# Exosomal Circular RNA as a Biomarker Platform for the Early Diagnosis of Immune-Mediated Demyelinating Disease

**DOI:** 10.3389/fgene.2019.00860

**Published:** 2019-09-27

**Authors:** Jinting He, Ming Ren, Haiqi Li, Le Yang, Xiaofeng Wang, Qiwei Yang

**Affiliations:** ^1^Department of Neurology, China-Japan Union Hospital of Jilin University, Changchun, China; ^2^Jilin Provincial Key Laboratory on Molecular and Chemical Genetics, Medical Research Center, Second Hospital of Jilin University, Changchun, China; ^3^Department of Orthopedics, Second Hospital of Jilin University, Changchun, China; ^4^Department of Endocrinology, The People’s Hospital of Jilin Province, Changchun, China; ^5^Department of Stomatology, China-Japan Union Hospital of Jilin University, Jilin University, Changchun, China

**Keywords:** exosome, circular RNA, immune-mediated demyelinating disease, cerebrospinal fluid, biomarker

## Abstract

Exosomes can pass through the blood-brain barrier and are present in the cerebrospinal fluid (CSF). The components in exosomes, such as DNA, RNA, protein, and lipids, change greatly and are closely related to disease progression. Circular RNA (circRNA) is stable in structure and has a long half-life in exosomes without degradation. Therefore, circRNA is considered an ideal biomarker and can be used to monitor a variety of central nervous system diseases. This study aimed to investigate the expression profiles of exosomal circRNA (exo-circRNA) in CSF from patients with immune-mediated demyelinating diseases to identify suitable biomarkers for the early diagnosis of immune-mediated demyelinating diseases. circRNA expression levels in exosomes obtained from five CSF samples from immune-mediated demyelinating disease patients and five paired CSF control samples were analyzed using a hybridization array. Hierarchical clustering analysis showed that 5,095 exo-circRNAs were differentially expressed between patients with immune-mediated demyelinating diseases and paired control samples. Of these exo-circRNAs, 26 were identified as significantly differentially expressed in CSF exosomes from patients with immune-mediated demyelinating diseases (FC ≥1.5 and p ≤ 0.05). Gene Ontology and Kyoto Encyclopedia of Genes and Genomes enrichment analysis indicated that the upregulation or activation of protein tyrosine phosphatase receptor type F (PTPRF) and RAD23 homolog B, nucleotide excision repair protein (RAD23B) may be associated with the occurrence and development of immune-mediated demyelinating diseases. Then, a competing endogenous RNA network was constructed and centered on the most upregulated/downregulated exo-circRNAs to predict their function in immune-mediated demyelinating diseases. In addition, reverse transcription quantitative polymerase chain reaction results stating that hsa_circ_0087862 and hsa_circ_0012077 were validated in an independent cohort of subjects. Canonical correlation analysis results indicated a potential connection between exosomal hsa_circ_0012077 expression level and immunoglobulin G levels in CSF. Finally, the receiver operating characteristic (ROC) curve showed that when hsa_circ_0087862 or hsa_circ_0012077 was employed alone for diagnosing immune-mediated demyelinating diseases, the diagnostic accuracy was 100%. In conclusion, based on this study, exosomal hsa_circ_0087862 and hsa_circ_0012077 in CSF could be used as suitable biomarkers for the diagnosis of immune-mediated demyelinating disease based on their expression levels. Moreover, the upregulation or activation of PTPRF and RAD23B was potentially associated with the occurrence and development of immune-mediated demyelinating diseases.

## Introduction

Demyelinating diseases are acquired diseases with varying etiologies and clinical manifestations but similar clinical characteristics (Hoftberger and Lassmann, 2017). The characteristic pathological change in these diseases is the demyelination of nerve fibers while neuronal cells remain intact. The primary role of myelin is to protect neurons and allow nerve impulses to be transmitted quickly and efficiently through neurons. The loss of myelin can affect the transmission of nerve impulses. Demyelinating diseases can occur in the central or peripheral nervous system. The main causes are as follows: 1, immune-mediated disorders, such as multiple sclerosis (MS) and acute infective polyradicular neuritis (also known as Guillain-Barré syndrome, GBS); 2, viral infection, such as progressive multifocal leukoencephalitis and subacute sclerosing panencephalitis; 3, nutritional disorders, such as central pontine myelin disintegration; and 4, hypoxia, such as delayed hypoxic demyelinating encephalopathy and progressive subcortical ischemic encephalopathy (Matute-Blanch et al., 2017). The diagnosis of demyelinating diseases in clinical work mostly refers to immune-mediated demyelinating diseases, including MS and GBS. In China, the main treatments in the early stages of immune-mediated demyelinating diseases are hormones and nutritional therapy (Neuroimmunity Branch of Chinese Society of Immunology and Neuroimmunology Group, 2018). In most of the other countries, immunomodulating therapies are the main treatments, but the efficacy is difficult to determine. In acute demyelinating disease, the nerve myelin sheath can be regenerated, and regeneration is rapid and complete (Galetta and Bhattacharyya, 2019). Although the regenerated myelin sheath is thinner than normal, it generally has little effect on functional recovery. In chronic demyelinating diseases, repeated demyelination and remyelination result in significant proliferation of Schwann cells, thickening of nerve fibers, and loss of axons, which may lead to incomplete recovery of nerve function (Khoo et al., 2019). Early diagnosis and monitoring can enable intervention in the early stage of the lesion or even when the lesion has not yet formed to reduce damage to the body and prevent the occurrence of sequelae.

Schwann cells release large amounts of extracellular vesicles and exosomes in the early stage of demyelinating diseases. Exosomes can pass through the blood-brain barrier; some exosomes enter the bloodstream (Li et al., 2015) while most exosomes are present in the cerebrospinal fluid (CSF). Exosomes contain a variety of components, including DNA, RNA, protein, and lipids, and these components slightly fluctuate with physiological conditions. In the disease state, these components change greatly, and the expression of certain substances is closely related to disease progression (Li et al., 2018b). In tumor patients, exosomes can be used to detect the mutation status of tumor DNA or the expression level of RNA (Figueroa et al., 2017; Shankar et al., 2017; Manda et al., 2018). Exosomes become important samples for monitoring the progression of central nervous system (CNS) diseases because nerve tissues are not available for biopsy. The circular RNA (circRNA) carried in the exosomes, because of its lack of an exonuclease recognition site, is not easily degraded (Memczak et al., 2013); thus, circRNA is considered an ideal biomarker and can be used to monitor a variety of CNS diseases (Lukiw, 2013; Qu et al., 2015; Lu and Xu, 2016). However, few studies have focused on exosomal circRNA (exo-circRNA), and most of these studies explored the role of exo-circRNA in neoplastic diseases (Greene et al., 2017). By contrast, changes in exo-circRNA content during demyelinating diseases have not been reported.

In the present study, we investigated the exo-circRNA expression profiles in the CSF of patients with immune-mediated demyelinating diseases. This study aimed to explore suitable biomarkers for the early diagnosis of immune-mediated demyelinating diseases. Moreover, analyzing exo-circRNA expression profiles can also aid in determining the molecular mechanism of immune-mediated demyelinating diseases.

## Materials and Methods

### Cerebrospinal Fluid Samples

Five CSF samples from patients with immune-mediated demyelinating disease (demyelinating disease group) and five paired adjacent CSF samples from patients who underwent lumbar puncture because of abnormal intracranial pressure (control group) were obtained from the Department of Neurology, China-Japan Union Hospital of Jilin University (Changchun, China). The clinical information and laboratory data of the patients/donors are shown in **Table 1**.

**Table 1:** Clinical information and laboratory data of the patients/control samples.

Factor	Immune-mediated demyelinating disease patients (n = 5)	Control samples (n = 5)
**Age (years)**		
Mean ± SD	45.80 ± 20.91	30.80 ± 19.88
Range	18–70	16–65
Median	54	21
**Sex**		
Male	1	2
Female	4	3
**Demyelinating type**		
MS	3	–
GBS	2	–
**Disease stage**		
Acute stage	5	–
Remission stage	0	–
**Disease progression**		
First episode	4	–
Recurrent	1	–
**Treatment**		
Yes	0	–
No	5	–
**Disease duration (days)**	16.60 ± 10.57	–
**Laboratory data**		
CSF red blood cells		
Negative	5	5
Positive	0	0
CSF leukocyte count (×10^6^/L)	5.10 ± 2.88	2.60 ± 2.88
CSF glucose (mmol/L)	3.72 ± 0.77	3.54 ± 1.00
CSF protein (g/L)	0.92 ± 0.96	0.49 ± 0.70
CSF chloride (mmol/L)	120.92 ± 2.60	122.90 ± 2.61
Thyroid stimulating hormone (mIU/L)	2.75 ± 1.13	2.72 ± 1.22
Free triiodothyronine (pmol/L)	4.74 ± 0.84	4.55 ± 0.64
Free thyroxine (pmol/L)	13.94 ± 2.41	14.02 ± 2.57
C-reactive protein (mg/L)	28.75 ± 6.80	19.34 ± 13.21
Folic acid (ng/ml)	14.90 ± 7.95	29.88 ± 14.98
Vitamin B12 (pg/ml)	550.00 ± 251.43	407.80 ± 110.87
Homocysteine (µmol/L)	36.00 ± 19.18	21.80 ± 16.38
CSF IgG (oligoclonal bands)		
Negative	1	5
Positive	4	0
Serum IgG (oligoclonal bands)		
Negative	5	5
Positive	0	0

### Exosome Enrichment

CSF (7 ml) from each sample was used for exosome enrichment using the ultracentrifugation method. The specific operation was as follows. CSF was centrifuged at 500 g for 5 min to eliminate cell components. The supernatant was further centrifuged at 2,000 g for 10 min to eliminate debris. The supernatant was further centrifuged at 10,000 g for 10 min to eliminate shed microvesicles and then filtered through a 0.22-μm membrane filter (Merck Millipore, Germany) and centrifuged at 100,000 g for 2 h. The pellet was resuspended in PBS and centrifuged at 100,000 g for 2 h; finally, the pellet was resuspended in PBS and stored at -80°C for further use. The enriched exosomes were identified by transmission electron microscopy (TEM) (Tecnai G2 Spirit 120KV, FEI, Hong Kong, China).

### circRNA Preparation

Total RNA from each sample was extracted by TRIzol reagent (Invitrogen, USA) according to the manufacturer’s instructions. Then, total RNAs were digested with RNase R (Epicentre, USA) to remove linear RNAs and enrich circular RNAs.

### Labeling and Hybridization

The labeled cRNAs were hybridized onto the Arraystar Human circRNA Array V2 (8×15K, Arraystar, USA). Sample labeling and array hybridization were performed according to the manufacturer’s instructions (Arraystar, USA). Briefly, enriched circRNAs were transcribed into fluorescent-labeled cRNAs by an Arraystar Super RNA Labeling Kit (Arraystar, USA) and then purified using an RNeasy Mini Kit (Qiagen, USA). A total of 1 μg of cRNA from each sample was fragmented by blocking agent and fragmentation buffer and incubated at 60°C for 30 min. Hybridization buffer was used to dilute the labeled cRNA. Then, the mixture was loaded onto a circRNA expression microarray slide. The slides were incubated at 65°C for 17 h in an Agilent Hybridization Oven (Agilent Technologies, USA). Next, the slides were washed, fixed, and scanned by Agilent Scanner G2505C (Agilent Technologies, USA).

### Hybridization Data Analysis

The hybridization data were extracted by Agilent Feature Extraction software (Agilent Technologies, USA). Raw data were imported into the R software Limma package for data quantile normalization and subsequent processing. Fold change (FC) was used as an indicator of circRNA differential expression between the immune-mediated demyelinating disease group and the control group. T-tests were employed to estimate the statistical significance of differences. The circRNA with FC ≥ 1.5 and p ≤ 0.05 were defined as significantly differentially expressed between the immune-mediated demyelinating disease group and the control group.

### Reverse Transcription Quantitative Polymerase Chain Reaction

A total of 30 ng of total RNA was used for cDNA synthesis using M-MuLV First Strand cDNA Synthesis Kit (Sangon Biotech, China) and for reverse transcription quantitative polymerase chain reaction (RT-qPCR) analysis using 2X SG Fast qPCR Master Mix (Low Rox) (Sangon Biotech, China), according to the manufacturers’ instructions. The primers were designed by Primer-BLAST online tool (https://www.ncbi.nlm.nih.gov/tools/primer-blast/index.cgi?LINK_LOC=BlastHome) and synthesized by Sangon Biotech, Inc. (China). The primer sequences are as follows:

hsa_circ_0087862 forward: 5’-GCAAGTAATTGCAGCCCTG​AG-3’, reverse: 5’-GCTGAGTTGTAGCTGGTGCT-3’; hsa_circ_0012077 forward: 5’-TCGTACGCTCTGCCAACTAC-3’, reverse: 5’-CATCCACTTCACGTAGGGCA-3’; hsa_circ_0001627 forward: 5’-GGTAGGGTTTTCTTGCTTCTGG-3’, reverse: 5’-CTGGGAGCTATGTGAACGAAC-3’; hsa_circ_0082352 forward: 5’-GAGACGAATGGGTTGTCCAGA-3’, reverse: 5’-ATCCACTGCTCTCCACTGGT-3’; and GAPDH, employed as a reference gene, forward: 5’- CGGACCAATACGACCAAATCCG -3’, reverse: 5’- AGCCACATCGCTCAGACACC -3’. RT-qPCR analysis was performed on a 7500 Fast Dx Real-Time PCR Instrument (Applied Biosystems, USA) and was repeated three times for each sample. The 2^-ΔΔCt^ method was employed to calculate the relative expression levels based on cycle threshold value (Ct) data. The two-tail Student’s t-test was used for comparing the relative expression levels between immune-mediated demyelinating disease samples and control samples.

### Bioinformatics Analysis

Detailed circRNA information was obtained from the circBase database (http://www.circbase.org/). The Circular RNA Interactome database (https://circinteractome.nia.nih.gov/) was employed to predict all miRNAs that were most likely associated with the chosen circRNAs. The circRNA/miRNA interaction was predicted with Arraystar’s miRNA target prediction software (Arraystar, USA) based on the TargetScan (http://www.targetscan.org/vert_71/) and miRanda (http://34.236.212.39/microrna/microrna/home.do) databases. The miRNA/mRNA interaction was predicted with TargetScan (http://www.targetscan.org/vert_71/). A circRNA-miRNA-mRNA network was constructed using Cytoscape 3.5.1. The OmicsBean database (http://www.omicsbean.cn/) was employed for Gene Ontology (GO) enrichment analysis and Kyoto Encyclopedia of Genes and Genomes (KEGG) analysis.

### Statistical Analysis

Statistical analyses were performed using SPSS version 21 (SPSS Inc., USA). The quantitative data are shown as the mean ± standard deviation (SD). The two-tail Student’s t-test was used for comparisons between two groups, p < 0.05 indicates a significant difference. The relationship between exo-circRNA expression levels (normalized intensity data of each circRNA in each sample from the hybridization data) and clinical laboratory data (the raw data in the clinical test report) was analyzed using canonical correlation analysis. A receiver operating characteristic (ROC) curve was used to validate diagnostic accuracy. In this study, no sample calculation and assessment of data outliers and data normality were performed, and the experiments were performed unblinded.

## Results

### Exosome Enrichment

**Figure 1:**
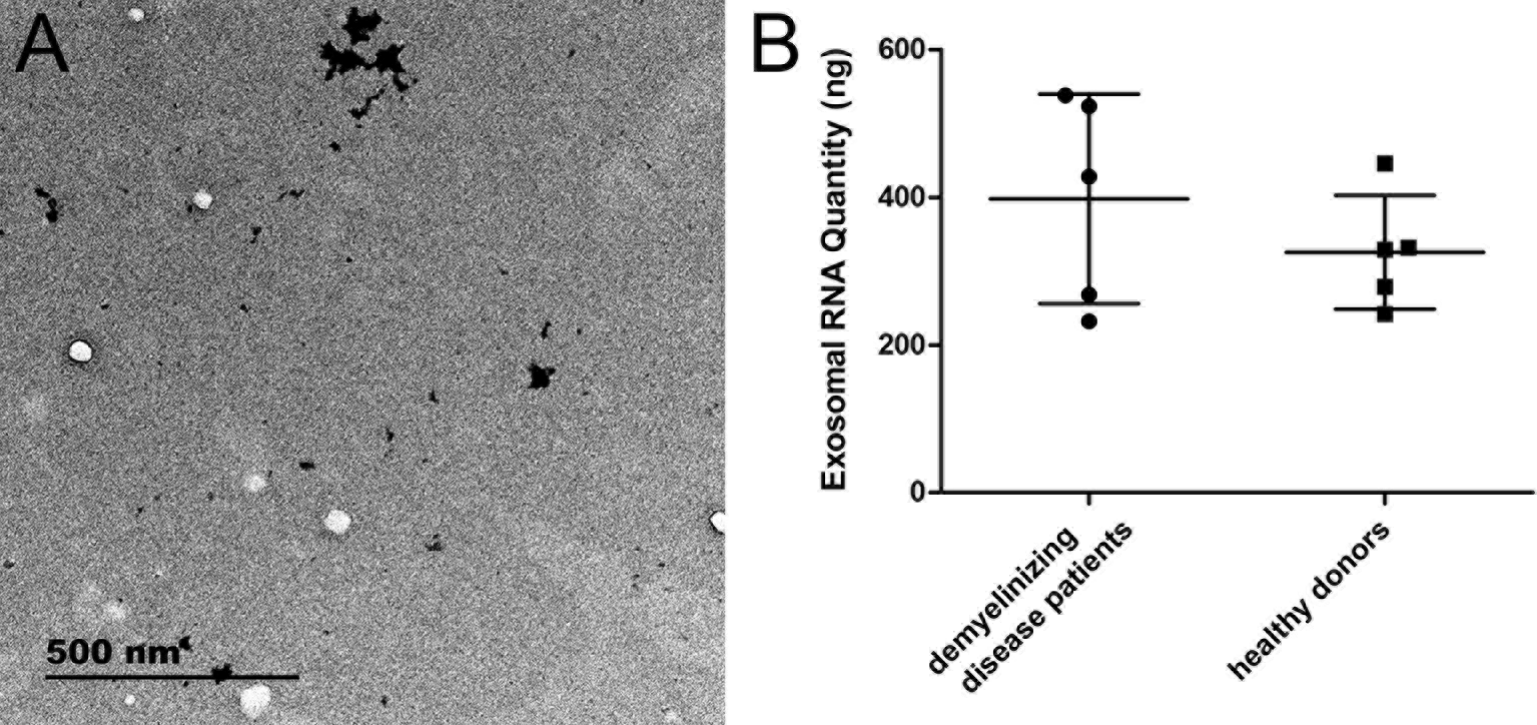
The characteristics of exosomes and exosomal RNA. **(A)** Representative TEM image of exosomes enriched from CSF. **(B)** The RNA quantity extracted from exosomes (n = 5 for each group).

Exosomes were successfully enriched from each CSF sample. Representative TEM images are presented in **Figure 1A**. This finding indicated that a certain amount of exosome is present in the CSF and can be enriched by conventional experimental techniques.

### circRNA Preparation

The quantity of total RNA extracted from patients with immune-mediated demyelinating disease and from healthy donors was 397.65 ± 141.91 ng and 325.35 ± 76.94 ng, respectively, with no significant difference between the two groups (**Figure 1B**). This result indicated that the quantity of RNA extracted from exosomes was sufficient for molecular detection techniques, such as RNA array analysis, RNA sequencing, and RT-qPCR.

### Labeling and Hybridization

The fluorescence of the hybridized microarray slide was clearly distinguishable. The microarray slide scanning images are shown in **Supplementary Figure 1**.

### exo-CircRNA Expression Profiles

**Figure 2:**
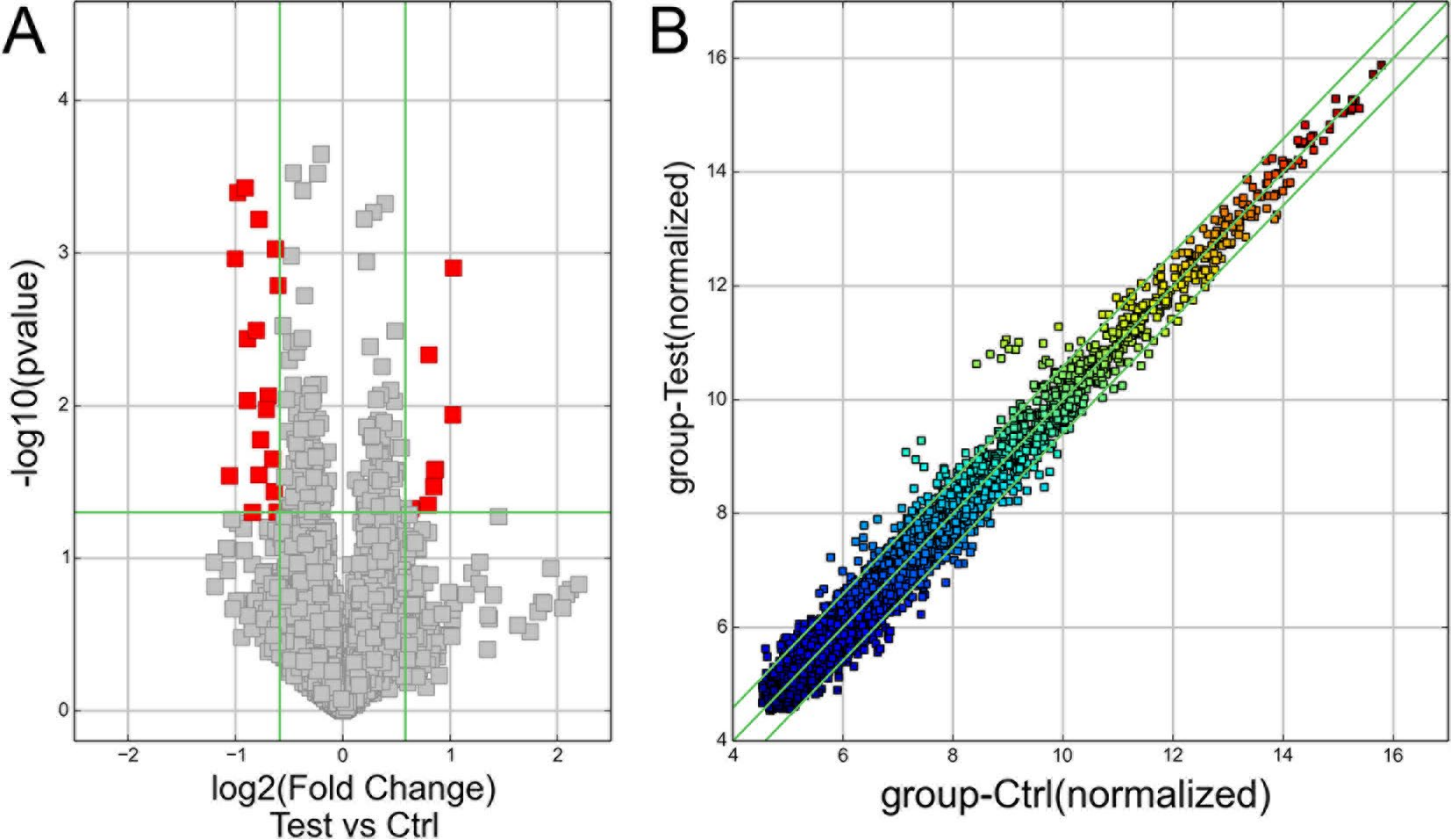
The exo-circRNA expression profile in exosomes. **(A)** The volcano plot graph of exo-circRNAs was constructed according to fold change values and p–values. The X axis represents the log_2_ (fold change) value of differential expression, and the Y axis represents the -log_10_ (padj) value of differential expression. The vertical lines correspond to 1.5-fold upregulation and downregulation between the immune-mediated demyelinating disease and control groups, and the horizontal line represents a p–value of 0.05. The red points in the plot represent significantly upregulated differentially expressed exo-circRNAs, and the gray points in the plot represent significantly downregulated differentially expressed exo-circRNAs. **(B)** A scatter plot graph of exo-circRNAs was constructed according to the normalized expression values of exo-circRNAs between the immune-mediated demyelinating disease and control groups.

**Figure 3:**
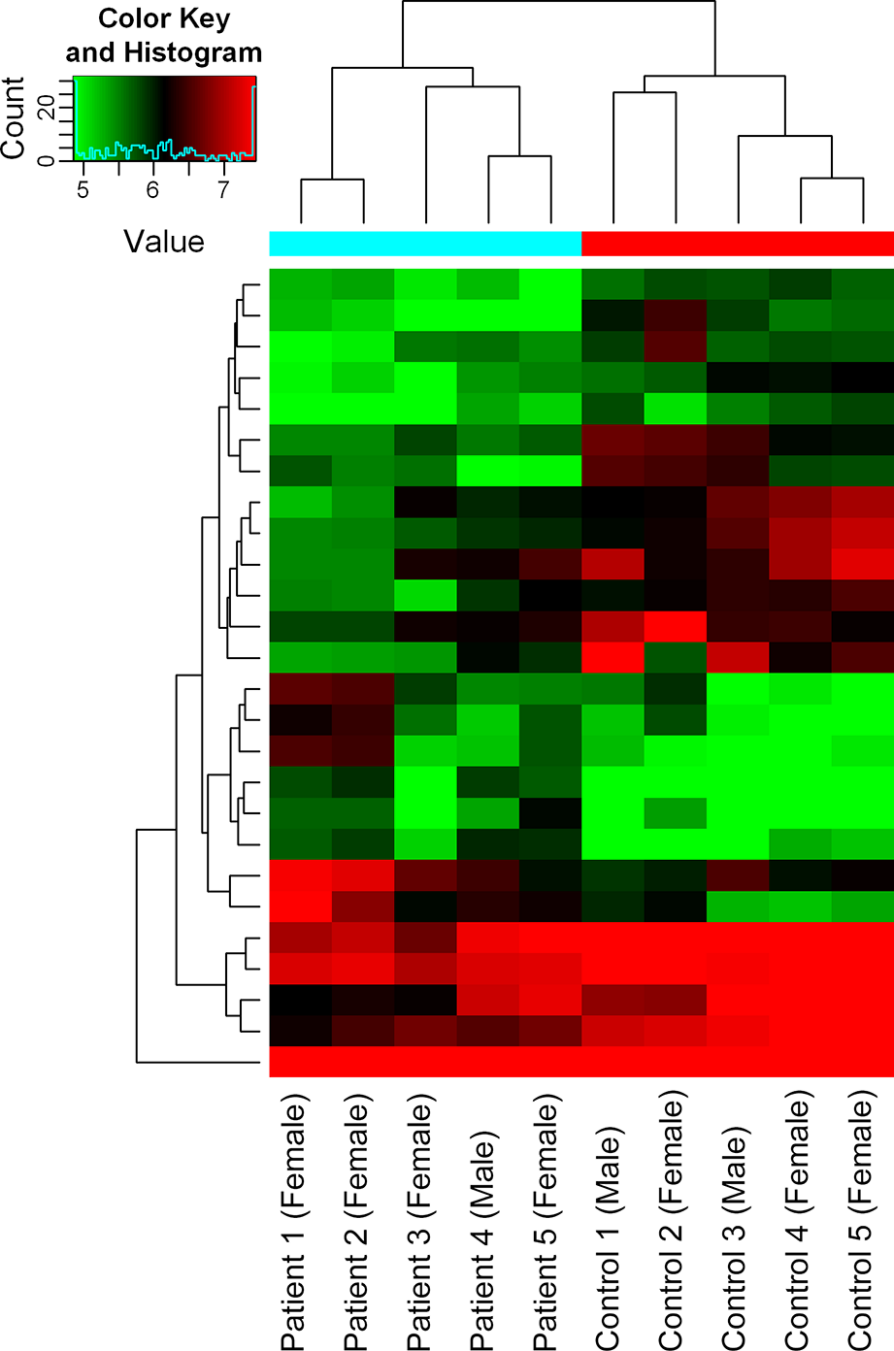
Hierarchical clustering analysis of exo-circRNAs. Hierarchical clustering analysis of exo-circRNAs that were differentially expressed with FC ≥1.5 and p ≤ 0.05 between the immune-mediated demyelinating disease and control groups. The X–axis represents samples (Patient 1–Patient 5: patients with immune-mediated demyelinating diseases; Control 1–Control 5: healthy donors), and the Y–axis represents differentially expressed exo-circRNAs. Expression values are represented in different colors; red indicates high expression levels, and green indicates low expression levels.

In total, 13,617 circRNAs were investigated to explore exo-circRNA expression profiling in CSF exosomes from patients with immune-mediated demyelinating disease. The exo-circRNA expression profiling between five patients with immune-mediated demyelinating disease and five adjacent control samples was revealed by hierarchical clustering. Overall, 5,095 exo-circRNAs were identified as differentially expressed. Of these exo-circRNAs, 2,364 were identified as upregulated, and 2,730 were identified as downregulated in CSF exosomes of patients with immune-mediated demyelinating disease. For the purpose of further screening for exo-circRNAs with significant differences in expression, FC ≥ 1.5 and p ≤ 0.05 were defined as cutoff values of significant differential expression. Volcano plot graphs (**Figure 2**) showed that 26 exo-circRNAs were differentially expressed in the CSF exosomes of patients with immune-mediated demyelinating disease. The expression profiles were demonstrated by a cluster heat map (**Figure 3**). Among these 26 exo-circRNAs, 8 were upregulated, and 18 were downregulated (**Table 2**). The details of these differentially expressed exo-circRNAs are listed in **Supplementary Table 1**.

**Table 2:** Significantly differentially expressed circRNAs between the immune-mediated demyelinating disease and control groups.

circRNA	FC	P–value	False Discover Rate	Regulation	Gene Symbol
hsa_circ_0087862	2.042157	0.001247068	0.423504188	up	RAD23B
hsa_circ_0012077	2.0347472	0.011459209	0.981034338	up	PTPRF
hsa_circ_0018659	1.819264	0.026043902	0.981034338	up	PALD1
hsa_circ_0026457	1.8080354	0.026340789	0.981034338	up	KRT5
hsa_circ_0081069	1.8001586	0.033936895	0.981034338	up	COL1A2
hsa_circ_0087856	1.7423365	0.004624728	0.872532088	up	RAD23B
hsa_circ_0008029	1.7347416	0.044784595	0.981034338	up	XLOC_005768
hsa_circ_0000559	1.5539134	0.047125197	0.981034338	up	FOXN3
hsa_circ_0001627	2.0787813	0.028786419	0.981034338	down	BACH2
hsa_circ_0082352	2.0060823	0.001084353	0.413057691	down	TMEM209
hsa_circ_0001355	1.9709762	0.000399538	0.304961321	down	RSRC1
hsa_circ_0014887	1.877371	0.000371477	0.304961321	down	COPA
hsa_circRNA_400660	1.8505931	0.003637707	0.806611429	down	CACUL1
hsa_circ_0000685	1.8500083	0.009224606	0.981034338	down	BOLA2
hsa_circ_0005735	1.7936504	0.049856633	0.981034338	down	EIF4G3
hsa_circ_0087571	1.7479558	0.003192816	0.806611429	down	HIATL1
hsa_circ_0001036	1.7188557	0.028378376	0.981034338	down	TGOLN2
hsa_circ_0007845	1.7176239	0.000598668	0.304961321	down	MLF2
hsa_circ_0014923	1.7011388	0.016684681	0.981034338	down	COPA
hsa_circ_0036599	1.6369447	0.010539743	0.981034338	down	SEC11A
hsa_circ_0004203	1.620143	0.008594196	0.981034338	down	ZNF778
hsa_circ_0000158	1.5709875	0.022347385	0.981034338	down	SELL
hsa_circ_0021569	1.5548393	0.036490975	0.981034338	down	QSER1
hsa_circRNA_404940	1.5434813	0.000934805	0.413057691	down	ARCN1
hsa_circ_0000254	1.5305835	0.049608482	0.981034338	down	RRP12
hsa_circ_0007395	1.5198962	0.001628428	0.518450704	down	KMT2E

### exo-circRNA Spliced Length Distribution

**Figure 4:**
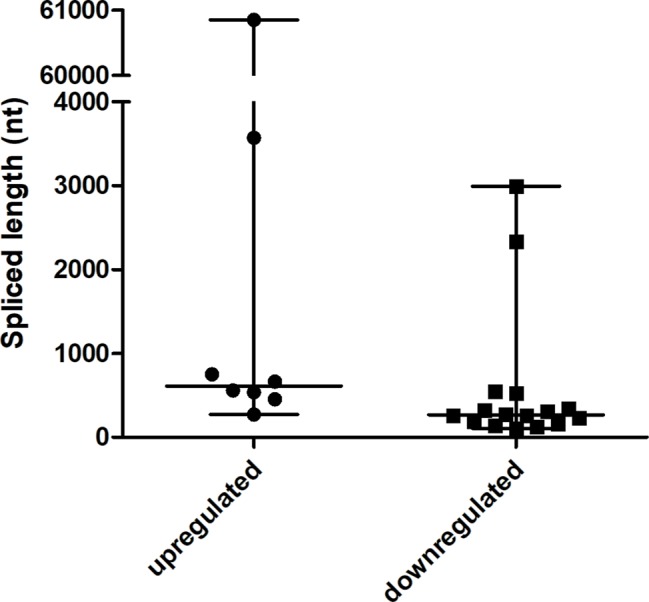
The spliced lengths of significant differentially expressed exo-circRNAs (n = 8 for the upregulated group, n = 18 for the downregulated group).

The spliced lengths of significant differentially expressed exo-circRNAs ranged from 108 to 60,853 nt with a median of 332.5 nt. There was no significant difference between upregulated and downregulated exo-circRNAs (**Figure 4**). Most exo-circRNAs were less than 1,000 nt in spliced length, but there were also large exo-circRNA fragments of up to 60,853 nt, such as hsa_circ_0008029. This finding indicates that small and large circRNA molecules are harbored in exosomes and can show significant differential expressions.

### Enrichment Analysis of Derived Gene Function of Differentially Expressed exo-CircRNA

**Figure 5:**
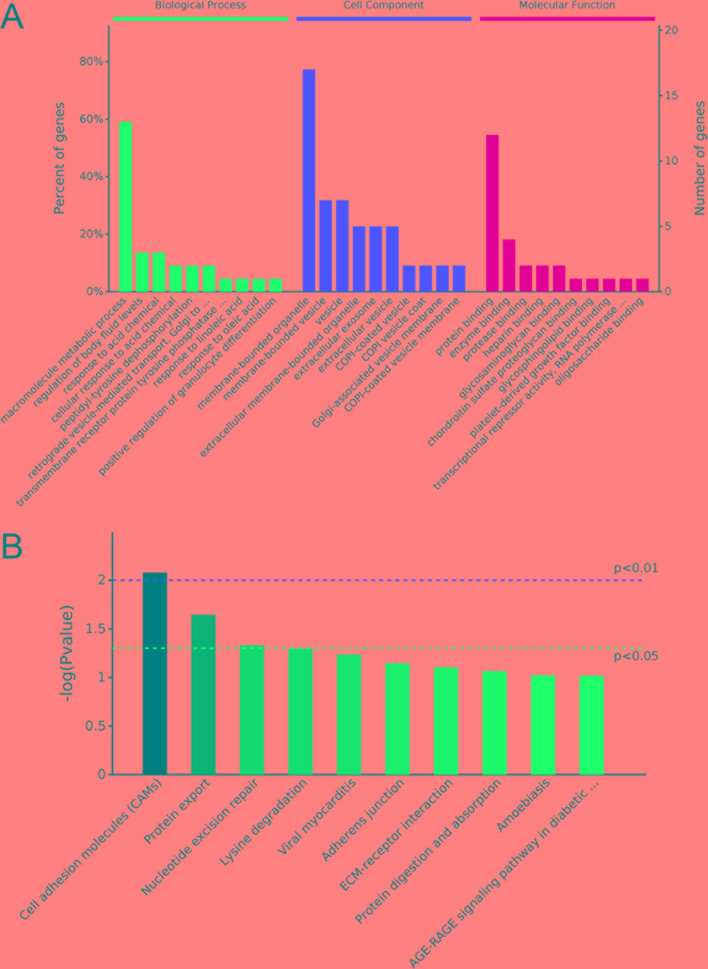
GO and KEGG analysis of parental gene function of differentially expressed exo-circRNAs. **(A)** GO analysis covers the three domains of biological process, cell component, and molecular function. The X axis represents the top 10 functions in each term, and the Y–axis represents the gene number and proportion of all annotated genes. **(B)** KEGG analysis determined the most important biochemical metabolic pathways and signal transduction pathways in which the genes are involved. The X–axis represents related pathways, and the Y–axis presents normalized p–values in this pathway.

According to the correspondence between circRNAs and derived genes, GO and KEGG enrichment analyses were performed to reveal the potential function of differentially expressed exo-circRNAs. GO classification showed that macromolecule metabolic process, membrane-bounded organelle, and protein binding were the major functions of these differentially expressed exo-circRNAs (**Figure 5A**). Protein tyrosine phosphatase receptor type F (PTPRF); PALD1; COL1A2; RAD23 homolog B, nucleotide excision repair protein (RAD23B); CACUL1; ZNF778; and KMT2E were involved in all three of these functions (**Supplementary Table 2**). KEGG enrichment revealed that cell adhesion molecules (CAMs), protein export, and nucleotide excision repair (corresponding to PTPRF/SELL, SEC11A, and RAD23B, respectively) were the main pathways by which these genes function (**Figure 5B**, **Supplementary Table 3**). Notably, both hsa_circ_0087862 and hsa_circ_0087856 were derived from RAD23B, whereas hsa_circ_0014887 and hsa_circ_0014923 were derived from coatomer protein complex subunit alpha (COPA) (**Table 2**).

### Expression Profiles of Significantly Differentially Expressed exo-circRNA

**Figure 6:**
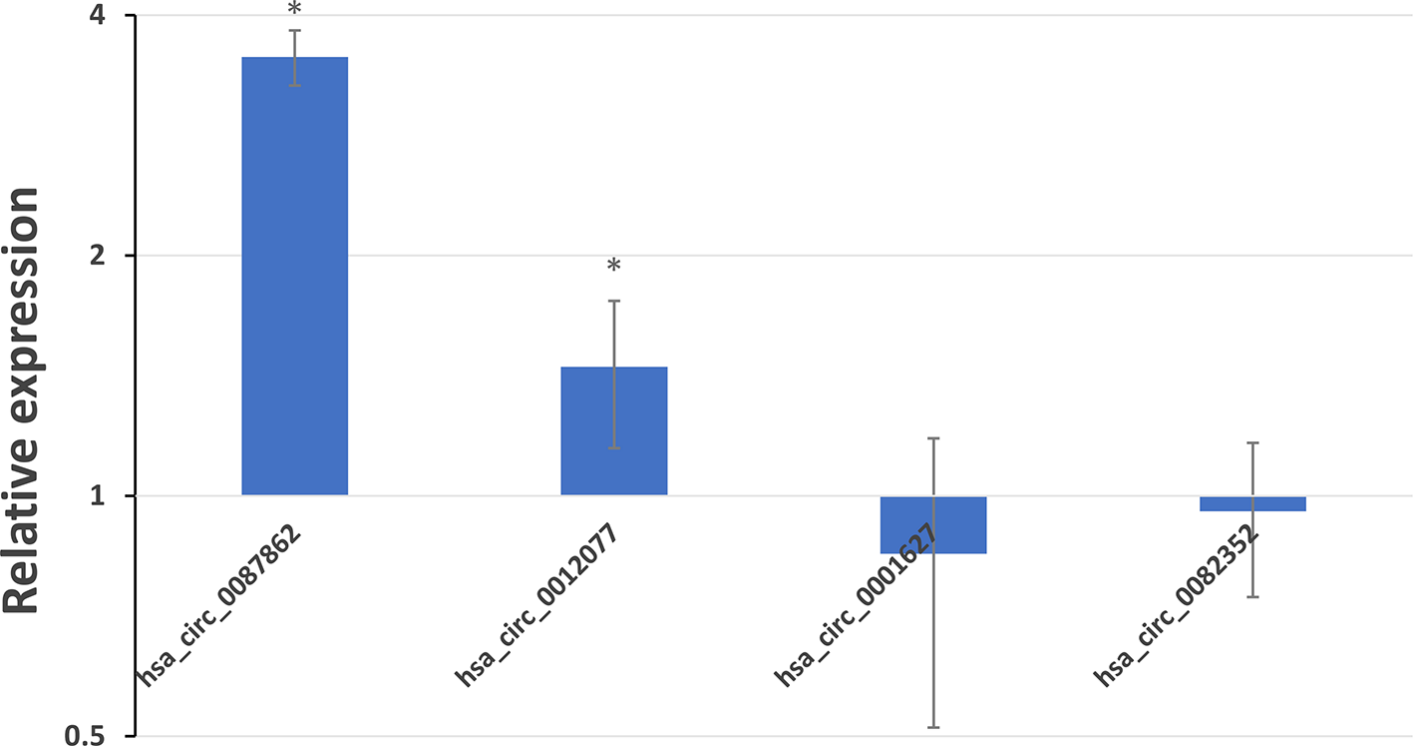
Expression profiles of hsa_circ_0087862, hsa_circ_0012077, hsa_circ_0001627, and hsa_circ_0082352 in immune-mediated demyelinating disease samples compared with control samples. The expression levels of the circRNAs were analyzed by the 2^−ΔΔCt^ method using RT-qPCR. The x–axis presents the circRNAs. The y–axis presents the relative expression level on a logarithmic scale based on 2; a relative expression level higher than 1.00 indicates upregulation, whereas a relative expression level lower than 1.00 indicates downregulation. The bars present the SD value of each group, * indicates the expression level is significant different between immune-mediated demyelinating disease samples and control samples.

Among all significantly differentially expressed exo-circRNAs, only four showed over a two-fold change. Hsa_circ_0087862 and hsa_circ_0012077 were upregulated 2.04- and 2.03-fold, respectively, and hsa_circ_0001627 and hsa_circ_0082352 were downregulated 2.08- and 2.01-fold, respectively. The results of hybridization data analysis were validated by RT-qPCR. RT-qPCR was performed in the same samples and another 10 paired samples (from five patients with immune-mediated demyelinating disease and five paired adjacent control samples; the clinical information and laboratory data of the patients/donors are shown in **Supplementary Table 4**). The expression levels in the control group were normalized to 1.00. Therefore, a relative expression level higher than 1.00 in the immune-mediated demyelinating disease group indicates an upregulation in immune-mediated demyelinating disease samples compared with control samples; in contrast, a relative expression level lower than 1.00 indicates a downregulation. As expected, the expressions of hsa_circ_0087862 and hsa_circ_0012077 in immune-mediated demyelinating disease samples were upregulated 3.55-fold and 1.45-fold, respectively, compared with control samples, with statistically significant differences (p < 0.05); the expressions of hsa_circ_0001627 and hsa_circ_0082352 in immune-mediated demyelinating disease samples were downregulated 1.18-fold and 1.05-fold, respectively, compared with control samples (**Figure 6**).

### Functional Prediction of Differentially Expressed exo-circRNA

**Figure 7:**
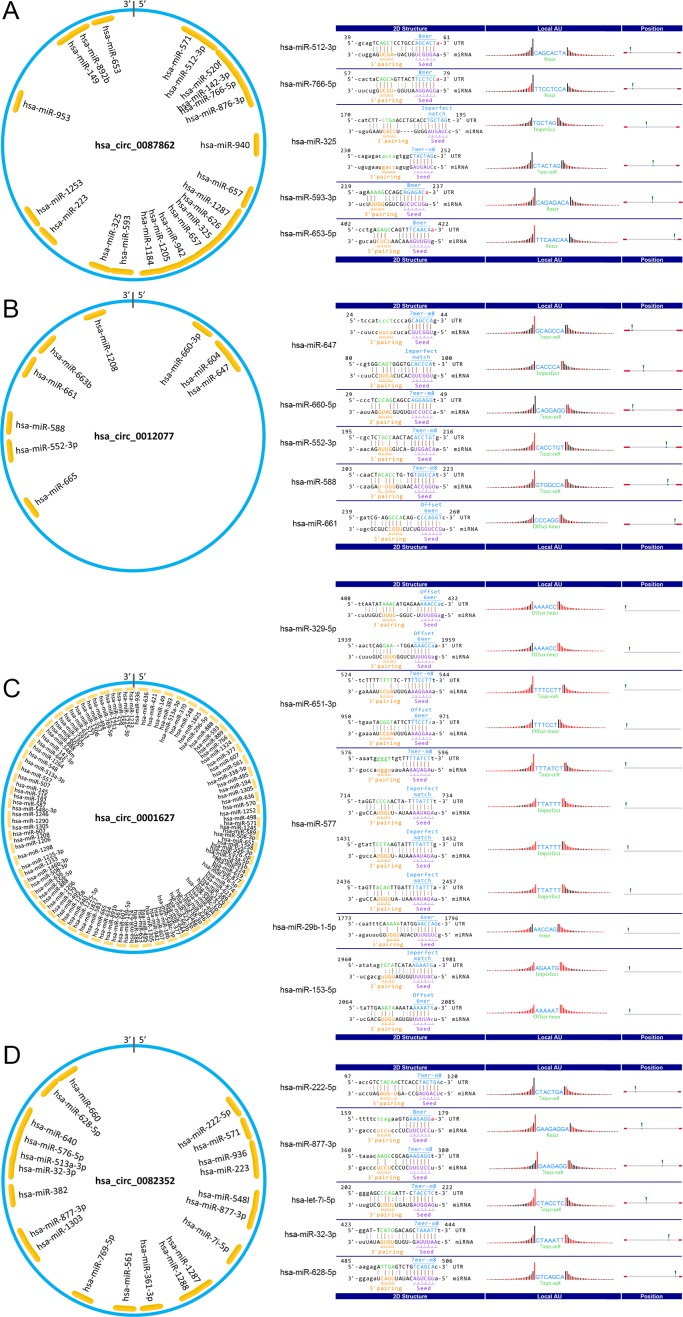
Pattern diagram of exo-circRNAs and their miRNA binding sites. The schematic model showing the putative binding sites for miRNAs of **(A)** hsa_circRNA_0087862, **(B)** hsa_circRNA_0012077, **(C)** hsa_circRNA_0001627, and **(D)** hsa_circRNA_0082352. The indigo circles represent the structure of exo-circRNAs, and the yellow fragments represent miRNAs.

The competing endogenous RNA (ceRNA) mechanism is regarded as one of the most important mechanisms by which circRNAs regulate gene expression (Salmena et al., 2011). CircRNAs function as molecular sponges for a miRNA to bind to its binding site and suppress the expression and function of all target genes of the respective miRNA family (Hansen et al., 2013; Memczak et al., 2013). To predict the function of differentially expressed exo-circRNAs, we constructed the circRNA/microRNA interaction according to the Circular RNA Interactome database and Arraystar’s miRNA target prediction software, which was based on TargetScan and miRanda. Hsa_circ_0087862 has a spliced length of 453 nt and was predicted to interact with 21 miRNAs, as shown in **Figure 7A**. Hsa_circ_0012077 has a spliced length of 270 nt and was predicted to interact with 9 miRNAs, as shown in **Figure 7B**. Hsa_circ_0001627 has a spliced length of 2,995 nt and was predicted to interact with 95 miRNAs, as shown in **Figure 7C**. Hsa_circ_0082352 has a spliced length of 547 nt and was predicted to interact with 20 miRNAs, as shown in **Figure 7D**.

**Figure 8:**
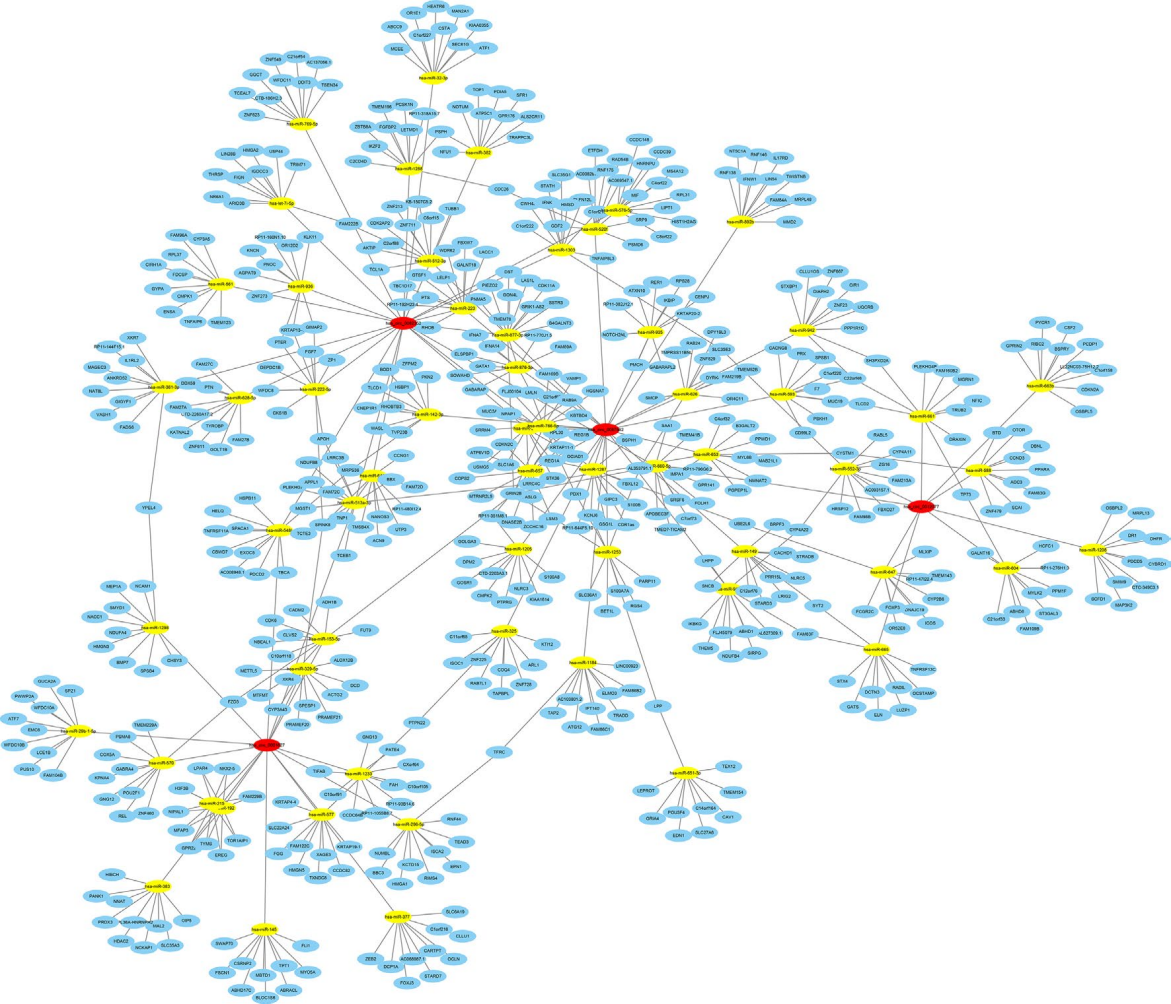
ceRNA analysis for differentially expressed exo-circRNAs. Four exo-circRNAs (red nodes), 60 miRNAs (yellow nodes) and 552 target genes (blue nodes) were involved in the ceRNA network. Solid lines represent the relationship between two nodes.

In addition, we constructed a ceRNA network. All predicted mRNAs of target genes were ranked according to the total context score, and only the top 10 target genes were selected for constructing the network. After removing duplicate items, there were a total of 60 miRNAs and 552 mRNAs of target genes involved in the ceRNA network (**Figure 8**). The ceRNA network shows that these four circRNAs are involved in the regulation of multiple gene expression. Notably, the target gene GRIN2B (glutamate ionotropic receptor NMDA type subunit 2B) could be regulated by as many as five miRNAs (hsa-miR-1253, hsa-miR-153-5p, hsa-miR-660-5p, hsa-miR-766-5p, and hsa-miR-877-3p), which are associated with hsa_circ_0087862, hsa_circ_0012077, hsa_circ_0001627, and hsa_circ_0082352.

**Figure 9:**
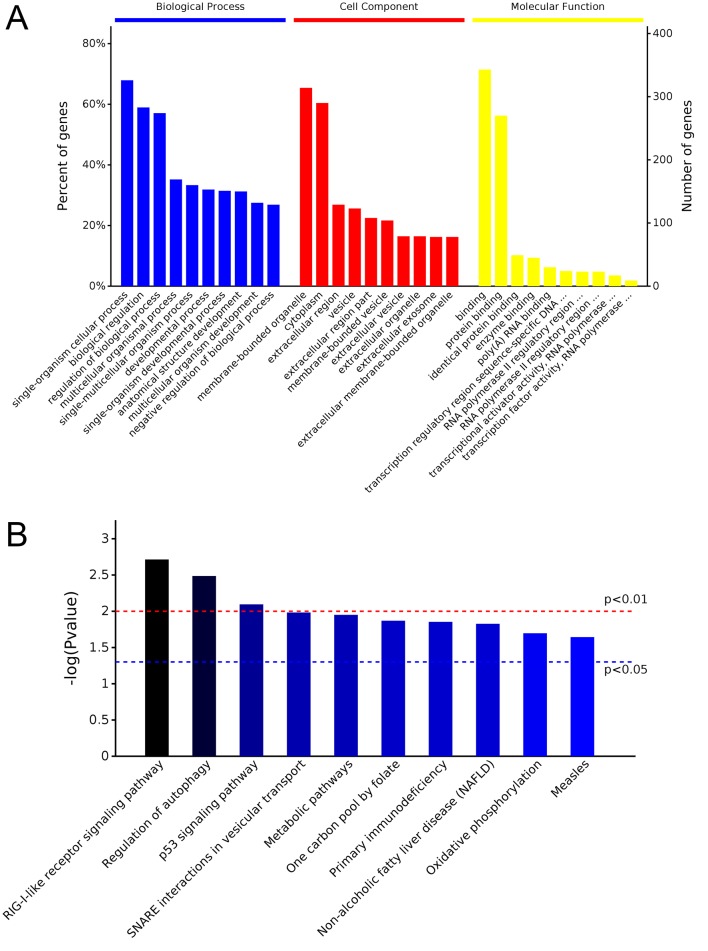
GO and KEGG analysis of potential target genes that may be regulated by differentially expressed exo-circRNAs. **(A)** GO analysis covers the three domains of biological process, cell component, and molecular function. The X–axis represents the top 10 functions in each term, and the Y–axis represents the gene number and proportion of all annotated genes. **(B)** KEGG analysis indicated the most important biochemical metabolic pathways and signal transduction pathways in which the genes are involved. The X–axis represents related pathways, and the Y–axis presents normalized p–values in this pathway.

Furthermore, we performed GO and KEGG analyses to determine the function of these target genes involved in the ceRNA network that may be regulated by differentially expressed exo-circRNAs. GO classification showed that single-organism cellular process, membrane-bounded organelle, and protein binding were the major functions of these differentially expressed exo-circRNAs (**Figure 9A**, **Supplementary Table 5**). KEGG enrichment revealed that RIG-I-like receptor signaling pathway, regulation of autophagy, and the p53 signaling pathway were the main pathways by which these genes function (**Figure 9B**, **Supplementary Table 6**).

### Diagnosis by exo-circRNA Expression Level

**Figure 10:**
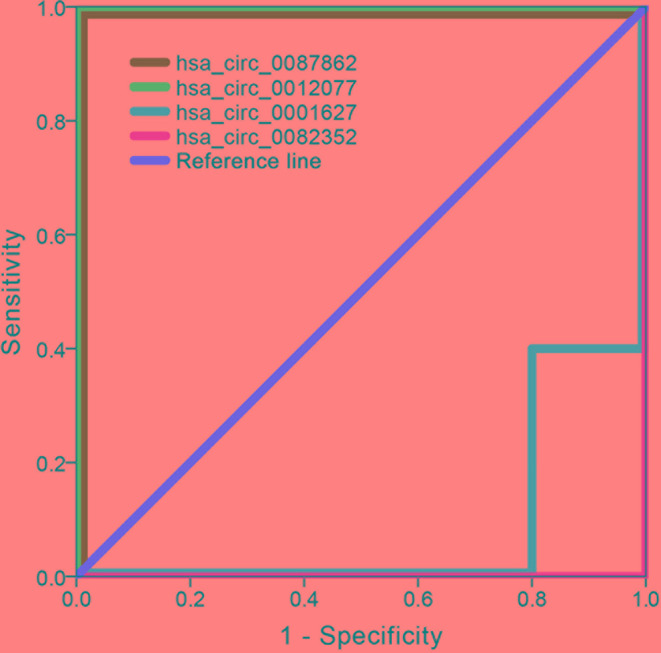
ROC curves of validating the diagnostic accuracy using the expression levels of hsa_circ_0087862, hsa_circ_0012077, hsa_circ_0001627, and hsa_circ_0082352 (n = 10).

The ROC curve was employed to validate the diagnostic accuracy using the expression levels of hsa_circ_0087862, hsa_circ_0012077, hsa_circ_0001627, and hsa_circ_0082352 (**Figure 10**). When hsa_circ_0087862 or hsa_circ_0012077, whose expression levels were statistically significantly different between immune-mediated demyelinating disease samples and control samples as validated by RT-qPCR as shown previously, was employed alone for diagnosing immune-mediated demyelinating disease, the area under the curve was 1.00, representing a diagnostic accuracy of 100%. These results indicate that the expression levels of exo-hsa_circ_0087862 and exo-hsa_circ_0012077 in CSF can be used as biomarkers for the diagnosis of immune-mediated demyelinating diseases.

### Correspondence Between exo-circRNA Expression and Clinical Laboratory Data

**Figure 11:**
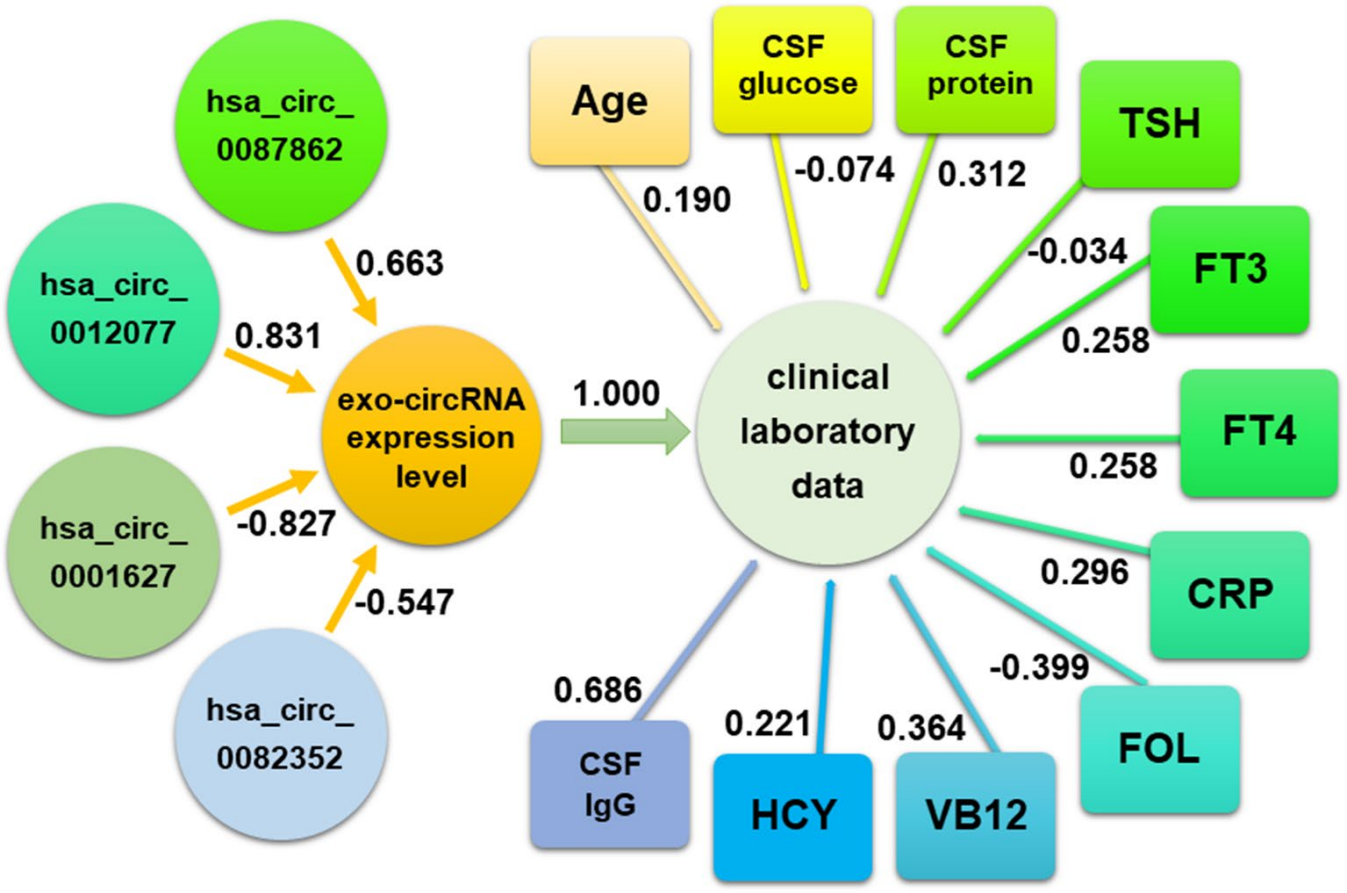
The correlation between exo-circRNA expression and clinical laboratory data was analyzed by canonical correlation analysis. The absolute value of correlation index approaching 1 represents a stronger correlation.

Canonical correlation analysis was performed to further explore the correlation between exo-circRNA expression level and clinical laboratory data in immune-mediated demyelinating disease. Canonical correlation analysis results revealed a strong correlation between clinical laboratory data and the expression levels of hsa_circ_0087862, hsa_circ_0012077, hsa_circ_0001627, and hsa_circ_0082352 (canonical correlation coefficient of 1.000). Among these exo-circRNAs, hsa_circ_0012077 has a great influence on the exo-circRNA expression level (canonical correlation coefficients of 0.831); CSF immunoglobulin G (IgG) levels had a great influence on the clinical laboratory data (canonical correlation coefficient of 0.686). The canonical correlation analysis structural diagram is shown in **Figure 11**.

## Discussion

A highly sensitive biomarker is essential for the early diagnosis and monitoring of immune-mediated demyelinating diseases. By virtue of their stable structure, exo-circRNAs in CSF are a suitable option. The exo-circRNA in CSF can be used as a molecular marker for the diagnosis of immune-mediated demyelinating diseases and reflects the degree of cell damage, metabolic status, and disease progression (Hou et al., 2018). Previous studies have suggested that exosomes are excreted from cells through exocytosis in the process of metabolism; thus, the level of circRNAs in exosomes may reflect the expression level of these circRNAs in cells and the physiological activity of the cells to a certain degree (Hou et al., 2018). Exosomes also represent an efficient way to transfer functional cargo from one cell to another and play biological roles in receptor cells. This action is called cell-to-cell communication or cross-talk between cells. The cross-talk ongoing between neurons and glia, such as microglia and immune cells of the brain in the CNS, mediates intercellular communication over long distances through exosomes in both healthy and diseased brains (Paolicelli et al., 2018).

In the present study, we investigated the exo-circRNA expression profiles *via* a circRNA microarray in the CSF of patients with immune-mediated demyelinating diseases. As a result, 5,095 exo-circRNAs were found to be differentially expressed in CSF exosomes from patients with immune-mediated demyelinating diseases compared with those from paired adjacent CSF samples. Our results revealed the amounts of circRNAs that can be detected in the exosome. Among these exo-circRNAs, 26 were identified as significantly differentially expressed (FC ≥ 1.5, p ≤ 0.05). By analyzing the length distribution of exo-circRNAs, we found that small and large circRNA molecules were harbored in exosomes. These results indicate that exosomes are ideal biological materials that can carry sufficient biomacromolecules and protect them from degradation (Noerholm et al., 2012; Li et al., 2018a). Based on the correspondence between circRNAs and their derived genes, GO and KEGG enrichment analysis were utilized to clarify the primary functions and pathways of the differentially expressed circRNAs. The enrichment analysis indicated that both PTPRF and RAD23B participated in the macromolecule metabolic process, membrane-bounded organelle function, and protein binding, which were the major functions according to the GO analysis of the differentially expressed exo-circRNAs. PTPRF and RAD23B also play roles in CAMs and nucleotide excision repair, respectively, which were the main pathways according to the KEGG enrichment. Furthermore, RAD23B was the derived gene of hsa_circ_0087862 (the most upregulated circRNA) and hsa_circ_0087856 (the third most upregulated circRNA). PTPRF was the derived gene of hsa_circ_0012077 (the second most upregulated circRNA). Previous studies have shown that the homologous gene of PTPRF, protein tyrosine phosphatase sigma, a receptor of chondroitin sulfate proteoglycans, is highly expressed in abnormal astrocytes in amyotrophic lateral sclerosis and inhibits their regeneration (Shijo et al., 2018). PTPRF also exhibited increased expression in injured spinal cord neurons (Lang et al., 2015). The mRNA levels of RAD23B were significantly higher in the brain tissue than in blood samples in neurodegenerative diseases, such as Alzheimer’s disease patients (Jensen et al., 2018). Based on the above findings, we conclude that the upregulation or activation of PTPRF and RAD23B may be associated with the occurrence and development of immune-mediated demyelinating diseases. However, the specific mechanism of PTPRF and RAD23B in immune-mediated demyelinating diseases remains to be confirmed.

The ceRNA hypothesis illustrates the way in which different types of coding and noncoding members of the transcriptome interact with each other *via* miRNAs. We constructed a ceRNA network to predict the functions of the differentially expressed circRNAs. In this study, we focused on the following exo-circRNAs with the most upregulated/downregulated expression levels: hsa_circ_0087862, hsa_circ_0012077, hsa_circ_0001627, and hsa_circ_0082352 indicated by hybridization data analysis. Functional prediction analysis indicated that 60 miRNAs and 552 mRNA of target genes were potentially regulated by these four exo-circRNAs. The predicted ceRNA network showed that the target gene GRIN2B was associated with all four circRNAs. The early expression of GRIN2B in development suggests a role in brain development, circuit formation, synaptic plasticity, and cellular migration and differentiation. Naturally occurring mutations within this gene are associated with neurodevelopmental disorders, including autism spectrum disorder, attention deficit hyperactivity disorder, epilepsy, and schizophrenia. Previous studies have shown that GRIN2B, a target gene of nuclear respiratory factor 1, plays a role in Alzheimer’s disease (Preciados et al., 2016). GO and KEGG analysis of target genes in ceRNA networks showed that GRIN2B is involved in most major biological functions and signaling pathways. These results suggest that GRIN2B may be an important target gene of circRNAs in immune-mediated demyelinating diseases in that it is regulated by circRNAs and can then promote disease.

Because these four exo-circRNAs are involved in the regulation of the expression of multiple genes, we investigated the correlation between their expression level and clinical laboratory data, which are most closely related to the state of immune-mediated demyelinating disease. Canonical correlation analysis can be used to reveal the correlation between two groups of variables and to measure the contribution of each variable to the overall correlation (Yang et al., 2018). Canonical correlation analysis results indicated that the expression levels of these four exo-circRNAs were strongly associated with the clinical laboratory data; especially, there was a strong correlation between the expression level of exo-hsa_circ_0012077 and IgG levels in CSF.

As the major aim of the present study, exo-circRNAs were examined as potential biomarkers for the diagnosis of immune-mediated demyelinating diseases based on their expression levels. The ROC curve results showed that the expression levels of exo-hsa_circ_0087862 or exo-hsa_circ_0012077 in CSF could accurately diagnose immune-mediated demyelinating diseases with a rate of 100%. In addition, RT-qPCR results validated that the expression level of exo-hsa_circ_0087862 and exo-hsa_circ_0012077 increased with statistically significant differences in immune-mediated demyelinating disease samples. These support the idea that exo-hsa_circ_0087862 and exo-hsa_circ_0012077 are promising candidates to be biomarkers for the early diagnosis of immune-mediated demyelinating diseases but still need more validation.

There are a few studies on diagnosing immune-mediated demyelinating diseases using circRNAs as biomarkers. An analysis of circRNAs expression profiles in peripheral blood leukocytes from multiple sclerosis patients and healthy controls showed that hsa_circ_0005402 and hsa_circ_0035560 are downregulated in patients with multiple sclerosis and could be used as biomarkers of the disease (Iparraguirre et al., 2017). However, the composition of leukocytes in blood is complex, and the proportion of cells is affected by many factors. CircRNAs may originate from different cell components, such as lymphocytes and granulocytes. The different proportions of cell components seriously affect the expression level of the circRNAs detected. In contrast, the source of CSF exosomes is relatively simple, which is more suitable for use for the early diagnosis of immune-mediated demyelinating diseases.

The limitation of this study is that the sample size (10 paired samples for hybridization data analysis, 10 more for RT-qPCR validation) is very small for a study involving human subjects, raising the possibility of a sampling bias. The incidence of immune-mediated demyelinating diseases is higher in females than in males, so in this study, we included four female patients and only one male patient. Future studies should be conducted in separate cohorts of subjects to replicate these results. Because of ethical limitations, we are not allowed to collect CSF lumbar puncture in a completely healthy individual. Therefore, another limitation of this study is using subjects with “abnormal intracranial pressure” as a control group. It is possible that using healthy controls would produce different results.

## Conclusion

In the present study, we investigated the exo-circRNA expression profiles in CSF of patients with immune-mediated demyelinating diseases. We found that exo-hsa_circ_0087862 and exo-hsa_circ_0012077 in CSF could be suitable biomarkers for the diagnosis of immune-mediated demyelinating diseases based on their expression levels. The expression levels of exo-circRNAs were strongly associated with the clinical laboratory data. The upregulation or activation of PTPRF and RAD23B was potentially associated with the occurrence and development of immune-mediated demyelinating disease. GRIN2B is suggested as an important target gene of circRNAs in immune-mediated demyelinating diseases because it is regulated by circRNAs and promotes disease. However, the specific mechanism remains to be confirmed.

## Data Availability

The raw data supporting the conclusions of this manuscript will be made available by the authors, without undue reservation, to any qualified researcher.

## Ethics Statement

The Ethics Committee of the China-Japan Union Hospital of Jilin University has a detailed understanding of all experimental protocols and approved all protocols used in this study. All involved methods were carried out in accordance with relevant guidelines and regulations of the Ethics Committee of the China-Japan Union Hospital of Jilin University. We informed all participants according to the informed consent for the use of their specimens, and written informed consent was obtained from each participant. The study conforms with The Code of Ethics of the World Medical Association (Declaration of Helsinki), printed in the British Medical Journal (18 July 1964).

## Author Contributions

All authors participated in caring for the patients. JH was responsible for collecting clinical data and critically revising the manuscript. HL and XW were responsible for collecting clinical data. LY was responsible for constructing and organizing pictures. MR was responsible for constructing and organizing pictures. QY was responsible for the intellectual content of the report, organizing pictures, and drafting the manuscript.

## Funding

The work was supported by grants from the Science and Technology Department of Jilin Province, P.R.C. (No. 20180520111JH); Jilin Provincial Department of Health funds (No.20152085); the Education Department of Jilin Province, P.R.C. (No. JJKH20190058KJ); Special Project of Medical and Health Talents in Jilin Province (2019SRCJ002); Bethune Project of Jilin University (2018B04); and the Second Hospital of Jilin University, P.R.C. (No. KYPY2018-02).

## Conflict of Interest Statement

The authors declare that the research was conducted in the absence of any commercial or financial relationships that could be construed as a potential conflict of interest.

## Abbreviations

MS, multiple sclerosis; GBS, Guillain-Barré syndrome; CSF, cerebrospinal fluid; circRNA, circular RNA; exo-circRNA, exosomal circRNA; CNS, central nervous system; TEM, transmission electron microscopy; FC, fold change; GO, Gene Ontology; KEGG, Kyoto Encyclopedia of Genes and Genomes; SD, standard deviation; ROC, receiver operating characteristic; PTPRF, protein tyrosine phosphatase receptor type F; RAD23B, RAD23 homolog B, nucleotide excision repair protein; CAMs, cell adhesion molecules; ceRNA, competing endogenous RNA; GRIN2B, glutamate ionotropic receptor NMDA type subunit 2B.

## Supplementary Material

Supplementary Figure 1 Microarray slide scanning images of exosomal circRNAs.

Supplementary Table 1 Information on differentially expressed circRNAs.

Supplementary Table 2 GO analysis on 25 parental genes of circRNAs.

Supplementary Table 3 KEGG enrichment on main pathways.

Supplementary Table 4 Clinical information and laboratory data of the patients/control samples employed for RT-qPCR validation.

Supplementary Table 5 GO analysis on potential target genes that may be regulated by differentially expressed circRNAs.

Supplementary Table 6 KEGG enrichment on potential target genes that may be regulated by differentially expressed circRNAs.
