# Supplementary figures and images for: Exosomal Circular RNA as a Biomarker Platform for the Early Diagnosis of Immune-Mediated Demyelinating Disease
Source: Front Genet. 2019 Sep 27;10:860. doi: 10.3389/fgene.2019.00860 (PMC6777646; doi:10.3389/fgene.2019.00860)

Patient 1

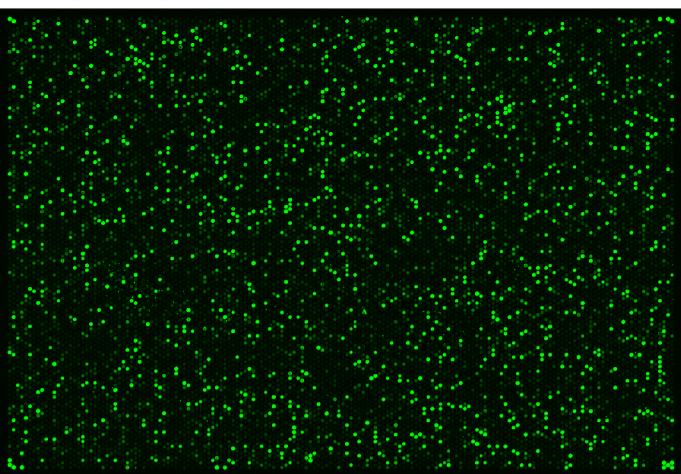

Control 1

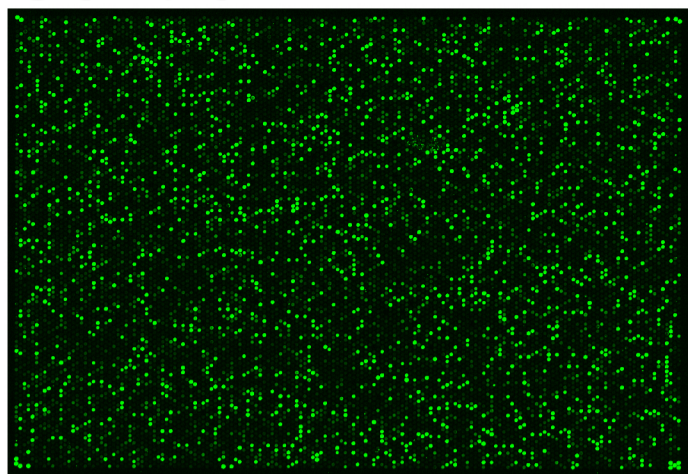

Patient 2

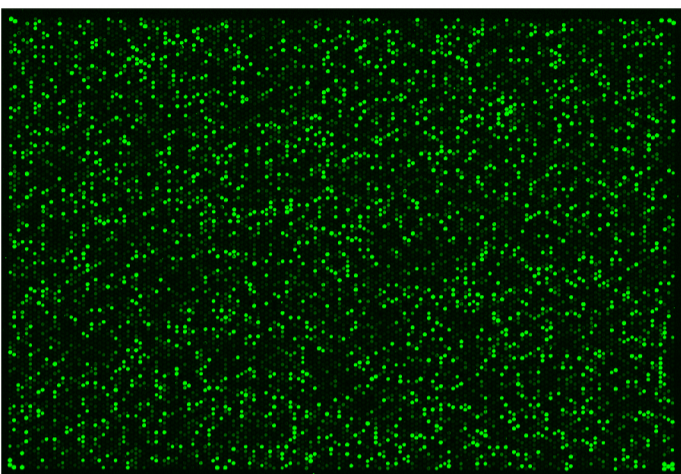

Control 2

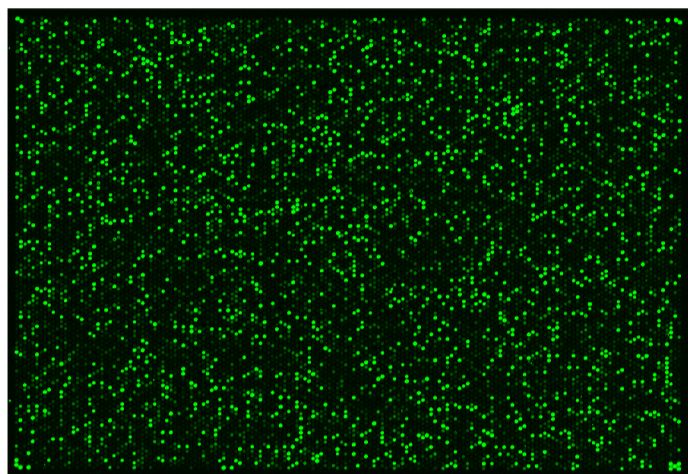

Patient 3

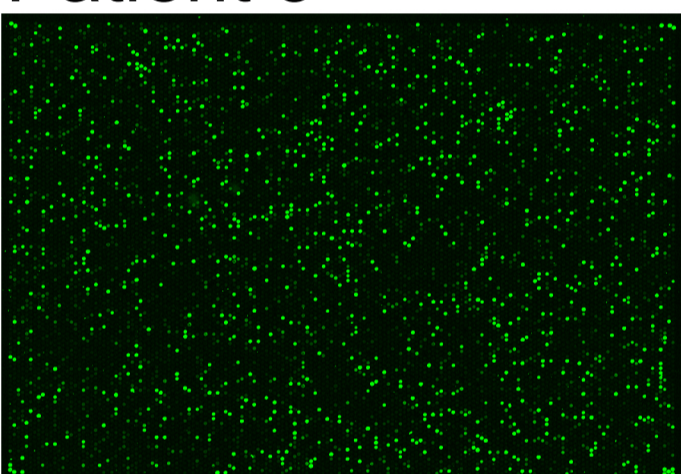

Control 3

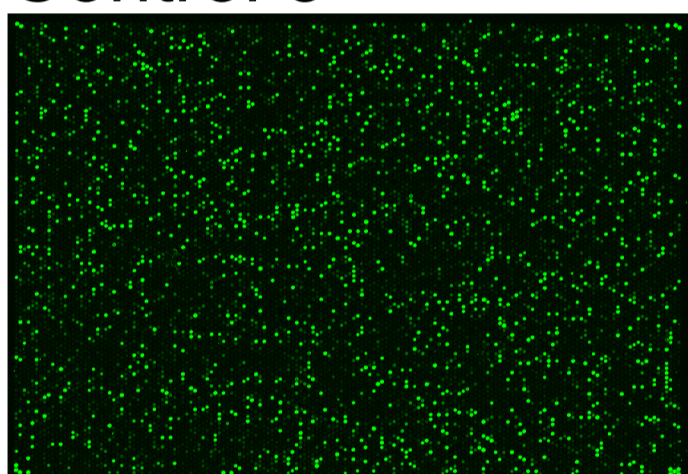

Patient 4

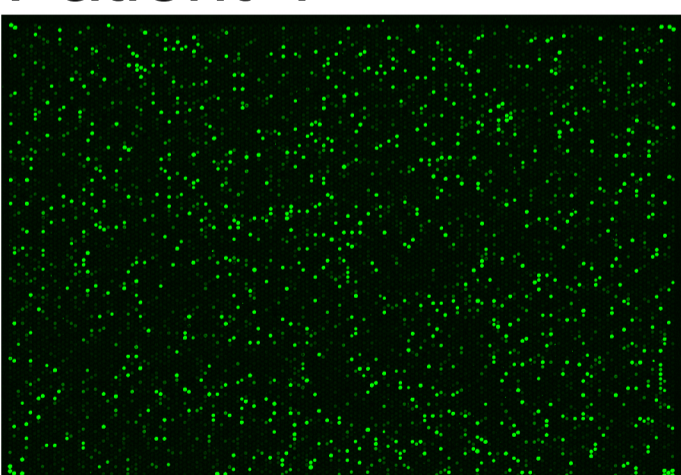

Control 4

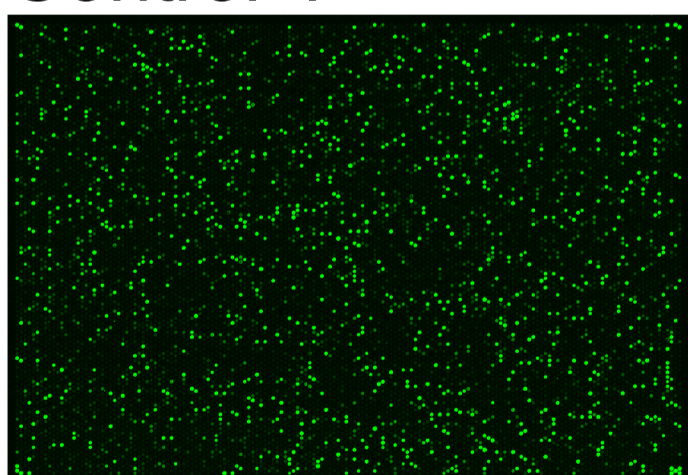

Patient 5

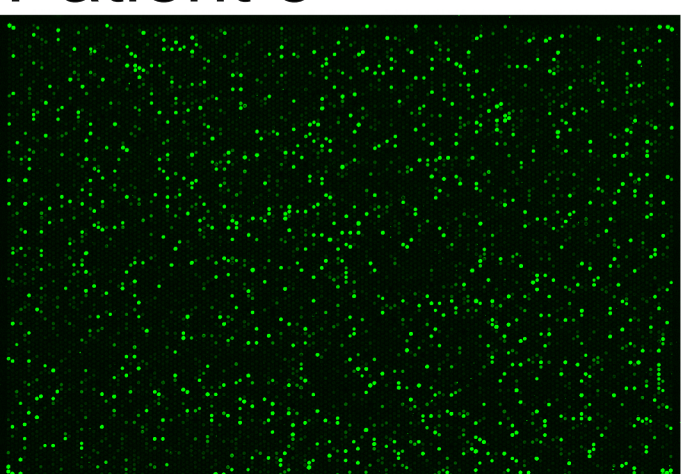

Control 5

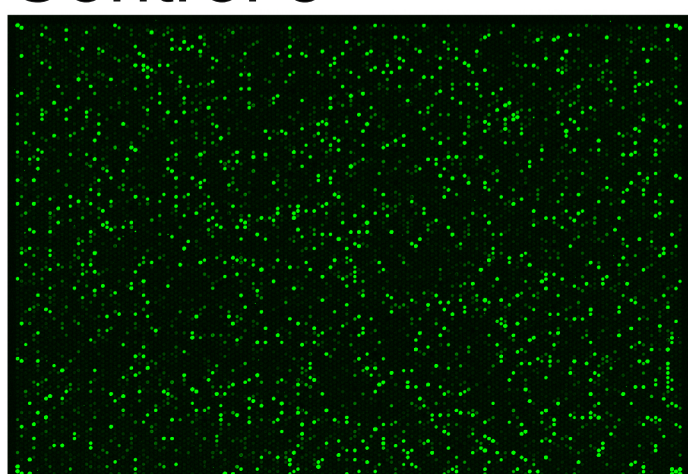

Supplement: Supplementary Figure 1 — Microarray slide scanning images of exosomal circRNAs. [file Image_1.pdf]
