# Supplementary material for: Exosomal Circular RNA as a Biomarker Platform for the Early Diagnosis of Immune-Mediated Demyelinating Disease
Source: Front Genet. 2019 Sep 27;10:860. doi: 10.3389/fgene.2019.00860 (PMC6777646; doi:10.3389/fgene.2019.00860)
Supplement: Supplementary Table 2 — GO analysis on 25 parental genes of circRNAs. [file Table_2.pdf]

Supplementary Table 2

ID	Biological Process	Cell Component	Molecular Function
PTPRF	GO:0006470,protein dephosphorylation|GO:0048679,regulation of axon regeneration|GO:0048678,response to axon injury|GO:0007165,signal transduction|GO:0007166,cell surface receptor signaling pathway|GO:0007167,enzyme linked receptor protein signaling pathway|GO:0031344,regulation of cell projection organization|GO:0071840,cellular component organization or biogenesis|GO:0051716,cellular response to stimulus|GO:0048869,cellular developmental process|GO:0009611,response to wounding|GO:0045664,regulation of neuron differentiation|GO:0044092,negative regulation of molecular function|GO:0031103,axon regeneration|GO:0031102,neuron projection regeneration|GO:0048583,regulation of response to stimulus|GO:0010975,regulation of neuron projection development|GO:0044700,single organism signaling|GO:0009605,response to external stimulus|GO:0044707,single-multicellular organism process|GO:0048870,cell motility|GO:0019538,protein metabolic process|GO:0033554,cellular response to stress|GO:0051100,negative regulation of binding|GO:0006928,movement of cell or subcellular component|GO:0051674,localization of cell|GO:0031175,neuron projection development|GO:0050789,regulation of biological process|GO:0044267,cellular protein metabolic process|GO:0044260,cellular macromolecule metabolic process|GO:0016043,cellular component organization|GO:0065007,biological regulation|GO:0065009,regulation of molecular function|GO:0016477,cell migration|GO:0007185,transmembrane receptor protein tyrosine phosphatase signaling pathway|GO:0061564,axon development|GO:0050793,regulation of developmental process|GO:0048468,cell development|GO:0050794,regulation of cellular process|GO:0043412,macromolecule modification|GO:0036211,protein modification process|GO:0043393,regulation of protein binding|GO:0051239,regulation of multicellular organismal process|GO:0080134,regulation of response to stress|GO:0080135,regulation of cellular response to stress|GO:0006950,response to stress|GO:0051960,regulation of nervous system development|GO:0008152,metabolic process|GO:0032101,regulation of response to external stimulus|GO:0016311,dephosphorylation|GO:0030154,cell differentiation|GO:0051128,regulation of cellular component organization|GO:0023052,signaling|GO:0035335,peptidyl-tyrosine dephosphorylation|GO:0044699,single-organism process|GO:0050767,regulation of neurogenesis|GO:0031099,regeneration|GO:0060284,regulation of cell development|GO:0070570,regulation of neuron projection regeneration|GO:0022610,biological adhesion|GO:1903034,regulation of response to wounding|GO:0032502,developmental process|GO:0032501,multicellular organismal process|GO:0009987,cellular process|GO:0045595,regulation of cell differentiation|GO:0051098,regulation of binding|GO:0043170,macromolecule metabolic process|GO:1900121,negative regulation of receptor binding|GO:1900120,regulation of receptor binding|GO:0048731,system development|GO:0050896,response to stimulus|GO:0030030,cell projection organization|GO:0007275,multicellular organism development|GO:0071704,organic substance metabolic process|GO:0048666,neuron development|GO:0030182,neuron differentiation|GO:0032091,negative regulation of protein binding|GO:0006464,cellular protein modification process|GO:0044767,single-organism developmental process|GO:0044763,single-organism cellular process|GO:0007155,cell adhesion|GO:0007154,cell communication|GO:0022008,neurogenesis|GO:0051179,localization|GO:0040011,locomotion|GO:0044238,primary metabolic process|GO:0048699,generation of neurons|GO:0007399,nervous system development|GO:0048856,anatomical structure development|GO:0044237,cellular metabolic process|GO:0006796,phosphate-containing compound metabolic process|GO:2000026,regulation of multicellular organismal development|GO:0006793,phosphorus metabolic process|GO:1901564,organonitrogen compound metabolic process|GO:0006807,nitrogen compound metabolic process	GO:0071944,cell periphery|GO:0031226,intrinsic component of plasma membrane|GO:0043226,organelle|GO:0031224,intrinsic component of membrane|GO:1903561,extracellular vesicle|GO:0031982,vesicle|GO:0043227,membrane-bounded organelle|GO:0016021,integral component of membrane|GO:0016020,membrane|GO:0031988,membrane-bounded vesicle|GO:0097458,neuron part|GO:0043005,neuron projection|GO:0044459,plasma membrane part|GO:0044297,cell body|GO:0036477,somatodendritic compartment|GO:0005887,integral component of plasma membrane|GO:0005886,plasma membrane|GO:0042995,cell projection|GO:0070062,extracellular exosome|GO:0043230,extracellular organelle|GO:0043025,neuronal cell body|GO:0044464,cell part|GO:0005623,cell|GO:0005576,extracellular region|GO:0044425,membrane part|GO:0065010,extracellular membrane-bounded organelle|GO:0044421,extracellular region part	GO:0038023,signaling receptor activity|GO:0035373,chondroitin sulfate proteoglycan binding|GO:0042578,phosphoric ester hydrolase activity|GO:0060089,molecular transducer activity|GO:0044877,macromolecular complex binding|GO:0004872,receptor activity|GO:0004871,signal transducer activity|GO:0016791,phosphatase activity|GO:0001948,glycoprotein binding|GO:1901681,sulfur compound binding|GO:0005539,glycosaminoglycan binding|GO:0043167,ion binding|GO:0032403,protein complex binding|GO:0016787,hydrolase activity|GO:0016788,hydrolase activity, acting on ester bonds|GO:0003824,catalytic activity|GO:0043394,proteoglycan binding|GO:0008201,heparin binding|GO:0097367,carbohydrate derivative binding|GO:0004725,protein tyrosine phosphatase activity|GO:0004721,phosphoprotein phosphatase activity|GO:0043168,anion binding|GO:0005001,transmembrane receptor protein tyrosine phosphatase activity|GO:0004888,transmembrane signaling receptor activity|GO:0005515,protein binding|GO:0019198,transmembrane receptor protein phosphatase activity|GO:0005488,binding|GO:0099600,transmembrane receptor activity|GO:0050839,cell adhesion molecule binding
PALD1	GO:0071704,organic substance metabolic process|GO:0044267,cellular protein metabolic process|GO:1901564,organonitrogen compound metabolic process|GO:0044260,cellular macromolecule metabolic process|GO:0044238,primary metabolic process|GO:0016311,dephosphorylation|GO:0006470,protein dephosphorylation|GO:0009987,cellular process|GO:0043412,macromolecule modification|GO:0006464,cellular protein modification process|GO:0043170,macromolecule metabolic process|GO:0019538,protein metabolic process|GO:0006796,phosphate-containing compound metabolic process|GO:0036211,protein modification process|GO:0006807,nitrogen compound metabolic process|GO:0044237,cellular metabolic process|GO:0008152,metabolic process|GO:0006793,phosphorus metabolic process|GO:0035335,peptidyl-tyrosine dephosphorylation	GO:0005737,cytoplasm|GO:0005829,cytosol|GO:0044464,cell part|GO:0005623,cell|GO:0005622,intracellular|GO:0044444,cytoplasmic part|GO:0044424,intracellular part|GO:0043231,intracellular membrane-bounded organelle|GO:0043226,organelle|GO:0043229,intracellular organelle|GO:0043227,membrane-bounded organelle|GO:0005634,nucleus	GO:0005488,binding|GO:0005515,protein binding|GO:0016787,hydrolase activity|GO:0016791,phosphatase activity|GO:0008138,protein tyrosine/serine/threonine phosphatase activity|GO:0004725,protein tyrosine phosphatase activity|GO:0004722,protein serine/threonine phosphatase activity|GO:0004721,phosphoprotein phosphatase activity|GO:0042578,phosphoric ester hydrolase activity|GO:0003824,catalytic activity|GO:0016788,hydrolase activity, acting on ester bonds
KRT5	GO:0032502,developmental process|GO:0022607,cellular component assembly|GO:0048856,anatomical structure development|GO:0060429,epithelium development|GO:0031581,hemidesmosome assembly|GO:0034330,cell junction organization|GO:0009888,tissue development|GO:0016043,cellular component organization|GO:0007044,cell-substrate junction assembly|GO:0044763,single-organism cellular process|GO:0071840,cellular component organization or biogenesis|GO:0009987,cellular process|GO:0008544,epidermis development|GO:0034329,cell junction assembly|GO:0044699,single-organism process|GO:0044085,cellular component biogenesis|GO:0008219,cell death|GO:0007275,multicellular organism development|GO:0032501,multicellular organismal process|GO:0048869,cellular developmental process|GO:0048513,animal organ development|GO:0030855,epithelial cell differentiation|GO:0043588,skin development|GO:0030154,cell differentiation|GO:0030216,keratinocyte differentiation|GO:0070268,cornification|GO:0044767,single-organism developmental process|GO:0012501,programmed cell death|GO:0006996,organelle organization|GO:0007010,cytoskeleton organization|GO:0044707,single-multicellular organism process|GO:0031424,keratinization|GO:0009913,epidermal cell differentiation|GO:0048731,system development	GO:0045095,keratin filament|GO:0043229,intracellular organelle|GO:0043228,non-membrane-bounded organelle|GO:0043227,membrane-bounded organelle|GO:0043226,organelle|GO:0005856,cytoskeleton|GO:0045111,intermediate filament cytoskeleton|GO:0031982,vesicle|GO:0005634,nucleus|GO:0016020,membrane|GO:0005739,mitochondrion|GO:0031988,membrane-bounded vesicle|GO:0044430,cytoskeletal part|GO:0005576,extracellular region|GO:0005737,cytoplasm|GO:0005882,intermediate filament|GO:0005886,plasma membrane|GO:0032991,macromolecular complex|GO:1903561,extracellular vesicle|GO:0070062,extracellular exosome|GO:0043234,protein complex|GO:0043230,extracellular organelle|GO:0043231,intracellular membrane-bounded organelle|GO:0043232,intracellular non-membrane-bounded organelle|GO:0005829,cytosol|GO:0044464,cell part|GO:0005623,cell|GO:0005622,intracellular|GO:0044446,intracellular organelle part|GO:0044444,cytoplasmic part|GO:0071944,cell periphery|GO:0044424,intracellular part|GO:0065010,extracellular membrane-bounded organelle|GO:0044421,extracellular region part|GO:0044422,organelle part|GO:0099512,supramolecular fiber|GO:0099513,polymeric cytoskeletal fiber	GO:0005200,structural constituent of cytoskeleton|GO:0005198,structural molecule activity|GO:0005488,binding|GO:0005515,protein binding|GO:0097110,scaffold protein binding
COL1A2	GO:0007599,hemostasis|GO:0007596,blood coagulation|GO:0001501,skeletal system development|GO:0072359,circulatory system development|GO:0072358,cardiovascular system development|GO:0007165,signal transduction|GO:0007166,cell surface receptor signaling pathway|GO:0007167,enzyme linked receptor protein signaling pathway|GO:0071840,cellular component organization or biogenesis|GO:0044712,single-organism catabolic process|GO:0043200,response to amino acid|GO:0044710,single-organism metabolic process|GO:0009611,response to wounding|GO:0048513,animal organ development|GO:0007179,transforming growth factor beta receptor signaling pathway|GO:0007178,transmembrane receptor protein serine/threonine kinase signaling pathway|GO:0051291,protein heterooligomerization|GO:0042221,response to chemical|GO:0010033,response to organic substance|GO:0030168,platelet activation|GO:0003008,system process|GO:0044700,single organism signaling|GO:0065008,regulation of biological quality|GO:0016192,vesicle-mediated transport|GO:0044707,single-multicellular organism process|GO:0048870,cell motility|GO:0010243,response to organonitrogen compound|GO:0002376,immune system process|GO:0030199,collagen fibril organization|GO:0030198,extracellular matrix organization|GO:0003013,circulatory system process|GO:0022607,cellular component assembly|GO:0006950,response to stress|GO:0006928,movement of cell or subcellular component|GO:0051674,localization of cell|GO:0035556,intracellular signal transduction|GO:0050789,regulation of biological process|GO:0009653,anatomical structure morphogenesis|GO:0001568,blood vessel development|GO:0007266,Rho protein signal transduction|GO:0016043,cellular component organization|GO:0065003,macromolecular complex assembly|GO:0065007,biological regulation|GO:0016477,cell migration|GO:0009887,animal organ morphogenesis|GO:0006810,transport|GO:0070206,protein trimerization|GO:0008015,blood circulation|GO:0070208,protein heterotrimerization|GO:0051716,cellular response to stimulus|GO:0042060,wound healing|GO:0050794,regulation of cellular process|GO:0001775,cell activation|GO:0050817,coagulation|GO:0008152,metabolic process|GO:0051234,establishment of localization|GO:0006897,endocytosis|GO:1901698,response to nitrogen compound|GO:1901699,cellular response to nitrogen compound|GO:0030574,collagen catabolic process|GO:0044763,single-organism cellular process|GO:0070271,protein complex biogenesis|GO:0070848,response to growth factor|GO:0023052,signaling|GO:0070887,cellular response to chemical stimulus|GO:0032963,collagen metabolic process|GO:0044243,multicellular organismal catabolic process|GO:0044699,single-organism process|GO:0009719,response to endogenous stimulus|GO:0001944,vasculature development|GO:0022617,extracellular matrix disassembly|GO:0032502,developmental process|GO:1901700,response to oxygen-containing compound|GO:0032501,multicellular organismal process|GO:0050878,regulation of body fluid levels|GO:0009987,cellular process|GO:0001101,response to acid chemical|GO:0044259,multicellular organismal macromolecule metabolic process|GO:0051259,protein oligomerization|GO:0071363,cellular response to growth factor stimulus|GO:0043170,macromolecule metabolic process|GO:0071560,cellular response to transforming growth factor beta stimulus|GO:0048731,system development|GO:0050896,response to stimulus|GO:0071495,cellular response to endogenous stimulus|GO:0006898,receptor-mediated endocytosis|GO:0043933,macromolecular complex subunit organization|GO:0071229,cellular response to acid chemical|GO:0050900,leukocyte migration|GO:0008217,regulation of blood pressure|GO:0007275,multicellular organism development|GO:0071822,protein complex subunit organization|GO:0071417,cellular response to organonitrogen compound|GO:0071704,organic substance metabolic process|GO:0071310,cellular response to organic substance|GO:0043062,extracellular structure organization|GO:0071559,response to transforming growth factor beta|GO:0043588,skin development|GO:0043589,skin morphogenesis|GO:0071230,cellular response to amino acid stimulus|GO:0006461,protein complex assembly|GO:0044767,single-organism developmental process|GO:0022411,cellular component disassembly|GO:0007154,cell communication|GO:0007265,Ras protein signal transduction|GO:0007264,small GTPase mediated signal transduction|GO:0009056,catabolic process|GO:0051179,localization|GO:0040011,locomotion|GO:1901701,cellular response to oxygen-containing compound|GO:0042476,odontogenesis|GO:0048856,anatomical structure development|GO:0044236,multicellular organism metabolic process|GO:0044085,cellular component biogenesis|GO:0048583,regulation of response to stimulus|GO:0002682,regulation of immune system process|GO:0006955,immune response|GO:0097435,fibril organization|GO:0050776,regulation of immune response|GO:0071345,cellular response to cytokine stimulus|GO:0019221,cytokine-mediated signaling pathway|GO:0034097,response to cytokine	GO:0005783,endoplasmic reticulum|GO:0044421,extracellular region part|GO:0031974,membrane-enclosed lumen|GO:0031012,extracellular matrix|GO:0043229,intracellular organelle|GO:0043227,membrane-bounded organelle|GO:0043226,organelle|GO:0005584,collagen type I trimer|GO:0070062,extracellular exosome|GO:0005583,fibrillar collagen trimer|GO:0005615,extracellular space|GO:0031988,membrane-bounded vesicle|GO:0044432,endoplasmic reticulum part|GO:0098644,complex of collagen trimers|GO:0098643,banded collagen fibril|GO:0005737,cytoplasm|GO:0005788,endoplasmic reticulum lumen|GO:0032991,macromolecular complex|GO:1903561,extracellular vesicle|GO:0031982,vesicle|GO:0043234,protein complex|GO:0043230,extracellular organelle|GO:0043231,intracellular membrane-bounded organelle|GO:0043233,organelle lumen|GO:0044420,extracellular matrix component|GO:0044464,cell part|GO:0005623,cell|GO:0005622,intracellular|GO:0044446,intracellular organelle part|GO:0070013,intracellular organelle lumen|GO:0044444,cytoplasmic part|GO:0005576,extracellular region|GO:0005581,collagen trimer|GO:0044424,intracellular part|GO:0065010,extracellular membrane-bounded organelle|GO:0005578,proteinaceous extracellular matrix|GO:0044422,organelle part|GO:0012505,endomembrane system	GO:0043169,cation binding|GO:0005201,extracellular matrix structural constituent|GO:0005198,structural molecule activity|GO:0030674,protein binding, bridging|GO:0043167,ion binding|GO:0046332,SMAD binding|GO:0019838,growth factor binding|GO:0005488,binding|GO:0005515,protein binding|GO:0048407,platelet-derived growth factor binding|GO:0046872,metal ion binding|GO:0042802,identical protein binding|GO:0060090,binding, bridging|GO:0019899,enzyme binding|GO:0002020,protease binding|GO:0030020,extracellular matrix structural constituent conferring tensile strength
RAD23B	GO:0009894,regulation of catabolic process|GO:0044238,primary metabolic process|GO:0080090,regulation of primary metabolic process|GO:0031329,regulation of cellular catabolic process|GO:0006950,response to stress|GO:0031323,regulation of cellular metabolic process|GO:0019953,sexual reproduction|GO:0090304,nucleic acid metabolic process|GO:0042176,regulation of protein catabolic process|GO:0006807,nitrogen compound metabolic process|GO:1901360,organic cyclic compound metabolic process|GO:0006725,cellular aromatic compound metabolic process|GO:0043632,modification-dependent macromolecule catabolic process|GO:0043170,macromolecule metabolic process|GO:0050789,regulation of biological process|GO:0007276,gamete generation|GO:0044349,DNA excision|GO:0006139,nucleobase-containing compound metabolic process|GO:0044710,single-organism metabolic process|GO:0044265,cellular macromolecule catabolic process|GO:0032504,multicellular organism reproduction|GO:0019222,regulation of metabolic process|GO:0044260,cellular macromolecule metabolic process|GO:0043161,proteasome-mediated ubiquitin-dependent protein catabolic process|GO:0071840,cellular component organization or biogenesis|GO:0044703,multi-organism reproductive process|GO:0051246,regulation of protein metabolic process|GO:0016043,cellular component organization|GO:0009057,macromolecule catabolic process|GO:0071704,organic substance metabolic process|GO:0065007,biological regulation|GO:0044699,single-organism process|GO:1901575,organic substance catabolic process|GO:0044267,cellular protein metabolic process|GO:0051704,multi-organism process|GO:0046483,heterocycle metabolic process|GO:0032501,multicellular organismal process|GO:0048609,multicellular organismal reproductive process|GO:1903362,regulation of cellular protein catabolic process|GO:0006281,DNA repair|GO:0060255,regulation of macromolecule metabolic process|GO:0061136,regulation of proteasomal protein catabolic process|GO:1903050,regulation of proteolysis involved in cellular protein catabolic process|GO:0009987,cellular process|GO:0019941,modification-dependent protein catabolic process|GO:0051716,cellular response to stimulus|GO:0006289,nucleotide-excision repair|GO:0006974,cellular response to DNA damage stimulus|GO:0022414,reproductive process|GO:0007283,spermatogenesis|GO:0044702,single organism reproductive process|GO:0030162,regulation of proteolysis|GO:0030163,protein catabolic process|GO:0008152,metabolic process|GO:0048232,male gamete generation|GO:0044257,cellular protein catabolic process|GO:0032434,regulation of proteasomal ubiquitin-dependent protein catabolic process|GO:0050794,regulation of cellular process|GO:0009056,catabolic process|GO:0006508,proteolysis|GO:0044248,cellular catabolic process|GO:0000003,reproduction|GO:0006996,organelle organization|GO:0051603,proteolysis involved in cellular protein catabolic process|GO:0000718,nucleotide-excision repair, DNA damage removal|GO:0032268,regulation of cellular protein metabolic process|GO:0051276,chromosome organization|GO:0019538,protein metabolic process|GO:0050896,response to stimulus|GO:0010498,proteasomal protein catabolic process|GO:0034641,cellular nitrogen compound metabolic process|GO:0044237,cellular metabolic process|GO:0000715,nucleotide-excision repair, DNA damage recognition|GO:0006511,ubiquitin-dependent protein catabolic process|GO:0033554,cellular response to stress|GO:0006259,DNA metabolic process|GO:0044763,single-organism cellular process|GO:0070647,protein modification by small protein conjugation or removal|GO:0070646,protein modification by small protein removal|GO:0048513,animal organ development|GO:0032392,DNA geometric change|GO:1901564,organonitrogen compound metabolic process|GO:1901565,organonitrogen compound catabolic process|GO:0044707,single-multicellular organism process|GO:0071103,DNA conformation change|GO:0006294,nucleotide-excision repair, preincision complex assembly|GO:0022607,cellular component assembly|GO:0006457,protein folding|GO:0048568,embryonic organ development|GO:0000717,nucleotide-excision repair, DNA duplex unwinding|GO:0070911,global genome nucleotide-excision repair|GO:0065003,macromolecular complex assembly|GO:0065004,protein-DNA complex assembly|GO:0016579,protein deubiquitination|GO:0043412,macromolecule modification|GO:0036211,protein modification process|GO:0009790,embryo development|GO:0032502,developmental process|GO:0032508,DNA duplex unwinding|GO:0048731,system development|GO:0043933,macromolecular complex subunit organization|GO:0034622,cellular macromolecular complex assembly|GO:0007275,multicellular organism development|GO:0071824,protein-DNA complex subunit organization|GO:0006464,cellular protein modification process|GO:0044767,single-organism developmental process|GO:0048856,anatomical structure development|GO:0044085,cellular component biogenesis	GO:0031974,membrane-enclosed lumen|GO:0043229,intracellular organelle|GO:0071942,XPC complex|GO:0000109,nucleotide-excision repair complex|GO:0043227,membrane-bounded organelle|GO:0043226,organelle|GO:0005737,cytoplasm|GO:0031981,nuclear lumen|GO:0005634,nucleus|GO:0005654,nucleoplasm|GO:0000502,proteasome complex|GO:0043234,protein complex|GO:0032991,macromolecular complex|GO:0043231,intracellular membrane-bounded organelle|GO:0043233,organelle lumen|GO:1990391,DNA repair complex|GO:0044464,cell part|GO:0005623,cell|GO:0005622,intracellular|GO:0044446,intracellular organelle part|GO:0070013,intracellular organelle lumen|GO:0044428,nuclear part|GO:0044424,intracellular part|GO:0044422,organelle part|GO:0005829,cytosol|GO:0044444,cytoplasmic part	GO:0043566,structure-specific DNA binding|GO:0005515,protein binding|GO:0003684,damaged DNA binding|GO:0097159,organic cyclic compound binding|GO:0031593,polyubiquitin binding|GO:0032182,ubiquitin-like protein binding|GO:0003697,single-stranded DNA binding|GO:0043130,ubiquitin binding|GO:0005488,binding|GO:0003676,nucleic acid binding|GO:0003677,DNA binding|GO:1901363,heterocyclic compound binding
XLOC_005768			
FOXN3	GO:0080090,regulation of primary metabolic process|GO:0019222,regulation of metabolic process|GO:0031326,regulation of cellular biosynthetic process|GO:0031323,regulation of cellular metabolic process|GO:0090304,nucleic acid metabolic process|GO:0044249,cellular biosynthetic process|GO:0034641,cellular nitrogen compound metabolic process|GO:0006807,nitrogen compound metabolic process|GO:0034645,cellular macromolecule biosynthetic process|GO:1901362,organic cyclic compound biosynthetic process|GO:0050789,regulation of biological process|GO:0097659,nucleic acid-templated transcription|GO:0032774,RNA biosynthetic process|GO:0006139,nucleobase-containing compound metabolic process|GO:0044260,cellular macromolecule metabolic process|GO:0071704,organic substance metabolic process|GO:2000112,regulation of cellular macromolecule biosynthetic process|GO:0060255,regulation of macromolecule metabolic process|GO:0010467,gene expression|GO:0065007,biological regulation|GO:1901360,organic cyclic compound metabolic process|GO:0010468,regulation of gene expression|GO:0018130,heterocycle biosynthetic process|GO:1901576,organic substance biosynthetic process|GO:0019219,regulation of nucleobase-containing compound metabolic process|GO:0006725,cellular aromatic compound metabolic process|GO:0009987,cellular process|GO:0009889,regulation of biosynthetic process|GO:1903506,regulation of nucleic acid-templated transcription|GO:0050794,regulation of cellular process|GO:0009058,biosynthetic process|GO:0009059,macromolecule biosynthetic process|GO:0051171,regulation of nitrogen compound metabolic process|GO:0008152,metabolic process|GO:2001141,regulation of RNA biosynthetic process|GO:0034654,nucleobase-containing compound biosynthetic process|GO:0046483,heterocycle metabolic process|GO:0016070,RNA metabolic process|GO:0044238,primary metabolic process|GO:0044271,cellular nitrogen compound biosynthetic process|GO:0051252,regulation of RNA metabolic process|GO:0044237,cellular metabolic process|GO:0043170,macromolecule metabolic process|GO:0006355,regulation of transcription, DNA-templated|GO:0010556,regulation of macromolecule biosynthetic process|GO:0006351,transcription, DNA-templated|GO:0019438,aromatic compound biosynthetic process	GO:0043231,intracellular membrane-bounded organelle|GO:0005634,nucleus|GO:0044464,cell part|GO:0005623,cell|GO:0005622,intracellular|GO:0043229,intracellular organelle|GO:0044424,intracellular part|GO:0043227,membrane-bounded organelle|GO:0043226,organelle	GO:0043565,sequence-specific DNA binding|GO:0003700,transcription factor activity, sequence-specific DNA binding|GO:0097159,organic cyclic compound binding|GO:0005488,binding|GO:0003676,nucleic acid binding|GO:0003677,DNA binding|GO:1901363,heterocyclic compound binding|GO:0001071,nucleic acid binding transcription factor activity
GeneSymbol			
BACH2	GO:0009892,negative regulation of metabolic process|GO:0080090,regulation of primary metabolic process|GO:0009890,negative regulation of biosynthetic process|GO:0031327,negative regulation of cellular biosynthetic process|GO:0031326,regulation of cellular biosynthetic process|GO:0031324,negative regulation of cellular metabolic process|GO:0031323,regulation of cellular metabolic process|GO:0090304,nucleic acid metabolic process|GO:0044249,cellular biosynthetic process|GO:0010629,negative regulation of gene expression|GO:0006807,nitrogen compound metabolic process|GO:0034645,cellular macromolecule biosynthetic process|GO:1901362,organic cyclic compound biosynthetic process|GO:0050789,regulation of biological process|GO:0097659,nucleic acid-templated transcription|GO:0032774,RNA biosynthetic process|GO:0006139,nucleobase-containing compound metabolic process|GO:0010605,negative regulation of macromolecule metabolic process|GO:0019222,regulation of metabolic process|GO:0044260,cellular macromolecule metabolic process|GO:0000122,negative regulation of transcription from RNA polymerase II promoter|GO:2000112,regulation of cellular macromolecule biosynthetic process|GO:2000113,negative regulation of cellular macromolecule biosynthetic process|GO:0060255,regulation of macromolecule metabolic process|GO:0010467,gene expression|GO:0010556,regulation of macromolecule biosynthetic process|GO:0065007,biological regulation|GO:1901360,organic cyclic compound metabolic process|GO:0006366,transcription from RNA polymerase II promoter|GO:0048519,negative regulation of biological process|GO:0010468,regulation of gene expression|GO:0018130,heterocycle biosynthetic process|GO:0045934,negative regulation of nucleobase-containing compound metabolic process|GO:1901576,organic substance biosynthetic process|GO:0019219,regulation of nucleobase-containing compound metabolic process|GO:0006725,cellular aromatic compound metabolic process|GO:0009987,cellular process|GO:0009889,regulation of biosynthetic process|GO:1903506,regulation of nucleic acid-templated transcription|GO:1903507,negative regulation of nucleic acid-templated transcription|GO:1902679,negative regulation of RNA biosynthetic process|GO:0071704,organic substance metabolic process|GO:0009058,biosynthetic process|GO:0009059,macromolecule biosynthetic process|GO:0045892,negative regulation of transcription, DNA-templated|GO:0051171,regulation of nitrogen compound metabolic process|GO:0051172,negative regulation of nitrogen compound metabolic process|GO:2001141,regulation of RNA biosynthetic process|GO:0034654,nucleobase-containing compound biosynthetic process|GO:0050794,regulation of cellular process|GO:0008152,metabolic process|GO:0046483,heterocycle metabolic process|GO:0016070,RNA metabolic process|GO:0044238,primary metabolic process|GO:0044271,cellular nitrogen compound biosynthetic process|GO:0051253,negative regulation of RNA metabolic process|GO:0051252,regulation of RNA metabolic process|GO:0034641,cellular nitrogen compound metabolic process|GO:0044237,cellular metabolic process|GO:0043170,macromolecule metabolic process|GO:0006355,regulation of transcription, DNA-templated|GO:0006357,regulation of transcription from RNA polymerase II promoter|GO:0006351,transcription, DNA-templated|GO:0019438,aromatic compound biosynthetic process|GO:0010558,negative regulation of macromolecule biosynthetic process|GO:0048523,negative regulation of cellular process|GO:0051169,nuclear transport|GO:0070647,protein modification by small protein conjugation or removal|GO:0032446,protein modification by small protein conjugation|GO:1901564,organonitrogen compound metabolic process|GO:0019538,protein metabolic process|GO:0016567,protein ubiquitination|GO:0006810,transport|GO:0043412,macromolecule modification|GO:0036211,protein modification process|GO:0051234,establishment of localization|GO:0046907,intracellular transport|GO:0044765,single-organism transport|GO:0044699,single-organism process|GO:1902593,single-organism nuclear import|GO:0044267,cellular protein metabolic process|GO:0006913,nucleocytoplasmic transport|GO:0006464,cellular protein modification process|GO:0051170,nuclear import|GO:0051649,establishment of localization in cell|GO:0051179,localization|GO:1902578,single-organism localization|GO:0051641,cellular localization|GO:1902582,single-organism intracellular transport	GO:0005737,cytoplasm|GO:0043231,intracellular membrane-bounded organelle|GO:0005634,nucleus|GO:0044464,cell part|GO:0005623,cell|GO:0005622,intracellular|GO:0043229,intracellular organelle|GO:0044424,intracellular part|GO:0043227,membrane-bounded organelle|GO:0043226,organelle|GO:0031461,cullin-RING ubiquitin ligase complex|GO:0043657,host cell|GO:0018995,host|GO:1902494,catalytic complex|GO:1990234,transferase complex|GO:0032991,macromolecular complex|GO:0031463,Cul3-RING ubiquitin ligase complex|GO:0043234,protein complex|GO:0044217,other organism part|GO:0044216,other organism cell|GO:0044215,other organism|GO:0005829,cytosol|GO:0000151,ubiquitin ligase complex|GO:0044444,cytoplasmic part	GO:0001012,RNA polymerase II regulatory region DNA binding|GO:0001158,enhancer sequence-specific DNA binding|GO:0005488,binding|GO:0003676,nucleic acid binding|GO:0003677,DNA binding|GO:1901363,heterocyclic compound binding|GO:0043565,sequence-specific DNA binding|GO:0000981,RNA polymerase II transcription factor activity, sequence-specific DNA binding|GO:0000980,RNA polymerase II distal enhancer sequence-specific DNA binding|GO:0001067,regulatory region nucleic acid binding|GO:0035326,enhancer binding|GO:0001206,transcriptional repressor activity, RNA polymerase II distal enhancer sequence-specific binding|GO:0044212,transcription regulatory region DNA binding|GO:0001227,transcriptional repressor activity, RNA polymerase II transcription regulatory region sequence-specific binding|GO:0003700,transcription factor activity, sequence-specific DNA binding|GO:0097159,organic cyclic compound binding|GO:0003705,transcription factor activity, RNA polymerase II distal enhancer sequence-specific binding|GO:0000976,transcription regulatory region sequence-specific DNA binding|GO:0000977,RNA polymerase II regulatory region sequence-specific DNA binding|GO:0000975,regulatory region DNA binding|GO:0001071,nucleic acid binding transcription factor activity|GO:0019787,ubiquitin-like protein transferase activity|GO:1990837,sequence-specific double-stranded DNA binding|GO:0016740,transferase activity|GO:0003690,double-stranded DNA binding|GO:0003824,catalytic activity|GO:0004842,ubiquitin-protein transferase activity
TMEM209		GO:0044425,membrane part|GO:0016021,integral component of membrane|GO:0016020,membrane|GO:0031224,intrinsic component of membrane	
CACUL1	GO:0042127,regulation of cell proliferation|GO:0044238,primary metabolic process|GO:0019220,regulation of phosphate metabolic process|GO:0080090,regulation of primary metabolic process|GO:0019222,regulation of metabolic process|GO:0033674,positive regulation of kinase activity|GO:0044699,single-organism process|GO:0031325,positive regulation of cellular metabolic process|GO:0031323,regulation of cellular metabolic process|GO:1903047,mitotic cell cycle process|GO:0044770,cell cycle phase transition|GO:0045860,positive regulation of protein kinase activity|GO:0044772,mitotic cell cycle phase transition|GO:0022402,cell cycle process|GO:0044237,cellular metabolic process|GO:0043632,modification-dependent macromolecule catabolic process|GO:0050789,regulation of biological process|GO:0043085,positive regulation of catalytic activity|GO:0009893,positive regulation of metabolic process|GO:0044267,cellular protein metabolic process|GO:1901575,organic substance catabolic process|GO:0044265,cellular macromolecule catabolic process|GO:0051347,positive regulation of transferase activity|GO:0010604,positive regulation of macromolecule metabolic process|GO:0044260,cellular macromolecule metabolic process|GO:0071704,organic substance metabolic process|GO:0010562,positive regulation of phosphorus metabolic process|GO:0043549,regulation of kinase activity|GO:0051246,regulation of protein metabolic process|GO:0051247,positive regulation of protein metabolic process|GO:0032446,protein modification by small protein conjugation|GO:0032270,positive regulation of cellular protein metabolic process|GO:0007049,cell cycle|GO:0044093,positive regulation of molecular function|GO:0031399,regulation of protein modification process|GO:0048518,positive regulation of biological process|GO:0065007,biological regulation|GO:0065009,regulation of molecular function|GO:0042787,protein ubiquitination involved in ubiquitin-dependent protein catabolic process|GO:0008284,positive regulation of cell proliferation|GO:0006468,protein phosphorylation|GO:0050790,regulation of catalytic activity|GO:0000278,mitotic cell cycle|GO:0045937,positive regulation of phosphate metabolic process|GO:0008283,cell proliferation|GO:0060255,regulation of macromolecule metabolic process|GO:0000082,G1/S transition of mitotic cell cycle|GO:0045859,regulation of protein kinase activity|GO:0019941,modification-dependent protein catabolic process|GO:0006464,cellular protein modification process|GO:0050794,regulation of cellular process|GO:0051174,regulation of phosphorus metabolic process|GO:0043412,macromolecule modification|GO:0036211,protein modification process|GO:0044763,single-organism cellular process|GO:0030163,protein catabolic process|GO:0008152,metabolic process|GO:0006508,proteolysis|GO:0044257,cellular protein catabolic process|GO:0044843,cell cycle G1/S phase transition|GO:0009056,catabolic process|GO:0009057,macromolecule catabolic process|GO:0044248,cellular catabolic process|GO:0070647,protein modification by small protein conjugation or removal|GO:0042325,regulation of phosphorylation|GO:0051603,proteolysis involved in cellular protein catabolic process|GO:0042327,positive regulation of phosphorylation|GO:0032268,regulation of cellular protein metabolic process|GO:0019538,protein metabolic process|GO:0016310,phosphorylation|GO:0031401,positive regulation of protein modification process|GO:0051338,regulation of transferase activity|GO:0043170,macromolecule metabolic process|GO:0016567,protein ubiquitination|GO:0006796,phosphate-containing compound metabolic process|GO:0006511,ubiquitin-dependent protein catabolic process|GO:0006793,phosphorus metabolic process|GO:0009987,cellular process|GO:0001932,regulation of protein phosphorylation|GO:0001934,positive regulation of protein phosphorylation|GO:0048522,positive regulation of cellular process|GO:0006807,nitrogen compound metabolic process|GO:1901564,organonitrogen compound metabolic process|GO:1901565,organonitrogen compound catabolic process	GO:0043234,protein complex|GO:0032991,macromolecular complex|GO:0043231,intracellular membrane-bounded organelle|GO:0005634,nucleus|GO:0044464,cell part|GO:0000151,ubiquitin ligase complex|GO:0000152,nuclear ubiquitin ligase complex|GO:0005622,intracellular|GO:0044446,intracellular organelle part|GO:1902494,catalytic complex|GO:0043229,intracellular organelle|GO:0044428,nuclear part|GO:0005623,cell|GO:1990234,transferase complex|GO:0044424,intracellular part|GO:0031461,cullin-RING ubiquitin ligase complex|GO:0043227,membrane-bounded organelle|GO:0043226,organelle|GO:0044422,organelle part|GO:0005680,anaphase-promoting complex	GO:0019787,ubiquitin-like protein transferase activity|GO:0031625,ubiquitin protein ligase binding|GO:0044389,ubiquitin-like protein ligase binding|GO:0019900,kinase binding|GO:0061630,ubiquitin protein ligase activity|GO:0019899,enzyme binding|GO:0019901,protein kinase binding|GO:0003824,catalytic activity|GO:0016740,transferase activity|GO:0005488,binding|GO:0005515,protein binding|GO:0004842,ubiquitin-protein transferase activity|GO:0061659,ubiquitin-like protein ligase activity
COPA	GO:0033036,macromolecule localization|GO:0008104,protein localization|GO:0022600,digestive system process|GO:0048200,Golgi transport vesicle coating|GO:0006901,vesicle coating|GO:0048205,COPI coating of Golgi vesicle|GO:0006903,vesicle targeting|GO:0061024,membrane organization|GO:0032941,secretion by tissue|GO:0051641,cellular localization|GO:0051640,organelle localization|GO:0051656,establishment of organelle localization|GO:0051650,establishment of vesicle localization|GO:0044699,single-organism process|GO:0070727,cellular macromolecule localization|GO:0006886,intracellular protein transport|GO:0048193,Golgi vesicle transport|GO:0016043,cellular component organization|GO:0030157,pancreatic juice secretion|GO:0065007,biological regulation|GO:0071840,cellular component organization or biogenesis|GO:0006810,transport|GO:0071702,organic substance transport|GO:0065008,regulation of biological quality|GO:0034613,cellular protein localization|GO:0048194,Golgi vesicle budding|GO:0032501,multicellular organismal process|GO:1902591,single-organism membrane budding|GO:0050878,regulation of body fluid levels|GO:0007589,body fluid secretion|GO:0007586,digestion|GO:0009987,cellular process|GO:0048199,vesicle targeting, to, from or within Golgi|GO:0006900,membrane budding|GO:0045184,establishment of protein localization|GO:0044765,single-organism transport|GO:0044802,single-organism membrane organization|GO:0051648,vesicle localization|GO:0051649,establishment of localization in cell|GO:0051668,localization within membrane|GO:0051234,establishment of localization|GO:0051179,localization|GO:1902578,single-organism localization|GO:0003008,system process|GO:0006996,organelle organization|GO:0046907,intracellular transport|GO:0046903,secretion|GO:0006890,retrograde vesicle-mediated transport, Golgi to ER|GO:0016192,vesicle-mediated transport|GO:0044707,single-multicellular organism process|GO:0044763,single-organism cellular process|GO:0016050,vesicle organization|GO:0035964,COPI-coated vesicle budding|GO:1902589,single-organism organelle organization|GO:0015031,protein transport|GO:1902582,single-organism intracellular transport|GO:1902580,single-organism cellular localization|GO:0015833,peptide transport|GO:0042886,amide transport|GO:0006888,ER to Golgi vesicle-mediated transport|GO:0071705,nitrogen compound transport|GO:0006891,intra-Golgi vesicle-mediated transport	GO:0031090,organelle membrane|GO:0043229,intracellular organelle|GO:0030117,membrane coat|GO:0005622,intracellular|GO:0030135,coated vesicle|GO:0043226,organelle|GO:0030137,COPI-coated vesicle|GO:0005737,cytoplasm|GO:0070062,extracellular exosome|GO:0030663,COPI-coated vesicle membrane|GO:0030662,coated vesicle membrane|GO:0016023,cytoplasmic, membrane-bounded vesicle|GO:0031410,cytoplasmic vesicle|GO:0098805,whole membrane|GO:0016020,membrane|GO:0044444,cytoplasmic part|GO:0031988,membrane-bounded vesicle|GO:0044433,cytoplasmic vesicle part|GO:0044431,Golgi apparatus part|GO:0005615,extracellular space|GO:0048475,coated membrane|GO:0005794,Golgi apparatus|GO:0098588,bounding membrane of organelle|GO:0005798,Golgi-associated vesicle|GO:0030659,cytoplasmic vesicle membrane|GO:0012505,endomembrane system|GO:0012506,vesicle membrane|GO:0032991,macromolecular complex|GO:0030120,vesicle coat|GO:1903561,extracellular vesicle|GO:0030126,COPI vesicle coat|GO:0031982,vesicle|GO:0043234,protein complex|GO:0043230,extracellular organelle|GO:0043231,intracellular membrane-bounded organelle|GO:0005829,cytosol|GO:0044464,cell part|GO:0005623,cell|GO:0000139,Golgi membrane|GO:0044446,intracellular organelle part|GO:0098796,membrane protein complex|GO:0043227,membrane-bounded organelle|GO:0005576,extracellular region|GO:0030660,Golgi-associated vesicle membrane|GO:0044424,intracellular part|GO:0044425,membrane part|GO:0065010,extracellular membrane-bounded organelle|GO:0044421,extracellular region part|GO:0044422,organelle part|GO:0005783,endoplasmic reticulum|GO:0005789,endoplasmic reticulum membrane|GO:0042175,nuclear outer membrane-endoplasmic reticulum membrane network|GO:0030133,transport vesicle|GO:0031984,organelle subcompartment|GO:0044432,endoplasmic reticulum part|GO:0098827,endoplasmic reticulum subcompartment|GO:0097708,intracellular vesicle|GO:0098791,Golgi subcompartment	GO:0005179,hormone activity|GO:0005198,structural molecule activity|GO:0005488,binding|GO:0005515,protein binding|GO:0005102,receptor binding
SEC11A	GO:0090087,regulation of peptide transport|GO:0008104,protein localization|GO:0051046,regulation of secretion|GO:0051049,regulation of transport|GO:0061024,membrane organization|GO:0071840,cellular component organization or biogenesis|GO:0070727,cellular macromolecule localization|GO:0043043,peptide biosynthetic process|GO:0046879,hormone secretion|GO:0033036,macromolecule localization|GO:0006605,protein targeting|GO:0045184,establishment of protein localization|GO:0090276,regulation of peptide hormone secretion|GO:0072657,protein localization to membrane|GO:0044700,single organism signaling|GO:1901564,organonitrogen compound metabolic process|GO:1901566,organonitrogen compound biosynthetic process|GO:0019538,protein metabolic process|GO:0015833,peptide transport|GO:0006613,cotranslational protein targeting to membrane|GO:0006612,protein targeting to membrane|GO:0006614,SRP-dependent cotranslational protein targeting to membrane|GO:0032940,secretion by cell|GO:0006807,nitrogen compound metabolic process|GO:0051223,regulation of protein transport|GO:0050789,regulation of biological process|GO:0030072,peptide hormone secretion|GO:0030073,insulin secretion|GO:1901576,organic substance biosynthetic process|GO:0050708,regulation of protein secretion|GO:0044260,cellular macromolecule metabolic process|GO:0006886,intracellular protein transport|GO:0016043,cellular component organization|GO:0065007,biological regulation|GO:0065008,regulation of biological quality|GO:0070201,regulation of establishment of protein localization|GO:0009306,protein secretion|GO:0006810,transport|GO:0050796,regulation of insulin secretion|GO:0050794,regulation of cellular process|GO:0070972,protein localization to endoplasmic reticulum|GO:0044802,single-organism membrane organization|GO:0008152,metabolic process|GO:0010817,regulation of hormone levels|GO:0046903,secretion|GO:0044271,cellular nitrogen compound biosynthetic process|GO:0051604,protein maturation|GO:0009058,biosynthetic process|GO:0045047,protein targeting to ER|GO:0006518,peptide metabolic process|GO:0044249,cellular biosynthetic process|GO:0034641,cellular nitrogen compound metabolic process|GO:0023052,signaling|GO:1903530,regulation of secretion by cell|GO:0034645,cellular macromolecule biosynthetic process|GO:0023051,regulation of signaling|GO:0010646,regulation of cell communication|GO:0044699,single-organism process|GO:0032880,regulation of protein localization|GO:0042886,amide transport|GO:0051234,establishment of localization|GO:0006508,proteolysis|GO:0072599,establishment of protein localization to endoplasmic reticulum|GO:0072594,establishment of protein localization to organelle|GO:0009987,cellular process|GO:0046883,regulation of hormone secretion|GO:0016485,protein processing|GO:0032879,regulation of localization|GO:0016482,cytosolic transport|GO:0043604,amide biosynthetic process|GO:0046907,intracellular transport|GO:0043603,cellular amide metabolic process|GO:0043170,macromolecule metabolic process|GO:0090150,establishment of protein localization to membrane|GO:0033365,protein localization to organelle|GO:0060341,regulation of cellular localization|GO:0071705,nitrogen compound transport|GO:0071704,organic substance metabolic process|GO:0010467,gene expression|GO:0071702,organic substance transport|GO:0044267,cellular protein metabolic process|GO:0034613,cellular protein localization|GO:0023061,signal release|GO:0006465,signal peptide processing|GO:0044765,single-organism transport|GO:0009059,macromolecule biosynthetic process|GO:0044763,single-organism cellular process|GO:0051649,establishment of localization in cell|GO:0007267,cell-cell signaling|GO:0007154,cell communication|GO:0051179,localization|GO:1902578,single-organism localization|GO:0051641,cellular localization|GO:0044238,primary metabolic process|GO:0002790,peptide secretion|GO:0002791,regulation of peptide secretion|GO:0044237,cellular metabolic process|GO:0009914,hormone transport|GO:0015031,protein transport|GO:1902582,single-organism intracellular transport|GO:1902580,single-organism cellular localization|GO:0006412,translation	GO:0005783,endoplasmic reticulum|GO:0005789,endoplasmic reticulum membrane|GO:0042175,nuclear outer membrane-endoplasmic reticulum membrane network|GO:0043229,intracellular organelle|GO:0043227,membrane-bounded organelle|GO:0043226,organelle|GO:0031224,intrinsic component of membrane|GO:0005737,cytoplasm|GO:0031982,vesicle|GO:0016021,integral component of membrane|GO:0016020,membrane|GO:0031988,membrane-bounded vesicle|GO:0044432,endoplasmic reticulum part|GO:0098588,bounding membrane of organelle|GO:0012505,endomembrane system|GO:1903561,extracellular vesicle|GO:0031090,organelle membrane|GO:0043230,extracellular organelle|GO:0043231,intracellular membrane-bounded organelle|GO:0070062,extracellular exosome|GO:0044464,cell part|GO:0005623,cell|GO:0005622,intracellular|GO:0044446,intracellular organelle part|GO:0044444,cytoplasmic part|GO:0005576,extracellular region|GO:0044424,intracellular part|GO:0044425,membrane part|GO:0065010,extracellular membrane-bounded organelle|GO:0044421,extracellular region part|GO:0044422,organelle part|GO:0005787,signal peptidase complex|GO:0031984,organelle subcompartment|GO:0098827,endoplasmic reticulum subcompartment|GO:0032991,macromolecular complex|GO:0043234,protein complex|GO:0098796,membrane protein complex	GO:0016787,hydrolase activity|GO:0017171,serine hydrolase activity|GO:0003824,catalytic activity|GO:0070011,peptidase activity, acting on L-amino acid peptides|GO:0008233,peptidase activity|GO:0008236,serine-type peptidase activity

ZNF778	GO:0080090,regulation of primary metabolic process|GO:0019222,regulation of metabolic process|GO:0031326,regulation of cellular biosynthetic process|GO:0031323,regulation of cellular metabolic process|GO:0090304,nucleic acid metabolic process|GO:0044249,cellular biosynthetic process|GO:0034641,cellular nitrogen compound metabolic process|GO:0006807,nitrogen compound metabolic process|GO:0034645,cellular macromolecule biosynthetic process|GO:1901362,organic cyclic compound biosynthetic process|GO:0050789,regulation of biological process|GO:0097659,nucleic acid-templated transcription|GO:0032774,RNA biosynthetic process|GO:0006139,nucleobase-containing compound metabolic process|GO:0044260,cellular macromolecule metabolic process|GO:0071704,organic substance metabolic process|GO:2000112,regulation of cellular macromolecule biosynthetic process|GO:0060255,regulation of macromolecule metabolic process|GO:0010467,gene expression|GO:0065007,biological regulation|GO:1901360,organic cyclic compound metabolic process|GO:0010468,regulation of gene expression|GO:0018130,heterocycle biosynthetic process|GO:1901576,organic substance biosynthetic process|GO:0019219,regulation of nucleobase-containing compound metabolic process|GO:0006725,cellular aromatic compound metabolic process|GO:0009987,cellular process|GO:0009889,regulation of biosynthetic process|GO:1903506,regulation of nucleic acid-templated transcription|GO:0050794,regulation of cellular process|GO:0009058,biosynthetic process|GO:0009059,macromolecule biosynthetic process|GO:0051171,regulation of nitrogen compound metabolic process|GO:0008152,metabolic process|GO:2001141,regulation of RNA biosynthetic process|GO:0034654,nucleobase-containing compound biosynthetic process|GO:0046483,heterocycle metabolic process|GO:0016070,RNA metabolic process|GO:0044238,primary metabolic process|GO:0044271,cellular nitrogen compound biosynthetic process|GO:0051252,regulation of RNA metabolic process|GO:0044237,cellular metabolic process|GO:0043170,macromolecule metabolic process|GO:0006355,regulation of transcription, DNA-templated|GO:0010556,regulation of macromolecule biosynthetic process|GO:0006351,transcription, DNA-templated|GO:0019438,aromatic compound biosynthetic process	GO:0043231,intracellular membrane-bounded organelle|GO:0005634,nucleus|GO:0044464,cell part|GO:0005623,cell|GO:0005622,intracellular|GO:0043229,intracellular organelle|GO:0044424,intracellular part|GO:0043227,membrane-bounded organelle|GO:0043226,organelle	GO:0043169,cation binding|GO:0005515,protein binding|GO:0097159,organic cyclic compound binding|GO:0043167,ion binding|GO:0005488,binding|GO:0003676,nucleic acid binding|GO:0003677,DNA binding|GO:0046872,metal ion binding|GO:1901363,heterocyclic compound binding|GO:0003700,transcription factor activity, sequence-specific DNA binding|GO:0001071,nucleic acid binding transcription factor activity|GO:0000981,RNA polymerase II transcription factor activity, sequence-specific DNA binding
KMT2E	GO:0006479,protein methylation|GO:0080090,regulation of primary metabolic process|GO:0019222,regulation of metabolic process|GO:0007165,signal transduction|GO:0051568,histone H3-K4 methylation|GO:1901362,organic cyclic compound biosynthetic process|GO:0071840,cellular component organization or biogenesis|GO:0006306,DNA methylation|GO:0044710,single-organism metabolic process|GO:0006304,DNA modification|GO:0045786,negative regulation of cell cycle|GO:0010604,positive regulation of macromolecule metabolic process|GO:0071704,organic substance metabolic process|GO:0048869,cellular developmental process|GO:0002262,myeloid cell homeostasis|GO:0040029,regulation of gene expression, epigenetic|GO:0018193,peptidyl-amino acid modification|GO:0030852,regulation of granulocyte differentiation|GO:0030851,granulocyte differentiation|GO:0030854,positive regulation of granulocyte differentiation|GO:0048518,positive regulation of biological process|GO:0048519,negative regulation of biological process|GO:0016571,histone methylation|GO:0010467,gene expression|GO:0030218,erythrocyte differentiation|GO:0034645,cellular macromolecule biosynthetic process|GO:1903708,positive regulation of hemopoiesis|GO:2001141,regulation of RNA biosynthetic process|GO:1903706,regulation of hemopoiesis|GO:0010033,response to organic substance|GO:0046483,heterocycle metabolic process|GO:0044700,single organism signaling|GO:0032259,methylation|GO:0048872,homeostasis of number of cells|GO:0044707,single-multicellular organism process|GO:0018205,peptidyl-lysine modification|GO:0002274,myeloid leukocyte activation|GO:0002376,immune system process|GO:0019538,protein metabolic process|GO:0019438,aromatic compound biosynthetic process|GO:0016568,chromatin modification|GO:0016569,covalent chromatin modification|GO:0001101,response to acid chemical|GO:0006305,DNA alkylation|GO:0009893,positive regulation of metabolic process|GO:0009891,positive regulation of biosynthetic process|GO:0008152,metabolic process|GO:0045321,leukocyte activation|GO:0010628,positive regulation of gene expression|GO:0050789,regulation of biological process|GO:0097659,nucleic acid-templated transcription|GO:0002761,regulation of myeloid leukocyte differentiation|GO:0044267,cellular protein metabolic process|GO:0002763,positive regulation of myeloid leukocyte differentiation|GO:0044260,cellular macromolecule metabolic process|GO:0018022,peptidyl-lysine methylation|GO:0016043,cellular component organization|GO:0002684,positive regulation of immune system process|GO:0002682,regulation of immune system process|GO:0016570,histone modification|GO:0065008,regulation of biological quality|GO:0018130,heterocycle biosynthetic process|GO:0050793,regulation of developmental process|GO:0009889,regulation of biosynthetic process|GO:0051716,cellular response to stimulus|GO:0050794,regulation of cellular process|GO:0007050,cell cycle arrest|GO:0001775,cell activation|GO:0044728,DNA methylation or demethylation|GO:0043414,macromolecule methylation|GO:0051239,regulation of multicellular organismal process|GO:0030097,hemopoiesis|GO:0034654,nucleobase-containing compound biosynthetic process|GO:0002521,leukocyte differentiation|GO:0002520,immune system development|GO:0016070,RNA metabolic process|GO:0044271,cellular nitrogen compound biosynthetic process|GO:0050896,response to stimulus|GO:0043412,macromolecule modification|GO:0006355,regulation of transcription, DNA-templated|GO:0010557,positive regulation of macromolecule biosynthetic process|GO:0045639,positive regulation of myeloid cell differentiation|GO:0006351,transcription, DNA-templated|GO:0045637,regulation of myeloid cell differentiation|GO:0032774,RNA biosynthetic process|GO:0030154,cell differentiation|GO:0044249,cellular biosynthetic process|GO:0034641,cellular nitrogen compound metabolic process|GO:0023052,signaling|GO:0070887,cellular response to chemical stimulus|GO:0042221,response to chemical|GO:0048384,retinoic acid receptor signaling pathway|GO:0007049,cell cycle|GO:0006139,nucleobase-containing compound metabolic process|GO:0051240,positive regulation of multicellular organismal process|GO:0042119,neutrophil activation|GO:0034968,histone lysine methylation|GO:0048513,animal organ development|GO:0036211,protein modification process|GO:1903508,positive regulation of nucleic acid-templated transcription|GO:1901700,response to oxygen-containing compound|GO:0032501,multicellular organismal process|GO:0044238,primary metabolic process|GO:0009987,cellular process|GO:0071396,cellular response to lipid|GO:1903506,regulation of nucleic acid-templated transcription|GO:0045597,positive regulation of cell differentiation|GO:0045595,regulation of cell differentiation|GO:0036230,granulocyte activation|GO:0045893,positive regulation of transcription, DNA-templated|GO:0090304,nucleic acid metabolic process|GO:0044699,single-organism process|GO:0051094,positive regulation of developmental process|GO:0051252,regulation of RNA metabolic process|GO:0051254,positive regulation of RNA metabolic process|GO:0043170,macromolecule metabolic process|GO:1902680,positive regulation of RNA biosynthetic process|GO:0008213,protein alkylation|GO:0006807,nitrogen compound metabolic process|GO:0030099,myeloid cell differentiation|GO:0048731,system development|GO:0032502,developmental process|GO:0048856,anatomical structure development|GO:0031328,positive regulation of cellular biosynthetic process|GO:0043933,macromolecular complex subunit organization|GO:0031326,regulation of cellular biosynthetic process|GO:0031325,positive regulation of cellular metabolic process|GO:0031323,regulation of cellular metabolic process|GO:0071229,cellular response to acid chemical|GO:0042592,homeostatic process|GO:0022402,cell cycle process|GO:0007275,multicellular organism development|GO:0030522,intracellular receptor signaling pathway|GO:0006325,chromatin organization|GO:0033993,response to lipid|GO:1901360,organic cyclic compound metabolic process|GO:2000112,regulation of cellular macromolecule biosynthetic process|GO:1902589,single-organism organelle organization|GO:0002573,myeloid leukocyte differentiation|GO:0060255,regulation of macromolecule metabolic process|GO:0071310,cellular response to organic substance|GO:0010556,regulation of macromolecule biosynthetic process|GO:0048534,hematopoietic or lymphoid organ development|GO:0010468,regulation of gene expression|GO:0034101,erythrocyte homeostasis|GO:0032526,response to retinoic acid|GO:0045935,positive regulation of nucleobase-containing compound metabolic process|GO:1901576,organic substance biosynthetic process|GO:0019219,regulation of nucleobase-containing compound metabolic process|GO:0006725,cellular aromatic compound metabolic process|GO:0006464,cellular protein modification process|GO:0044767,single-organism developmental process|GO:0009058,biosynthetic process|GO:0009059,macromolecule biosynthetic process|GO:0044763,single-organism cellular process|GO:0051171,regulation of nitrogen compound metabolic process|GO:0051173,positive regulation of nitrogen compound metabolic process|GO:0007154,cell communication|GO:0002443,leukocyte mediated immunity|GO:0002444,myeloid leukocyte mediated immunity|GO:0002446,neutrophil mediated immunity|GO:0006996,organelle organization|GO:1901701,cellular response to oxygen-containing compound|GO:0051276,chromosome organization|GO:0051726,regulation of cell cycle|GO:0065007,biological regulation|GO:0044237,cellular metabolic process|GO:1902107,positive regulation of leukocyte differentiation|GO:0071300,cellular response to retinoic acid|GO:1902105,regulation of leukocyte differentiation|GO:2000026,regulation of multicellular organismal development|GO:0002252,immune effector process|GO:0006259,DNA metabolic process|GO:0048523,negative regulation of cellular process|GO:0048522,positive regulation of cellular process|GO:1901564,organonitrogen compound metabolic process|GO:0045931,positive regulation of mitotic cell cycle|GO:0090068,positive regulation of cell cycle process|GO:0007346,regulation of mitotic cell cycle|GO:0000278,mitotic cell cycle|GO:0045787,positive regulation of cell cycle|GO:0030219,megakaryocyte differentiation|GO:0045652,regulation of megakaryocyte differentiation|GO:0010564,regulation of cell cycle process|GO:1903047,mitotic cell cycle process|GO:2000045,regulation of G1/S transition of mitotic cell cycle|GO:1902806,regulation of cell cycle G1/S phase transition|GO:1901987,regulation of cell cycle phase transition|GO:0044772,mitotic cell cycle phase transition|GO:0044770,cell cycle phase transition|GO:1901990,regulation of mitotic cell cycle	GO:0031974,membrane-enclosed lumen|GO:0043229,intracellular organelle|GO:0071944,cell periphery|GO:0005623,cell|GO:0043227,membrane-bounded organelle|GO:0043226,organelle|GO:0034708,methyltransferase complex|GO:0016607,nuclear speck|GO:0016604,nuclear body|GO:0045171,intercellular bridge|GO:0031981,nuclear lumen|GO:0005634,nucleus|GO:0016020,membrane|GO:0005654,nucleoplasm|GO:0044451,nucleoplasm part|GO:0070688,MLL5-L complex|GO:0005737,cytoplasm|GO:1902494,catalytic complex|GO:1990234,transferase complex|GO:0005886,plasma membrane|GO:0043234,protein complex|GO:0032991,macromolecular complex|GO:0043231,intracellular membrane-bounded organelle|GO:0043233,organelle lumen|GO:0044464,cell part|GO:0035097,histone methyltransferase complex|GO:0005622,intracellular|GO:0044446,intracellular organelle part|GO:0070013,intracellular organelle lumen|GO:0005576,extracellular region|GO:0044428,nuclear part|GO:0044424,intracellular part|GO:0044421,extracellular region part|GO:0044422,organelle part|GO:0005856,cytoskeleton|GO:0015630,microtubule cytoskeleton|GO:0043228,non-membrane-bounded organelle|GO:0043232,intracellular non-membrane-bounded organelle|GO:0005815,microtubule organizing center|GO:0044430,cytoskeletal part|GO:0000785,chromatin|GO:0035327,transcriptionally active chromatin|GO:0044427,chromosomal part|GO:0005694,chromosome	GO:0018024,histone-lysine N-methyltransferase activity|GO:0042054,histone methyltransferase activity|GO:0016278,lysine N-methyltransferase activity|GO:0016279,protein-lysine N-methyltransferase activity|GO:0008276,protein methyltransferase activity|GO:0008270,zinc ion binding|GO:0008168,methyltransferase activity|GO:0043169,cation binding|GO:0046914,transition metal ion binding|GO:0003713,transcription coactivator activity|GO:0003712,transcription cofactor activity|GO:0043167,ion binding|GO:0016740,transferase activity|GO:0016741,transferase activity, transferring one-carbon groups|GO:0000989,transcription factor activity, transcription factor binding|GO:0000988,transcription factor activity, protein binding|GO:0046872,metal ion binding|GO:0042800,histone methyltransferase activity (H3-K4 specific)|GO:0008757,S-adenosylmethionine-dependent methyltransferase activity|GO:0003824,catalytic activity|GO:0008170,N-methyltransferase activity|GO:0019899,enzyme binding|GO:0005515,protein binding|GO:0005488,binding|GO:0042393,histone binding|GO:0035064,methylated histone binding
HIATL1	GO:0006810,transport|GO:0009987,cellular process|GO:0051179,localization|GO:0044765,single-organism transport|GO:0044763,single-organism cellular process|GO:1902578,single-organism localization|GO:0051234,establishment of localization|GO:0055085,transmembrane transport|GO:0044699,single-organism process	GO:0044425,membrane part|GO:0016021,integral component of membrane|GO:0016020,membrane|GO:0031224,intrinsic component of membrane	GO:0022891,substrate-specific transmembrane transporter activity|GO:0005215,transporter activity|GO:0022857,transmembrane transporter activity|GO:0022892,substrate-specific transporter activity
TGOLN2		GO:0031974,membrane-enclosed lumen|GO:0043229,intracellular organelle|GO:0043227,membrane-bounded organelle|GO:0043226,organelle|GO:0005737,cytoplasm|GO:0031984,organelle subcompartment|GO:0031981,nuclear lumen|GO:0005634,nucleus|GO:0005654,nucleoplasm|GO:0044431,Golgi apparatus part|GO:0005794,Golgi apparatus|GO:0012505,endomembrane system|GO:0043231,intracellular membrane-bounded organelle|GO:0043233,organelle lumen|GO:0005802,trans-Golgi network|GO:0044464,cell part|GO:0005623,cell|GO:0005622,intracellular|GO:0044446,intracellular organelle part|GO:0070013,intracellular organelle lumen|GO:0044444,cytoplasmic part|GO:0044428,nuclear part|GO:0098791,Golgi subcompartment|GO:0044424,intracellular part|GO:0044422,organelle part|GO:0031224,intrinsic component of membrane|GO:0016021,integral component of membrane|GO:0016020,membrane|GO:0044425,membrane part	
MLF2	GO:0006952,defense response|GO:0006950,response to stress|GO:0050896,response to stimulus	GO:0005737,cytoplasm|GO:0043231,intracellular membrane-bounded organelle|GO:0005634,nucleus|GO:0016020,membrane|GO:0044464,cell part|GO:0005623,cell|GO:0005622,intracellular|GO:0043229,intracellular organelle|GO:0044424,intracellular part|GO:0043227,membrane-bounded organelle|GO:0043226,organelle	GO:0005488,binding|GO:0005515,protein binding
RSRC1	GO:0090304,nucleic acid metabolic process|GO:0034641,cellular nitrogen compound metabolic process|GO:0006807,nitrogen compound metabolic process|GO:0044237,cellular metabolic process|GO:1901360,organic cyclic compound metabolic process|GO:0006139,nucleobase-containing compound metabolic process|GO:0000380,alternative mRNA splicing, via spliceosome|GO:0044260,cellular macromolecule metabolic process|GO:0071704,organic substance metabolic process|GO:0010467,gene expression|GO:0008380,RNA splicing|GO:0044238,primary metabolic process|GO:0009987,cellular process|GO:0006725,cellular aromatic compound metabolic process|GO:0000375,RNA splicing, via transesterification reactions|GO:0000377,RNA splicing, via transesterification reactions with bulged adenosine as nucleophile|GO:0008152,metabolic process|GO:0046483,heterocycle metabolic process|GO:0016070,RNA metabolic process|GO:0016071,mRNA metabolic process|GO:0000398,mRNA splicing, via spliceosome|GO:0043170,macromolecule metabolic process|GO:0006396,RNA processing|GO:0006397,mRNA processing		
BOLA2	GO:0016310,phosphorylation|GO:0090304,nucleic acid metabolic process|GO:0034641,cellular nitrogen compound metabolic process|GO:0006807,nitrogen compound metabolic process|GO:0044699,single-organism process|GO:0006139,nucleobase-containing compound metabolic process|GO:0044710,single-organism metabolic process|GO:0006302,double-strand break repair|GO:0000725,recombinational repair|GO:0000724,double-strand break repair via homologous recombination|GO:0071704,organic substance metabolic process|GO:1901360,organic cyclic compound metabolic process|GO:0006281,DNA repair|GO:0009987,cellular process|GO:0006725,cellular aromatic compound metabolic process|GO:0051716,cellular response to stimulus|GO:0006974,cellular response to DNA damage stimulus|GO:0006950,response to stress|GO:0044763,single-organism cellular process|GO:0008152,metabolic process|GO:0046483,heterocycle metabolic process|GO:0006310,DNA recombination|GO:0044238,primary metabolic process|GO:0044260,cellular macromolecule metabolic process|GO:0050896,response to stimulus|GO:0044237,cellular metabolic process|GO:0043170,macromolecule metabolic process|GO:0006796,phosphate-containing compound metabolic process|GO:0033554,cellular response to stress|GO:0006793,phosphorus metabolic process|GO:0006259,DNA metabolic process		GO:0016772,transferase activity, transferring phosphorus-containing groups|GO:0016301,kinase activity|GO:0003824,catalytic activity|GO:0016740,transferase activity|GO:0005488,binding|GO:0005515,protein binding|GO:0046983,protein dimerization activity
SELL	GO:0007599,hemostasis|GO:0048584,positive regulation of response to stimulus|GO:0048583,regulation of response to stimulus|GO:0007610,behavior|GO:0050920,regulation of chemotaxis|GO:0050921,positive regulation of chemotaxis|GO:0060326,cell chemotaxis|GO:0051716,cellular response to stimulus|GO:0042330,taxis|GO:0009611,response to wounding|GO:0048518,positive regulation of biological process|GO:0065007,biological regulation|GO:0030595,leukocyte chemotaxis|GO:0007596,blood coagulation|GO:0030593,neutrophil chemotaxis|GO:0006935,chemotaxis|GO:0034201,response to oleic acid|GO:0010033,response to organic substance|GO:0065008,regulation of biological quality|GO:0044707,single-multicellular organism process|GO:0009605,response to external stimulus|GO:0048870,cell motility|GO:0010243,response to organonitrogen compound|GO:0002376,immune system process|GO:0006928,movement of cell or subcellular component|GO:1902624,positive regulation of neutrophil migration|GO:0051674,localization of cell|GO:1902622,regulation of neutrophil migration|GO:0042981,regulation of apoptotic process|GO:0050789,regulation of biological process|GO:0002687,positive regulation of leukocyte migration|GO:0002684,positive regulation of immune system process|GO:0002685,regulation of leukocyte migration|GO:0002682,regulation of immune system process|GO:0014070,response to organic cyclic compound|GO:0016477,cell migration|GO:0002688,regulation of leukocyte chemotaxis|GO:0016265,obsolete death|GO:0042060,wound healing|GO:0050794,regulation of cellular process|GO:0012501,programmed cell death|GO:0006950,response to stress|GO:0050817,coagulation|GO:0050795,regulation of behavior|GO:0006955,immune response|GO:1901698,response to nitrogen compound|GO:0036296,response to increased oxygen levels|GO:2000145,regulation of cell motility|GO:0002690,positive regulation of leukocyte chemotaxis|GO:2000147,positive regulation of cell motility|GO:0032103,positive regulation of response to external stimulus|GO:0032101,regulation of response to external stimulus|GO:0070887,cellular response to chemical stimulus|GO:0014074,response to purine-containing compound|GO:0044699,single-organism process|GO:0009719,response to endogenous stimulus|GO:0097530,granulocyte migration|GO:0022610,biological adhesion|GO:1901700,response to oxygen-containing compound|GO:0032501,multicellular organismal process|GO:0050878,regulation of body fluid levels|GO:0051272,positive regulation of cellular component movement|GO:0009987,cellular process|GO:0051270,regulation of cellular component movement|GO:0001101,response to acid chemical|GO:0032879,regulation of localization|GO:0050776,regulation of immune response|GO:0070542,response to fatty acid|GO:0070543,response to linoleic acid|GO:0033198,response to ATP|GO:0097529,myeloid leukocyte migration|GO:0050896,response to stimulus|GO:1990266,neutrophil migration|GO:0050900,leukocyte migration|GO:0008219,cell death|GO:0010941,regulation of cell death|GO:0055093,response to hyperoxia|GO:0090023,positive regulation of neutrophil chemotaxis|GO:0090022,regulation of neutrophil chemotaxis|GO:0033993,response to lipid|GO:0043067,regulation of programmed cell death|GO:0071622,regulation of granulocyte chemotaxis|GO:0071621,granulocyte chemotaxis|GO:0046683,response to organophosphorus|GO:0071624,positive regulation of granulocyte chemotaxis|GO:0030335,positive regulation of cell migration|GO:0030334,regulation of cell migration|GO:0006915,apoptotic process|GO:0044763,single-organism cellular process|GO:0007155,cell adhesion|GO:0042221,response to chemical|GO:0051179,localization|GO:0070482,response to oxygen levels|GO:0040011,locomotion|GO:0009628,response to abiotic stimulus|GO:0040012,regulation of locomotion|GO:0040017,positive regulation of locomotion|GO:0048520,positive regulation of behavior|GO:0048522,positive regulation of cellular process|GO:0006887,exocytosis|GO:0042119,neutrophil activation|GO:0002446,neutrophil mediated immunity|GO:0045321,leukocyte activation|GO:0002283,neutrophil activation involved in immune response|GO:0002443,leukocyte mediated immunity|GO:0002263,cell activation involved in immune response|GO:0045055,regulated exocytosis|GO:0044765,single-organism transport|GO:0002366,leukocyte activation involved in immune response|GO:0006810,transport|GO:0001775,cell activation|GO:0036230,granulocyte activation|GO:0032940,secretion by cell|GO:0051234,establishment of localization|GO:0002444,myeloid leukocyte mediated immunity|GO:1902578,single-organism localization|GO:0046903,secretion|GO:0016192,vesicle-mediated transport|GO:0002275,myeloid cell activation involved in immune response|GO:0002274,myeloid leukocyte activation|GO:0043312,neutrophil degranulation|GO:0043299,leukocyte degranulation|GO:0002252,immune effector process|GO:0098602,single organism cell adhesion|GO:0016337,single organismal cell-cell adhesion|GO:0098609,cell-cell adhesion|GO:0045123,cellular extravasation|GO:0016339,calcium-dependent cell-cell adhesion via plasma membrane cell adhesion molecules|GO:0061756,leukocyte adhesion to vascular endothelial cell|GO:0050901,leukocyte tethering or rolling|GO:0007159,leukocyte cell-cell adhesion|GO:0098742,cell-cell adhesion via plasma-membrane adhesion molecules	GO:0009897,external side of plasma membrane|GO:0016020,membrane|GO:0031226,intrinsic component of plasma membrane|GO:0016021,integral component of membrane|GO:0098552,side of membrane|GO:0044464,cell part|GO:0005623,cell|GO:0071944,cell periphery|GO:0005887,integral component of plasma membrane|GO:0005886,plasma membrane|GO:0044425,membrane part|GO:0044459,plasma membrane part|GO:0009986,cell surface|GO:0031224,intrinsic component of membrane|GO:0043229,intracellular organelle|GO:0043227,membrane-bounded organelle|GO:0043226,organelle|GO:0005737,cytoplasm|GO:0030667,secretory granule membrane|GO:0031090,organelle membrane|GO:0016023,cytoplasmic, membrane-bounded vesicle|GO:0031410,cytoplasmic vesicle|GO:0031988,membrane-bounded vesicle|GO:0044433,cytoplasmic vesicle part|GO:0099503,secretory vesicle|GO:0030141,secretory granule|GO:0098588,bounding membrane of organelle|GO:0030659,cytoplasmic vesicle membrane|GO:0097708,intracellular vesicle|GO:0012506,vesicle membrane|GO:0031982,vesicle|GO:0043231,intracellular membrane-bounded organelle|GO:0005622,intracellular|GO:0044446,intracellular organelle part|GO:0044444,cytoplasmic part|GO:0044424,intracellular part|GO:0098805,whole membrane|GO:0044422,organelle part|GO:0012505,endomembrane system	GO:1901681,sulfur compound binding|GO:0043168,anion binding|GO:0002020,protease binding|GO:0097367,carbohydrate derivative binding|GO:0043208,glycosphingolipid binding|GO:0046625,sphingolipid binding|GO:0030246,carbohydrate binding|GO:0019899,enzyme binding|GO:0043167,ion binding|GO:0005539,glycosaminoglycan binding|GO:0005515,protein binding|GO:0008289,lipid binding|GO:0051861,glycolipid binding|GO:0050839,cell adhesion molecule binding|GO:0008201,heparin binding|GO:0005488,binding|GO:0043169,cation binding|GO:0046872,metal ion binding|GO:0070492,oligosaccharide binding|GO:0005509,calcium ion binding
QSER1			
ARCN1	GO:0033036,macromolecule localization|GO:0006890,retrograde vesicle-mediated transport, Golgi to ER|GO:0016192,vesicle-mediated transport|GO:0046907,intracellular transport|GO:0048193,Golgi vesicle transport|GO:0006810,transport|GO:0045184,establishment of protein localization|GO:0044765,single-organism transport|GO:0008104,protein localization|GO:1902578,single-organism localization|GO:0071702,organic substance transport|GO:0015031,protein transport|GO:1902582,single-organism intracellular transport|GO:0051234,establishment of localization|GO:0051649,establishment of localization in cell|GO:0051179,localization|GO:0044699,single-organism process|GO:0051641,cellular localization|GO:0042886,amide transport|GO:0071705,nitrogen compound transport|GO:0015833,peptide transport	GO:0043229,intracellular organelle|GO:0030117,membrane coat|GO:0005622,intracellular|GO:0030135,coated vesicle|GO:0043226,organelle|GO:0030137,COPI-coated vesicle|GO:0005737,cytoplasm|GO:0030660,Golgi-associated vesicle membrane|GO:0030663,COPI-coated vesicle membrane|GO:0030662,coated vesicle membrane|GO:0016023,cytoplasmic, membrane-bounded vesicle|GO:0031410,cytoplasmic vesicle|GO:0098805,whole membrane|GO:0016020,membrane|GO:0044444,cytoplasmic part|GO:0031988,membrane-bounded vesicle|GO:0044433,cytoplasmic vesicle part|GO:0044431,Golgi apparatus part|GO:0048475,coated membrane|GO:0005794,Golgi apparatus|GO:0098588,bounding membrane of organelle|GO:0005798,Golgi-associated vesicle|GO:0030659,cytoplasmic vesicle membrane|GO:0012505,endomembrane system|GO:0012506,vesicle membrane|GO:0030120,vesicle coat|GO:0030126,COPI vesicle coat|GO:0031982,vesicle|GO:0043234,protein complex|GO:0032991,macromolecular complex|GO:0043231,intracellular membrane-bounded organelle|GO:0044464,cell part|GO:0005623,cell|GO:0000139,Golgi membrane|GO:0044446,intracellular organelle part|GO:0098796,membrane protein complex|GO:0043227,membrane-bounded organelle|GO:0031090,organelle membrane|GO:0044424,intracellular part|GO:0044425,membrane part|GO:0044422,organelle part|GO:0031984,organelle subcompartment|GO:0097708,intracellular vesicle|GO:0098791,Golgi subcompartment	
RRP12		GO:0016020,membrane|GO:0031974,membrane-enclosed lumen|GO:0031975,envelope|GO:0043229,intracellular organelle|GO:0043228,non-membrane-bounded organelle|GO:0043227,membrane-bounded organelle|GO:0043226,organelle|GO:0031224,intrinsic component of membrane|GO:0031090,organelle membrane|GO:0031981,nuclear lumen|GO:0005730,nucleolus|GO:0016021,integral component of membrane|GO:0005635,nuclear envelope|GO:0031965,nuclear membrane|GO:0031967,organelle envelope|GO:0012505,endomembrane system|GO:0043231,intracellular membrane-bounded organelle|GO:0043232,intracellular non-membrane-bounded organelle|GO:0043233,organelle lumen|GO:0044464,cell part|GO:0005623,cell|GO:0005622,intracellular|GO:0044446,intracellular organelle part|GO:0070013,intracellular organelle lumen|GO:0044428,nuclear part|GO:0044424,intracellular part|GO:0044425,membrane part|GO:0005634,nucleus|GO:0044422,organelle part	GO:0097159,organic cyclic compound binding|GO:0044822,poly(A) RNA binding|GO:0005488,binding|GO:0003676,nucleic acid binding|GO:1901363,heterocyclic compound binding|GO:0003723,RNA binding
EIF4G3	GO:0044238,primary metabolic process|GO:0019221,cytokine-mediated signaling pathway|GO:0019222,regulation of metabolic process|GO:0051246,regulation of protein metabolic process|GO:0031323,regulation of cellular metabolic process|GO:1901564,organonitrogen compound metabolic process|GO:0044249,cellular biosynthetic process|GO:0034641,cellular nitrogen compound metabolic process|GO:0043043,peptide biosynthetic process|GO:0023052,signaling|GO:0007165,signal transduction|GO:0007166,cell surface receptor signaling pathway|GO:0042221,response to chemical|GO:1901576,organic substance biosynthetic process|GO:0070887,cellular response to chemical stimulus|GO:0050789,regulation of biological process|GO:0044699,single-organism process|GO:0080090,regulation of primary metabolic process|GO:0044267,cellular protein metabolic process|GO:0051716,cellular response to stimulus|GO:0044260,cellular macromolecule metabolic process|GO:0010608,posttranscriptional regulation of gene expression|GO:2000112,regulation of cellular macromolecule biosynthetic process|GO:0010467,gene expression|GO:0044419,interspecies interaction between organisms|GO:0071704,organic substance metabolic process|GO:0071310,cellular response to organic substance|GO:0065007,biological regulation|GO:0031326,regulation of cellular biosynthetic process|GO:0010468,regulation of gene expression|GO:0034097,response to cytokine|GO:0043604,amide biosynthetic process|GO:0060255,regulation of macromolecule metabolic process|GO:0006446,regulation of translational initiation|GO:0009987,cellular process|GO:0009889,regulation of biosynthetic process|GO:0034248,regulation of cellular amide metabolic process|GO:0006807,nitrogen compound metabolic process|GO:0034645,cellular macromolecule biosynthetic process|GO:0044764,multi-organism cellular process|GO:0044763,single-organism cellular process|GO:0051171,regulation of nitrogen compound metabolic process|GO:0008152,metabolic process|GO:0007154,cell communication|GO:0050794,regulation of cellular process|GO:0009059,macromolecule biosynthetic process|GO:0010033,response to organic substance|GO:0051704,multi-organism process|GO:0044700,single organism signaling|GO:0032268,regulation of cellular protein metabolic process|GO:0044271,cellular nitrogen compound biosynthetic process|GO:1901566,organonitrogen compound biosynthetic process|GO:0043603,cellular amide metabolic process|GO:0071345,cellular response to cytokine stimulus|GO:0050896,response to stimulus|GO:0009058,biosynthetic process|GO:0044237,cellular metabolic process|GO:0043170,macromolecule metabolic process|GO:0019538,protein metabolic process|GO:0010556,regulation of macromolecule biosynthetic process|GO:0016032,viral process|GO:0006417,regulation of translation|GO:0044403,symbiosis, encompassing mutualism through parasitism|GO:0006518,peptide metabolic process|GO:0006413,translational initiation|GO:0006412,translation|GO:0009894,regulation of catabolic process|GO:0009895,negative regulation of catabolic process|GO:0009892,negative regulation of metabolic process|GO:0031329,regulation of cellular catabolic process|GO:0031324,negative regulation of cellular metabolic process|GO:0044248,cellular catabolic process|GO:0048519,negative regulation of biological process|GO:0010506,regulation of autophagy|GO:0031330,negative regulation of cellular catabolic process|GO:0009056,catabolic process|GO:0006914,autophagy|GO:0010507,negative regulation of autophagy|GO:0048523,negative regulation of cellular process	GO:0043234,protein complex|GO:0005737,cytoplasm|GO:0032991,macromolecular complex|GO:0016281,eukaryotic translation initiation factor 4F complex|GO:0044464,cell part|GO:0005623,cell|GO:0005622,intracellular|GO:0044444,cytoplasmic part|GO:0044424,intracellular part|GO:0005829,cytosol	GO:0005488,binding|GO:0000339,RNA cap binding|GO:0044822,poly(A) RNA binding|GO:0008135,translation factor activity, RNA binding|GO:0003723,RNA binding|GO:0003676,nucleic acid binding|GO:0097159,organic cyclic compound binding|GO:1901363,heterocyclic compound binding|GO:0003743,translation initiation factor activity
