# Supplementary material for: Exosomal Circular RNA as a Biomarker Platform for the Early Diagnosis of Immune-Mediated Demyelinating Disease
Source: Front Genet. 2019 Sep 27;10:860. doi: 10.3389/fgene.2019.00860 (PMC6777646; doi:10.3389/fgene.2019.00860)
Supplement: Supplementary Table 3 — KEGG enrichment on main pathways. [file Table_3.pdf]

**Supplementary Table 3**

| Pathway Name | Pathway ID | Pvalue | Pvalue_adjusted | Genes | Count | Pop Hit | List_Total | Background Genes | Class |
|---|---|---|---|---|---|---|---|---|---|
| Cell adhesion molecules (CAMs) | hsa04514 | 8.34E-03 | 1.42E-01 | PTPRF\|1;SELL\|1 | 2 | 146 | 7 | 7057 | Environmental Information Processing |
| Protein export | hsa03060 | 2.26E-02 | 1.60E-01 | SEC11A\|1 | 1 | 23 | 7 | 7057 | Genetic Information Processing |
| Nucleotide excision repair | hsa03420 | 4.67E-02 | 1.60E-01 | RAD23B\|1 | 1 | 48 | 7 | 7057 | Genetic Information Processing |
| Lysine degradation | hsa00310 | 5.05E-02 | 1.60E-01 | KMT2E\|1 | 1 | 52 | 7 | 7057 | Metabolism |
| Viral myocarditis | hsa05416 | 5.80E-02 | 1.60E-01 | EIF4G3\|1 | 1 | 60 | 7 | 7057 | Human Diseases |
| Adherens junction | hsa04520 | 7.12E-02 | 1.60E-01 | PTPRF\|1 | 1 | 74 | 7 | 7057 | Cellular Processes |
| ECM-receptor interaction | hsa04512 | 7.86E-02 | 1.60E-01 | COL1A2\|1 | 1 | 82 | 7 | 7057 | Environmental Information Processing |
| Protein digestion and absorption | hsa04974 | 8.60E-02 | 1.60E-01 | COL1A2\|1 | 1 | 90 | 7 | 7057 | Organismal Systems |
| Amoebiasis | hsa05146 | 9.51E-02 | 1.60E-01 | COL1A2\|1 | 1 | 100 | 7 | 7057 | Human Diseases |
| AGE-RAGE signaling pathway in diabetic complications | hsa04933 | 9.60E-02 | 1.60E-01 | COL1A2\|1 | 1 | 101 | 7 | 7057 | - |
| Insulin resistance | hsa04931 | 1.03E-01 | 1.60E-01 | PTPRF\|1 | 1 | 109 | 7 | 7057 | - |
| Platelet activation | hsa04611 | 1.15E-01 | 1.63E-01 | COL1A2\|1 | 1 | 122 | 7 | 7057 | Organismal Systems |
| Insulin signaling pathway | hsa04910 | 1.30E-01 | 1.70E-01 | PTPRF\|1 | 1 | 139 | 7 | 7057 | Organismal Systems |
| Protein processing in endoplasmic reticulum | hsa04141 | 1.54E-01 | 1.80E-01 | RAD23B\|1 | 1 | 166 | 7 | 7057 | Genetic Information Processing |
| RNA transport | hsa03013 | 1.59E-01 | 1.80E-01 | EIF4G3\|1 | 1 | 172 | 7 | 7057 | Genetic Information Processing |
| Focal adhesion | hsa04510 | 1.85E-01 | 1.96E-01 | COL1A2\|1 | 1 | 203 | 7 | 7057 | Cellular Processes |
| PI3K-Akt signaling pathway | hsa04151 | 2.94E-01 | 2.94E-01 | COL1A2\|1 | 1 | 342 | 7 | 7057 | Environmental Information Processing |
