# Supplementary material for: Exosomal Circular RNA as a Biomarker Platform for the Early Diagnosis of Immune-Mediated Demyelinating Disease
Source: Front Genet. 2019 Sep 27;10:860. doi: 10.3389/fgene.2019.00860 (PMC6777646; doi:10.3389/fgene.2019.00860)
Supplement: Supplementary Table 4 — Clinical information and laboratory data of the patients/control samples employed for RT-qPCR validation. [file Table_4.pdf]

Supplementary Table 4

Clinical information and laboratory data of the patients/control samples employed for RT-qPCR validation.

Factor	Immune-mediated demyelinating disease patients (n=5)	Control samples (n=5)
**Age (years)**		
Mean±SD	44.20±15.45	33.40±13.39
Range	25-64	20-55
Median	41	33
**Sex**		
Male	2	2
Female	3	3
**Demyelinating type**		
MS	4	-
GBS	1	-
**Disease stage**		
Acute stage	5	-
Remission stage	0	-
**Disease progression**		
First-episode	5	-
Recurrent	0	-
**Treatment**		
Yes	0	-
No	5	-
**Disease duration (days)**	15.80±8.38	-
**Laboratory data**		
CSF red blood cells		
Negative	5	5
Positive	0	0

CSF leukocyte count (×10^6/L)	5.30±2.77	2.80±1.92
CSF glucose (mmol/L)	3.62±0.63	3.24±0.58
CSF protein (g/L)	073±0.52	0.48±0.64
CSF chloride (mmol/L)	121.80±3.96	122.60±2.41
Thyroid stimulating hormone (TSH) (mIU/L)	2.74±1.19	2.77±0.91
Free triiodothyronine (FT3) (pmol/L)	5.12±0.57	4.40±0.70
Free thyroxine (FT4) (pmol/L)	13.76±1.61	14.40±2.17
C-reactive protein (CRP) (mg/L)	30.76±8.44	19.30±14.98
Folic acid (FOL) (ng/ml)	17.20±7.98	25.80±11.50
Vitamin B12 (VB12) (pg/ml)	507.80±225.24	413.80±88.30
Homocysteine (HCY) (μmol/L)	39.80±14.31	24.20±15.02
CSF IgG (oligoclonal bands)		
Negative	0	5
Positive	5	0
Serum IgG (oligoclonal bands)		
Negative	5	5
Positive	0	0
