# Supplementary material for: Exosomal Circular RNA as a Biomarker Platform for the Early Diagnosis of Immune-Mediated Demyelinating Disease
Source: Front Genet. 2019 Sep 27;10:860. doi: 10.3389/fgene.2019.00860 (PMC6777646; doi:10.3389/fgene.2019.00860)
Supplement: Supplementary Table 5 — GO analysis on potential target genes that may be regulated by differentially expressed circRNAs. [file Table_5.pdf]

Supplementary Table 5

<table>
<tr><th>ID</th><th>Biological Process</th><th>Cell Component</th><th>Molecular Function</th></tr>
<tr><td>GABRA4</td><td>GO:0042391,regulation of membrane potential|GO:0006810,transport|GO:0048583,regulation of response to stimulus|GO:0006820,anion transport|GO:0006821,chloride transport|GO:0023052,signaling|GO:0007165,signal transduction|GO:0042221,response to chemical|GO:0051179,localization|GO:0007275,multicellular organism development|GO:0044699,single-organism process|GO:0007417,central nervous system development|GO:0051716,cellular response to stimulus|GO:0007214,gamma-aminobutyric acid signaling pathway|GO:0050789,regulation of biological process|GO:0065007,biological regulation|GO:0098661,inorganic anion transmembrane transport|GO:0098660,inorganic ion transmembrane transport|GO:0015698,inorganic anion transport|GO:0065008,regulation of biological quality|GO:0007186,G-protein coupled receptor signaling pathway|GO:0050877,neurological system process|GO:0032502,developmental process|GO:0032501,multicellular organismal process|GO:1902476,chloride transmembrane transport|GO:0006811,ion transport|GO:0009987,cellular process|GO:0050794,regulation of cellular process|GO:0044767,single-organism developmental process|GO:0034220,ion transmembrane transport|GO:0044765,single-organism transport|GO:0044763,single-organism cellular process|GO:0007268,chemical synaptic transmission|GO:0007267,cell-cell signaling|GO:0007154,cell communication|GO:2001023,regulation of response to drug|GO:0051234,establishment of localization|GO:0055085,transmembrane transport|GO:1902578,single-organism localization|GO:0003008,system process|GO:0044700,single organism signaling|GO:0044707,single-multicellular organism process|GO:0007399,nervous system development|GO:0050896,response to stimulus|GO:0048856,anatomical structure development|GO:0042493,response to drug|GO:0098656,anion transmembrane transport|GO:0048731,system development</td><td>GO:0098590,plasma membrane region|GO:0034707,chloride channel complex|GO:0071944,cell periphery|GO:0034702,ion channel complex|GO:0045202,synapse|GO:0031226,intrinsic component of plasma membrane|GO:0031224,intrinsic component of membrane|GO:0097060,synaptic membrane|GO:0016021,integral component of membrane|GO:0016020,membrane|GO:0097458,neuron part|GO:0044456,synapse part|GO:0030054,cell junction|GO:0043005,neuron projection|GO:0044459,plasma membrane part|GO:1902710,GABA receptor complex|GO:1902711,GABA-A receptor complex|GO:1902495,transmembrane transporter complex|GO:0098589,membrane region|GO:1990351,transporter complex|GO:0045211,postsynaptic membrane|GO:0005887,integral component of plasma membrane|GO:0005886,plasma membrane|GO:0042995,cell projection|GO:0043234,protein complex|GO:0043235,receptor complex|GO:0032991,macromolecular complex|GO:0044464,cell part|GO:0005623,cell|GO:0098796,membrane protein complex|GO:0098794,postsynapse|GO:0044425,membrane part|GO:0098805,whole membrane</td><td>GO:0038023,signaling receptor activity|GO:0016917,GABA receptor activity|GO:0060089,molecular transducer activity|GO:0004872,receptor activity|GO:0004871,signal transducer activity|GO:0008503,benzodiazepine receptor activity|GO:0008509,anion transmembrane transporter activity|GO:0015276,ligand-gated ion channel activity|GO:0022857,transmembrane transporter activity|GO:0015103,inorganic anion transmembrane transporter activity|GO:0004890,GABA-A receptor activity|GO:0005253,anion channel activity|GO:0005254,chloride channel activity|GO:0015108,chloride transmembrane transporter activity|GO:0030594,neurotransmitter receptor activity|GO:0005215,transporter activity|GO:0005230,extracellular ligand-gated ion channel activity|GO:0005216,ion channel activity|GO:0005237,inhibitory extracellular ligand-gated ion channel activity|GO:0022803,passive transmembrane transporter activity|GO:0022891,substrate-specific transmembrane transporter activity|GO:0022892,substrate-specific transporter activity|GO:0015075,ion transmembrane transporter activity|GO:0004888,transmembrane signaling receptor activity|GO:0015267,channel activity|GO:0022836,gated channel activity|GO:0022834,ligand-gated channel activity|GO:0022838,substrate-specific channel activity|GO:0099600,transmembrane receptor activity</td></tr>
<tr><td>POU2F1</td><td>GO:0048856,anatomical structure development|GO:0009892,negative regulation of metabolic process|GO:0080090,regulation of primary metabolic process|GO:0009890,negative regulation of biosynthetic process|GO:0061008,hepaticobiliary system development|GO:0031327,negative regulation of cellular biosynthetic process|GO:0031326,regulation of cellular biosynthetic process|GO:0031324,negative regulation of cellular metabolic process|GO:0031323,regulation of cellular metabolic process|GO:0090304,nucleic acid metabolic process|GO:0044249,cellular biosynthetic process|GO:0010629,negative regulation of gene expression|GO:0006807,nitrogen compound metabolic process|GO:0001889,liver development|GO:0034645,cellular macromolecule biosynthetic process|GO:1901576,organic substance biosynthetic process|GO:1901362,organic cyclic compound biosynthetic process|GO:0050789,regulation of biological process|GO:0044699,single-organism process|GO:0032774,RNA biosynthetic process|GO:0006139,nucleobase-containing compound metabolic process|GO:0010605,negative regulation of macromolecule metabolic process|GO:0019222,regulation of metabolic process|GO:0044260,cellular macromolecule metabolic process|GO:0071704,organic substance metabolic process|GO:0031099,regeneration|GO:1901360,organic cyclic compound metabolic process|GO:2000112,regulation of cellular macromolecule biosynthetic process|GO:2000113,negative regulation of cellular macromolecule biosynthetic process|GO:0007275,multicellular organism development|GO:0060255,regulation of macromolecule metabolic process|GO:0010467,gene expression|GO:0048513,animal organ development|GO:0065007,biological regulation|GO:0097659,nucleic acid-templated transcription|GO:0048519,negative regulation of biological process|GO:0006383,transcription from RNA polymerase III promoter|GO:0010468,regulation of gene expression|GO:0018130,heterocycle biosynthetic process|GO:0032502,developmental process|GO:0032501,multicellular organismal process|GO:0045934,negative regulation of nucleobase-containing compound metabolic process|GO:0008152,metabolic process|GO:0031100,animal organ regeneration|GO:0019219,regulation of nucleobase-containing compound metabolic process|GO:0006725,cellular aromatic compound metabolic process|GO:0009987,cellular process|GO:0009889,regulation of biosynthetic process|GO:1903506,regulation of nucleic acid-templated transcription|GO:1903507,negative regulation of nucleic acid-templated transcription|GO:1902679,negative regulation of RNA biosynthetic process|GO:0009058,biosynthetic process|GO:0009059,macromolecule biosynthetic process|GO:0045892,negative regulation of transcription, DNA-templated|GO:0051171,regulation of nitrogen compound metabolic process|GO:0051172,negative regulation of nitrogen compound metabolic process|GO:2001141,regulation of RNA biosynthetic process|GO:0034654,nucleobase-containing compound biosynthetic process|GO:0050794,regulation of cellular process|GO:0044238,primary metabolic process|GO:0046483,heterocycle metabolic process|GO:0016070,RNA metabolic process|GO:0044767,single-organism developmental process|GO:0044271,cellular nitrogen compound biosynthetic process|GO:0044707,single-multicellular organism process|GO:0019438,aromatic compound biosynthetic process|GO:0097421,liver regeneration|GO:0051253,negative regulation of RNA metabolic process|GO:0051252,regulation of RNA metabolic process|GO:0034641,cellular nitrogen compound metabolic process|GO:0044237,cellular metabolic process|GO:0043170,macromolecule metabolic process|GO:0006355,regulation of transcription, DNA-templated|GO:0010556,regulation of macromolecule biosynthetic process|GO:0006351,transcription, DNA-templated|GO:0048731,system development|GO:0048732,gland development|GO:0010558,negative regulation of macromolecule biosynthetic process|GO:0048523,negative regulation of cellular process|GO:0010604,positive regulation of macromolecule metabolic process|GO:0048518,positive regulation of biological process|GO:0009893,positive regulation of metabolic process|GO:0009891,positive regulation of biosynthetic process|GO:0010628,positive regulation of gene expression|GO:0034660,ncRNA metabolic process|GO:0006366,transcription from RNA polymerase II promoter|GO:0009301,snRNA transcription|GO:0016073,snRNA metabolic process|GO:0010557,positive regulation of macromolecule biosynthetic process|GO:1903508,positive regulation of nucleic acid-templated transcription|GO:0045893,positive regulation of transcription, DNA-templated|GO:0051254,positive regulation of RNA metabolic process|GO:1902680,positive regulation of RNA biosynthetic process|GO:0045944,positive regulation of transcription from RNA polymerase II promoter|GO:0042795,snRNA transcription from RNA polymerase II promoter|GO:0031328,positive regulation of cellular biosynthetic process|GO:0031325,positive regulation of cellular metabolic process|GO:0098781,ncRNA transcription|GO:0006357,regulation of transcription from RNA polymerase II promoter|GO:0045935,positive regulation of nucleobase-containing compound metabolic process|GO:0051173,positive regulation of nitrogen compound metabolic process|GO:0048522,positive regulation of cellular process|GO:0044700,single organism signaling|GO:0051716,cellular response to stimulus|GO:0019221,cytokine-mediated signaling pathway|GO:0071345,cellular response to cytokine stimulus|GO:0050896,response to stimulus|GO:0071310,cellular response to organic substance|GO:0023052,signaling|GO:0007165,signal transduction|GO:0007166,cell surface receptor signaling pathway|GO:0042221,response to chemical|GO:0007154,cell communication|GO:0070887,cellular response to chemical stimulus|GO:0010033,response to organic substance|GO:0034097,response to cytokine</td><td>GO:0044446,intracellular organelle part|GO:0043231,intracellular membrane-bounded organelle|GO:0031981,nuclear lumen|GO:0043233,organelle lumen|GO:0005634,nucleus|GO:0044464,cell part|GO:0031974,membrane-enclosed lumen|GO:0005622,intracellular|GO:0005654,nucleoplasm|GO:0070013,intracellular organelle lumen|GO:0043229,intracellular organelle|GO:0044428,nuclear part|GO:0005623,cell|GO:0044424,intracellular part|GO:0043227,membrane-bounded organelle|GO:0043226,organelle|GO:0044422,organelle part|GO:0005783,endoplasmic reticulum|GO:0005737,cytoplasm|GO:0044798,nuclear transcription factor complex|GO:0012505,endomembrane system|GO:0043234,protein complex|GO:0032991,macromolecular complex|GO:0090575,RNA polymerase II transcription factor complex|GO:0044444,cytoplasmic part|GO:0005667,transcription factor complex</td><td>GO:0044212,transcription regulatory region DNA binding|GO:0005515,protein binding|GO:0043565,sequence-specific DNA binding|GO:0003700,transcription factor activity, sequence-specific DNA binding|GO:0097159,organic cyclic compound binding|GO:0003677,DNA binding|GO:0001067,regulatory region nucleic acid binding|GO:0000976,transcription regulatory region sequence-specific DNA binding|GO:0005488,binding|GO:0003676,nucleic acid binding|GO:0000975,regulatory region DNA binding|GO:0044877,macromolecular complex binding|GO:0005102,receptor binding|GO:0003682,chromatin binding|GO:1901363,heterocyclic compound binding|GO:0001071,nucleic acid binding transcription factor activity|GO:0001012,RNA polymerase II regulatory region DNA binding|GO:1990837,sequence-specific double-stranded DNA binding|GO:0003690,double-stranded DNA binding|GO:0000983,transcription factor activity, RNA polymerase II core promoter sequence-specific|GO:0000981,RNA polymerase II transcription factor activity, sequence-specific DNA binding|GO:0001047,core promoter binding|GO:0001046,core promoter sequence-specific DNA binding|GO:0000977,RNA polymerase II regulatory region sequence-specific DNA binding|GO:0000979,RNA polymerase II core promoter sequence-specific DNA binding|GO:0000987,core promoter proximal region sequence-specific DNA binding|GO:0000978,RNA polymerase II core promoter proximal region sequence-specific DNA binding|GO:0001159,core promoter proximal region DNA binding</td></tr>
<tr><td>REL</td><td>GO:0080090,regulation of primary metabolic process|GO:0019222,regulation of metabolic process|GO:0048584,positive regulation of response to stimulus|GO:0048583,regulation of response to stimulus|GO:0042035,regulation of cytokine biosynthetic process|GO:0007165,signal transduction|GO:1901362,organic cyclic compound biosynthetic process|GO:1901360,organic cyclic compound metabolic process|GO:0051716,cellular response to stimulus|GO:0010605,negative regulation of macromolecule metabolic process|GO:0010604,positive regulation of macromolecule metabolic process|GO:0009966,regulation of signal transduction|GO:0009967,positive regulation of signal transduction|GO:0048518,positive regulation of biological process|GO:0048519,negative regulation of biological process|GO:0043122,regulation of I-kappaB kinase/NF-kappaB signaling|GO:0043123,positive regulation of I-kappaB kinase/NF-kappaB signaling|GO:0032655,regulation of interleukin-12 production|GO:0060255,regulation of macromolecule metabolic process|GO:0060548,negative regulation of cell death|GO:0042221,response to chemical|GO:2001141,regulation of RNA biosynthetic process|GO:0010033,response to organic substance|GO:0046483,heterocycle metabolic process|GO:0044700,single organism signaling|GO:0042089,cytokine biosynthetic process|GO:0044707,single-multicellular organism process|GO:0019538,protein metabolic process|GO:0002376,immune system process|GO:0032735,positive regulation of interleukin-12 production|GO:0019438,aromatic compound biosynthetic process|GO:0009892,negative regulation of metabolic process|GO:0009893,positive regulation of metabolic process|GO:0009890,negative regulation of biosynthetic process|GO:0009891,positive regulation of biosynthetic process|GO:0038061,NIK/NF-kappaB signaling|GO:0032648,regulation of interferon-beta production|GO:0008152,metabolic process|GO:0010629,negative regulation of gene expression|GO:0010628,positive regulation of gene expression|GO:0035556,intracellular signal transduction|GO:0050789,regulation of biological process|GO:0097659,nucleic acid-templated transcription|GO:0045934,negative regulation of nucleobase-containing compound metabolic process|GO:0001816,cytokine production|GO:0042090,interleukin-12 biosynthetic process|GO:0044260,cellular macromolecule metabolic process|GO:0070997,neuron death|GO:0065007,biological regulation|GO:0006366,transcription from RNA polymerase II promoter|GO:0045087,innate immune response|GO:0018130,heterocycle biosynthetic process|GO:0016265,obsolete death|GO:0034097,response to cytokine|GO:0009889,regulation of biosynthetic process|GO:0050794,regulation of cellular process|GO:0006952,defense response|GO:0006950,response to stress|GO:0032480,negative regulation of type I interferon production|GO:0051239,regulation of multicellular organismal process|GO:0006955,immune response|GO:0034654,nucleobase-containing compound biosynthetic process|GO:1902533,positive regulation of intracellular signal transduction|GO:1901214,regulation of neuron death|GO:1901215,negative regulation of neuron death|GO:0016070,RNA metabolic process|GO:0044271,cellular nitrogen compound biosynthetic process|GO:0050896,response to stimulus|GO:0006355,regulation of transcription, DNA-templated|GO:0010557,positive regulation of macromolecule biosynthetic process|GO:0010556,regulation of macromolecule biosynthetic process|GO:0006351,transcription, DNA-templated|GO:0010558,negative regulation of macromolecule biosynthetic process|GO:0006954,inflammatory response|GO:0032774,RNA biosynthetic process|GO:0023056,positive regulation of signaling|GO:0044249,cellular biosynthetic process|GO:0034641,cellular nitrogen compound metabolic process|GO:0023052,signaling|GO:0034645,cellular macromolecule biosynthetic process|GO:0023051,regulation of signaling|GO:0010647,positive regulation of cell communication|GO:0010646,regulation of cell communication|GO:0044699,single-organism process|GO:0006139,nucleobase-containing compound metabolic process|GO:1902531,regulation of intracellular signal transduction|GO:0000122,negative regulation of transcription from RNA polymerase II promoter|GO:0045075,regulation of interleukin-12 biosynthetic process|GO:0051240,positive regulation of multicellular organismal process|GO:0051241,negative regulation of multicellular organismal process|GO:0051246,regulation of protein metabolic process|GO:0051247,positive regulation of protein metabolic process|GO:1903508,positive regulation of nucleic acid-templated transcription|GO:0032501,multicellular organismal process|GO:0009987,cellular process|GO:0006725,cellular aromatic compound metabolic process|GO:1903506,regulation of nucleic acid-templated transcription|GO:1903507,negative regulation of nucleic acid-templated transcription|GO:0045892,negative regulation of transcription, DNA-templated|GO:0045893,positive regulation of transcription, DNA-templated|GO:0051253,negative regulation of RNA metabolic process|GO:0051252,regulation of RNA metabolic process|GO:0007249,I-kappaB kinase/NF-kappaB signaling|GO:0051254,positive regulation of RNA metabolic process|GO:0043170,macromolecule metabolic process|GO:1902680,positive regulation of RNA biosynthetic process|GO:0001817,regulation of cytokine production|GO:0006807,nitrogen compound metabolic process|GO:0045944,positive regulation of transcription from RNA polymerase II promoter|GO:0032479,regulation of type I interferon production|GO:0001818,negative regulation of cytokine production|GO:0001819,positive regulation of cytokine production|GO:0032608,interferon-beta production|GO:0031328,positive regulation of cellular biosynthetic process|GO:0031327,negative regulation of cellular biosynthetic process|GO:0031326,regulation of cellular biosynthetic process|GO:0031325,positive regulation of cellular metabolic process|GO:0031324,negative regulation of cellular metabolic process|GO:0031323,regulation of cellular metabolic process|GO:0032606,type I interferon production|GO:0090304,nucleic acid metabolic process|GO:0008219,cell death|GO:0010941,regulation of cell death|GO:2000112,regulation of cellular macromolecule biosynthetic process|GO:2000113,negative regulation of cellular macromolecule biosynthetic process|GO:0071704,organic substance metabolic process|GO:0010467,gene expression|GO:0006357,regulation of transcription from RNA polymerase II promoter|GO:0010468,regulation of gene expression|GO:0045935,positive regulation of nucleobase-containing compound metabolic process|GO:0042108,positive regulation of cytokine biosynthetic process|GO:1901576,organic substance biosynthetic process|GO:0019219,regulation of nucleobase-containing compound metabolic process|GO:0045084,positive regulation of interleukin-12 biosynthetic process|GO:0042107,cytokine metabolic process|GO:1902679,negative regulation of RNA biosynthetic process|GO:0009058,biosynthetic process|GO:0009059,macromolecule biosynthetic process|GO:0044763,single-organism cellular process|GO:0051171,regulation of nitrogen compound metabolic process|GO:0051172,negative regulation of nitrogen compound metabolic process|GO:0051173,positive regulation of nitrogen compound metabolic process|GO:0007154,cell communication|GO:0032615,interleukin-12 production|GO:0044238,primary metabolic process|GO:0044237,cellular metabolic process|GO:0032688,negative regulation of interferon-beta production|GO:0048523,negative regulation of cellular process|GO:0048522,positive regulation of cellular process</td><td>GO:0031974,membrane-enclosed lumen|GO:0043229,intracellular organelle|GO:0043227,membrane-bounded organelle|GO:0043226,organelle|GO:0005737,cytoplasm|GO:0031981,nuclear lumen|GO:0005634,nucleus|GO:0005654,nucleoplasm|GO:0033256,I-kappaB/NF-kappaB complex|GO:0043234,protein complex|GO:0032991,macromolecular complex|GO:0043231,intracellular membrane-bounded organelle|GO:0043233,organelle lumen|GO:0005829,cytosol|GO:0044464,cell part|GO:0005623,cell|GO:0005622,intracellular|GO:0044446,intracellular organelle part|GO:0070013,intracellular organelle lumen|GO:0044444,cytoplasmic part|GO:0044428,nuclear part|GO:0005667,transcription factor complex|GO:0044424,intracellular part|GO:0044422,organelle part</td><td>GO:0001012,RNA polymerase II regulatory region DNA binding|GO:0001158,enhancer sequence-specific DNA binding|GO:0044877,macromolecular complex binding|GO:0005488,binding|GO:0003676,nucleic acid binding|GO:0003677,DNA binding|GO:1901363,heterocyclic compound binding|GO:0043565,sequence-specific DNA binding|GO:0003705,transcription factor activity, RNA polymerase II distal enhancer sequence-specific binding|GO:0000981,RNA polymerase II transcription factor activity, sequence-specific DNA binding|GO:0000980,RNA polymerase II distal enhancer sequence-specific DNA binding|GO:0001067,regulatory region nucleic acid binding|GO:0035326,enhancer binding|GO:0001205,transcriptional activator activity, RNA polymerase II distal enhancer sequence-specific binding|GO:0044212,transcription regulatory region DNA binding|GO:0003700,transcription factor activity, sequence-specific DNA binding|GO:0097159,organic cyclic compound binding|GO:0001228,transcriptional activator activity, RNA polymerase II transcription regulatory region sequence-specific binding|GO:0000976,transcription regulatory region sequence-specific DNA binding|GO:0000977,RNA polymerase II regulatory region sequence-specific DNA binding|GO:0005515,protein binding|GO:0000975,regulatory region DNA binding|GO:0003682,chromatin binding|GO:0001071,nucleic acid binding transcription factor activity|GO:1990837,sequence-specific double-stranded DNA binding|GO:0003690,double-stranded DNA binding</td></tr>
<tr><td>FZD3</td><td>GO:0048468,cell development|GO:0060322,head development|GO:0007164,establishment of tissue polarity|GO:0007165,signal transduction|GO:0007166,cell surface receptor signaling pathway|GO:0098773,skin epidermis development|GO:0071840,cellular component organization or biogenesis|GO:0051716,cellular response to stimulus|GO:0045786,negative regulation of cell cycle|GO:0042330,taxis|GO:0048869,cellular developmental process|GO:0048513,animal organ development|GO:0010720,positive regulation of cell development|GO:0048518,positive regulation of biological process|GO:0048519,negative regulation of biological process|GO:0043583,ear development|GO:0042127,regulation of cell proliferation|GO:0048729,tissue morphogenesis|GO:0061548,ganglion development|GO:0044700,single organism signaling|GO:0060548,negative regulation of cell death|GO:0045992,negative regulation of embryonic development|GO:0045995,regulation of embryonic development|GO:0097485,neuron projection guidance|GO:0035567,non-canonical Wnt signaling pathway|GO:0014020,primary neural tube formation|GO:0009605,response to external stimulus|GO:0044707,single-multicellular organism process|GO:0048870,cell motility|GO:0016055,Wnt signaling pathway|GO:0048666,neuron development|GO:0048562,embryonic organ morphogenesis|GO:0048667,cell morphogenesis involved in neuron differentiation|GO:0048568,embryonic organ development|GO:0006928,movement of cell or subcellular component|GO:0048485,sympathetic nervous system development|GO:0001764,neuron migration|GO:0048483,autonomic nervous system development|GO:0031175,neuron projection development|GO:0051179,localization|GO:0042981,regulation of apoptotic process|GO:0050789,regulation of biological process|GO:0007346,regulation of mitotic cell cycle|GO:0000904,cell morphogenesis involved in differentiation|GO:0000902,cell morphogenesis|GO:0036342,post-anal tail morphogenesis|GO:1900118,negative regulation of execution phase of apoptosis|GO:0065007,biological regulation|GO:0044699,single-organism process|GO:2000648,positive regulation of stem cell proliferation|GO:0001841,neural tube formation|GO:0001843,neural tube closure|GO:0016477,cell migration|GO:0007186,G-protein coupled receptor signaling pathway|GO:1900117,regulation of execution phase of apoptosis|GO:0048646,anatomical structure formation involved in morphogenesis|GO:0016265,obsolete death|GO:0009887,animal organ morphogenesis|GO:0061564,axon development|GO:0050793,regulation of developmental process|GO:0009888,tissue development|GO:0050794,regulation of cellular process|GO:0060606,tube closure|GO:0012501,programmed cell death|GO:0051239,regulation of multicellular organismal process|GO:0042633,hair cycle|GO:0042303,molting cycle|GO:0007420,brain development|GO:0007423,sensory organ development|GO:0060070,canonical Wnt signaling pathway|GO:0050896,response to stimulus|GO:0051962,positive regulation of nervous system development|GO:0051960,regulation of nervous system development|GO:0071679,commissural neuron axon guidance|GO:0001942,hair follicle development|GO:0030154,cell differentiation|GO:0035148,tube formation|GO:0009790,embryo development|GO:0009792,embryo development ending in birth or egg hatching|GO:0023052,signaling|GO:0009794,regulation of mitotic cell cycle, embryonic|GO:0042221,response to chemical|GO:0007411,axon guidance|GO:0008544,epidermis development|GO:0022008,neurogenesis|GO:0007049,cell cycle|GO:0007417,central nervous system development|GO:0050767,regulation of neurogenesis|GO:0009653,anatomical structure morphogenesis|GO:0051240,positive regulation of multicellular organismal process|GO:0051241,negative regulation of multicellular organismal process|GO:0060284,regulation of cell development|GO:0050769,positive regulation of neurogenesis|GO:0030030,cell projection organization|GO:2000177,regulation of neural precursor cell proliferation|GO:0060562,epithelial tube morphogenesis|GO:0043009,chordate embryonic development|GO:0045976,negative regulation of mitotic cell cycle, embryonic|GO:0072091,regulation of stem cell proliferation|GO:0033278,cell proliferation in midbrain|GO:0008284,positive regulation of cell proliferation|GO:0042471,ear morphogenesis|GO:0032501,multicellular organismal process|GO:0035239,tube morphogenesis|GO:0008283,cell proliferation|GO:0060429,epithelium development|GO:0045597,positive regulation of cell differentiation|GO:0045595,regulation of cell differentiation|GO:0040011,locomotion|GO:0007409,axonogenesis|GO:0007405,neuroblast proliferation|GO:0032990,cell part morphogenesis|GO:0051093,negative regulation of developmental process|GO:0048839,inner ear development|GO:0051094,positive regulation of developmental process|GO:0022404,molting cycle process|GO:2000179,positive regulation of neural precursor cell proliferation|GO:0051674,localization of cell|GO:0048731,system development|GO:0072089,stem cell proliferation|GO:0016043,cellular component organization|GO:0032502,developmental process|GO:0016331,morphogenesis of embryonic epithelium|GO:0048812,neuron projection morphogenesis|GO:0097194,execution phase of apoptosis|GO:0072175,epithelial tube formation|GO:0022405,hair cycle process|GO:0045448,mitotic cell cycle, embryonic|GO:0008219,cell death|GO:0010941,regulation of cell death|GO:0007275,multicellular organism development|GO:0006935,chemotaxis|GO:0002009,morphogenesis of an epithelium|GO:0002052,positive regulation of neuroblast proliferation|GO:0061549,sympathetic ganglion development|GO:0032989,cellular component morphogenesis|GO:0043067,regulation of programmed cell death|GO:0043066,negative regulation of apoptotic process|GO:1902692,regulation of neuroblast proliferation|GO:0061351,neural precursor cell proliferation|GO:0043069,negative regulation of programmed cell death|GO:0043588,skin development|GO:0090596,sensory organ morphogenesis|GO:0048598,embryonic morphogenesis|GO:0045930,negative regulation of mitotic cell cycle|GO:0009987,cellular process|GO:0021915,neural tube development|GO:0000278,mitotic cell cycle|GO:0030182,neuron differentiation|GO:0006915,apoptotic process|GO:0044767,single-organism developmental process|GO:0001738,morphogenesis of a polarized epithelium|GO:0044763,single-organism cellular process|GO:0007154,cell communication|GO:0035295,tube development|GO:0001736,establishment of planar polarity|GO:0001838,embryonic epithelial tube formation|GO:0048699,generation of neurons|GO:0042472,inner ear morphogenesis|GO:0048858,cell projection morphogenesis|GO:0007399,nervous system development|GO:0051726,regulation of cell cycle|GO:0048856,anatomical structure development|GO:2000026,regulation of multicellular organismal development|GO:0030901,midbrain development|GO:0048523,negative regulation of cellular process|GO:0048522,positive regulation of cellular process|GO:1904693,midbrain morphogenesis|GO:1905114,cell surface receptor signaling pathway involved in cell-cell signaling|GO:0090175,regulation of establishment of planar polarity|GO:1904938,planar cell polarity pathway involved in axon guidance|GO:0022603,regulation of anatomical structure morphogenesis|GO:0036515,serotonergic neuron axon guidance|GO:0036514,dopaminergic neuron axon guidance|GO:0007223,Wnt signaling pathway, calcium modulating pathway|GO:0060071,Wnt signaling pathway, planar cell polarity pathway|GO:0042493,response to drug|GO:0198738,cell-cell signaling by wnt|GO:0007267,cell-cell signaling|GO:0009628,response to abiotic stimulus|GO:0048854,brain morphogenesis|GO:0051602,response to electrical stimulus|GO:2000027,regulation of organ morphogenesis</td><td>GO:0098590,plasma membrane region|GO:0016020,membrane|GO:0032433,filopodium tip|GO:0030425,dendrite|GO:0030424,axon|GO:0030175,filopodium|GO:0071944,cell periphery|GO:0045202,synapse|GO:0031224,intrinsic component of membrane|GO:0005737,cytoplasm|GO:0045177,apical part of cell|GO:0016021,integral component of membrane|GO:0048786,presynaptic active zone|GO:0097458,neuron part|GO:0044456,synapse part|GO:0043005,neuron projection|GO:0043025,neuronal cell body|GO:0016328,lateral plasma membrane|GO:0016324,apical plasma membrane|GO:0009986,cell surface|GO:0098589,membrane region|GO:0044297,cell body|GO:0036477,somatodendritic compartment|GO:0005886,plasma membrane|GO:0042995,cell projection|GO:0044459,plasma membrane part|GO:0044463,cell projection part|GO:0044464,cell part|GO:0005623,cell|GO:0005622,intracellular|GO:0098793,presynapse|GO:0044424,intracellular part|GO:0044425,membrane part|GO:0098805,whole membrane|GO:0098858,actin-based cell projection</td><td>GO:0005488,binding|GO:0004930,G-protein coupled receptor activity|GO:0038023,signaling receptor activity|GO:0005515,protein binding|GO:0042813,Wnt-activated receptor activity|GO:0060089,molecular transducer activity|GO:0004888,transmembrane signaling receptor activity|GO:0030165,PDZ domain binding|GO:0004872,receptor activity|GO:0004871,signal transducer activity|GO:0017147,Wnt-protein binding|GO:0019904,protein domain specific binding|GO:0099600,transmembrane receptor activity</td></tr>
<tr><td>TMEM229</td><td>GO:0080090,regulation of primary metabolic process|GO:0019222,regulation of metabolic process|GO:0031326,regulation of cellular biosynthetic process|GO:0031323,regulation of cellular metabolic process|GO:0090304,nucleic acid metabolic process|GO:0044249,cellular biosynthetic process|GO:0034641,cellular nitrogen compound metabolic process|GO:0006807,nitrogen compound metabolic process|GO:0034645,cellular macromolecule biosynthetic process|GO:1901362,organic cyclic compound biosynthetic process|GO:0050789,regulation of biological process|GO:0097659,nucleic acid-templated transcription|GO:0032774,RNA biosynthetic process|GO:0006139,nucleobase-containing compound metabolic process|GO:0044260,cellular macromolecule metabolic process|GO:0071704,organic substance metabolic process|GO:2000112,regulation of cellular macromolecule biosynthetic process|GO:0060255,regulation of macromolecule metabolic process|GO:0010467,gene expression|GO:0065007,biological regulation|GO:1901360,organic cyclic compound metabolic process|GO:0010468,regulation of gene expression|GO:0018130,heterocycle biosynthetic process|GO:1901576,organic substance biosynthetic process|GO:0019219,regulation of nucleobase-containing compound metabolic process|GO:0006725,cellular aromatic compound metabolic process|GO:0009987,cellular process|GO:0009889,regulation of biosynthetic process|GO:1903506,regulation of nucleic acid-templated transcription|GO:0050794,regulation of cellular process|GO:0009058,biosynthetic process|GO:0009059,macromolecule biosynthetic process|GO:0051171,regulation of nitrogen compound metabolic process|GO:0008152,metabolic process|GO:2001141,regulation of RNA biosynthetic process|GO:0034654,nucleobase-containing compound biosynthetic process|GO:0046483,heterocycle metabolic process|GO:0016070,RNA metabolic process|GO:0044238,primary metabolic process|GO:0044271,cellular nitrogen compound biosynthetic process|GO:0051252,regulation of RNA metabolic process|GO:0044237,cellular metabolic process|GO:0043170,macromolecule metabolic process|GO:0006355,regulation of transcription, DNA-templated|GO:0010556,regulation of macromolecule biosynthetic process|GO:0006351,transcription, DNA-templated|GO:0019438,aromatic compound biosynthetic process|GO:0019080,viral gene expression|GO:0019083,viral transcription|GO:0050434,positive regulation of viral transcription|GO:0044419,interspecies interaction between organisms|GO:0046782,regulation of viral transcription|GO:0048518,positive regulation of biological process|GO:0051704,multi-organism process|GO:0050792,regulation of viral process|GO:0043902,positive regulation of multi-organism process|GO:0043903,regulation of symbiosis, encompassing mutualism through parasitism|GO:0043900,regulation of multi-organism process|GO:0044403,symbiosis, encompassing mutualism through parasitism|GO:0016032,viral process|GO:0048524,positive regulation of viral process</td><td>GO:0018995,host|GO:0033648,host intracellular membrane-bounded organelle|GO:0044217,other organism part|GO:0044216,other organism cell|GO:0044215,other organism|GO:0043656,intracellular region of host|GO:0016021,integral component of membrane|GO:0016020,membrane|GO:0033646,host intracellular part|GO:0033647,host intracellular organelle|GO:0043657,host cell|GO:0033643,host cell part|GO:0042025,host cell nucleus|GO:0044425,membrane part|GO:0031224,intrinsic component of membrane</td><td>GO:0003700,transcription factor activity, sequence-specific DNA binding|GO:0001071,nucleic acid binding transcription factor activity|GO:0001070,RNA binding transcription factor activity</td></tr>
<tr><td>PSMA8</td><td>GO:0080090,regulation of primary metabolic process|GO:0019222,regulation of metabolic process|GO:0048585,negative regulation of response to stimulus|GO:0048584,positive regulation of response to stimulus|GO:0048583,regulation of response to stimulus|GO:0031349,positive regulation of defense response|GO:0030111,regulation of Wnt signaling pathway|GO:0007165,signal transduction|GO:0007166,cell surface receptor signaling pathway|GO:1902402,signal transduction involved in mitotic DNA damage checkpoint|GO:1902403,signal transduction involved in mitotic DNA integrity checkpoint|GO:1902400,intracellular signal transduction involved in G1 DNA damage checkpoint|GO:0031347,regulation of defense response|GO:0044710,single-organism metabolic process|GO:2000134,negative regulation of G1/S transition of mitotic cell cycle|GO:0010604,positive regulation of macromolecule metabolic process|GO:0009968,negative regulation of signal transduction|GO:0072422,signal transduction involved in DNA damage checkpoint|GO:0009966,regulation of signal transduction|GO:0009967,positive regulation of signal transduction|GO:0070647,protein modification by small protein conjugation or removal|GO:0010467,gene expression|GO:0044419,interspecies interaction between organisms|GO:0032446,protein modification by small protein conjugation|GO:0033238,regulation of cellular amine metabolic process|GO:0043632,modification-dependent macromolecule catabolic process|GO:0044093,positive regulation of molecular function|GO:0048518,positive regulation of biological process|GO:0048519,negative regulation of biological process|GO:0060255,regulation of macromolecule metabolic process|GO:0060548,negative regulation of cell death|GO:0006952,defense response|GO:0050776,regulation of immune response|GO:0030162,regulation of proteolysis|GO:0030163,protein catabolic process|GO:0043436,oxoacid metabolic process|GO:0072413,signal transduction involved in mitotic cell cycle checkpoint|GO:0051704,multi-organism process|GO:0044700,single organism signaling|GO:0002429,immune response-activating cell surface receptor signaling pathway|GO:1901564,organonitrogen compound metabolic process|GO:0019538,protein metabolic process|GO:0022402,cell cycle process|GO:0016055,Wnt signaling pathway|GO:0051351,positive regulation of ligase activity|GO:0050789,regulation of biological process|GO:0016567,protein ubiquitination|GO:0007154,cell communication|GO:0012501,programmed cell death|GO:0033554,cellular response to stress|GO:1901991,negative regulation of mitotic cell cycle phase transition|GO:0044783,G1 DNA damage checkpoint|GO:0060828,regulation of canonical Wnt signaling pathway|GO:1901990,regulation of mitotic cell cycle phase transition|GO:0009896,positive regulation of catabolic process|GO:0090090,negative regulation of canonical Wnt signaling pathway|GO:0009894,regulation of catabolic process|GO:0009893,positive regulation of metabolic process|GO:0090263,positive regulation of canonical Wnt signaling pathway|GO:0030177,positive regulation of Wnt signaling pathway|GO:0065007,biological regulation|GO:0048002,antigen processing and presentation of peptide antigen|GO:0035556,intracellular signal transduction|GO:0072331,signal transduction by p53 class mediator|GO:0030178,negative regulation of Wnt signaling pathway|GO:0072401,signal transduction involved in DNA integrity checkpoint|GO:0000278,mitotic cell cycle|GO:0090068,positive regulation of cell cycle process|GO:0007346,regulation of mitotic cell cycle|GO:0051340,regulation of ligase activity|GO:0051347,positive regulation of transferase activity|GO:0002764,immune response-regulating signaling pathway|GO:0031570,DNA integrity checkpoint|GO:0031571,mitotic G1 DNA damage checkpoint|GO:0002768,immune response-regulating cell surface receptor signaling pathway|GO:0002684,positive regulation of immune system process|GO:0002682,regulation of immune system process|GO:0007049,cell cycle|GO:0044267,cellular protein metabolic process|GO:0065009,regulation of molecular function|GO:0044281,small molecule metabolic process|GO:0000082,G1/S transition of mitotic cell cycle|GO:0016265,obsolete death|GO:0019882,antigen processing and presentation|GO:0019884,antigen processing and presentation of exogenous antigen|GO:0009308,amine metabolic process|GO:0050790,regulation of catalytic activity|GO:0051716,cellular response to stimulus|GO:0050794,regulation of cellular process|GO:0007050,cell cycle arrest|GO:0060070,canonical Wnt signaling pathway|GO:0043412,macromolecule modification|GO:0036211,protein modification process|GO:0008152,metabolic process|GO:0006955,immune response|GO:0045786,negative regulation of cell cycle|GO:0034641,cellular nitrogen compound metabolic process|GO:1901988,negative regulation of cell cycle phase transition|GO:0045787,positive regulation of cell cycle|GO:0051603,proteolysis involved in cellular protein catabolic process|GO:0002757,immune response-activating signal transduction|GO:0051443,positive regulation of ubiquitin-protein transferase activity|GO:0080134,regulation of response to stress|GO:0031401,positive regulation of protein modification process|GO:0002758,innate immune response-activating signal transduction|GO:0010499,proteasomal ubiquitin-independent protein catabolic process|GO:0051338,regulation of transferase activity|GO:0002218,activation of innate immune response|GO:0006511,ubiquitin-dependent protein catabolic process|GO:0010564,regulation of cell cycle process|GO:0051247,positive regulation of protein metabolic process|GO:0023056,positive regulation of signaling|GO:0023057,negative regulation of signaling|GO:0042176,regulation of protein catabolic process|GO:0023052,signaling|GO:0010648,negative regulation of cell communication|GO:0023051,regulation of signaling|GO:0010647,positive regulation of cell communication|GO:0010646,regulation of cell communication|GO:0044819,mitotic G1/S transition checkpoint|GO:0000209,protein polyubiquitination|GO:0043085,positive regulation of catalytic activity|GO:0043161,proteasome-mediated ubiquitin-dependent protein catabolic process|GO:0072395,signal transduction involved in cell cycle checkpoint|GO:0051246,regulation of protein metabolic process|GO:0010565,regulation of cellular ketone metabolic process|GO:1903320,regulation of protein modification by small protein conjugation or removal|GO:0002376,immune system process|GO:1903322,positive regulation of protein modification by small protein conjugation or removal|GO:0032270,positive regulation of cellular protein metabolic process|GO:0031398,positive regulation of protein ubiquitination|GO:0031399,regulation of protein modification process|GO:0031396,regulation of protein ubiquitination|GO:0006508,proteolysis|GO:1901987,regulation of cell cycle phase transition|GO:0002220,innate immune response activating cell surface receptor signaling pathway|GO:0002223,stimulatory C-type lectin receptor signaling pathway|GO:0042590,antigen processing and presentation of exogenous peptide antigen via MHC class I|GO:1903052,positive regulation of proteolysis involved in cellular protein catabolic process|GO:0031331,positive regulation of cellular catabolic process|GO:1903050,regulation of proteolysis involved in cellular protein catabolic process|GO:0009987,cellular process|GO:0019941,modification-dependent protein catabolic process|GO:0031145,anaphase-promoting complex-dependent catabolic process|GO:0044260,cellular macromolecule metabolic process|GO:0006974,cellular response to DNA damage stimulus|GO:0044106,cellular amine metabolic process|GO:0006977,DNA damage response, signal transduction by p53 class mediator resulting in cell cycle arrest|GO:1903047,mitotic cell cycle process|GO:0044257,cellular protein catabolic process|GO:0016032,viral process|GO:0044764,multi-organism cellular process|GO:0044699,single-organism process|GO:0032268,regulation of cellular protein metabolic process|GO:2000045,regulation of G1/S transition of mitotic cell cycle|GO:0006082,organic acid metabolic process|GO:0051726,regulation of cell cycle|GO:0050778,positive regulation of immune response|GO:0006950,response to stress|GO:0043170,macromolecule metabolic process|GO:0006807,nitrogen compound metabolic process|GO:0045862,positive regulation of proteolysis|GO:0050896,response to stimulus|GO:0051438,regulation of ubiquitin-protein transferase activity|GO:0031329,regulation of cellular catabolic process|GO:0010498,proteasomal protein catabolic process|GO:0031325,positive regulation of cellular metabolic process|GO:0031323,regulation of cellular metabolic process|GO:0019752,carboxylic acid metabolic process|GO:0044770,cell cycle phase transition|GO:0010948,negative regulation of cell cycle process|GO:0044773,mitotic DNA damage checkpoint|GO:0051439,regulation of ubiquitin-protein ligase activity involved in mitotic cell cycle|GO:2000060,positive regulation of protein ubiquitination involved</td><td>GO:0019773,proteasome core complex, alpha-subunit complex|GO:0043229,intracellular organelle|GO:0000502,proteasome complex|GO:0043227,membrane-bounded organelle|GO:0043226,organelle|GO:0005737,cytoplasm|GO:0031982,vesicle|GO:0005634,nucleus|GO:0031988,membrane-bounded vesicle|GO:1990111,spermatoproteasome complex|GO:0032991,macromolecular complex|GO:1903561,extracellular vesicle|GO:0005839,proteasome core complex|GO:0043234,protein complex|GO:0043230,extracellular organelle|GO:0043231,intracellular membrane-bounded organelle|GO:0070062,extracellular exosome|GO:0005829,cytosol|GO:0044464,cell part|GO:0005623,cell|GO:0005622,intracellular|GO:0044444,cytoplasmic part|GO:0005576,extracellular region|GO:0044424,intracellular part|GO:0065010,extracellular membrane-bounded organelle|GO:0044421,extracellular region part</td><td>GO:0016787,hydrolase activity|GO:0004175,endopeptidase activity|GO:0004298,threonine-type endopeptidase activity|GO:0003824,catalytic activity|GO:0070003,threonine-type peptidase activity|GO:0070011,peptidase activity, acting on L-amino acid peptides|GO:0008233,peptidase activity</td></tr>
</table>

GNG12	GO:0021537,telencephalon development|GO:0071495,cellular response to endogenous stimulus|GO:0044707,single-multicellular organism process|GO:0033762,response to glucagon|GO:0050896,response to stimulus|GO:1901652,response to peptide|GO:0060322,head development|GO:0023052,signaling|GO:0007165,signal transduction|GO:0070887,cellular response to chemical stimulus|GO:0042221,response to chemical|GO:0006091,generation of precursor metabolites and energy|GO:0021987,cerebral cortex development|GO:0044699,single-organism process|GO:0007417,central nervous system development|GO:0009719,response to endogenous stimulus|GO:0044710,single-organism metabolic process|GO:0071375,cellular response to peptide hormone stimulus|GO:0043207,response to external biotic stimulus|GO:0071377,cellular response to glucagon stimulus|GO:0033993,response to lipid|GO:0071417,cellular response to organonitrogen compound|GO:0009617,response to bacterium|GO:0050789,regulation of biological process|GO:0071310,cellular response to organic substance|GO:0048513,animal organ development|GO:0006112,energy reserve metabolic process|GO:0065007,biological regulation|GO:0051704,multi-organism process|GO:0007186,G-protein coupled receptor signaling pathway|GO:0009605,response to external stimulus|GO:0032502,developmental process|GO:1901699,cellular response to nitrogen compound|GO:0032501,multicellular organismal process|GO:0032496,response to lipopolysaccharide|GO:1901701,cellular response to oxygen-containing compound|GO:0009987,cellular process|GO:0051716,cellular response to stimulus|GO:0050794,regulation of cellular process|GO:0044767,single-organism developmental process|GO:0021543,pallium development|GO:0032870,cellular response to hormone stimulus|GO:0044763,single-organism cellular process|GO:1901700,response to oxygen-containing compound|GO:0008152,metabolic process|GO:0007268,chemical synaptic transmission|GO:0007267,cell-cell signaling|GO:0007154,cell communication|GO:0043434,response to peptide hormone|GO:0051707,response to other organism|GO:0010033,response to organic substance|GO:0055114,oxidation-reduction process|GO:0015980,energy derivation by oxidation of organic compounds|GO:0002237,response to molecule of bacterial origin|GO:0044700,single organism signaling|GO:0009607,response to biotic stimulus|GO:0007420,brain development|GO:0007399,nervous system development|GO:0009725,response to hormone|GO:0010243,response to organonitrogen compound|GO:0048856,anatomical structure development|GO:0044237,cellular metabolic process|GO:0030900,forebrain development|GO:1901653,cellular response to peptide|GO:0007275,multicellular organism development|GO:0048731,system development|GO:1901698,response to nitrogen compound|GO:0044281,small molecule metabolic process	GO:0019897,extrinsic component of plasma membrane|GO:0015629,actin cytoskeleton|GO:0009898,cytoplasmic side of plasma membrane|GO:0019898,extrinsic component of membrane|GO:0043229,intracellular organelle|GO:0043228,non-membrane-bounded organelle|GO:0098552,side of membrane|GO:0043227,membrane-bounded organelle|GO:0043226,organelle|GO:0005856,cytoskeleton|GO:0044446,intracellular organelle part|GO:0031982,vesicle|GO:0005834,heterotrimeric G-protein complex|GO:0016020,membrane|GO:0031988,membrane-bounded vesicle|GO:0044430,cytoskeletal part|GO:0044459,plasma membrane part|GO:0005576,extracellular region|GO:1902494,catalytic complex|GO:0098562,cytoplasmic side of membrane|GO:0005884,actin filament|GO:0005886,plasma membrane|GO:0032991,macromolecular complex|GO:1903561,extracellular vesicle|GO:0031234,extrinsic component of cytoplasmic side of plasma membrane|GO:0070062,extracellular exosome|GO:0043234,protein complex|GO:0043230,extracellular organelle|GO:0043232,intracellular non-membrane-bounded organelle|GO:0044464,cell part|GO:0005623,cell|GO:0005622,intracellular|GO:0098797,plasma membrane protein complex|GO:0098796,membrane protein complex|GO:0071944,cell periphery|GO:0044424,intracellular part|GO:0044425,membrane part|GO:0065010,extracellular membrane-bounded organelle|GO:0044421,extracellular region part|GO:0044422,organelle part|GO:0099512,supramolecular fiber|GO:0099513,polymeric cytoskeletal fiber	GO:0043168,anion binding|GO:0005515,protein binding|GO:0043167,ion binding|GO:0060089,molecular transducer activity|GO:0030165,PDZ domain binding|GO:0019904,protein domain specific binding|GO:0004871,signal transducer activity|GO:0042301,phosphate ion binding|GO:0005488,binding|GO:0016787,hydrolase activity|GO:0016818,hydrolase activity, acting on acid anhydrides, in phosphorus-containing anhydrides|GO:0003824,catalytic activity|GO:0003924,GTPase activity|GO:0017111,nucleoside-triphosphatase activity|GO:0016817,hydrolase activity, acting on acid anhydrides|GO:0016462,pyrophosphatase activity
KPNA4	GO:0034504,protein localization to nucleus|GO:0010035,response to inorganic substance|GO:0051169,nuclear transport|GO:0008104,protein localization|GO:0019221,cytokine-mediated signaling pathway|GO:0051170,nuclear import|GO:0000302,response to reactive oxygen species|GO:0023052,signaling|GO:0007165,signal transduction|GO:0007166,cell surface receptor signaling pathway|GO:0007154,cell communication|GO:0070887,cellular response to chemical stimulus|GO:0050789,regulation of biological process|GO:0044699,single-organism process|GO:0051716,cellular response to stimulus|GO:0051234,establishment of localization|GO:0042221,response to chemical|GO:0070727,cellular macromolecule localization|GO:0006886,intracellular protein transport|GO:0010033,response to organic substance|GO:0051641,cellular localization|GO:0006979,response to oxidative stress|GO:0071310,cellular response to organic substance|GO:0065007,biological regulation|GO:0006810,transport|GO:0071702,organic substance transport|GO:0006913,nucleocytoplasmic transport|GO:0033036,macromolecule localization|GO:0034613,cellular protein localization|GO:0009987,cellular process|GO:0034097,response to cytokine|GO:1902593,single-organism nuclear import|GO:0072594,establishment of protein localization to organelle|GO:0017038,protein import|GO:0006606,protein import into nucleus|GO:0006607,NLS-bearing protein import into nucleus|GO:0050794,regulation of cellular process|GO:0045184,establishment of protein localization|GO:0033365,protein localization to organelle|GO:0044765,single-organism transport|GO:0044763,single-organism cellular process|GO:0051649,establishment of localization in cell|GO:0044744,protein targeting to nucleus|GO:0006605,protein targeting|GO:0051179,localization|GO:1902578,single-organism localization|GO:0016482,cytosolic transport|GO:1901700,response to oxygen-containing compound|GO:0044700,single organism signaling|GO:0046907,intracellular transport|GO:0042542,response to hydrogen peroxide|GO:0071345,cellular response to cytokine stimulus|GO:0050896,response to stimulus|GO:0006950,response to stress|GO:0015031,protein transport|GO:1902582,single-organism intracellular transport|GO:1902580,single-organism cellular localization|GO:0019048,modulation by virus of host morphology or physiology|GO:0015833,peptide transport|GO:0044403,symbiosis, encompassing mutualism through parasitism|GO:0044419,interspecies interaction between organisms|GO:0042886,amide transport|GO:0075733,intracellular transport of virus|GO:0071705,nitrogen compound transport|GO:0019058,viral life cycle|GO:0051817,modification of morphology or physiology of other organism involved in symbiotic interaction|GO:0051704,multi-organism process|GO:0019054,modulation by virus of host process|GO:0044766,multi-organism transport|GO:0051701,interaction with host|GO:1902579,multi-organism localization|GO:0044068,modulation by symbiont of host cellular process|GO:0046794,transport of virus|GO:0016032,viral process|GO:1902583,multi-organism intracellular transport|GO:0044003,modification by symbiont of host morphology or physiology|GO:0035821,modification of morphology or physiology of other organism	GO:0031974,membrane-enclosed lumen|GO:0043229,intracellular organelle|GO:0043227,membrane-bounded organelle|GO:0043226,organelle|GO:0005737,cytoplasm|GO:0070062,extracellular exosome|GO:0031981,nuclear lumen|GO:0005634,nucleus|GO:0031988,membrane-bounded vesicle|GO:0005654,nucleoplasm|GO:1903561,extracellular vesicle|GO:0031982,vesicle|GO:0043230,extracellular organelle|GO:0043231,intracellular membrane-bounded organelle|GO:0043233,organelle lumen|GO:0005829,cytosol|GO:0044464,cell part|GO:0005623,cell|GO:0005622,intracellular|GO:0044446,intracellular organelle part|GO:0070013,intracellular organelle lumen|GO:0044444,cytoplasmic part|GO:0005576,extracellular region|GO:0044428,nuclear part|GO:0044424,intracellular part|GO:0065010,extracellular membrane-bounded organelle|GO:0044421,extracellular region part|GO:0044422,organelle part|GO:0044217,other organism part|GO:0031975,envelope|GO:0044216,other organism cell|GO:0005635,nuclear envelope|GO:0043657,host cell|GO:0018995,host|GO:0031967,organelle envelope|GO:0012505,endomembrane system|GO:0032991,macromolecular complex|GO:0043234,protein complex|GO:0044215,other organism|GO:0005643,nuclear pore|GO:0016020,membrane|GO:0031965,nuclear membrane|GO:0031090,organelle	GO:0008565,protein transporter activity|GO:0022892,substrate-specific transporter activity|GO:0005215,transporter activity|GO:0005488,binding|GO:0005515,protein binding|GO:0042277,peptide binding|GO:0005048,signal sequence binding|GO:0008139,nuclear localization sequence binding|GO:0033218,amide binding|GO:0015197,peptide transporter activity|GO:0061608,nuclear import signal receptor activity|GO:0005487,nucleocytoplasmic transporter activity
COX5A	GO:0009167,purine ribonucleoside monophosphate metabolic process|GO:0044281,small molecule metabolic process|GO:0009161,ribonucleoside monophosphate metabolic process|GO:1901362,organic cyclic compound biosynthetic process|GO:1901360,organic cyclic compound metabolic process|GO:0044710,single-organism metabolic process|GO:0009199,ribonucleoside triphosphate metabolic process|GO:0022900,electron transport chain|GO:0022904,respiratory electron transport chain|GO:0009205,purine ribonucleoside triphosphate metabolic process|GO:0046034,ATP metabolic process|GO:0098660,inorganic ion transmembrane transport|GO:0046128,purine ribonucleoside metabolic process|GO:0055114,oxidation-reduction process|GO:0046483,heterocycle metabolic process|GO:1901564,organonitrogen compound metabolic process|GO:0006163,purine nucleotide metabolic process|GO:0019637,organophosphate metabolic process|GO:0019438,aromatic compound biosynthetic process|GO:0006119,oxidative phosphorylation|GO:0009141,nucleoside triphosphate metabolic process|GO:0009144,purine nucleoside triphosphate metabolic process|GO:0006807,nitrogen compound metabolic process|GO:0097659,nucleic acid-templated transcription|GO:1901576,organic substance biosynthetic process|GO:0015992,proton transport|GO:0044260,cellular macromolecule metabolic process|GO:0098662,inorganic cation transmembrane transport|GO:0006367,transcription initiation from RNA polymerase II promoter|GO:0006366,transcription from RNA polymerase II promoter|GO:0018130,heterocycle biosynthetic process|GO:0006818,hydrogen transport|GO:0006812,cation transport|GO:0006811,ion transport|GO:0006810,transport|GO:0009150,purine ribonucleotide metabolic process|GO:0045333,cellular respiration|GO:0008152,metabolic process|GO:0034654,nucleobase-containing compound biosynthetic process|GO:0051234,establishment of localization|GO:0016070,RNA metabolic process|GO:0044271,cellular nitrogen compound biosynthetic process|GO:0015980,energy derivation by oxidation of organic compounds|GO:0009058,biosynthetic process|GO:0006351,transcription, DNA-templated|GO:0006352,DNA-templated transcription, initiation|GO:0009117,nucleotide metabolic process|GO:0006753,nucleoside phosphate metabolic process|GO:0032774,RNA biosynthetic process|GO:1902600,hydrogen ion transmembrane transport|GO:0016310,phosphorylation|GO:0044249,cellular biosynthetic process|GO:0034641,cellular nitrogen compound metabolic process|GO:0034645,cellular macromolecule biosynthetic process|GO:0009123,nucleoside monophosphate metabolic process|GO:0009126,purine nucleoside monophosphate metabolic process|GO:0009259,ribonucleotide metabolic process|GO:0044699,single-organism process|GO:0006139,nucleobase-containing compound metabolic process|GO:0042278,purine nucleoside metabolic process|GO:0051179,localization|GO:0015672,monovalent inorganic cation transport|GO:0009987,cellular process|GO:0006725,cellular aromatic compound metabolic process|GO:0055085,transmembrane transport|GO:0055086,nucleobase-containing small molecule metabolic process|GO:1901135,carbohydrate derivative metabolic process|GO:0006123,mitochondrial electron transport, cytochrome c to oxygen|GO:0043170,macromolecule metabolic process|GO:0090304,nucleic acid metabolic process|GO:0019693,ribose phosphate metabolic process|GO:0072521,purine-containing compound metabolic process|GO:0006091,generation of precursor metabolites and energy|GO:0071704,organic substance metabolic process|GO:0010467,gene expression|GO:0034220,ion transmembrane transport|GO:0044765,single-organism transport|GO:0009059,macromolecule biosynthetic process|GO:0044763,single-organism cellular process|GO:0009116,nucleoside metabolic process|GO:0009119,ribonucleoside metabolic process|GO:0042775,mitochondrial ATP synthesis coupled electron transport|GO:0042773,ATP synthesis coupled electron transport|GO:1902578,single-organism localization|GO:0044238,primary metabolic process|GO:0044237,cellular metabolic process|GO:1901657,glycosyl compound metabolic process|GO:0006796,phosphate-containing compound metabolic process|GO:0006793,phosphorus metabolic process|GO:0098655,cation transmembrane transport	GO:0044464,cell part|GO:0031975,envelope|GO:0043229,intracellular organelle|GO:0070069,cytochrome complex|GO:0031988,membrane-bounded vesicle|GO:0043227,membrane-bounded organelle|GO:0043226,organelle|GO:0005737,cytoplasm|GO:0005751,mitochondrial respiratory chain complex IV|GO:0031982,vesicle|GO:0098796,membrane protein complex|GO:0043209,myelin sheath|GO:0016020,membrane|GO:0098803,respiratory chain complex|GO:0044444,cytoplasmic part|GO:0005739,mitochondrion|GO:0098800,inner mitochondrial membrane protein complex|GO:0044455,mitochondrial membrane part|GO:0031967,organelle envelope|GO:0031966,mitochondrial membrane|GO:0032991,macromolecular complex|GO:1903561,extracellular vesicle|GO:0031090,organelle membrane|GO:0043234,protein complex|GO:0043230,extracellular organelle|GO:0043231,intracellular membrane-bounded organelle|GO:0070062,extracellular exosome|GO:0019866,organelle inner membrane|GO:0005623,cell|GO:0005622,intracellular|GO:0044446,intracellular organelle part|GO:0005743,mitochondrial inner membrane|GO:0005740,mitochondrial envelope|GO:0005576,extracellular region|GO:0005746,mitochondrial respiratory chain|GO:0044429,mitochondrial part|GO:0044424,intracellular part|GO:0044425,membrane part|GO:0070469,respiratory chain|GO:0045277,respiratory chain complex IV|GO:0065010,extracellular membrane-bounded organelle|GO:0044421,extracellular region part|GO:0044422,organelle part|GO:0098798,mitochondrial protein complex	GO:0005488,binding|GO:0016491,oxidoreductase activity|GO:0043169,cation binding|GO:0015077,monovalent inorganic cation transmembrane transporter activity|GO:0043167,ion binding|GO:0046872,metal ion binding|GO:0015002,heme-copper terminal oxidase activity|GO:0004129,cytochrome-c oxidase activity|GO:0005215,transporter activity|GO:0008324,cation transmembrane transporter activity|GO:0003824,catalytic activity|GO:0009055,electron carrier activity|GO:0022891,substrate-specific transmembrane transporter activity|GO:0022890,inorganic cation transmembrane transporter activity|GO:0022892,substrate-specific transporter activity|GO:0015075,ion transmembrane transporter activity|GO:0022857,transmembrane transporter activity|GO:0016676,oxidoreductase activity, acting on a heme group of donors, oxygen as acceptor|GO:0005515,protein binding|GO:0016675,oxidoreductase activity, acting on a heme group of donors|GO:0015078,hydrogen ion transmembrane transporter activity
TNFRSF11	GO:0032310,prostaglandin secretion|GO:0019220,regulation of phosphate metabolic process|GO:0019221,cytokine-mediated signaling pathway|GO:0019222,regulation of metabolic process|GO:0051047,positive regulation of secretion|GO:0051049,regulation of transport|GO:0071812,positive regulation of fever generation by positive regulation of prostaglandin secretion|GO:0048583,regulation of response to stimulus|GO:0032846,positive regulation of homeostatic process|GO:0032844,regulation of homeostatic process|GO:0031349,positive regulation of defense response|GO:0006820,anion transport|GO:0001503,ossification|GO:0007165,signal transduction|GO:0071848,positive regulation of ERK1 and ERK2 cascade via TNFSF11-mediated signaling|GO:0060326,cell chemotaxis|GO:0071847,TNFSF11-mediated signaling pathway|GO:0051234,establishment of localization|GO:1901362,organic cyclic compound biosynthetic process|GO:0023014,signal transduction by protein phosphorylation|GO:0080090,regulation of primary metabolic process|GO:0044710,single-organism metabolic process|GO:0050729,positive regulation of inflammatory response|GO:0010604,positive regulation of macromolecule metabolic process|GO:0042330,taxis|GO:0009966,regulation of signal transduction|GO:0009967,positive regulation of signal transduction|GO:0006869,lipid transport|GO:0000165,MAPK cascade|GO:0050727,regulation of inflammatory response|GO:0048511,rhythmic process|GO:0048513,animal organ development|GO:0032370,positive regulation of lipid transport|GO:0044093,positive regulation of molecular function|GO:0016310,phosphorylation|GO:0048518,positive regulation of biological process|GO:0002526,acute inflammatory response|GO:0033036,macromolecule localization|GO:0042127,regulation of cell proliferation|GO:0030595,leukocyte chemotaxis|GO:0048584,positive regulation of response to stimulus|GO:0015718,monocarboxylic acid transport|GO:0006935,chemotaxis|GO:0071810,regulation of fever generation by regulation of prostaglandin secretion|GO:0060255,regulation of macromolecule metabolic process|GO:0015849,organic acid transport|GO:0045859,regulation of protein kinase activity|GO:0006952,defense response|GO:0015711,organic anion transport|GO:0046330,positive regulation of JNK cascade|GO:0032268,regulation of cellular protein metabolic process|GO:0042221,response to chemical|GO:2001141,regulation of RNA biosynthetic process|GO:0002673,regulation of acute inflammatory response|GO:0010876,lipid localization|GO:0010033,response to organic substance|GO:0002675,positive regulation of acute inflammatory response|GO:0046483,heterocycle metabolic process|GO:0042325,regulation of phosphorylation|GO:0044700,single organism signaling|GO:0042327,positive regulation of phosphorylation|GO:0065008,regulation of biological quality|GO:0009607,response to biotic stimulus|GO:0044707,single-multicellular organism process|GO:0048871,multicellular organismal homeostasis|GO:0048870,cell motility|GO:0019538,protein metabolic process|GO:0060086,circadian temperature homeostasis|GO:0034641,cellular nitrogen compound metabolic process|GO:0070555,response to interleukin-1|GO:0002376,immune system process|GO:0032305,positive regulation of icosanoid secretion|GO:0032306,regulation of prostaglandin secretion|GO:0033554,cellular response to stress|GO:0032303,regulation of icosanoid secretion|GO:0019438,aromatic compound biosynthetic process|GO:0032308,positive regulation of prostaglandin secretion|GO:0032309,icosanoid secretion|GO:1903793,positive regulation of anion transport|GO:0032496,response to lipopolysaccharide|GO:0007166,cell surface receptor signaling pathway|GO:0009891,positive regulation of biosynthetic process|GO:0006950,response to stress|GO:0006928,movement of cell or subcellular component|GO:0008152,metabolic process|GO:0009314,response to radiation|GO:0010628,positive regulation of gene expression|GO:0006807,nitrogen compound metabolic process|GO:0050896,response to stimulus|GO:0046328,regulation of JNK cascade|GO:0035556,intracellular signal transduction|GO:0071900,regulation of protein serine/threonine kinase activity|GO:0006955,immune response|GO:0050789,regulation of biological process|GO:0097659,nucleic acid-templated transcription|GO:0034097,response to cytokine|GO:0009605,response to external stimulus|GO:0044267,cellular protein metabolic process|GO:0090304,nucleic acid metabolic process|GO:0051347,positive regulation of transferase activity|GO:1901571,fatty acid derivative transport|GO:0071356,cellular response to tumor necrosis factor|GO:0043549,regulation of kinase activity|GO:0015732,prostaglandin transport|GO:0065007,biological regulation|GO:0031347,regulation of defense response|GO:0065009,regulation of molecular function|GO:0016477,cell migration|GO:0030879,mammary gland development|GO:0018130,heterocycle biosynthetic process|GO:0006810,transport|GO:0007623,circadian rhythm|GO:0015908,fatty acid transport|GO:0006139,nucleobase-containing compound metabolic process|GO:0006811,ion transport|GO:0050790,regulation of catalytic activity|GO:0009889,regulation of biosynthetic process|GO:0051716,cellular response to stimulus|GO:0050794,regulation of cellular process|GO:0043410,positive regulation of MAPK cascade|GO:0006953,acute-phase response|GO:0043412,macromolecule modification|GO:0036211,protein modification process|GO:0051239,regulation of multicellular organismal process|GO:0048731,system development|GO:0032890,regulation of organic acid transport|GO:1902533,positive regulation of intracellular signal transduction|GO:0032892,positive regulation of organic acid transport|GO:1902531,regulation of intracellular signal transduction|GO:0044238,primary metabolic process|GO:0002521,leukocyte differentiation|GO:0002520,immune system development|GO:0016070,RNA metabolic process|GO:0044767,single-organism developmental process|GO:0046903,secretion|GO:0070374,positive regulation of ERK1 and ERK2 cascade|GO:0044271,cellular nitrogen compound biosynthetic process|GO:0031649,heat generation|GO:0032368,regulation of lipid transport|GO:0071345,cellular response to cytokine stimulus|GO:0080134,regulation of response to stress|GO:0031401,positive regulation of protein modification process|GO:0009058,biosynthetic process|GO:0051338,regulation of transferase activity|GO:0032502,developmental process|GO:0006355,regulation of transcription, DNA-templated|GO:0010557,positive regulation of macromolecule biosynthetic process|GO:0010556,regulation of macromolecule biosynthetic process|GO:0006351,transcription, DNA-templated|GO:0048869,cellular developmental process|GO:0051046,regulation of secretion|GO:0043207,response to external biotic stimulus|GO:0009611,response to wounding|GO:0051240,positive regulation of multicellular organismal process|GO:0051171,regulation of nitrogen compound metabolic process|GO:0006954,inflammatory response|GO:0009617,response to bacterium|GO:0032103,positive regulation of response to external stimulus|GO:0032101,regulation of response to external stimulus|GO:0044699,single-organism process|GO:0033209,tumor necrosis factor-mediated signaling pathway|GO:0031098,stress-activated protein kinase signaling cascade|GO:0023056,positive regulation of signaling|GO:0043406,positive regulation of MAP kinase activity|GO:0043405,regulation of MAP kinase activity|GO:0023052,signaling|GO:0070887,cellular response to chemical stimulus|GO:0023051,regulation of signaling|GO:0010647,positive regulation of cell communication|GO:0010646,regulation of cell communication|GO:0001660,fever generation|GO:0043085,positive regulation of catalytic activity|GO:0043408,regulation of MAPK cascade|GO:0009893,positive regulation of metabolic process|GO:0031652,positive regulation of heat generation|GO:0007254,JNK cascade|GO:0031650,regulation of heat generation|GO:0051674,localization of cell|GO:0010562,positive regulation of phosphorus metabolic process|GO:0033674,positive regulation of kinase activity|GO:0051246,regulation of protein metabolic process|GO:0051247,positive regulation of protein metabolic process|GO:0010467,gene expression|GO:0032270,positive regulation of cellular protein metabolic process|GO:0031399,regulation of protein modification process|GO:0061377,mammary gland lobule development|GO:1903034,regulation of response to wounding|GO:1903036,positive regulation of response to wounding|GO:1903508,positive regulation of nucleic acid-templated transcription|GO:1901700,response to oxygen-containing compound|GO:0032501,multicellular organismal process|GO:2000193,positive regulation of fatty acid transport|GO:2000191,regulation of fatty acid transport|GO:0070302,regulation of stress-activated protein kinase signaling cascade|GO:0030316,osteoclast	GO:0009897,external side of plasma membrane|GO:0016020,membrane|GO:0016021,integral component of membrane|GO:0098552,side of membrane|GO:0044464,cell part|GO:0005623,cell|GO:0071944,cell periphery|GO:0005886,plasma membrane|GO:0044425,membrane part|GO:0044459,plasma membrane part|GO:0009986,cell surface|GO:0031224,intrinsic component of membrane|GO:0005737,cytoplasm|GO:0031226,intrinsic component of plasma membrane|GO:0005622,intracellular|GO:0044444,cytoplasmic part|GO:0005887,integral component of plasma membrane|GO:0044424,intracellular part|GO:0005829,cytosol	GO:0005488,binding|GO:0043169,cation binding|GO:0038023,signaling receptor activity|GO:0005515,protein binding|GO:0060089,molecular transducer activity|GO:0019955,cytokine binding|GO:0043167,ion binding|GO:0005035,death receptor activity|GO:0004888,transmembrane signaling receptor activity|GO:0005031,tumor necrosis factor-activated receptor activity|GO:0004871,signal transducer activity|GO:0046872,metal ion binding|GO:0004872,receptor activity|GO:0099600,transmembrane receptor activity
TBCA	GO:0022607,cellular component assembly|GO:0006457,protein folding|GO:0070271,protein complex biogenesis|GO:0043933,macromolecular complex subunit organization|GO:0006458,'de novo' protein folding|GO:0034622,cellular macromolecular complex assembly|GO:0071840,cellular component organization or biogenesis|GO:0007021,tubulin complex assembly|GO:0044267,cellular protein metabolic process|GO:0007023,post-chaperonin tubulin folding pathway|GO:0044260,cellular macromolecule metabolic process|GO:0051084,'de novo' posttranslational protein folding|GO:0016043,cellular component organization|GO:0065003,macromolecular complex assembly|GO:0071704,organic substance metabolic process|GO:0009987,cellular process|GO:0006461,protein complex assembly|GO:0008152,metabolic process|GO:0043623,cellular protein complex assembly|GO:0044238,primary metabolic process|GO:0071822,protein complex subunit organization|GO:0019538,protein metabolic process|GO:0044237,cellular metabolic process|GO:0043170,macromolecule metabolic process|GO:0044085,cellular component biogenesis	GO:0031974,membrane-enclosed lumen|GO:0043229,intracellular organelle|GO:0043228,non-membrane-bounded organelle|GO:0005874,microtubule|GO:0043226,organelle|GO:0005856,cytoskeleton|GO:0070062,extracellular exosome|GO:0031981,nuclear lumen|GO:0005730,nucleolus|GO:0043227,membrane-bounded organelle|GO:0005634,nucleus|GO:0031988,membrane-bounded vesicle|GO:0044430,cytoskeletal part|GO:0005737,cytoplasm|GO:0015630,microtubule cytoskeleton|GO:0032991,macromolecular complex|GO:1903561,extracellular vesicle|GO:0031982,vesicle|GO:0043234,protein complex|GO:0043230,extracellular organelle|GO:0043231,intracellular membrane-bounded organelle|GO:0043232,intracellular non-membrane-bounded organelle|GO:0043233,organelle lumen|GO:0044464,cell part|GO:0005623,cell|GO:0005622,intracellular|GO:0044446,intracellular organelle part|GO:0070013,intracellular organelle lumen|GO:0005576,extracellular region|GO:0044428,nuclear part|GO:0044424,intracellular part|GO:0065010,extracellular membrane-bounded organelle|GO:0044421,extracellular region part|GO:0044422,organelle part|GO:0099512,supramolecular fiber|GO:0099513,polymeric cytoskeletal fiber	GO:0015631,tubulin binding|GO:0005515,protein binding|GO:0008092,cytoskeletal protein binding|GO:0097159,organic cyclic compound binding|GO:0044822,poly(A) RNA binding|GO:0051087,chaperone binding|GO:0051082,unfolded protein binding|GO:0003723,RNA binding|GO:0003676,nucleic acid binding|GO:1901363,heterocyclic compound binding|GO:0005488,binding|GO:0048487,beta-tubulin binding
SPACA1	GO:0022607,cellular component assembly|GO:0030154,cell differentiation|GO:0048468,cell development|GO:0019953,sexual reproduction|GO:0010927,cellular component assembly involved in morphogenesis|GO:0007276,gamete generation|GO:0009653,anatomical structure morphogenesis|GO:0044699,single-organism process|GO:0070925,organelle assembly|GO:0000003,reproduction|GO:0048869,cellular developmental process|GO:0071840,cellular component organization or biogenesis|GO:0010256,endomembrane system organization|GO:0016043,cellular component organization|GO:0032989,cellular component morphogenesis|GO:0048515,spermatid differentiation|GO:0048646,anatomical structure formation involved in morphogenesis|GO:0032502,developmental process|GO:0032501,multicellular organismal process|GO:0048609,multicellular organismal reproductive process|GO:0032504,multicellular organism reproduction|GO:0009987,cellular process|GO:0044767,single-organism developmental process|GO:0003006,developmental process involved in reproduction|GO:0007283,spermatogenesis|GO:0044763,single-organism cellular process|GO:0022412,cellular process involved in reproduction in multicellular organism|GO:0048232,male gamete generation|GO:0001675,acrosome assembly|GO:0051704,multi-organism process|GO:0006996,organelle organization|GO:0007281,germ cell development|GO:0044703,multi-organism reproductive process|GO:0044702,single organism reproductive process|GO:0022414,reproductive process|GO:0048856,anatomical structure development|GO:0016050,vesicle organization|GO:1902589,single-organism organelle organization|GO:0044085,cellular component biogenesis|GO:0007286,spermatid development|GO:0033363,secretory granule organization|GO:0051707,response to other organism|GO:0006950,response to stress|GO:0044248,cellular catabolic process|GO:0044036,cell wall macromolecule metabolic process|GO:0044237,cellular metabolic process|GO:0050829,defense response to Gram-negative bacterium|GO:0044260,cellular macromolecule metabolic process|GO:0016998,cell wall macromolecule catabolic process|GO:0009617,response to bacterium|GO:0071554,cell wall organization or biogenesis|GO:0071704,organic substance metabolic process|GO:0006952,defense response|GO:0008152,metabolic process|GO:0009056,catabolic process|GO:0009607,response to biotic stimulus|GO:0043207,response to external biotic stimulus|GO:0009605,response to external stimulus|GO:0050896,response to stimulus|GO:0050830,defense response to Gram-positive bacterium|GO:0043170,macromolecule metabolic process|GO:0098542,defense response to other organism|GO:0042742,defense response to bacterium	GO:0002080,acrosomal membrane|GO:0043229,intracellular organelle|GO:0001669,acrosomal vesicle|GO:0043227,membrane-bounded organelle|GO:0043226,organelle|GO:0031224,intrinsic component of membrane|GO:0005737,cytoplasm|GO:0030667,secretory granule membrane|GO:0031982,vesicle|GO:0016023,cytoplasmic, membrane-bounded vesicle|GO:0031410,cytoplasmic vesicle|GO:0016021,integral component of membrane|GO:0016020,membrane|GO:0097223,sperm part|GO:0031988,membrane-bounded vesicle|GO:0044433,cytoplasmic vesicle part|GO:0030141,secretory granule|GO:0098588,bounding membrane of organelle|GO:0030659,cytoplasmic vesicle membrane|GO:0012505,endomembrane system|GO:0012506,vesicle membrane|GO:0031090,organelle membrane|GO:0043231,intracellular membrane-bounded organelle|GO:0044464,cell part|GO:0005623,cell|GO:0005622,intracellular|GO:0044446,intracellular organelle part|GO:0044444,cytoplasmic part|GO:0044424,intracellular part|GO:0044425,membrane part|GO:0098805,whole membrane|GO:0044422,organelle part|GO:0044421,extracellular region part|GO:0005615,extracellular space|GO:0099503,secretory vesicle|GO:0097708,intracellular vesicle|GO:0005576,extracellular region|GO:0002079,inner acrosomal membrane|GO:0098589,membrane region	GO:0016787,hydrolase activity|GO:0003824,catalytic activity|GO:0016798,hydrolase activity, acting on glycosyl bonds|GO:0003796,lysozyme activity|GO:0004553,hydrolase activity, hydrolyzing O-glycosyl compounds
AC008948			
HSPB11	GO:0032502,developmental process|GO:0030323,respiratory tube development|GO:0030324,lung development|GO:0051234,establishment of localization|GO:0030030,cell projection organization|GO:0030031,cell projection assembly|GO:0010927,cellular component assembly involved in morphogenesis|GO:0001501,skeletal system development|GO:0072359,circulatory system development|GO:0072358,cardiovascular system development|GO:0023052,signaling|GO:0007165,signal transduction|GO:0007166,cell surface receptor signaling pathway|GO:0009798,axis specification|GO:0032989,cellular component morphogenesis|GO:0050789,regulation of biological process|GO:0044699,single-organism process|GO:0007389,pattern specification process|GO:0051716,cellular response to stimulus|GO:0009653,anatomical structure morphogenesis|GO:0000902,cell morphogenesis|GO:0048869,cellular developmental process|GO:0016043,cellular component organization|GO:0007275,multicellular organism development|GO:0008104,protein localization|GO:0048513,animal organ development|GO:0065007,biological regulation|GO:0071840,cellular component organization or biogenesis|GO:0006810,transport|GO:0071702,organic substance transport|GO:0033036,macromolecule localization|GO:0048646,anatomical structure formation involved in morphogenesis|GO:0060541,respiratory system development|GO:0042384,cilium assembly|GO:0022607,cellular component assembly|GO:0060271,cilium morphogenesis|GO:0007507,heart development|GO:0045184,establishment of protein localization|GO:0032501,multicellular organismal process|GO:0009987,cellular process|GO:0050794,regulation of cellular process|GO:0044767,single-organism developmental process|GO:0044763,single-organism cellular process|GO:0003002,regionalization|GO:0007154,cell communication|GO:0035295,tube development|GO:0070925,organelle assembly|GO:0051179,localization|GO:0032990,cell part morphogenesis|GO:0006996,organelle organization|GO:0044700,single organism signaling|GO:0048858,cell projection morphogenesis|GO:0044707,single-multicellular organism process|GO:0007224,smoothened signaling pathway|GO:0050896,response to stimulus|GO:0048856,anatomical structure development|GO:0060972,left/right pattern formation|GO:0070986,left/right axis specification|GO:1902589,single-organism organelle organization|GO:0044085,cellular component biogenesis|GO:0015031,protein transport|GO:0048731,system development|GO:0044782,cilium organization|GO:0030705,cytoskeleton-dependent intracellular transport|GO:0051649,establishment of localization in cell|GO:0042073,intraciliary transport|GO:0070727,cellular macromolecule localization|GO:0006886,intracellular protein transport|GO:0051641,cellular localization|GO:0071705,nitrogen compound transport|GO:0046907,intracellular transport|GO:0034613,cellular protein localization|GO:0031503,protein complex localization|GO:0015833,peptide transport|GO:0042886,amide transport|GO:0044765,single-organism transport|GO:0010970,transport along microtubule|GO:1902582,single-organism intracellular transport|GO:1902578,single-organism localization|GO:0098840,protein transport along microtubule|GO:0035735,intraciliary transport involved in cilium	GO:0042995,cell projection|GO:0043229,intracellular organelle|GO:0043228,non-membrane-bounded organelle|GO:0005929,cilium|GO:0005815,microtubule organizing center|GO:0043227,membrane-bounded organelle|GO:0043226,organelle|GO:0005856,cytoskeleton|GO:0072372,primary cilium|GO:0031982,vesicle|GO:0005813,centrosome|GO:0031988,membrane-bounded vesicle|GO:0044430,cytoskeletal part|GO:0030992,intraciliary transport particle B|GO:0015630,microtubule cytoskeleton|GO:0030990,intraciliary transport particle|GO:0032991,macromolecular complex|GO:1903561,extracellular vesicle|GO:0070062,extracellular exosome|GO:0043234,protein complex|GO:0043230,extracellular organelle|GO:0043232,intracellular non-membrane-bounded organelle|GO:0044463,cell projection part|GO:0044464,cell part|GO:0005623,cell|GO:0005622,intracellular|GO:0044446,intracellular organelle part|GO:0097542,ciliary tip|GO:0005576,extracellular region|GO:0044441,ciliary part|GO:0044424,intracellular part|GO:0065010,extracellular membrane-bounded organelle|GO:0044421,extracellular region part|GO:0044422,organelle part	GO:0043169,cation binding|GO:0043167,ion binding|GO:0005488,binding|GO:0005515,protein binding|GO:0046872,metal ion binding
PDCD2	GO:0080090,regulation of primary metabolic process|GO:0019222,regulation of metabolic process|GO:0010604,positive regulation of macromolecule metabolic process|GO:0048869,cellular developmental process|GO:0043067,regulation of programmed cell death|GO:0048513,animal organ development|GO:0044093,positive regulation of molecular function|GO:0043281,regulation of cysteine-type endopeptidase activity involved in apoptotic process|GO:0002682,regulation of immune system process|GO:0042127,regulation of cell proliferation|GO:0060255,regulation of macromolecule metabolic process|GO:1903708,positive regulation of hemopoiesis|GO:0030162,regulation of proteolysis|GO:1903706,regulation of hemopoiesis|GO:0010467,gene expression|GO:0044707,single-multicellular organism process|GO:0019538,protein metabolic process|GO:0002376,immune system process|GO:0009893,positive regulation of metabolic process|GO:0043170,macromolecule metabolic process|GO:0042981,regulation of apoptotic process|GO:0050789,regulation of biological process|GO:0044267,cellular protein metabolic process|GO:0051345,positive regulation of hydrolase activity|GO:0006919,activation of cysteine-type endopeptidase activity involved in apoptotic process|GO:0002684,positive regulation of immune system process|GO:0065007,biological regulation|GO:0044699,single-organism process|GO:2000648,positive regulation of stem cell proliferation|GO:0065009,regulation of molecular function|GO:0016265,obsolete death|GO:0050793,regulation of developmental process|GO:0050790,regulation of catalytic activity|GO:0050794,regulation of cellular process|GO:0012501,programmed cell death|GO:0051239,regulation of multicellular organismal process|GO:0030097,hemopoiesis|GO:0002520,immune system development|GO:0051336,regulation of hydrolase activity|GO:0051604,protein maturation|GO:0008152,metabolic process|GO:0030154,cell differentiation|GO:0043085,positive regulation of catalytic activity|GO:0051240,positive regulation of multicellular organismal process|GO:0051246,regulation of protein metabolic process|GO:0051247,positive regulation of protein metabolic process|GO:1902035,positive regulation of hematopoietic stem cell proliferation|GO:0032270,positive regulation of cellular protein metabolic process|GO:1902033,regulation of hematopoietic stem cell proliferation|GO:0072091,regulation of stem cell proliferation|GO:0008284,positive regulation of cell proliferation|GO:0032501,multicellular organismal process|GO:0008283,cell proliferation|GO:0009987,cellular process|GO:0048518,positive regulation of biological process|GO:0045595,regulation of cell differentiation|GO:0043280,positive regulation of cysteine-type endopeptidase activity involved in apoptotic process|GO:0016485,protein processing|GO:0006508,proteolysis|GO:0071425,hematopoietic stem cell proliferation|GO:0032268,regulation of cellular protein metabolic process|GO:0051094,positive regulation of developmental process|GO:0045862,positive regulation of proteolysis|GO:0048731,system development|GO:0072089,stem cell proliferation|GO:0032502,developmental process|GO:0031325,positive regulation of cellular metabolic process|GO:0031323,regulation of cellular metabolic process|GO:2001056,positive regulation of cysteine-type endopeptidase activity|GO:0010942,positive regulation of cell death|GO:0008219,cell death|GO:0010941,regulation of cell death|GO:0007275,multicellular organism development|GO:0097202,activation of cysteine-type endopeptidase activity|GO:2000116,regulation of cysteine-type endopeptidase activity|GO:1901532,regulation of hematopoietic progenitor cell differentiation|GO:0031638,zymogen activation|GO:0043065,positive regulation of apoptotic process|GO:0071704,organic substance metabolic process|GO:0002244,hematopoietic progenitor cell differentiation|GO:0048534,hematopoietic or lymphoid organ development|GO:0043068,positive regulation of programmed cell death|GO:0052547,regulation of peptidase activity|GO:0052548,regulation of endopeptidase activity|GO:0006915,apoptotic process|GO:0044767,single-organism developmental process|GO:0044763,single-organism cellular process|GO:0010950,positive regulation of endopeptidase activity|GO:0010952,positive regulation of peptidase activity|GO:0044238,primary metabolic process|GO:0044260,cellular macromolecule metabolic process|GO:0048856,anatomical structure development|GO:0044237,cellular metabolic process|GO:2000026,regulation of multicellular organismal development|GO:0048522,positive regulation of cellular process|GO:1901564,organonitrogen compound metabolic process|GO:0006807,nitrogen compound metabolic process	GO:0043231,intracellular membrane-bounded organelle|GO:0005737,cytoplasm|GO:0005622,intracellular|GO:0043230,extracellular organelle|GO:0031982,vesicle|GO:0044421,extracellular region part|GO:0043227,membrane-bounded organelle|GO:0005634,nucleus|GO:0044464,cell part|GO:0005623,cell|GO:0031988,membrane-bounded vesicle|GO:0043229,intracellular organelle|GO:0005576,extracellular region|GO:0044424,intracellular part|GO:1903561,extracellular vesicle|GO:0065010,extracellular membrane-bounded organelle|GO:0043226,organelle|GO:0070062,extracellular exosome	GO:0043169,cation binding|GO:0005515,protein binding|GO:0097159,organic cyclic compound binding|GO:0019899,enzyme binding|GO:0043167,ion binding|GO:0005488,binding|GO:0003676,nucleic acid binding|GO:0003677,DNA binding|GO:0046872,metal ion binding|GO:1901363,heterocyclic compound binding

<table>
<tr><td></td><td></td><td></td><td></td></tr>
<tr><td>MRPS36</td><td>GO:0043933,macromolecular complex subunit organization|GO:0044249,cellular biosynthetic process|GO:0034641,cellular nitrogen compound metabolic process|GO:0006807,nitrogen compound metabolic process|GO:0034645,cellular macromolecule biosynthetic process|GO:1901576,organic substance biosynthetic process|GO:0043241,protein complex disassembly|GO:1901566,organonitrogen compound biosynthetic process|GO:0071840,cellular component organization or biogenesis|GO:0044267,cellular protein metabolic process|GO:0044710,single-organism metabolic process|GO:0044711,single-organism biosynthetic process|GO:0071822,protein complex subunit organization|GO:0043043,peptide biosynthetic process|GO:0016043,cellular component organization|GO:0071704,organic substance metabolic process|GO:0010467,gene expression|GO:0007005,mitochondrion organization|GO:0032543,mitochondrial translation|GO:0070126,mitochondrial translational termination|GO:0070124,mitochondrial translational initiation|GO:0070125,mitochondrial translational elongation|GO:0044238,primary metabolic process|GO:0009987,cellular process|GO:0032984,macromolecular complex disassembly|GO:0009058,biosynthetic process|GO:0009059,macromolecule biosynthetic process|GO:0022411,cellular component disassembly|GO:0008152,metabolic process|GO:1901564,organonitrogen compound metabolic process|GO:0043624,cellular protein complex disassembly|GO:0006518,peptide metabolic process|GO:0006996,organelle organization|GO:0043604,amide biosynthetic process|GO:0044699,single-organism process|GO:0044271,cellular nitrogen compound biosynthetic process|GO:0044260,cellular macromolecule metabolic process|GO:0043603,cellular amide metabolic process|GO:0019538,protein metabolic process|GO:0044237,cellular metabolic process|GO:0043170,macromolecule metabolic process|GO:1902589,single-organism organelle organization|GO:0044763,single-organism cellular process|GO:0006415,translational termination|GO:0006414,translational elongation|GO:0006413,translational initiation|GO:0006412,translation</td><td>GO:0015935,small ribosomal subunit|GO:0044464,cell part|GO:0031974,membrane-enclosed lumen|GO:0031975,envelope|GO:0043229,intracellular organelle|GO:0043228,non-membrane-bounded organelle|GO:0043227,membrane-bounded organelle|GO:0043226,organelle|GO:0005737,cytoplasm|GO:0031090,organelle membrane|GO:0070013,intracellular organelle lumen|GO:0016020,membrane|GO:0044444,cytoplasmic part|GO:0005739,mitochondrion|GO:0030529,intracellular ribonucleoprotein complex|GO:0005759,mitochondrial matrix|GO:0044429,mitochondrial part|GO:0000314,organellar small ribosomal subunit|GO:0000313,organellar ribosome|GO:0031967,organelle envelope|GO:0044391,ribosomal subunit|GO:0031966,mitochondrial membrane|GO:0005840,ribosome|GO:0032991,macromolecular complex|GO:0043231,intracellular membrane-bounded organelle|GO:0043232,intracellular non-membrane-bounded organelle|GO:0043233,organelle lumen|GO:0019866,organelle inner membrane|GO:0005623,cell|GO:0005622,intracellular|GO:0044446,intracellular organelle part|GO:0005743,mitochondrial inner membrane|GO:0005740,mitochondrial envelope|GO:0005761,mitochondrial ribosome|GO:0005763,mitochondrial small ribosomal subunit|GO:0044424,intracellular part|GO:0044422,organelle part|GO:1990904,ribonucleoprotein complex|GO:1990204,oxidoreductase complex|GO:0009353,mitochondrial oxoglutarate dehydrogenase complex|GO:0045240,dihydrolipoyl dehydrogenase complex|GO:0005947,mitochondrial alpha-ketoglutarate dehydrogenase complex|GO:0045252,oxoglutarate dehydrogenase complex|GO:1902494,catalytic complex|GO:0030062,mitochondrial tricarboxylic acid cycle enzyme complex|GO:1990234,transferase complex|GO:0045239,tricarboxylic acid cycle enzyme complex|GO:0043234,protein complex|GO:0098798,mitochondrial protein complex</td><td>GO:0003735,structural constituent of ribosome|GO:0005198,structural molecule activity</td></tr>
<tr><td>CBWD7</td><td></td><td></td><td>GO:0043168,anion binding|GO:0005488,binding|GO:0030554,adenyl nucleotide binding|GO:0035639,purine ribonucleoside triphosphate binding|GO:0097367,carbohydrate derivative binding|GO:1901363,heterocyclic compound binding|GO:1901265,nucleoside phosphate binding|GO:0000166,nucleotide binding|GO:0036094,small molecule binding|GO:0032553,ribonucleotide binding|GO:0032559,adenyl ribonucleotide binding|GO:0005524,ATP binding|GO:0032549,ribonucleoside binding|GO:0032555,purine ribonucleotide binding|GO:0017076,purine nucleotide binding|GO:0043167,ion binding|GO:0097159,organic cyclic compound binding|GO:0032550,purine ribonucleoside binding|GO:0001883,purine nucleoside</td></tr>
<tr><td>EXOC5</td><td>GO:0044238,primary metabolic process|GO:0022406,membrane docking|GO:0061024,membrane organization|GO:0032940,secretion by cell|GO:0008104,protein localization|GO:0071840,cellular component organization or biogenesis|GO:0044267,cellular protein metabolic process|GO:0044260,cellular macromolecule metabolic process|GO:0006887,exocytosis|GO:0016043,cellular component organization|GO:0071704,organic substance metabolic process|GO:0044699,single-organism process|GO:0071702,organic substance transport|GO:0033036,macromolecule localization|GO:0006810,transport|GO:0048193,Golgi vesicle transport|GO:0009987,cellular process|GO:0045184,establishment of protein localization|GO:0048278,vesicle docking|GO:0044765,single-organism transport|GO:0044763,single-organism cellular process|GO:0008152,metabolic process|GO:0051649,establishment of localization in cell|GO:0051234,establishment of localization|GO:0051179,localization|GO:1902578,single-organism localization|GO:0051641,cellular localization|GO:0006996,organelle organization|GO:0006892,post-Golgi vesicle-mediated transport|GO:0046903,secretion|GO:0016192,vesicle-mediated transport|GO:0046907,intracellular transport|GO:0019538,protein metabolic process|GO:0044237,cellular metabolic process|GO:0043170,macromolecule metabolic process|GO:0015031,protein transport|GO:1902582,single-organism intracellular transport|GO:0015833,peptide transport|GO:0042886,amide transport|GO:0071705,nitrogen compound transport|GO:0006893,Golgi to plasma membrane transport|GO:0098876,vesicle-mediated transport to the plasma membrane|GO:0060249,anatomical structure homeostasis|GO:0042592,homeostatic process|GO:0007009,plasma membrane organization|GO:0032501,multicellular organismal process|GO:0007164,establishment of tissue polarity|GO:0008219,cell death|GO:0009653,anatomical structure morphogenesis|GO:0044707,single-multicellular organism process|GO:0002009,morphogenesis of an epithelium|GO:1904019,epithelial cell apoptotic process|GO:1990778,protein localization to cell periphery|GO:0070727,cellular macromolecule localization|GO:0010256,endomembrane system organization|GO:0065007,biological regulation|GO:0048729,tissue morphogenesis|GO:0065008,regulation of biological quality|GO:0034613,cellular protein localization|GO:0032502,developmental process|GO:0060429,epithelium development|GO:0006915,apoptotic process|GO:0009888,tissue development|GO:0044767,single-organism developmental process|GO:0012501,programmed cell death|GO:0001738,morphogenesis of a polarized epithelium|GO:0044802,single-organism membrane organization|GO:0072657,protein localization to membrane|GO:0001894,tissue homeostasis|GO:0072659,protein localization to plasma membrane|GO:0001736,establishment of planar polarity|GO:0048873,homeostasis of number of cells within a tissue|GO:0048872,homeostasis of number of cells|GO:0048871,multicellular organismal homeostasis|GO:0048856,anatomical structure development|GO:1902580,single-organism cellular localization</td><td>GO:0005737,cytoplasm|GO:0005829,cytosol|GO:0044464,cell part|GO:0005623,cell|GO:0005622,intracellular|GO:0044444,cytoplasmic part|GO:0044424,intracellular part|GO:0043234,protein complex|GO:0032991,macromolecular complex|GO:0000145,exocyst|GO:0099568,cytoplasmic region|GO:0071944,cell periphery|GO:0005938,cell cortex|GO:0044448,cell cortex part|GO:0030496,midbody</td><td>GO:0017160,Ral GTPase binding|GO:0047485,protein N-terminus binding|GO:0019899,enzyme binding|GO:0017016,Ras GTPase binding|GO:0031267,small GTPase binding|GO:0051020,GTPase binding|GO:0005488,binding|GO:0005515,protein binding</td></tr>
<tr><td>HELQ</td><td>GO:0090304,nucleic acid metabolic process|GO:0034641,cellular nitrogen compound metabolic process|GO:0006807,nitrogen compound metabolic process|GO:1901360,organic cyclic compound metabolic process|GO:0006139,nucleobase-containing compound metabolic process|GO:0044710,single-organism metabolic process|GO:0006302,double-strand break repair|GO:0000725,recombinational repair|GO:0000724,double-strand break repair via homologous recombination|GO:0071704,organic substance metabolic process|GO:0044699,single-organism process|GO:0006281,DNA repair|GO:0009987,cellular process|GO:0006725,cellular aromatic compound metabolic process|GO:0051716,cellular response to stimulus|GO:0006974,cellular response to DNA damage stimulus|GO:0006950,response to stress|GO:0044763,single-organism cellular process|GO:0008152,metabolic process|GO:0046483,heterocycle metabolic process|GO:0006310,DNA recombination|GO:0044238,primary metabolic process|GO:0044260,cellular macromolecule metabolic process|GO:0050896,response to stimulus|GO:0044237,cellular metabolic process|GO:0043170,macromolecule metabolic process|GO:0033554,cellular response to stress|GO:0006259,DNA metabolic process|GO:0010501,RNA secondary structure unwinding|GO:0016070,RNA metabolic process</td><td>GO:0044446,intracellular organelle part|GO:0043231,intracellular membrane-bounded organelle|GO:0031981,nuclear lumen|GO:0043233,organelle lumen|GO:0005634,nucleus|GO:0005730,nucleolus|GO:0044464,cell part|GO:0043232,intracellular non-membrane-bounded organelle|GO:0031974,membrane-enclosed lumen|GO:0005622,intracellular|GO:0005654,nucleoplasm|GO:0070013,intracellular organelle lumen|GO:0043229,intracellular organelle|GO:0043228,non-membrane-bounded organelle|GO:0044428,nuclear part|GO:0005623,cell|GO:0044424,intracellular part|GO:0043227,membrane-bounded organelle|GO:0043226,organelle|GO:0044422,organelle part|GO:0005737,cytoplasm</td><td>GO:0035639,purine ribonucleoside triphosphate binding|GO:0005488,binding|GO:0003676,nucleic acid binding|GO:0043167,ion binding|GO:1901363,heterocyclic compound binding|GO:1901265,nucleoside phosphate binding|GO:0001882,nucleoside binding|GO:0043168,anion binding|GO:0000166,nucleotide binding|GO:0001883,purine nucleoside binding|GO:0032549,ribonucleoside binding|GO:0017076,purine nucleotide binding|GO:0005524,ATP binding|GO:0016787,hydrolase activity|GO:0017111,nucleoside-triphosphatase activity|GO:0004386,helicase activity|GO:0036094,small molecule binding|GO:0003824,catalytic activity|GO:0016818,hydrolase activity, acting on acid anhydrides, in phosphorus-containing anhydrides|GO:0030554,adenyl nucleotide binding|GO:0097367,carbohydrate derivative binding|GO:0097159,organic cyclic compound binding|GO:0016817,hydrolase activity, acting on acid anhydrides|GO:0016462,pyrophosphatase activity|GO:0032559,adenyl ribonucleotide binding|GO:0032555,purine ribonucleotide binding|GO:0032550,purine ribonucleoside binding|GO:0032553,ribonucleotide binding|GO:0016887,ATPase activity|GO:0008026,ATP-dependent helicase activity|GO:0003677,DNA binding|GO:0008186,RNA-dependent ATPase activity|GO:0042623,ATPase activity, coupled|GO:0004004,ATP-dependent RNA helicase activity|GO:0070035,purine NTP-dependent helicase activity|GO:0003724,RNA helicase activity</td></tr>
<tr><td>BBX</td><td></td><td>GO:0005737,cytoplasm|GO:0044446,intracellular organelle part|GO:0043231,intracellular membrane-bounded organelle|GO:0031981,nuclear lumen|GO:0043233,organelle lumen|GO:0005634,nucleus|GO:0044464,cell part|GO:0031974,membrane-enclosed lumen|GO:0005622,intracellular|GO:0005654,nucleoplasm|GO:0070013,intracellular organelle lumen|GO:0043229,intracellular organelle|GO:0044428,nuclear part|GO:0005623,cell|GO:0044424,intracellular part|GO:0043227,membrane-bounded organelle|GO:0043226,organelle|GO:0044422,organelle part|GO:0005829,cytosol|GO:0044444,cytoplasmic part</td><td>GO:0097159,organic cyclic compound binding|GO:0005488,binding|GO:0003676,nucleic acid binding|GO:0003677,DNA binding|GO:1901363,heterocyclic compound binding</td></tr>
<tr><td>TCTE3</td><td>GO:0051234,establishment of localization|GO:0006810,transport|GO:0051179,localization|GO:0008152,metabolic process</td><td>GO:0043229,intracellular organelle|GO:0043228,non-membrane-bounded organelle|GO:0030286,dynein complex|GO:0005874,microtubule|GO:0043226,organelle|GO:0005856,cytoskeleton|GO:0016020,membrane|GO:0005875,microtubule associated complex|GO:0044430,cytoskeletal part|GO:0005737,cytoplasm|GO:0015630,microtubule cytoskeleton|GO:1902494,catalytic complex|GO:0043234,protein complex|GO:0032991,macromolecular complex|GO:0043232,intracellular non-membrane-bounded organelle|GO:0044464,cell part|GO:0005623,cell|GO:0005622,intracellular|GO:0044446,intracellular organelle part|GO:0044424,intracellular part|GO:0044422,organelle part|GO:0099512,supramolecular fiber|GO:0099513,polymeric cytoskeletal fiber</td><td>GO:0016787,hydrolase activity|GO:0016818,hydrolase activity, acting on acid anhydrides, in phosphorus-containing anhydrides|GO:0003824,catalytic activity|GO:0016817,hydrolase activity, acting on acid anhydrides|GO:0017111,nucleoside-triphosphatase activity|GO:0016462,pyrophosphatase activity|GO:0003774,motor activity</td></tr>
<tr><td>APPL1</td><td>GO:0008104,protein localization|GO:0051049,regulation of transport|GO:0061024,membrane organization|GO:0007009,plasma membrane organization|GO:0007165,signal transduction|GO:0007166,cell surface receptor signaling pathway|GO:0007167,enzyme linked receptor protein signaling pathway|GO:0007169,transmembrane receptor protein tyrosine kinase signaling pathway|GO:0090003,regulation of establishment of protein localization to plasma membrane|GO:0090002,establishment of protein localization to plasma membrane|GO:0071840,cellular component organization or biogenesis|GO:0051716,cellular response to stimulus|GO:0070727,cellular macromolecule localization|GO:0010256,endomembrane system organization|GO:0033036,macromolecule localization|GO:0045184,establishment of protein localization|GO:0072657,protein localization to membrane|GO:0043434,response to peptide hormone|GO:0072659,protein localization to plasma membrane|GO:0010033,response to organic substance|GO:0044700,single organism signaling|GO:0010243,response to organonitrogen compound|GO:0046323,glucose import|GO:0046324,regulation of glucose import|GO:0050789,regulation of biological process|GO:0008645,hexose transport|GO:0016043,cellular component organization|GO:0008643,carbohydrate transport|GO:0065007,biological regulation|GO:0044699,single-organism process|GO:0016265,obsolete death|GO:0070201,regulation of establishment of protein localization|GO:0006915,apoptotic process|GO:0006810,transport|GO:0050794,regulation of cellular process|GO:0012501,programmed cell death|GO:0044802,single-organism membrane organization|GO:1903729,regulation of plasma membrane organization|GO:0051234,establishment of localization|GO:1901698,response to nitrogen compound|GO:1901699,cellular response to nitrogen compound|GO:0051128,regulation of cellular component organization|GO:1903827,regulation of cellular protein localization|GO:0023052,signaling|GO:0070887,cellular response to chemical stimulus|GO:0042221,response to chemical|GO:1990778,protein localization to cell periphery|GO:0007049,cell cycle|GO:0032880,regulation of protein localization|GO:0009719,response to endogenous stimulus|GO:0071375,cellular response to peptide hormone stimulus|GO:0071495,cellular response to endogenous stimulus|GO:0008286,insulin receptor signaling pathway|GO:0038034,signal transduction in absence of ligand|GO:0008283,cell proliferation|GO:0009987,cellular process|GO:1904375,regulation of protein localization to cell periphery|GO:0032870,cellular response to hormone stimulus|GO:0032879,regulation of localization|GO:0009725,response to hormone|GO:0050896,response to stimulus|GO:0060341,regulation of cellular localization|GO:0097190,apoptotic signaling pathway|GO:0097191,extrinsic apoptotic signaling pathway|GO:0097192,extrinsic apoptotic signaling pathway in absence of ligand|GO:0010827,regulation of glucose transport|GO:0032869,cellular response to insulin stimulus|GO:0032868,response to insulin|GO:0008219,cell death|GO:0071417,cellular response to organonitrogen compound|GO:0015758,glucose transport|GO:0071310,cellular response to organic substance|GO:0071702,organic substance transport|GO:1903076,regulation of protein localization to plasma membrane|GO:0034613,cellular protein localization|GO:0044765,single-organism transport|GO:0044763,single-organism cellular process|GO:0007154,cell communication|GO:0051179,localization|GO:1902578,single-organism localization|GO:0051641,cellular localization|GO:1901700,response to oxygen-containing compound|GO:1901701,cellular response to oxygen-containing compound|GO:0090150,establishment of protein localization to membrane|GO:1901652,response to peptide|GO:1901653,cellular response to peptide|GO:0015749,monosaccharide transport|GO:1902580,single-organism cellular localization</td><td>GO:0044421,extracellular region part|GO:0031974,membrane-enclosed lumen|GO:0043229,intracellular organelle|GO:0005622,intracellular|GO:0043227,membrane-bounded organelle|GO:0043226,organelle|GO:0010008,endosome membrane|GO:0005737,cytoplasm|GO:1903561,extracellular vesicle|GO:0070603,SWI/SNF superfamily-type complex|GO:0031982,vesicle|GO:0031981,nuclear lumen|GO:0005634,nucleus|GO:0016020,membrane|GO:0031988,membrane-bounded vesicle|GO:0005654,nucleoplasm|GO:0044451,nucleoplasm part|GO:0032991,macromolecular complex|GO:0031901,early endosome membrane|GO:1902494,catalytic complex|GO:0017053,transcriptional repressor complex|GO:0098588,bounding membrane of organelle|GO:0000118,histone deacetylase complex|GO:0012505,endomembrane system|GO:0012506,vesicle membrane|GO:0090568,nuclear transcriptional repressor complex|GO:0098805,whole membrane|GO:0090545,CHD-type complex|GO:0070062,extracellular exosome|GO:0043234,protein complex|GO:0043230,extracellular organelle|GO:0016581,NuRD complex|GO:0043233,organelle lumen|GO:0005768,endosome|GO:0005829,cytosol|GO:0044464,cell part|GO:0005623,cell|GO:0043231,intracellular membrane-bounded organelle|GO:0044446,intracellular organelle part|GO:0070013,intracellular organelle lumen|GO:0044444,cytoplasmic part|GO:0005576,extracellular region|GO:0044428,nuclear part|GO:0031090,organelle membrane|GO:0044440,endosomal part|GO:0044424,intracellular part|GO:0065010,extracellular membrane-bounded organelle|GO:0005769,early endosome|GO:0044422,organelle part|GO:0044433,cytoplasmic vesicle part|GO:0031410,cytoplasmic</td><td>GO:0019899,enzyme binding|GO:0019901,protein kinase binding|GO:0019900,kinase binding|GO:0005488,binding|GO:0005515,protein binding|GO:0042802,identical protein binding|GO:0043422,protein kinase B binding</td></tr>
<tr><td>APOH</td><td>GO:0007599,hemostasis|GO:0019222,regulation of metabolic process|GO:0048585,negative regulation of response to stimulus|GO:0048584,positive regulation of response to stimulus|GO:0048583,regulation of response to stimulus|GO:0050680,negative regulation of epithelial cell proliferation|GO:0031638,zymogen activation|GO:0072359,circulatory system development|GO:0072358,cardiovascular system development|GO:0034197,triglyceride transport|GO:0034196,acylglycerol transport|GO:0044707,single-multicellular organism process|GO:0044710,single-organism metabolic process|GO:0006869,lipid transport|GO:0010467,gene expression|GO:0048513,animal organ development|GO:0048514,blood vessel morphogenesis|GO:0044093,positive regulation of molecular function|GO:0048518,positive regulation of biological process|GO:0048519,negative regulation of biological process|GO:0033036,macromolecule localization|GO:0007597,blood coagulation, intrinsic pathway|GO:0033033,negative regulation of myeloid cell apoptotic process|GO:0033032,regulation of myeloid cell apoptotic process|GO:0042127,regulation of cell proliferation|GO:0007596,blood coagulation|GO:0031100,animal organ regeneration|GO:0060548,negative regulation of cell death|GO:0046486,glycerolipid metabolic process|GO:0010876,lipid localization|GO:0065008,regulation of biological quality|GO:0010631,epithelial cell migration|GO:0010632,regulation of epithelial cell migration|GO:0010633,negative regulation of epithelial cell migration|GO:0019538,protein metabolic process|GO:0048870,cell motility|GO:0030193,regulation of blood coagulation|GO:0030195,negative regulation of blood coagulation|GO:0030194,positive regulation of blood coagulation|GO:0009893,positive regulation of metabolic process|GO:0022603,regulation of anatomical structure morphogenesis|GO:0006928,movement of cell or subcellular component|GO:0050820,positive regulation of coagulation|GO:1901342,regulation of vasculature development|GO:1901343,negative regulation of vasculature development|GO:0043170,macromolecule metabolic process|GO:0042981,regulation of apoptotic process|GO:0050789,regulation of biological process|GO:0043542,endothelial cell migration|GO:0051345,positive regulation of hydrolase activity|GO:0001568,blood vessel development|GO:1900046,regulation of hemostasis|GO:1900047,negative regulation of hemostasis|GO:0065007,biological regulation|GO:0044699,single-organism process|GO:0065009,regulation of molecular function|GO:0016477,cell migration|GO:1900048,positive regulation of hemostasis|GO:0048646,anatomical structure formation involved in morphogenesis|GO:0016265,obsolete death|GO:0006810,transport|GO:0006629,lipid metabolic process|GO:0050793,regulation of developmental process|GO:0050790,regulation of catalytic activity|GO:0042060,wound healing|GO:0050794,regulation of cellular process|GO:0012501,programmed cell death|GO:0006950,response to stress|GO:0050817,coagulation|GO:0051239,regulation of multicellular organismal process|GO:0010656,negative regulation of muscle cell apoptotic process|GO:0010657,muscle cell apoptotic process|GO:0051234,establishment of localization|GO:0050818,regulation of coagulation|GO:0050819,negative regulation of coagulation|GO:0051336,regulation of hydrolase activity|GO:0042730,fibrinolysis|GO:0051604,protein maturation|GO:0080134,regulation of response to stress|GO:0009605,response to external stimulus|GO:2000145,regulation of cell motility|GO:2000146,negative regulation of cell motility|GO:0061365,positive regulation of triglyceride lipase activity|GO:0060193,positive regulation of lipase activity|GO:0060191,regulation of lipase activity|GO:0032102,negative regulation of response to external stimulus|GO:0008152,metabolic process|GO:0032101,regulation of response to external stimulus|GO:0031099,regeneration|GO:0006639,acylglycerol metabolic process|GO:0006638,neutral lipid metabolic process|GO:0009611,response to wounding|GO:0051004,regulation of lipoprotein lipase activity|GO:0051006,positive regulation of lipoprotein lipase activity|GO:0001667,ameboidal-type cell migration|GO:0009653,anatomical structure morphogenesis|GO:0043085,positive regulation of catalytic activity|GO:0034391,regulation of smooth muscle cell apoptotic process|GO:0034390,smooth muscle cell apoptotic process|GO:0051240,positive regulation of multicellular organismal process|GO:0051241,negative regulation of multicellular organismal process|GO:0001944,vasculature development|GO:0051917,regulation of fibrinolysis|GO:0034392,negative regulation of smooth muscle cell apoptotic process|GO:1903034,regulation of response to wounding|GO:1903035,negative regulation of response to wounding|GO:0051918,negative regulation of fibrinolysis|GO:0032502,developmental process|GO:0008285,negative regulation of cell proliferation|GO:0032501,multicellular organismal process|GO:0050878,regulation of body fluid levels|GO:0006641,triglyceride metabolic process|GO:0009987,cellular process|GO:0051271,negative regulation of cellular component movement|GO:0040012,regulation of locomotion|GO:0006508,proteolysis|GO:0016485,protein processing|GO:0044255,cellular lipid metabolic process|GO:0032879,regulation of localization|GO:0090132,epithelium migration|GO:0090130,tissue migration|GO:0051093,negative regulation of developmental process|GO:0050678,regulation of epithelial cell proliferation|GO:0050673,epithelial cell proliferation|GO:0051674,localization of cell|GO:1903036,positive regulation of response to wounding|GO:0048731,system development|GO:0050896,response to stimulus|GO:0016525,negative regulation of angiogenesis|GO:2000181,negative regulation of blood vessel morphogenesis|GO:0010660,regulation of muscle cell apoptotic process|GO:0061041,regulation of wound healing|GO:0061045,negative regulation of wound healing|GO:0090303,positive regulation of wound healing|GO:0008219,cell death|GO:0010941,regulation of cell death|GO:0007275,multicellular organism development|GO:0001525,angiogenesis|GO:0072376,protein activation cascade|GO:0072378,blood coagulation, fibrin clot formation|GO:0008283,cell proliferation|GO:0031639,plasminogen activation|GO:0045765,regulation of angiogenesis|GO:0071704,organic substance metabolic process|GO:0043067,regulation of programmed cell death|GO:0043066,negative regulation of apoptotic process|GO:0071702,organic substance transport|GO:0043069,negative regulation of programmed cell death|GO:0097285,obsolete cell-type specific apoptotic process|GO:0030336,negative regulation of cell migration|GO:0030334,regulation of cell migration|GO:0006915,apoptotic process|GO:0044767,single-organism developmental process|GO:0044765,single-organism transport|GO:0044763,single-organism cellular process|GO:0051179,localization|GO:1902578,single-organism localization|GO:0040011,locomotion|GO:0044238,primary metabolic process|GO:0040013,negative regulation of locomotion|GO:0051270,regulation of cellular component movement|GO:0010594,regulation of endothelial cell migration|GO:0010596,negative regulation of endothelial cell migration|GO:0048856,anatomical structure development|GO:0044237,cellular metabolic process|GO:2000026,regulation of multicellular organismal development|GO:0001937,negative regulation of endothelial cell proliferation|GO:0033028,myeloid cell apoptotic process|GO:0001935,endothelial cell proliferation|GO:0048523,negative regulation of cellular process|GO:0001936,regulation of endothelial cell proliferation|GO:0016192,vesicle-mediated transport|GO:1901564,organonitrogen compound metabolic process|GO:0006887,exocytosis|GO:0045055,regulated exocytosis|GO:0032103,positive regulation of response to external stimulus|GO:0032940,secretion by cell|GO:0006807,nitrogen compound metabolic process|GO:0002576,platelet degranulation|GO:0046903,secretion</td><td>GO:0034358,plasma lipoprotein particle|GO:0031012,extracellular matrix|GO:1990777,lipoprotein particle|GO:0043227,membrane-bounded organelle|GO:0043226,organelle|GO:0042627,chylomicron|GO:0070062,extracellular exosome|GO:0031988,membrane-bounded vesicle|GO:0034361,very-low-density lipoprotein particle|GO:0034364,high-density lipoprotein particle|GO:0009986,cell surface|GO:0031982,vesicle|GO:0032991,macromolecular complex|GO:1903561,extracellular vesicle|GO:0005615,extracellular space|GO:0032994,protein-lipid complex|GO:0043230,extracellular organelle|GO:0044464,cell part|GO:0005623,cell|GO:0034385,triglyceride-rich plasma lipoprotein particle|GO:0005576,extracellular region|GO:0065010,extracellular membrane-bounded organelle|GO:0044421,extracellular region part|GO:0031974,membrane-enclosed lumen|GO:0043229,intracellular organelle|GO:0060205,cytoplasmic membrane-bounded vesicle lumen|GO:0005737,cytoplasm|GO:0031983,vesicle lumen|GO:0016023,cytoplasmic, membrane-bounded vesicle|GO:0031410,cytoplasmic vesicle|GO:0044433,cytoplasmic vesicle part|GO:0042827,platelet dense granule|GO:0005622,intracellular|GO:0099503,secretory vesicle|GO:0030141,secretory granule|GO:0097708,intracellular vesicle|GO:0034774,secretory granule lumen|GO:0043231,intracellular membrane-bounded organelle|GO:0043233,organelle lumen|GO:0031089,platelet dense granule lumen|GO:0044446,intracellular organelle part|GO:0044444,cytoplasmic part|GO:0044424,intracellular part|GO:0044422,organelle part|GO:0012505,endomembrane system</td><td>GO:1901681,sulfur compound binding|GO:0043168,anion binding|GO:0005543,phospholipid binding|GO:0097367,carbohydrate derivative binding|GO:0030234,enzyme regulator activity|GO:0060230,lipoprotein lipase activator activity|GO:0008289,lipid binding|GO:0008201,heparin binding|GO:0005539,glycosaminoglycan binding|GO:0043167,ion binding|GO:0008047,enzyme activator activity|GO:0005515,protein binding|GO:0005488,binding|GO:0098772,molecular function regulator|GO:0060229,lipase activator activity|GO:0042802,identical protein binding|GO:0001948,glycoprotein binding</td></tr>
<tr><td>TCEB1</td><td>GO:0080090,regulation of primary metabolic process|GO:0019222,regulation of metabolic process|GO:1901362,organic cyclic compound biosynthetic process|GO:1901360,organic cyclic compound metabolic process|GO:0051716,cellular response to stimulus|GO:0010604,positive regulation of macromolecule metabolic process|GO:0070647,protein modification by small protein conjugation or removal|GO:0044419,interspecies interaction between organisms|GO:0032446,protein modification by small protein conjugation|GO:0019058,viral life cycle|GO:0048518,positive regulation of biological process|GO:0051704,multi-organism process|GO:0060255,regulation of macromolecule metabolic process|GO:0071456,cellular response to hypoxia|GO:0032784,regulation of DNA-templated transcription, elongation|GO:0032786,positive regulation of DNA-templated transcription, elongation|GO:0006950,response to stress|GO:0030163,protein catabolic process|GO:2001141,regulation of RNA biosynthetic process|GO:0044403,symbiosis, encompassing mutualism through parasitism|GO:0046483,heterocycle metabolic process|GO:0019538,protein metabolic process|GO:0016567,protein ubiquitination|GO:0033554,cellular response to stress|GO:0019438,aromatic compound biosynthetic process|GO:0009893,positive regulation of metabolic process|GO:0009891,positive regulation of biosynthetic process|GO:0019080,viral gene expression|GO:0019083,viral transcription|GO:0010628,positive regulation of gene expression|GO:0044033,multi-organism metabolic process|GO:0061418,regulation of transcription from RNA polymerase II promoter in response to hypoxia|GO:0050789,regulation of biological process|GO:0097659,nucleic acid-templated transcription|GO:0044267,cellular protein metabolic process|GO:1901575,organic substance catabolic process|GO:0044265,cellular macromolecule catabolic process|GO:0044260,cellular macromolecule metabolic process|GO:0006357,regulation of transcription from RNA polymerase II promoter|GO:0071453,cellular response to oxygen levels|GO:0065007,biological regulation|GO:0006366,transcription from RNA polymerase II promoter|GO:0006368,transcription elongation from RNA polymerase II promoter|GO:0018130,heterocycle biosynthetic process|GO:0050792,regulation of viral process|GO:0009889,regulation of biosynthetic process|GO:0050794,regulation of cellular process|GO:0043902,positive regulation of multi-organism process|GO:0043903,regulation of symbiosis, encompassing mutualism through parasitism|GO:0043900,regulation of multi-organism process|GO:0036211,protein modification process|GO:0008152,metabolic process|GO:0034654,nucleobase-containing compound biosynthetic process|GO:0016070,RNA metabolic process|GO:0051603,proteolysis involved in cellular protein catabolic process|GO:0044271,cellular nitrogen compound biosynthetic process|GO:0036293,response to decreased oxygen levels|GO:0050896,response to stimulus|GO:0043412,macromolecule modification|GO:0036294,cellular response to decreased oxygen levels|GO:0006354,DNA-templated transcription, elongation|GO:0006355,regulation of transcription, DNA-templated|GO:0010557,positive regulation of macromolecule biosynthetic process|GO:0006511,ubiquitin-dependent protein catabolic process|GO:0006351,transcription, DNA-templated|GO:0032774,RNA biosynthetic process|GO:0044248,cellular catabolic process|GO:0044249,cellular biosynthetic process|GO:0034641,cellular nitrogen compound metabolic process|GO:0070887,cellular response to chemical stimulus|GO:0050434,positive regulation of viral transcription|GO:0001666,response to hypoxia|GO:0032968,positive regulation of transcription elongation from RNA polymerase II promoter|GO:0006139,nucleobase-containing compound metabolic process|GO:1903900,regulation of viral life cycle|GO:1903902,positive regulation of viral life cycle|GO:0043618,regulation of transcription from RNA polymerase II promoter in response to stress|GO:0046782,regulation of viral transcription|GO:0006508,proteolysis|GO:0070482,response to oxygen levels|GO:1903508,positive regulation of nucleic acid-templated transcription|GO:0034243,regulation of transcription elongation from RNA polymerase II promoter|GO:0009628,response to abiotic stimulus|GO:0009987,cellular process|GO:0019941,modification-dependent protein catabolic process|GO:1903506,regulation of nucleic acid-templated transcription|GO:0034645,cellular macromolecule biosynthetic process|GO:0045893,positive regulation of transcription, DNA-templated|GO:0044257,cellular protein catabolic process|GO:0051252,regulation of RNA metabolic process|GO:0051254,positive regulation of RNA metabolic process|GO:0009059,macromolecule biosynthetic process|GO:0043170,macromolecule metabolic process|GO:1902680,positive regulation of RNA biosynthetic process|GO:0006807,nitrogen compound metabolic process|GO:0045944,positive regulation of transcription from RNA polymerase II promoter|GO:0031328,positive regulation of cellular biosynthetic process|GO:0031326,regulation of cellular biosynthetic process|GO:0031325,positive regulation of cellular metabolic process|GO:0031323,regulation of cellular metabolic process|GO:0090304,nucleic acid metabolic process|GO:0043632,modification-dependent macromolecule catabolic process|GO:2000112,regulation of cellular macromolecule biosynthetic process|GO:0071704,organic substance metabolic process|GO:0010467,gene expression|GO:0010556,regulation of macromolecule biosynthetic process|GO:0042787,protein ubiquitination involved in ubiquitin-dependent protein catabolic process|GO:0010468,regulation of gene expression|GO:0045935,positive regulation of nucleobase-containing compound metabolic process|GO:1901576,organic substance biosynthetic process|GO:0019219,regulation of nucleobase-containing compound metabolic process|GO:0006725,cellular aromatic compound metabolic process|GO:0006464,cellular protein modification process|GO:0009058,biosynthetic process|GO:0044764,multi-organism cellular process|GO:0051171,regulation of nitrogen compound metabolic process|GO:0051173,positive regulation of nitrogen compound metabolic process|GO:0042221,response to chemical|GO:0009056,catabolic process|GO:0009057,macromolecule catabolic process|GO:0043620,regulation of DNA-templated transcription in response to stress|GO:0044238,primary metabolic process|GO:0044237,cellular metabolic process|GO:0016032,viral process|GO:0048524,positive regulation of viral process|GO:0048522,positive regulation of cellular process|GO:1901564,organonitrogen compound metabolic process|GO:1901565,organonitrogen compound catabolic process|GO:0043687,post-translational protein modification</td><td>GO:0008023,transcription elongation factor complex|GO:0031974,membrane-enclosed lumen|GO:0043229,intracellular organelle|GO:0043227,membrane-bounded organelle|GO:0043226,organelle|GO:0005737,cytoplasm|GO:0070449,elongin complex|GO:0031981,nuclear lumen|GO:0005634,nucleus|GO:0005654,nucleoplasm|GO:0044451,nucleoplasm part|GO:0043234,protein complex|GO:0032991,macromolecular complex|GO:0043231,intracellular membrane-bounded organelle|GO:0043233,organelle lumen|GO:0005829,cytosol|GO:0044464,cell part|GO:0005623,cell|GO:0005622,intracellular|GO:0044446,intracellular organelle part|GO:0070013,intracellular organelle lumen|GO:0044444,cytoplasmic part|GO:0044428,nuclear part|GO:0044424,intracellular part|GO:0044422,organelle part</td><td>GO:0019787,ubiquitin-like protein transferase activity|GO:0003824,catalytic activity|GO:0016740,transferase activity|GO:0005488,binding|GO:0005515,protein binding|GO:0004842,ubiquitin-protein transferase activity</td></tr>
<tr><td>PLEKHG7</td><td>GO:0009893,positive regulation of metabolic process|GO:0019222,regulation of metabolic process|GO:0035023,regulation of Rho protein signal transduction|GO:0048583,regulation of response to stimulus|GO:0023052,signaling|GO:0007165,signal transduction|GO:0023051,regulation of signaling|GO:0035556,intracellular signal transduction|GO:0010646,regulation of cell communication|GO:0043087,regulation of GTPase activity|GO:0050789,regulation of biological process|GO:0043085,positive regulation of catalytic activity|GO:0051716,cellular response to stimulus|GO:1902531,regulation of intracellular signal transduction|GO:0043547,positive regulation of GTPase activity|GO:0051345,positive regulation of hydrolase activity|GO:0009966,regulation of signal transduction|GO:0065007,biological regulation|GO:0044699,single-organism process|GO:0044093,positive regulation of molecular function|GO:0048518,positive regulation of biological process|GO:0065009,regulation of molecular function|GO:0051056,regulation of small GTPase mediated signal transduction|GO:0009987,cellular process|GO:0046578,regulation of Ras protein signal transduction|GO:0050790,regulation of catalytic activity|GO:0050794,regulation of cellular process|GO:0044763,single-organism cellular process|GO:0008152,metabolic process|GO:0007266,Rho protein signal transduction|GO:0007265,Ras protein signal transduction|GO:0007264,small GTPase mediated signal transduction|GO:0051336,regulation of hydrolase activity|GO:0044700,single organism signaling|GO:0050896,response to stimulus|GO:0007154,cell communication</td><td></td><td>GO:0005089,Rho guanyl-nucleotide exchange factor activity|GO:0005088,Ras guanyl-nucleotide exchange factor activity|GO:0098772,molecular function regulator|GO:0005085,guanyl-nucleotide exchange factor activity</td></tr>
</table>

LRRC4C	GO:0022604,regulation of cell morphogenesis|GO:0032502,developmental process|GO:0044707,single-multicellular organism process|GO:0051239,regulation of multicellular organismal process|GO:0009653,anatomical structure morphogenesis|GO:0022603,regulation of anatomical structure morphogenesis|GO:0030030,cell projection organization|GO:0030154,cell differentiation|GO:0010975,regulation of neuron projection development|GO:0051128,regulation of cellular component organization|GO:0016043,cellular component organization|GO:0031175,neuron projection development|GO:0032989,cellular component morphogenesis|GO:0050789,regulation of biological process|GO:0044699,single-organism process|GO:0050767,regulation of neurogenesis|GO:0000904,cell morphogenesis involved in differentiation|GO:0000902,cell morphogenesis|GO:0051960,regulation of nervous system development|GO:0048869,cellular developmental process|GO:0060284,regulation of cell development|GO:0031344,regulation of cell projection organization|GO:0045664,regulation of neuron differentiation|GO:0010769,regulation of cell morphogenesis involved in differentiation|GO:0065007,biological regulation|GO:0071840,cellular component organization or biogenesis|GO:0048666,neuron development|GO:0048667,cell morphogenesis involved in neuron differentiation|GO:0032501,multicellular organismal process|GO:0061564,axon development|GO:0030182,neuron differentiation|GO:0050793,regulation of developmental process|GO:0048468,cell development|GO:0009987,cellular process|GO:0050794,regulation of cellular process|GO:0044767,single-organism developmental process|GO:0045595,regulation of cell differentiation|GO:0044763,single-organism cellular process|GO:0007409,axonogenesis|GO:0022008,neurogenesis|GO:0032990,cell part morphogenesis|GO:0048858,cell projection morphogenesis|GO:0048699,generation of neurons|GO:0050770,regulation of axonogenesis|GO:0007399,nervous system development|GO:0048856,anatomical structure development|GO:0048812,neuron projection morphogenesis|GO:2000026,regulation of multicellular organismal development|GO:0007275,multicellular organism development|GO:0048731,system development|GO:0051348,negative regulation of transferase activity|GO:0019220,regulation of phosphate metabolic process|GO:0019221,cytokine-mediated signaling pathway|GO:0019222,regulation of metabolic process|GO:0048585,negative regulation of response to stimulus|GO:0048583,regulation of response to stimulus|GO:0007165,signal transduction|GO:0007166,cell surface receptor signaling pathway|GO:0080090,regulation of primary metabolic process|GO:0051716,cellular response to stimulus|GO:0010605,negative regulation of macromolecule metabolic process|GO:0009968,negative regulation of signal transduction|GO:0009966,regulation of signal transduction|GO:0044092,negative regulation of molecular function|GO:0048519,negative regulation of biological process|GO:0060255,regulation of macromolecule metabolic process|GO:0045859,regulation of protein kinase activity|GO:0042221,response to chemical|GO:0010033,response to organic substance|GO:0042325,regulation of phosphorylation|GO:0044700,single organism signaling|GO:0042326,negative regulation of phosphorylation|GO:1901564,organonitrogen compound metabolic process|GO:0019538,protein metabolic process|GO:0033673,negative regulation of kinase activity|GO:0009892,negative regulation of metabolic process|GO:0006807,nitrogen compound metabolic process|GO:0035556,intracellular signal transduction|GO:0044267,cellular protein metabolic process|GO:0044260,cellular macromolecule metabolic process|GO:0046425,regulation of JAK-STAT cascade|GO:0065009,regulation of molecular function|GO:0034097,response to cytokine|GO:0050790,regulation of catalytic activity|GO:0043412,macromolecule modification|GO:0036211,protein modification process|GO:1902532,negative regulation of intracellular signal transduction|GO:1902531,regulation of intracellular signal transduction|GO:0071345,cellular response to cytokine stimulus|GO:0031400,negative regulation of protein modification process|GO:0051338,regulation of transferase activity|GO:0008152,metabolic process|GO:0016310,phosphorylation|GO:0023057,negative regulation of signaling|GO:0023052,signaling|GO:0010648,negative regulation of cell communication|GO:0070887,cellular response to chemical stimulus|GO:0023051,regulation of signaling|GO:0010646,regulation of cell communication|GO:0043086,negative regulation of catalytic activity|GO:0051248,negative regulation of protein metabolic process|GO:0010563,negative regulation of phosphorus metabolic process|GO:0051246,regulation of protein metabolic process|GO:0007259,JAK-STAT cascade|GO:0043549,regulation of kinase activity|GO:0031399,regulation of protein modification process|GO:0032269,negative regulation of cellular protein metabolic process|GO:0032268,regulation of cellular protein metabolic process|GO:0043170,macromolecule metabolic process|GO:1904893,negative regulation of STAT cascade|GO:1904892,regulation of STAT cascade|GO:0050896,response to stimulus|GO:0031324,negative regulation of cellular metabolic process|GO:0031323,regulation of cellular metabolic process|GO:0071704,organic substance metabolic process|GO:0071310,cellular response to organic substance|GO:0097696,STAT cascade|GO:0006468,protein phosphorylation|GO:0006469,negative regulation of protein kinase activity|GO:0046426,negative regulation of JAK-STAT cascade|GO:0045936,negative regulation of phosphate metabolic process|GO:0006464,cellular protein modification process|GO:0051174,regulation of phosphorus metabolic process|GO:0007154,cell communication|GO:0044238,primary metabolic process|GO:0044237,cellular metabolic process|GO:0006796,phosphate-containing compound metabolic process|GO:0006793,phosphorus metabolic process|GO:0001933,negative regulation of protein phosphorylation|GO:0001932,regulation of protein phosphorylation|GO:0048523,negative regulation of cellular process|GO:0050808,synapse organization|GO:0098609,cell-cell adhesion|GO:0099560,synaptic membrane adhesion|GO:0007155,cell adhesion|GO:0022610,biological adhesion|GO:0098742,cell-cell adhesion via plasma-membrane adhesion molecules	GO:0098590,plasma membrane region|GO:0071944,cell periphery|GO:0045202,synapse|GO:0031224,intrinsic component of membrane|GO:0097060,synaptic membrane|GO:0005615,extracellular space|GO:0016021,integral component of membrane|GO:0016020,membrane|GO:0098805,whole membrane|GO:0044456,synapse part|GO:0030054,cell junction|GO:0044459,plasma membrane part|GO:0005576,extracellular region|GO:0098589,membrane region|GO:0045211,postsynaptic membrane|GO:0005886,plasma membrane|GO:0044464,cell part|GO:0005623,cell|GO:0098794,postsynapse|GO:0044425,membrane part|GO:0044421,extracellular region part|GO:0005737,cytoplasm|GO:0005622,intracellular|GO:0044424,intracellular part	GO:0005488,binding|GO:0005515,protein binding|GO:0019210,kinase inhibitor activity|GO:0019207,kinase regulator activity|GO:0019887,protein kinase regulator activity|GO:0030234,enzyme regulator activity|GO:0004860,protein kinase inhibitor activity|GO:0004857,enzyme inhibitor activity|GO:0098772,molecular function regulator|GO:0050839,cell adhesion molecule binding|GO:0098631,protein binding involved in cell adhesion|GO:0098632,protein binding involved in cell-cell adhesion
LRRC3B		GO:0044425,membrane part|GO:0016021,integral component of membrane|GO:0016020,membrane|GO:0031224,intrinsic component of membrane	
MGST1	GO:0033327,Leydig cell differentiation|GO:0008584,male gonad development|GO:0044281,small molecule metabolic process|GO:0044707,single-multicellular organism process|GO:0071840,cellular component organization or biogenesis|GO:0044710,single-organism metabolic process|GO:0044711,single-organism biosynthetic process|GO:0043207,response to external biotic stimulus|GO:0006982,response to lipid hydroperoxide|GO:0048869,cellular developmental process|GO:0009617,response to bacterium|GO:0045137,development of primary sexual characteristics|GO:0048513,animal organ development|GO:0003006,developmental process involved in reproduction|GO:1901564,organonitrogen compound metabolic process|GO:0051707,response to other organism|GO:0010033,response to organic substance|GO:0055114,oxidation-reduction process|GO:0051704,multi-organism process|GO:0044702,single organism reproductive process|GO:0009607,response to biotic stimulus|GO:0009605,response to external stimulus|GO:1901566,organonitrogen compound biosynthetic process|GO:0010243,response to organonitrogen compound|GO:0009410,response to xenobiotic stimulus|GO:0071447,cellular response to hydroperoxide|GO:0033554,cellular response to stress|GO:0071449,cellular response to lipid hydroperoxide|GO:0022607,cellular component assembly|GO:0006805,xenobiotic metabolic process|GO:0006807,nitrogen compound metabolic process|GO:0032496,response to lipopolysaccharide|GO:1901576,organic substance biosynthetic process|GO:0008406,gonad development|GO:0016043,cellular component organization|GO:0065003,macromolecular complex assembly|GO:0070207,protein homotrimerization|GO:0070206,protein trimerization|GO:0046546,development of primary male sexual characteristics|GO:0051716,cellular response to stimulus|GO:0006950,response to stress|GO:0008152,metabolic process|GO:0046661,male sex differentiation|GO:0006575,cellular modified amino acid metabolic process|GO:0007548,sex differentiation|GO:0044272,sulfur compound biosynthetic process|GO:1901698,response to nitrogen compound|GO:0071466,cellular response to xenobiotic stimulus|GO:0006518,peptide metabolic process|GO:0070271,protein complex biogenesis|GO:0030154,cell differentiation|GO:0044249,cellular biosynthetic process|GO:0034641,cellular nitrogen compound metabolic process|GO:0070887,cellular response to chemical stimulus|GO:0044699,single-organism process|GO:0009719,response to endogenous stimulus|GO:1901685,glutathione derivative metabolic process|GO:1901687,glutathione derivative biosynthetic process|GO:0043933,macromolecular complex subunit organization|GO:0006749,glutathione metabolic process|GO:0032502,developmental process|GO:0032501,multicellular organismal process|GO:0048608,reproductive structure development|GO:0009987,cellular process|GO:0006979,response to oxidative stress|GO:0051259,protein oligomerization|GO:0043603,cellular amide metabolic process|GO:0042493,response to drug|GO:0002237,response to molecule of bacterial origin|GO:0033194,response to hydroperoxide|GO:0048731,system development|GO:0050896,response to stimulus|GO:0061458,reproductive system development|GO:0007275,multicellular organism development|GO:0071822,protein complex subunit organization|GO:0033993,response to lipid|GO:0051260,protein homooligomerization|GO:0071704,organic substance metabolic process|GO:0006461,protein complex assembly|GO:0044767,single-organism developmental process|GO:0022414,reproductive process|GO:0009058,biosynthetic process|GO:0044763,single-organism cellular process|GO:0042221,response to chemical|GO:0034599,cellular response to oxidative stress|GO:1901700,response to oxygen-containing compound|GO:1901701,cellular response to oxygen-containing compound|GO:0048856,anatomical structure development|GO:0044237,cellular metabolic process|GO:0044085,cellular component biogenesis|GO:0006790,sulfur compound metabolic process|GO:0009636,response to toxic substance|GO:0000003,reproduction|GO:0002263,cell activation involved in immune response|GO:0098869,cellular oxidant detoxification|GO:1990748,cellular detoxification|GO:0016192,vesicle-mediated transport|GO:0002275,myeloid cell activation involved in immune response|GO:0002274,myeloid leukocyte activation|GO:0002376,immune system process|GO:0045321,leukocyte activation|GO:0032940,secretion by cell|GO:0098754,detoxification|GO:0002283,neutrophil activation involved in immune response|GO:0006887,exocytosis|GO:0045055,regulated exocytosis|GO:0002366,leukocyte activation involved in immune response|GO:0006810,transport|GO:0001775,cell activation|GO:0006955,immune response|GO:0051234,establishment of localization|GO:0046903,secretion|GO:0002446,neutrophil mediated immunity|GO:0036230,granulocyte activation|GO:0042119,neutrophil activation|GO:0043312,neutrophil degranulation|GO:0044765,single-organism transport|GO:0002443,leukocyte mediated immunity|GO:0002444,myeloid leukocyte mediated immunity|GO:0051179,localization|GO:1902578,single-organism localization|GO:0043299,leukocyte degranulation|GO:0002252,immune effector process	GO:0005783,endoplasmic reticulum|GO:0044464,cell part|GO:0005789,endoplasmic reticulum membrane|GO:0042579,microbody|GO:0043229,intracellular organelle|GO:0043227,membrane-bounded organelle|GO:0043226,organelle|GO:0031224,intrinsic component of membrane|GO:0005737,cytoplasm|GO:0045177,apical part of cell|GO:0005778,peroxisomal membrane|GO:0031090,organelle membrane|GO:0005634,nucleus|GO:0016020,membrane|GO:0005777,peroxisome|GO:0044444,cytoplasmic part|GO:0005739,mitochondrion|GO:0098805,whole membrane|GO:0044432,endoplasmic reticulum part|GO:0044439,peroxisomal part|GO:0044438,microbody part|GO:0031903,microbody membrane|GO:0031968,organelle outer membrane|GO:0098588,bounding membrane of organelle|GO:0031967,organelle envelope|GO:0031966,mitochondrial membrane|GO:0012505,endomembrane system|GO:0031975,envelope|GO:0043231,intracellular membrane-bounded organelle|GO:0042175,nuclear outer membrane-endoplasmic reticulum membrane network|GO:0019866,organelle inner membrane|GO:0019867,outer membrane|GO:0005623,cell|GO:0005622,intracellular|GO:0044446,intracellular organelle part|GO:0005743,mitochondrial inner membrane|GO:0005740,mitochondrial envelope|GO:0005741,mitochondrial outer membrane|GO:0044429,mitochondrial part|GO:0044424,intracellular part|GO:0044425,membrane part|GO:0016021,integral component of membrane|GO:0044422,organelle part|GO:0000323,lytic vacuole|GO:0071944,cell periphery|GO:0031988,membrane-bounded vesicle|GO:0035577,azurophil granule membrane|GO:0042582,azurophil granule|GO:0005764,lysosome|GO:0030667,secretory granule membrane|GO:0031982,vesicle|GO:0005773,vacuole|GO:0031410,cytoplasmic vesicle|GO:0005766,primary lysosome|GO:0005774,vacuolar membrane|GO:0044433,cytoplasmic vesicle part|GO:0098827,endoplasmic reticulum subcompartment|GO:0044437,vacuolar part|GO:0099503,secretory vesicle|GO:0016023,cytoplasmic, membrane-bounded vesicle|GO:0030141,secretory granule|GO:0030659,cytoplasmic vesicle membrane|GO:0031984,organelle subcompartment|GO:0097708,intracellular vesicle|GO:0012506,vesicle membrane|GO:0005886,plasma membrane|GO:0005765,lysosomal membrane|GO:0098852,lytic vacuole membrane	GO:0005488,binding|GO:0016491,oxidoreductase activity|GO:1901681,sulfur compound binding|GO:0004364,glutathione transferase activity|GO:0042277,peptide binding|GO:0043167,ion binding|GO:0016765,transferase activity, transferring alkyl or aryl (other than methyl) groups|GO:0016740,transferase activity|GO:0016684,oxidoreductase activity, acting on peroxide as acceptor|GO:1900750,oligopeptide binding|GO:0042802,identical protein binding|GO:0042803,protein homodimerization activity|GO:0043168,anion binding|GO:0033218,amide binding|GO:0016209,antioxidant activity|GO:0003824,catalytic activity|GO:0072341,modified amino acid binding|GO:0005515,protein binding|GO:0046983,protein dimerization activity|GO:0004601,peroxidase activity|GO:0004602,glutathione peroxidase activity|GO:0043295,glutathione binding
NDUFB8	GO:0009141,nucleoside triphosphate metabolic process|GO:0009144,purine nucleoside triphosphate metabolic process|GO:0009167,purine ribonucleoside monophosphate metabolic process|GO:0034641,cellular nitrogen compound metabolic process|GO:0006807,nitrogen compound metabolic process|GO:0044281,small molecule metabolic process|GO:0009161,ribonucleoside monophosphate metabolic process|GO:0009123,nucleoside monophosphate metabolic process|GO:0006091,generation of precursor metabolites and energy|GO:0009126,purine nucleoside monophosphate metabolic process|GO:0009259,ribonucleotide metabolic process|GO:0044699,single-organism process|GO:0006139,nucleobase-containing compound metabolic process|GO:0044710,single-organism metabolic process|GO:0042775,mitochondrial ATP synthesis coupled electron transport|GO:1901360,organic cyclic compound metabolic process|GO:0042278,purine nucleoside metabolic process|GO:0071704,organic substance metabolic process|GO:0009199,ribonucleoside triphosphate metabolic process|GO:0022900,electron transport chain|GO:0055086,nucleobase-containing small molecule metabolic process|GO:0022904,respiratory electron transport chain|GO:0006119,oxidative phosphorylation|GO:0009205,purine ribonucleoside triphosphate metabolic process|GO:0046034,ATP metabolic process|GO:0009987,cellular process|GO:0006725,cellular aromatic compound metabolic process|GO:0009150,purine ribonucleotide metabolic process|GO:0072521,purine-containing compound metabolic process|GO:0045333,cellular respiration|GO:0009116,nucleoside metabolic process|GO:0008152,metabolic process|GO:0009119,ribonucleoside metabolic process|GO:0046128,purine ribonucleoside metabolic process|GO:0042773,ATP synthesis coupled electron transport|GO:0055114,oxidation-reduction process|GO:0046483,heterocycle metabolic process|GO:0044238,primary metabolic process|GO:1901564,organonitrogen compound metabolic process|GO:0015980,energy derivation by oxidation of organic compounds|GO:0016310,phosphorylation|GO:0044763,single-organism cellular process|GO:0019693,ribose phosphate metabolic process|GO:0006120,mitochondrial electron transport, NADH to ubiquinone|GO:0006163,purine nucleotide metabolic process|GO:1901657,glycosyl compound metabolic process|GO:0006796,phosphate-containing compound metabolic process|GO:0006793,phosphorus metabolic process|GO:0019637,organophosphate metabolic process|GO:0009117,nucleotide metabolic process|GO:0044237,cellular metabolic process|GO:0006753,nucleoside phosphate metabolic process|GO:1901135,carbohydrate derivative metabolic process|GO:0022607,cellular component assembly|GO:0070271,protein complex biogenesis|GO:0043933,macromolecular complex subunit organization|GO:0034622,cellular macromolecular complex assembly|GO:0071840,cellular component organization or biogenesis|GO:0071822,protein complex subunit organization|GO:0032981,mitochondrial respiratory chain complex I assembly|GO:0010257,NADH dehydrogenase complex assembly|GO:0016043,cellular component organization|GO:0065003,macromolecular complex assembly|GO:0007005,mitochondrion organization|GO:0033108,mitochondrial respiratory chain complex assembly|GO:0006461,protein complex assembly|GO:0043623,cellular protein complex assembly|GO:0006996,organelle organization|GO:0097031,mitochondrial respiratory chain complex I biogenesis|GO:0044085,cellular component biogenesis	GO:0005783,endoplasmic reticulum|GO:0044464,cell part|GO:1990204,oxidoreductase complex|GO:0043229,intracellular organelle|GO:0043227,membrane-bounded organelle|GO:0043226,organelle|GO:0031224,intrinsic component of membrane|GO:0005737,cytoplasm|GO:0031090,organelle membrane|GO:0098796,membrane protein complex|GO:0016021,integral component of membrane|GO:0016020,membrane|GO:0098803,respiratory chain complex|GO:0044444,cytoplasmic part|GO:0005739,mitochondrion|GO:0098800,inner mitochondrial membrane protein complex|GO:0044455,mitochondrial membrane part|GO:0044429,mitochondrial part|GO:1902494,catalytic complex|GO:0031967,organelle envelope|GO:0031966,mitochondrial membrane|GO:0012505,endomembrane system|GO:0031975,envelope|GO:0043234,protein complex|GO:0045271,respiratory chain complex I|GO:0032991,macromolecular complex|GO:0043231,intracellular membrane-bounded organelle|GO:0030964,NADH dehydrogenase complex|GO:0019866,organelle inner membrane|GO:0005623,cell|GO:0005622,intracellular|GO:0044446,intracellular organelle part|GO:0005743,mitochondrial inner membrane|GO:0005740,mitochondrial envelope|GO:0005746,mitochondrial respiratory chain|GO:0005747,mitochondrial respiratory chain complex I|GO:0044424,intracellular part|GO:0044425,membrane part|GO:0070469,respiratory chain|GO:0044422,organelle part|GO:0098798,mitochondrial protein complex|GO:0005759,mitochondrial matrix|GO:0070013,intracellular organelle lumen|GO:0031974,membrane-	GO:0003824,catalytic activity|GO:0008137,NADH dehydrogenase (ubiquinone) activity|GO:0016655,oxidoreductase activity, acting on NAD(P)H, quinone or similar compound as acceptor|GO:0050136,NADH dehydrogenase (quinone) activity|GO:0016651,oxidoreductase activity, acting on NAD(P)H|GO:0003954,NADH dehydrogenase activity|GO:0016491,oxidoreductase activity
NCKAP1	GO:0008105,asymmetric protein localization|GO:0008104,protein localization|GO:0006909,phagocytosis|GO:0051234,establishment of localization|GO:0048584,positive regulation of response to stimulus|GO:0048583,regulation of response to stimulus|GO:0007492,endoderm development|GO:0008064,regulation of actin polymerization or depolymerization|GO:0060491,regulation of cell projection assembly|GO:0072358,cardiovascular system development|GO:0007165,signal transduction|GO:0007166,cell surface receptor signaling pathway|GO:0007167,enzyme linked receptor protein signaling pathway|GO:0007169,transmembrane receptor protein tyrosine kinase signaling pathway|GO:0031344,regulation of cell projection organization|GO:0071840,cellular component organization or biogenesis|GO:0031346,positive regulation of cell projection organization|GO:0051716,cellular response to stimulus|GO:0001756,somitogenesis|GO:0051495,positive regulation of cytoskeleton organization|GO:0007369,gastrulation|GO:0051493,regulation of cytoskeleton organization|GO:0044419,interspecies interaction between organisms|GO:0048513,animal organ development|GO:0048518,positive regulation of biological process|GO:0065007,biological regulation|GO:0033036,macromolecule localization|GO:0048570,notochord morphogenesis|GO:0008078,mesodermal cell migration|GO:0022607,cellular component assembly|GO:0044700,single organism signaling|GO:0006952,defense response|GO:0002429,immune response-activating cell surface receptor signaling pathway|GO:0003002,regionalization|GO:0030041,actin filament polymerization|GO:0035148,tube formation|GO:0051704,multi-organism process|GO:0014020,primary neural tube formation|GO:0007440,foregut morphogenesis|GO:0016192,vesicle-mediated transport|GO:0044707,single-multicellular organism process|GO:0048870,cell motility|GO:0010638,positive regulation of organelle organization|GO:0072359,circulatory system development|GO:0002376,immune system process|GO:0009952,anterior/posterior pattern specification|GO:0034315,regulation of Arp2/3 complex-mediated actin nucleation|GO:0048340,paraxial mesoderm morphogenesis|GO:0032535,regulation of cellular component size|GO:0051130,positive regulation of cellular component organization|GO:0048562,embryonic organ morphogenesis|GO:0048565,digestive tract development|GO:0048568,embryonic organ development|GO:0006928,movement of cell or subcellular component|GO:0050821,protein stabilization|GO:0051674,localization of cell|GO:0000578,embryonic axis specification|GO:0072132,mesenchyme morphogenesis|GO:0035556,intracellular signal transduction|GO:0050789,regulation of biological process|GO:0002764,immune response-regulating signaling pathway|GO:0016601,Rac protein signal transduction|GO:0002431,Fc receptor mediated stimulatory signaling pathway|GO:0002768,immune response-regulating cell surface receptor signaling pathway|GO:0002433,immune response-regulating cell surface receptor signaling pathway involved in phagocytosis|GO:0016043,cellular component organization|GO:0090066,regulation of anatomical structure size|GO:0065003,macromolecular complex assembly|GO:0002684,positive regulation of immune system process|GO:0002682,regulation of immune system process|GO:0002252,immune effector process|GO:0001841,neural tube formation|GO:0001843,neural tube closure|GO:0007417,central nervous system development|GO:0048646,anatomical structure formation involved in morphogenesis|GO:0016265,obsolete death|GO:0009887,animal organ morphogenesis|GO:0006461,protein complex assembly|GO:0009880,embryonic pattern specification|GO:0006810,transport|GO:0009888,tissue development|GO:0050794,regulation of cellular process|GO:0060606,tube closure|GO:0012501,programmed cell death|GO:0006950,response to stress|GO:0045176,apical protein localization|GO:0032956,regulation of actin cytoskeleton organization|GO:0048010,vascular endothelial growth factor receptor signaling pathway|GO:0043254,regulation of protein complex assembly|GO:0007350,blastoderm segmentation|GO:0007354,zygotic determination of anterior/posterior axis, embryo|GO:0038096,Fc-gamma receptor signaling pathway involved in phagocytosis|GO:0008154,actin polymerization or depolymerization|GO:0038094,Fc-gamma receptor signaling pathway|GO:0002757,immune response-activating signal transduction|GO:0006897,endocytosis|GO:0038093,Fc receptor signaling pathway|GO:0050896,response to stimulus|GO:0031647,regulation of protein stability|GO:0051127,positive regulation of actin nucleation|GO:0051125,regulation of actin nucleation|GO:0033043,regulation of organelle organization|GO:0007498,mesoderm development|GO:0042074,cell migration involved in gastrulation|GO:0006955,immune response|GO:0051128,regulation of cellular component organization|GO:0055123,digestive system development|GO:0009790,embryo development|GO:0009792,embryo development ending in birth or egg hatching|GO:0023052,signaling|GO:0034314,Arp2/3 complex-mediated actin nucleation|GO:0007154,cell communication|GO:0009798,axis specification|GO:0001667,ameboidal-type cell migration|GO:0009653,anatomical structure morphogenesis|GO:0007351,tripartite regional subdivision|GO:0044699,single-organism process|GO:0032880,regulation of protein localization|GO:0045175,basal protein localization|GO:0001701,in utero embryonic development|GO:0030952,establishment or maintenance of cytoskeleton polarity|GO:0030950,establishment or maintenance of actin cytoskeleton polarity|GO:0043933,macromolecular complex subunit organization|GO:0032271,regulation of protein polymerization|GO:0032273,positive regulation of protein polymerization|GO:0060562,epithelial tube morphogenesis|GO:0043009,chordate embryonic development|GO:0009948,anterior/posterior axis specification|GO:0032502,developmental process|GO:0001838,embryonic epithelial tube formation|GO:0006996,organelle organization|GO:2000601,positive regulation of Arp2/3 complex-mediated actin nucleation|GO:0035239,tube morphogenesis|GO:0048332,mesoderm morphogenesis|GO:0007015,actin filament organization|GO:0032970,regulation of actin filament-based process|GO:0031334,positive regulation of protein complex assembly|GO:0048339,paraxial mesoderm development|GO:0007163,establishment or maintenance of cell polarity|GO:0040011,locomotion|GO:0010172,embryonic body morphogenesis|GO:0010171,body morphogenesis|GO:0032879,regulation of localization|GO:0061053,somite development|GO:0051258,protein polymerization|GO:0050776,regulation of immune response|GO:0065008,regulation of biological quality|GO:0050778,positive regulation of immune response|GO:0048731,system development|GO:0009987,cellular process|GO:0048546,digestive tract morphogenesis|GO:0016331,morphogenesis of embryonic epithelium|GO:0048617,embryonic foregut morphogenesis|GO:0030030,cell projection organization|GO:0030031,cell projection assembly|GO:0030032,lamellipodium assembly|GO:0030036,actin cytoskeleton organization|GO:0072175,epithelial tube formation|GO:0032501,multicellular organismal process|GO:0016477,cell migration|GO:0034622,cellular macromolecular complex assembly|GO:0008219,cell death|GO:0035282,segmentation|GO:0007389,pattern specification process|GO:0030832,regulation of actin filament length|GO:0030833,regulation of actin filament polymerization|GO:0045010,actin nucleation|GO:0030838,positive regulation of actin filament polymerization|GO:0007275,multicellular organism development|GO:0070271,protein complex biogenesis|GO:0048729,tissue morphogenesis|GO:0048598,embryonic morphogenesis|GO:0060429,epithelium development|GO:0021915,neural tube development|GO:0016032,viral process|GO:0030029,actin filament-based process|GO:0002009,morphogenesis of an epithelium|GO:0045087,innate immune response|GO:0006915,apoptotic process|GO:0035050,embryonic heart tube development|GO:0044767,single-organism developmental process|GO:0008595,anterior/posterior axis specification, embryo|GO:0044765,single-organism transport|GO:0044764,multi-organism cellular process|GO:0044763,single-organism cellular process|GO:0097581,lamellipodium organization|GO:0060485,mesenchyme development|GO:0007265,Ras protein signal transduction|GO:0007264,small GTPase mediated signal transduction|GO:0043623,cellular protein complex assembly|GO:0051179,localization|GO:1902578,single-organism localization|GO:0010591,regulation of lamellipodium assembly|GO:0007507,heart development|GO:0010592,positive regulation of lamellipodium assembly|GO:0007010,cytoskeleton organization|GO:0071822,protein complex subunit	GO:0098590,plasma membrane region|GO:0044421,extracellular region part|GO:0071944,cell periphery|GO:0031209,SCAR complex|GO:0005924,cell-substrate adherens junction|GO:0005925,focal adhesion|GO:0043227,membrane-bounded organelle|GO:0043226,organelle|GO:0030054,cell junction|GO:0030055,cell-substrate junction|GO:0005737,cytoplasm|GO:0042995,cell projection|GO:0031982,vesicle|GO:0016021,integral component of membrane|GO:0016020,membrane|GO:0031988,membrane-bounded vesicle|GO:0031224,intrinsic component of membrane|GO:0044459,plasma membrane part|GO:0070161,anchoring junction|GO:0031258,lamellipodium membrane|GO:0030027,lamellipodium|GO:0031256,leading edge membrane|GO:0098589,membrane region|GO:0031253,cell projection membrane|GO:0031252,cell leading edge|GO:0005912,adherens junction|GO:0005886,plasma membrane|GO:0032991,macromolecular complex|GO:1903561,extracellular vesicle|GO:0070062,extracellular exosome|GO:0043234,protein complex|GO:0043230,extracellular organelle|GO:0005829,cytosol|GO:0044463,cell projection part|GO:0044464,cell part|GO:0005623,cell|GO:0005622,intracellular|GO:0044444,cytoplasmic part|GO:0005576,extracellular region|GO:0044424,intracellular part|GO:0044425,membrane part|GO:0065010,extracellular membrane-bounded organelle|GO:0098805,whole membrane|GO:0099512,supramolecular fiber|GO:0015629,actin cytoskeleton|GO:0043229,intracellular organelle|GO:0043228,non-membrane-bounded organelle|GO:0099513,polymeric cytoskeletal fiber|GO:0005856,cytoskeleton|GO:0001726,ruffle|GO:0044430,cytoskeletal part|GO:0031941,filamentous actin|GO:0005884,actin filament|GO:0043232,intracellular non-membrane-bounded organelle|GO:0044446,intracellular organelle part|GO:0044422,organelle part	GO:0051020,GTPase binding|GO:0017048,Rho GTPase binding|GO:0017016,Ras GTPase binding|GO:0031267,small GTPase binding|GO:0048365,Rac GTPase binding|GO:0044877,macromolecular complex binding|GO:0019899,enzyme binding|GO:0005515,protein binding|GO:0032403,protein complex binding|GO:0005488,binding
MAL2	GO:0008104,protein localization|GO:0061024,membrane organization|GO:0001766,membrane raft polarization|GO:0007272,ensheathment of neurons|GO:0007275,multicellular organism development|GO:0044699,single-organism process|GO:0042552,myelination|GO:0045056,transcytosis|GO:0016043,cellular component organization|GO:0031579,membrane raft organization|GO:0071840,cellular component organization or biogenesis|GO:0006810,transport|GO:0033036,macromolecule localization|GO:0032502,developmental process|GO:0032501,multicellular organismal process|GO:0051641,cellular localization|GO:0031580,membrane raft distribution|GO:0044767,single-organism developmental process|GO:0044765,single-organism transport|GO:0044763,single-organism cellular process|GO:0008366,axon ensheathment|GO:0048731,system development|GO:0051668,localization within membrane|GO:0051234,establishment of localization|GO:0051179,localization|GO:1902578,single-organism localization|GO:0016192,vesicle-mediated transport|GO:0007399,nervous system development|GO:0044707,single-multicellular organism process|GO:0048856,anatomical structure development|GO:0044802,single-organism membrane organization|GO:0009987,cellular process|GO:1902580,single-organism cellular localization|GO:0051665,membrane raft localization	GO:0098590,plasma membrane region|GO:0044421,extracellular region part|GO:0071944,cell periphery|GO:0043227,membrane-bounded organelle|GO:0043226,organelle|GO:0031224,intrinsic component of membrane|GO:0005737,cytoplasm|GO:0045177,apical part of cell|GO:0031982,vesicle|GO:0098805,whole membrane|GO:0016020,membrane|GO:0031988,membrane-bounded vesicle|GO:0048471,perinuclear region of cytoplasm|GO:0044459,plasma membrane part|GO:0016324,apical plasma membrane|GO:0098589,membrane region|GO:0012505,endomembrane system|GO:0005886,plasma membrane|GO:1903561,extracellular vesicle|GO:0070062,extracellular exosome|GO:0043230,extracellular organelle|GO:0045121,membrane raft|GO:0044464,cell part|GO:0005623,cell|GO:0005622,intracellular|GO:0044444,cytoplasmic part|GO:0005576,extracellular region|GO:0044424,intracellular part|GO:0044425,membrane part|GO:0065010,extracellular membrane-bounded organelle|GO:0016021,integral component of	GO:0005488,binding|GO:0005515,protein binding|GO:0019911,structural constituent of myelin sheath|GO:0005198,structural molecule activity
RPL36A	GO:0008104,protein localization|GO:0061024,membrane organization|GO:1901362,organic cyclic compound biosynthetic process|GO:1901360,organic cyclic compound metabolic process|GO:1901361,organic cyclic compound catabolic process|GO:0070727,cellular macromolecule localization|GO:0043043,peptide biosynthetic process|GO:0044419,interspecies interaction between organisms|GO:0019058,viral life cycle|GO:0033036,macromolecule localization|GO:0051704,multi-organism process|GO:0006605,protein targeting|GO:0045184,establishment of protein localization|GO:0072657,protein localization to membrane|GO:0044403,symbiosis, encompassing mutualism through parasitism|GO:0046483,heterocycle metabolic process|GO:1901564,organonitrogen compound metabolic process|GO:1901566,organonitrogen compound biosynthetic process|GO:0019538,protein metabolic process|GO:0019439,aromatic compound catabolic process|GO:0019438,aromatic compound biosynthetic process|GO:0019080,viral gene expression|GO:0006613,cotranslational protein targeting to membrane|GO:0006612,protein targeting to membrane|GO:0006614,SRP-dependent cotranslational protein targeting to membrane|GO:0006807,nitrogen compound metabolic process|GO:0044033,multi-organism metabolic process|GO:1901576,organic substance biosynthetic process|GO:1901575,organic substance catabolic process|GO:0044265,cellular macromolecule catabolic process|GO:0044260,cellular macromolecule metabolic process|GO:0006886,intracellular protein transport|GO:0016043,cellular component organization|GO:0071840,cellular component organization or biogenesis|GO:0018130,heterocycle biosynthetic process|GO:0006810,transport|GO:0046700,heterocycle catabolic process|GO:0044802,single-organism membrane organization|GO:0008152,metabolic process|GO:0034654,nucleobase-containing compound biosynthetic process|GO:0034655,nucleobase-containing compound catabolic process|GO:0051234,establishment of localization|GO:0016070,RNA metabolic process|GO:0016071,mRNA metabolic process|GO:0044271,cellular nitrogen compound biosynthetic process|GO:0044270,cellular nitrogen compound catabolic process|GO:0046907,intracellular transport|GO:0044765,single-organism transport|GO:0045047,protein targeting to ER|GO:0044764,multi-organism cellular process|GO:0022411,cellular component disassembly|GO:0006518,peptide metabolic process|GO:0032774,RNA biosynthetic process|GO:0044248,cellular catabolic process|GO:0044249,cellular biosynthetic process|GO:0034641,cellular nitrogen compound metabolic process|GO:0034645,cellular macromolecule biosynthetic process|GO:0043241,protein complex disassembly|GO:0044699,single-organism process|GO:0006139,nucleobase-containing compound metabolic process|GO:0070972,protein localization to endoplasmic reticulum|GO:0051179,localization|GO:0002181,cytoplasmic translation|GO:0019083,viral transcription|GO:0072599,establishment of protein localization to endoplasmic reticulum|GO:0072594,establishment of protein localization to organelle|GO:0009987,cellular process|GO:0006725,cellular aromatic compound metabolic process|GO:0016482,cytosolic transport|GO:0043604,amide biosynthetic process|GO:0043603,cellular amide metabolic process|GO:0043170,macromolecule metabolic process|GO:0000956,nuclear-transcribed mRNA catabolic process|GO:0033365,protein localization to organelle|GO:0000184,nuclear-transcribed mRNA catabolic process, nonsense-mediated decay|GO:0043933,macromolecular complex subunit organization|GO:0090304,nucleic acid metabolic process|GO:0032984,macromolecular complex disassembly|GO:0071822,protein complex subunit organization|GO:0071704,organic substance metabolic process|GO:0010467,gene expression|GO:0071702,organic substance transport|GO:0006401,RNA catabolic process|GO:0006402,mRNA catabolic process|GO:0044267,cellular protein metabolic process|GO:0034613,cellular protein localization|GO:0009058,biosynthetic process|GO:0009059,macromolecule biosynthetic process|GO:0044763,single-organism cellular process|GO:0051649,establishment of localization in cell|GO:0043624,cellular protein complex disassembly|GO:0009056,catabolic process|GO:0009057,macromolecule catabolic process|GO:1902578,single-organism localization|GO:0051641,cellular localization|GO:0044238,primary metabolic process|GO:1902580,single-organism cellular localization|GO:0090150,establishment of protein localization to membrane|GO:0044237,cellular metabolic process|GO:0016032,viral process|GO:0015031,protein transport|GO:1902582,single-organism intracellular transport|GO:0006415,translational termination|GO:0006414,translational elongation|GO:0006413,translational initiation|GO:0006412,translation|GO:0019222,regulation of metabolic process|GO:0010605,negative regulation of macromolecule metabolic process|GO:0048519,negative regulation of biological process|GO:0006364,rRNA processing|GO:0034470,ncRNA processing|GO:0060255,regulation of macromolecule metabolic process|GO:0009892,negative regulation of metabolic process|GO:0010629,negative regulation of gene expression|GO:0034660,ncRNA metabolic process|GO:0050789,regulation of biological process|GO:0042254,ribosome biogenesis|GO:0065007,biological regulation|GO:0016072,rRNA metabolic process|GO:0015833,peptide transport|GO:0042886,amide transport|GO:0022613,ribonucleoprotein complex biogenesis|GO:0071705,nitrogen compound transport|GO:0010468,regulation of gene expression|GO:0044085,cellular component biogenesis|GO:0006396,RNA processing	GO:0022626,cytosolic ribosome|GO:0022625,cytosolic large ribosomal subunit|GO:0043229,intracellular organelle|GO:0043228,non-membrane-bounded organelle|GO:0015934,large ribosomal subunit|GO:0043226,organelle|GO:0005737,cytoplasm|GO:0030529,intracellular ribonucleoprotein complex|GO:0044391,ribosomal subunit|GO:0032991,macromolecular complex|GO:0005840,ribosome|GO:0043232,intracellular non-membrane-bounded organelle|GO:0005829,cytosol|GO:0044464,cell part|GO:0005623,cell|GO:0005622,intracellular|GO:0044446,intracellular organelle part|GO:0044444,cytoplasmic part|GO:0044445,cytosolic part|GO:0044424,intracellular part|GO:0044422,organelle part|GO:0016020,membrane|GO:0071944,cell periphery|GO:0005886,plasma membrane|GO:1990904,ribonucleoprotein complex|GO:0005844,polysome|GO:0005783,endoplasmic reticulum|GO:0012505,endomembrane system|GO:0042788,polysomal ribosome|GO:0043227,membrane-bounded organelle|GO:0043231,intracellular membrane-bounded organelle	GO:0097159,organic cyclic compound binding|GO:0044822,poly(A) RNA binding|GO:0005198,structural molecule activity|GO:0003735,structural constituent of ribosome|GO:0003723,RNA binding|GO:0003676,nucleic acid binding|GO:1901363,heterocyclic compound binding|GO:0005488,binding|GO:0005515,protein binding

PRDX3	GO:0043549,regulation of kinase activity|GO:0019220,regulation of phosphate metabolic process|GO:0080090,regulation of primary metabolic process|GO:0019222,regulation of metabolic process|GO:1901576,organic substance biosynthetic process|GO:1901362,organic cyclic compound biosynthetic process|GO:0007005,mitochondrion organization|GO:0044712,single-organism catabolic process|GO:0044710,single-organism metabolic process|GO:0010605,negative regulation of macromolecule metabolic process|GO:0010604,positive regulation of macromolecule metabolic process|GO:0043207,response to external biotic stimulus|GO:0048869,cellular developmental process|GO:0009617,response to bacterium|GO:0010467,gene expression|GO:0018193,peptidyl-amino acid modification|GO:0006979,response to oxidative stress|GO:0010466,negative regulation of peptidase activity|GO:0044093,positive regulation of molecular function|GO:0044092,negative regulation of molecular function|GO:0043281,regulation of cysteine-type endopeptidase activity involved in apoptotic process|GO:0048519,negative regulation of biological process|GO:0051704,multi-organism process|GO:0042127,regulation of cell proliferation|GO:0060255,regulation of macromolecule metabolic process|GO:0060548,negative regulation of cell death|GO:0032269,negative regulation of cellular protein metabolic process|GO:0003006,developmental process involved in reproduction|GO:0051090,regulation of sequence-specific DNA binding transcription factor activity|GO:0030162,regulation of proteolysis|GO:2001141,regulation of RNA biosynthetic process|GO:0010035,response to inorganic substance|GO:0051707,response to other organism|GO:0010033,response to organic substance|GO:0055114,oxidation-reduction process|GO:0046483,heterocycle metabolic process|GO:0042325,regulation of phosphorylation|GO:0044703,multi-organism reproductive process|GO:0042326,negative regulation of phosphorylation|GO:0009607,response to biotic stimulus|GO:0009605,response to external stimulus|GO:0044707,single-multicellular organism process|GO:0044706,multi-multicellular organism process|GO:0019538,protein metabolic process|GO:0002376,immune system process|GO:0033554,cellular response to stress|GO:0019438,aromatic compound biosynthetic process|GO:0033993,response to lipid|GO:0033673,negative regulation of kinase activity|GO:0042391,regulation of membrane potential|GO:0009892,negative regulation of metabolic process|GO:0009893,positive regulation of metabolic process|GO:0009891,positive regulation of biosynthetic process|GO:0000302,response to reactive oxygen species|GO:0010628,positive regulation of gene expression|GO:0042743,hydrogen peroxide metabolic process|GO:0000003,reproduction|GO:0042981,regulation of apoptotic process|GO:0050789,regulation of biological process|GO:0097659,nucleic acid-templated transcription|GO:0044267,cellular protein metabolic process|GO:0051346,negative regulation of hydrolase activity|GO:0044260,cellular macromolecule metabolic process|GO:0051348,negative regulation of transferase activity|GO:0016043,cellular component organization|GO:0019219,regulation of nucleobase-containing compound metabolic process|GO:0065007,biological regulation|GO:0071840,cellular component organization or biogenesis|GO:0065009,regulation of molecular function|GO:0065008,regulation of biological quality|GO:0018130,heterocycle biosynthetic process|GO:0016265,obsolete death|GO:0018158,protein oxidation|GO:0050790,regulation of catalytic activity|GO:0009889,regulation of biosynthetic process|GO:0051716,cellular response to stimulus|GO:0050794,regulation of cellular process|GO:0012501,programmed cell death|GO:0043412,macromolecule modification|GO:0036211,protein modification process|GO:1901700,response to oxygen-containing compound|GO:0008152,metabolic process|GO:0048731,system development|GO:0034654,nucleobase-containing compound biosynthetic process|GO:0044238,primary metabolic process|GO:0002520,immune system development|GO:0051336,regulation of hydrolase activity|GO:0044767,single-organism developmental process|GO:0044271,cellular nitrogen compound biosynthetic process|GO:0042542,response to hydrogen peroxide|GO:0050896,response to stimulus|GO:0006950,response to stress|GO:0051338,regulation of transferase activity|GO:0032502,developmental process|GO:0006355,regulation of transcription, DNA-templated|GO:0010557,positive regulation of macromolecule biosynthetic process|GO:0010556,regulation of macromolecule biosynthetic process|GO:0006351,transcription, DNA-templated|GO:0032774,RNA biosynthetic process|GO:0016310,phosphorylation|GO:0030154,cell differentiation|GO:0018198,peptidyl-cysteine modification|GO:0044248,cellular catabolic process|GO:0044249,cellular biosynthetic process|GO:0034641,cellular nitrogen compound metabolic process|GO:0070887,cellular response to chemical stimulus|GO:0043086,negative regulation of catalytic activity|GO:0044699,single-organism process|GO:0006139,nucleobase-containing compound metabolic process|GO:0051248,negative regulation of protein metabolic process|GO:0010563,negative regulation of phosphorus metabolic process|GO:0051246,regulation of protein metabolic process|GO:0048513,animal organ development|GO:0006508,proteolysis|GO:0008284,positive regulation of cell proliferation|GO:0016070,RNA metabolic process|GO:0018171,peptidyl-cysteine oxidation|GO:0032501,multicellular organismal process|GO:0048608,reproductive structure development|GO:0048609,multicellular organismal reproductive process|GO:0032504,multicellular organism reproduction|GO:0008283,cell proliferation|GO:0009987,cellular process|GO:0006725,cellular aromatic compound metabolic process|GO:1903506,regulation of nucleic acid-templated transcription|GO:0048518,positive regulation of biological process|GO:0034645,cellular macromolecule biosynthetic process|GO:0045893,positive regulation of transcription, DNA-templated|GO:0001893,maternal placenta development|GO:0001890,placenta development|GO:0051092,positive regulation of NF-kappaB transcription factor activity|GO:0051091,positive regulation of sequence-specific DNA binding transcription factor activity|GO:0032268,regulation of cellular protein metabolic process|GO:0007565,female pregnancy|GO:0051252,regulation of RNA metabolic process|GO:0051254,positive regulation of RNA metabolic process|GO:0043170,macromolecule metabolic process|GO:1902680,positive regulation of RNA biosynthetic process|GO:0042744,hydrogen peroxide catabolic process|GO:0002237,response to molecule of bacterial origin|GO:0030099,myeloid cell differentiation|GO:0030097,hemopoiesis|GO:0045861,negative regulation of proteolysis|GO:1903508,positive regulation of nucleic acid-templated transcription|GO:0072593,reactive oxygen species metabolic process|GO:0031328,positive regulation of cellular biosynthetic process|GO:0061458,reproductive system development|GO:0031326,regulation of cellular biosynthetic process|GO:0031325,positive regulation of cellular metabolic process|GO:0031324,negative regulation of cellular metabolic process|GO:0031323,regulation of cellular metabolic process|GO:0006807,nitrogen compound metabolic process|GO:0090304,nucleic acid metabolic process|GO:0060135,maternal process involved in female pregnancy|GO:0008219,cell death|GO:0010941,regulation of cell death|GO:0007275,multicellular organism development|GO:0043154,negative regulation of cysteine-type endopeptidase activity involved in apoptotic process|GO:2000116,regulation of cysteine-type endopeptidase activity|GO:2000117,negative regulation of cysteine-type endopeptidase activity|GO:1901360,organic cyclic compound metabolic process|GO:2000112,regulation of cellular macromolecule biosynthetic process|GO:0071704,organic substance metabolic process|GO:0043067,regulation of programmed cell death|GO:0043066,negative regulation of apoptotic process|GO:0048534,hematopoietic or lymphoid organ development|GO:0043069,negative regulation of programmed cell death|GO:0010468,regulation of gene expression|GO:0044702,single organism reproductive process|GO:0045935,positive regulation of nucleobase-containing compound metabolic process|GO:0032496,response to lipopolysaccharide|GO:0045936,negative regulation of phosphate metabolic process|GO:0052548,regulation of endopeptidase activity|GO:0006915,apoptotic process|GO:0034614,cellular response to reactive oxygen species|GO:0006464,cellular protein modification process|GO:0051174,regulation of phosphorus metabolic process|GO:0022414,reproductive process|GO:0009058,biosynthetic process|GO:0009059,macromolecule biosynthetic process|GO:0044763,single-organism cellular process|GO:0051171,regulation of nitrogen compound metabolic	GO:0044421,extracellular region part|GO:0031974,membrane-enclosed lumen|GO:0043229,intracellular organelle|GO:0043227,membrane-bounded organelle|GO:0043226,organelle|GO:0005737,cytoplasm|GO:0070062,extracellular exosome|GO:0043209,myelin sheath|GO:1902911,protein kinase complex|GO:0005739,mitochondrion|GO:0031988,membrane-bounded vesicle|GO:0005576,extracellular region|GO:0008385,IkappaB kinase complex|GO:1902494,catalytic complex|GO:0061695,transferase complex, transferring phosphorus-containing groups|GO:1902554,serine/threonine protein kinase complex|GO:0005759,mitochondrial matrix|GO:0012505,endomembrane system|GO:1990234,transferase complex|GO:0032991,macromolecular complex|GO:1903561,extracellular vesicle|GO:0031982,vesicle|GO:0043234,protein complex|GO:0043230,extracellular organelle|GO:0043231,intracellular membrane-bounded organelle|GO:0043233,organelle lumen|GO:0065010,extracellular membrane-bounded organelle|GO:0005829,cytosol|GO:0044464,cell part|GO:0005623,cell|GO:0005622,intracellular|GO:0044446,intracellular organelle part|GO:0070013,intracellular organelle lumen|GO:0044444,cytoplasmic part|GO:0044445,cytosolic part|GO:0044429,mitochondrial part|GO:0044424,intracellular part|GO:0005768,endosome|GO:0005769,early endosome|GO:0044422,organelle part|GO:0031410,cytoplasmic vesicle|GO:0097708,intracellular vesicle	GO:0008022,protein C-terminus binding|GO:0004866,endopeptidase inhibitor activity|GO:0019899,enzyme binding|GO:0061135,endopeptidase regulator activity|GO:0008379,thioredoxin peroxidase activity|GO:0004857,enzyme inhibitor activity|GO:0098772,molecular function regulator|GO:0016491,oxidoreductase activity|GO:0008785,alkyl hydroperoxide reductase activity|GO:0043028,cysteine-type endopeptidase regulator activity involved in apoptotic process|GO:0042802,identical protein binding|GO:0016667,oxidoreductase activity, acting on a sulfur group of donors|GO:0016668,oxidoreductase activity, acting on a sulfur group of donors, NAD(P) as acceptor|GO:0016684,oxidoreductase activity, acting on peroxide as acceptor|GO:0016209,antioxidant activity|GO:0043027,cysteine-type endopeptidase inhibitor activity involved in apoptotic process|GO:0030234,enzyme regulator activity|GO:0061134,peptidase regulator activity|GO:0019901,protein kinase binding|GO:0003824,catalytic activity|GO:0030414,peptidase inhibitor activity|GO:0004869,cysteine-type endopeptidase inhibitor activity|GO:0019900,kinase binding|GO:0051920,peroxiredoxin activity|GO:0016651,oxidoreductase activity, acting on NAD(P)H|GO:0005515,protein binding|GO:0004601,peroxidase activity|GO:0005488,binding
SLC35A3	GO:0015931,nucleobase-containing compound transport|GO:0006862,nucleotide transport|GO:0006820,anion transport|GO:0006807,nitrogen compound metabolic process|GO:0044281,small molecule metabolic process|GO:0051179,localization|GO:0044699,single-organism process|GO:1901264,carbohydrate derivative transport|GO:0006139,nucleobase-containing compound metabolic process|GO:0044710,single-organism metabolic process|GO:0015992,proton transport|GO:1901360,organic cyclic compound metabolic process|GO:0006796,phosphate-containing compound metabolic process|GO:0008643,carbohydrate transport|GO:0071705,nitrogen compound transport|GO:0071704,organic substance metabolic process|GO:0055086,nucleobase-containing small molecule metabolic process|GO:0006810,transport|GO:1990569,UDP-N-acetylglucosamine transmembrane transport|GO:1902578,single-organism localization|GO:0019637,organophosphate metabolic process|GO:0015672,monovalent inorganic cation transport|GO:0006818,hydrogen transport|GO:0044238,primary metabolic process|GO:0009225,nucleotide-sugar metabolic process|GO:0006812,cation transport|GO:0006811,ion transport|GO:0006793,phosphorus metabolic process|GO:0006725,cellular aromatic compound metabolic process|GO:0015711,organic anion transport|GO:0034220,ion transmembrane transport|GO:0044765,single-organism transport|GO:0009117,nucleotide metabolic process|GO:0008152,metabolic process|GO:0051234,establishment of localization|GO:0055085,transmembrane transport|GO:0072531,pyrimidine-containing compound transmembrane transport|GO:0046483,heterocycle metabolic process|GO:1901679,nucleotide transmembrane transport|GO:0015788,UDP-N-acetylglucosamine transport|GO:0071702,organic substance transport|GO:0006040,amino sugar metabolic process|GO:0015781,pyrimidine nucleotide-sugar transport|GO:0015780,nucleotide-sugar transport|GO:0034641,cellular nitrogen compound metabolic process|GO:0044237,cellular metabolic process|GO:0006047,UDP-N-acetylglucosamine metabolic process|GO:0090481,pyrimidine nucleotide-sugar transmembrane transport|GO:0015748,organophosphate ester transport|GO:0098656,anion transmembrane transport|GO:0044763,single-organism cellular process|GO:0009987,cellular process|GO:0006753,nucleoside phosphate metabolic process|GO:1901135,carbohydrate derivative metabolic process	GO:0043229,intracellular organelle|GO:0005622,intracellular|GO:0043227,membrane-bounded organelle|GO:0043226,organelle|GO:0031224,intrinsic component of membrane|GO:0005737,cytoplasm|GO:0031090,organelle membrane|GO:0016021,integral component of membrane|GO:0016020,membrane|GO:0044431,Golgi apparatus part|GO:0005794,Golgi apparatus|GO:0098588,bounding membrane of organelle|GO:0012505,endomembrane system|GO:0043231,intracellular membrane-bounded organelle|GO:0044464,cell part|GO:0005623,cell|GO:0000139,Golgi membrane|GO:0044446,intracellular organelle part|GO:0044444,cytoplasmic part|GO:0044424,intracellular part|GO:0044425,membrane part|GO:0044422,organelle part|GO:0031984,organelle subcompartment|GO:0098791,Golgi subcompartment	GO:0015932,nucleobase-containing compound transmembrane transporter activity|GO:0015605,organophosphate ester transmembrane transporter activity|GO:0005488,binding|GO:0015291,secondary active transmembrane transporter activity|GO:0015215,nucleotide transmembrane transporter activity|GO:0008509,anion transmembrane transporter activity|GO:0005462,UDP-N-acetylglucosamine transmembrane transporter activity|GO:0015165,pyrimidine nucleotide-sugar transmembrane transporter activity|GO:0005402,cation:sugar symporter activity|GO:0015144,carbohydrate transmembrane transporter activity|GO:0022804,active transmembrane transporter activity|GO:0015295,solute:proton symporter activity|GO:0015294,solute:cation symporter activity|GO:0005338,nucleotide-sugar transmembrane transporter activity|GO:0005351,sugar:proton symporter activity|GO:0015293,symporter activity|GO:0051119,sugar transmembrane transporter activity|GO:0005215,transporter activity|GO:0008324,cation transmembrane transporter activity|GO:0022891,substrate-specific transmembrane transporter activity|GO:0022892,substrate-specific transporter activity|GO:0008514,organic anion transmembrane transporter activity|GO:1901677,phosphate transmembrane transporter activity|GO:0015075,ion transmembrane transporter activity|GO:1901476,carbohydrate transporter activity|GO:0022857,transmembrane transporter
HDAC2	GO:0007599,hemostasis|GO:0034605,cellular response to heat|GO:0007596,blood coagulation|GO:0016358,dendrite development|GO:2000736,regulation of stem cell differentiation|GO:0098773,skin epidermis development|GO:0070933,histone H4 deacetylation|GO:0070932,histone H3 deacetylation|GO:0051716,cellular response to stimulus|GO:0014031,mesenchymal cell development|GO:0043207,response to external biotic stimulus|GO:0018212,peptidyl-tyrosine modification|GO:0016458,gene silencing|GO:0030855,epithelial cell differentiation|GO:0006473,protein acetylation|GO:0060548,negative regulation of cell death|GO:0046483,heterocycle metabolic process|GO:0042325,regulation of phosphorylation|GO:0042327,positive regulation of phosphorylation|GO:0009607,response to biotic stimulus|GO:0009605,response to external stimulus|GO:0019538,protein metabolic process|GO:0040029,regulation of gene expression, epigenetic|GO:0051130,positive regulation of cellular component organization|GO:0009892,negative regulation of metabolic process|GO:0009893,positive regulation of metabolic process|GO:0009890,negative regulation of biosynthetic process|GO:0009891,positive regulation of biosynthetic process|GO:0032760,positive regulation of tumor necrosis factor production|GO:0000302,response to reactive oxygen species|GO:0010869,regulation of receptor biosynthetic process|GO:0031175,neuron projection development|GO:0071868,cellular response to monoamine stimulus|GO:0071869,response to catecholamine|GO:0035556,intracellular signal transduction|GO:0050789,regulation of biological process|GO:0000904,cell morphogenesis involved in differentiation|GO:0000902,cell morphogenesis|GO:0001817,regulation of cytokine production|GO:1901360,organic cyclic compound metabolic process|GO:0060789,hair follicle placode formation|GO:0060788,ectodermal placode formation|GO:0007623,circadian rhythm|GO:0048709,oligodendrocyte differentiation|GO:0043412,macromolecule modification|GO:0042060,wound healing|GO:0009266,response to temperature stimulus|GO:0016070,RNA metabolic process|GO:0035094,response to nicotine|GO:0001975,response to amphetamine|GO:0014897,striated muscle hypertrophy|GO:0010557,positive regulation of macromolecule biosynthetic process|GO:0010556,regulation of macromolecule biosynthetic process|GO:0048869,cellular developmental process|GO:0097305,response to alcohol|GO:0010558,negative regulation of macromolecule biosynthetic process|GO:0043392,negative regulation of DNA binding|GO:0048149,behavioral response to ethanol|GO:0071242,cellular response to ammonium ion|GO:0051128,regulation of cellular component organization|GO:0010001,glial cell differentiation|GO:0098732,macromolecule deacylation|GO:0014074,response to purine-containing compound|GO:0014075,response to amine|GO:0014070,response to organic cyclic compound|GO:0000122,negative regulation of transcription from RNA polymerase II promoter|GO:0060284,regulation of cell development|GO:0008284,positive regulation of cell proliferation|GO:0035601,protein deacylation|GO:0050878,regulation of body fluid levels|GO:0008283,cell proliferation|GO:0001654,eye development|GO:0044259,multicellular organismal macromolecule metabolic process|GO:0044253,positive regulation of multicellular organismal metabolic process|GO:0006979,response to oxidative stress|GO:0006338,chromatin remodeling|GO:0042542,response to hydrogen peroxide|GO:1903351,cellular response to dopamine|GO:1903350,response to dopamine|GO:0030030,cell projection organization|GO:0018108,peptidyl-tyrosine phosphorylation|GO:0022404,molting cycle process|GO:0022405,hair cycle process|GO:0032652,regulation of interleukin-1 production|GO:0008219,cell death|GO:0055093,response to hyperoxia|GO:0007275,multicellular organism development|GO:0033993,response to lipid|GO:2000112,regulation of cellular macromolecule biosynthetic process|GO:2000113,negative regulation of cellular macromolecule biosynthetic process|GO:0043067,regulation of programmed cell death|GO:0043066,negative regulation of apoptotic process|GO:0061198,fungiform papilla formation|GO:0061197,fungiform papilla morphogenesis|GO:0061196,fungiform papilla development|GO:0043069,negative regulation of programmed cell death|GO:0048598,embryonic morphogenesis|GO:0006468,protein phosphorylation|GO:0019219,regulation of nucleobase-containing compound metabolic process|GO:0006464,cellular protein modification process|GO:0044767,single-organism developmental process|GO:0044763,single-organism cellular process|GO:0060485,mesenchyme development|GO:0042509,regulation of tyrosine phosphorylation of STAT protein|GO:1901701,cellular response to oxygen-containing compound|GO:0051276,chromosome organization|GO:0009913,epidermal cell differentiation|GO:0042503,tyrosine phosphorylation of Stat3 protein|GO:0048856,anatomical structure development|GO:0001837,epithelial to mesenchymal transition|GO:0006796,phosphate-containing compound metabolic process|GO:2000026,regulation of multicellular organismal development|GO:2000756,regulation of peptidyl-lysine acetylation|GO:2000757,negative regulation of peptidyl-lysine acetylation|GO:0043112,receptor metabolic process|GO:0048523,negative regulation of cellular process|GO:0048522,positive regulation of cellular process|GO:0007610,behavior|GO:0003012,muscle system process|GO:0007165,signal transduction|GO:0007166,cell surface receptor signaling pathway|GO:0007167,enzyme linked receptor protein signaling pathway|GO:0007169,transmembrane receptor protein tyrosine kinase signaling pathway|GO:0031345,negative regulation of cell projection organization|GO:0031344,regulation of cell projection organization|GO:0044710,single-organism metabolic process|GO:0044711,single-organism biosynthetic process|GO:0045786,negative regulation of cell cycle|GO:0070848,response to growth factor|GO:0043433,negative regulation of sequence-specific DNA binding transcription factor activity|GO:0045665,negative regulation of neuron differentiation|GO:0045664,regulation of neuron differentiation|GO:0060998,regulation of dendritic spine development|GO:0044092,negative regulation of molecular function|GO:0060996,dendritic spine development|GO:2001141,regulation of RNA biosynthetic process|GO:0010035,response to inorganic substance|GO:0051707,response to other organism|GO:0045814,negative regulation of gene expression, epigenetic|GO:0051704,multi-organism process|GO:0044249,cellular biosynthetic process|GO:0018205,peptidyl-lysine modification|GO:0050730,regulation of peptidyl-tyrosine phosphorylation|GO:0016568,chromatin modification|GO:0016569,covalent chromatin modification|GO:0032526,response to retinoic acid|GO:0061000,negative regulation of dendritic spine development|GO:0010629,negative regulation of gene expression|GO:0010628,positive regulation of gene expression|GO:0044267,cellular protein metabolic process|GO:0009653,anatomical structure morphogenesis|GO:0044260,cellular macromolecule metabolic process|GO:0044699,single-organism process|GO:0006366,transcription from RNA polymerase II promoter|GO:0009887,animal organ morphogenesis|GO:0050793,regulation of developmental process|GO:0009889,regulation of biosynthetic process|GO:0009888,tissue development|GO:0031000,response to caffeine|GO:0050794,regulation of cellular process|GO:0051239,regulation of multicellular organismal process|GO:0071870,cellular response to catecholamine stimulus|GO:1902679,negative regulation of RNA biosynthetic process|GO:0010718,positive regulation of epithelial to mesenchymal transition|GO:0010717,regulation of epithelial to mesenchymal transition|GO:0050896,response to stimulus|GO:0010714,positive regulation of collagen metabolic process|GO:0010712,regulation of collagen metabolic process|GO:0051962,positive regulation of nervous system development|GO:0014896,muscle hypertrophy|GO:0051960,regulation of nervous system development|GO:0043010,camera-type eye development|GO:0010562,positive regulation of phosphorus metabolic process|GO:0051246,regulation of protein metabolic process|GO:0032964,collagen biosynthetic process|GO:0032965,regulation of collagen biosynthetic process|GO:0032967,positive regulation of collagen biosynthetic process|GO:0070887,cellular response to chemical stimulus|GO:0032963,collagen metabolic process|GO:0007049,cell cycle|GO:0050767,regulation of neurogenesis|GO:0051248,negative regulation of protein metabolic process|GO:0051240,positive regulation of multicellular organismal process|GO:0051241,negative regulation of multicellular organismal process|GO:0003300,cardiac muscle hypertrophy|GO:0051247,positive regulation of protein metabolic	GO:0031974,membrane-enclosed lumen|GO:0043229,intracellular organelle|GO:0043228,non-membrane-bounded organelle|GO:0000785,chromatin|GO:0005623,cell|GO:0000228,nuclear chromosome|GO:0043227,membrane-bounded organelle|GO:0043226,organelle|GO:0034708,methyltransferase complex|GO:0005737,cytoplasm|GO:0070822,Sin3-type complex|GO:0070603,SWI/SNF superfamily-type complex|GO:0031519,PcG protein complex|GO:0031981,nuclear lumen|GO:0005634,nucleus|GO:0005654,nucleoplasm|GO:0044454,nuclear chromosome part|GO:0044451,nucleoplasm part|GO:0005694,chromosome|GO:1902494,catalytic complex|GO:0017053,transcriptional repressor complex|GO:0000118,histone deacetylase complex|GO:0000790,nuclear chromatin|GO:1990234,transferase complex|GO:0090568,nuclear transcriptional repressor complex|GO:0032991,macromolecular complex|GO:0090545,CHD-type complex|GO:0043231,intracellular membrane-bounded organelle|GO:0043234,protein complex|GO:0035098,ESC/E(Z) complex|GO:0016580,Sin3 complex|GO:0016581,NuRD complex|GO:0043232,intracellular non-membrane-bounded organelle|GO:0043233,organelle lumen|GO:0044464,cell part|GO:0035097,histone methyltransferase complex|GO:0005622,intracellular|GO:0044446,intracellular organelle part|GO:0070013,intracellular organelle lumen|GO:0044428,nuclear part|GO:0044424,intracellular part|GO:0044427,chromosomal part|GO:0044422,organelle part	GO:0032041,NAD-dependent histone deacetylase activity (H3-K14 specific)|GO:0031078,histone deacetylase activity (H3-K14 specific)|GO:0031072,heat shock protein binding|GO:0001012,RNA polymerase II regulatory region DNA binding|GO:0019899,enzyme binding|GO:0001159,core promoter proximal region DNA binding|GO:0001158,enhancer sequence-specific DNA binding|GO:0031491,nucleosome binding|GO:0031490,chromatin DNA binding|GO:0031492,nucleosomal DNA binding|GO:0044877,macromolecular complex binding|GO:0005488,binding|GO:0003676,nucleic acid binding|GO:0003677,DNA binding|GO:1901363,heterocyclic compound binding|GO:0070491,repressing transcription factor binding|GO:0043565,sequence-specific DNA binding|GO:0043566,structure-specific DNA binding|GO:0004407,histone deacetylase activity|GO:0000987,core promoter proximal region sequence-specific DNA binding|GO:0008134,transcription factor binding|GO:0000980,RNA polymerase II distal enhancer sequence-specific DNA binding|GO:0016787,hydrolase activity|GO:0019213,deacetylase activity|GO:0001067,regulatory region nucleic acid binding|GO:0044822,poly(A) RNA binding|GO:0017136,NAD-dependent histone deacetylase activity|GO:0051059,NF-kappaB binding|GO:0003824,catalytic activity|GO:0035326,enhancer binding|GO:0001047,core promoter binding|GO:0001103,RNA polymerase II repressing transcription factor binding|GO:0000978,RNA polymerase II core promoter proximal region sequence-specific DNA binding|GO:0001085,RNA polymerase II transcription factor binding|GO:0044212,transcription regulatory region DNA binding|GO:0097159,organic cyclic compound binding|GO:0016810,hydrolase activity, acting on carbon-nitrogen (but not peptide) bonds|GO:0016811,hydrolase activity, acting on carbon-nitrogen (but not peptide) bonds, in linear amides|GO:0032403,protein complex binding|GO:0034979,NAD-dependent protein deacetylase activity|GO:0000976,transcription regulatory region sequence-specific DNA binding|GO:0000977,RNA polymerase II regulatory region sequence-specific DNA binding|GO:0005515,protein binding|GO:0000975,regulatory region DNA binding|GO:0003723,RNA binding|GO:0033558,protein deacetylase activity|GO:0003682,chromatin binding|GO:1990837,sequence-specific double-stranded DNA binding|GO:0003690,double-stranded DNA binding|GO:1990841,promoter-specific chromatin binding|GO:0042826,histone deacetylase binding
PANK1	GO:1901566,organonitrogen compound biosynthetic process|GO:0055086,nucleobase-containing small molecule metabolic process|GO:0015936,coenzyme A metabolic process|GO:0015937,coenzyme A biosynthetic process|GO:0006732,coenzyme metabolic process|GO:0015939,pantothenate metabolic process|GO:0044249,cellular biosynthetic process|GO:0034641,cellular nitrogen compound metabolic process|GO:0009165,nucleotide biosynthetic process|GO:0009108,coenzyme biosynthetic process|GO:0009163,nucleoside biosynthetic process|GO:0072521,purine-containing compound metabolic process|GO:0072522,purine-containing compound biosynthetic process|GO:1901362,organic cyclic compound biosynthetic process|GO:0046129,purine ribonucleoside biosynthetic process|GO:0044699,single-organism process|GO:0006139,nucleobase-containing compound metabolic process|GO:0044710,single-organism metabolic process|GO:0044711,single-organism biosynthetic process|GO:0009987,cellular process|GO:0090407,organophosphate biosynthetic process|GO:1901360,organic cyclic compound metabolic process|GO:0042278,purine nucleoside metabolic process|GO:0033875,ribonucleoside bisphosphate metabolic process|GO:0051188,cofactor biosynthetic process|GO:0009259,ribonucleotide metabolic process|GO:0071704,organic substance metabolic process|GO:0034032,purine nucleoside bisphosphate metabolic process|GO:0016310,phosphorylation|GO:0046483,heterocycle metabolic process|GO:0044281,small molecule metabolic process|GO:0018130,heterocycle biosynthetic process|GO:0006767,water-soluble vitamin metabolic process|GO:0006766,vitamin metabolic process|GO:0046390,ribose phosphate biosynthetic process|GO:1901576,organic substance biosynthetic process|GO:0006793,phosphorus metabolic process|GO:0006725,cellular aromatic compound metabolic process|GO:0009152,purine ribonucleotide biosynthetic process|GO:0009150,purine ribonucleotide metabolic process|GO:0009260,ribonucleotide biosynthetic process|GO:0009058,biosynthetic process|GO:0051186,cofactor metabolic process|GO:0009117,nucleotide metabolic process|GO:0009116,nucleoside metabolic process|GO:0008152,metabolic process|GO:0019438,aromatic compound biosynthetic process|GO:0034654,nucleobase-containing compound biosynthetic process|GO:1901564,organonitrogen compound metabolic process|GO:0009119,ribonucleoside metabolic process|GO:0046128,purine ribonucleoside metabolic process|GO:0034030,ribonucleoside bisphosphate biosynthetic process|GO:0042455,ribonucleoside biosynthetic process|GO:0034033,purine nucleoside bisphosphate biosynthetic process|GO:0044238,primary metabolic process|GO:0044271,cellular nitrogen compound biosynthetic process|GO:0006575,cellular modified amino acid metabolic process|GO:0043603,cellular amide metabolic process|GO:1901137,carbohydrate derivative biosynthetic process|GO:1901135,carbohydrate derivative metabolic process|GO:0033866,nucleoside bisphosphate biosynthetic process|GO:0019693,ribose phosphate metabolic process|GO:0033865,nucleoside bisphosphate metabolic process|GO:0006163,purine nucleotide metabolic process|GO:1901657,glycosyl compound metabolic process|GO:0006796,phosphate-containing compound metabolic process|GO:0006807,nitrogen compound metabolic process|GO:0042451,purine nucleoside biosynthetic process|GO:1901293,nucleoside phosphate biosynthetic process|GO:0006164,purine nucleotide biosynthetic process|GO:0019637,organophosphate metabolic process|GO:0044763,single-organism cellular process|GO:0044237,cellular metabolic process|GO:0006753,nucleoside phosphate metabolic process|GO:1901659,glycosyl compound biosynthetic process	GO:0055037,recycling endosome|GO:0043229,intracellular organelle|GO:0071944,cell periphery|GO:0030117,membrane coat|GO:0030118,clathrin coat|GO:0043227,membrane-bounded organelle|GO:0043226,organelle|GO:0005737,cytoplasm|GO:0005634,nucleus|GO:0016020,membrane|GO:0048475,coated membrane|GO:0012505,endomembrane system|GO:0043234,protein complex|GO:0032991,macromolecular complex|GO:0043231,intracellular membrane-bounded organelle|GO:0005829,cytosol|GO:0044464,cell part|GO:0005623,cell|GO:0005622,intracellular|GO:0098796,membrane protein complex|GO:0044444,cytoplasmic part|GO:0044424,intracellular part|GO:0044425,membrane part|GO:0005768,endosome|GO:0031982,vesicle|GO:0031410,cytoplasmic vesicle|GO:0097708,intracellular vesicle	GO:0035639,purine ribonucleoside triphosphate binding|GO:0005488,binding|GO:0043167,ion binding|GO:1901363,heterocyclic compound binding|GO:1901265,nucleoside phosphate binding|GO:0001882,nucleoside binding|GO:0043168,anion binding|GO:0000166,nucleotide binding|GO:0016740,transferase activity|GO:0001883,purine nucleoside binding|GO:0032549,ribonucleoside binding|GO:0017076,purine nucleotide binding|GO:0005524,ATP binding|GO:0016301,kinase activity|GO:0036094,small molecule binding|GO:0003824,catalytic activity|GO:0030554,adenyl nucleotide binding|GO:0016773,phosphotransferase activity, alcohol group as acceptor|GO:0016772,transferase activity, transferring phosphorus-containing groups|GO:0097367,carbohydrate derivative binding|GO:0032559,adenyl ribonucleotide binding|GO:0097159,organic cyclic compound binding|GO:0032555,purine ribonucleotide binding|GO:0004594,pantothenate kinase activity|GO:0032550,purine ribonucleoside binding|GO:0032553,ribonucleotide binding
HIBCH	GO:0019752,carboxylic acid metabolic process|GO:0044248,cellular catabolic process|GO:0034641,cellular nitrogen compound metabolic process|GO:0006807,nitrogen compound metabolic process|GO:0044281,small molecule metabolic process|GO:0044282,small molecule catabolic process|GO:0044699,single-organism process|GO:0044712,single-organism catabolic process|GO:0044710,single-organism metabolic process|GO:0006520,cellular amino acid metabolic process|GO:0071704,organic substance metabolic process|GO:1901605,alpha-amino acid metabolic process|GO:1901606,alpha-amino acid catabolic process|GO:0044238,primary metabolic process|GO:0009987,cellular process|GO:1901575,organic substance catabolic process|GO:0044763,single-organism cellular process|GO:0008152,metabolic process|GO:0043436,oxoacid metabolic process|GO:1901564,organonitrogen compound metabolic process|GO:0009056,catabolic process|GO:0009063,cellular amino acid catabolic process|GO:0006573,valine metabolic process|GO:0006574,valine catabolic process|GO:1901565,organonitrogen compound catabolic process|GO:0006082,organic acid metabolic process|GO:0046395,carboxylic acid catabolic process|GO:0016054,organic acid catabolic process|GO:0044237,cellular metabolic process|GO:0009081,branched-chain amino acid metabolic process|GO:0009083,branched-chain amino acid catabolic process	GO:0031974,membrane-enclosed lumen|GO:0043229,intracellular organelle|GO:0043227,membrane-bounded organelle|GO:0043226,organelle|GO:0005737,cytoplasm|GO:0070062,extracellular exosome|GO:0005739,mitochondrion|GO:0031988,membrane-bounded vesicle|GO:0005759,mitochondrial matrix|GO:1903561,extracellular vesicle|GO:0031982,vesicle|GO:0043230,extracellular organelle|GO:0043231,intracellular membrane-bounded organelle|GO:0043233,organelle lumen|GO:0044464,cell part|GO:0005623,cell|GO:0005622,intracellular|GO:0044446,intracellular organelle part|GO:0070013,intracellular organelle lumen|GO:0044444,cytoplasmic part|GO:0005576,extracellular region|GO:0044429,mitochondrial part|GO:0044424,intracellular part|GO:0065010,extracellular membrane-bounded organelle|GO:0044421,extracellular region part|GO:0044422,organelle part	GO:0016787,hydrolase activity|GO:0016289,CoA hydrolase activity|GO:0016790,thiolester hydrolase activity|GO:0016788,hydrolase activity, acting on ester bonds|GO:0003824,catalytic activity|GO:0003860,3-hydroxyisobutyryl-CoA hydrolase activity
NNAT	GO:0090087,regulation of peptide transport|GO:0032024,positive regulation of insulin secretion|GO:0008104,protein localization|GO:0051046,regulation of secretion|GO:0051047,positive regulation of secretion|GO:0051049,regulation of transport|GO:0060322,head development|GO:0048869,cellular developmental process|GO:0046879,hormone secretion|GO:0048513,animal organ development|GO:0048518,positive regulation of biological process|GO:0033036,macromolecule localization|GO:0051050,positive regulation of transport|GO:0045184,establishment of protein localization|GO:0090276,regulation of peptide hormone secretion|GO:0090277,positive regulation of peptide hormone secretion|GO:0044700,single organism signaling|GO:0065008,regulation of biological quality|GO:0044707,single-multicellular organism process|GO:0019538,protein metabolic process|GO:0023051,regulation of signaling|GO:0032940,secretion by cell|GO:0051222,positive regulation of protein transport|GO:0051223,regulation of protein transport|GO:0050789,regulation of biological process|GO:0030072,peptide hormone secretion|GO:0030073,insulin secretion|GO:0044267,cellular protein metabolic process|GO:0050708,regulation of protein secretion|GO:0044260,cellular macromolecule metabolic process|GO:0065007,biological regulation|GO:0032880,regulation of protein localization|GO:0070201,regulation of establishment of protein localization|GO:0009306,protein secretion|GO:0006810,transport|GO:0050796,regulation of insulin secretion|GO:0050794,regulation of cellular process|GO:0043412,macromolecule modification|GO:0036211,protein modification process|GO:0006464,cellular protein modification process|GO:0008152,metabolic process|GO:0051234,establishment of localization|GO:0046903,secretion|GO:0007420,brain development|GO:0050714,positive regulation of protein secretion|GO:0030154,cell differentiation|GO:0023056,positive regulation of signaling|GO:0015833,peptide transport|GO:0023052,signaling|GO:1903530,regulation of secretion by cell|GO:1903532,positive regulation of secretion by cell|GO:0010647,positive regulation of cell communication|GO:0010646,regulation of cell communication|GO:0044699,single-organism process|GO:0007417,central nervous system development|GO:0042886,amide transport|GO:0032502,developmental process|GO:0009249,protein lipoylation|GO:0032501,multicellular organismal process|GO:0009987,cellular process|GO:0046883,regulation of hormone secretion|GO:0046887,positive regulation of hormone secretion|GO:0032879,regulation of localization|GO:0043170,macromolecule metabolic process|GO:0048731,system development|GO:0002793,positive regulation of peptide secretion|GO:0060341,regulation of cellular localization|GO:0018065,protein-cofactor linkage|GO:0007275,multicellular organism development|GO:0071705,nitrogen compound transport|GO:0071704,organic substance metabolic process|GO:0071702,organic substance transport|GO:0030182,neuron differentiation|GO:0023061,signal release|GO:0010817,regulation of hormone levels|GO:0044767,single-organism developmental process|GO:0044765,single-organism transport|GO:0044763,single-organism cellular process|GO:0051649,establishment of localization in cell|GO:0007267,cell-cell signaling|GO:0007154,cell communication|GO:0022008,neurogenesis|GO:0051179,localization|GO:1902578,single-organism localization|GO:0051641,cellular localization|GO:0044238,primary metabolic process|GO:0048699,generation of neurons|GO:0007399,nervous system development|GO:0002790,peptide secretion|GO:0002791,regulation of peptide secretion|GO:0048856,anatomical structure development|GO:0044237,cellular metabolic process|GO:0009914,hormone transport|GO:0015031,protein transport|GO:0048522,positive regulation of cellular process|GO:1901564,organonitrogen compound metabolic process|GO:0006807,nitrogen compound metabolic process|GO:1904951,positive regulation of establishment of protein localization	GO:0005737,cytoplasm|GO:0044464,cell part|GO:0005623,cell|GO:0005622,intracellular|GO:0044424,intracellular part	
OIP5	GO:0034728,nucleosome organization|GO:0034080,CENP-A containing nucleosome assembly|GO:0022607,cellular component assembly|GO:0070271,protein complex biogenesis|GO:0043933,macromolecular complex subunit organization|GO:0034508,centromere complex assembly|GO:1903047,mitotic cell cycle process|GO:0022402,cell cycle process|GO:0016043,cellular component organization|GO:0031497,chromatin assembly|GO:0034622,cellular macromolecular complex assembly|GO:0071840,cellular component organization or biogenesis|GO:0007067,mitotic nuclear division|GO:0044699,single-organism process|GO:0071824,protein-DNA complex subunit organization|GO:0071822,protein complex subunit organization|GO:0006325,chromatin organization|GO:0043486,histone exchange|GO:0071103,DNA conformation change|GO:0043044,ATP-dependent chromatin remodeling|GO:0000280,nuclear division|GO:0006323,DNA packaging|GO:0065003,macromolecular complex assembly|GO:0051301,cell division|GO:0065004,protein-DNA complex assembly|GO:0031055,chromatin remodeling at centromere|GO:0009987,cellular process|GO:0000278,mitotic cell cycle|GO:0034724,DNA replication-independent nucleosome organization|GO:0006461,protein complex assembly|GO:0044763,single-organism cellular process|GO:0007154,cell communication|GO:0006996,organelle organization|GO:0007049,cell cycle|GO:0051276,chromosome organization|GO:0006338,chromatin remodeling|GO:0006336,DNA replication-independent nucleosome assembly|GO:0006334,nucleosome assembly|GO:0006333,chromatin assembly or disassembly|GO:1902589,single-organism organelle organization|GO:0044085,cellular component biogenesis|GO:0048285,organelle fission|GO:0061641,CENP-A containing chromatin organization|GO:0016568,chromatin modification|GO:0007059,chromosome segregation	GO:0031974,membrane-enclosed lumen|GO:0043229,intracellular organelle|GO:0043228,non-membrane-bounded organelle|GO:0000785,chromatin|GO:0005622,intracellular|GO:0043227,membrane-bounded organelle|GO:0043226,organelle|GO:0005737,cytoplasm|GO:0016604,nuclear body|GO:0031981,nuclear lumen|GO:0005634,nucleus|GO:0005654,nucleoplasm|GO:0044451,nucleoplasm part|GO:0005694,chromosome|GO:0000775,chromosome, centromeric region|GO:0098687,chromosomal region|GO:0032991,macromolecular complex|GO:0043231,intracellular membrane-bounded organelle|GO:0043232,intracellular non-membrane-bounded organelle|GO:0043233,organelle lumen|GO:0044464,cell part|GO:0005623,cell|GO:0010369,chromocenter|GO:0044446,intracellular organelle part|GO:0070013,intracellular organelle lumen|GO:0044428,nuclear part|GO:0044424,intracellular part|GO:0015030,Cajal body|GO:0044427,chromosomal part|GO:0044422,organelle part|GO:0016607,nuclear speck|GO:0005829,cytosol|GO:0044444,cytoplasmic part	GO:0005488,binding|GO:0005515,protein binding|GO:0043169,cation binding|GO:0043167,ion binding|GO:0046872,metal ion binding|GO:0042802,identical protein binding
SLC6A19	GO:0009991,response to extracellular stimulus|GO:0006865,amino acid transport|GO:0031667,response to nutrient levels|GO:0006820,anion transport|GO:0044699,single-organism process|GO:0051179,localization|GO:0071705,nitrogen compound transport|GO:0071702,organic substance transport|GO:0007584,response to nutrient|GO:0006810,transport|GO:0015849,organic acid transport|GO:0006811,ion transport|GO:0009987,cellular process|GO:0015711,organic anion transport|GO:0015804,neutral amino acid transport|GO:0034220,ion transmembrane transport|GO:0044765,single-organism transport|GO:0044763,single-organism cellular process|GO:0006836,neurotransmitter transport|GO:0042221,response to chemical|GO:0051234,establishment of localization|GO:0055085,transmembrane transport|GO:1902578,single-organism localization|GO:0046942,carboxylic acid transport|GO:0009605,response to external stimulus|GO:0003333,amino acid transmembrane transport|GO:1903825,organic acid transmembrane transport|GO:0098656,anion transmembrane transport|GO:0050896,response to stimulus|GO:1905039,carboxylic acid transmembrane transport	GO:0098590,plasma membrane region|GO:0044421,extracellular region part|GO:0005903,brush border|GO:0031226,intrinsic component of plasma membrane|GO:0043226,organelle|GO:0031224,intrinsic component of membrane|GO:0042995,cell projection|GO:0031982,vesicle|GO:0043227,membrane-bounded organelle|GO:0098805,whole membrane|GO:0016020,membrane|GO:0031988,membrane-bounded vesicle|GO:0005576,extracellular region|GO:0044459,plasma membrane part|GO:0098589,membrane region|GO:0031253,cell projection membrane|GO:0005887,integral component of plasma membrane|GO:0005886,plasma membrane|GO:1903561,extracellular vesicle|GO:0070062,extracellular exosome|GO:0043230,extracellular organelle|GO:0044463,cell projection part|GO:0044464,cell part|GO:0005623,cell|GO:0031526,brush border membrane|GO:0071944,cell periphery|GO:0044425,membrane part|GO:0065010,extracellular membrane-bounded organelle|GO:0016021,integral component of membrane|GO:0098862,cluster of actin-based cell projections|GO:0016324,apical plasma membrane|GO:0045177,apical part of cell	GO:0005328,neurotransmitter:sodium symporter activity|GO:0005326,neurotransmitter transporter activity|GO:0005342,organic acid transmembrane transporter activity|GO:0015081,sodium ion transmembrane transporter activity|GO:0008509,anion transmembrane transporter activity|GO:0022857,transmembrane transporter activity|GO:0015370,solute:sodium symporter activity|GO:0046873,metal ion transmembrane transporter activity|GO:0022804,active transmembrane transporter activity|GO:0015294,solute:cation symporter activity|GO:0015291,secondary active transmembrane transporter activity|GO:0015293,symporter activity|GO:0005215,transporter activity|GO:0008324,cation transmembrane transporter activity|GO:0046943,carboxylic acid transmembrane transporter activity|GO:0022891,substrate-specific transmembrane transporter activity|GO:0022890,inorganic cation transmembrane transporter activity|GO:0022892,substrate-specific transporter activity|GO:0008514,organic anion transmembrane transporter activity|GO:0015075,ion transmembrane transporter activity|GO:0015077,monovalent inorganic cation transmembrane transporter activity|GO:0015175,neutral amino acid transmembrane transporter activity|GO:0015171,amino acid transmembrane transporter activity

CARTPT	GO:0090087,regulation of peptide transport|GO:0019226,transmission of nerve impulse|GO:0008104,protein localization|GO:0019220,regulation of phosphate metabolic process|GO:0080090,regulation of primary metabolic process|GO:0019222,regulation of metabolic process|GO:0051047,positive regulation of secretion|GO:0051048,negative regulation of secretion|GO:0051049,regulation of transport|GO:0048585,negative regulation of response to stimulus|GO:0048584,positive regulation of response to stimulus|GO:0048583,regulation of response to stimulus|GO:0032147,activation of protein kinase activity|GO:0007610,behavior|GO:0032844,regulation of homeostatic process|GO:0044710,single-organism metabolic process|GO:0007165,signal transduction|GO:0051937,catecholamine transport|GO:0001894,tissue homeostasis|GO:0023014,signal transduction by protein phosphorylation|GO:0009267,cellular response to starvation|GO:0007218,neuropeptide signaling pathway|GO:0010604,positive regulation of macromolecule metabolic process|GO:0071704,organic substance metabolic process|GO:0009966,regulation of signal transduction|GO:0009967,positive regulation of signal transduction|GO:0060249,anatomical structure homeostasis|GO:0000165,MAPK cascade|GO:0045453,bone resorption|GO:0046879,hormone secretion|GO:0048511,rhythmic process|GO:0048513,animal organ development|GO:0048869,cellular developmental process|GO:0044093,positive regulation of molecular function|GO:0048518,positive regulation of biological process|GO:0048519,negative regulation of biological process|GO:0033036,macromolecule localization|GO:0051954,positive regulation of amine transport|GO:0019725,cellular homeostasis|GO:0015844,monoamine transport|GO:0051051,negative regulation of transport|GO:0051050,positive regulation of transport|GO:0060255,regulation of macromolecule metabolic process|GO:0045859,regulation of protein kinase activity|GO:0032922,circadian regulation of gene expression|GO:0031668,cellular response to extracellular stimulus|GO:0048771,tissue remodeling|GO:0045184,establishment of protein localization|GO:0090276,regulation of peptide hormone secretion|GO:0032845,negative regulation of homeostatic process|GO:0090278,negative regulation of peptide hormone secretion|GO:1903707,negative regulation of hemopoiesis|GO:0003008,system process|GO:0042325,regulation of phosphorylation|GO:0044700,single organism signaling|GO:0042327,positive regulation of phosphorylation|GO:0031669,cellular response to nutrient levels|GO:0044707,single-multicellular organism process|GO:0048871,multicellular organismal homeostasis|GO:0019538,protein metabolic process|GO:0044708,single-organism behavior|GO:0008015,blood circulation|GO:0031667,response to nutrient levels|GO:0048878,chemical homeostasis|GO:0002376,immune system process|GO:0007154,cell communication|GO:0003013,circulatory system process|GO:0046849,bone remodeling|GO:0033554,cellular response to stress|GO:1903531,negative regulation of secretion by cell|GO:0051649,establishment of localization in cell|GO:1903530,regulation of secretion by cell|GO:0007631,feeding behavior|GO:0051716,cellular response to stimulus|GO:0009893,positive regulation of metabolic process|GO:0033674,positive regulation of kinase activity|GO:1903532,positive regulation of secretion by cell|GO:0070201,regulation of establishment of protein localization|GO:0008152,metabolic process|GO:0032940,secretion by cell|GO:0065007,biological regulation|GO:0035556,intracellular signal transduction|GO:0051223,regulation of protein transport|GO:0050789,regulation of biological process|GO:0048242,epinephrine secretion|GO:0030073,insulin secretion|GO:0002761,regulation of myeloid leukocyte differentiation|GO:0044267,cellular protein metabolic process|GO:0002762,negative regulation of myeloid leukocyte differentiation|GO:0051347,positive regulation of transferase activity|GO:0032095,regulation of response to food|GO:0032096,negative regulation of response to food|GO:0032098,regulation of appetite|GO:0032099,negative regulation of appetite|GO:0043549,regulation of kinase activity|GO:0009306,protein secretion|GO:0046851,negative regulation of bone remodeling|GO:0046850,regulation of bone remodeling|GO:0002682,regulation of immune system process|GO:0002683,negative regulation of immune system process|GO:0065009,regulation of molecular function|GO:0032880,regulation of protein localization|GO:0007186,G-protein coupled receptor signaling pathway|GO:0051971,positive regulation of transmission of nerve impulse|GO:0048241,epinephrine transport|GO:0032812,positive regulation of epinephrine secretion|GO:0033605,positive regulation of catecholamine secretion|GO:0007623,circadian rhythm|GO:0051046,regulation of secretion|GO:0050793,regulation of developmental process|GO:0050790,regulation of catalytic activity|GO:0050796,regulation of insulin secretion|GO:0050794,regulation of cellular process|GO:0043410,positive regulation of MAPK cascade|GO:0043412,macromolecule modification|GO:0036211,protein modification process|GO:0006464,cellular protein modification process|GO:0051239,regulation of multicellular organismal process|GO:0048731,system development|GO:1902533,positive regulation of intracellular signal transduction|GO:1902531,regulation of intracellular signal transduction|GO:0002521,leukocyte differentiation|GO:0002520,immune system development|GO:0044767,single-organism developmental process|GO:0046903,secretion|GO:0051240,positive regulation of multicellular organismal process|GO:0031644,regulation of neurological system process|GO:0031646,positive regulation of neurological system process|GO:0031401,positive regulation of protein modification process|GO:0015850,organic hydroxy compound transport|GO:0006950,response to stress|GO:0051338,regulation of transferase activity|GO:0009605,response to external stimulus|GO:0045638,negative regulation of myeloid cell differentiation|GO:0051952,regulation of amine transport|GO:0051969,regulation of transmission of nerve impulse|GO:0045637,regulation of myeloid cell differentiation|GO:0032107,regulation of response to nutrient levels|GO:0032104,regulation of response to extracellular stimulus|GO:0032105,negative regulation of response to extracellular stimulus|GO:0032102,negative regulation of response to external stimulus|GO:0001678,cellular glucose homeostasis|GO:0035637,multicellular organismal signaling|GO:0032101,regulation of response to external stimulus|GO:0016310,phosphorylation|GO:0030154,cell differentiation|GO:0032108,negative regulation of response to nutrient levels|GO:0023056,positive regulation of signaling|GO:0023057,negative regulation of signaling|GO:0015833,peptide transport|GO:0023052,signaling|GO:0010648,negative regulation of cell communication|GO:0015837,amine transport|GO:0023051,regulation of signaling|GO:0010647,positive regulation of cell communication|GO:0010646,regulation of cell communication|GO:0050432,catecholamine secretion|GO:0050433,regulation of catecholamine secretion|GO:0043085,positive regulation of catalytic activity|GO:0043408,regulation of MAPK cascade|GO:1903706,regulation of hemopoiesis|GO:0042886,amide transport|GO:0051234,establishment of localization|GO:0044057,regulation of system process|GO:0010562,positive regulation of phosphorus metabolic process|GO:0051241,negative regulation of multicellular organismal process|GO:0051246,regulation of protein metabolic process|GO:0051247,positive regulation of protein metabolic process|GO:0032270,positive regulation of cellular protein metabolic process|GO:0006810,transport|GO:0031399,regulation of protein modification process|GO:0032502,developmental process|GO:0032501,multicellular organismal process|GO:0044238,primary metabolic process|GO:0050877,neurological system process|GO:0030316,osteoclast differentiation|GO:0045596,negative regulation of cell differentiation|GO:0045595,regulation of cell differentiation|GO:0046883,regulation of hormone secretion|GO:0046887,positive regulation of hormone secretion|GO:0046888,negative regulation of hormone secretion|GO:0055082,cellular chemical homeostasis|GO:0032879,regulation of localization|GO:0014060,regulation of epinephrine secretion|GO:0051093,negative regulation of developmental process|GO:0042221,response to chemical|GO:0044699,single-organism process|GO:0032268,regulation of cellular protein metabolic process|GO:0065008,regulation of biological quality|GO:0043170,macromolecule metabolic process|GO:0045777,positive regulation of blood pressure|GO:0030099,myeloid cell differentiation|GO:0030097,hemopoiesis|GO:0009987,cellular process|GO:0045860,positive regulation of protein kinase activity|GO:0050896,response to stimulus|GO:0000186,activation of MAPKK activity|GO:0009991,response to extracellular	GO:0005737,cytoplasm|GO:0031982,vesicle|GO:0016023,cytoplasmic, membrane-bounded vesicle|GO:0030141,secretory granule|GO:0044464,cell part|GO:0044444,cytoplasmic part|GO:0005623,cell|GO:0031988,membrane-bounded vesicle|GO:0044421,extracellular region part|GO:0043229,intracellular organelle|GO:0005576,extracellular region|GO:0005615,extracellular space|GO:0012505,endomembrane system|GO:0031410,cytoplasmic vesicle|GO:0044424,intracellular part|GO:0005622,intracellular|GO:0043227,membrane-bounded organelle|GO:0043226,organelle|GO:0043231,intracellular membrane-bounded organelle|GO:0099503,secretory vesicle|GO:0097708,intracellular vesicle	GO:0005488,binding|GO:0005515,protein binding|GO:0005179,hormone activity|GO:0005184,neuropeptide hormone activity|GO:0005102,receptor binding
AC068987	GO:0042391,regulation of membrane potential|GO:0019226,transmission of nerve impulse|GO:0051234,establishment of localization|GO:0035637,multicellular organismal signaling|GO:0051049,regulation of transport|GO:0050877,neurological system process|GO:0019228,neuronal action potential|GO:0051899,membrane depolarization|GO:0023052,signaling|GO:0001508,action potential|GO:0034765,regulation of ion transmembrane transport|GO:0050789,regulation of biological process|GO:0043269,regulation of ion transport|GO:0032879,regulation of localization|GO:0051179,localization|GO:0035725,sodium ion transmembrane transport|GO:0065007,biological regulation|GO:0034762,regulation of transmembrane transport|GO:0006810,transport|GO:0098660,inorganic ion transmembrane transport|GO:0065008,regulation of biological quality|GO:0015672,monovalent inorganic cation transport|GO:0098662,inorganic cation transmembrane transport|GO:0032501,multicellular organismal process|GO:0006812,cation transport|GO:0006811,ion transport|GO:0009987,cellular process|GO:0006814,sodium ion transport|GO:0034220,ion transmembrane transport|GO:0044765,single-organism transport|GO:0044763,single-organism cellular process|GO:0007154,cell communication|GO:0050794,regulation of cellular process|GO:0055085,transmembrane transport|GO:1902578,single-organism localization|GO:0003008,system process|GO:0044700,single organism signaling|GO:0044699,single-organism process|GO:0044707,single-multicellular organism process|GO:0086010,membrane depolarization during action potential|GO:0030001,metal ion transport|GO:0098655,cation transmembrane transport|GO:0060078,regulation of postsynaptic membrane potential	GO:0034706,sodium channel complex|GO:0071944,cell periphery|GO:0034702,ion channel complex|GO:0034703,cation channel complex|GO:0031226,intrinsic component of plasma membrane|GO:0031224,intrinsic component of membrane|GO:0016021,integral component of membrane|GO:0016020,membrane|GO:0044459,plasma membrane part|GO:1902495,transmembrane transporter complex|GO:1990351,transporter complex|GO:0005887,integral component of plasma membrane|GO:0005886,plasma membrane|GO:0001518,voltage-gated sodium channel complex|GO:0043234,protein complex|GO:0032991,macromolecular complex|GO:0044464,cell part|GO:0005623,cell|GO:0098797,plasma membrane protein complex|GO:0098796,membrane protein complex|GO:0044425,membrane part	GO:0005261,cation channel activity|GO:0015081,sodium ion transmembrane transporter activity|GO:0015077,monovalent inorganic cation transmembrane transporter activity|GO:0022843,voltage-gated cation channel activity|GO:0022803,passive transmembrane transporter activity|GO:0046873,metal ion transmembrane transporter activity|GO:0005272,sodium channel activity|GO:0005215,transporter activity|GO:0005216,ion channel activity|GO:0008324,cation transmembrane transporter activity|GO:0022891,substrate-specific transmembrane transporter activity|GO:0022890,inorganic cation transmembrane transporter activity|GO:0022892,substrate-specific transporter activity|GO:0005244,voltage-gated ion channel activity|GO:0015075,ion transmembrane transporter activity|GO:0022832,voltage-gated channel activity|GO:0022857,transmembrane transporter activity|GO:0015267,channel activity|GO:0022836,gated channel activity|GO:0005248,voltage-gated sodium channel activity|GO:0022838,substrate-specific channel activity|GO:1905030,voltage-gated ion channel activity involved in regulation of postsynaptic membrane
OCLN	GO:0042278,purine nucleoside metabolic process|GO:0008152,metabolic process|GO:0006921,cellular component disassembly involved in execution phase of apoptosis|GO:0070271,protein complex biogenesis|GO:0097194,execution phase of apoptosis|GO:0034330,cell junction organization|GO:0019752,carboxylic acid metabolic process|GO:0006807,nitrogen compound metabolic process|GO:0034641,cellular nitrogen compound metabolic process|GO:0022607,cellular component assembly|GO:0007043,cell-cell junction assembly|GO:0044281,small molecule metabolic process|GO:0072521,purine-containing compound metabolic process|GO:0008219,cell death|GO:0046498,S-adenosylhomocysteine metabolic process|GO:0044699,single-organism process|GO:0006139,nucleobase-containing compound metabolic process|GO:0044710,single-organism metabolic process|GO:0051186,cofactor metabolic process|GO:0071822,protein complex subunit organization|GO:0071704,organic substance metabolic process|GO:1901360,organic cyclic compound metabolic process|GO:0006520,cellular amino acid metabolic process|GO:0016043,cellular component organization|GO:0065003,macromolecular complex assembly|GO:0043933,macromolecular complex subunit organization|GO:0071840,cellular component organization or biogenesis|GO:0032259,methylation|GO:1901605,alpha-amino acid metabolic process|GO:0046500,S-adenosylmethionine metabolic process|GO:0016265,obsolete death|GO:0006461,protein complex assembly|GO:0006732,coenzyme metabolic process|GO:0006915,apoptotic process|GO:0006725,cellular aromatic compound metabolic process|GO:0012501,programmed cell death|GO:0022411,cellular component disassembly|GO:0009116,nucleoside metabolic process|GO:0045216,cell-cell junction organization|GO:0043436,oxoacid metabolic process|GO:0009119,ribonucleoside metabolic process|GO:0046128,purine ribonucleoside metabolic process|GO:0034329,cell junction assembly|GO:0055086,nucleobase-containing small molecule metabolic process|GO:0046483,heterocycle metabolic process|GO:0044238,primary metabolic process|GO:1901564,organonitrogen compound metabolic process|GO:0006575,cellular modified amino acid metabolic process|GO:0006082,organic acid metabolic process|GO:1901135,carbohydrate derivative metabolic process|GO:0070830,bicellular tight junction assembly|GO:0044237,cellular metabolic process|GO:1901657,glycosyl compound metabolic process|GO:0044085,cellular component biogenesis|GO:0006790,sulfur compound metabolic process|GO:0044763,single-organism cellular process|GO:0009987,cellular process|GO:0043297,apical junction assembly|GO:0070887,cellular response to chemical stimulus|GO:0051716,cellular response to stimulus|GO:0071356,cellular response to tumor necrosis factor|GO:0010033,response to organic substance|GO:0071310,cellular response to organic substance|GO:0034097,response to cytokine|GO:0034612,response to tumor necrosis factor|GO:0042221,response to chemical|GO:0071345,cellular response to cytokine stimulus|GO:0050896,response to stimulus|GO:0070673,response to interleukin-18|GO:1901888,regulation of cell junction assembly|GO:0051128,regulation of cellular component organization|GO:0044087,regulation of cellular component biogenesis|GO:2000810,regulation of bicellular tight junction assembly|GO:0065007,biological regulation|GO:0050794,regulation of cellular process|GO:0050789,regulation of biological process	GO:0098590,plasma membrane region|GO:0030139,endocytic vesicle|GO:0043229,intracellular organelle|GO:0071944,cell periphery|GO:0043227,membrane-bounded organelle|GO:0005923,bicellular tight junction|GO:0031224,intrinsic component of membrane|GO:0005737,cytoplasm|GO:0045177,apical part of cell|GO:0031982,vesicle|GO:0016023,cytoplasmic, membrane-bounded vesicle|GO:0031410,cytoplasmic vesicle|GO:0016021,integral component of membrane|GO:0016020,membrane|GO:0031988,membrane-bounded vesicle|GO:0030054,cell junction|GO:0044459,plasma membrane part|GO:0016327,apicolateral plasma membrane|GO:0016324,apical plasma membrane|GO:0070160,occluding junction|GO:0098589,membrane region|GO:0005911,cell-cell junction|GO:0005886,plasma membrane|GO:0043231,intracellular membrane-bounded organelle|GO:0005829,cytosol|GO:0044464,cell part|GO:0005623,cell|GO:0005622,intracellular|GO:0044444,cytoplasmic part|GO:0043226,organelle|GO:0044424,intracellular part|GO:0044425,membrane part|GO:0043296,apical junction complex|GO:0098805,whole membrane|GO:0016328,lateral plasma membrane|GO:0097708,intracellular vesicle	GO:0008119,thiopurine S-methyltransferase activity|GO:0008172,S-methyltransferase activity|GO:0003824,catalytic activity|GO:0016740,transferase activity|GO:0016741,transferase activity, transferring one-carbon groups|GO:0019904,protein domain specific binding|GO:0005515,protein binding|GO:0008757,S-adenosylmethionine-dependent methyltransferase activity|GO:0008168,methyltransferase activity|GO:0005488,binding
ZEB2	GO:0080090,regulation of primary metabolic process|GO:0019222,regulation of metabolic process|GO:0032438,melanosome organization|GO:0048066,developmental pigmentation|GO:1901362,organic cyclic compound biosynthetic process|GO:0044707,single-multicellular organism process|GO:0071840,cellular component organization or biogenesis|GO:0044710,single-organism metabolic process|GO:0044711,single-organism biosynthetic process|GO:0010605,negative regulation of macromolecule metabolic process|GO:0010604,positive regulation of macromolecule metabolic process|GO:0044550,secondary metabolite biosynthetic process|GO:0048869,cellular developmental process|GO:0010467,gene expression|GO:0030856,regulation of epithelial cell differentiation|GO:0030855,epithelial cell differentiation|GO:0048518,positive regulation of biological process|GO:0048519,negative regulation of biological process|GO:0030858,positive regulation of epithelial cell differentiation|GO:0002065,columnar/cuboidal epithelial cell differentiation|GO:0060255,regulation of macromolecule metabolic process|GO:0050932,regulation of pigment cell differentiation|GO:0050931,pigment cell differentiation|GO:0048070,regulation of developmental pigmentation|GO:2001141,regulation of RNA biosynthetic process|GO:0046483,heterocycle metabolic process|GO:0016477,cell migration|GO:0010631,epithelial cell migration|GO:1900378,positive regulation of secondary metabolite biosynthetic process|GO:0048870,cell motility|GO:1900376,regulation of secondary metabolite biosynthetic process|GO:0016050,vesicle organization|GO:0019438,aromatic compound biosynthetic process|GO:0009892,negative regulation of metabolic process|GO:0048087,positive regulation of developmental pigmentation|GO:0009890,negative regulation of biosynthetic process|GO:0009891,positive regulation of biosynthetic process|GO:0050942,positive regulation of pigment cell differentiation|GO:0006928,movement of cell or subcellular component|GO:0008152,metabolic process|GO:0010629,negative regulation of gene expression|GO:0010628,positive regulation of gene expression|GO:0050789,regulation of biological process|GO:0097659,nucleic acid-templated transcription|GO:1901576,organic substance biosynthetic process|GO:0044260,cellular macromolecule metabolic process|GO:0016043,cellular component organization|GO:0046189,phenol-containing compound biosynthetic process|GO:0065007,biological regulation|GO:1901360,organic cyclic compound metabolic process|GO:0006366,transcription from RNA polymerase II promoter|GO:0065009,regulation of molecular function|GO:0048023,positive regulation of melanin biosynthetic process|GO:0018130,heterocycle biosynthetic process|GO:0006582,melanin metabolic process|GO:0050793,regulation of developmental process|GO:0050790,regulation of catalytic activity|GO:0009889,regulation of biosynthetic process|GO:0009888,tissue development|GO:0050794,regulation of cellular process|GO:0042438,melanin biosynthetic process|GO:0051239,regulation of multicellular organismal process|GO:0034654,nucleobase-containing compound biosynthetic process|GO:0042440,pigment metabolic process|GO:0046148,pigment biosynthetic process|GO:0016070,RNA metabolic process|GO:0044767,single-organism developmental process|GO:0044271,cellular nitrogen compound biosynthetic process|GO:0006355,regulation of transcription, DNA-templated|GO:0010557,positive regulation of macromolecule biosynthetic process|GO:0010556,regulation of macromolecule biosynthetic process|GO:0006351,transcription, DNA-templated|GO:0045636,positive regulation of melanocyte differentiation|GO:0010558,negative regulation of macromolecule biosynthetic process|GO:0045634,regulation of melanocyte differentiation|GO:1901615,organic hydroxy compound metabolic process|GO:0032774,RNA biosynthetic process|GO:0033043,regulation of organelle organization|GO:0030154,cell differentiation|GO:0051128,regulation of cellular component organization|GO:0044249,cellular biosynthetic process|GO:0034641,cellular nitrogen compound metabolic process|GO:0034645,cellular macromolecule biosynthetic process|GO:0001667,ameboidal-type cell migration|GO:0048021,regulation of melanin biosynthetic process|GO:0044699,single-organism process|GO:0009893,positive regulation of metabolic process|GO:0006139,nucleobase-containing compound metabolic process|GO:0000122,negative regulation of transcription from RNA polymerase II promoter|GO:1902913,positive regulation of neuroepithelial cell differentiation|GO:0048753,pigment granule organization|GO:0060563,neuroepithelial cell differentiation|GO:1903508,positive regulation of nucleic acid-templated transcription|GO:0040011,locomotion|GO:0032501,multicellular organismal process|GO:0030318,melanocyte differentiation|GO:0044238,primary metabolic process|GO:0060429,epithelium development|GO:1903056,regulation of melanosome organization|GO:1903506,regulation of nucleic acid-templated transcription|GO:1903507,negative regulation of nucleic acid-templated transcription|GO:0045597,positive regulation of cell differentiation|GO:0045595,regulation of cell differentiation|GO:0045892,negative regulation of transcription, DNA-templated|GO:0045893,positive regulation of transcription, DNA-templated|GO:0018958,phenol-containing compound metabolic process|GO:0090132,epithelium migration|GO:0090130,tissue migration|GO:0043473,pigmentation|GO:0051674,localization of cell|GO:0051094,positive regulation of developmental process|GO:0051253,negative regulation of RNA metabolic process|GO:0051252,regulation of RNA metabolic process|GO:0051254,positive regulation of RNA metabolic process|GO:0043170,macromolecule metabolic process|GO:1902680,positive regulation of RNA biosynthetic process|GO:0006807,nitrogen compound metabolic process|GO:0045944,positive regulation of transcription from RNA polymerase II promoter|GO:0097324,melanocyte migration|GO:0048731,system development|GO:0032502,developmental process|GO:0031328,positive regulation of cellular biosynthetic process|GO:0031327,negative regulation of cellular biosynthetic process|GO:0031326,regulation of cellular biosynthetic process|GO:0031325,positive regulation of cellular metabolic process|GO:0031324,negative regulation of cellular metabolic process|GO:0031323,regulation of cellular metabolic process|GO:0090304,nucleic acid metabolic process|GO:0007275,multicellular organism development|GO:2000112,regulation of cellular macromolecule biosynthetic process|GO:2000113,negative regulation of cellular macromolecule biosynthetic process|GO:0071704,organic substance metabolic process|GO:0033059,cellular pigmentation|GO:0006357,regulation of transcription from RNA polymerase II promoter|GO:0010468,regulation of gene expression|GO:0009987,cellular process|GO:0045935,positive regulation of nucleobase-containing compound metabolic process|GO:0045934,negative regulation of nucleobase-containing compound metabolic process|GO:0019219,regulation of nucleobase-containing compound metabolic process|GO:0006725,cellular aromatic compound metabolic process|GO:0019748,secondary metabolic process|GO:1902679,negative regulation of RNA biosynthetic process|GO:0009058,biosynthetic process|GO:0009059,macromolecule biosynthetic process|GO:0044763,single-organism cellular process|GO:0051171,regulation of nitrogen compound metabolic process|GO:0051172,negative regulation of nitrogen compound metabolic process|GO:0051173,positive regulation of nitrogen compound metabolic process|GO:0051179,localization|GO:0006996,organelle organization|GO:0043455,regulation of secondary metabolic process|GO:0007399,nervous system development|GO:0048856,anatomical structure development|GO:0044237,cellular metabolic process|GO:1901617,organic hydroxy compound biosynthetic process|GO:2000026,regulation of multicellular organismal development|GO:0048523,negative regulation of cellular process|GO:0048522,positive regulation of cellular process|GO:0019220,regulation of phosphate metabolic process|GO:0048588,developmental cell growth|GO:0048584,positive regulation of response to stimulus|GO:0048583,regulation of response to stimulus|GO:0001756,somitogenesis|GO:0030111,regulation of Wnt signaling pathway|GO:0060322,head development|GO:0007165,signal transduction|GO:0007166,cell surface receptor signaling pathway|GO:0030182,neuron differentiation|GO:1905114,cell surface receptor signaling pathway involved in cell-cell signaling|GO:0023014,signal transduction by protein phosphorylation|GO:0031346,positive regulation of cell projection organization|GO:0014032,neural crest cell development|GO:0014033,neural crest cell differentiation|GO:0009966,regulation of signal transduction|GO:0009967,positive regulation of signal	GO:0043231,intracellular membrane-bounded organelle|GO:0005634,nucleus|GO:0044464,cell part|GO:0005623,cell|GO:0005622,intracellular|GO:0043229,intracellular organelle|GO:0044424,intracellular part|GO:0043227,membrane-bounded organelle|GO:0043226,organelle|GO:0031974,membrane-enclosed lumen|GO:0043228,non-membrane-bounded organelle|GO:0005737,cytoplasm|GO:0031981,nuclear lumen|GO:0005730,nucleolus|GO:0043232,intracellular non-membrane-bounded organelle|GO:0043233,organelle lumen|GO:0005829,cytosol|GO:0044446,intracellular organelle part|GO:0070013,intracellular organelle lumen|GO:0044444,cytoplasmic part|GO:0044428,nuclear part|GO:0044422,organelle part|GO:0044454,nuclear chromosome part|GO:0000790,nuclear chromatin|GO:0000785,chromatin|GO:0000228,nuclear chromosome|GO:0044427,chromosomal part|GO:0005694,chromosome	GO:0030234,enzyme regulator activity|GO:0043169,cation binding|GO:0046872,metal ion binding|GO:0097159,organic cyclic compound binding|GO:0019208,phosphatase regulator activity|GO:0043167,ion binding|GO:0005488,binding|GO:0003676,nucleic acid binding|GO:0003677,DNA binding|GO:0098772,molecular function regulator|GO:1901363,heterocyclic compound binding|GO:0001228,transcriptional activator activity, RNA polymerase II transcription regulatory region sequence-specific binding|GO:0001071,nucleic acid binding transcription factor activity|GO:0070412,R-SMAD binding|GO:0000981,RNA polymerase II transcription factor activity, sequence-specific DNA binding|GO:0046332,SMAD binding|GO:0001205,transcriptional activator activity, RNA polymerase II distal enhancer sequence-specific binding|GO:0001227,transcriptional repressor activity, RNA polymerase II transcription regulatory region sequence-specific binding|GO:0003700,transcription factor activity, sequence-specific DNA binding|GO:0003705,transcription factor activity, RNA polymerase II distal enhancer sequence-specific binding|GO:0005515,protein binding|GO:0043565,sequence-specific DNA binding
DCP1A	GO:0000184,nuclear-transcribed mRNA catabolic process, nonsense-mediated decay|GO:0090304,nucleic acid metabolic process|GO:0034641,cellular nitrogen compound metabolic process|GO:0006807,nitrogen compound metabolic process|GO:1901360,organic cyclic compound metabolic process|GO:1901361,organic cyclic compound catabolic process|GO:0006139,nucleobase-containing compound metabolic process|GO:1901575,organic substance catabolic process|GO:0044265,cellular macromolecule catabolic process|GO:0000288,nuclear-transcribed mRNA catabolic process, deadenylation-dependent decay|GO:0044260,cellular macromolecule metabolic process|GO:0009056,catabolic process|GO:0071704,organic substance metabolic process|GO:0010467,gene expression|GO:0006401,RNA catabolic process|GO:0006402,mRNA catabolic process|GO:0044238,primary metabolic process|GO:0009987,cellular process|GO:0006725,cellular aromatic compound metabolic process|GO:0046700,heterocycle catabolic process|GO:0008152,metabolic process|GO:0034655,nucleobase-containing compound catabolic process|GO:0043928,exonucleolytic nuclear-transcribed mRNA catabolic process involved in deadenylation-dependent decay|GO:0009057,macromolecule catabolic process|GO:0044248,cellular catabolic process|GO:0046483,heterocycle metabolic process|GO:0016070,RNA metabolic process|GO:0016071,mRNA metabolic process|GO:0000291,nuclear-transcribed mRNA catabolic process, exonucleolytic|GO:0044270,cellular nitrogen compound catabolic process|GO:0044237,cellular metabolic process|GO:0043170,macromolecule metabolic process|GO:0000956,nuclear-transcribed mRNA catabolic process|GO:0019439,aromatic compound catabolic process|GO:0033036,macromolecule localization|GO:0009892,negative regulation of metabolic process|GO:0019222,regulation of metabolic process|GO:0090305,nucleic acid phosphodiester bond hydrolysis|GO:0010629,negative regulation of gene expression|GO:0051641,cellular localization|GO:0051179,localization|GO:0050789,regulation of biological process|GO:0043085,positive regulation of catalytic activity|GO:0043488,regulation of mRNA stability|GO:0043487,regulation of RNA stability|GO:0010608,posttranscriptional regulation of gene expression|GO:0008104,protein localization|GO:0065007,biological regulation|GO:0044093,positive regulation of molecular function|GO:0048519,negative regulation of biological process|GO:0065008,regulation of biological quality|GO:0010468,regulation of gene expression|GO:0034613,cellular protein localization|GO:0060255,regulation of macromolecule metabolic process|GO:0050790,regulation of catalytic activity|GO:0033365,protein localization to organelle|GO:0010605,negative regulation of macromolecule metabolic process|GO:1903608,protein localization to cytoplasmic stress granule|GO:0065009,regulation of molecular function|GO:0031086,nuclear-transcribed mRNA catabolic process, deadenylation-independent decay|GO:0031087,deadenylation-independent decapping of nuclear-transcribed mRNA|GO:0000290,deadenylation-dependent decapping of nuclear-transcribed mRNA|GO:0070727,cellular macromolecule localization	GO:0005737,cytoplasm|GO:0005829,cytosol|GO:0035770,ribonucleoprotein granule|GO:0043231,intracellular membrane-bounded organelle|GO:0043232,intracellular non-membrane-bounded organelle|GO:0044464,cell part|GO:0005634,nucleus|GO:0016020,membrane|GO:0000932,P-body|GO:0044444,cytoplasmic part|GO:0005623,cell|GO:0030529,intracellular ribonucleoprotein complex|GO:0043229,intracellular organelle|GO:0043228,non-membrane-bounded organelle|GO:0036464,cytoplasmic ribonucleoprotein granule|GO:0044424,intracellular part|GO:0032991,macromolecular complex|GO:0005622,intracellular|GO:0043227,membrane-bounded organelle|GO:0043226,organelle|GO:1990904,ribonucleoprotein complex	GO:0016787,hydrolase activity|GO:0003824,catalytic activity|GO:0005488,binding|GO:0005515,protein binding|GO:0042802,identical protein binding|GO:0019894,kinesin binding|GO:0008092,cytoskeletal protein binding|GO:0097159,organic cyclic compound binding|GO:0030234,enzyme regulator activity|GO:0008047,enzyme activator activity|GO:0003676,nucleic acid binding|GO:0003723,RNA binding|GO:0098772,molecular function regulator|GO:1901363,heterocyclic compound binding|GO:0003729,mRNA binding
C1orf216			
FOXJ3	GO:0048856,anatomical structure development|GO:0080090,regulation of primary metabolic process|GO:0019222,regulation of metabolic process|GO:0031326,regulation of cellular biosynthetic process|GO:0030154,cell differentiation|GO:0031323,regulation of cellular metabolic process|GO:0090304,nucleic acid metabolic process|GO:0044249,cellular biosynthetic process|GO:0034641,cellular nitrogen compound metabolic process|GO:0006807,nitrogen compound metabolic process|GO:0034645,cellular macromolecule biosynthetic process|GO:0009653,anatomical structure morphogenesis|GO:1901362,organic cyclic compound biosynthetic process|GO:0050789,regulation of biological process|GO:0044699,single-organism process|GO:0032774,RNA biosynthetic process|GO:0006139,nucleobase-containing compound metabolic process|GO:0044260,cellular macromolecule metabolic process|GO:0071704,organic substance metabolic process|GO:0048869,cellular developmental process|GO:1901360,organic cyclic compound metabolic process|GO:2000112,regulation of cellular macromolecule biosynthetic process|GO:0060255,regulation of macromolecule metabolic process|GO:0010467,gene expression|GO:0006357,regulation of transcription from RNA polymerase II promoter|GO:0065007,biological regulation|GO:0097659,nucleic acid-templated transcription|GO:0006366,transcription from RNA polymerase II promoter|GO:0010468,regulation of gene expression|GO:0018130,heterocycle biosynthetic process|GO:0032502,developmental process|GO:1901576,organic substance biosynthetic process|GO:0019219,regulation of nucleobase-containing compound metabolic process|GO:0006725,cellular aromatic compound metabolic process|GO:0009987,cellular process|GO:0009889,regulation of biosynthetic process|GO:1903506,regulation of nucleic acid-templated transcription|GO:0050794,regulation of cellular process|GO:0044767,single-organism developmental process|GO:0009058,biosynthetic process|GO:0009059,macromolecule biosynthetic process|GO:0044763,single-organism cellular process|GO:0051171,regulation of nitrogen compound metabolic process|GO:0008152,metabolic process|GO:2001141,regulation of RNA biosynthetic process|GO:0034654,nucleobase-containing compound biosynthetic process|GO:0046483,heterocycle metabolic process|GO:0016070,RNA metabolic process|GO:0044238,primary metabolic process|GO:0044271,cellular nitrogen compound biosynthetic process|GO:0051252,regulation of RNA metabolic process|GO:0044237,cellular metabolic process|GO:0043170,macromolecule metabolic process|GO:0006355,regulation of transcription, DNA-templated|GO:0010556,regulation of macromolecule biosynthetic process|GO:0006351,transcription, DNA-templated|GO:0019438,aromatic compound biosynthetic process|GO:1903508,positive regulation of nucleic acid-templated transcription|GO:0045935,positive regulation of nucleobase-containing compound metabolic process|GO:0010604,positive regulation of macromolecule metabolic process|GO:0031328,positive regulation of cellular biosynthetic process|GO:0031325,positive regulation of cellular metabolic process|GO:0009891,positive regulation of biosynthetic process|GO:0051254,positive regulation of RNA metabolic process|GO:1902680,positive regulation of RNA biosynthetic process|GO:0010557,positive regulation of macromolecule biosynthetic process|GO:0010628,positive regulation of gene expression|GO:0045944,positive regulation of transcription from RNA polymerase II promoter|GO:0045893,positive regulation of transcription, DNA-templated|GO:0051173,positive regulation of nitrogen compound metabolic process|GO:0048518,positive regulation of biological process|GO:0048522,positive regulation of cellular process|GO:0009893,positive regulation of metabolic process	GO:0043231,intracellular membrane-bounded organelle|GO:0005634,nucleus|GO:0044464,cell part|GO:0005623,cell|GO:0005622,intracellular|GO:0043229,intracellular organelle|GO:0044424,intracellular part|GO:0043227,membrane-bounded organelle|GO:0043226,organelle	GO:0043565,sequence-specific DNA binding|GO:0003700,transcription factor activity, sequence-specific DNA binding|GO:0097159,organic cyclic compound binding|GO:0005488,binding|GO:0003676,nucleic acid binding|GO:0003677,DNA binding|GO:1901363,heterocyclic compound binding|GO:0000981,RNA polymerase II transcription factor activity, sequence-specific DNA binding|GO:0001071,nucleic acid binding transcription factor activity|GO:0001228,transcriptional activator activity, RNA polymerase II transcription regulatory region sequence-specific binding
STARD7	GO:0033157,regulation of intracellular protein transport|GO:0008104,protein localization|GO:0032388,positive regulation of intracellular transport|GO:0060341,regulation of cellular localization|GO:0033043,regulation of organelle organization|GO:1903651,positive regulation of cytoplasmic transport|GO:0032386,regulation of intracellular transport|GO:0051649,establishment of localization in cell|GO:0051128,regulation of cellular component organization|GO:1903827,regulation of cellular protein localization|GO:0010821,regulation of mitochondrion organization|GO:1903533,regulation of protein targeting|GO:0010822,positive regulation of mitochondrion organization|GO:0071840,cellular component organization or biogenesis|GO:0051222,positive regulation of protein transport|GO:0051223,regulation of protein transport|GO:0050789,regulation of biological process|GO:1903829,positive regulation of cellular protein localization|GO:0032880,regulation of protein localization|GO:0051234,establishment of localization|GO:0070727,cellular macromolecule localization|GO:0006605,protein targeting|GO:0051641,cellular localization|GO:0016043,cellular component organization|GO:0065007,biological regulation|GO:0044699,single-organism process|GO:0051049,regulation of transport|GO:0048518,positive regulation of biological process|GO:0016482,cytosolic transport|GO:0034613,cellular protein localization|GO:0051130,positive regulation of cellular component organization|GO:0070201,regulation of establishment of protein localization|GO:0006810,transport|GO:1903649,regulation of cytoplasmic transport|GO:0051050,positive regulation of transport|GO:0072594,establishment of protein localization to organelle|GO:0006626,protein targeting to mitochondrion|GO:0009987,cellular process|GO:0090316,positive regulation of intracellular protein transport|GO:0050794,regulation of cellular process|GO:0045184,establishment of protein localization|GO:1903747,regulation of establishment of protein localization to mitochondrion|GO:1903955,positive regulation of protein targeting to mitochondrion|GO:0071702,organic substance transport|GO:0072655,establishment of protein localization to mitochondrion|GO:0006839,mitochondrial transport|GO:0032879,regulation of localization|GO:0051179,localization|GO:1902578,single-organism localization|GO:1903749,positive regulation of establishment of protein localization to mitochondrion|GO:0006996,organelle organization|GO:0070585,protein localization to mitochondrion|GO:0033036,macromolecule localization|GO:0046907,intracellular transport|GO:0007005,mitochondrion organization|GO:0010638,positive regulation of organelle organization|GO:0044765,single-organism transport|GO:0033365,protein localization to organelle|GO:1903214,regulation of protein targeting to mitochondrion|GO:0015031,protein transport|GO:1902582,single-organism intracellular transport|GO:1902580,single-organism cellular localization|GO:0006886,intracellular protein transport|GO:0048522,positive regulation of cellular process|GO:0044710,single-organism metabolic process|GO:0044711,single-organism biosynthetic process|GO:0046486,glycerolipid metabolic process|GO:1901564,organonitrogen compound metabolic process|GO:1901566,organonitrogen compound biosynthetic process|GO:0019637,organophosphate metabolic process|GO:0006807,nitrogen compound metabolic process|GO:1901576,organic substance biosynthetic process|GO:0097164,ammonium ion metabolic process|GO:0006629,lipid metabolic process|GO:0008152,metabolic process|GO:0090407,organophosphate biosynthetic process|GO:0008654,phospholipid biosynthetic process|GO:0044249,cellular biosynthetic process|GO:0015833,peptide transport|GO:1904951,positive regulation of establishment of protein localization|GO:0042886,amide transport|GO:0006644,phospholipid metabolic process|GO:0044255,cellular lipid metabolic process|GO:0046474,glycerophospholipid biosynthetic process|GO:0046470,phosphatidylcholine metabolic process|GO:0006656,phosphatidylcholine biosynthetic process|GO:0006650,glycerophospholipid metabolic process|GO:0045017,glycerolipid biosynthetic process|GO:0071705,nitrogen compound transport|GO:0071704,organic substance metabolic process|GO:0009058,biosynthetic process|GO:0044763,single-organism cellular process|GO:0008610,lipid biosynthetic process|GO:0044238,primary metabolic process|GO:0044237,cellular metabolic process|GO:0006796,phosphate-containing compound	GO:0005737,cytoplasm|GO:0043231,intracellular membrane-bounded organelle|GO:0044464,cell part|GO:0043229,intracellular organelle|GO:0005739,mitochondrion|GO:0005622,intracellular|GO:0044444,cytoplasmic part|GO:0005623,cell|GO:0044424,intracellular part|GO:0043227,membrane-bounded organelle|GO:0043226,organelle|GO:0031975,envelope|GO:0031090,organelle membrane|GO:0098805,whole membrane|GO:0016020,membrane|GO:0005740,mitochondrial envelope|GO:0031968,organelle outer membrane|GO:0098588,bounding membrane of organelle|GO:0031967,organelle envelope|GO:0031966,mitochondrial membrane|GO:0019867,outer membrane|GO:0044446,intracellular organelle part|GO:0005741,mitochondrial outer membrane|GO:0044429,mitochondrial part|GO:0044422,organelle part	GO:0008289,lipid binding|GO:0005488,binding
CLLU1		GO:0005737,cytoplasm|GO:0044464,cell part|GO:0005623,cell|GO:0005622,intracellular|GO:0044424,intracellular part	

<table>
<tr><td>HMGA1</td><td>GO:0033044,regulation of chromosome organization|GO:0080090,regulation of primary metabolic process|GO:0019222,regulation of metabolic process|GO:0048584,positive regulation of response to stimulus|GO:0048583,regulation of response to stimulus|GO:1901362,organic cyclic compound biosynthetic process|GO:0071840,cellular component organization or biogenesis|GO:0044710,single-organism metabolic process|GO:0010605,negative regulation of macromolecule metabolic process|GO:0010604,positive regulation of macromolecule metabolic process|GO:0043207,response to external biotic stimulus|GO:0009615,response to virus|GO:0070271,protein complex biogenesis|GO:0040029,regulation of gene expression, epigenetic|GO:0044419,interspecies interaction between organisms|GO:0016458,gene silencing|GO:0006260,DNA replication|GO:0006261,DNA-dependent DNA replication|GO:0048518,positive regulation of biological process|GO:0048519,negative regulation of biological process|GO:0051704,multi-organism process|GO:0006268,DNA unwinding involved in DNA replication|GO:0034728,nucleosome organization|GO:0042127,regulation of cell proliferation|GO:0032392,DNA geometric change|GO:0006284,base-excision repair|GO:0006281,DNA repair|GO:0060255,regulation of macromolecule metabolic process|GO:0006366,transcription from RNA polymerase II promoter|GO:2001141,regulation of RNA biosynthetic process|GO:0009607,response to biotic stimulus|GO:0051707,response to other organism|GO:0046483,heterocycle metabolic process|GO:0031507,heterochromatin assembly|GO:0009605,response to external stimulus|GO:0010639,negative regulation of organelle organization|GO:0033554,cellular response to stress|GO:0019438,aromatic compound biosynthetic process|GO:0016568,chromatin modification|GO:0071103,DNA conformation change|GO:0090342,regulation of cell aging|GO:0090343,positive regulation of cell aging|GO:0009892,negative regulation of metabolic process|GO:0009893,positive regulation of metabolic process|GO:0009890,negative regulation of biosynthetic process|GO:0009891,positive regulation of biosynthetic process|GO:0006333,chromatin assembly or disassembly|GO:0010629,negative regulation of gene expression|GO:0010628,positive regulation of gene expression|GO:0050789,regulation of biological process|GO:0097659,nucleic acid-templated transcription|GO:1901576,organic substance biosynthetic process|GO:0044260,cellular macromolecule metabolic process|GO:0016043,cellular component organization|GO:0065003,macromolecular complex assembly|GO:0065007,biological regulation|GO:1901360,organic cyclic compound metabolic process|GO:1903308,regulation of chromatin modification|GO:1903309,negative regulation of chromatin modification|GO:0018130,heterocycle biosynthetic process|GO:0050793,regulation of developmental process|GO:0009889,regulation of biosynthetic process|GO:0051716,cellular response to stimulus|GO:0050794,regulation of cellular process|GO:0006950,response to stress|GO:0008152,metabolic process|GO:0034654,nucleobase-containing compound biosynthetic process|GO:0090402,oncogene-induced cell senescence|GO:0035986,senescence-associated heterochromatin focus assembly|GO:0016070,RNA metabolic process|GO:0044767,single-organism developmental process|GO:0044271,cellular nitrogen compound biosynthetic process|GO:0080134,regulation of response to stress|GO:0080135,regulation of cellular response to stress|GO:0006355,regulation of transcription, DNA-templated|GO:0010557,positive regulation of macromolecule biosynthetic process|GO:0010556,regulation of macromolecule biosynthetic process|GO:0006351,transcription, DNA-templated|GO:0044763,single-organism cellular process|GO:0010558,negative regulation of macromolecule biosynthetic process|GO:2001251,negative regulation of chromosome organization|GO:0031936,negative regulation of chromatin silencing|GO:0031935,regulation of chromatin silencing|GO:0032774,RNA biosynthetic process|GO:0033043,regulation of organelle organization|GO:0051129,negative regulation of cellular component organization|GO:0051128,regulation of cellular component organization|GO:0044249,cellular biosynthetic process|GO:0034641,cellular nitrogen compound metabolic process|GO:0022607,cellular component assembly|GO:0031497,chromatin assembly|GO:0031498,chromatin disassembly|GO:0043241,protein complex disassembly|GO:0044699,single-organism process|GO:0006139,nucleobase-containing compound metabolic process|GO:0006342,chromatin silencing|GO:0019043,establishment of viral latency|GO:0043933,macromolecular complex subunit organization|GO:1903508,positive regulation of nucleic acid-templated transcription|GO:0008285,negative regulation of cell proliferation|GO:0008283,cell proliferation|GO:0032508,DNA duplex unwinding|GO:0009987,cellular process|GO:0006725,cellular aromatic compound metabolic process|GO:1903506,regulation of nucleic acid-templated transcription|GO:1903507,negative regulation of nucleic acid-templated transcription|GO:0006974,cellular response to DNA damage stimulus|GO:0034645,cellular macromolecule biosynthetic process|GO:0045892,negative regulation of transcription, DNA-templated|GO:0045893,positive regulation of transcription, DNA-templated|GO:0007568,aging|GO:0007569,cell aging|GO:0006338,chromatin remodeling|GO:0051094,positive regulation of developmental process|GO:0006337,nucleosome disassembly|GO:0051253,negative regulation of RNA metabolic process|GO:0051252,regulation of RNA metabolic process|GO:0051254,positive regulation of RNA metabolic process|GO:0009059,macromolecule biosynthetic process|GO:0043170,macromolecule metabolic process|GO:1902680,positive regulation of RNA biosynthetic process|GO:0006807,nitrogen compound metabolic process|GO:0045944,positive regulation of transcription from RNA polymerase II promoter|GO:2000774,positive regulation of cellular senescence|GO:2000772,regulation of cellular senescence|GO:0050896,response to stimulus|GO:1902275,regulation of chromatin organization|GO:0032502,developmental process|GO:0019042,viral latency|GO:0031328,positive regulation of cellular biosynthetic process|GO:0031327,negative regulation of cellular biosynthetic process|GO:0031326,regulation of cellular biosynthetic process|GO:0031325,positive regulation of cellular metabolic process|GO:0031324,negative regulation of cellular metabolic process|GO:0031323,regulation of cellular metabolic process|GO:0090304,nucleic acid metabolic process|GO:0032986,protein-DNA complex disassembly|GO:0032984,macromolecular complex disassembly|GO:0071822,protein complex subunit organization|GO:0006325,chromatin organization|GO:0075713,establishment of integrated proviral latency|GO:0070828,heterochromatin organization|GO:2000112,regulation of cellular macromolecule biosynthetic process|GO:2000113,negative regulation of cellular macromolecule biosynthetic process|GO:0006323,DNA packaging|GO:0071704,organic substance metabolic process|GO:0010467,gene expression|GO:0006357,regulation of transcription from RNA polymerase II promoter|GO:0045814,negative regulation of gene expression, epigenetic|GO:0045815,positive regulation of gene expression, epigenetic|GO:0010468,regulation of gene expression|GO:0071824,protein-DNA complex subunit organization|GO:0045935,positive regulation of nucleobase-containing compound metabolic process|GO:0045934,negative regulation of nucleobase-containing compound metabolic process|GO:0019219,regulation of nucleobase-containing compound metabolic process|GO:0006461,protein complex assembly|GO:1902679,negative regulation of RNA biosynthetic process|GO:0009058,biosynthetic process|GO:0044764,multi-organism cellular process|GO:0022411,cellular component disassembly|GO:0051171,regulation of nitrogen compound metabolic process|GO:0051172,negative regulation of nitrogen compound metabolic process|GO:0051173,positive regulation of nitrogen compound metabolic process|GO:0060969,negative regulation of gene silencing|GO:0060968,regulation of gene silencing|GO:0006996,organelle organization|GO:0044238,primary metabolic process|GO:0051276,chromosome organization|GO:0090398,cellular senescence|GO:0044237,cellular metabolic process|GO:1902589,single-organism organelle organization|GO:0044085,cellular component biogenesis|GO:0016032,viral process|GO:0006259,DNA metabolic process|GO:0044403,symbiosis, encompassing mutualism through parasitism|GO:0048523,negative regulation of cellular process|GO:0048522,positive regulation of cellular process|GO:0051169,nuclear transport|GO:0048869,cellular developmental process|GO:0006810,transport|GO:0051234,establishment of localization|GO:0046907,intracellular</td><td>GO:0031974,membrane-enclosed lumen|GO:0043229,intracellular organelle|GO:0043228,non-membrane-bounded organelle|GO:0000785,chromatin|GO:0005924,cell-substrate adherens junction|GO:0005925,focal adhesion|GO:0043227,membrane-bounded organelle|GO:0043226,organelle|GO:0030054,cell junction|GO:0030055,cell-substrate junction|GO:0005737,cytoplasm|GO:0031981,nuclear lumen|GO:0005634,nucleus|GO:0005654,nucleoplasm|GO:0044798,nuclear transcription factor complex|GO:0005694,chromosome|GO:0070161,anchoring junction|GO:0000792,heterochromatin|GO:0005912,adherens junction|GO:0035985,senescence-associated heterochromatin focus|GO:0043234,protein complex|GO:0032991,macromolecular complex|GO:0043231,intracellular membrane-bounded organelle|GO:0043232,intracellular non-membrane-bounded organelle|GO:0043233,organelle lumen|GO:0005829,cytosol|GO:0044464,cell part|GO:0090575,RNA polymerase II transcription factor complex|GO:0005623,cell|GO:0005622,intracellular|GO:0044446,intracellular organelle part|GO:0070013,intracellular organelle lumen|GO:0044444,cytoplasmic part|GO:0044428,nuclear part|GO:0005667,transcription factor complex|GO:0044424,intracellular part|GO:0044427,chromosomal part|GO:0044422,organelle part|GO:0043657,host cell|GO:0044215,other organism|GO:0018995,host|GO:0044217,other organism part|GO:0044216,other organism cell</td><td>GO:0019899,enzyme binding|GO:0003712,transcription cofactor activity|GO:0044877,macromolecular complex binding|GO:0005488,binding|GO:0003676,nucleic acid binding|GO:0003677,DNA binding|GO:1901363,heterocyclic compound binding|GO:0043565,sequence-specific DNA binding|GO:0097159,organic cyclic compound binding|GO:0016829,lyase activity|GO:0003705,transcription factor activity, RNA polymerase II distal enhancer sequence-specific binding|GO:0003713,transcription coactivator activity|GO:0004520,endodeoxyribonuclease activity|GO:0003700,transcription factor activity, sequence-specific DNA binding|GO:0000989,transcription factor activity, transcription factor binding|GO:0000988,transcription factor activity, protein binding|GO:0000981,RNA polymerase II transcription factor activity, sequence-specific DNA binding|GO:0016787,hydrolase activity|GO:0008134,transcription factor binding|GO:0035257,nuclear hormone receptor binding|GO:0016788,hydrolase activity, acting on ester bonds|GO:0030374,ligand-dependent nuclear receptor transcription coactivator activity|GO:0003906,DNA-(apurinic or apyrimidinic site) lyase activity|GO:0003824,catalytic activity|GO:0046965,retinoid X receptor binding|GO:0042975,peroxisome proliferator activated receptor binding|GO:0051427,hormone receptor binding|GO:0001205,transcriptional activator activity, RNA polymerase II distal enhancer sequence-specific binding|GO:0051575,5'-deoxyribose-5-phosphate lyase activity|GO:0004536,deoxyribonuclease activity|GO:0016835,carbon-oxygen lyase activity|GO:0001228,transcriptional activator activity, RNA polymerase II transcription regulatory region sequence-specific binding|GO:0004518,nuclease activity|GO:0005515,protein binding|GO:0004519,endonuclease activity|GO:0042974,retinoic acid receptor binding|GO:0003680,AT DNA binding|GO:0005102,receptor binding|GO:0003682,chromatin binding|GO:0001071,nucleic acid binding transcription factor activity|GO:1990837,sequence-specific double-stranded DNA binding|GO:0003690,double-stranded DNA binding|GO:0000975,regulatory region DNA binding|GO:0001067,regulatory region nucleic acid binding|GO:0035326,enhancer binding|GO:0001158,enhancer sequence-specific DNA binding|GO:0044212,transcription regulatory region DNA binding|GO:0000976,transcription regulatory region sequence-specific DNA binding</td></tr>
<tr><td>RNF44</td><td>GO:0044248,cellular catabolic process|GO:0006807,nitrogen compound metabolic process|GO:0043632,modification-dependent macromolecule catabolic process|GO:0044267,cellular protein metabolic process|GO:1901575,organic substance catabolic process|GO:0044265,cellular macromolecule catabolic process|GO:0044260,cellular macromolecule metabolic process|GO:0071704,organic substance metabolic process|GO:0043161,proteasome-mediated ubiquitin-dependent protein catabolic process|GO:0070647,protein modification by small protein conjugation or removal|GO:0032446,protein modification by small protein conjugation|GO:0006508,proteolysis|GO:0044238,primary metabolic process|GO:0009987,cellular process|GO:0019941,modification-dependent protein catabolic process|GO:0006464,cellular protein modification process|GO:0043412,macromolecule modification|GO:0036211,protein modification process|GO:0030163,protein catabolic process|GO:0008152,metabolic process|GO:0044257,cellular protein catabolic process|GO:0009056,catabolic process|GO:0009057,macromolecule catabolic process|GO:0051603,proteolysis involved in cellular protein catabolic process|GO:1901564,organonitrogen compound metabolic process|GO:1901565,organonitrogen compound catabolic process|GO:0019538,protein metabolic process|GO:0010498,proteasomal protein catabolic process|GO:0044237,cellular metabolic process|GO:0043170,macromolecule metabolic process|GO:0016567,protein ubiquitination|GO:0006511,ubiquitin-dependent protein catabolic process</td><td></td><td>GO:0043169,cation binding|GO:0046914,transition metal ion binding|GO:0043167,ion binding|GO:0005488,binding|GO:0046872,metal ion binding|GO:0008270,zinc ion binding|GO:0019787,ubiquitin-like protein transferase activity|GO:0061630,ubiquitin protein ligase activity|GO:0003824,catalytic activity|GO:0016740,transferase activity|GO:0004842,ubiquitin-protein transferase activity|GO:0061659,ubiquitin-like protein ligase activity</td></tr>
<tr><td>EPN1</td><td>GO:0048568,embryonic organ development|GO:0048585,negative regulation of response to stimulus|GO:0007167,enzyme linked receptor protein signaling pathway|GO:0048583,regulation of response to stimulus|GO:0042059,negative regulation of epidermal growth factor receptor signaling pathway|GO:0042058,regulation of epidermal growth factor receptor signaling pathway|GO:0023057,negative regulation of signaling|GO:0009792,embryo development ending in birth or egg hatching|GO:0023052,signaling|GO:0010648,negative regulation of cell communication|GO:0007166,cell surface receptor signaling pathway|GO:0023051,regulation of signaling|GO:0007169,transmembrane receptor protein tyrosine kinase signaling pathway|GO:1901184,regulation of ERBB signaling pathway|GO:1901185,negative regulation of ERBB signaling pathway|GO:0050789,regulation of biological process|GO:0044699,single-organism process|GO:0051716,cellular response to stimulus|GO:0007219,Notch signaling pathway|GO:0010646,regulation of cell communication|GO:0009968,negative regulation of signal transduction|GO:0009966,regulation of signal transduction|GO:0007275,multicellular organism development|GO:0048513,animal organ development|GO:0065007,biological regulation|GO:0006810,transport|GO:0048519,negative regulation of biological process|GO:0043009,chordate embryonic development|GO:0044706,multi-multicellular organism process|GO:0032502,developmental process|GO:0032501,multicellular organismal process|GO:0007165,signal transduction|GO:0009987,cellular process|GO:0050794,regulation of cellular process|GO:0044767,single-organism developmental process|GO:0022414,reproductive process|GO:0001701,in utero embryonic development|GO:0007173,epidermal growth factor receptor signaling pathway|GO:0007154,cell communication|GO:0051234,establishment of localization|GO:0051179,localization|GO:0051704,multi-organism process|GO:0044700,single organism signaling|GO:0044703,multi-organism reproductive process|GO:0006897,endocytosis|GO:0016192,vesicle-mediated transport|GO:0044707,single-multicellular organism process|GO:0009790,embryo development|GO:0007565,female pregnancy|GO:0050896,response to stimulus|GO:0048856,anatomical structure development|GO:0044763,single-organism cellular process|GO:0048731,system development|GO:0048523,negative regulation of cellular process|GO:0038127,ERBB signaling pathway|GO:0071840,cellular component organization or biogenesis|GO:0000003,reproduction|GO:0016043,cellular component organization|GO:0061024,membrane organization|GO:2000181,negative regulation of blood vessel morphogenesis|GO:0022603,regulation of anatomical structure morphogenesis|GO:0072359,circulatory system development|GO:0072358,cardiovascular system development|GO:1903670,regulation of sprouting angiogenesis|GO:1903671,negative regulation of sprouting angiogenesis|GO:0009653,anatomical structure morphogenesis|GO:0001525,angiogenesis|GO:0001568,blood vessel development|GO:0051241,negative regulation of multicellular organismal process|GO:0001944,vasculature development|GO:0045765,regulation of angiogenesis|GO:0048514,blood vessel morphogenesis|GO:0002040,sprouting angiogenesis|GO:0048646,anatomical structure formation involved in morphogenesis|GO:0050793,regulation of developmental process|GO:0051239,regulation of multicellular organismal process|GO:0051093,negative regulation of developmental process|GO:1901342,regulation of vasculature development|GO:1901343,negative regulation of vasculature development|GO:2000026,regulation of multicellular organismal development|GO:0016525,negative regulation of angiogenesis</td><td>GO:0030424,axon|GO:0043229,intracellular organelle|GO:0071944,cell periphery|GO:0045202,synapse|GO:0043227,membrane-bounded organelle|GO:0043226,organelle|GO:0005737,cytoplasm|GO:0044306,neuron projection terminus|GO:0043679,axon terminus|GO:0005634,nucleus|GO:0016020,membrane|GO:0097458,neuron part|GO:0044456,synapse part|GO:0043005,neuron projection|GO:0098589,membrane region|GO:0012505,endomembrane system|GO:0005886,plasma membrane|GO:0042995,cell projection|GO:0043195,terminal bouton|GO:0043231,intracellular membrane-bounded organelle|GO:0044463,cell projection part|GO:0044464,cell part|GO:0005623,cell|GO:0005622,intracellular|GO:0033267,axon part|GO:0044424,intracellular part|GO:0044425,membrane part|GO:0098805,whole membrane|GO:0005905,clathrin-coated pit|GO:0005829,cytosol|GO:0044444,cytoplasmic part</td><td>GO:0008134,transcription factor binding|GO:0008289,lipid binding|GO:0005488,binding|GO:0005515,protein binding|GO:0044325,ion channel binding</td></tr>
<tr><td>TFRC</td><td>GO:0008104,protein localization|GO:0060249,anatomical structure homeostasis|GO:0032846,positive regulation of homeostatic process|GO:0032844,regulation of homeostatic process|GO:0055072,iron ion homeostasis|GO:0006826,iron ion transport|GO:0055076,transition metal ion homeostasis|GO:0098771,inorganic ion homeostasis|GO:0010042,response to manganese ion|GO:0055082,cellular chemical homeostasis|GO:0071840,cellular component organization or biogenesis|GO:0045780,positive regulation of bone resorption|GO:0048869,cellular developmental process|GO:0045453,bone resorption|GO:0044419,interspecies interaction between organisms|GO:0048513,animal organ development|GO:0048518,positive regulation of biological process|GO:0033036,macromolecule localization|GO:0019725,cellular homeostasis|GO:0051704,multi-organism process|GO:0048771,tissue remodeling|GO:0045184,establishment of protein localization|GO:0010038,response to metal ion|GO:0010039,response to iron ion|GO:0010035,response to inorganic substance|GO:0010033,response to organic substance|GO:0016192,vesicle-mediated transport|GO:0048871,multicellular organismal homeostasis|GO:0044707,single-multicellular organism process|GO:0031667,response to nutrient levels|GO:0048878,chemical homeostasis|GO:0002376,immune system process|GO:0046849,bone remodeling|GO:0050789,regulation of biological process|GO:0046916,cellular transition metal ion homeostasis|GO:0016043,cellular component organization|GO:0046852,positive regulation of bone remodeling|GO:0046850,regulation of bone remodeling|GO:0065007,biological regulation|GO:0065008,regulation of biological quality|GO:0006812,cation transport|GO:0006811,ion transport|GO:0006810,transport|GO:0051716,cellular response to stimulus|GO:0050794,regulation of cellular process|GO:0006952,defense response|GO:0006953,acute-phase response|GO:0006950,response to stress|GO:0051239,regulation of multicellular organismal process|GO:0030097,hemopoiesis|GO:0002526,acute inflammatory response|GO:0051234,establishment of localization|GO:0002521,leukocyte differentiation|GO:0002520,immune system development|GO:0006897,endocytosis|GO:0036293,response to decreased oxygen levels|GO:0050896,response to stimulus|GO:0006898,receptor-mediated endocytosis|GO:0033572,transferrin transport|GO:0006954,inflammatory response|GO:0050801,ion homeostasis|GO:0030154,cell differentiation|GO:0051128,regulation of cellular component organization|GO:0070887,cellular response to chemical stimulus|GO:0042221,response to chemical|GO:0001666,response to hypoxia|GO:0044699,single-organism process|GO:0051240,positive regulation of multicellular organismal process|GO:0000041,transition metal ion transport|GO:0032502,developmental process|GO:0032501,multicellular organismal process|GO:0006879,cellular iron ion homeostasis|GO:0008283,cell proliferation|GO:0006875,cellular metal ion homeostasis|GO:0030316,osteoclast differentiation|GO:0006873,cellular ion homeostasis|GO:0001558,regulation of cell growth|GO:0030001,metal ion transport|GO:0001101,response to acid chemical|GO:0030003,cellular cation homeostasis|GO:0055080,cation homeostasis|GO:0001894,tissue homeostasis|GO:0055085,transmembrane transport|GO:0007568,aging|GO:0072512,trivalent inorganic cation transport|GO:0042493,response to drug|GO:0015682,ferric iron transport|GO:0030099,myeloid cell differentiation|GO:0048731,system development|GO:0009987,cellular process|GO:0042127,regulation of cell proliferation|GO:0009991,response to extracellular stimulus|GO:1990267,response to transition metal nanoparticle|GO:0042592,homeostatic process|GO:0035690,cellular response to drug|GO:0007275,multicellular organism development|GO:0040007,growth|GO:0033993,response to lipid|GO:0040008,regulation of growth|GO:0002573,myeloid leukocyte differentiation|GO:0046688,response to copper ion|GO:0071702,organic substance transport|GO:0048534,hematopoietic or lymphoid organ development|GO:0009605,response to external stimulus|GO:0097286,iron ion import|GO:0032526,response to retinoic acid|GO:0034103,regulation of tissue remodeling|GO:0034105,positive regulation of tissue remodeling|GO:0007584,response to nutrient|GO:0044767,single-organism developmental process|GO:0044765,single-organism transport|GO:0044764,multi-organism cellular process|GO:0044763,single-organism cellular process|GO:0055065,metal ion homeostasis|GO:0016049,cell growth|GO:0051179,localization|GO:1902578,single-organism localization|GO:0070482,response to oxygen levels|GO:1901700,response to oxygen-containing compound|GO:0009628,response to abiotic stimulus|GO:0048856,anatomical structure development|GO:0045124,regulation of bone resorption|GO:0016032,viral process|GO:0015031,protein transport|GO:0044403,symbiosis, encompassing mutualism through parasitism|GO:0044238,primary metabolic process|GO:0034112,positive regulation of homotypic cell-cell adhesion|GO:0019222,regulation of metabolic process|GO:0034110,regulation of homotypic cell-cell adhesion|GO:0048584,positive regulation of response to stimulus|GO:0048583,regulation of response to stimulus|GO:0002706,regulation of lymphocyte mediated immunity|GO:0002705,positive regulation of leukocyte mediated immunity|GO:0002703,regulation of leukocyte mediated immunity|GO:0002702,positive regulation of production of molecular mediator of immune response|GO:0002700,regulation of production of molecular mediator of immune response|GO:0002381,immunoglobulin production involved in immunoglobulin mediated immune response|GO:0080090,regulation of primary metabolic process|GO:0071705,nitrogen compound transport|GO:0002440,production of molecular mediator of immune response|GO:0010604,positive regulation of macromolecule metabolic process|GO:0045785,positive regulation of cell adhesion|GO:0002208,somatic diversification of immunoglobulins involved in immune response|GO:0045830,positive regulation of isotype switching|GO:0019724,B cell mediated immunity|GO:0051054,positive regulation of DNA metabolic process|GO:0051052,regulation of DNA metabolic process|GO:0060255,regulation of macromolecule metabolic process|GO:0002824,positive regulation of adaptive immune response based on somatic recombination of immune receptors built from immunoglobulin superfamily domains|GO:0002822,regulation of adaptive immune response based on somatic recombination of immune receptors built from immunoglobulin superfamily domains|GO:0042129,regulation of T cell proliferation|GO:0002821,positive regulation of adaptive immune response|GO:0046649,lymphocyte activation|GO:0051701,interaction with host|GO:0002200,somatic diversification of immune receptors|GO:0046483,heterocycle metabolic process|GO:0061024,membrane organization|GO:0000018,regulation of DNA recombination|GO:0002377,immunoglobulin production|GO:0016445,somatic diversification of immunoglobulins|GO:0016444,somatic cell DNA recombination|GO:0016447,somatic recombination of immunoglobulin gene segments|GO:0046718,viral entry into host cell|GO:0009893,positive regulation of metabolic process|GO:0045191,regulation of isotype switching|GO:0050671,positive regulation of lymphocyte proliferation|GO:0032943,mononuclear cell proliferation|GO:0006807,nitrogen compound metabolic process|GO:0032946,positive regulation of mononuclear cell proliferation|GO:0032944,regulation of mononuclear cell proliferation|GO:0046651,lymphocyte proliferation|GO:0006955,immune response|GO:0044260,cellular macromolecule metabolic process|GO:0002285,lymphocyte activation involved in immune response|GO:0042098,T cell proliferation|GO:0002684,positive regulation of immune system process|GO:0002682,regulation of immune system process|GO:0045911,positive regulation of DNA recombination|GO:0002708,positive regulation of lymphocyte mediated immunity|GO:0002204,somatic recombination of immunoglobulin genes involved in immune response|GO:0098602,single organism cell adhesion|GO:0030890,positive regulation of B cell proliferation|GO:0050793,regulation of developmental process|GO:0044710,single-organism metabolic process|GO:0002889,regulation of immunoglobulin mediated immune response|GO:0001775,cell activation|GO:0016064,immunoglobulin mediated immune response|GO:0002449,lymphocyte mediated immunity|GO:0002694,regulation of leukocyte activation|GO:0002697,regulation of immune effector process|GO:0002696,positive regulation of leukocyte activation|GO:0098609,cell-cell adhesion|GO:0015833,peptide transport|GO:0007159,leukocyte cell-cell adhesion|GO:0002699,positive regulation of immune</td><td>GO:0009897,external side of plasma membrane|GO:0055038,recycling endosome membrane|GO:0044421,extracellular region part|GO:0055037,recycling endosome|GO:0098552,side of membrane|GO:0048770,pigment granule|GO:0043229,intracellular organelle|GO:0071944,cell periphery|GO:0005905,clathrin-coated pit|GO:1990712,HFE-transferrin receptor complex|GO:0043227,membrane-bounded organelle|GO:0043226,organelle|GO:0031224,intrinsic component of membrane|GO:0010008,endosome membrane|GO:0005737,cytoplasm|GO:0098797,plasma membrane protein complex|GO:0070062,extracellular exosome|GO:0016023,cytoplasmic, membrane-bounded vesicle|GO:0031410,cytoplasmic vesicle|GO:0016021,integral component of membrane|GO:0016020,membrane|GO:0098802,plasma membrane receptor complex|GO:0005739,mitochondrion|GO:0031988,membrane-bounded vesicle|GO:0005615,extracellular space|GO:0048471,perinuclear region of cytoplasm|GO:0044459,plasma membrane part|GO:0009986,cell surface|GO:0031226,intrinsic component of plasma membrane|GO:0043235,receptor complex|GO:0098588,bounding membrane of organelle|GO:0098589,membrane region|GO:0031982,vesicle|GO:0012505,endomembrane system|GO:0005887,integral component of plasma membrane|GO:0005886,plasma membrane|GO:0032991,macromolecular complex|GO:1903561,extracellular vesicle|GO:0031090,organelle membrane|GO:0043234,protein complex|GO:0042470,melanosome|GO:0043230,extracellular organelle|GO:0043231,intracellular membrane-bounded organelle|GO:0005768,endosome|GO:0044464,cell part|GO:0005623,cell|GO:0005622,intracellular|GO:0044446,intracellular organelle part|GO:0098796,membrane protein complex|GO:0044444,cytoplasmic part|GO:0005576,extracellular region|GO:0044440,endosomal part|GO:0044424,intracellular part|GO:0044425,membrane part|GO:0065010,extracellular membrane-bounded organelle|GO:0098805,whole membrane|GO:0044422,organelle part|GO:0072562,blood microparticle|GO:0098590,plasma membrane region|GO:0030135,coated vesicle|GO:0030136,clathrin-coated vesicle|GO:0030665,clathrin-coated vesicle membrane|GO:0030662,coated vesicle membrane|GO:0044433,cytoplasmic vesicle part|GO:0016323,basolateral plasma membrane|GO:0030659,cytoplasmic vesicle membrane|GO:0097708,intracellular vesicle|GO:0012506,vesicle membrane|GO:0005769,early endosome</td><td>GO:0038024,cargo receptor activity|GO:0031072,heat shock protein binding|GO:0060089,molecular transducer activity|GO:0004872,receptor activity|GO:0003676,nucleic acid binding|GO:1901363,heterocyclic compound binding|GO:0008565,protein transporter activity|GO:0022884,macromolecule transmembrane transporter activity|GO:0046915,transition metal ion transmembrane transporter activity|GO:0030544,Hsp70 protein binding|GO:0051087,chaperone binding|GO:0015399,primary active transmembrane transporter activity|GO:0003725,double-stranded RNA binding|GO:0004998,transferrin receptor activity|GO:0046873,metal ion transmembrane transporter activity|GO:0022804,active transmembrane transporter activity|GO:0042803,protein homodimerization activity|GO:0001948,glycoprotein binding|GO:0015450,P-P-bond-hydrolysis-driven protein transmembrane transporter activity|GO:0042802,identical protein binding|GO:0005215,transporter activity|GO:0044822,poly(A) RNA binding|GO:0008324,cation transmembrane transporter activity|GO:0008320,protein transmembrane transporter activity|GO:0015091,ferric iron transmembrane transporter activity|GO:0022891,substrate-specific transmembrane transporter activity|GO:0022890,inorganic cation transmembrane transporter activity|GO:0022892,substrate-specific transporter activity|GO:0072510,trivalent inorganic cation transmembrane transporter activity|GO:0097367,carbohydrate derivative binding|GO:0097159,organic cyclic compound binding|GO:0015075,ion transmembrane transporter activity|GO:0022857,transmembrane transporter activity|GO:0005381,iron ion transmembrane transporter activity|GO:0033570,transferrin transmembrane transporter activity|GO:0003723,RNA binding|GO:0005515,protein binding|GO:0046983,protein dimerization activity|GO:0015405,P-P-bond-hydrolysis-driven transmembrane transporter activity|GO:0005488,binding|GO:0042887,amide transmembrane transporter activity|GO:0015197,peptide transporter activity|GO:1904680,peptide transmembrane transporter activity|GO:0001618,virus receptor activity</td></tr>
<tr><td>TEAD3</td><td>GO:2000736,regulation of stem cell differentiation|GO:0080090,regulation of primary metabolic process|GO:0019222,regulation of metabolic process|GO:2000738,positive regulation of stem cell differentiation|GO:0048468,cell development|GO:0007165,signal transduction|GO:1901362,organic cyclic compound biosynthetic process|GO:1901360,organic cyclic compound metabolic process|GO:0051716,cellular response to stimulus|GO:0048863,stem cell differentiation|GO:0048864,stem cell development|GO:0010604,positive regulation of macromolecule metabolic process|GO:0048869,cellular developmental process|GO:0010720,positive regulation of cell development|GO:2000036,regulation of stem cell population maintenance|GO:0048518,positive regulation of biological process|GO:0048519,negative regulation of biological process|GO:0051704,multi-organism process|GO:0044700,single organism signaling|GO:0060255,regulation of macromolecule metabolic process|GO:2001141,regulation of RNA biosynthetic process|GO:0046483,heterocycle metabolic process|GO:0019827,stem cell population maintenance|GO:0044703,multi-organism reproductive process|GO:0044707,single-multicellular organism process|GO:0044706,multi-multicellular organism process|GO:0019438,aromatic compound biosynthetic process|GO:0009893,positive regulation of metabolic process|GO:0009891,positive regulation of biosynthetic process|GO:0008152,metabolic process|GO:0010628,positive regulation of gene expression|GO:0035556,intracellular signal transduction|GO:0050789,regulation of biological process|GO:0097659,nucleic acid-templated transcription|GO:1901576,organic substance biosynthetic process|GO:0044260,cellular macromolecule metabolic process|GO:0065007,biological regulation|GO:0006367,transcription initiation from RNA polymerase II promoter|GO:0006366,transcription from RNA polymerase II promoter|GO:0018130,heterocycle biosynthetic process|GO:0050793,regulation of developmental process|GO:0009889,regulation of biosynthetic process|GO:0050794,regulation of cellular process|GO:0098727,maintenance of cell number|GO:0051239,regulation of multicellular organismal process|GO:0034654,nucleobase-containing compound biosynthetic process|GO:0016070,RNA metabolic process|GO:0044271,cellular nitrogen compound biosynthetic process|GO:0050896,response to stimulus|GO:0006355,regulation of transcription, DNA-templated|GO:0010557,positive regulation of macromolecule biosynthetic process|GO:0010556,regulation of macromolecule biosynthetic process|GO:0006351,transcription, DNA-templated|GO:0006352,DNA-templated transcription, initiation|GO:0032774,RNA biosynthetic process|GO:0030154,cell differentiation|GO:0044249,cellular biosynthetic process|GO:0034641,cellular nitrogen compound metabolic process|GO:0023052,signaling|GO:0034645,cellular macromolecule biosynthetic process|GO:0044699,single-organism process|GO:0006139,nucleobase-containing compound metabolic process|GO:0051240,positive regulation of multicellular organismal process|GO:0060284,regulation of cell development|GO:0032502,developmental process|GO:0032501,multicellular organismal process|GO:0009987,cellular process|GO:0006725,cellular aromatic compound metabolic process|GO:1903506,regulation of nucleic acid-templated transcription|GO:0045597,positive regulation of cell differentiation|GO:0045596,negative regulation of cell differentiation|GO:0045595,regulation of cell differentiation|GO:0045893,positive regulation of transcription, DNA-templated|GO:0035329,hippo signaling|GO:1902459,positive regulation of stem cell population maintenance|GO:0051093,negative regulation of developmental process|GO:0051094,positive regulation of developmental process|GO:0007565,female pregnancy|GO:0051252,regulation of RNA metabolic process|GO:0051254,positive regulation of RNA metabolic process|GO:0043170,macromolecule metabolic process|GO:1902680,positive regulation of RNA biosynthetic process|GO:0006807,nitrogen compound metabolic process|GO:0045944,positive regulation of transcription from RNA polymerase II promoter|GO:1903508,positive regulation of nucleic acid-templated transcription|GO:0031328,positive regulation of cellular biosynthetic process|GO:0031326,regulation of cellular biosynthetic process|GO:0031325,positive regulation of cellular metabolic process|GO:0031323,regulation of cellular metabolic process|GO:0090304,nucleic acid metabolic process|GO:2000112,regulation of cellular macromolecule biosynthetic process|GO:0071704,organic substance metabolic process|GO:0010467,gene expression|GO:0006357,regulation of transcription from RNA polymerase II promoter|GO:0010468,regulation of gene expression|GO:0045935,positive regulation of nucleobase-containing compound metabolic process|GO:0019219,regulation of nucleobase-containing compound metabolic process|GO:0044767,single-organism developmental process|GO:0022414,reproductive process|GO:0009058,biosynthetic process|GO:0009059,macromolecule biosynthetic process|GO:0044763,single-organism cellular process|GO:0051171,regulation of nitrogen compound metabolic process|GO:0051173,positive regulation of nitrogen compound metabolic process|GO:0007154,cell communication|GO:0044238,primary metabolic process|GO:0048856,anatomical structure development|GO:0044237,cellular metabolic process|GO:0048523,negative regulation of cellular process|GO:0048522,positive regulation of cellular process|GO:0048103,somatic stem cell division|GO:0048513,animal organ development|GO:0055059,asymmetric neuroblast division|GO:0048568,embryonic organ development|GO:0055057,neuroblast division|GO:0098722,asymmetric stem cell division|GO:0072089,stem cell proliferation|GO:0000003,reproduction|GO:0045165,cell fate commitment|GO:0036445,neuronal stem cell division|GO:0009790,embryo development|GO:0008283,cell proliferation|GO:0007405,neuroblast proliferation|GO:0048731,system development|GO:0017145,stem cell division|GO:0008356,asymmetric cell division|GO:0007275,multicellular organism development|GO:0051301,cell division|GO:0061351,neural precursor cell proliferation|GO:0022008,neurogenesis|GO:0048699,generation of</td><td>GO:0043234,protein complex|GO:0044446,intracellular organelle part|GO:0032991,macromolecular complex|GO:0043231,intracellular membrane-bounded organelle|GO:0031981,nuclear lumen|GO:0043233,organelle lumen|GO:0005634,nucleus|GO:0044464,cell part|GO:0031974,membrane-enclosed lumen|GO:0005622,intracellular|GO:0005654,nucleoplasm|GO:0070013,intracellular organelle lumen|GO:0043229,intracellular organelle|GO:0044428,nuclear part|GO:0005623,cell|GO:0005667,transcription factor complex|GO:0044424,intracellular part|GO:0043227,membrane-bounded organelle|GO:0043226,organelle|GO:0044422,organelle part</td><td>GO:0001085,RNA polymerase II transcription factor binding|GO:0003676,nucleic acid binding|GO:0003700,transcription factor activity, sequence-specific DNA binding|GO:0097159,organic cyclic compound binding|GO:0008134,transcription factor binding|GO:0005488,binding|GO:0005515,protein binding|GO:0003677,DNA binding|GO:1901363,heterocyclic compound binding|GO:0001071,nucleic acid binding transcription factor activity|GO:0044212,transcription regulatory region DNA binding|GO:0043565,sequence-specific DNA binding|GO:0001067,regulatory region nucleic acid binding|GO:0000975,regulatory region DNA binding|GO:0000981,RNA polymerase II transcription factor activity, sequence-specific DNA binding</td></tr>
<tr><td>BBC3</td><td>GO:0016265,obsolete death|GO:0009987,cellular process|GO:0006915,apoptotic process|GO:0012501,programmed cell death|GO:0044763,single-organism cellular process|GO:0008219,cell death|GO:0044699,single-organism process|GO:2001235,positive regulation of apoptotic signaling pathway|GO:0010822,positive regulation of mitochondrion organization|GO:2001233,regulation of apoptotic signaling pathway|GO:0048584,positive regulation of response to stimulus|GO:0097190,apoptotic signaling pathway|GO:0090200,positive regulation of release of cytochrome c from mitochondria|GO:0097193,intrinsic apoptotic signaling pathway|GO:0023056,positive regulation of signaling|GO:0023052,signaling|GO:0007165,signal transduction|GO:0023051,regulation of signaling|GO:0010942,positive regulation of cell death|GO:0010647,positive regulation of cell communication|GO:0010646,regulation of cell communication|GO:0042981,regulation of apoptotic process|GO:0035556,intracellular signal transduction|GO:0007005,mitochondrion organization|GO:0051716,cellular response to stimulus|GO:0010941,regulation of cell death|GO:0009966,regulation of signal transduction|GO:0009967,positive regulation of signal transduction|GO:0033043,regulation of organelle organization|GO:0016043,cellular component organization|GO:0050789,regulation of biological process|GO:0043065,positive regulation of apoptotic process|GO:0043067,regulation of programmed cell death|GO:0065007,biological regulation|GO:0071840,cellular component organization or biogenesis|GO:0048518,positive regulation of biological process|GO:0043068,positive regulation of programmed cell death|GO:0051130,positive regulation of cellular component organization|GO:0090199,regulation of release of cytochrome c from mitochondria|GO:0048583,regulation of response to stimulus|GO:0050794,regulation of cellular process|GO:0007154,cell communication|GO:0051128,regulation of cellular component organization|GO:0006996,organelle organization|GO:0044700,single organism signaling|GO:0008637,apoptotic mitochondrial changes|GO:0050896,response to stimulus|GO:0010638,positive regulation of organelle organization|GO:0001836,release of cytochrome c from mitochondria|GO:0010821,regulation of mitochondrion organization|GO:0048522,positive regulation of cellular process</td><td>GO:0005737,cytoplasm|GO:0043231,intracellular membrane-bounded organelle|GO:0044464,cell part|GO:0043229,intracellular organelle|GO:0005739,mitochondrion|GO:0005622,intracellular|GO:0044444,cytoplasmic part|GO:0005623,cell|GO:0044424,intracellular part|GO:0043227,membrane-bounded organelle|GO:0043226,organelle|GO:0000323,lytic vacuole|GO:0005773,vacuole|GO:0005764,lysosome</td><td>GO:0001948,glycoprotein binding|GO:0097367,carbohydrate derivative binding|GO:0051117,ATPase binding|GO:0019899,enzyme binding|GO:0005488,binding|GO:0005515,protein binding</td></tr>
<tr><td>KCTD15</td><td>GO:0051259,protein oligomerization|GO:0022607,cellular component assembly|GO:0071822,protein complex subunit organization|GO:0070271,protein complex biogenesis|GO:0043933,macromolecular complex subunit organization|GO:0006461,protein complex assembly|GO:0051260,protein homooligomerization|GO:0016043,cellular component organization|GO:0065003,macromolecular complex assembly|GO:0044085,cellular component biogenesis|GO:0009987,cellular process|GO:0071840,cellular component organization or biogenesis</td><td></td><td></td></tr>
<tr><td>ISCA2</td><td>GO:0022607,cellular component assembly|GO:0009987,cellular process|GO:0009058,biosynthetic process|GO:0016043,cellular component organization|GO:0016226,iron-sulfur cluster assembly|GO:0031163,metallo-sulfur cluster assembly|GO:0044085,cellular component biogenesis|GO:0008152,metabolic process|GO:0071840,cellular component organization or biogenesis|GO:0044249,cellular biosynthetic process|GO:0006807,nitrogen compound metabolic process|GO:0044281,small molecule metabolic process|GO:0044699,single-organism process|GO:0044710,single-organism metabolic process|GO:0051186,cofactor metabolic process|GO:0051188,cofactor biosynthetic process|GO:0071704,organic substance metabolic process|GO:0010467,gene expression|GO:0097428,protein maturation by iron-sulfur cluster transfer|GO:0044238,primary metabolic process|GO:1901564,organonitrogen compound metabolic process|GO:0051604,protein maturation|GO:0019538,protein metabolic process|GO:0044237,cellular metabolic process|GO:0043170,macromolecule metabolic process|GO:0006790,sulfur compound metabolic process</td><td>GO:0005737,cytoplasm|GO:0043231,intracellular membrane-bounded organelle|GO:0044464,cell part|GO:0043229,intracellular organelle|GO:0005739,mitochondrion|GO:0005622,intracellular|GO:0044444,cytoplasmic part|GO:0005623,cell|GO:0044424,intracellular part|GO:0043227,membrane-bounded organelle|GO:0043226,organelle|GO:0043233,organelle lumen|GO:0031974,membrane-enclosed lumen|GO:0044446,intracellular organelle part|GO:0070013,intracellular organelle lumen|GO:0044429,mitochondrial part|GO:0005759,mitochondrial matrix|GO:0044422,organelle part</td><td>GO:0043169,cation binding|GO:0051540,metal cluster binding|GO:0051536,iron-sulfur cluster binding|GO:0043167,ion binding|GO:0005488,binding|GO:0005515,protein binding|GO:0046872,metal ion binding|GO:0005198,structural molecule activity|GO:0046914,transition metal ion binding|GO:0051537,2 iron, 2 sulfur cluster binding|GO:0005506,iron ion binding|GO:0051539,4 iron, 4 sulfur cluster binding</td></tr>
<tr><td>RIMS4</td><td>GO:0042391,regulation of membrane potential|GO:0051234,establishment of localization|GO:0051648,vesicle localization|GO:0060341,regulation of cellular localization|GO:0051049,regulation of transport|GO:0006836,neurotransmitter transport|GO:0097480,establishment of synaptic vesicle localization|GO:0050804,modulation of synaptic transmission|GO:0032940,secretion by cell|GO:0023052,signaling|GO:1903530,regulation of secretion by cell|GO:0023051,regulation of signaling|GO:0051588,regulation of neurotransmitter transport|GO:0010646,regulation of cell communication|GO:0050789,regulation of biological process|GO:0044699,single-organism process|GO:0048489,synaptic vesicle transport|GO:0051656,establishment of organelle localization|GO:0051046,regulation of secretion|GO:0051649,establishment of localization in cell|GO:0006887,exocytosis|GO:0051650,establishment of vesicle localization|GO:0065007,biological regulation|GO:1902578,single-organism localization|GO:0097479,synaptic vesicle localization|GO:0065008,regulation of biological quality|GO:0001505,regulation of neurotransmitter levels|GO:0017157,regulation of exocytosis|GO:0006810,transport|GO:0009987,cellular process|GO:0023061,signal release|GO:0060627,regulation of vesicle-mediated transport|GO:0044765,single-organism transport|GO:0046928,regulation of neurotransmitter secretion|GO:0044763,single-organism cellular process|GO:0007269,neurotransmitter secretion|GO:0007268,chemical synaptic transmission|GO:0007267,cell-cell signaling|GO:0007154,cell communication|GO:0032879,regulation of localization|GO:0051179,localization|GO:0050794,regulation of cellular process|GO:0051641,cellular localization|GO:0044700,single organism signaling|GO:0046903,secretion|GO:0016192,vesicle-mediated transport|GO:0016079,synaptic vesicle exocytosis|GO:1902803,regulation of synaptic vesicle transport|GO:0051640,organelle localization|GO:1902580,single-organism cellular localization|GO:2000300,regulation of synaptic vesicle exocytosis|GO:0099643,signal release from synapse|GO:0032386,regulation of intracellular transport|GO:0045055,regulated exocytosis|GO:0017158,regulation of calcium ion-dependent exocytosis|GO:1903305,regulation of regulated secretory pathway|GO:0017156,calcium ion regulated exocytosis|GO:0099504,synaptic vesicle cycle|GO:1903649,regulation of cytoplasmic transport|GO:0016482,cytosolic transport|GO:0046907,intracellular transport|GO:0048791,calcium ion-regulated exocytosis of neurotransmitter|GO:1902582,single-organism intracellular transport|GO:0098916,anterograde trans-synaptic signaling|GO:0099531,presynaptic process involved in chemical synaptic transmission|GO:0099536,synaptic signaling|GO:0099537,trans-synaptic signaling</td><td>GO:0097060,synaptic membrane|GO:0098590,plasma membrane region|GO:0098589,membrane region|GO:0044464,cell part|GO:0098805,whole membrane|GO:0048786,presynaptic active zone|GO:0071944,cell periphery|GO:0005623,cell|GO:0097458,neuron part|GO:0044456,synapse part|GO:0098793,presynapse|GO:0045202,synapse|GO:0005886,plasma membrane|GO:0044425,membrane part|GO:0016020,membrane|GO:0044459,plasma membrane part|GO:0030054,cell junction</td><td>GO:0044325,ion channel binding|GO:0005488,binding|GO:0005515,protein binding</td></tr>
<tr><td>NUMBL</td><td>GO:0019221,cytokine-mediated signaling pathway|GO:0048468,cell development|GO:0048103,somatic stem cell division|GO:0060322,head development|GO:0007165,signal transduction|GO:0007166,cell surface receptor signaling pathway|GO:0071840,cellular component organization or biogenesis|GO:0051716,cellular response to stimulus|GO:0021591,ventricular system development|GO:0007409,axonogenesis|GO:0048869,cellular developmental process|GO:0048513,animal organ development|GO:0010720,positive regulation of cell development|GO:0048518,positive regulation of biological process|GO:0010033,response to organic substance|GO:0044700,single organism signaling|GO:0044707,single-multicellular organism process|GO:0019538,protein metabolic process|GO:0021846,cell proliferation in forebrain|GO:0021849,neuroblast division in subventricular zone|GO:0021537,telencephalon development|GO:0055057,neuroblast division|GO:0031175,neuron projection development|GO:0050789,regulation of biological process|GO:0000904,cell morphogenesis involved in differentiation|GO:0000902,cell morphogenesis|GO:0016043,cellular component organization|GO:0065007,biological regulation|GO:0061564,axon development|GO:0034097,response to cytokine|GO:0050793,regulation of developmental process|GO:0050794,regulation of cellular process|GO:0051239,regulation of multicellular organismal process|GO:0072089,stem cell proliferation|GO:0007420,brain development|GO:0071345,cellular response to cytokine stimulus|GO:0050896,response to stimulus|GO:0051962,positive regulation of nervous system development|GO:0048812,neuron projection morphogenesis|GO:0008152,metabolic process|GO:0030154,cell differentiation|GO:0036445,neuronal stem cell division|GO:0023052,signaling|GO:0070887,cellular response to chemical stimulus|GO:0042221,response to chemical|GO:0009653,anatomical structure morphogenesis|GO:0044699,single-organism process|GO:0007417,central nervous system development|GO:0050767,regulation of neurogenesis|GO:0051240,positive regulation of multicellular organismal process|GO:0060284,regulation of cell development|GO:0050769,positive regulation of neurogenesis|GO:0032502,developmental process|GO:0032501,multicellular organismal process|GO:0008283,cell proliferation|GO:0009987,cellular process|GO:0045597,positive regulation of cell differentiation|GO:0045595,regulation of cell differentiation|GO:0045216,cell-cell junction organization|GO:0007405,neuroblast proliferation|GO:0021670,lateral ventricle development|GO:0048858,cell projection morphogenesis|GO:0051094,positive regulation of developmental process|GO:0043170,macromolecule metabolic process|GO:0048731,system development|GO:0017145,stem cell division|GO:0030030,cell projection organization|GO:0034332,adherens junction organization|GO:0034330,cell junction organization|GO:0007275,multicellular organism development|GO:0051301,cell division|GO:0032989,cellular component morphogenesis|GO:0071704,organic substance metabolic process|GO:0071310,cellular response to organic substance|GO:0051960,regulation of nervous system development|GO:0061351,neural precursor cell proliferation|GO:2000026,regulation of multicellular organismal development|GO:0021872,forebrain generation of neurons|GO:0021873,forebrain neuroblast division|GO:0048666,neuron development|GO:0048667,cell morphogenesis involved in neuron differentiation|GO:0030182,neuron differentiation|GO:0044767,single-organism developmental process|GO:0044763,single-organism cellular process|GO:0007154,cell communication|GO:0022008,neurogenesis|GO:0044238,primary metabolic process|GO:0048699,generation of neurons|GO:0032990,cell part morphogenesis|GO:0007399,nervous system development|GO:0048856,anatomical structure development|GO:0030900,forebrain development|GO:0048522,positive regulation of cellular process|GO:1901564,organonitrogen compound metabolic process|GO:0006807,nitrogen compound metabolic process</td><td>GO:0005737,cytoplasm|GO:0044464,cell part|GO:0005623,cell|GO:0005622,intracellular|GO:0044424,intracellular part</td><td>GO:0005488,binding|GO:0005515,protein binding</td></tr>
<tr><td>FAM229B</td><td></td><td></td><td></td></tr>
</table>

EREG	GO:0042035,regulation of cytokine biosynthetic process|GO:0051716,cellular response to stimulus|GO:0048589,developmental growth|GO:0000165,MAPK cascade|GO:0045137,development of primary sexual characteristics|GO:0030855,epithelial cell differentiation|GO:0007088,regulation of mitotic nuclear division|GO:0048468,cell development|GO:0045859,regulation of protein kinase activity|GO:0007281,germ cell development|GO:0050678,regulation of epithelial cell proliferation|GO:0046483,heterocycle metabolic process|GO:0042325,regulation of phosphorylation|GO:0042327,positive regulation of phosphorylation|GO:0042089,cytokine biosynthetic process|GO:0019538,protein metabolic process|GO:0010638,positive regulation of organelle organization|GO:0006275,regulation of DNA replication|GO:0051783,regulation of nuclear division|GO:0042698,ovulation cycle|GO:0051781,positive regulation of cell division|GO:0051785,positive regulation of nuclear division|GO:0042692,muscle cell differentiation|GO:0009892,negative regulation of metabolic process|GO:0009893,positive regulation of metabolic process|GO:0009890,negative regulation of biosynthetic process|GO:0009891,positive regulation of biosynthetic process|GO:0071902,positive regulation of protein serine/threonine kinase activity|GO:0035556,intracellular signal transduction|GO:0071900,regulation of protein serine/threonine kinase activity|GO:0050789,regulation of biological process|GO:0051347,positive regulation of transferase activity|GO:0045408,regulation of interleukin-6 biosynthetic process|GO:0002684,positive regulation of immune system process|GO:0002682,regulation of immune system process|GO:1901360,organic cyclic compound metabolic process|GO:0018130,heterocycle biosynthetic process|GO:2000273,positive regulation of receptor activity|GO:0043410,positive regulation of MAPK cascade|GO:0043412,macromolecule modification|GO:0000003,reproduction|GO:0016070,RNA metabolic process|GO:0071345,cellular response to cytokine stimulus|GO:0010557,positive regulation of macromolecule biosynthetic process|GO:0010556,regulation of macromolecule biosynthetic process|GO:0048869,cellular developmental process|GO:0010558,negative regulation of macromolecule biosynthetic process|GO:0051246,regulation of protein metabolic process|GO:0048146,positive regulation of fibroblast proliferation|GO:0048145,regulation of fibroblast proliferation|GO:0048144,fibroblast proliferation|GO:0051128,regulation of cellular component organization|GO:0038127,ERBB signaling pathway|GO:0007126,meiotic nuclear division|GO:0000280,nuclear division|GO:0045742,positive regulation of epidermal growth factor receptor signaling pathway|GO:0045740,positive regulation of DNA replication|GO:0045741,positive regulation of epidermal growth factor-activated receptor activity|GO:0032675,regulation of interleukin-6 production|GO:0008284,positive regulation of cell proliferation|GO:0008285,negative regulation of cell proliferation|GO:0008283,cell proliferation|GO:0051150,regulation of smooth muscle cell differentiation|GO:0051151,negative regulation of smooth muscle cell differentiation|GO:0001550,ovarian cumulus expansion|GO:0001556,oocyte maturation|GO:0018108,peptidyl-tyrosine phosphorylation|GO:0022402,cell cycle process|GO:0007275,multicellular organism development|GO:0051301,cell division|GO:2000112,regulation of cellular macromolecule biosynthetic process|GO:2000113,negative regulation of cellular macromolecule biosynthetic process|GO:0051302,regulation of cell division|GO:0007276,gamete generation|GO:0048599,oocyte development|GO:0006468,protein phosphorylation|GO:0045089,positive regulation of innate immune response|GO:0000278,mitotic cell cycle|GO:0019219,regulation of nucleobase-containing compound metabolic process|GO:0045087,innate immune response|GO:0006464,cellular protein modification process|GO:0044767,single-organism developmental process|GO:0044763,single-organism cellular process|GO:1901700,response to oxygen-containing compound|GO:0003006,developmental process involved in reproduction|GO:0009913,epidermal cell differentiation|GO:0048856,anatomical structure development|GO:0018212,peptidyl-tyrosine modification|GO:0006796,phosphate-containing compound metabolic process|GO:0006793,phosphorus metabolic process|GO:0048523,negative regulation of cellular process|GO:0048522,positive regulation of cellular process|GO:0032147,activation of protein kinase activity|GO:0031349,positive regulation of defense response|GO:0009299,mRNA transcription|GO:0007165,signal transduction|GO:0007166,cell surface receptor signaling pathway|GO:0007167,enzyme linked receptor protein signaling pathway|GO:0007169,transmembrane receptor protein tyrosine kinase signaling pathway|GO:0031347,regulation of defense response|GO:0044710,single-organism metabolic process|GO:0045787,positive regulation of cell cycle|GO:0070848,response to growth factor|GO:0044093,positive regulation of molecular function|GO:0051054,positive regulation of DNA metabolic process|GO:0051052,regulation of DNA metabolic process|GO:0030216,keratinocyte differentiation|GO:2001141,regulation of RNA biosynthetic process|GO:0010033,response to organic substance|GO:0051704,multi-organism process|GO:0045840,positive regulation of mitotic nuclear division|GO:0010629,negative regulation of gene expression|GO:0006807,nitrogen compound metabolic process|GO:0045088,regulation of innate immune response|GO:0044267,cellular protein metabolic process|GO:0009653,anatomical structure morphogenesis|GO:0002764,immune response-regulating signaling pathway|GO:0044260,cellular macromolecule metabolic process|GO:0002768,immune response-regulating cell surface receptor signaling pathway|GO:0001568,blood vessel development|GO:0044344,cellular response to fibroblast growth factor stimulus|GO:0007049,cell cycle|GO:0061097,regulation of protein tyrosine kinase activity|GO:0061098,positive regulation of protein tyrosine kinase activity|GO:0007186,G-protein coupled receptor signaling pathway|GO:0009887,animal organ morphogenesis|GO:0050793,regulation of developmental process|GO:0050790,regulation of catalytic activity|GO:0009889,regulation of biosynthetic process|GO:0009888,tissue development|GO:0050794,regulation of cellular process|GO:0051239,regulation of multicellular organismal process|GO:0046660,female sex differentiation|GO:1902679,negative regulation of RNA biosynthetic process|GO:0007548,sex differentiation|GO:0050896,response to stimulus|GO:0051338,regulation of transferase activity|GO:0010562,positive regulation of phosphorus metabolic process|GO:0033043,regulation of organelle organization|GO:0001944,vasculature development|GO:0019953,sexual reproduction|GO:0043406,positive regulation of MAP kinase activity|GO:0043405,regulation of MAP kinase activity|GO:0070887,cellular response to chemical stimulus|GO:0044699,single-organism process|GO:0043408,regulation of MAPK cascade|GO:0051240,positive regulation of multicellular organismal process|GO:0010564,regulation of cell cycle process|GO:0051247,positive regulation of protein metabolic process|GO:0031399,regulation of protein modification process|GO:0048608,reproductive structure development|GO:0048609,multicellular organismal reproductive process|GO:0048731,system development|GO:0061458,reproductive system development|GO:0051145,smooth muscle cell differentiation|GO:0051147,regulation of muscle cell differentiation|GO:0051148,negative regulation of muscle cell differentiation|GO:0001525,angiogenesis|GO:0043588,skin development|GO:0045931,positive regulation of mitotic cell cycle|GO:0045935,positive regulation of nucleobase-containing compound metabolic process|GO:0045934,negative regulation of nucleobase-containing compound metabolic process|GO:0045937,positive regulation of phosphate metabolic process|GO:0022414,reproductive process|GO:0022412,cellular process involved in reproduction in multicellular organism|GO:0007267,cell-cell signaling|GO:0042221,response to chemical|GO:0042226,interleukin-6 biosynthetic process|GO:0006996,organelle organization|GO:0044238,primary metabolic process|GO:0044237,cellular metabolic process|GO:0032755,positive regulation of interleukin-6 production|GO:0006259,DNA metabolic process|GO:0019220,regulation of phosphate metabolic process|GO:0019221,cytokine-mediated signaling pathway|GO:0019222,regulation of metabolic process|GO:0048584,positive regulation of response to stimulus|GO:0048583,regulation of response to stimulus|GO:0048469,cell maturation|GO:0072359,circulatory system development|GO:0072358,cardiovascular system development|GO:1901362,organic cyclic compound biosynthetic process|GO:0071840,cellular component organization or biogenesis|GO:0009966,regulation of signal	GO:0005615,extracellular space|GO:0031226,intrinsic component of plasma membrane|GO:0016021,integral component of membrane|GO:0016020,membrane|GO:0044464,cell part|GO:0005623,cell|GO:0005622,intracellular|GO:0071944,cell periphery|GO:0005887,integral component of plasma membrane|GO:0005886,plasma membrane|GO:0044425,membrane part|GO:0044459,plasma membrane part|GO:0044421,extracellular region part|GO:0031224,intrinsic component of membrane|GO:0005576,extracellular region	GO:0008083,growth factor activity|GO:0070851,growth factor receptor binding|GO:0005154,epidermal growth factor receptor binding|GO:0005488,binding|GO:0005515,protein binding|GO:0005102,receptor binding|GO:0046934,phosphatidylinositol-4,5-bisphosphate 3-kinase activity|GO:0005088,Ras guanyl-nucleotide exchange factor activity|GO:0003824,catalytic activity|GO:0016773,phosphotransferase activity, alcohol group as acceptor|GO:0016772,transferase activity, transferring phosphorus-containing groups|GO:0016301,kinase activity|GO:0005085,guanyl-nucleotide exchange factor activity|GO:0052813,phosphatidylinositol bisphosphate kinase activity|GO:0016740,transferase activity|GO:0004713,protein tyrosine kinase activity|GO:0035004,phosphatidylinositol 3-kinase activity|GO:0004672,protein kinase activity|GO:0098772,molecular function regulator
LPAR4	GO:0044700,single organism signaling|GO:0051716,cellular response to stimulus|GO:0050896,response to stimulus|GO:0009987,cellular process|GO:0044763,single-organism cellular process|GO:0050794,regulation of cellular process|GO:0065007,biological regulation|GO:0023052,signaling|GO:0007165,signal transduction|GO:0007154,cell communication|GO:0007186,G-protein coupled receptor signaling pathway|GO:0050789,regulation of biological process|GO:0044699,single-organism process|GO:0019725,cellular homeostasis|GO:0010647,positive regulation of cell communication|GO:0035023,regulation of Rho protein signal transduction|GO:0046578,regulation of Ras protein signal transduction|GO:0046579,positive regulation of Ras protein signal transduction|GO:0023056,positive regulation of signaling|GO:0035025,positive regulation of Rho protein signal transduction|GO:0023051,regulation of signaling|GO:0098771,inorganic ion homeostasis|GO:0035556,intracellular signal transduction|GO:0010646,regulation of cell communication|GO:0007265,Ras protein signal transduction|GO:0072507,divalent inorganic cation homeostasis|GO:1902531,regulation of intracellular signal transduction|GO:0072503,cellular divalent inorganic cation homeostasis|GO:0009966,regulation of signal transduction|GO:0009967,positive regulation of signal transduction|GO:0055074,calcium ion homeostasis|GO:0042592,homeostatic process|GO:0006874,cellular calcium ion homeostasis|GO:0048518,positive regulation of biological process|GO:0065008,regulation of biological quality|GO:0050801,ion homeostasis|GO:0051057,positive regulation of small GTPase mediated signal transduction|GO:0051056,regulation of small GTPase mediated signal transduction|GO:0048584,positive regulation of response to stimulus|GO:0006875,cellular metal ion homeostasis|GO:0048583,regulation of response to stimulus|GO:0006873,cellular ion homeostasis|GO:0030003,cellular cation homeostasis|GO:0055065,metal ion homeostasis|GO:0055080,cation homeostasis|GO:0007266,Rho protein signal transduction|GO:1902533,positive regulation of intracellular signal transduction|GO:0007264,small GTPase mediated signal transduction|GO:0051480,regulation of cytosolic calcium ion concentration|GO:0007200,phospholipase C-activating G-protein coupled receptor signaling pathway|GO:0051482,positive regulation of cytosolic calcium ion concentration involved in phospholipase C-activating G-protein coupled signaling pathway|GO:0007204,positive regulation of cytosolic calcium ion concentration|GO:0048878,chemical homeostasis|GO:0055082,cellular chemical homeostasis|GO:0048522,positive regulation of cellular process	GO:0031226,intrinsic component of plasma membrane|GO:0016021,integral component of membrane|GO:0016020,membrane|GO:0044464,cell part|GO:0005623,cell|GO:0071944,cell periphery|GO:0005887,integral component of plasma membrane|GO:0005886,plasma membrane|GO:0044425,membrane part|GO:0044459,plasma membrane part|GO:0031224,intrinsic component of membrane|GO:0044446,intracellular organelle part|GO:0016604,nuclear body|GO:0043231,intracellular membrane-bounded organelle|GO:0031981,nuclear lumen|GO:0043233,organelle lumen|GO:0005634,nucleus|GO:0031974,membrane-enclosed lumen|GO:0005622,intracellular|GO:0005654,nucleoplasm|GO:0070013,intracellular organelle lumen|GO:0043229,intracellular organelle|GO:0044451,nucleoplasm part|GO:0044424,intracellular part|GO:0044428,nuclear part|GO:0043227,membrane-bounded organelle|GO:0043226,organelle|GO:0044422,organelle part	GO:0004930,G-protein coupled receptor activity|GO:0038023,signaling receptor activity|GO:0008289,lipid binding|GO:0060089,molecular transducer activity|GO:0004888,transmembrane signaling receptor activity|GO:0004872,receptor activity|GO:0004871,signal transducer activity|GO:0005488,binding|GO:0043168,anion binding|GO:0097367,carbohydrate derivative binding|GO:0005543,phospholipid binding|GO:0070915,lysophosphatidic acid receptor activity|GO:0045125,bioactive lipid receptor activity|GO:0035727,lysophosphatidic acid binding|GO:0043167,ion binding|GO:0099600,transmembrane receptor activity
NIPAL1	GO:0070838,divalent metal ion transport|GO:0006812,cation transport|GO:0006811,ion transport|GO:0006810,transport|GO:0044765,single-organism transport|GO:0015693,magnesium ion transport|GO:0030001,metal ion transport|GO:0044699,single-organism process|GO:0072511,divalent inorganic cation transport|GO:0051234,establishment of localization|GO:0051179,localization|GO:1902578,single-organism localization|GO:0009987,cellular process|GO:1903830,magnesium ion transmembrane transport|GO:0044763,single-organism cellular process|GO:0055085,transmembrane transport	GO:0044425,membrane part|GO:0016021,integral component of membrane|GO:0016020,membrane|GO:0031224,intrinsic component of membrane	GO:0022891,substrate-specific transmembrane transporter activity|GO:0022890,inorganic cation transmembrane transporter activity|GO:0022892,substrate-specific transporter activity|GO:0005215,transporter activity|GO:0008324,cation transmembrane transporter activity|GO:0072509,divalent inorganic cation transmembrane transporter activity|GO:0015075,ion transmembrane transporter activity|GO:0022857,transmembrane transporter activity|GO:0046873,metal ion transmembrane transporter activity|GO:0015095,magnesium ion transmembrane transporter activity
TYMS	GO:0009211,pyrimidine deoxyribonucleoside triphosphate metabolic process|GO:0009212,pyrimidine deoxyribonucleoside triphosphate biosynthetic process|GO:0080090,regulation of primary metabolic process|GO:0019222,regulation of metabolic process|GO:0009219,pyrimidine deoxyribonucleotide metabolic process|GO:0048468,cell development|GO:0000083,regulation of transcription involved in G1/S transition of mitotic cell cycle|GO:0001501,skeletal system development|GO:0009165,nucleotide biosynthetic process|GO:0009162,deoxyribonucleoside monophosphate metabolic process|GO:0009163,nucleoside biosynthetic process|GO:0009130,pyrimidine nucleoside monophosphate biosynthetic process|GO:0046134,pyrimidine nucleoside biosynthetic process|GO:1901362,organic cyclic compound biosynthetic process|GO:0051701,interaction with host|GO:1901360,organic cyclic compound metabolic process|GO:0043200,response to amino acid|GO:0044710,single-organism metabolic process|GO:0044711,single-organism biosynthetic process|GO:0048589,developmental growth|GO:0048869,cellular developmental process|GO:0044419,interspecies interaction between organisms|GO:0048511,rhythmic process|GO:0048513,animal organ development|GO:0051817,modification of morphology or physiology of other organism involved in symbiotic interaction|GO:0001101,response to acid chemical|GO:0030855,epithelial cell differentiation|GO:0051704,multi-organism process|GO:0002066,columnar/cuboidal epithelial cell development|GO:0002064,epithelial cell development|GO:0002065,columnar/cuboidal epithelial cell differentiation|GO:0006760,folic acid-containing compound metabolic process|GO:0009200,deoxyribonucleoside triphosphate metabolic process|GO:0060255,regulation of macromolecule metabolic process|GO:0000082,G1/S transition of mitotic cell cycle|GO:0048469,cell maturation|GO:0051593,response to folic acid|GO:0009177,pyrimidine deoxyribonucleoside monophosphate biosynthetic process|GO:0009176,pyrimidine deoxyribonucleoside monophosphate metabolic process|GO:2001141,regulation of RNA biosynthetic process|GO:0043436,oxoacid metabolic process|GO:0031100,animal organ regeneration|GO:0010033,response to organic substance|GO:0046483,heterocycle metabolic process|GO:0051216,cartilage development|GO:1901564,organonitrogen compound metabolic process|GO:0006231,dTMP biosynthetic process|GO:0044707,single-multicellular organism process|GO:0010243,response to organonitrogen compound|GO:0031667,response to nutrient levels|GO:0046683,response to organophosphorus|GO:0019637,organophosphate metabolic process|GO:0002070,epithelial cell maturation|GO:1901135,carbohydrate derivative metabolic process|GO:0044281,small molecule metabolic process|GO:0009148,pyrimidine nucleoside triphosphate biosynthetic process|GO:0060575,intestinal epithelial cell differentiation|GO:0060576,intestinal epithelial cell development|GO:0020021,immortalization of host cell|GO:0048565,digestive tract development|GO:0009141,nucleoside triphosphate metabolic process|GO:0009142,nucleoside triphosphate biosynthetic process|GO:0009147,pyrimidine nucleoside triphosphate metabolic process|GO:0014070,response to organic cyclic compound|GO:0006807,nitrogen compound metabolic process|GO:0046653,tetrahydrofolate metabolic process|GO:0021700,developmental maturation|GO:0050789,regulation of biological process|GO:0097659,nucleic acid-templated transcription|GO:0009605,response to external stimulus|GO:0043603,cellular amide metabolic process|GO:0090304,nucleic acid metabolic process|GO:0051186,cofactor metabolic process|GO:0044260,cellular macromolecule metabolic process|GO:0006206,pyrimidine nucleobase metabolic process|GO:0042558,pteridine-containing compound metabolic process|GO:0065007,biological regulation|GO:0007049,cell cycle|GO:0006366,transcription from RNA polymerase II promoter|GO:0065008,regulation of biological quality|GO:0018130,heterocycle biosynthetic process|GO:0009719,response to endogenous stimulus|GO:0007623,circadian rhythm|GO:0034097,response to cytokine|GO:0009394,2'-deoxyribonucleotide metabolic process|GO:0009157,deoxyribonucleoside monophosphate biosynthetic process|GO:0009889,regulation of biosynthetic process|GO:0009888,tissue development|GO:0050794,regulation of cellular process|GO:0009263,deoxyribonucleotide biosynthetic process|GO:0009262,deoxyribonucleotide metabolic process|GO:0009265,2'-deoxyribonucleotide biosynthetic process|GO:0008152,metabolic process|GO:0019438,aromatic compound biosynthetic process|GO:0034654,nucleobase-containing compound biosynthetic process|GO:0090407,organophosphate biosynthetic process|GO:0006575,cellular modified amino acid metabolic process|GO:0016070,RNA metabolic process|GO:0044271,cellular nitrogen compound biosynthetic process|GO:0045471,response to ethanol|GO:1901698,response to nitrogen compound|GO:0006213,pyrimidine nucleoside metabolic process|GO:0006355,regulation of transcription, DNA-templated|GO:0006357,regulation of transcription from RNA polymerase II promoter|GO:1901293,nucleoside phosphate biosynthetic process|GO:0044763,single-organism cellular process|GO:0006753,nucleoside phosphate metabolic process|GO:0097305,response to alcohol|GO:0009116,nucleoside metabolic process|GO:0060574,intestinal epithelial cell maturation|GO:0032774,RNA biosynthetic process|GO:0006732,coenzyme metabolic process|GO:0044699,single-organism process|GO:0030154,cell differentiation|GO:0055123,digestive system development|GO:0044249,cellular biosynthetic process|GO:0034641,cellular nitrogen compound metabolic process|GO:0009129,pyrimidine nucleoside monophosphate metabolic process|GO:0034645,cellular macromolecule biosynthetic process|GO:0042221,response to chemical|GO:0009123,nucleoside monophosphate metabolic process|GO:1901566,organonitrogen compound biosynthetic process|GO:0009124,nucleoside monophosphate biosynthetic process|GO:0006220,pyrimidine nucleotide metabolic process|GO:0006221,pyrimidine nucleotide biosynthetic process|GO:0009636,response to toxic substance|GO:0046385,deoxyribose phosphate biosynthetic process|GO:0031099,regeneration|GO:0002069,columnar/cuboidal epithelial cell maturation|GO:0033273,response to vitamin|GO:0032259,methylation|GO:1901605,alpha-amino acid metabolic process|GO:0032502,developmental process|GO:0032501,multicellular organismal process|GO:0019048,modulation by virus of host morphology or physiology|GO:0019088,immortalization of host cell by virus|GO:0060429,epithelium development|GO:0006725,cellular aromatic compound metabolic process|GO:1903506,regulation of nucleic acid-templated transcription|GO:0046073,dTMP metabolic process|GO:0046075,dTTP metabolic process|GO:1903047,mitotic cell cycle process|GO:0046078,dUMP metabolic process|GO:0019087,transformation of host cell by virus|GO:0055086,nucleobase-containing small molecule metabolic process|GO:0072528,pyrimidine-containing compound biosynthetic process|GO:0006235,dTTP biosynthetic process|GO:0007568,aging|GO:0006082,organic acid metabolic process|GO:1901137,carbohydrate derivative biosynthetic process|GO:0009725,response to hormone|GO:0019860,uracil metabolic process|GO:0051252,regulation of RNA metabolic process|GO:0042493,response to drug|GO:0009059,macromolecule biosynthetic process|GO:0043170,macromolecule metabolic process|GO:0006139,nucleobase-containing compound metabolic process|GO:0032570,response to progesterone|GO:0048731,system development|GO:0050896,response to stimulus|GO:0048545,response to steroid hormone|GO:0009991,response to extracellular stimulus|GO:0051384,response to glucocorticoid|GO:0031326,regulation of cellular biosynthetic process|GO:0031323,regulation of cellular metabolic process|GO:0019752,carboxylic acid metabolic process|GO:0044770,cell cycle phase transition|GO:0044772,mitotic cell cycle phase transition|GO:0022402,cell cycle process|GO:0007275,multicellular organism development|GO:0009202,deoxyribonucleoside triphosphate biosynthetic process|GO:0040007,growth|GO:0033993,response to lipid|GO:0006520,cellular amino acid metabolic process|GO:2000112,regulation of cellular macromolecule biosynthetic process|GO:1901654,response to ketone|GO:0071704,organic substance metabolic process|GO:0010467,gene expression|GO:0010556,regulation of macromolecule biosynthetic process|GO:0031960,response to corticosteroid|GO:0033189,response to vitamin A|GO:0072527,pyrimidine-containing compound metabolic process|GO:0010468,regulation of gene expression|GO:0006351,transcription, DNA-templated|GO:0009987,cellular process|GO:0000278,mitotic cell	GO:0044464,cell part|GO:0031974,membrane-enclosed lumen|GO:0031975,envelope|GO:0043229,intracellular organelle|GO:0043228,non-membrane-bounded organelle|GO:0043227,membrane-bounded organelle|GO:0043226,organelle|GO:0005737,cytoplasm|GO:0031090,organelle membrane|GO:0031981,nuclear lumen|GO:0005730,nucleolus|GO:0005634,nucleus|GO:0016020,membrane|GO:0044444,cytoplasmic part|GO:0005739,mitochondrion|GO:0005654,nucleoplasm|GO:0005759,mitochondrial matrix|GO:0031967,organelle envelope|GO:0031966,mitochondrial membrane|GO:0070013,intracellular organelle lumen|GO:0043231,intracellular membrane-bounded organelle|GO:0043232,intracellular non-membrane-bounded organelle|GO:0043233,organelle lumen|GO:0005829,cytosol|GO:0019866,organelle inner membrane|GO:0005623,cell|GO:0005622,intracellular|GO:0044446,intracellular organelle part|GO:0005743,mitochondrial inner membrane|GO:0005740,mitochondrial envelope|GO:0044428,nuclear part|GO:0044429,mitochondrial part|GO:0044424,intracellular part|GO:0044422,organelle part	GO:0008144,drug binding|GO:0005488,binding|GO:0003676,nucleic acid binding|GO:0004799,thymidylate synthase activity|GO:0008168,methyltransferase activity|GO:0000166,nucleotide binding|GO:0016597,amino acid binding|GO:1901363,heterocyclic compound binding|GO:1901265,nucleoside phosphate binding|GO:0043168,anion binding|GO:0005542,folic acid binding|GO:0043167,ion binding|GO:0016740,transferase activity|GO:0016741,transferase activity, transferring one-carbon groups|GO:0042802,identical protein binding|GO:0042803,protein homodimerization activity|GO:0033218,amide binding|GO:0044822,poly(A) RNA binding|GO:0036094,small molecule binding|GO:0003824,catalytic activity|GO:0048037,cofactor binding|GO:0072341,modified amino acid binding|GO:0043177,organic acid binding|GO:0097159,organic cyclic compound binding|GO:0031406,carboxylic acid binding|GO:0019842,vitamin binding|GO:0042083,5,10-methylenetetrahydrofolate-dependent methyltransferase activity|GO:0003723,RNA binding|GO:0005515,protein binding|GO:0046983,protein dimerization activity|GO:0003729,mRNA binding|GO:0000900,translation repressor activity, nucleic acid binding|GO:0030371,translation repressor activity|GO:0045182,translation regulator activity|GO:1990825,sequence-specific mRNA binding|GO:0090079,translation regulator activity, nucleic acid binding
MFAP3	GO:0009987,cellular process|GO:0016043,cellular component organization|GO:0030198,extracellular matrix organization|GO:0044763,single-organism cellular process|GO:0044699,single-organism process|GO:0043062,extracellular structure organization|GO:0071840,cellular component organization or biogenesis	GO:0016021,integral component of membrane|GO:0016020,membrane|GO:0044464,cell part|GO:0005623,cell|GO:0071944,cell periphery|GO:0005886,plasma membrane|GO:0044425,membrane part|GO:0031224,intrinsic component of membrane|GO:0005576,extracellular region	
GPR22	GO:0050896,response to stimulus|GO:0023052,signaling|GO:0007165,signal transduction|GO:0070887,cellular response to chemical stimulus|GO:0042221,response to chemical|GO:0050789,regulation of biological process|GO:0044699,single-organism process|GO:0009719,response to endogenous stimulus|GO:0051716,cellular response to stimulus|GO:0071310,cellular response to organic substance|GO:0065007,biological regulation|GO:0007186,G-protein coupled receptor signaling pathway|GO:0071495,cellular response to endogenous stimulus|GO:0009987,cellular process|GO:0050794,regulation of cellular process|GO:0032870,cellular response to hormone stimulus|GO:0044763,single-organism cellular process|GO:0007154,cell communication|GO:0010033,response to organic substance|GO:1901700,response to oxygen-containing compound|GO:0044700,single organism signaling|GO:0009725,response to hormone|GO:0010243,response to organonitrogen compound|GO:1901652,response to peptide|GO:1901698,response to nitrogen compound	GO:0031226,intrinsic component of plasma membrane|GO:0016021,integral component of membrane|GO:0016020,membrane|GO:0044464,cell part|GO:0005623,cell|GO:0071944,cell periphery|GO:0005887,integral component of plasma membrane|GO:0005886,plasma membrane|GO:0044425,membrane part|GO:0044459,plasma membrane part|GO:0031224,intrinsic component of membrane	GO:0004930,G-protein coupled receptor activity|GO:0038023,signaling receptor activity|GO:0033218,amide binding|GO:0060089,molecular transducer activity|GO:0004888,transmembrane signaling receptor activity|GO:0004872,receptor activity|GO:0004871,signal transducer activity|GO:0042277,peptide binding|GO:0005488,binding|GO:0099600,transmembrane receptor activity
NKX2			
H3F3B	GO:0007599,hemostasis|GO:0080090,regulation of primary metabolic process|GO:0019222,regulation of metabolic process|GO:0007596,blood coagulation|GO:0060322,head development|GO:0007165,signal transduction|GO:1901362,organic cyclic compound biosynthetic process|GO:0071840,cellular component organization or biogenesis|GO:0006306,DNA methylation|GO:0051716,cellular response to stimulus|GO:0006304,DNA modification|GO:0010605,negative regulation of macromolecule metabolic process|GO:0070271,protein complex biogenesis|GO:0040029,regulation of gene expression, epigenetic|GO:0016458,gene silencing|GO:0048513,animal organ development|GO:0048518,positive regulation of biological process|GO:0048519,negative regulation of biological process|GO:0060255,regulation of macromolecule metabolic process|GO:2001141,regulation of RNA biosynthetic process|GO:0010033,response to organic substance|GO:0046483,heterocycle metabolic process|GO:0044700,single organism signaling|GO:0032259,methylation|GO:0044707,single-multicellular organism process|GO:0019438,aromatic compound biosynthetic process|GO:0051253,negative regulation of RNA metabolic process|GO:0071103,DNA conformation change|GO:0022607,cellular component assembly|GO:0009892,negative regulation of metabolic process|GO:0034645,cellular macromolecule biosynthetic process|GO:0009890,negative regulation of biosynthetic process|GO:0010629,negative regulation of gene expression|GO:0006807,nitrogen compound metabolic process|GO:0035556,intracellular signal transduction|GO:0050789,regulation of biological process|GO:0097659,nucleic acid-templated transcription|GO:1901576,organic substance biosynthetic process|GO:0016049,cell growth|GO:0044260,cellular macromolecule metabolic process|GO:0016043,cellular component organization|GO:0065003,macromolecular complex assembly|GO:0065007,biological regulation|GO:1901360,organic cyclic compound metabolic process|GO:0065004,protein-DNA complex assembly|GO:0065008,regulation of biological quality|GO:0018130,heterocycle biosynthetic process|GO:0009889,regulation of biosynthetic process|GO:0006305,DNA alkylation|GO:0042060,wound healing|GO:0050794,regulation of cellular process|GO:0043412,macromolecule modification|GO:0050817,coagulation|GO:0043414,macromolecule methylation|GO:0008152,metabolic process|GO:0034654,nucleobase-containing compound biosynthetic process|GO:0016070,RNA metabolic process|GO:0044767,single-organism developmental process|GO:0044271,cellular nitrogen compound biosynthetic process|GO:0007420,brain development|GO:0050896,response to stimulus|GO:0006950,response to stress|GO:0006355,regulation of transcription, DNA-templated|GO:0010556,regulation of macromolecule biosynthetic process|GO:0006351,transcription, DNA-templated|GO:0009611,response to wounding|GO:0010558,negative regulation of macromolecule biosynthetic process|GO:0034728,nucleosome organization|GO:0032774,RNA biosynthetic process|GO:0044728,DNA methylation or demethylation|GO:0032776,DNA methylation on cytosine|GO:0030307,positive regulation of cell growth|GO:0034724,DNA replication-independent nucleosome organization|GO:0051128,regulation of cellular component organization|GO:0044249,cellular biosynthetic process|GO:0034641,cellular nitrogen compound metabolic process|GO:0023052,signaling|GO:0031497,chromatin assembly|GO:0042221,response to chemical|GO:0044699,single-organism process|GO:0007417,central nervous system development|GO:0009719,response to endogenous stimulus|GO:0006139,nucleobase-containing compound metabolic process|GO:0006342,chromatin silencing|GO:0043933,macromolecular complex subunit organization|GO:0032502,developmental process|GO:0032501,multicellular organismal process|GO:0050878,regulation of body fluid levels|GO:0009987,cellular process|GO:0006725,cellular aromatic compound metabolic process|GO:1903506,regulation of nucleic acid-templated transcription|GO:1903507,negative regulation of nucleic acid-templated transcription|GO:0001558,regulation of cell growth|GO:0045892,negative regulation of transcription, DNA-templated|GO:0006336,DNA replication-independent nucleosome assembly|GO:0009725,response to hormone|GO:0006334,nucleosome assembly|GO:0051252,regulation of RNA metabolic process|GO:0006333,chromatin assembly or disassembly|GO:0043170,macromolecule metabolic process|GO:0048731,system development|GO:0000183,chromatin silencing at rDNA|GO:0045927,positive regulation of growth|GO:0031327,negative regulation of cellular biosynthetic process|GO:0031326,regulation of cellular biosynthetic process|GO:0031324,negative regulation of cellular metabolic process|GO:0031323,regulation of cellular metabolic process|GO:0090304,nucleic acid metabolic process|GO:0034622,cellular macromolecular complex assembly|GO:0007275,multicellular organism development|GO:0071824,protein-DNA complex subunit organization|GO:0040007,growth|GO:0071822,protein complex subunit organization|GO:0006325,chromatin organization|GO:0040008,regulation of growth|GO:2000112,regulation of cellular macromolecule biosynthetic process|GO:2000113,negative regulation of cellular macromolecule biosynthetic process|GO:0006323,DNA packaging|GO:0071704,organic substance metabolic process|GO:0010467,gene expression|GO:0045814,negative regulation of gene expression, epigenetic|GO:0010468,regulation of gene expression|GO:0045934,negative regulation of nucleobase-containing compound metabolic process|GO:0019219,regulation of nucleobase-containing compound metabolic process|GO:0006461,protein complex assembly|GO:1902679,negative regulation of RNA biosynthetic process|GO:0009058,biosynthetic process|GO:0009059,macromolecule biosynthetic process|GO:0044763,single-organism cellular process|GO:0051171,regulation of nitrogen compound metabolic process|GO:0051172,negative regulation of nitrogen compound metabolic process|GO:0007154,cell communication|GO:0007264,small GTPase mediated signal transduction|GO:0006996,organelle organization|GO:0044238,primary metabolic process|GO:0051276,chromosome organization|GO:0007399,nervous system development|GO:0048856,anatomical structure development|GO:0044237,cellular metabolic process|GO:0044085,cellular component biogenesis|GO:0006259,DNA metabolic process|GO:0048523,negative regulation of cellular process|GO:0048522,positive regulation of cellular process|GO:0001503,ossification|GO:0007286,spermatid development|GO:0048468,cell development|GO:0008584,male gonad development|GO:0007292,female gamete generation|GO:0031055,chromatin remodeling at centromere|GO:0035264,multicellular organism growth|GO:0010604,positive regulation of macromolecule metabolic process|GO:0010608,posttranscriptional regulation of gene expression|GO:0043043,peptide biosynthetic process|GO:0045137,development of primary sexual characteristics|GO:0048515,spermatid differentiation|GO:0051704,multi-organism process|GO:0007281,germ cell development|GO:0003006,developmental process involved in reproduction|GO:0060147,regulation of posttranscriptional gene silencing|GO:1901564,organonitrogen compound metabolic process|GO:0044703,multi-organism reproductive process|GO:0044702,single organism reproductive process|GO:0031507,heterochromatin assembly|GO:0043603,cellular amide metabolic process|GO:0044706,multi-multicellular organism process|GO:0019538,protein metabolic process|GO:0031509,telomeric heterochromatin assembly|GO:0031508,pericentric heterochromatin assembly|GO:0009566,fertilization|GO:0010639,negative regulation of organelle organization|GO:0016441,posttranscriptional gene silencing|GO:0042692,muscle cell differentiation|GO:0009893,positive regulation of metabolic process|GO:0010628,positive regulation of gene expression|GO:0048589,developmental growth|GO:0044267,cellular protein metabolic process|GO:0008406,gonad development|GO:0048477,oogenesis|GO:0046546,development of primary male sexual characteristics|GO:0007566,embryo implantation|GO:0046661,male sex differentiation|GO:0000003,reproduction|GO:0007548,sex differentiation|GO:0022414,reproductive process|GO:0048869,cellular developmental process|GO:0006518,peptide metabolic process|GO:0031047,gene silencing by RNA|GO:0033044,regulation of chromosome organization|GO:0033043,regulation of organelle organization|GO:0030154,cell differentiation|GO:0019953,sexual reproduction|GO:0051129,negative regulation of cellular component organization|GO:0030261,chromosome condensation|GO:0061061,muscle structure development|GO:1901566,organonitrogen compound biosynthetic process|GO:0051248,negative regulation of protein	GO:0031974,membrane-enclosed lumen|GO:0044815,DNA packaging complex|GO:0043229,intracellular organelle|GO:0043228,non-membrane-bounded organelle|GO:0000785,chromatin|GO:0000786,nucleosome|GO:0000788,nuclear nucleosome|GO:0000228,nuclear chromosome|GO:0043227,membrane-bounded organelle|GO:0043226,organelle|GO:0070062,extracellular exosome|GO:0031981,nuclear lumen|GO:0005634,nucleus|GO:0031988,membrane-bounded vesicle|GO:0005654,nucleoplasm|GO:0044454,nuclear chromosome part|GO:0005694,chromosome|GO:0000790,nuclear chromatin|GO:0032991,macromolecular complex|GO:1903561,extracellular vesicle|GO:0031982,vesicle|GO:0043234,protein complex|GO:0043230,extracellular organelle|GO:0043231,intracellular membrane-bounded organelle|GO:0043232,intracellular non-membrane-bounded organelle|GO:0043233,organelle lumen|GO:0044464,cell part|GO:0005623,cell|GO:0005622,intracellular|GO:0044446,intracellular organelle part|GO:0070013,intracellular organelle lumen|GO:0005576,extracellular region|GO:0044428,nuclear part|GO:0044424,intracellular part|GO:0032993,protein-DNA complex|GO:0044427,chromosomal part|GO:0065010,extracellular membrane-bounded organelle|GO:0044421,extracellular region part|GO:0044422,organelle part|GO:0000781,chromosome, telomeric region|GO:0000784,nuclear chromosome, telomeric region|GO:0001740,Barr body|GO:0000805,X chromosome|GO:0000803,sex chromosome|GO:0098687,chromosomal region	GO:0042393,histone binding|GO:0001012,RNA polymerase II regulatory region DNA binding|GO:0001158,enhancer sequence-specific DNA binding|GO:0031491,nucleosome binding|GO:0031490,chromatin DNA binding|GO:0031492,nucleosomal DNA binding|GO:0044877,macromolecular complex binding|GO:0005488,binding|GO:0003676,nucleic acid binding|GO:0003677,DNA binding|GO:1901363,heterocyclic compound binding|GO:0043565,sequence-specific DNA binding|GO:0043566,structure-specific DNA binding|GO:0032403,protein complex binding|GO:0000980,RNA polymerase II distal enhancer sequence-specific DNA binding|GO:0046982,protein heterodimerization activity|GO:0001067,regulatory region nucleic acid binding|GO:0035326,enhancer binding|GO:0001047,core promoter binding|GO:0001046,core promoter sequence-specific DNA binding|GO:0044212,transcription regulatory region DNA binding|GO:0003682,chromatin binding|GO:0097159,organic cyclic compound binding|GO:0000976,transcription regulatory region sequence-specific DNA binding|GO:0000977,RNA polymerase II regulatory region sequence-specific DNA binding|GO:0005515,protein binding|GO:0000975,regulatory region DNA binding|GO:0046983,protein dimerization activity|GO:0000979,RNA polymerase II core promoter sequence-specific DNA binding|GO:1990837,sequence-specific double-stranded DNA binding|GO:0003690,double-stranded DNA binding
TOR1AIP1		GO:0044425,membrane part|GO:0016021,integral component of membrane|GO:0016020,membrane|GO:0031224,intrinsic component of membrane	
CSRNP2	GO:0019220,regulation of phosphate metabolic process|GO:0080090,regulation of primary metabolic process|GO:0019222,regulation of metabolic process|GO:0006470,protein dephosphorylation|GO:1901576,organic substance biosynthetic process|GO:1901362,organic cyclic compound biosynthetic process|GO:1901360,organic cyclic compound metabolic process|GO:0010605,negative regulation of macromolecule metabolic process|GO:0010604,positive regulation of macromolecule metabolic process|GO:0044092,negative regulation of molecular function|GO:0048518,positive regulation of biological process|GO:0048519,negative regulation of biological process|GO:0060255,regulation of macromolecule metabolic process|GO:2001141,regulation of RNA biosynthetic process|GO:0046483,heterocycle metabolic process|GO:0019538,protein metabolic process|GO:0019438,aromatic compound biosynthetic process|GO:0009892,negative regulation of metabolic process|GO:0009893,positive regulation of metabolic process|GO:0009891,positive regulation of biosynthetic process|GO:0010628,positive regulation of gene expression|GO:0050789,regulation of biological process|GO:0097659,nucleic acid-templated transcription|GO:0044267,cellular protein metabolic process|GO:0051346,negative regulation of hydrolase activity|GO:0044260,cellular macromolecule metabolic process|GO:0065007,biological regulation|GO:0006366,transcription from RNA polymerase II promoter|GO:0065009,regulation of molecular function|GO:0018130,heterocycle biosynthetic process|GO:0016265,obsolete death|GO:0050790,regulation of catalytic activity|GO:0009889,regulation of biosynthetic process|GO:0050794,regulation of cellular process|GO:0035308,negative regulation of protein dephosphorylation|GO:0043412,macromolecule modification|GO:0036211,protein modification process|GO:0008152,metabolic process|GO:0034654,nucleobase-containing compound biosynthetic process|GO:0035303,regulation of dephosphorylation|GO:0035305,negative regulation of dephosphorylation|GO:0035304,regulation of protein dephosphorylation|GO:0016070,RNA metabolic process|GO:0044271,cellular nitrogen compound biosynthetic process|GO:0012501,programmed cell death|GO:0031400,negative regulation of protein modification process|GO:0006355,regulation of transcription, DNA-templated|GO:0010557,positive regulation of macromolecule biosynthetic process|GO:0010556,regulation of macromolecule biosynthetic process|GO:0006351,transcription, DNA-templated|GO:0032774,RNA biosynthetic process|GO:0016311,dephosphorylation|GO:0010921,regulation of phosphatase activity|GO:0010923,negative regulation of phosphatase activity|GO:0044249,cellular biosynthetic process|GO:0034641,cellular nitrogen compound metabolic process|GO:0034645,cellular macromolecule biosynthetic process|GO:0043086,negative regulation of catalytic activity|GO:0044699,single-organism process|GO:0006139,nucleobase-containing compound metabolic process|GO:0051248,negative regulation of protein metabolic process|GO:0010563,negative regulation of phosphorus metabolic process|GO:0051246,regulation of protein metabolic process|GO:0031399,regulation of protein modification process|GO:1903508,positive regulation of nucleic acid-templated transcription|GO:0051336,regulation of hydrolase activity|GO:0009987,cellular process|GO:0006725,cellular aromatic compound metabolic process|GO:1903506,regulation of nucleic acid-templated transcription|GO:0045893,positive regulation of transcription, DNA-templated|GO:0032269,negative regulation of cellular protein metabolic process|GO:0032268,regulation of cellular protein metabolic process|GO:0051252,regulation of RNA metabolic process|GO:0051254,positive regulation of RNA metabolic process|GO:0043170,macromolecule metabolic process|GO:1902680,positive regulation of RNA biosynthetic process|GO:0006807,nitrogen compound metabolic process|GO:0045944,positive regulation of transcription from RNA polymerase II promoter|GO:0031328,positive regulation of cellular biosynthetic process|GO:0031326,regulation of cellular biosynthetic process|GO:0031325,positive regulation of cellular metabolic process|GO:0031324,negative regulation of cellular metabolic process|GO:0031323,regulation of cellular metabolic process|GO:0090304,nucleic acid metabolic process|GO:0008219,cell death|GO:2000112,regulation of cellular macromolecule biosynthetic process|GO:0071704,organic substance metabolic process|GO:0010467,gene expression|GO:0006357,regulation of transcription from RNA polymerase II promoter|GO:0010468,regulation of gene expression|GO:0045935,positive regulation of nucleobase-containing compound metabolic process|GO:0045936,negative regulation of phosphate metabolic process|GO:0019219,regulation of nucleobase-containing compound metabolic process|GO:0006915,apoptotic process|GO:0006464,cellular protein modification process|GO:0051174,regulation of phosphorus metabolic process|GO:0009058,biosynthetic process|GO:0009059,macromolecule biosynthetic process|GO:0044763,single-organism cellular process|GO:0051171,regulation of nitrogen compound metabolic process|GO:0051173,positive regulation of nitrogen compound metabolic process|GO:0044238,primary metabolic process|GO:0044237,cellular metabolic process|GO:0006796,phosphate-containing compound metabolic process|GO:0006793,phosphorus metabolic process|GO:0048523,negative regulation of cellular process|GO:0048522,positive regulation of cellular process	GO:0043231,intracellular membrane-bounded organelle|GO:0005634,nucleus|GO:0044464,cell part|GO:0005623,cell|GO:0005622,intracellular|GO:0043229,intracellular organelle|GO:0044424,intracellular part|GO:0043227,membrane-bounded organelle|GO:0043226,organelle	GO:0043565,sequence-specific DNA binding|GO:0005515,protein binding|GO:0003700,transcription factor activity, sequence-specific DNA binding|GO:0097159,organic cyclic compound binding|GO:0001228,transcriptional activator activity, RNA polymerase II transcription regulatory region sequence-specific binding|GO:0019899,enzyme binding|GO:0019902,phosphatase binding|GO:0005488,binding|GO:0003676,nucleic acid binding|GO:0003677,DNA binding|GO:1901363,heterocyclic compound binding|GO:0000981,RNA polymerase II transcription factor activity, sequence-specific DNA binding|GO:0001071,nucleic acid binding transcription factor activity

FSCN1	GO:0022607,cellular component assembly|GO:0061572,actin filament bundle organization|GO:0043933,macromolecular complex subunit organization|GO:0030036,actin cytoskeleton organization|GO:0006928,movement of cell or subcellular component|GO:0051674,localization of cell|GO:0071840,cellular component organization or biogenesis|GO:0071822,protein complex subunit organization|GO:0016043,cellular component organization|GO:0044699,single-organism process|GO:0016477,cell migration|GO:0040011,locomotion|GO:0030029,actin filament-based process|GO:0008283,cell proliferation|GO:0009987,cellular process|GO:0051017,actin filament bundle assembly|GO:0044763,single-organism cellular process|GO:0051179,localization|GO:0006996,organelle organization|GO:0007015,actin filament organization|GO:0007010,cytoskeleton organization|GO:0048870,cell motility|GO:1902589,single-organism organelle organization|GO:0044085,cellular component biogenesis|GO:0060491,regulation of cell projection assembly|GO:0007163,establishment or maintenance of cell polarity|GO:0031344,regulation of cell projection organization|GO:0031346,positive regulation of cell projection organization|GO:0070271,protein complex biogenesis|GO:0051493,regulation of cytoskeleton organization|GO:0051491,positive regulation of filopodium assembly|GO:0048518,positive regulation of biological process|GO:0043062,extracellular structure organization|GO:0010638,positive regulation of organelle organization|GO:0030198,extracellular matrix organization|GO:0061245,establishment or maintenance of bipolar cell polarity|GO:0050789,regulation of biological process|GO:0061162,establishment of monopolar cell polarity|GO:0035088,establishment or maintenance of apical/basal cell polarity|GO:0035089,establishment of apical/basal cell polarity|GO:0065003,macromolecular complex assembly|GO:0065007,biological regulation|GO:0051130,positive regulation of cellular component organization|GO:0050794,regulation of cellular process|GO:0032956,regulation of actin cytoskeleton organization|GO:0043254,regulation of protein complex assembly|GO:0010715,regulation of extracellular matrix disassembly|GO:0022411,cellular component disassembly|GO:0033043,regulation of organelle organization|GO:0051128,regulation of cellular component organization|GO:0007043,cell-cell junction assembly|GO:0030010,establishment of cell polarity|GO:0009653,anatomical structure morphogenesis|GO:0071803,positive regulation of podosome assembly|GO:0071800,podosome assembly|GO:0071801,regulation of podosome assembly|GO:0097435,fibril organization|GO:0022617,extracellular matrix disassembly|GO:0032502,developmental process|GO:0010591,regulation of lamellipodium assembly|GO:0051489,regulation of filopodium assembly|GO:1903053,regulation of extracellular matrix organization|GO:0032970,regulation of actin filament-based process|GO:0031334,positive regulation of protein complex assembly|GO:1903055,positive regulation of extracellular matrix organization|GO:0045216,cell-cell junction organization|GO:0034329,cell junction assembly|GO:0030030,cell projection organization|GO:0030031,cell projection assembly|GO:0030032,lamellipodium assembly|GO:0034330,cell junction organization|GO:1902115,regulation of organelle assembly|GO:1902117,positive regulation of organelle assembly|GO:0090091,positive regulation of extracellular matrix disassembly|GO:0061339,establishment or maintenance of monopolar cell polarity|GO:0006461,protein complex assembly|GO:0097581,lamellipodium organization|GO:0070925,organelle assembly|GO:0010592,positive regulation of lamellipodium assembly|GO:0048856,anatomical structure development|GO:0044087,regulation of cellular component biogenesis|GO:1902745,positive regulation of lamellipodium organization|GO:0044089,positive regulation of cellular component biogenesis|GO:1902743,regulation of lamellipodium organization|GO:0048522,positive regulation of cellular process|GO:0044700,single organism signaling|GO:0051716,cellular response to stimulus|GO:0019221,cytokine-mediated signaling pathway|GO:0071345,cellular response to cytokine stimulus|GO:0050896,response to stimulus|GO:0071310,cellular response to organic substance|GO:0023052,signaling|GO:0007165,signal transduction|GO:0007166,cell surface receptor signaling pathway|GO:0042221,response to chemical|GO:0007154,cell communication|GO:0070887,cellular response to chemical stimulus|GO:0010033,response to organic substance|GO:0034097,response to cytokine	GO:0032432,actin filament bundle|GO:0015629,actin cytoskeleton|GO:0030175,filopodium|GO:0043229,intracellular organelle|GO:0043228,non-membrane-bounded organelle|GO:0005622,intracellular|GO:0043227,membrane-bounded organelle|GO:0043226,organelle|GO:0030054,cell junction|GO:0042641,actomyosin|GO:0005856,cytoskeleton|GO:0001725,stress fiber|GO:0042995,cell projection|GO:0031982,vesicle|GO:0043209,myelin sheath|GO:0031988,membrane-bounded vesicle|GO:0044430,cytoskeletal part|GO:0071437,invadopodium|GO:1903561,extracellular vesicle|GO:0070062,extracellular exosome|GO:0043230,extracellular organelle|GO:0043232,intracellular non-membrane-bounded organelle|GO:0005829,cytosol|GO:0044464,cell part|GO:0005623,cell|GO:0005737,cytoplasm|GO:0044446,intracellular organelle part|GO:0044444,cytoplasmic part|GO:0005576,extracellular region|GO:0044424,intracellular part|GO:0065010,extracellular membrane-bounded organelle|GO:0044421,extracellular region part|GO:0044422,organelle part|GO:0098590,plasma membrane region|GO:0099512,supramolecular fiber|GO:0030427,site of polarized growth|GO:0030426,growth cone|GO:0001726,ruffle|GO:0002102,podosome|GO:0098805,whole membrane|GO:0016020,membrane|GO:0097517,contractile actin filament bundle|GO:0097458,neuron part|GO:0043005,neuron projection|GO:0044459,plasma membrane part|GO:0031941,filamentous actin|GO:0030027,lamellipodium|GO:0098589,membrane region|GO:0031253,cell projection membrane|GO:0031252,cell leading edge|GO:0005884,actin filament|GO:0005911,cell-cell junction|GO:0005886,plasma membrane|GO:0032991,macromolecular complex|GO:0099513,polymeric cytoskeletal fiber|GO:0043234,protein complex|GO:0005902,microvillus|GO:0071944,cell periphery|GO:0098858,actin-based cell projection|GO:0044425,membrane part	GO:0005488,binding|GO:0030674,protein binding, bridging|GO:0005515,protein binding|GO:0008092,cytoskeletal protein binding|GO:0097159,organic cyclic compound binding|GO:0044822,poly(A) RNA binding|GO:0051015,actin filament binding|GO:0008144,drug binding|GO:0003779,actin binding|GO:0044877,macromolecular complex binding|GO:0003723,RNA binding|GO:0003676,nucleic acid binding|GO:0032403,protein complex binding|GO:1901363,heterocyclic compound binding|GO:0060090,binding, bridging|GO:0045296,cadherin binding|GO:0050839,cell adhesion molecule binding
ABHD17C	GO:0008152,metabolic process|GO:0008104,protein localization|GO:0060341,regulation of cellular localization|GO:0035372,protein localization to microtubule|GO:0044380,protein localization to cytoskeleton|GO:1903827,regulation of cellular protein localization|GO:0044249,cellular biosynthetic process|GO:0006807,nitrogen compound metabolic process|GO:0002084,protein depalmitoylation|GO:0042157,lipoprotein metabolic process|GO:0034645,cellular macromolecule biosynthetic process|GO:0098732,macromolecule deacylation|GO:0072698,protein localization to microtubule cytoskeleton|GO:1901576,organic substance biosynthetic process|GO:0042158,lipoprotein biosynthetic process|GO:0042159,lipoprotein catabolic process|GO:0050789,regulation of biological process|GO:0032880,regulation of protein localization|GO:0043543,protein acylation|GO:1901575,organic substance catabolic process|GO:0044260,cellular macromolecule metabolic process|GO:0070727,cellular macromolecule localization|GO:0051641,cellular localization|GO:0051179,localization|GO:0071704,organic substance metabolic process|GO:1902817,negative regulation of protein localization to microtubule|GO:1902816,regulation of protein localization to microtubule|GO:0065007,biological regulation|GO:0048519,negative regulation of biological process|GO:1903828,negative regulation of cellular protein localization|GO:0034613,cellular protein localization|GO:0035601,protein deacylation|GO:0044267,cellular protein metabolic process|GO:0009987,cellular process|GO:0006464,cellular protein modification process|GO:0050794,regulation of cellular process|GO:0043412,macromolecule modification|GO:0036211,protein modification process|GO:0030163,protein catabolic process|GO:0032879,regulation of localization|GO:0009056,catabolic process|GO:0009057,macromolecule catabolic process|GO:1901565,organonitrogen compound catabolic process|GO:0006497,protein lipidation|GO:0044238,primary metabolic process|GO:0033036,macromolecule localization|GO:1901564,organonitrogen compound metabolic process|GO:0018345,protein palmitoylation|GO:1901566,organonitrogen compound biosynthetic process|GO:0098734,macromolecule depalmitoylation|GO:0019538,protein metabolic process|GO:0009058,biosynthetic process|GO:0044237,cellular metabolic process|GO:0043170,macromolecule metabolic process|GO:0009059,macromolecule biosynthetic process|GO:0033365,protein localization to organelle	GO:0098590,plasma membrane region|GO:0055038,recycling endosome membrane|GO:0055037,recycling endosome|GO:0099572,postsynaptic specialization|GO:0043229,intracellular organelle|GO:0043228,non-membrane-bounded organelle|GO:0045202,synapse|GO:0098984,neuron to neuron synapse|GO:0043227,membrane-bounded organelle|GO:0043226,organelle|GO:0030054,cell junction|GO:0010008,endosome membrane|GO:0097060,synaptic membrane|GO:0031982,vesicle|GO:0031410,cytoplasmic vesicle|GO:0098805,whole membrane|GO:0016020,membrane|GO:0044309,neuron spine|GO:0044433,cytoplasmic vesicle part|GO:0097458,neuron part|GO:0044456,synapse part|GO:0043005,neuron projection|GO:0044459,plasma membrane part|GO:0032279,asymmetric synapse|GO:0005737,cytoplasm|GO:0098588,bounding membrane of organelle|GO:0098589,membrane region|GO:0071944,cell periphery|GO:0045211,postsynaptic membrane|GO:0014069,postsynaptic density|GO:0097708,intracellular vesicle|GO:0005886,plasma membrane|GO:0036477,somatodendritic compartment|GO:0031090,organelle membrane|GO:0030425,dendrite|GO:0043232,intracellular non-membrane-bounded organelle|GO:0043197,dendritic spine|GO:0044464,cell part|GO:0005623,cell|GO:0005622,intracellular|GO:0044446,intracellular organelle part|GO:0044444,cytoplasmic part|GO:0098794,postsynapse|GO:0044440,endosomal part|GO:0044424,intracellular part|GO:0044425,membrane part|GO:0005768,endosome|GO:0044422,organelle part|GO:0012505,endomembrane system	GO:0016787,hydrolase activity|GO:0003824,catalytic activity|GO:0098599,palmitoyl hydrolase activity|GO:0008474,palmitoyl-(protein) hydrolase activity|GO:0016790,thiolester hydrolase activity|GO:0016788,hydrolase activity, acting on ester bonds
MYO5A	GO:0008152,metabolic process|GO:0007017,microtubule-based process|GO:0009987,cellular process|GO:0007018,microtubule-based movement|GO:0006928,movement of cell or subcellular component|GO:0044763,single-organism cellular process|GO:0044699,single-organism process	GO:0005856,cytoskeleton|GO:0043234,protein complex|GO:0043228,non-membrane-bounded organelle|GO:0015629,actin cytoskeleton|GO:0043232,intracellular non-membrane-bounded organelle|GO:0044464,cell part|GO:0005623,cell|GO:0005622,intracellular|GO:0044446,intracellular organelle part|GO:0043229,intracellular organelle|GO:0016459,myosin complex|GO:0044430,cytoskeletal part|GO:0044424,intracellular part|GO:0032991,macromolecular complex|GO:0043226,organelle|GO:0044422,organelle part	GO:0035639,purine ribonucleoside triphosphate binding|GO:0005488,binding|GO:0043167,ion binding|GO:1901363,heterocyclic compound binding|GO:1901265,nucleoside phosphate binding|GO:0001882,nucleoside binding|GO:0043168,anion binding|GO:0000166,nucleotide binding|GO:0001883,purine nucleoside binding|GO:0032549,ribonucleoside binding|GO:0017076,purine nucleotide binding|GO:0003774,motor activity|GO:0005524,ATP binding|GO:0016787,hydrolase activity|GO:0017111,nucleoside-triphosphatase activity|GO:0036094,small molecule binding|GO:0003824,catalytic activity|GO:0016818,hydrolase activity, acting on acid anhydrides, in phosphorus-containing anhydrides|GO:0030554,adenyl nucleotide binding|GO:0097367,carbohydrate derivative binding|GO:0097159,organic cyclic compound binding|GO:0016817,hydrolase activity, acting on acid anhydrides|GO:0016462,pyrophosphatase activity|GO:0032559,adenyl ribonucleotide binding|GO:0032555,purine ribonucleotide binding|GO:0032550,purine ribonucleoside binding|GO:0032553,ribonucleotide binding|GO:0015631,tubulin binding|GO:0008092,cytoskeletal protein binding|GO:0008017,microtubule binding|GO:0051015,actin filament binding|GO:0003779,actin binding|GO:0044877,macromolecular complex binding|GO:0005515,protein binding|GO:0032403,protein complex binding|GO:0003777,microtubule motor
FLI1	GO:0007599,hemostasis|GO:0080090,regulation of primary metabolic process|GO:0019222,regulation of metabolic process|GO:0048468,cell development|GO:0001501,skeletal system development|GO:0003013,circulatory system process|GO:1901362,organic cyclic compound biosynthetic process|GO:1901360,organic cyclic compound metabolic process|GO:0010604,positive regulation of macromolecule metabolic process|GO:0048869,cellular developmental process|GO:0010467,gene expression|GO:0048513,animal organ development|GO:0048518,positive regulation of biological process|GO:0003008,system process|GO:0060255,regulation of macromolecule metabolic process|GO:0030219,megakaryocyte differentiation|GO:2001141,regulation of RNA biosynthetic process|GO:0046483,heterocycle metabolic process|GO:0044707,single-multicellular organism process|GO:0002376,immune system process|GO:0019438,aromatic compound biosynthetic process|GO:0009893,positive regulation of metabolic process|GO:0009891,positive regulation of biosynthetic process|GO:0098751,bone cell development|GO:0010628,positive regulation of gene expression|GO:0050789,regulation of biological process|GO:0097659,nucleic acid-templated transcription|GO:1901576,organic substance biosynthetic process|GO:0044260,cellular macromolecule metabolic process|GO:0035855,megakaryocyte development|GO:0065007,biological regulation|GO:0006366,transcription from RNA polymerase II promoter|GO:0065008,regulation of biological quality|GO:0018130,heterocycle biosynthetic process|GO:0009887,animal organ morphogenesis|GO:0008015,blood circulation|GO:0009889,regulation of biosynthetic process|GO:0050794,regulation of cellular process|GO:0008152,metabolic process|GO:0030097,hemopoiesis|GO:0034654,nucleobase-containing compound biosynthetic process|GO:0002520,immune system development|GO:0016070,RNA metabolic process|GO:0044271,cellular nitrogen compound biosynthetic process|GO:0006355,regulation of transcription, DNA-templated|GO:0010557,positive regulation of macromolecule biosynthetic process|GO:0010556,regulation of macromolecule biosynthetic process|GO:0006351,transcription, DNA-templated|GO:0032774,RNA biosynthetic process|GO:0030154,cell differentiation|GO:0044249,cellular biosynthetic process|GO:0034641,cellular nitrogen compound metabolic process|GO:0034645,cellular macromolecule biosynthetic process|GO:0009653,anatomical structure morphogenesis|GO:0044699,single-organism process|GO:0006139,nucleobase-containing compound metabolic process|GO:0032502,developmental process|GO:0032501,multicellular organismal process|GO:0050878,regulation of body fluid levels|GO:0009987,cellular process|GO:0006725,cellular aromatic compound metabolic process|GO:1903506,regulation of nucleic acid-templated transcription|GO:0045893,positive regulation of transcription, DNA-templated|GO:0051252,regulation of RNA metabolic process|GO:0051254,positive regulation of RNA metabolic process|GO:0043170,macromolecule metabolic process|GO:1902680,positive regulation of RNA biosynthetic process|GO:0006807,nitrogen compound metabolic process|GO:0045944,positive regulation of transcription from RNA polymerase II promoter|GO:0030099,myeloid cell differentiation|GO:0048731,system development|GO:1903508,positive regulation of nucleic acid-templated transcription|GO:0031328,positive regulation of cellular biosynthetic process|GO:0031326,regulation of cellular biosynthetic process|GO:0031325,positive regulation of cellular metabolic process|GO:0031323,regulation of cellular metabolic process|GO:0060348,bone development|GO:0090304,nucleic acid metabolic process|GO:0061515,myeloid cell development|GO:0007275,multicellular organism development|GO:2000112,regulation of cellular macromolecule biosynthetic process|GO:0071704,organic substance metabolic process|GO:0002244,hematopoietic progenitor cell differentiation|GO:0006357,regulation of transcription from RNA polymerase II promoter|GO:0048534,hematopoietic or lymphoid organ development|GO:0010468,regulation of gene expression|GO:0045935,positive regulation of nucleobase-containing compound metabolic process|GO:0019219,regulation of nucleobase-containing compound metabolic process|GO:0044767,single-organism developmental process|GO:0009058,biosynthetic process|GO:0009059,macromolecule biosynthetic process|GO:0044763,single-organism cellular process|GO:0051171,regulation of nitrogen compound metabolic process|GO:0051173,positive regulation of nitrogen compound metabolic process|GO:0044238,primary metabolic process|GO:0048856,anatomical structure development|GO:0044237,cellular metabolic process|GO:0048522,positive regulation of cellular process	GO:0043231,intracellular membrane-bounded organelle|GO:0005634,nucleus|GO:0044464,cell part|GO:0005623,cell|GO:0005622,intracellular|GO:0043229,intracellular organelle|GO:0044424,intracellular part|GO:0043227,membrane-bounded organelle|GO:0043226,organelle|GO:0031974,membrane-enclosed lumen|GO:0005737,cytoplasm|GO:0016604,nuclear body|GO:0031981,nuclear lumen|GO:0005654,nucleoplasm|GO:0044451,nucleoplasm part|GO:0043233,organelle lumen|GO:0005829,cytosol|GO:0044446,intracellular organelle part|GO:0070013,intracellular organelle lumen|GO:0044444,cytoplasmic part|GO:0044428,nuclear part|GO:0044422,organelle part	GO:0001012,RNA polymerase II regulatory region DNA binding|GO:0001159,core promoter proximal region DNA binding|GO:0001158,enhancer sequence-specific DNA binding|GO:0001077,transcriptional activator activity, RNA polymerase II core promoter proximal region sequence-specific binding|GO:0044877,macromolecular complex binding|GO:0005488,binding|GO:0003676,nucleic acid binding|GO:0003677,DNA binding|GO:1901363,heterocyclic compound binding|GO:0043565,sequence-specific DNA binding|GO:0000987,core promoter proximal region sequence-specific DNA binding|GO:0000982,transcription factor activity, RNA polymerase II core promoter proximal region sequence-specific binding|GO:0000981,RNA polymerase II transcription factor activity, sequence-specific DNA binding|GO:0000980,RNA polymerase II distal enhancer sequence-specific DNA binding|GO:0001067,regulatory region nucleic acid binding|GO:0035326,enhancer binding|GO:0044212,transcription regulatory region DNA binding|GO:0003700,transcription factor activity, sequence-specific DNA binding|GO:0097159,organic cyclic compound binding|GO:0001228,transcriptional activator activity, RNA polymerase II transcription regulatory region sequence-specific binding|GO:0000976,transcription regulatory region sequence-specific DNA binding|GO:0000977,RNA polymerase II regulatory region sequence-specific DNA binding|GO:0005515,protein binding|GO:0000975,regulatory region DNA binding|GO:0000978,RNA polymerase II core promoter proximal region sequence-specific DNA binding|GO:0003682,chromatin binding|GO:0001071,nucleic acid binding transcription factor activity|GO:1990837,sequence-specific double-stranded DNA binding|GO:0003690,double-
ABRACL			
MBTD1	GO:0048856,anatomical structure development|GO:0080090,regulation of primary metabolic process|GO:0019222,regulation of metabolic process|GO:0043933,macromolecular complex subunit organization|GO:0031326,regulation of cellular biosynthetic process|GO:0031323,regulation of cellular metabolic process|GO:0090304,nucleic acid metabolic process|GO:0009790,embryo development|GO:0034641,cellular nitrogen compound metabolic process|GO:0009792,embryo development ending in birth or egg hatching|GO:0006807,nitrogen compound metabolic process|GO:0019438,aromatic compound biosynthetic process|GO:0034645,cellular macromolecule biosynthetic process|GO:1901362,organic cyclic compound biosynthetic process|GO:0050789,regulation of biological process|GO:0044699,single-organism process|GO:0032774,RNA biosynthetic process|GO:0006139,nucleobase-containing compound metabolic process|GO:0044260,cellular macromolecule metabolic process|GO:0071704,organic substance metabolic process|GO:0006325,chromatin organization|GO:1901360,organic cyclic compound metabolic process|GO:2000112,regulation of cellular macromolecule biosynthetic process|GO:0016043,cellular component organization|GO:0007275,multicellular organism development|GO:0060255,regulation of macromolecule metabolic process|GO:0010467,gene expression|GO:0065007,biological regulation|GO:0071840,cellular component organization or biogenesis|GO:0043009,chordate embryonic development|GO:0044249,cellular biosynthetic process|GO:0010468,regulation of gene expression|GO:0018130,heterocycle biosynthetic process|GO:0032502,developmental process|GO:0016070,RNA metabolic process|GO:0048706,embryonic skeletal system development|GO:1901576,organic substance biosynthetic process|GO:0019219,regulation of nucleobase-containing compound metabolic process|GO:0006725,cellular aromatic compound metabolic process|GO:0032501,multicellular organismal process|GO:0009987,cellular process|GO:0009889,regulation of biosynthetic process|GO:1903506,regulation of nucleic acid-templated transcription|GO:0050794,regulation of cellular process|GO:0044767,single-organism developmental process|GO:0009058,biosynthetic process|GO:0009059,macromolecule biosynthetic process|GO:0051171,regulation of nitrogen compound metabolic process|GO:0008152,metabolic process|GO:2001141,regulation of RNA biosynthetic process|GO:0034654,nucleobase-containing compound biosynthetic process|GO:0046483,heterocycle metabolic process|GO:0006996,organelle organization|GO:0044238,primary metabolic process|GO:0044271,cellular nitrogen compound biosynthetic process|GO:0051276,chromosome organization|GO:0044707,single-multicellular organism process|GO:0001501,skeletal system development|GO:0051252,regulation of RNA metabolic process|GO:0044237,cellular metabolic process|GO:0043170,macromolecule metabolic process|GO:0006355,regulation of transcription, DNA-templated|GO:0010556,regulation of macromolecule biosynthetic process|GO:0006351,transcription, DNA-templated|GO:0048731,system development|GO:0097659,nucleic acid-templated transcription|GO:0016568,chromatin modification|GO:0043412,macromolecule modification|GO:0016569,covalent chromatin modification	GO:0043231,intracellular membrane-bounded organelle|GO:0005634,nucleus|GO:0044464,cell part|GO:0005623,cell|GO:0005622,intracellular|GO:0043229,intracellular organelle|GO:0044424,intracellular part|GO:0043227,membrane-bounded organelle|GO:0043226,organelle	GO:0043169,cation binding|GO:0046914,transition metal ion binding|GO:0043167,ion binding|GO:0005488,binding|GO:0046872,metal ion binding|GO:0008270,zinc ion binding|GO:0042393,histone binding|GO:0035064,methylated histone binding|GO:0005515,protein binding
BLOC1S6	GO:0008104,protein localization|GO:0007599,hemostasis|GO:0006904,vesicle docking involved in exocytosis|GO:0032438,melanosome organization|GO:0007596,blood coagulation|GO:0048468,cell development|GO:0048066,developmental pigmentation|GO:0061025,membrane fusion|GO:0061024,membrane organization|GO:0001505,regulation of neurotransmitter levels|GO:0051656,establishment of organelle localization|GO:0051650,establishment of vesicle localization|GO:0071840,cellular component organization or biogenesis|GO:0051716,cellular response to stimulus|GO:0048869,cellular developmental process|GO:0009611,response to wounding|GO:0030855,epithelial cell differentiation|GO:0048518,positive regulation of biological process|GO:0065007,biological regulation|GO:0097479,synaptic vesicle localization|GO:0033036,macromolecule localization|GO:0002065,columnar/cuboidal epithelial cell differentiation|GO:0050932,regulation of pigment cell differentiation|GO:0050931,pigment cell differentiation|GO:0048070,regulation of developmental pigmentation|GO:0045184,establishment of protein localization|GO:0048278,vesicle docking|GO:0010970,transport along microtubule|GO:0006836,neurotransmitter transport|GO:0097480,establishment of synaptic vesicle localization|GO:0016197,endosomal transport|GO:0044700,single organism signaling|GO:0016192,vesicle-mediated transport|GO:0044707,single-multicellular organism process|GO:0016050,vesicle organization|GO:0002376,immune system process|GO:0048087,positive regulation of developmental pigmentation|GO:0050942,positive regulation of pigment cell differentiation|GO:0006928,movement of cell or subcellular component|GO:0045321,leukocyte activation|GO:0032940,secretion by cell|GO:0031175,neuron projection development|GO:0051179,localization|GO:0021700,developmental maturation|GO:0050789,regulation of biological process|GO:0048489,synaptic vesicle transport|GO:0006887,exocytosis|GO:0008088,axo-dendritic transport|GO:0008089,anterograde axonal transport|GO:0051875,pigment granule localization|GO:0009306,protein secretion|GO:0002684,positive regulation of immune system process|GO:0002682,regulation of immune system process|GO:0035646,endosome to melanosome transport|GO:0065008,regulation of biological quality|GO:0032814,regulation of natural killer cell activation|GO:0032816,positive regulation of natural killer cell activation|GO:0050793,regulation of developmental process|GO:0006810,transport|GO:0009888,tissue development|GO:0042060,wound healing|GO:0050794,regulation of cellular process|GO:0001775,cell activation|GO:0050817,coagulation|GO:0048490,anterograde synaptic vesicle transport|GO:0051234,establishment of localization|GO:0006892,post-Golgi vesicle-mediated transport|GO:0046903,secretion|GO:0046907,intracellular transport|GO:0016079,synaptic vesicle exocytosis|GO:0050896,response to stimulus|GO:0006950,response to stress|GO:0002694,regulation of leukocyte activation|GO:0002696,positive regulation of leukocyte activation|GO:0051648,vesicle localization|GO:0030154,cell differentiation|GO:0023052,signaling|GO:0044699,single-organism process|GO:0016043,cellular component organization|GO:0051249,regulation of lymphocyte activation|GO:0051649,establishment of localization in cell|GO:0043485,endosome to pigment granule transport|GO:0043482,cellular pigment accumulation|GO:0030705,cytoskeleton-dependent intracellular transport|GO:0030030,cell projection organization|GO:0051640,organelle localization|GO:0060563,neuroepithelial cell differentiation|GO:0032402,melanosome transport|GO:0032401,establishment of melanosome localization|GO:0032400,melanosome localization|GO:0032502,developmental process|GO:0032501,multicellular organismal process|GO:0030318,melanocyte differentiation|GO:0050878,regulation of body fluid levels|GO:0016081,synaptic vesicle docking|GO:0060429,epithelium development|GO:0033299,secretion of lysosomal enzymes|GO:0045595,regulation of cell differentiation|GO:0016482,cytosolic transport|GO:0043473,pigmentation|GO:0043476,pigment accumulation|GO:0051094,positive regulation of developmental process|GO:0051251,positive regulation of lymphocyte activation|GO:0051905,establishment of pigment granule localization|GO:0051904,pigment granule transport|GO:0048731,system development|GO:0050865,regulation of cell activation|GO:0048753,pigment granule organization|GO:0050867,positive regulation of cell activation|GO:0048757,pigment granule maturation|GO:0022406,membrane docking|GO:0007275,multicellular organism development|GO:0033059,cellular pigmentation|GO:0071702,organic substance transport|GO:0009605,response to external stimulus|GO:0048666,neuron development|GO:0009987,cellular process|GO:0030182,neuron differentiation|GO:0048193,Golgi vesicle transport|GO:0023061,signal release|GO:0044767,single-organism developmental process|GO:0044765,single-organism transport|GO:0044763,single-organism cellular process|GO:0007269,neurotransmitter secretion|GO:0007268,chemical synaptic transmission|GO:0007267,cell-cell signaling|GO:0007154,cell communication|GO:0022008,neurogenesis|GO:0030101,natural killer cell activation|GO:1902578,single-organism localization|GO:0051641,cellular localization|GO:0006996,organelle organization|GO:0045597,positive regulation of cell differentiation|GO:0048699,generation of neurons|GO:0007017,microtubule-based process|GO:0007399,nervous system development|GO:0048856,anatomical structure development|GO:0007018,microtubule-based movement|GO:0046649,lymphocyte activation|GO:0015031,protein transport|GO:1902582,single-organism intracellular transport|GO:1902580,single-organism cellular localization|GO:0048522,positive regulation of cellular process|GO:0099517,synaptic vesicle transport along microtubule|GO:0099514,synaptic vesicle cytoskeletal transport|GO:0099518,vesicle cytoskeletal trafficking|GO:0099504,synaptic vesicle cycle|GO:0098916,anterograde trans-synaptic signaling|GO:0045055,regulated exocytosis|GO:0072384,organelle transport along microtubule|GO:0098930,axonal transport|GO:0099531,presynaptic process involved in chemical synaptic transmission|GO:0047496,vesicle transport along microtubule|GO:0099536,synaptic signaling|GO:0099537,trans-synaptic signaling|GO:0099643,signal release from synapse|GO:0017156,calcium	GO:0031974,membrane-enclosed lumen|GO:0019898,extrinsic component of membrane|GO:0043226,organelle|GO:0043229,intracellular organelle|GO:0030133,transport vesicle|GO:0043227,membrane-bounded organelle|GO:0031201,SNARE complex|GO:0005737,cytoplasm|GO:0031982,vesicle|GO:0031981,nuclear lumen|GO:0070013,intracellular organelle lumen|GO:0016023,cytoplasmic, membrane-bounded vesicle|GO:0031410,cytoplasmic vesicle|GO:0005634,nucleus|GO:0016020,membrane|GO:0031988,membrane-bounded vesicle|GO:0005654,nucleoplasm|GO:0012505,endomembrane system|GO:0032991,macromolecular complex|GO:0031083,BLOC-1 complex|GO:0043234,protein complex|GO:0031082,BLOC complex|GO:0043231,intracellular membrane-bounded organelle|GO:0043233,organelle lumen|GO:0005829,cytosol|GO:0044464,cell part|GO:0005623,cell|GO:0005622,intracellular|GO:0044446,intracellular organelle part|GO:0098796,membrane protein complex|GO:0044444,cytoplasmic part|GO:0044445,cytosolic part|GO:0044428,nuclear part|GO:0044424,intracellular part|GO:0044425,membrane part|GO:0005768,endosome|GO:0044422,organelle part|GO:0045202,synapse|GO:1904115,axon cytoplasm|GO:0097458,neuron part|GO:0044456,synapse part|GO:0043005,neuron projection|GO:0032838,cell projection cytoplasm|GO:0099568,cytoplasmic region|GO:0097708,intracellular vesicle|GO:0030424,axon|GO:0098793,presynapse|GO:0033267,axon part	GO:0008092,cytoskeletal protein binding|GO:0000149,SNARE binding|GO:0051015,actin filament binding|GO:0003779,actin binding|GO:0019905,syntaxin binding|GO:0005488,binding|GO:0005515,protein binding|GO:0032403,protein complex binding|GO:0044877,macromolecular complex binding|GO:0046983,protein dimerization activity|GO:0042802,identical protein binding|GO:0042803,protein homodimerization activity
SWAP70	GO:0044238,primary metabolic process|GO:0019220,regulation of phosphate metabolic process|GO:0080090,regulation of primary metabolic process|GO:0019222,regulation of metabolic process|GO:0002312,B cell activation involved in immune response|GO:0048584,positive regulation of response to stimulus|GO:0048583,regulation of response to stimulus|GO:0006139,nucleobase-containing compound metabolic process|GO:0007610,behavior|GO:0008064,regulation of actin polymerization or depolymerization|GO:0007162,negative regulation of cell adhesion|GO:0008104,protein localization|GO:0050920,regulation of chemotaxis|GO:0050921,positive regulation of chemotaxis|GO:0060326,cell chemotaxis|GO:0098771,inorganic ion homeostasis|GO:0006796,phosphate-containing compound metabolic process|GO:0071840,cellular component organization or biogenesis|GO:0061572,actin filament bundle organization|GO:0032231,regulation of actin filament bundle assembly|GO:0044710,single-organism metabolic process|GO:0032233,positive regulation of actin filament bundle assembly|GO:0010605,negative regulation of macromolecule metabolic process|GO:0042330,taxis|GO:0071704,organic substance metabolic process|GO:0051495,positive regulation of cytoskeleton organization|GO:0051494,negative regulation of cytoskeleton organization|GO:0051493,regulation of cytoskeleton organization|GO:0002208,somatic diversification of immunoglobulins involved in immune response|GO:0033043,regulation of organelle organization|GO:0002366,leukocyte activation involved in immune response|GO:0006470,protein dephosphorylation|GO:0048518,positive regulation of biological process|GO:0048519,negative regulation of biological process|GO:0007015,actin filament organization|GO:0050801,ion homeostasis|GO:0019725,cellular homeostasis|GO:0019724,B cell mediated immunity|GO:0030595,leukocyte chemotaxis|GO:0006935,chemotaxis|GO:0060255,regulation of macromolecule metabolic process|GO:1902309,negative regulation of peptidyl-serine dephosphorylation|GO:1902308,regulation of peptidyl-serine dephosphorylation|GO:1901880,negative regulation of protein depolymerization|GO:0006928,movement of cell or subcellular component|GO:0046649,lymphocyte activation|GO:0030042,actin filament depolymerization|GO:0046483,heterocycle metabolic process|GO:0016477,cell migration|GO:0009605,response to external stimulus|GO:0044707,single-multicellular organism process|GO:0048870,cell motility|GO:0019538,protein metabolic process|GO:0010638,positive regulation of organelle organization|GO:0010639,negative regulation of organelle organization|GO:0048878,chemical homeostasis|GO:0043242,negative regulation of protein complex disassembly|GO:0002377,immunoglobulin production|GO:0002204,somatic recombination of immunoglobulin genes involved in immune response|GO:0002250,adaptive immune response|GO:0016445,somatic diversification of immunoglobulins|GO:0016444,somatic cell DNA recombination|GO:0016447,somatic recombination of immunoglobulin gene segments|GO:0055074,calcium ion homeostasis|GO:0033036,macromolecule localization|GO:0022607,cellular component assembly|GO:0009892,negative regulation of metabolic process|GO:1901879,regulation of protein depolymerization|GO:0007204,positive regulation of cytosolic calcium ion concentration|GO:0045321,leukocyte activation|GO:0065007,biological regulation|GO:0050789,regulation of biological process|GO:0044267,cellular protein metabolic process|GO:0044260,cellular macromolecule metabolic process|GO:0002285,lymphocyte activation involved in immune response|GO:0016043,cellular component organization|GO:0090066,regulation of anatomical structure size|GO:0002687,positive regulation of leukocyte migration|GO:0002684,positive regulation of immune system process|GO:0002685,regulation of leukocyte migration|GO:0002682,regulation of immune system process|GO:1901360,organic cyclic compound metabolic process|GO:0002200,somatic diversification of immune receptors|GO:0060753,regulation of mast cell chemotaxis|GO:0002688,regulation of leukocyte chemotaxis|GO:0051130,positive regulation of cellular component organization|GO:0098602,single organism cell adhesion|GO:0072507,divalent inorganic cation homeostasis|GO:0098609,cell-cell adhesion|GO:0051716,cellular response to stimulus|GO:0050795,regulation of behavior|GO:0050794,regulation of cellular process|GO:0008154,actin polymerization or depolymerization|GO:0035308,negative regulation of protein dephosphorylation|GO:0001775,cell activation|GO:0036211,protein modification process|GO:0002263,cell activation involved in immune response|GO:0008152,metabolic process|GO:0032956,regulation of actin cytoskeleton organization|GO:0035303,regulation of dephosphorylation|GO:0035305,negative regulation of dephosphorylation|GO:0035304,regulation of protein dephosphorylation|GO:0002520,immune system development|GO:0044767,single-organism developmental process|GO:0031400,negative regulation of protein modification process|GO:0043412,macromolecule modification|GO:2000145,regulation of cell motility|GO:0002690,positive regulation of leukocyte chemotaxis|GO:2000147,positive regulation of cell motility|GO:0044763,single-organism cellular process|GO:0002551,mast cell chemotaxis|GO:0032103,positive regulation of response to external stimulus|GO:0032101,regulation of response to external stimulus|GO:0016311,dephosphorylation|GO:0030155,regulation of cell adhesion|GO:0006955,immune response|GO:0051129,negative regulation of cellular component organization|GO:0051128,regulation of cellular component organization|GO:0002440,production of molecular mediator of immune response|GO:0034641,cellular nitrogen compound metabolic process|GO:0070887,cellular response to chemical stimulus|GO:0060754,positive regulation of mast cell chemotaxis|GO:0043244,regulation of protein complex disassembly|GO:0043241,protein complex disassembly|GO:0044699,single-organism process|GO:0032880,regulation of protein localization|GO:0032535,regulation of cellular component size|GO:0051248,negative regulation of protein metabolic process|GO:0016064,immunoglobulin mediated immune response|GO:0072503,cellular divalent inorganic cation homeostasis|GO:0010563,negative regulation of phosphorus metabolic process|GO:0051246,regulation of protein metabolic process|GO:0031399,regulation of protein modification process|GO:0022610,biological adhesion|GO:0032502,developmental process|GO:0006310,DNA recombination|GO:0045190,isotype switching|GO:0002562,somatic diversification of immune receptors via germline recombination within a single locus|GO:0006875,cellular metal ion homeostasis|GO:0006874,cellular calcium ion homeostasis|GO:0032970,regulation of actin filament-based process|GO:0051017,actin filament bundle assembly|GO:0006873,cellular ion homeostasis|GO:0051270,regulation of cellular component movement|GO:0040011,locomotion|GO:0030003,cellular cation homeostasis|GO:0055080,cation homeostasis|GO:0055082,cellular chemical homeostasis|GO:0032879,regulation of localization|GO:0090304,nucleic acid metabolic process|GO:0032269,negative regulation of cellular protein metabolic process|GO:0032268,regulation of cellular protein metabolic process|GO:0051674,localization of cell|GO:0065008,regulation of biological quality|GO:0002460,adaptive immune response based on somatic recombination of immune receptors built from immunoglobulin superfamily domains|GO:0043170,macromolecule metabolic process|GO:0006807,nitrogen compound metabolic process|GO:0048731,system development|GO:0009987,cellular process|GO:0097529,myeloid leukocyte migration|GO:0050896,response to stimulus|GO:0016337,single organismal cell-cell adhesion|GO:0043933,macromolecular complex subunit organization|GO:0002376,immune system process|GO:0031324,negative regulation of cellular metabolic process|GO:0031323,regulation of cellular metabolic process|GO:0042113,B cell activation|GO:0030036,actin cytoskeleton organization|GO:0042592,homeostatic process|GO:0022407,regulation of cell-cell adhesion|GO:0050900,leukocyte migration|GO:0032501,multicellular organismal process|GO:0007275,multicellular organism development|GO:0022408,negative regulation of cell-cell adhesion|GO:0097531,mast cell migration|GO:0030832,regulation of actin filament length|GO:0030834,regulation of actin filament depolymerization|GO:0030835,negative regulation of actin filament depolymerization|GO:0071822,protein complex subunit organization|GO:0070262,peptidyl-serine dephosphorylation|GO:0051261,protein depolymerization|GO:0002443,leukocyte mediated immunity|GO:0033628,regulation of cell adhesion mediated by integrin|GO:0033629,negative regulation of cell adhesion	GO:0005737,cytoplasm|GO:0005829,cytosol|GO:0005622,intracellular|GO:0043231,intracellular membrane-bounded organelle|GO:0030027,lamellipodium|GO:0005634,nucleus|GO:0016020,membrane|GO:0044464,cell part|GO:0044444,cytoplasmic part|GO:0005623,cell|GO:0031252,cell leading edge|GO:0043229,intracellular organelle|GO:0071944,cell periphery|GO:0005886,plasma membrane|GO:0044424,intracellular part|GO:0042995,cell projection|GO:0043227,membrane-bounded organelle|GO:0043226,organelle|GO:0005856,cytoskeleton|GO:0043232,intracellular non-membrane-bounded organelle|GO:0043228,non-membrane-bounded organelle	GO:0035639,purine ribonucleoside triphosphate binding|GO:0005488,binding|GO:0003676,nucleic acid binding|GO:0003677,DNA binding|GO:0000166,nucleotide binding|GO:1901363,heterocyclic compound binding|GO:1901265,nucleoside phosphate binding|GO:0001882,nucleoside binding|GO:0043168,anion binding|GO:0043169,cation binding|GO:0043167,ion binding|GO:0005509,calcium ion binding|GO:0032549,ribonucleoside binding|GO:0017076,purine nucleotide binding|GO:0046872,metal ion binding|GO:0005524,ATP binding|GO:0001883,purine nucleoside binding|GO:0036094,small molecule binding|GO:0030554,adenyl nucleotide binding|GO:0097367,carbohydrate derivative binding|GO:0097159,organic cyclic compound binding|GO:0032559,adenyl ribonucleotide binding|GO:0005515,protein binding|GO:0032555,purine ribonucleotide binding|GO:0032550,purine ribonucleoside binding|GO:0032553,ribonucleotide binding|GO:0050839,cell adhesion molecule binding|GO:0045296,cadherin binding
TPT1		GO:0005737,cytoplasm|GO:0044464,cell part|GO:0005623,cell|GO:0005622,intracellular|GO:0044424,intracellular part|GO:0005829,cytosol|GO:0044444,cytoplasmic part	
YPEL4		GO:0043231,intracellular membrane-bounded organelle|GO:0031981,nuclear lumen|GO:0043233,organelle lumen|GO:0005634,nucleus|GO:0005730,nucleolus|GO:0044464,cell part|GO:0043232,intracellular non-membrane-bounded organelle|GO:0031974,membrane-enclosed lumen|GO:0005622,intracellular|GO:0044446,intracellular organelle part|GO:0070013,intracellular organelle lumen|GO:0043229,intracellular organelle|GO:0043228,non-membrane-bounded organelle|GO:0044428,nuclear part|GO:0005623,cell|GO:0044424,intracellular part|GO:0043227,membrane-bounded organelle|GO:0043226,organelle|GO:0044422,organelle part	GO:0043169,cation binding|GO:0046872,metal ion binding|GO:0005488,binding|GO:0043167,ion binding

BMP7	GO:0033157,regulation of intracellular protein transport|GO:0051169,nuclear transport|GO:0007409,axonogenesis|GO:0051049,regulation of transport|GO:0016358,dendrite development|GO:0001501,skeletal system development|GO:0001503,ossification|GO:2000736,regulation of stem cell differentiation|GO:0051716,cellular response to stimulus|GO:0014031,mesenchymal cell development|GO:0003156,regulation of animal organ formation|GO:0001666,response to hypoxia|GO:0000165,MAPK cascade|GO:0018210,peptidyl-threonine modification|GO:0008104,protein localization|GO:0030850,prostate gland development|GO:0030855,epithelial cell differentiation|GO:0007088,regulation of mitotic nuclear division|GO:0032386,regulation of intracellular transport|GO:0032387,negative regulation of intracellular transport|GO:0060548,negative regulation of cell death|GO:0033280,response to vitamin D|GO:0045859,regulation of protein kinase activity|GO:0035272,exocrine system development|GO:0097485,neuron projection guidance|GO:0046483,heterocycle metabolic process|GO:0042325,regulation of phosphorylation|GO:0042327,positive regulation of phosphorylation|GO:0042326,negative regulation of phosphorylation|GO:2000121,regulation of removal of superoxide radicals|GO:0019538,protein metabolic process|GO:0010639,negative regulation of organelle organization|GO:0051783,regulation of nuclear division|GO:0051782,negative regulation of cell division|GO:0051784,negative regulation of nuclear division|GO:0051130,positive regulation of cellular component organization|GO:0048562,embryonic organ morphogenesis|GO:0009892,negative regulation of metabolic process|GO:0009893,positive regulation of metabolic process|GO:0009890,negative regulation of biosynthetic process|GO:0009891,positive regulation of biosynthetic process|GO:0000305,response to oxygen radical|GO:0048568,embryonic organ development|GO:0031076,embryonic camera-type eye development|GO:0000303,response to superoxide|GO:0051254,positive regulation of RNA metabolic process|GO:1901342,regulation of vasculature development|GO:1901343,negative regulation of vasculature development|GO:0031175,neuron projection development|GO:0035556,intracellular signal transduction|GO:0051223,regulation of protein transport|GO:0051224,negative regulation of protein transport|GO:0050789,regulation of biological process|GO:0009880,embryonic pattern specification|GO:0000904,cell morphogenesis involved in differentiation|GO:0000902,cell morphogenesis|GO:0006886,intracellular protein transport|GO:0051348,negative regulation of transferase activity|GO:0070887,cellular response to chemical stimulus|GO:1901360,organic cyclic compound metabolic process|GO:0018130,heterocycle biosynthetic process|GO:0048705,skeletal system morphogenesis|GO:0048704,embryonic skeletal system morphogenesis|GO:0070201,regulation of establishment of protein localization|GO:0070169,positive regulation of biomineral tissue development|GO:1903649,regulation of cytoplasmic transport|GO:0098609,cell-cell adhesion|GO:0090194,negative regulation of glomerulus development|GO:2000377,regulation of reactive oxygen species metabolic process|GO:0090192,regulation of glomerulus development|GO:0043412,macromolecule modification|GO:0048863,stem cell differentiation|GO:0060601,lateral sprouting from an epithelium|GO:0016070,RNA metabolic process|GO:0072028,nephron morphogenesis|GO:0010557,positive regulation of macromolecule biosynthetic process|GO:0010556,regulation of macromolecule biosynthetic process|GO:0048869,cellular developmental process|GO:0097305,response to alcohol|GO:0010558,negative regulation of macromolecule biosynthetic process|GO:0007498,mesoderm development|GO:0051129,negative regulation of cellular component organization|GO:0051128,regulation of cellular component organization|GO:1903827,regulation of cellular protein localization|GO:0022008,neurogenesis|GO:0014070,response to organic cyclic compound|GO:1903828,negative regulation of cellular protein localization|GO:0009653,anatomical structure morphogenesis|GO:0000280,nuclear division|GO:0060284,regulation of cell development|GO:0045746,negative regulation of Notch signaling pathway|GO:0033273,response to vitamin|GO:0008285,negative regulation of cell proliferation|GO:0035239,tube morphogenesis|GO:0008283,cell proliferation|GO:2000036,regulation of stem cell population maintenance|GO:1902455,negative regulation of stem cell population maintenance|GO:0048518,positive regulation of biological process|GO:0001654,eye development|GO:0001655,urogenital system development|GO:0001656,metanephros development|GO:0001657,ureteric bud development|GO:0006979,response to oxidative stress|GO:0044699,single-organism process|GO:0018107,peptidyl-threonine phosphorylation|GO:0060341,regulation of cellular localization|GO:0030030,cell projection organization|GO:0022407,regulation of cell-cell adhesion|GO:0022402,cell cycle process|GO:0008219,cell death|GO:0051302,regulation of cell division|GO:0051301,cell division|GO:0022409,positive regulation of cell-cell adhesion|GO:0032355,response to estradiol|GO:0033993,response to lipid|GO:0072203,cell proliferation involved in metanephros development|GO:2000112,regulation of cellular macromolecule biosynthetic process|GO:2000113,negative regulation of cellular macromolecule biosynthetic process|GO:0007275,multicellular organism development|GO:0043065,positive regulation of apoptotic process|GO:0043067,regulation of programmed cell death|GO:0043066,negative regulation of apoptotic process|GO:0043062,extracellular structure organization|GO:0043069,negative regulation of programmed cell death|GO:0097285,obsolete cell-type specific apoptotic process|GO:0048598,embryonic morphogenesis|GO:0090092,regulation of transmembrane receptor protein serine/threonine kinase signaling pathway|GO:0006469,negative regulation of protein kinase activity|GO:0000278,mitotic cell cycle|GO:0019219,regulation of nucleobase-containing compound metabolic process|GO:0048592,eye morphogenesis|GO:0048593,camera-type eye morphogenesis|GO:0090317,negative regulation of intracellular protein transport|GO:0048596,embryonic camera-type eye morphogenesis|GO:0044767,single-organism developmental process|GO:0044765,single-organism transport|GO:0044763,single-organism cellular process|GO:0060485,mesenchyme development|GO:1902882,regulation of response to oxidative stress|GO:0040011,locomotion|GO:1901701,cellular response to oxygen-containing compound|GO:0048858,cell projection morphogenesis|GO:0048856,anatomical structure development|GO:1900104,regulation of hyaluranon cable assembly|GO:0035295,tube development|GO:0006796,phosphate-containing compound metabolic process|GO:2000026,regulation of multicellular organismal development|GO:2000027,regulation of organ morphogenesis|GO:0006793,phosphorus metabolic process|GO:0071773,cellular response to BMP stimulus|GO:0048523,negative regulation of cellular process|GO:0048522,positive regulation of cellular process|GO:0034116,positive regulation of heterotypic cell-cell adhesion|GO:0034114,regulation of heterotypic cell-cell adhesion|GO:0034113,heterotypic cell-cell adhesion|GO:2000242,negative regulation of reproductive process|GO:2000241,regulation of reproductive process|GO:0007165,signal transduction|GO:0007166,cell surface receptor signaling pathway|GO:0007167,enzyme linked receptor protein signaling pathway|GO:0031344,regulation of cell projection organization|GO:0031346,positive regulation of cell projection organization|GO:0072111,cell proliferation involved in kidney development|GO:0072110,glomerular mesangial cell proliferation|GO:0044710,single-organism metabolic process|GO:0045786,negative regulation of cell cycle|GO:0045785,positive regulation of cell adhesion|GO:0007369,gastrulation|GO:0070848,response to growth factor|GO:0045665,negative regulation of neuron differentiation|GO:0045664,regulation of neuron differentiation|GO:0045667,regulation of osteoblast differentiation|GO:0045666,positive regulation of neuron differentiation|GO:0061138,morphogenesis of a branching epithelium|GO:0044092,negative regulation of molecular function|GO:0060993,kidney morphogenesis|GO:0033036,macromolecule localization|GO:0045669,positive regulation of osteoblast differentiation|GO:0006935,chemotaxis|GO:0051051,negative regulation of transport|GO:0044700,single organism signaling|GO:2001141,regulation of RNA biosynthetic process|GO:0010035,response to inorganic substance|GO:0042992,negative regulation of transcription factor import into nucleus|GO:0010033,response to organic substance|GO:0042990,regulation of transcription factor import into nucleus|GO:0042991,transcription factor import into nucleus|GO:0031503,protein complex localization|GO:0019827,stem cell population maintenance|GO:0009605,response	GO:0005615,extracellular space|GO:0044421,extracellular region part|GO:0031988,membrane-bounded vesicle|GO:0005576,extracellular region|GO:0031012,extracellular matrix|GO:0043227,membrane-bounded organelle|GO:0043226,organelle|GO:0031982,vesicle	GO:1901681,sulfur compound binding|GO:0043168,anion binding|GO:0005125,cytokine activity|GO:0008083,growth factor activity|GO:0033612,receptor serine/threonine kinase binding|GO:0097367,carbohydrate derivative binding|GO:0005160,transforming growth factor beta receptor binding|GO:0005102,receptor binding|GO:0070700,BMP receptor binding|GO:0043167,ion binding|GO:0005539,glycosaminoglycan binding|GO:0005488,binding|GO:0005515,protein binding|GO:0005126,cytokine receptor binding|GO:0008201,heparin binding|GO:0070696,transmembrane receptor protein serine/threonine kinase binding
NCAM1	GO:0042221,response to chemical|GO:0032502,developmental process|GO:0044707,single-multicellular organism process|GO:0019221,cytokine-mediated signaling pathway|GO:0030030,cell projection organization|GO:0030154,cell differentiation|GO:0048468,cell development|GO:0006928,movement of cell or subcellular component|GO:0023052,signaling|GO:0007165,signal transduction|GO:0007166,cell surface receptor signaling pathway|GO:0031175,neuron projection development|GO:0007411,axon guidance|GO:0032989,cellular component morphogenesis|GO:0006955,immune response|GO:0007275,multicellular organism development|GO:0044699,single-organism process|GO:0034097,response to cytokine|GO:0051716,cellular response to stimulus|GO:0000904,cell morphogenesis involved in differentiation|GO:0000902,cell morphogenesis|GO:0042330,taxis|GO:0009653,anatomical structure morphogenesis|GO:0048869,cellular developmental process|GO:0016043,cellular component organization|GO:0050789,regulation of biological process|GO:0071310,cellular response to organic substance|GO:0030198,extracellular matrix organization|GO:0065007,biological regulation|GO:0071840,cellular component organization or biogenesis|GO:0034341,response to interferon-gamma|GO:0043062,extracellular structure organization|GO:0022610,biological adhesion|GO:0009605,response to external stimulus|GO:0048666,neuron development|GO:0048667,cell morphogenesis involved in neuron differentiation|GO:0032501,multicellular organismal process|GO:0061564,axon development|GO:0006935,chemotaxis|GO:0030182,neuron differentiation|GO:0045087,innate immune response|GO:0009987,cellular process|GO:0006952,defense response|GO:0050794,regulation of cellular process|GO:0044767,single-organism developmental process|GO:0060333,interferon-gamma-mediated signaling pathway|GO:0006950,response to stress|GO:0044763,single-organism cellular process|GO:0070887,cellular response to chemical stimulus|GO:0007409,axonogenesis|GO:0071346,cellular response to interferon-gamma|GO:0007155,cell adhesion|GO:0007154,cell communication|GO:0022008,neurogenesis|GO:0097485,neuron projection guidance|GO:0010033,response to organic substance|GO:0032990,cell part morphogenesis|GO:0040011,locomotion|GO:0044700,single organism signaling|GO:0048699,generation of neurons|GO:0048858,cell projection morphogenesis|GO:0007399,nervous system development|GO:0071345,cellular response to cytokine stimulus|GO:0050896,response to stimulus|GO:0048856,anatomical structure development|GO:0002376,immune system process|GO:0048812,neuron projection morphogenesis|GO:0048731,system development|GO:0044710,single-organism metabolic process|GO:0000165,MAPK cascade|GO:0044419,interspecies interaction between organisms|GO:0019058,viral life cycle|GO:0044093,positive regulation of molecular function|GO:0051701,interaction with host|GO:0051704,multi-organism process|GO:1901564,organonitrogen compound metabolic process|GO:0019538,protein metabolic process|GO:0046718,viral entry into host cell|GO:0006807,nitrogen compound metabolic process|GO:0035556,intracellular signal transduction|GO:0044267,cellular protein metabolic process|GO:0043547,positive regulation of GTPase activity|GO:0051345,positive regulation of hydrolase activity|GO:0023014,signal transduction by protein phosphorylation|GO:0065009,regulation of molecular function|GO:0043085,positive regulation of catalytic activity|GO:0050790,regulation of catalytic activity|GO:0043412,macromolecule modification|GO:0036211,protein modification process|GO:0008152,metabolic process|GO:0051336,regulation of hydrolase activity|GO:0016310,phosphorylation|GO:0030260,entry into host cell|GO:0043087,regulation of GTPase activity|GO:0043170,macromolecule metabolic process|GO:0051828,entry into other organism involved in symbiotic interaction|GO:0071704,organic substance metabolic process|GO:0006468,protein phosphorylation|GO:0006464,cellular protein modification process|GO:0044238,primary metabolic process|GO:0044260,cellular macromolecule metabolic process|GO:0044237,cellular metabolic process|GO:0006796,phosphate-containing compound metabolic process|GO:0044409,entry into host|GO:0016032,viral process|GO:0006793,phosphorus metabolic process|GO:0044403,symbiosis, encompassing mutualism through parasitism|GO:0051806,entry into cell of other organism involved in symbiotic interaction|GO:0009966,regulation of signal transduction|GO:0071526,semaphorin-plexin signaling pathway|GO:0048583,regulation of response to stimulus|GO:2001260,regulation of semaphorin-plexin signaling pathway|GO:0023051,regulation of signaling|GO:0010646,regulation of cell communication|GO:0071679,commissural neuron axon guidance	GO:0009897,external side of plasma membrane|GO:0043229,intracellular organelle|GO:0071944,cell periphery|GO:0098552,side of membrane|GO:0005622,intracellular|GO:0043227,membrane-bounded organelle|GO:0043226,organelle|GO:0031224,intrinsic component of membrane|GO:0031225,anchored component of membrane|GO:0005737,cytoplasm|GO:0031982,vesicle|GO:0016021,integral component of membrane|GO:0016020,membrane|GO:0031988,membrane-bounded vesicle|GO:0044431,Golgi apparatus part|GO:0044459,plasma membrane part|GO:0009986,cell surface|GO:0005794,Golgi apparatus|GO:0098588,bounding membrane of organelle|GO:0012505,endomembrane system|GO:0005886,plasma membrane|GO:1903561,extracellular vesicle|GO:0031090,organelle membrane|GO:0043230,extracellular organelle|GO:0043231,intracellular membrane-bounded organelle|GO:0070062,extracellular exosome|GO:0044464,cell part|GO:0005623,cell|GO:0000139,Golgi membrane|GO:0044446,intracellular organelle part|GO:0044444,cytoplasmic part|GO:0005576,extracellular region|GO:0044424,intracellular part|GO:0044425,membrane part|GO:0065010,extracellular membrane-bounded organelle|GO:0044421,extracellular region part|GO:0044422,organelle part|GO:0031984,organelle subcompartment|GO:0005829,cytosol|GO:0098791,Golgi subcompartment	GO:0005515,protein binding|GO:0042802,identical protein binding|GO:0005488,binding|GO:0005088,Ras guanyl-nucleotide exchange factor activity|GO:0005085,guanyl-nucleotide exchange factor activity|GO:0001618,virus receptor activity|GO:0060089,molecular transducer activity|GO:0004872,receptor activity|GO:0098772,molecular function regulator
MEP1A	GO:0044238,primary metabolic process|GO:0032501,multicellular organismal process|GO:0044707,single-multicellular organism process|GO:0071704,organic substance metabolic process|GO:0007586,digestion|GO:0043170,macromolecule metabolic process|GO:0019538,protein metabolic process|GO:0008152,metabolic process|GO:0006508,proteolysis|GO:0044699,single-organism process|GO:1901564,organonitrogen compound metabolic process|GO:0006807,nitrogen compound metabolic process	GO:0071944,cell periphery|GO:0031226,intrinsic component of plasma membrane|GO:0043226,organelle|GO:0031224,intrinsic component of membrane|GO:0031982,vesicle|GO:0043227,membrane-bounded organelle|GO:0016021,integral component of membrane|GO:0016020,membrane|GO:0031988,membrane-bounded vesicle|GO:0017090,meprin A complex|GO:0005615,extracellular space|GO:0044459,plasma membrane part|GO:0005887,integral component of plasma membrane|GO:0005886,plasma membrane|GO:0032991,macromolecular complex|GO:1903561,extracellular vesicle|GO:0070062,extracellular exosome|GO:0043234,protein complex|GO:0043230,extracellular organelle|GO:0044464,cell part|GO:0005623,cell|GO:0098796,membrane protein complex|GO:0005576,extracellular region|GO:0044425,membrane part|GO:0065010,extracellular membrane-bounded organelle|GO:0044421,extracellular region part	GO:0016787,hydrolase activity|GO:0004175,endopeptidase activity|GO:0004222,metalloendopeptidase activity|GO:0046914,transition metal ion binding|GO:0043169,cation binding|GO:0043167,ion binding|GO:0003824,catalytic activity|GO:0070011,peptidase activity, acting on L-amino acid peptides|GO:0008270,zinc ion binding|GO:0005515,protein binding|GO:0008233,peptidase activity|GO:0046872,metal ion binding|GO:0008237,metallopeptidase activity|GO:0005488,binding
NACC1	GO:0009892,negative regulation of metabolic process|GO:0080090,regulation of primary metabolic process|GO:0009890,negative regulation of biosynthetic process|GO:0031327,negative regulation of cellular biosynthetic process|GO:0031326,regulation of cellular biosynthetic process|GO:0031324,negative regulation of cellular metabolic process|GO:0031323,regulation of cellular metabolic process|GO:0090304,nucleic acid metabolic process|GO:0044249,cellular biosynthetic process|GO:0010629,negative regulation of gene expression|GO:0006807,nitrogen compound metabolic process|GO:0034645,cellular macromolecule biosynthetic process|GO:1901362,organic cyclic compound biosynthetic process|GO:0050789,regulation of biological process|GO:0097659,nucleic acid-templated transcription|GO:0032774,RNA biosynthetic process|GO:0006139,nucleobase-containing compound metabolic process|GO:0010605,negative regulation of macromolecule metabolic process|GO:0019222,regulation of metabolic process|GO:0044260,cellular macromolecule metabolic process|GO:0000122,negative regulation of transcription from RNA polymerase II promoter|GO:2000112,regulation of cellular macromolecule biosynthetic process|GO:2000113,negative regulation of cellular macromolecule biosynthetic process|GO:0060255,regulation of macromolecule metabolic process|GO:0010467,gene expression|GO:0010556,regulation of macromolecule biosynthetic process|GO:0065007,biological regulation|GO:1901360,organic cyclic compound metabolic process|GO:0006366,transcription from RNA polymerase II promoter|GO:0048519,negative regulation of biological process|GO:0010468,regulation of gene expression|GO:0018130,heterocycle biosynthetic process|GO:0045934,negative regulation of nucleobase-containing compound metabolic process|GO:1901576,organic substance biosynthetic process|GO:0019219,regulation of nucleobase-containing compound metabolic process|GO:0006725,cellular aromatic compound metabolic process|GO:0009987,cellular process|GO:0009889,regulation of biosynthetic process|GO:1903506,regulation of nucleic acid-templated transcription|GO:1903507,negative regulation of nucleic acid-templated transcription|GO:1902679,negative regulation of RNA biosynthetic process|GO:0071704,organic substance metabolic process|GO:0009058,biosynthetic process|GO:0009059,macromolecule biosynthetic process|GO:0045892,negative regulation of transcription, DNA-templated|GO:0051171,regulation of nitrogen compound metabolic process|GO:0051172,negative regulation of nitrogen compound metabolic process|GO:2001141,regulation of RNA biosynthetic process|GO:0034654,nucleobase-containing compound biosynthetic process|GO:0050794,regulation of cellular process|GO:0008152,metabolic process|GO:0046483,heterocycle metabolic process|GO:0016070,RNA metabolic process|GO:0044238,primary metabolic process|GO:0044271,cellular nitrogen compound biosynthetic process|GO:0051253,negative regulation of RNA metabolic process|GO:0051252,regulation of RNA metabolic process|GO:0034641,cellular nitrogen compound metabolic process|GO:0044237,cellular metabolic process|GO:0043170,macromolecule metabolic process|GO:0006355,regulation of transcription, DNA-templated|GO:0006357,regulation of transcription from RNA polymerase II promoter|GO:0006351,transcription, DNA-templated|GO:0019438,aromatic compound biosynthetic process|GO:0010558,negative regulation of macromolecule biosynthetic process|GO:0048523,negative regulation of cellular process	GO:0044446,intracellular organelle part|GO:0043231,intracellular membrane-bounded organelle|GO:0031981,nuclear lumen|GO:0043233,organelle lumen|GO:0005634,nucleus|GO:0044464,cell part|GO:0031974,membrane-enclosed lumen|GO:0005622,intracellular|GO:0005654,nucleoplasm|GO:0070013,intracellular organelle lumen|GO:0043229,intracellular organelle|GO:0044428,nuclear part|GO:0030054,cell junction|GO:0044424,intracellular part|GO:0005623,cell|GO:0043227,membrane-bounded organelle|GO:0043226,organelle|GO:0044422,organelle part	GO:0044212,transcription regulatory region DNA binding|GO:0001227,transcriptional repressor activity, RNA polymerase II transcription regulatory region sequence-specific binding|GO:0001071,nucleic acid binding transcription factor activity|GO:0043565,sequence-specific DNA binding|GO:0003700,transcription factor activity, sequence-specific DNA binding|GO:0001012,RNA polymerase II regulatory region DNA binding|GO:0003677,DNA binding|GO:0001067,regulatory region nucleic acid binding|GO:0000976,transcription regulatory region sequence-specific DNA binding|GO:0000977,RNA polymerase II regulatory region sequence-specific DNA binding|GO:0003676,nucleic acid binding|GO:0000975,regulatory region DNA binding|GO:0097159,organic cyclic compound binding|GO:1901363,heterocyclic compound binding|GO:0000981,RNA polymerase II transcription factor activity, sequence-specific DNA binding|GO:0005488,binding
SMYD1	GO:0006479,protein methylation|GO:0080090,regulation of primary metabolic process|GO:0019222,regulation of metabolic process|GO:0010831,positive regulation of myotube differentiation|GO:0072359,circulatory system development|GO:0072358,cardiovascular system development|GO:1901362,organic cyclic compound biosynthetic process|GO:0071840,cellular component organization or biogenesis|GO:0044710,single-organism metabolic process|GO:0010605,negative regulation of macromolecule metabolic process|GO:0048869,cellular developmental process|GO:0007519,skeletal muscle tissue development|GO:0018193,peptidyl-amino acid modification|GO:0048513,animal organ development|GO:0045661,regulation of myoblast differentiation|GO:0045663,positive regulation of myoblast differentiation|GO:0048518,positive regulation of biological process|GO:0048519,negative regulation of biological process|GO:0060255,regulation of macromolecule metabolic process|GO:2001141,regulation of RNA biosynthetic process|GO:0046483,heterocycle metabolic process|GO:0044707,single-multicellular organism process|GO:0018205,peptidyl-lysine modification|GO:0019538,protein metabolic process|GO:0019438,aromatic compound biosynthetic process|GO:0042692,muscle cell differentiation|GO:0016568,chromatin modification|GO:0016569,covalent chromatin modification|GO:0009892,negative regulation of metabolic process|GO:0009890,negative regulation of biosynthetic process|GO:0018022,peptidyl-lysine methylation|GO:0010629,negative regulation of gene expression|GO:0065007,biological regulation|GO:0050789,regulation of biological process|GO:0097659,nucleic acid-templated transcription|GO:0044267,cellular protein metabolic process|GO:0044260,cellular macromolecule metabolic process|GO:0016043,cellular component organization|GO:0016571,histone methylation|GO:0016570,histone modification|GO:0035914,skeletal muscle cell differentiation|GO:0032259,methylation|GO:0018130,heterocycle biosynthetic process|GO:0050793,regulation of developmental process|GO:0009889,regulation of biosynthetic process|GO:0007517,muscle organ development|GO:0050794,regulation of cellular process|GO:0043412,macromolecule modification|GO:0036211,protein modification process|GO:0043414,macromolecule methylation|GO:0008152,metabolic process|GO:0034654,nucleobase-containing compound biosynthetic process|GO:0016070,RNA metabolic process|GO:0044767,single-organism developmental process|GO:0044271,cellular nitrogen compound biosynthetic process|GO:0006355,regulation of transcription, DNA-templated|GO:0010556,regulation of macromolecule biosynthetic process|GO:0006351,transcription, DNA-templated|GO:0010558,negative regulation of macromolecule biosynthetic process|GO:0032774,RNA biosynthetic process|GO:0030154,cell differentiation|GO:0044249,cellular biosynthetic process|GO:0034641,cellular nitrogen compound metabolic process|GO:0034645,cellular macromolecule biosynthetic process|GO:0061061,muscle structure development|GO:0044699,single-organism process|GO:0006139,nucleobase-containing compound metabolic process|GO:0031327,negative regulation of cellular biosynthetic process|GO:0034968,histone lysine methylation|GO:0032502,developmental process|GO:0032501,multicellular organismal process|GO:0044238,primary metabolic process|GO:0014902,myotube differentiation|GO:0009987,cellular process|GO:0006725,cellular aromatic compound metabolic process|GO:1903506,regulation of nucleic acid-templated transcription|GO:1903507,negative regulation of nucleic acid-templated transcription|GO:0045597,positive regulation of cell differentiation|GO:0051153,regulation of striated muscle cell differentiation|GO:0045595,regulation of cell differentiation|GO:0045892,negative regulation of transcription, DNA-templated|GO:0010830,regulation of myotube differentiation|GO:0051155,positive regulation of striated muscle cell differentiation|GO:0008213,protein alkylation|GO:0006338,chromatin remodeling|GO:0051094,positive regulation of developmental process|GO:0051253,negative regulation of RNA metabolic process|GO:0051252,regulation of RNA metabolic process|GO:0043170,macromolecule metabolic process|GO:0006807,nitrogen compound metabolic process|GO:0048731,system development|GO:0043933,macromolecular complex subunit organization|GO:0031326,regulation of cellular biosynthetic process|GO:0031324,negative regulation of cellular metabolic process|GO:0031323,regulation of cellular metabolic process|GO:0090304,nucleic acid metabolic process|GO:0051149,positive regulation of muscle cell differentiation|GO:0014706,striated muscle tissue development|GO:0051147,regulation of muscle cell differentiation|GO:0051146,striated muscle cell differentiation|GO:0045445,myoblast differentiation|GO:0007275,multicellular organism development|GO:0009888,tissue development|GO:0006325,chromatin organization|GO:1901360,organic cyclic compound metabolic process|GO:2000112,regulation of cellular macromolecule biosynthetic process|GO:2000113,negative regulation of cellular macromolecule biosynthetic process|GO:0071704,organic substance metabolic process|GO:0010467,gene expression|GO:0010468,regulation of gene expression|GO:0045934,negative regulation of nucleobase-containing compound metabolic process|GO:1901576,organic substance biosynthetic process|GO:0019219,regulation of nucleobase-containing compound metabolic process|GO:0006464,cellular protein modification process|GO:1902679,negative regulation of RNA biosynthetic process|GO:0009058,biosynthetic process|GO:0009059,macromolecule biosynthetic process|GO:0044763,single-organism cellular process|GO:0051171,regulation of nitrogen compound metabolic process|GO:0051172,negative regulation of nitrogen compound metabolic process|GO:0006996,organelle organization|GO:0007507,heart development|GO:0051276,chromosome organization|GO:0048856,anatomical structure development|GO:0044237,cellular metabolic process|GO:1902589,single-organism organelle organization|GO:0060537,muscle tissue development|GO:0060538,skeletal muscle organ development|GO:0048523,negative regulation of cellular process|GO:0048522,positive regulation of cellular process|GO:1901564,organonitrogen compound metabolic process	GO:0005737,cytoplasm|GO:0043231,intracellular membrane-bounded organelle|GO:0005634,nucleus|GO:0044464,cell part|GO:0005623,cell|GO:0005622,intracellular|GO:0043229,intracellular organelle|GO:0044424,intracellular part|GO:0043227,membrane-bounded organelle|GO:0043226,organelle	GO:0018024,histone-lysine N-methyltransferase activity|GO:0042054,histone methyltransferase activity|GO:0016278,lysine N-methyltransferase activity|GO:0016279,protein-lysine N-methyltransferase activity|GO:0008276,protein methyltransferase activity|GO:0005488,binding|GO:0003676,nucleic acid binding|GO:0003677,DNA binding|GO:0008168,methyltransferase activity|GO:1901363,heterocyclic compound binding|GO:0043169,cation binding|GO:0003714,transcription corepressor activity|GO:0003712,transcription cofactor activity|GO:0043167,ion binding|GO:0016740,transferase activity|GO:0016741,transferase activity, transferring one-carbon groups|GO:0000989,transcription factor activity, transcription factor binding|GO:0000988,transcription factor activity, protein binding|GO:0046872,metal ion binding|GO:0008757,S-adenosylmethionine-dependent methyltransferase activity|GO:0003824,catalytic activity|GO:0008170,N-methyltransferase activity|GO:0097159,organic cyclic compound binding|GO:0005515,protein binding
SPSB4	GO:0023052,signaling|GO:0007165,signal transduction|GO:0035556,intracellular signal transduction|GO:0050789,regulation of biological process|GO:0044699,single-organism process|GO:0044267,cellular protein metabolic process|GO:0051716,cellular response to stimulus|GO:0044260,cellular macromolecule metabolic process|GO:0071704,organic substance metabolic process|GO:0070647,protein modification by small protein conjugation or removal|GO:0032446,protein modification by small protein conjugation|GO:0065007,biological regulation|GO:0044238,primary metabolic process|GO:0009987,cellular process|GO:0006464,cellular protein modification process|GO:0050794,regulation of cellular process|GO:0043412,macromolecule modification|GO:0036211,protein modification process|GO:0044763,single-organism cellular process|GO:0008152,metabolic process|GO:0007154,cell communication|GO:0044700,single organism signaling|GO:0019538,protein metabolic process|GO:0050896,response to stimulus|GO:0044237,cellular metabolic process|GO:0043170,macromolecule metabolic process|GO:0016567,protein ubiquitination|GO:0006807,nitrogen compound metabolic process|GO:0043687,post-translational protein modification|GO:1901564,organonitrogen compound metabolic process	GO:0005737,cytoplasm|GO:0044464,cell part|GO:0005623,cell|GO:0005622,intracellular|GO:0044424,intracellular part|GO:0005829,cytosol|GO:0044444,cytoplasmic part	GO:0005488,binding|GO:0005515,protein binding
NDUFA4	GO:0051234,establishment of localization|GO:1902600,hydrogen ion transmembrane transport|GO:0009141,nucleoside triphosphate metabolic process|GO:0009144,purine nucleoside triphosphate metabolic process|GO:0009167,purine ribonucleoside monophosphate metabolic process|GO:0034641,cellular nitrogen compound metabolic process|GO:0006807,nitrogen compound metabolic process|GO:0044281,small molecule metabolic process|GO:0009161,ribonucleoside monophosphate metabolic process|GO:0009123,nucleoside monophosphate metabolic process|GO:0006091,generation of precursor metabolites and energy|GO:0009126,purine nucleoside monophosphate metabolic process|GO:0009259,ribonucleotide metabolic process|GO:0044699,single-organism process|GO:0044237,cellular metabolic process|GO:0006139,nucleobase-containing compound metabolic process|GO:0044710,single-organism metabolic process|GO:0042775,mitochondrial ATP synthesis coupled electron transport|GO:0015992,proton transport|GO:0071704,organic substance metabolic process|GO:0009987,cellular process|GO:1901360,organic cyclic compound metabolic process|GO:0042278,purine nucleoside metabolic process|GO:0055085,transmembrane transport|GO:0098662,inorganic cation transmembrane transport|GO:0009199,ribonucleoside triphosphate metabolic process|GO:0022900,electron transport chain|GO:0055086,nucleobase-containing small molecule metabolic process|GO:0006810,transport|GO:0022904,respiratory electron transport chain|GO:1902578,single-organism localization|GO:0015672,monovalent inorganic cation transport|GO:0006119,oxidative phosphorylation|GO:0009205,purine ribonucleoside triphosphate metabolic process|GO:0006818,hydrogen transport|GO:0046034,ATP metabolic process|GO:0006811,ion transport|GO:0098660,inorganic ion transmembrane transport|GO:0006725,cellular aromatic compound metabolic process|GO:0009150,purine ribonucleotide metabolic process|GO:0072521,purine-containing compound metabolic process|GO:0034220,ion transmembrane transport|GO:0044765,single-organism transport|GO:0045333,cellular respiration|GO:0009116,nucleoside metabolic process|GO:0008152,metabolic process|GO:0051179,localization|GO:0009119,ribonucleoside metabolic process|GO:0046128,purine ribonucleoside metabolic process|GO:0042773,ATP synthesis coupled electron transport|GO:0055114,oxidation-reduction process|GO:0046483,heterocycle metabolic process|GO:0044238,primary metabolic process|GO:0098655,cation transmembrane transport|GO:1901564,organonitrogen compound metabolic process|GO:0015980,energy derivation by oxidation of organic compounds|GO:0016310,phosphorylation|GO:0044763,single-organism cellular process|GO:0019693,ribose phosphate metabolic process|GO:0006120,mitochondrial electron transport, NADH to ubiquinone|GO:0006163,purine nucleotide metabolic process|GO:1901657,glycosyl compound metabolic process|GO:0006796,phosphate-containing compound metabolic process|GO:0006793,phosphorus metabolic process|GO:0019637,organophosphate metabolic process|GO:0009117,nucleotide metabolic process|GO:0006812,cation transport|GO:0006753,nucleoside phosphate metabolic process|GO:1901135,carbohydrate derivative metabolic process|GO:0006123,mitochondrial electron transport, cytochrome c to oxygen	GO:0044464,cell part|GO:0031090,organelle membrane|GO:1990204,oxidoreductase complex|GO:0043229,intracellular organelle|GO:0070069,cytochrome complex|GO:0031988,membrane-bounded vesicle|GO:0043227,membrane-bounded organelle|GO:0043226,organelle|GO:0005737,cytoplasm|GO:0005751,mitochondrial respiratory chain complex IV|GO:0031982,vesicle|GO:0098796,membrane protein complex|GO:0031975,envelope|GO:0016020,membrane|GO:0098803,respiratory chain complex|GO:0044444,cytoplasmic part|GO:0005739,mitochondrion|GO:0098800,inner mitochondrial membrane protein complex|GO:0044455,mitochondrial membrane part|GO:0044429,mitochondrial part|GO:1902494,catalytic complex|GO:0031967,organelle envelope|GO:0031966,mitochondrial membrane|GO:0032991,macromolecular complex|GO:1903561,extracellular vesicle|GO:0070062,extracellular exosome|GO:0043234,protein complex|GO:0045271,respiratory chain complex I|GO:0043230,extracellular organelle|GO:0043231,intracellular membrane-bounded organelle|GO:0030964,NADH dehydrogenase complex|GO:0019866,organelle inner membrane|GO:0005623,cell|GO:0005622,intracellular|GO:0044446,intracellular organelle part|GO:0005743,mitochondrial inner membrane|GO:0005740,mitochondrial envelope|GO:0005576,extracellular region|GO:0005746,mitochondrial respiratory chain|GO:0005747,mitochondrial respiratory chain complex I|GO:0044424,intracellular part|GO:0044425,membrane part|GO:0070469,respiratory chain|GO:0045277,respiratory chain complex IV|GO:0065010,extracellular membrane-bounded organelle|GO:0044421,extracellular region part|GO:0044422,organelle part|GO:0098798,mitochondrial protein complex	GO:0044877,macromolecular complex binding|GO:0005488,binding|GO:0003954,NADH dehydrogenase activity|GO:0016491,oxidoreductase activity|GO:0015077,monovalent inorganic cation transmembrane transporter activity|GO:0050136,NADH dehydrogenase (quinone) activity|GO:0008137,NADH dehydrogenase (ubiquinone) activity|GO:0032403,protein complex binding|GO:0015002,heme-copper terminal oxidase activity|GO:0004129,cytochrome-c oxidase activity|GO:0005215,transporter activity|GO:0008324,cation transmembrane transporter activity|GO:0003824,catalytic activity|GO:0009055,electron carrier activity|GO:0022891,substrate-specific transmembrane transporter activity|GO:0022890,inorganic cation transmembrane transporter activity|GO:0022892,substrate-specific transporter activity|GO:0015075,ion transmembrane transporter activity|GO:0016655,oxidoreductase activity, acting on NAD(P)H, quinone or similar compound as acceptor|GO:0022857,transmembrane transporter activity|GO:0016676,oxidoreductase activity, acting on a heme group of donors, oxygen as acceptor|GO:0016651,oxidoreductase activity, acting on NAD(P)H|GO:0005515,protein binding|GO:0016675,oxidoreductase activity, acting on a heme group of donors|GO:0015078,hydrogen ion transmembrane transporter activity
HMGN3	GO:0090087,regulation of peptide transport|GO:0008104,protein localization|GO:0080090,regulation of primary metabolic process|GO:0019222,regulation of metabolic process|GO:0035773,insulin secretion involved in cellular response to glucose stimulus|GO:0051049,regulation of transport|GO:0048583,regulation of response to stimulus|GO:0032844,regulation of homeostatic process|GO:1901362,organic cyclic compound biosynthetic process|GO:0071840,cellular component organization or biogenesis|GO:0051716,cellular response to stimulus|GO:0010604,positive regulation of macromolecule metabolic process|GO:0010467,gene expression|GO:0046879,hormone secretion|GO:0071331,cellular response to hexose stimulus|GO:0071333,cellular response to glucose stimulus|GO:0048518,positive regulation of biological process|GO:0033036,macromolecule localization|GO:0019725,cellular homeostasis|GO:0060255,regulation of macromolecule metabolic process|GO:0045184,establishment of protein localization|GO:0090276,regulation of peptide hormone secretion|GO:0042221,response to chemical|GO:2001141,regulation of RNA biosynthetic process|GO:0010033,response to organic substance|GO:0046483,heterocycle metabolic process|GO:0044700,single organism signaling|GO:0034284,response to monosaccharide|GO:0071322,cellular response to carbohydrate stimulus|GO:0071326,cellular response to monosaccharide stimulus|GO:0048878,chemical homeostasis|GO:0019438,aromatic compound biosynthetic process|GO:0016568,chromatin modification|GO:0009893,positive regulation of metabolic process|GO:0009891,positive regulation of biosynthetic process|GO:0008152,metabolic process|GO:0032940,secretion by cell|GO:0010628,positive regulation of gene expression|GO:0051223,regulation of protein transport|GO:0050789,regulation of biological process|GO:0030072,peptide hormone secretion|GO:0030073,insulin secretion|GO:1901576,organic substance biosynthetic process|GO:0050708,regulation of protein secretion|GO:0044260,cellular macromolecule metabolic process|GO:0016043,cellular component organization|GO:0065007,biological regulation|GO:1901360,organic cyclic compound metabolic process|GO:0006366,transcription from RNA polymerase II promoter|GO:0065008,regulation of biological quality|GO:0018130,heterocycle biosynthetic process|GO:0070201,regulation of establishment of protein localization|GO:0009306,protein secretion|GO:0006810,transport|GO:0009889,regulation of biosynthetic process|GO:0050796,regulation of insulin secretion|GO:0050794,regulation of cellular process|GO:1901700,response to oxygen-containing compound|GO:0001678,cellular glucose homeostasis|GO:0034654,nucleobase-containing compound biosynthetic process|GO:0034641,cellular nitrogen compound metabolic process|GO:0061178,regulation of insulin secretion involved in cellular response to glucose stimulus|GO:0016070,RNA metabolic process|GO:0046903,secretion|GO:0044271,cellular nitrogen compound biosynthetic process|GO:0050896,response to stimulus|GO:0009058,biosynthetic process|GO:0006355,regulation of transcription, DNA-templated|GO:0010557,positive regulation of macromolecule biosynthetic process|GO:0010556,regulation of macromolecule biosynthetic process|GO:0006351,transcription, DNA-templated|GO:0051046,regulation of secretion|GO:0032774,RNA biosynthetic process|GO:0051649,establishment of localization in cell|GO:0044249,cellular biosynthetic process|GO:0015833,peptide transport|GO:0023052,signaling|GO:1903530,regulation of secretion by cell|GO:0070887,cellular response to chemical stimulus|GO:0023051,regulation of signaling|GO:0010646,regulation of cell communication|GO:0044699,single-organism process|GO:0032880,regulation of protein localization|GO:0006139,nucleobase-containing compound metabolic process|GO:0042886,amide transport|GO:0051234,establishment of localization|GO:1903508,positive regulation of nucleic acid-templated transcription|GO:0009746,response to hexose|GO:0044238,primary metabolic process|GO:0009987,cellular process|GO:0006725,cellular aromatic compound metabolic process|GO:1903506,regulation of nucleic acid-templated transcription|GO:0034645,cellular macromolecule biosynthetic process|GO:0046883,regulation of hormone secretion|GO:0045893,positive regulation of transcription, DNA-templated|GO:0055082,cellular chemical homeostasis|GO:0032879,regulation of localization|GO:0009743,response to carbohydrate|GO:0090304,nucleic acid metabolic process|GO:0051252,regulation of RNA metabolic process|GO:0051254,positive regulation of RNA metabolic process|GO:0043170,macromolecule metabolic process|GO:1902680,positive regulation of RNA biosynthetic process|GO:0006807,nitrogen compound metabolic process|GO:0045944,positive regulation of transcription from RNA polymerase II promoter|GO:0060341,regulation of cellular localization|GO:0031328,positive regulation of cellular biosynthetic process|GO:0043933,macromolecular complex subunit organization|GO:0031326,regulation of cellular biosynthetic process|GO:0031325,positive regulation of cellular metabolic process|GO:0031323,regulation of cellular metabolic process|GO:0042592,homeostatic process|GO:0042593,glucose homeostasis|GO:0009749,response to glucose|GO:0006325,chromatin organization|GO:2000112,regulation of cellular macromolecule biosynthetic process|GO:0033500,carbohydrate homeostasis|GO:0071705,nitrogen compound transport|GO:0071704,organic substance metabolic process|GO:0071310,cellular response to organic substance|GO:0006357,regulation of transcription from RNA polymerase II promoter|GO:0097659,nucleic acid-templated transcription|GO:0071702,organic substance transport|GO:0010468,regulation of gene expression|GO:0045935,positive regulation of nucleobase-containing compound metabolic process|GO:0019219,regulation of nucleobase-containing compound metabolic process|GO:0023061,signal release|GO:0010817,regulation of hormone levels|GO:0044765,single-organism transport|GO:0009059,macromolecule biosynthetic process|GO:0044763,single-organism cellular process|GO:0051171,regulation of nitrogen compound metabolic process|GO:0051173,positive regulation of nitrogen compound metabolic process|GO:0007267,cell-cell signaling|GO:0007154,cell communication|GO:0051179,localization|GO:1902578,single-organism localization|GO:0051641,cellular localization|GO:0006996,organelle organization|GO:1901701,cellular response to oxygen-containing compound|GO:0051276,chromosome organization|GO:0002790,peptide secretion|GO:0002791,regulation of peptide secretion|GO:0044237,cellular metabolic process|GO:0009914,hormone transport|GO:0015031,protein transport|GO:0048522,positive regulation of cellular process|GO:0016569,covalent chromatin	GO:0031974,membrane-enclosed lumen|GO:0043229,intracellular organelle|GO:0043228,non-membrane-bounded organelle|GO:0000785,chromatin|GO:0043227,membrane-bounded organelle|GO:0043226,organelle|GO:0005737,cytoplasm|GO:0031981,nuclear lumen|GO:0005634,nucleus|GO:0005654,nucleoplasm|GO:0005694,chromosome|GO:0032991,macromolecular complex|GO:0043231,intracellular membrane-bounded organelle|GO:0043232,intracellular non-membrane-bounded organelle|GO:0043233,organelle lumen|GO:0044464,cell part|GO:0005623,cell|GO:0005622,intracellular|GO:0044446,intracellular organelle part|GO:0070013,intracellular organelle lumen|GO:0044428,nuclear part|GO:0044424,intracellular part|GO:0044427,chromosomal part|GO:0044422,organelle part|GO:0005829,cytosol|GO:0044444,cytoplasmic part	GO:0043566,structure-specific DNA binding|GO:0005515,protein binding|GO:0046966,thyroid hormone receptor binding|GO:0097159,organic cyclic compound binding|GO:0008134,transcription factor binding|GO:0035257,nuclear hormone receptor binding|GO:0031491,nucleosome binding|GO:0031490,chromatin DNA binding|GO:0031492,nucleosomal DNA binding|GO:0044877,macromolecular complex binding|GO:0005488,binding|GO:0003676,nucleic acid binding|GO:0003677,DNA binding|GO:0032403,protein complex binding|GO:0051427,hormone receptor binding|GO:0005102,receptor binding|GO:0003682,chromatin binding|GO:1901363,heterocyclic compound binding

<table>
<tr><td></td><td></td><td></td><td></td></tr>
<tr><td>CHSY3</td><td>GO:0030206,chondroitin sulfate biosynthetic process|GO:0030204,chondroitin sulfate metabolic process|GO:0030203,glycosaminoglycan metabolic process|GO:0044237,cellular metabolic process|GO:1903510,mucopolysaccharide metabolic process|GO:0044249,cellular biosynthetic process|GO:0006807,nitrogen compound metabolic process|GO:0044281,small molecule metabolic process|GO:0034645,cellular macromolecule biosynthetic process|GO:0009100,glycoprotein metabolic process|GO:0009101,glycoprotein biosynthetic process|GO:0044699,single-organism process|GO:1901576,organic substance biosynthetic process|GO:0044710,single-organism metabolic process|GO:0044711,single-organism biosynthetic process|GO:0044260,cellular macromolecule metabolic process|GO:0071704,organic substance metabolic process|GO:0009987,cellular process|GO:0030166,proteoglycan biosynthetic process|GO:0009058,biosynthetic process|GO:0009059,macromolecule biosynthetic process|GO:0044763,single-organism cellular process|GO:0008152,metabolic process|GO:0043436,oxoacid metabolic process|GO:1901564,organonitrogen compound metabolic process|GO:0044238,primary metabolic process|GO:0005975,carbohydrate metabolic process|GO:1901566,organonitrogen compound biosynthetic process|GO:0006082,organic acid metabolic process|GO:0044272,sulfur compound biosynthetic process|GO:0019538,protein metabolic process|GO:0050650,chondroitin sulfate proteoglycan biosynthetic process|GO:0050654,chondroitin sulfate proteoglycan metabolic process|GO:0043170,macromolecule metabolic process|GO:1901137,carbohydrate derivative biosynthetic process|GO:0006029,proteoglycan metabolic process|GO:0006790,sulfur compound metabolic process|GO:0006022,aminoglycan metabolic process|GO:0006023,aminoglycan biosynthetic process|GO:1901135,carbohydrate derivative metabolic process|GO:0006024,glycosaminoglycan biosynthetic process</td><td>GO:0043229,intracellular organelle|GO:0005622,intracellular|GO:0043227,membrane-bounded organelle|GO:0043226,organelle|GO:0031224,intrinsic component of membrane|GO:0005737,cytoplasm|GO:0031985,Golgi cisterna|GO:0031984,organelle subcompartment|GO:0031090,organelle membrane|GO:0016021,integral component of membrane|GO:0016020,membrane|GO:0044431,Golgi apparatus part|GO:0005795,Golgi stack|GO:0005794,Golgi apparatus|GO:0098588,bounding membrane of organelle|GO:0012505,endomembrane system|GO:0032580,Golgi cisterna membrane|GO:0043231,intracellular membrane-bounded organelle|GO:0044464,cell part|GO:0005623,cell|GO:0000139,Golgi membrane|GO:0044446,intracellular organelle part|GO:0044444,cytoplasmic part|GO:0098791,Golgi subcompartment|GO:0044424,intracellular part|GO:0044425,membrane part|GO:0044422,organelle part</td><td>GO:0003824,catalytic activity|GO:0016740,transferase activity|GO:0016757,transferase activity, transferring glycosyl groups|GO:0016758,transferase activity, transferring hexosyl groups|GO:0008376,acetylgalactosaminyltransferase activity|GO:0008194,UDP-glycosyltransferase activity|GO:0050510,N-acetylgalactosaminyl-proteoglycan 3-beta-glucuronosyltransferase activity|GO:0015020,glucuronosyltransferase activity</td></tr>
<tr><td>C10orf105</td><td></td><td>GO:0044425,membrane part|GO:0016021,integral component of membrane|GO:0016020,membrane|GO:0031224,intrinsic component of membrane</td><td></td></tr>
<tr><td>CCDC64B</td><td></td><td></td><td>GO:0017137,Rab GTPase binding|GO:0019899,enzyme binding|GO:0017016,Ras GTPase binding|GO:0031267,small GTPase binding|GO:0051020,GTPase</td></tr>
<tr><td>FAH</td><td>GO:0009065,glutamine family amino acid catabolic process|GO:0019752,carboxylic acid metabolic process|GO:0009063,cellular amino acid catabolic process|GO:0009074,aromatic amino acid family catabolic process|GO:0034641,cellular nitrogen compound metabolic process|GO:0006807,nitrogen compound metabolic process|GO:0044281,small molecule metabolic process|GO:0044282,small molecule catabolic process|GO:0009064,glutamine family amino acid metabolic process|GO:0044699,single-organism process|GO:1901361,organic cyclic compound catabolic process|GO:0044712,single-organism catabolic process|GO:0044710,single-organism metabolic process|GO:0006520,cellular amino acid metabolic process|GO:0006527,arginine catabolic process|GO:0006525,arginine metabolic process|GO:0071704,organic substance metabolic process|GO:1901360,organic cyclic compound metabolic process|GO:1901605,alpha-amino acid metabolic process|GO:1901606,alpha-amino acid catabolic process|GO:0009987,cellular process|GO:0044238,primary metabolic process|GO:1902222,erythrose 4-phosphate/phosphoenolpyruvate family amino acid catabolic process|GO:1902221,erythrose 4-phosphate/phosphoenolpyruvate family amino acid metabolic process|GO:0006725,cellular aromatic compound metabolic process|GO:1901575,organic substance catabolic process|GO:0044763,single-organism cellular process|GO:0008152,metabolic process|GO:0043436,oxoacid metabolic process|GO:0009056,catabolic process|GO:0009072,aromatic amino acid family metabolic process|GO:1901565,organonitrogen compound catabolic process|GO:0006570,tyrosine metabolic process|GO:0044248,cellular catabolic process|GO:0006572,tyrosine catabolic process|GO:1901564,organonitrogen compound metabolic process|GO:0006082,organic acid metabolic process|GO:0006558,L-phenylalanine metabolic process|GO:0006559,L-phenylalanine catabolic process|GO:0046395,carboxylic acid catabolic process|GO:0016054,organic acid catabolic process|GO:0044237,cellular metabolic process|GO:0019439,aromatic compound catabolic process</td><td>GO:0005737,cytoplasm|GO:0005622,intracellular|GO:0043230,extracellular organelle|GO:0031982,vesicle|GO:0044421,extracellular region part|GO:0043227,membrane-bounded organelle|GO:0005829,cytosol|GO:0044464,cell part|GO:0005623,cell|GO:0031988,membrane-bounded vesicle|GO:0044444,cytoplasmic part|GO:0005576,extracellular region|GO:0044424,intracellular part|GO:1903561,extracellular vesicle|GO:0065010,extracellular membrane-bounded organelle|GO:0043226,organelle|GO:0070062,extracellular exosome</td><td>GO:0016823,hydrolase activity, acting on acid carbon-carbon bonds, in ketonic substances|GO:0016787,hydrolase activity|GO:0016822,hydrolase activity, acting on acid carbon-carbon bonds|GO:0043169,cation binding|GO:0043167,ion binding|GO:0004334,fumarylacetoacetase activity|GO:0003824,catalytic activity|GO:0005488,binding|GO:0005515,protein binding|GO:0046872,metal ion binding</td></tr>
<tr><td>TIFAB</td><td>GO:0007610,behavior|GO:0007611,learning or memory|GO:0001501,skeletal system development|GO:0003012,muscle system process|GO:0007165,signal transduction|GO:0021603,cranial nerve formation|GO:0021602,cranial nerve morphogenesis|GO:0007517,muscle organ development|GO:0050885,neuromuscular process controlling balance|GO:0000003,reproduction|GO:0009612,response to mechanical stimulus|GO:0048513,animal organ development|GO:0048806,genitalia development|GO:0098583,learned vocalization behavior|GO:0006936,muscle contraction|GO:0014821,phasic smooth muscle contraction|GO:0006939,smooth muscle contraction|GO:0003006,developmental process involved in reproduction|GO:0003002,regionalization|GO:0003008,system process|GO:0044700,single organism signaling|GO:0044702,single organism reproductive process|GO:0009605,response to external stimulus|GO:0044707,single-multicellular organism process|GO:0021648,vestibulocochlear nerve morphogenesis|GO:0044708,single-organism behavior|GO:0090075,relaxation of muscle|GO:1901078,negative regulation of relaxation of muscle|GO:0090596,sensory organ morphogenesis|GO:1901077,regulation of relaxation of muscle|GO:0048562,embryonic organ morphogenesis|GO:0022600,digestive system process|GO:0007638,mechanosensory behavior|GO:0098598,learned vocalization behavior or vocal learning|GO:0097094,craniofacial suture morphogenesis|GO:0061061,muscle structure development|GO:0000578,embryonic axis specification|GO:0031223,auditory behavior|GO:0035556,intracellular signal transduction|GO:0050789,regulation of biological process|GO:0090102,cochlea development|GO:0090103,cochlea morphogenesis|GO:0009948,anterior/posterior axis specification|GO:0065007,biological regulation|GO:0048646,anatomical structure formation involved in morphogenesis|GO:0048705,skeletal system morphogenesis|GO:0009887,animal organ morphogenesis|GO:0009880,embryonic pattern specification|GO:0050793,regulation of developmental process|GO:1904888,cranial skeletal system development|GO:0051716,cellular response to stimulus|GO:0050794,regulation of cellular process|GO:0051239,regulation of multicellular organismal process|GO:0021545,cranial nerve development|GO:0007351,tripartite regional subdivision|GO:0007350,blastoderm segmentation|GO:0007356,thorax and anterior abdomen determination|GO:0007354,zygotic determination of anterior/posterior axis, embryo|GO:0007548,sex differentiation|GO:0009952,anterior/posterior pattern specification|GO:0007423,sensory organ development|GO:0022414,reproductive process|GO:0050896,response to stimulus|GO:0050890,cognition|GO:0030432,peristalsis|GO:0048634,regulation of muscle organ development|GO:0009790,embryo development|GO:0023052,signaling|GO:0021559,trigeminal nerve development|GO:0009798,axis specification|GO:0009653,anatomical structure morphogenesis|GO:0021650,vestibulocochlear nerve formation|GO:0044699,single-organism process|GO:0044057,regulation of system process|GO:0051241,negative regulation of multicellular organismal process|GO:0048568,embryonic organ development|GO:0010996,response to auditory stimulus|GO:0032502,developmental process|GO:0032501,multicellular organismal process|GO:0048608,reproductive structure development|GO:0050877,neurological system process|GO:0009987,cellular process|GO:0021562,vestibulocochlear nerve development|GO:0048519,negative regulation of biological process|GO:0021675,nerve development|GO:0060021,palate development|GO:0048839,inner ear development|GO:0007249,I-kappaB kinase/NF-kappaB signaling|GO:0048731,system development|GO:0061458,reproductive system development|GO:0060349,bone morphogenesis|GO:0060348,bone development|GO:0050905,neuromuscular process|GO:0035282,segmentation|GO:0007275,multicellular organism development|GO:0007389,pattern specification process|GO:0043583,ear development|GO:0071626,mastication|GO:0071625,vocalization behavior|GO:0048598,embryonic morphogenesis|GO:0007586,digestion|GO:0044767,single-organism developmental process|GO:0008595,anterior/posterior axis specification, embryo|GO:0090257,regulation of muscle system process|GO:0035112,genitalia morphogenesis|GO:0007154,cell communication|GO:0042471,ear morphogenesis|GO:0009628,response to abiotic stimulus|GO:0042472,inner ear morphogenesis|GO:0007399,nervous system development|GO:0048856,anatomical structure development|GO:2000026,regulation of multicellular organismal development</td><td>GO:0044464,cell part|GO:0005623,cell|GO:0005622,intracellular</td><td></td></tr>
<tr><td>RP11</td><td></td><td></td><td></td></tr>
<tr><td>C10orf91</td><td></td><td></td><td></td></tr>
<tr><td>GNG13</td><td>GO:0051716,cellular response to stimulus|GO:0033762,response to glucagon|GO:0050896,response to stimulus|GO:0023052,signaling|GO:0007165,signal transduction|GO:0070887,cellular response to chemical stimulus|GO:0042221,response to chemical|GO:0006091,generation of precursor metabolites and energy|GO:0050909,sensory perception of taste|GO:0050789,regulation of biological process|GO:0044699,single-organism process|GO:0009719,response to endogenous stimulus|GO:0044710,single-organism metabolic process|GO:0071375,cellular response to peptide hormone stimulus|GO:0071377,cellular response to glucagon stimulus|GO:0071417,cellular response to organonitrogen compound|GO:0071310,cellular response to organic substance|GO:0065007,biological regulation|GO:0006112,energy reserve metabolic process|GO:0007186,G-protein coupled receptor signaling pathway|GO:0071495,cellular response to endogenous stimulus|GO:0032501,multicellular organismal process|GO:1901701,cellular response to oxygen-containing compound|GO:0050877,neurological system process|GO:0007606,sensory perception of chemical stimulus|GO:0007600,sensory perception|GO:0050794,regulation of cellular process|GO:0032870,cellular response to hormone stimulus|GO:0044763,single-organism cellular process|GO:0008152,metabolic process|GO:0007154,cell communication|GO:0043434,response to peptide hormone|GO:0010033,response to organic substance|GO:0055114,oxidation-reduction process|GO:0003008,system process|GO:1901700,response to oxygen-containing compound|GO:0044700,single organism signaling|GO:0015980,energy derivation by oxidation of organic compounds|GO:0044707,single-multicellular organism process|GO:0007200,phospholipase C-activating G-protein coupled receptor signaling pathway|GO:0010243,response to organonitrogen compound|GO:1901699,cellular response to nitrogen compound|GO:0044237,cellular metabolic process|GO:1901652,response to peptide|GO:1901653,cellular response to peptide|GO:0009725,response to hormone|GO:0009987,cellular process|GO:1901698,response to nitrogen compound|GO:0044281,small molecule metabolic process</td><td>GO:0019897,extrinsic component of plasma membrane|GO:0009898,cytoplasmic side of plasma membrane|GO:0019898,extrinsic component of membrane|GO:0071944,cell periphery|GO:0098552,side of membrane|GO:0005834,heterotrimeric G-protein complex|GO:0016020,membrane|GO:0097458,neuron part|GO:0043005,neuron projection|GO:0044459,plasma membrane part|GO:1902494,catalytic complex|GO:0098562,cytoplasmic side of membrane|GO:0030425,dendrite|GO:0005886,plasma membrane|GO:0042995,cell projection|GO:0031234,extrinsic component of cytoplasmic side of plasma membrane|GO:0043234,protein complex|GO:0032991,macromolecular complex|GO:0044464,cell part|GO:0005623,cell|GO:0005622,intracellular|GO:0098797,plasma membrane protein complex|GO:0098796,membrane protein complex|GO:0044424,intracellular part|GO:0044425,membrane</td><td>GO:0031681,G-protein beta-subunit binding|GO:0004871,signal transducer activity|GO:0060089,molecular transducer activity|GO:0005488,binding|GO:0005515,protein binding|GO:0016787,hydrolase activity|GO:0016818,hydrolase activity, acting on acid anhydrides, in phosphorus-containing anhydrides|GO:0003824,catalytic activity|GO:0003924,GTPase activity|GO:0017111,nucleoside-triphosphatase activity|GO:0016817,hydrolase activity, acting on acid anhydrides|GO:0016462,pyrophosphatase activity</td></tr>
<tr><td>CXorf64</td><td></td><td></td><td></td></tr>
<tr><td>PATE4</td><td>GO:0044700,single organism signaling|GO:0009987,cellular process|GO:0050896,response to stimulus|GO:0007268,chemical synaptic transmission|GO:0044763,single-organism cellular process|GO:0009611,response to wounding|GO:0050804,modulation of synaptic transmission|GO:0006950,response to stress|GO:0065007,biological regulation|GO:0023052,signaling|GO:0044699,single-organism process|GO:0023051,regulation of signaling|GO:0007267,cell-cell signaling|GO:0007154,cell communication|GO:0010646,regulation of cell communication|GO:0050789,regulation of biological process|GO:0050794,regulation of cellular process|GO:0048583,regulation of response to stimulus|GO:0007165,signal transduction|GO:0051716,cellular response to stimulus|GO:0009966,regulation of signal transduction|GO:0065009,regulation of molecular function|GO:0010469,regulation of receptor activity|GO:0098916,anterograde trans-synaptic signaling|GO:0099601,regulation of neurotransmitter receptor activity|GO:0099536,synaptic signaling|GO:0099537,trans-synaptic signaling</td><td>GO:0043229,intracellular organelle|GO:0001669,acrosomal vesicle|GO:0043227,membrane-bounded organelle|GO:0043226,organelle|GO:0005737,cytoplasm|GO:0031982,vesicle|GO:0016023,cytoplasmic, membrane-bounded vesicle|GO:0031410,cytoplasmic vesicle|GO:0097223,sperm part|GO:0031988,membrane-bounded vesicle|GO:0030141,secretory granule|GO:0012505,endomembrane system|GO:0005615,extracellular space|GO:0043231,intracellular membrane-bounded organelle|GO:0044464,cell part|GO:0005623,cell|GO:0005622,intracellular|GO:0044444,cytoplasmic part|GO:0005576,extracellular region|GO:0044424,intracellular part|GO:0044421,extracellular region part|GO:0099503,secretory vesicle|GO:0097708,intracellular vesicle</td><td>GO:0099602,neurotransmitter receptor regulator activity|GO:0098772,molecular function regulator|GO:0030548,acetylcholine receptor regulator activity|GO:0030545,receptor regulator activity</td></tr>
<tr><td>METTL5</td><td>GO:0032259,methylation|GO:0008152,metabolic process</td><td></td><td>GO:0097159,organic cyclic compound binding|GO:0003824,catalytic activity|GO:0016740,transferase activity|GO:0016741,transferase activity, transferring one-carbon groups|GO:0005488,binding|GO:0003676,nucleic acid binding|GO:0008168,methyltransferase</td></tr>
<tr><td>MTFMT</td><td>GO:0044238,primary metabolic process|GO:0009451,RNA modification|GO:0090304,nucleic acid metabolic process|GO:0044249,cellular biosynthetic process|GO:0034641,cellular nitrogen compound metabolic process|GO:0006807,nitrogen compound metabolic process|GO:0034645,cellular macromolecule biosynthetic process|GO:0034660,ncRNA metabolic process|GO:0071840,cellular component organization or biogenesis|GO:0034470,ncRNA processing|GO:0006139,nucleobase-containing compound metabolic process|GO:0044710,single-organism metabolic process|GO:0044711,single-organism biosynthetic process|GO:0044260,cellular macromolecule metabolic process|GO:1901360,organic cyclic compound metabolic process|GO:0043043,peptide biosynthetic process|GO:0016043,cellular component organization|GO:0071704,organic substance metabolic process|GO:0010467,gene expression|GO:0007005,mitochondrion organization|GO:0006400,tRNA modification|GO:0044267,cellular protein metabolic process|GO:0032543,mitochondrial translation|GO:0016070,RNA metabolic process|GO:0070124,mitochondrial translational initiation|GO:0008033,tRNA processing|GO:0043604,amide biosynthetic process|GO:0009987,cellular process|GO:0006725,cellular aromatic compound metabolic process|GO:0006464,cellular protein modification process|GO:0071951,conversion of methionyl-tRNA to N-formyl-methionyl-tRNA|GO:0043412,macromolecule modification|GO:0036211,protein modification process|GO:0044763,single-organism cellular process|GO:0008152,metabolic process|GO:1901564,organonitrogen compound metabolic process|GO:0046483,heterocycle metabolic process|GO:0006996,organelle organization|GO:0019988,charged-tRNA amino acid modification|GO:0044699,single-organism process|GO:0044271,cellular nitrogen compound biosynthetic process|GO:1901566,organonitrogen compound biosynthetic process|GO:0043603,cellular amide metabolic process|GO:0019538,protein metabolic process|GO:0009058,biosynthetic process|GO:0044237,cellular metabolic process|GO:0043170,macromolecule metabolic process|GO:0006399,tRNA metabolic process|GO:1902589,single-organism organelle organization|GO:0009059,macromolecule biosynthetic process|GO:1901576,organic substance biosynthetic process|GO:0006518,peptide metabolic process|GO:0006413,translational</td><td>GO:0005737,cytoplasm|GO:0043231,intracellular membrane-bounded organelle|GO:0044464,cell part|GO:0043229,intracellular organelle|GO:0005739,mitochondrion|GO:0005622,intracellular|GO:0044444,cytoplasmic part|GO:0005623,cell|GO:0044424,intracellular part|GO:0043227,membrane-bounded organelle|GO:0043226,organelle</td><td>GO:0004479,methionyl-tRNA formyltransferase activity|GO:0016742,hydroxymethyl-, formyl- and related transferase activity|GO:0003824,catalytic activity|GO:0016740,transferase activity|GO:0016741,transferase activity, transferring one-carbon groups</td></tr>
<tr><td>SPESP1</td><td>GO:0044801,single-organism membrane fusion|GO:0019953,sexual reproduction|GO:0061025,membrane fusion|GO:0061024,membrane organization|GO:0032940,secretion by cell|GO:0007275,multicellular organism development|GO:0044699,single-organism process|GO:0007340,acrosome reaction|GO:0000003,reproduction|GO:0007342,fusion of sperm to egg plasma membrane|GO:0006887,exocytosis|GO:0016043,cellular component organization|GO:0071840,cellular component organization or biogenesis|GO:0006810,transport|GO:0051704,multi-organism process|GO:0017156,calcium ion regulated exocytosis|GO:0032502,developmental process|GO:0032501,multicellular organismal process|GO:0032504,multicellular organism reproduction|GO:0009987,cellular process|GO:0044703,multi-organism reproductive process|GO:0044767,single-organism developmental process|GO:0022414,reproductive process|GO:0044765,single-organism transport|GO:0044763,single-organism cellular process|GO:0007338,single fertilization|GO:0022412,cellular process involved in reproduction in multicellular organism|GO:0045026,plasma membrane fusion|GO:0051234,establishment of localization|GO:0051179,localization|GO:1902578,single-organism localization|GO:0051641,cellular localization|GO:0046903,secretion|GO:0044702,single organism reproductive process|GO:0016192,vesicle-mediated transport|GO:0044707,single-multicellular organism process|GO:0009566,fertilization|GO:0044802,single-organism membrane organization|GO:0051649,establishment of localization in cell|GO:0048856,anatomical structure development|GO:0008037,cell recognition|GO:0009988,cell-cell recognition|GO:0035036,sperm-egg recognition</td><td>GO:0005737,cytoplasm|GO:0031982,vesicle|GO:0016023,cytoplasmic, membrane-bounded vesicle|GO:0030141,secretory granule|GO:0044464,cell part|GO:0044444,cytoplasmic part|GO:0005623,cell|GO:0031988,membrane-bounded vesicle|GO:0097223,sperm part|GO:0043229,intracellular organelle|GO:0001669,acrosomal vesicle|GO:0012505,endomembrane system|GO:0031410,cytoplasmic vesicle|GO:0044424,intracellular part|GO:0005622,intracellular|GO:0043227,membrane-bounded organelle|GO:0043226,organelle|GO:0043231,intracellular membrane-bounded organelle|GO:0099503,secretory vesicle|GO:0097708,intracellular vesicle</td><td></td></tr>
<tr><td>SPINK8</td><td>GO:0009892,negative regulation of metabolic process|GO:0080090,regulation of primary metabolic process|GO:0019222,regulation of metabolic process|GO:0031324,negative regulation of cellular metabolic process|GO:0031323,regulation of cellular metabolic process|GO:0043086,negative regulation of catalytic activity|GO:0044267,cellular protein metabolic process|GO:0051248,negative regulation of protein metabolic process|GO:0010605,negative regulation of macromolecule metabolic process|GO:0051346,negative regulation of hydrolase activity|GO:0044260,cellular macromolecule metabolic process|GO:0071704,organic substance metabolic process|GO:0051246,regulation of protein metabolic process|GO:0050789,regulation of biological process|GO:0060255,regulation of macromolecule metabolic process|GO:0010466,negative regulation of peptidase activity|GO:0065007,biological regulation|GO:0044092,negative regulation of molecular function|GO:0048519,negative regulation of biological process|GO:0065009,regulation of molecular function|GO:0009987,cellular process|GO:0052547,regulation of peptidase activity|GO:0052548,regulation of endopeptidase activity|GO:0050790,regulation of catalytic activity|GO:0050794,regulation of cellular process|GO:0030162,regulation of proteolysis|GO:0008152,metabolic process|GO:0006508,proteolysis|GO:0010951,negative regulation of endopeptidase activity|GO:0051336,regulation of hydrolase activity|GO:0044238,primary metabolic process|GO:0032269,negative regulation of cellular protein metabolic process|GO:0032268,regulation of cellular protein metabolic process|GO:0019538,protein metabolic process|GO:0044237,cellular metabolic process|GO:0043170,macromolecule metabolic process|GO:0045861,negative regulation of proteolysis|GO:0048523,negative regulation of cellular process|GO:0051049,regulation of transport|GO:0019953,sexual reproduction|GO:0051704,multi-organism process|GO:0060046,regulation of acrosome reaction|GO:0006807,nitrogen compound metabolic process|GO:1903530,regulation of secretion by cell|GO:0051641,cellular localization|GO:0051234,establishment of localization|GO:0051179,localization|GO:0045055,regulated exocytosis|GO:0044699,single-organism process|GO:0044765,single-organism transport|GO:1900003,regulation of serine-type endopeptidase activity|GO:1900004,negative regulation of serine-type endopeptidase activity|GO:0060341,regulation of cellular localization|GO:0006887,exocytosis|GO:0017158,regulation of calcium ion-dependent exocytosis|GO:1902578,single-organism localization|GO:0006810,transport|GO:1903305,regulation of regulated secretory pathway|GO:0017156,calcium ion regulated exocytosis|GO:0017157,regulation of exocytosis|GO:2000241,regulation of reproductive process|GO:0051046,regulation of secretion|GO:0044703,multi-organism reproductive process|GO:0060627,regulation of vesicle-mediated transport|GO:0022414,reproductive process|GO:0043900,regulation of multi-organism process|GO:0044702,single organism reproductive process|GO:0007338,single fertilization|GO:1902571,regulation of serine-type peptidase activity|GO:0032879,regulation of localization|GO:0000003,reproduction|GO:1901564,organonitrogen compound metabolic process|GO:0016192,vesicle-mediated transport|GO:0046903,secretion|GO:0009566,fertilization|GO:0032940,secretion by cell|GO:0044763,single-organism cellular process|GO:0007340,acrosome reaction|GO:1902572,negative regulation of serine-type peptidase activity</td><td>GO:0005576,extracellular region|GO:0043229,intracellular organelle|GO:0001669,acrosomal vesicle|GO:0043227,membrane-bounded organelle|GO:0043226,organelle|GO:0005737,cytoplasm|GO:0005615,extracellular space|GO:0016023,cytoplasmic, membrane-bounded vesicle|GO:0031410,cytoplasmic vesicle|GO:0097223,sperm part|GO:0031988,membrane-bounded vesicle|GO:0099503,secretory vesicle|GO:0030141,secretory granule|GO:0012505,endomembrane system|GO:0031982,vesicle|GO:0043231,intracellular membrane-bounded organelle|GO:0044464,cell part|GO:0005623,cell|GO:0005622,intracellular|GO:0044444,cytoplasmic part|GO:0044424,intracellular part|GO:0044421,extracellular region part|GO:0097708,intracellular vesicle</td><td>GO:0004866,endopeptidase inhibitor activity|GO:0061135,endopeptidase regulator activity|GO:0061134,peptidase regulator activity|GO:0030414,peptidase inhibitor activity|GO:0004867,serine-type endopeptidase inhibitor activity|GO:0004857,enzyme inhibitor activity|GO:0098772,molecular function regulator|GO:0030234,enzyme regulator activity</td></tr>
<tr><td>ACTG2</td><td>GO:0009893,positive regulation of metabolic process|GO:0019222,regulation of metabolic process|GO:0003012,muscle system process|GO:0010628,positive regulation of gene expression|GO:0072132,mesenchyme morphogenesis|GO:0009653,anatomical structure morphogenesis|GO:0007275,multicellular organism development|GO:0044699,single-organism process|GO:0010604,positive regulation of macromolecule metabolic process|GO:0050789,regulation of biological process|GO:0071704,organic substance metabolic process|GO:0010467,gene expression|GO:0048513,animal organ development|GO:0065007,biological regulation|GO:0048729,tissue morphogenesis|GO:0048518,positive regulation of biological process|GO:0090130,tissue migration|GO:0010468,regulation of gene expression|GO:0032502,developmental process|GO:0009887,animal organ morphogenesis|GO:0032501,multicellular organismal process|GO:0006936,muscle contraction|GO:0060255,regulation of macromolecule metabolic process|GO:0009888,tissue development|GO:0044767,single-organism developmental process|GO:0008152,metabolic process|GO:0060485,mesenchyme development|GO:0090131,mesenchyme migration|GO:0003008,system process|GO:0044707,single-multicellular organism process|GO:0048856,anatomical structure development|GO:0043170,macromolecule metabolic process|GO:0048731,system development</td><td>GO:0015629,actin cytoskeleton|GO:0030175,filopodium|GO:0043229,intracellular organelle|GO:0043228,non-membrane-bounded organelle|GO:0044430,cytoskeletal part|GO:0043227,membrane-bounded organelle|GO:0043226,organelle|GO:0005856,cytoskeleton|GO:0042995,cell projection|GO:0032982,myosin filament|GO:0031982,vesicle|GO:0005615,extracellular space|GO:0072562,blood microparticle|GO:0031988,membrane-bounded vesicle|GO:0016459,myosin complex|GO:0005576,extracellular region|GO:0005737,cytoplasm|GO:0030027,lamellipodium|GO:0044297,cell body|GO:0031252,cell leading edge|GO:0032991,macromolecular complex|GO:1903561,extracellular vesicle|GO:0070062,extracellular exosome|GO:0043234,protein complex|GO:0043230,extracellular organelle|GO:0043232,intracellular non-membrane-bounded organelle|GO:0005829,cytosol|GO:0044464,cell part|GO:0005623,cell|GO:0005622,intracellular|GO:0044446,intracellular organelle part|GO:0044444,cytoplasmic part|GO:0071944,cell periphery|GO:0044424,intracellular part|GO:0065010,extracellular membrane-bounded organelle|GO:0044421,extracellular region part|GO:0044422,organelle part|GO:0098858,actin-based cell projection</td><td>GO:0043168,anion binding|GO:0005488,binding|GO:0030554,adenyl nucleotide binding|GO:0035639,purine ribonucleoside triphosphate binding|GO:0097367,carbohydrate derivative binding|GO:1901363,heterocyclic compound binding|GO:1901265,nucleoside phosphate binding|GO:0000166,nucleotide binding|GO:0036094,small molecule binding|GO:0032553,ribonucleotide binding|GO:0032559,adenyl ribonucleotide binding|GO:0005524,ATP binding|GO:0032549,ribonucleoside binding|GO:0032555,purine ribonucleotide binding|GO:0017076,purine nucleotide binding|GO:0043167,ion binding|GO:0097159,organic cyclic compound binding|GO:0032550,purine ribonucleoside binding|GO:0001883,purine nucleoside binding|GO:0001882,nucleoside binding</td></tr>
<tr><td>DCD</td><td>GO:0042742,defense response to bacterium|GO:0043207,response to external biotic stimulus|GO:0044364,disruption of cells of other organism|GO:0009617,response to bacterium|GO:0071704,organic substance metabolic process|GO:0065007,biological regulation|GO:0006508,proteolysis|GO:0001906,cell killing|GO:0065008,regulation of biological quality|GO:0006952,defense response|GO:0050832,defense response to fungus|GO:0006950,response to stress|GO:0098542,defense response to other organism|GO:0008152,metabolic process|GO:0051707,response to other organism|GO:0051704,multi-organism process|GO:0044238,primary metabolic process|GO:0009607,response to biotic stimulus|GO:0009605,response to external stimulus|GO:0019538,protein metabolic process|GO:0009620,response to fungus|GO:0050896,response to stimulus|GO:0031640,killing of cells of other organism|GO:0043170,macromolecule metabolic process|GO:0035821,modification of morphology or physiology of other organism|GO:0019730,antimicrobial humoral response|GO:0006807,nitrogen compound metabolic process|GO:0050829,defense response to Gram-negative bacterium|GO:0044419,interspecies interaction between organisms|GO:0050830,defense response to Gram-positive bacterium|GO:0006955,immune response|GO:0006959,humoral immune response|GO:1901564,organonitrogen compound metabolic process|GO:0002376,immune system process</td><td>GO:0043230,extracellular organelle|GO:0031982,vesicle|GO:0044421,extracellular region part|GO:0043227,membrane-bounded organelle|GO:0031988,membrane-bounded vesicle|GO:0005576,extracellular region|GO:1903561,extracellular vesicle|GO:0065010,extracellular membrane-bounded organelle|GO:0043226,organelle|GO:0070062,extracellular exosome|GO:0005615,extracellular space|GO:0031012,extracellular matrix</td><td>GO:0016787,hydrolase activity|GO:0005515,protein binding|GO:0097159,organic cyclic compound binding|GO:0044822,poly(A) RNA binding|GO:0003824,catalytic activity|GO:0003723,RNA binding|GO:0003676,nucleic acid binding|GO:0008233,peptidase activity|GO:1901363,heterocyclic compound binding|GO:0005488,binding</td></tr>
<tr><td>CYP3A43</td><td>GO:0044710,single-organism metabolic process|GO:0008152,metabolic process|GO:0009410,response to xenobiotic stimulus|GO:0009987,cellular process|GO:0055114,oxidation-reduction process|GO:0051716,cellular response to stimulus|GO:0044237,cellular metabolic process|GO:0006805,xenobiotic metabolic process|GO:0071466,cellular response to xenobiotic stimulus|GO:0044281,small molecule metabolic process|GO:0070887,cellular response to chemical stimulus|GO:0042221,response to chemical|GO:0050896,response to stimulus|GO:0044699,single-organism process</td><td>GO:0005783,endoplasmic reticulum|GO:0005789,endoplasmic reticulum membrane|GO:0042175,nuclear outer membrane-endoplasmic reticulum membrane network|GO:0043229,intracellular organelle|GO:0043227,membrane-bounded organelle|GO:0043226,organelle|GO:0005737,cytoplasm|GO:0044446,intracellular organelle part|GO:0031090,organelle membrane|GO:0016020,membrane|GO:0044432,endoplasmic reticulum part|GO:0098588,bounding membrane of organelle|GO:0012505,endomembrane system|GO:0043231,intracellular membrane-bounded organelle|GO:0044464,cell part|GO:0005623,cell|GO:0005622,intracellular|GO:0044444,cytoplasmic part|GO:0044424,intracellular part|GO:0044425,membrane part|GO:0044422,organelle part|GO:0031984,organelle</td><td>GO:0043169,cation binding|GO:0004497,monooxygenase activity|GO:0070330,aromatase activity|GO:0046914,transition metal ion binding|GO:0020037,heme binding|GO:0046906,tetrapyrrole binding|GO:0097159,organic cyclic compound binding|GO:0016705,oxidoreductase activity, acting on paired donors, with incorporation or reduction of molecular oxygen|GO:0016712,oxidoreductase activity, acting on paired donors, with incorporation or reduction of molecular oxygen, reduced flavin or flavoprotein as one donor, and incorporation of one atom of oxygen|GO:0003824,catalytic activity|GO:0043167,ion binding|GO:0005488,binding|GO:0005506,iron ion binding|GO:0046872,metal ion binding|GO:1901363,heterocyclic compound binding|GO:0016491,oxidoreductase activity</td></tr>
<tr><td>PRAMEF20</td><td>GO:0080090,regulation of primary metabolic process|GO:0019222,regulation of metabolic process|GO:0048585,negative regulation of response to stimulus|GO:0048583,regulation of response to stimulus|GO:0007165,signal transduction|GO:1901362,organic cyclic compound biosynthetic process|GO:1901360,organic cyclic compound metabolic process|GO:0051716,cellular response to stimulus|GO:0010605,negative regulation of macromolecule metabolic process|GO:0009968,negative regulation of signal transduction|GO:0009966,regulation of signal transduction|GO:0048869,cellular developmental process|GO:0010467,gene expression|GO:0048518,positive regulation of biological process|GO:0048519,negative regulation of biological process|GO:0042127,regulation of cell proliferation|GO:0060255,regulation of macromolecule metabolic process|GO:0060548,negative regulation of cell death|GO:2001141,regulation of RNA biosynthetic process|GO:0046483,heterocycle metabolic process|GO:0044700,single organism signaling|GO:0044249,cellular biosynthetic process|GO:0019438,aromatic compound biosynthetic process|GO:0009892,negative regulation of metabolic process|GO:0009890,negative regulation of biosynthetic process|GO:0010629,negative regulation of gene expression|GO:0006807,nitrogen compound metabolic process|GO:0042981,regulation of apoptotic process|GO:0050789,regulation of biological process|GO:0097659,nucleic acid-templated transcription|GO:1901576,organic substance biosynthetic process|GO:0044260,cellular macromolecule metabolic process|GO:0065007,biological regulation|GO:0044699,single-organism process|GO:0018130,heterocycle biosynthetic process|GO:0016265,obsolete death|GO:0050793,regulation of developmental process|GO:0009889,regulation of biosynthetic process|GO:0050794,regulation of cellular process|GO:0012501,programmed cell death|GO:0008152,metabolic process|GO:0034654,nucleobase-containing compound biosynthetic process|GO:0016070,RNA metabolic process|GO:1902679,negative regulation of RNA biosynthetic process|GO:0044271,cellular nitrogen compound biosynthetic process|GO:0050896,response to stimulus|GO:0006355,regulation of transcription, DNA-templated|GO:0010556,regulation of macromolecule biosynthetic process|GO:0006351,transcription, DNA-templated|GO:0010558,negative regulation of macromolecule biosynthetic process|GO:0032774,RNA biosynthetic process|GO:0030154,cell differentiation|GO:0023057,negative regulation of signaling|GO:0034641,cellular nitrogen compound metabolic process|GO:0023052,signaling|GO:0010648,negative regulation of cell communication|GO:0034645,cellular macromolecule biosynthetic process|GO:0023051,regulation of signaling|GO:0010646,regulation of cell communication|GO:0048385,regulation of retinoic acid receptor signaling pathway|GO:0048384,retinoic acid receptor signaling pathway|GO:0048387,negative regulation of retinoic acid receptor signaling pathway|GO:0006139,nucleobase-containing compound metabolic process|GO:0008284,positive regulation of cell proliferation|GO:0008283,cell proliferation|GO:0009987,cellular process|GO:0006725,cellular aromatic compound metabolic process|GO:1903506,regulation of nucleic acid-templated transcription|GO:1903507,negative regulation of nucleic acid-templated transcription|GO:0045596,negative regulation of cell differentiation|GO:0045595,regulation of cell differentiation|GO:0045892,negative regulation of transcription, DNA-templated|GO:0051093,negative regulation of developmental process|GO:0051253,negative regulation of RNA metabolic process|GO:0051252,regulation of RNA metabolic process|GO:0043170,macromolecule metabolic process|GO:0032502,developmental process|GO:0031327,negative regulation of cellular biosynthetic process|GO:0031326,regulation of cellular biosynthetic process|GO:0031324,negative regulation of cellular metabolic process|GO:0031323,regulation of cellular metabolic process|GO:0090304,nucleic acid metabolic process|GO:0008219,cell death|GO:0010941,regulation of cell death|GO:0030522,intracellular receptor signaling pathway|GO:2000112,regulation of cellular macromolecule biosynthetic process|GO:2000113,negative regulation of cellular macromolecule biosynthetic process|GO:0071704,organic substance metabolic process|GO:0043067,regulation of programmed cell death|GO:0043066,negative regulation of apoptotic process|GO:0043069,negative regulation of programmed cell death|GO:0010468,regulation of gene expression|GO:0045934,negative regulation of nucleobase-containing compound metabolic process|GO:0019219,regulation of nucleobase-containing compound metabolic process|GO:0006915,apoptotic process|GO:0044767,single-organism developmental process|GO:0009058,biosynthetic process|GO:0009059,macromolecule biosynthetic process|GO:0044763,single-organism cellular process|GO:0051171,regulation of nitrogen compound metabolic process|GO:0051172,negative regulation of nitrogen compound metabolic process|GO:0007154,cell communication|GO:0044238,primary metabolic process|GO:0044237,cellular metabolic process|GO:0048523,negative regulation of cellular process|GO:0048522,positive regulation of cellular process</td><td></td><td>GO:0008134,transcription factor binding|GO:0035257,nuclear hormone receptor binding|GO:0005488,binding|GO:0005515,protein binding|GO:0042974,retinoic acid receptor binding|GO:0051427,hormone receptor binding|GO:0005102,receptor binding</td></tr>
</table>

ALOX12B	GO:0019220,regulation of phosphate metabolic process|GO:0080090,regulation of primary metabolic process|GO:0019222,regulation of metabolic process|GO:0051047,positive regulation of secretion|GO:0051049,regulation of transport|GO:0048584,positive regulation of response to stimulus|GO:0048583,regulation of response to stimulus|GO:0061436,establishment of skin barrier|GO:0007165,signal transduction|GO:0044283,small molecule biosynthetic process|GO:1901576,organic substance biosynthetic process|GO:0023014,signal transduction by protein phosphorylation|GO:0006636,unsaturated fatty acid biosynthetic process|GO:0044710,single-organism metabolic process|GO:0044711,single-organism biosynthetic process|GO:0010604,positive regulation of macromolecule metabolic process|GO:0009966,regulation of signal transduction|GO:0009967,positive regulation of signal transduction|GO:0043651,linoleic acid metabolic process|GO:0000165,MAPK cascade|GO:0048513,animal organ development|GO:0048518,positive regulation of biological process|GO:0006665,sphingolipid metabolic process|GO:0051050,positive regulation of transport|GO:0060255,regulation of macromolecule metabolic process|GO:0032787,monocarboxylic acid metabolic process|GO:0043436,oxoacid metabolic process|GO:0042759,long-chain fatty acid biosynthetic process|GO:0055114,oxidation-reduction process|GO:0042325,regulation of phosphorylation|GO:0044700,single organism signaling|GO:0042327,positive regulation of phosphorylation|GO:1901564,organonitrogen compound metabolic process|GO:0044707,single-multicellular organism process|GO:0048871,multicellular organismal homeostasis|GO:1901568,fatty acid derivative metabolic process|GO:0016053,organic acid biosynthetic process|GO:0048878,chemical homeostasis|GO:0019538,protein metabolic process|GO:0033559,unsaturated fatty acid metabolic process|GO:0006672,ceramide metabolic process|GO:0044281,small molecule metabolic process|GO:0009893,positive regulation of metabolic process|GO:0008152,metabolic process|GO:0010628,positive regulation of gene expression|GO:0006807,nitrogen compound metabolic process|GO:0042157,lipoprotein metabolic process|GO:0035556,intracellular signal transduction|GO:0050789,regulation of biological process|GO:0044267,cellular protein metabolic process|GO:0044260,cellular macromolecule metabolic process|GO:0065007,biological regulation|GO:0065008,regulation of biological quality|GO:0030148,sphingolipid biosynthetic process|GO:0006810,transport|GO:0006629,lipid metabolic process|GO:0033561,regulation of water loss via skin|GO:0051716,cellular response to stimulus|GO:0050794,regulation of cellular process|GO:0043410,positive regulation of MAPK cascade|GO:0043412,macromolecule modification|GO:0036211,protein modification process|GO:0051239,regulation of multicellular organismal process|GO:0001676,long-chain fatty acid metabolic process|GO:1902533,positive regulation of intracellular signal transduction|GO:1902531,regulation of intracellular signal transduction|GO:0044767,single-organism developmental process|GO:0046903,secretion|GO:0044271,cellular nitrogen compound biosynthetic process|GO:0046394,carboxylic acid biosynthetic process|GO:0050896,response to stimulus|GO:0031401,positive regulation of protein modification process|GO:0050891,multicellular organismal water homeostasis|GO:0006690,icosanoid metabolic process|GO:0043603,cellular amide metabolic process|GO:0051046,regulation of secretion|GO:0051240,positive regulation of multicellular organismal process|GO:0006633,fatty acid biosynthetic process|GO:0006631,fatty acid metabolic process|GO:0051122,hepoxilin biosynthetic process|GO:0051121,hepoxilin metabolic process|GO:0016310,phosphorylation|GO:0023056,positive regulation of signaling|GO:0044249,cellular biosynthetic process|GO:0034641,cellular nitrogen compound metabolic process|GO:0023052,signaling|GO:0034645,cellular macromolecule biosynthetic process|GO:0023051,regulation of signaling|GO:0010647,positive regulation of cell communication|GO:0010646,regulation of cell communication|GO:1901566,organonitrogen compound biosynthetic process|GO:0044699,single-organism process|GO:0043408,regulation of MAPK cascade|GO:0051234,establishment of localization|GO:0010562,positive regulation of phosphorus metabolic process|GO:0051246,regulation of protein metabolic process|GO:0051247,positive regulation of protein metabolic process|GO:0032270,positive regulation of cellular protein metabolic process|GO:0042158,lipoprotein biosynthetic process|GO:0031399,regulation of protein modification process|GO:0046467,membrane lipid biosynthetic process|GO:0032502,developmental process|GO:0032501,multicellular organismal process|GO:0006643,membrane lipid metabolic process|GO:0050878,regulation of body fluid levels|GO:0009987,cellular process|GO:0009058,biosynthetic process|GO:0044255,cellular lipid metabolic process|GO:0032879,regulation of localization|GO:0043604,amide biosynthetic process|GO:0032268,regulation of cellular protein metabolic process|GO:0006082,organic acid metabolic process|GO:0043170,macromolecule metabolic process|GO:0032941,secretion by tissue|GO:0048731,system development|GO:0031325,positive regulation of cellular metabolic process|GO:0031323,regulation of cellular metabolic process|GO:0019752,carboxylic acid metabolic process|GO:0042592,homeostatic process|GO:0007275,multicellular organism development|GO:0001934,positive regulation of protein phosphorylation|GO:0072330,monocarboxylic acid biosynthetic process|GO:0071704,organic substance metabolic process|GO:0010467,gene expression|GO:0019372,lipoxygenase pathway|GO:0010468,regulation of gene expression|GO:0043588,skin development|GO:0006468,protein phosphorylation|GO:0045937,positive regulation of phosphate metabolic process|GO:0007589,body fluid secretion|GO:0006464,cellular protein modification process|GO:0051174,regulation of phosphorus metabolic process|GO:0044765,single-organism transport|GO:0009059,macromolecule biosynthetic process|GO:0044763,single-organism cellular process|GO:0030104,water homeostasis|GO:0007154,cell communication|GO:0051179,localization|GO:1902578,single-organism localization|GO:0008610,lipid biosynthetic process|GO:0006497,protein lipidation|GO:0044238,primary metabolic process|GO:0070257,positive regulation of mucus secretion|GO:0048856,anatomical structure development|GO:0044237,cellular metabolic process|GO:0070255,regulation of mucus secretion|GO:0006796,phosphate-containing compound metabolic process|GO:0019369,arachidonic acid metabolic process|GO:0006793,phosphorus metabolic process|GO:0001932,regulation of protein phosphorylation|GO:0070254,mucus secretion|GO:0046513,ceramide biosynthetic process|GO:0048522,positive	GO:0005737,cytoplasm|GO:0005829,cytosol|GO:0044464,cell part|GO:0005623,cell|GO:0005622,intracellular|GO:0044444,cytoplasmic part|GO:0044424,intracellular part	GO:0043169,cation binding|GO:0043167,ion binding|GO:0046914,transition metal ion binding|GO:0051213,dioxygenase activity|GO:0004052,arachidonate 12-lipoxygenase activity|GO:1990136,linoleate 9S-lipoxygenase activity|GO:0016702,oxidoreductase activity, acting on single donors with incorporation of molecular oxygen, incorporation of two atoms of oxygen|GO:0016701,oxidoreductase activity, acting on single donors with incorporation of molecular oxygen|GO:0003824,catalytic activity|GO:0005488,binding|GO:0005515,protein binding|GO:0005506,iron ion binding|GO:0046872,metal ion binding|GO:0016491,oxidoreductase activity
PUS10	GO:0009451,RNA modification|GO:0090304,nucleic acid metabolic process|GO:0034641,cellular nitrogen compound metabolic process|GO:0006807,nitrogen compound metabolic process|GO:0008033,tRNA processing|GO:0034660,ncRNA metabolic process|GO:0001522,pseudouridine synthesis|GO:1901360,organic cyclic compound metabolic process|GO:0006139,nucleobase-containing compound metabolic process|GO:0044260,cellular macromolecule metabolic process|GO:0071704,organic substance metabolic process|GO:0010467,gene expression|GO:0034470,ncRNA processing|GO:0009987,cellular process|GO:0006725,cellular aromatic compound metabolic process|GO:0043412,macromolecule modification|GO:0008152,metabolic process|GO:0046483,heterocycle metabolic process|GO:0016070,RNA metabolic process|GO:0044238,primary metabolic process|GO:0044237,cellular metabolic process|GO:0043170,macromolecule metabolic process|GO:0006399,tRNA metabolic process|GO:0006396,RNA processing|GO:0031119,tRNA pseudouridine synthesis|GO:0006400,tRNA modification		GO:0016853,isomerase activity|GO:0097159,organic cyclic compound binding|GO:0009982,pseudouridine synthase activity|GO:0003824,catalytic activity|GO:0003723,RNA binding|GO:0003676,nucleic acid binding|GO:0016866,intramolecular transferase activity|GO:1901363,heterocyclic compound binding|GO:0005488,binding
PWWP2A		GO:0043231,intracellular membrane-bounded organelle|GO:0005634,nucleus|GO:0044464,cell part|GO:0005623,cell|GO:0005622,intracellular|GO:0043229,intracellular organelle|GO:0044424,intracellular part|GO:0043227,membrane-bounded organelle|GO:0043226,organelle	GO:0005488,binding|GO:0005515,protein binding|GO:0003682,chromatin binding|GO:0042393,histone binding
ATF7			GO:0043169,cation binding|GO:0097159,organic cyclic compound binding|GO:0043167,ion binding|GO:0005488,binding|GO:0003676,nucleic acid binding|GO:0046872,metal ion binding|GO:1901363,heterocyclic compound binding
FAM104B			
WFDC10B	GO:0009892,negative regulation of metabolic process|GO:0080090,regulation of primary metabolic process|GO:0019222,regulation of metabolic process|GO:0031324,negative regulation of cellular metabolic process|GO:0031323,regulation of cellular metabolic process|GO:0043086,negative regulation of catalytic activity|GO:0044267,cellular protein metabolic process|GO:0051248,negative regulation of protein metabolic process|GO:0010605,negative regulation of macromolecule metabolic process|GO:0051346,negative regulation of hydrolase activity|GO:0044260,cellular macromolecule metabolic process|GO:0051246,regulation of protein metabolic process|GO:0050789,regulation of biological process|GO:0071704,organic substance metabolic process|GO:0010466,negative regulation of peptidase activity|GO:0065007,biological regulation|GO:0044092,negative regulation of molecular function|GO:0048519,negative regulation of biological process|GO:0065009,regulation of molecular function|GO:0009987,cellular process|GO:0052547,regulation of peptidase activity|GO:0060255,regulation of macromolecule metabolic process|GO:0050790,regulation of catalytic activity|GO:0050794,regulation of cellular process|GO:0030162,regulation of proteolysis|GO:0008152,metabolic process|GO:0006508,proteolysis|GO:0051336,regulation of hydrolase activity|GO:0044238,primary metabolic process|GO:0032269,negative regulation of cellular protein metabolic process|GO:0032268,regulation of cellular protein metabolic process|GO:0019538,protein metabolic process|GO:0044237,cellular metabolic process|GO:0043170,macromolecule metabolic process|GO:0045861,negative regulation of proteolysis|GO:0048523,negative regulation of cellular process|GO:0006807,nitrogen compound metabolic	GO:0005576,extracellular region	GO:0030234,enzyme regulator activity|GO:0061134,peptidase regulator activity|GO:0030414,peptidase inhibitor activity|GO:0004857,enzyme inhibitor activity|GO:0098772,molecular function regulator
SPZ1	GO:0080090,regulation of primary metabolic process|GO:0019222,regulation of metabolic process|GO:0031326,regulation of cellular biosynthetic process|GO:0031323,regulation of cellular metabolic process|GO:0090304,nucleic acid metabolic process|GO:0044249,cellular biosynthetic process|GO:0034641,cellular nitrogen compound metabolic process|GO:0006807,nitrogen compound metabolic process|GO:0034645,cellular macromolecule biosynthetic process|GO:1901362,organic cyclic compound biosynthetic process|GO:0050789,regulation of biological process|GO:0097659,nucleic acid-templated transcription|GO:0032774,RNA biosynthetic process|GO:0006139,nucleobase-containing compound metabolic process|GO:0044260,cellular macromolecule metabolic process|GO:0071704,organic substance metabolic process|GO:2000112,regulation of cellular macromolecule biosynthetic process|GO:0060255,regulation of macromolecule metabolic process|GO:0010467,gene expression|GO:0065007,biological regulation|GO:1901360,organic cyclic compound metabolic process|GO:0010468,regulation of gene expression|GO:0018130,heterocycle biosynthetic process|GO:1901576,organic substance biosynthetic process|GO:0019219,regulation of nucleobase-containing compound metabolic process|GO:0006725,cellular aromatic compound metabolic process|GO:0009987,cellular process|GO:0009889,regulation of biosynthetic process|GO:1903506,regulation of nucleic acid-templated transcription|GO:0050794,regulation of cellular process|GO:0009058,biosynthetic process|GO:0009059,macromolecule biosynthetic process|GO:0051171,regulation of nitrogen compound metabolic process|GO:0008152,metabolic process|GO:2001141,regulation of RNA biosynthetic process|GO:0034654,nucleobase-containing compound biosynthetic process|GO:0046483,heterocycle metabolic process|GO:0016070,RNA metabolic process|GO:0044238,primary metabolic process|GO:0044271,cellular nitrogen compound biosynthetic process|GO:0051252,regulation of RNA metabolic process|GO:0044237,cellular metabolic process|GO:0043170,macromolecule metabolic process|GO:0006355,regulation of transcription, DNA-templated|GO:0010556,regulation of macromolecule biosynthetic process|GO:0006351,transcription, DNA-templated|GO:0019438,aromatic compound biosynthetic process	GO:0005737,cytoplasm|GO:0043231,intracellular membrane-bounded organelle|GO:0005634,nucleus|GO:0044464,cell part|GO:0005623,cell|GO:0005622,intracellular|GO:0043229,intracellular organelle|GO:0044424,intracellular part|GO:0043227,membrane-bounded organelle|GO:0043226,organelle	GO:0003676,nucleic acid binding|GO:0003700,transcription factor activity, sequence-specific DNA binding|GO:0097159,organic cyclic compound binding|GO:0005488,binding|GO:0005515,protein binding|GO:0003677,DNA binding|GO:1901363,heterocyclic compound binding|GO:0001071,nucleic acid binding transcription factor activity
WFDC10A	GO:0009892,negative regulation of metabolic process|GO:0080090,regulation of primary metabolic process|GO:0019222,regulation of metabolic process|GO:0031324,negative regulation of cellular metabolic process|GO:0031323,regulation of cellular metabolic process|GO:0043086,negative regulation of catalytic activity|GO:0044267,cellular protein metabolic process|GO:0051248,negative regulation of protein metabolic process|GO:0010605,negative regulation of macromolecule metabolic process|GO:0051346,negative regulation of hydrolase activity|GO:0044260,cellular macromolecule metabolic process|GO:0071704,organic substance metabolic process|GO:0051246,regulation of protein metabolic process|GO:0050789,regulation of biological process|GO:0060255,regulation of macromolecule metabolic process|GO:0010466,negative regulation of peptidase activity|GO:0065007,biological regulation|GO:0044092,negative regulation of molecular function|GO:0048519,negative regulation of biological process|GO:0065009,regulation of molecular function|GO:0009987,cellular process|GO:0052547,regulation of peptidase activity|GO:0052548,regulation of endopeptidase activity|GO:0050790,regulation of catalytic activity|GO:0050794,regulation of cellular process|GO:0030162,regulation of proteolysis|GO:0008152,metabolic process|GO:0006508,proteolysis|GO:0010951,negative regulation of endopeptidase activity|GO:0051336,regulation of hydrolase activity|GO:0044238,primary metabolic process|GO:0032269,negative regulation of cellular protein metabolic process|GO:0032268,regulation of cellular protein metabolic process|GO:0019538,protein metabolic process|GO:0044237,cellular metabolic process|GO:0043170,macromolecule metabolic process|GO:0045861,negative regulation of proteolysis|GO:0048523,negative regulation of cellular process|GO:0006807,nitrogen compound metabolic process|GO:1901564,organonitrogen compound metabolic process	GO:0005576,extracellular region	GO:0004866,endopeptidase inhibitor activity|GO:0061135,endopeptidase regulator activity|GO:0061134,peptidase regulator activity|GO:0030414,peptidase inhibitor activity|GO:0004867,serine-type endopeptidase inhibitor activity|GO:0004857,enzyme inhibitor activity|GO:0098772,molecular function regulator|GO:0030234,enzyme regulator activity
GUCA2A	GO:0019220,regulation of phosphate metabolic process|GO:0080090,regulation of primary metabolic process|GO:0019222,regulation of metabolic process|GO:0009165,nucleotide biosynthetic process|GO:0044281,small molecule metabolic process|GO:1901362,organic cyclic compound biosynthetic process|GO:0045981,positive regulation of nucleotide metabolic process|GO:1901360,organic cyclic compound metabolic process|GO:0044710,single-organism metabolic process|GO:0044711,single-organism biosynthetic process|GO:0052652,cyclic purine nucleotide metabolic process|GO:0044093,positive regulation of molecular function|GO:0048518,positive regulation of biological process|GO:0009190,cyclic nucleotide biosynthetic process|GO:0031279,regulation of cyclase activity|GO:0046483,heterocycle metabolic process|GO:1901564,organonitrogen compound metabolic process|GO:1901566,organonitrogen compound biosynthetic process|GO:1900373,positive regulation of purine nucleotide biosynthetic process|GO:1900371,regulation of purine nucleotide biosynthetic process|GO:0006163,purine nucleotide metabolic process|GO:0006164,purine nucleotide biosynthetic process|GO:0019637,organophosphate metabolic process|GO:0019438,aromatic compound biosynthetic process|GO:0009893,positive regulation of metabolic process|GO:0009891,positive regulation of biosynthetic process|GO:0006807,nitrogen compound metabolic process|GO:0050789,regulation of biological process|GO:1901576,organic substance biosynthetic process|GO:0051349,positive regulation of lyase activity|GO:0065007,biological regulation|GO:0043085,positive regulation of catalytic activity|GO:0065009,regulation of molecular function|GO:0018130,heterocycle biosynthetic process|GO:0050790,regulation of catalytic activity|GO:0009889,regulation of biosynthetic process|GO:0009152,purine ribonucleotide biosynthetic process|GO:0050794,regulation of cellular process|GO:0009260,ribonucleotide biosynthetic process|GO:0008152,metabolic process|GO:0034654,nucleobase-containing compound biosynthetic process|GO:0009150,purine ribonucleotide metabolic process|GO:0090407,organophosphate biosynthetic process|GO:0006182,cGMP biosynthetic process|GO:0044271,cellular nitrogen compound biosynthetic process|GO:0046390,ribose phosphate biosynthetic process|GO:0051339,regulation of lyase activity|GO:1901293,nucleoside phosphate biosynthetic process|GO:0044763,single-organism cellular process|GO:0006753,nucleoside phosphate metabolic process|GO:0044249,cellular biosynthetic process|GO:0034641,cellular nitrogen compound metabolic process|GO:0046068,cGMP metabolic process|GO:0009259,ribonucleotide metabolic process|GO:0044699,single-organism process|GO:0006139,nucleobase-containing compound metabolic process|GO:0010562,positive regulation of phosphorus metabolic process|GO:0030810,positive regulation of nucleotide biosynthetic process|GO:0044238,primary metabolic process|GO:0009987,cellular process|GO:0006725,cellular aromatic compound metabolic process|GO:0030799,regulation of cyclic nucleotide metabolic process|GO:0055086,nucleobase-containing small molecule metabolic process|GO:0030808,regulation of nucleotide biosynthetic process|GO:1901137,carbohydrate derivative biosynthetic process|GO:0030804,positive regulation of cyclic nucleotide biosynthetic process|GO:1901135,carbohydrate derivative metabolic process|GO:0030801,positive regulation of cyclic nucleotide metabolic process|GO:0030802,regulation of cyclic nucleotide biosynthetic process|GO:0031284,positive regulation of guanylate cyclase activity|GO:0009187,cyclic nucleotide metabolic process|GO:0031281,positive regulation of cyclase activity|GO:0031282,regulation of guanylate cyclase activity|GO:0031328,positive regulation of cellular biosynthetic process|GO:0031326,regulation of cellular biosynthetic process|GO:0031325,positive regulation of cellular metabolic process|GO:0031323,regulation of cellular metabolic process|GO:0044237,cellular metabolic process|GO:0072521,purine-containing compound metabolic process|GO:0072522,purine-containing compound biosynthetic process|GO:0071704,organic substance metabolic process|GO:0045935,positive regulation of nucleobase-containing compound metabolic process|GO:0045937,positive regulation of phosphate metabolic process|GO:0019219,regulation of nucleobase-containing compound metabolic process|GO:0051174,regulation of phosphorus metabolic process|GO:0009058,biosynthetic process|GO:0009117,nucleotide metabolic process|GO:0051171,regulation of nitrogen compound metabolic process|GO:0051173,positive regulation of nitrogen compound metabolic process|GO:0030826,regulation of cGMP biosynthetic process|GO:0030825,positive regulation of cGMP metabolic process|GO:1900542,regulation of purine nucleotide metabolic process|GO:0030823,regulation of cGMP metabolic process|GO:1900544,positive regulation of purine nucleotide metabolic process|GO:0019693,ribose phosphate metabolic process|GO:0030828,positive regulation of cGMP biosynthetic process|GO:0006796,phosphate-containing compound metabolic process|GO:0006793,phosphorus metabolic process|GO:0006140,regulation of nucleotide metabolic process|GO:0048522,positive regulation of cellular process|GO:0044707,single-multicellular organism process|GO:0032501,multicellular organismal	GO:0043230,extracellular organelle|GO:0031982,vesicle|GO:0044421,extracellular region part|GO:0043227,membrane-bounded organelle|GO:0031988,membrane-bounded vesicle|GO:0005576,extracellular region|GO:1903561,extracellular vesicle|GO:0065010,extracellular membrane-bounded organelle|GO:0043226,organelle|GO:0070062,extracellular exosome	GO:0005179,hormone activity|GO:0030249,guanylate cyclase regulator activity|GO:0030234,enzyme regulator activity|GO:0030250,guanylate cyclase activator activity|GO:0008047,enzyme activator activity|GO:0005515,protein binding|GO:0005488,binding|GO:0005102,receptor binding|GO:0098772,molecular function regulator|GO:0010851,cyclase regulator activity|GO:0010853,cyclase activator activity
LCE1B	GO:0030154,cell differentiation|GO:0008544,epidermis development|GO:0007275,multicellular organism development|GO:0044699,single-organism process|GO:0048869,cellular developmental process|GO:0048513,animal organ development|GO:0030855,epithelial cell differentiation|GO:0043588,skin development|GO:0032502,developmental process|GO:0032501,multicellular organismal process|GO:0030216,keratinocyte differentiation|GO:0060429,epithelium development|GO:0009888,tissue development|GO:0044767,single-organism developmental process|GO:0048731,system development|GO:0044707,single-multicellular organism process|GO:0031424,keratinization|GO:0048856,anatomical structure development|GO:0009913,epidermal cell differentiation|GO:0044763,single-organism cellular process|GO:0009987,cellular process|GO:0018149,peptide cross-linking|GO:0006807,nitrogen compound metabolic process|GO:0044267,cellular protein metabolic process|GO:0044260,cellular macromolecule metabolic process|GO:0071704,organic substance metabolic process|GO:0019538,protein metabolic process|GO:0006464,cellular protein modification process|GO:0043412,macromolecule modification|GO:0036211,protein modification process|GO:0008152,metabolic process|GO:0044238,primary metabolic process|GO:1901564,organonitrogen compound metabolic process|GO:0044237,cellular metabolic process|GO:0043170,macromolecule metabolic process	GO:0005737,cytoplasm|GO:0016020,membrane|GO:0044464,cell part|GO:0005623,cell|GO:0005622,intracellular|GO:0071944,cell periphery|GO:0005886,plasma membrane|GO:0044424,intracellular part|GO:0001533,cornified envelope	GO:0005488,binding|GO:0005515,protein binding|GO:0005198,structural molecule activity
EMC6	GO:0009991,response to extracellular stimulus|GO:0022607,cellular component assembly|GO:0006457,protein folding|GO:0008152,metabolic process|GO:0016236,macroautophagy|GO:0044248,cellular catabolic process|GO:0042594,response to starvation|GO:0071840,cellular component organization or biogenesis|GO:0044712,single-organism catabolic process|GO:0044267,cellular protein metabolic process|GO:0044710,single-organism metabolic process|GO:0044260,cellular macromolecule metabolic process|GO:0016043,cellular component organization|GO:0000045,autophagosome assembly|GO:0071704,organic substance metabolic process|GO:0044699,single-organism process|GO:0071496,cellular response to external stimulus|GO:0044238,primary metabolic process|GO:0006914,autophagy|GO:0009987,cellular process|GO:0051716,cellular response to stimulus|GO:0006950,response to stress|GO:0044763,single-organism cellular process|GO:0009267,cellular response to starvation|GO:0007154,cell communication|GO:0070925,organelle assembly|GO:0009056,catabolic process|GO:0006996,organelle organization|GO:0007033,vacuole organization|GO:0031668,cellular response to extracellular stimulus|GO:0031669,cellular response to nutrient levels|GO:0009605,response to external stimulus|GO:0019538,protein metabolic process|GO:0050896,response to stimulus|GO:0031667,response to nutrient levels|GO:0044237,cellular metabolic process|GO:0043170,macromolecule metabolic process|GO:0044085,cellular component biogenesis|GO:0033554,cellular response to stress|GO:0034975,protein folding in endoplasmic reticulum|GO:1905037,autophagosome organization	GO:0005783,endoplasmic reticulum|GO:0005789,endoplasmic reticulum membrane|GO:0042175,nuclear outer membrane-endoplasmic reticulum membrane network|GO:0043229,intracellular organelle|GO:0030176,integral component of endoplasmic reticulum membrane|GO:0031301,integral component of organelle membrane|GO:0031300,intrinsic component of organelle membrane|GO:0043227,membrane-bounded organelle|GO:0043226,organelle|GO:0031224,intrinsic component of membrane|GO:0097631,integral component of omegasome membrane|GO:0097630,intrinsic component of omegasome membrane|GO:0031090,organelle membrane|GO:0016021,integral component of membrane|GO:0016020,membrane|GO:0072546,ER membrane protein complex|GO:0031227,intrinsic component of endoplasmic reticulum membrane|GO:0044432,endoplasmic reticulum part|GO:1990462,omegasome|GO:0005737,cytoplasm|GO:0098588,bounding membrane of organelle|GO:1903349,omegasome membrane|GO:0012505,endomembrane system|GO:0043234,protein complex|GO:0032991,macromolecular complex|GO:0043231,intracellular membrane-bounded organelle|GO:0044464,cell part|GO:0005623,cell|GO:0005622,intracellular|GO:0044446,intracellular organelle part|GO:0098796,membrane protein complex|GO:0044444,cytoplasmic part|GO:0044424,intracellular part|GO:0044425,membrane part|GO:0044422,organelle part|GO:0031984,organelle	GO:0005488,binding|GO:0005515,protein binding
XKR4		GO:0044425,membrane part|GO:0016021,integral component of membrane|GO:0016020,membrane|GO:0031224,intrinsic component of membrane	
CADM2	GO:0008037,cell recognition|GO:0022607,cellular component assembly|GO:0022610,biological adhesion|GO:0098609,cell-cell adhesion|GO:0034332,adherens junction organization|GO:0009987,cellular process|GO:0034330,cell junction organization|GO:0016043,cellular component organization|GO:0044085,cellular component biogenesis|GO:0044763,single-organism cellular process|GO:0071840,cellular component organization or biogenesis|GO:0045216,cell-cell junction organization|GO:0007155,cell adhesion|GO:0007157,heterophilic cell-cell adhesion via plasma membrane cell adhesion molecules|GO:0007156,homophilic cell adhesion via plasma membrane adhesion molecules|GO:0098742,cell-cell adhesion via plasma-membrane adhesion molecules|GO:0034329,cell junction assembly|GO:0044699,single-organism process	GO:0005911,cell-cell junction|GO:0030424,axon|GO:0071944,cell periphery|GO:0045202,synapse|GO:0031226,intrinsic component of plasma membrane|GO:0031224,intrinsic component of membrane|GO:0016021,integral component of membrane|GO:0016020,membrane|GO:0097458,neuron part|GO:0030054,cell junction|GO:0043005,neuron projection|GO:0044459,plasma membrane part|GO:0070161,anchoring junction|GO:0005913,cell-cell adherens junction|GO:0005912,adherens junction|GO:0005887,integral component of plasma membrane|GO:0005886,plasma membrane|GO:0042995,cell projection|GO:0044464,cell part|GO:0005623,cell|GO:0044425,membrane part	GO:0005488,binding|GO:0060089,molecular transducer activity|GO:0004872,receptor activity|GO:0005515,protein binding|GO:0046983,protein dimerization activity|GO:0050839,cell adhesion molecule binding|GO:0005102,receptor binding|GO:0042802,identical protein binding|GO:0042803,protein homodimerization activity
NBEAL1	GO:0016197,endosomal transport|GO:0046907,intracellular transport|GO:0006810,transport|GO:0044765,single-organism transport|GO:1902578,single-organism localization|GO:0051649,establishment of localization in cell|GO:1902582,single-organism intracellular transport|GO:0051234,establishment of localization|GO:0051179,localization|GO:0044699,single-organism process|GO:0051641,cellular localization	GO:0016020,membrane|GO:0044464,cell part|GO:0005623,cell|GO:0005622,intracellular|GO:0012505,endomembrane system|GO:0044425,membrane part|GO:0019898,extrinsic component of membrane	GO:0043168,anion binding|GO:0005543,phospholipid binding|GO:0008289,lipid binding|GO:0043167,ion binding|GO:0005488,binding
FUT9	GO:0019320,hexose catabolic process|GO:0046365,monosaccharide catabolic process|GO:0044249,cellular biosynthetic process|GO:0032501,multicellular organismal process|GO:0034645,cellular macromolecule biosynthetic process|GO:0009100,glycoprotein metabolic process|GO:0007275,multicellular organism development|GO:0044699,single-organism process|GO:0044712,single-organism catabolic process|GO:0044267,cellular protein metabolic process|GO:0044710,single-organism metabolic process|GO:0006486,protein glycosylation|GO:0042354,L-fucose metabolic process|GO:0042355,L-fucose catabolic process|GO:0009101,glycoprotein biosynthetic process|GO:0071704,organic substance metabolic process|GO:0032502,developmental process|GO:0036065,fucosylation|GO:0019317,fucose catabolic process|GO:1901576,organic substance biosynthetic process|GO:0070085,glycosylation|GO:0009987,cellular process|GO:0019318,hexose metabolic process|GO:1901575,organic substance catabolic process|GO:0006464,cellular protein modification process|GO:0044260,cellular macromolecule metabolic process|GO:0044767,single-organism developmental process|GO:0043412,macromolecule modification|GO:0043413,macromolecule glycosylation|GO:0044763,single-organism cellular process|GO:0008152,metabolic process|GO:0044723,single-organism carbohydrate metabolic process|GO:0009059,macromolecule biosynthetic process|GO:0044724,single-organism carbohydrate catabolic process|GO:0044238,primary metabolic process|GO:0005975,carbohydrate metabolic process|GO:0044707,single-multicellular organism process|GO:0007399,nervous system development|GO:0019538,protein metabolic process|GO:0005996,monosaccharide metabolic process|GO:1901135,carbohydrate derivative metabolic process|GO:0048856,anatomical structure development|GO:0016052,carbohydrate catabolic process|GO:0009058,biosynthetic process|GO:0044237,cellular metabolic process|GO:0043170,macromolecule metabolic process|GO:1901137,carbohydrate derivative biosynthetic process|GO:0036211,protein modification process|GO:0006004,fucose metabolic process|GO:0048731,system development|GO:0009056,catabolic process|GO:0006807,nitrogen compound metabolic process|GO:0044281,small molecule metabolic process|GO:0044282,small molecule catabolic process|GO:1901566,organonitrogen compound biosynthetic process|GO:1901564,organonitrogen compound metabolic process	GO:0043229,intracellular organelle|GO:0005622,intracellular|GO:0043227,membrane-bounded organelle|GO:0043226,organelle|GO:0031224,intrinsic component of membrane|GO:0005737,cytoplasm|GO:0031985,Golgi cisterna|GO:0031984,organelle subcompartment|GO:0031090,organelle membrane|GO:0016021,integral component of membrane|GO:0016020,membrane|GO:0044431,Golgi apparatus part|GO:0005795,Golgi stack|GO:0005794,Golgi apparatus|GO:0098588,bounding membrane of organelle|GO:0012505,endomembrane system|GO:0032580,Golgi cisterna membrane|GO:0043231,intracellular membrane-bounded organelle|GO:0044464,cell part|GO:0005623,cell|GO:0000139,Golgi membrane|GO:0044446,intracellular organelle part|GO:0044444,cytoplasmic part|GO:0098791,Golgi subcompartment|GO:0044424,intracellular part|GO:0044425,membrane part|GO:0044422,organelle part	GO:0008417,fucosyltransferase activity|GO:0003824,catalytic activity|GO:0016740,transferase activity|GO:0016757,transferase activity, transferring glycosyl groups|GO:0016758,transferase activity, transferring hexosyl groups|GO:0046920,alpha-(1->3)-fucosyltransferase activity
CDK6	GO:0019222,regulation of metabolic process|GO:0048585,negative regulation of response to stimulus|GO:0048583,regulation of response to stimulus|GO:0050680,negative regulation of epithelial cell proliferation|GO:0032844,regulation of homeostatic process|GO:0007160,cell-matrix adhesion|GO:0060322,head development|GO:0007165,signal transduction|GO:0007166,cell surface receptor signaling pathway|GO:0051716,cellular response to stimulus|GO:0007219,Notch signaling pathway|GO:0045786,negative regulation of cell cycle|GO:0010604,positive regulation of macromolecule metabolic process|GO:0043207,response to external biotic stimulus|GO:0045785,positive regulation of cell adhesion|GO:0048869,cellular developmental process|GO:0002262,myeloid cell homeostasis|GO:0010467,gene expression|GO:0071704,organic substance metabolic process|GO:0045667,regulation of osteoblast differentiation|GO:0048513,animal organ development|GO:0030855,epithelial cell differentiation|GO:0048518,positive regulation of biological process|GO:0048519,negative regulation of biological process|GO:0002066,columnar/cuboidal epithelial cell development|GO:0030155,regulation of cell adhesion|GO:0002064,epithelial cell development|GO:0002065,columnar/cuboidal epithelial cell differentiation|GO:0042127,regulation of cell proliferation|GO:0031589,cell-substrate adhesion|GO:0031016,pancreas development|GO:0030217,T cell differentiation|GO:0060255,regulation of macromolecule metabolic process|GO:0048468,cell development|GO:0035270,endocrine system development|GO:0046649,lymphocyte activation|GO:0007568,aging|GO:1903706,regulation of hemopoiesis|GO:1903707,negative regulation of hemopoiesis|GO:0051704,multi-organism process|GO:0044700,single organism signaling|GO:0065008,regulation of biological quality|GO:0009607,response to biotic stimulus|GO:0048872,homeostasis of number of cells|GO:0044707,single-multicellular organism process|GO:0048870,cell motility|GO:0019538,protein metabolic process|GO:0014002,astrocyte development|GO:0002376,immune system process|GO:0007154,cell communication|GO:0001503,ossification|GO:0033554,cellular response to stress|GO:0021537,telencephalon development|GO:0090342,regulation of cell aging|GO:0090344,negative regulation of cell aging|GO:0009893,positive regulation of metabolic process|GO:0021782,glial cell development|GO:0006950,response to stress|GO:0043697,cell dedifferentiation|GO:0006928,movement of cell or subcellular component|GO:0008152,metabolic process|GO:0045321,leukocyte activation|GO:0010628,positive regulation of gene expression|GO:0043170,macromolecule metabolic process|GO:0050789,regulation of biological process|GO:0009605,response to external stimulus|GO:0044267,cellular protein metabolic process|GO:0044260,cellular macromolecule metabolic process|GO:0045646,regulation of erythrocyte differentiation|GO:0098609,cell-cell adhesion|GO:0002682,regulation of immune system process|GO:0002683,negative regulation of immune system process|GO:0010811,positive regulation of cell-substrate adhesion|GO:0071593,lymphocyte aggregation|GO:0071594,thymocyte aggregation|GO:0000082,G1/S transition of mitotic cell cycle|GO:0042063,gliogenesis|GO:0098602,single organism cell adhesion|GO:0033077,T cell differentiation in thymus|GO:0050793,regulation of developmental process|GO:0016310,phosphorylation|GO:0048708,astrocyte differentiation|GO:0050794,regulation of cellular process|GO:0021542,dentate gyrus development|GO:0021543,pallium development|GO:0001775,cell activation|GO:0048863,stem cell differentiation|GO:0051239,regulation of multicellular organismal process|GO:0048731,system development|GO:0021591,ventricular system development|GO:0030278,regulation of ossification|GO:0030279,negative regulation of ossification|GO:0002521,leukocyte differentiation|GO:0002520,immune system development|GO:0007050,cell cycle arrest|GO:0007420,brain development|GO:0080134,regulation of response to stress|GO:0080135,regulation of cellular response to stress|GO:0043412,macromolecule modification|GO:0065007,biological regulation|GO:0045638,negative regulation of myeloid cell differentiation|GO:0036211,protein modification process|GO:2000145,regulation of cell motility|GO:0009615,response to virus|GO:0070489,T cell aggregation|GO:0045637,regulation of myeloid cell differentiation|GO:0031018,endocrine pancreas development|GO:0043696,dedifferentiation|GO:0048146,positive regulation of fibroblast proliferation|GO:0048145,regulation of fibroblast proliferation|GO:0048144,fibroblast proliferation|GO:0021766,hippocampus development|GO:0023052,signaling|GO:0010001,glial cell differentiation|GO:0021761,limbic system development|GO:0007049,cell cycle|GO:0007417,central nervous system development|GO:0003309,type B pancreatic cell differentiation|GO:0050896,response to stimulus|GO:0051241,negative regulation of multicellular organismal process|GO:0002068,glandular epithelial cell development|GO:0042592,homeostatic process|GO:0022610,biological adhesion|GO:0045668,negative regulation of osteoblast differentiation|GO:0008284,positive regulation of cell proliferation|GO:0008285,negative regulation of cell proliferation|GO:0032501,multicellular organismal process|GO:0008283,cell proliferation|GO:0060429,epithelium development|GO:0045596,negative regulation of cell differentiation|GO:0045595,regulation of cell differentiation|GO:0051270,regulation of cellular component movement|GO:1903047,mitotic cell cycle process|GO:0060218,hematopoietic stem cell differentiation|GO:0032879,regulation of localization|GO:0021670,lateral ventricle development|GO:0051093,negative regulation of developmental process|GO:0035883,enteroendocrine cell differentiation|GO:0044699,single-organism process|GO:0050678,regulation of epithelial cell proliferation|GO:0007569,cell aging|GO:0051707,response to other organism|GO:0001954,positive regulation of cell-matrix adhesion|GO:0044763,single-organism cellular process|GO:0001952,regulation of cell-matrix adhesion|GO:0050673,epithelial cell proliferation|GO:0051674,localization of cell|GO:0030098,lymphocyte differentiation|GO:0030099,myeloid cell differentiation|GO:0030097,hemopoiesis|GO:2000772,regulation of cellular senescence|GO:2000773,negative regulation of cellular senescence|GO:0032502,developmental process|GO:0048856,anatomical structure development|GO:0016337,single organismal cell-cell adhesion|GO:0042110,T cell activation|GO:0044770,cell cycle phase transition|GO:0044772,mitotic cell cycle phase transition|GO:0022402,cell cycle process|GO:0007275,multicellular organism development|GO:0001649,osteoblast differentiation|GO:0009888,tissue development|GO:0030218,erythrocyte differentiation|GO:0002244,hematopoietic progenitor cell differentiation|GO:0051301,cell division|GO:2000026,regulation of multicellular organismal development|GO:0048534,hematopoietic or lymphoid organ development|GO:0010468,regulation of gene expression|GO:0034101,erythrocyte homeostasis|GO:0006468,protein phosphorylation|GO:0009987,cellular process|GO:0000278,mitotic cell cycle|GO:0010810,regulation of cell-substrate adhesion|GO:0034109,homotypic cell-cell adhesion|GO:0006793,phosphorus metabolic process|GO:0006464,cellular protein modification process|GO:0044767,single-organism developmental process|GO:0007159,leukocyte cell-cell adhesion|GO:0007155,cell adhesion|GO:0044843,cell cycle G1/S phase transition|GO:0022008,neurogenesis|GO:0070486,leukocyte aggregation|GO:0051179,localization|GO:0040011,locomotion|GO:0044238,primary metabolic process|GO:0048699,generation of neurons|GO:0040012,regulation of locomotion|GO:0007399,nervous system development|GO:0051726,regulation of cell cycle|GO:0090398,cellular senescence|GO:0044237,cellular metabolic process|GO:0006796,phosphate-containing compound metabolic process|GO:0030900,forebrain development|GO:0003323,type B pancreatic cell development|GO:0030154,cell differentiation|GO:0002067,glandular epithelial cell differentiation|GO:0048523,negative regulation of cellular process|GO:0048522,positive regulation of cellular process|GO:2000736,regulation of stem cell differentiation|GO:0080090,regulation of primary metabolic process|GO:0000086,G2/M transition of mitotic cell cycle|GO:0030224,monocyte differentiation|GO:1901362,organic cyclic compound biosynthetic process|GO:1901360,organic cyclic compound metabolic process|GO:0032774,RNA biosynthetic process|GO:0010605,negative regulation of macromolecule metabolic process|GO:0071310,cellular response to organic substance|GO:0010033,response to organic substance|GO:1904628,cellular response to phorbol 13-acetate 12-myristate|GO:0034645,cellular macromolecule biosynthetic	GO:0000307,cyclin-dependent protein kinase holoenzyme complex|GO:0031974,membrane-enclosed lumen|GO:0043229,intracellular organelle|GO:0043228,non-membrane-bounded organelle|GO:0043227,membrane-bounded organelle|GO:0043226,organelle|GO:0005856,cytoskeleton|GO:0001726,ruffle|GO:0031981,nuclear lumen|GO:0005813,centrosome|GO:0005634,nucleus|GO:0005815,microtubule organizing center|GO:0005654,nucleoplasm|GO:0044430,cytoskeletal part|GO:0005737,cytoplasm|GO:0015630,microtubule cytoskeleton|GO:1902494,catalytic complex|GO:0061695,transferase complex, transferring phosphorus-containing groups|GO:0031252,cell leading edge|GO:1990234,transferase complex|GO:0042995,cell projection|GO:0043234,protein complex|GO:0032991,macromolecular complex|GO:0043231,intracellular membrane-bounded organelle|GO:0043232,intracellular non-membrane-bounded organelle|GO:0043233,organelle lumen|GO:0005829,cytosol|GO:0044464,cell part|GO:0005623,cell|GO:0005622,intracellular|GO:0044446,intracellular organelle part|GO:0070013,intracellular organelle lumen|GO:0044444,cytoplasmic part|GO:0044428,nuclear part|GO:1902911,protein kinase complex|GO:0044424,intracellular part|GO:0044422,organelle part|GO:0097132,cyclin D2-CDK6 complex|GO:1902554,serine/threonine protein kinase complex	GO:0035639,purine ribonucleoside triphosphate binding|GO:0004672,protein kinase activity|GO:0004693,cyclin-dependent protein serine/threonine kinase activity|GO:0043167,ion binding|GO:1901363,heterocyclic compound binding|GO:0001883,purine nucleoside binding|GO:0001882,nucleoside binding|GO:0043168,anion binding|GO:0004674,protein serine/threonine kinase activity|GO:0000166,nucleotide binding|GO:0097472,cyclin-dependent protein kinase activity|GO:0016740,transferase activity|GO:1901265,nucleoside phosphate binding|GO:0032549,ribonucleoside binding|GO:0017076,purine nucleotide binding|GO:0005524,ATP binding|GO:0030332,cyclin binding|GO:0016301,kinase activity|GO:0036094,small molecule binding|GO:0003824,catalytic activity|GO:0030554,adenyl nucleotide binding|GO:0016773,phosphotransferase activity, alcohol group as acceptor|GO:0016772,transferase activity, transferring phosphorus-containing groups|GO:0097367,carbohydrate derivative binding|GO:0032559,adenyl ribonucleotide binding|GO:0005515,protein binding|GO:0097159,organic cyclic compound binding|GO:0032555,purine ribonucleotide binding|GO:0032550,purine ribonucleoside binding|GO:0032553,ribonucleotide binding|GO:0005488,binding|GO:0098770,FBXO family protein binding

ADH1B	GO:0071704,organic substance metabolic process|GO:0044710,single-organism metabolic process|GO:0008152,metabolic process|GO:0006805,xenobiotic metabolic process|GO:0044699,single-organism process|GO:0009410,response to xenobiotic stimulus|GO:0009987,cellular process|GO:0051716,cellular response to stimulus|GO:0044237,cellular metabolic process|GO:0006066,alcohol metabolic process|GO:0006067,ethanol metabolic process|GO:0071466,cellular response to xenobiotic stimulus|GO:0044281,small molecule metabolic process|GO:0070887,cellular response to chemical stimulus|GO:0042221,response to chemical|GO:0034308,primary alcohol metabolic process|GO:1901615,organic hydroxy compound metabolic process|GO:0050896,response to stimulus|GO:0055114,oxidation-reduction process|GO:0006069,ethanol oxidation	GO:0005737,cytoplasm|GO:0005829,cytosol|GO:0044464,cell part|GO:0005623,cell|GO:0005622,intracellular|GO:0044444,cytoplasmic part|GO:0044424,intracellular part|GO:0031974,membrane-enclosed lumen|GO:0043229,intracellular organelle|GO:0071944,cell periphery|GO:0043227,membrane-bounded organelle|GO:0043226,organelle|GO:0031981,nuclear lumen|GO:0005634,nucleus|GO:0016020,membrane|GO:0005654,nucleoplasm|GO:0005886,plasma membrane|GO:0043231,intracellular membrane-bounded organelle|GO:0043233,organelle lumen|GO:0044446,intracellular organelle part|GO:0070013,intracellular organelle lumen|GO:0044428,nuclear part|GO:0044422,organelle part	GO:0005488,binding|GO:0043169,cation binding|GO:0016614,oxidoreductase activity, acting on CH-OH group of donors|GO:0046914,transition metal ion binding|GO:0016616,oxidoreductase activity, acting on the CH-OH group of donors, NAD or NADP as acceptor|GO:0004024,alcohol dehydrogenase activity, zinc-dependent|GO:0004022,alcohol dehydrogenase (NAD) activity|GO:0043167,ion binding|GO:0003824,catalytic activity|GO:0008270,zinc ion binding|GO:0046872,metal ion binding|GO:0016491,oxidoreductase activity
GRIN2B	GO:0051234,establishment of localization|GO:0007612,learning|GO:0007613,memory|GO:0007610,behavior|GO:0007611,learning or memory|GO:0007165,signal transduction|GO:0007166,cell surface receptor signaling pathway|GO:0007167,enzyme linked receptor protein signaling pathway|GO:0007169,transmembrane receptor protein tyrosine kinase signaling pathway|GO:0048266,behavioral response to pain|GO:0071840,cellular component organization or biogenesis|GO:0048265,response to pain|GO:0051716,cellular response to stimulus|GO:0042330,taxis|GO:0009966,regulation of signal transduction|GO:0048869,cellular developmental process|GO:0009612,response to mechanical stimulus|GO:0007215,glutamate receptor signaling pathway|GO:0048513,animal organ development|GO:0048518,positive regulation of biological process|GO:0051704,multi-organism process|GO:0006935,chemotaxis|GO:0048583,regulation of response to stimulus|GO:0007600,sensory perception|GO:0042221,response to chemical|GO:0070838,divalent metal ion transport|GO:0097485,neuron projection guidance|GO:0010033,response to organic substance|GO:0051705,multi-organism behavior|GO:0003008,system process|GO:0044700,single organism signaling|GO:0009605,response to external stimulus|GO:0044707,single-multicellular organism process|GO:0044708,single-organism behavior|GO:0070588,calcium ion transmembrane transport|GO:0010737,protein kinase A signaling|GO:0033555,multicellular organismal response to stress|GO:0010738,regulation of protein kinase A signaling|GO:0007631,feeding behavior|GO:0009581,detection of external stimulus|GO:0006928,movement of cell or subcellular component|GO:0048167,regulation of synaptic plasticity|GO:0031175,neuron projection development|GO:0035556,intracellular signal transduction|GO:0050789,regulation of biological process|GO:0000904,cell morphogenesis involved in differentiation|GO:0000902,cell morphogenesis|GO:0016043,cellular component organization|GO:0001967,suckling behavior|GO:0001964,startle response|GO:0002209,behavioral defense response|GO:0065007,biological regulation|GO:0098662,inorganic cation transmembrane transport|GO:0098660,inorganic ion transmembrane transport|GO:0019233,sensory perception of pain|GO:0065008,regulation of biological quality|GO:0061564,axon development|GO:0006812,cation transport|GO:0006811,ion transport|GO:0006810,transport|GO:0006816,calcium ion transport|GO:0048468,cell development|GO:0050794,regulation of cellular process|GO:0006952,defense response|GO:0006950,response to stress|GO:0060079,excitatory postsynaptic potential|GO:0060078,regulation of postsynaptic membrane potential|GO:0048013,ephrin receptor signaling pathway|GO:1902531,regulation of intracellular signal transduction|GO:0051606,detection of stimulus|GO:0007423,sensory organ development|GO:0045471,response to ethanol|GO:0050896,response to stimulus|GO:0050890,cognition|GO:0048812,neuron projection morphogenesis|GO:0097305,response to alcohol|GO:0050966,detection of mechanical stimulus involved in sensory perception of pain|GO:0042391,regulation of membrane potential|GO:0050803,regulation of synapse structure or activity|GO:0030154,cell differentiation|GO:0050806,positive regulation of synaptic transmission|GO:0050804,modulation of synaptic transmission|GO:0023056,positive regulation of signaling|GO:0009790,embryo development|GO:0009792,embryo development ending in birth or egg hatching|GO:0023052,signaling|GO:0023051,regulation of signaling|GO:0007411,axon guidance|GO:0010647,positive regulation of cell communication|GO:0010646,regulation of cell communication|GO:0044699,single-organism process|GO:0001662,behavioral fear response|GO:0001701,in utero embryonic development|GO:0009653,anatomical structure morphogenesis|GO:0043009,chordate embryonic development|GO:0032502,developmental process|GO:1901700,response to oxygen-containing compound|GO:0050974,detection of mechanical stimulus involved in sensory perception|GO:0035235,ionotropic glutamate receptor signaling pathway|GO:0050877,neurological system process|GO:0009987,cellular process|GO:0030001,metal ion transport|GO:0007409,axonogenesis|GO:0055085,transmembrane transport|GO:0048858,cell projection morphogenesis|GO:0050954,sensory perception of mechanical stimulus|GO:0072511,divalent inorganic cation transport|GO:0060291,long-term synaptic potentiation|GO:0048731,system development|GO:0030030,cell projection organization|GO:0032501,multicellular organismal process|GO:0050906,detection of stimulus involved in sensory perception|GO:0050905,neuromuscular process|GO:0007275,multicellular organism development|GO:0050982,detection of mechanical stimulus|GO:0042596,fear response|GO:0032989,cellular component morphogenesis|GO:0048666,neuron development|GO:0048667,cell morphogenesis involved in neuron differentiation|GO:0009582,detection of abiotic stimulus|GO:0030182,neuron differentiation|GO:0044767,single-organism developmental process|GO:0034220,ion transmembrane transport|GO:0044765,single-organism transport|GO:0044763,single-organism cellular process|GO:0007268,chemical synaptic transmission|GO:0007267,cell-cell signaling|GO:0007154,cell communication|GO:0022008,neurogenesis|GO:0051179,localization|GO:1902578,single-organism localization|GO:0040011,locomotion|GO:0009628,response to abiotic stimulus|GO:0048699,generation of neurons|GO:0032990,cell part morphogenesis|GO:0007399,nervous system development|GO:0048856,anatomical structure development|GO:0098655,cation transmembrane transport|GO:0048522,positive regulation of cellular process|GO:0051049,regulation of transport|GO:0006468,protein phosphorylation|GO:0006807,nitrogen compound metabolic process|GO:0043087,regulation of GTPase activity|GO:1905114,cell surface receptor signaling pathway involved in cell-cell signaling|GO:0023014,signal transduction by protein phosphorylation|GO:0044267,cellular protein metabolic process|GO:0044710,single-organism metabolic process|GO:0043547,positive regulation of GTPase activity|GO:0051345,positive regulation of hydrolase activity|GO:0000165,MAPK cascade|GO:0006796,phosphate-containing compound metabolic process|GO:0071704,organic substance metabolic process|GO:0034762,regulation of transmembrane transport|GO:0044093,positive regulation of molecular function|GO:0016310,phosphorylation|GO:0065009,regulation of molecular function|GO:1901564,organonitrogen compound metabolic process|GO:0043085,positive regulation of catalytic activity|GO:0099565,chemical synaptic transmission, postsynaptic|GO:0044238,primary metabolic process|GO:0050790,regulation of catalytic activity|GO:0006464,cellular protein modification process|GO:0036211,protein modification process|GO:0008152,metabolic process|GO:0032879,regulation of localization|GO:0051336,regulation of hydrolase activity|GO:0034765,regulation of ion transmembrane transport|GO:0044260,cellular macromolecule metabolic process|GO:0019538,protein metabolic process|GO:0043412,macromolecule modification|GO:0044237,cellular metabolic process|GO:0043170,macromolecule metabolic process|GO:0098916,anterograde trans-synaptic signaling|GO:0043269,regulation of ion transport|GO:0006793,phosphorus metabolic process|GO:0099536,synaptic signaling|GO:0099537,trans-synaptic signaling|GO:0006875,cellular metal ion homeostasis|GO:0022607,cellular component assembly|GO:0080090,regulation of primary metabolic process|GO:0019222,regulation of metabolic process|GO:0070271,protein complex biogenesis|GO:0043933,macromolecular complex subunit organization|GO:0043954,cellular component maintenance|GO:0031325,positive regulation of cellular metabolic process|GO:0051649,establishment of localization in cell|GO:0031323,regulation of cellular metabolic process|GO:2001056,positive regulation of cysteine-type endopeptidase activity|GO:0051128,regulation of cellular component organization|GO:0042592,homeostatic process|GO:0098771,inorganic ion homeostasis|GO:0060322,head development|GO:0055074,calcium ion homeostasis|GO:0051641,cellular localization|GO:0010942,positive regulation of cell death|GO:0008219,cell death|GO:0010941,regulation of cell death|GO:0031345,negative regulation of cell projection organization|GO:0031344,regulation of cell projection organization|GO:0009893,positive regulation of metabolic process|GO:0097061,dendritic spine organization|GO:0097062,dendritic spine maintenance|GO:0010604,positive regulation of macromolecule metabolic process|GO:0071822,protein complex subunit organization|GO:2000116,regulation of cysteine-type	GO:0008021,synaptic vesicle|GO:0098590,plasma membrane region|GO:0043229,intracellular organelle|GO:0071944,cell periphery|GO:0034702,ion channel complex|GO:0045202,synapse|GO:0043227,membrane-bounded organelle|GO:0043226,organelle|GO:0030054,cell junction|GO:0097060,synaptic membrane|GO:0031982,vesicle|GO:0016023,cytoplasmic, membrane-bounded vesicle|GO:0031410,cytoplasmic vesicle|GO:0016021,integral component of membrane|GO:0016020,membrane|GO:0098802,plasma membrane receptor complex|GO:0031988,membrane-bounded vesicle|GO:0097458,neuron part|GO:0044456,synapse part|GO:0031224,intrinsic component of membrane|GO:0043005,neuron projection|GO:0044425,membrane part|GO:0044459,plasma membrane part|GO:0005737,cytoplasm|GO:0031226,intrinsic component of plasma membrane|GO:1902495,transmembrane transporter complex|GO:0008328,ionotropic glutamate receptor complex|GO:0009986,cell surface|GO:0098589,membrane region|GO:1990351,transporter complex|GO:0045211,postsynaptic membrane|GO:0014069,postsynaptic density|GO:0005887,integral component of plasma membrane|GO:0005886,plasma membrane|GO:0042995,cell projection|GO:0097481,neuronal postsynaptic density|GO:0043234,protein complex|GO:0043235,receptor complex|GO:0032991,macromolecular complex|GO:0043231,intracellular membrane-bounded organelle|GO:0044463,cell projection part|GO:0044464,cell part|GO:0005623,cell|GO:0005622,intracellular|GO:0098797,plasma membrane protein complex|GO:0098796,membrane protein complex|GO:0044444,cytoplasmic part|GO:0098794,postsynapse|GO:0098793,presynapse|GO:0044424,intracellular part|GO:0017146,NMDA selective glutamate receptor complex|GO:0098805,whole membrane|GO:0098878,neurotransmitter receptor complex|GO:0034703,cation channel complex|GO:0098984,neuron to neuron synapse|GO:0043228,non-membrane-bounded organelle|GO:0043232,intracellular non-membrane-bounded organelle|GO:0032279,asymmetric synapse|GO:0099572,postsynaptic specialization	GO:0005488,binding|GO:0038023,signaling receptor activity|GO:0005262,calcium channel activity|GO:0060089,molecular transducer activity|GO:0008066,glutamate receptor activity|GO:0004872,receptor activity|GO:0004871,signal transducer activity|GO:0005261,cation channel activity|GO:0043167,ion binding|GO:0004972,NMDA glutamate receptor activity|GO:0004970,ionotropic glutamate receptor activity|GO:0016594,glycine binding|GO:0043168,anion binding|GO:0015085,calcium ion transmembrane transporter activity|GO:0046914,transition metal ion binding|GO:0016597,amino acid binding|GO:0015276,ligand-gated ion channel activity|GO:0072509,divalent inorganic cation transmembrane transporter activity|GO:0022857,transmembrane transporter activity|GO:0022803,passive transmembrane transporter activity|GO:0046873,metal ion transmembrane transporter activity|GO:0046872,metal ion binding|GO:0005216,ion channel activity|GO:0005231,excitatory extracellular ligand-gated ion channel activity|GO:0043169,cation binding|GO:0005215,transporter activity|GO:0005230,extracellular ligand-gated ion channel activity|GO:0008324,cation transmembrane transporter activity|GO:0005234,extracellular-glutamate-gated ion channel activity|GO:0036094,small molecule binding|GO:0022891,substrate-specific transmembrane transporter activity|GO:0022890,inorganic cation transmembrane transporter activity|GO:0022892,substrate-specific transporter activity|GO:0043177,organic acid binding|GO:0031406,carboxylic acid binding|GO:0015075,ion transmembrane transporter activity|GO:0004888,transmembrane signaling receptor activity|GO:0015267,channel activity|GO:0022836,gated channel activity|GO:0005515,protein binding|GO:0022834,ligand-gated channel activity|GO:0022838,substrate-specific channel activity|GO:0008270,zinc ion binding|GO:0005088,Ras guanyl-nucleotide exchange factor activity|GO:0005085,guanyl-nucleotide exchange factor activity|GO:0098772,molecular function regulator|GO:0022824,transmitter-gated ion channel activity|GO:0030594,neurotransmitter receptor activity|GO:0022832,voltage-gated channel activity|GO:0022835,transmitter-gated channel activity|GO:0005244,voltage-gated ion channel activity|GO:0099600,transmembrane receptor activity|GO:0099604,ligand-gated calcium channel activity|GO:0016595,glutamate binding|GO:0022849,glutamate-gated calcium ion channel activity
CLVS2	GO:0061024,membrane organization|GO:0007040,lysosome organization|GO:0071840,cellular component organization or biogenesis|GO:0016043,cellular component organization|GO:0044699,single-organism process|GO:0007033,vacuole organization|GO:0009987,cellular process|GO:0048193,Golgi vesicle transport|GO:0006810,transport|GO:0044765,single-organism transport|GO:0051649,establishment of localization in cell|GO:0051234,establishment of localization|GO:0051179,localization|GO:1902578,single-organism localization|GO:0051641,cellular localization|GO:0006996,organelle organization|GO:0006892,post-Golgi vesicle-mediated transport|GO:0016192,vesicle-mediated transport|GO:0046907,intracellular transport|GO:0080171,lytic vacuole organization|GO:1902582,single-organism intracellular transport	GO:0030139,endocytic vesicle|GO:0031984,organelle subcompartment|GO:0043229,intracellular organelle|GO:0005622,intracellular|GO:0030135,coated vesicle|GO:0043226,organelle|GO:0030136,clathrin-coated vesicle|GO:0030665,clathrin-coated vesicle membrane|GO:0030666,endocytic vesicle membrane|GO:0031982,vesicle|GO:0030662,coated vesicle membrane|GO:0016023,cytoplasmic, membrane-bounded vesicle|GO:0031410,cytoplasmic vesicle|GO:0098805,whole membrane|GO:0016020,membrane|GO:0030669,clathrin-coated endocytic vesicle membrane|GO:0044444,cytoplasmic part|GO:0031988,membrane-bounded vesicle|GO:0044433,cytoplasmic vesicle part|GO:0044431,Golgi apparatus part|GO:0005737,cytoplasm|GO:0010008,endosome membrane|GO:0031901,early endosome membrane|GO:0005794,Golgi apparatus|GO:0044440,endosomal part|GO:0098588,bounding membrane of organelle|GO:0030659,cytoplasmic vesicle membrane|GO:0032588,trans-Golgi network membrane|GO:0045334,clathrin-coated endocytic vesicle|GO:0012505,endomembrane system|GO:0012506,vesicle membrane|GO:0031090,organelle membrane|GO:0043231,intracellular membrane-bounded organelle|GO:0005802,trans-Golgi network|GO:0044464,cell part|GO:0005623,cell|GO:0000139,Golgi membrane|GO:0044446,intracellular organelle part|GO:0043227,membrane-bounded organelle|GO:0098791,Golgi subcompartment|GO:0044424,intracellular part|GO:0005768,endosome|GO:0005769,early	GO:0043168,anion binding|GO:0080025,phosphatidylinositol-3,5-bisphosphate binding|GO:0035091,phosphatidylinositol binding|GO:0005215,transporter activity|GO:0005543,phospholipid binding|GO:0008289,lipid binding|GO:0043167,ion binding|GO:1902936,phosphatidylinositol bisphosphate binding|GO:0005488,binding|GO:1901981,phosphatidylinositol phosphate binding
C10orf118			
LPP		GO:0005737,cytoplasm|GO:0070161,anchoring junction|GO:0044464,cell part|GO:0005623,cell|GO:0005622,intracellular|GO:0005912,adherens junction|GO:0044424,intracellular part|GO:0005924,cell-substrate adherens junction|GO:0005925,focal adhesion|GO:0030054,cell junction|GO:0030055,cell-substrate junction|GO:0005829,cytosol|GO:0044444,cytoplasmic part	
POU3F4	GO:0080090,regulation of primary metabolic process|GO:0019222,regulation of metabolic process|GO:0060322,head development|GO:1901362,organic cyclic compound biosynthetic process|GO:1901360,organic cyclic compound metabolic process|GO:0071704,organic substance metabolic process|GO:0048869,cellular developmental process|GO:0010467,gene expression|GO:0048513,animal organ development|GO:0048519,negative regulation of biological process|GO:0046483,heterocycle metabolic process|GO:0060255,regulation of macromolecule metabolic process|GO:0060548,negative regulation of cell death|GO:0021879,forebrain neuron differentiation|GO:0007600,sensory perception|GO:2001141,regulation of RNA biosynthetic process|GO:0003008,system process|GO:0044707,single-multicellular organism process|GO:0044249,cellular biosynthetic process|GO:0021872,forebrain generation of neurons|GO:0097285,obsolete cell-type specific apoptotic process|GO:0019438,aromatic compound biosynthetic process|GO:0048562,embryonic organ morphogenesis|GO:0048568,embryonic organ development|GO:0006807,nitrogen compound metabolic process|GO:0042981,regulation of apoptotic process|GO:0050789,regulation of biological process|GO:0097659,nucleic acid-templated transcription|GO:1901576,organic substance biosynthetic process|GO:0090102,cochlea development|GO:0090103,cochlea morphogenesis|GO:0044260,cellular macromolecule metabolic process|GO:0065007,biological regulation|GO:0006366,transcription from RNA polymerase II promoter|GO:0018130,heterocycle biosynthetic process|GO:0016265,obsolete death|GO:0009887,animal organ morphogenesis|GO:0050954,sensory perception of mechanical stimulus|GO:0009889,regulation of biosynthetic process|GO:0050794,regulation of cellular process|GO:0012501,programmed cell death|GO:0008152,metabolic process|GO:0034654,nucleobase-containing compound biosynthetic process|GO:0016070,RNA metabolic process|GO:0097152,mesenchymal cell apoptotic process|GO:0044271,cellular nitrogen compound biosynthetic process|GO:0007420,brain development|GO:0007423,sensory organ development|GO:0006355,regulation of transcription, DNA-templated|GO:0006357,regulation of transcription from RNA polymerase II promoter|GO:0006351,transcription, DNA-templated|GO:0032774,RNA biosynthetic process|GO:0030154,cell differentiation|GO:0009790,embryo development|GO:0034641,cellular nitrogen compound metabolic process|GO:0034645,cellular macromolecule biosynthetic process|GO:0009653,anatomical structure morphogenesis|GO:0044699,single-organism process|GO:0007417,central nervous system development|GO:0006139,nucleobase-containing compound metabolic process|GO:0021953,central nervous system neuron differentiation|GO:0032502,developmental process|GO:0032501,multicellular organismal process|GO:0010556,regulation of macromolecule biosynthetic process|GO:2001054,negative regulation of mesenchymal cell apoptotic process|GO:0009987,cellular process|GO:0006725,cellular aromatic compound metabolic process|GO:1903506,regulation of nucleic acid-templated transcription|GO:0048839,inner ear development|GO:0051252,regulation of RNA metabolic process|GO:0043170,macromolecule metabolic process|GO:0048731,system development|GO:0031326,regulation of cellular biosynthetic process|GO:2001053,regulation of mesenchymal cell apoptotic process|GO:0031323,regulation of cellular metabolic process|GO:0090304,nucleic acid metabolic process|GO:0008219,cell death|GO:0010941,regulation of cell death|GO:0007275,multicellular organism development|GO:2000112,regulation of cellular macromolecule biosynthetic process|GO:0007605,sensory perception of sound|GO:0043067,regulation of programmed cell death|GO:0043066,negative regulation of apoptotic process|GO:0043583,ear development|GO:0050877,neurological system process|GO:0043069,negative regulation of programmed cell death|GO:0010468,regulation of gene expression|GO:0090596,sensory organ morphogenesis|GO:0048598,embryonic morphogenesis|GO:0030182,neuron differentiation|GO:0019219,regulation of nucleobase-containing compound metabolic process|GO:0006915,apoptotic process|GO:0044767,single-organism developmental process|GO:0009058,biosynthetic process|GO:0009059,macromolecule biosynthetic process|GO:0044763,single-organism cellular process|GO:0051171,regulation of nitrogen compound metabolic process|GO:0022008,neurogenesis|GO:0042471,ear morphogenesis|GO:0044238,primary metabolic process|GO:0048699,generation of neurons|GO:0042472,inner ear morphogenesis|GO:0007399,nervous system development|GO:0048856,anatomical structure development|GO:0044237,cellular metabolic process|GO:0030900,forebrain development|GO:0048523,negative regulation of cellular process	GO:0043231,intracellular membrane-bounded organelle|GO:0005634,nucleus|GO:0044464,cell part|GO:0005623,cell|GO:0005622,intracellular|GO:0043229,intracellular organelle|GO:0044424,intracellular part|GO:0043227,membrane-bounded organelle|GO:0043226,organelle	GO:0044212,transcription regulatory region DNA binding|GO:0043566,structure-specific DNA binding|GO:0001071,nucleic acid binding transcription factor activity|GO:0043565,sequence-specific DNA binding|GO:0003700,transcription factor activity, sequence-specific DNA binding|GO:0001012,RNA polymerase II regulatory region DNA binding|GO:0003677,DNA binding|GO:0001067,regulatory region nucleic acid binding|GO:0000976,transcription regulatory region sequence-specific DNA binding|GO:0000977,RNA polymerase II regulatory region sequence-specific DNA binding|GO:0003676,nucleic acid binding|GO:0000975,regulatory region DNA binding|GO:0003680,AT DNA binding|GO:0003690,double-stranded DNA binding|GO:0097159,organic cyclic compound binding|GO:1901363,heterocyclic compound binding|GO:0005488,binding|GO:1990837,sequence-specific double-stranded DNA binding|GO:0000981,RNA polymerase II transcription factor activity, sequence-specific DNA binding
GRIA4	GO:0035249,synaptic transmission, glutamatergic|GO:0023052,signaling|GO:0007165,signal transduction|GO:0007166,cell surface receptor signaling pathway|GO:0007270,neuron-neuron synaptic transmission|GO:0051179,localization|GO:0050789,regulation of biological process|GO:0044699,single-organism process|GO:0051716,cellular response to stimulus|GO:0007215,glutamate receptor signaling pathway|GO:0065007,biological regulation|GO:0006810,transport|GO:0035235,ionotropic glutamate receptor signaling pathway|GO:0006811,ion transport|GO:0009987,cellular process|GO:0050794,regulation of cellular process|GO:0034220,ion transmembrane transport|GO:0044765,single-organism transport|GO:0044763,single-organism cellular process|GO:0007268,chemical synaptic transmission|GO:0007267,cell-cell signaling|GO:0007154,cell communication|GO:0051234,establishment of localization|GO:0055085,transmembrane transport|GO:1902578,single-organism localization|GO:0044700,single organism signaling|GO:0050896,response to stimulus|GO:0042391,regulation of membrane potential|GO:1905114,cell surface receptor signaling pathway involved in cell-cell signaling|GO:0060079,excitatory postsynaptic potential|GO:0065008,regulation of biological quality|GO:0099565,chemical synaptic transmission, postsynaptic|GO:0060078,regulation of postsynaptic membrane potential|GO:0098916,anterograde trans-synaptic signaling|GO:0099536,synaptic signaling|GO:0099537,trans-synaptic signaling	GO:0098590,plasma membrane region|GO:0042995,cell projection|GO:0032281,AMPA glutamate receptor complex|GO:0005887,integral component of plasma membrane|GO:0044421,extracellular region part|GO:0030425,dendrite|GO:0030139,endocytic vesicle|GO:0043229,intracellular organelle|GO:0071944,cell periphery|GO:0034702,ion channel complex|GO:0045202,synapse|GO:0043227,membrane-bounded organelle|GO:0043226,organelle|GO:0030054,cell junction|GO:0097060,synaptic membrane|GO:0098797,plasma membrane protein complex|GO:0030666,endocytic vesicle membrane|GO:0031982,vesicle|GO:0016023,cytoplasmic, membrane-bounded vesicle|GO:0031410,cytoplasmic vesicle|GO:0016021,integral component of membrane|GO:0016020,membrane|GO:0098802,plasma membrane receptor complex|GO:0031988,membrane-bounded vesicle|GO:0044433,cytoplasmic vesicle part|GO:0097458,neuron part|GO:0044456,synapse part|GO:0031224,intrinsic component of membrane|GO:0043005,neuron projection|GO:0044459,plasma membrane part|GO:0005576,extracellular region|GO:0005737,cytoplasm|GO:0031226,intrinsic component of plasma membrane|GO:1902495,transmembrane transporter complex|GO:0008328,ionotropic glutamate receptor complex|GO:0098588,bounding membrane of organelle|GO:0098589,membrane region|GO:1990351,transporter complex|GO:0030659,cytoplasmic vesicle membrane|GO:0036477,somatodendritic compartment|GO:0012506,vesicle membrane|GO:0005886,plasma membrane|GO:0032991,macromolecular complex|GO:1903561,extracellular vesicle|GO:0031090,organelle membrane|GO:0043234,protein complex|GO:0043235,receptor complex|GO:0043230,extracellular organelle|GO:0043231,intracellular membrane-bounded organelle|GO:0045211,postsynaptic membrane|GO:0044464,cell part|GO:0005623,cell|GO:0005622,intracellular|GO:0044446,intracellular organelle part|GO:0098796,membrane protein complex|GO:0044444,cytoplasmic part|GO:0098794,postsynapse|GO:0044424,intracellular part|GO:0044425,membrane part|GO:0098805,whole membrane|GO:0044422,organelle part|GO:0097708,intracellular vesicle|GO:0098878,neurotransmitter receptor complex|GO:0034703,cation channel complex|GO:0044297,cell	GO:0038023,signaling receptor activity|GO:0060089,molecular transducer activity|GO:0008066,glutamate receptor activity|GO:0004872,receptor activity|GO:0004871,signal transducer activity|GO:0004970,ionotropic glutamate receptor activity|GO:0004971,AMPA glutamate receptor activity|GO:0015276,ligand-gated ion channel activity|GO:0022857,transmembrane transporter activity|GO:0022803,passive transmembrane transporter activity|GO:0005216,ion channel activity|GO:0005215,transporter activity|GO:0005230,extracellular ligand-gated ion channel activity|GO:0005231,excitatory extracellular ligand-gated ion channel activity|GO:0005234,extracellular-glutamate-gated ion channel activity|GO:0022891,substrate-specific transmembrane transporter activity|GO:0022892,substrate-specific transporter activity|GO:0015075,ion transmembrane transporter activity|GO:0004888,transmembrane signaling receptor activity|GO:0015267,channel activity|GO:0022836,gated channel activity|GO:0022834,ligand-gated channel activity|GO:0022838,substrate-specific channel activity|GO:0022824,transmitter-gated ion channel activity|GO:0030594,neurotransmitter receptor activity|GO:0022835,transmitter-gated channel activity|GO:0099600,transmembrane receptor activity
EDN1	GO:0007599,hemostasis|GO:0051046,regulation of secretion|GO:0051047,positive regulation of secretion|GO:0051048,negative regulation of secretion|GO:0051049,regulation of transport|GO:0046887,positive regulation of hormone secretion|GO:0001501,skeletal system development|GO:0009165,nucleotide biosynthetic process|GO:0044281,small molecule metabolic process|GO:0044283,small molecule biosynthetic process|GO:0098771,inorganic ion homeostasis|GO:0044321,response to leptin|GO:0014032,neural crest cell development|GO:0014033,neural crest cell differentiation|GO:0051716,cellular response to stimulus|GO:0014031,mesenchymal cell development|GO:0000003,reproduction|GO:0043207,response to external biotic stimulus|GO:0001666,response to hypoxia|GO:0051495,positive regulation of cytoskeleton organization|GO:0000165,MAPK cascade|GO:0034694,response to prostaglandin|GO:0034696,response to prostaglandin F|GO:0007088,regulation of mitotic nuclear division|GO:0030595,leukocyte chemotaxis|GO:0007596,blood coagulation|GO:0030593,neutrophil chemotaxis|GO:0060548,negative regulation of cell death|GO:0045859,regulation of protein kinase activity|GO:0019229,regulation of vasoconstriction|GO:0046330,positive regulation of JNK cascade|GO:0071559,response to transforming growth factor beta|GO:0009628,response to abiotic stimulus|GO:0046483,heterocycle metabolic process|GO:0010634,positive regulation of epithelial cell migration|GO:0042327,positive regulation of phosphorylation|GO:0009607,response to biotic stimulus|GO:0044062,regulation of excretion|GO:0010632,regulation of epithelial cell migration|GO:0019538,protein metabolic process|GO:0010638,positive regulation of organelle organization|GO:0051783,regulation of nuclear division|GO:0051781,positive regulation of cell division|GO:0045823,positive regulation of heart contraction|GO:0051785,positive regulation of nuclear division|GO:0042692,muscle cell differentiation|GO:0033993,response to lipid|GO:0070542,response to fatty acid|GO:0016265,obsolete death|GO:0048562,embryonic organ morphogenesis|GO:0009892,negative regulation of metabolic process|GO:0009893,positive regulation of metabolic process|GO:0009890,negative regulation of biosynthetic process|GO:0009891,positive regulation of biosynthetic process|GO:0006950,response to stress|GO:0048568,embryonic organ development|GO:0000302,response to reactive oxygen species|GO:0010869,regulation of receptor biosynthetic process|GO:0071902,positive regulation of protein serine/threonine kinase activity|GO:0035556,intracellular signal transduction|GO:0071900,regulation of protein serine/threonine kinase activity|GO:0050789,regulation of biological process|GO:0030072,peptide hormone secretion|GO:0035994,response to muscle stretch|GO:0051341,regulation of oxidoreductase activity|GO:0051347,positive regulation of transferase activity|GO:0008645,hexose transport|GO:0032310,prostaglandin secretion|GO:0008643,carbohydrate transport|GO:0002687,positive regulation of leukocyte migration|GO:0002684,positive regulation of immune system process|GO:0002685,regulation of leukocyte migration|GO:0002682,regulation of immune system process|GO:1901360,organic cyclic compound metabolic process|GO:0022603,regulation of anatomical structure morphogenesis|GO:0002688,regulation of leukocyte chemotaxis|GO:0018130,heterocycle biosynthetic process|GO:2000379,positive regulation of reactive oxygen species metabolic process|GO:0006629,lipid metabolic process|GO:0043200,response to amino acid|GO:0015833,peptide transport|GO:2000377,regulation of reactive oxygen species metabolic process|GO:0043410,positive regulation of MAPK cascade|GO:0009260,ribonucleotide biosynthetic process|GO:1903524,positive regulation of blood circulation|GO:1903522,regulation of blood circulation|GO:0071277,cellular response to calcium ion|GO:0032956,regulation of actin cytoskeleton organization|GO:0032890,regulation of organic acid transport|GO:0060760,positive regulation of response to cytokine stimulus|GO:0032892,positive regulation of organic acid transport|GO:0016070,RNA metabolic process|GO:0050817,coagulation|GO:0071345,cellular response to cytokine stimulus|GO:0071347,cellular response to interleukin-1|GO:0071346,cellular response to interferon-gamma|GO:0035094,response to nicotine|GO:0033574,response to testosterone|GO:0006690,icosanoid metabolic process|GO:0010557,positive regulation of macromolecule biosynthetic process|GO:0010556,regulation of macromolecule biosynthetic process|GO:1901293,nucleoside phosphate biosynthetic process|GO:0048869,cellular developmental process|GO:0006753,nucleoside phosphate metabolic process|GO:0051384,response to glucocorticoid|GO:0010558,negative regulation of macromolecule biosynthetic process|GO:0071248,cellular response to metal ion|GO:0051493,regulation of cytoskeleton organization|GO:0071241,cellular response to inorganic substance|GO:0051128,regulation of cellular component organization|GO:0009187,cyclic nucleotide metabolic process|GO:0014070,response to organic cyclic compound|GO:0030818,negative regulation of cAMP biosynthetic process|GO:0000122,negative regulation of transcription from RNA polymerase II promoter|GO:0042481,regulation of odontogenesis|GO:0042482,positive regulation of odontogenesis|GO:0030815,negative regulation of cAMP metabolic process|GO:0000280,nuclear division|GO:0060284,regulation of cell development|GO:1904407,positive regulation of nitric oxide metabolic process|GO:0015672,monovalent inorganic cation transport|GO:0008284,positive regulation of cell proliferation|GO:0055001,muscle cell development|GO:0055002,striated muscle cell development|GO:0035239,tube morphogenesis|GO:0050878,regulation of body fluid levels|GO:0008283,cell proliferation|GO:0006875,cellular metal ion homeostasis|GO:0006874,cellular calcium ion homeostasis|GO:0046058,cAMP metabolic process|GO:0051153,regulation of striated muscle cell differentiation|GO:0046883,regulation of hormone secretion|GO:0045214,sarcomere organization|GO:0051155,positive regulation of striated muscle cell differentiation|GO:0046888,negative regulation of hormone secretion|GO:0044255,cellular lipid metabolic process|GO:2001057,reactive nitrogen species metabolic process|GO:0006979,response to oxidative stress|GO:0009190,cyclic nucleotide biosynthetic process|GO:0050900,leukocyte migration|GO:0008217,regulation of blood pressure|GO:0072593,reactive oxygen species metabolic process|GO:0031032,actomyosin structure organization|GO:0060341,regulation of cellular localization|GO:0060024,rhythmic synaptic transmission|GO:1990266,neutrophil migration|GO:0030036,actin cytoskeleton organization|GO:0042592,homeostatic process|GO:0061041,regulation of wound healing|GO:0035690,cellular response to drug|GO:0022402,cell cycle process|GO:0061045,negative regulation of wound healing|GO:0008219,cell death|GO:0046209,nitric oxide metabolic process|GO:0051302,regulation of cell division|GO:0051301,cell division|GO:0051930,regulation of sensory perception of pain|GO:0051931,regulation of sensory perception|GO:2000112,regulation of cellular macromolecule biosynthetic process|GO:2000113,negative regulation of cellular macromolecule biosynthetic process|GO:0007275,multicellular organism development|GO:0043067,regulation of programmed cell death|GO:0043066,negative regulation of apoptotic process|GO:0043500,muscle adaptation|GO:0015849,organic acid transport|GO:0043069,negative regulation of programmed cell death|GO:0097285,obsolete cell-type specific apoptotic process|GO:0048598,embryonic morphogenesis|GO:0006468,protein phosphorylation|GO:0000278,mitotic cell cycle|GO:0019219,regulation of nucleobase-containing compound metabolic process|GO:0045087,innate immune response|GO:0006464,cellular protein modification process|GO:0007600,sensory perception|GO:0044767,single-organism developmental process|GO:0044765,single-organism transport|GO:0044763,single-organism cellular process|GO:0090257,regulation of muscle system process|GO:0060485,mesenchyme development|GO:1901362,organic cyclic compound biosynthetic process|GO:0040011,locomotion|GO:0051272,positive regulation of cellular component movement|GO:0051270,regulation of cellular component movement|GO:0010595,positive regulation of endothelial cell migration|GO:0010594,regulation of endothelial cell migration|GO:0040017,positive regulation of locomotion|GO:0006164,purine nucleotide biosynthetic process|GO:0008361,regulation of cell size|GO:0019693,ribose phosphate metabolic process|GO:0009914,hormone transport|GO:0006796,phosphate-containing compound metabolic process|GO:2000021,regulation of ion homeostasis|GO:2000026,regulation		
iption, DNA-templated|GO:0045893,positive regulation of transcription, DNA-templated|GO:0030003,cellular cation homeostasis|GO:0055080,cation homeostasis|GO:0055082,cellular chemical homeostasis|GO:0055085,transmembrane transport|GO:0055086,nucleobase-containing small molecule metabolic process|GO:003226		http://www.uniprot.org/uniprot/P05305	FALSE

LEPROT	GO:0033157,regulation of intracellular protein transport|GO:0008104,protein localization|GO:0032388,positive regulation of intracellular transport|GO:0051049,regulation of transport|GO:0048585,negative regulation of response to stimulus|GO:0048583,regulation of response to stimulus|GO:0007165,signal transduction|GO:0007166,cell surface receptor signaling pathway|GO:0007167,enzyme linked receptor protein signaling pathway|GO:0007169,transmembrane receptor protein tyrosine kinase signaling pathway|GO:0071840,cellular component organization or biogenesis|GO:0051716,cellular response to stimulus|GO:0009968,negative regulation of signal transduction|GO:0070727,cellular macromolecule localization|GO:0009966,regulation of signal transduction|GO:0048518,positive regulation of biological process|GO:0048519,negative regulation of biological process|GO:0016482,cytosolic transport|GO:0032386,regulation of intracellular transport|GO:0051050,positive regulation of transport|GO:0006605,protein targeting|GO:0045184,establishment of protein localization|GO:1903955,positive regulation of protein targeting to mitochondrion|GO:0072655,establishment of protein localization to mitochondrion|GO:0006839,mitochondrial transport|GO:0043434,response to peptide hormone|GO:0010033,response to organic substance|GO:0044700,single organism signaling|GO:0060398,regulation of growth hormone receptor signaling pathway|GO:0060396,growth hormone receptor signaling pathway|GO:0010638,positive regulation of organelle organization|GO:2000008,regulation of protein localization to cell surface|GO:2000009,negative regulation of protein localization to cell surface|GO:0070887,cellular response to chemical stimulus|GO:1903651,positive regulation of cytoplasmic transport|GO:0046426,negative regulation of JAK-STAT cascade|GO:0035556,intracellular signal transduction|GO:0051223,regulation of protein transport|GO:0050789,regulation of biological process|GO:0006886,intracellular protein transport|GO:0016043,cellular component organization|GO:0065007,biological regulation|GO:0044699,single-organism process|GO:1903828,negative regulation of cellular protein localization|GO:0051130,positive regulation of cellular component organization|GO:0070201,regulation of establishment of protein localization|GO:1903649,regulation of cytoplasmic transport|GO:0006626,protein targeting to mitochondrion|GO:0006810,transport|GO:0050794,regulation of cellular process|GO:1902532,negative regulation of intracellular signal transduction|GO:1902531,regulation of intracellular signal transduction|GO:0046907,intracellular transport|GO:1901698,response to nitrogen compound|GO:1901699,cellular response to nitrogen compound|GO:0060416,response to growth hormone|GO:0033043,regulation of organelle organization|GO:0051128,regulation of cellular component organization|GO:1903827,regulation of cellular protein localization|GO:0023057,negative regulation of signaling|GO:0023052,signaling|GO:0010648,negative regulation of cell communication|GO:1903533,regulation of protein targeting|GO:0023051,regulation of signaling|GO:0070585,protein localization to mitochondrion|GO:0007005,mitochondrion organization|GO:0010646,regulation of cell communication|GO:1903829,positive regulation of cellular protein localization|GO:0032880,regulation of protein localization|GO:0009719,response to endogenous stimulus|GO:0051234,establishment of localization|GO:0071375,cellular response to peptide hormone stimulus|GO:0071378,cellular response to growth hormone stimulus|GO:0034394,protein localization to cell surface|GO:0007259,JAK-STAT cascade|GO:0071495,cellular response to endogenous stimulus|GO:1901700,response to oxygen-containing compound|GO:0072594,establishment of protein localization to organelle|GO:0009987,cellular process|GO:1903747,regulation of establishment of protein localization to mitochondrion|GO:0032870,cellular response to hormone stimulus|GO:0032879,regulation of localization|GO:1903749,positive regulation of establishment of protein localization to mitochondrion|GO:0042221,response to chemical|GO:0033036,macromolecule localization|GO:0009725,response to hormone|GO:0033365,protein localization to organelle|GO:0050896,response to stimulus|GO:0060341,regulation of cellular localization|GO:0010821,regulation of mitochondrion organization|GO:0010822,positive regulation of mitochondrion organization|GO:0051222,positive regulation of protein transport|GO:0071417,cellular response to organonitrogen compound|GO:0071310,cellular response to organic substance|GO:0071702,organic substance transport|GO:1901652,response to peptide|GO:0060400,negative regulation of growth hormone receptor signaling pathway|GO:0046425,regulation of JAK-STAT cascade|GO:0034613,cellular protein localization|GO:0090316,positive regulation of intracellular protein transport|GO:0044765,single-organism transport|GO:0044763,single-organism cellular process|GO:0010243,response to organonitrogen compound|GO:0051649,establishment of localization in cell|GO:0007154,cell communication|GO:0051179,localization|GO:1902578,single-organism localization|GO:0051641,cellular localization|GO:0006996,organelle organization|GO:1901701,cellular response to oxygen-containing compound|GO:1903214,regulation of protein targeting to mitochondrion|GO:1901653,cellular response to peptide|GO:0015031,protein transport|GO:1902582,single-organism intracellular transport|GO:1902580,single-organism cellular localization|GO:0048523,negative regulation of cellular process|GO:0048522,positive regulation of cellular process|GO:0016197,endosomal transport|GO:0016192,vesicle-mediated transport|GO:0045324,late endosome to vacuole transport|GO:0071985,multivesicular body sorting pathway|GO:0015833,peptide transport|GO:1904951,positive regulation of establishment of protein localization|GO:0042886,amide transport|GO:0032509,endosome transport via multivesicular body sorting pathway|GO:0007034,vacuolar transport|GO:1904893,negative regulation of STAT cascade|GO:1904892,regulation of STAT cascade|GO:0032511,late endosome to vacuole transport via multivesicular	GO:0043229,intracellular organelle|GO:0005622,intracellular|GO:0043227,membrane-bounded organelle|GO:0043226,organelle|GO:0031224,intrinsic component of membrane|GO:0010008,endosome membrane|GO:0005737,cytoplasm|GO:0031090,organelle membrane|GO:0016021,integral component of membrane|GO:0016020,membrane|GO:0044431,Golgi apparatus part|GO:0005794,Golgi apparatus|GO:0098588,bounding membrane of organelle|GO:0012505,endomembrane system|GO:0043231,intracellular membrane-bounded organelle|GO:0044464,cell part|GO:0005623,cell|GO:0000139,Golgi membrane|GO:0044446,intracellular organelle part|GO:0044444,cytoplasmic part|GO:0044440,endosomal part|GO:0044424,intracellular part|GO:0044425,membrane part|GO:0005768,endosome|GO:0098805,whole membrane|GO:0044422,organelle part|GO:0031984,organelle subcompartment|GO:0031410,cytoplasmic vesicle|GO:0044433,cytoplasmic vesicle part|GO:0097708,intracellular vesicle|GO:0031982,vesicle|GO:0098791,Golgi subcompartment	GO:0005102,receptor binding|GO:0005488,binding|GO:0005515,protein binding
TMEM154		GO:0044425,membrane part|GO:0016021,integral component of membrane|GO:0016020,membrane|GO:0031224,intrinsic component of membrane	
C14orf164	GO:1903046,meiotic cell cycle process|GO:0006807,nitrogen compound metabolic process|GO:0022402,cell cycle process|GO:0018193,peptidyl-amino acid modification|GO:0044699,single-organism process|GO:0044267,cellular protein metabolic process|GO:0045143,homologous chromosome segregation|GO:0051321,meiotic cell cycle|GO:0000003,reproduction|GO:0044260,cellular macromolecule metabolic process|GO:0071704,organic substance metabolic process|GO:0071840,cellular component organization or biogenesis|GO:0045132,meiotic chromosome segregation|GO:0070647,protein modification by small protein conjugation or removal|GO:0007129,synapsis|GO:0016043,cellular component organization|GO:0032446,protein modification by small protein conjugation|GO:0007049,cell cycle|GO:0009987,cellular process|GO:0006464,cellular protein modification process|GO:0022414,reproductive process|GO:0043412,macromolecule modification|GO:0036211,protein modification process|GO:0044763,single-organism cellular process|GO:0008152,metabolic process|GO:0007059,chromosome segregation|GO:0016925,protein sumoylation|GO:0070192,chromosome organization involved in meiotic cell cycle|GO:0006996,organelle organization|GO:0044238,primary metabolic process|GO:0044702,single organism reproductive process|GO:1901564,organonitrogen compound metabolic process|GO:0051276,chromosome organization|GO:0018205,peptidyl-lysine modification|GO:0098813,nuclear chromosome segregation|GO:0044237,cellular metabolic process|GO:0043170,macromolecule metabolic process|GO:0019538,protein metabolic process|GO:1902589,single-organism organelle organization		GO:0043169,cation binding|GO:0046914,transition metal ion binding|GO:0043167,ion binding|GO:0005488,binding|GO:0046872,metal ion binding|GO:0008270,zinc ion binding|GO:0019787,ubiquitin-like protein transferase activity|GO:0019789,SUMO transferase activity|GO:0003824,catalytic activity|GO:0016740,transferase activity
TEX12	GO:0000711,meiotic DNA repair synthesis|GO:0008152,metabolic process|GO:0006950,response to stress|GO:0007130,synaptonemal complex assembly|GO:1903046,meiotic cell cycle process|GO:0090304,nucleic acid metabolic process|GO:0044249,cellular biosynthetic process|GO:0034641,cellular nitrogen compound metabolic process|GO:0022607,cellular component assembly|GO:0022402,cell cycle process|GO:0034645,cellular macromolecule biosynthetic process|GO:1901576,organic substance biosynthetic process|GO:1901362,organic cyclic compound biosynthetic process|GO:0044699,single-organism process|GO:0006139,nucleobase-containing compound metabolic process|GO:0044710,single-organism metabolic process|GO:0044711,single-organism biosynthetic process|GO:0051321,meiotic cell cycle|GO:0000003,reproduction|GO:0044260,cellular macromolecule metabolic process|GO:0071840,cellular component organization or biogenesis|GO:0045132,meiotic chromosome segregation|GO:0000280,nuclear division|GO:0007129,synapsis|GO:0016043,cellular component organization|GO:0071704,organic substance metabolic process|GO:0007049,cell cycle|GO:0046483,heterocycle metabolic process|GO:0018130,heterocycle biosynthetic process|GO:0006281,DNA repair|GO:0044271,cellular nitrogen compound biosynthetic process|GO:0009987,cellular process|GO:0006725,cellular aromatic compound metabolic process|GO:0007126,meiotic nuclear division|GO:0006974,cellular response to DNA damage stimulus|GO:0006807,nitrogen compound metabolic process|GO:0009058,biosynthetic process|GO:0007127,meiosis I|GO:0044763,single-organism cellular process|GO:0051716,cellular response to stimulus|GO:0034654,nucleobase-containing compound biosynthetic process|GO:0007059,chromosome segregation|GO:0045143,homologous chromosome segregation|GO:0009059,macromolecule biosynthetic process|GO:0051276,chromosome organization|GO:0006996,organelle organization|GO:0044238,primary metabolic process|GO:0070193,synaptonemal complex organization|GO:0070192,chromosome organization involved in meiotic cell cycle|GO:0050896,response to stimulus|GO:0098813,nuclear chromosome segregation|GO:0000731,DNA synthesis involved in DNA repair|GO:0044237,cellular metabolic process|GO:0043170,macromolecule metabolic process|GO:1902589,single-organism organelle organization|GO:0044085,cellular component biogenesis|GO:0048285,organelle fission|GO:0033554,cellular response to stress|GO:0006259,DNA metabolic process|GO:0019438,aromatic compound biosynthetic process|GO:1901360,organic cyclic compound metabolic process|GO:0071897,DNA biosynthetic process|GO:0022414,reproductive process|GO:0044702,single organism reproductive process	GO:0031974,membrane-enclosed lumen|GO:0043229,intracellular organelle|GO:0043228,non-membrane-bounded organelle|GO:0000228,nuclear chromosome|GO:0043227,membrane-bounded organelle|GO:0043226,organelle|GO:0031981,nuclear lumen|GO:0005634,nucleus|GO:0000801,central element|GO:0044454,nuclear chromosome part|GO:0005694,chromosome|GO:0000793,condensed chromosome|GO:0000794,condensed nuclear chromosome|GO:0000795,synaptonemal complex|GO:0043231,intracellular membrane-bounded organelle|GO:0043232,intracellular non-membrane-bounded organelle|GO:0043233,organelle lumen|GO:0044464,cell part|GO:0005623,cell|GO:0005622,intracellular|GO:0044446,intracellular organelle part|GO:0070013,intracellular organelle lumen|GO:0044428,nuclear part|GO:0044424,intracellular part|GO:0044427,chromosomal part|GO:0044422,organelle part	
CAV1	GO:0007599,hemostasis|GO:0007595,lactation|GO:0051049,regulation of transport|GO:0006907,pinocytosis|GO:0007596,blood coagulation|GO:0043933,macromolecular complex subunit organization|GO:0044281,small molecule metabolic process|GO:0060402,calcium ion transport into cytosol|GO:0032388,positive regulation of intracellular transport|GO:0051651,maintenance of location in cell|GO:0044854,plasma membrane raft assembly|GO:0044857,plasma membrane raft organization|GO:0051716,cellular response to stimulus|GO:0019222,regulation of metabolic process|GO:0043207,response to external biotic stimulus|GO:0001666,response to hypoxia|GO:1904294,positive regulation of ERAD pathway|GO:0051495,positive regulation of cytoskeleton organization|GO:0000165,MAPK cascade|GO:1904292,regulation of ERAD pathway|GO:0030856,regulation of epithelial cell differentiation|GO:0030857,negative regulation of epithelial cell differentiation|GO:0030855,epithelial cell differentiation|GO:0071455,cellular response to hyperoxia|GO:0048585,negative regulation of response to stimulus|GO:0060544,regulation of necroptotic process|GO:0060547,negative regulation of necrotic cell death|GO:0060546,negative regulation of necroptotic process|GO:0048583,regulation of response to stimulus|GO:0045859,regulation of protein kinase activity|GO:0031109,microtubule polymerization or depolymerization|GO:0019229,regulation of vasoconstriction|GO:0071363,cellular response to growth factor stimulus|GO:0046486,glycerolipid metabolic process|GO:0046483,heterocycle metabolic process|GO:0042325,regulation of phosphorylation|GO:0042327,positive regulation of phosphorylation|GO:0042326,negative regulation of phosphorylation|GO:0009607,response to biotic stimulus|GO:0097178,ruffle assembly|GO:0019538,protein metabolic process|GO:0010638,positive regulation of organelle organization|GO:0060491,regulation of cell projection assembly|GO:2000286,receptor internalization involved in canonical Wnt signaling pathway|GO:0072358,cardiovascular system development|GO:0043331,response to dsRNA|GO:0043330,response to exogenous dsRNA|GO:0009896,positive regulation of catabolic process|GO:0016265,obsolete death|GO:0009894,regulation of catabolic process|GO:0032410,negative regulation of transporter activity|GO:0009893,positive regulation of metabolic process|GO:0009890,negative regulation of biosynthetic process|GO:0032413,negative regulation of ion transmembrane transporter activity|GO:0090263,positive regulation of canonical Wnt signaling pathway|GO:0060306,regulation of membrane repolarization|GO:0031952,regulation of protein autophosphorylation|GO:0031953,negative regulation of protein autophosphorylation|GO:0001508,action potential|GO:0030177,positive regulation of Wnt signaling pathway|GO:0043393,regulation of protein binding|GO:0035556,intracellular signal transduction|GO:0071900,regulation of protein serine/threonine kinase activity|GO:0030178,negative regulation of Wnt signaling pathway|GO:0032091,negative regulation of protein binding|GO:0051341,regulation of oxidoreductase activity|GO:0051345,positive regulation of hydrolase activity|GO:0051348,negative regulation of transferase activity|GO:0051828,entry into other organism involved in symbiotic interaction|GO:1903409,reactive oxygen species biosynthetic process|GO:0002684,positive regulation of immune system process|GO:0002682,regulation of immune system process|GO:1901360,organic cyclic compound metabolic process|GO:1903598,positive regulation of gap junction assembly|GO:0010332,response to gamma radiation|GO:0018130,heterocycle biosynthetic process|GO:2000378,negative regulation of reactive oxygen species metabolic process|GO:0032769,negative regulation of monooxygenase activity|GO:0006629,lipid metabolic process|GO:1903649,regulation of cytoplasmic transport|GO:0098609,cell-cell adhesion|GO:0016310,phosphorylation|GO:2000377,regulation of reactive oxygen species metabolic process|GO:0009888,tissue development|GO:1903524,positive regulation of blood circulation|GO:1903522,regulation of blood circulation|GO:0042060,wound healing|GO:0009267,cellular response to starvation|GO:0060761,negative regulation of response to cytokine stimulus|GO:0016070,RNA metabolic process|GO:0086098,angiotensin-activated signaling pathway involved in heart process|GO:0071345,cellular response to cytokine stimulus|GO:0010556,regulation of macromolecule biosynthetic process|GO:0044767,single-organism developmental process|GO:2001258,negative regulation of cation channel activity|GO:0048869,cellular developmental process|GO:2001257,regulation of cation channel activity|GO:0010558,negative regulation of macromolecule biosynthetic process|GO:0051493,regulation of cytoskeleton organization|GO:0072584,caveolin-mediated endocytosis|GO:0051129,negative regulation of cellular component organization|GO:0051128,regulation of cellular component organization|GO:0042176,regulation of protein catabolic process|GO:0018105,peptidyl-serine phosphorylation|GO:0006511,ubiquitin-dependent protein catabolic process|GO:0051235,maintenance of location|GO:0014070,response to organic cyclic compound|GO:0003057,regulation of the force of heart contraction by chemical signal|GO:0038083,peptidyl-tyrosine autophosphorylation|GO:0000122,negative regulation of transcription from RNA polymerase II promoter|GO:1900027,regulation of ruffle assembly|GO:0034330,cell junction organization|GO:0060284,regulation of cell development|GO:0019058,viral life cycle|GO:1904406,negative regulation of nitric oxide metabolic process|GO:0002224,toll-like receptor signaling pathway|GO:0030260,entry into host cell|GO:0002221,pattern recognition receptor signaling pathway|GO:0008284,positive regulation of cell proliferation|GO:0008285,negative regulation of cell proliferation|GO:0050878,regulation of body fluid levels|GO:0008283,cell proliferation|GO:0006875,cellular metal ion homeostasis|GO:0006874,cellular calcium ion homeostasis|GO:0050870,positive regulation of T cell activation|GO:0043412,macromolecule modification|GO:0040011,locomotion|GO:0045216,cell-cell junction organization|GO:0044257,cellular protein catabolic process|GO:0044255,cellular lipid metabolic process|GO:0034329,cell junction assembly|GO:0072511,divalent inorganic cation transport|GO:0050900,leukocyte migration|GO:1901699,cellular response to nitrogen compound|GO:0060341,regulation of cellular localization|GO:0030030,cell projection organization|GO:0030031,cell projection assembly|GO:0097190,apoptotic signaling pathway|GO:0097191,extrinsic apoptotic signaling pathway|GO:0018108,peptidyl-tyrosine phosphorylation|GO:0097193,intrinsic apoptotic signaling pathway|GO:0042592,homeostatic process|GO:0022407,regulation of cell-cell adhesion|GO:0061041,regulation of wound healing|GO:0042594,response to starvation|GO:0086064,cell communication by electrical coupling involved in cardiac conduction|GO:0086065,cell communication involved in cardiac conduction|GO:0055093,response to hyperoxia|GO:0008219,cell death|GO:0022409,positive regulation of cell-cell adhesion|GO:0046209,nitric oxide metabolic process|GO:0002218,activation of innate immune response|GO:0071559,response to transforming growth factor beta|GO:0031100,animal organ regeneration|GO:2000112,regulation of cellular macromolecule biosynthetic process|GO:2000113,negative regulation of cellular macromolecule biosynthetic process|GO:0007275,multicellular organism development|GO:0043065,positive regulation of apoptotic process|GO:0043067,regulation of programmed cell death|GO:0043066,negative regulation of apoptotic process|GO:0043269,regulation of ion transport|GO:0060548,negative regulation of cell death|GO:0043069,negative regulation of programmed cell death|GO:0043068,positive regulation of programmed cell death|GO:0090090,negative regulation of canonical Wnt signaling pathway|GO:0090092,regulation of transmembrane receptor protein serine/threonine kinase signaling pathway|GO:0006469,negative regulation of protein kinase activity|GO:0045089,positive regulation of innate immune response|GO:0045088,regulation of innate immune response|GO:0019217,regulation of fatty acid metabolic process|GO:0019216,regulation of lipid metabolic process|GO:0019219,regulation of nucleobase-containing compound metabolic process|GO:0045087,innate immune response|GO:0006461,protein complex assembly|GO:1903071,positive regulation of ER-associated ubiquitin-dependent protein catabolic process|GO:0006464,cellular protein modification process|GO:0010959,regulation of metal ion transport|GO:0044766,multi-organism transport|GO:0044765,single-organism transport|GO:0044764,multi-organism cellular process|GO:0044763,single-organism cellular process|GO:0090257,regulation of muscle system process|GO:0019941,modification-dependent protein catabolic process|GO:0060401,cytosolic		
ion|GO:0030048,actin filament-based movement|GO:0046649,lymphocyte activation|GO:0030162,regulation of proteolysis|GO:0030163,protein catabolic process|GO:0030514,negative regulation of BMP signaling pathway|GO:0010876,lipid localization|GO:0030510,regulation of BMP signaling pathway|GO:0042522,regulation o		http://www.uniprot.org/uniprot/Q03135	FALSE
SLC27A6	GO:0006631,fatty acid metabolic process|GO:0006869,lipid transport|GO:0019752,carboxylic acid metabolic process|GO:0001676,long-chain fatty acid metabolic process|GO:0006820,anion transport|GO:0044281,small molecule metabolic process|GO:0015718,monocarboxylic acid transport|GO:0044699,single-organism process|GO:0015908,fatty acid transport|GO:0044710,single-organism metabolic process|GO:0051179,localization|GO:0071704,organic substance metabolic process|GO:0071702,organic substance transport|GO:0033036,macromolecule localization|GO:0006810,transport|GO:0015909,long-chain fatty acid transport|GO:0006629,lipid metabolic process|GO:0015849,organic acid transport|GO:0006811,ion transport|GO:0009987,cellular process|GO:0015711,organic anion transport|GO:0032787,monocarboxylic acid metabolic process|GO:0044765,single-organism transport|GO:0044763,single-organism cellular process|GO:0008152,metabolic process|GO:0000038,very long-chain fatty acid metabolic process|GO:0043436,oxoacid metabolic process|GO:0044255,cellular lipid metabolic process|GO:0051234,establishment of localization|GO:0010876,lipid localization|GO:0055085,transmembrane transport|GO:1902578,single-organism localization|GO:0046942,carboxylic acid transport|GO:0044238,primary metabolic process|GO:0006082,organic acid metabolic process|GO:0044237,cellular metabolic process	GO:0042383,sarcolemma|GO:0016021,integral component of membrane|GO:0016020,membrane|GO:0044464,cell part|GO:0005623,cell|GO:0071944,cell periphery|GO:0005886,plasma membrane|GO:0044425,membrane part|GO:0031224,intrinsic component of membrane	GO:0015645,fatty acid ligase activity|GO:0097159,organic cyclic compound binding|GO:0031957,very long-chain fatty acid-CoA ligase activity|GO:0000166,nucleotide binding|GO:0036094,small molecule binding|GO:0003824,catalytic activity|GO:0004467,long-chain fatty acid-CoA ligase activity|GO:0005488,binding|GO:0016874,ligase activity|GO:1901363,heterocyclic compound binding|GO:1901265,nucleoside phosphate binding|GO:0016877,ligase activity, forming carbon-sulfur bonds|GO:0005319,lipid transporter activity|GO:0022892,substrate-specific transporter activity|GO:0005215,transporter activity|GO:0015245,fatty acid transporter activity
SLC22A24	GO:0006810,transport|GO:0006811,ion transport|GO:0009987,cellular process|GO:0015711,organic anion transport|GO:0051179,localization|GO:0044765,single-organism transport|GO:0034220,ion transmembrane transport|GO:0006820,anion transport|GO:0044763,single-organism cellular process|GO:0044699,single-organism process|GO:0071702,organic substance transport|GO:0098656,anion transmembrane transport|GO:0051234,establishment of localization|GO:0043252,sodium-independent organic anion transport|GO:0055085,transmembrane transport|GO:1902578,single-organism localization|GO:0015849,organic acid transport|GO:1903825,organic acid transmembrane transport|GO:0071705,nitrogen compound transport|GO:0015747,urate transport|GO:0015698,inorganic anion transport	GO:0031226,intrinsic component of plasma membrane|GO:0016021,integral component of membrane|GO:0016020,membrane|GO:0044464,cell part|GO:0005623,cell|GO:0071944,cell periphery|GO:0005887,integral component of plasma membrane|GO:0005886,plasma membrane|GO:0044425,membrane part|GO:0044459,plasma membrane part|GO:0031224,intrinsic component of membrane	GO:0022891,substrate-specific transmembrane transporter activity|GO:0015297,antiporter activity|GO:0022892,substrate-specific transporter activity|GO:0015291,secondary active transmembrane transporter activity|GO:0015301,anion:anion antiporter activity|GO:0008514,organic anion transmembrane transporter activity|GO:0005215,transporter activity|GO:0008509,anion transmembrane transporter activity|GO:0015347,sodium-independent organic anion transmembrane transporter activity|GO:0015075,ion transmembrane transporter activity|GO:0022857,transmembrane transporter activity|GO:0005452,inorganic anion exchanger activity|GO:0022804,active transmembrane transporter activity|GO:1901702,salt transmembrane transporter activity|GO:0005342,organic acid transmembrane transporter activity|GO:0015103,inorganic anion transmembrane transporter activity|GO:0015143,urate transmembrane transporter activity
KRTAP19			
XAGE3			GO:0005488,binding|GO:0005515,protein binding
FAM122C			
CCDC82			
FGG	GO:2001234,negative regulation of apoptotic signaling pathway|GO:0034116,positive regulation of heterotypic cell-cell adhesion|GO:2001236,regulation of extrinsic apoptotic signaling pathway|GO:2001237,negative regulation of extrinsic apoptotic signaling pathway|GO:0019220,regulation of phosphate metabolic process|GO:0080090,regulation of primary metabolic process|GO:0019222,regulation of metabolic process|GO:2001233,regulation of apoptotic signaling pathway|GO:0051049,regulation of transport|GO:0048585,negative regulation of response to stimulus|GO:0048584,positive regulation of response to stimulus|GO:0048583,regulation of response to stimulus|GO:0019229,regulation of vasoconstriction|GO:0007160,cell-matrix adhesion|GO:0034109,homotypic cell-cell adhesion|GO:0045907,positive regulation of vasoconstriction|GO:0007165,signal transduction|GO:0007166,cell surface receptor signaling pathway|GO:0003018,vascular process in circulatory system|GO:0034113,heterotypic cell-cell adhesion|GO:0023014,signal transduction by protein phosphorylation|GO:0044710,single-organism metabolic process|GO:0010604,positive regulation of macromolecule metabolic process|GO:0009968,negative regulation of signal transduction|GO:0045785,positive regulation of cell adhesion|GO:0009966,regulation of signal transduction|GO:0009967,positive regulation of signal transduction|GO:0051047,positive regulation of secretion|GO:0000165,MAPK cascade|GO:0010467,gene expression|GO:0046879,hormone secretion|GO:0010720,positive regulation of cell development|GO:0048518,positive regulation of biological process|GO:0048519,negative regulation of biological process|GO:0033036,macromolecule localization|GO:0007599,hemostasis|GO:0031589,cell-substrate adhesion|GO:0007596,blood coagulation|GO:0051050,positive regulation of transport|GO:0060255,regulation of macromolecule metabolic process|GO:0060548,negative regulation of cell death|GO:0071702,organic substance transport|GO:0009914,hormone transport|GO:0045184,establishment of protein localization|GO:0090276,regulation of peptide hormone secretion|GO:0090277,positive regulation of peptide hormone secretion|GO:0007155,cell adhesion|GO:0010038,response to metal ion|GO:0010035,response to inorganic substance|GO:0030168,platelet activation|GO:0003008,system process|GO:0042325,regulation of phosphorylation|GO:0044700,single organism signaling|GO:0042327,positive regulation of phosphorylation|GO:0065008,regulation of biological quality|GO:0051716,cellular response to stimulus|GO:0016192,vesicle-mediated transport|GO:0044707,single-multicellular organism process|GO:0019538,protein metabolic process|GO:0008015,blood circulation|GO:0030198,extracellular matrix organization|GO:0050880,regulation of blood vessel size|GO:0030193,regulation of blood coagulation|GO:0098609,cell-cell adhesion|GO:0003013,circulatory system process|GO:0030195,negative regulation of blood coagulation|GO:1903530,regulation of secretion by cell|GO:0022604,regulation of cell morphogenesis|GO:0016265,obsolete death|GO:0050896,response to stimulus|GO:0022607,cellular component assembly|GO:0009893,positive regulation of metabolic process|GO:0022603,regulation of anatomical structure morphogenesis|GO:0006950,response to stress|GO:1903532,positive regulation of secretion by cell|GO:0008104,protein localization|GO:0032940,secretion by cell|GO:0065007,biological regulation|GO:0042310,vasoconstriction|GO:0035556,intracellular signal transduction|GO:0051223,regulation of protein transport|GO:0042981,regulation of apoptotic process|GO:0050789,regulation of biological process|GO:0030072,peptide hormone secretion|GO:0009605,response to external stimulus|GO:0044267,cellular protein metabolic process|GO:0000904,cell morphogenesis involved in differentiation|GO:0050708,regulation of protein secretion|GO:0000902,cell morphogenesis|GO:0044260,cellular macromolecule metabolic process|GO:0006887,exocytosis|GO:0035150,regulation of tube size|GO:0016043,cellular component organization|GO:0090066,regulation of anatomical structure size|GO:0065003,macromolecular complex assembly|GO:0009306,protein secretion|GO:1900046,regulation of hemostasis|GO:1900047,negative regulation of hemostasis|GO:1904035,regulation of epithelial cell apoptotic process|GO:0071840,cellular component organization or biogenesis|GO:1904036,negative regulation of epithelial cell apoptotic process|GO:0032880,regulation of protein localization|GO:0051130,positive regulation of cellular component organization|GO:0070201,regulation of establishment of protein localization|GO:0098602,single organism cell adhesion|GO:0050793,regulation of developmental process|GO:0006810,transport|GO:0042886,amide transport|GO:0048468,cell development|GO:0050794,regulation of cellular process|GO:0043410,positive regulation of MAPK cascade|GO:0012501,programmed cell death|GO:1903524,positive regulation of blood circulation|GO:0050817,coagulation|GO:1903522,regulation of blood circulation|GO:0006464,cellular protein modification process|GO:0051239,regulation of multicellular organismal process|GO:1902533,positive regulation of intracellular signal transduction|GO:1902531,regulation of intracellular signal transduction|GO:0050818,regulation of coagulation|GO:0050819,negative regulation of coagulation|GO:0048856,anatomical structure development|GO:0042060,wound healing|GO:0044767,single-organism developmental process|GO:0042730,fibrinolysis|GO:0072577,endothelial cell apoptotic process|GO:0051604,protein maturation|GO:0050714,positive regulation of protein secretion|GO:0043412,macromolecule modification|GO:0080134,regulation of response to stress|GO:0031401,positive regulation of protein modification process|GO:0001775,cell activation|GO:0034114,regulation of heterotypic cell-cell adhesion|GO:0036211,protein modification process|GO:0048869,cellular developmental process|GO:0051046,regulation of secretion|GO:0009611,response to wounding|GO:0051240,positive regulation of multicellular organismal process|GO:1902042,negative regulation of extrinsic apoptotic signaling pathway via death domain receptors|GO:1902041,regulation of extrinsic apoptotic signaling pathway via death domain receptors|GO:0032102,negative regulation of response to external stimulus|GO:0008152,metabolic process|GO:0032101,regulation of response to external stimulus|GO:0016310,phosphorylation|GO:0030155,regulation of cell adhesion|GO:0030154,cell differentiation|GO:0051128,regulation of cellular component organization|GO:0023056,positive regulation of signaling|GO:0023057,negative regulation of signaling|GO:0015833,peptide transport|GO:0023052,signaling|GO:0010648,negative regulation of cell communication|GO:0023051,regulation of signaling|GO:0051592,response to calcium ion|GO:0010647,positive regulation of cell communication|GO:0010646,regulation of cell communication|GO:0044699,single-organism process|GO:0043408,regulation of MAPK cascade|GO:1904019,epithelial cell apoptotic process|GO:0008625,extrinsic apoptotic signaling pathway via death domain receptors|GO:0044057,regulation of system process|GO:1900026,positive regulation of substrate adhesion-dependent cell spreading|GO:0010562,positive regulation of phosphorus metabolic process|GO:0051241,negative regulation of multicellular organismal process|GO:0051246,regulation of protein metabolic process|GO:0051247,positive regulation of protein metabolic process|GO:0060284,regulation of cell development|GO:0010769,regulation of cell morphogenesis involved in differentiation|GO:0032270,positive regulation of cellular protein metabolic process|GO:0031399,regulation of protein modification process|GO:0006508,proteolysis|GO:0022610,biological adhesion|GO:1903034,regulation of response to wounding|GO:1903035,negative regulation of response to wounding|GO:0017157,regulation of exocytosis|GO:0032502,developmental process|GO:0032501,multicellular organismal process|GO:0050878,regulation of body fluid levels|GO:0031323,regulation of cellular metabolic process|GO:0009987,cellular process|GO:2000351,regulation of endothelial cell apoptotic process|GO:2000352,negative regulation of endothelial cell apoptotic process|GO:0060627,regulation of vesicle-mediated transport|GO:0045597,positive regulation of cell differentiation|GO:0045595,regulation of cell differentiation|GO:0046883,regulation of hormone secretion|GO:0046887,positive regulation of hormone secretion|GO:0016485,protein processing|GO:0046903,secretion|GO:0032879,regulation of localization|GO:0051258,protein polymerization|GO:0032268,regulation of cellular protein metabolic process|GO:0006461,protein complex	GO:0009897,external side of plasma membrane|GO:0098552,side of membrane|GO:0031974,membrane-enclosed lumen|GO:0043229,intracellular organelle|GO:0071944,cell periphery|GO:0060205,cytoplasmic membrane-bounded vesicle lumen|GO:0043227,membrane-bounded organelle|GO:0043226,organelle|GO:0005737,cytoplasm|GO:0031983,vesicle lumen|GO:0070062,extracellular exosome|GO:0031093,platelet alpha granule lumen|GO:0016023,cytoplasmic, membrane-bounded vesicle|GO:0031410,cytoplasmic vesicle|GO:0016020,membrane|GO:0044444,cytoplasmic part|GO:0072562,blood microparticle|GO:0031988,membrane-bounded vesicle|GO:0044433,cytoplasmic vesicle part|GO:0005615,extracellular space|GO:0005886,plasma membrane|GO:0044459,plasma membrane part|GO:0032991,macromolecular complex|GO:0030141,secretory granule|GO:0009986,cell surface|GO:0005938,cell cortex|GO:0034774,secretory granule lumen|GO:0031091,platelet alpha granule|GO:1903561,extracellular vesicle|GO:0031982,vesicle|GO:0043234,protein complex|GO:0043230,extracellular organelle|GO:0043231,intracellular membrane-bounded organelle|GO:0043233,organelle lumen|GO:0044464,cell part|GO:0005623,cell|GO:0005622,intracellular|GO:0044446,intracellular organelle part|GO:0005577,fibrinogen complex|GO:0005576,extracellular region|GO:0044424,intracellular part|GO:0044425,membrane part|GO:0065010,extracellular membrane-bounded organelle|GO:0044421,extracellular region part|GO:0044422,organelle part|GO:0012505,endomembrane system|GO:0005783,endoplasmic reticulum|GO:0005788,endoplasmic reticulum lumen|GO:0044432,endoplasmic reticulum part|GO:0099503,secretory vesicle|GO:0070013,intracellular organelle lumen|GO:0097708,intracellular vesicle	GO:0043169,cation binding|GO:0030674,protein binding, bridging|GO:0050839,cell adhesion molecule binding|GO:0005198,structural molecule activity|GO:0043167,ion binding|GO:0005488,binding|GO:0005515,protein binding|GO:0005102,receptor binding|GO:0046872,metal ion binding|GO:0060090,binding, bridging|GO:0042803,protein homodimerization activity|GO:0046983,protein dimerization activity|GO:0042802,identical protein binding
HMGN5		GO:0031974,membrane-enclosed lumen|GO:0043229,intracellular organelle|GO:0043228,non-membrane-bounded organelle|GO:0000785,chromatin|GO:0043227,membrane-bounded organelle|GO:0043226,organelle|GO:0031981,nuclear lumen|GO:0005730,nucleolus|GO:0005634,nucleus|GO:0005694,chromosome|GO:0032991,macromolecular complex|GO:0043231,intracellular membrane-bounded organelle|GO:0043232,intracellular non-membrane-bounded organelle|GO:0043233,organelle lumen|GO:0044464,cell part|GO:0005623,cell|GO:0005622,intracellular|GO:0044446,intracellular organelle part|GO:0070013,intracellular organelle lumen|GO:0044428,nuclear part|GO:0044424,intracellular part|GO:0044427,chromosomal	GO:0043566,structure-specific DNA binding|GO:0005515,protein binding|GO:0097159,organic cyclic compound binding|GO:0031491,nucleosome binding|GO:0031490,chromatin DNA binding|GO:0031492,nucleosomal DNA binding|GO:0044877,macromolecular complex binding|GO:0005488,binding|GO:0003676,nucleic acid binding|GO:0003677,DNA binding|GO:0032403,protein complex binding|GO:0003682,chromatin binding|GO:1901363,heterocyclic compound binding
KRTAP4			

PTPN22	GO:0051046,regulation of secretion|GO:0051047,positive regulation of secretion|GO:0051048,negative regulation of secretion|GO:0051049,regulation of transport|GO:0051716,cellular response to stimulus|GO:0043207,response to external biotic stimulus|GO:0000165,MAPK cascade|GO:0045859,regulation of protein kinase activity|GO:0042325,regulation of phosphorylation|GO:0042327,positive regulation of phosphorylation|GO:0042326,negative regulation of phosphorylation|GO:0009607,response to biotic stimulus|GO:0009605,response to external stimulus|GO:0019538,protein metabolic process|GO:1900165,negative regulation of interleukin-6 secretion|GO:0009894,regulation of catabolic process|GO:0009895,negative regulation of catabolic process|GO:0009892,negative regulation of metabolic process|GO:0009893,positive regulation of metabolic process|GO:0006950,response to stress|GO:0046651,lymphocyte proliferation|GO:0051222,positive regulation of protein transport|GO:0071900,regulation of protein serine/threonine kinase activity|GO:0051224,negative regulation of protein transport|GO:0050789,regulation of biological process|GO:0051348,negative regulation of transferase activity|GO:0002684,positive regulation of immune system process|GO:0002682,regulation of immune system process|GO:0002683,negative regulation of immune system process|GO:0070201,regulation of establishment of protein localization|GO:0098602,single organism cell adhesion|GO:0098609,cell-cell adhesion|GO:0043410,positive regulation of MAPK cascade|GO:0032481,positive regulation of type I interferon production|GO:0002521,leukocyte differentiation|GO:0002520,immune system development|GO:0050710,negative regulation of cytokine secretion|GO:0050715,positive regulation of cytokine secretion|GO:0050714,positive regulation of protein secretion|GO:0048869,cellular developmental process|GO:0034142,toll-like receptor 4 signaling pathway|GO:0035556,intracellular signal transduction|GO:0034139,regulation of toll-like receptor 3 signaling pathway|GO:0002224,toll-like receptor signaling pathway|GO:0002221,pattern recognition receptor signaling pathway|GO:0032677,regulation of interleukin-8 production|GO:0008283,cell proliferation|GO:0043508,negative regulation of JUN kinase activity|GO:0060341,regulation of cellular localization|GO:0022407,regulation of cell-cell adhesion|GO:1901222,regulation of NIK/NF-kappaB signaling|GO:0007275,multicellular organism development|GO:0022408,negative regulation of cell-cell adhesion|GO:0032717,negative regulation of interleukin-8 production|GO:0033993,response to lipid|GO:0070661,leukocyte proliferation|GO:0070663,regulation of leukocyte proliferation|GO:0006468,protein phosphorylation|GO:0006469,negative regulation of protein kinase activity|GO:0045089,positive regulation of innate immune response|GO:0045088,regulation of innate immune response|GO:0045087,innate immune response|GO:0006464,cellular protein modification process|GO:0044767,single-organism developmental process|GO:0044765,single-organism transport|GO:0044763,single-organism cellular process|GO:0030101,natural killer cell activation|GO:1901700,response to oxygen-containing compound|GO:1901701,cellular response to oxygen-containing compound|GO:0048856,anatomical structure development|GO:0006796,phosphate-containing compound metabolic process|GO:0006793,phosphorus metabolic process|GO:0048523,negative regulation of cellular process|GO:0048522,positive regulation of cellular process|GO:0008104,protein localization|GO:0034111,negative regulation of homotypic cell-cell adhesion|GO:0034110,regulation of homotypic cell-cell adhesion|GO:0031349,positive regulation of defense response|GO:0007162,negative regulation of cell adhesion|GO:0007165,signal transduction|GO:0007166,cell surface receptor signaling pathway|GO:0031347,regulation of defense response|GO:0044710,single-organism metabolic process|GO:0044092,negative regulation of molecular function|GO:0033036,macromolecule localization|GO:0070534,protein K63-linked ubiquitination|GO:0051051,negative regulation of transport|GO:0051050,positive regulation of transport|GO:0051707,response to other organism|GO:0010033,response to organic substance|GO:0051704,multi-organism process|GO:0044248,cellular catabolic process|GO:0002429,immune response-activating cell surface receptor signaling pathway|GO:0031663,lipopolysaccharide-mediated signaling pathway|GO:0016567,protein ubiquitination|GO:0045321,leukocyte activation|GO:0010629,negative regulation of gene expression|GO:0010628,positive regulation of gene expression|GO:0046329,negative regulation of JNK cascade|GO:0046328,regulation of JNK cascade|GO:1902523,positive regulation of protein K63-linked ubiquitination|GO:0044267,cellular protein metabolic process|GO:0002764,immune response-regulating signaling pathway|GO:0044260,cellular macromolecule metabolic process|GO:0002768,immune response-regulating cell surface receptor signaling pathway|GO:0035644,phosphoanandamide dephosphorylation|GO:0043409,negative regulation of MAPK cascade|GO:0043408,regulation of MAPK cascade|GO:0032814,regulation of natural killer cell activation|GO:0032817,regulation of natural killer cell proliferation|GO:0050790,regulation of catalytic activity|GO:0050794,regulation of cellular process|GO:0051239,regulation of multicellular organismal process|GO:0051234,establishment of localization|GO:0050896,response to stimulus|GO:0051338,regulation of transferase activity|GO:0002695,negative regulation of leukocyte activation|GO:0002694,regulation of leukocyte activation|GO:0032715,negative regulation of interleukin-6 production|GO:0010562,positive regulation of phosphorus metabolic process|GO:0010563,negative regulation of phosphorus metabolic process|GO:1902914,regulation of protein polyubiquitination|GO:0051247,positive regulation of protein metabolic process|GO:0043407,negative regulation of MAP kinase activity|GO:0043405,regulation of MAP kinase activity|GO:1903531,negative regulation of secretion by cell|GO:1903530,regulation of secretion by cell|GO:0070887,cellular response to chemical stimulus|GO:1903532,positive regulation of secretion by cell|GO:0035335,peptidyl-tyrosine dephosphorylation|GO:0000209,protein polyubiquitination|GO:0044699,single-organism process|GO:0032880,regulation of protein localization|GO:0051248,negative regulation of protein metabolic process|GO:0051249,regulation of lymphocyte activation|GO:0051240,positive regulation of multicellular organismal process|GO:0051241,negative regulation of multicellular organismal process|GO:0051246,regulation of protein metabolic process|GO:0031098,stress-activated protein kinase signaling cascade|GO:1902916,positive regulation of protein polyubiquitination|GO:1903038,negative regulation of leukocyte cell-cell adhesion|GO:0031398,positive regulation of protein ubiquitination|GO:0031399,regulation of protein modification process|GO:0031396,regulation of protein ubiquitination|GO:0001787,natural killer cell proliferation|GO:1903037,regulation of leukocyte cell-cell adhesion|GO:0070302,regulation of stress-activated protein kinase signaling cascade|GO:0070303,negative regulation of stress-activated protein kinase signaling cascade|GO:0071396,cellular response to lipid|GO:0034123,positive regulation of toll-like receptor signaling pathway|GO:0034121,regulation of toll-like receptor signaling pathway|GO:0002237,response to molecule of bacterial origin|GO:0048731,system development|GO:0070374,positive regulation of ERK1 and ERK2 cascade|GO:0070372,regulation of ERK1 and ERK2 cascade|GO:0070371,ERK1 and ERK2 cascade|GO:0016337,single organismal cell-cell adhesion|GO:0050868,negative regulation of T cell activation|GO:0050865,regulation of cell activation|GO:0050866,negative regulation of cell activation|GO:0050860,negative regulation of T cell receptor signaling pathway|GO:0050863,regulation of T cell activation|GO:0071901,negative regulation of protein serine/threonine kinase activity|GO:0051223,regulation of protein transport|GO:0045937,positive regulation of phosphate metabolic process|GO:0045936,negative regulation of phosphate metabolic process|GO:0070489,T cell aggregation|GO:0042221,response to chemical|GO:0070486,leukocyte aggregation|GO:0044238,primary metabolic process|GO:0044237,cellular metabolic process|GO:0019220,regulation of phosphate metabolic process|GO:0019222,regulation of metabolic process|GO:0006470,protein dephosphorylation|GO:0048585,negative regulation of response to stimulus|GO:0048584,positive regulation of response to stimulus|GO:0048583,regulation of response to stimulus|GO:0009968,negative regulation of signal transduction|GO:0009966,regulation of signal transduction|GO:0009967,positive regulation of signal transduction|GO:0070432,regulation of nucleotide-binding oligomerization domain containing 2 signaling pathway|GO:0070433,negative regulation of nucleotide-	GO:0098562,cytoplasmic side of membrane|GO:0005737,cytoplasm|GO:0016020,membrane|GO:0043231,intracellular membrane-bounded organelle|GO:0044459,plasma membrane part|GO:0005634,nucleus|GO:0005886,plasma membrane|GO:0044464,cell part|GO:0044444,cytoplasmic part|GO:0009898,cytoplasmic side of plasma membrane|GO:0005622,intracellular|GO:0043229,intracellular organelle|GO:0071944,cell periphery|GO:0005623,cell|GO:0098552,side of membrane|GO:0044424,intracellular part|GO:0044425,membrane part|GO:0048471,perinuclear region of cytoplasm|GO:0043227,membrane-bounded organelle|GO:0043226,organelle|GO:0005829,cytosol	GO:0005488,binding|GO:0016787,hydrolase activity|GO:0019899,enzyme binding|GO:0031625,ubiquitin protein ligase binding|GO:0016791,phosphatase activity|GO:0019900,kinase binding|GO:0004725,protein tyrosine phosphatase activity|GO:0017124,SH3 domain binding|GO:0016788,hydrolase activity, acting on ester bonds|GO:0042578,phosphoric ester hydrolase activity|GO:0003824,catalytic activity|GO:0004721,phosphoprotein phosphatase activity|GO:0005515,protein binding|GO:0044389,ubiquitin-like protein ligase binding|GO:0019904,protein domain specific binding
TXNDC8	GO:0034599,cellular response to oxidative stress|GO:0032502,developmental process|GO:0022607,cellular component assembly|GO:0006457,protein folding|GO:0048232,male gamete generation|GO:0044237,cellular metabolic process|GO:0018904,ether metabolic process|GO:0030154,cell differentiation|GO:0048468,cell development|GO:0019953,sexual reproduction|GO:0010927,cellular component assembly involved in morphogenesis|GO:0042592,homeostatic process|GO:0044281,small molecule metabolic process|GO:0070887,cellular response to chemical stimulus|GO:0009653,anatomical structure morphogenesis|GO:0000103,sulfate assimilation|GO:0050789,regulation of biological process|GO:0007276,gamete generation|GO:0044267,cellular protein metabolic process|GO:0044710,single-organism metabolic process|GO:0070925,organelle assembly|GO:0000003,reproduction|GO:0044260,cellular macromolecule metabolic process|GO:0048869,cellular developmental process|GO:0071840,cellular component organization or biogenesis|GO:0044703,multi-organism reproductive process|GO:0010256,endomembrane system organization|GO:0016043,cellular component organization|GO:0032989,cellular component morphogenesis|GO:0071704,organic substance metabolic process|GO:0065007,biological regulation|GO:0048515,spermatid differentiation|GO:0006662,glycerol ether metabolic process|GO:0065008,regulation of biological quality|GO:0048646,anatomical structure formation involved in morphogenesis|GO:0019725,cellular homeostasis|GO:0032501,multicellular organismal process|GO:0048609,multicellular organismal reproductive process|GO:0032504,multicellular organism reproduction|GO:0044238,primary metabolic process|GO:0009987,cellular process|GO:0051716,cellular response to stimulus|GO:0050794,regulation of cellular process|GO:0044767,single-organism developmental process|GO:0003006,developmental process involved in reproduction|GO:0007283,spermatogenesis|GO:0044763,single-organism cellular process|GO:0008152,metabolic process|GO:0022412,cellular process involved in reproduction in multicellular organism|GO:0042221,response to chemical|GO:0045454,cell redox homeostasis|GO:0001675,acrosome assembly|GO:0006979,response to oxidative stress|GO:0055114,oxidation-reduction process|GO:0051704,multi-organism process|GO:0006996,organelle organization|GO:0007281,germ cell development|GO:0044699,single-organism process|GO:0044702,single organism reproductive process|GO:0044707,single-multicellular organism process|GO:0022414,reproductive process|GO:0019538,protein metabolic process|GO:0050896,response to stimulus|GO:0048856,anatomical structure development|GO:0006950,response to stress|GO:0016050,vesicle organization|GO:0043170,macromolecule metabolic process|GO:1902589,single-organism organelle organization|GO:0044085,cellular component biogenesis|GO:0006790,sulfur compound metabolic process|GO:0033554,cellular response to stress|GO:0007275,multicellular organism development|GO:0007286,spermatid development|GO:0033363,secretory granule organization|GO:0098754,detoxification|GO:0009636,response to toxic substance|GO:0098869,cellular oxidant detoxification|GO:1990748,cellular	GO:0043229,intracellular organelle|GO:0001669,acrosomal vesicle|GO:0005929,cilium|GO:0043227,membrane-bounded organelle|GO:0043226,organelle|GO:0005737,cytoplasm|GO:1903561,extracellular vesicle|GO:0070062,extracellular exosome|GO:0016023,cytoplasmic, membrane-bounded vesicle|GO:0031410,cytoplasmic vesicle|GO:0036126,sperm flagellum|GO:0097223,sperm part|GO:0005739,mitochondrion|GO:0031988,membrane-bounded vesicle|GO:0005794,Golgi apparatus|GO:0030141,secretory granule|GO:0012505,endomembrane system|GO:0042995,cell projection|GO:0031982,vesicle|GO:0043230,extracellular organelle|GO:0043231,intracellular membrane-bounded organelle|GO:0044464,cell part|GO:0005623,cell|GO:0005622,intracellular|GO:0031514,motile cilium|GO:0044444,cytoplasmic part|GO:0005576,extracellular region|GO:0044424,intracellular part|GO:0065010,extracellular membrane-bounded organelle|GO:0044421,extracellular region part|GO:0099503,secretory vesicle|GO:0097708,intracellular vesicle	GO:0003824,catalytic activity|GO:0016671,oxidoreductase activity, acting on a sulfur group of donors, disulfide as acceptor|GO:0016667,oxidoreductase activity, acting on a sulfur group of donors|GO:0015036,disulfide oxidoreductase activity|GO:0015035,protein disulfide oxidoreductase activity|GO:0016491,oxidoreductase activity|GO:0016209,antioxidant activity|GO:0047134,protein-disulfide reductase activity|GO:0016651,oxidoreductase activity, acting on NAD(P)H|GO:0016668,oxidoreductase activity, acting on a sulfur group of donors, NAD(P) as acceptor|GO:0004791,thioredoxin-disulfide reductase activity
ZNF273	GO:0080090,regulation of primary metabolic process|GO:0019222,regulation of metabolic process|GO:0031326,regulation of cellular biosynthetic process|GO:0031323,regulation of cellular metabolic process|GO:0090304,nucleic acid metabolic process|GO:0044249,cellular biosynthetic process|GO:0034641,cellular nitrogen compound metabolic process|GO:0006807,nitrogen compound metabolic process|GO:0034645,cellular macromolecule biosynthetic process|GO:1901362,organic cyclic compound biosynthetic process|GO:0050789,regulation of biological process|GO:0097659,nucleic acid-templated transcription|GO:0032774,RNA biosynthetic process|GO:0006139,nucleobase-containing compound metabolic process|GO:0044260,cellular macromolecule metabolic process|GO:0071704,organic substance metabolic process|GO:2000112,regulation of cellular macromolecule biosynthetic process|GO:0060255,regulation of macromolecule metabolic process|GO:0010467,gene expression|GO:0065007,biological regulation|GO:1901360,organic cyclic compound metabolic process|GO:0010468,regulation of gene expression|GO:0018130,heterocycle biosynthetic process|GO:1901576,organic substance biosynthetic process|GO:0019219,regulation of nucleobase-containing compound metabolic process|GO:0006725,cellular aromatic compound metabolic process|GO:0009987,cellular process|GO:0009889,regulation of biosynthetic process|GO:1903506,regulation of nucleic acid-templated transcription|GO:0050794,regulation of cellular process|GO:0009058,biosynthetic process|GO:0009059,macromolecule biosynthetic process|GO:0051171,regulation of nitrogen compound metabolic process|GO:0008152,metabolic process|GO:2001141,regulation of RNA biosynthetic process|GO:0034654,nucleobase-containing compound biosynthetic process|GO:0046483,heterocycle metabolic process|GO:0016070,RNA metabolic process|GO:0044238,primary metabolic process|GO:0044271,cellular nitrogen compound biosynthetic process|GO:0051252,regulation of RNA metabolic process|GO:0044237,cellular metabolic process|GO:0043170,macromolecule metabolic process|GO:0006355,regulation of transcription, DNA-templated|GO:0010556,regulation of macromolecule biosynthetic process|GO:0006351,transcription, DNA-templated|GO:0019438,aromatic compound biosynthetic process	GO:0043231,intracellular membrane-bounded organelle|GO:0005634,nucleus|GO:0044464,cell part|GO:0005623,cell|GO:0005622,intracellular|GO:0043229,intracellular organelle|GO:0044424,intracellular part|GO:0043227,membrane-bounded organelle|GO:0043226,organelle	GO:0043169,cation binding|GO:0005515,protein binding|GO:0097159,organic cyclic compound binding|GO:0043167,ion binding|GO:0005488,binding|GO:0003676,nucleic acid binding|GO:0003677,DNA binding|GO:0046872,metal ion binding|GO:1901363,heterocyclic compound binding|GO:0003700,transcription factor activity, sequence-specific DNA binding|GO:0001071,nucleic acid binding transcription factor activity|GO:0000981,RNA polymerase II transcription factor activity, sequence-specific DNA binding
KLK11	GO:0044238,primary metabolic process|GO:0019538,protein metabolic process|GO:0043170,macromolecule metabolic process|GO:0071704,organic substance metabolic process|GO:0008152,metabolic process|GO:0006508,proteolysis|GO:1901564,organonitrogen compound metabolic process|GO:0006807,nitrogen compound metabolic process	GO:0043229,intracellular organelle|GO:0043227,membrane-bounded organelle|GO:0043226,organelle|GO:0005737,cytoplasm|GO:0031982,vesicle|GO:0031988,membrane-bounded vesicle|GO:0005794,Golgi apparatus|GO:0012505,endomembrane system|GO:1903561,extracellular vesicle|GO:0005615,extracellular space|GO:0043230,extracellular organelle|GO:0043231,intracellular membrane-bounded organelle|GO:0070062,extracellular exosome|GO:0044464,cell part|GO:0005623,cell|GO:0005622,intracellular|GO:0044444,cytoplasmic part|GO:0005576,extracellular region|GO:0044424,intracellular part|GO:0065010,extracellular membrane-bounded organelle|GO:0044421,extracellular region part	GO:0004252,serine-type endopeptidase activity|GO:0004175,endopeptidase activity|GO:0017171,serine hydrolase activity|GO:0016787,hydrolase activity|GO:0003824,catalytic activity|GO:0070011,peptidase activity, acting on L-amino acid peptides|GO:0008233,peptidase activity|GO:0008236,serine-type peptidase activity
OR12D2	GO:0009593,detection of chemical stimulus|GO:0050906,detection of stimulus involved in sensory perception|GO:0007165,signal transduction|GO:0042221,response to chemical|GO:0050789,regulation of biological process|GO:0044699,single-organism process|GO:0051716,cellular response to stimulus|GO:0065007,biological regulation|GO:0007186,G-protein coupled receptor signaling pathway|GO:0032501,multicellular organismal process|GO:0007608,sensory perception of smell|GO:0050877,neurological system process|GO:0007606,sensory perception of chemical stimulus|GO:0007600,sensory perception|GO:0050794,regulation of cellular process|GO:0050911,detection of chemical stimulus involved in sensory perception of smell|GO:0044763,single-organism cellular process|GO:0007154,cell communication|GO:0003008,system process|GO:0044700,single organism signaling|GO:0051606,detection of stimulus|GO:0044707,single-multicellular organism process|GO:0050896,response to stimulus|GO:0023052,signaling|GO:0009987,cellular process|GO:0050907,detection of chemical stimulus involved in sensory perception	GO:0016021,integral component of membrane|GO:0016020,membrane|GO:0044464,cell part|GO:0005623,cell|GO:0071944,cell periphery|GO:0005886,plasma membrane|GO:0044425,membrane part|GO:0031224,intrinsic component of membrane	GO:0004930,G-protein coupled receptor activity|GO:0038023,signaling receptor activity|GO:0060089,molecular transducer activity|GO:0004888,transmembrane signaling receptor activity|GO:0004872,receptor activity|GO:0004871,signal transducer activity|GO:0004984,olfactory receptor activity|GO:0099600,transmembrane receptor activity
PTER	GO:0032502,developmental process|GO:0060429,epithelium development|GO:0048869,cellular developmental process|GO:0030154,cell differentiation|GO:0009888,tissue development|GO:0044767,single-organism developmental process|GO:0044763,single-organism cellular process|GO:0008152,metabolic process|GO:0030855,epithelial cell differentiation|GO:0009987,cellular process|GO:0009056,catabolic process|GO:0044699,single-organism process|GO:0048856,anatomical structure development	GO:0043230,extracellular organelle|GO:0031982,vesicle|GO:0044421,extracellular region part|GO:0043227,membrane-bounded organelle|GO:0031988,membrane-bounded vesicle|GO:0005576,extracellular region|GO:1903561,extracellular vesicle|GO:0065010,extracellular membrane-bounded organelle|GO:0043226,organelle|GO:0070062,extracellular exosome	GO:0016787,hydrolase activity|GO:0046914,transition metal ion binding|GO:0043169,cation binding|GO:0016788,hydrolase activity, acting on ester bonds|GO:0043167,ion binding|GO:0003824,catalytic activity|GO:0008270,zinc ion binding|GO:0046872,metal ion binding|GO:0005488,binding
AGPAT9	GO:0006654,phosphatidic acid biosynthetic process|GO:0044238,primary metabolic process|GO:0006650,glycerophospholipid metabolic process|GO:0046341,CDP-diacylglycerol metabolic process|GO:0006639,acylglycerol metabolic process|GO:0006638,neutral lipid metabolic process|GO:0048583,regulation of response to stimulus|GO:0044249,cellular biosynthetic process|GO:0023052,signaling|GO:0007165,signal transduction|GO:0023051,regulation of signaling|GO:1901576,organic substance biosynthetic process|GO:0035556,intracellular signal transduction|GO:0010646,regulation of cell communication|GO:0044255,cellular lipid metabolic process|GO:0050789,regulation of biological process|GO:0044699,single-organism process|GO:0045017,glycerolipid biosynthetic process|GO:0044710,single-organism metabolic process|GO:0044711,single-organism biosynthetic process|GO:0009966,regulation of signal transduction|GO:0016024,CDP-diacylglycerol biosynthetic process|GO:0071704,organic substance metabolic process|GO:0065007,biological regulation|GO:0046463,acylglycerol biosynthetic process|GO:0046460,neutral lipid biosynthetic process|GO:0006644,phospholipid metabolic process|GO:0006629,lipid metabolic process|GO:0006641,triglyceride metabolic process|GO:0009987,cellular process|GO:0031929,TOR signaling|GO:0051716,cellular response to stimulus|GO:0050794,regulation of cellular process|GO:0009058,biosynthetic process|GO:0044763,single-organism cellular process|GO:0008152,metabolic process|GO:0046474,glycerophospholipid biosynthetic process|GO:0007154,cell communication|GO:0046486,glycerolipid metabolic process|GO:1902531,regulation of intracellular signal transduction|GO:0090407,organophosphate biosynthetic process|GO:0008610,lipid biosynthetic process|GO:0044700,single organism signaling|GO:0008654,phospholipid biosynthetic process|GO:0050896,response to stimulus|GO:0044237,cellular metabolic process|GO:0006796,phosphate-containing compound metabolic process|GO:0019432,triglyceride biosynthetic process|GO:0006793,phosphorus metabolic process|GO:0019637,organophosphate metabolic process|GO:0032006,regulation of TOR signaling|GO:0046473,phosphatidic acid metabolic process|GO:0044281,small molecule metabolic process	GO:0005783,endoplasmic reticulum|GO:0005789,endoplasmic reticulum membrane|GO:0042175,nuclear outer membrane-endoplasmic reticulum membrane network|GO:0043229,intracellular organelle|GO:0043227,membrane-bounded organelle|GO:0043226,organelle|GO:0031224,intrinsic component of membrane|GO:0005737,cytoplasm|GO:0031090,organelle membrane|GO:0016021,integral component of membrane|GO:0016020,membrane|GO:0044432,endoplasmic reticulum part|GO:0098588,bounding membrane of organelle|GO:0012505,endomembrane system|GO:0043231,intracellular membrane-bounded organelle|GO:0044464,cell part|GO:0005623,cell|GO:0005622,intracellular|GO:0044446,intracellular organelle part|GO:0044444,cytoplasmic part|GO:0044424,intracellular part|GO:0044425,membrane part|GO:0044422,organelle part|GO:0031984,organelle subcompartment|GO:0098827,endoplasmic reticulum subcompartment	GO:0004366,glycerol-3-phosphate O-acyltransferase activity|GO:0016740,transferase activity|GO:0003824,catalytic activity|GO:0003841,1-acylglycerol-3-phosphate O-acyltransferase activity|GO:0071617,lysophospholipid acyltransferase activity|GO:0042171,lysophosphatidic acid acyltransferase activity|GO:0016411,acylglycerol O-acyltransferase activity|GO:0008374,O-acyltransferase activity|GO:0016746,transferase activity, transferring acyl groups|GO:0016747,transferase activity, transferring acyl groups other than amino-acyl groups
KNCN		GO:0098590,plasma membrane region|GO:0032437,cuticular plate|GO:0043229,intracellular organelle|GO:0043228,non-membrane-bounded organelle|GO:0005929,cilium|GO:0005815,microtubule organizing center|GO:0043226,organelle|GO:0031224,intrinsic component of membrane|GO:0015629,actin cytoskeleton|GO:0005856,cytoskeleton|GO:0036064,ciliary basal body|GO:0045177,apical part of cell|GO:0072372,primary cilium|GO:0016021,integral component of membrane|GO:0016020,membrane|GO:0031513,nonmotile primary cilium|GO:0097458,neuron part|GO:0044430,cytoskeletal part|GO:0044459,plasma membrane part|GO:0032421,stereocilium bundle|GO:0005737,cytoplasm|GO:0015630,microtubule cytoskeleton|GO:0016324,apical plasma membrane|GO:0098589,membrane region|GO:0044297,cell body|GO:0005938,cell cortex|GO:0005886,plasma membrane|GO:0042995,cell projection|GO:0060091,kinocilium|GO:0005622,intracellular|GO:0043232,intracellular non-membrane-bounded organelle|GO:0030863,cortical cytoskeleton|GO:0043025,neuronal cell body|GO:0044463,cell projection part|GO:0044464,cell part|GO:0005623,cell|GO:0030864,cortical actin cytoskeleton|GO:0044446,intracellular organelle part|GO:0044444,cytoplasmic part|GO:0071944,cell periphery|GO:0044448,cell cortex part|GO:0044441,ciliary part|GO:0044424,intracellular part|GO:0044425,membrane part|GO:0098805,whole membrane|GO:0044422,organelle part|GO:0036477,somatodendritic compartment|GO:0098862,cluster of actin-based cell projections|GO:0099568,cytoplasmic region	
PNOC	GO:0023052,signaling|GO:0007165,signal transduction|GO:0050789,regulation of biological process|GO:0044699,single-organism process|GO:0007218,neuropeptide signaling pathway|GO:0065007,biological regulation|GO:0051704,multi-organism process|GO:0007186,G-protein coupled receptor signaling pathway|GO:0032501,multicellular organismal process|GO:0050877,neurological system process|GO:0009987,cellular process|GO:0051716,cellular response to stimulus|GO:0007600,sensory perception|GO:0022414,reproductive process|GO:0044763,single-organism cellular process|GO:0007268,chemical synaptic transmission|GO:0007267,cell-cell signaling|GO:0007154,cell communication|GO:0050794,regulation of cellular process|GO:0003008,system process|GO:0044700,single organism signaling|GO:0044703,multi-organism reproductive process|GO:0044707,single-multicellular organism process|GO:0044706,multi-multicellular organism process|GO:0007565,female pregnancy|GO:0050896,response to stimulus|GO:0000003,reproduction|GO:0098916,anterograde trans-synaptic signaling|GO:0099536,synaptic signaling|GO:0099537,trans-synaptic signaling	GO:0005576,extracellular region|GO:0044306,neuron projection terminus|GO:0043679,axon terminus|GO:0044297,cell body|GO:0016020,membrane|GO:0071944,cell periphery|GO:0030425,dendrite|GO:0005623,cell|GO:0097458,neuron part|GO:0044456,synapse part|GO:0098793,presynapse|GO:0045202,synapse|GO:0043005,neuron projection|GO:0005886,plasma membrane|GO:0044464,cell part|GO:0043025,neuronal cell body|GO:0036477,somatodendritic compartment	GO:0005179,hormone activity|GO:0005184,neuropeptide hormone activity|GO:0001515,opioid peptide activity|GO:0005488,binding|GO:0005515,protein binding|GO:0005102,receptor binding|GO:0031628,opioid receptor binding|GO:0001664,G-protein coupled receptor binding
GIMAP2		GO:0005737,cytoplasm|GO:0016020,membrane|GO:0043232,intracellular non-membrane-bounded organelle|GO:0016021,integral component of membrane|GO:0005811,lipid particle|GO:0044464,cell part|GO:0005623,cell|GO:0005622,intracellular|GO:0043229,intracellular organelle|GO:0043228,non-membrane-bounded organelle|GO:0044424,intracellular part|GO:0044425,membrane part|GO:0043226,organelle|GO:0031224,intrinsic component of membrane	GO:0043168,anion binding|GO:0005488,binding|GO:0019001,guanyl nucleotide binding|GO:0035639,purine ribonucleoside triphosphate binding|GO:0097367,carbohydrate derivative binding|GO:0043167,ion binding|GO:1901363,heterocyclic compound binding|GO:1901265,nucleoside phosphate binding|GO:0000166,nucleotide binding|GO:0036094,small molecule binding|GO:0032561,guanyl ribonucleotide binding|GO:0032553,ribonucleotide binding|GO:0032549,ribonucleoside binding|GO:0032555,purine ribonucleotide binding|GO:0017076,purine nucleotide binding|GO:0005525,GTP binding|GO:0097159,organic cyclic compound binding|GO:0032550,purine ribonucleoside binding|GO:0001883,purine nucleoside binding|GO:0001882,nucleoside binding|GO:0005515,protein binding|GO:0042802,identical protein binding
KRTAP13			
C21orf54			
ZNF549	GO:0080090,regulation of primary metabolic process|GO:0019222,regulation of metabolic process|GO:0031326,regulation of cellular biosynthetic process|GO:0031323,regulation of cellular metabolic process|GO:0090304,nucleic acid metabolic process|GO:0044249,cellular biosynthetic process|GO:0034641,cellular nitrogen compound metabolic process|GO:0006807,nitrogen compound metabolic process|GO:0034645,cellular macromolecule biosynthetic process|GO:1901362,organic cyclic compound biosynthetic process|GO:0050789,regulation of biological process|GO:0097659,nucleic acid-templated transcription|GO:0032774,RNA biosynthetic process|GO:0006139,nucleobase-containing compound metabolic process|GO:0044260,cellular macromolecule metabolic process|GO:0071704,organic substance metabolic process|GO:2000112,regulation of cellular macromolecule biosynthetic process|GO:0060255,regulation of macromolecule metabolic process|GO:0010467,gene expression|GO:0065007,biological regulation|GO:1901360,organic cyclic compound metabolic process|GO:0010468,regulation of gene expression|GO:0018130,heterocycle biosynthetic process|GO:1901576,organic substance biosynthetic process|GO:0019219,regulation of nucleobase-containing compound metabolic process|GO:0006725,cellular aromatic compound metabolic process|GO:0009987,cellular process|GO:0009889,regulation of biosynthetic process|GO:1903506,regulation of nucleic acid-templated transcription|GO:0050794,regulation of cellular process|GO:0009058,biosynthetic process|GO:0009059,macromolecule biosynthetic process|GO:0051171,regulation of nitrogen compound metabolic process|GO:0008152,metabolic process|GO:2001141,regulation of RNA biosynthetic process|GO:0034654,nucleobase-containing compound biosynthetic process|GO:0046483,heterocycle metabolic process|GO:0016070,RNA metabolic process|GO:0044238,primary metabolic process|GO:0044271,cellular nitrogen compound biosynthetic process|GO:0051252,regulation of RNA metabolic process|GO:0044237,cellular metabolic process|GO:0043170,macromolecule metabolic process|GO:0006355,regulation of transcription, DNA-templated|GO:0010556,regulation of macromolecule biosynthetic process|GO:0006351,transcription, DNA-templated|GO:0019438,aromatic compound biosynthetic process	GO:0044446,intracellular organelle part|GO:0043231,intracellular membrane-bounded organelle|GO:0031981,nuclear lumen|GO:0043233,organelle lumen|GO:0005634,nucleus|GO:0044464,cell part|GO:0031974,membrane-enclosed lumen|GO:0005622,intracellular|GO:0005654,nucleoplasm|GO:0070013,intracellular organelle lumen|GO:0043229,intracellular organelle|GO:0044428,nuclear part|GO:0005623,cell|GO:0044424,intracellular part|GO:0043227,membrane-bounded organelle|GO:0043226,organelle|GO:0044422,organelle part	GO:0044212,transcription regulatory region DNA binding|GO:0001071,nucleic acid binding transcription factor activity|GO:0043565,sequence-specific DNA binding|GO:0003700,transcription factor activity, sequence-specific DNA binding|GO:0001012,RNA polymerase II regulatory region DNA binding|GO:0003677,DNA binding|GO:0001067,regulatory region nucleic acid binding|GO:0043169,cation binding|GO:0043167,ion binding|GO:0000976,transcription regulatory region sequence-specific DNA binding|GO:0000977,RNA polymerase II regulatory region sequence-specific DNA binding|GO:0003676,nucleic acid binding|GO:0000975,regulatory region DNA binding|GO:0046872,metal ion binding|GO:0097159,organic cyclic compound binding|GO:1901363,heterocyclic compound binding|GO:0005488,binding|GO:0000981,RNA polymerase II transcription factor activity, sequence-specific DNA binding
WFDC11		GO:0005576,extracellular region	

DDIT3	GO:2001235,positive regulation of apoptotic signaling pathway|GO:0080090,regulation of primary metabolic process|GO:0019222,regulation of metabolic process|GO:2001233,regulation of apoptotic signaling pathway|GO:2000677,regulation of transcription regulatory region DNA binding|GO:0048585,negative regulation of response to stimulus|GO:0048584,positive regulation of response to stimulus|GO:0048583,regulation of response to stimulus|GO:0051283,negative regulation of sequestering of calcium ion|GO:0032845,negative regulation of homeostatic process|GO:0032844,regulation of homeostatic process|GO:0009299,mRNA transcription|GO:0030111,regulation of Wnt signaling pathway|GO:0051898,negative regulation of protein kinase B signaling|GO:0007165,signal transduction|GO:0007166,cell surface receptor signaling pathway|GO:0048262,determination of dorsal/ventral asymmetry|GO:0048263,determination of dorsal identity|GO:0044324,regulation of transcription involved in anterior/posterior axis specification|GO:1901362,organic cyclic compound biosynthetic process|GO:0051896,regulation of protein kinase B signaling|GO:1901360,organic cyclic compound metabolic process|GO:0036003,positive regulation of transcription from RNA polymerase II promoter in response to stress|GO:0006984,ER-nucleus signaling pathway|GO:0051716,cellular response to stimulus|GO:0006986,response to unfolded protein|GO:0010605,negative regulation of macromolecule metabolic process|GO:0010604,positive regulation of macromolecule metabolic process|GO:0009968,negative regulation of signal transduction|GO:0009966,regulation of signal transduction|GO:0009967,positive regulation of signal transduction|GO:1901216,positive regulation of neuron death|GO:0071310,cellular response to organic substance|GO:0051208,sequestering of calcium ion|GO:0051209,release of sequestered calcium ion into cytosol|GO:0045661,regulation of myoblast differentiation|GO:0045662,negative regulation of myoblast differentiation|GO:0048518,positive regulation of biological process|GO:0048519,negative regulation of biological process|GO:0016482,cytosolic transport|GO:0050801,ion homeostasis|GO:0019725,cellular homeostasis|GO:0009058,biosynthetic process|GO:0010467,gene expression|GO:0060255,regulation of macromolecule metabolic process|GO:0051282,regulation of sequestering of calcium ion|GO:0034645,cellular macromolecule biosynthetic process|GO:0042221,response to chemical|GO:0003002,regionalization|GO:2001141,regulation of RNA biosynthetic process|GO:0072511,divalent inorganic cation transport|GO:0043433,negative regulation of sequence-specific DNA binding transcription factor activity|GO:0071695,anatomical structure maturation|GO:0046483,heterocycle metabolic process|GO:0044257,cellular protein catabolic process|GO:0044700,single organism signaling|GO:0009607,response to biotic stimulus|GO:0044707,single-multicellular organism process|GO:0030968,endoplasmic reticulum unfolded protein response|GO:0019438,aromatic compound biosynthetic process|GO:0019538,protein metabolic process|GO:0016055,Wnt signaling pathway|GO:0072359,circulatory system development|GO:0030163,protein catabolic process|GO:0043068,positive regulation of programmed cell death|GO:0043523,regulation of neuron apoptotic process|GO:0033554,cellular response to stress|GO:0006511,ubiquitin-dependent protein catabolic process|GO:0097285,obsolete cell-type specific apoptotic process|GO:0042692,muscle cell differentiation|GO:0001955,blood vessel maturation|GO:0060828,regulation of canonical Wnt signaling pathway|GO:0043525,positive regulation of neuron apoptotic process|GO:0051098,regulation of binding|GO:0009892,negative regulation of metabolic process|GO:0009893,positive regulation of metabolic process|GO:0009890,negative regulation of biosynthetic process|GO:0009891,positive regulation of biosynthetic process|GO:0055065,metal ion homeostasis|GO:1902235,regulation of endoplasmic reticulum stress-induced intrinsic apoptotic signaling pathway|GO:0032792,negative regulation of CREB transcription factor activity|GO:1902237,positive regulation of endoplasmic reticulum stress-induced intrinsic apoptotic signaling pathway|GO:0007204,positive regulation of cytosolic calcium ion concentration|GO:0008152,metabolic process|GO:0061061,muscle structure development|GO:0010629,negative regulation of gene expression|GO:0010628,positive regulation of gene expression|GO:0098771,inorganic ion homeostasis|GO:0035556,intracellular signal transduction|GO:0048878,chemical homeostasis|GO:0042981,regulation of apoptotic process|GO:0030178,negative regulation of Wnt signaling pathway|GO:0097659,nucleic acid-templated transcription|GO:0060401,cytosolic calcium ion transport|GO:0009605,response to external stimulus|GO:0001816,cytokine production|GO:1901575,organic substance catabolic process|GO:0044265,cellular macromolecule catabolic process|GO:0044260,cellular macromolecule metabolic process|GO:0070997,neuron death|GO:0001568,blood vessel development|GO:2000016,negative regulation of determination of dorsal identity|GO:2000015,regulation of determination of dorsal identity|GO:0035966,response to topologically incorrect protein|GO:0006873,cellular ion homeostasis|GO:0044699,single-organism process|GO:0006366,transcription from RNA polymerase II promoter|GO:0071216,cellular response to biotic stimulus|GO:0065009,regulation of molecular function|GO:0065008,regulation of biological quality|GO:0018130,heterocycle biosynthetic process|GO:0016265,obsolete death|GO:1990440,positive regulation of transcription from RNA polymerase II promoter in response to endoplasmic reticulum stress|GO:1902582,single-organism intracellular transport|GO:0006139,nucleobase-containing compound metabolic process|GO:0050793,regulation of developmental process|GO:0006812,cation transport|GO:0006811,ion transport|GO:0006810,transport|GO:0009889,regulation of biosynthetic process|GO:0006816,calcium ion transport|GO:0050794,regulation of cellular process|GO:0007050,cell cycle arrest|GO:0060070,canonical Wnt signaling pathway|GO:0006950,response to stress|GO:0043392,negative regulation of DNA binding|GO:0051239,regulation of multicellular organismal process|GO:1903026,negative regulation of RNA polymerase II regulatory region sequence-specific DNA binding|GO:1902532,negative regulation of intracellular signal transduction|GO:1902533,positive regulation of intracellular signal transduction|GO:1901214,regulation of neuron death|GO:1902531,regulation of intracellular signal transduction|GO:0016070,RNA metabolic process|GO:0044767,single-organism developmental process|GO:0034976,response to endoplasmic reticulum stress|GO:0009953,dorsal/ventral pattern formation|GO:0006983,ER overload response|GO:0046907,intracellular transport|GO:0070059,intrinsic apoptotic signaling pathway in response to endoplasmic reticulum stress|GO:0043491,protein kinase B signaling|GO:0080134,regulation of response to stress|GO:0080135,regulation of cellular response to stress|GO:0010498,proteasomal protein catabolic process|GO:0044765,single-organism transport|GO:0006355,regulation of transcription, DNA-templated|GO:0010557,positive regulation of macromolecule biosynthetic process|GO:0006357,regulation of transcription from RNA polymerase II promoter|GO:0006351,transcription, DNA-templated|GO:0048869,cellular developmental process|GO:0055082,cellular chemical homeostasis|GO:2000678,negative regulation of transcription regulatory region DNA binding|GO:0010558,negative regulation of macromolecule biosynthetic process|GO:0032774,RNA biosynthetic process|GO:0043632,modification-dependent macromolecule catabolic process|GO:0055074,calcium ion homeostasis|GO:0030154,cell differentiation|GO:0023056,positive regulation of signaling|GO:0023057,negative regulation of signaling|GO:0034641,cellular nitrogen compound metabolic process|GO:0023052,signaling|GO:0010648,negative regulation of cell communication|GO:0070887,cellular response to chemical stimulus|GO:0023051,regulation of signaling|GO:0009799,specification of symmetry|GO:0009798,axis specification|GO:0010647,positive regulation of cell communication|GO:0010646,regulation of cell communication|GO:0009952,anterior/posterior pattern specification|GO:0051235,maintenance of location|GO:0007049,cell cycle|GO:0072358,cardiovascular system development|GO:0072507,divalent inorganic cation homeostasis|GO:0051234,establishment of localization|GO:0072503,cellular divalent inorganic cation homeostasis|GO:0051649,establishment of localization in cell|GO:0000122,negative regulation of transcription from RNA polymerase II promoter|GO:0043161,proteasome-mediated ubiquitin-dependent protein catabolic process|GO:0051240,positive	GO:0031974,membrane-enclosed lumen|GO:1990617,CHOP-ATF4 complex|GO:0043229,intracellular organelle|GO:0036488,CHOP-C/EBP complex|GO:0043227,membrane-bounded organelle|GO:0043226,organelle|GO:0005737,cytoplasm|GO:0005654,nucleoplasm|GO:0031981,nuclear lumen|GO:0005634,nucleus|GO:0005770,late endosome|GO:0035976,transcription factor AP-1 complex|GO:0044798,nuclear transcription factor complex|GO:0012505,endomembrane system|GO:1990622,CHOP-ATF3 complex|GO:0043234,protein complex|GO:0032991,macromolecular complex|GO:0043231,intracellular membrane-bounded organelle|GO:0032993,protein-DNA complex|GO:0043233,organelle lumen|GO:0005829,cytosol|GO:0044464,cell part|GO:0090575,RNA polymerase II transcription factor complex|GO:0005623,cell|GO:0005622,intracellular|GO:0044446,intracellular organelle part|GO:0070013,intracellular organelle lumen|GO:0044444,cytoplasmic part|GO:0044428,nuclear part|GO:0005667,transcription factor complex|GO:0044424,intracellular part|GO:0005768,endosome|GO:0044422,organelle part|GO:0031982,vesicle|GO:0031410,cytoplasmic vesicle|GO:0097708,intracellular vesicle	GO:0008140,cAMP response element binding protein binding|GO:0001012,RNA polymerase II regulatory region DNA binding|GO:0001159,core promoter proximal region DNA binding|GO:0043522,leucine zipper domain binding|GO:0001077,transcriptional activator activity, RNA polymerase II core promoter proximal region sequence-specific binding|GO:0005488,binding|GO:0003676,nucleic acid binding|GO:0003677,DNA binding|GO:1901363,heterocyclic compound binding|GO:0043565,sequence-specific DNA binding|GO:0003714,transcription corepressor activity|GO:0003712,transcription cofactor activity|GO:0000989,transcription factor activity, transcription factor binding|GO:0000988,transcription factor activity, protein binding|GO:0000987,core promoter proximal region sequence-specific DNA binding|GO:0000982,transcription factor activity, RNA polymerase II core promoter proximal region sequence-specific binding|GO:0042802,identical protein binding|GO:0042803,protein homodimerization activity|GO:0008134,transcription factor binding|GO:0001067,regulatory region nucleic acid binding|GO:0019904,protein domain specific binding|GO:0030275,LRR domain binding|GO:0000978,RNA polymerase II core promoter proximal region sequence-specific DNA binding|GO:0044212,transcription regulatory region DNA binding|GO:0003700,transcription factor activity, sequence-specific DNA binding|GO:0097159,organic cyclic compound binding|GO:0001071,nucleic acid binding transcription factor activity|GO:0001228,transcriptional activator activity, RNA polymerase II transcription regulatory region sequence-specific binding|GO:0000976,transcription regulatory region sequence-specific DNA binding|GO:0000977,RNA polymerase II regulatory region sequence-specific DNA binding|GO:0005515,protein binding|GO:0000975,regulatory region DNA binding|GO:0046983,protein dimerization activity|GO:0046982,protein heterodimerization activity|GO:0000981,RNA polymerase II transcription factor activity, sequence-specific DNA binding|GO:1990837,sequence-specific double-stranded DNA binding|GO:0003690,double-stranded DNA binding
TCEAL7	GO:0009892,negative regulation of metabolic process|GO:0080090,regulation of primary metabolic process|GO:0009890,negative regulation of biosynthetic process|GO:0031327,negative regulation of cellular biosynthetic process|GO:0031326,regulation of cellular biosynthetic process|GO:0031324,negative regulation of cellular metabolic process|GO:0031323,regulation of cellular metabolic process|GO:0090304,nucleic acid metabolic process|GO:0044249,cellular biosynthetic process|GO:0010629,negative regulation of gene expression|GO:0006807,nitrogen compound metabolic process|GO:0034645,cellular macromolecule biosynthetic process|GO:0043170,macromolecule metabolic process|GO:1901362,organic cyclic compound biosynthetic process|GO:0050789,regulation of biological process|GO:0097659,nucleic acid-templated transcription|GO:0032774,RNA biosynthetic process|GO:0006139,nucleobase-containing compound metabolic process|GO:0010605,negative regulation of macromolecule metabolic process|GO:0019222,regulation of metabolic process|GO:0044260,cellular macromolecule metabolic process|GO:0071704,organic substance metabolic process|GO:2000112,regulation of cellular macromolecule biosynthetic process|GO:2000113,negative regulation of cellular macromolecule biosynthetic process|GO:0060255,regulation of macromolecule metabolic process|GO:0010467,gene expression|GO:0065007,biological regulation|GO:1901360,organic cyclic compound metabolic process|GO:0044092,negative regulation of molecular function|GO:0048519,negative regulation of biological process|GO:0065009,regulation of molecular function|GO:0010468,regulation of gene expression|GO:0018130,heterocycle biosynthetic process|GO:0045934,negative regulation of nucleobase-containing compound metabolic process|GO:1901576,organic substance biosynthetic process|GO:0019219,regulation of nucleobase-containing compound metabolic process|GO:0006725,cellular aromatic compound metabolic process|GO:0009987,cellular process|GO:0009889,regulation of biosynthetic process|GO:1903506,regulation of nucleic acid-templated transcription|GO:1903507,negative regulation of nucleic acid-templated transcription|GO:1902679,negative regulation of RNA biosynthetic process|GO:0009058,biosynthetic process|GO:0009059,macromolecule biosynthetic process|GO:0045892,negative regulation of transcription, DNA-templated|GO:0051171,regulation of nitrogen compound metabolic process|GO:0051172,negative regulation of nitrogen compound metabolic process|GO:2001141,regulation of RNA biosynthetic process|GO:0034654,nucleobase-containing compound biosynthetic process|GO:0050794,regulation of cellular process|GO:0008152,metabolic process|GO:0043433,negative regulation of sequence-specific DNA binding transcription factor activity|GO:0046483,heterocycle metabolic process|GO:0016070,RNA metabolic process|GO:0044238,primary metabolic process|GO:0051090,regulation of sequence-specific DNA binding transcription factor activity|GO:0044271,cellular nitrogen compound biosynthetic process|GO:0051253,negative regulation of RNA metabolic process|GO:0051252,regulation of RNA metabolic process|GO:0034641,cellular nitrogen compound metabolic process|GO:0044237,cellular metabolic process|GO:0032088,negative regulation of NF-kappaB transcription factor activity|GO:0006355,regulation of transcription, DNA-templated|GO:0010556,regulation of macromolecule biosynthetic process|GO:0006351,transcription, DNA-templated|GO:0019438,aromatic compound biosynthetic process|GO:0010558,negative regulation of macromolecule biosynthetic process|GO:0048523,negative regulation of cellular process|GO:0006366,transcription from RNA polymerase II promoter|GO:0006357,regulation of transcription from RNA polymerase II promoter	GO:0043231,intracellular membrane-bounded organelle|GO:0005634,nucleus|GO:0044464,cell part|GO:0005623,cell|GO:0005622,intracellular|GO:0043229,intracellular organelle|GO:0044424,intracellular part|GO:0043227,membrane-bounded organelle|GO:0043226,organelle	GO:0005488,binding|GO:0019904,protein domain specific binding|GO:0005515,protein binding|GO:0050699,WW domain binding
ZNF623	GO:0080090,regulation of primary metabolic process|GO:0019222,regulation of metabolic process|GO:0031326,regulation of cellular biosynthetic process|GO:0031323,regulation of cellular metabolic process|GO:0090304,nucleic acid metabolic process|GO:0044249,cellular biosynthetic process|GO:0034641,cellular nitrogen compound metabolic process|GO:0006807,nitrogen compound metabolic process|GO:0034645,cellular macromolecule biosynthetic process|GO:1901362,organic cyclic compound biosynthetic process|GO:0050789,regulation of biological process|GO:0097659,nucleic acid-templated transcription|GO:0032774,RNA biosynthetic process|GO:0006139,nucleobase-containing compound metabolic process|GO:0044260,cellular macromolecule metabolic process|GO:0071704,organic substance metabolic process|GO:2000112,regulation of cellular macromolecule biosynthetic process|GO:0060255,regulation of macromolecule metabolic process|GO:0010467,gene expression|GO:0065007,biological regulation|GO:1901360,organic cyclic compound metabolic process|GO:0010468,regulation of gene expression|GO:0018130,heterocycle biosynthetic process|GO:1901576,organic substance biosynthetic process|GO:0019219,regulation of nucleobase-containing compound metabolic process|GO:0006725,cellular aromatic compound metabolic process|GO:0009987,cellular process|GO:0009889,regulation of biosynthetic process|GO:1903506,regulation of nucleic acid-templated transcription|GO:0050794,regulation of cellular process|GO:0009058,biosynthetic process|GO:0009059,macromolecule biosynthetic process|GO:0051171,regulation of nitrogen compound metabolic process|GO:0008152,metabolic process|GO:2001141,regulation of RNA biosynthetic process|GO:0034654,nucleobase-containing compound biosynthetic process|GO:0046483,heterocycle metabolic process|GO:0016070,RNA metabolic process|GO:0044238,primary metabolic process|GO:0044271,cellular nitrogen compound biosynthetic process|GO:0051252,regulation of RNA metabolic process|GO:0044237,cellular metabolic process|GO:0043170,macromolecule metabolic process|GO:0006355,regulation of transcription, DNA-templated|GO:0010556,regulation of macromolecule biosynthetic process|GO:0006351,transcription, DNA-templated|GO:0019438,aromatic compound biosynthetic process	GO:0043231,intracellular membrane-bounded organelle|GO:0005634,nucleus|GO:0044464,cell part|GO:0005623,cell|GO:0005622,intracellular|GO:0043229,intracellular organelle|GO:0044424,intracellular part|GO:0043227,membrane-bounded organelle|GO:0043226,organelle	GO:0043169,cation binding|GO:0003700,transcription factor activity, sequence-specific DNA binding|GO:0097159,organic cyclic compound binding|GO:0043167,ion binding|GO:0005488,binding|GO:0003676,nucleic acid binding|GO:0003677,DNA binding|GO:0046872,metal ion binding|GO:1901363,heterocyclic compound binding|GO:0001071,nucleic acid binding transcription factor activity|GO:0000981,RNA polymerase II transcription factor activity, sequence-specific DNA binding
GGCT	GO:0042398,cellular modified amino acid biosynthetic process|GO:0044237,cellular metabolic process|GO:0097190,apoptotic signaling pathway|GO:1901564,organonitrogen compound metabolic process|GO:0006805,xenobiotic metabolic process|GO:0034641,cellular nitrogen compound metabolic process|GO:0006807,nitrogen compound metabolic process|GO:0023052,signaling|GO:0007165,signal transduction|GO:0070887,cellular response to chemical stimulus|GO:0042221,response to chemical|GO:0008219,cell death|GO:0050789,regulation of biological process|GO:0007005,mitochondrion organization|GO:1901576,organic substance biosynthetic process|GO:0044710,single-organism metabolic process|GO:0044711,single-organism biosynthetic process|GO:1901685,glutathione derivative metabolic process|GO:1901687,glutathione derivative biosynthetic process|GO:0071840,cellular component organization or biogenesis|GO:0043043,peptide biosynthetic process|GO:0016043,cellular component organization|GO:0071704,organic substance metabolic process|GO:0065007,biological regulation|GO:0044699,single-organism process|GO:0006749,glutathione metabolic process|GO:1901566,organonitrogen compound biosynthetic process|GO:0019184,nonribosomal peptide biosynthetic process|GO:0016265,obsolete death|GO:0009987,cellular process|GO:0043604,amide biosynthetic process|GO:0006915,apoptotic process|GO:0051716,cellular response to stimulus|GO:0050794,regulation of cellular process|GO:0012501,programmed cell death|GO:0009058,biosynthetic process|GO:0044763,single-organism cellular process|GO:0008152,metabolic process|GO:0007154,cell communication|GO:0006996,organelle organization|GO:0044700,single organism signaling|GO:0008637,apoptotic mitochondrial changes|GO:0044271,cellular nitrogen compound biosynthetic process|GO:0006575,cellular modified amino acid metabolic process|GO:0043603,cellular amide metabolic process|GO:0044272,sulfur compound biosynthetic process|GO:0050896,response to stimulus|GO:0009410,response to xenobiotic stimulus|GO:0001836,release of cytochrome c from mitochondria|GO:0071466,cellular response to xenobiotic stimulus|GO:0006790,sulfur compound metabolic process|GO:0006750,glutathione biosynthetic process|GO:0006518,peptide metabolic process|GO:0044249,cellular biosynthetic process|GO:0044281,small	GO:0005737,cytoplasm|GO:0005622,intracellular|GO:0043230,extracellular organelle|GO:0031982,vesicle|GO:0044421,extracellular region part|GO:0043227,membrane-bounded organelle|GO:0005829,cytosol|GO:0044464,cell part|GO:0005623,cell|GO:0031988,membrane-bounded vesicle|GO:0044444,cytoplasmic part|GO:0005576,extracellular region|GO:0044424,intracellular part|GO:1903561,extracellular vesicle|GO:0065010,extracellular membrane-bounded organelle|GO:0043226,organelle|GO:0070062,extracellular exosome	GO:0003824,catalytic activity|GO:0016740,transferase activity|GO:0016746,transferase activity, transferring acyl groups|GO:0005488,binding|GO:0005515,protein binding|GO:0016755,transferase activity, transferring amino-acyl groups|GO:0046983,protein dimerization activity|GO:0003839,gamma-glutamylcyclotransferase activity|GO:0042802,identical protein binding|GO:0042803,protein homodimerization activity
TSEN34	GO:0090304,nucleic acid metabolic process|GO:0090305,nucleic acid phosphodiester bond hydrolysis|GO:0034641,cellular nitrogen compound metabolic process|GO:0006807,nitrogen compound metabolic process|GO:0034660,ncRNA metabolic process|GO:1901360,organic cyclic compound metabolic process|GO:0034470,ncRNA processing|GO:0006139,nucleobase-containing compound metabolic process|GO:0044260,cellular macromolecule metabolic process|GO:0071704,organic substance metabolic process|GO:0010467,gene expression|GO:0006388,tRNA splicing, via endonucleolytic cleavage and ligation|GO:0008380,RNA splicing|GO:0016070,RNA metabolic process|GO:0008033,tRNA processing|GO:0044238,primary metabolic process|GO:0009987,cellular process|GO:0006725,cellular aromatic compound metabolic process|GO:0008152,metabolic process|GO:0000379,tRNA-type intron splice site recognition and cleavage|GO:0046483,heterocycle metabolic process|GO:0090502,RNA phosphodiester bond hydrolysis, endonucleolytic|GO:0016071,mRNA metabolic process|GO:0000394,RNA splicing, via endonucleolytic cleavage and ligation|GO:0090501,RNA phosphodiester bond hydrolysis|GO:0044237,cellular metabolic process|GO:0043170,macromolecule metabolic process|GO:0006399,tRNA metabolic process|GO:0006396,RNA processing|GO:0006397,mRNA processing	GO:0031974,membrane-enclosed lumen|GO:0043229,intracellular organelle|GO:0043228,non-membrane-bounded organelle|GO:0043227,membrane-bounded organelle|GO:0043226,organelle|GO:0031981,nuclear lumen|GO:0005730,nucleolus|GO:0005634,nucleus|GO:0005654,nucleoplasm|GO:1902494,catalytic complex|GO:1902555,endoribonuclease complex|GO:0000214,tRNA-intron endonuclease complex|GO:0043234,protein complex|GO:0032991,macromolecular complex|GO:0043231,intracellular membrane-bounded organelle|GO:0043232,intracellular non-membrane-bounded organelle|GO:0043233,organelle lumen|GO:0044464,cell part|GO:0005623,cell|GO:0005622,intracellular|GO:0044446,intracellular organelle part|GO:0070013,intracellular organelle lumen|GO:0044428,nuclear part|GO:0044424,intracellular	GO:0016787,hydrolase activity|GO:0004549,tRNA-specific ribonuclease activity|GO:0003676,nucleic acid binding|GO:0003824,catalytic activity|GO:0000213,tRNA-intron endonuclease activity|GO:0004519,endonuclease activity|GO:0016829,lyase activity|GO:0004540,ribonuclease activity|GO:0004521,endoribonuclease activity|GO:0016788,hydrolase activity, acting on ester bonds|GO:0016892,endoribonuclease activity, producing 3'-phosphomonoesters|GO:0004518,nuclease activity|GO:0005488,binding|GO:0016894,endonuclease activity, active with either ribo- or deoxyribonucleic acids and producing 3'-phosphomonoesters|GO:0097159,organic cyclic compound binding|GO:1901363,heterocyclic compound binding
AC137056	GO:0006631,fatty acid metabolic process|GO:0006637,acyl-CoA metabolic process|GO:0006732,coenzyme metabolic process|GO:0019752,carboxylic acid metabolic process|GO:0042592,homeostatic process|GO:0042593,glucose homeostasis|GO:0044281,small molecule metabolic process|GO:0035337,fatty-acyl-CoA metabolic process|GO:0044699,single-organism process|GO:0055090,acylglycerol homeostasis|GO:0044710,single-organism metabolic process|GO:0051186,cofactor metabolic process|GO:0033500,carbohydrate homeostasis|GO:0071704,organic substance metabolic process|GO:0065007,biological regulation|GO:0065008,regulation of biological quality|GO:0035383,thioester metabolic process|GO:0070328,triglyceride homeostasis|GO:0006629,lipid metabolic process|GO:0009987,cellular process|GO:0032787,monocarboxylic acid metabolic process|GO:0055088,lipid homeostasis|GO:0044763,single-organism cellular process|GO:0008152,metabolic process|GO:0043436,oxoacid metabolic process|GO:0044255,cellular lipid metabolic process|GO:0044238,primary metabolic process|GO:0006082,organic acid metabolic process|GO:0036112,medium-chain fatty-acyl-CoA metabolic process|GO:0044237,cellular metabolic process|GO:0048878,chemical homeostasis|GO:0006790,sulfur compound metabolic process|GO:0006793,phosphorus metabolic process|GO:0006633,fatty acid biosynthetic process|GO:0044283,small molecule biosynthetic process|GO:0072330,monocarboxylic acid biosynthetic process|GO:1901576,organic substance biosynthetic process|GO:0044711,single-organism biosynthetic process|GO:0009058,biosynthetic process|GO:0008610,lipid biosynthetic process|GO:0044249,cellular biosynthetic process|GO:0046394,carboxylic acid biosynthetic process|GO:0016053,organic acid biosynthetic process|GO:1901568,fatty acid derivative metabolic process	GO:0005737,cytoplasm|GO:0043231,intracellular membrane-bounded organelle|GO:0043233,organelle lumen|GO:0044464,cell part|GO:0044444,cytoplasmic part|GO:0031974,membrane-enclosed lumen|GO:0005622,intracellular|GO:0044446,intracellular organelle part|GO:0070013,intracellular organelle lumen|GO:0043229,intracellular organelle|GO:0005739,mitochondrion|GO:0044429,mitochondrial part|GO:0005759,mitochondrial matrix|GO:0044424,intracellular part|GO:0005623,cell|GO:0043227,membrane-bounded organelle|GO:0043226,organelle|GO:0044422,organelle part	GO:0035639,purine ribonucleoside triphosphate binding|GO:0005488,binding|GO:0043167,ion binding|GO:1901363,heterocyclic compound binding|GO:1901265,nucleoside phosphate binding|GO:0001882,nucleoside binding|GO:0043168,anion binding|GO:0043169,cation binding|GO:0000166,nucleotide binding|GO:0001883,purine nucleoside binding|GO:0032549,ribonucleoside binding|GO:0017076,purine nucleotide binding|GO:0046872,metal ion binding|GO:0005524,ATP binding|GO:0016878,acid-thiol ligase activity|GO:0003824,catalytic activity|GO:0047760,butyrate-CoA ligase activity|GO:0016874,ligase activity|GO:0016877,ligase activity, forming carbon-sulfur bonds|GO:0036094,small molecule binding|GO:0030554,adenyl nucleotide binding|GO:0097367,carbohydrate derivative binding|GO:0097159,organic cyclic compound binding|GO:0032559,adenyl ribonucleotide binding|GO:0032555,purine ribonucleotide binding|GO:0032550,purine ribonucleoside binding|GO:0032553,ribonucleotide binding|GO:0015645,fatty acid ligase activity|GO:0004321,fatty-acyl-CoA synthase activity|GO:0016740,transferase activity|GO:0016746,transferase activity, transferring acyl groups|GO:0016747,transferase activity, transferring acyl groups other than amino-acyl groups|GO:0016405,CoA-ligase activity|GO:0003996,acyl-CoA ligase activity|GO:0016408,C-acyltransferase
CTB	GO:0009311,oligosaccharide metabolic process|GO:0009313,oligosaccharide catabolic process|GO:0046348,amino sugar catabolic process|GO:0006807,nitrogen compound metabolic process|GO:0044699,single-organism process|GO:0044712,single-organism catabolic process|GO:0044710,single-organism metabolic process|GO:0071704,organic substance metabolic process|GO:0006030,chitin metabolic process|GO:0006032,chitin catabolic process|GO:1901575,organic substance catabolic process|GO:1901072,glucosamine-containing compound catabolic process|GO:0008152,metabolic process|GO:0044723,single-organism carbohydrate metabolic process|GO:1901564,organonitrogen compound metabolic process|GO:0009056,catabolic process|GO:0009057,macromolecule catabolic process|GO:0044724,single-organism carbohydrate catabolic process|GO:0044238,primary metabolic process|GO:0005975,carbohydrate metabolic process|GO:1901565,organonitrogen compound catabolic process|GO:0006040,amino sugar metabolic process|GO:1901136,carbohydrate derivative catabolic process|GO:1901135,carbohydrate derivative metabolic process|GO:0016052,carbohydrate catabolic process|GO:0043170,macromolecule metabolic process|GO:0006022,aminoglycan metabolic process|GO:1901071,glucosamine-containing compound metabolic process|GO:0006026,aminoglycan catabolic process	GO:0000323,lytic vacuole|GO:0043229,intracellular organelle|GO:0043227,membrane-bounded organelle|GO:0043226,organelle|GO:0005737,cytoplasm|GO:0031982,vesicle|GO:0005773,vacuole|GO:0031988,membrane-bounded vesicle|GO:1903561,extracellular vesicle|GO:0005615,extracellular space|GO:0043230,extracellular organelle|GO:0043231,intracellular membrane-bounded organelle|GO:0070062,extracellular exosome|GO:0044464,cell part|GO:0005623,cell|GO:0005622,intracellular|GO:0005764,lysosome|GO:0044444,cytoplasmic part|GO:0005576,extracellular region|GO:0044424,intracellular part|GO:0065010,extracellular membrane-bounded organelle|GO:0044421,extracellular region part	GO:0016787,hydrolase activity|GO:0097367,carbohydrate derivative binding|GO:0016798,hydrolase activity, acting on glycosyl bonds|GO:0003824,catalytic activity|GO:0008061,chitin binding|GO:0004553,hydrolase activity, hydrolyzing O-glycosyl compounds|GO:0004568,chitinase activity|GO:0005488,binding
APOBEC3H	GO:0080090,regulation of primary metabolic process|GO:0019222,regulation of metabolic process|GO:0048583,regulation of response to stimulus|GO:0009451,RNA modification|GO:0009164,nucleoside catabolic process|GO:0044281,small molecule metabolic process|GO:0046135,pyrimidine nucleoside catabolic process|GO:0046131,pyrimidine ribonucleoside metabolic process|GO:0031347,regulation of defense response|GO:0050688,regulation of defense response to virus|GO:0044712,single-organism catabolic process|GO:0044710,single-organism metabolic process|GO:0006304,DNA modification|GO:0010605,negative regulation of macromolecule metabolic process|GO:0043207,response to external biotic stimulus|GO:0071704,organic substance metabolic process|GO:0009615,response to virus|GO:0040029,regulation of gene expression, epigenetic|GO:0044419,interspecies interaction between organisms|GO:0019058,viral life cycle|GO:0010528,regulation of transposition|GO:0010529,negative regulation of transposition|GO:0002682,regulation of immune system process|GO:0046483,heterocycle metabolic process|GO:0051053,negative regulation of DNA metabolic process|GO:0051052,regulation of DNA metabolic process|GO:0060255,regulation of macromolecule metabolic process|GO:0009607,response to biotic stimulus|GO:0051707,response to other organism|GO:0042454,ribonucleoside catabolic process|GO:0070988,demethylation|GO:1901564,organonitrogen compound metabolic process|GO:1901565,organonitrogen compound catabolic process|GO:0009605,response to external stimulus|GO:0039694,viral RNA genome replication|GO:0039692,single stranded viral RNA replication via double stranded DNA intermediate|GO:0009972,cytidine deamination|GO:0002376,immune system process|GO:0019439,aromatic compound catabolic process|GO:0051253,negative regulation of RNA metabolic process|GO:0009892,negative regulation of metabolic process|GO:0006950,response to stress|GO:0002831,regulation of response to biotic stimulus|GO:0006807,nitrogen compound metabolic process|GO:0044033,multi-organism metabolic process|GO:0050789,regulation of biological process|GO:0046087,cytidine metabolic process|GO:1901575,organic substance catabolic process|GO:0044260,cellular macromolecule metabolic process|GO:0065007,biological regulation|GO:1901360,organic cyclic compound metabolic process|GO:0046133,pyrimidine ribonucleoside catabolic process|GO:0050792,regulation of viral process|GO:1901135,carbohydrate derivative metabolic process|GO:0050794,regulation of cellular process|GO:0006952,defense response|GO:0043903,regulation of symbiosis, encompassing mutualism through parasitism|GO:0043900,regulation of multi-organism process|GO:0044728,DNA methylation or demethylation|GO:0008152,metabolic process|GO:0006955,immune response|GO:0034655,nucleobase-containing compound catabolic process|GO:0046700,heterocycle catabolic process|GO:0016070,RNA metabolic process|GO:0044270,cellular nitrogen compound catabolic process|GO:0039703,RNA replication|GO:0006216,cytidine catabolic process|GO:0043412,macromolecule modification|GO:0006213,pyrimidine nucleoside metabolic process|GO:0002697,regulation of immune effector process|GO:0043901,negative regulation of multi-organism process|GO:0009116,nucleoside metabolic process|GO:0032101,regulation of response to external stimulus|GO:0050896,response to stimulus|GO:0044248,cellular catabolic process|GO:0034641,cellular nitrogen compound metabolic process|GO:0044699,single-organism process|GO:0006139,nucleobase-containing compound metabolic process|GO:1903900,regulation of viral life cycle|GO:1903901,negative regulation of viral life cycle|GO:0016553,base conversion or substitution editing|GO:0045071,negative regulation of viral genome replication|GO:0019079,viral genome replication|GO:0009987,cellular process|GO:0006725,cellular aromatic compound metabolic process|GO:0098542,defense response to other organism|GO:0048519,negative regulation of biological process|GO:0055086,nucleobase-containing small molecule metabolic process|GO:0051607,defense response to virus|GO:0080111,DNA demethylation|GO:1901136,carbohydrate derivative catabolic process|GO:0045069,regulation of viral genome replication|GO:0051252,regulation of RNA metabolic process|GO:0043170,macromolecule metabolic process|GO:0045869,negative regulation of single stranded viral RNA replication via double stranded DNA intermediate|GO:0002230,positive regulation of defense response to virus by host|GO:0080134,regulation of response to stress|GO:0035510,DNA dealkylation|GO:0031324,negative regulation of cellular metabolic process|GO:0031323,regulation of cellular metabolic process|GO:0045091,regulation of single stranded viral RNA replication via double stranded DNA intermediate|GO:0090304,nucleic acid metabolic process|GO:0072529,pyrimidine-containing compound catabolic process|GO:0072527,pyrimidine-containing compound metabolic process|GO:1901361,organic cyclic compound catabolic process|GO:0032196,transposition|GO:0070383,DNA cytosine deamination|GO:0010467,gene expression|GO:0051704,multi-organism process|GO:0010468,regulation of gene expression|GO:0045934,negative regulation of nucleobase-containing compound metabolic process|GO:0019219,regulation of nucleobase-containing compound metabolic process|GO:0045087,innate immune response|GO:0050691,regulation of defense response to virus by host|GO:0048525,negative regulation of viral process|GO:0044764,multi-organism cellular process|GO:0044763,single-organism cellular process|GO:0051171,regulation of nitrogen compound metabolic process|GO:0051172,negative regulation of nitrogen compound metabolic process|GO:0009119,ribonucleoside metabolic process|GO:0009056,catabolic process|GO:0044238,primary metabolic process|GO:0045006,DNA deamination|GO:0044237,cellular metabolic process|GO:1901657,glycosyl compound metabolic process|GO:0048523,negative regulation of cellular process|GO:0016032,viral process|GO:0002252,immune effector process|GO:0006259,DNA metabolic process|GO:0044403,symbiosis, encompassing mutualism through parasitism|GO:1901658,glycosyl compound catabolic process	GO:0035770,ribonucleoprotein granule|GO:0000932,P-body|GO:0043229,intracellular organelle|GO:0043228,non-membrane-bounded organelle|GO:0036464,cytoplasmic ribonucleoprotein granule|GO:0043227,membrane-bounded organelle|GO:0043226,organelle|GO:0005737,cytoplasm|GO:0005634,nucleus|GO:0030529,intracellular ribonucleoprotein complex|GO:0030895,apolipoprotein B mRNA editing enzyme complex|GO:0043234,protein complex|GO:0032991,macromolecular complex|GO:0043231,intracellular membrane-bounded organelle|GO:0043232,intracellular non-membrane-bounded organelle|GO:0044464,cell part|GO:0005623,cell|GO:0005622,intracellular|GO:0044446,intracellular organelle part|GO:0044444,cytoplasmic part|GO:0044428,nuclear part|GO:0044424,intracellular part|GO:0044422,organelle part|GO:1990904,ribonucleoprotein complex	GO:0005488,binding|GO:0016787,hydrolase activity|GO:0005515,protein binding|GO:0046914,transition metal ion binding|GO:0043169,cation binding|GO:0097159,organic cyclic compound binding|GO:0016810,hydrolase activity, acting on carbon-nitrogen (but not peptide) bonds|GO:0043167,ion binding|GO:1901363,heterocyclic compound binding|GO:0016814,hydrolase activity, acting on carbon-nitrogen (but not peptide) bonds, in cyclic amidines|GO:0004126,cytidine deaminase activity|GO:0003824,catalytic activity|GO:0019239,deaminase activity|GO:0003723,RNA binding|GO:0003676,nucleic acid binding|GO:0046872,metal ion binding|GO:0044822,poly(A) RNA binding|GO:0008270,zinc ion binding|GO:0042802,identical protein binding
GPR141	GO:0044700,single organism signaling|GO:0051716,cellular response to stimulus|GO:0009987,cellular process|GO:0050896,response to stimulus|GO:0065007,biological regulation|GO:0044763,single-organism cellular process|GO:0050794,regulation of cellular process|GO:0050789,regulation of biological process|GO:0007188,adenylate cyclase-modulating G-protein coupled receptor signaling pathway|GO:0007189,adenylate cyclase-activating G-protein coupled receptor signaling pathway|GO:0023052,signaling|GO:0007165,signal transduction|GO:0007154,cell communication|GO:0007186,G-protein coupled receptor signaling pathway|GO:0007187,G-protein coupled receptor signaling pathway, coupled to cyclic nucleotide second messenger|GO:0044699,single-organism process	GO:0031226,intrinsic component of plasma membrane|GO:0016021,integral component of membrane|GO:0016020,membrane|GO:0044464,cell part|GO:0005623,cell|GO:0071944,cell periphery|GO:0005887,integral component of plasma membrane|GO:0005886,plasma membrane|GO:0044425,membrane part|GO:0044459,plasma membrane part|GO:0031224,intrinsic component of membrane	GO:0004930,G-protein coupled receptor activity|GO:0038023,signaling receptor activity|GO:0060089,molecular transducer activity|GO:0004888,transmembrane signaling receptor activity|GO:0004872,receptor activity|GO:0004871,signal transducer activity|GO:0099600,transmembrane receptor activity
SAA1	GO:0040011,locomotion|GO:0042221,response to chemical|GO:0006935,chemotaxis|GO:0042330,taxis|GO:0050896,response to stimulus|GO:0006952,defense response|GO:0006953,acute-phase response|GO:0006950,response to stress|GO:0006954,inflammatory response|GO:0002526,acute inflammatory response|GO:0050918,positive chemotaxis|GO:0009605,response to external stimulus	GO:0032994,protein-lipid complex|GO:0032991,macromolecular complex|GO:0005615,extracellular space|GO:0005576,extracellular region|GO:1990777,lipoprotein particle|GO:0034364,high-density lipoprotein particle|GO:0044421,extracellular region part|GO:0034358,plasma lipoprotein particle	GO:0042056,chemoattractant activity
C7orf73			
FOLH1	GO:0006732,coenzyme metabolic process|GO:0019752,carboxylic acid metabolic process|GO:0034641,cellular nitrogen compound metabolic process|GO:0006807,nitrogen compound metabolic process|GO:0044281,small molecule metabolic process|GO:0044699,single-organism process|GO:0044710,single-organism metabolic process|GO:0051186,cofactor metabolic process|GO:1901360,organic cyclic compound metabolic process|GO:0006520,cellular amino acid metabolic process|GO:0042558,pteridine-containing compound metabolic process|GO:0071704,organic substance metabolic process|GO:0006082,organic acid metabolic process|GO:0006508,proteolysis|GO:1901605,alpha-amino acid metabolic process|GO:0006760,folic acid-containing compound metabolic process|GO:0009987,cellular process|GO:0006725,cellular aromatic compound metabolic process|GO:0044763,single-organism cellular process|GO:0008152,metabolic process|GO:0043436,oxoacid metabolic process|GO:0046483,heterocycle metabolic process|GO:0044238,primary metabolic process|GO:1901564,organonitrogen compound metabolic process|GO:0006575,cellular modified amino acid metabolic process|GO:0043603,cellular amide metabolic process|GO:0019538,protein metabolic process|GO:0044237,cellular metabolic process|GO:0043170,macromolecule metabolic process|GO:0018410,C-terminal protein amino acid modification|GO:0018200,peptidyl-glutamic acid modification|GO:0044249,cellular biosynthetic process|GO:0044283,small molecule biosynthetic process|GO:1901576,organic substance biosynthetic process|GO:1901566,organonitrogen compound biosynthetic process|GO:0044267,cellular protein metabolic process|GO:0044711,single-organism biosynthetic process|GO:0044260,cellular macromolecule metabolic process|GO:0018193,peptidyl-amino acid modification|GO:0043687,post-translational protein modification|GO:0035608,protein deglutamylation|GO:0035609,C-terminal protein deglutamylation|GO:0006464,cellular protein modification process|GO:0009058,biosynthetic process|GO:0043412,macromolecule modification|GO:0008652,cellular amino acid biosynthetic process|GO:0046394,carboxylic acid biosynthetic process|GO:0016053,organic acid biosynthetic process|GO:0036211,protein modification process	GO:0071944,cell periphery|GO:0031226,intrinsic component of plasma membrane|GO:0043226,organelle|GO:0031224,intrinsic component of membrane|GO:0005737,cytoplasm|GO:0031982,vesicle|GO:0043227,membrane-bounded organelle|GO:0016021,integral component of membrane|GO:0016020,membrane|GO:0031988,membrane-bounded vesicle|GO:0044459,plasma membrane part|GO:0005887,integral component of plasma membrane|GO:0005886,plasma membrane|GO:1903561,extracellular vesicle|GO:0070062,extracellular exosome|GO:0043230,extracellular organelle|GO:0044464,cell part|GO:0005623,cell|GO:0005622,intracellular|GO:0005576,extracellular region|GO:0044424,intracellular part|GO:0044425,membrane part|GO:0065010,extracellular membrane-bounded organelle|GO:0044421,extracellular	GO:0016787,hydrolase activity|GO:0003824,catalytic activity|GO:0004180,carboxypeptidase activity|GO:0016805,dipeptidase activity|GO:0043169,cation binding|GO:0043167,ion binding|GO:0008238,exopeptidase activity|GO:0070011,peptidase activity, acting on L-amino acid peptides|GO:0005488,binding|GO:0008233,peptidase activity|GO:0046872,metal ion binding|GO:0008237,metallopeptidase activity|GO:0043168,anion binding|GO:1900750,oligopeptide binding|GO:0042277,peptide binding|GO:1904493,tetrahydrofolyl-poly(glutamate) polymer binding|GO:0004181,metallocarboxypeptidase activity|GO:1904492,Ac-Asp-Glu binding|GO:0033218,amide binding|GO:0008235,metalloexopeptidase activity

TMED7	GO:0033036,macromolecule localization|GO:0008104,protein localization|GO:0006810,transport|GO:0045184,establishment of protein localization|GO:0071702,organic substance transport|GO:0015031,protein transport|GO:0051234,establishment of localization|GO:0051179,localization|GO:0042886,amide transport|GO:0006890,retrograde vesicle-mediated transport, Golgi to ER|GO:0006888,ER to Golgi vesicle-mediated transport|GO:0016192,vesicle-mediated transport|GO:0046907,intracellular transport|GO:0048193,Golgi vesicle transport|GO:0071705,nitrogen compound transport|GO:0015833,peptide transport|GO:0051649,establishment of localization in cell|GO:0051641,cellular localization	GO:0005783,endoplasmic reticulum|GO:0044421,extracellular region part|GO:0033116,endoplasmic reticulum-Golgi intermediate compartment membrane|GO:0005789,endoplasmic reticulum membrane|GO:0043226,organelle|GO:0042175,nuclear outer membrane-endoplasmic reticulum membrane network|GO:0043229,intracellular organelle|GO:0030117,membrane coat|GO:0030133,transport vesicle|GO:0005622,intracellular|GO:0030135,coated vesicle|GO:0030134,ER to Golgi transport vesicle|GO:0030137,COPI-coated vesicle|GO:0005737,cytoplasm|GO:0070062,extracellular exosome|GO:0030663,COPI-coated vesicle membrane|GO:0030662,coated vesicle membrane|GO:0016023,cytoplasmic, membrane-bounded vesicle|GO:0031410,cytoplasmic vesicle|GO:0016021,integral component of membrane|GO:0016020,membrane|GO:0044444,cytoplasmic part|GO:0031988,membrane-bounded vesicle|GO:0044433,cytoplasmic vesicle part|GO:0044432,endoplasmic reticulum part|GO:0044431,Golgi apparatus part|GO:0031224,intrinsic component of membrane|GO:0031982,vesicle|GO:0048475,coated membrane|GO:0005794,Golgi apparatus|GO:0005793,endoplasmic reticulum-Golgi intermediate compartment|GO:0098588,bounding membrane of organelle|GO:0005798,Golgi-associated vesicle|GO:0030658,transport vesicle membrane|GO:0030659,cytoplasmic vesicle membrane|GO:0012505,endomembrane system|GO:0012506,vesicle membrane|GO:0012507,ER to Golgi transport vesicle membrane|GO:0032991,macromolecular complex|GO:0030120,vesicle coat|GO:1903561,extracellular vesicle|GO:0030126,COPI vesicle coat|GO:0030127,COPII vesicle coat|GO:0030660,Golgi-associated vesicle membrane|GO:0043234,protein complex|GO:0043230,extracellular organelle|GO:0043231,intracellular membrane-bounded organelle|GO:0044464,cell part|GO:0005623,cell|GO:0000139,Golgi membrane|GO:0044446,intracellular organelle part|GO:0098796,membrane protein complex|GO:0043227,membrane-bounded organelle|GO:0005576,extracellular region|GO:0031090,organelle membrane|GO:0044424,intracellular part|GO:0044425,membrane part|GO:0065010,extracellular membrane-bounded organelle|GO:0098805,whole membrane|GO:0044422,organelle part|GO:0031984,organelle subcompartment|GO:0098827,endoplasmic reticulum subcompartment|GO:0097708,intracellular vesicle|GO:0098791,Golgi subcompartment	
SRSF6	GO:0051169,nuclear transport|GO:0051168,nuclear export|GO:0080090,regulation of primary metabolic process|GO:0019222,regulation of metabolic process|GO:0050684,regulation of mRNA processing|GO:0050686,negative regulation of mRNA processing|GO:0048583,regulation of response to stimulus|GO:0071166,ribonucleoprotein complex localization|GO:1901362,organic cyclic compound biosynthetic process|GO:0071840,cellular component organization or biogenesis|GO:0000245,spliceosomal complex assembly|GO:0010605,negative regulation of macromolecule metabolic process|GO:0071427,mRNA-containing ribonucleoprotein complex export from nucleus|GO:0048869,cellular developmental process|GO:0009611,response to wounding|GO:0048513,animal organ development|GO:0030856,regulation of epithelial cell differentiation|GO:0030857,negative regulation of epithelial cell differentiation|GO:0030855,epithelial cell differentiation|GO:0048518,positive regulation of biological process|GO:0048519,negative regulation of biological process|GO:0033036,macromolecule localization|GO:0042127,regulation of cell proliferation|GO:0030216,keratinocyte differentiation|GO:0060255,regulation of macromolecule metabolic process|GO:0060548,negative regulation of cell death|GO:0071702,organic substance transport|GO:0046483,heterocycle metabolic process|GO:0044707,single-multicellular organism process|GO:0000398,mRNA splicing, via spliceosome|GO:0006405,RNA export from nucleus|GO:0006376,mRNA splice site selection|GO:0006406,mRNA export from nucleus|GO:0019438,aromatic compound biosynthetic process|GO:0022607,cellular component assembly|GO:0009892,negative regulation of metabolic process|GO:0022603,regulation of anatomical structure morphogenesis|GO:0008152,metabolic process|GO:0010629,negative regulation of gene expression|GO:0006807,nitrogen compound metabolic process|GO:2000794,regulation of epithelial cell proliferation involved in lung morphogenesis|GO:0051028,mRNA transport|GO:0043170,macromolecule metabolic process|GO:0050789,regulation of biological process|GO:0097659,nucleic acid-templated transcription|GO:1901576,organic substance biosynthetic process|GO:0009653,anatomical structure morphogenesis|GO:0000381,regulation of alternative mRNA splicing, via spliceosome|GO:0000380,alternative mRNA splicing, via spliceosome|GO:0044260,cellular macromolecule metabolic process|GO:0016043,cellular component organization|GO:0065003,macromolecular complex assembly|GO:0065007,biological regulation|GO:1901360,organic cyclic compound metabolic process|GO:0006366,transcription from RNA polymerase II promoter|GO:0006369,termination of RNA polymerase II transcription|GO:0018130,heterocycle biosynthetic process|GO:0016265,obsolete death|GO:0009887,animal organ morphogenesis|GO:0050793,regulation of developmental process|GO:0006810,transport|GO:0009888,tissue development|GO:0042060,wound healing|GO:0050794,regulation of cellular process|GO:0006950,response to stress|GO:0051239,regulation of multicellular organismal process|GO:0034654,nucleobase-containing compound biosynthetic process|GO:0043484,regulation of RNA splicing|GO:0051234,establishment of localization|GO:0016070,RNA metabolic process|GO:0016071,mRNA metabolic process|GO:0050658,RNA transport|GO:0044271,cellular nitrogen compound biosynthetic process|GO:0046907,intracellular transport|GO:0080134,regulation of response to stress|GO:0009058,biosynthetic process|GO:0032502,developmental process|GO:0009059,macromolecule biosynthetic process|GO:0006351,transcription, DNA-templated|GO:0006353,DNA-templated transcription, termination|GO:1903311,regulation of mRNA metabolic process|GO:1903312,negative regulation of mRNA metabolic process|GO:0015931,nucleobase-containing compound transport|GO:0033119,negative regulation of RNA splicing|GO:0032774,RNA biosynthetic process|GO:0030154,cell differentiation|GO:0044249,cellular biosynthetic process|GO:0034641,cellular nitrogen compound metabolic process|GO:0034645,cellular macromolecule biosynthetic process|GO:0051236,establishment of RNA localization|GO:0048024,regulation of mRNA splicing, via spliceosome|GO:0048025,negative regulation of mRNA splicing, via spliceosome|GO:0008544,epidermis development|GO:0044699,single-organism process|GO:0043616,keratinocyte proliferation|GO:0006139,nucleobase-containing compound metabolic process|GO:0051240,positive regulation of multicellular organismal process|GO:0051241,negative regulation of multicellular organismal process|GO:0022618,ribonucleoprotein complex assembly|GO:0022613,ribonucleoprotein complex biogenesis|GO:1903034,regulation of response to wounding|GO:0008380,RNA splicing|GO:0008284,positive regulation of cell proliferation|GO:0032501,multicellular organismal process|GO:0060425,lung morphogenesis|GO:0060428,lung epithelium development|GO:0060429,epithelium development|GO:0006725,cellular aromatic compound metabolic process|GO:0010837,regulation of keratinocyte proliferation|GO:0045595,regulation of cell differentiation|GO:0016482,cytosolic transport|GO:0051093,negative regulation of developmental process|GO:0050678,regulation of epithelial cell proliferation|GO:0050679,positive regulation of epithelial cell proliferation|GO:0051094,positive regulation of developmental process|GO:0051253,negative regulation of RNA metabolic process|GO:0051252,regulation of RNA metabolic process|GO:0050673,epithelial cell proliferation|GO:0048731,system development|GO:0045616,regulation of keratinocyte differentiation|GO:0045617,negative regulation of keratinocyte differentiation|GO:0050896,response to stimulus|GO:0060541,respiratory system development|GO:0030323,respiratory tube development|GO:0030324,lung development|GO:0043933,macromolecular complex subunit organization|GO:0031324,negative regulation of cellular metabolic process|GO:0031323,regulation of cellular metabolic process|GO:0050657,nucleic acid transport|GO:0090304,nucleic acid metabolic process|GO:0061041,regulation of wound healing|GO:0034622,cellular macromolecular complex assembly|GO:0045683,negative regulation of epidermis development|GO:0045682,regulation of epidermis development|GO:0008219,cell death|GO:0010941,regulation of cell death|GO:0007275,multicellular organism development|GO:0071826,ribonucleoprotein complex subunit organization|GO:0006396,RNA processing|GO:0008283,cell proliferation|GO:0071705,nitrogen compound transport|GO:0071704,organic substance metabolic process|GO:0010467,gene expression|GO:0045605,negative regulation of epidermal cell differentiation|GO:0045604,regulation of epidermal cell differentiation|GO:0006403,RNA localization|GO:0060501,positive regulation of epithelial cell proliferation involved in lung morphogenesis|GO:0010468,regulation of gene expression|GO:0043588,skin development|GO:0060502,epithelial cell proliferation involved in lung morphogenesis|GO:0009987,cellular process|GO:0045934,negative regulation of nucleobase-containing compound metabolic process|GO:0019219,regulation of nucleobase-containing compound metabolic process|GO:0031124,mRNA 3'-end processing|GO:0006913,nucleocytoplasmic transport|GO:0031123,RNA 3'-end processing|GO:0044767,single-organism developmental process|GO:0000375,RNA splicing, via transesterification reactions|GO:0044765,single-organism transport|GO:0000377,RNA splicing, via transesterification reactions with bulged adenosine as nucleophile|GO:0044763,single-organism cellular process|GO:0051171,regulation of nitrogen compound metabolic process|GO:0051172,negative regulation of nitrogen compound metabolic process|GO:0051649,establishment of localization in cell|GO:0035295,tube development|GO:0051179,localization|GO:1902578,single-organism localization|GO:0051641,cellular localization|GO:0044238,primary metabolic process|GO:0071426,ribonucleoprotein complex export from nucleus|GO:0045596,negative regulation of cell differentiation|GO:0009913,epidermal cell differentiation|GO:0048856,anatomical structure development|GO:0044237,cellular metabolic process|GO:0044085,cellular component biogenesis|GO:2000026,regulation of multicellular organismal development|GO:2000027,regulation of organ morphogenesis|GO:1902582,single-organism intracellular transport|GO:0006397,mRNA processing|GO:0048523,negative regulation of cellular process|GO:0048522,positive regulation of cellular process|GO:0043434,response to peptide hormone|GO:0010033,response to organic substance|GO:0010243,response to organonitrogen compound|GO:0032868,response to insulin|GO:0042981,regulation of apoptotic process|GO:1904036,negative regulation of	GO:0016607,nuclear speck|GO:0044446,intracellular organelle part|GO:0016604,nuclear body|GO:0043231,intracellular membrane-bounded organelle|GO:0031981,nuclear lumen|GO:0043233,organelle lumen|GO:0005634,nucleus|GO:0044464,cell part|GO:0031974,membrane-enclosed lumen|GO:0005622,intracellular|GO:0005654,nucleoplasm|GO:0070013,intracellular organelle lumen|GO:0043229,intracellular organelle|GO:0044451,nucleoplasm part|GO:0005623,cell|GO:0044424,intracellular part|GO:0044428,nuclear part|GO:0043227,membrane-bounded organelle|GO:0043226,organelle|GO:0044422,organelle part|GO:0043657,host cell|GO:0044216,other organism cell|GO:0018995,host|GO:0044217,other organism part|GO:0044215,other organism	GO:0036002,pre-mRNA binding|GO:0005515,protein binding|GO:0097159,organic cyclic compound binding|GO:0044822,poly(A) RNA binding|GO:0000166,nucleotide binding|GO:0036094,small molecule binding|GO:0003723,RNA binding|GO:0003676,nucleic acid binding|GO:1901363,heterocyclic compound binding|GO:1901265,nucleoside phosphate binding|GO:0005488,binding
TMEM41B	GO:0032502,developmental process|GO:0032501,multicellular organismal process|GO:0007399,nervous system development|GO:0044707,single-multicellular organism process|GO:0048856,anatomical structure development|GO:0044767,single-organism developmental process|GO:0048731,system development|GO:0007275,multicellular organism development|GO:0044699,single-organism process	GO:0044425,membrane part|GO:0016021,integral component of membrane|GO:0016020,membrane|GO:0031224,intrinsic component of membrane	
UTP3	GO:0043933,macromolecular complex subunit organization|GO:0006807,nitrogen compound metabolic process|GO:0090304,nucleic acid metabolic process|GO:0034641,cellular nitrogen compound metabolic process|GO:0060322,head development|GO:1901360,organic cyclic compound metabolic process|GO:0034660,ncRNA metabolic process|GO:0007275,multicellular organism development|GO:0044699,single-organism process|GO:0007417,central nervous system development|GO:0006139,nucleobase-containing compound metabolic process|GO:0044260,cellular macromolecule metabolic process|GO:0042274,ribosomal small subunit biogenesis|GO:0006325,chromatin organization|GO:0071840,cellular component organization or biogenesis|GO:0042254,ribosome biogenesis|GO:0016043,cellular component organization|GO:0071704,organic substance metabolic process|GO:0010467,gene expression|GO:0048513,animal organ development|GO:0006364,rRNA processing|GO:0022613,ribonucleoprotein complex biogenesis|GO:0046483,heterocycle metabolic process|GO:0032502,developmental process|GO:0016070,RNA metabolic process|GO:0032501,multicellular organismal process|GO:0034470,ncRNA processing|GO:0009987,cellular process|GO:0006725,cellular aromatic compound metabolic process|GO:0044767,single-organism developmental process|GO:0000462,maturation of SSU-rRNA from tricistronic rRNA transcript (SSU-rRNA, 5.8S rRNA, LSU-rRNA)|GO:0008152,metabolic process|GO:0048731,system development|GO:0051276,chromosome organization|GO:0006996,organelle organization|GO:0044238,primary metabolic process|GO:0016072,rRNA metabolic process|GO:0007420,brain development|GO:0044707,single-multicellular organism process|GO:0030490,maturation of SSU-rRNA|GO:0048856,anatomical structure development|GO:0044237,cellular metabolic process|GO:0043170,macromolecule metabolic process|GO:0044085,cellular component biogenesis|GO:0006396,RNA processing|GO:0007399,nervous system development|GO:0016568,chromatin modification|GO:0043412,macromolecule modification|GO:0016569,covalent chromatin modification	GO:0032040,small-subunit processome|GO:0031974,membrane-enclosed lumen|GO:0030684,preribosome|GO:0043229,intracellular organelle|GO:0043228,non-membrane-bounded organelle|GO:0043227,membrane-bounded organelle|GO:0043226,organelle|GO:0031981,nuclear lumen|GO:0005730,nucleolus|GO:0005634,nucleus|GO:0030529,intracellular ribonucleoprotein complex|GO:0032991,macromolecular complex|GO:0043231,intracellular membrane-bounded organelle|GO:0043232,intracellular non-membrane-bounded organelle|GO:0043233,organelle lumen|GO:0044464,cell part|GO:0005623,cell|GO:0005622,intracellular|GO:0044446,intracellular organelle part|GO:0070013,intracellular organelle lumen|GO:0044428,nuclear part|GO:0044424,intracellular part|GO:0044422,organelle part|GO:0005654,nucleoplasm|GO:1990904,ribonucleoprotein complex	GO:0003676,nucleic acid binding|GO:0097159,organic cyclic compound binding|GO:0044822,poly(A) RNA binding|GO:0003723,RNA binding|GO:0005515,protein binding|GO:1901363,heterocyclic compound binding|GO:0005488,binding
NANOS3	GO:2001234,negative regulation of apoptotic signaling pathway|GO:0080090,regulation of primary metabolic process|GO:0019222,regulation of metabolic process|GO:2001233,regulation of apoptotic signaling pathway|GO:0048585,negative regulation of response to stimulus|GO:0048583,regulation of response to stimulus|GO:0007292,female gamete generation|GO:0007165,signal transduction|GO:1901360,organic cyclic compound metabolic process|GO:1901361,organic cyclic compound catabolic process|GO:0051716,cellular response to stimulus|GO:0010605,negative regulation of macromolecule metabolic process|GO:0010604,positive regulation of macromolecule metabolic process|GO:0009968,negative regulation of signal transduction|GO:0009966,regulation of signal transduction|GO:0010608,posttranscriptional regulation of gene expression|GO:0043043,peptide biosynthetic process|GO:0010467,gene expression|GO:0048518,positive regulation of biological process|GO:0048519,negative regulation of biological process|GO:0051704,multi-organism process|GO:0031331,positive regulation of cellular catabolic process|GO:0060255,regulation of macromolecule metabolic process|GO:0060548,negative regulation of cell death|GO:0061013,regulation of mRNA catabolic process|GO:0061014,positive regulation of mRNA catabolic process|GO:0007281,germ cell development|GO:0003006,developmental process involved in reproduction|GO:0007283,spermatogenesis|GO:0046483,heterocycle metabolic process|GO:0044700,single organism signaling|GO:0044703,multi-organism reproductive process|GO:0044702,single organism reproductive process|GO:1901564,organonitrogen compound metabolic process|GO:1901566,organonitrogen compound biosynthetic process|GO:0044707,single-multicellular organism process|GO:0044249,cellular biosynthetic process|GO:0019538,protein metabolic process|GO:0019439,aromatic compound catabolic process|GO:0009896,positive regulation of catabolic process|GO:0009894,regulation of catabolic process|GO:0009892,negative regulation of metabolic process|GO:0009893,positive regulation of metabolic process|GO:0009890,negative regulation of biosynthetic process|GO:0010629,negative regulation of gene expression|GO:0006807,nitrogen compound metabolic process|GO:0042981,regulation of apoptotic process|GO:0050789,regulation of biological process|GO:0044267,cellular protein metabolic process|GO:1901575,organic substance catabolic process|GO:0044265,cellular macromolecule catabolic process|GO:0044260,cellular macromolecule metabolic process|GO:0065007,biological regulation|GO:0044699,single-organism process|GO:0048477,oogenesis|GO:0016265,obsolete death|GO:0009889,regulation of biosynthetic process|GO:0048468,cell development|GO:0050794,regulation of cellular process|GO:0012501,programmed cell death|GO:0008152,metabolic process|GO:0034655,nucleobase-containing compound catabolic process|GO:0046700,heterocycle catabolic process|GO:0000003,reproduction|GO:0016070,RNA metabolic process|GO:0016071,mRNA metabolic process|GO:0044271,cellular nitrogen compound biosynthetic process|GO:0044270,cellular nitrogen compound catabolic process|GO:0050896,response to stimulus|GO:0010556,regulation of macromolecule biosynthetic process|GO:0048869,cellular developmental process|GO:0006518,peptide metabolic process|GO:1903311,regulation of mRNA metabolic process|GO:0010558,negative regulation of macromolecule biosynthetic process|GO:1903313,positive regulation of mRNA metabolic process|GO:0030154,cell differentiation|GO:0019953,sexual reproduction|GO:0044248,cellular catabolic process|GO:0023057,negative regulation of signaling|GO:0034641,cellular nitrogen compound metabolic process|GO:0023052,signaling|GO:0010648,negative regulation of cell communication|GO:0034645,cellular macromolecule biosynthetic process|GO:0023051,regulation of signaling|GO:0010646,regulation of cell communication|GO:0007049,cell cycle|GO:0006139,nucleobase-containing compound metabolic process|GO:0051248,negative regulation of protein metabolic process|GO:0000288,nuclear-transcribed mRNA catabolic process, deadenylation-dependent decay|GO:0051246,regulation of protein metabolic process|GO:0032502,developmental process|GO:0032501,multicellular organismal process|GO:0048609,multicellular organismal reproductive process|GO:0032504,multicellular organism reproduction|GO:0031323,regulation of cellular metabolic process|GO:0009987,cellular process|GO:0006725,cellular aromatic compound metabolic process|GO:0034249,negative regulation of cellular amide metabolic process|GO:0034248,regulation of cellular amide metabolic process|GO:0048232,male gamete generation|GO:0043604,amide biosynthetic process|GO:0032269,negative regulation of cellular protein metabolic process|GO:0032268,regulation of cellular protein metabolic process|GO:0043603,cellular amide metabolic process|GO:0051252,regulation of RNA metabolic process|GO:0051254,positive regulation of RNA metabolic process|GO:0043170,macromolecule metabolic process|GO:0000956,nuclear-transcribed mRNA catabolic process|GO:0017148,negative regulation of translation|GO:0031329,regulation of cellular catabolic process|GO:0031327,negative regulation of cellular biosynthetic process|GO:0031326,regulation of cellular biosynthetic process|GO:0031325,positive regulation of cellular metabolic process|GO:0031324,negative regulation of cellular metabolic process|GO:0097190,apoptotic signaling pathway|GO:0090304,nucleic acid metabolic process|GO:0008219,cell death|GO:0010941,regulation of cell death|GO:0007275,multicellular organism development|GO:0007276,gamete generation|GO:2000112,regulation of cellular macromolecule biosynthetic process|GO:2000113,negative regulation of cellular macromolecule biosynthetic process|GO:0071704,organic substance metabolic process|GO:0043067,regulation of programmed cell death|GO:0043066,negative regulation of apoptotic process|GO:1900153,positive regulation of nuclear-transcribed mRNA catabolic process, deadenylation-dependent decay|GO:1900151,regulation of nuclear-transcribed mRNA catabolic process, deadenylation-dependent decay|GO:0006401,RNA catabolic process|GO:0006402,mRNA catabolic process|GO:0043069,negative regulation of programmed cell death|GO:0010468,regulation of gene expression|GO:0045935,positive regulation of nucleobase-containing compound metabolic process|GO:1901576,organic substance biosynthetic process|GO:0019219,regulation of nucleobase-containing compound metabolic process|GO:0006915,apoptotic process|GO:0044767,single-organism developmental process|GO:0022414,reproductive process|GO:0009058,biosynthetic process|GO:0009059,macromolecule biosynthetic process|GO:0044763,single-organism cellular process|GO:0051171,regulation of nitrogen compound metabolic process|GO:0051172,negative regulation of nitrogen compound metabolic process|GO:0022412,cellular process involved in reproduction in multicellular organism|GO:0007154,cell communication|GO:0009056,catabolic process|GO:0009057,macromolecule catabolic process|GO:0044238,primary metabolic process|GO:0051173,positive regulation of nitrogen compound metabolic process|GO:0051726,regulation of cell cycle|GO:0048856,anatomical structure development|GO:0044237,cellular metabolic	GO:0010494,cytoplasmic stress granule|GO:0005737,cytoplasm|GO:0035770,ribonucleoprotein granule|GO:0043231,intracellular membrane-bounded organelle|GO:0043232,intracellular non-membrane-bounded organelle|GO:0044464,cell part|GO:0005634,nucleus|GO:0000932,P-body|GO:0044444,cytoplasmic part|GO:0005623,cell|GO:0030529,intracellular ribonucleoprotein complex|GO:0043229,intracellular organelle|GO:0043228,non-membrane-bounded organelle|GO:0036464,cytoplasmic ribonucleoprotein granule|GO:0044424,intracellular part|GO:0032991,macromolecular complex|GO:0048471,perinuclear region of cytoplasm|GO:0005622,intracellular|GO:0043227,membrane-bounded organelle|GO:0043226,organelle|GO:1990904,ribonucleoprotein complex	GO:0005488,binding|GO:0043169,cation binding|GO:0005515,protein binding|GO:0046914,transition metal ion binding|GO:0097159,organic cyclic compound binding|GO:0043167,ion binding|GO:0008270,zinc ion binding|GO:0003676,nucleic acid binding|GO:0046872,metal ion binding|GO:1901363,heterocyclic compound binding|GO:0003723,RNA binding
TNP1	GO:0080090,regulation of primary metabolic process|GO:0019222,regulation of metabolic process|GO:0007286,spermatid development|GO:0048468,cell development|GO:0007290,spermatid nucleus elongation|GO:1901362,organic cyclic compound biosynthetic process|GO:0071840,cellular component organization or biogenesis|GO:0044710,single-organism metabolic process|GO:0010605,negative regulation of macromolecule metabolic process|GO:0000003,reproduction|GO:0048869,cellular developmental process|GO:0043044,ATP-dependent chromatin remodeling|GO:0040029,regulation of gene expression, epigenetic|GO:0016458,gene silencing|GO:0048515,spermatid differentiation|GO:0048519,negative regulation of biological process|GO:0051704,multi-organism process|GO:0006281,DNA repair|GO:0060255,regulation of macromolecule metabolic process|GO:0007281,germ cell development|GO:0003006,developmental process involved in reproduction|GO:0007283,spermatogenesis|GO:2001141,regulation of RNA biosynthetic process|GO:0007289,spermatid nucleus differentiation|GO:0046483,heterocycle metabolic process|GO:0044703,multi-organism reproductive process|GO:0044702,single organism reproductive process|GO:0044707,single-multicellular organism process|GO:0048870,cell motility|GO:0000012,single strand break repair|GO:0009566,fertilization|GO:0033554,cellular response to stress|GO:0019438,aromatic compound biosynthetic process|GO:0016568,chromatin modification|GO:0022607,cellular component assembly|GO:0009892,negative regulation of metabolic process|GO:0009890,negative regulation of biosynthetic process|GO:0006928,movement of cell or subcellular component|GO:0010629,negative regulation of gene expression|GO:0006807,nitrogen compound metabolic process|GO:0050789,regulation of biological process|GO:0097659,nucleic acid-templated transcription|GO:1901576,organic substance biosynthetic process|GO:0044260,cellular macromolecule metabolic process|GO:0016043,cellular component organization|GO:0065007,biological regulation|GO:1901360,organic cyclic compound metabolic process|GO:0018130,heterocycle biosynthetic process|GO:0009889,regulation of biosynthetic process|GO:0051716,cellular response to stimulus|GO:0050794,regulation of cellular process|GO:0006950,response to stress|GO:0008152,metabolic process|GO:0034654,nucleobase-containing compound biosynthetic process|GO:0016070,RNA metabolic process|GO:0044767,single-organism developmental process|GO:0044271,cellular nitrogen compound biosynthetic process|GO:0050896,response to stimulus|GO:0006355,regulation of transcription, DNA-templated|GO:0010556,regulation of macromolecule biosynthetic process|GO:0006351,transcription, DNA-templated|GO:0043486,histone exchange|GO:0022411,cellular component disassembly|GO:0010558,negative regulation of macromolecule biosynthetic process|GO:0034728,nucleosome organization|GO:0032774,RNA biosynthetic process|GO:0030154,cell differentiation|GO:0019953,sexual reproduction|GO:0044249,cellular biosynthetic process|GO:0034641,cellular nitrogen compound metabolic process|GO:0034645,cellular macromolecule biosynthetic process|GO:0031498,chromatin disassembly|GO:0043241,protein complex disassembly|GO:0044699,single-organism process|GO:0006139,nucleobase-containing compound metabolic process|GO:0006342,chromatin silencing|GO:0043933,macromolecular complex subunit organization|GO:0032502,developmental process|GO:0040011,locomotion|GO:0035039,male pronucleus assembly|GO:0032501,multicellular organismal process|GO:0048609,multicellular organismal reproductive process|GO:0032504,multicellular organism reproduction|GO:0044238,primary metabolic process|GO:0031323,regulation of cellular metabolic process|GO:0030317,flagellated sperm motility|GO:0009987,cellular process|GO:0006725,cellular aromatic compound metabolic process|GO:1903506,regulation of nucleic acid-templated transcription|GO:1903507,negative regulation of nucleic acid-templated transcription|GO:0006974,cellular response to DNA damage stimulus|GO:0045892,negative regulation of transcription, DNA-templated|GO:0007338,single fertilization|GO:0048232,male gamete generation|GO:0006338,chromatin remodeling|GO:0006337,nucleosome disassembly|GO:0051253,negative regulation of RNA metabolic process|GO:0051252,regulation of RNA metabolic process|GO:0006333,chromatin assembly or disassembly|GO:0043170,macromolecule metabolic process|GO:0051674,localization of cell|GO:0031327,negative regulation of cellular biosynthetic process|GO:0031326,regulation of cellular biosynthetic process|GO:0031324,negative regulation of cellular metabolic process|GO:0035042,fertilization, exchange of chromosomal proteins|GO:0035041,sperm chromatin decondensation|GO:0090304,nucleic acid metabolic process|GO:0007275,multicellular organism development|GO:0007276,gamete generation|GO:0032986,protein-DNA complex disassembly|GO:0032984,macromolecular complex disassembly|GO:0071822,protein complex subunit organization|GO:0006325,chromatin organization|GO:2000112,regulation of cellular macromolecule biosynthetic process|GO:2000113,negative regulation of cellular macromolecule biosynthetic process|GO:0071704,organic substance metabolic process|GO:0010467,gene expression|GO:0045814,negative regulation of gene expression, epigenetic|GO:0010468,regulation of gene expression|GO:0071824,protein-DNA complex subunit organization|GO:0045934,negative regulation of nucleobase-containing compound metabolic process|GO:0019219,regulation of nucleobase-containing compound metabolic process|GO:1902679,negative regulation of RNA biosynthetic process|GO:0022414,reproductive process|GO:0009058,biosynthetic process|GO:0009059,macromolecule biosynthetic process|GO:0044763,single-organism cellular process|GO:0051171,regulation of nitrogen compound metabolic process|GO:0051172,negative regulation of nitrogen compound metabolic process|GO:0022412,cellular process involved in reproduction in multicellular organism|GO:0070925,organelle assembly|GO:0051179,localization|GO:0006996,organelle organization|GO:0006997,nucleus organization|GO:0051276,chromosome organization|GO:0048856,anatomical structure development|GO:0044237,cellular metabolic process|GO:1902589,single-organism organelle organization|GO:0044085,cellular component biogenesis|GO:0006259,DNA metabolic process|GO:0048523,negative regulation of cellular process|GO:0010604,positive regulation of macromolecule metabolic process|GO:0048518,positive regulation of biological process|GO:0030162,regulation of proteolysis|GO:1901564,organonitrogen compound metabolic process|GO:0019538,protein metabolic process|GO:0071103,DNA conformation change|GO:0009893,positive regulation of metabolic process|GO:0010628,positive regulation of gene expression|GO:0044267,cellular protein metabolic process|GO:0070613,regulation of protein processing|GO:0051604,protein maturation|GO:0035093,spermatogenesis, exchange of chromosomal proteins|GO:0035092,sperm chromatin condensation|GO:1903319,positive regulation of protein maturation|GO:1903317,regulation of protein maturation|GO:0051246,regulation of protein metabolic process|GO:0051247,positive regulation of protein metabolic process|GO:0032270,positive regulation of cellular protein metabolic process|GO:0006508,proteolysis|GO:0016485,protein processing|GO:0032268,regulation of cellular protein metabolic process|GO:0045862,positive regulation of	GO:0044815,DNA packaging complex|GO:0043229,intracellular organelle|GO:0043228,non-membrane-bounded organelle|GO:0000785,chromatin|GO:0000786,nucleosome|GO:0043227,membrane-bounded organelle|GO:0043226,organelle|GO:0005634,nucleus|GO:0005694,chromosome|GO:0001673,male germ cell nucleus|GO:0043234,protein complex|GO:0032991,macromolecular complex|GO:0043231,intracellular membrane-bounded organelle|GO:0043232,intracellular non-membrane-bounded organelle|GO:0044464,cell part|GO:0005623,cell|GO:0005622,intracellular|GO:0044446,intracellular organelle part|GO:0043073,germ cell nucleus|GO:0044424,intracellular part|GO:0032993,protein-DNA complex|GO:0044427,chromosomal part|GO:0044422,organelle part|GO:0031974,membrane-enclosed lumen|GO:0000788,nuclear nucleosome|GO:0000228,nuclear chromosome|GO:0031981,nuclear lumen|GO:0044454,nuclear chromosome part|GO:0000790,nuclear chromatin|GO:0043233,organelle lumen|GO:0070013,intracellular organelle lumen|GO:0044428,nuclear part	GO:0003676,nucleic acid binding|GO:0097159,organic cyclic compound binding|GO:0005488,binding|GO:0005515,protein binding|GO:0003677,DNA binding|GO:1901363,heterocyclic compound binding
ACN9	GO:0080090,regulation of primary metabolic process|GO:0019222,regulation of metabolic process|GO:0031326,regulation of cellular biosynthetic process|GO:0031323,regulation of cellular metabolic process|GO:0010906,regulation of glucose metabolic process|GO:0044249,cellular biosynthetic process|GO:0044281,small molecule metabolic process|GO:0050789,regulation of biological process|GO:0044699,single-organism process|GO:0006094,gluconeogenesis|GO:1901576,organic substance biosynthetic process|GO:0044710,single-organism metabolic process|GO:0044711,single-organism biosynthetic process|GO:0044262,cellular carbohydrate metabolic process|GO:0010565,regulation of cellular ketone metabolic process|GO:0071704,organic substance metabolic process|GO:0065007,biological regulation|GO:0046364,monosaccharide biosynthetic process|GO:0009987,cellular process|GO:0009889,regulation of biosynthetic process|GO:0006111,regulation of gluconeogenesis|GO:0019318,hexose metabolic process|GO:0019319,hexose biosynthetic process|GO:0050794,regulation of cellular process|GO:0009058,biosynthetic process|GO:0044763,single-organism cellular process|GO:0010675,regulation of cellular carbohydrate metabolic process|GO:0008152,metabolic process|GO:0044723,single-organism carbohydrate metabolic process|GO:0043255,regulation of carbohydrate biosynthetic process|GO:0044238,primary metabolic process|GO:0005975,carbohydrate metabolic process|GO:0042180,cellular ketone metabolic process|GO:0005996,monosaccharide metabolic process|GO:0044237,cellular metabolic process|GO:0016051,carbohydrate biosynthetic process|GO:0006006,glucose metabolic process|GO:0006109,regulation of carbohydrate metabolic process|GO:0022607,cellular component assembly|GO:0070271,protein complex biogenesis|GO:0043933,macromolecular complex subunit organization|GO:0044283,small molecule biosynthetic process|GO:0019752,carboxylic acid metabolic process|GO:0034622,cellular macromolecular complex assembly|GO:0007005,mitochondrion organization|GO:0071822,protein complex subunit organization|GO:0071840,cellular component organization or biogenesis|GO:0016043,cellular component organization|GO:0065003,macromolecular complex assembly|GO:0034553,mitochondrial respiratory chain complex II assembly|GO:0034552,respiratory chain complex II assembly|GO:0033108,mitochondrial respiratory chain complex assembly|GO:0006461,protein complex assembly|GO:0043436,oxoacid metabolic process|GO:0043623,cellular protein complex assembly|GO:0006979,response to oxidative stress|GO:0006996,organelle organization|GO:0043648,dicarboxylic acid metabolic process|GO:0006082,organic acid metabolic process|GO:0097032,mitochondrial respiratory chain complex II biogenesis|GO:0050896,response to stimulus|GO:0006950,response to stress|GO:0006105,succinate metabolic process|GO:0044085,cellular component biogenesis	GO:0031970,organelle envelope lumen|GO:0031974,membrane-enclosed lumen|GO:0031975,envelope|GO:0043229,intracellular organelle|GO:0043227,membrane-bounded organelle|GO:0043226,organelle|GO:0005737,cytoplasm|GO:0005740,mitochondrial envelope|GO:0005739,mitochondrion|GO:0005759,mitochondrial matrix|GO:0005758,mitochondrial intermembrane space|GO:0031967,organelle envelope|GO:0043231,intracellular membrane-bounded organelle|GO:0043233,organelle lumen|GO:0044464,cell part|GO:0005623,cell|GO:0005622,intracellular|GO:0044446,intracellular organelle part|GO:0070013,intracellular organelle lumen|GO:0044444,cytoplasmic part|GO:0044429,mitochondrial part|GO:0044424,intracellular part|GO:0044422,organelle part	
FAM72C		GO:0005737,cytoplasm|GO:0043231,intracellular membrane-bounded organelle|GO:0005829,cytosol|GO:0044464,cell part|GO:0043229,intracellular organelle|GO:0005623,cell|GO:0005622,intracellular|GO:0044444,cytoplasmic part|GO:0044424,intracellular part|GO:0043227,membrane-bounded organelle|GO:0043226,organelle	

TMSB4X	GO:0006887,exocytosis|GO:0007599,hemostasis|GO:0033043,regulation of organelle organization|GO:0043933,macromolecular complex subunit organization|GO:0006810,transport|GO:0007596,blood coagulation|GO:0051129,negative regulation of cellular component organization|GO:0051128,regulation of cellular component organization|GO:0008064,regulation of actin polymerization or depolymerization|GO:0032940,secretion by cell|GO:0022607,cellular component assembly|GO:0042989,sequestering of actin monomers|GO:0051641,cellular localization|GO:0034622,cellular macromolecular complex assembly|GO:0071840,cellular component organization or biogenesis|GO:0008104,protein localization|GO:0051651,maintenance of location in cell|GO:0050789,regulation of biological process|GO:0044699,single-organism process|GO:0065003,macromolecular complex assembly|GO:0030832,regulation of actin filament length|GO:0030833,regulation of actin filament polymerization|GO:0050878,regulation of body fluid levels|GO:0071822,protein complex subunit organization|GO:0030837,negative regulation of actin filament polymerization|GO:0002576,platelet degranulation|GO:0051494,negative regulation of cytoskeleton organization|GO:0051493,regulation of cytoskeleton organization|GO:0009611,response to wounding|GO:0016043,cellular component organization|GO:0090066,regulation of anatomical structure size|GO:0044765,single-organism transport|GO:0065007,biological regulation|GO:0032271,regulation of protein polymerization|GO:0032272,negative regulation of protein polymerization|GO:1902578,single-organism localization|GO:0048519,negative regulation of biological process|GO:0065008,regulation of biological quality|GO:0051649,establishment of localization in cell|GO:0034613,cellular protein localization|GO:0032970,regulation of actin filament-based process|GO:0032507,maintenance of protein location in cell|GO:0030029,actin filament-based process|GO:0051258,protein polymerization|GO:0032501,multicellular organismal process|GO:0031333,negative regulation of protein complex assembly|GO:0042060,wound healing|GO:0050794,regulation of cellular process|GO:0008154,actin polymerization or depolymerization|GO:0045185,maintenance of protein location|GO:0001775,cell activation|GO:0050817,coagulation|GO:0044763,single-organism cellular process|GO:0030036,actin cytoskeleton organization|GO:0032956,regulation of actin cytoskeleton organization|GO:0030041,actin filament polymerization|GO:0043254,regulation of protein complex assembly|GO:0051234,establishment of localization|GO:0043623,cellular protein complex assembly|GO:0051179,localization|GO:0030168,platelet activation|GO:0016192,vesicle-mediated transport|GO:0006996,organelle organization|GO:0007015,actin filament organization|GO:0046903,secretion|GO:0033036,macromolecule localization|GO:0007010,cytoskeleton organization|GO:0006461,protein complex assembly|GO:0044707,single-multicellular organism process|GO:0051235,maintenance of location|GO:0070271,protein complex biogenesis|GO:0070727,cellular macromolecule localization|GO:0010639,negative regulation of organelle organization|GO:0044087,regulation of cellular component biogenesis|GO:1902589,single-organism organelle organization|GO:0044085,cellular component biogenesis|GO:0032535,regulation of cellular component size|GO:0006950,response to stress|GO:0009987,cellular process|GO:0050896,response to stimulus|GO:0048523,negative regulation of cellular process|GO:0033157,regulation of intracellular protein transport|GO:0051169,nuclear transport|GO:0051046,regulation of secretion|GO:0051048,negative regulation of secretion|GO:0051049,regulation of transport|GO:0009167,purine ribonucleoside monophosphate metabolic process|GO:0009165,nucleotide biosynthetic process|GO:0044281,small molecule metabolic process|GO:0009161,ribonucleoside monophosphate metabolic process|GO:0009168,purine ribonucleoside monophosphate biosynthetic process|GO:0051716,cellular response to stimulus|GO:0009199,ribonucleoside triphosphate metabolic process|GO:0032386,regulation of intracellular transport|GO:0032387,negative regulation of intracellular transport|GO:0032101,regulation of response to external stimulus|GO:0046483,heterocycle metabolic process|GO:0010634,positive regulation of epithelial cell migration|GO:0010631,epithelial cell migration|GO:0010632,regulation of epithelial cell migration|GO:1904589,regulation of protein import|GO:0051252,regulation of RNA metabolic process|GO:0009893,positive regulation of metabolic process|GO:0009891,positive regulation of biosynthetic process|GO:0051220,cytoplasmic sequestering of protein|GO:0035556,intracellular signal transduction|GO:0051223,regulation of protein transport|GO:0051224,negative regulation of protein transport|GO:2000677,regulation of transcription regulatory region DNA binding|GO:0006886,intracellular protein transport|GO:1901360,organic cyclic compound metabolic process|GO:0018130,heterocycle biosynthetic process|GO:0070201,regulation of establishment of protein localization|GO:0009306,protein secretion|GO:0009260,ribonucleotide biosynthetic process|GO:0016070,RNA metabolic process|GO:0050710,negative regulation of cytokine secretion|GO:0071345,cellular response to cytokine stimulus|GO:0010556,regulation of macromolecule biosynthetic process|GO:1901293,nucleoside phosphate biosynthetic process|GO:0006753,nucleoside phosphate metabolic process|GO:0006754,ATP biosynthetic process|GO:0033209,tumor necrosis factor-mediated signaling pathway|GO:1903827,regulation of cellular protein localization|GO:1903828,negative regulation of cellular protein localization|GO:0030810,positive regulation of nucleotide biosynthetic process|GO:0032677,regulation of interleukin-8 production|GO:0048518,positive regulation of biological process|GO:0060341,regulation of cellular localization|GO:1901223,negative regulation of NIK/NF-kappaB signaling|GO:1901222,regulation of NIK/NF-kappaB signaling|GO:2000112,regulation of cellular macromolecule biosynthetic process|GO:0043062,extracellular structure organization|GO:0009605,response to external stimulus|GO:0019219,regulation of nucleobase-containing compound metabolic process|GO:0090317,negative regulation of intracellular protein transport|GO:0040011,locomotion|GO:0051272,positive regulation of cellular component movement|GO:0051270,regulation of cellular component movement|GO:0010595,positive regulation of endothelial cell migration|GO:0010594,regulation of endothelial cell migration|GO:0040017,positive regulation of locomotion|GO:0043536,positive regulation of blood vessel endothelial cell migration|GO:0019693,ribose phosphate metabolic process|GO:0006796,phosphate-containing compound metabolic process|GO:0006793,phosphorus metabolic process|GO:0006140,regulation of nucleotide metabolic process|GO:0048522,positive regulation of cellular process|GO:1903578,regulation of ATP metabolic process|GO:0007165,signal transduction|GO:0007166,cell surface receptor signaling pathway|GO:0031347,regulation of defense response|GO:0044710,single-organism metabolic process|GO:0044711,single-organism biosynthetic process|GO:0050727,regulation of inflammatory response|GO:0044092,negative regulation of molecular function|GO:0006935,chemotaxis|GO:0051051,negative regulation of transport|GO:0046034,ATP metabolic process|GO:2001028,positive regulation of endothelial cell chemotaxis|GO:2001141,regulation of RNA biosynthetic process|GO:0042994,cytoplasmic sequestering of transcription factor|GO:0042992,negative regulation of transcription factor import into nucleus|GO:0010033,response to organic substance|GO:0042990,regulation of transcription factor import into nucleus|GO:0042991,transcription factor import into nucleus|GO:0031503,protein complex localization|GO:1901566,organonitrogen compound biosynthetic process|GO:0044249,cellular biosynthetic process|GO:1900373,positive regulation of purine nucleotide biosynthetic process|GO:1900371,regulation of purine nucleotide biosynthetic process|GO:1903533,regulation of protein targeting|GO:0006807,nitrogen compound metabolic process|GO:0044260,cellular macromolecule metabolic process|GO:0035767,endothelial cell chemotaxis|GO:0009889,regulation of biosynthetic process|GO:0051239,regulation of multicellular organismal process|GO:0090407,organophosphate biosynthetic process|GO:0046390,ribose phosphate biosynthetic process|GO:0032088,negative regulation of NF-kappaB transcription factor activity|GO:0032717,negative regulation of interleukin-8 production|GO:2000145,regulation of cell motility|GO:2000147,positive regulation of cell motility|GO:0010562,positive regulation of phosphorus metabolic process|GO:0051171,regulation of nitrogen compound metabolic	GO:0031974,membrane-enclosed lumen|GO:0043229,intracellular organelle|GO:0043228,non-membrane-bounded organelle|GO:0060205,cytoplasmic membrane-bounded vesicle lumen|GO:0043227,membrane-bounded organelle|GO:0043226,organelle|GO:0005856,cytoskeleton|GO:0031983,vesicle lumen|GO:0031982,vesicle|GO:0031093,platelet alpha granule lumen|GO:0016023,cytoplasmic, membrane-bounded vesicle|GO:0031410,cytoplasmic vesicle|GO:0031988,membrane-bounded vesicle|GO:0044433,cytoplasmic vesicle part|GO:0005737,cytoplasm|GO:0030141,secretory granule|GO:0012505,endomembrane system|GO:0034774,secretory granule lumen|GO:0031091,platelet alpha granule|GO:0043231,intracellular membrane-bounded organelle|GO:0043232,intracellular non-membrane-bounded organelle|GO:0043233,organelle lumen|GO:0044464,cell part|GO:0005623,cell|GO:0005622,intracellular|GO:0044446,intracellular organelle part|GO:0044444,cytoplasmic part|GO:0005576,extracellular region|GO:0044424,intracellular part|GO:0044422,organelle part|GO:0005634,nucleus|GO:0099503,secretory vesicle|GO:0097708,intracellular vesicle|GO:0005829,cytosol	GO:0005488,binding|GO:0005515,protein binding|GO:0008092,cytoskeletal protein binding|GO:0097159,organic cyclic compound binding|GO:0044822,poly(A) RNA binding|GO:0003779,actin binding|GO:0003785,actin monomer binding|GO:0003676,nucleic acid binding|GO:1901363,heterocyclic compound binding|GO:0003723,RNA binding|GO:0019899,enzyme binding
CCNG1	GO:1904029,regulation of cyclin-dependent protein kinase activity|GO:0019220,regulation of phosphate metabolic process|GO:0080090,regulation of primary metabolic process|GO:0019222,regulation of metabolic process|GO:0000280,nuclear division|GO:0031323,regulation of cellular metabolic process|GO:1903047,mitotic cell cycle process|GO:0022402,cell cycle process|GO:0044237,cellular metabolic process|GO:0007067,mitotic nuclear division|GO:0071900,regulation of protein serine/threonine kinase activity|GO:0050789,regulation of biological process|GO:0007049,cell cycle|GO:0044267,cellular protein metabolic process|GO:0044260,cellular macromolecule metabolic process|GO:0071840,cellular component organization or biogenesis|GO:0043549,regulation of kinase activity|GO:0051246,regulation of protein metabolic process|GO:0006796,phosphate-containing compound metabolic process|GO:0016043,cellular component organization|GO:0071704,organic substance metabolic process|GO:0065007,biological regulation|GO:0051301,cell division|GO:0031399,regulation of protein modification process|GO:0065009,regulation of molecular function|GO:0006996,organelle organization|GO:0006468,protein phosphorylation|GO:0045859,regulation of protein kinase activity|GO:0000278,mitotic cell cycle|GO:0060255,regulation of macromolecule metabolic process|GO:0050790,regulation of catalytic activity|GO:0016310,phosphorylation|GO:0006464,cellular protein modification process|GO:0050794,regulation of cellular process|GO:0051174,regulation of phosphorus metabolic process|GO:0043412,macromolecule modification|GO:0036211,protein modification process|GO:0044763,single-organism cellular process|GO:0008152,metabolic process|GO:0042325,regulation of phosphorylation|GO:0044238,primary metabolic process|GO:0044699,single-organism process|GO:0032268,regulation of cellular protein metabolic process|GO:0051726,regulation of cell cycle|GO:0019538,protein metabolic process|GO:0000079,regulation of cyclin-dependent protein serine/threonine kinase activity|GO:0051338,regulation of transferase activity|GO:0043170,macromolecule metabolic process|GO:1902589,single-organism organelle organization|GO:0048285,organelle fission|GO:0006793,phosphorus metabolic process|GO:0009987,cellular process|GO:0001932,regulation of protein phosphorylation|GO:0006807,nitrogen compound metabolic process|GO:1901564,organonitrogen compound metabolic process	GO:0043231,intracellular membrane-bounded organelle|GO:0005634,nucleus|GO:0044464,cell part|GO:0005623,cell|GO:0005622,intracellular|GO:0043229,intracellular organelle|GO:0044424,intracellular part|GO:0043227,membrane-bounded organelle|GO:0043226,organelle|GO:0044446,intracellular organelle part|GO:0031981,nuclear lumen|GO:0043233,organelle lumen|GO:0031974,membrane-enclosed lumen|GO:0005654,nucleoplasm|GO:0070013,intracellular organelle lumen|GO:0044428,nuclear part|GO:0044422,organelle part	GO:0005488,binding|GO:0005515,protein binding
FAM72D		GO:0005737,cytoplasm|GO:0043231,intracellular membrane-bounded organelle|GO:0005829,cytosol|GO:0044464,cell part|GO:0043229,intracellular organelle|GO:0005623,cell|GO:0005622,intracellular|GO:0044444,cytoplasmic part|GO:0044424,intracellular part|GO:0043227,membrane-bounded organelle|GO:0043226,organelle	
HNRNPU	GO:0032922,circadian regulation of gene expression|GO:0008152,metabolic process|GO:0019222,regulation of metabolic process|GO:0071704,organic substance metabolic process|GO:0030154,cell differentiation|GO:0090304,nucleic acid metabolic process|GO:0034641,cellular nitrogen compound metabolic process|GO:0001503,ossification|GO:0044237,cellular metabolic process|GO:0070934,CRD-mediated mRNA stabilization|GO:0043170,macromolecule metabolic process|GO:0050789,regulation of biological process|GO:0044699,single-organism process|GO:0006139,nucleobase-containing compound metabolic process|GO:0043489,RNA stabilization|GO:0043488,regulation of mRNA stability|GO:0043487,regulation of RNA stability|GO:0010608,posttranscriptional regulation of gene expression|GO:1901360,organic cyclic compound metabolic process|GO:0048511,rhythmic process|GO:0010467,gene expression|GO:0065007,biological regulation|GO:0001649,osteoblast differentiation|GO:0065008,regulation of biological quality|GO:0010468,regulation of gene expression|GO:0032502,developmental process|GO:0032501,multicellular organismal process|GO:0007623,circadian rhythm|GO:0044238,primary metabolic process|GO:0060255,regulation of macromolecule metabolic process|GO:0009987,cellular process|GO:0006725,cellular aromatic compound metabolic process|GO:0044767,single-organism developmental process|GO:0000375,RNA splicing, via transesterification reactions|GO:0000377,RNA splicing, via transesterification reactions with bulged adenosine as nucleophile|GO:0044763,single-organism cellular process|GO:0048255,mRNA stabilization|GO:0046483,heterocycle metabolic process|GO:0016070,RNA metabolic process|GO:0016071,mRNA metabolic process|GO:0044260,cellular macromolecule metabolic process|GO:0044707,single-multicellular organism process|GO:0000398,mRNA splicing, via spliceosome|GO:0008380,RNA splicing|GO:0006807,nitrogen compound metabolic process|GO:0048869,cellular developmental process|GO:0006396,RNA processing|GO:0006397,mRNA processing|GO:0080090,regulation of primary metabolic process|GO:0060249,anatomical structure homeostasis|GO:0032845,negative regulation of homeostatic process|GO:0032844,regulation of homeostatic process|GO:1901362,organic cyclic compound biosynthetic process|GO:0071840,cellular component organization or biogenesis|GO:0007004,telomere maintenance via telomerase|GO:0010605,negative regulation of macromolecule metabolic process|GO:0048519,negative regulation of biological process|GO:0051053,negative regulation of DNA metabolic process|GO:0051052,regulation of DNA metabolic process|GO:0006278,RNA-dependent DNA biosynthetic process|GO:0010639,negative regulation of organelle organization|GO:0019438,aromatic compound biosynthetic process|GO:0009892,negative regulation of metabolic process|GO:0009890,negative regulation of biosynthetic process|GO:1901576,organic substance biosynthetic process|GO:0016043,cellular component organization|GO:0018130,heterocycle biosynthetic process|GO:2000279,negative regulation of DNA biosynthetic process|GO:2000278,regulation of DNA biosynthetic process|GO:0009889,regulation of biosynthetic process|GO:0050794,regulation of cellular process|GO:0034654,nucleobase-containing compound biosynthetic process|GO:0044271,cellular nitrogen compound biosynthetic process|GO:0010556,regulation of macromolecule biosynthetic process|GO:0010558,negative regulation of macromolecule biosynthetic process|GO:2001251,negative regulation of chromosome organization|GO:0033044,regulation of chromosome organization|GO:0033043,regulation of organelle organization|GO:0051129,negative regulation of cellular component organization|GO:0051128,regulation of cellular component organization|GO:0044249,cellular biosynthetic process|GO:0034645,cellular macromolecule biosynthetic process|GO:0000723,telomere maintenance|GO:0010833,telomere maintenance via telomere lengthening|GO:0009059,macromolecule biosynthetic process|GO:0071897,DNA biosynthetic process|GO:0031327,negative regulation of cellular biosynthetic process|GO:0031326,regulation of cellular biosynthetic process|GO:0031324,negative regulation of cellular metabolic process|GO:0031323,regulation of cellular metabolic process|GO:0042592,homeostatic process|GO:2000112,regulation of cellular macromolecule biosynthetic process|GO:2000113,negative regulation of cellular macromolecule biosynthetic process|GO:0032210,regulation of telomere maintenance via telomerase|GO:0032211,negative regulation of telomere maintenance via telomerase|GO:0045934,negative regulation of nucleobase-containing compound metabolic process|GO:0019219,regulation of nucleobase-containing compound metabolic process|GO:1904356,regulation of telomere maintenance via telomere lengthening|GO:1904357,negative regulation of telomere maintenance via telomere lengthening|GO:0009058,biosynthetic process|GO:0051171,regulation of nitrogen compound metabolic process|GO:0051172,negative regulation of nitrogen compound metabolic process|GO:0006996,organelle organization|GO:0051276,chromosome organization|GO:0032205,negative regulation of telomere maintenance|GO:0032204,regulation of telomere maintenance|GO:0032200,telomere organization|GO:0048523,negative regulation of cellular process|GO:0006259,DNA metabolic process|GO:0051348,negative regulation of transferase activity|GO:0019220,regulation of phosphate metabolic process|GO:2000737,negative regulation of stem cell differentiation|GO:0050684,regulation of mRNA processing|GO:0035372,protein localization to microtubule|GO:0048468,cell development|GO:0009048,dosage compensation by inactivation of X chromosome|GO:0072359,circulatory system development|GO:0033047,regulation of mitotic sister chromatid segregation|GO:2000736,regulation of stem cell differentiation|GO:0051651,maintenance of location in cell|GO:0044710,single-organism metabolic process|GO:0048863,stem cell differentiation|GO:0090169,regulation of spindle assembly|GO:0070727,cellular macromolecule localization|GO:0051495,positive regulation of cytoskeleton organization|GO:0051493,regulation of cytoskeleton organization|GO:0040029,regulation of gene expression, epigenetic|GO:0008104,protein localization|GO:0048513,animal organ development|GO:0044093,positive regulation of molecular function|GO:0044092,negative regulation of molecular function|GO:0048518,positive regulation of biological process|GO:0007088,regulation of mitotic nuclear division|GO:0042127,regulation of cell proliferation|GO:0006793,phosphorus metabolic process|GO:0032781,positive regulation of ATPase activity|GO:0032784,regulation of DNA-templated transcription, elongation|GO:0032785,negative regulation of DNA-templated transcription, elongation|GO:0045185,maintenance of protein location|GO:2001141,regulation of RNA biosynthetic process|GO:0010033,response to organic substance|GO:0042325,regulation of phosphorylation|GO:0071383,cellular response to steroid hormone stimulus|GO:0042326,negative regulation of phosphorylation|GO:0051716,cellular response to stimulus|GO:1901673,regulation of mitotic spindle assembly|GO:0010638,positive regulation of organelle organization|GO:0051783,regulation of nuclear division|GO:0051781,positive regulation of cell division|GO:0051785,positive regulation of nuclear division|GO:0009725,response to hormone|GO:0070507,regulation of microtubule cytoskeleton organization|GO:0033673,negative regulation of kinase activity|GO:0045840,positive regulation of mitotic nuclear division|GO:0022607,cellular component assembly|GO:0009893,positive regulation of metabolic process|GO:0009891,positive regulation of biosynthetic process|GO:0061061,muscle structure development|GO:0010629,negative regulation of gene expression|GO:0010628,positive regulation of gene expression|GO:0000226,microtubule cytoskeleton organization|GO:1902425,positive regulation of attachment of mitotic spindle microtubules to kinetochore|GO:0007067,mitotic nuclear division|GO:0051225,spindle assembly|GO:0097659,nucleic acid-templated transcription|GO:1902423,regulation of mitotic attachment of spindle microtubules to kinetochore|GO:0090068,positive regulation of cell cycle process|GO:0007346,regulation of mitotic cell cycle|GO:0000381,regulation of alternative mRNA splicing, via spliceosome|GO:0000380,alternative mRNA splicing, via spliceosome|GO:0051345,positive regulation of hydrolase activity|GO:0043549,regulation of kinase activity|GO:0065003,macromolecular complex assembly|GO:0043085,positive regulation of catalytic activity|GO:2000648,positive regulation of stem cell proliferation|GO:0006366,transcription from RNA polymerase II promoter|GO:0006368,transcription elongation from RNA polymerase II	GO:0035770,ribonucleoprotein granule|GO:0031974,membrane-enclosed lumen|GO:0043229,intracellular organelle|GO:0043228,non-membrane-bounded organelle|GO:0036464,cytoplasmic ribonucleoprotein granule|GO:0070937,CRD-mediated mRNA stability complex|GO:0043227,membrane-bounded organelle|GO:0043226,organelle|GO:0005737,cytoplasm|GO:0031981,nuclear lumen|GO:0005634,nucleus|GO:0016020,membrane|GO:0030529,intracellular ribonucleoprotein complex|GO:0005654,nucleoplasm|GO:0009986,cell surface|GO:0071013,catalytic step 2 spliceosome|GO:0043234,protein complex|GO:0032991,macromolecular complex|GO:0043231,intracellular membrane-bounded organelle|GO:0043232,intracellular non-membrane-bounded organelle|GO:0043233,organelle lumen|GO:0044464,cell part|GO:0005623,cell|GO:0005622,intracellular|GO:0044446,intracellular organelle part|GO:0070013,intracellular organelle lumen|GO:0044444,cytoplasmic part|GO:0044428,nuclear part|GO:0044422,organelle part|GO:0044424,intracellular part|GO:0005681,spliceosomal complex|GO:0031012,extracellular matrix|GO:0005697,telomerase holoenzyme complex|GO:1990904,ribonucleoprotein complex|GO:0005576,extracellular region|GO:0044421,extracellular region part|GO:0098687,chromosomal region|GO:0099512,supramolecular fiber|GO:0099513,polymeric cytoskeletal fiber|GO:0098577,inactive sex chromosome|GO:0000228,nuclear chromosome|GO:0005815,microtubule organizing center|GO:0005874,microtubule|GO:1990498,mitotic spindle microtubule|GO:0034399,nuclear periphery|GO:0016604,nuclear body|GO:0005813,centrosome|GO:0000803,sex chromosome|GO:0000922,spindle pole|GO:0044430,cytoskeletal part|GO:0044451,nucleoplasm part|GO:0005876,spindle microtubule|GO:0016363,nuclear matrix|GO:0005694,chromosome|GO:0016607,nuclear speck|GO:0015630,microtubule cytoskeleton|GO:1990023,mitotic spindle midzone|GO:0000779,condensed chromosome, centromeric region|GO:0072686,mitotic spindle|GO:0051233,spindle midzone|GO:0000775,chromosome, centromeric region|GO:0000776,kinetochore|GO:0000777,condensed chromosome kinetochore|GO:0000793,condensed chromosome|GO:0005856,cytoskeleton|GO:0030496,midbody|GO:0044427,chromosomal part|GO:0005819,spindle	GO:0035639,purine ribonucleoside triphosphate binding|GO:0005488,binding|GO:0003676,nucleic acid binding|GO:0003677,DNA binding|GO:0043167,ion binding|GO:1901363,heterocyclic compound binding|GO:1901265,nucleoside phosphate binding|GO:0001882,nucleoside binding|GO:0043168,anion binding|GO:0000166,nucleotide binding|GO:0001883,purine nucleoside binding|GO:0032549,ribonucleoside binding|GO:0017076,purine nucleotide binding|GO:0005524,ATP binding|GO:0044822,poly(A) RNA binding|GO:0001067,regulatory region nucleic acid binding|GO:0036094,small molecule binding|GO:0001047,core promoter binding|GO:0044212,transcription regulatory region DNA binding|GO:0030554,adenyl nucleotide binding|GO:0097367,carbohydrate derivative binding|GO:0097159,organic cyclic compound binding|GO:0032559,adenyl ribonucleotide binding|GO:0003723,RNA binding|GO:0005515,protein binding|GO:0000975,regulatory region DNA binding|GO:0032555,purine ribonucleotide binding|GO:0032550,purine ribonucleoside binding|GO:0032553,ribonucleotide binding|GO:0070034,telomerase RNA binding|GO:0008143,poly(A) binding|GO:0001012,RNA polymerase II regulatory region DNA binding|GO:0001159,core promoter proximal region DNA binding|GO:0031490,chromatin DNA binding|GO:0034046,poly(G) binding|GO:0044877,macromolecular complex binding|GO:0008187,poly-pyrimidine tract binding|GO:0036002,pre-mRNA binding|GO:1990837,sequence-specific double-stranded DNA binding|GO:0070063,RNA polymerase binding|GO:0017069,snRNA binding|GO:0003714,transcription corepressor activity|GO:0003712,transcription cofactor activity|GO:0001098,basal transcription machinery binding|GO:0001099,basal RNA polymerase II transcription machinery binding|GO:0003779,actin binding|GO:0003730,mRNA 3'-UTR binding|GO:0000989,transcription factor activity, transcription factor binding|GO:0000988,transcription factor activity, protein binding|GO:0000987,core promoter proximal region sequence-specific DNA binding|GO:0003690,double-stranded DNA binding|GO:0003697,single-stranded DNA binding|GO:0042802,identical protein binding|GO:0043565,sequence-specific DNA binding|GO:0017130,poly(C) RNA binding|GO:0008134,transcription factor binding|GO:0043021,ribonucleoprotein complex binding|GO:0019899,enzyme binding|GO:1990841,promoter-specific chromatin binding|GO:0001085,RNA polymerase II transcription factor binding|GO:0003682,chromatin binding|GO:0008092,cytoskeletal protein binding|GO:0001097,TFIIH-class transcription factor binding|GO:0070717,poly-purine tract binding|GO:0001091,RNA polymerase II basal transcription factor binding|GO:0032403,protein complex binding|GO:0003727,single-stranded RNA binding|GO:0003725,double-stranded RNA binding|GO:0000976,transcription regulatory region sequence-specific DNA binding|GO:0000977,RNA
HIST1H2A	GO:0009892,negative regulation of metabolic process|GO:0080090,regulation of primary metabolic process|GO:0009890,negative regulation of biosynthetic process|GO:0031327,negative regulation of cellular biosynthetic process|GO:0031326,regulation of cellular biosynthetic process|GO:0031324,negative regulation of cellular metabolic process|GO:0031323,regulation of cellular metabolic process|GO:0040029,regulation of gene expression, epigenetic|GO:0090304,nucleic acid metabolic process|GO:0044249,cellular biosynthetic process|GO:0010629,negative regulation of gene expression|GO:0006807,nitrogen compound metabolic process|GO:0034645,cellular macromolecule biosynthetic process|GO:0010467,gene expression|GO:1901362,organic cyclic compound biosynthetic process|GO:0050789,regulation of biological process|GO:0071840,cellular component organization or biogenesis|GO:0032774,RNA biosynthetic process|GO:0006139,nucleobase-containing compound metabolic process|GO:0010605,negative regulation of macromolecule metabolic process|GO:0019222,regulation of metabolic process|GO:0044260,cellular macromolecule metabolic process|GO:0071704,organic substance metabolic process|GO:0006325,chromatin organization|GO:0006342,chromatin silencing|GO:1901360,organic cyclic compound metabolic process|GO:2000112,regulation of cellular macromolecule biosynthetic process|GO:2000113,negative regulation of cellular macromolecule biosynthetic process|GO:0016043,cellular component organization|GO:0019219,regulation of nucleobase-containing compound metabolic process|GO:0016458,gene silencing|GO:0065007,biological regulation|GO:0044699,single-organism process|GO:0048519,negative regulation of biological process|GO:0045814,negative regulation of gene expression, epigenetic|GO:0010468,regulation of gene expression|GO:0018130,heterocycle biosynthetic process|GO:0016070,RNA metabolic process|GO:0045934,negative regulation of nucleobase-containing compound metabolic process|GO:1901576,organic substance biosynthetic process|GO:0060255,regulation of macromolecule metabolic process|GO:0006725,cellular aromatic compound metabolic process|GO:0009987,cellular process|GO:0009889,regulation of biosynthetic process|GO:1903506,regulation of nucleic acid-templated transcription|GO:1903507,negative regulation of nucleic acid-templated transcription|GO:1902679,negative regulation of RNA biosynthetic process|GO:0009058,biosynthetic process|GO:0009059,macromolecule biosynthetic process|GO:0045892,negative regulation of transcription, DNA-templated|GO:0051171,regulation of nitrogen compound metabolic process|GO:0051172,negative regulation of nitrogen compound metabolic process|GO:2001141,regulation of RNA biosynthetic process|GO:0034654,nucleobase-containing compound biosynthetic process|GO:0050794,regulation of cellular process|GO:0008152,metabolic process|GO:0046483,heterocycle metabolic process|GO:0006996,organelle organization|GO:0044238,primary metabolic process|GO:0044271,cellular nitrogen compound biosynthetic process|GO:0051276,chromosome organization|GO:0019438,aromatic compound biosynthetic process|GO:0051253,negative regulation of RNA metabolic process|GO:0051252,regulation of RNA metabolic process|GO:0034641,cellular nitrogen compound metabolic process|GO:0044237,cellular metabolic process|GO:0043170,macromolecule metabolic process|GO:0006355,regulation of transcription, DNA-templated|GO:0010556,regulation of macromolecule biosynthetic process|GO:0006351,transcription, DNA-templated|GO:0044763,single-organism cellular process|GO:0097659,nucleic acid-templated transcription|GO:0010558,negative regulation of macromolecule biosynthetic process|GO:0048523,negative regulation of cellular process	GO:0044815,DNA packaging complex|GO:0043229,intracellular organelle|GO:0043228,non-membrane-bounded organelle|GO:0000785,chromatin|GO:0000786,nucleosome|GO:0043227,membrane-bounded organelle|GO:0043226,organelle|GO:0031982,vesicle|GO:0005634,nucleus|GO:0031988,membrane-bounded vesicle|GO:0005694,chromosome|GO:0032991,macromolecular complex|GO:1903561,extracellular vesicle|GO:0070062,extracellular exosome|GO:0043234,protein complex|GO:0043230,extracellular organelle|GO:0043231,intracellular membrane-bounded organelle|GO:0043232,intracellular non-membrane-bounded organelle|GO:0044464,cell part|GO:0005623,cell|GO:0005622,intracellular|GO:0044446,intracellular organelle part|GO:0005576,extracellular region|GO:0044424,intracellular part|GO:0032993,protein-DNA complex|GO:0044427,chromosomal part|GO:0065010,extracellular membrane-bounded organelle|GO:0044421,extracellular region part|GO:0044422,organelle part|GO:0031974,membrane-enclosed lumen|GO:0000228,nuclear chromosome|GO:0031981,nuclear lumen|GO:0044454,nuclear chromosome part|GO:0000790,nuclear chromatin|GO:0043233,organelle lumen|GO:0070013,intracellular organelle lumen|GO:0044428,nuclear part	GO:0005515,protein binding|GO:0097159,organic cyclic compound binding|GO:0019899,enzyme binding|GO:0005488,binding|GO:0003676,nucleic acid binding|GO:0003677,DNA binding|GO:0046983,protein dimerization activity|GO:0046982,protein heterodimerization activity|GO:1901363,heterocyclic compound binding
C4orf22			
C8orf22			
CCDC148			GO:0005488,binding|GO:0005515,protein binding
CCDC39	GO:0072359,circulatory system development|GO:0072358,cardiovascular system development|GO:0003341,cilium movement|GO:0071840,cellular component organization or biogenesis|GO:0048869,cellular developmental process|GO:0007368,determination of left/right symmetry|GO:0048513,animal organ development|GO:0060541,respiratory system development|GO:0003007,heart morphogenesis|GO:0003356,regulation of cilium beat frequency|GO:0010927,cellular component assembly involved in morphogenesis|GO:0003352,regulation of cilium movement|GO:0003351,epithelial cilium movement|GO:0071910,determination of liver left/right asymmetry|GO:0044707,single-multicellular organism process|GO:0048870,cell motility|GO:0044782,cilium organization|GO:0036159,inner dynein arm assembly|GO:0048562,embryonic organ morphogenesis|GO:0048565,digestive tract development|GO:0061008,hepaticobiliary system development|GO:0048568,embryonic organ development|GO:0006928,movement of cell or subcellular component|GO:0051674,localization of cell|GO:0000226,microtubule cytoskeleton organization|GO:0071907,determination of digestive tract left/right asymmetry|GO:0050789,regulation of biological process|GO:0009653,anatomical structure morphogenesis|GO:0000902,cell morphogenesis|GO:0035082,axoneme assembly|GO:0016043,cellular component organization|GO:0065003,macromolecular complex assembly|GO:0065007,biological regulation|GO:0048646,anatomical structure formation involved in morphogenesis|GO:0009887,animal organ morphogenesis|GO:0042384,cilium assembly|GO:0060271,cilium morphogenesis|GO:0009888,tissue development|GO:0050794,regulation of cellular process|GO:0001578,microtubule bundle formation|GO:0044763,single-organism cellular process|GO:0070271,protein complex biogenesis|GO:0031016,pancreas development|GO:0060287,epithelial cilium movement involved in determination of left/right asymmetry|GO:0055123,digestive system development|GO:0009790,embryo development|GO:0022607,cellular component assembly|GO:0009799,specification of symmetry|GO:0032886,regulation of microtubule-based process|GO:0060285,cilium-dependent cell motility|GO:0044699,single-organism process|GO:0070286,axonemal dynein complex assembly|GO:0001947,heart looping|GO:0043933,macromolecular complex subunit organization|GO:0060562,epithelial tube morphogenesis|GO:0061371,determination of heart left/right asymmetry|GO:0044458,motile cilium assembly|GO:0032502,developmental process|GO:0040011,locomotion|GO:0032501,multicellular organismal process|GO:0035239,tube morphogenesis|GO:0003143,embryonic heart tube morphogenesis|GO:0030317,flagellated sperm motility|GO:0060429,epithelium development|GO:0051270,regulation of cellular component movement|GO:0032879,regulation of localization|GO:0048858,cell projection morphogenesis|GO:0048731,system development|GO:0048732,gland development|GO:0030323,respiratory tube development|GO:0030324,lung development|GO:0030030,cell projection organization|GO:0030031,cell projection assembly|GO:0060632,regulation of microtubule-based movement|GO:0001889,liver development|GO:0034622,cellular macromolecular complex assembly|GO:0007275,multicellular organism development|GO:0007389,pattern specification process|GO:0071822,protein complex subunit organization|GO:0032989,cellular component morphogenesis|GO:0048729,tissue morphogenesis|GO:0048598,embryonic morphogenesis|GO:0009987,cellular process|GO:0002009,morphogenesis of an epithelium|GO:0006461,protein complex assembly|GO:0035050,embryonic heart tube development|GO:0044767,single-organism developmental process|GO:0001539,cilium or flagellum-dependent cell motility|GO:0035469,determination of pancreatic left/right asymmetry|GO:0035295,tube development|GO:0070925,organelle assembly|GO:0043623,cellular protein complex assembly|GO:0051179,localization|GO:0006996,organelle organization|GO:0007507,heart development|GO:0007017,microtubule-based process|GO:0007010,cytoskeleton organization|GO:0032990,cell part morphogenesis|GO:0048856,anatomical structure development|GO:0007018,microtubule-based movement|GO:1902589,single-organism organelle organization|GO:0044085,cellular component	GO:0005856,cytoskeleton|GO:0005737,cytoplasm|GO:0044463,cell projection part|GO:0032838,cell projection cytoplasm|GO:0043232,intracellular non-membrane-bounded organelle|GO:0005929,cilium|GO:0044464,cell part|GO:0044444,cytoplasmic part|GO:0005623,cell|GO:0043226,organelle|GO:0043229,intracellular organelle|GO:0043228,non-membrane-bounded organelle|GO:0097014,ciliary plasm|GO:0044441,ciliary part|GO:0044424,intracellular part|GO:0042995,cell projection|GO:0005930,axoneme|GO:0044422,organelle part|GO:0005622,intracellular|GO:0044446,intracellular organelle part|GO:0044430,cytoskeletal part	
AC069547	GO:2001234,negative regulation of apoptotic signaling pathway|GO:2001236,regulation of extrinsic apoptotic signaling pathway|GO:2001237,negative regulation of extrinsic apoptotic signaling pathway|GO:2001233,regulation of apoptotic signaling pathway|GO:0048585,negative regulation of response to stimulus|GO:0048583,regulation of response to stimulus|GO:0097191,extrinsic apoptotic signaling pathway|GO:0023057,negative regulation of signaling|GO:0023052,signaling|GO:0007165,signal transduction|GO:0007166,cell surface receptor signaling pathway|GO:0023051,regulation of signaling|GO:0008219,cell death|GO:0010646,regulation of cell communication|GO:0042981,regulation of apoptotic process|GO:0050789,regulation of biological process|GO:0044699,single-organism process|GO:0051716,cellular response to stimulus|GO:0010941,regulation of cell death|GO:0009968,negative regulation of signal transduction|GO:0009966,regulation of signal transduction|GO:0043067,regulation of programmed cell death|GO:0043066,negative regulation of apoptotic process|GO:0065007,biological regulation|GO:0048519,negative regulation of biological process|GO:0097190,apoptotic signaling pathway|GO:0043069,negative regulation of programmed cell death|GO:0016265,obsolete death|GO:0009987,cellular process|GO:0060548,negative regulation of cell death|GO:0006915,apoptotic process|GO:0050794,regulation of cellular process|GO:0012501,programmed cell death|GO:0044763,single-organism cellular process|GO:0007154,cell communication|GO:0044700,single organism signaling|GO:0050896,response to stimulus|GO:0048523,negative regulation of cellular process|GO:0010648,negative regulation of cell communication|GO:0019222,regulation of metabolic process|GO:0044710,single-organism metabolic process|GO:0044711,single-organism biosynthetic process|GO:0010604,positive regulation of macromolecule metabolic process|GO:0009617,response to bacterium|GO:0048518,positive regulation of biological process|GO:0051704,multi-organism process|GO:0006665,sphingolipid metabolic process|GO:0060255,regulation of macromolecule metabolic process|GO:0042221,response to chemical|GO:0009607,response to biotic stimulus|GO:0051707,response to other organism|GO:0010033,response to organic substance|GO:0010467,gene expression|GO:1901564,organonitrogen compound metabolic process|GO:0009605,response to external stimulus|GO:1901566,organonitrogen compound biosynthetic process|GO:0044249,cellular biosynthetic process|GO:0006672,ceramide metabolic process|GO:0009893,positive regulation of metabolic process|GO:0010628,positive regulation of gene expression|GO:0032496,response to lipopolysaccharide|GO:0034097,response to cytokine|GO:1901576,organic substance biosynthetic process|GO:0016049,cell growth|GO:0071356,cellular response to tumor necrosis factor|GO:0071219,cellular response to molecule of bacterial origin|GO:0071216,cellular response to biotic stimulus|GO:0030148,sphingolipid biosynthetic process|GO:0006629,lipid metabolic process|GO:0006952,defense response|GO:0006950,response to stress|GO:0008152,metabolic process|GO:0044271,cellular nitrogen compound biosynthetic process|GO:0043207,response to external biotic stimulus|GO:0071345,cellular response to cytokine stimulus|GO:0006954,inflammatory response|GO:0034641,cellular nitrogen compound metabolic process|GO:0070887,cellular response to chemical stimulus|GO:0046467,membrane lipid biosynthetic process|GO:0006643,membrane lipid metabolic process|GO:0044238,primary metabolic process|GO:0071396,cellular response to lipid|GO:0044255,cellular lipid metabolic process|GO:0043604,amide biosynthetic process|GO:0043603,cellular amide metabolic process|GO:0043170,macromolecule metabolic process|GO:0006807,nitrogen compound metabolic process|GO:0002237,response to molecule of bacterial origin|GO:0071222,cellular response to lipopolysaccharide|GO:0040007,growth|GO:0033993,response to lipid|GO:0071704,organic substance metabolic process|GO:0071310,cellular response to organic substance|GO:0010468,regulation of gene expression|GO:0034612,response to tumor necrosis factor|GO:0009058,biosynthetic process|GO:0008610,lipid biosynthetic process|GO:1901700,response to oxygen-containing compound|GO:1901701,cellular response to oxygen-containing compound|GO:0044237,cellular metabolic process|GO:0046513,ceramide	GO:0044425,membrane part|GO:0016021,integral component of membrane|GO:0016020,membrane|GO:0031224,intrinsic component of membrane	GO:0016780,phosphotransferase activity, for other substituted phosphate groups|GO:0016772,transferase activity, transferring phosphorus-containing groups|GO:0003824,catalytic activity|GO:0016740,transferase activity|GO:0002950,ceramide phosphoethanolamine synthase activity|GO:0033188,sphingomyelin synthase activity
LIPT1	GO:0044267,cellular protein metabolic process|GO:0044710,single-organism metabolic process|GO:0006629,lipid metabolic process|GO:0044260,cellular macromolecule metabolic process|GO:0044238,primary metabolic process|GO:0019538,protein metabolic process|GO:0009987,cellular process|GO:0018065,protein-cofactor linkage|GO:0009249,protein lipoylation|GO:0006464,cellular protein modification process|GO:0043170,macromolecule metabolic process|GO:0071704,organic substance metabolic process|GO:0043412,macromolecule modification|GO:0036211,protein modification process|GO:0044237,cellular metabolic process|GO:0008152,metabolic process|GO:0044699,single-organism process|GO:1901564,organonitrogen compound metabolic process|GO:0006807,nitrogen compound metabolic process|GO:0034641,cellular nitrogen compound metabolic process	GO:0005737,cytoplasm|GO:0043231,intracellular membrane-bounded organelle|GO:0044464,cell part|GO:0043229,intracellular organelle|GO:0005739,mitochondrion|GO:0005622,intracellular|GO:0044444,cytoplasmic part|GO:0005623,cell|GO:0044424,intracellular part|GO:0043227,membrane-bounded organelle|GO:0043226,organelle|GO:0043233,organelle lumen|GO:0031974,membrane-enclosed lumen|GO:0044446,intracellular organelle part|GO:0070013,intracellular organelle lumen|GO:0044429,mitochondrial part|GO:0005759,mitochondrial matrix|GO:0044422,organelle part	GO:0003824,catalytic activity|GO:0016740,transferase activity|GO:0016746,transferase activity, transferring acyl groups|GO:0016979,lipoate-protein ligase activity|GO:0016879,ligase activity, forming carbon-nitrogen bonds|GO:0016874,ligase activity
MS4A12	GO:0044699,single-organism process|GO:0006810,transport|GO:0070838,divalent metal ion transport|GO:0051234,establishment of localization|GO:0072511,divalent inorganic cation transport|GO:0006811,ion transport|GO:0009987,cellular process|GO:0070588,calcium ion transmembrane transport|GO:0006816,calcium ion transport|GO:0051179,localization|GO:0034220,ion transmembrane transport|GO:0044765,single-organism transport|GO:0044763,single-organism cellular process|GO:0098662,inorganic cation transmembrane transport|GO:0098660,inorganic ion transmembrane transport|GO:0030001,metal ion transport|GO:0006812,cation transport|GO:0098655,cation transmembrane transport|GO:0055085,transmembrane transport|GO:1902578,single-organism localization	GO:0044425,membrane part|GO:0016021,integral component of membrane|GO:0016020,membrane|GO:0031224,intrinsic component of membrane	GO:0005261,cation channel activity|GO:0005262,calcium channel activity|GO:0005488,binding|GO:0015085,calcium ion transmembrane transporter activity|GO:0015279,store-operated calcium channel activity|GO:0015276,ligand-gated ion channel activity|GO:0072509,divalent inorganic cation transmembrane transporter activity|GO:0022803,passive transmembrane transporter activity|GO:0046873,metal ion transmembrane transporter activity|GO:0005215,transporter activity|GO:0005216,ion channel activity|GO:0008324,cation transmembrane transporter activity|GO:0022891,substrate-specific transmembrane transporter activity|GO:0022890,inorganic cation transmembrane transporter activity|GO:0022892,substrate-specific transporter activity|GO:0015075,ion transmembrane transporter activity|GO:0022857,transmembrane transporter activity|GO:0015267,channel activity|GO:0022836,gated channel activity|GO:0005515,protein binding|GO:0022834,ligand-gated channel

RPL31	GO:0008104,protein localization|GO:0061024,membrane organization|GO:1901362,organic cyclic compound biosynthetic process|GO:1901360,organic cyclic compound metabolic process|GO:1901361,organic cyclic compound catabolic process|GO:0070727,cellular macromolecule localization|GO:0043043,peptide biosynthetic process|GO:0044419,interspecies interaction between organisms|GO:0019058,viral life cycle|GO:0033036,macromolecule localization|GO:0051704,multi-organism process|GO:0006605,protein targeting|GO:0045184,establishment of protein localization|GO:0072657,protein localization to membrane|GO:0044403,symbiosis, encompassing mutualism through parasitism|GO:0046483,heterocycle metabolic process|GO:1901564,organonitrogen compound metabolic process|GO:1901566,organonitrogen compound biosynthetic process|GO:0019538,protein metabolic process|GO:0019439,aromatic compound catabolic process|GO:0019438,aromatic compound biosynthetic process|GO:0019080,viral gene expression|GO:0006613,cotranslational protein targeting to membrane|GO:0006612,protein targeting to membrane|GO:0006614,SRP-dependent cotranslational protein targeting to membrane|GO:0006807,nitrogen compound metabolic process|GO:0044033,multi-organism metabolic process|GO:1901576,organic substance biosynthetic process|GO:1901575,organic substance catabolic process|GO:0044265,cellular macromolecule catabolic process|GO:0044260,cellular macromolecule metabolic process|GO:0006886,intracellular protein transport|GO:0016043,cellular component organization|GO:0071840,cellular component organization or biogenesis|GO:0018130,heterocycle biosynthetic process|GO:0006810,transport|GO:0046700,heterocycle catabolic process|GO:0044802,single-organism membrane organization|GO:0008152,metabolic process|GO:0034654,nucleobase-containing compound biosynthetic process|GO:0034655,nucleobase-containing compound catabolic process|GO:0051234,establishment of localization|GO:0016070,RNA metabolic process|GO:0016071,mRNA metabolic process|GO:0044271,cellular nitrogen compound biosynthetic process|GO:0044270,cellular nitrogen compound catabolic process|GO:0046907,intracellular transport|GO:0044765,single-organism transport|GO:0045047,protein targeting to ER|GO:0044764,multi-organism cellular process|GO:0022411,cellular component disassembly|GO:0006518,peptide metabolic process|GO:0032774,RNA biosynthetic process|GO:0044248,cellular catabolic process|GO:0044249,cellular biosynthetic process|GO:0034641,cellular nitrogen compound metabolic process|GO:0034645,cellular macromolecule biosynthetic process|GO:0043241,protein complex disassembly|GO:0044699,single-organism process|GO:0006139,nucleobase-containing compound metabolic process|GO:0070972,protein localization to endoplasmic reticulum|GO:0051179,localization|GO:0002181,cytoplasmic translation|GO:0019083,viral transcription|GO:0072599,establishment of protein localization to endoplasmic reticulum|GO:0072594,establishment of protein localization to organelle|GO:0009987,cellular process|GO:0006725,cellular aromatic compound metabolic process|GO:0016482,cytosolic transport|GO:0043604,amide biosynthetic process|GO:0043603,cellular amide metabolic process|GO:0043170,macromolecule metabolic process|GO:0000956,nuclear-transcribed mRNA catabolic process|GO:0033365,protein localization to organelle|GO:0000184,nuclear-transcribed mRNA catabolic process, nonsense-mediated decay|GO:0043933,macromolecular complex subunit organization|GO:0090304,nucleic acid metabolic process|GO:0032984,macromolecular complex disassembly|GO:0071822,protein complex subunit organization|GO:0071704,organic substance metabolic process|GO:0010467,gene expression|GO:0071702,organic substance transport|GO:0006401,RNA catabolic process|GO:0006402,mRNA catabolic process|GO:0044267,cellular protein metabolic process|GO:0034613,cellular protein localization|GO:0009058,biosynthetic process|GO:0009059,macromolecule biosynthetic process|GO:0044763,single-organism cellular process|GO:0051649,establishment of localization in cell|GO:0043624,cellular protein complex disassembly|GO:0009056,catabolic process|GO:0009057,macromolecule catabolic process|GO:1902578,single-organism localization|GO:0051641,cellular localization|GO:0044238,primary metabolic process|GO:1902580,single-organism cellular localization|GO:0090150,establishment of protein localization to membrane|GO:0044237,cellular metabolic process|GO:0016032,viral process|GO:0015031,protein transport|GO:1902582,single-organism intracellular transport|GO:0006415,translational termination|GO:0006414,translational elongation|GO:0006413,translational initiation|GO:0006412,translation|GO:0019222,regulation of metabolic process|GO:0010605,negative regulation of macromolecule metabolic process|GO:0048519,negative regulation of biological process|GO:0006364,rRNA processing|GO:0034470,ncRNA processing|GO:0060255,regulation of macromolecule metabolic process|GO:0009892,negative regulation of metabolic process|GO:0010629,negative regulation of gene expression|GO:0034660,ncRNA metabolic process|GO:0050789,regulation of biological process|GO:0042254,ribosome biogenesis|GO:0065007,biological regulation|GO:0016072,rRNA metabolic process|GO:0015833,peptide transport|GO:0042886,amide transport|GO:0022613,ribonucleoprotein complex biogenesis|GO:0071705,nitrogen compound transport|GO:0010468,regulation of gene	GO:0015934,large ribosomal subunit|GO:0022625,cytosolic large ribosomal subunit|GO:0043229,intracellular organelle|GO:0043228,non-membrane-bounded organelle|GO:0022626,cytosolic ribosome|GO:0005924,cell-substrate adherens junction|GO:0005925,focal adhesion|GO:0043227,membrane-bounded organelle|GO:0043226,organelle|GO:0030054,cell junction|GO:0030055,cell-substrate junction|GO:0005737,cytoplasm|GO:0031982,vesicle|GO:0016020,membrane|GO:0031988,membrane-bounded vesicle|GO:0005576,extracellular region|GO:0030529,intracellular ribonucleoprotein complex|GO:0070161,anchoring junction|GO:0044391,ribosomal subunit|GO:0005912,adherens junction|GO:0032991,macromolecular complex|GO:1903561,extracellular vesicle|GO:0070062,extracellular exosome|GO:0043230,extracellular organelle|GO:0005840,ribosome|GO:0043232,intracellular non-membrane-bounded organelle|GO:0005829,cytosol|GO:0044464,cell part|GO:0005623,cell|GO:0005622,intracellular|GO:0044446,intracellular organelle part|GO:0044444,cytoplasmic part|GO:0044445,cytosolic part|GO:0044424,intracellular part|GO:0065010,extracellular membrane-bounded organelle|GO:0044421,extracellular region part|GO:0044422,organelle part|GO:1990904,ribonucleoprotein complex|GO:0005844,polysome|GO:0042788,polysomal ribosome	GO:0097159,organic cyclic compound binding|GO:0044822,poly(A) RNA binding|GO:0005198,structural molecule activity|GO:0003735,structural constituent of ribosome|GO:0003723,RNA binding|GO:0003676,nucleic acid binding|GO:1901363,heterocyclic compound binding|GO:0005488,binding|GO:0005515,protein binding
KBTBD4	GO:0080090,regulation of primary metabolic process|GO:0019222,regulation of metabolic process|GO:0051246,regulation of protein metabolic process|GO:0044248,cellular catabolic process|GO:0006807,nitrogen compound metabolic process|GO:0043632,modification-dependent macromolecule catabolic process|GO:0050789,regulation of biological process|GO:0044267,cellular protein metabolic process|GO:1901575,organic substance catabolic process|GO:0044265,cellular macromolecule catabolic process|GO:0044260,cellular macromolecule metabolic process|GO:0071704,organic substance metabolic process|GO:0043161,proteasome-mediated ubiquitin-dependent protein catabolic process|GO:0070647,protein modification by small protein conjugation or removal|GO:0032446,protein modification by small protein conjugation|GO:0065007,biological regulation|GO:0042787,protein ubiquitination involved in ubiquitin-dependent protein catabolic process|GO:0044238,primary metabolic process|GO:0060255,regulation of macromolecule metabolic process|GO:0009987,cellular process|GO:0019941,modification-dependent protein catabolic process|GO:0006464,cellular protein modification process|GO:0043412,macromolecule modification|GO:0036211,protein modification process|GO:0030162,regulation of proteolysis|GO:0030163,protein catabolic process|GO:0008152,metabolic process|GO:0006508,proteolysis|GO:0044257,cellular protein catabolic process|GO:0009056,catabolic process|GO:0009057,macromolecule catabolic process|GO:0051603,proteolysis involved in cellular protein catabolic process|GO:1901564,organonitrogen compound metabolic process|GO:1901565,organonitrogen compound catabolic process|GO:0019538,protein metabolic process|GO:0010498,proteasomal protein catabolic process|GO:0044237,cellular metabolic process|GO:0043170,macromolecule metabolic process|GO:0016567,protein ubiquitination|GO:0006511,ubiquitin-dependent protein catabolic process	GO:0043234,protein complex|GO:0005737,cytoplasm|GO:0032991,macromolecular complex|GO:0043231,intracellular membrane-bounded organelle|GO:0005634,nucleus|GO:0044464,cell part|GO:0000151,ubiquitin ligase complex|GO:0005623,cell|GO:0005622,intracellular|GO:0043229,intracellular organelle|GO:0019005,SCF ubiquitin ligase complex|GO:1990234,transferase complex|GO:0044424,intracellular part|GO:0031461,cullin-RING ubiquitin ligase complex|GO:0043227,membrane-bounded organelle|GO:0043226,organelle|GO:1902494,catalytic complex	GO:0031625,ubiquitin protein ligase binding|GO:0044389,ubiquitin-like protein ligase binding|GO:0019899,enzyme binding|GO:0005488,binding|GO:0005515,protein binding
SLC1A6	GO:0089711,L-glutamate transmembrane transport|GO:0042391,regulation of membrane potential|GO:0089712,L-aspartate transmembrane transport|GO:0015813,L-glutamate transport|GO:0015810,aspartate transport|GO:0046717,acid secretion|GO:1903825,organic acid transmembrane transport|GO:0023052,signaling|GO:0001505,regulation of neurotransmitter levels|GO:0051641,cellular localization|GO:0044699,single-organism process|GO:0006835,dicarboxylic acid transport|GO:0071705,nitrogen compound transport|GO:0051649,establishment of localization in cell|GO:0051179,localization|GO:0044765,single-organism transport|GO:0065007,biological regulation|GO:0006810,transport|GO:0071702,organic substance transport|GO:0065008,regulation of biological quality|GO:0006811,ion transport|GO:0006865,amino acid transport|GO:0015849,organic acid transport|GO:1902475,L-alpha-amino acid transmembrane transport|GO:0009987,cellular process|GO:0015711,organic anion transport|GO:0023061,signal release|GO:0014047,glutamate secretion|GO:0034220,ion transmembrane transport|GO:0006820,anion transport|GO:0015807,L-amino acid transport|GO:0044763,single-organism cellular process|GO:0007269,neurotransmitter secretion|GO:0007268,chemical synaptic transmission|GO:0007267,cell-cell signaling|GO:0007154,cell communication|GO:0051234,establishment of localization|GO:0055085,transmembrane transport|GO:1902578,single-organism localization|GO:0046942,carboxylic acid transport|GO:0044700,single organism signaling|GO:0046903,secretion|GO:0070778,L-aspartate transport|GO:0003333,amino acid transmembrane transport|GO:0032940,secretion by cell|GO:0098656,anion transmembrane transport|GO:0015800,acidic amino acid transport|GO:0006836,neurotransmitter transport|GO:0015740,C4-dicarboxylate transport|GO:0089718,amino acid import across plasma membrane|GO:0098712,L-glutamate import across plasma membrane|GO:1905039,carboxylic acid transmembrane transport|GO:0098739,import across plasma membrane|GO:1903802,L-glutamate(1-) import into cell|GO:0051938,L-glutamate import|GO:1902837,amino acid import into cell|GO:0098657,import into cell|GO:1990123,L-glutamate import into cell|GO:0043090,amino acid import|GO:0043092,L-amino acid import|GO:0098916,anterograde trans-synaptic signaling|GO:0099536,synaptic signaling|GO:0099537,trans-synaptic signaling	GO:0043229,intracellular organelle|GO:0071944,cell periphery|GO:0031226,intrinsic component of plasma membrane|GO:0043226,organelle|GO:0031224,intrinsic component of membrane|GO:0005737,cytoplasm|GO:0043227,membrane-bounded organelle|GO:0016021,integral component of membrane|GO:0016020,membrane|GO:0044459,plasma membrane part|GO:0005794,Golgi apparatus|GO:0012505,endomembrane system|GO:0005887,integral component of plasma membrane|GO:0005886,plasma membrane|GO:0043231,intracellular membrane-bounded organelle|GO:0044464,cell part|GO:0005623,cell|GO:0005622,intracellular|GO:0044444,cytoplasmic part|GO:0044424,intracellular part|GO:0044425,membrane part|GO:0043228,non-membrane-bounded organelle|GO:0005856,cytoskeleton|GO:0045111,intermediate filament cytoskeleton|GO:0043232,intracellular non-membrane-bounded organelle	GO:0005342,organic acid transmembrane transporter activity|GO:0005343,organic acid:sodium symporter activity|GO:0015183,L-aspartate transmembrane transporter activity|GO:0015081,sodium ion transmembrane transporter activity|GO:0015556,C4-dicarboxylate transmembrane transporter activity|GO:0008509,anion transmembrane transporter activity|GO:0022857,transmembrane transporter activity|GO:0015370,solute:sodium symporter activity|GO:0005215,transporter activity|GO:0046873,metal ion transmembrane transporter activity|GO:0017153,sodium:dicarboxylate symporter activity|GO:0022804,active transmembrane transporter activity|GO:0015294,solute:cation symporter activity|GO:0015296,anion:cation symporter activity|GO:0015291,secondary active transmembrane transporter activity|GO:0015293,symporter activity|GO:0005310,dicarboxylic acid transmembrane transporter activity|GO:0005313,L-glutamate transmembrane transporter activity|GO:0008324,cation transmembrane transporter activity|GO:0046943,carboxylic acid transmembrane transporter activity|GO:0022891,substrate-specific transmembrane transporter activity|GO:0022890,inorganic cation transmembrane transporter activity|GO:0022892,substrate-specific transporter activity|GO:0008514,organic anion transmembrane transporter activity|GO:0015075,ion transmembrane transporter activity|GO:0015077,monovalent inorganic cation transmembrane transporter activity|GO:0015179,L-amino acid transmembrane transporter activity|GO:0015171,amino acid transmembrane transporter activity|GO:0015172,acidic amino acid transmembrane transporter activity|GO:0005314,high-affinity glutamate transmembrane transporter
USMG5		GO:0044464,cell part|GO:0031975,envelope|GO:0043229,intracellular organelle|GO:0031988,membrane-bounded vesicle|GO:0043227,membrane-bounded organelle|GO:0043226,organelle|GO:0031224,intrinsic component of membrane|GO:0005737,cytoplasm|GO:0031982,vesicle|GO:0098796,membrane protein complex|GO:0016021,integral component of membrane|GO:0016020,membrane|GO:0044444,cytoplasmic part|GO:0005739,mitochondrion|GO:0098800,inner mitochondrial membrane protein complex|GO:0044455,mitochondrial membrane part|GO:0005753,mitochondrial proton-transporting ATP synthase complex|GO:0031967,organelle envelope|GO:0031966,mitochondrial membrane|GO:0032991,macromolecular complex|GO:1903561,extracellular vesicle|GO:0031090,organelle membrane|GO:0043234,protein complex|GO:0043230,extracellular organelle|GO:0043231,intracellular membrane-bounded organelle|GO:0070062,extracellular exosome|GO:0019866,organelle inner membrane|GO:0045259,proton-transporting ATP synthase complex|GO:0005623,cell|GO:0005622,intracellular|GO:0044446,intracellular organelle part|GO:0005743,mitochondrial inner membrane|GO:0005740,mitochondrial envelope|GO:0005576,extracellular region|GO:0044429,mitochondrial part|GO:0044424,intracellular part|GO:0044425,membrane part|GO:0016469,proton-transporting two-sector ATPase complex|GO:0065010,extracellular membrane-bounded organelle|GO:0044421,extracellular region part|GO:0044422,organelle part|GO:0098798,mitochondrial protein complex	
STK36	GO:0008589,regulation of smoothened signaling pathway|GO:0080090,regulation of primary metabolic process|GO:0019222,regulation of metabolic process|GO:0048584,positive regulation of response to stimulus|GO:0048583,regulation of response to stimulus|GO:0060322,head development|GO:0007165,signal transduction|GO:0007166,cell surface receptor signaling pathway|GO:0003341,cilium movement|GO:1901362,organic cyclic compound biosynthetic process|GO:0071840,cellular component organization or biogenesis|GO:0051716,cellular response to stimulus|GO:0010604,positive regulation of macromolecule metabolic process|GO:0009966,regulation of signal transduction|GO:0009967,positive regulation of signal transduction|GO:0048513,animal organ development|GO:0044093,positive regulation of molecular function|GO:0048518,positive regulation of biological process|GO:0044700,single organism signaling|GO:0060255,regulation of macromolecule metabolic process|GO:2001141,regulation of RNA biosynthetic process|GO:0003351,epithelial cilium movement|GO:0046483,heterocycle metabolic process|GO:0009791,post-embryonic development|GO:0044707,single-multicellular organism process|GO:0019538,protein metabolic process|GO:0019438,aromatic compound biosynthetic process|GO:0044782,cilium organization|GO:0022607,cellular component assembly|GO:0009893,positive regulation of metabolic process|GO:0009891,positive regulation of biosynthetic process|GO:0006928,movement of cell or subcellular component|GO:0010628,positive regulation of gene expression|GO:0035556,intracellular signal transduction|GO:0050789,regulation of biological process|GO:0097659,nucleic acid-templated transcription|GO:0044267,cellular protein metabolic process|GO:0009653,anatomical structure morphogenesis|GO:0000902,cell morphogenesis|GO:0044260,cellular macromolecule metabolic process|GO:0016043,cellular component organization|GO:0065007,biological regulation|GO:1901360,organic cyclic compound metabolic process|GO:0065009,regulation of molecular function|GO:0048646,anatomical structure formation involved in morphogenesis|GO:0018130,heterocycle biosynthetic process|GO:0042384,cilium assembly|GO:0060271,cilium morphogenesis|GO:0009889,regulation of biosynthetic process|GO:0050794,regulation of cellular process|GO:0043412,macromolecule modification|GO:0036211,protein modification process|GO:0008152,metabolic process|GO:0034654,nucleobase-containing compound biosynthetic process|GO:0016070,RNA metabolic process|GO:0007227,signal transduction downstream of smoothened|GO:0007420,brain development|GO:0007224,smoothened signaling pathway|GO:0050896,response to stimulus|GO:0007228,positive regulation of hh target transcription factor activity|GO:0006355,regulation of transcription, DNA-templated|GO:0010557,positive regulation of macromolecule biosynthetic process|GO:0010556,regulation of macromolecule biosynthetic process|GO:0006351,transcription, DNA-templated|GO:0048869,cellular developmental process|GO:0032774,RNA biosynthetic process|GO:0016310,phosphorylation|GO:0010927,cellular component assembly involved in morphogenesis|GO:0023056,positive regulation of signaling|GO:0044249,cellular biosynthetic process|GO:0034641,cellular nitrogen compound metabolic process|GO:0023052,signaling|GO:0034645,cellular macromolecule biosynthetic process|GO:0023051,regulation of signaling|GO:0010647,positive regulation of cell communication|GO:0010646,regulation of cell communication|GO:0044699,single-organism process|GO:0007417,central nervous system development|GO:0006139,nucleobase-containing compound metabolic process|GO:0030031,cell projection assembly|GO:1903508,positive regulation of nucleic acid-templated transcription|GO:0032501,multicellular organismal process|GO:0009987,cellular process|GO:0006725,cellular aromatic compound metabolic process|GO:1903506,regulation of nucleic acid-templated transcription|GO:0045893,positive regulation of transcription, DNA-templated|GO:0044271,cellular nitrogen compound biosynthetic process|GO:0048858,cell projection morphogenesis|GO:0051091,positive regulation of sequence-specific DNA binding transcription factor activity|GO:0051090,regulation of sequence-specific DNA binding transcription factor activity|GO:0051252,regulation of RNA metabolic process|GO:0051254,positive regulation of RNA metabolic process|GO:0043170,macromolecule metabolic process|GO:1902680,positive regulation of RNA biosynthetic process|GO:0006807,nitrogen compound metabolic process|GO:0048731,system development|GO:0032502,developmental process|GO:0031328,positive regulation of cellular biosynthetic process|GO:0030030,cell projection organization|GO:0031326,regulation of cellular biosynthetic process|GO:0031325,positive regulation of cellular metabolic process|GO:0031323,regulation of cellular metabolic process|GO:0090304,nucleic acid metabolic process|GO:0045880,positive regulation of smoothened signaling pathway|GO:0007275,multicellular organism development|GO:2000112,regulation of cellular macromolecule biosynthetic process|GO:0006796,phosphate-containing compound metabolic process|GO:0032989,cellular component morphogenesis|GO:0071704,organic substance metabolic process|GO:0010467,gene expression|GO:0010468,regulation of gene expression|GO:0006468,protein phosphorylation|GO:0045935,positive regulation of nucleobase-containing compound metabolic process|GO:1901576,organic substance biosynthetic process|GO:0019219,regulation of nucleobase-containing compound metabolic process|GO:0006464,cellular protein modification process|GO:0044767,single-organism developmental process|GO:0009058,biosynthetic process|GO:0009059,macromolecule biosynthetic process|GO:0044763,single-organism cellular process|GO:0051171,regulation of nitrogen compound metabolic process|GO:0051173,positive regulation of nitrogen compound metabolic process|GO:0007154,cell communication|GO:0070925,organelle assembly|GO:0006996,organelle organization|GO:0044238,primary metabolic process|GO:0007017,microtubule-based process|GO:0032990,cell part morphogenesis|GO:0007399,nervous system development|GO:0048856,anatomical structure development|GO:0007018,microtubule-based movement|GO:0044237,cellular metabolic process|GO:1902589,single-organism organelle organization|GO:0044085,cellular component biogenesis|GO:0006793,phosphorus	GO:0005737,cytoplasm|GO:0043231,intracellular membrane-bounded organelle|GO:0005634,nucleus|GO:0044464,cell part|GO:0005623,cell|GO:0005622,intracellular|GO:0043229,intracellular organelle|GO:0044424,intracellular part|GO:0043227,membrane-bounded organelle|GO:0043226,organelle|GO:0005829,cytosol|GO:0044444,cytoplasmic part	GO:0035639,purine ribonucleoside triphosphate binding|GO:0004672,protein kinase activity|GO:0043167,ion binding|GO:1901363,heterocyclic compound binding|GO:0001883,purine nucleoside binding|GO:0001882,nucleoside binding|GO:0043168,anion binding|GO:0043169,cation binding|GO:0000287,magnesium ion binding|GO:0000166,nucleotide binding|GO:0004674,protein serine/threonine kinase activity|GO:0016740,transferase activity|GO:1901265,nucleoside phosphate binding|GO:0032549,ribonucleoside binding|GO:0017076,purine nucleotide binding|GO:0046872,metal ion binding|GO:0005524,ATP binding|GO:0008134,transcription factor binding|GO:0016301,kinase activity|GO:0036094,small molecule binding|GO:0003824,catalytic activity|GO:0030554,adenyl nucleotide binding|GO:0016773,phosphotransferase activity, alcohol group as acceptor|GO:0016772,transferase activity, transferring phosphorus-containing groups|GO:0097367,carbohydrate derivative binding|GO:0032559,adenyl ribonucleotide binding|GO:0005515,protein binding|GO:0097159,organic cyclic compound binding|GO:0032555,purine ribonucleotide binding|GO:0032550,purine ribonucleoside binding|GO:0032553,ribonucleotide binding|GO:0005488,binding
C21orf67		GO:0005737,cytoplasm|GO:0005783,endoplasmic reticulum|GO:0044464,cell part|GO:0043229,intracellular organelle|GO:0005623,cell|GO:0005622,intracellular|GO:0044444,cytoplasmic part|GO:0012505,endomembrane system|GO:0044424,intracellular part|GO:0043227,membrane-bounded organelle|GO:0043226,organelle|GO:0043231,intracellular membrane-bounded organelle	
FASLG	GO:2001235,positive regulation of apoptotic signaling pathway|GO:2001236,regulation of extrinsic apoptotic signaling pathway|GO:0080090,regulation of primary metabolic process|GO:0019222,regulation of metabolic process|GO:2001233,regulation of apoptotic signaling pathway|GO:0048584,positive regulation of response to stimulus|GO:0048583,regulation of response to stimulus|GO:0045765,regulation of angiogenesis|GO:0016485,protein processing|GO:0072359,circulatory system development|GO:0043523,regulation of neuron apoptotic process|GO:0007165,signal transduction|GO:0007166,cell surface receptor signaling pathway|GO:0007167,enzyme linked receptor protein signaling pathway|GO:0098771,inorganic ion homeostasis|GO:0007169,transmembrane receptor protein tyrosine kinase signaling pathway|GO:1901362,organic cyclic compound biosynthetic process|GO:0071840,cellular component organization or biogenesis|GO:0032774,RNA biosynthetic process|GO:0043065,positive regulation of apoptotic process|GO:0051716,cellular response to stimulus|GO:0010605,negative regulation of macromolecule metabolic process|GO:0010604,positive regulation of macromolecule metabolic process|GO:0043207,response to external biotic stimulus|GO:0009966,regulation of signal transduction|GO:0009967,positive regulation of signal transduction|GO:0009617,response to bacterium|GO:0010256,endomembrane system organization|GO:0010467,gene expression|GO:0019438,aromatic compound biosynthetic process|GO:0043066,negative regulation of apoptotic process|GO:0048514,blood vessel morphogenesis|GO:0044093,positive regulation of molecular function|GO:0044092,negative regulation of molecular function|GO:0043281,regulation of cysteine-type endopeptidase activity involved in apoptotic process|GO:0043280,positive regulation of cysteine-type endopeptidase activity involved in apoptotic process|GO:0051704,multi-organism process|GO:0043122,regulation of I-kappaB kinase/NF-kappaB signaling|GO:0043123,positive regulation of I-kappaB kinase/NF-kappaB signaling|GO:0042127,regulation of cell proliferation|GO:0007032,endosome organization|GO:0070231,T cell apoptotic process|GO:0060255,regulation of macromolecule metabolic process|GO:0060548,negative regulation of cell death|GO:0070227,lymphocyte apoptotic process|GO:0042221,response to chemical|GO:0030162,regulation of proteolysis|GO:2001141,regulation of RNA biosynthetic process|GO:0051707,response to other organism|GO:0010033,response to organic substance|GO:0046483,heterocycle metabolic process|GO:0044700,single organism signaling|GO:0009607,response to biotic stimulus|GO:0009605,response to external stimulus|GO:0044707,single-multicellular organism process|GO:0048388,endosomal lumen acidification|GO:0019538,protein metabolic process|GO:0030644,cellular chloride ion homeostasis|GO:0043068,positive regulation of programmed cell death|GO:0002376,immune system process|GO:0007154,cell communication|GO:0072358,cardiovascular system development|GO:0097285,obsolete cell-type specific apoptotic process|GO:0043525,positive regulation of neuron apoptotic process|GO:0006925,inflammatory cell apoptotic process|GO:0009892,negative regulation of metabolic process|GO:0009893,positive regulation of metabolic process|GO:0009890,negative regulation of biosynthetic process|GO:0022603,regulation of anatomical structure morphogenesis|GO:0008152,metabolic process|GO:0010629,negative regulation of gene expression|GO:1901343,negative regulation of vasculature development|GO:1901186,positive regulation of ERBB signaling pathway|GO:1901184,regulation of ERBB signaling pathway|GO:0048878,chemical homeostasis|GO:0042981,regulation of apoptotic process|GO:0050789,regulation of biological process|GO:0097659,nucleic acid-templated transcription|GO:0044267,cellular protein metabolic process|GO:0009653,anatomical structure morphogenesis|GO:0051346,negative regulation of hydrolase activity|GO:0051345,positive regulation of hydrolase activity|GO:0070997,neuron death|GO:0006885,regulation of pH|GO:0001568,blood vessel development|GO:0006919,activation of cysteine-type endopeptidase activity involved in apoptotic process|GO:0006955,immune response|GO:0016043,cellular component organization|GO:0052548,regulation of endopeptidase activity|GO:1904035,regulation of epithelial cell apoptotic process|GO:0044699,single-organism process|GO:1904037,positive regulation of epithelial cell apoptotic process|GO:0006366,transcription from RNA polymerase II promoter|GO:0019725,cellular homeostasis|GO:0065009,regulation of molecular function|GO:0065008,regulation of biological quality|GO:0048646,anatomical structure formation involved in morphogenesis|GO:0018130,heterocycle biosynthetic process|GO:0016265,obsolete death|GO:0009887,animal organ morphogenesis|GO:0050793,regulation of developmental process|GO:0050790,regulation of catalytic activity|GO:0009889,regulation of biosynthetic process|GO:1904019,epithelial cell apoptotic process|GO:0048468,cell development|GO:0050794,regulation of cellular process|GO:0012501,programmed cell death|GO:1901700,response to oxygen-containing compound|GO:0051239,regulation of multicellular organismal process|GO:0048731,system development|GO:1901216,positive regulation of neuron death|GO:1902533,positive regulation of intracellular signal transduction|GO:1901214,regulation of neuron death|GO:1902531,regulation of intracellular signal transduction|GO:0051336,regulation of hydrolase activity|GO:0044767,single-organism developmental process|GO:0072577,endothelial cell apoptotic process|GO:0007423,sensory organ development|GO:0001654,eye development|GO:0050896,response to stimulus|GO:0009058,biosynthetic process|GO:0032502,developmental process|GO:0006355,regulation of transcription, DNA-templated|GO:0006357,regulation of transcription from RNA polymerase II promoter|GO:0006351,transcription, DNA-templated|GO:0048869,cellular developmental process|GO:0097300,programmed necrotic cell death|GO:0010558,negative regulation of macromolecule biosynthetic process|GO:0055067,monovalent inorganic cation homeostasis|GO:1902041,regulation of extrinsic apoptotic signaling pathway via death domain receptors|GO:0055064,chloride ion homeostasis|GO:0001944,vasculature development|GO:0050801,ion homeostasis|GO:0030154,cell differentiation|GO:0044271,cellular nitrogen compound biosynthetic process|GO:0070848,response to growth factor|GO:0023056,positive regulation of signaling|GO:0007267,cell-cell signaling|GO:0034641,cellular nitrogen compound metabolic process|GO:0023052,signaling|GO:0038127,ERBB signaling pathway|GO:0034645,cellular macromolecule biosynthetic process|GO:0023051,regulation of signaling|GO:0051453,regulation of intracellular pH|GO:0051452,intracellular pH reduction|GO:0010647,positive regulation of cell communication|GO:0010646,regulation of cell communication|GO:0043086,negative regulation of catalytic activity|GO:0043085,positive regulation of catalytic activity|GO:0006139,nucleobase-containing compound metabolic process|GO:0051248,negative regulation of protein metabolic process|GO:0008625,extrinsic apoptotic signaling pathway via death domain receptors|GO:0000122,negative regulation of transcription from RNA polymerase II promoter|GO:0051241,negative regulation of multicellular organismal process|GO:0051246,regulation of protein metabolic process|GO:0051247,positive regulation of protein metabolic process|GO:2000353,positive regulation of endothelial cell apoptotic process|GO:0048513,animal organ development|GO:0045742,positive regulation of epidermal growth factor receptor signaling pathway|GO:0031638,zymogen activation|GO:0032270,positive regulation of cellular protein metabolic process|GO:0071887,leukocyte apoptotic process|GO:0008284,positive regulation of cell proliferation|GO:0016070,RNA metabolic process|GO:0070266,necroptotic process|GO:0010939,regulation of necrotic cell death|GO:0010556,regulation of macromolecule biosynthetic process|GO:0031323,regulation of cellular metabolic process|GO:0032501,multicellular organismal process|GO:0046666,retinal cell programmed cell death|GO:0006725,cellular aromatic compound metabolic process|GO:2000351,regulation of endothelial cell apoptotic process|GO:1903506,regulation of nucleic acid-templated transcription|GO:1903507,negative regulation of nucleic acid-templated transcription|GO:0048518,positive regulation of biological process|GO:0045892,negative regulation of transcription, DNA-templated|GO:0030003,cellular cation homeostasis|GO:0048519,negative regulation of biological process|GO:0055080,cation homeostasis|GO:0055081,anion homeostasis|GO:0055082,cellular chemical homeostasis|GO:0055083,monovalent inorganic	GO:0098590,plasma membrane region|GO:0000323,lytic vacuole|GO:0009897,external side of plasma membrane|GO:0044421,extracellular region part|GO:0060205,cytoplasmic membrane-bounded vesicle lumen|GO:0031974,membrane-enclosed lumen|GO:0005901,caveola|GO:0043229,intracellular organelle|GO:0071944,cell periphery|GO:0005773,vacuole|GO:0044853,plasma membrane raft|GO:0043227,membrane-bounded organelle|GO:0043226,organelle|GO:0031224,intrinsic component of membrane|GO:0005737,cytoplasm|GO:0005764,lysosome|GO:0043202,lysosomal lumen|GO:0031983,vesicle lumen|GO:0070062,extracellular exosome|GO:0016023,cytoplasmic, membrane-bounded vesicle|GO:0031410,cytoplasmic vesicle|GO:0005634,nucleus|GO:0016020,membrane|GO:0005775,vacuolar lumen|GO:0031988,membrane-bounded vesicle|GO:0044433,cytoplasmic vesicle part|GO:0044437,vacuolar part|GO:0048471,perinuclear region of cytoplasm|GO:0044459,plasma membrane part|GO:0031226,intrinsic component of plasma membrane|GO:0009986,cell surface|GO:0098589,membrane region|GO:0005615,extracellular space|GO:0098552,side of membrane|GO:0005887,integral component of plasma membrane|GO:0005886,plasma membrane|GO:0098805,whole membrane|GO:1903561,extracellular vesicle|GO:0031982,vesicle|GO:0043230,extracellular organelle|GO:0043231,intracellular membrane-bounded organelle|GO:0043233,organelle lumen|GO:0045121,membrane raft|GO:0044464,cell part|GO:0005623,cell|GO:0005622,intracellular|GO:0044446,intracellular organelle part|GO:0070013,intracellular organelle lumen|GO:0044444,cytoplasmic part|GO:0005576,extracellular region|GO:0044424,intracellular part|GO:0044425,membrane part|GO:0065010,extracellular membrane-bounded organelle|GO:0016021,integral component of membrane|GO:0044422,organelle part|GO:0097708,intracellular vesicle|GO:0098857,membrane microdomain	GO:0005126,cytokine receptor binding|GO:0032813,tumor necrosis factor receptor superfamily binding|GO:0005123,death receptor binding|GO:0005164,tumor necrosis factor receptor binding|GO:0005125,cytokine activity|GO:0005488,binding|GO:0005515,protein binding|GO:0005102,receptor binding
RAB9A	GO:0008104,protein localization|GO:0006909,phagocytosis|GO:0051046,regulation of secretion|GO:0051047,positive regulation of secretion|GO:0051049,regulation of transport|GO:0006906,vesicle fusion|GO:0061025,membrane fusion|GO:0061024,membrane organization|GO:0007165,signal transduction|GO:0071840,cellular component organization or biogenesis|GO:0052204,negative regulation of molecular function in other organism involved in symbiotic interaction|GO:0051716,cellular response to stimulus|GO:0070727,cellular macromolecule localization|GO:0044419,interspecies interaction between organisms|GO:0051817,modification of morphology or physiology of other organism involved in symbiotic interaction|GO:0044092,negative regulation of molecular function|GO:0048518,positive regulation of biological process|GO:0033036,macromolecule localization|GO:0051050,positive regulation of transport|GO:0052405,negative regulation by host of symbiont molecular function|GO:0045184,establishment of protein localization|GO:0051702,interaction with symbiont|GO:0050794,regulation of cellular process|GO:0051704,multi-organism process|GO:0016197,endosomal transport|GO:0044700,single organism signaling|GO:0044359,modulation of molecular function in other organism|GO:0016192,vesicle-mediated transport|GO:0090174,organelle membrane fusion|GO:0016050,vesicle organization|GO:0022607,cellular component assembly|GO:0007040,lysosome organization|GO:0032940,secretion by cell|GO:0044403,symbiosis, encompassing mutualism through parasitism|GO:0035556,intracellular signal transduction|GO:0050789,regulation of biological process|GO:0006886,intracellular protein transport|GO:0006887,exocytosis|GO:0016043,cellular component organization|GO:0065007,biological regulation|GO:0052205,modulation of molecular function in other organism involved in symbiotic interaction|GO:0065009,regulation of molecular function|GO:0065008,regulation of biological quality|GO:0001845,phagolysosome assembly|GO:0006810,transport|GO:0052428,modification by host of symbiont molecular function|GO:0032482,Rab protein signal transduction|GO:0044802,single-organism membrane organization|GO:0042147,retrograde transport, endosome to Golgi|GO:0044801,single-organism membrane fusion|GO:0051234,establishment of localization|GO:0046903,secretion|GO:0006897,endocytosis|GO:0046907,intracellular transport|GO:0050896,response to stimulus|GO:0023052,signaling|GO:1903530,regulation of secretion by cell|GO:1903532,positive regulation of secretion by cell|GO:0044699,single-organism process|GO:0032880,regulation of protein localization|GO:0044362,negative regulation of molecular function in other organism|GO:0070925,organelle assembly|GO:0051851,modification by host of symbiont morphology or physiology|GO:0017157,regulation of exocytosis|GO:0009987,cellular process|GO:0060627,regulation of vesicle-mediated transport|GO:0032879,regulation of localization|GO:0007033,vacuole organization|GO:0045921,positive regulation of exocytosis|GO:0060341,regulation of cellular localization|GO:0090385,phagosome-lysosome fusion|GO:0090382,phagosome maturation|GO:0044085,cellular component biogenesis|GO:0071702,organic substance transport|GO:0034613,cellular protein localization|GO:0044765,single-organism transport|GO:0044763,single-organism cellular process|GO:0051649,establishment of localization in cell|GO:0007154,cell communication|GO:0007265,Ras protein signal transduction|GO:0007264,small GTPase mediated signal transduction|GO:0051179,localization|GO:1902578,single-organism localization|GO:0051641,cellular localization|GO:0006996,organelle organization|GO:1902589,single-organism organelle organization|GO:0080171,lytic vacuole organization|GO:0048284,organelle fusion|GO:0015031,protein transport|GO:1902582,single-organism intracellular transport|GO:0035821,modification of morphology or physiology of other organism|GO:0048522,positive regulation of cellular process|GO:0042886,amide transport|GO:0071705,nitrogen compound transport|GO:0016482,cytosolic transport|GO:0015833,peptide transport	GO:0016020,membrane|GO:0000323,lytic vacuole|GO:0005783,endoplasmic reticulum|GO:0005789,endoplasmic reticulum membrane|GO:0030139,endocytic vesicle|GO:0042175,nuclear outer membrane-endoplasmic reticulum membrane network|GO:0043229,intracellular organelle|GO:0071944,cell periphery|GO:0005774,vacuolar membrane|GO:0043227,membrane-bounded organelle|GO:0043226,organelle|GO:0005737,cytoplasm|GO:0005764,lysosome|GO:0030666,endocytic vesicle membrane|GO:0070062,extracellular exosome|GO:0016023,cytoplasmic, membrane-bounded vesicle|GO:0031410,cytoplasmic vesicle|GO:0098805,whole membrane|GO:0005770,late endosome|GO:0031988,membrane-bounded vesicle|GO:0044433,cytoplasmic vesicle part|GO:0044432,endoplasmic reticulum part|GO:0044431,Golgi apparatus part|GO:0044437,vacuolar part|GO:0005794,Golgi apparatus|GO:0005622,intracellular|GO:0098588,bounding membrane of organelle|GO:0030659,cytoplasmic vesicle membrane|GO:0045335,phagocytic vesicle|GO:0012505,endomembrane system|GO:0012506,vesicle membrane|GO:0005886,plasma membrane|GO:1903561,extracellular vesicle|GO:0031982,vesicle|GO:0043230,extracellular organelle|GO:0043231,intracellular membrane-bounded organelle|GO:0030670,phagocytic vesicle membrane|GO:0005768,endosome|GO:0044464,cell part|GO:0005623,cell|GO:0000139,Golgi membrane|GO:0044446,intracellular organelle part|GO:0005773,vacuole|GO:0044444,cytoplasmic part|GO:0005576,extracellular region|GO:0031090,organelle membrane|GO:0044424,intracellular part|GO:0044425,membrane part|GO:0065010,extracellular membrane-bounded organelle|GO:0044421,extracellular region part|GO:0044422,organelle part|GO:0048770,pigment granule|GO:0030133,transport vesicle|GO:0031984,organelle subcompartment|GO:0098827,endoplasmic reticulum subcompartment|GO:0032588,trans-Golgi network membrane|GO:0097708,intracellular vesicle|GO:0042470,melanosome|GO:0005829,cytosol|GO:0005802,trans-Golgi network|GO:0098791,Golgi subcompartment	GO:0035639,purine ribonucleoside triphosphate binding|GO:0005488,binding|GO:0043167,ion binding|GO:1901363,heterocyclic compound binding|GO:1901265,nucleoside phosphate binding|GO:0001882,nucleoside binding|GO:0043168,anion binding|GO:0016462,pyrophosphatase activity|GO:0000166,nucleotide binding|GO:0032561,guanyl ribonucleotide binding|GO:0001883,purine nucleoside binding|GO:0032549,ribonucleoside binding|GO:0017076,purine nucleotide binding|GO:0005525,GTP binding|GO:0016787,hydrolase activity|GO:0003924,GTPase activity|GO:0017111,nucleoside-triphosphatase activity|GO:0036094,small molecule binding|GO:0003824,catalytic activity|GO:0016818,hydrolase activity, acting on acid anhydrides, in phosphorus-containing anhydrides|GO:0097367,carbohydrate derivative binding|GO:0097159,organic cyclic compound binding|GO:0016817,hydrolase activity, acting on acid anhydrides|GO:0019001,guanyl nucleotide binding|GO:0019003,GDP binding|GO:0005515,protein binding|GO:0032555,purine ribonucleotide binding|GO:0032550,purine ribonucleoside binding|GO:0032553,ribonucleotide binding|GO:0042802,identical protein binding
REG1B	GO:0044699,single-organism process|GO:0008283,cell proliferation	GO:0043230,extracellular organelle|GO:0031982,vesicle|GO:0044421,extracellular region part|GO:0043227,membrane-bounded organelle|GO:0031988,membrane-bounded vesicle|GO:0005576,extracellular region|GO:1903561,extracellular vesicle|GO:0065010,extracellular membrane-bounded organelle|GO:0043226,organelle|GO:0070062,extracellular exosome	GO:0030246,carbohydrate binding|GO:0005488,binding

<table>
<tr><th></th><th></th><th></th><th></th></tr>
<tr><td>REG1A</td><td>GO:0008284,positive regulation of cell proliferation|GO:0042127,regulation of cell proliferation|GO:0008283,cell proliferation|GO:0009987,cellular process|GO:0050794,regulation of cellular process|GO:0065007,biological regulation|GO:0048518,positive regulation of biological process|GO:0050789,regulation of biological process|GO:0044699,single-organism process|GO:0048522,positive regulation of cellular process</td><td>GO:0043230,extracellular organelle|GO:0031982,vesicle|GO:0044421,extracellular region part|GO:0043227,membrane-bounded organelle|GO:0031988,membrane-bounded vesicle|GO:0005576,extracellular region|GO:1903561,extracellular vesicle|GO:0065010,extracellular membrane-bounded organelle|GO:0043226,organelle|GO:0070062,extracellular exosome</td><td>GO:0008083,growth factor activity|GO:0030246,carbohydrate binding|GO:0005488,binding|GO:0005515,protein binding|GO:0005102,receptor binding</td></tr>
<tr><td>OCIAD1</td><td></td><td></td><td></td></tr>
<tr><td>RPL37</td><td>GO:0008104,protein localization|GO:0061024,membrane organization|GO:1901362,organic cyclic compound biosynthetic process|GO:1901360,organic cyclic compound metabolic process|GO:1901361,organic cyclic compound catabolic process|GO:0070727,cellular macromolecule localization|GO:0043043,peptide biosynthetic process|GO:0044419,interspecies interaction between organisms|GO:0019058,viral life cycle|GO:0033036,macromolecule localization|GO:0051704,multi-organism process|GO:0006605,protein targeting|GO:0045184,establishment of protein localization|GO:0072657,protein localization to membrane|GO:0044403,symbiosis, encompassing mutualism through parasitism|GO:0046483,heterocycle metabolic process|GO:1901564,organonitrogen compound metabolic process|GO:1901566,organonitrogen compound biosynthetic process|GO:0019538,protein metabolic process|GO:0019439,aromatic compound catabolic process|GO:0019438,aromatic compound biosynthetic process|GO:0019080,viral gene expression|GO:0006613,cotranslational protein targeting to membrane|GO:0006612,protein targeting to membrane|GO:0006614,SRP-dependent cotranslational protein targeting to membrane|GO:0006807,nitrogen compound metabolic process|GO:0044033,multi-organism metabolic process|GO:1901576,organic substance biosynthetic process|GO:1901575,organic substance catabolic process|GO:0044265,cellular macromolecule catabolic process|GO:0044260,cellular macromolecule metabolic process|GO:0006886,intracellular protein transport|GO:0016043,cellular component organization|GO:0071840,cellular component organization or biogenesis|GO:0018130,heterocycle biosynthetic process|GO:0006810,transport|GO:0046700,heterocycle catabolic process|GO:0044802,single-organism membrane organization|GO:0008152,metabolic process|GO:0034654,nucleobase-containing compound biosynthetic process|GO:0034655,nucleobase-containing compound catabolic process|GO:0051234,establishment of localization|GO:0016070,RNA metabolic process|GO:0016071,mRNA metabolic process|GO:0044271,cellular nitrogen compound biosynthetic process|GO:0044270,cellular nitrogen compound catabolic process|GO:0046907,intracellular transport|GO:0044765,single-organism transport|GO:0045047,protein targeting to ER|GO:0044764,multi-organism cellular process|GO:0022411,cellular component disassembly|GO:0006518,peptide metabolic process|GO:0032774,RNA biosynthetic process|GO:0044248,cellular catabolic process|GO:0044249,cellular biosynthetic process|GO:0034641,cellular nitrogen compound metabolic process|GO:0034645,cellular macromolecule biosynthetic process|GO:0043241,protein complex disassembly|GO:0044699,single-organism process|GO:0006139,nucleobase-containing compound metabolic process|GO:0070972,protein localization to endoplasmic reticulum|GO:0051179,localization|GO:0019083,viral transcription|GO:0072599,establishment of protein localization to endoplasmic reticulum|GO:0072594,establishment of protein localization to organelle|GO:0009987,cellular process|GO:0006725,cellular aromatic compound metabolic process|GO:0016482,cytosolic transport|GO:0043604,amide biosynthetic process|GO:0043603,cellular amide metabolic process|GO:0043170,macromolecule metabolic process|GO:0000956,nuclear-transcribed mRNA catabolic process|GO:0033365,protein localization to organelle|GO:0000184,nuclear-transcribed mRNA catabolic process, nonsense-mediated decay|GO:0043933,macromolecular complex subunit organization|GO:0090304,nucleic acid metabolic process|GO:0032984,macromolecular complex disassembly|GO:0071822,protein complex subunit organization|GO:0071704,organic substance metabolic process|GO:0010467,gene expression|GO:0071702,organic substance transport|GO:0006401,RNA catabolic process|GO:0006402,mRNA catabolic process|GO:0044267,cellular protein metabolic process|GO:0034613,cellular protein localization|GO:0009058,biosynthetic process|GO:0009059,macromolecule biosynthetic process|GO:0044763,single-organism cellular process|GO:0051649,establishment of localization in cell|GO:0043624,cellular protein complex disassembly|GO:0009056,catabolic process|GO:0009057,macromolecule catabolic process|GO:1902578,single-organism localization|GO:0051641,cellular localization|GO:0044238,primary metabolic process|GO:1902580,single-organism cellular localization|GO:0090150,establishment of protein localization to membrane|GO:0044237,cellular metabolic process|GO:0016032,viral process|GO:0015031,protein transport|GO:1902582,single-organism intracellular transport|GO:0006415,translational termination|GO:0006414,translational elongation|GO:0006413,translational initiation|GO:0006412,translation|GO:0019222,regulation of metabolic process|GO:0010605,negative regulation of macromolecule metabolic process|GO:0048519,negative regulation of biological process|GO:0006364,rRNA processing|GO:0034470,ncRNA processing|GO:0060255,regulation of macromolecule metabolic process|GO:0009892,negative regulation of metabolic process|GO:0010629,negative regulation of gene expression|GO:0034660,ncRNA metabolic process|GO:0050789,regulation of biological process|GO:0042254,ribosome biogenesis|GO:0065007,biological regulation|GO:0016072,rRNA metabolic process|GO:0015833,peptide transport|GO:0042886,amide transport|GO:0022613,ribonucleoprotein complex biogenesis|GO:0071705,nitrogen compound transport|GO:0010468,regulation of gene expression|GO:0044085,cellular component</td><td>GO:0022626,cytosolic ribosome|GO:0022625,cytosolic large ribosomal subunit|GO:0043229,intracellular organelle|GO:0043228,non-membrane-bounded organelle|GO:0015934,large ribosomal subunit|GO:0043226,organelle|GO:0005737,cytoplasm|GO:0030529,intracellular ribonucleoprotein complex|GO:0044391,ribosomal subunit|GO:0032991,macromolecular complex|GO:0005840,ribosome|GO:0043232,intracellular non-membrane-bounded organelle|GO:0005829,cytosol|GO:0044464,cell part|GO:0005623,cell|GO:0005622,intracellular|GO:0044446,intracellular organelle part|GO:0044444,cytoplasmic part|GO:0044445,cytosolic part|GO:0044424,intracellular part|GO:0044422,organelle part|GO:1990904,ribonucleoprotein complex</td><td>GO:0043169,cation binding|GO:0046872,metal ion binding|GO:0097159,organic cyclic compound binding|GO:0019843,rRNA binding|GO:0003735,structural constituent of ribosome|GO:0043167,ion binding|GO:0003723,RNA binding|GO:0003676,nucleic acid binding|GO:0005198,structural molecule activity|GO:1901363,heterocyclic compound binding|GO:0005488,binding</td></tr>
<tr><td>ENSA</td><td>GO:0044699,single-organism process|GO:0009987,cellular process|GO:0050790,regulation of catalytic activity|GO:0065007,biological regulation|GO:0051301,cell division|GO:0044092,negative regulation of molecular function|GO:0044763,single-organism cellular process|GO:0065009,regulation of molecular function|GO:0043086,negative regulation of catalytic activity|GO:0007049,cell cycle</td><td>GO:0005737,cytoplasm|GO:0044464,cell part|GO:0005623,cell|GO:0005622,intracellular|GO:0044424,intracellular part</td><td>GO:0019212,phosphatase inhibitor activity|GO:0019888,protein phosphatase regulator activity|GO:0030234,enzyme regulator activity|GO:0004864,protein phosphatase inhibitor activity|GO:0004857,enzyme inhibitor activity|GO:0098772,molecular function regulator|GO:0019208,phosphatase</td></tr>
<tr><td>TMEM123</td><td>GO:0070265,necrotic cell death|GO:0016265,obsolete death|GO:0070267,oncosis|GO:0071840,cellular component organization or biogenesis|GO:0009987,cellular process|GO:0045793,positive regulation of cell size|GO:0016043,cellular component organization|GO:0090066,regulation of anatomical structure size|GO:0008361,regulation of cell size|GO:0044763,single-organism cellular process|GO:0044699,single-organism process|GO:0065007,biological regulation|GO:0008219,cell death|GO:0065008,regulation of biological quality|GO:0032535,regulation of cellular component size</td><td>GO:0009897,external side of plasma membrane|GO:0016020,membrane|GO:0016021,integral component of membrane|GO:0098552,side of membrane|GO:0044464,cell part|GO:0005623,cell|GO:0071944,cell periphery|GO:0005886,plasma membrane|GO:0044425,membrane part|GO:0044459,plasma membrane part|GO:0009986,cell surface|GO:0031224,intrinsic component of membrane|GO:0043227,membrane-bounded organelle|GO:0043226,organelle|GO:0005737,cytoplasm|GO:0031982,vesicle|GO:0031410,cytoplasmic vesicle|GO:0097708,intracellular vesicle|GO:0005622,intracellular|GO:0044444,cytoplasmic part|GO:0044424,intracellular part</td><td>GO:0060089,molecular transducer activity|GO:0004872,receptor activity|GO:0005515,protein binding|GO:0005488,binding|GO:0038023,signaling receptor activity|GO:0004871,signal transducer activity</td></tr>
<tr><td>FDCSP</td><td></td><td>GO:0005576,extracellular region</td><td></td></tr>
<tr><td>GYPA</td><td>GO:0046718,viral entry into host cell|GO:0040011,locomotion|GO:0009987,cellular process|GO:0052192,movement in environment of other organism involved in symbiotic interaction|GO:0044764,multi-organism cellular process|GO:0044419,interspecies interaction between organisms|GO:0030260,entry into host cell|GO:0051828,entry into other organism involved in symbiotic interaction|GO:0019058,viral life cycle|GO:0016032,viral process|GO:0052126,movement in host environment|GO:0044403,symbiosis, encompassing mutualism through parasitism|GO:0051701,interaction with host|GO:0051704,multi-organism process|GO:0051806,entry into cell of other organism involved in symbiotic interaction|GO:0044409,entry into host|GO:0006928,movement of cell or subcellular component|GO:0050900,leukocyte migration|GO:0051674,localization of cell|GO:0044699,single-organism process|GO:0016477,cell migration|GO:0044763,single-organism cellular process|GO:0051179,localization|GO:0048870,cell motility|GO:0002376,immune system process</td><td>GO:0043230,extracellular organelle|GO:0031982,vesicle|GO:0044421,extracellular region part|GO:0043227,membrane-bounded organelle|GO:0016021,integral component of membrane|GO:0016020,membrane|GO:0044464,cell part|GO:0005623,cell|GO:0031988,membrane-bounded vesicle|GO:0071944,cell periphery|GO:0005576,extracellular region|GO:0005886,plasma membrane|GO:0044425,membrane part|GO:1903561,extracellular vesicle|GO:0065010,extracellular membrane-bounded organelle|GO:0043226,organelle|GO:0031224,intrinsic component of membrane|GO:0070062,extracellular exosome</td><td>GO:0001618,virus receptor activity|GO:0060089,molecular transducer activity|GO:0005488,binding|GO:0005515,protein binding|GO:0042802,identical protein binding|GO:0004872,receptor activity</td></tr>
<tr><td>FAM96A</td><td>GO:0022607,cellular component assembly|GO:0009987,cellular process|GO:0009058,biosynthetic process|GO:0016043,cellular component organization|GO:0016226,iron-sulfur cluster assembly|GO:0031163,metallo-sulfur cluster assembly|GO:0044085,cellular component biogenesis|GO:0044763,single-organism cellular process|GO:0071840,cellular component organization or biogenesis|GO:0008152,metabolic process|GO:0007059,chromosome segregation|GO:0044699,single-organism process</td><td></td><td></td></tr>
<tr><td>TNFAIP6</td><td>GO:0032102,negative regulation of response to external stimulus|GO:0032101,regulation of response to external stimulus|GO:0048585,negative regulation of response to stimulus|GO:0048583,regulation of response to stimulus|GO:0009611,response to wounding|GO:0031348,negative regulation of defense response|GO:0023052,signaling|GO:0007165,signal transduction|GO:0050789,regulation of biological process|GO:0044699,single-organism process|GO:0050728,negative regulation of inflammatory response|GO:0007155,cell adhesion|GO:0050727,regulation of inflammatory response|GO:0065007,biological regulation|GO:0031347,regulation of defense response|GO:0048519,negative regulation of biological process|GO:0022610,biological adhesion|GO:1903034,regulation of response to wounding|GO:1903035,negative regulation of response to wounding|GO:0009987,cellular process|GO:0051716,cellular response to stimulus|GO:0050794,regulation of cellular process|GO:0006952,defense response|GO:0006950,response to stress|GO:0044763,single-organism cellular process|GO:0006954,inflammatory response|GO:0007267,cell-cell signaling|GO:0007154,cell communication|GO:0044700,single organism signaling|GO:0009605,response to external stimulus|GO:0080134,regulation of response to stress|GO:0050896,response to stimulus|GO:0007292,female gamete generation|GO:0000003,reproduction|GO:0002263,cell activation involved in immune response|GO:0048518,positive regulation of biological process|GO:0051704,multi-organism process|GO:0044703,multi-organism reproductive process|GO:0044702,single organism reproductive process|GO:0016192,vesicle-mediated transport|GO:0048870,cell motility|GO:0002275,myeloid cell activation involved in immune response|GO:0002274,myeloid leukocyte activation|GO:0002376,immune system process|GO:0006928,movement of cell or subcellular component|GO:0045321,leukocyte activation|GO:0032940,secretion by cell|GO:0051674,localization of cell|GO:0002283,neutrophil activation involved in immune response|GO:0006887,exocytosis|GO:0045055,regulated exocytosis|GO:0002366,leukocyte activation involved in immune response|GO:0016477,cell migration|GO:0006810,transport|GO:0006955,immune response|GO:0051234,establishment of localization|GO:0046903,secretion|GO:0001775,cell activation|GO:2000145,regulation of cell motility|GO:2000147,positive regulation of cell motility|GO:0019953,sexual reproduction|GO:1902578,single-organism localization|GO:0032501,multicellular organismal process|GO:0048609,multicellular organismal reproductive process|GO:0032504,multicellular organism reproduction|GO:0051270,regulation of cellular component movement|GO:0036230,granulocyte activation|GO:0032879,regulation of localization|GO:0042119,neutrophil activation|GO:0043312,neutrophil degranulation|GO:0007276,gamete generation|GO:0030728,ovulation|GO:0030335,positive regulation of cell migration|GO:0030334,regulation of cell migration|GO:0022414,reproductive process|GO:0044765,single-organism transport|GO:0002443,leukocyte mediated immunity|GO:0002444,myeloid leukocyte mediated immunity|GO:0051179,localization|GO:0002446,neutrophil mediated immunity|GO:0040011,locomotion|GO:0051272,positive regulation of cellular component movement|GO:0040012,regulation of locomotion|GO:0040017,positive regulation of locomotion|GO:0043299,leukocyte degranulation|GO:0002252,immune effector process|GO:0048522,positive regulation of cellular process</td><td>GO:0101002,ficolin-1-rich granule|GO:0031974,membrane-enclosed lumen|GO:1904813,ficolin-1-rich granule lumen|GO:0043229,intracellular organelle|GO:0043227,membrane-bounded organelle|GO:0043226,organelle|GO:0005737,cytoplasm|GO:0070820,tertiary granule|GO:0031982,vesicle|GO:0016023,cytoplasmic, membrane-bounded vesicle|GO:0031410,cytoplasmic vesicle|GO:0031988,membrane-bounded vesicle|GO:0044433,cytoplasmic vesicle part|GO:1904724,tertiary granule lumen|GO:0099503,secretory vesicle|GO:0030141,secretory granule|GO:0097708,intracellular vesicle|GO:0005615,extracellular space|GO:0043231,intracellular membrane-bounded organelle|GO:0044464,cell part|GO:0005623,cell|GO:0005622,intracellular|GO:0044446,intracellular organelle part|GO:0044444,cytoplasmic part|GO:0005576,extracellular region|GO:0044424,intracellular part|GO:0044421,extracellular region part|GO:0044422,organelle part|GO:0012505,endomembrane system</td><td>GO:0005540,hyaluronic acid binding|GO:0005539,glycosaminoglycan binding|GO:0097367,carbohydrate derivative binding|GO:0005488,binding|GO:0043168,anion binding|GO:0031406,carboxylic acid binding|GO:0043167,ion binding|GO:0036094,small molecule binding|GO:0043177,organic acid binding|GO:0005515,protein binding</td></tr>
<tr><td>CMPK1</td><td>GO:0044710,single-organism metabolic process|GO:0009259,ribonucleotide metabolic process|GO:0008152,metabolic process|GO:0009218,pyrimidine ribonucleotide metabolic process|GO:0009141,nucleoside triphosphate metabolic process|GO:0009142,nucleoside triphosphate biosynthetic process|GO:1901564,organonitrogen compound metabolic process|GO:0072528,pyrimidine-containing compound biosynthetic process|GO:0046134,pyrimidine nucleoside biosynthetic process|GO:0034641,cellular nitrogen compound metabolic process|GO:0009165,nucleotide biosynthetic process|GO:0006807,nitrogen compound metabolic process|GO:0009163,nucleoside biosynthetic process|GO:0009161,ribonucleoside monophosphate metabolic process|GO:0046112,nucleobase biosynthetic process|GO:0009123,nucleoside monophosphate metabolic process|GO:1901362,organic cyclic compound biosynthetic process|GO:0046131,pyrimidine ribonucleoside metabolic process|GO:0046132,pyrimidine ribonucleoside biosynthetic process|GO:0072527,pyrimidine-containing compound metabolic process|GO:0006220,pyrimidine nucleotide metabolic process|GO:0006221,pyrimidine nucleotide biosynthetic process|GO:0006222,UMP biosynthetic process|GO:0044711,single-organism biosynthetic process|GO:0019856,pyrimidine nucleobase biosynthetic process|GO:0071704,organic substance metabolic process|GO:0006206,pyrimidine nucleobase metabolic process|GO:0006207,'de novo' pyrimidine nucleobase biosynthetic process|GO:1901360,organic cyclic compound metabolic process|GO:0046939,nucleotide phosphorylation|GO:0046049,UMP metabolic process|GO:1901576,organic substance biosynthetic process|GO:0009112,nucleobase metabolic process|GO:0009124,nucleoside monophosphate biosynthetic process|GO:0016310,phosphorylation|GO:0044249,cellular biosynthetic process|GO:0044281,small molecule metabolic process|GO:0018130,heterocycle biosynthetic process|GO:0019693,ribose phosphate metabolic process|GO:0006139,nucleobase-containing compound metabolic process|GO:0009156,ribonucleoside monophosphate biosynthetic process|GO:0009987,cellular process|GO:0006725,cellular aromatic compound metabolic process|GO:0009220,pyrimidine ribonucleotide biosynthetic process|GO:0055086,nucleobase-containing small molecule metabolic process|GO:0009174,pyrimidine ribonucleoside monophosphate biosynthetic process|GO:0009058,biosynthetic process|GO:0009117,nucleotide metabolic process|GO:0009116,nucleoside metabolic process|GO:0009173,pyrimidine ribonucleoside monophosphate metabolic process|GO:0019438,aromatic compound biosynthetic process|GO:0015949,nucleobase-containing small molecule interconversion|GO:0009130,pyrimidine nucleoside monophosphate biosynthetic process|GO:0009119,ribonucleoside metabolic process|GO:0009132,nucleoside diphosphate metabolic process|GO:0090407,organophosphate biosynthetic process|GO:0042455,ribonucleoside biosynthetic process|GO:0046483,heterocycle metabolic process|GO:0044238,primary metabolic process|GO:0044699,single-organism process|GO:0044271,cellular nitrogen compound biosynthetic process|GO:1901566,organonitrogen compound biosynthetic process|GO:0009260,ribonucleotide biosynthetic process|GO:1901137,carbohydrate derivative biosynthetic process|GO:1901135,carbohydrate derivative metabolic process|GO:0046390,ribose phosphate biosynthetic process|GO:0006213,pyrimidine nucleoside metabolic process|GO:1901657,glycosyl compound metabolic process|GO:0006796,phosphate-containing compound metabolic process|GO:0009129,pyrimidine nucleoside monophosphate metabolic process|GO:1901293,nucleoside phosphate biosynthetic process|GO:0006165,nucleoside diphosphate phosphorylation|GO:0006793,phosphorus metabolic process|GO:0019637,organophosphate metabolic process|GO:0044763,single-organism cellular process|GO:0044237,cellular metabolic process|GO:0006753,nucleoside phosphate metabolic process|GO:0034654,nucleobase-containing compound biosynthetic process|GO:1901659,glycosyl compound biosynthetic process|GO:0046940,nucleoside monophosphate phosphorylation</td><td>GO:0031974,membrane-enclosed lumen|GO:0043229,intracellular organelle|GO:0043228,non-membrane-bounded organelle|GO:0043227,membrane-bounded organelle|GO:0043226,organelle|GO:0005737,cytoplasm|GO:0070062,extracellular exosome|GO:0031981,nuclear lumen|GO:0005730,nucleolus|GO:0005634,nucleus|GO:0031988,membrane-bounded vesicle|GO:0005654,nucleoplasm|GO:1903561,extracellular vesicle|GO:0031982,vesicle|GO:0043230,extracellular organelle|GO:0043231,intracellular membrane-bounded organelle|GO:0043232,intracellular non-membrane-bounded organelle|GO:0043233,organelle lumen|GO:0005829,cytosol|GO:0044464,cell part|GO:0005623,cell|GO:0005622,intracellular|GO:0044446,intracellular organelle part|GO:0070013,intracellular organelle lumen|GO:0044444,cytoplasmic part|GO:0005576,extracellular region|GO:0044428,nuclear part|GO:0044424,intracellular part|GO:0065010,extracellular membrane-bounded organelle|GO:0044421,extracellular region part|GO:0044422,organelle part</td><td>GO:0019201,nucleotide kinase activity|GO:0019206,nucleoside kinase activity|GO:0019205,nucleobase-containing compound kinase activity|GO:0035639,purine ribonucleoside triphosphate binding|GO:0005488,binding|GO:0009041,uridylate kinase activity|GO:0043167,ion binding|GO:1901363,heterocyclic compound binding|GO:0001883,purine nucleoside binding|GO:0001882,nucleoside binding|GO:0043168,anion binding|GO:0000166,nucleotide binding|GO:0016740,transferase activity|GO:1901265,nucleoside phosphate binding|GO:0032549,ribonucleoside binding|GO:0017076,purine nucleotide binding|GO:0005524,ATP binding|GO:0016301,kinase activity|GO:0004127,cytidylate kinase activity|GO:0036094,small molecule binding|GO:0003824,catalytic activity|GO:0004849,uridine kinase activity|GO:0032559,adenyl ribonucleotide binding|GO:0030554,adenyl nucleotide binding|GO:0097367,carbohydrate derivative binding|GO:0016772,transferase activity, transferring phosphorus-containing groups|GO:0016776,phosphotransferase activity, phosphate group as acceptor|GO:0004550,nucleoside diphosphate kinase activity|GO:0097159,organic cyclic compound binding|GO:0032555,purine ribonucleotide binding|GO:0032550,purine ribonucleoside binding|GO:0032553,ribonucleotide binding|GO:0050145,nucleoside phosphate kinase activity</td></tr>
<tr><td>CIRH1A</td><td>GO:0080090,regulation of primary metabolic process|GO:0019222,regulation of metabolic process|GO:0031326,regulation of cellular biosynthetic process|GO:0031323,regulation of cellular metabolic process|GO:0090304,nucleic acid metabolic process|GO:0044249,cellular biosynthetic process|GO:0034641,cellular nitrogen compound metabolic process|GO:0006807,nitrogen compound metabolic process|GO:0034645,cellular macromolecule biosynthetic process|GO:0034660,ncRNA metabolic process|GO:1901362,organic cyclic compound biosynthetic process|GO:0050789,regulation of biological process|GO:0071840,cellular component organization or biogenesis|GO:0032774,RNA biosynthetic process|GO:0006139,nucleobase-containing compound metabolic process|GO:0044260,cellular macromolecule metabolic process|GO:0042274,ribosomal small subunit biogenesis|GO:1901360,organic cyclic compound metabolic process|GO:2000112,regulation of cellular macromolecule biosynthetic process|GO:0042254,ribosome biogenesis|GO:0097659,nucleic acid-templated transcription|GO:0060255,regulation of macromolecule metabolic process|GO:0010467,gene expression|GO:0044085,cellular component biogenesis|GO:0065007,biological regulation|GO:0006364,rRNA processing|GO:0022613,ribonucleoprotein complex biogenesis|GO:0010468,regulation of gene expression|GO:0018130,heterocycle biosynthetic process|GO:0034470,ncRNA processing|GO:1901576,organic substance biosynthetic process|GO:0019219,regulation of nucleobase-containing compound metabolic process|GO:0006725,cellular aromatic compound metabolic process|GO:0009987,cellular process|GO:0009889,regulation of biosynthetic process|GO:1903506,regulation of nucleic acid-templated transcription|GO:0050794,regulation of cellular process|GO:0071704,organic substance metabolic process|GO:0009058,biosynthetic process|GO:0009059,macromolecule biosynthetic process|GO:0051171,regulation of nitrogen compound metabolic process|GO:0008152,metabolic process|GO:2001141,regulation of RNA biosynthetic process|GO:0034654,nucleobase-containing compound biosynthetic process|GO:0046483,heterocycle metabolic process|GO:0016070,RNA metabolic process|GO:0044238,primary metabolic process|GO:0016072,rRNA metabolic process|GO:0044271,cellular nitrogen compound biosynthetic process|GO:0030490,maturation of SSU-rRNA|GO:0051252,regulation of RNA metabolic process|GO:0044237,cellular metabolic process|GO:0043170,macromolecule metabolic process|GO:0006355,regulation of transcription, DNA-templated|GO:0010556,regulation of macromolecule biosynthetic process|GO:0006351,transcription, DNA-templated|GO:0019438,aromatic compound biosynthetic process|GO:0006396,RNA processing</td><td>GO:0031974,membrane-enclosed lumen|GO:0043229,intracellular organelle|GO:0043228,non-membrane-bounded organelle|GO:0043227,membrane-bounded organelle|GO:0043226,organelle|GO:0031981,nuclear lumen|GO:0005730,nucleolus|GO:0005634,nucleus|GO:0044452,nucleolar part|GO:0034455,t-UTP complex|GO:0043234,protein complex|GO:0032991,macromolecular complex|GO:0043231,intracellular membrane-bounded organelle|GO:0043232,intracellular non-membrane-bounded organelle|GO:0043233,organelle lumen|GO:0044464,cell part|GO:0005623,cell|GO:0005622,intracellular|GO:0044446,intracellular organelle part|GO:0070013,intracellular organelle lumen|GO:0044428,nuclear part|GO:0044424,intracellular part|GO:0044422,organelle part|GO:0005654,nucleoplasm|GO:0005694,chromosome|GO:0001650,fibrillar center</td><td>GO:0003676,nucleic acid binding|GO:0097159,organic cyclic compound binding|GO:0044822,poly(A) RNA binding|GO:0003723,RNA binding|GO:0005515,protein binding|GO:1901363,heterocyclic compound binding|GO:0005488,binding</td></tr>
<tr><td>CYP3A5</td><td>GO:0008202,steroid metabolic process|GO:0044248,cellular catabolic process|GO:0006805,xenobiotic metabolic process|GO:0034641,cellular nitrogen compound metabolic process|GO:0006807,nitrogen compound metabolic process|GO:0044281,small molecule metabolic process|GO:0070887,cellular response to chemical stimulus|GO:0044699,single-organism process|GO:0044710,single-organism metabolic process|GO:0009822,alkaloid catabolic process|GO:0009820,alkaloid metabolic process|GO:0071704,organic substance metabolic process|GO:1901360,organic cyclic compound metabolic process|GO:1901575,organic substance catabolic process|GO:0006629,lipid metabolic process|GO:0044238,primary metabolic process|GO:0009987,cellular process|GO:0051716,cellular response to stimulus|GO:0008152,metabolic process|GO:0042221,response to chemical|GO:0009056,catabolic process|GO:0055114,oxidation-reduction process|GO:0042737,drug catabolic process|GO:0070988,demethylation|GO:0070989,oxidative demethylation|GO:1901564,organonitrogen compound metabolic process|GO:0044270,cellular nitrogen compound catabolic process|GO:0050896,response to stimulus|GO:0009410,response to xenobiotic stimulus|GO:0044237,cellular metabolic process|GO:0071466,cellular response to xenobiotic stimulus|GO:0017144,drug metabolic process|GO:1901565,organonitrogen compound catabolic process|GO:0002933,lipid hydroxylation|GO:0044763,single-organism cellular process|GO:0044255,cellular lipid metabolic process|GO:0030258,lipid modification</td><td>GO:0005783,endoplasmic reticulum|GO:0005789,endoplasmic reticulum membrane|GO:0042175,nuclear outer membrane-endoplasmic reticulum membrane network|GO:0043229,intracellular organelle|GO:0043227,membrane-bounded organelle|GO:0043226,organelle|GO:0005737,cytoplasm|GO:0044446,intracellular organelle part|GO:0031090,organelle membrane|GO:0016020,membrane|GO:0044432,endoplasmic reticulum part|GO:0098588,bounding membrane of organelle|GO:0012505,endomembrane system|GO:0043231,intracellular membrane-bounded organelle|GO:0044464,cell part|GO:0005623,cell|GO:0005622,intracellular|GO:0044444,cytoplasmic part|GO:0044424,intracellular part|GO:0044425,membrane part|GO:0044422,organelle part|GO:0031984,organelle</td><td>GO:0043169,cation binding|GO:0004497,monooxygenase activity|GO:0070330,aromatase activity|GO:0046914,transition metal ion binding|GO:0020037,heme binding|GO:0046906,tetrapyrrole binding|GO:0097159,organic cyclic compound binding|GO:0016705,oxidoreductase activity, acting on paired donors, with incorporation or reduction of molecular oxygen|GO:0016712,oxidoreductase activity, acting on paired donors, with incorporation or reduction of molecular oxygen, reduced flavin or flavoprotein as one donor, and incorporation of one atom of oxygen|GO:0003824,catalytic activity|GO:0019825,oxygen binding|GO:0043167,ion binding|GO:0005488,binding|GO:0005506,iron ion binding|GO:0046872,metal ion binding|GO:1901363,heterocyclic compound binding|GO:0016491,oxidoreductase activity</td></tr>
<tr><td>NFU1</td><td>GO:0022607,cellular component assembly|GO:0009987,cellular process|GO:0009058,biosynthetic process|GO:0016043,cellular component organization|GO:0016226,iron-sulfur cluster assembly|GO:0031163,metallo-sulfur cluster assembly|GO:0044085,cellular component biogenesis|GO:0008152,metabolic process|GO:0071840,cellular component organization or biogenesis|GO:0044237,cellular metabolic process|GO:0051188,cofactor biosynthetic process|GO:0044249,cellular biosynthetic process|GO:0006790,sulfur compound metabolic process|GO:0051186,cofactor metabolic process</td><td></td><td>GO:0043169,cation binding|GO:0051540,metal cluster binding|GO:0046914,transition metal ion binding|GO:0051536,iron-sulfur cluster binding|GO:0043167,ion binding|GO:0005488,binding|GO:0005506,iron ion binding|GO:0046872,metal ion binding</td></tr>
<tr><td>NOTUM</td><td>GO:0048585,negative regulation of response to stimulus|GO:0098732,macromolecule deacylation|GO:0048583,regulation of response to stimulus|GO:0023057,negative regulation of signaling|GO:0030111,regulation of Wnt signaling pathway|GO:0023052,signaling|GO:0007165,signal transduction|GO:0007166,cell surface receptor signaling pathway|GO:0023051,regulation of signaling|GO:0010646,regulation of cell communication|GO:0050789,regulation of biological process|GO:0044699,single-organism process|GO:0042157,lipoprotein metabolic process|GO:0044267,cellular protein metabolic process|GO:0051716,cellular response to stimulus|GO:0042159,lipoprotein catabolic process|GO:0009968,negative regulation of signal transduction|GO:0009966,regulation of signal transduction|GO:0030178,negative regulation of Wnt signaling pathway|GO:0071704,organic substance metabolic process|GO:0065007,biological regulation|GO:0048519,negative regulation of biological process|GO:0035601,protein deacylation|GO:0044238,primary metabolic process|GO:0009987,cellular process|GO:1901575,organic substance catabolic process|GO:0006464,cellular protein modification process|GO:0050794,regulation of cellular process|GO:0043412,macromolecule modification|GO:0036211,protein modification process|GO:0044763,single-organism cellular process|GO:0030163,protein catabolic process|GO:0008152,metabolic process|GO:0007154,cell communication|GO:0009056,catabolic process|GO:0009057,macromolecule catabolic process|GO:0044700,single organism signaling|GO:0044260,cellular macromolecule metabolic process|GO:0019538,protein metabolic process|GO:0050896,response to stimulus|GO:0016055,Wnt signaling pathway|GO:0044237,cellular metabolic process|GO:0043170,macromolecule metabolic process|GO:1990697,protein depalmitoleylation|GO:0048523,negative regulation of cellular process|GO:0010648,negative regulation of cell communication|GO:0006807,nitrogen compound metabolic process|GO:1905114,cell surface receptor signaling pathway involved in cell-cell signaling|GO:0198738,cell-cell signaling by wnt|GO:0090090,negative regulation of canonical Wnt signaling pathway|GO:0043687,post-translational protein modification|GO:0060070,canonical Wnt signaling pathway|GO:0007267,cell-cell signaling|GO:1901565,organonitrogen compound catabolic process|GO:1901564,organonitrogen compound metabolic process|GO:0060828,regulation of canonical Wnt signaling pathway</td><td>GO:0005576,extracellular region|GO:0005737,cytoplasm|GO:0012505,endomembrane system|GO:0005783,endoplasmic reticulum|GO:0044432,endoplasmic reticulum part|GO:0043233,organelle lumen|GO:0044464,cell part|GO:0044444,cytoplasmic part|GO:0031974,membrane-enclosed lumen|GO:0005622,intracellular|GO:0044446,intracellular organelle part|GO:0070013,intracellular organelle lumen|GO:0043229,intracellular organelle|GO:0005788,endoplasmic reticulum lumen|GO:0044424,intracellular part|GO:0005623,cell|GO:0043227,membrane-bounded organelle|GO:0043226,organelle|GO:0044422,organelle part|GO:0043231,intracellular membrane-bounded organelle</td><td>GO:0016787,hydrolase activity|GO:0016788,hydrolase activity, acting on ester bonds|GO:0003824,catalytic activity|GO:0052689,carboxylic ester hydrolase activity|GO:1990699,palmitoleyl hydrolase activity|GO:0008081,phosphoric diester hydrolase activity|GO:0042578,phosphoric ester hydrolase activity|GO:0004629,phospholipase C activity|GO:0004620,phospholipase activity|GO:0016298,lipase activity</td></tr>
<tr><td>ALS2CR11</td><td></td><td></td><td>GO:0005488,binding|GO:0005515,protein binding</td></tr>
<tr><td>PDIA5</td><td>GO:0019725,cellular homeostasis|GO:0051716,cellular response to stimulus|GO:0050896,response to stimulus|GO:0009987,cellular process|GO:0050794,regulation of cellular process|GO:0042592,homeostatic process|GO:0006950,response to stress|GO:0044763,single-organism cellular process|GO:0033554,cellular response to stress|GO:0065007,biological regulation|GO:0045454,cell redox homeostasis|GO:0065008,regulation of biological quality|GO:0050789,regulation of biological process|GO:0044699,single-organism process|GO:0034976,response to endoplasmic reticulum stress</td><td>GO:0005623,cell</td><td></td></tr>
<tr><td>TRAPPC3L</td><td>GO:0006810,transport|GO:0016192,vesicle-mediated transport|GO:0051234,establishment of localization|GO:0051179,localization|GO:0048193,Golgi vesicle transport</td><td>GO:0005737,cytoplasm|GO:0005794,Golgi apparatus|GO:0005783,endoplasmic reticulum|GO:0044464,cell part|GO:0044444,cytoplasmic part|GO:0005623,cell|GO:0005622,intracellular|GO:0043229,intracellular organelle|GO:0012505,endomembrane system|GO:0044424,intracellular part|GO:0043227,membrane-bounded organelle|GO:0043226,organelle|GO:0043231,intracellular membrane-bounded organelle|GO:0043234,protein complex|GO:0032991,macromolecular complex|GO:0030008,TRAPP complex</td><td></td></tr>
<tr><td>ATP5C1</td><td>GO:0009167,purine ribonucleoside monophosphate metabolic process|GO:0009165,nucleotide biosynthetic process|GO:0009163,nucleoside biosynthetic process|GO:0009161,ribonucleoside monophosphate metabolic process|GO:1901362,organic cyclic compound biosynthetic process|GO:0042451,purine nucleoside biosynthetic process|GO:0009168,purine ribonucleoside monophosphate biosynthetic process|GO:0044710,single-organism metabolic process|GO:0044711,single-organism biosynthetic process|GO:0009199,ribonucleoside triphosphate metabolic process|GO:0022900,electron transport chain|GO:0042455,ribonucleoside biosynthetic process|GO:0022904,respiratory electron transport chain|GO:0009206,purine ribonucleoside triphosphate biosynthetic process|GO:0009205,purine ribonucleoside triphosphate metabolic process|GO:0009201,ribonucleoside triphosphate biosynthetic process|GO:0046034,ATP metabolic process|GO:0006839,mitochondrial transport|GO:0046129,purine ribonucleoside biosynthetic process|GO:0046128,purine ribonucleoside metabolic process|GO:0055114,oxidation-reduction process|GO:0046483,heterocycle metabolic process|GO:1901564,organonitrogen compound metabolic process|GO:1901566,organonitrogen compound biosynthetic process|GO:0006163,purine nucleotide metabolic process|GO:0006164,purine nucleotide biosynthetic process|GO:0019637,organophosphate metabolic process|GO:0019438,aromatic compound biosynthetic process|GO:0006119,oxidative phosphorylation|GO:0044281,small molecule metabolic process|GO:0009141,nucleoside triphosphate metabolic process|GO:0009142,nucleoside triphosphate biosynthetic process|GO:0009144,purine nucleoside triphosphate metabolic process|GO:0009145,purine nucleoside triphosphate biosynthetic process|GO:0006807,nitrogen compound metabolic process|GO:1901576,organic substance biosynthetic process|GO:0015992,proton transport|GO:0009127,purine nucleoside monophosphate biosynthetic process|GO:0098662,inorganic cation transmembrane transport|GO:0098660,inorganic ion transmembrane transport|GO:0018130,heterocycle biosynthetic process|GO:0006818,hydrogen transport|GO:0009156,ribonucleoside monophosphate biosynthetic process|GO:0006811,ion transport|GO:0006810,transport|GO:0009152,purine ribonucleotide biosynthetic process|GO:0009150,purine ribonucleotide metabolic process|GO:0009260,ribonucleotide biosynthetic process|GO:0045333,cellular respiration|GO:0008152,metabolic process|GO:0034654,nucleobase-containing compound biosynthetic process|GO:0051234,establishment of localization|GO:0090407,organophosphate biosynthetic process|GO:0015985,energy coupled proton transport, down electrochemical gradient|GO:0015986,ATP synthesis coupled proton transport|GO:0044271,cellular nitrogen compound biosynthetic process|GO:0015980,energy derivation by oxidation of organic compounds|GO:0046907,intracellular transport|GO:0046390,ribose phosphate biosynthetic process|GO:0009058,biosynthetic process|GO:1901293,nucleoside phosphate biosynthetic process|GO:0009117,nucleotide metabolic process|GO:0006812,cation transport|GO:0006753,nucleoside phosphate metabolic process|GO:0006754,ATP biosynthetic process|GO:1902600,hydrogen ion transmembrane transport|GO:0016310,phosphorylation|GO:0044249,cellular biosynthetic process|GO:0034641,cellular nitrogen compound metabolic process|GO:0009123,nucleoside monophosphate metabolic process|GO:0009126,purine nucleoside monophosphate metabolic process|GO:0009259,ribonucleotide metabolic process|GO:0009124,nucleoside monophosphate biosynthetic process|GO:0006139,nucleobase-containing compound metabolic process|GO:0042278,purine nucleoside metabolic process|GO:0015672,monovalent inorganic cation transport|GO:0009987,cellular process|GO:0006725,cellular aromatic compound metabolic process|GO:0055085,transmembrane transport|GO:0055086,nucleobase-containing small molecule metabolic process|GO:0044699,single-organism process|GO:1901137,carbohydrate derivative biosynthetic process|GO:1901135,carbohydrate derivative metabolic process|GO:0044237,cellular metabolic process|GO:0072521,purine-containing compound metabolic process|GO:0072522,purine-containing compound biosynthetic process|GO:0006091,generation of precursor metabolites and energy|GO:1901360,organic cyclic compound metabolic process|GO:0071704,organic substance metabolic process|GO:1990542,mitochondrial transmembrane transport|GO:0034220,ion transmembrane transport|GO:0044765,single-organism transport|GO:0044763,single-organism cellular process|GO:0009116,nucleoside metabolic process|GO:0051649,establishment of localization in cell|GO:0042776,mitochondrial ATP synthesis coupled proton transport|GO:0009119,ribonucleoside metabolic process|GO:0051179,localization|GO:1902578,single-organism localization|GO:0051641,cellular localization|GO:0044238,primary metabolic process|GO:0019693,ribose phosphate metabolic process|GO:1901657,glycosyl compound metabolic process|GO:0006796,phosphate-containing compound metabolic process|GO:0006793,phosphorus metabolic process|GO:1902582,single-organism intracellular transport|GO:0098655,cation transmembrane transport|GO:1901659,glycosyl compound biosynthetic process|GO:0061024,membrane organization|GO:0007007,inner mitochondrial membrane organization|GO:0007006,mitochondrial membrane organization|GO:0071840,cellular component organization or biogenesis|GO:0016043,cellular component organization|GO:0044802,single-organism membrane organization|GO:0042407,cristae formation|GO:0007005,mitochondrion organization|GO:1902589,single-organism organelle organization|GO:0006996,organelle organization</td><td>GO:0098796,membrane protein complex|GO:0044464,cell part|GO:0031974,membrane-enclosed lumen|GO:0031975,envelope|GO:0043229,intracellular organelle|GO:0031988,membrane-bounded vesicle|GO:0043227,membrane-bounded organelle|GO:0043226,organelle|GO:0045261,proton-transporting ATP synthase complex, catalytic core F(1)|GO:0033178,proton-transporting two-sector ATPase complex, catalytic domain|GO:0070062,extracellular exosome|GO:0005743,mitochondrial inner membrane|GO:0043209,myelin sheath|GO:0016020,membrane|GO:0044444,cytoplasmic part|GO:0005739,mitochondrion|GO:0098800,inner mitochondrial membrane protein complex|GO:0044455,mitochondrial membrane part|GO:0005753,mitochondrial proton-transporting ATP synthase complex|GO:0005759,mitochondrial matrix|GO:0005737,cytoplasm|GO:0000275,mitochondrial proton-transporting ATP synthase complex, catalytic core F(1)|GO:0031967,organelle envelope|GO:0031966,mitochondrial membrane|GO:0032991,macromolecular complex|GO:1903561,extracellular vesicle|GO:0031982,vesicle|GO:0043234,protein complex|GO:0031090,organelle membrane|GO:0043230,extracellular organelle|GO:0043231,intracellular membrane-bounded organelle|GO:0043233,organelle lumen|GO:0019866,organelle inner membrane|GO:0045259,proton-transporting ATP synthase complex|GO:0005623,cell|GO:0005622,intracellular|GO:0044446,intracellular organelle part|GO:0070013,intracellular organelle lumen|GO:0005740,mitochondrial envelope|GO:0005576,extracellular region|GO:0044429,mitochondrial part|GO:0044424,intracellular part|GO:0044425,membrane part|GO:0016469,proton-transporting two-sector ATPase complex|GO:0065010,extracellular membrane-bounded organelle|GO:0044421,extracellular region part|GO:0044422,organelle part|GO:0098798,mitochondrial protein complex</td><td>GO:0003676,nucleic acid binding|GO:0036442,hydrogen-exporting ATPase activity|GO:0005488,binding|GO:0016887,ATPase activity|GO:1901363,heterocyclic compound binding|GO:0042625,ATPase coupled ion transmembrane transporter activity|GO:0042626,ATPase activity, coupled to transmembrane movement of substances|GO:0016820,hydrolase activity, acting on acid anhydrides, catalyzing transmembrane movement of substances|GO:0046933,proton-transporting ATP synthase activity, rotational mechanism|GO:0015077,monovalent inorganic cation transmembrane transporter activity|GO:0015399,primary active transmembrane transporter activity|GO:0022804,active transmembrane transporter activity|GO:0016787,hydrolase activity|GO:0005215,transporter activity|GO:0044822,poly(A) RNA binding|GO:0008324,cation transmembrane transporter activity|GO:0017111,nucleoside-triphosphatase activity|GO:0003824,catalytic activity|GO:0046961,proton-transporting ATPase activity, rotational mechanism|GO:0044769,ATPase activity, coupled to transmembrane movement of ions, rotational mechanism|GO:0022891,substrate-specific transmembrane transporter activity|GO:0022890,inorganic cation transmembrane transporter activity|GO:0016818,hydrolase activity, acting on acid anhydrides, in phosphorus-containing anhydrides|GO:0022892,substrate-specific transporter activity|GO:0097159,organic cyclic compound binding|GO:0043492,ATPase activity, coupled to movement of substances|GO:0042623,ATPase activity, coupled|GO:0016817,hydrolase activity, acting on acid anhydrides|GO:0019829,cation-transporting ATPase activity|GO:0015075,ion transmembrane transporter activity|GO:0016462,pyrophosphatase activity|GO:0022857,transmembrane transporter activity|GO:0015078,hydrogen ion transmembrane transporter activity|GO:0003723,RNA binding|GO:0015405,P-P-bond-hydrolysis-driven transmembrane transporter activity|GO:0022853,active ion transmembrane transporter activity</td></tr>
</table>

SFR1	GO:0090304,nucleic acid metabolic process|GO:0034641,cellular nitrogen compound metabolic process|GO:0006807,nitrogen compound metabolic process|GO:1901360,organic cyclic compound metabolic process|GO:0006139,nucleobase-containing compound metabolic process|GO:0044710,single-organism metabolic process|GO:0006302,double-strand break repair|GO:0000725,recombinational repair|GO:0000724,double-strand break repair via homologous recombination|GO:0071704,organic substance metabolic process|GO:0044699,single-organism process|GO:0006281,DNA repair|GO:0009987,cellular process|GO:0006725,cellular aromatic compound metabolic process|GO:0051716,cellular response to stimulus|GO:0006974,cellular response to DNA damage stimulus|GO:0006950,response to stress|GO:0044763,single-organism cellular process|GO:0008152,metabolic process|GO:0046483,heterocycle metabolic process|GO:0006310,DNA recombination|GO:0044238,primary metabolic process|GO:0044260,cellular macromolecule metabolic process|GO:0050896,response to stimulus|GO:0044237,cellular metabolic process|GO:0043170,macromolecule metabolic process|GO:0033554,cellular response to stress|GO:0006259,DNA metabolic process|GO:0080090,regulation of primary metabolic process|GO:0019222,regulation of metabolic process|GO:1901362,organic cyclic compound biosynthetic process|GO:0010604,positive regulation of macromolecule metabolic process|GO:0071310,cellular response to organic substance|GO:0048518,positive regulation of biological process|GO:0060255,regulation of macromolecule metabolic process|GO:2001141,regulation of RNA biosynthetic process|GO:0010033,response to organic substance|GO:0009725,response to hormone|GO:0019438,aromatic compound biosynthetic process|GO:0009893,positive regulation of metabolic process|GO:0009891,positive regulation of biosynthetic process|GO:0010628,positive regulation of gene expression|GO:0050789,regulation of biological process|GO:0097659,nucleic acid-templated transcription|GO:1901576,organic substance biosynthetic process|GO:0070887,cellular response to chemical stimulus|GO:0065007,biological regulation|GO:0018130,heterocycle biosynthetic process|GO:0009889,regulation of biosynthetic process|GO:0050794,regulation of cellular process|GO:0034654,nucleobase-containing compound biosynthetic process|GO:0016070,RNA metabolic process|GO:0044271,cellular nitrogen compound biosynthetic process|GO:0006355,regulation of transcription, DNA-templated|GO:0010557,positive regulation of macromolecule biosynthetic process|GO:0010556,regulation of macromolecule biosynthetic process|GO:0006351,transcription, DNA-templated|GO:0032774,RNA biosynthetic process|GO:0044249,cellular biosynthetic process|GO:0034645,cellular macromolecule biosynthetic process|GO:0014070,response to organic cyclic compound|GO:0009719,response to endogenous stimulus|GO:0071383,cellular response to steroid hormone stimulus|GO:0071495,cellular response to endogenous stimulus|GO:1903506,regulation of nucleic acid-templated transcription|GO:0032870,cellular response to hormone stimulus|GO:0045893,positive regulation of transcription, DNA-templated|GO:0071407,cellular response to organic cyclic compound|GO:0051252,regulation of RNA metabolic process|GO:0051254,positive regulation of RNA metabolic process|GO:1902680,positive regulation of RNA biosynthetic process|GO:0048545,response to steroid hormone|GO:1903508,positive regulation of nucleic acid-templated transcription|GO:0031328,positive regulation of cellular biosynthetic process|GO:0031326,regulation of cellular biosynthetic process|GO:0031325,positive regulation of cellular metabolic process|GO:0031323,regulation of cellular metabolic process|GO:0033993,response to lipid|GO:2000112,regulation of cellular macromolecule biosynthetic process|GO:0010467,gene expression|GO:0010468,regulation of gene expression|GO:0071391,cellular response to estrogen stimulus|GO:0045935,positive regulation of nucleobase-containing compound metabolic process|GO:0019219,regulation of nucleobase-containing compound metabolic process|GO:0071396,cellular response to lipid|GO:0009058,biosynthetic process|GO:0009059,macromolecule biosynthetic process|GO:0051171,regulation of nitrogen compound metabolic process|GO:0051173,positive regulation of nitrogen compound metabolic process|GO:0043627,response to	GO:0033061,DNA recombinase mediator complex|GO:0032798,Swi5-Sfr1 complex|GO:0031974,membrane-enclosed lumen|GO:0043229,intracellular organelle|GO:0043228,non-membrane-bounded organelle|GO:0000785,chromatin|GO:0000228,nuclear chromosome|GO:0043227,membrane-bounded organelle|GO:0043226,organelle|GO:0031981,nuclear lumen|GO:0005634,nucleus|GO:0044454,nuclear chromosome part|GO:0005694,chromosome|GO:0000790,nuclear chromatin|GO:0043234,protein complex|GO:0032991,macromolecular complex|GO:0043231,intracellular membrane-bounded organelle|GO:0043232,intracellular non-membrane-bounded organelle|GO:0043233,organelle lumen|GO:0044464,cell part|GO:0005623,cell|GO:0005622,intracellular|GO:0044446,intracellular organelle part|GO:0070013,intracellular organelle lumen|GO:0044428,nuclear part|GO:0044424,intracellular part|GO:0044427,chromosomal part|GO:0044422,organelle part	GO:0005488,binding|GO:0005515,protein binding|GO:0000989,transcription factor activity, transcription factor binding|GO:0003713,transcription coactivator activity|GO:0003712,transcription cofactor activity|GO:0030374,ligand-dependent nuclear receptor transcription coactivator activity|GO:0000988,transcription factor activity, protein binding
GPR176	GO:0044700,single organism signaling|GO:0051716,cellular response to stimulus|GO:0050896,response to stimulus|GO:0009987,cellular process|GO:0044763,single-organism cellular process|GO:0050794,regulation of cellular process|GO:0065007,biological regulation|GO:0023052,signaling|GO:0007165,signal transduction|GO:0007268,chemical synaptic transmission|GO:0007267,cell-cell signaling|GO:0007154,cell communication|GO:0007186,G-protein coupled receptor signaling pathway|GO:0050789,regulation of biological process|GO:0044699,single-organism process|GO:0019220,regulation of phosphate metabolic process|GO:0080090,regulation of primary metabolic process|GO:0019222,regulation of metabolic process|GO:0007610,behavior|GO:0009165,nucleotide biosynthetic process|GO:0045980,negative regulation of nucleotide metabolic process|GO:1901360,organic cyclic compound metabolic process|GO:0044710,single-organism metabolic process|GO:0044711,single-organism biosynthetic process|GO:0052652,cyclic purine nucleotide metabolic process|GO:0048511,rhythmic process|GO:0048512,circadian behavior|GO:0048519,negative regulation of biological process|GO:0009190,cyclic nucleotide biosynthetic process|GO:0006171,cAMP biosynthetic process|GO:0046483,heterocycle metabolic process|GO:1901564,organonitrogen compound metabolic process|GO:1901566,organonitrogen compound biosynthetic process|GO:1900372,negative regulation of purine nucleotide biosynthetic process|GO:1900371,regulation of purine nucleotide biosynthetic process|GO:0006163,purine nucleotide metabolic process|GO:0098916,anterograde trans-synaptic signaling|GO:0006164,purine nucleotide biosynthetic process|GO:0019637,organophosphate metabolic process|GO:0019438,aromatic compound biosynthetic process|GO:0044281,small molecule metabolic process|GO:0009892,negative regulation of metabolic process|GO:0009890,negative regulation of biosynthetic process|GO:0006807,nitrogen compound metabolic process|GO:1901576,organic substance biosynthetic process|GO:1901362,organic cyclic compound biosynthetic process|GO:0009150,purine ribonucleotide metabolic process|GO:0018130,heterocycle biosynthetic process|GO:0007623,circadian rhythm|GO:0007622,rhythmic behavior|GO:0009889,regulation of biosynthetic process|GO:0009152,purine ribonucleotide biosynthetic process|GO:0009260,ribonucleotide biosynthetic process|GO:0008152,metabolic process|GO:0034654,nucleobase-containing compound biosynthetic process|GO:0090407,organophosphate biosynthetic process|GO:0044271,cellular nitrogen compound biosynthetic process|GO:0046390,ribose phosphate biosynthetic process|GO:1901293,nucleoside phosphate biosynthetic process|GO:0006753,nucleoside phosphate metabolic process|GO:0099536,synaptic signaling|GO:0099537,trans-synaptic signaling|GO:0030815,negative regulation of cAMP metabolic process|GO:0044249,cellular biosynthetic process|GO:0034641,cellular nitrogen compound metabolic process|GO:0009259,ribonucleotide metabolic process|GO:0006139,nucleobase-containing compound metabolic process|GO:0030818,negative regulation of cAMP biosynthetic process|GO:0030817,regulation of cAMP biosynthetic process|GO:0030814,regulation of cAMP metabolic process|GO:0010563,negative regulation of phosphorus metabolic process|GO:0006725,cellular aromatic compound metabolic process|GO:0030799,regulation of cyclic nucleotide metabolic process|GO:0055086,nucleobase-containing small molecule metabolic process|GO:0030809,negative regulation of nucleotide biosynthetic process|GO:0030808,regulation of nucleotide biosynthetic process|GO:1901137,carbohydrate derivative biosynthetic process|GO:1901135,carbohydrate derivative metabolic process|GO:0030800,negative regulation of cyclic nucleotide metabolic process|GO:0030803,negative regulation of cyclic nucleotide biosynthetic process|GO:0030802,regulation of cyclic nucleotide biosynthetic process|GO:0009187,cyclic nucleotide metabolic process|GO:0031327,negative regulation of cellular biosynthetic process|GO:0031326,regulation of cellular biosynthetic process|GO:0031324,negative regulation of cellular metabolic process|GO:0031323,regulation of cellular metabolic process|GO:0044237,cellular metabolic process|GO:0072521,purine-containing compound metabolic process|GO:0072522,purine-containing compound biosynthetic process|GO:0071704,organic substance metabolic process|GO:0046058,cAMP metabolic process|GO:0045934,negative regulation of nucleobase-containing compound metabolic process|GO:0045936,negative regulation of phosphate metabolic process|GO:0019219,regulation of nucleobase-containing compound metabolic process|GO:0051174,regulation of phosphorus metabolic process|GO:0009058,biosynthetic process|GO:0009117,nucleotide metabolic process|GO:0051171,regulation of nitrogen compound metabolic process|GO:0051172,negative regulation of nitrogen compound metabolic process|GO:0044238,primary metabolic process|GO:1900543,negative regulation of purine nucleotide metabolic process|GO:1900542,regulation of purine nucleotide metabolic process|GO:0019693,ribose phosphate metabolic process|GO:0006796,phosphate-containing compound metabolic process|GO:0006793,phosphorus metabolic process|GO:0006140,regulation of nucleotide metabolic process|GO:0048523,negative regulation of cellular process|GO:0007193,adenylate cyclase-inhibiting G-protein coupled receptor signaling pathway|GO:0007188,adenylate cyclase-modulating G-protein coupled receptor signaling pathway|GO:0007187,G-	GO:0031226,intrinsic component of plasma membrane|GO:0016021,integral component of membrane|GO:0016020,membrane|GO:0044464,cell part|GO:0005623,cell|GO:0071944,cell periphery|GO:0005887,integral component of plasma membrane|GO:0005886,plasma membrane|GO:0044425,membrane part|GO:0044459,plasma membrane part|GO:0031224,intrinsic component of membrane	GO:0004930,G-protein coupled receptor activity|GO:0038023,signaling receptor activity|GO:0060089,molecular transducer activity|GO:0004888,transmembrane signaling receptor activity|GO:0004872,receptor activity|GO:0004871,signal transducer activity|GO:0099600,transmembrane receptor activity
TOP1	GO:0071103,DNA conformation change|GO:0032502,developmental process|GO:0032922,circadian regulation of gene expression|GO:0019222,regulation of metabolic process|GO:0071704,organic substance metabolic process|GO:0043933,macromolecular complex subunit organization|GO:0016310,phosphorylation|GO:0090304,nucleic acid metabolic process|GO:0009790,embryo development|GO:0034641,cellular nitrogen compound metabolic process|GO:0006807,nitrogen compound metabolic process|GO:1901360,organic cyclic compound metabolic process|GO:0034645,cellular macromolecule biosynthetic process|GO:0042221,response to chemical|GO:0008219,cell death|GO:0050789,regulation of biological process|GO:0044699,single-organism process|GO:0006139,nucleobase-containing compound metabolic process|GO:0044260,cellular macromolecule metabolic process|GO:0006265,DNA topological change|GO:0006325,chromatin organization|GO:0071840,cellular component organization or biogenesis|GO:0016043,cellular component organization|GO:0007275,multicellular organism development|GO:0044419,interspecies interaction between organisms|GO:0048511,rhythmic process|GO:0010467,gene expression|GO:0065007,biological regulation|GO:0051301,cell division|GO:0006260,DNA replication|GO:0051704,multi-organism process|GO:0046483,heterocycle metabolic process|GO:0010468,regulation of gene expression|GO:0016265,obsolete death|GO:0032501,multicellular organismal process|GO:0007623,circadian rhythm|GO:1901576,organic substance biosynthetic process|GO:0060255,regulation of macromolecule metabolic process|GO:0009987,cellular process|GO:0006725,cellular aromatic compound metabolic process|GO:0044767,single-organism developmental process|GO:0012501,programmed cell death|GO:0009058,biosynthetic process|GO:0044764,multi-organism cellular process|GO:0044763,single-organism cellular process|GO:0008152,metabolic process|GO:0007059,chromosome segregation|GO:0051276,chromosome organization|GO:0006996,organelle organization|GO:0044238,primary metabolic process|GO:0006338,chromatin remodeling|GO:0044707,single-multicellular organism process|GO:0040016,embryonic cleavage|GO:0050896,response to stimulus|GO:0048856,anatomical structure development|GO:0042493,response to drug|GO:0044237,cellular metabolic process|GO:0043170,macromolecule metabolic process|GO:0006796,phosphate-containing compound metabolic process|GO:0009059,macromolecule biosynthetic process|GO:0016032,viral process|GO:0006793,phosphorus metabolic process|GO:0006259,DNA metabolic process|GO:0044403,symbiosis, encompassing mutualism through parasitism|GO:0044249,cellular biosynthetic process|GO:0016568,chromatin modification|GO:1901564,organonitrogen compound metabolic process|GO:0018105,peptidyl-serine phosphorylation|GO:0018193,peptidyl-amino acid modification|GO:0070647,protein modification by small protein conjugation or removal|GO:0006468,protein phosphorylation|GO:0044267,cellular protein metabolic process|GO:0032446,protein modification by small protein conjugation|GO:0006464,cellular protein modification process|GO:0043412,macromolecule modification|GO:0036211,protein modification process|GO:0016925,protein sumoylation|GO:0018209,peptidyl-serine modification|GO:0018205,peptidyl-lysine modification|GO:0019538,protein metabolic process	GO:0097458,neuron part|GO:0035770,ribonucleoprotein granule|GO:0044464,cell part|GO:0031974,membrane-enclosed lumen|GO:0043229,intracellular organelle|GO:0043228,non-membrane-bounded organelle|GO:0036464,cytoplasmic ribonucleoprotein granule|GO:0000228,nuclear chromosome|GO:0043227,membrane-bounded organelle|GO:0043226,organelle|GO:0005737,cytoplasm|GO:0043204,perikaryon|GO:0031981,nuclear lumen|GO:0005730,nucleolus|GO:0005634,nucleus|GO:0030529,intracellular ribonucleoprotein complex|GO:0005654,nucleoplasm|GO:0044454,nuclear chromosome part|GO:0005657,replication fork|GO:0005694,chromosome|GO:0044297,cell body|GO:0031298,replication fork protection complex|GO:0036477,somatodendritic compartment|GO:0043234,protein complex|GO:0032991,macromolecular complex|GO:0043231,intracellular membrane-bounded organelle|GO:0043232,intracellular non-membrane-bounded organelle|GO:0043233,organelle lumen|GO:0043025,neuronal cell body|GO:0000932,P-body|GO:0005623,cell|GO:0005622,intracellular|GO:0044446,intracellular organelle part|GO:0070013,intracellular organelle lumen|GO:0044444,cytoplasmic part|GO:0043596,nuclear replication fork|GO:0044428,nuclear part|GO:0044424,intracellular part|GO:0044427,chromosomal part|GO:0044422,organelle part|GO:0009330,DNA topoisomerase complex (ATP-hydrolyzing)|GO:1990904,ribonucleoprotein complex|GO:0044452,nucleolar part|GO:0001650,fibrillar center|GO:0032993,protein-DNA complex	GO:0003676,nucleic acid binding|GO:0061505,DNA topoisomerase II activity|GO:0003917,DNA topoisomerase type I activity|GO:0003916,DNA topoisomerase activity|GO:0044877,macromolecular complex binding|GO:0005488,binding|GO:0016887,ATPase activity|GO:0003677,DNA binding|GO:0003918,DNA topoisomerase type II (ATP-hydrolyzing) activity|GO:0043565,sequence-specific DNA binding|GO:0042623,ATPase activity, coupled|GO:1901363,heterocyclic compound binding|GO:0016787,hydrolase activity|GO:0044212,transcription regulatory region DNA binding|GO:0044822,poly(A) RNA binding|GO:0001067,regulatory region nucleic acid binding|GO:0017111,nucleoside-triphosphatase activity|GO:0003824,catalytic activity|GO:0001047,core promoter binding|GO:0001046,core promoter sequence-specific DNA binding|GO:0008094,DNA-dependent ATPase activity|GO:0016818,hydrolase activity, acting on acid anhydrides, in phosphorus-containing anhydrides|GO:0016853,isomerase activity|GO:0097159,organic cyclic compound binding|GO:0016817,hydrolase activity, acting on acid anhydrides|GO:0016462,pyrophosphatase activity|GO:0000976,transcription regulatory region sequence-specific DNA binding|GO:0003723,RNA binding|GO:0005515,protein binding|GO:0000975,regulatory region DNA binding|GO:0003682,chromatin binding|GO:0017076,purine nucleotide binding|GO:0035639,purine ribonucleoside triphosphate binding|GO:0043167,ion binding|GO:1901265,nucleoside phosphate binding|GO:0043168,anion binding|GO:0043566,structure-specific DNA binding|GO:1990837,sequence-specific double-stranded DNA binding|GO:0097100,supercoiled DNA binding|GO:0000166,nucleotide binding|GO:0004674,protein serine/threonine kinase activity|GO:0016740,transferase activity|GO:0004672,protein kinase activity|GO:0032559,adenyl ribonucleotide binding|GO:0003690,double-stranded DNA binding|GO:0003697,single-stranded DNA binding|GO:0005524,ATP binding|GO:0016301,kinase activity|GO:0036094,small molecule binding|GO:0019904,protein domain specific binding|GO:0030554,adenyl nucleotide binding|GO:0016773,phosphotransferase activity, alcohol group as acceptor|GO:0016772,transferase activity, transferring phosphorus-containing groups|GO:0097367,carbohydrate derivative binding|GO:0032555,purine ribonucleotide binding|GO:0032553,ribonucleotide binding|GO:0000987,core promoter proximal region sequence-specific DNA binding|GO:0000978,RNA polymerase II core promoter proximal region sequence-specific DNA binding|GO:0001012,RNA polymerase II regulatory region DNA binding|GO:0000977,RNA polymerase II regulatory region sequence-specific DNA
PSPH	GO:0009991,response to extracellular stimulus|GO:0009628,response to abiotic stimulus|GO:0016311,dephosphorylation|GO:0019752,carboxylic acid metabolic process|GO:0031667,response to nutrient levels|GO:0044249,cellular biosynthetic process|GO:0034641,cellular nitrogen compound metabolic process|GO:0006807,nitrogen compound metabolic process|GO:0044281,small molecule metabolic process|GO:0044283,small molecule biosynthetic process|GO:0009069,serine family amino acid metabolic process|GO:0044699,single-organism process|GO:0009719,response to endogenous stimulus|GO:1901576,organic substance biosynthetic process|GO:0044710,single-organism metabolic process|GO:0006564,L-serine biosynthetic process|GO:0006563,L-serine metabolic process|GO:0033993,response to lipid|GO:0006520,cellular amino acid metabolic process|GO:1901654,response to ketone|GO:0009612,response to mechanical stimulus|GO:0071704,organic substance metabolic process|GO:0014070,response to organic cyclic compound|GO:1901605,alpha-amino acid metabolic process|GO:0009605,response to external stimulus|GO:1901607,alpha-amino acid biosynthetic process|GO:0044238,primary metabolic process|GO:0009987,cellular process|GO:0009058,biosynthetic process|GO:0044711,single-organism biosynthetic process|GO:0044763,single-organism cellular process|GO:0008152,metabolic process|GO:0043436,oxoacid metabolic process|GO:0042221,response to chemical|GO:0009070,serine family amino acid biosynthetic process|GO:0010033,response to organic substance|GO:1901700,response to oxygen-containing compound|GO:0008652,cellular amino acid biosynthetic process|GO:1901564,organonitrogen compound metabolic process|GO:1901566,organonitrogen compound biosynthetic process|GO:0006082,organic acid metabolic process|GO:0046394,carboxylic acid biosynthetic process|GO:0009725,response to hormone|GO:0050896,response to stimulus|GO:0016053,organic acid biosynthetic process|GO:0044237,cellular metabolic process|GO:0033574,response to testosterone|GO:0006796,phosphate-containing compound metabolic process|GO:0006793,phosphorus metabolic process|GO:0097305,response to alcohol|GO:0048545,response to steroid hormone	GO:0005737,cytoplasm|GO:0005829,cytosol|GO:0044464,cell part|GO:0005623,cell|GO:0005622,intracellular|GO:0097458,neuron part|GO:0044444,cytoplasmic part|GO:0043005,neuron projection|GO:0044424,intracellular part|GO:0042995,cell projection	GO:0005488,binding|GO:0016787,hydrolase activity|GO:0043167,ion binding|GO:0016791,phosphatase activity|GO:0043169,cation binding|GO:0000287,magnesium ion binding|GO:0016788,hydrolase activity, acting on ester bonds|GO:0004647,phosphoserine phosphatase activity|GO:0042578,phosphoric ester hydrolase activity|GO:0003824,catalytic activity|GO:0005509,calcium ion binding|GO:0005515,protein binding|GO:0046983,protein dimerization activity|GO:0046872,metal ion binding|GO:0042802,identical protein binding|GO:0042803,protein homodimerization activity
GIGYF1	GO:0044700,single organism signaling|GO:0051716,cellular response to stimulus|GO:0009987,cellular process|GO:0050896,response to stimulus|GO:0065007,biological regulation|GO:0044763,single-organism cellular process|GO:0050794,regulation of cellular process|GO:0007154,cell communication|GO:0048009,insulin-like growth factor receptor signaling pathway|GO:0023052,signaling|GO:0007165,signal transduction|GO:0007166,cell surface receptor signaling pathway|GO:0007167,enzyme linked receptor protein signaling pathway|GO:0007169,transmembrane receptor protein tyrosine kinase signaling pathway|GO:0050789,regulation of biological process|GO:0044699,single-organism process	GO:0043234,protein complex|GO:0032991,macromolecular complex	GO:0005488,binding|GO:0005515,protein binding
IL1RL2	GO:0019221,cytokine-mediated signaling pathway|GO:0034110,regulation of homotypic cell-cell adhesion|GO:0048583,regulation of response to stimulus|GO:0007165,signal transduction|GO:0007166,cell surface receptor signaling pathway|GO:0031347,regulation of defense response|GO:0034112,positive regulation of homotypic cell-cell adhesion|GO:0051716,cellular response to stimulus|GO:0045785,positive regulation of cell adhesion|GO:0048869,cellular developmental process|GO:0050727,regulation of inflammatory response|GO:0048513,animal organ development|GO:0048518,positive regulation of biological process|GO:0065007,biological regulation|GO:0030217,T cell differentiation|GO:1903708,positive regulation of hemopoiesis|GO:0046649,lymphocyte activation|GO:1903706,regulation of hemopoiesis|GO:0010033,response to organic substance|GO:0044700,single organism signaling|GO:0009605,response to external stimulus|GO:0044707,single-multicellular organism process|GO:0002376,immune system process|GO:0045321,leukocyte activation|GO:0050789,regulation of biological process|GO:0098609,cell-cell adhesion|GO:0002684,positive regulation of immune system process|GO:0002682,regulation of immune system process|GO:0034109,homotypic cell-cell adhesion|GO:0071593,lymphocyte aggregation|GO:0098602,single organism cell adhesion|GO:0034097,response to cytokine|GO:0050793,regulation of developmental process|GO:0050794,regulation of cellular process|GO:0006952,defense response|GO:0001775,cell activation|GO:0051239,regulation of multicellular organismal process|GO:0030097,hemopoiesis|GO:0002521,leukocyte differentiation|GO:0002520,immune system development|GO:0071345,cellular response to cytokine stimulus|GO:0080134,regulation of response to stress|GO:0006950,response to stress|GO:0002694,regulation of leukocyte activation|GO:0002696,positive regulation of leukocyte activation|GO:0044763,single-organism cellular process|GO:0006954,inflammatory response|GO:0032101,regulation of response to external stimulus|GO:0050896,response to stimulus|GO:0030155,regulation of cell adhesion|GO:0030154,cell differentiation|GO:0009611,response to wounding|GO:0045580,regulation of T cell differentiation|GO:0045582,positive regulation of T cell differentiation|GO:0023052,signaling|GO:0070887,cellular response to chemical stimulus|GO:0042221,response to chemical|GO:0044699,single-organism process|GO:0051249,regulation of lymphocyte activation|GO:0051240,positive regulation of multicellular organismal process|GO:1903039,positive regulation of leukocyte cell-cell adhesion|GO:0045621,positive regulation of lymphocyte differentiation|GO:0022610,biological adhesion|GO:1903034,regulation of response to wounding|GO:0032675,regulation of interleukin-6 production|GO:1903037,regulation of leukocyte cell-cell adhesion|GO:0032502,developmental process|GO:0032501,multicellular organismal process|GO:0009987,cellular process|GO:0050870,positive regulation of T cell activation|GO:0045597,positive regulation of cell differentiation|GO:0045595,regulation of cell differentiation|GO:0042110,T cell activation|GO:0006955,immune response|GO:0051094,positive regulation of developmental process|GO:0051251,positive regulation of lymphocyte activation|GO:0001816,cytokine production|GO:0001817,regulation of cytokine production|GO:0045619,regulation of lymphocyte differentiation|GO:0030098,lymphocyte differentiation|GO:0048731,system development|GO:0071347,cellular response to interleukin-1|GO:0001819,positive regulation of cytokine production|GO:0006968,cellular defense response|GO:0016337,single organismal cell-cell adhesion|GO:0050865,regulation of cell activation|GO:0050867,positive regulation of cell activation|GO:0050863,regulation of T cell activation|GO:0022407,regulation of cell-cell adhesion|GO:0070498,interleukin-1-mediated signaling pathway|GO:0007275,multicellular organism development|GO:0022409,positive regulation of cell-cell adhesion|GO:0070555,response to interleukin-1|GO:0071310,cellular response to organic substance|GO:0048534,hematopoietic or lymphoid organ development|GO:0032635,interleukin-6 production|GO:0045087,innate immune response|GO:0044767,single-organism developmental process|GO:0070489,T cell aggregation|GO:0007155,cell adhesion|GO:0007154,cell communication|GO:0070486,leukocyte aggregation|GO:0007159,leukocyte cell-cell adhesion|GO:0048856,anatomical structure development|GO:1902107,positive regulation of leukocyte differentiation|GO:1902105,regulation of leukocyte differentiation|GO:2000026,regulation of multicellular organismal development|GO:0032755,positive regulation of interleukin-6 production|GO:0048522,positive regulation of cellular process	GO:0031226,intrinsic component of plasma membrane|GO:0016021,integral component of membrane|GO:0016020,membrane|GO:0044464,cell part|GO:0005623,cell|GO:0071944,cell periphery|GO:0005887,integral component of plasma membrane|GO:0005886,plasma membrane|GO:0044425,membrane part|GO:0044459,plasma membrane part|GO:0031224,intrinsic component of membrane	GO:0038023,signaling receptor activity|GO:0004888,transmembrane signaling receptor activity|GO:0060089,molecular transducer activity|GO:0004909,interleukin-1, Type I, activating receptor activity|GO:0004908,interleukin-1 receptor activity|GO:0004872,receptor activity|GO:0004871,signal transducer activity|GO:0004896,cytokine receptor activity|GO:0099600,transmembrane receptor activity
XKR7		GO:0044425,membrane part|GO:0016021,integral component of membrane|GO:0016020,membrane|GO:0031224,intrinsic component of membrane	
NAT8L	GO:0008152,metabolic process|GO:0019752,carboxylic acid metabolic process|GO:0044249,cellular biosynthetic process|GO:0006807,nitrogen compound metabolic process|GO:0044281,small molecule metabolic process|GO:0044283,small molecule biosynthetic process|GO:0044699,single-organism process|GO:1901576,organic substance biosynthetic process|GO:0044710,single-organism metabolic process|GO:0044711,single-organism biosynthetic process|GO:0006520,cellular amino acid metabolic process|GO:0071704,organic substance metabolic process|GO:0044238,primary metabolic process|GO:0009987,cellular process|GO:0009058,biosynthetic process|GO:0044763,single-organism cellular process|GO:0043436,oxoacid metabolic process|GO:0008652,cellular amino acid biosynthetic process|GO:1901564,organonitrogen compound metabolic process|GO:0006082,organic acid metabolic process|GO:1901566,organonitrogen compound biosynthetic process|GO:0046394,carboxylic acid biosynthetic process|GO:0016053,organic acid biosynthetic process|GO:0044237,cellular metabolic process	GO:0005783,endoplasmic reticulum|GO:0044464,cell part|GO:0005789,endoplasmic reticulum membrane|GO:0042175,nuclear outer membrane-endoplasmic reticulum membrane network|GO:0043229,intracellular organelle|GO:0043227,membrane-bounded organelle|GO:0043226,organelle|GO:0031224,intrinsic component of membrane|GO:0005737,cytoplasm|GO:0031090,organelle membrane|GO:0016021,integral component of membrane|GO:0016020,membrane|GO:0044444,cytoplasmic part|GO:0005739,mitochondrion|GO:0044432,endoplasmic reticulum part|GO:0005791,rough endoplasmic reticulum|GO:0098588,bounding membrane of organelle|GO:0031967,organelle envelope|GO:0031966,mitochondrial membrane|GO:0012505,endomembrane system|GO:0031975,envelope|GO:0043231,intracellular membrane-bounded organelle|GO:0030867,rough endoplasmic reticulum membrane|GO:0005623,cell|GO:0005622,intracellular|GO:0044446,intracellular organelle part|GO:0005740,mitochondrial envelope|GO:0044429,mitochondrial part|GO:0044424,intracellular part|GO:0044425,membrane part|GO:0044422,organelle part|GO:0031974,membrane-enclosed lumen|GO:0031984,organelle subcompartment|GO:0098827,endoplasmic reticulum subcompartment|GO:0005759,mitochondrial matrix|GO:0043233,organelle lumen|GO:0070013,intracellular organelle	GO:0017188,aspartate N-acetyltransferase activity|GO:0008080,N-acetyltransferase activity|GO:0016740,transferase activity|GO:0003824,catalytic activity|GO:0016407,acetyltransferase activity|GO:0016746,transferase activity, transferring acyl groups|GO:0016747,transferase activity, transferring acyl groups other than amino-acyl groups|GO:0016410,N-acyltransferase activity
MAGEC3			
VASH1	GO:0048583,regulation of response to stimulus|GO:0050680,negative regulation of epithelial cell proliferation|GO:0072359,circulatory system development|GO:0072358,cardiovascular system development|GO:0044707,single-multicellular organism process|GO:0051716,cellular response to stimulus|GO:0045786,negative regulation of cell cycle|GO:0009611,response to wounding|GO:0048514,blood vessel morphogenesis|GO:0048519,negative regulation of biological process|GO:0042127,regulation of cell proliferation|GO:0043537,negative regulation of blood vessel endothelial cell migration|GO:0043535,regulation of blood vessel endothelial cell migration|GO:0043534,blood vessel endothelial cell migration|GO:0050678,regulation of epithelial cell proliferation|GO:0010631,epithelial cell migration|GO:0010632,regulation of epithelial cell migration|GO:0048870,cell motility|GO:0010633,negative regulation of epithelial cell migration|GO:0033554,cellular response to stress|GO:0090342,regulation of cell aging|GO:0022603,regulation of anatomical structure morphogenesis|GO:0006928,movement of cell or subcellular component|GO:1901342,regulation of vasculature development|GO:1901343,negative regulation of vasculature development|GO:0050789,regulation of biological process|GO:0043542,endothelial cell migration|GO:0001568,blood vessel development|GO:0065007,biological regulation|GO:0044699,single-organism process|GO:0016477,cell migration|GO:0048646,anatomical structure formation involved in morphogenesis|GO:0050793,regulation of developmental process|GO:0050794,regulation of cellular process|GO:0007050,cell cycle arrest|GO:0006950,response to stress|GO:0051239,regulation of multicellular organismal process|GO:0080134,regulation of response to stress|GO:0080135,regulation of cellular response to stress|GO:2000145,regulation of cell motility|GO:2000146,negative regulation of cell motility|GO:0001667,ameboidal-type cell migration|GO:0009653,anatomical structure morphogenesis|GO:0007049,cell cycle|GO:0051241,negative regulation of multicellular organismal process|GO:0001944,vasculature development|GO:0001945,lymph vessel development|GO:0001946,lymphangiogenesis|GO:0032502,developmental process|GO:0008285,negative regulation of cell proliferation|GO:0032501,multicellular organismal process|GO:0008283,cell proliferation|GO:0009987,cellular process|GO:0040013,negative regulation of locomotion|GO:0051270,regulation of cellular component movement|GO:0032879,regulation of localization|GO:0090132,epithelium migration|GO:0090130,tissue migration|GO:0051093,negative regulation of developmental process|GO:0007568,aging|GO:0007569,cell aging|GO:0010596,negative regulation of endothelial cell migration|GO:0050673,epithelial cell proliferation|GO:0051674,localization of cell|GO:0048731,system development|GO:2000772,regulation of cellular senescence|GO:0050896,response to stimulus|GO:0016525,negative regulation of angiogenesis|GO:0048856,anatomical structure development|GO:2000181,negative regulation of blood vessel morphogenesis|GO:0022402,cell cycle process|GO:0001525,angiogenesis|GO:0007275,multicellular organism development|GO:0036303,lymph vessel morphogenesis|GO:0045765,regulation of angiogenesis|GO:0030336,negative regulation of cell migration|GO:0030334,regulation of cell migration|GO:1901490,regulation of lymphangiogenesis|GO:1901491,negative regulation of lymphangiogenesis|GO:0044767,single-organism developmental process|GO:0044763,single-organism cellular process|GO:0051179,localization|GO:0040011,locomotion|GO:0051271,negative regulation of cellular component movement|GO:0040012,regulation of locomotion|GO:0010594,regulation of endothelial cell migration|GO:0051726,regulation of cell cycle|GO:0090398,cellular senescence|GO:2000026,regulation of multicellular organismal development|GO:0001937,negative regulation of endothelial cell proliferation|GO:0001935,endothelial cell proliferation|GO:0048523,negative regulation of cellular process|GO:0001936,regulation of endothelial cell proliferation|GO:0060711,labyrinthine layer development|GO:0060716,labyrinthine layer blood vessel development|GO:0000003,reproduction|GO:0048869,cellular developmental process|GO:0048513,animal organ development|GO:0003006,developmental process involved in reproduction|GO:0044702,single organism reproductive process|GO:0048568,embryonic organ development|GO:0060674,placenta blood vessel development|GO:0009790,embryo development|GO:0009792,embryo development ending in birth or egg hatching|GO:0001701,in utero embryonic development|GO:0043009,chordate embryonic development|GO:0048608,reproductive structure development|GO:0001892,embryonic placenta development|GO:0001890,placenta development|GO:0061458,reproductive system development|GO:0022414,reproductive process	GO:0005737,cytoplasm|GO:0045177,apical part of cell|GO:0005783,endoplasmic reticulum|GO:0044421,extracellular region part|GO:0005615,extracellular space|GO:0044464,cell part|GO:0043229,intracellular organelle|GO:0005623,cell|GO:0005622,intracellular|GO:0044444,cytoplasmic part|GO:0005576,extracellular region|GO:0012505,endomembrane system|GO:0044424,intracellular part|GO:0043227,membrane-bounded organelle|GO:0043226,organelle|GO:0043231,intracellular membrane-bounded organelle	GO:0005488,binding|GO:0005515,protein binding
ANKRD52			GO:0005488,binding|GO:0005515,protein binding
FADS6	GO:0006633,fatty acid biosynthetic process|GO:0006631,fatty acid metabolic process|GO:0019752,carboxylic acid metabolic process|GO:0044249,cellular biosynthetic process|GO:0044281,small molecule metabolic process|GO:0044283,small molecule biosynthetic process|GO:0072330,monocarboxylic acid biosynthetic process|GO:0044699,single-organism process|GO:1901576,organic substance biosynthetic process|GO:0044710,single-organism metabolic process|GO:0044711,single-organism biosynthetic process|GO:0071704,organic substance metabolic process|GO:0006629,lipid metabolic process|GO:0009987,cellular process|GO:0032787,monocarboxylic acid metabolic process|GO:0009058,biosynthetic process|GO:0044763,single-organism cellular process|GO:0008152,metabolic process|GO:0043436,oxoacid metabolic process|GO:0044255,cellular lipid metabolic process|GO:0055114,oxidation-reduction process|GO:0008610,lipid biosynthetic process|GO:0044238,primary metabolic process|GO:0006082,organic acid metabolic process|GO:0046394,carboxylic acid biosynthetic process|GO:0016053,organic acid biosynthetic process|GO:0044237,cellular metabolic process	GO:0044425,membrane part|GO:0016021,integral component of membrane|GO:0016020,membrane|GO:0031224,intrinsic component of membrane	GO:0003824,catalytic activity|GO:0016491,oxidoreductase activity

FBXW7	GO:0033157,regulation of intracellular protein transport|GO:0010564,regulation of cell cycle process|GO:0051049,regulation of transport|GO:0032388,positive regulation of intracellular transport|GO:0051716,cellular response to stimulus|GO:0000165,MAPK cascade|GO:0032386,regulation of intracellular transport|GO:0060541,respiratory system development|GO:0045859,regulation of protein kinase activity|GO:0006839,mitochondrial transport|GO:0046486,glycerolipid metabolic process|GO:0046483,heterocycle metabolic process|GO:0042325,regulation of phosphorylation|GO:0042327,positive regulation of phosphorylation|GO:0009605,response to external stimulus|GO:0019538,protein metabolic process|GO:0010638,positive regulation of organelle organization|GO:0006275,regulation of DNA replication|GO:0010883,regulation of lipid storage|GO:0009896,positive regulation of catabolic process|GO:0016265,obsolete death|GO:0009894,regulation of catabolic process|GO:0009892,negative regulation of metabolic process|GO:0009893,positive regulation of metabolic process|GO:0009890,negative regulation of biosynthetic process|GO:0009891,positive regulation of biosynthetic process|GO:1903008,organelle disassembly|GO:0010868,negative regulation of triglyceride biosynthetic process|GO:0051222,positive regulation of protein transport|GO:0051223,regulation of protein transport|GO:0050789,regulation of biological process|GO:0010866,regulation of triglyceride biosynthetic process|GO:0051340,regulation of ligase activity|GO:0051347,positive regulation of transferase activity|GO:0006886,intracellular protein transport|GO:1901360,organic cyclic compound metabolic process|GO:0018130,heterocycle biosynthetic process|GO:2000273,positive regulation of receptor activity|GO:0070201,regulation of establishment of protein localization|GO:0006629,lipid metabolic process|GO:1903649,regulation of cytoplasmic transport|GO:0006626,protein targeting to mitochondrion|GO:0043410,positive regulation of MAPK cascade|GO:0043412,macromolecule modification|GO:0009267,cellular response to starvation|GO:0007059,chromosome segregation|GO:0016070,RNA metabolic process|GO:0010557,positive regulation of macromolecule biosynthetic process|GO:0010556,regulation of macromolecule biosynthetic process|GO:0048869,cellular developmental process|GO:0010558,negative regulation of macromolecule biosynthetic process|GO:0018212,peptidyl-tyrosine modification|GO:0051128,regulation of cellular component organization|GO:1903827,regulation of cellular protein localization|GO:0042176,regulation of protein catabolic process|GO:0009411,response to UV|GO:0009416,response to light stimulus|GO:0038127,ERBB signaling pathway|GO:0051235,maintenance of location|GO:1903829,positive regulation of cellular protein localization|GO:0045746,negative regulation of Notch signaling pathway|GO:0045742,positive regulation of epidermal growth factor receptor signaling pathway|GO:0045741,positive regulation of epidermal growth factor-activated receptor activity|GO:0008285,negative regulation of cell proliferation|GO:0008283,cell proliferation|GO:0006974,cellular response to DNA damage stimulus|GO:0044257,cellular protein catabolic process|GO:0044255,cellular lipid metabolic process|GO:0006979,response to oxidative stress|GO:0001889,liver development|GO:0060341,regulation of cellular localization|GO:0097190,apoptotic signaling pathway|GO:0006366,transcription from RNA polymerase II promoter|GO:0018108,peptidyl-tyrosine phosphorylation|GO:0097193,intrinsic apoptotic signaling pathway|GO:0042592,homeostatic process|GO:0022402,cell cycle process|GO:2000060,positive regulation of protein ubiquitination involved in ubiquitin-dependent protein catabolic process|GO:0042594,response to starvation|GO:0010822,positive regulation of mitochondrion organization|GO:0008219,cell death|GO:0007275,multicellular organism development|GO:2000112,regulation of cellular macromolecule biosynthetic process|GO:2000113,negative regulation of cellular macromolecule biosynthetic process|GO:2000058,regulation of protein ubiquitination involved in ubiquitin-dependent protein catabolic process|GO:0043065,positive regulation of apoptotic process|GO:0043067,regulation of programmed cell death|GO:0043068,positive regulation of programmed cell death|GO:1903223,positive regulation of oxidative stress-induced neuron death|GO:0006468,protein phosphorylation|GO:1902177,positive regulation of oxidative stress-induced intrinsic apoptotic signaling pathway|GO:0019216,regulation of lipid metabolic process|GO:0019219,regulation of nucleobase-containing compound metabolic process|GO:0090316,positive regulation of intracellular protein transport|GO:0006464,cellular protein modification process|GO:0044767,single-organism developmental process|GO:0044765,single-organism transport|GO:0044764,multi-organism cellular process|GO:0044763,single-organism cellular process|GO:1902884,positive regulation of response to oxidative stress|GO:1902882,regulation of response to oxidative stress|GO:0051276,chromosome organization|GO:0036473,cell death in response to oxidative stress|GO:0048856,anatomical structure development|GO:0006796,phosphate-containing compound metabolic process|GO:2000026,regulation of multicellular organismal development|GO:2000027,regulation of organ morphogenesis|GO:0006793,phosphorus metabolic process|GO:0048523,negative regulation of cellular process|GO:0048522,positive regulation of cellular process|GO:0008104,protein localization|GO:0051603,proteolysis involved in cellular protein catabolic process|GO:0043523,regulation of neuron apoptotic process|GO:0007165,signal transduction|GO:0007166,cell surface receptor signaling pathway|GO:0007167,enzyme linked receptor protein signaling pathway|GO:0007169,transmembrane receptor protein tyrosine kinase signaling pathway|GO:0007005,mitochondrion organization|GO:0036480,neuron intrinsic apoptotic signaling pathway in response to oxidative stress|GO:0044712,single-organism catabolic process|GO:0044710,single-organism metabolic process|GO:0044711,single-organism biosynthetic process|GO:0045786,negative regulation of cell cycle|GO:0032101,regulation of response to external stimulus|GO:0044093,positive regulation of molecular function|GO:0009314,response to radiation|GO:0033036,macromolecule localization|GO:0051055,negative regulation of lipid biosynthetic process|GO:0051053,negative regulation of DNA metabolic process|GO:0051052,regulation of DNA metabolic process|GO:0051050,positive regulation of transport|GO:1903955,positive regulation of protein targeting to mitochondrion|GO:2001141,regulation of RNA biosynthetic process|GO:0072655,establishment of protein localization to mitochondrion|GO:0051704,multi-organism process|GO:0044248,cellular catabolic process|GO:0031668,cellular response to extracellular stimulus|GO:0031669,cellular response to nutrient levels|GO:0044249,cellular biosynthetic process|GO:0031667,response to nutrient levels|GO:0097285,obsolete cell-type specific apoptotic process|GO:0016567,protein ubiquitination|GO:0044786,cell cycle DNA replication|GO:1903533,regulation of protein targeting|GO:0061008,hepaticobiliary system development|GO:0034644,cellular response to UV|GO:0050821,protein stabilization|GO:0010628,positive regulation of gene expression|GO:0044267,cellular protein metabolic process|GO:0009653,anatomical structure morphogenesis|GO:0044260,cellular macromolecule metabolic process|GO:0070997,neuron death|GO:0001568,blood vessel development|GO:1902175,regulation of oxidative stress-induced intrinsic apoptotic signaling pathway|GO:0007049,cell cycle|GO:0061097,regulation of protein tyrosine kinase activity|GO:0061098,positive regulation of protein tyrosine kinase activity|GO:0043408,regulation of MAPK cascade|GO:0009887,animal organ morphogenesis|GO:0034613,cellular protein localization|GO:0050793,regulation of developmental process|GO:0050790,regulation of catalytic activity|GO:0009889,regulation of biosynthetic process|GO:0050794,regulation of cellular process|GO:0051239,regulation of multicellular organismal process|GO:1901216,positive regulation of neuron death|GO:1901214,regulation of neuron death|GO:0051234,establishment of localization|GO:0071501,cellular response to sterol depletion|GO:0050896,response to stimulus|GO:0010498,proteasomal protein catabolic process|GO:0051338,regulation of transferase activity|GO:0006511,ubiquitin-dependent protein catabolic process|GO:0033674,positive regulation of kinase activity|GO:0032107,regulation of response to nutrient levels|GO:0032104,regulation of response to extracellular stimulus|GO:0071482,cellular response to light stimulus|GO:0033043,regulation of organelle organization|GO:0051246,regulation of protein metabolic process|GO:0006639,acylglycerol metabolic process|GO:0006638,neutral lipid metabolic	GO:1990452,Parkin-FBXW7-Cul1 ubiquitin ligase complex|GO:0005783,endoplasmic reticulum|GO:0031974,membrane-enclosed lumen|GO:0043229,intracellular organelle|GO:0043228,non-membrane-bounded organelle|GO:0043227,membrane-bounded organelle|GO:0043226,organelle|GO:0005737,cytoplasm|GO:0031981,nuclear lumen|GO:0005730,nucleolus|GO:0005634,nucleus|GO:0005654,nucleoplasm|GO:0005794,Golgi apparatus|GO:1902494,catalytic complex|GO:0012505,endomembrane system|GO:1990234,transferase complex|GO:0031461,cullin-RING ubiquitin ligase complex|GO:0043234,protein complex|GO:0032991,macromolecular complex|GO:0043231,intracellular membrane-bounded organelle|GO:0043232,intracellular non-membrane-bounded organelle|GO:0043233,organelle lumen|GO:0044464,cell part|GO:0000151,ubiquitin ligase complex|GO:0005623,cell|GO:0005622,intracellular|GO:0044446,intracellular organelle part|GO:0070013,intracellular organelle lumen|GO:0044444,cytoplasmic part|GO:0044428,nuclear part|GO:0019005,SCF ubiquitin ligase complex|GO:0044424,intracellular part|GO:0044422,organelle part|GO:0005829,cytosol	GO:0030674,protein binding, bridging|GO:0031625,ubiquitin protein ligase binding|GO:0044389,ubiquitin-like protein ligase binding|GO:0030332,cyclin binding|GO:0060090,binding, bridging|GO:0030234,enzyme regulator activity|GO:0001071,nucleic acid binding transcription factor activity|GO:0019899,enzyme binding|GO:0003700,transcription factor activity, sequence-specific DNA binding|GO:0097027,ubiquitin-protein transferase activator activity|GO:0005488,binding|GO:0005515,protein binding|GO:0055103,ligase regulator activity|GO:0098772,molecular function regulator|GO:0042802,identical protein binding|GO:0055106,ubiquitin-protein transferase regulator activity|GO:0045309,protein phosphorylated amino acid binding|GO:0019787,ubiquitin-like protein transferase activity|GO:0016740,transferase activity|GO:0003824,catalytic activity|GO:0050816,phosphothreonine binding|GO:0004842,ubiquitin-protein transferase activity|GO:0051219,phosphoprotein binding
RHOB	GO:0008104,protein localization|GO:0007599,hemostasis|GO:0051234,establishment of localization|GO:0007596,blood coagulation|GO:0048583,regulation of response to stimulus|GO:0072359,circulatory system development|GO:0072358,cardiovascular system development|GO:0007165,signal transduction|GO:0071840,cellular component organization or biogenesis|GO:0051716,cellular response to stimulus|GO:0010035,response to inorganic substance|GO:0045786,negative regulation of cell cycle|GO:0042330,taxis|GO:0070727,cellular macromolecule localization|GO:0009966,regulation of signal transduction|GO:0048869,cellular developmental process|GO:0009611,response to wounding|GO:0048514,blood vessel morphogenesis|GO:0048518,positive regulation of biological process|GO:0048519,negative regulation of biological process|GO:0016482,cytosolic transport|GO:0051056,regulation of small GTPase mediated signal transduction|GO:0030154,cell differentiation|GO:0006935,chemotaxis|GO:0048468,cell development|GO:0045184,establishment of protein localization|GO:0042221,response to chemical|GO:0097485,neuron projection guidance|GO:0030168,platelet activation|GO:0000910,cytokinesis|GO:0044700,single organism signaling|GO:0071214,cellular response to abiotic stimulus|GO:0009605,response to external stimulus|GO:0044707,single-multicellular organism process|GO:0097285,obsolete cell-type specific apoptotic process|GO:0007154,cell communication|GO:0033554,cellular response to stress|GO:0006927,obsolete transformed cell apoptotic process|GO:0070887,cellular response to chemical stimulus|GO:0022603,regulation of anatomical structure morphogenesis|GO:0006928,movement of cell or subcellular component|GO:1901342,regulation of vasculature development|GO:0031175,neuron projection development|GO:0035556,intracellular signal transduction|GO:0042981,regulation of apoptotic process|GO:0050789,regulation of biological process|GO:0000904,cell morphogenesis involved in differentiation|GO:0000902,cell morphogenesis|GO:0001568,blood vessel development|GO:0016043,cellular component organization|GO:0065007,biological regulation|GO:0044699,single-organism process|GO:0065008,regulation of biological quality|GO:0048646,anatomical structure formation involved in morphogenesis|GO:0016265,obsolete death|GO:0034613,cellular protein localization|GO:0061564,axon development|GO:0050793,regulation of developmental process|GO:0006810,transport|GO:0042060,wound healing|GO:0050794,regulation of cellular process|GO:0012501,programmed cell death|GO:0001775,cell activation|GO:0050817,coagulation|GO:0051239,regulation of multicellular organismal process|GO:1902531,regulation of intracellular signal transduction|GO:0046907,intracellular transport|GO:0042542,response to hydrogen peroxide|GO:0050896,response to stimulus|GO:0006950,response to stress|GO:0048812,neuron projection morphogenesis|GO:0071478,cellular response to radiation|GO:0034599,cellular response to oxidative stress|GO:0009314,response to radiation|GO:0008333,endosome to lysosome transport|GO:0023052,signaling|GO:0007041,lysosomal transport|GO:0023051,regulation of signaling|GO:0007411,axon guidance|GO:0010646,regulation of cell communication|GO:0007265,Ras protein signal transduction|GO:0007049,cell cycle|GO:0071479,cellular response to ionizing radiation|GO:1904018,positive regulation of vasculature development|GO:0010212,response to ionizing radiation|GO:0009653,anatomical structure morphogenesis|GO:0051240,positive regulation of multicellular organismal process|GO:0001944,vasculature development|GO:0022610,biological adhesion|GO:0032502,developmental process|GO:1901700,response to oxygen-containing compound|GO:0032501,multicellular organismal process|GO:0050878,regulation of body fluid levels|GO:0009628,response to abiotic stimulus|GO:0070301,cellular response to hydrogen peroxide|GO:0007409,axonogenesis|GO:0000302,response to reactive oxygen species|GO:0006979,response to oxidative stress|GO:0048858,cell projection morphogenesis|GO:0033036,macromolecule localization|GO:0007034,vacuolar transport|GO:0051094,positive regulation of developmental process|GO:0016197,endosomal transport|GO:0048731,system development|GO:0030030,cell projection organization|GO:0022402,cell cycle process|GO:0010942,positive regulation of cell death|GO:0008219,cell death|GO:0001525,angiogenesis|GO:0007275,multicellular organism development|GO:0051301,cell division|GO:0010941,regulation of cell death|GO:0045765,regulation of angiogenesis|GO:0045766,positive regulation of angiogenesis|GO:0032989,cellular component morphogenesis|GO:0043065,positive regulation of apoptotic process|GO:0043067,regulation of programmed cell death|GO:0071702,organic substance transport|GO:0043068,positive regulation of programmed cell death|GO:0048666,neuron development|GO:0048667,cell morphogenesis involved in neuron differentiation|GO:0009987,cellular process|GO:0030182,neuron differentiation|GO:0006915,apoptotic process|GO:0034614,cellular response to reactive oxygen species|GO:0044767,single-organism developmental process|GO:0044765,single-organism transport|GO:0044763,single-organism cellular process|GO:0051649,establishment of localization in cell|GO:0007155,cell adhesion|GO:0007266,Rho protein signal transduction|GO:0022008,neurogenesis|GO:0007264,small GTPase mediated signal transduction|GO:0051179,localization|GO:1902578,single-organism localization|GO:0051641,cellular localization|GO:0040011,locomotion|GO:1901701,cellular response to oxygen-containing compound|GO:0048699,generation of neurons|GO:0032990,cell part morphogenesis|GO:0007399,nervous system development|GO:0051726,regulation of cell cycle|GO:0048856,anatomical structure development|GO:2000026,regulation of multicellular organismal development|GO:0015031,protein transport|GO:1902582,single-organism intracellular transport|GO:0006886,intracellular protein transport|GO:0048523,negative regulation of cellular process|GO:0048522,positive regulation of cellular process|GO:0071705,nitrogen compound transport|GO:0061154,endothelial tube morphogenesis|GO:0016192,vesicle-mediated transport|GO:0016477,cell migration|GO:0010631,epithelial cell migration|GO:0010632,regulation of epithelial cell migration|GO:0048870,cell motility|GO:0010634,positive regulation of epithelial cell migration|GO:0051674,localization of cell|GO:0043542,endothelial cell migration|GO:0009888,tissue development|GO:0003159,morphogenesis of an endothelium|GO:2000145,regulation of cell motility|GO:2000147,positive regulation of cell motility|GO:2000146,negative regulation of cell motility|GO:0042886,amide transport|GO:0015833,peptide transport|GO:0001667,ameboidal-type cell migration|GO:0035295,tube development|GO:0060562,epithelial tube morphogenesis|GO:0035239,tube morphogenesis|GO:0060429,epithelium development|GO:0040013,negative regulation of locomotion|GO:0051270,regulation of cellular component movement|GO:0003158,endothelium development|GO:0032879,regulation of localization|GO:0090132,epithelium migration|GO:0090130,tissue migration|GO:0002009,morphogenesis of an epithelium|GO:0048729,tissue morphogenesis|GO:0030336,negative regulation of cell migration|GO:0030335,positive regulation of cell migration|GO:0030334,regulation of cell migration|GO:0051272,positive regulation of cellular component movement|GO:0051271,negative regulation of cellular component movement|GO:0040012,regulation of locomotion|GO:0010595,positive regulation of endothelial cell migration|GO:0010594,regulation of endothelial cell migration|GO:0040017,positive regulation of locomotion|GO:0000278,mitotic cell cycle|GO:0007186,G-protein coupled receptor signaling	GO:0016020,membrane|GO:0044421,extracellular region part|GO:0043229,intracellular organelle|GO:0071944,cell periphery|GO:0005924,cell-substrate adherens junction|GO:0005925,focal adhesion|GO:0043227,membrane-bounded organelle|GO:0043226,organelle|GO:0030054,cell junction|GO:0010008,endosome membrane|GO:0005737,cytoplasm|GO:0097610,cell surface furrow|GO:0031982,vesicle|GO:0005634,nucleus|GO:0005770,late endosome|GO:0070062,extracellular exosome|GO:0031988,membrane-bounded vesicle|GO:0030055,cell-substrate junction|GO:0031902,late endosome membrane|GO:0098588,bounding membrane of organelle|GO:0070161,anchoring junction|GO:0032155,cell division site part|GO:0032154,cleavage furrow|GO:0032153,cell division site|GO:0005912,adherens junction|GO:0005886,plasma membrane|GO:0098805,whole membrane|GO:1903561,extracellular vesicle|GO:0009986,cell surface|GO:0031090,organelle membrane|GO:0043230,extracellular organelle|GO:0043231,intracellular membrane-bounded organelle|GO:0065010,extracellular membrane-bounded organelle|GO:0005829,cytosol|GO:0044464,cell part|GO:0005623,cell|GO:0005622,intracellular|GO:0044446,intracellular organelle part|GO:0044444,cytoplasmic part|GO:0005576,extracellular region|GO:0044440,endosomal part|GO:0044424,intracellular part|GO:0005768,endosome|GO:0005769,early endosome|GO:0044422,organelle part|GO:0012505,endomembrane system|GO:0031410,cytoplasmic vesicle|GO:0044433,cytoplasmic vesicle part|GO:0097708,intracellular vesicle	GO:0035639,purine ribonucleoside triphosphate binding|GO:0005488,binding|GO:0043167,ion binding|GO:1901363,heterocyclic compound binding|GO:1901265,nucleoside phosphate binding|GO:0001882,nucleoside binding|GO:0043168,anion binding|GO:0016462,pyrophosphatase activity|GO:0000166,nucleotide binding|GO:0032561,guanyl ribonucleotide binding|GO:0001883,purine nucleoside binding|GO:0032549,ribonucleoside binding|GO:0017076,purine nucleotide binding|GO:0005525,GTP binding|GO:0016787,hydrolase activity|GO:0003924,GTPase activity|GO:0017111,nucleoside-triphosphatase activity|GO:0036094,small molecule binding|GO:0003824,catalytic activity|GO:0016818,hydrolase activity, acting on acid anhydrides, in phosphorus-containing anhydrides|GO:0097367,carbohydrate derivative binding|GO:0097159,organic cyclic compound binding|GO:0016817,hydrolase activity, acting on acid anhydrides|GO:0019001,guanyl nucleotide binding|GO:0019003,GDP binding|GO:0005515,protein binding|GO:0032555,purine ribonucleotide binding|GO:0032550,purine ribonucleoside binding|GO:0032553,ribonucleotide binding
LELP1	GO:0018149,peptide cross-linking|GO:0030154,cell differentiation|GO:0006807,nitrogen compound metabolic process|GO:0008544,epidermis development|GO:0007275,multicellular organism development|GO:0044699,single-organism process|GO:0044267,cellular protein metabolic process|GO:0044260,cellular macromolecule metabolic process|GO:0048869,cellular developmental process|GO:0071704,organic substance metabolic process|GO:0048513,animal organ development|GO:0030855,epithelial cell differentiation|GO:0043588,skin development|GO:0032502,developmental process|GO:0032501,multicellular organismal process|GO:0030216,keratinocyte differentiation|GO:0060429,epithelium development|GO:0009888,tissue development|GO:0006464,cellular protein modification process|GO:0044767,single-organism developmental process|GO:0043412,macromolecule modification|GO:0036211,protein modification process|GO:0044763,single-organism cellular process|GO:0008152,metabolic process|GO:0044238,primary metabolic process|GO:1901564,organonitrogen compound metabolic process|GO:0044707,single-multicellular organism process|GO:0009913,epidermal cell differentiation|GO:0048856,anatomical structure development|GO:0044237,cellular metabolic process|GO:0043170,macromolecule metabolic process|GO:0019538,protein metabolic process|GO:0048731,system development|GO:0009987,cellular process	GO:0005737,cytoplasm|GO:0016020,membrane|GO:0044464,cell part|GO:0005623,cell|GO:0005622,intracellular|GO:0071944,cell periphery|GO:0005886,plasma membrane|GO:0044424,intracellular part|GO:0001533,cornified envelope	GO:0005198,structural molecule activity
PTS	GO:0006729,tetrahydrobiopterin biosynthetic process|GO:0019222,regulation of metabolic process|GO:0006732,coenzyme metabolic process|GO:0019752,carboxylic acid metabolic process|GO:2001057,reactive nitrogen species metabolic process|GO:0044249,cellular biosynthetic process|GO:0034641,cellular nitrogen compound metabolic process|GO:0006807,nitrogen compound metabolic process|GO:0009108,coenzyme biosynthetic process|GO:0044281,small molecule metabolic process|GO:0042559,pteridine-containing compound biosynthetic process|GO:1901362,organic cyclic compound biosynthetic process|GO:0007275,multicellular organism development|GO:0044699,single-organism process|GO:0007417,central nervous system development|GO:1901576,organic substance biosynthetic process|GO:0051341,regulation of oxidoreductase activity|GO:0051186,cofactor metabolic process|GO:0006520,cellular amino acid metabolic process|GO:0051188,cofactor biosynthetic process|GO:0042558,pteridine-containing compound metabolic process|GO:0050789,regulation of biological process|GO:0071704,organic substance metabolic process|GO:0065007,biological regulation|GO:1901360,organic cyclic compound metabolic process|GO:0065009,regulation of molecular function|GO:1901566,organonitrogen compound biosynthetic process|GO:0018130,heterocycle biosynthetic process|GO:0032502,developmental process|GO:0032501,multicellular organismal process|GO:0032768,regulation of monooxygenase activity|GO:0050790,regulation of catalytic activity|GO:0006725,cellular aromatic compound metabolic process|GO:0044710,single-organism metabolic process|GO:0072593,reactive oxygen species metabolic process|GO:0044767,single-organism developmental process|GO:0009058,biosynthetic process|GO:0046146,tetrahydrobiopterin metabolic process|GO:0044763,single-organism cellular process|GO:0008152,metabolic process|GO:0019438,aromatic compound biosynthetic process|GO:0043436,oxoacid metabolic process|GO:1901564,organonitrogen compound metabolic process|GO:0046483,heterocycle metabolic process|GO:0050999,regulation of nitric-oxide synthase activity|GO:0044238,primary metabolic process|GO:0044271,cellular nitrogen compound biosynthetic process|GO:0044707,single-multicellular organism process|GO:0006082,organic acid metabolic process|GO:0048856,anatomical structure development|GO:0007399,nervous system development|GO:0044237,cellular metabolic process|GO:0048731,system development|GO:0009987,cellular process|GO:0046209,nitric oxide metabolic process	GO:0005737,cytoplasm|GO:0043231,intracellular membrane-bounded organelle|GO:0005829,cytosol|GO:0044464,cell part|GO:0044444,cytoplasmic part|GO:0005739,mitochondrion|GO:0005622,intracellular|GO:0043229,intracellular organelle|GO:0005623,cell|GO:0044424,intracellular part|GO:0043227,membrane-bounded organelle|GO:0043226,organelle	GO:0005488,binding|GO:0043169,cation binding|GO:0016838,carbon-oxygen lyase activity, acting on phosphates|GO:0016835,carbon-oxygen lyase activity|GO:0016829,lyase activity|GO:0043167,ion binding|GO:0003824,catalytic activity|GO:0042802,identical protein binding|GO:0005515,protein binding|GO:0046983,protein dimerization activity|GO:0046872,metal ion binding|GO:0003874,6-pyruvoyltetrahydropterin synthase activity|GO:0042803,protein homodimerization activity
TBC1D17	GO:0022604,regulation of cell morphogenesis|GO:0022607,cellular component assembly|GO:0051234,establishment of localization|GO:0009893,positive regulation of metabolic process|GO:0019222,regulation of metabolic process|GO:0022603,regulation of anatomical structure morphogenesis|GO:0030030,cell projection organization|GO:0030031,cell projection assembly|GO:0006810,transport|GO:0051128,regulation of cellular component organization|GO:0044248,cellular catabolic process|GO:0060491,regulation of cell projection assembly|GO:0008152,metabolic process|GO:0051641,cellular localization|GO:0051179,localization|GO:0032989,cellular component morphogenesis|GO:0043087,regulation of GTPase activity|GO:0050789,regulation of biological process|GO:0043085,positive regulation of catalytic activity|GO:0000902,cell morphogenesis|GO:0070925,organelle assembly|GO:0043547,positive regulation of GTPase activity|GO:0051345,positive regulation of hydrolase activity|GO:0009653,anatomical structure morphogenesis|GO:0048869,cellular developmental process|GO:0071840,cellular component organization or biogenesis|GO:0033043,regulation of organelle organization|GO:0016043,cellular component organization|GO:0031344,regulation of cell projection organization|GO:0008104,protein localization|GO:0065007,biological regulation|GO:1902115,regulation of organelle assembly|GO:0044093,positive regulation of molecular function|GO:0071702,organic substance transport|GO:0048518,positive regulation of biological process|GO:1902017,regulation of cilium assembly|GO:0033036,macromolecule localization|GO:0048646,anatomical structure formation involved in morphogenesis|GO:0032502,developmental process|GO:0016197,endosomal transport|GO:0042384,cilium assembly|GO:0060271,cilium morphogenesis|GO:0050793,regulation of developmental process|GO:0006914,autophagy|GO:0050790,regulation of catalytic activity|GO:0050794,regulation of cellular process|GO:0044767,single-organism developmental process|GO:0044765,single-organism transport|GO:0044763,single-organism cellular process|GO:0042147,retrograde transport, endosome to Golgi|GO:0051649,establishment of localization in cell|GO:0010927,cellular component assembly involved in morphogenesis|GO:0009056,catabolic process|GO:0065009,regulation of molecular function|GO:1902578,single-organism localization|GO:0032990,cell part morphogenesis|GO:0051336,regulation of hydrolase activity|GO:0045184,establishment of protein localization|GO:0044699,single-organism process|GO:0048858,cell projection morphogenesis|GO:0046907,intracellular transport|GO:0048856,anatomical structure development|GO:0044237,cellular metabolic process|GO:0044087,regulation of cellular component biogenesis|GO:1902589,single-organism organelle organization|GO:0044085,cellular component biogenesis|GO:0006996,organelle organization|GO:0015031,protein transport|GO:1902582,single-organism intracellular transport|GO:0009987,cellular process|GO:0044782,cilium organization|GO:0016192,vesicle-mediated transport|GO:0051049,regulation of transport|GO:0006906,vesicle fusion|GO:0061025,membrane fusion|GO:0061024,membrane organization|GO:0070727,cellular macromolecule localization|GO:0090174,organelle membrane fusion|GO:0016050,vesicle organization|GO:0006886,intracellular protein transport|GO:0044802,single-organism membrane organization|GO:0044801,single-organism membrane fusion|GO:0015833,peptide transport|GO:0042886,amide transport|GO:0031338,regulation of vesicle fusion|GO:0060627,regulation of vesicle-mediated transport|GO:0032879,regulation of localization|GO:0016482,cytosolic transport|GO:0090630,activation of GTPase activity|GO:0071705,nitrogen compound transport|GO:0034613,cellular protein	GO:0005737,cytoplasm|GO:0031982,vesicle|GO:0005773,vacuole|GO:0031410,cytoplasmic vesicle|GO:0005829,cytosol|GO:0044444,cytoplasmic part|GO:0044464,cell part|GO:0005776,autophagosome|GO:0005623,cell|GO:0005622,intracellular|GO:0043229,intracellular organelle|GO:0044424,intracellular part|GO:0043227,membrane-bounded organelle|GO:0043226,organelle|GO:0043231,intracellular membrane-bounded organelle|GO:0055037,recycling endosome|GO:0012505,endomembrane system|GO:0097708,intracellular vesicle|GO:0005768,endosome	GO:0030234,enzyme regulator activity|GO:0005096,GTPase activator activity|GO:0030695,GTPase regulator activity|GO:0008047,enzyme activator activity|GO:0005515,protein binding|GO:0098772,molecular function regulator|GO:0060589,nucleoside-triphosphatase regulator activity|GO:0005488,binding|GO:0031267,small GTPase binding|GO:0017137,Rab GTPase binding|GO:0019899,enzyme binding|GO:0017016,Ras GTPase binding|GO:0051020,GTPase binding
WDR62	GO:0021537,telencephalon development|GO:0044707,single-multicellular organism process|GO:0043933,macromolecular complex subunit organization|GO:0030154,cell differentiation|GO:1903047,mitotic cell cycle process|GO:0060322,head development|GO:0000226,microtubule cytoskeleton organization|GO:0007275,multicellular organism development|GO:0007049,cell cycle|GO:0007417,central nervous system development|GO:0071822,protein complex subunit organization|GO:0048869,cellular developmental process|GO:0071840,cellular component organization or biogenesis|GO:0016043,cellular component organization|GO:0021987,cerebral cortex development|GO:0048513,animal organ development|GO:0044699,single-organism process|GO:0032502,developmental process|GO:0032501,multicellular organismal process|GO:0000278,mitotic cell cycle|GO:0009987,cellular process|GO:0044767,single-organism developmental process|GO:0007051,spindle organization|GO:0007052,mitotic spindle organization|GO:0044763,single-organism cellular process|GO:0048731,system development|GO:0022008,neurogenesis|GO:0006996,organelle organization|GO:0007017,microtubule-based process|GO:0007010,cytoskeleton organization|GO:0007420,brain development|GO:0007399,nervous system development|GO:0021543,pallium development|GO:0048856,anatomical structure development|GO:1902589,single-organism organelle organization|GO:0030900,forebrain development|GO:0022402,cell cycle process|GO:0022607,cellular component assembly|GO:0006807,nitrogen compound metabolic process|GO:0090304,nucleic acid metabolic process|GO:0034641,cellular nitrogen compound metabolic process|GO:0098534,centriole assembly|GO:0007067,mitotic nuclear division|GO:0006139,nucleobase-containing compound metabolic process|GO:0044260,cellular macromolecule metabolic process|GO:1901360,organic cyclic compound metabolic process|GO:0000280,nuclear division|GO:0071704,organic substance metabolic process|GO:0010467,gene expression|GO:1902850,microtubule cytoskeleton organization involved in mitosis|GO:0046483,heterocycle metabolic process|GO:0008380,RNA splicing|GO:0048285,organelle fission|GO:0016070,RNA metabolic process|GO:0031023,microtubule organizing center organization|GO:0051298,centrosome duplication|GO:0051297,centrosome organization|GO:0006725,cellular aromatic compound metabolic process|GO:0008152,metabolic process|GO:0070925,organelle assembly|GO:0044238,primary metabolic process|GO:0044237,cellular metabolic process|GO:0043170,macromolecule metabolic process|GO:0044085,cellular component biogenesis|GO:0007098,centrosome cycle|GO:0007099,centriole replication|GO:0006396,RNA processing	GO:0031974,membrane-enclosed lumen|GO:0043229,intracellular organelle|GO:0043228,non-membrane-bounded organelle|GO:0043227,membrane-bounded organelle|GO:0043226,organelle|GO:0005856,cytoskeleton|GO:0005819,spindle|GO:0031981,nuclear lumen|GO:0005634,nucleus|GO:0000922,spindle pole|GO:0005654,nucleoplasm|GO:0044430,cytoskeletal part|GO:0005737,cytoplasm|GO:0015630,microtubule cytoskeleton|GO:0043231,intracellular membrane-bounded organelle|GO:0043232,intracellular non-membrane-bounded organelle|GO:0043233,organelle lumen|GO:0044464,cell part|GO:0005623,cell|GO:0005622,intracellular|GO:0044446,intracellular organelle part|GO:0070013,intracellular organelle lumen|GO:0044428,nuclear part|GO:0044424,intracellular part|GO:0044422,organelle part|GO:0097525,spliceosomal snRNP complex|GO:0005813,centrosome|GO:0005814,centriole|GO:0005815,microtubule organizing center|GO:0044450,microtubule organizing center part|GO:0030529,intracellular ribonucleoprotein complex|GO:0071011,precatalytic spliceosome|GO:0071013,catalytic step 2 spliceosome|GO:0032991,macromolecular complex|GO:0030532,small nuclear ribonucleoprotein complex|GO:1990904,ribonucleoprotein	GO:0005488,binding|GO:0005515,protein binding|GO:0003676,nucleic acid binding|GO:0097159,organic cyclic compound binding|GO:1901363,heterocyclic compound binding|GO:0003723,RNA binding
LACC1		GO:0005737,cytoplasm|GO:0043231,intracellular membrane-bounded organelle|GO:0044464,cell part|GO:0005777,peroxisome|GO:0044444,cytoplasmic part|GO:0005623,cell|GO:0005622,intracellular|GO:0042579,microbody|GO:0043229,intracellular organelle|GO:0044424,intracellular part|GO:0043227,membrane-bounded organelle|GO:0043226,organelle	GO:0043169,cation binding|GO:0005507,copper ion binding|GO:0046914,transition metal ion binding|GO:0043167,ion binding|GO:0005488,binding|GO:0005515,protein binding|GO:0046872,metal ion binding
GTSF1	GO:0032502,developmental process|GO:0044703,multi-organism reproductive process|GO:0044702,single organism reproductive process|GO:0048609,multicellular organismal reproductive process|GO:0032504,multicellular organism reproduction|GO:0048869,cellular developmental process|GO:0030154,cell differentiation|GO:0019953,sexual reproduction|GO:0044767,single-organism developmental process|GO:0022414,reproductive process|GO:0007283,spermatogenesis|GO:0032501,multicellular organismal process|GO:0044763,single-organism cellular process|GO:0044699,single-organism process|GO:0048232,male gamete generation|GO:0009987,cellular process|GO:0051704,multi-organism process|GO:0007276,gamete generation|GO:0000003,reproduction	GO:0005737,cytoplasm|GO:0044464,cell part|GO:0005623,cell|GO:0005622,intracellular|GO:0044424,intracellular part	GO:0043169,cation binding|GO:0046872,metal ion binding|GO:0005488,binding|GO:0043167,ion binding|GO:0097159,organic cyclic compound binding|GO:0003676,nucleic acid binding|GO:1901363,heterocyclic compound binding
GALNT18	GO:0044249,cellular biosynthetic process|GO:0034645,cellular macromolecule biosynthetic process|GO:0009100,glycoprotein metabolic process|GO:0009101,glycoprotein biosynthetic process|GO:0044699,single-organism process|GO:0044267,cellular protein metabolic process|GO:0044710,single-organism metabolic process|GO:0044260,cellular macromolecule metabolic process|GO:0071704,organic substance metabolic process|GO:1901576,organic substance biosynthetic process|GO:0009987,cellular process|GO:0070085,glycosylation|GO:0006464,cellular protein modification process|GO:0043412,macromolecule modification|GO:0036211,protein modification process|GO:0044763,single-organism cellular process|GO:0008152,metabolic process|GO:0044723,single-organism carbohydrate metabolic process|GO:0009059,macromolecule biosynthetic process|GO:0044238,primary metabolic process|GO:0005975,carbohydrate metabolic process|GO:0006486,protein glycosylation|GO:0019538,protein metabolic process|GO:1901135,carbohydrate derivative metabolic process|GO:0009058,biosynthetic process|GO:0044237,cellular metabolic process|GO:0043170,macromolecule metabolic process|GO:1901137,carbohydrate derivative biosynthetic process|GO:0043413,macromolecule glycosylation|GO:0006807,nitrogen compound metabolic process|GO:1901564,organonitrogen compound metabolic process|GO:1901566,organonitrogen compound biosynthetic process|GO:0006493,protein O-linked glycosylation	GO:0043229,intracellular organelle|GO:0005622,intracellular|GO:0043227,membrane-bounded organelle|GO:0043226,organelle|GO:0031224,intrinsic component of membrane|GO:0005737,cytoplasm|GO:0031090,organelle membrane|GO:0016021,integral component of membrane|GO:0016020,membrane|GO:0044431,Golgi apparatus part|GO:0005794,Golgi apparatus|GO:0098588,bounding membrane of organelle|GO:0012505,endomembrane system|GO:0043231,intracellular membrane-bounded organelle|GO:0044464,cell part|GO:0005623,cell|GO:0000139,Golgi membrane|GO:0044446,intracellular organelle part|GO:0044444,cytoplasmic part|GO:0044424,intracellular part|GO:0044425,membrane part|GO:0044422,organelle part|GO:0031984,organelle subcompartment|GO:0098791,Golgi subcompartment	GO:0043169,cation binding|GO:0004653,polypeptide N-acetylgalactosaminyltransferase activity|GO:0030246,carbohydrate binding|GO:0043167,ion binding|GO:0003824,catalytic activity|GO:0016740,transferase activity|GO:0005488,binding|GO:0016757,transferase activity, transferring glycosyl groups|GO:0016758,transferase activity, transferring hexosyl groups|GO:0008376,acetylgalactosaminyltransferase activity|GO:0046872,metal ion binding|GO:0008194,UDP-glycosyltransferase activity
SLC35E3	GO:1901679,nucleotide transmembrane transport|GO:0006810,transport|GO:0071702,organic substance transport|GO:0015931,nucleobase-containing compound transport|GO:0006811,ion transport|GO:0009987,cellular process|GO:0006862,nucleotide transport|GO:0044765,single-organism transport|GO:0051179,localization|GO:0071705,nitrogen compound transport|GO:0034220,ion transmembrane transport|GO:0006820,anion transport|GO:0044763,single-organism cellular process|GO:0044699,single-organism process|GO:0015748,organophosphate ester transport|GO:0098656,anion transmembrane transport|GO:0051234,establishment of localization|GO:0055085,transmembrane transport|GO:1902578,single-organism localization|GO:1901264,carbohydrate derivative transport	GO:0044425,membrane part|GO:0016021,integral component of membrane|GO:0016020,membrane|GO:0031224,intrinsic component of membrane	GO:0022891,substrate-specific transmembrane transporter activity|GO:0015932,nucleobase-containing compound transmembrane transporter activity|GO:0022892,substrate-specific transporter activity|GO:0015291,secondary active transmembrane transporter activity|GO:0015215,nucleotide transmembrane transporter activity|GO:0005215,transporter activity|GO:0005338,nucleotide-sugar transmembrane transporter activity|GO:0015297,antiporter activity|GO:0015605,organophosphate ester transmembrane transporter activity|GO:0022857,transmembrane transporter activity|GO:1901505,carbohydrate derivative transporter activity|GO:1901677,phosphate transmembrane transporter activity|GO:0022804,active transmembrane transporter activity
C1orf213			
TNFAIP8L3	GO:0009987,cellular process|GO:0006915,apoptotic process|GO:0050794,regulation of cellular process|GO:0012501,programmed cell death|GO:0043067,regulation of programmed cell death|GO:0065007,biological regulation|GO:0044763,single-organism cellular process|GO:0008219,cell death|GO:0010941,regulation of cell death|GO:0042981,regulation of apoptotic process|GO:0050789,regulation of biological process|GO:0044699,single-organism process	GO:0005737,cytoplasm|GO:0044446,intracellular organelle part|GO:0005829,cytosol|GO:0043231,intracellular membrane-bounded organelle|GO:0031981,nuclear lumen|GO:0043233,organelle lumen|GO:0005634,nucleus|GO:0044464,cell part|GO:0044444,cytoplasmic part|GO:0031974,membrane-enclosed lumen|GO:0005622,intracellular|GO:0005654,nucleoplasm|GO:0070013,intracellular organelle lumen|GO:0043229,intracellular organelle|GO:0044428,nuclear part|GO:0005623,cell|GO:0044424,intracellular part|GO:0043227,membrane-bounded organelle|GO:0043226,organelle|GO:0044422,organelle part	
STATH	GO:0032502,developmental process|GO:0009617,response to bacterium|GO:0022600,digestive system process|GO:0032941,secretion by tissue|GO:0042742,defense response to bacterium|GO:0030500,regulation of bone mineralization|GO:0030502,negative regulation of bone mineralization|GO:0007275,multicellular organism development|GO:0044699,single-organism process|GO:0051234,establishment of localization|GO:0043207,response to external biotic stimulus|GO:0051241,negative regulation of multicellular organismal process|GO:0030282,bone mineralization|GO:0007586,digestion|GO:0048513,animal organ development|GO:0065007,biological regulation|GO:0048519,negative regulation of biological process|GO:0051704,multi-organism process|GO:0065008,regulation of biological quality|GO:0046541,saliva secretion|GO:0070168,negative regulation of biomineral tissue development|GO:0032501,multicellular organismal process|GO:0050878,regulation of body fluid levels|GO:0007589,body fluid secretion|GO:0050793,regulation of developmental process|GO:0006810,transport|GO:0070167,regulation of biomineral tissue development|GO:0009888,tissue development|GO:0044767,single-organism developmental process|GO:0044765,single-organism transport|GO:0098542,defense response to other organism|GO:0051239,regulation of multicellular organismal process|GO:0031214,biomineral tissue development|GO:0030278,regulation of ossification|GO:0030279,negative regulation of ossification|GO:0051707,response to other organism|GO:0051179,localization|GO:1902578,single-organism localization|GO:0003008,system process|GO:0051093,negative regulation of developmental process|GO:0006952,defense response|GO:0046903,secretion|GO:0009607,response to biotic stimulus|GO:0009605,response to external stimulus|GO:0044707,single-multicellular organism process|GO:0050896,response to stimulus|GO:0048856,anatomical structure development|GO:0006950,response to stress|GO:0050789,regulation of biological process|GO:0001503,ossification|GO:2000026,regulation of multicellular organismal development|GO:0048731,system development	GO:0005576,extracellular region	GO:0005201,extracellular matrix structural constituent|GO:0030021,extracellular matrix structural constituent conferring compression resistance|GO:0005198,structural molecule activity|GO:0046848,hydroxyapatite binding|GO:0005488,binding|GO:0005515,protein binding|GO:0030345,structural constituent of tooth enamel|GO:0030197,extracellular matrix constituent, lubricant activity
SLFN12L		GO:0044425,membrane part|GO:0016021,integral component of membrane|GO:0016020,membrane|GO:0031224,intrinsic component of membrane	GO:0043168,anion binding|GO:0005488,binding|GO:0030554,adenyl nucleotide binding|GO:0035639,purine ribonucleoside triphosphate binding|GO:0097367,carbohydrate derivative binding|GO:1901363,heterocyclic compound binding|GO:1901265,nucleoside phosphate binding|GO:0000166,nucleotide binding|GO:0036094,small molecule binding|GO:0032553,ribonucleotide binding|GO:0032559,adenyl ribonucleotide binding|GO:0005524,ATP binding|GO:0032549,ribonucleoside binding|GO:0032555,purine ribonucleotide binding|GO:0017076,purine nucleotide binding|GO:0043167,ion binding|GO:0097159,organic cyclic compound binding|GO:0032550,purine ribonucleoside binding|GO:0001883,purine nucleoside

CWH43	GO:0006650,glycerophospholipid metabolic process|GO:0044249,cellular biosynthetic process|GO:0042157,lipoprotein metabolic process|GO:0034645,cellular macromolecule biosynthetic process|GO:1901576,organic substance biosynthetic process|GO:0042158,lipoprotein biosynthetic process|GO:0044255,cellular lipid metabolic process|GO:0044699,single-organism process|GO:0006629,lipid metabolic process|GO:0045017,glycerolipid biosynthetic process|GO:0044710,single-organism metabolic process|GO:0044711,single-organism biosynthetic process|GO:0044260,cellular macromolecule metabolic process|GO:0090407,organophosphate biosynthetic process|GO:0071704,organic substance metabolic process|GO:0006505,GPI anchor metabolic process|GO:0006506,GPI anchor biosynthetic process|GO:0046467,membrane lipid biosynthetic process|GO:0006664,glycolipid metabolic process|GO:1903509,liposaccharide metabolic process|GO:0006644,phospholipid metabolic process|GO:0006643,membrane lipid metabolic process|GO:0044267,cellular protein metabolic process|GO:0009987,cellular process|GO:0009247,glycolipid biosynthetic process|GO:0006464,cellular protein modification process|GO:0043412,macromolecule modification|GO:0036211,protein modification process|GO:0046488,phosphatidylinositol metabolic process|GO:0008152,metabolic process|GO:0046474,glycerophospholipid biosynthetic process|GO:0046486,glycerolipid metabolic process|GO:0006661,phosphatidylinositol biosynthetic process|GO:0008610,lipid biosynthetic process|GO:0006497,protein lipidation|GO:0044238,primary metabolic process|GO:0008654,phospholipid biosynthetic process|GO:0019538,protein metabolic process|GO:1901135,carbohydrate derivative metabolic process|GO:0009058,biosynthetic process|GO:0044237,cellular metabolic process|GO:0043170,macromolecule metabolic process|GO:1901137,carbohydrate derivative biosynthetic process|GO:0006796,phosphate-containing compound metabolic process|GO:0009059,macromolecule biosynthetic process|GO:0006793,phosphorus metabolic process|GO:0019637,organophosphate metabolic process|GO:0044763,single-organism cellular process|GO:0006807,nitrogen compound metabolic process|GO:1901564,organonitrogen compound metabolic process|GO:1901566,organonitrogen compound biosynthetic process	GO:0044425,membrane part|GO:0016021,integral component of membrane|GO:0016020,membrane|GO:0031224,intrinsic component of membrane	
HMSD	GO:0009892,negative regulation of metabolic process|GO:0080090,regulation of primary metabolic process|GO:0019222,regulation of metabolic process|GO:0031324,negative regulation of cellular metabolic process|GO:0031323,regulation of cellular metabolic process|GO:0043086,negative regulation of catalytic activity|GO:0044267,cellular protein metabolic process|GO:0051248,negative regulation of protein metabolic process|GO:0010605,negative regulation of macromolecule metabolic process|GO:0051346,negative regulation of hydrolase activity|GO:0044260,cellular macromolecule metabolic process|GO:0071704,organic substance metabolic process|GO:0051246,regulation of protein metabolic process|GO:0050789,regulation of biological process|GO:0060255,regulation of macromolecule metabolic process|GO:0010466,negative regulation of peptidase activity|GO:0065007,biological regulation|GO:0044092,negative regulation of molecular function|GO:0048519,negative regulation of biological process|GO:0065009,regulation of molecular function|GO:0009987,cellular process|GO:0052547,regulation of peptidase activity|GO:0052548,regulation of endopeptidase activity|GO:0050790,regulation of catalytic activity|GO:0050794,regulation of cellular process|GO:0030162,regulation of proteolysis|GO:0008152,metabolic process|GO:0006508,proteolysis|GO:0010951,negative regulation of endopeptidase activity|GO:0051336,regulation of hydrolase activity|GO:0044238,primary metabolic process|GO:0032269,negative regulation of cellular protein metabolic process|GO:0032268,regulation of cellular protein metabolic process|GO:0019538,protein metabolic process|GO:0044237,cellular metabolic process|GO:0043170,macromolecule metabolic process|GO:0045861,negative regulation of proteolysis|GO:0048523,negative regulation of cellular process|GO:0006955,immune response|GO:0002684,positive regulation of immune system process|GO:0002682,regulation of immune system process|GO:0048518,positive regulation of biological process|GO:0048584,positive regulation of response to stimulus|GO:0048583,regulation of response to stimulus|GO:0050776,regulation of immune response|GO:1901564,organonitrogen compound metabolic process|GO:0050896,response to stimulus|GO:0050778,positive regulation of immune response|GO:0002376,immune system process|GO:0006807,nitrogen	GO:0005737,cytoplasm|GO:0005615,extracellular space|GO:0044464,cell part|GO:0005623,cell|GO:0005622,intracellular|GO:0005576,extracellular region|GO:0044424,intracellular part|GO:0044421,extracellular region part	GO:0004866,endopeptidase inhibitor activity|GO:0061135,endopeptidase regulator activity|GO:0061134,peptidase regulator activity|GO:0030414,peptidase inhibitor activity|GO:0004867,serine-type endopeptidase inhibitor activity|GO:0004857,enzyme inhibitor activity|GO:0098772,molecular function regulator|GO:0030234,enzyme regulator activity
IFNK	GO:0019220,regulation of phosphate metabolic process|GO:0019221,cytokine-mediated signaling pathway|GO:0019222,regulation of metabolic process|GO:0048584,positive regulation of response to stimulus|GO:0048583,regulation of response to stimulus|GO:0031349,positive regulation of defense response|GO:0007165,signal transduction|GO:0007166,cell surface receptor signaling pathway|GO:1901576,organic substance biosynthetic process|GO:1901362,organic cyclic compound biosynthetic process|GO:0031347,regulation of defense response|GO:0080090,regulation of primary metabolic process|GO:0051716,cellular response to stimulus|GO:0010604,positive regulation of macromolecule metabolic process|GO:0043207,response to external biotic stimulus|GO:0009966,regulation of signal transduction|GO:0048869,cellular developmental process|GO:0002263,cell activation involved in immune response|GO:0010467,gene expression|GO:0018193,peptidyl-amino acid modification|GO:0010033,response to organic substance|GO:0048513,animal organ development|GO:0048518,positive regulation of biological process|GO:0048519,negative regulation of biological process|GO:0051704,multi-organism process|GO:0042127,regulation of cell proliferation|GO:0033141,positive regulation of peptidyl-serine phosphorylation of STAT protein|GO:0060255,regulation of macromolecule metabolic process|GO:0042221,response to chemical|GO:0032268,regulation of cellular protein metabolic process|GO:2001141,regulation of RNA biosynthetic process|GO:0009607,response to biotic stimulus|GO:0051707,response to other organism|GO:0002323,natural killer cell activation involved in immune response|GO:0046483,heterocycle metabolic process|GO:0042325,regulation of phosphorylation|GO:0044700,single organism signaling|GO:0042327,positive regulation of phosphorylation|GO:0018209,peptidyl-serine modification|GO:0009605,response to external stimulus|GO:0044707,single-multicellular organism process|GO:0019538,protein metabolic process|GO:0002376,immune system process|GO:0043331,response to dsRNA|GO:0043330,response to exogenous dsRNA|GO:0019438,aromatic compound biosynthetic process|GO:0050896,response to stimulus|GO:0009893,positive regulation of metabolic process|GO:0006950,response to stress|GO:0032943,mononuclear cell proliferation|GO:0014070,response to organic cyclic compound|GO:0065007,biological regulation|GO:0046651,lymphocyte proliferation|GO:0035556,intracellular signal transduction|GO:0050789,regulation of biological process|GO:0097659,nucleic acid-templated transcription|GO:0044267,cellular protein metabolic process|GO:0002286,T cell activation involved in immune response|GO:0044260,cellular macromolecule metabolic process|GO:0002285,lymphocyte activation involved in immune response|GO:0046425,regulation of JAK-STAT cascade|GO:0002684,positive regulation of immune system process|GO:0002366,leukocyte activation involved in immune response|GO:0002682,regulation of immune system process|GO:1901360,organic cyclic compound metabolic process|GO:0071593,lymphocyte aggregation|GO:0018130,heterocycle biosynthetic process|GO:0098602,single organism cell adhesion|GO:0034097,response to cytokine|GO:0098609,cell-cell adhesion|GO:0009889,regulation of biosynthetic process|GO:0050794,regulation of cellular process|GO:0006952,defense response|GO:0001775,cell activation|GO:0036211,protein modification process|GO:0008152,metabolic process|GO:0048731,system development|GO:0034654,nucleobase-containing compound biosynthetic process|GO:1902531,regulation of intracellular signal transduction|GO:0002521,leukocyte differentiation|GO:0002520,immune system development|GO:0016070,RNA metabolic process|GO:0044767,single-organism developmental process|GO:0044271,cellular nitrogen compound biosynthetic process|GO:0051607,defense response to virus|GO:0071345,cellular response to cytokine stimulus|GO:0080134,regulation of response to stress|GO:0031401,positive regulation of protein modification process|GO:0043412,macromolecule modification|GO:0006355,regulation of transcription, DNA-templated|GO:0010556,regulation of macromolecule biosynthetic process|GO:0006351,transcription, DNA-templated|GO:0009615,response to virus|GO:0007159,leukocyte cell-cell adhesion|GO:0032774,RNA biosynthetic process|GO:0016310,phosphorylation|GO:0030154,cell differentiation|GO:0031326,regulation of cellular biosynthetic process|GO:0044249,cellular biosynthetic process|GO:0034641,cellular nitrogen compound metabolic process|GO:0023052,signaling|GO:0070887,cellular response to chemical stimulus|GO:0023051,regulation of signaling|GO:0016337,single organismal cell-cell adhesion|GO:0010646,regulation of cell communication|GO:0044699,single-organism process|GO:0006139,nucleobase-containing compound metabolic process|GO:0006959,humoral immune response|GO:0010562,positive regulation of phosphorus metabolic process|GO:0051246,regulation of protein metabolic process|GO:0051247,positive regulation of protein metabolic process|GO:0007259,JAK-STAT cascade|GO:0032270,positive regulation of cellular protein metabolic process|GO:0031399,regulation of protein modification process|GO:0022610,biological adhesion|GO:0032502,developmental process|GO:0008285,negative regulation of cell proliferation|GO:0032501,multicellular organismal process|GO:0008283,cell proliferation|GO:0009987,cellular process|GO:0006725,cellular aromatic compound metabolic process|GO:1903506,regulation of nucleic acid-templated transcription|GO:0034645,cellular macromolecule biosynthetic process|GO:0098542,defense response to other organism|GO:0006955,immune response|GO:0050776,regulation of immune response|GO:0045321,leukocyte activation|GO:0051252,regulation of RNA metabolic process|GO:0044763,single-organism cellular process|GO:0050778,positive regulation of immune response|GO:0043170,macromolecule metabolic process|GO:0006807,nitrogen compound metabolic process|GO:0030098,lymphocyte differentiation|GO:0030097,hemopoiesis|GO:1901698,response to nitrogen compound|GO:0018105,peptidyl-serine phosphorylation|GO:0033138,positive regulation of peptidyl-serine phosphorylation|GO:0033139,regulation of peptidyl-serine phosphorylation of STAT protein|GO:0033135,regulation of peptidyl-serine phosphorylation|GO:0031325,positive regulation of cellular metabolic process|GO:0031323,regulation of cellular metabolic process|GO:0042113,B cell activation|GO:0042110,T cell activation|GO:0090304,nucleic acid metabolic process|GO:0007275,multicellular organism development|GO:2000112,regulation of cellular macromolecule biosynthetic process|GO:0070661,leukocyte proliferation|GO:0071704,organic substance metabolic process|GO:0071310,cellular response to organic substance|GO:0048534,hematopoietic or lymphoid organ development|GO:0010468,regulation of gene expression|GO:0006468,protein phosphorylation|GO:0045089,positive regulation of innate immune response|GO:0045088,regulation of innate immune response|GO:0045937,positive regulation of phosphate metabolic process|GO:0030183,B cell differentiation|GO:0019219,regulation of nucleobase-containing compound metabolic process|GO:0034109,homotypic cell-cell adhesion|GO:0045087,innate immune response|GO:0002252,immune effector process|GO:0042100,B cell proliferation|GO:0006464,cellular protein modification process|GO:0051174,regulation of phosphorus metabolic process|GO:0009058,biosynthetic process|GO:0009059,macromolecule biosynthetic process|GO:0070489,T cell aggregation|GO:0051171,regulation of nitrogen compound metabolic process|GO:0007155,cell adhesion|GO:0007154,cell communication|GO:0070486,leukocyte aggregation|GO:0030101,natural killer cell activation|GO:0044238,primary metabolic process|GO:0042501,serine phosphorylation of STAT protein|GO:0048856,anatomical structure development|GO:0044237,cellular metabolic process|GO:0006796,phosphate-containing compound metabolic process|GO:0046649,lymphocyte activation|GO:0002250,adaptive immune response|GO:0006793,phosphorus metabolic process|GO:0001932,regulation of protein phosphorylation|GO:0001934,positive regulation of protein phosphorylation|GO:0048523,negative regulation of cellular process|GO:0048522,positive regulation of cellular process|GO:1901564,organonitrogen compound metabolic process|GO:1904892,regulation of STAT cascade|GO:0097696,STAT cascade	GO:0005576,extracellular region|GO:0005615,extracellular space|GO:0044421,extracellular region part	GO:0005126,cytokine receptor binding|GO:0005125,cytokine activity|GO:0005132,type I interferon receptor binding|GO:0044877,macromolecular complex binding|GO:0005488,binding|GO:0005515,protein binding|GO:0032403,protein complex binding|GO:0005102,receptor binding
C1orf222			
SLC35G1	GO:0050801,ion homeostasis|GO:0042592,homeostatic process|GO:0098771,inorganic ion homeostasis|GO:0055074,calcium ion homeostasis|GO:1990034,calcium ion export from cell|GO:0044699,single-organism process|GO:0072507,divalent inorganic cation homeostasis|GO:0072503,cellular divalent inorganic cation homeostasis|GO:1901660,calcium ion export|GO:0065007,biological regulation|GO:0006812,cation transport|GO:0065008,regulation of biological quality|GO:0019725,cellular homeostasis|GO:0006810,transport|GO:0006875,cellular metal ion homeostasis|GO:0006874,cellular calcium ion homeostasis|GO:0006811,ion transport|GO:0009987,cellular process|GO:0006873,cellular ion homeostasis|GO:0044765,single-organism transport|GO:0044763,single-organism cellular process|GO:0030003,cellular cation homeostasis|GO:0055065,metal ion homeostasis|GO:0055080,cation homeostasis|GO:0055082,cellular chemical homeostasis|GO:0051234,establishment of localization|GO:0051179,localization|GO:1902578,single-organism localization|GO:0051480,regulation of cytosolic calcium ion concentration|GO:0048878,chemical homeostasis|GO:0030001,metal ion transport|GO:0006816,calcium ion transport|GO:0072511,divalent inorganic cation transport|GO:0070838,divalent metal ion transport	GO:0005783,endoplasmic reticulum|GO:0005789,endoplasmic reticulum membrane|GO:0042175,nuclear outer membrane-endoplasmic reticulum membrane network|GO:0043229,intracellular organelle|GO:0071944,cell periphery|GO:0043227,membrane-bounded organelle|GO:0043226,organelle|GO:0031224,intrinsic component of membrane|GO:0005737,cytoplasm|GO:0031090,organelle membrane|GO:0016021,integral component of membrane|GO:0016020,membrane|GO:0044432,endoplasmic reticulum part|GO:0098588,bounding membrane of organelle|GO:0012505,endomembrane system|GO:0005886,plasma membrane|GO:0043231,intracellular membrane-bounded organelle|GO:0005829,cytosol|GO:0044464,cell part|GO:0005623,cell|GO:0005622,intracellular|GO:0044446,intracellular organelle part|GO:0044444,cytoplasmic part|GO:0044424,intracellular part|GO:0044425,membrane part|GO:0044422,organelle part|GO:0031984,organelle subcompartment|GO:0098827,endoplasmic reticulum subcompartment	GO:0005488,binding|GO:0005515,protein binding
TMEM196		GO:0044425,membrane part|GO:0016021,integral component of membrane|GO:0016020,membrane|GO:0031224,intrinsic component of membrane	
LETMD1		GO:0031974,membrane-enclosed lumen|GO:0043229,intracellular organelle|GO:0043227,membrane-bounded organelle|GO:0043226,organelle|GO:0031224,intrinsic component of membrane|GO:0005737,cytoplasm|GO:0031981,nuclear lumen|GO:0016021,integral component of membrane|GO:0016020,membrane|GO:0005739,mitochondrion|GO:0005654,nucleoplasm|GO:0043231,intracellular membrane-bounded organelle|GO:0043233,organelle lumen|GO:0044464,cell part|GO:0005623,cell|GO:0005622,intracellular|GO:0044446,intracellular organelle part|GO:0070013,intracellular organelle lumen|GO:0044444,cytoplasmic part|GO:0044428,nuclear part|GO:0044424,intracellular part|GO:0044425,membrane part|GO:0005634,nucleus|GO:0044422,organelle part	GO:0043021,ribonucleoprotein complex binding|GO:0043022,ribosome binding|GO:0044877,macromolecular complex binding|GO:0005488,binding
IKZF2			GO:0043169,cation binding|GO:0097159,organic cyclic compound binding|GO:0043167,ion binding|GO:0005488,binding|GO:0003676,nucleic acid binding|GO:0046872,metal ion binding|GO:1901363,heterocyclic compound binding
C2CD4D	GO:0051648,vesicle localization|GO:0051046,regulation of secretion|GO:0051049,regulation of transport|GO:0006836,neurotransmitter transport|GO:0097480,establishment of synaptic vesicle localization|GO:0032940,secretion by cell|GO:0023052,signaling|GO:1903530,regulation of secretion by cell|GO:0051656,establishment of organelle localization|GO:0050789,regulation of biological process|GO:0044699,single-organism process|GO:0048489,synaptic vesicle transport|GO:0051234,establishment of localization|GO:0060341,regulation of cellular localization|GO:0051649,establishment of localization in cell|GO:0006887,exocytosis|GO:0051650,establishment of vesicle localization|GO:0017158,regulation of calcium ion-dependent exocytosis|GO:0065007,biological regulation|GO:1902578,single-organism localization|GO:0097479,synaptic vesicle localization|GO:0065008,regulation of biological quality|GO:0017156,calcium ion regulated exocytosis|GO:0017157,regulation of exocytosis|GO:0006810,transport|GO:0009987,cellular process|GO:0023061,signal release|GO:0060627,regulation of vesicle-mediated transport|GO:0044765,single-organism transport|GO:0044763,single-organism cellular process|GO:0007269,neurotransmitter secretion|GO:0007268,chemical synaptic transmission|GO:0007267,cell-cell signaling|GO:0007154,cell communication|GO:0032879,regulation of localization|GO:0051179,localization|GO:0050794,regulation of cellular process|GO:0051641,cellular localization|GO:0044700,single organism signaling|GO:0046903,secretion|GO:0016192,vesicle-mediated transport|GO:0016079,synaptic vesicle exocytosis|GO:0048791,calcium ion-regulated exocytosis of neurotransmitter|GO:0051640,organelle localization|GO:1902580,single-organism cellular localization|GO:0001505,regulation of neurotransmitter levels|GO:0099643,signal release from synapse|GO:0006906,vesicle fusion|GO:0061025,membrane fusion|GO:0061024,membrane organization|GO:0071840,cellular component organization or biogenesis|GO:0016043,cellular component organization|GO:0044801,single-organism membrane fusion|GO:0045055,regulated exocytosis|GO:1903305,regulation of regulated secretory pathway|GO:0099504,synaptic vesicle cycle|GO:0044802,single-organism membrane organization|GO:0006996,organelle organization|GO:0046907,intracellular transport|GO:0090174,organelle membrane fusion|GO:0016050,vesicle organization|GO:1902582,single-organism intracellular transport|GO:1902589,single-organism organelle organization|GO:0098916,anterograde trans-synaptic signaling|GO:0048284,organelle fusion|GO:0099531,presynaptic process involved in chemical synaptic	GO:0016020,membrane|GO:0044464,cell part|GO:0005623,cell|GO:0071944,cell periphery|GO:0005886,plasma membrane	GO:0043168,anion binding|GO:0043169,cation binding|GO:0000149,SNARE binding|GO:0005543,phospholipid binding|GO:0005544,calcium-dependent phospholipid binding|GO:0008289,lipid binding|GO:0043167,ion binding|GO:0019905,syntaxin binding|GO:0005509,calcium ion binding|GO:0005515,protein binding|GO:0030276,clathrin binding|GO:0046872,metal ion binding|GO:0005488,binding
FGFBP2		GO:0005576,extracellular region|GO:0005615,extracellular space|GO:0044421,extracellular region part	GO:0019838,growth factor binding|GO:0005515,protein binding|GO:0005488,binding
ZBTB8A	GO:0080090,regulation of primary metabolic process|GO:0019222,regulation of metabolic process|GO:0031326,regulation of cellular biosynthetic process|GO:0031323,regulation of cellular metabolic process|GO:0090304,nucleic acid metabolic process|GO:0044249,cellular biosynthetic process|GO:0034641,cellular nitrogen compound metabolic process|GO:0006807,nitrogen compound metabolic process|GO:0034645,cellular macromolecule biosynthetic process|GO:1901362,organic cyclic compound biosynthetic process|GO:0050789,regulation of biological process|GO:0097659,nucleic acid-templated transcription|GO:0032774,RNA biosynthetic process|GO:0006139,nucleobase-containing compound metabolic process|GO:0044260,cellular macromolecule metabolic process|GO:0071704,organic substance metabolic process|GO:2000112,regulation of cellular macromolecule biosynthetic process|GO:0060255,regulation of macromolecule metabolic process|GO:0010467,gene expression|GO:0065007,biological regulation|GO:1901360,organic cyclic compound metabolic process|GO:0010468,regulation of gene expression|GO:0018130,heterocycle biosynthetic process|GO:1901576,organic substance biosynthetic process|GO:0019219,regulation of nucleobase-containing compound metabolic process|GO:0006725,cellular aromatic compound metabolic process|GO:0009987,cellular process|GO:0009889,regulation of biosynthetic process|GO:1903506,regulation of nucleic acid-templated transcription|GO:0050794,regulation of cellular process|GO:0009058,biosynthetic process|GO:0009059,macromolecule biosynthetic process|GO:0051171,regulation of nitrogen compound metabolic process|GO:0008152,metabolic process|GO:2001141,regulation of RNA biosynthetic process|GO:0034654,nucleobase-containing compound biosynthetic process|GO:0046483,heterocycle metabolic process|GO:0016070,RNA metabolic process|GO:0044238,primary metabolic process|GO:0044271,cellular nitrogen compound biosynthetic process|GO:0051252,regulation of RNA metabolic process|GO:0044237,cellular metabolic process|GO:0043170,macromolecule metabolic process|GO:0006355,regulation of transcription, DNA-templated|GO:0010556,regulation of macromolecule biosynthetic process|GO:0006351,transcription, DNA-templated|GO:0019438,aromatic compound biosynthetic process	GO:0043231,intracellular membrane-bounded organelle|GO:0005634,nucleus|GO:0044464,cell part|GO:0005623,cell|GO:0005622,intracellular|GO:0043229,intracellular organelle|GO:0044424,intracellular part|GO:0043227,membrane-bounded organelle|GO:0043226,organelle	GO:0043169,cation binding|GO:0005515,protein binding|GO:0097159,organic cyclic compound binding|GO:0043167,ion binding|GO:0005488,binding|GO:0003676,nucleic acid binding|GO:0003677,DNA binding|GO:0046872,metal ion binding|GO:1901363,heterocyclic compound binding
CDC26	GO:0080090,regulation of primary metabolic process|GO:0032434,regulation of proteasomal ubiquitin-dependent protein catabolic process|GO:0032435,negative regulation of proteasomal ubiquitin-dependent protein catabolic process|GO:0044257,cellular protein catabolic process|GO:0000280,nuclear division|GO:0010965,regulation of mitotic sister chromatid separation|GO:0071840,cellular component organization or biogenesis|GO:0044712,single-organism catabolic process|GO:0044710,single-organism metabolic process|GO:0010605,negative regulation of macromolecule metabolic process|GO:0019222,regulation of metabolic process|GO:0071704,organic substance metabolic process|GO:0070647,protein modification by small protein conjugation or removal|GO:0032446,protein modification by small protein conjugation|GO:1901799,negative regulation of proteasomal protein catabolic process|GO:0044092,negative regulation of molecular function|GO:0048518,positive regulation of biological process|GO:0045839,negative regulation of mitotic nuclear division|GO:0007088,regulation of mitotic nuclear division|GO:0060255,regulation of macromolecule metabolic process|GO:0030162,regulation of proteolysis|GO:0030163,protein catabolic process|GO:1902099,regulation of metaphase/anaphase transition of cell cycle|GO:0019538,protein metabolic process|GO:0022402,cell cycle process|GO:0010639,negative regulation of organelle organization|GO:0051352,negative regulation of ligase activity|GO:0051783,regulation of nuclear division|GO:0051782,negative regulation of cell division|GO:0051784,negative regulation of nuclear division|GO:0044784,metaphase/anaphase transition of cell cycle|GO:0009896,positive regulation of catabolic process|GO:0009894,regulation of catabolic process|GO:0009895,negative regulation of catabolic process|GO:0009892,negative regulation of metabolic process|GO:0009893,positive regulation of metabolic process|GO:0051351,positive regulation of ligase activity|GO:0050789,regulation of biological process|GO:2000816,negative regulation of mitotic sister chromatid separation|GO:0007067,mitotic nuclear division|GO:0030071,regulation of mitotic metaphase/anaphase transition|GO:0031577,spindle checkpoint|GO:0007346,regulation of mitotic cell cycle|GO:0051340,regulation of ligase activity|GO:0051347,positive regulation of transferase activity|GO:0044260,cellular macromolecule metabolic process|GO:0016567,protein ubiquitination|GO:0051348,negative regulation of transferase activity|GO:0016043,cellular component organization|GO:0000209,protein polyubiquitination|GO:0065007,biological regulation|GO:0007049,cell cycle|GO:0044267,cellular protein metabolic process|GO:0065009,regulation of molecular function|GO:0071174,mitotic spindle checkpoint|GO:0071173,spindle assembly checkpoint|GO:0050790,regulation of catalytic activity|GO:0050794,regulation of cellular process|GO:1901987,regulation of cell cycle phase transition|GO:0043412,macromolecule modification|GO:0036211,protein modification process|GO:0008152,metabolic process|GO:0045786,negative regulation of cell cycle|GO:1901988,negative regulation of cell cycle phase transition|GO:0010604,positive regulation of macromolecule metabolic process|GO:0051444,negative regulation of ubiquitin-protein transferase activity|GO:0051603,proteolysis involved in cellular protein catabolic process|GO:0051443,positive regulation of ubiquitin-protein transferase activity|GO:0031400,negative regulation of protein modification process|GO:0031401,positive regulation of protein modification process|GO:0010498,proteasomal protein catabolic process|GO:0051338,regulation of transferase activity|GO:0045732,positive regulation of protein catabolic process|GO:0006511,ubiquitin-dependent protein catabolic process|GO:2001251,negative regulation of chromosome organization|GO:0033044,regulation of chromosome organization|GO:0045841,negative regulation of mitotic metaphase/anaphase transition|GO:0033046,negative regulation of sister chromatid segregation|GO:0033047,regulation of mitotic sister chromatid segregation|GO:0033043,regulation of organelle organization|GO:0010564,regulation of cell cycle process|GO:0033048,negative regulation of mitotic sister chromatid segregation|GO:0051129,negative regulation of cellular component organization|GO:0051128,regulation of cellular component organization|GO:0044248,cellular catabolic process|GO:0042176,regulation of protein catabolic process|GO:0042177,negative regulation of protein catabolic process|GO:1901991,negative regulation of mitotic cell cycle phase transition|GO:1901990,regulation of mitotic cell cycle phase transition|GO:0007059,chromosome segregation|GO:0043086,negative regulation of catalytic activity|GO:0043085,positive regulation of catalytic activity|GO:0051248,negative regulation of protein metabolic process|GO:0070979,protein K11-linked ubiquitination|GO:0043161,proteasome-mediated ubiquitin-dependent protein catabolic process|GO:0051246,regulation of protein metabolic process|GO:0051247,positive regulation of protein metabolic process|GO:1903320,regulation of protein modification by small protein conjugation or removal|GO:1903321,negative regulation of protein modification by small protein conjugation or removal|GO:1903322,positive regulation of protein modification by small protein conjugation or removal|GO:0032270,positive regulation of cellular protein metabolic process|GO:0031398,positive regulation of protein ubiquitination|GO:0031399,regulation of protein modification process|GO:0031396,regulation of protein ubiquitination|GO:0031397,negative regulation of protein ubiquitination|GO:0043632,modification-dependent macromolecule catabolic process|GO:0044093,positive regulation of molecular function|GO:0031330,negative regulation of cellular catabolic process|GO:0031331,positive regulation of cellular catabolic process|GO:1903050,regulation of proteolysis involved in cellular protein catabolic process|GO:1903051,negative regulation of proteolysis involved in cellular protein catabolic process|GO:0019941,modification-dependent protein catabolic process|GO:0031145,anaphase-promoting complex-dependent catabolic process|GO:0048519,negative regulation of biological process|GO:0051985,negative regulation of chromosome segregation|GO:0006508,proteolysis|GO:0051983,regulation of chromosome segregation|GO:0032269,negative regulation of cellular protein metabolic process|GO:0032268,regulation of cellular protein metabolic process|GO:0000819,sister chromatid segregation|GO:0098813,nuclear chromosome segregation|GO:0044772,mitotic cell cycle phase transition|GO:0043170,macromolecule metabolic process|GO:0045862,positive regulation of proteolysis|GO:0045861,negative regulation of proteolysis|GO:0033045,regulation of sister chromatid segregation|GO:0051438,regulation of ubiquitin-protein transferase activity|GO:0031329,regulation of cellular catabolic process|GO:0031325,positive regulation of cellular metabolic process|GO:0031324,negative regulation of cellular metabolic process|GO:0031323,regulation of cellular metabolic process|GO:1903047,mitotic cell cycle process|GO:0044770,cell cycle phase transition|GO:0010948,negative regulation of cell cycle process|GO:0051439,regulation of ubiquitin-protein ligase activity involved in mitotic cell cycle|GO:2000060,positive regulation of protein ubiquitination involved in ubiquitin-dependent protein catabolic process|GO:0051306,mitotic sister chromatid separation|GO:0051437,positive regulation of ubiquitin-protein ligase activity involved in regulation of mitotic cell cycle transition|GO:0051436,negative regulation of ubiquitin-protein ligase activity involved in mitotic cell cycle|GO:0051302,regulation of cell division|GO:0051301,cell division|GO:0051304,chromosome separation|GO:0044699,single-organism process|GO:2000058,regulation of protein ubiquitination involved in ubiquitin-dependent protein catabolic process|GO:1903052,positive regulation of proteolysis involved in cellular protein catabolic process|GO:0042787,protein ubiquitination involved in ubiquitin-dependent protein catabolic process|GO:1903364,positive regulation of cellular protein catabolic process|GO:0045930,negative regulation of mitotic cell cycle|GO:0009987,cellular process|GO:0000278,mitotic cell cycle|GO:1903362,regulation of cellular protein catabolic process|GO:1903363,negative regulation of cellular protein catabolic process|GO:0061136,regulation of proteasomal protein catabolic process|GO:1901575,organic substance catabolic process|GO:0006464,cellular protein modification process|GO:0044265,cellular macromolecule catabolic process|GO:0044763,single-organism cellular process|GO:0009056,catabolic process|GO:0009057,macromolecule catabolic process|GO:0006996,organelle organization|GO:0044238,primary metabolic process|GO:0000070,mitotic sister chromatid	GO:0031974,membrane-enclosed lumen|GO:0043229,intracellular organelle|GO:0005623,cell|GO:0043227,membrane-bounded organelle|GO:0043226,organelle|GO:0005737,cytoplasm|GO:0031981,nuclear lumen|GO:0005634,nucleus|GO:0005654,nucleoplasm|GO:1902494,catalytic complex|GO:1990234,transferase complex|GO:0031461,cullin-RING ubiquitin ligase complex|GO:0043234,protein complex|GO:0032991,macromolecular complex|GO:0043231,intracellular membrane-bounded organelle|GO:0043233,organelle lumen|GO:0005829,cytosol|GO:0044464,cell part|GO:0000151,ubiquitin ligase complex|GO:0000152,nuclear ubiquitin ligase complex|GO:0005622,intracellular|GO:0044446,intracellular organelle part|GO:0070013,intracellular organelle lumen|GO:0044444,cytoplasmic part|GO:0044428,nuclear part|GO:0044424,intracellular part|GO:0044422,organelle part|GO:0005680,anaphase-promoting complex	GO:0005488,binding|GO:0005515,protein binding
PCSK1N	GO:0009991,response to extracellular stimulus|GO:0051716,cellular response to stimulus|GO:0009892,negative regulation of metabolic process|GO:0080090,regulation of primary metabolic process|GO:0019222,regulation of metabolic process|GO:0044092,negative regulation of molecular function|GO:0031324,negative regulation of cellular metabolic process|GO:0031323,regulation of cellular metabolic process|GO:0016485,protein processing|GO:0034641,cellular nitrogen compound metabolic process|GO:0006807,nitrogen compound metabolic process|GO:0023052,signaling|GO:0007165,signal transduction|GO:0043086,negative regulation of catalytic activity|GO:0044699,single-organism process|GO:0042445,hormone metabolic process|GO:0007218,neuropeptide signaling pathway|GO:0051604,protein maturation|GO:0010605,negative regulation of macromolecule metabolic process|GO:0051346,negative regulation of hydrolase activity|GO:0010817,regulation of hormone levels|GO:0071704,organic substance metabolic process|GO:0009987,cellular process|GO:0051246,regulation of protein metabolic process|GO:0050789,regulation of biological process|GO:0060255,regulation of macromolecule metabolic process|GO:0010467,gene expression|GO:0010466,negative regulation of peptidase activity|GO:0065007,biological regulation|GO:0002021,response to dietary excess|GO:0048519,negative regulation of biological process|GO:0065009,regulation of molecular function|GO:0065008,regulation of biological quality|GO:0007186,G-protein coupled receptor signaling pathway|GO:0009605,response to external stimulus|GO:0009266,response to temperature stimulus|GO:0032501,multicellular organismal process|GO:0052547,regulation of peptidase activity|GO:0052548,regulation of endopeptidase activity|GO:0044260,cellular macromolecule metabolic process|GO:0050790,regulation of catalytic activity|GO:0051248,negative regulation of protein metabolic process|GO:0050794,regulation of cellular process|GO:0006950,response to stress|GO:0030162,regulation of proteolysis|GO:0008152,metabolic process|GO:0006508,proteolysis|GO:0010951,negative regulation of endopeptidase activity|GO:0007154,cell communication|GO:0016486,peptide hormone processing|GO:0044267,cellular protein metabolic process|GO:0006518,peptide metabolic process|GO:0009628,response to abiotic stimulus|GO:0051336,regulation of hydrolase activity|GO:0044700,single organism signaling|GO:0032269,negative regulation of cellular protein metabolic process|GO:0032268,regulation of cellular protein metabolic process|GO:1901564,organonitrogen compound metabolic process|GO:0043603,cellular amide metabolic process|GO:0044707,single-multicellular organism process|GO:0019538,protein metabolic process|GO:0050896,response to stimulus|GO:0031667,response to nutrient levels|GO:0044237,cellular metabolic process|GO:0043170,macromolecule metabolic process|GO:0009409,response to cold|GO:0044238,primary metabolic process|GO:0044763,single-organism cellular process|GO:0045861,negative regulation of proteolysis|GO:0048523,negative regulation of cellular process	GO:0043229,intracellular organelle|GO:0043227,membrane-bounded organelle|GO:0043226,organelle|GO:0005737,cytoplasm|GO:0031984,organelle subcompartment|GO:0070062,extracellular exosome|GO:0016023,cytoplasmic, membrane-bounded vesicle|GO:0031410,cytoplasmic vesicle|GO:0031988,membrane-bounded vesicle|GO:0044431,Golgi apparatus part|GO:0005615,extracellular space|GO:0005794,Golgi apparatus|GO:0030141,secretory granule|GO:0012505,endomembrane system|GO:1903561,extracellular vesicle|GO:0031982,vesicle|GO:0043230,extracellular organelle|GO:0043231,intracellular membrane-bounded organelle|GO:0005802,trans-Golgi network|GO:0044464,cell part|GO:0005623,cell|GO:0005622,intracellular|GO:0044446,intracellular organelle part|GO:0044444,cytoplasmic part|GO:0005576,extracellular region|GO:0098791,Golgi subcompartment|GO:0044424,intracellular part|GO:0065010,extracellular membrane-bounded organelle|GO:0044421,extracellular region part|GO:0044422,organelle part|GO:0099503,secretory vesicle|GO:0097708,intracellular vesicle	GO:0004866,endopeptidase inhibitor activity|GO:0061135,endopeptidase regulator activity|GO:0061134,peptidase regulator activity|GO:0005102,receptor binding|GO:0005515,protein binding|GO:0030414,peptidase inhibitor activity|GO:0004867,serine-type endopeptidase inhibitor activity|GO:0004857,enzyme inhibitor activity|GO:0098772,molecular function regulator|GO:0030234,enzyme regulator activity|GO:0005488,binding
DST	GO:0006996,organelle organization|GO:0007010,cytoskeleton organization|GO:0009987,cellular process|GO:0016043,cellular component organization|GO:0071840,cellular component organization or biogenesis	GO:0005856,cytoskeleton|GO:0043232,intracellular non-membrane-bounded organelle|GO:0044464,cell part|GO:0005623,cell|GO:0005622,intracellular|GO:0043229,intracellular organelle|GO:0043228,non-membrane-bounded organelle|GO:0044424,intracellular part|GO:0043226,organelle	GO:0003779,actin binding|GO:0005488,binding|GO:0005515,protein binding|GO:0008092,cytoskeletal protein binding

LAS1L	GO:0090304,nucleic acid metabolic process|GO:0034641,cellular nitrogen compound metabolic process|GO:0006807,nitrogen compound metabolic process|GO:0034660,ncRNA metabolic process|GO:1901360,organic cyclic compound metabolic process|GO:0006139,nucleobase-containing compound metabolic process|GO:0044260,cellular macromolecule metabolic process|GO:0071840,cellular component organization or biogenesis|GO:0042254,ribosome biogenesis|GO:0071704,organic substance metabolic process|GO:0010467,gene expression|GO:0006364,rRNA processing|GO:0022613,ribonucleoprotein complex biogenesis|GO:0034470,ncRNA processing|GO:0009987,cellular process|GO:0006725,cellular aromatic compound metabolic process|GO:0008152,metabolic process|GO:0046483,heterocycle metabolic process|GO:0016070,RNA metabolic process|GO:0044238,primary metabolic process|GO:0016072,rRNA metabolic process|GO:0044237,cellular metabolic process|GO:0043170,macromolecule metabolic process|GO:0044085,cellular component biogenesis|GO:0006396,RNA processing|GO:0090305,nucleic acid phosphodiester bond hydrolysis|GO:0000470,maturation of LSU-rRNA|GO:0000478,endonucleolytic cleavage involved in rRNA processing|GO:0042273,ribosomal large subunit biogenesis|GO:0006325,chromatin organization|GO:0016043,cellular component organization|GO:0090502,RNA phosphodiester bond hydrolysis, endonucleolytic|GO:0000460,maturation of 5.8S rRNA|GO:0000469,cleavage involved in rRNA processing|GO:0006996,organelle organization|GO:0090501,RNA phosphodiester bond hydrolysis|GO:0051276,chromosome organization	GO:0031974,membrane-enclosed lumen|GO:0043229,intracellular organelle|GO:0043228,non-membrane-bounded organelle|GO:0005623,cell|GO:0043227,membrane-bounded organelle|GO:0043226,organelle|GO:0034708,methyltransferase complex|GO:0005856,cytoskeleton|GO:0031981,nuclear lumen|GO:0005730,nucleolus|GO:0005634,nucleus|GO:0016020,membrane|GO:0005815,microtubule organizing center|GO:0005654,nucleoplasm|GO:0044430,cytoskeletal part|GO:0044451,nucleoplasm part|GO:0071339,MLL1 complex|GO:0005737,cytoplasm|GO:0015630,microtubule cytoskeleton|GO:1902494,catalytic complex|GO:1990234,transferase complex|GO:0043234,protein complex|GO:0032991,macromolecular complex|GO:0043231,intracellular membrane-bounded organelle|GO:0043232,intracellular non-membrane-bounded organelle|GO:0043233,organelle lumen|GO:0044464,cell part|GO:0035097,histone methyltransferase complex|GO:0005622,intracellular|GO:0044446,intracellular organelle part|GO:0070013,intracellular organelle lumen|GO:0044428,nuclear part|GO:0044424,intracellular part|GO:0044422,organelle part|GO:0044665,MLL1/2 complex|GO:0030687,preribosome, large subunit precursor|GO:0030684,preribosome|GO:0030529,intracellular	GO:0097159,organic cyclic compound binding|GO:0044822,poly(A) RNA binding|GO:0005488,binding|GO:0003676,nucleic acid binding|GO:1901363,heterocyclic compound binding|GO:0003723,RNA binding
PNMA5	GO:0016265,obsolete death|GO:0009987,cellular process|GO:0012501,programmed cell death|GO:0050789,regulation of biological process|GO:0006915,apoptotic process|GO:0050794,regulation of cellular process|GO:0043065,positive regulation of apoptotic process|GO:0048518,positive regulation of biological process|GO:0043067,regulation of programmed cell death|GO:0044763,single-organism cellular process|GO:0010942,positive regulation of cell death|GO:0065007,biological regulation|GO:0008219,cell death|GO:0010941,regulation of cell death|GO:0042981,regulation of apoptotic process|GO:0043068,positive regulation of programmed cell death|GO:0044699,single-organism process|GO:0048522,positive regulation of cellular process		GO:0005515,protein binding|GO:0042802,identical protein binding|GO:0005488,binding
B4GALNT3	GO:0008152,metabolic process	GO:0043229,intracellular organelle|GO:0005622,intracellular|GO:0043227,membrane-bounded organelle|GO:0043226,organelle|GO:0031224,intrinsic component of membrane|GO:0005737,cytoplasm|GO:0031985,Golgi cisterna|GO:0031984,organelle subcompartment|GO:0031090,organelle membrane|GO:0016021,integral component of membrane|GO:0016020,membrane|GO:0044431,Golgi apparatus part|GO:0005795,Golgi stack|GO:0005794,Golgi apparatus|GO:0098588,bounding membrane of organelle|GO:0012505,endomembrane system|GO:0032580,Golgi cisterna membrane|GO:0043231,intracellular membrane-bounded organelle|GO:0044464,cell part|GO:0005623,cell|GO:0000139,Golgi membrane|GO:0044446,intracellular organelle part|GO:0044444,cytoplasmic part|GO:0098791,Golgi subcompartment|GO:0044424,intracellular part|GO:0044425,membrane part|GO:0044422,organelle part	GO:0033842,N-acetyl-beta-glucosaminyl-glycoprotein 4-beta-N-acetylgalactosaminyltransferase activity|GO:0003824,catalytic activity|GO:0016740,transferase activity|GO:0016757,transferase activity, transferring glycosyl groups|GO:0016758,transferase activity, transferring hexosyl groups|GO:0008376,acetylgalactosaminyltransferase activity|GO:0008194,UDP-glycosyltransferase activity
CDK11A	GO:0044267,cellular protein metabolic process|GO:0006468,protein phosphorylation|GO:0044260,cellular macromolecule metabolic process|GO:0044238,primary metabolic process|GO:0019538,protein metabolic process|GO:0016310,phosphorylation|GO:0009987,cellular process|GO:0043412,macromolecule modification|GO:0006464,cellular protein modification process|GO:0043170,macromolecule metabolic process|GO:0071704,organic substance metabolic process|GO:0006796,phosphate-containing compound metabolic process|GO:0036211,protein modification process|GO:0044237,cellular metabolic process|GO:0008152,metabolic process|GO:0006793,phosphorus metabolic process|GO:1901564,organonitrogen compound metabolic process|GO:0006807,nitrogen compound metabolic process	GO:0005737,cytoplasm|GO:0044446,intracellular organelle part|GO:0043231,intracellular membrane-bounded organelle|GO:0031981,nuclear lumen|GO:0043233,organelle lumen|GO:0005634,nucleus|GO:0044464,cell part|GO:0031974,membrane-enclosed lumen|GO:0005622,intracellular|GO:0005654,nucleoplasm|GO:0070013,intracellular organelle lumen|GO:0043229,intracellular organelle|GO:0044428,nuclear part|GO:0005623,cell|GO:0044424,intracellular part|GO:0043227,membrane-bounded organelle|GO:0043226,organelle|GO:0044422,organelle part	GO:0035639,purine ribonucleoside triphosphate binding|GO:0004672,protein kinase activity|GO:0000166,nucleotide binding|GO:1901363,heterocyclic compound binding|GO:0001883,purine nucleoside binding|GO:0001882,nucleoside binding|GO:0043168,anion binding|GO:0043167,ion binding|GO:0016740,transferase activity|GO:1901265,nucleoside phosphate binding|GO:0032549,ribonucleoside binding|GO:0017076,purine nucleotide binding|GO:0005524,ATP binding|GO:0016301,kinase activity|GO:0036094,small molecule binding|GO:0003824,catalytic activity|GO:0030554,adenyl nucleotide binding|GO:0016773,phosphotransferase activity, alcohol group as acceptor|GO:0016772,transferase activity, transferring phosphorus-containing groups|GO:0097367,carbohydrate derivative binding|GO:0032559,adenyl ribonucleotide binding|GO:0097159,organic cyclic compound binding|GO:0032555,purine ribonucleotide binding|GO:0032550,purine ribonucleoside binding|GO:0032553,ribonucleotide binding|GO:0005488,binding
GON4L	GO:0080090,regulation of primary metabolic process|GO:0019222,regulation of metabolic process|GO:1901362,organic cyclic compound biosynthetic process|GO:1901360,organic cyclic compound metabolic process|GO:0010605,negative regulation of macromolecule metabolic process|GO:0048869,cellular developmental process|GO:0048513,animal organ development|GO:0048519,negative regulation of biological process|GO:0060255,regulation of macromolecule metabolic process|GO:0046649,lymphocyte activation|GO:2001141,regulation of RNA biosynthetic process|GO:0046483,heterocycle metabolic process|GO:0044707,single-multicellular organism process|GO:0002376,immune system process|GO:0019438,aromatic compound biosynthetic process|GO:0009892,negative regulation of metabolic process|GO:0009890,negative regulation of biosynthetic process|GO:0045321,leukocyte activation|GO:0010629,negative regulation of gene expression|GO:0006807,nitrogen compound metabolic process|GO:0050789,regulation of biological process|GO:0097659,nucleic acid-templated transcription|GO:1901576,organic substance biosynthetic process|GO:0044260,cellular macromolecule metabolic process|GO:0065007,biological regulation|GO:0018130,heterocycle biosynthetic process|GO:0009889,regulation of biosynthetic process|GO:0050794,regulation of cellular process|GO:0001775,cell activation|GO:0008152,metabolic process|GO:0030097,hemopoiesis|GO:0034654,nucleobase-containing compound biosynthetic process|GO:0002521,leukocyte differentiation|GO:0002520,immune system development|GO:0016070,RNA metabolic process|GO:0044767,single-organism developmental process|GO:0044271,cellular nitrogen compound biosynthetic process|GO:0006355,regulation of transcription, DNA-templated|GO:0010556,regulation of macromolecule biosynthetic process|GO:0006351,transcription, DNA-templated|GO:0010558,negative regulation of macromolecule biosynthetic process|GO:0032774,RNA biosynthetic process|GO:0030154,cell differentiation|GO:0044249,cellular biosynthetic process|GO:0034641,cellular nitrogen compound metabolic process|GO:0034645,cellular macromolecule biosynthetic process|GO:0044699,single-organism process|GO:0006139,nucleobase-containing compound metabolic process|GO:0032502,developmental process|GO:0032501,multicellular organismal process|GO:0009987,cellular process|GO:0006725,cellular aromatic compound metabolic process|GO:1903506,regulation of nucleic acid-templated transcription|GO:1903507,negative regulation of nucleic acid-templated transcription|GO:0045892,negative regulation of transcription, DNA-templated|GO:0051253,negative regulation of RNA metabolic process|GO:0051252,regulation of RNA metabolic process|GO:0043170,macromolecule metabolic process|GO:0030098,lymphocyte differentiation|GO:0048731,system development|GO:0031327,negative regulation of cellular biosynthetic process|GO:0031326,regulation of cellular biosynthetic process|GO:0031324,negative regulation of cellular metabolic process|GO:0031323,regulation of cellular metabolic process|GO:0042113,B cell activation|GO:0090304,nucleic acid metabolic process|GO:0007275,multicellular organism development|GO:2000112,regulation of cellular macromolecule biosynthetic process|GO:2000113,negative regulation of cellular macromolecule biosynthetic process|GO:0071704,organic substance metabolic process|GO:0010467,gene expression|GO:0048534,hematopoietic or lymphoid organ development|GO:0010468,regulation of gene expression|GO:0045934,negative regulation of nucleobase-containing compound metabolic process|GO:0030183,B cell differentiation|GO:0019219,regulation of nucleobase-containing compound metabolic process|GO:1902679,negative regulation of RNA biosynthetic process|GO:0009058,biosynthetic process|GO:0009059,macromolecule biosynthetic process|GO:0044763,single-organism cellular process|GO:0051171,regulation of nitrogen compound metabolic process|GO:0051172,negative regulation of nitrogen compound metabolic process|GO:0044238,primary metabolic process|GO:0048856,anatomical structure development|GO:0044237,cellular metabolic process|GO:0048523,negative regulation of cellular process	GO:0044446,intracellular organelle part|GO:0016604,nuclear body|GO:0043231,intracellular membrane-bounded organelle|GO:0031981,nuclear lumen|GO:0043233,organelle lumen|GO:0005634,nucleus|GO:0044464,cell part|GO:0031974,membrane-enclosed lumen|GO:0005622,intracellular|GO:0005654,nucleoplasm|GO:0070013,intracellular organelle lumen|GO:0043229,intracellular organelle|GO:0044451,nucleoplasm part|GO:0005623,cell|GO:0044424,intracellular part|GO:0044428,nuclear part|GO:0043227,membrane-bounded organelle|GO:0043226,organelle|GO:0044422,organelle part	GO:0003714,transcription corepressor activity|GO:0003712,transcription cofactor activity|GO:0000989,transcription factor activity, transcription factor binding|GO:0000988,transcription factor activity, protein binding
GRIK1	GO:0051046,regulation of secretion|GO:0051047,positive regulation of secretion|GO:0051049,regulation of transport|GO:0007610,behavior|GO:0006820,anion transport|GO:0007165,signal transduction|GO:0007166,cell surface receptor signaling pathway|GO:0060080,inhibitory postsynaptic potential|GO:0034765,regulation of ion transmembrane transport|GO:0048266,behavioral response to pain|GO:0034762,regulation of transmembrane transport|GO:0048265,response to pain|GO:0032230,positive regulation of synaptic transmission, GABAergic|GO:0051716,cellular response to stimulus|GO:0007215,glutamate receptor signaling pathway|GO:0048518,positive regulation of biological process|GO:0048519,negative regulation of biological process|GO:0051957,positive regulation of amino acid transport|GO:0051954,positive regulation of amine transport|GO:0051955,regulation of amino acid transport|GO:0015718,monocarboxylic acid transport|GO:0051050,positive regulation of transport|GO:0015849,organic acid transport|GO:0015711,organic anion transport|GO:0048172,regulation of short-term neuronal synaptic plasticity|GO:0044700,single organism signaling|GO:0044707,single-multicellular organism process|GO:0044708,single-organism behavior|GO:0060079,excitatory postsynaptic potential|GO:0051899,membrane depolarization|GO:0032229,negative regulation of synaptic transmission, GABAergic|GO:0032228,regulation of synaptic transmission, GABAergic|GO:0050789,regulation of biological process|GO:0033555,multicellular organismal response to stress|GO:0042391,regulation of membrane potential|GO:1903793,positive regulation of anion transport|GO:0046717,acid secretion|GO:0048167,regulation of synaptic plasticity|GO:0035249,synaptic transmission, glutamatergic|GO:0065007,biological regulation|GO:0065008,regulation of biological quality|GO:0006811,ion transport|GO:0006810,transport|GO:0050794,regulation of cellular process|GO:0006950,response to stress|GO:0032890,regulation of organic acid transport|GO:0060078,regulation of postsynaptic membrane potential|GO:0032892,positive regulation of organic acid transport|GO:0051234,establishment of localization|GO:0046903,secretion|GO:0050896,response to stimulus|GO:0051967,negative regulation of synaptic transmission, glutamatergic|GO:0051966,regulation of synaptic transmission, glutamatergic|GO:0050803,regulation of synapse structure or activity|GO:0006865,amino acid transport|GO:0050806,positive regulation of synaptic transmission|GO:0050805,negative regulation of synaptic transmission|GO:0050804,modulation of synaptic transmission|GO:0023056,positive regulation of signaling|GO:0023057,negative regulation of signaling|GO:0023052,signaling|GO:0010648,negative regulation of cell communication|GO:0015837,amine transport|GO:0023051,regulation of signaling|GO:0010647,positive regulation of cell communication|GO:0010646,regulation of cell communication|GO:0044699,single-organism process|GO:0007417,central nervous system development|GO:0032502,developmental process|GO:0032501,multicellular organismal process|GO:0035235,ionotropic glutamate receptor signaling pathway|GO:0009987,cellular process|GO:0043270,positive regulation of ion transport|GO:0051952,regulation of amine transport|GO:0032879,regulation of localization|GO:0055085,transmembrane transport|GO:0048731,system development|GO:0015812,gamma-aminobutyric acid transport|GO:0014051,gamma-aminobutyric acid secretion|GO:0014052,regulation of gamma-aminobutyric acid secretion|GO:0014054,positive regulation of gamma-aminobutyric acid secretion|GO:0007270,neuron-neuron synaptic transmission|GO:0044070,regulation of anion transport|GO:0007275,multicellular organism development|GO:0043269,regulation of ion transport|GO:0051932,synaptic transmission, GABAergic|GO:0048168,regulation of neuronal synaptic plasticity|GO:0071705,nitrogen compound transport|GO:0071702,organic substance transport|GO:0044767,single-organism developmental process|GO:0034220,ion transmembrane transport|GO:0044765,single-organism transport|GO:0044763,single-organism cellular process|GO:0007268,chemical synaptic transmission|GO:0007267,cell-cell signaling|GO:0007154,cell communication|GO:0051179,localization|GO:1902578,single-organism localization|GO:0046942,carboxylic acid transport|GO:0030534,adult behavior|GO:0007399,nervous system development|GO:0048856,anatomical structure development|GO:0048523,negative regulation of cellular process|GO:0048522,positive regulation of cellular process|GO:1905114,cell surface receptor signaling pathway involved in cell-cell signaling|GO:0071804,cellular potassium ion transport|GO:0071805,potassium ion transmembrane transport|GO:0006813,potassium ion transport|GO:0035725,sodium ion transmembrane transport|GO:0098662,inorganic cation transmembrane transport|GO:0098660,inorganic ion transmembrane transport|GO:0015672,monovalent inorganic cation transport|GO:0030001,metal ion transport|GO:0099565,chemical synaptic transmission, postsynaptic|GO:0006812,cation transport|GO:0006814,sodium ion transport|GO:0098916,anterograde trans-synaptic signaling|GO:0098655,cation transmembrane transport|GO:0099536,synaptic signaling|GO:0099537,trans-synaptic signaling	GO:0098590,plasma membrane region|GO:0030425,dendrite|GO:0030424,axon|GO:0071944,cell periphery|GO:0034702,ion channel complex|GO:0045202,synapse|GO:0031226,intrinsic component of plasma membrane|GO:0030054,cell junction|GO:0097060,synaptic membrane|GO:0043679,axon terminus|GO:0044306,neuron projection terminus|GO:0032983,kainate selective glutamate receptor complex|GO:0016021,integral component of membrane|GO:0016020,membrane|GO:0098802,plasma membrane receptor complex|GO:0097458,neuron part|GO:0044456,synapse part|GO:0031224,intrinsic component of membrane|GO:0043005,neuron projection|GO:0044459,plasma membrane part|GO:1902495,transmembrane transporter complex|GO:0008328,ionotropic glutamate receptor complex|GO:0044297,cell body|GO:0098589,membrane region|GO:1990351,transporter complex|GO:0045211,postsynaptic membrane|GO:0014069,postsynaptic density|GO:0036477,somatodendritic compartment|GO:0005887,integral component of plasma membrane|GO:0005886,plasma membrane|GO:0032991,macromolecular complex|GO:0042995,cell projection|GO:0097481,neuronal postsynaptic density|GO:0043234,protein complex|GO:0043235,receptor complex|GO:0043195,terminal bouton|GO:0042734,presynaptic membrane|GO:0043025,neuronal cell body|GO:0044463,cell projection part|GO:0044464,cell part|GO:0005623,cell|GO:0098797,plasma membrane protein complex|GO:0098796,membrane protein complex|GO:0098794,postsynapse|GO:0033267,axon part|GO:0044425,membrane part|GO:0098805,whole membrane	GO:0038023,signaling receptor activity|GO:0008144,drug binding|GO:0060089,molecular transducer activity|GO:0008066,glutamate receptor activity|GO:0004872,receptor activity|GO:0004871,signal transducer activity|GO:0005261,cation channel activity|GO:0016595,glutamate binding|GO:0016597,amino acid binding|GO:0004970,ionotropic glutamate receptor activity|GO:0043168,anion binding|GO:0015277,kainate selective glutamate receptor activity|GO:0015276,ligand-gated ion channel activity|GO:0043167,ion binding|GO:0022857,transmembrane transporter activity|GO:0022843,voltage-gated cation channel activity|GO:0022803,passive transmembrane transporter activity|GO:0005216,ion channel activity|GO:0042802,identical protein binding|GO:0042803,protein homodimerization activity|GO:0005231,excitatory extracellular ligand-gated ion channel activity|GO:0005215,transporter activity|GO:0005230,extracellular ligand-gated ion channel activity|GO:0008324,cation transmembrane transporter activity|GO:0005234,extracellular-glutamate-gated ion channel activity|GO:0036094,small molecule binding|GO:0022891,substrate-specific transmembrane transporter activity|GO:0022892,substrate-specific transporter activity|GO:0005244,voltage-gated ion channel activity|GO:0043177,organic acid binding|GO:0031406,carboxylic acid binding|GO:0015075,ion transmembrane transporter activity|GO:0022832,voltage-gated channel activity|GO:0004888,transmembrane signaling receptor activity|GO:0015267,channel activity|GO:0022836,gated channel activity|GO:0005515,protein binding|GO:0022834,ligand-gated channel activity|GO:0046983,protein dimerization activity|GO:0022838,substrate-specific channel activity|GO:0005488,binding|GO:0005267,potassium channel activity|GO:0030594,neurotransmitter receptor activity|GO:0015081,sodium ion transmembrane transporter activity|GO:0022824,transmitter-gated ion channel activity|GO:0046873,metal ion transmembrane transporter activity|GO:0005272,sodium channel activity|GO:0022890,inorganic cation transmembrane transporter activity|GO:0015077,monovalent inorganic cation transmembrane transporter activity|GO:0022835,transmitter-gated channel activity|GO:0015079,potassium ion transmembrane transporter activity|GO:0099600,transmembrane receptor activity
PIEZO2	GO:0006810,transport|GO:0006811,ion transport|GO:0009987,cellular process|GO:0051179,localization|GO:0034220,ion transmembrane transport|GO:0044765,single-organism transport|GO:0044763,single-organism cellular process|GO:0044699,single-organism process|GO:0051234,establishment of localization|GO:0055085,transmembrane transport|GO:1902578,single-organism localization	GO:0044425,membrane part|GO:0016021,integral component of membrane|GO:0016020,membrane|GO:0031224,intrinsic component of membrane	GO:0022891,substrate-specific transmembrane transporter activity|GO:0022833,mechanically gated channel activity|GO:0022892,substrate-specific transporter activity|GO:0005215,transporter activity|GO:0005216,ion channel activity|GO:0015075,ion transmembrane transporter activity|GO:0022857,transmembrane transporter activity|GO:0015267,channel activity|GO:0022836,gated channel activity|GO:0022803,passive transmembrane transporter activity|GO:0022838,substrate-specific channel activity|GO:0008381,mechanically-gated ion channel activity
SSTR3	GO:0038169,somatostatin receptor signaling pathway|GO:0060322,head development|GO:0007165,signal transduction|GO:0071840,cellular component organization or biogenesis|GO:0009755,hormone-mediated signaling pathway|GO:0051716,cellular response to stimulus|GO:0000003,reproduction|GO:0048513,animal organ development|GO:0048519,negative regulation of biological process|GO:0042127,regulation of cell proliferation|GO:0031960,response to corticosteroid|GO:0038170,somatostatin signaling pathway|GO:0007283,spermatogenesis|GO:0043434,response to peptide hormone|GO:0010033,response to organic substance|GO:0051704,multi-organism process|GO:0044700,single organism signaling|GO:0044703,multi-organism reproductive process|GO:0044702,single organism reproductive process|GO:0009605,response to external stimulus|GO:0044707,single-multicellular organism process|GO:0010243,response to organonitrogen compound|GO:0031667,response to nutrient levels|GO:0009725,response to hormone|GO:0022037,metencephalon development|GO:0050789,regulation of biological process|GO:0016043,cellular component organization|GO:0065007,biological regulation|GO:0014070,response to organic cyclic compound|GO:0007186,G-protein coupled receptor signaling pathway|GO:0007187,G-protein coupled receptor signaling pathway, coupled to cyclic nucleotide second messenger|GO:0016265,obsolete death|GO:0050794,regulation of cellular process|GO:0012501,programmed cell death|GO:0006950,response to stress|GO:1901700,response to oxygen-containing compound|GO:0021549,cerebellum development|GO:0007420,brain development|GO:1901698,response to nitrogen compound|GO:1901699,cellular response to nitrogen compound|GO:0097305,response to alcohol|GO:0097306,cellular response to alcohol|GO:0019953,sexual reproduction|GO:0007267,cell-cell signaling|GO:0023052,signaling|GO:0070887,cellular response to chemical stimulus|GO:0042221,response to chemical|GO:0044699,single-organism process|GO:0007417,central nervous system development|GO:0009719,response to endogenous stimulus|GO:0071375,cellular response to peptide hormone stimulus|GO:0008628,hormone-mediated apoptotic signaling pathway|GO:0032502,developmental process|GO:0008285,negative regulation of cell proliferation|GO:0032501,multicellular organismal process|GO:0048609,multicellular organismal reproductive process|GO:0032504,multicellular organism reproduction|GO:0008283,cell proliferation|GO:0071392,cellular response to estradiol stimulus|GO:0071385,cellular response to glucocorticoid stimulus|GO:0071391,cellular response to estrogen stimulus|GO:0071396,cellular response to lipid|GO:0032870,cellular response to hormone stimulus|GO:0048232,male gamete generation|GO:0071407,cellular response to organic cyclic compound|GO:0048731,system development|GO:0050896,response to stimulus|GO:0048545,response to steroid hormone|GO:0071495,cellular response to endogenous stimulus|GO:0009991,response to extracellular stimulus|GO:0051384,response to glucocorticoid|GO:0071383,cellular response to steroid hormone stimulus|GO:0097190,apoptotic signaling pathway|GO:0071384,cellular response to corticosteroid stimulus|GO:0042594,response to starvation|GO:0008219,cell death|GO:0007275,multicellular organism development|GO:0007276,gamete generation|GO:0032355,response to estradiol|GO:0033993,response to lipid|GO:0071417,cellular response to organonitrogen compound|GO:0071310,cellular response to organic substance|GO:0030900,forebrain development|GO:0009987,cellular process|GO:0006915,apoptotic process|GO:0044767,single-organism developmental process|GO:0022414,reproductive process|GO:0044763,single-organism cellular process|GO:0043627,response to estrogen|GO:0007154,cell communication|GO:0006996,organelle organization|GO:1901701,cellular response to oxygen-containing compound|GO:0007399,nervous system development|GO:0048856,anatomical structure development|GO:1901652,response to peptide|GO:1901653,cellular response to peptide|GO:0030902,hindbrain development|GO:0048523,negative regulation of cellular process|GO:0007218,neuropeptide signaling pathway|GO:0098916,anterograde trans-synaptic signaling|GO:0099536,synaptic signaling|GO:0007268,chemical synaptic	GO:0098590,plasma membrane region|GO:0060170,ciliary membrane|GO:0071944,cell periphery|GO:0005929,cilium|GO:0031226,intrinsic component of plasma membrane|GO:0043226,organelle|GO:0031224,intrinsic component of membrane|GO:0005737,cytoplasm|GO:0072372,primary cilium|GO:0031090,organelle membrane|GO:0043227,membrane-bounded organelle|GO:0016021,integral component of membrane|GO:0016020,membrane|GO:0031513,nonmotile primary cilium|GO:0044459,plasma membrane part|GO:0098588,bounding membrane of organelle|GO:0098589,membrane region|GO:0031253,cell projection membrane|GO:0005887,integral component of plasma membrane|GO:0005886,plasma membrane|GO:0042995,cell projection|GO:0044463,cell projection part|GO:0044464,cell part|GO:0005623,cell|GO:0005622,intracellular|GO:0044441,ciliary part|GO:0044424,intracellular part|GO:0044425,membrane part|GO:0098805,whole membrane|GO:0044422,organelle part|GO:0097458,neuron part|GO:0043005,neuron projection	GO:0008528,G-protein coupled peptide receptor activity|GO:0004930,G-protein coupled receptor activity|GO:0030594,neurotransmitter receptor activity|GO:0038023,signaling receptor activity|GO:0005515,protein binding|GO:0060089,molecular transducer activity|GO:0004888,transmembrane signaling receptor activity|GO:0001653,peptide receptor activity|GO:0004872,receptor activity|GO:0004871,signal transducer activity|GO:0008188,neuropeptide receptor activity|GO:0004994,somatostatin receptor activity|GO:0005488,binding|GO:0033218,amide binding|GO:0042923,neuropeptide binding|GO:0042277,peptide binding|GO:0005102,receptor binding|GO:0099600,transmembrane receptor activity
ZNF611	GO:0080090,regulation of primary metabolic process|GO:0019222,regulation of metabolic process|GO:0031326,regulation of cellular biosynthetic process|GO:0031323,regulation of cellular metabolic process|GO:0090304,nucleic acid metabolic process|GO:0044249,cellular biosynthetic process|GO:0034641,cellular nitrogen compound metabolic process|GO:0006807,nitrogen compound metabolic process|GO:0034645,cellular macromolecule biosynthetic process|GO:1901362,organic cyclic compound biosynthetic process|GO:0050789,regulation of biological process|GO:0097659,nucleic acid-templated transcription|GO:0032774,RNA biosynthetic process|GO:0006139,nucleobase-containing compound metabolic process|GO:0044260,cellular macromolecule metabolic process|GO:0071704,organic substance metabolic process|GO:2000112,regulation of cellular macromolecule biosynthetic process|GO:0060255,regulation of macromolecule metabolic process|GO:0010467,gene expression|GO:0065007,biological regulation|GO:1901360,organic cyclic compound metabolic process|GO:0010468,regulation of gene expression|GO:0018130,heterocycle biosynthetic process|GO:1901576,organic substance biosynthetic process|GO:0019219,regulation of nucleobase-containing compound metabolic process|GO:0006725,cellular aromatic compound metabolic process|GO:0009987,cellular process|GO:0009889,regulation of biosynthetic process|GO:1903506,regulation of nucleic acid-templated transcription|GO:0050794,regulation of cellular process|GO:0009058,biosynthetic process|GO:0009059,macromolecule biosynthetic process|GO:0051171,regulation of nitrogen compound metabolic process|GO:0008152,metabolic process|GO:2001141,regulation of RNA biosynthetic process|GO:0034654,nucleobase-containing compound biosynthetic process|GO:0046483,heterocycle metabolic process|GO:0016070,RNA metabolic process|GO:0044238,primary metabolic process|GO:0044271,cellular nitrogen compound biosynthetic process|GO:0051252,regulation of RNA metabolic process|GO:0044237,cellular metabolic process|GO:0043170,macromolecule metabolic process|GO:0006355,regulation of transcription, DNA-templated|GO:0010556,regulation of macromolecule biosynthetic process|GO:0006351,transcription, DNA-templated|GO:0019438,aromatic compound biosynthetic process		GO:0003700,transcription factor activity, sequence-specific DNA binding|GO:0001071,nucleic acid binding transcription factor activity
CTD			
PTN	GO:0008104,protein localization|GO:0019222,regulation of metabolic process|GO:0007612,learning|GO:0021510,spinal cord development|GO:0007610,behavior|GO:0007611,learning or memory|GO:0061024,membrane organization|GO:0060322,head development|GO:0007165,signal transduction|GO:0007166,cell surface receptor signaling pathway|GO:0007167,enzyme linked receptor protein signaling pathway|GO:0031344,regulation of cell projection organization|GO:0072657,protein localization to membrane|GO:0031346,positive regulation of cell projection organization|GO:0051716,cellular response to stimulus|GO:0045785,positive regulation of cell adhesion|GO:0001666,response to hypoxia|GO:0048869,cellular developmental process|GO:0070848,response to growth factor|GO:0045664,regulation of neuron differentiation|GO:0045666,positive regulation of neuron differentiation|GO:0010720,positive regulation of cell development|GO:0044092,negative regulation of molecular function|GO:0048518,positive regulation of biological process|GO:0048519,negative regulation of biological process|GO:0033036,macromolecule localization|GO:0042127,regulation of cell proliferation|GO:0031589,cell-substrate adhesion|GO:0030154,cell differentiation|GO:0048468,cell development|GO:0033280,response to vitamin D|GO:0007528,neuromuscular junction development|GO:0042221,response to chemical|GO:0010976,positive regulation of neuron projection development|GO:0010975,regulation of neuron projection development|GO:0001754,eye photoreceptor cell differentiation|GO:0051128,regulation of cellular component organization|GO:0009628,response to abiotic stimulus|GO:0010033,response to organic substance|GO:0003008,system process|GO:0044700,single organism signaling|GO:0031668,cellular response to extracellular stimulus|GO:0031669,cellular response to nutrient levels|GO:0009605,response to external stimulus|GO:0044707,single-multicellular organism process|GO:0044708,single-organism behavior|GO:0031667,response to nutrient levels|GO:0046530,photoreceptor cell differentiation|GO:0051781,positive regulation of cell division|GO:0001503,ossification|GO:0003407,neural retina development|GO:0033554,cellular response to stress|GO:0042391,regulation of membrane potential|GO:0009892,negative regulation of metabolic process|GO:0070887,cellular response to chemical stimulus|GO:0022603,regulation of anatomical structure morphogenesis|GO:0048167,regulation of synaptic plasticity|GO:0031175,neuron projection development|GO:0050789,regulation of biological process|GO:0071295,cellular response to vitamin|GO:0009653,anatomical structure morphogenesis|GO:0031670,cellular response to nutrient|GO:0016043,cellular component organization|GO:1990089,response to nerve growth factor|GO:0071453,cellular response to oxygen levels|GO:0065007,biological regulation|GO:0071840,cellular component organization or biogenesis|GO:0071456,cellular response to hypoxia|GO:0065009,regulation of molecular function|GO:0065008,regulation of biological quality|GO:0007584,response to nutrient|GO:0007185,transmembrane receptor protein tyrosine phosphatase signaling pathway|GO:0051130,positive regulation of cellular component organization|GO:0009887,animal organ morphogenesis|GO:0034613,cellular protein localization|GO:0050793,regulation of developmental process|GO:0050790,regulation of catalytic activity|GO:0009888,tissue development|GO:0050794,regulation of cellular process|GO:0006950,response to stress|GO:0044802,single-organism membrane organization|GO:0051239,regulation of multicellular organismal process|GO:0036119,response to platelet-derived growth factor|GO:0007423,sensory organ development|GO:0072576,liver morphogenesis|GO:0072575,epithelial cell proliferation involved in liver morphogenesis|GO:0072574,hepatocyte proliferation|GO:0036293,response to decreased oxygen levels|GO:0050896,response to stimulus|GO:0070727,cellular macromolecule localization|GO:0050890,cognition|GO:0071340,skeletal muscle acetylcholine-gated channel clustering|GO:0036294,cellular response to decreased oxygen levels|GO:0032502,developmental process|GO:0051962,positive regulation of nervous system development|GO:0051960,regulation of nervous system development|GO:0007275,multicellular organism development|GO:0043010,camera-type eye development|GO:0097306,cellular response to alcohol|GO:0050808,synapse organization|GO:0008152,metabolic process|GO:0050803,regulation of synapse structure or activity|GO:0030155,regulation of cell adhesion|GO:0031214,biomineral tissue development|GO:0050806,positive regulation of synaptic transmission|GO:0050804,modulation of synaptic transmission|GO:0023056,positive regulation of signaling|GO:0007155,cell adhesion|GO:0023052,signaling|GO:0060041,retina development in camera-type eye|GO:0060042,retina morphogenesis in camera-type eye|GO:0023051,regulation of signaling|GO:0030324,lung development|GO:0010647,positive regulation of cell communication|GO:0010646,regulation of cell communication|GO:0043086,negative regulation of catalytic activity|GO:0044699,single-organism process|GO:0007417,central nervous system development|GO:0009719,response to endogenous stimulus|GO:0050767,regulation of neurogenesis|GO:0001941,postsynaptic membrane organization|GO:0051240,positive regulation of multicellular organismal process|GO:0061008,hepaticobiliary system development|GO:0036120,cellular response to platelet-derived growth factor stimulus|GO:0060284,regulation of cell development|GO:0050769,positive regulation of neurogenesis|GO:0022008,neurogenesis|GO:0048513,animal organ development|GO:0033273,response to vitamin|GO:0022612,gland morphogenesis|GO:0022610,biological adhesion|GO:0008284,positive regulation of cell proliferation|GO:0032501,multicellular organismal process|GO:0008283,cell proliferation|GO:0050877,neurological system process|GO:0045837,negative regulation of membrane potential|GO:0071396,cellular response to lipid|GO:0045597,positive regulation of cell differentiation|GO:0045595,regulation of cell differentiation|GO:0001654,eye development|GO:0060219,camera-type eye photoreceptor cell differentiation|GO:0007420,brain development|GO:0050678,regulation of epithelial cell proliferation|GO:0050679,positive regulation of epithelial cell proliferation|GO:0051094,positive regulation of developmental process|GO:0060291,long-term synaptic potentiation|GO:0071407,cellular response to organic cyclic compound|GO:0042493,response to drug|GO:0050673,epithelial cell proliferation|GO:0014070,response to organic cyclic compound|GO:0048731,system development|GO:0048732,gland development|GO:0060541,respiratory system development|GO:0009991,response to extracellular stimulus|GO:0030323,respiratory tube development|GO:2000347,positive regulation of hepatocyte proliferation|GO:2000345,regulation of hepatocyte proliferation|GO:0030030,cell projection organization|GO:0071496,cellular response to external stimulus|GO:0001889,liver development|GO:0060221,retinal rod cell differentiation|GO:0051302,regulation of cell division|GO:0051301,cell division|GO:0033993,response to lipid|GO:0070482,response to oxygen levels|GO:0030282,bone mineralization|GO:0071310,cellular response to organic substance|GO:0048729,tissue morphogenesis|GO:0071305,cellular response to vitamin D|GO:0014823,response to activity|GO:0090596,sensory organ morphogenesis|GO:0048666,neuron development|GO:0009987,cellular process|GO:0030182,neuron differentiation|GO:0010810,regulation of cell-substrate adhesion|GO:0010811,positive regulation of cell-substrate adhesion|GO:0048592,eye morphogenesis|GO:0048593,camera-type eye morphogenesis|GO:0044767,single-organism developmental process|GO:0071363,cellular response to growth factor stimulus|GO:0044763,single-organism cellular process|GO:0007268,chemical synaptic transmission|GO:0007267,cell-cell signaling|GO:0007154,cell communication|GO:0035295,tube development|GO:0051179,localization|GO:1902578,single-organism localization|GO:0051641,cellular localization|GO:1901700,response to oxygen-containing compound|GO:1901701,cellular response to oxygen-containing compound|GO:0048699,generation of neurons|GO:0007399,nervous system development|GO:0097305,response to alcohol|GO:0048856,anatomical structure development|GO:0071495,cellular response to endogenous stimulus|GO:2000026,regulation of multicellular organismal development|GO:2000027,regulation of organ morphogenesis|GO:0030902,hindbrain development|GO:0043113,receptor clustering|GO:1902580,single-organism cellular localization|GO:0048522,positive regulation of cellular process|GO:0050680,negative regulation of epithelial cell proliferation|GO:0072359,circulatory system	GO:0005737,cytoplasm|GO:0005783,endoplasmic reticulum|GO:0044421,extracellular region part|GO:0005615,extracellular space|GO:0016020,membrane|GO:0044464,cell part|GO:0044444,cytoplasmic part|GO:0005623,cell|GO:0005622,intracellular|GO:0043229,intracellular organelle|GO:0005576,extracellular region|GO:0012505,endomembrane system|GO:0044424,intracellular part|GO:0048471,perinuclear region of cytoplasm|GO:0043227,membrane-bounded organelle|GO:0043226,organelle|GO:0043231,intracellular membrane-bounded organelle|GO:0031012,extracellular matrix|GO:0031594,neuromuscular junction|GO:0045202,synapse|GO:0009986,cell surface|GO:0005604,basement membrane|GO:0044420,extracellular matrix component|GO:0005578,proteinaceous extracellular matrix|GO:0032991,macromolecular complex	GO:1901681,sulfur compound binding|GO:0043168,anion binding|GO:0019212,phosphatase inhibitor activity|GO:0008083,growth factor activity|GO:0019888,protein phosphatase regulator activity|GO:0097367,carbohydrate derivative binding|GO:0030234,enzyme regulator activity|GO:0035373,chondroitin sulfate proteoglycan binding|GO:0005102,receptor binding|GO:0008201,heparin binding|GO:0005539,glycosaminoglycan binding|GO:0043167,ion binding|GO:0043394,proteoglycan binding|GO:0004864,protein phosphatase inhibitor activity|GO:0005488,binding|GO:0005515,protein binding|GO:0004857,enzyme inhibitor activity|GO:0098772,molecular function regulator|GO:0019208,phosphatase regulator activity|GO:0001948,glycoprotein binding|GO:0035374,chondroitin sulfate binding|GO:0038085,vascular endothelial growth factor binding|GO:1904399,heparan sulfate binding|GO:0019838,growth factor binding|GO:0019901,protein kinase binding|GO:0019900,kinase binding|GO:0019899,enzyme binding

DDX59	GO:0016070,RNA metabolic process|GO:0006139,nucleobase-containing compound metabolic process|GO:0044260,cellular macromolecule metabolic process|GO:0044238,primary metabolic process|GO:0009987,cellular process|GO:0006725,cellular aromatic compound metabolic process|GO:0044237,cellular metabolic process|GO:0043170,macromolecule metabolic process|GO:0090304,nucleic acid metabolic process|GO:0071704,organic substance metabolic process|GO:0034641,cellular nitrogen compound metabolic process|GO:0006807,nitrogen compound metabolic process|GO:0008152,metabolic process|GO:0010501,RNA secondary structure unwinding|GO:1901360,organic cyclic compound metabolic process|GO:0046483,heterocycle metabolic process	GO:0005737,cytoplasm|GO:0043231,intracellular membrane-bounded organelle|GO:0005634,nucleus|GO:0044464,cell part|GO:0005623,cell|GO:0005622,intracellular|GO:0043229,intracellular organelle|GO:0044424,intracellular part|GO:0043227,membrane-bounded organelle|GO:0043226,organelle|GO:0043232,intracellular non-membrane-bounded organelle|GO:0031981,nuclear lumen|GO:0043233,organelle lumen|GO:0005730,nucleolus|GO:0031974,membrane-enclosed lumen|GO:0044446,intracellular organelle part|GO:0070013,intracellular organelle lumen|GO:0043228,non-membrane-bounded organelle|GO:0044428,nuclear part|GO:0044422,organelle part	GO:0016887,ATPase activity|GO:0008026,ATP-dependent helicase activity|GO:0035639,purine ribonucleoside triphosphate binding|GO:0005488,binding|GO:0003676,nucleic acid binding|GO:0008186,RNA-dependent ATPase activity|GO:0043167,ion binding|GO:1901363,heterocyclic compound binding|GO:0001883,purine nucleoside binding|GO:0001882,nucleoside binding|GO:0043168,anion binding|GO:0043169,cation binding|GO:0042623,ATPase activity, coupled|GO:0000166,nucleotide binding|GO:1901265,nucleoside phosphate binding|GO:0032549,ribonucleoside binding|GO:0017076,purine nucleotide binding|GO:0046872,metal ion binding|GO:0005524,ATP binding|GO:0016787,hydrolase activity|GO:0017111,nucleoside-triphosphatase activity|GO:0004004,ATP-dependent RNA helicase activity|GO:0004386,helicase activity|GO:0036094,small molecule binding|GO:0003824,catalytic activity|GO:0016818,hydrolase activity, acting on acid anhydrides, in phosphorus-containing anhydrides|GO:0030554,adenyl nucleotide binding|GO:0097367,carbohydrate derivative binding|GO:0097159,organic cyclic compound binding|GO:0016817,hydrolase activity, acting on acid anhydrides|GO:0070035,purine NTP-dependent helicase activity|GO:0003724,RNA helicase activity|GO:0032559,adenyl ribonucleotide binding|GO:0003723,RNA binding|GO:0032555,purine ribonucleotide binding|GO:0016462,pyrophosphatase activity|GO:0032550,purine ribonucleoside binding|GO:0032553,ribonucleotide binding
KATNAL2	GO:0006996,organelle organization|GO:0007017,microtubule-based process|GO:0007010,cytoskeleton organization|GO:0071822,protein complex subunit organization|GO:0051013,microtubule severing|GO:0009987,cellular process|GO:0016043,cellular component organization|GO:0031122,cytoplasmic microtubule organization|GO:0043933,macromolecular complex subunit organization|GO:1902589,single-organism organelle organization|GO:0044763,single-organism cellular process|GO:0071840,cellular component organization or biogenesis|GO:0008152,metabolic process|GO:0000226,microtubule cytoskeleton organization|GO:0044699,single-organism process	GO:0005856,cytoskeleton|GO:0043234,protein complex|GO:0015630,microtubule cytoskeleton|GO:0043228,non-membrane-bounded organelle|GO:0043232,intracellular non-membrane-bounded organelle|GO:0044464,cell part|GO:0005623,cell|GO:0005737,cytoplasm|GO:0044446,intracellular organelle part|GO:0043229,intracellular organelle|GO:0044430,cytoskeletal part|GO:0044424,intracellular part|GO:0032991,macromolecular complex|GO:0005622,intracellular|GO:0005874,microtubule|GO:0043226,organelle|GO:0044422,organelle part|GO:0099512,supramolecular fiber|GO:0099513,polymeric cytoskeletal fiber|GO:0043227,membrane-bounded organelle|GO:0005634,nucleus|GO:0043231,intracellular membrane-bounded organelle|GO:0005819,spindle|GO:0000922,spindle pole	GO:0035639,purine ribonucleoside triphosphate binding|GO:0044877,macromolecular complex binding|GO:0005488,binding|GO:0016887,ATPase activity|GO:0043167,ion binding|GO:1901363,heterocyclic compound binding|GO:0001883,purine nucleoside binding|GO:0001882,nucleoside binding|GO:0043168,anion binding|GO:0042623,ATPase activity, coupled|GO:0008568,microtubule-severing ATPase activity|GO:0000166,nucleotide binding|GO:1901265,nucleoside phosphate binding|GO:0032549,ribonucleoside binding|GO:0032403,protein complex binding|GO:0017076,purine nucleotide binding|GO:0005524,ATP binding|GO:0016787,hydrolase activity|GO:0015631,tubulin binding|GO:0008017,microtubule binding|GO:0017111,nucleoside-triphosphatase activity|GO:0036094,small molecule binding|GO:0003824,catalytic activity|GO:0016818,hydrolase activity, acting on acid anhydrides, in phosphorus-containing anhydrides|GO:0030554,adenyl nucleotide binding|GO:0097367,carbohydrate derivative binding|GO:0097159,organic cyclic compound binding|GO:0008092,cytoskeletal protein binding|GO:0016817,hydrolase activity, acting on acid anhydrides|GO:0016462,pyrophosphatase activity|GO:0032559,adenyl ribonucleotide binding|GO:0005515,protein binding|GO:0032555,purine ribonucleotide binding|GO:0032550,purine ribonucleoside binding|GO:0032553,ribonucleotide binding
FAM27B			
TYROBP	GO:0048583,regulation of response to stimulus|GO:0001501,skeletal system development|GO:0007165,signal transduction|GO:0007166,cell surface receptor signaling pathway|GO:0002573,myeloid leukocyte differentiation|GO:0071840,cellular component organization or biogenesis|GO:0051716,cellular response to stimulus|GO:0042330,taxis|GO:0048869,cellular developmental process|GO:0002263,cell activation involved in immune response|GO:0048513,animal organ development|GO:0065007,biological regulation|GO:0006935,chemotaxis|GO:1903010,regulation of bone development|GO:0048468,cell development|GO:0097485,neuron projection guidance|GO:1903706,regulation of hemopoiesis|GO:0044700,single organism signaling|GO:0009605,response to external stimulus|GO:0044707,single-multicellular organism process|GO:0002275,myeloid cell activation involved in immune response|GO:0002274,myeloid leukocyte activation|GO:0002376,immune system process|GO:0006928,movement of cell or subcellular component|GO:0045321,leukocyte activation|GO:0098751,bone cell development|GO:0031175,neuron projection development|GO:0035556,intracellular signal transduction|GO:0006955,immune response|GO:0050789,regulation of biological process|GO:0002761,regulation of myeloid leukocyte differentiation|GO:0002283,neutrophil activation involved in immune response|GO:0002281,macrophage activation involved in immune response|GO:0000902,cell morphogenesis|GO:0016043,cellular component organization|GO:0002366,leukocyte activation involved in immune response|GO:0002682,regulation of immune system process|GO:0061564,axon development|GO:0050793,regulation of developmental process|GO:0050794,regulation of cellular process|GO:0006952,defense response|GO:0001775,cell activation|GO:0051239,regulation of multicellular organismal process|GO:0030097,hemopoiesis|GO:0002521,leukocyte differentiation|GO:0002520,immune system development|GO:0007229,integrin-mediated signaling pathway|GO:0006950,response to stress|GO:0048812,neuron projection morphogenesis|GO:0045637,regulation of myeloid cell differentiation|GO:0030154,cell differentiation|GO:0023052,signaling|GO:0042221,response to chemical|GO:0007411,axon guidance|GO:0009653,anatomical structure morphogenesis|GO:0044699,single-organism process|GO:0060284,regulation of cell development|GO:0030030,cell projection organization|GO:0032502,developmental process|GO:0032501,multicellular organismal process|GO:0030316,osteoclast differentiation|GO:0045595,regulation of cell differentiation|GO:0036230,granulocyte activation|GO:0007409,axonogenesis|GO:0048858,cell projection morphogenesis|GO:0050776,regulation of immune response|GO:0036035,osteoclast development|GO:0030099,myeloid cell differentiation|GO:0048731,system development|GO:0050896,response to stimulus|GO:0006968,cellular defense response|GO:0042119,neutrophil activation|GO:0042116,macrophage activation|GO:0060348,bone development|GO:0061515,myeloid cell development|GO:0007275,multicellular organism development|GO:0032989,cellular component morphogenesis|GO:0048534,hematopoietic or lymphoid organ development|GO:0048666,neuron development|GO:0048667,cell morphogenesis involved in neuron differentiation|GO:0009987,cellular process|GO:2001204,regulation of osteoclast development|GO:0030182,neuron differentiation|GO:0045087,innate immune response|GO:0044767,single-organism developmental process|GO:0000904,cell morphogenesis involved in differentiation|GO:0044763,single-organism cellular process|GO:0007154,cell communication|GO:0022008,neurogenesis|GO:0040011,locomotion|GO:0048699,generation of neurons|GO:0032990,cell part morphogenesis|GO:0007399,nervous system development|GO:0048856,anatomical structure development|GO:1902105,regulation of leukocyte differentiation|GO:2000026,regulation of multicellular organismal development|GO:0045670,regulation of osteoclast differentiation|GO:0002252,immune effector process|GO:0016192,vesicle-mediated transport|GO:0006887,exocytosis|GO:0045055,regulated exocytosis|GO:0006810,transport|GO:0051234,establishment of localization|GO:0046903,secretion|GO:0002446,neutrophil mediated immunity|GO:0032940,secretion by cell|GO:0043312,neutrophil degranulation|GO:0044765,single-organism transport|GO:0002443,leukocyte mediated immunity|GO:0002444,myeloid leukocyte mediated immunity|GO:0051179,localization|GO:1902578,single-organism localization|GO:0043299,leukocyte degranulation	GO:0031226,intrinsic component of plasma membrane|GO:0016021,integral component of membrane|GO:0016020,membrane|GO:0044464,cell part|GO:0005623,cell|GO:0005622,intracellular|GO:0071944,cell periphery|GO:0005887,integral component of plasma membrane|GO:0005886,plasma membrane|GO:0044425,membrane part|GO:0044459,plasma membrane part|GO:0009986,cell surface|GO:0031224,intrinsic component of membrane|GO:0043229,intracellular organelle|GO:0043226,organelle|GO:0005737,cytoplasm|GO:0030667,secretory granule membrane|GO:0031090,organelle membrane|GO:0016023,cytoplasmic, membrane-bounded vesicle|GO:0031410,cytoplasmic vesicle|GO:0044444,cytoplasmic part|GO:0031988,membrane-bounded vesicle|GO:0044433,cytoplasmic vesicle part|GO:0099503,secretory vesicle|GO:0030141,secretory granule|GO:0098588,bounding membrane of organelle|GO:0030659,cytoplasmic vesicle membrane|GO:0097708,intracellular vesicle|GO:0012506,vesicle membrane|GO:0031982,vesicle|GO:0043231,intracellular membrane-bounded organelle|GO:0044446,intracellular organelle part|GO:0043227,membrane-bounded organelle|GO:0044424,intracellular part|GO:0098805,whole membrane|GO:0044422,organelle part|GO:0012505,endomembrane system	GO:0004871,signal transducer activity|GO:0005057,signal transducer activity, downstream of receptor|GO:0060089,molecular transducer activity|GO:0005488,binding|GO:0005515,protein binding|GO:0005102,receptor binding|GO:0042802,identical protein binding
GOLT1B	GO:0008104,protein localization|GO:0010647,positive regulation of cell communication|GO:0048584,positive regulation of response to stimulus|GO:0048583,regulation of response to stimulus|GO:0023056,positive regulation of signaling|GO:0023052,signaling|GO:0007165,signal transduction|GO:0023051,regulation of signaling|GO:0035556,intracellular signal transduction|GO:0010646,regulation of cell communication|GO:0050789,regulation of biological process|GO:0044699,single-organism process|GO:0044710,single-organism metabolic process|GO:0051234,establishment of localization|GO:0009966,regulation of signal transduction|GO:0009967,positive regulation of signal transduction|GO:0071704,organic substance metabolic process|GO:0065007,biological regulation|GO:0071702,organic substance transport|GO:0048518,positive regulation of biological process|GO:0033036,macromolecule localization|GO:0043122,regulation of I-kappaB kinase/NF-kappaB signaling|GO:0043123,positive regulation of I-kappaB kinase/NF-kappaB signaling|GO:0006810,transport|GO:0006629,lipid metabolic process|GO:0044238,primary metabolic process|GO:0009987,cellular process|GO:0051716,cellular response to stimulus|GO:0050794,regulation of cellular process|GO:0045184,establishment of protein localization|GO:0044763,single-organism cellular process|GO:0008152,metabolic process|GO:1902533,positive regulation of intracellular signal transduction|GO:1902531,regulation of intracellular signal transduction|GO:0051179,localization|GO:0044700,single organism signaling|GO:0016192,vesicle-mediated transport|GO:0050896,response to stimulus|GO:0007249,I-kappaB kinase/NF-kappaB signaling|GO:0007154,cell communication|GO:0015031,protein transport|GO:0048522,positive regulation of cellular process|GO:0015833,peptide transport|GO:0071705,nitrogen compound transport|GO:0042886,amide transport	GO:0005783,endoplasmic reticulum|GO:0043229,intracellular organelle|GO:0005622,intracellular|GO:0043227,membrane-bounded organelle|GO:0043226,organelle|GO:0031224,intrinsic component of membrane|GO:0005737,cytoplasm|GO:0031090,organelle membrane|GO:0016021,integral component of membrane|GO:0016020,membrane|GO:0044431,Golgi apparatus part|GO:0005794,Golgi apparatus|GO:0098588,bounding membrane of organelle|GO:0012505,endomembrane system|GO:0043231,intracellular membrane-bounded organelle|GO:0044464,cell part|GO:0005623,cell|GO:0000139,Golgi membrane|GO:0044446,intracellular organelle part|GO:0044444,cytoplasmic part|GO:0044424,intracellular part|GO:0044425,membrane part|GO:0044422,organelle part|GO:0031984,organelle subcompartment|GO:0098791,Golgi subcompartment	GO:0016787,hydrolase activity|GO:0003824,catalytic activity|GO:0016788,hydrolase activity, acting on ester bonds|GO:0060089,molecular transducer activity|GO:0004871,signal transducer activity|GO:0016298,lipase activity
WFDC8	GO:0009892,negative regulation of metabolic process|GO:0080090,regulation of primary metabolic process|GO:0019222,regulation of metabolic process|GO:0031324,negative regulation of cellular metabolic process|GO:0031323,regulation of cellular metabolic process|GO:0043086,negative regulation of catalytic activity|GO:0044267,cellular protein metabolic process|GO:0051248,negative regulation of protein metabolic process|GO:0010605,negative regulation of macromolecule metabolic process|GO:0051346,negative regulation of hydrolase activity|GO:0044260,cellular macromolecule metabolic process|GO:0071704,organic substance metabolic process|GO:0051246,regulation of protein metabolic process|GO:0050789,regulation of biological process|GO:0060255,regulation of macromolecule metabolic process|GO:0010466,negative regulation of peptidase activity|GO:0065007,biological regulation|GO:0044092,negative regulation of molecular function|GO:0048519,negative regulation of biological process|GO:0065009,regulation of molecular function|GO:0009987,cellular process|GO:0052547,regulation of peptidase activity|GO:0052548,regulation of endopeptidase activity|GO:0050790,regulation of catalytic activity|GO:0050794,regulation of cellular process|GO:0030162,regulation of proteolysis|GO:0008152,metabolic process|GO:0006508,proteolysis|GO:0010951,negative regulation of endopeptidase activity|GO:0051336,regulation of hydrolase activity|GO:0044238,primary metabolic process|GO:0032269,negative regulation of cellular protein metabolic process|GO:0032268,regulation of cellular protein metabolic process|GO:0019538,protein metabolic process|GO:0044237,cellular metabolic process|GO:0043170,macromolecule metabolic process|GO:0045861,negative regulation of proteolysis|GO:0048523,negative regulation of cellular process|GO:0006807,nitrogen compound metabolic process|GO:1901564,organonitrogen compound metabolic process	GO:0005576,extracellular region	GO:0004866,endopeptidase inhibitor activity|GO:0061135,endopeptidase regulator activity|GO:0061134,peptidase regulator activity|GO:0030414,peptidase inhibitor activity|GO:0004867,serine-type endopeptidase inhibitor activity|GO:0004857,enzyme inhibitor activity|GO:0098772,molecular function regulator|GO:0030234,enzyme regulator activity
ZP1	GO:0008037,cell recognition|GO:0044703,multi-organism reproductive process|GO:0044702,single organism reproductive process|GO:0009988,cell-cell recognition|GO:0000003,reproduction|GO:0032504,multicellular organism reproduction|GO:0009987,cellular process|GO:0009566,fertilization|GO:0035036,sperm-egg recognition|GO:0019953,sexual reproduction|GO:0022414,reproductive process|GO:0032501,multicellular organismal process|GO:0044763,single-organism cellular process|GO:0007338,single fertilization|GO:0007339,binding of sperm to zona pellucida|GO:0044699,single-organism process|GO:0051704,multi-organism process	GO:0044421,extracellular region part|GO:0031012,extracellular matrix|GO:0043229,intracellular organelle|GO:0071944,cell periphery|GO:0043227,membrane-bounded organelle|GO:0043226,organelle|GO:0031224,intrinsic component of membrane|GO:0005737,cytoplasm|GO:0031982,vesicle|GO:0016023,cytoplasmic, membrane-bounded vesicle|GO:0031410,cytoplasmic vesicle|GO:0016021,integral component of membrane|GO:0016020,membrane|GO:0031988,membrane-bounded vesicle|GO:0005794,Golgi apparatus|GO:0030141,secretory granule|GO:0012505,endomembrane system|GO:0005886,plasma membrane|GO:0043231,intracellular membrane-bounded organelle|GO:0044464,cell part|GO:0005623,cell|GO:0005622,intracellular|GO:0044444,cytoplasmic part|GO:0005576,extracellular region|GO:0044424,intracellular part|GO:0044425,membrane part|GO:0005578,proteinaceous extracellular	
DEPDC1B	GO:0009893,positive regulation of metabolic process|GO:0019222,regulation of metabolic process|GO:0048584,positive regulation of response to stimulus|GO:0048583,regulation of response to stimulus|GO:0006928,movement of cell or subcellular component|GO:0023056,positive regulation of signaling|GO:0030111,regulation of Wnt signaling pathway|GO:0051674,localization of cell|GO:0023052,signaling|GO:0007165,signal transduction|GO:0007166,cell surface receptor signaling pathway|GO:0023051,regulation of signaling|GO:0035556,intracellular signal transduction|GO:0010646,regulation of cell communication|GO:0043087,regulation of GTPase activity|GO:0010647,positive regulation of cell communication|GO:0043085,positive regulation of catalytic activity|GO:0051716,cellular response to stimulus|GO:1902531,regulation of intracellular signal transduction|GO:0043547,positive regulation of GTPase activity|GO:0051345,positive regulation of hydrolase activity|GO:0009966,regulation of signal transduction|GO:0009967,positive regulation of signal transduction|GO:0050789,regulation of biological process|GO:0065007,biological regulation|GO:0044699,single-organism process|GO:0044093,positive regulation of molecular function|GO:0048518,positive regulation of biological process|GO:0065009,regulation of molecular function|GO:0016477,cell migration|GO:0051056,regulation of small GTPase mediated signal transduction|GO:0009987,cellular process|GO:0050790,regulation of catalytic activity|GO:0050794,regulation of cellular process|GO:0044763,single-organism cellular process|GO:0040011,locomotion|GO:0008152,metabolic process|GO:0007154,cell communication|GO:0007264,small GTPase mediated signal transduction|GO:0051179,localization|GO:0051336,regulation of hydrolase activity|GO:0044700,single organism signaling|GO:0048870,cell motility|GO:0050896,response to stimulus|GO:0016055,Wnt signaling pathway|GO:0030177,positive regulation of Wnt signaling pathway|GO:0048522,positive regulation of cellular process|GO:0198738,cell-cell signaling by wnt|GO:1905114,cell surface receptor signaling pathway involved in cell-cell signaling|GO:0007267,cell-cell signaling	GO:0005737,cytoplasm|GO:0005829,cytosol|GO:0044464,cell part|GO:0005623,cell|GO:0005622,intracellular|GO:0044444,cytoplasmic part|GO:0044424,intracellular part	GO:0030234,enzyme regulator activity|GO:0005096,GTPase activator activity|GO:0030695,GTPase regulator activity|GO:0008047,enzyme activator activity|GO:0098772,molecular function regulator|GO:0060589,nucleoside-triphosphatase regulator activity
FGF7	GO:0019538,protein metabolic process|GO:0044238,primary metabolic process|GO:0019220,regulation of phosphate metabolic process|GO:0080090,regulation of primary metabolic process|GO:0019222,regulation of metabolic process|GO:0060249,anatomical structure homeostasis|GO:0060429,epithelium development|GO:0051549,positive regulation of keratinocyte migration|GO:0048583,regulation of response to stimulus|GO:0051607,defense response to virus|GO:0008585,female gonad development|GO:0009607,response to biotic stimulus|GO:0008104,protein localization|GO:0007165,signal transduction|GO:0007166,cell surface receptor signaling pathway|GO:0007167,enzyme linked receptor protein signaling pathway|GO:0007169,transmembrane receptor protein tyrosine kinase signaling pathway|GO:0098773,skin epidermis development|GO:0018193,peptidyl-amino acid modification|GO:1901362,organic cyclic compound biosynthetic process|GO:0001894,tissue homeostasis|GO:0031347,regulation of defense response|GO:0050688,regulation of defense response to virus|GO:0002009,morphogenesis of an epithelium|GO:0044710,single-organism metabolic process|GO:0044711,single-organism biosynthetic process|GO:0090407,organophosphate biosynthetic process|GO:0010604,positive regulation of macromolecule metabolic process|GO:0042330,taxis|GO:0071704,organic substance metabolic process|GO:0071363,cellular response to growth factor stimulus|GO:1901653,cellular response to peptide|GO:0018212,peptidyl-tyrosine modification|GO:0010467,gene expression|GO:0045137,development of primary sexual characteristics|GO:0010033,response to organic substance|GO:0048511,rhythmic process|GO:0048513,animal organ development|GO:0043129,surfactant homeostasis|GO:0048518,positive regulation of biological process|GO:0065007,biological regulation|GO:0033036,macromolecule localization|GO:0042127,regulation of cell proliferation|GO:0042325,regulation of phosphorylation|GO:0038179,neurotrophin signaling pathway|GO:0048729,tissue morphogenesis|GO:0060255,regulation of macromolecule metabolic process|GO:0071375,cellular response to peptide hormone stimulus|GO:0048875,chemical homeostasis within a tissue|GO:0034645,cellular macromolecule biosynthetic process|GO:0003006,developmental process involved in reproduction|GO:0042221,response to chemical|GO:0032268,regulation of cellular protein metabolic process|GO:0007173,epidermal growth factor receptor signaling pathway|GO:0070848,response to growth factor|GO:2001141,regulation of RNA biosynthetic process|GO:0060665,regulation of branching involved in salivary gland morphogenesis by mesenchymal-epithelial signaling|GO:0046486,glycerolipid metabolic process|GO:0051707,response to other organism|GO:0031069,hair follicle morphogenesis|GO:0046483,heterocycle metabolic process|GO:0010634,positive regulation of epithelial cell migration|GO:0044700,single organism signaling|GO:0042327,positive regulation of phosphorylation|GO:0044702,single organism reproductive process|GO:0051716,cellular response to stimulus|GO:0010631,epithelial cell migration|GO:0010632,regulation of epithelial cell migration|GO:0048870,cell motility|GO:0050731,positive regulation of peptidyl-tyrosine phosphorylation|GO:0050730,regulation of peptidyl-tyrosine phosphorylation|GO:0010243,response to organonitrogen compound|GO:0048871,multicellular organismal homeostasis|GO:0006935,chemotaxis|GO:0048878,chemical homeostasis|GO:0002376,immune system process|GO:0042698,ovulation cycle|GO:0051781,positive regulation of cell division|GO:0019637,organophosphate metabolic process|GO:0019438,aromatic compound biosynthetic process|GO:0044281,small molecule metabolic process|GO:1901698,response to nitrogen compound|GO:0044707,single-multicellular organism process|GO:0009893,positive regulation of metabolic process|GO:0022602,ovulation cycle process|GO:0009891,positive regulation of biosynthetic process|GO:0006950,response to stress|GO:0044283,small molecule biosynthetic process|GO:0002831,regulation of response to biotic stimulus|GO:0006468,protein phosphorylation|GO:0006928,movement of cell or subcellular component|GO:0008152,metabolic process|GO:0032940,secretion by cell|GO:0010628,positive regulation of gene expression|GO:0006807,nitrogen compound metabolic process|GO:2000794,regulation of epithelial cell proliferation involved in lung morphogenesis|GO:0035556,intracellular signal transduction|GO:0043170,macromolecule metabolic process|GO:0050789,regulation of biological process|GO:0097659,nucleic acid-templated transcription|GO:0009605,response to external stimulus|GO:0044267,cellular protein metabolic process|GO:0007435,salivary gland morphogenesis|GO:0002764,immune response-regulating signaling pathway|GO:0044260,cellular macromolecule metabolic process|GO:0007431,salivary gland development|GO:0009987,cellular process|GO:0002768,immune response-regulating cell surface receptor signaling pathway|GO:0008406,gonad development|GO:0044344,cellular response to fibroblast growth factor stimulus|GO:0016043,cellular component organization|GO:0048589,developmental growth|GO:0002682,regulation of immune system process|GO:0071840,cellular component organization or biogenesis|GO:0018108,peptidyl-tyrosine phosphorylation|GO:0002252,immune effector process|GO:0016477,cell migration|GO:0022603,regulation of anatomical structure morphogenesis|GO:0071495,cellular response to endogenous stimulus|GO:0018130,heterocycle biosynthetic process|GO:0009719,response to endogenous stimulus|GO:0009887,animal organ morphogenesis|GO:0046545,development of primary female sexual characteristics|GO:0006629,lipid metabolic process|GO:0009308,amine metabolic process|GO:0050793,regulation of developmental process|GO:0006810,transport|GO:0009889,regulation of biosynthetic process|GO:0009888,tissue development|GO:0042060,wound healing|GO:0050794,regulation of cellular process|GO:0006952,defense response|GO:0043900,regulation of multi-organism process|GO:0036211,protein modification process|GO:0042439,ethanolamine-containing compound metabolic process|GO:1901700,response to oxygen-containing compound|GO:0051239,regulation of multicellular organismal process|GO:0048731,system development|GO:0048011,neurotrophin TRK receptor signaling pathway|GO:0006576,cellular biogenic amine metabolic process|GO:0051234,establishment of localization|GO:0048015,phosphatidylinositol-mediated signaling|GO:0048732,gland development|GO:0048017,inositol lipid-mediated signaling|GO:0000003,reproduction|GO:0016070,RNA metabolic process|GO:0044767,single-organism developmental process|GO:0046903,secretion|GO:0038095,Fc-epsilon receptor signaling pathway|GO:0044271,cellular nitrogen compound biosynthetic process|GO:0038093,Fc receptor signaling pathway|GO:0008654,phospholipid biosynthetic process|GO:0080134,regulation of response to stress|GO:0031401,positive regulation of protein modification process|GO:0043412,macromolecule modification|GO:0032502,developmental process|GO:0006355,regulation of transcription, DNA-templated|GO:0010557,positive regulation of macromolecule biosynthetic process|GO:0010556,regulation of macromolecule biosynthetic process|GO:2000145,regulation of cell motility|GO:0006351,transcription, DNA-templated|GO:2000147,positive regulation of cell motility|GO:0009615,response to virus|GO:0071774,response to fibroblast growth factor|GO:0043207,response to external biotic stimulus|GO:0009611,response to wounding|GO:0051240,positive regulation of multicellular organismal process|GO:0061033,secretion by lung epithelial cell involved in lung growth|GO:0032774,RNA biosynthetic process|GO:0032101,regulation of response to external stimulus|GO:0060541,respiratory system development|GO:0016310,phosphorylation|GO:0051649,establishment of localization in cell|GO:1901615,organic hydroxy compound metabolic process|GO:0042633,hair cycle|GO:0034641,cellular nitrogen compound metabolic process|GO:0030323,respiratory tube development|GO:0023052,signaling|GO:0038127,ERBB signaling pathway|GO:0070887,cellular response to chemical stimulus|GO:0050918,positive chemotaxis|GO:0001667,ameboidal-type cell migration|GO:0008544,epidermis development|GO:0008543,fibroblast growth factor receptor signaling pathway|GO:1901566,organonitrogen compound biosynthetic process|GO:0044699,single-organism process|GO:0043616,keratinocyte proliferation|GO:0006139,nucleobase-containing compound metabolic process|GO:0050896,response to stimulus|GO:0001541,ovarian follicle development|GO:0051674,localization of cell|GO:0035265,organ growth|GO:0046660,female sex differentiation|GO:0010562,positive regulation of phosphorus metabolic	GO:0005737,cytoplasm|GO:0005794,Golgi apparatus|GO:0043231,intracellular membrane-bounded organelle|GO:0044421,extracellular region part|GO:0044464,cell part|GO:0044444,cytoplasmic part|GO:0005623,cell|GO:0005622,intracellular|GO:0043229,intracellular organelle|GO:0005576,extracellular region|GO:0012505,endomembrane system|GO:0044424,intracellular part|GO:0043227,membrane-bounded organelle|GO:0043226,organelle|GO:0005615,extracellular space	GO:1901681,sulfur compound binding|GO:0042056,chemoattractant activity|GO:0008083,growth factor activity|GO:0070851,growth factor receptor binding|GO:0097367,carbohydrate derivative binding|GO:0043167,ion binding|GO:0005539,glycosaminoglycan binding|GO:0043168,anion binding|GO:0005488,binding|GO:0005515,protein binding|GO:0005102,receptor binding|GO:0005104,fibroblast growth factor receptor binding|GO:0008201,heparin binding|GO:0005111,type 2 fibroblast growth factor receptor binding|GO:0005088,Ras guanyl-nucleotide exchange factor activity|GO:0005085,guanyl-nucleotide exchange factor activity|GO:0035004,phosphatidylinositol 3-kinase activity|GO:0098772,molecular function regulator|GO:0046934,phosphatidylinositol-4,5-bisphosphate 3-kinase activity|GO:0052813,phosphatidylinositol bisphosphate kinase activity|GO:0016740,transferase activity|GO:0004713,protein tyrosine kinase activity|GO:0004672,protein kinase activity|GO:0016303,1-phosphatidylinositol-3-kinase activity|GO:0016301,kinase activity|GO:0003824,catalytic activity|GO:0052742,phosphatidylinositol kinase activity|GO:0016772,transferase activity, transferring phosphorus-containing groups|GO:0016773,phosphotransferase activity, alcohol group as acceptor
CKS1B	GO:1904029,regulation of cyclin-dependent protein kinase activity|GO:0019220,regulation of phosphate metabolic process|GO:0080090,regulation of primary metabolic process|GO:0019222,regulation of metabolic process|GO:0016310,phosphorylation|GO:0031323,regulation of cellular metabolic process|GO:1903047,mitotic cell cycle process|GO:0044770,cell cycle phase transition|GO:0044772,mitotic cell cycle phase transition|GO:0022402,cell cycle process|GO:0044237,cellular metabolic process|GO:0071900,regulation of protein serine/threonine kinase activity|GO:0050789,regulation of biological process|GO:0007049,cell cycle|GO:0044267,cellular protein metabolic process|GO:0044260,cellular macromolecule metabolic process|GO:0043549,regulation of kinase activity|GO:0051246,regulation of protein metabolic process|GO:0071704,organic substance metabolic process|GO:0065007,biological regulation|GO:0051301,cell division|GO:0031399,regulation of protein modification process|GO:0065009,regulation of molecular function|GO:0006468,protein phosphorylation|GO:0045859,regulation of protein kinase activity|GO:0000278,mitotic cell cycle|GO:0008283,cell proliferation|GO:0060255,regulation of macromolecule metabolic process|GO:0000082,G1/S transition of mitotic cell cycle|GO:0050790,regulation of catalytic activity|GO:0006464,cellular protein modification process|GO:0050794,regulation of cellular process|GO:0051174,regulation of phosphorus metabolic process|GO:0043412,macromolecule modification|GO:0036211,protein modification process|GO:0044763,single-organism cellular process|GO:0008152,metabolic process|GO:0044843,cell cycle G1/S phase transition|GO:0042325,regulation of phosphorylation|GO:0044238,primary metabolic process|GO:0044699,single-organism process|GO:0032268,regulation of cellular protein metabolic process|GO:0051726,regulation of cell cycle|GO:0019538,protein metabolic process|GO:0000079,regulation of cyclin-dependent protein serine/threonine kinase activity|GO:0051338,regulation of transferase activity|GO:0043170,macromolecule metabolic process|GO:0006796,phosphate-containing compound metabolic process|GO:0006793,phosphorus metabolic process|GO:0009987,cellular process|GO:0001932,regulation of protein phosphorylation|GO:1901362,organic cyclic compound biosynthetic process|GO:1901360,organic cyclic compound metabolic process|GO:0010604,positive regulation of macromolecule metabolic process|GO:0044093,positive regulation of molecular function|GO:0048518,positive regulation of biological process|GO:2001141,regulation of RNA biosynthetic process|GO:0046483,heterocycle metabolic process|GO:0042327,positive regulation of phosphorylation|GO:1901564,organonitrogen compound metabolic process|GO:0019438,aromatic compound biosynthetic process|GO:0009893,positive regulation of metabolic process|GO:0033674,positive regulation of kinase activity|GO:0010628,positive regulation of gene expression|GO:0071902,positive regulation of protein serine/threonine kinase activity|GO:0097659,nucleic acid-templated transcription|GO:0007346,regulation of mitotic cell cycle|GO:0051347,positive regulation of transferase activity|GO:1904031,positive regulation of cyclin-dependent protein kinase activity|GO:0043085,positive regulation of catalytic activity|GO:0018130,heterocycle biosynthetic process|GO:0009889,regulation of biosynthetic process|GO:0034654,nucleobase-containing compound biosynthetic process|GO:0045787,positive regulation of cell cycle|GO:0016070,RNA metabolic process|GO:0044271,cellular nitrogen compound biosynthetic process|GO:0031401,positive regulation of protein modification process|GO:0006355,regulation of transcription, DNA-templated|GO:0010557,positive regulation of macromolecule biosynthetic process|GO:0010556,regulation of macromolecule biosynthetic process|GO:0045737,positive regulation of cyclin-dependent protein serine/threonine kinase activity|GO:0006351,transcription, DNA-templated|GO:0032774,RNA biosynthetic process|GO:0044249,cellular biosynthetic process|GO:0034641,cellular nitrogen compound metabolic process|GO:0034645,cellular macromolecule biosynthetic process|GO:0006139,nucleobase-containing compound metabolic process|GO:0010562,positive regulation of phosphorus metabolic process|GO:0009891,positive regulation of biosynthetic process|GO:0051247,positive regulation of protein metabolic process|GO:0032270,positive regulation of cellular protein metabolic process|GO:1903508,positive regulation of nucleic acid-templated transcription|GO:0006725,cellular aromatic compound metabolic process|GO:1903506,regulation of nucleic acid-templated transcription|GO:0045893,positive regulation of transcription, DNA-templated|GO:0090304,nucleic acid metabolic process|GO:0051252,regulation of RNA metabolic process|GO:0051254,positive regulation of RNA metabolic process|GO:1902680,positive regulation of RNA biosynthetic process|GO:0006807,nitrogen compound metabolic process|GO:0045860,positive regulation of protein kinase activity|GO:0031328,positive regulation of cellular biosynthetic process|GO:0031326,regulation of cellular biosynthetic process|GO:0031325,positive regulation of cellular metabolic process|GO:2000112,regulation of cellular macromolecule biosynthetic process|GO:0010467,gene expression|GO:0010468,regulation of gene expression|GO:0045935,positive regulation of nucleobase-containing compound metabolic process|GO:0045937,positive regulation of phosphate metabolic process|GO:1901576,organic substance biosynthetic process|GO:0019219,regulation of nucleobase-containing compound metabolic process|GO:0009058,biosynthetic process|GO:0009059,macromolecule biosynthetic process|GO:0051171,regulation of nitrogen compound metabolic process|GO:0051173,positive regulation of nitrogen compound metabolic process|GO:0001934,positive regulation of protein phosphorylation|GO:0048522,positive	GO:0044446,intracellular organelle part|GO:0043231,intracellular membrane-bounded organelle|GO:0031981,nuclear lumen|GO:0043233,organelle lumen|GO:0005634,nucleus|GO:0044464,cell part|GO:0031974,membrane-enclosed lumen|GO:0005622,intracellular|GO:0005654,nucleoplasm|GO:0070013,intracellular organelle lumen|GO:0043229,intracellular organelle|GO:0044428,nuclear part|GO:0005623,cell|GO:0044424,intracellular part|GO:0043227,membrane-bounded organelle|GO:0043226,organelle|GO:0044422,organelle part|GO:0000307,cyclin-dependent protein kinase holoenzyme complex|GO:1902911,protein kinase complex|GO:1902494,catalytic complex|GO:0061695,transferase complex, transferring phosphorus-containing groups|GO:1902554,serine/threonine protein kinase complex|GO:1990234,transferase complex|GO:0031461,cullin-RING ubiquitin ligase complex|GO:0043234,protein complex|GO:0032991,macromolecular complex|GO:0000151,ubiquitin ligase complex|GO:0019005,SCF ubiquitin ligase complex	GO:0019207,kinase regulator activity|GO:0019887,protein kinase regulator activity|GO:0030234,enzyme regulator activity|GO:0016538,cyclin-dependent protein serine/threonine kinase regulator activity|GO:0005488,binding|GO:0005515,protein binding|GO:0098772,molecular function regulator|GO:0061575,cyclin-dependent protein serine/threonine kinase activator activity|GO:0042393,histone binding|GO:0019900,kinase binding|GO:0019209,kinase activator activity|GO:0032182,ubiquitin-like protein binding|GO:0019899,enzyme binding|GO:0019901,protein kinase binding|GO:0043130,ubiquitin binding|GO:0008047,enzyme activator activity|GO:0030295,protein kinase activator activity|GO:0043539,protein serine/threonine kinase activator activity
HEATR6			GO:0097159,organic cyclic compound binding|GO:0044822,poly(A) RNA binding|GO:0005488,binding|GO:0003676,nucleic acid binding|GO:1901363,heterocyclic compound binding|GO:0003723,RNA binding

MAN2A1	GO:0048468,cell development|GO:0071840,cellular component organization or biogenesis|GO:0044710,single-organism metabolic process|GO:0048869,cellular developmental process|GO:0018196,peptidyl-asparagine modification|GO:0018193,peptidyl-amino acid modification|GO:0048513,animal organ development|GO:0010720,positive regulation of cell development|GO:0048518,positive regulation of biological process|GO:0060541,respiratory system development|GO:0044707,single-multicellular organism process|GO:0044249,cellular biosynthetic process|GO:0019538,protein metabolic process|GO:0060042,retina morphogenesis in camera-type eye|GO:0061008,hepaticobiliary system development|GO:0050789,regulation of biological process|GO:0044267,cellular protein metabolic process|GO:0044260,cellular macromolecule metabolic process|GO:0016043,cellular component organization|GO:0065007,biological regulation|GO:0007005,mitochondrion organization|GO:0009887,animal organ morphogenesis|GO:0048593,camera-type eye morphogenesis|GO:0050793,regulation of developmental process|GO:0019318,hexose metabolic process|GO:0009888,tissue development|GO:0050794,regulation of cellular process|GO:0043412,macromolecule modification|GO:0043413,macromolecule glycosylation|GO:0051239,regulation of multicellular organismal process|GO:0044723,single-organism carbohydrate metabolic process|GO:0007423,sensory organ development|GO:0051962,positive regulation of nervous system development|GO:0051960,regulation of nervous system development|GO:0006517,protein deglycosylation|GO:0043010,camera-type eye development|GO:0008152,metabolic process|GO:0030154,cell differentiation|GO:0009311,oligosaccharide metabolic process|GO:0009790,embryo development|GO:0009792,embryo development ending in birth or egg hatching|GO:0060041,retina development in camera-type eye|GO:0034645,cellular macromolecule biosynthetic process|GO:0009653,anatomical structure morphogenesis|GO:0035295,tube development|GO:0044699,single-organism process|GO:0050767,regulation of neurogenesis|GO:0001701,in utero embryonic development|GO:0051240,positive regulation of multicellular organismal process|GO:0060284,regulation of cell development|GO:0050769,positive regulation of neurogenesis|GO:0036211,protein modification process|GO:0043009,chordate embryonic development|GO:0032502,developmental process|GO:0032501,multicellular organismal process|GO:0043687,post-translational protein modification|GO:0009987,cellular process|GO:0045597,positive regulation of cell differentiation|GO:0045595,regulation of cell differentiation|GO:0001654,eye development|GO:0007033,vacuole organization|GO:0007399,nervous system development|GO:0051094,positive regulation of developmental process|GO:1901137,carbohydrate derivative biosynthetic process|GO:0005996,monosaccharide metabolic process|GO:1901135,carbohydrate derivative metabolic process|GO:0043170,macromolecule metabolic process|GO:0048731,system development|GO:0048732,gland development|GO:0030323,respiratory tube development|GO:0030324,lung development|GO:0001889,liver development|GO:0009100,glycoprotein metabolic process|GO:0009101,glycoprotein biosynthetic process|GO:0006486,protein glycosylation|GO:0006487,protein N-linked glycosylation|GO:0007275,multicellular organism development|GO:0006013,mannose metabolic process|GO:0071704,organic substance metabolic process|GO:0048729,tissue morphogenesis|GO:0018279,protein N-linked glycosylation via asparagine|GO:0090596,sensory organ morphogenesis|GO:1901576,organic substance biosynthetic process|GO:0048592,eye morphogenesis|GO:0007585,respiratory gaseous exchange|GO:0070085,glycosylation|GO:0006464,cellular protein modification process|GO:0044767,single-organism developmental process|GO:0009058,biosynthetic process|GO:0009059,macromolecule biosynthetic process|GO:0044763,single-organism cellular process|GO:0022008,neurogenesis|GO:0006996,organelle organization|GO:0044238,primary metabolic process|GO:0048699,generation of neurons|GO:0005975,carbohydrate metabolic process|GO:0006491,N-glycan processing|GO:0048856,anatomical structure development|GO:0044237,cellular metabolic process|GO:2000026,regulation of multicellular organismal development|GO:0048286,lung alveolus development|GO:0048522,positive regulation of cellular process|GO:0044281,small molecule metabolic process|GO:1901564,organonitrogen compound metabolic process|GO:1901566,organonitrogen compound	GO:0043229,intracellular organelle|GO:0005622,intracellular|GO:0043227,membrane-bounded organelle|GO:0043226,organelle|GO:0031224,intrinsic component of membrane|GO:0005737,cytoplasm|GO:0031982,vesicle|GO:0016021,integral component of membrane|GO:0016020,membrane|GO:0031988,membrane-bounded vesicle|GO:0044431,Golgi apparatus part|GO:0005794,Golgi apparatus|GO:0098588,bounding membrane of organelle|GO:0012505,endomembrane system|GO:1903561,extracellular vesicle|GO:0031090,organelle membrane|GO:0043230,extracellular organelle|GO:0043231,intracellular membrane-bounded organelle|GO:0005801,cis-Golgi network|GO:0070062,extracellular exosome|GO:0044464,cell part|GO:0005623,cell|GO:0000139,Golgi membrane|GO:0044446,intracellular organelle part|GO:0044444,cytoplasmic part|GO:0005576,extracellular region|GO:0044424,intracellular part|GO:0044425,membrane part|GO:0065010,extracellular membrane-bounded organelle|GO:0044421,extracellular region part|GO:0044422,organelle part|GO:0031984,organelle subcompartment|GO:0005615,extracellular space|GO:0098791,Golgi subcompartment|GO:0005795,Golgi stack|GO:0005797,Golgi medial cisterna|GO:0031985,Golgi cisterna	GO:0016787,hydrolase activity|GO:0015924,mannosyl-oligosaccharide mannosidase activity|GO:0015923,mannosidase activity|GO:0046914,transition metal ion binding|GO:0043169,cation binding|GO:0043167,ion binding|GO:0016799,hydrolase activity, hydrolyzing N-glycosyl compounds|GO:0016798,hydrolase activity, acting on glycosyl bonds|GO:0030246,carbohydrate binding|GO:0004572,mannosyl-oligosaccharide 1,3-1,6-alpha-mannosidase activity|GO:0003824,catalytic activity|GO:0008270,zinc ion binding|GO:0004553,hydrolase activity, hydrolyzing O-glycosyl compounds|GO:0046872,metal ion binding|GO:0004559,alpha-mannosidase activity|GO:0005488,binding
KIAA0355			
CSTA	GO:0018149,peptide cross-linking|GO:0009892,negative regulation of metabolic process|GO:0080090,regulation of primary metabolic process|GO:0019222,regulation of metabolic process|GO:0044092,negative regulation of molecular function|GO:0060429,epithelium development|GO:0031324,negative regulation of cellular metabolic process|GO:0031323,regulation of cellular metabolic process|GO:0030154,cell differentiation|GO:0007155,cell adhesion|GO:0032501,multicellular organismal process|GO:0016337,single organismal cell-cell adhesion|GO:0008544,epidermis development|GO:0043086,negative regulation of catalytic activity|GO:0044699,single-organism process|GO:0044267,cellular protein metabolic process|GO:0051248,negative regulation of protein metabolic process|GO:0010605,negative regulation of macromolecule metabolic process|GO:0051346,negative regulation of hydrolase activity|GO:0044260,cellular macromolecule metabolic process|GO:0071704,organic substance metabolic process|GO:0009987,cellular process|GO:0048869,cellular developmental process|GO:0051246,regulation of protein metabolic process|GO:0050789,regulation of biological process|GO:0060255,regulation of macromolecule metabolic process|GO:0010466,negative regulation of peptidase activity|GO:0065007,biological regulation|GO:0030855,epithelial cell differentiation|GO:0048519,negative regulation of biological process|GO:0065009,regulation of molecular function|GO:0022610,biological adhesion|GO:0043588,skin development|GO:0032502,developmental process|GO:0098602,single organism cell adhesion|GO:0030216,keratinocyte differentiation|GO:0052547,regulation of peptidase activity|GO:0052548,regulation of endopeptidase activity|GO:0050790,regulation of catalytic activity|GO:0009888,tissue development|GO:0006464,cellular protein modification process|GO:0050794,regulation of cellular process|GO:0044767,single-organism developmental process|GO:0043412,macromolecule modification|GO:0036211,protein modification process|GO:0030162,regulation of proteolysis|GO:0008152,metabolic process|GO:0006508,proteolysis|GO:0010951,negative regulation of endopeptidase activity|GO:0044238,primary metabolic process|GO:0051336,regulation of hydrolase activity|GO:0048513,animal organ development|GO:0032269,negative regulation of cellular protein metabolic process|GO:0032268,regulation of cellular protein metabolic process|GO:0044707,single-multicellular organism process|GO:0009913,epidermal cell differentiation|GO:0048856,anatomical structure development|GO:0044763,single-organism cellular process|GO:0044237,cellular metabolic process|GO:0043170,macromolecule metabolic process|GO:0019538,protein metabolic process|GO:0007275,multicellular organism development|GO:0048731,system development|GO:0045861,negative regulation of proteolysis|GO:0098609,cell-cell adhesion|GO:0048523,negative regulation of cellular process|GO:0008219,cell death|GO:0070268,cornification|GO:0012501,programmed cell death|GO:1901564,organonitrogen compound metabolic process|GO:0031424,keratinization|GO:0006807,nitrogen compound metabolic process	GO:0031974,membrane-enclosed lumen|GO:0043229,intracellular organelle|GO:0071944,cell periphery|GO:0043227,membrane-bounded organelle|GO:0043226,organelle|GO:0005737,cytoplasm|GO:0070062,extracellular exosome|GO:0031981,nuclear lumen|GO:0005615,extracellular space|GO:0005634,nucleus|GO:0016020,membrane|GO:0031988,membrane-bounded vesicle|GO:0005654,nucleoplasm|GO:0005886,plasma membrane|GO:1903561,extracellular vesicle|GO:0001533,cornified envelope|GO:0031982,vesicle|GO:0043230,extracellular organelle|GO:0043231,intracellular membrane-bounded organelle|GO:0043233,organelle lumen|GO:0044464,cell part|GO:0005623,cell|GO:0005622,intracellular|GO:0044446,intracellular organelle part|GO:0070013,intracellular organelle lumen|GO:0005576,extracellular region|GO:0044428,nuclear part|GO:0044424,intracellular part|GO:0065010,extracellular membrane-bounded organelle|GO:0044421,extracellular region part|GO:0044422,organelle part|GO:0031012,extracellular matrix|GO:0005829,cytosol|GO:0044444,cytoplasmic part	GO:0005488,binding|GO:0030674,protein binding, bridging|GO:0004866,endopeptidase inhibitor activity|GO:0061135,endopeptidase regulator activity|GO:0061134,peptidase regulator activity|GO:0019899,enzyme binding|GO:0005198,structural molecule activity|GO:0002020,protease binding|GO:0005515,protein binding|GO:0030414,peptidase inhibitor activity|GO:0004869,cysteine-type endopeptidase inhibitor activity|GO:0004857,enzyme inhibitor activity|GO:0098772,molecular function regulator|GO:0030234,enzyme regulator activity|GO:0060090,binding, bridging
SEC61G	GO:0008104,protein localization|GO:0061024,membrane organization|GO:0007165,signal transduction|GO:0071840,cellular component organization or biogenesis|GO:0051716,cellular response to stimulus|GO:0006986,response to unfolded protein|GO:0070727,cellular macromolecule localization|GO:0043043,peptide biosynthetic process|GO:0071310,cellular response to organic substance|GO:0033036,macromolecule localization|GO:0006605,protein targeting|GO:0045184,establishment of protein localization|GO:0072657,protein localization to membrane|GO:0010033,response to organic substance|GO:0044700,single organism signaling|GO:1901564,organonitrogen compound metabolic process|GO:0030968,endoplasmic reticulum unfolded protein response|GO:1901566,organonitrogen compound biosynthetic process|GO:0019538,protein metabolic process|GO:0002376,immune system process|GO:0033554,cellular response to stress|GO:0070887,cellular response to chemical stimulus|GO:0006613,cotranslational protein targeting to membrane|GO:0006612,protein targeting to membrane|GO:0006614,SRP-dependent cotranslational protein targeting to membrane|GO:0006807,nitrogen compound metabolic process|GO:0048002,antigen processing and presentation of peptide antigen|GO:0050789,regulation of biological process|GO:1901576,organic substance biosynthetic process|GO:0044260,cellular macromolecule metabolic process|GO:0006886,intracellular protein transport|GO:0016043,cellular component organization|GO:0065007,biological regulation|GO:0019882,antigen processing and presentation|GO:0019884,antigen processing and presentation of exogenous antigen|GO:0006810,transport|GO:0050794,regulation of cellular process|GO:0006950,response to stress|GO:0044802,single-organism membrane organization|GO:0008152,metabolic process|GO:0051234,establishment of localization|GO:0044271,cellular nitrogen compound biosynthetic process|GO:0046907,intracellular transport|GO:0050896,response to stimulus|GO:0009058,biosynthetic process|GO:0045047,protein targeting to ER|GO:0006518,peptide metabolic process|GO:0044249,cellular biosynthetic process|GO:0034641,cellular nitrogen compound metabolic process|GO:0023052,signaling|GO:0034645,cellular macromolecule biosynthetic process|GO:0007154,cell communication|GO:0044699,single-organism process|GO:0070972,protein localization to endoplasmic reticulum|GO:0071806,protein transmembrane transport|GO:0072599,establishment of protein localization to endoplasmic reticulum|GO:0072594,establishment of protein localization to organelle|GO:0009987,cellular process|GO:0055085,transmembrane transport|GO:0016482,cytosolic transport|GO:0043604,amide biosynthetic process|GO:0043603,cellular amide metabolic process|GO:0043170,macromolecule metabolic process|GO:0035967,cellular response to topologically incorrect protein|GO:0035966,response to topologically incorrect protein|GO:0033365,protein localization to organelle|GO:0034976,response to endoplasmic reticulum stress|GO:0042590,antigen processing and presentation of exogenous peptide antigen via MHC class I|GO:0034620,cellular response to unfolded protein|GO:0002474,antigen processing and presentation of peptide antigen via MHC class I|GO:0002479,antigen processing and presentation of exogenous peptide antigen via MHC class I, TAP-dependent|GO:0002478,antigen processing and presentation of exogenous peptide antigen|GO:0071704,organic substance metabolic process|GO:0010467,gene expression|GO:0071702,organic substance transport|GO:0044267,cellular protein metabolic process|GO:0034613,cellular protein localization|GO:0036498,IRE1-mediated unfolded protein response|GO:0044765,single-organism transport|GO:0009059,macromolecule biosynthetic process|GO:0044763,single-organism cellular process|GO:0051649,establishment of localization in cell|GO:0042221,response to chemical|GO:0051179,localization|GO:1902578,single-organism localization|GO:0051641,cellular localization|GO:0044238,primary metabolic process|GO:0090150,establishment of protein localization to membrane|GO:0044237,cellular metabolic process|GO:0015031,protein transport|GO:1902582,single-organism intracellular transport|GO:1902580,single-organism cellular localization|GO:0006412,translation|GO:0015833,peptide transport|GO:0071705,nitrogen compound transport|GO:0042886,amide transport	GO:0005783,endoplasmic reticulum|GO:0005789,endoplasmic reticulum membrane|GO:0042175,nuclear outer membrane-endoplasmic reticulum membrane network|GO:0043229,intracellular organelle|GO:0043227,membrane-bounded organelle|GO:0043226,organelle|GO:0031224,intrinsic component of membrane|GO:0005737,cytoplasm|GO:0031090,organelle membrane|GO:0016021,integral component of membrane|GO:0016020,membrane|GO:0044432,endoplasmic reticulum part|GO:0098588,bounding membrane of organelle|GO:0012505,endomembrane system|GO:0043231,intracellular membrane-bounded organelle|GO:0005829,cytosol|GO:0044464,cell part|GO:0005623,cell|GO:0005622,intracellular|GO:0044446,intracellular organelle part|GO:0044444,cytoplasmic part|GO:0044424,intracellular part|GO:0044425,membrane part|GO:0044422,organelle part|GO:0031984,organelle subcompartment|GO:0098827,endoplasmic reticulum subcompartment	GO:0008565,protein transporter activity|GO:0022891,substrate-specific transmembrane transporter activity|GO:0015450,P-P-bond-hydrolysis-driven protein transmembrane transporter activity|GO:0022884,macromolecule transmembrane transporter activity|GO:0005215,transporter activity|GO:0008320,protein transmembrane transporter activity|GO:0015399,primary active transmembrane transporter activity|GO:0022857,transmembrane transporter activity|GO:0022892,substrate-specific transporter activity|GO:0005488,binding|GO:0005515,protein binding|GO:0015405,P-P-bond-hydrolysis-driven transmembrane transporter activity|GO:0022804,active transmembrane transporter activity|GO:0042887,amide transmembrane transporter activity|GO:0015197,peptide transporter activity|GO:1904680,peptide transmembrane transporter activity
ABCC9	GO:0023052,signaling|GO:0007165,signal transduction|GO:0010107,potassium ion import|GO:0050789,regulation of biological process|GO:0044699,single-organism process|GO:0051716,cellular response to stimulus|GO:0071804,cellular potassium ion transport|GO:0071805,potassium ion transmembrane transport|GO:0009615,response to virus|GO:0051179,localization|GO:0065007,biological regulation|GO:0098662,inorganic cation transmembrane transport|GO:0098660,inorganic ion transmembrane transport|GO:0015672,monovalent inorganic cation transport|GO:0006810,transport|GO:0006813,potassium ion transport|GO:0006812,cation transport|GO:0006811,ion transport|GO:0009987,cellular process|GO:0050794,regulation of cellular process|GO:0006952,defense response|GO:0034220,ion transmembrane transport|GO:0044765,single-organism transport|GO:0030001,metal ion transport|GO:0008152,metabolic process|GO:0007268,chemical synaptic transmission|GO:0007267,cell-cell signaling|GO:0007154,cell communication|GO:0051234,establishment of localization|GO:0051707,response to other organism|GO:0055085,transmembrane transport|GO:1902578,single-organism localization|GO:0051704,multi-organism process|GO:0044700,single organism signaling|GO:0009607,response to biotic stimulus|GO:0051607,defense response to virus|GO:0009605,response to external stimulus|GO:0098542,defense response to other organism|GO:0050896,response to stimulus|GO:0043207,response to external biotic stimulus|GO:0006950,response to stress|GO:0002376,immune system process|GO:0002252,immune effector process|GO:0044763,single-organism cellular process|GO:0098655,cation transmembrane transport|GO:0042221,response to chemical|GO:0035637,multicellular organismal signaling|GO:0042493,response to drug|GO:0060047,heart contraction|GO:0023051,regulation of signaling|GO:1903779,regulation of cardiac conduction|GO:0065009,regulation of molecular function|GO:0032501,multicellular organismal process|GO:0061337,cardiac conduction|GO:0008015,blood circulation|GO:0008016,regulation of heart contraction|GO:1903522,regulation of blood circulation|GO:0051239,regulation of multicellular organismal process|GO:0003008,system process|GO:0044057,regulation of system process|GO:0044707,single-multicellular organism process|GO:0003013,circulatory system process|GO:0003015,heart process	GO:0030016,myofibril|GO:0030017,sarcomere|GO:0043229,intracellular organelle|GO:0043228,non-membrane-bounded organelle|GO:0034702,ion channel complex|GO:0034703,cation channel complex|GO:0042383,sarcolemma|GO:0031226,intrinsic component of plasma membrane|GO:0043226,organelle|GO:0031224,intrinsic component of membrane|GO:0005737,cytoplasm|GO:0016021,integral component of membrane|GO:0016020,membrane|GO:0044459,plasma membrane part|GO:1902495,transmembrane transporter complex|GO:0008282,ATP-sensitive potassium channel complex|GO:1990351,transporter complex|GO:0008076,voltage-gated potassium channel complex|GO:0044424,intracellular part|GO:0005887,integral component of plasma membrane|GO:0005886,plasma membrane|GO:0043234,protein complex|GO:0032991,macromolecular complex|GO:0043232,intracellular non-membrane-bounded organelle|GO:0071944,cell periphery|GO:0044464,cell part|GO:0005623,cell|GO:0005622,intracellular|GO:0098797,plasma membrane protein complex|GO:0098796,membrane protein complex|GO:0044444,cytoplasmic part|GO:0034705,potassium channel complex|GO:0043292,contractile fiber|GO:0044425,membrane part|GO:0044422,organelle part|GO:0044449,contractile fiber part|GO:0099512,supramolecular fiber	GO:0005267,potassium channel activity|GO:0038023,signaling receptor activity|GO:0016887,ATPase activity|GO:0035639,purine ribonucleoside triphosphate binding|GO:0060089,molecular transducer activity|GO:0004872,receptor activity|GO:0004871,signal transducer activity|GO:0005261,cation channel activity|GO:0044325,ion channel binding|GO:0098772,molecular function regulator|GO:1901363,heterocyclic compound binding|GO:0001883,purine nucleoside binding|GO:0001882,nucleoside binding|GO:0043168,anion binding|GO:0042626,ATPase activity, coupled to transmembrane movement of substances|GO:0016820,hydrolase activity, acting on acid anhydrides, catalyzing transmembrane movement of substances|GO:0042623,ATPase activity, coupled|GO:0043167,ion binding|GO:0004888,transmembrane signaling receptor activity|GO:0000166,nucleotide binding|GO:0015399,primary active transmembrane transporter activity|GO:1901265,nucleoside phosphate binding|GO:0032549,ribonucleoside binding|GO:0016247,channel regulator activity|GO:0022803,passive transmembrane transporter activity|GO:0046873,metal ion transmembrane transporter activity|GO:0015459,potassium channel regulator activity|GO:0022804,active transmembrane transporter activity|GO:0016787,hydrolase activity|GO:0022857,transmembrane transporter activity|GO:0008281,sulfonylurea receptor activity|GO:0005215,transporter activity|GO:0005216,ion channel activity|GO:0008324,cation transmembrane transporter activity|GO:0017111,nucleoside-triphosphatase activity|GO:0036094,small molecule binding|GO:0003824,catalytic activity|GO:0017076,purine nucleotide binding|GO:0022891,substrate-specific transmembrane transporter activity|GO:0022890,inorganic cation transmembrane transporter activity|GO:0016818,hydrolase activity, acting on acid anhydrides, in phosphorus-containing anhydrides|GO:0022892,substrate-specific transporter activity|GO:0030554,adenyl nucleotide binding|GO:0097367,carbohydrate derivative binding|GO:0097159,organic cyclic compound binding|GO:0043492,ATPase activity, coupled to movement of substances|GO:0016817,hydrolase activity, acting on acid anhydrides|GO:0032550,purine ribonucleoside binding|GO:0015075,ion transmembrane transporter activity|GO:0016462,pyrophosphatase activity|GO:0015077,monovalent inorganic cation transmembrane transporter activity|GO:0015267,channel activity|GO:0032559,adenyl ribonucleotide binding|GO:0005524,ATP binding|GO:0005515,protein binding|GO:0032555,purine ribonucleotide binding|GO:0022838,substrate-specific channel activity|GO:0015079,potassium ion transmembrane transporter activity|GO:0015405,P-P-bond-hydrolysis-driven transmembrane transporter activity|GO:0032553,ribonucleotide binding|GO:0005488,binding|GO:0043225,ATPase-coupled anion transmembrane transporter activity|GO:0042625,ATPase coupled ion transmembrane transporter activity|GO:0008509,anion transmembrane transporter activity|GO:0022853,active ion transmembrane transporter activity|GO:0099600,transmembrane
MCEE	GO:0034440,lipid oxidation|GO:0006631,fatty acid metabolic process|GO:0009259,ribonucleotide metabolic process|GO:0006635,fatty acid beta-oxidation|GO:0006732,coenzyme metabolic process|GO:0044237,cellular metabolic process|GO:0019752,carboxylic acid metabolic process|GO:0044248,cellular catabolic process|GO:0009062,fatty acid catabolic process|GO:0046491,L-methylmalonyl-CoA metabolic process|GO:0006807,nitrogen compound metabolic process|GO:0044281,small molecule metabolic process|GO:0044282,small molecule catabolic process|GO:0072521,purine-containing compound metabolic process|GO:0044242,cellular lipid catabolic process|GO:0035337,fatty-acyl-CoA metabolic process|GO:0009119,ribonucleoside metabolic process|GO:0044699,single-organism process|GO:0044712,single-organism catabolic process|GO:0006139,nucleobase-containing compound metabolic process|GO:0044710,single-organism metabolic process|GO:0051186,cofactor metabolic process|GO:0015936,coenzyme A metabolic process|GO:0009056,catabolic process|GO:1901360,organic cyclic compound metabolic process|GO:0042278,purine nucleoside metabolic process|GO:0033875,ribonucleoside bisphosphate metabolic process|GO:0016042,lipid catabolic process|GO:0009150,purine ribonucleotide metabolic process|GO:0071704,organic substance metabolic process|GO:0034032,purine nucleoside bisphosphate metabolic process|GO:0019626,short-chain fatty acid catabolic process|GO:0006637,acyl-CoA metabolic process|GO:0035383,thioester metabolic process|GO:0006629,lipid metabolic process|GO:0009987,cellular process|GO:0006725,cellular aromatic compound metabolic process|GO:1901575,organic substance catabolic process|GO:0032787,monocarboxylic acid metabolic process|GO:0055086,nucleobase-containing small molecule metabolic process|GO:0009117,nucleotide metabolic process|GO:0009116,nucleoside metabolic process|GO:0008152,metabolic process|GO:0072329,monocarboxylic acid catabolic process|GO:0043436,oxoacid metabolic process|GO:0019395,fatty acid oxidation|GO:0044255,cellular lipid metabolic process|GO:0046128,purine ribonucleoside metabolic process|GO:0030258,lipid modification|GO:0055114,oxidation-reduction process|GO:0046483,heterocycle metabolic process|GO:0044238,primary metabolic process|GO:1901564,organonitrogen compound metabolic process|GO:0006082,organic acid metabolic process|GO:0046395,carboxylic acid catabolic process|GO:0016054,organic acid catabolic process|GO:0034641,cellular nitrogen compound metabolic process|GO:0019693,ribose phosphate metabolic process|GO:0033865,nucleoside bisphosphate metabolic process|GO:0006163,purine nucleotide metabolic process|GO:1901657,glycosyl compound metabolic process|GO:0006796,phosphate-containing compound metabolic process|GO:0006790,sulfur compound metabolic process|GO:0006793,phosphorus metabolic process|GO:0019637,organophosphate metabolic process|GO:0044763,single-organism cellular process|GO:0006753,nucleoside phosphate metabolic process|GO:1901135,carbohydrate derivative metabolic process|GO:0046459,short-chain fatty acid metabolic process	GO:0005737,cytoplasm|GO:0043231,intracellular membrane-bounded organelle|GO:0043233,organelle lumen|GO:0044464,cell part|GO:0044444,cytoplasmic part|GO:0031974,membrane-enclosed lumen|GO:0005622,intracellular|GO:0044446,intracellular organelle part|GO:0070013,intracellular organelle lumen|GO:0043229,intracellular organelle|GO:0005739,mitochondrion|GO:0044429,mitochondrial part|GO:0005759,mitochondrial matrix|GO:0044424,intracellular part|GO:0005623,cell|GO:0043227,membrane-bounded organelle|GO:0043226,organelle|GO:0044422,organelle part	GO:0043169,cation binding|GO:0016854,racemase and epimerase activity|GO:0016853,isomerase activity|GO:0004493,methylmalonyl-CoA epimerase activity|GO:0043167,ion binding|GO:0003824,catalytic activity|GO:0005488,binding|GO:0005515,protein binding|GO:0046872,metal ion binding
C1orf227			
OR1E1	GO:0009593,detection of chemical stimulus|GO:0050906,detection of stimulus involved in sensory perception|GO:0007165,signal transduction|GO:0042221,response to chemical|GO:0050789,regulation of biological process|GO:0044699,single-organism process|GO:0051716,cellular response to stimulus|GO:0065007,biological regulation|GO:0007186,G-protein coupled receptor signaling pathway|GO:0032501,multicellular organismal process|GO:0007608,sensory perception of smell|GO:0050877,neurological system process|GO:0007606,sensory perception of chemical stimulus|GO:0007600,sensory perception|GO:0050794,regulation of cellular process|GO:0050911,detection of chemical stimulus involved in sensory perception of smell|GO:0044763,single-organism cellular process|GO:0007154,cell communication|GO:0003008,system process|GO:0044700,single organism signaling|GO:0051606,detection of stimulus|GO:0044707,single-multicellular organism process|GO:0050896,response to stimulus|GO:0023052,signaling|GO:0009987,cellular process|GO:0050907,detection of chemical stimulus involved in sensory perception	GO:0016021,integral component of membrane|GO:0016020,membrane|GO:0044464,cell part|GO:0005623,cell|GO:0071944,cell periphery|GO:0005886,plasma membrane|GO:0044425,membrane part|GO:0031224,intrinsic component of membrane	GO:0004930,G-protein coupled receptor activity|GO:0038023,signaling receptor activity|GO:0060089,molecular transducer activity|GO:0004888,transmembrane signaling receptor activity|GO:0004872,receptor activity|GO:0004871,signal transducer activity|GO:0004984,olfactory receptor activity|GO:0099600,transmembrane receptor activity
ATF1	GO:0032025,response to cobalt ion|GO:0080090,regulation of primary metabolic process|GO:0019222,regulation of metabolic process|GO:0048584,positive regulation of response to stimulus|GO:0048583,regulation of response to stimulus|GO:0034645,cellular macromolecule biosynthetic process|GO:0031349,positive regulation of defense response|GO:0007165,signal transduction|GO:0007166,cell surface receptor signaling pathway|GO:0007167,enzyme linked receptor protein signaling pathway|GO:0007169,transmembrane receptor protein tyrosine kinase signaling pathway|GO:1901362,organic cyclic compound biosynthetic process|GO:0031344,regulation of cell projection organization|GO:0023014,signal transduction by protein phosphorylation|GO:0031346,positive regulation of cell projection organization|GO:0044710,single-organism metabolic process|GO:0010604,positive regulation of macromolecule metabolic process|GO:0048869,cellular developmental process|GO:0071840,cellular component organization or biogenesis|GO:0000165,MAPK cascade|GO:0010467,gene expression|GO:0045664,regulation of neuron differentiation|GO:0045666,positive regulation of neuron differentiation|GO:0006260,DNA replication|GO:0010720,positive regulation of cell development|GO:0035666,TRIF-dependent toll-like receptor signaling pathway|GO:0065007,biological regulation|GO:0051054,positive regulation of DNA metabolic process|GO:0038179,neurotrophin signaling pathway|GO:0051052,regulation of DNA metabolic process|GO:0060255,regulation of macromolecule metabolic process|GO:0048468,cell development|GO:0034162,toll-like receptor 9 signaling pathway|GO:0042221,response to chemical|GO:0034166,toll-like receptor 10 signaling pathway|GO:0010976,positive regulation of neuron projection development|GO:0010975,regulation of neuron projection development|GO:2001141,regulation of RNA biosynthetic process|GO:0010035,response to inorganic substance|GO:0010033,response to organic substance|GO:0046483,heterocycle metabolic process|GO:0044700,single organism signaling|GO:0044707,single-multicellular organism process|GO:0044249,cellular biosynthetic process|GO:0019538,protein metabolic process|GO:0006355,regulation of transcription, DNA-templated|GO:0006275,regulation of DNA replication|GO:0002376,immune system process|GO:0033554,cellular response to stress|GO:0019438,aromatic compound biosynthetic process|GO:0010038,response to metal ion|GO:0022607,cellular component assembly|GO:0009893,positive regulation of metabolic process|GO:0009891,positive regulation of biosynthetic process|GO:0006950,response to stress|GO:0051254,positive regulation of RNA metabolic process|GO:0008152,metabolic process|GO:0014070,response to organic cyclic compound|GO:0010628,positive regulation of gene expression|GO:0031175,neuron projection development|GO:0045935,positive regulation of nucleobase-containing compound metabolic process|GO:0035556,intracellular signal transduction|GO:0050789,regulation of biological process|GO:0097659,nucleic acid-templated transcription|GO:0044267,cellular protein metabolic process|GO:0002764,immune response-regulating signaling pathway|GO:0044260,cellular macromolecule metabolic process|GO:0016043,cellular component organization|GO:0065003,macromolecular complex assembly|GO:0002684,positive regulation of immune system process|GO:0002682,regulation of immune system process|GO:0031347,regulation of defense response|GO:0006366,transcription from RNA polymerase II promoter|GO:0018130,heterocycle biosynthetic process|GO:0051130,positive regulation of cellular component organization|GO:0006139,nucleobase-containing compound metabolic process|GO:0050793,regulation of developmental process|GO:0009889,regulation of biosynthetic process|GO:0051716,cellular response to stimulus|GO:0050794,regulation of cellular process|GO:0006952,defense response|GO:0034146,toll-like receptor 5 signaling pathway|GO:0036211,protein modification process|GO:0034142,toll-like receptor 4 signaling pathway|GO:0006955,immune response|GO:0048011,neurotrophin TRK receptor signaling pathway|GO:0016070,RNA metabolic process|GO:0002755,MyD88-dependent toll-like receptor signaling pathway|GO:0002756,MyD88-independent toll-like receptor signaling pathway|GO:0002757,immune response-activating signal transduction|GO:0044271,cellular nitrogen compound biosynthetic process|GO:0080134,regulation of response to stress|GO:0002758,innate immune response-activating signal transduction|GO:0043412,macromolecule modification|GO:0002218,activation of innate immune response|GO:0010557,positive regulation of macromolecule biosynthetic process|GO:0010556,regulation of macromolecule biosynthetic process|GO:0006351,transcription, DNA-templated|GO:0051239,regulation of multicellular organismal process|GO:0070271,protein complex biogenesis|GO:0016310,phosphorylation|GO:0030154,cell differentiation|GO:0051128,regulation of cellular component organization|GO:0038123,toll-like receptor TLR1:TLR2 signaling pathway|GO:0034641,cellular nitrogen compound metabolic process|GO:0023052,signaling|GO:0038124,toll-like receptor TLR6:TLR2 signaling pathway|GO:0051960,regulation of nervous system development|GO:0070887,cellular response to chemical stimulus|GO:0070848,response to growth factor|GO:0044699,single-organism process|GO:0050767,regulation of neurogenesis|GO:0051240,positive regulation of multicellular organismal process|GO:0031098,stress-activated protein kinase signaling cascade|GO:0060284,regulation of cell development|GO:0050769,positive regulation of neurogenesis|GO:0043933,macromolecular complex subunit organization|GO:0045740,positive regulation of DNA replication|GO:0031326,regulation of cellular biosynthetic process|GO:0002224,toll-like receptor signaling pathway|GO:0002221,pattern recognition receptor signaling pathway|GO:1903508,positive regulation of nucleic acid-templated transcription|GO:0032501,multicellular organismal process|GO:0009987,cellular process|GO:0006725,cellular aromatic compound metabolic process|GO:1903506,regulation of nucleic acid-templated transcription|GO:0045597,positive regulation of cell differentiation|GO:0048518,positive regulation of biological process|GO:0045595,regulation of cell differentiation|GO:0045893,positive regulation of transcription, DNA-templated|GO:0050776,regulation of immune response|GO:0071363,cellular response to growth factor stimulus|GO:0051094,positive regulation of developmental process|GO:0051252,regulation of RNA metabolic process|GO:0050778,positive regulation of immune response|GO:0009059,macromolecule biosynthetic process|GO:0043170,macromolecule metabolic process|GO:1902680,positive regulation of RNA biosynthetic process|GO:0006807,nitrogen compound metabolic process|GO:0045944,positive regulation of transcription from RNA polymerase II promoter|GO:0048731,system development|GO:0050896,response to stimulus|GO:0032502,developmental process|GO:0032774,RNA biosynthetic process|GO:0034138,toll-like receptor 3 signaling pathway|GO:0031328,positive regulation of cellular biosynthetic process|GO:0030030,cell projection organization|GO:0034134,toll-like receptor 2 signaling pathway|GO:0031325,positive regulation of cellular metabolic process|GO:0031323,regulation of cellular metabolic process|GO:0090304,nucleic acid metabolic process|GO:0034622,cellular macromolecular complex assembly|GO:0007275,multicellular organism development|GO:0071822,protein complex subunit organization|GO:0051962,positive regulation of nervous system development|GO:1901360,organic cyclic compound metabolic process|GO:2000112,regulation of cellular macromolecule biosynthetic process|GO:0071704,organic substance metabolic process|GO:0071310,cellular response to organic substance|GO:0006357,regulation of transcription from RNA polymerase II promoter|GO:0010468,regulation of gene expression|GO:0048666,neuron development|GO:0006468,protein phosphorylation|GO:0045089,positive regulation of innate immune response|GO:0045088,regulation of innate immune response|GO:0030182,neuron differentiation|GO:1901576,organic substance biosynthetic process|GO:0019219,regulation of nucleobase-containing compound metabolic process|GO:0045087,innate immune response|GO:0006461,protein complex assembly|GO:0006464,cellular protein modification process|GO:0044767,single-organism developmental process|GO:0009058,biosynthetic process|GO:0051403,stress-activated MAPK cascade|GO:0044763,single-organism cellular process|GO:0051171,regulation of nitrogen compound metabolic process|GO:0051173,positive regulation of nitrogen compound metabolic process|GO:0007154,cell communication|GO:0022008,neurogenesis|GO:0043623,cellular protein complex	GO:0031974,membrane-enclosed lumen|GO:0043229,intracellular organelle|GO:1990590,ATF1-ATF4 transcription factor complex|GO:0043227,membrane-bounded organelle|GO:0043226,organelle|GO:0031981,nuclear lumen|GO:0005634,nucleus|GO:0005654,nucleoplasm|GO:0044798,nuclear transcription factor complex|GO:0043234,protein complex|GO:0032991,macromolecular complex|GO:0043231,intracellular membrane-bounded organelle|GO:0043233,organelle lumen|GO:0044464,cell part|GO:0090575,RNA polymerase II transcription factor complex|GO:0005623,cell|GO:0005622,intracellular|GO:0044446,intracellular organelle part|GO:0070013,intracellular organelle lumen|GO:0044428,nuclear part|GO:0005667,transcription factor complex|GO:0044424,intracellular part|GO:0044422,organelle part	GO:0001012,RNA polymerase II regulatory region DNA binding|GO:0003705,transcription factor activity, RNA polymerase II distal enhancer sequence-specific binding|GO:0044877,macromolecular complex binding|GO:0001071,nucleic acid binding transcription factor activity|GO:0003676,nucleic acid binding|GO:0003677,DNA binding|GO:1901363,heterocyclic compound binding|GO:0043565,sequence-specific DNA binding|GO:0032403,protein complex binding|GO:0000981,RNA polymerase II transcription factor activity, sequence-specific DNA binding|GO:0001067,regulatory region nucleic acid binding|GO:0044212,transcription regulatory region DNA binding|GO:0003700,transcription factor activity, sequence-specific DNA binding|GO:0097159,organic cyclic compound binding|GO:0001228,transcriptional activator activity, RNA polymerase II transcription regulatory region sequence-specific binding|GO:0000976,transcription regulatory region sequence-specific DNA binding|GO:0000977,RNA polymerase II regulatory region sequence-specific DNA binding|GO:0005515,protein binding|GO:0000975,regulatory region DNA binding|GO:0046983,protein dimerization activity|GO:0046982,protein heterodimerization activity|GO:0005488,binding|GO:1990837,sequence-specific double-stranded DNA binding|GO:0003690,double-stranded DNA binding|GO:0042802,identical protein binding

HMGA2	GO:0001501,skeletal system development|GO:2001038,regulation of cellular response to drug|GO:2001032,regulation of double-strand break repair via nonhomologous end joining|GO:2001033,negative regulation of double-strand break repair via nonhomologous end joining|GO:0051716,cellular response to stimulus|GO:0014031,mesenchymal cell development|GO:0006303,double-strand break repair via nonhomologous end joining|GO:0006302,double-strand break repair|GO:0043207,response to external biotic stimulus|GO:0006284,base-excision repair|GO:0006282,regulation of DNA repair|GO:0006281,DNA repair|GO:0060548,negative regulation of cell death|GO:0046483,heterocycle metabolic process|GO:0009607,response to biotic stimulus|GO:0009605,response to external stimulus|GO:0019538,protein metabolic process|GO:0090342,regulation of cell aging|GO:0090343,positive regulation of cell aging|GO:0009892,negative regulation of metabolic process|GO:0009893,positive regulation of metabolic process|GO:0009890,negative regulation of biosynthetic process|GO:0009891,positive regulation of biosynthetic process|GO:0019080,viral gene expression|GO:0019083,viral transcription|GO:0044033,multi-organism metabolic process|GO:0050789,regulation of biological process|GO:0000904,cell morphogenesis involved in differentiation|GO:0000902,cell morphogenesis|GO:1901360,organic cyclic compound metabolic process|GO:0018130,heterocycle biosynthetic process|GO:0007050,cell cycle arrest|GO:0043412,macromolecule modification|GO:2000780,negative regulation of double-strand break repair|GO:0032897,negative regulation of viral transcription|GO:0016070,RNA metabolic process|GO:1901987,regulation of cell cycle phase transition|GO:0039703,RNA replication|GO:0010557,positive regulation of macromolecule biosynthetic process|GO:0010556,regulation of macromolecule biosynthetic process|GO:0010558,negative regulation of macromolecule biosynthetic process|GO:0007492,endoderm development|GO:0007498,mesoderm development|GO:1901991,negative regulation of mitotic cell cycle phase transition|GO:1901990,regulation of mitotic cell cycle phase transition|GO:0031497,chromatin assembly|GO:0044818,mitotic G2/M transition checkpoint|GO:0000122,negative regulation of transcription from RNA polymerase II promoter|GO:0000280,nuclear division|GO:0045071,negative regulation of viral genome replication|GO:0060284,regulation of cell development|GO:0048513,animal organ development|GO:0072091,regulation of stem cell proliferation|GO:0008284,positive regulation of cell proliferation|GO:0008283,cell proliferation|GO:0006974,cellular response to DNA damage stimulus|GO:0043921,modulation by host of viral transcription|GO:0043922,negative regulation by host of viral transcription|GO:0006338,chromatin remodeling|GO:0006333,chromatin assembly or disassembly|GO:0018105,peptidyl-serine phosphorylation|GO:0035690,cellular response to drug|GO:0022402,cell cycle process|GO:0052472,modulation by host of symbiont transcription|GO:0045444,fat cell differentiation|GO:0008219,cell death|GO:0007275,multicellular organism development|GO:0051301,cell division|GO:2000112,regulation of cellular macromolecule biosynthetic process|GO:2000113,negative regulation of cellular macromolecule biosynthetic process|GO:0043065,positive regulation of apoptotic process|GO:0043067,regulation of programmed cell death|GO:0043066,negative regulation of apoptotic process|GO:0043069,negative regulation of programmed cell death|GO:0043068,positive regulation of programmed cell death|GO:0048598,embryonic morphogenesis|GO:0006468,protein phosphorylation|GO:0000278,mitotic cell cycle|GO:0019219,regulation of nucleobase-containing compound metabolic process|GO:0006464,cellular protein modification process|GO:0044767,single-organism developmental process|GO:0044764,multi-organism cellular process|GO:0044763,single-organism cellular process|GO:0060485,mesenchyme development|GO:0031049,programmed DNA elimination|GO:0010389,regulation of G2/M transition of mitotic cell cycle|GO:0051276,chromosome organization|GO:0048856,anatomical structure development|GO:0001837,epithelial to mesenchymal transition|GO:0006796,phosphate-containing compound metabolic process|GO:0006793,phosphorus metabolic process|GO:0048525,negative regulation of viral process|GO:0048523,negative regulation of cellular process|GO:0048522,positive regulation of cellular process|GO:0000086,G2/M transition of mitotic cell cycle|GO:0042769,DNA damage response, detection of DNA damage|GO:0044710,single-organism metabolic process|GO:0045786,negative regulation of cell cycle|GO:0045787,positive regulation of cell cycle|GO:0007369,gastrulation|GO:0044093,positive regulation of molecular function|GO:0044092,negative regulation of molecular function|GO:0002062,chondrocyte differentiation|GO:0051053,negative regulation of DNA metabolic process|GO:0051052,regulation of DNA metabolic process|GO:2001141,regulation of RNA biosynthetic process|GO:2001021,negative regulation of response to DNA damage stimulus|GO:0051702,interaction with symbiont|GO:2001023,regulation of response to drug|GO:2001022,positive regulation of response to DNA damage stimulus|GO:0051707,response to other organism|GO:0051704,multi-organism process|GO:0019827,stem cell population maintenance|GO:0031507,heterochromatin assembly|GO:0044249,cellular biosynthetic process|GO:0039694,viral RNA genome replication|GO:0039692,single stranded viral RNA replication via double stranded DNA intermediate|GO:0016568,chromatin modification|GO:0016569,covalent chromatin modification|GO:0010629,negative regulation of gene expression|GO:0010628,positive regulation of gene expression|GO:0044267,cellular protein metabolic process|GO:0044260,cellular macromolecule metabolic process|GO:0044699,single-organism process|GO:0006366,transcription from RNA polymerase II promoter|GO:0050793,regulation of developmental process|GO:0050792,regulation of viral process|GO:0009889,regulation of biosynthetic process|GO:0009888,tissue development|GO:0050794,regulation of cellular process|GO:0035988,chondrocyte proliferation|GO:0051239,regulation of multicellular organismal process|GO:0090402,oncogene-induced cell senescence|GO:0035987,endodermal cell differentiation|GO:0035986,senescence-associated heterochromatin focus assembly|GO:1903901,negative regulation of viral life cycle|GO:0052312,modulation of transcription in other organism involved in symbiotic interaction|GO:0050896,response to stimulus|GO:0071481,cellular response to X-ray|GO:0009314,response to radiation|GO:0070887,cellular response to chemical stimulus|GO:0007049,cell cycle|GO:0071479,cellular response to ionizing radiation|GO:0071478,cellular response to radiation|GO:0010212,response to ionizing radiation|GO:0010564,regulation of cell cycle process|GO:0035978,histone H2A-S139 phosphorylation|GO:2000683,regulation of cellular response to X-ray|GO:2000685,positive regulation of cellular response to X-ray|GO:0045069,regulation of viral genome replication|GO:0042493,response to drug|GO:1902680,positive regulation of RNA biosynthetic process|GO:0048731,system development|GO:0072089,stem cell proliferation|GO:0043933,macromolecular complex subunit organization|GO:0044772,mitotic cell cycle phase transition|GO:0006325,chromatin organization|GO:0006323,DNA packaging|GO:0071158,positive regulation of cell cycle arrest|GO:0071156,regulation of cell cycle arrest|GO:0045930,negative regulation of mitotic cell cycle|GO:0045935,positive regulation of nucleobase-containing compound metabolic process|GO:0045934,negative regulation of nucleobase-containing compound metabolic process|GO:0007267,cell-cell signaling|GO:0042221,response to chemical|GO:0006996,organelle organization|GO:0009628,response to abiotic stimulus|GO:0044237,cellular metabolic process|GO:0006259,DNA metabolic process|GO:0044403,symbiosis, encompassing mutualism through parasitism|GO:2000736,regulation of stem cell differentiation|GO:0019222,regulation of metabolic process|GO:0048585,negative regulation of response to stimulus|GO:0048584,positive regulation of response to stimulus|GO:0048583,regulation of response to stimulus|GO:0031052,chromosome breakage|GO:1901362,organic cyclic compound biosynthetic process|GO:0071840,cellular component organization or biogenesis|GO:0048863,stem cell differentiation|GO:0048864,stem cell development|GO:0048869,cellular developmental process|GO:0019058,viral life cycle|GO:0051817,modification of morphology or physiology of other organism involved in symbiotic interaction|GO:2000036,regulation of stem cell population maintenance|GO:0048518,positive regulation of biological process|GO:0048519,negative regulation of biological process|GO:0042127,regulation of cell	GO:0031974,membrane-enclosed lumen|GO:0043229,intracellular organelle|GO:0043228,non-membrane-bounded organelle|GO:0000785,chromatin|GO:0000228,nuclear chromosome|GO:0043227,membrane-bounded organelle|GO:0043226,organelle|GO:0031981,nuclear lumen|GO:0005634,nucleus|GO:0005654,nucleoplasm|GO:0005694,chromosome|GO:0000792,heterochromatin|GO:0035985,senescence-associated heterochromatin focus|GO:0043234,protein complex|GO:0032991,macromolecular complex|GO:0043231,intracellular membrane-bounded organelle|GO:0043232,intracellular non-membrane-bounded organelle|GO:0043233,organelle lumen|GO:0044464,cell part|GO:0005623,cell|GO:0005622,intracellular|GO:0044446,intracellular organelle part|GO:0070013,intracellular organelle lumen|GO:0044428,nuclear part|GO:0044422,organelle part|GO:0044424,intracellular part|GO:0032993,protein-DNA complex|GO:0044427,chromosomal part|GO:0071141,SMAD protein complex	GO:0046332,SMAD binding|GO:0031491,nucleosome binding|GO:0031490,chromatin DNA binding|GO:0031492,nucleosomal DNA binding|GO:0044877,macromolecular complex binding|GO:0005488,binding|GO:0003676,nucleic acid binding|GO:0003677,DNA binding|GO:0001078,transcriptional repressor activity, RNA polymerase II core promoter proximal region sequence-specific binding|GO:1901363,heterocyclic compound binding|GO:0019904,protein domain specific binding|GO:0043565,sequence-specific DNA binding|GO:0043566,structure-specific DNA binding|GO:0097159,organic cyclic compound binding|GO:0016829,lyase activity|GO:0004520,endodeoxyribonuclease activity|GO:0070742,C2H2 zinc finger domain binding|GO:0004674,protein serine/threonine kinase activity|GO:0016740,transferase activity|GO:0000989,transcription factor activity, transcription factor binding|GO:0004672,protein kinase activity|GO:0032403,protein complex binding|GO:0000982,transcription factor activity, RNA polymerase II core promoter proximal region sequence-specific binding|GO:0000981,RNA polymerase II transcription factor activity, sequence-specific DNA binding|GO:0000975,regulatory region DNA binding|GO:0016787,hydrolase activity|GO:0008134,transcription factor binding|GO:0016301,kinase activity|GO:0001067,regulatory region nucleic acid binding|GO:0016788,hydrolase activity, acting on ester bonds|GO:0035500,MH2 domain binding|GO:0003906,DNA-(apurinic or apyrimidinic site) lyase activity|GO:0003824,catalytic activity|GO:0008301,DNA binding, bending|GO:0035501,MH1 domain binding|GO:0001047,core promoter binding|GO:0003700,transcription factor activity, sequence-specific DNA binding|GO:0000988,transcription factor activity, protein binding|GO:0051575,5'-deoxyribose-5-phosphate lyase activity|GO:0044212,transcription regulatory region DNA binding|GO:0001227,transcriptional repressor activity, RNA polymerase II transcription regulatory region sequence-specific binding|GO:0004536,deoxyribonuclease activity|GO:0016835,carbon-oxygen lyase activity|GO:0001071,nucleic acid binding transcription factor activity|GO:0001228,transcriptional activator activity, RNA polymerase II transcription regulatory region sequence-specific binding|GO:0004518,nuclease activity|GO:0001077,transcriptional activator activity, RNA polymerase II core promoter proximal region sequence-specific binding|GO:0004519,endonuclease activity|GO:0005515,protein binding|GO:0016772,transferase activity, transferring phosphorus-containing groups|GO:0003680,AT DNA binding|GO:0035497,cAMP response element binding|GO:0003682,chromatin binding|GO:0016773,phosphotransferase activity, alcohol group as acceptor|GO:0004677,DNA-dependent protein kinase activity|GO:1990837,sequence-specific double-stranded DNA binding|GO:0003690,double-stranded DNA binding|GO:0001012,RNA polymerase II regulatory region DNA binding|GO:0001159,core
ARID3B	GO:0032774,RNA biosynthetic process|GO:0080090,regulation of primary metabolic process|GO:0019222,regulation of metabolic process|GO:0031328,positive regulation of cellular biosynthetic process|GO:0031326,regulation of cellular biosynthetic process|GO:0031325,positive regulation of cellular metabolic process|GO:0051173,positive regulation of nitrogen compound metabolic process|GO:0031323,regulation of cellular metabolic process|GO:0090304,nucleic acid metabolic process|GO:0044249,cellular biosynthetic process|GO:0034641,cellular nitrogen compound metabolic process|GO:0010628,positive regulation of gene expression|GO:0006807,nitrogen compound metabolic process|GO:0034645,cellular macromolecule biosynthetic process|GO:1901362,organic cyclic compound biosynthetic process|GO:0050789,regulation of biological process|GO:0097659,nucleic acid-templated transcription|GO:0009893,positive regulation of metabolic process|GO:0006139,nucleobase-containing compound metabolic process|GO:0010604,positive regulation of macromolecule metabolic process|GO:0044260,cellular macromolecule metabolic process|GO:0071704,organic substance metabolic process|GO:0009891,positive regulation of biosynthetic process|GO:2000112,regulation of cellular macromolecule biosynthetic process|GO:0060255,regulation of macromolecule metabolic process|GO:0010467,gene expression|GO:0010556,regulation of macromolecule biosynthetic process|GO:0065007,biological regulation|GO:1901360,organic cyclic compound metabolic process|GO:0006366,transcription from RNA polymerase II promoter|GO:0048518,positive regulation of biological process|GO:0010468,regulation of gene expression|GO:0018130,heterocycle biosynthetic process|GO:1903508,positive regulation of nucleic acid-templated transcription|GO:0045935,positive regulation of nucleobase-containing compound metabolic process|GO:1901576,organic substance biosynthetic process|GO:0019219,regulation of nucleobase-containing compound metabolic process|GO:0006725,cellular aromatic compound metabolic process|GO:0009987,cellular process|GO:0009889,regulation of biosynthetic process|GO:1903506,regulation of nucleic acid-templated transcription|GO:0050794,regulation of cellular process|GO:0009058,biosynthetic process|GO:0009059,macromolecule biosynthetic process|GO:0045893,positive regulation of transcription, DNA-templated|GO:0008152,metabolic process|GO:2001141,regulation of RNA biosynthetic process|GO:0034654,nucleobase-containing compound biosynthetic process|GO:0046483,heterocycle metabolic process|GO:0016070,RNA metabolic process|GO:0044238,primary metabolic process|GO:0044271,cellular nitrogen compound biosynthetic process|GO:0051252,regulation of RNA metabolic process|GO:0051254,positive regulation of RNA metabolic process|GO:0044237,cellular metabolic process|GO:0043170,macromolecule metabolic process|GO:1902680,positive regulation of RNA biosynthetic process|GO:0006355,regulation of transcription, DNA-templated|GO:0010557,positive regulation of macromolecule biosynthetic process|GO:0006357,regulation of transcription from RNA polymerase II promoter|GO:0045944,positive regulation of transcription from RNA polymerase II promoter|GO:0006351,transcription, DNA-templated|GO:0019438,aromatic compound biosynthetic process|GO:0048522,positive regulation of cellular process|GO:0051171,regulation of nitrogen compound metabolic process	GO:0044446,intracellular organelle part|GO:0043231,intracellular membrane-bounded organelle|GO:0031981,nuclear lumen|GO:0043233,organelle lumen|GO:0005634,nucleus|GO:0044464,cell part|GO:0031974,membrane-enclosed lumen|GO:0005622,intracellular|GO:0005654,nucleoplasm|GO:0070013,intracellular organelle lumen|GO:0043229,intracellular organelle|GO:0044428,nuclear part|GO:0005623,cell|GO:0044424,intracellular part|GO:0043227,membrane-bounded organelle|GO:0043226,organelle|GO:0044422,organelle part	GO:0001012,RNA polymerase II regulatory region DNA binding|GO:0001159,core promoter proximal region DNA binding|GO:0001158,enhancer sequence-specific DNA binding|GO:0001077,transcriptional activator activity, RNA polymerase II core promoter proximal region sequence-specific binding|GO:0005488,binding|GO:0003676,nucleic acid binding|GO:0003677,DNA binding|GO:1901363,heterocyclic compound binding|GO:0043565,sequence-specific DNA binding|GO:0003705,transcription factor activity, RNA polymerase II distal enhancer sequence-specific binding|GO:0000987,core promoter proximal region sequence-specific DNA binding|GO:0000982,transcription factor activity, RNA polymerase II core promoter proximal region sequence-specific binding|GO:0000981,RNA polymerase II transcription factor activity, sequence-specific DNA binding|GO:0000980,RNA polymerase II distal enhancer sequence-specific DNA binding|GO:0001067,regulatory region nucleic acid binding|GO:0035326,enhancer binding|GO:0001205,transcriptional activator activity, RNA polymerase II distal enhancer sequence-specific binding|GO:0044212,transcription regulatory region DNA binding|GO:0003700,transcription factor activity, sequence-specific DNA binding|GO:0097159,organic cyclic compound binding|GO:0001228,transcriptional activator activity, RNA polymerase II transcription regulatory region sequence-specific binding|GO:0000976,transcription regulatory region sequence-specific DNA binding|GO:0000977,RNA polymerase II regulatory region sequence-specific DNA binding|GO:0000975,regulatory region DNA binding|GO:0000978,RNA polymerase II core promoter proximal region sequence-specific DNA binding|GO:0001071,nucleic acid binding transcription factor
LIN28B	GO:0080090,regulation of primary metabolic process|GO:0019222,regulation of metabolic process|GO:0031050,dsRNA fragmentation|GO:0031054,pre-miRNA processing|GO:1901362,organic cyclic compound biosynthetic process|GO:1901360,organic cyclic compound metabolic process|GO:1901361,organic cyclic compound catabolic process|GO:0044710,single-organism metabolic process|GO:0010605,negative regulation of macromolecule metabolic process|GO:0010608,posttranscriptional regulation of gene expression|GO:0010467,gene expression|GO:0016458,gene silencing|GO:0048519,negative regulation of biological process|GO:0034470,ncRNA processing|GO:0060255,regulation of macromolecule metabolic process|GO:2001141,regulation of RNA biosynthetic process|GO:0010033,response to organic substance|GO:0046483,heterocycle metabolic process|GO:0043331,response to dsRNA|GO:0019439,aromatic compound catabolic process|GO:0019438,aromatic compound biosynthetic process|GO:0009892,negative regulation of metabolic process|GO:0034645,cellular macromolecule biosynthetic process|GO:0010629,negative regulation of gene expression|GO:0006807,nitrogen compound metabolic process|GO:0034661,ncRNA catabolic process|GO:0034660,ncRNA metabolic process|GO:0050789,regulation of biological process|GO:0097659,nucleic acid-templated transcription|GO:0070918,production of small RNA involved in gene silencing by RNA|GO:1901576,organic substance biosynthetic process|GO:1901575,organic substance catabolic process|GO:0044265,cellular macromolecule catabolic process|GO:0044260,cellular macromolecule metabolic process|GO:0071359,cellular response to dsRNA|GO:0065007,biological regulation|GO:0014070,response to organic cyclic compound|GO:0018130,heterocycle biosynthetic process|GO:0009889,regulation of biosynthetic process|GO:0051716,cellular response to stimulus|GO:0050794,regulation of cellular process|GO:0016441,posttranscriptional gene silencing|GO:0008152,metabolic process|GO:0034654,nucleobase-containing compound biosynthetic process|GO:0034655,nucleobase-containing compound catabolic process|GO:0046700,heterocycle catabolic process|GO:0016070,RNA metabolic process|GO:0044271,cellular nitrogen compound biosynthetic process|GO:0044270,cellular nitrogen compound catabolic process|GO:1901698,response to nitrogen compound|GO:1901699,cellular response to nitrogen compound|GO:0006355,regulation of transcription, DNA-templated|GO:0010556,regulation of macromolecule biosynthetic process|GO:0006351,transcription, DNA-templated|GO:0051171,regulation of nitrogen compound metabolic process|GO:0032774,RNA biosynthetic process|GO:0040029,regulation of gene expression, epigenetic|GO:0044248,cellular catabolic process|GO:0044249,cellular biosynthetic process|GO:0034641,cellular nitrogen compound metabolic process|GO:0070887,cellular response to chemical stimulus|GO:0044699,single-organism process|GO:0006139,nucleobase-containing compound metabolic process|GO:0009987,cellular process|GO:0006725,cellular aromatic compound metabolic process|GO:1903506,regulation of nucleic acid-templated transcription|GO:0071407,cellular response to organic cyclic compound|GO:0051252,regulation of RNA metabolic process|GO:0043170,macromolecule metabolic process|GO:0050896,response to stimulus|GO:0031326,regulation of cellular biosynthetic process|GO:0031323,regulation of cellular metabolic process|GO:0090304,nucleic acid metabolic process|GO:0010586,miRNA metabolic process|GO:0010587,miRNA catabolic process|GO:2000112,regulation of cellular macromolecule biosynthetic process|GO:0071704,organic substance metabolic process|GO:0071310,cellular response to organic substance|GO:0006401,RNA catabolic process|GO:0010468,regulation of gene expression|GO:0019219,regulation of nucleobase-containing compound metabolic process|GO:0031123,RNA 3'-end processing|GO:0009058,biosynthetic process|GO:0009059,macromolecule biosynthetic process|GO:0044763,single-organism cellular process|GO:0031047,gene silencing by RNA|GO:0042221,response to chemical|GO:0009056,catabolic process|GO:0009057,macromolecule catabolic process|GO:0044238,primary metabolic process|GO:0035194,posttranscriptional gene silencing by RNA|GO:0035195,gene silencing by miRNA|GO:0035196,production of miRNAs involved in gene silencing by miRNA|GO:0044237,cellular metabolic process|GO:0006396,RNA processing|GO:0043043,peptide biosynthetic process|GO:1901564,organonitrogen compound metabolic process|GO:1901566,organonitrogen compound biosynthetic process|GO:0019538,protein metabolic process|GO:0009890,negative regulation of biosynthetic process|GO:0044267,cellular protein metabolic process|GO:0006518,peptide metabolic process|GO:0010558,negative regulation of macromolecule biosynthetic process|GO:0051248,negative regulation of protein metabolic process|GO:0051246,regulation of protein metabolic process|GO:0034249,negative regulation of cellular amide metabolic process|GO:0034248,regulation of cellular amide metabolic process|GO:0043604,amide biosynthetic process|GO:0032269,negative regulation of cellular protein metabolic process|GO:0032268,regulation of cellular protein metabolic process|GO:0043603,cellular amide metabolic process|GO:0017148,negative regulation of translation|GO:0031327,negative regulation of cellular biosynthetic process|GO:0031324,negative regulation of cellular metabolic process|GO:2000113,negative regulation of cellular macromolecule biosynthetic process|GO:0051172,negative regulation of nitrogen compound metabolic process|GO:0048523,negative regulation of cellular process|GO:0006417,regulation of translation|GO:0006412,translation	GO:0016234,inclusion body|GO:0016235,aggresome|GO:0043229,intracellular organelle|GO:0043228,non-membrane-bounded organelle|GO:0043227,membrane-bounded organelle|GO:0043226,organelle|GO:0005737,cytoplasm|GO:0031981,nuclear lumen|GO:0005730,nucleolus|GO:0005634,nucleus|GO:0005739,mitochondrion|GO:0005654,nucleoplasm|GO:0031974,membrane-enclosed lumen|GO:0043231,intracellular membrane-bounded organelle|GO:0043232,intracellular non-membrane-bounded organelle|GO:0043233,organelle lumen|GO:0044464,cell part|GO:0005623,cell|GO:0005622,intracellular|GO:0044446,intracellular organelle part|GO:0070013,intracellular organelle lumen|GO:0044444,cytoplasmic part|GO:0044428,nuclear part|GO:0044424,intracellular part|GO:0044422,organelle part|GO:0005829,cytosol	GO:0005488,binding|GO:0043169,cation binding|GO:0005515,protein binding|GO:0046914,transition metal ion binding|GO:0097159,organic cyclic compound binding|GO:0044822,poly(A) RNA binding|GO:0043167,ion binding|GO:0008270,zinc ion binding|GO:0003676,nucleic acid binding|GO:0003677,DNA binding|GO:0046872,metal ion binding|GO:1901363,heterocyclic compound binding|GO:0003723,RNA binding
FIGN	GO:0080090,regulation of primary metabolic process|GO:0019222,regulation of metabolic process|GO:0043933,macromolecular complex subunit organization|GO:0048583,regulation of response to stimulus|GO:0006139,nucleobase-containing compound metabolic process|GO:1903047,mitotic cell cycle process|GO:0090304,nucleic acid metabolic process|GO:0034641,cellular nitrogen compound metabolic process|GO:0006807,nitrogen compound metabolic process|GO:0022402,cell cycle process|GO:0007067,mitotic nuclear division|GO:0050789,regulation of biological process|GO:0007049,cell cycle|GO:0000278,mitotic cell cycle|GO:0010569,regulation of double-strand break repair via homologous recombination|GO:0044710,single-organism metabolic process|GO:0006302,double-strand break repair|GO:0000725,recombinational repair|GO:0000724,double-strand break repair via homologous recombination|GO:0071840,cellular component organization or biogenesis|GO:0000280,nuclear division|GO:0016043,cellular component organization|GO:0060255,regulation of macromolecule metabolic process|GO:0065007,biological regulation|GO:0051301,cell division|GO:0006310,DNA recombination|GO:0006282,regulation of DNA repair|GO:0051052,regulation of DNA metabolic process|GO:0006281,DNA repair|GO:0019219,regulation of nucleobase-containing compound metabolic process|GO:0031323,regulation of cellular metabolic process|GO:0044260,cellular macromolecule metabolic process|GO:0009987,cellular process|GO:0006725,cellular aromatic compound metabolic process|GO:0051716,cellular response to stimulus|GO:0050794,regulation of cellular process|GO:0006974,cellular response to DNA damage stimulus|GO:0071704,organic substance metabolic process|GO:0006950,response to stress|GO:0044763,single-organism cellular process|GO:0051171,regulation of nitrogen compound metabolic process|GO:0008152,metabolic process|GO:2001020,regulation of response to DNA damage stimulus|GO:0031122,cytoplasmic microtubule organization|GO:0046483,heterocycle metabolic process|GO:0006996,organelle organization|GO:0044238,primary metabolic process|GO:0044699,single-organism process|GO:0007017,microtubule-based process|GO:0007010,cytoskeleton organization|GO:0071822,protein complex subunit organization|GO:0080134,regulation of response to stress|GO:0080135,regulation of cellular response to stress|GO:0000018,regulation of DNA recombination|GO:0044237,cellular metabolic process|GO:0043170,macromolecule metabolic process|GO:1902589,single-organism organelle organization|GO:0048285,organelle fission|GO:0033554,cellular response to stress|GO:2000779,regulation of double-strand break repair|GO:0006259,DNA metabolic process|GO:0000226,microtubule cytoskeleton organization|GO:1901360,organic cyclic compound metabolic process|GO:0050896,response to stimulus	GO:0031974,membrane-enclosed lumen|GO:0043229,intracellular organelle|GO:0043228,non-membrane-bounded organelle|GO:0005622,intracellular|GO:0043227,membrane-bounded organelle|GO:0043226,organelle|GO:0034399,nuclear periphery|GO:0031981,nuclear lumen|GO:0005874,microtubule|GO:0005634,nucleus|GO:0005815,microtubule organizing center|GO:0044430,cytoskeletal part|GO:0016363,nuclear matrix|GO:0005856,cytoskeleton|GO:0015630,microtubule cytoskeleton|GO:0043234,protein complex|GO:0032991,macromolecular complex|GO:0043231,intracellular membrane-bounded organelle|GO:0043232,intracellular non-membrane-bounded organelle|GO:0043233,organelle lumen|GO:0044464,cell part|GO:0005623,cell|GO:0005737,cytoplasm|GO:0044446,intracellular organelle part|GO:0070013,intracellular organelle lumen|GO:0044428,nuclear part|GO:0044424,intracellular part|GO:0044422,organelle part|GO:0099512,supramolecular fiber|GO:0099513,polymeric cytoskeletal fiber	GO:0008022,protein C-terminus binding|GO:0035639,purine ribonucleoside triphosphate binding|GO:0005488,binding|GO:0016887,ATPase activity|GO:0043167,ion binding|GO:1901363,heterocyclic compound binding|GO:1901265,nucleoside phosphate binding|GO:0001882,nucleoside binding|GO:0043168,anion binding|GO:0042623,ATPase activity, coupled|GO:0008568,microtubule-severing ATPase activity|GO:0000166,nucleotide binding|GO:0001883,purine nucleoside binding|GO:0032549,ribonucleoside binding|GO:0017076,purine nucleotide binding|GO:0005524,ATP binding|GO:0016787,hydrolase activity|GO:0017111,nucleoside-triphosphatase activity|GO:0036094,small molecule binding|GO:0003824,catalytic activity|GO:0016818,hydrolase activity, acting on acid anhydrides, in phosphorus-containing anhydrides|GO:0030554,adenyl nucleotide binding|GO:0097367,carbohydrate derivative binding|GO:0097159,organic cyclic compound binding|GO:0016817,hydrolase activity, acting on acid anhydrides|GO:0016462,pyrophosphatase activity|GO:0032559,adenyl ribonucleotide binding|GO:0005515,protein binding|GO:0032555,purine ribonucleotide binding|GO:0032550,purine ribonucleoside binding|GO:0032553,ribonucleotide binding
TRIM71	GO:0080090,regulation of primary metabolic process|GO:0019222,regulation of metabolic process|GO:0000082,G1/S transition of mitotic cell cycle|GO:0007165,signal transduction|GO:0007166,cell surface receptor signaling pathway|GO:0007167,enzyme linked receptor protein signaling pathway|GO:0007169,transmembrane receptor protein tyrosine kinase signaling pathway|GO:1901360,organic cyclic compound metabolic process|GO:0051716,cellular response to stimulus|GO:0010605,negative regulation of macromolecule metabolic process|GO:0010608,posttranscriptional regulation of gene expression|GO:0043043,peptide biosynthetic process|GO:0040029,regulation of gene expression, epigenetic|GO:0010033,response to organic substance|GO:0032446,protein modification by small protein conjugation|GO:0016458,gene silencing|GO:0048518,positive regulation of biological process|GO:0048519,negative regulation of biological process|GO:0046483,heterocycle metabolic process|GO:0042127,regulation of cell proliferation|GO:0048729,tissue morphogenesis|GO:0044700,single organism signaling|GO:0060255,regulation of macromolecule metabolic process|GO:0061158,3'-UTR-mediated mRNA destabilization|GO:0060147,regulation of posttranscriptional gene silencing|GO:0042221,response to chemical|GO:0040033,negative regulation of translation, ncRNA-mediated|GO:0035278,miRNA mediated inhibition of translation|GO:0060148,positive regulation of posttranscriptional gene silencing|GO:0061157,mRNA destabilization|GO:0043604,amide biosynthetic process|GO:0014020,primary neural tube formation|GO:1901564,organonitrogen compound metabolic process|GO:1901566,organonitrogen compound biosynthetic process|GO:0044707,single-multicellular organism process|GO:0044249,cellular biosynthetic process|GO:0019538,protein metabolic process|GO:0016567,protein ubiquitination|GO:0051865,protein autoubiquitination|GO:0016441,posttranscriptional gene silencing|GO:2000637,positive regulation of gene silencing by miRNA|GO:0009892,negative regulation of metabolic process|GO:0034645,cellular macromolecule biosynthetic process|GO:0009890,negative regulation of biosynthetic process|GO:0010629,negative regulation of gene expression|GO:0006807,nitrogen compound metabolic process|GO:0034660,ncRNA metabolic process|GO:0050789,regulation of biological process|GO:0044267,cellular protein metabolic process|GO:0044260,cellular macromolecule metabolic process|GO:0044344,cellular response to fibroblast growth factor stimulus|GO:0065007,biological regulation|GO:0044699,single-organism process|GO:0001841,neural tube formation|GO:0001843,neural tube closure|GO:0065008,regulation of biological quality|GO:0048646,anatomical structure formation involved in morphogenesis|GO:0009889,regulation of biosynthetic process|GO:0009888,tissue development|GO:0050794,regulation of cellular process|GO:0060606,tube closure|GO:0043412,macromolecule modification|GO:0036211,protein modification process|GO:0008152,metabolic process|GO:0016070,RNA metabolic process|GO:0044271,cellular nitrogen compound biosynthetic process|GO:0050896,response to stimulus|GO:0010556,regulation of macromolecule biosynthetic process|GO:0071774,response to fibroblast growth factor|GO:0006518,peptide metabolic process|GO:0010558,negative regulation of macromolecule biosynthetic process|GO:0051171,regulation of nitrogen compound metabolic process|GO:0070647,protein modification by small protein conjugation or removal|GO:0035148,tube formation|GO:0009790,embryo development|GO:0034641,cellular nitrogen compound metabolic process|GO:0009792,embryo development ending in birth or egg hatching|GO:0023052,signaling|GO:0070887,cellular response to chemical stimulus|GO:0007154,cell communication|GO:0009653,anatomical structure morphogenesis|GO:0008543,fibroblast growth factor receptor signaling pathway|GO:0070848,response to growth factor|GO:0007049,cell cycle|GO:0009719,response to endogenous stimulus|GO:0006139,nucleobase-containing compound metabolic process|GO:0051248,negative regulation of protein metabolic process|GO:0043488,regulation of mRNA stability|GO:0043487,regulation of RNA stability|GO:0051246,regulation of protein metabolic process|GO:2000177,regulation of neural precursor cell proliferation|GO:0060562,epithelial tube morphogenesis|GO:0043009,chordate embryonic development|GO:0032502,developmental process|GO:0032501,multicellular organismal process|GO:0035239,tube morphogenesis|GO:0008283,cell proliferation|GO:0060429,epithelium development|GO:0006725,cellular aromatic compound metabolic process|GO:0034249,negative regulation of cellular amide metabolic process|GO:0034248,regulation of cellular amide metabolic process|GO:0045974,regulation of translation, ncRNA-mediated|GO:0090304,nucleic acid metabolic process|GO:0032269,negative regulation of cellular protein metabolic process|GO:0032268,regulation of cellular protein metabolic process|GO:0071363,cellular response to growth factor stimulus|GO:0043603,cellular amide metabolic process|GO:0050779,RNA destabilization|GO:0044772,mitotic cell cycle phase transition|GO:0009059,macromolecule biosynthetic process|GO:0043170,macromolecule metabolic process|GO:0017148,negative regulation of translation|GO:0048731,system development|GO:0072089,stem cell proliferation|GO:0071495,cellular response to endogenous stimulus|GO:0016331,morphogenesis of embryonic epithelium|GO:0031327,negative regulation of cellular biosynthetic process|GO:0031326,regulation of cellular biosynthetic process|GO:0031324,negative regulation of cellular metabolic process|GO:0031323,regulation of cellular metabolic process|GO:1903047,mitotic cell cycle process|GO:0044770,cell cycle phase transition|GO:0072175,epithelial tube formation|GO:0022402,cell cycle process|GO:0010467,gene expression|GO:0007275,multicellular organism development|GO:0002009,morphogenesis of an epithelium|GO:0010586,miRNA metabolic process|GO:2000112,regulation of cellular macromolecule biosynthetic process|GO:2000113,negative regulation of cellular macromolecule biosynthetic process|GO:0071704,organic substance metabolic process|GO:0071310,cellular response to organic substance|GO:0061351,neural precursor cell proliferation|GO:0010468,regulation of gene expression|GO:0048598,embryonic morphogenesis|GO:0009987,cellular process|GO:0021915,neural tube development|GO:0000278,mitotic cell cycle|GO:1901576,organic substance biosynthetic process|GO:0006464,cellular protein modification process|GO:0044767,single-organism developmental process|GO:0060966,regulation of gene silencing by RNA|GO:0009058,biosynthetic process|GO:0060964,regulation of gene silencing by miRNA|GO:0044763,single-organism cellular process|GO:0031047,gene silencing by RNA|GO:0051172,negative regulation of nitrogen compound metabolic process|GO:0044843,cell cycle G1/S phase transition|GO:0035295,tube development|GO:0060968,regulation of gene silencing|GO:0001838,embryonic epithelial tube formation|GO:0044238,primary metabolic process|GO:0007399,nervous system development|GO:0035194,posttranscriptional gene silencing by RNA|GO:0035195,gene silencing by miRNA|GO:0048856,anatomical structure development|GO:0044237,cellular metabolic process|GO:0006417,regulation of translation|GO:0006412,translation|GO:0048523,negative regulation of cellular process|GO:0048522,positive regulation of cellular process|GO:0071359,cellular response to dsRNA|GO:0071407,cellular response to organic cyclic compound|GO:0035196,production of miRNAs involved in gene silencing by miRNA|GO:1901699,cellular response to nitrogen compound|GO:0031050,dsRNA fragmentation|GO:0043331,response to dsRNA|GO:0014070,response to organic cyclic compound|GO:1901698,response to nitrogen compound|GO:0006396,RNA processing|GO:0070918,production of	GO:0005737,cytoplasm|GO:0035770,ribonucleoprotein granule|GO:0043232,intracellular non-membrane-bounded organelle|GO:0044464,cell part|GO:0000932,P-body|GO:0044444,cytoplasmic part|GO:0005623,cell|GO:0030529,intracellular ribonucleoprotein complex|GO:0043229,intracellular organelle|GO:0043228,non-membrane-bounded organelle|GO:0036464,cytoplasmic ribonucleoprotein granule|GO:0044424,intracellular part|GO:0032991,macromolecular complex|GO:0005622,intracellular|GO:0043226,organelle|GO:1990904,ribonucleoprotein complex	GO:0019787,ubiquitin-like protein transferase activity|GO:0043169,cation binding|GO:0035198,miRNA binding|GO:0045182,translation regulator activity|GO:0046914,transition metal ion binding|GO:0016874,ligase activity|GO:0030371,translation repressor activity|GO:0061630,ubiquitin protein ligase activity|GO:0008270,zinc ion binding|GO:0043167,ion binding|GO:0003824,catalytic activity|GO:0016740,transferase activity|GO:0003723,RNA binding|GO:0003676,nucleic acid binding|GO:0097159,organic cyclic compound binding|GO:0005488,binding|GO:0046872,metal ion binding|GO:0004842,ubiquitin-protein transferase activity|GO:1901363,heterocyclic compound binding|GO:0061659,ubiquitin-like protein ligase activity
NR6A1	GO:0080090,regulation of primary metabolic process|GO:0019222,regulation of metabolic process|GO:0031326,regulation of cellular biosynthetic process|GO:0071383,cellular response to steroid hormone stimulus|GO:0031323,regulation of cellular metabolic process|GO:0090304,nucleic acid metabolic process|GO:0044249,cellular biosynthetic process|GO:0034641,cellular nitrogen compound metabolic process|GO:0006807,nitrogen compound metabolic process|GO:0023052,signaling|GO:0007165,signal transduction|GO:0043401,steroid hormone mediated signaling pathway|GO:0042221,response to chemical|GO:0006725,cellular aromatic compound metabolic process|GO:1901576,organic substance biosynthetic process|GO:0070887,cellular response to chemical stimulus|GO:1901362,organic cyclic compound biosynthetic process|GO:0050789,regulation of biological process|GO:0044699,single-organism process|GO:0032774,RNA biosynthetic process|GO:0009755,hormone-mediated signaling pathway|GO:0006139,nucleobase-containing compound metabolic process|GO:0051716,cellular response to stimulus|GO:0044260,cellular macromolecule metabolic process|GO:0071704,organic substance metabolic process|GO:0033993,response to lipid|GO:1901360,organic cyclic compound metabolic process|GO:2000112,regulation of cellular macromolecule biosynthetic process|GO:0010467,gene expression|GO:0060255,regulation of macromolecule metabolic process|GO:0071310,cellular response to organic substance|GO:0014070,response to organic cyclic compound|GO:0065007,biological regulation|GO:0097659,nucleic acid-templated transcription|GO:0010468,regulation of gene expression|GO:0018130,heterocycle biosynthetic process|GO:0071495,cellular response to endogenous stimulus|GO:0009719,response to endogenous stimulus|GO:0044238,primary metabolic process|GO:0019219,regulation of nucleobase-containing compound metabolic process|GO:0071396,cellular response to lipid|GO:0009987,cellular process|GO:0009889,regulation of biosynthetic process|GO:1903506,regulation of nucleic acid-templated transcription|GO:0050794,regulation of cellular process|GO:0034645,cellular macromolecule biosynthetic process|GO:0032870,cellular response to hormone stimulus|GO:0009059,macromolecule biosynthetic process|GO:0044763,single-organism cellular process|GO:0051171,regulation of nitrogen compound metabolic process|GO:0008152,metabolic process|GO:2001141,regulation of RNA biosynthetic process|GO:0034654,nucleobase-containing compound biosynthetic process|GO:0007154,cell communication|GO:0010033,response to organic substance|GO:0046483,heterocycle metabolic process|GO:0016070,RNA metabolic process|GO:0044700,single organism signaling|GO:0044271,cellular nitrogen compound biosynthetic process|GO:0071407,cellular response to organic cyclic compound|GO:0050896,response to stimulus|GO:0051252,regulation of RNA metabolic process|GO:0009058,biosynthetic process|GO:0044237,cellular metabolic process|GO:0043170,macromolecule metabolic process|GO:0006355,regulation of transcription, DNA-templated|GO:0010556,regulation of macromolecule biosynthetic process|GO:0006351,transcription, DNA-templated|GO:0009725,response to hormone|GO:0019438,aromatic compound biosynthetic process|GO:0048545,response to steroid hormone	GO:0043231,intracellular membrane-bounded organelle|GO:0005634,nucleus|GO:0044464,cell part|GO:0005623,cell|GO:0005622,intracellular|GO:0043229,intracellular organelle|GO:0044424,intracellular part|GO:0043227,membrane-bounded organelle|GO:0043226,organelle	GO:0005488,binding|GO:0043565,sequence-specific DNA binding|GO:0038023,signaling receptor activity|GO:0003676,nucleic acid binding|GO:0046914,transition metal ion binding|GO:0043169,cation binding|GO:0003700,transcription factor activity, sequence-specific DNA binding|GO:0097159,organic cyclic compound binding|GO:0043167,ion binding|GO:0001071,nucleic acid binding transcription factor activity|GO:0003707,steroid hormone receptor activity|GO:0060089,molecular transducer activity|GO:0004872,receptor activity|GO:0004871,signal transducer activity|GO:0003677,DNA binding|GO:0046872,metal ion binding|GO:1901363,heterocyclic compound binding|GO:0008270,zinc ion binding
THRSP	GO:0032774,RNA biosynthetic process|GO:0080090,regulation of primary metabolic process|GO:0019222,regulation of metabolic process|GO:0031326,regulation of cellular biosynthetic process|GO:0090207,regulation of triglyceride metabolic process|GO:0006638,neutral lipid metabolic process|GO:0031323,regulation of cellular metabolic process|GO:0006139,nucleobase-containing compound metabolic process|GO:0090304,nucleic acid metabolic process|GO:0044249,cellular biosynthetic process|GO:0034641,cellular nitrogen compound metabolic process|GO:0006807,nitrogen compound metabolic process|GO:0034645,cellular macromolecule biosynthetic process|GO:0046890,regulation of lipid biosynthetic process|GO:1901576,organic substance biosynthetic process|GO:0043170,macromolecule metabolic process|GO:0044255,cellular lipid metabolic process|GO:0050789,regulation of biological process|GO:0044699,single-organism process|GO:0010866,regulation of triglyceride biosynthetic process|GO:0045017,glycerolipid biosynthetic process|GO:0044710,single-organism metabolic process|GO:0044711,single-organism biosynthetic process|GO:0044260,cellular macromolecule metabolic process|GO:0071704,organic substance metabolic process|GO:1901362,organic cyclic compound biosynthetic process|GO:1901360,organic cyclic compound metabolic process|GO:0006641,triglyceride metabolic process|GO:2000112,regulation of cellular macromolecule biosynthetic process|GO:0060255,regulation of macromolecule metabolic process|GO:0010467,gene expression|GO:0010556,regulation of macromolecule biosynthetic process|GO:0065007,biological regulation|GO:0097659,nucleic acid-templated transcription|GO:0046483,heterocycle metabolic process|GO:0010468,regulation of gene expression|GO:0046460,neutral lipid biosynthetic process|GO:0018130,heterocycle biosynthetic process|GO:0006629,lipid metabolic process|GO:0019216,regulation of lipid metabolic process|GO:0019219,regulation of nucleobase-containing compound metabolic process|GO:0006725,cellular aromatic compound metabolic process|GO:0006639,acylglycerol metabolic process|GO:0009987,cellular process|GO:0009889,regulation of biosynthetic process|GO:1903506,regulation of nucleic acid-templated transcription|GO:0050794,regulation of cellular process|GO:0009058,biosynthetic process|GO:0009059,macromolecule biosynthetic process|GO:0044763,single-organism cellular process|GO:0051171,regulation of nitrogen compound metabolic process|GO:0008152,metabolic process|GO:2001141,regulation of RNA biosynthetic process|GO:0034654,nucleobase-containing compound biosynthetic process|GO:0046486,glycerolipid metabolic process|GO:0008610,lipid biosynthetic process|GO:0016070,RNA metabolic process|GO:0044238,primary metabolic process|GO:0044271,cellular nitrogen compound biosynthetic process|GO:0051252,regulation of RNA metabolic process|GO:0044237,cellular metabolic process|GO:0046463,acylglycerol biosynthetic process|GO:0006355,regulation of transcription, DNA-templated|GO:0019432,triglyceride biosynthetic process|GO:0006351,transcription, DNA-templated|GO:0019438,aromatic compound biosynthetic process|GO:0006820,anion transport|GO:0051641,cellular localization|GO:1990542,mitochondrial transmembrane transport|GO:0033036,macromolecule localization|GO:0015718,monocarboxylic acid transport|GO:0015849,organic acid transport|GO:0015711,organic anion transport|GO:0006839,mitochondrial transport|GO:0010876,lipid localization|GO:1902001,fatty acid transmembrane transport|GO:0015909,long-chain fatty acid transport|GO:0015908,fatty acid transport|GO:0006811,ion transport|GO:0006810,transport|GO:0051234,establishment of localization|GO:0046907,intracellular transport|GO:0032365,intracellular lipid transport|GO:0044765,single-organism transport|GO:0006869,lipid transport|GO:1903825,organic acid transmembrane transport|GO:0055085,transmembrane transport|GO:1905039,carboxylic acid transmembrane transport|GO:0071702,organic substance transport|GO:0006853,carnitine shuttle|GO:0034220,ion transmembrane transport|GO:0051649,establishment of localization in cell|GO:0051179,localization|GO:1902578,single-organism localization|GO:0046942,carboxylic acid transport|GO:0098656,anion transmembrane transport|GO:1902582,single-organism intracellular transport	GO:0005737,cytoplasm|GO:0044446,intracellular organelle part|GO:0005829,cytosol|GO:0043231,intracellular membrane-bounded organelle|GO:0031981,nuclear lumen|GO:0043233,organelle lumen|GO:0005634,nucleus|GO:0044464,cell part|GO:0044444,cytoplasmic part|GO:0031974,membrane-enclosed lumen|GO:0005622,intracellular|GO:0005654,nucleoplasm|GO:0070013,intracellular organelle lumen|GO:0043229,intracellular organelle|GO:0044428,nuclear part|GO:0005623,cell|GO:0044424,intracellular part|GO:0043227,membrane-bounded organelle|GO:0043226,organelle|GO:0044422,organelle part	GO:0005488,binding|GO:0005515,protein binding|GO:0046983,protein dimerization activity|GO:0042802,identical protein binding|GO:0042803,protein homodimerization activity

USP44	GO:0080090,regulation of primary metabolic process|GO:0032434,regulation of proteasomal ubiquitin-dependent protein catabolic process|GO:0032435,negative regulation of proteasomal ubiquitin-dependent protein catabolic process|GO:0044257,cellular protein catabolic process|GO:0000280,nuclear division|GO:0010965,regulation of mitotic sister chromatid separation|GO:0071840,cellular component organization or biogenesis|GO:0044712,single-organism catabolic process|GO:0044710,single-organism metabolic process|GO:0010605,negative regulation of macromolecule metabolic process|GO:0019222,regulation of metabolic process|GO:0070647,protein modification by small protein conjugation or removal|GO:0070646,protein modification by small protein removal|GO:0032446,protein modification by small protein conjugation|GO:1901799,negative regulation of proteasomal protein catabolic process|GO:0044092,negative regulation of molecular function|GO:0048519,negative regulation of biological process|GO:0007088,regulation of mitotic nuclear division|GO:0060255,regulation of macromolecule metabolic process|GO:0030162,regulation of proteolysis|GO:0030163,protein catabolic process|GO:1902099,regulation of metaphase/anaphase transition of cell cycle|GO:0019538,protein metabolic process|GO:0010639,negative regulation of organelle organization|GO:0051352,negative regulation of ligase activity|GO:0051783,regulation of nuclear division|GO:0051782,negative regulation of cell division|GO:0051784,negative regulation of nuclear division|GO:0044784,metaphase/anaphase transition of cell cycle|GO:0045841,negative regulation of mitotic metaphase/anaphase transition|GO:0009894,regulation of catabolic process|GO:0009895,negative regulation of catabolic process|GO:0009892,negative regulation of metabolic process|GO:0090266,regulation of mitotic cell cycle spindle assembly checkpoint|GO:2000816,negative regulation of mitotic sister chromatid separation|GO:0007067,mitotic nuclear division|GO:0030071,regulation of mitotic metaphase/anaphase transition|GO:0031577,spindle checkpoint|GO:0007346,regulation of mitotic cell cycle|GO:0051340,regulation of ligase activity|GO:0044260,cellular macromolecule metabolic process|GO:0016567,protein ubiquitination|GO:0051348,negative regulation of transferase activity|GO:0016043,cellular component organization|GO:0065007,biological regulation|GO:0044699,single-organism process|GO:0044267,cellular protein metabolic process|GO:0065009,regulation of molecular function|GO:0016579,protein deubiquitination|GO:0071174,mitotic spindle checkpoint|GO:0071173,spindle assembly checkpoint|GO:0050790,regulation of catalytic activity|GO:0050794,regulation of cellular process|GO:1901987,regulation of cell cycle phase transition|GO:0043412,macromolecule modification|GO:0036211,protein modification process|GO:0008152,metabolic process|GO:0045786,negative regulation of cell cycle|GO:1901988,negative regulation of cell cycle phase transition|GO:0051444,negative regulation of ubiquitin-protein transferase activity|GO:0051603,proteolysis involved in cellular protein catabolic process|GO:0031400,negative regulation of protein modification process|GO:0010498,proteasomal protein catabolic process|GO:0051338,regulation of transferase activity|GO:0006511,ubiquitin-dependent protein catabolic process|GO:1901976,regulation of cell cycle checkpoint|GO:2001251,negative regulation of chromosome organization|GO:0033044,regulation of chromosome organization|GO:0033045,regulation of sister chromatid segregation|GO:0033046,negative regulation of sister chromatid segregation|GO:0033047,regulation of mitotic sister chromatid segregation|GO:0033043,regulation of organelle organization|GO:0010564,regulation of cell cycle process|GO:0033048,negative regulation of mitotic sister chromatid segregation|GO:0051129,negative regulation of cellular component organization|GO:0051128,regulation of cellular component organization|GO:0044248,cellular catabolic process|GO:0042176,regulation of protein catabolic process|GO:0042177,negative regulation of protein catabolic process|GO:1901991,negative regulation of mitotic cell cycle phase transition|GO:1901990,regulation of mitotic cell cycle phase transition|GO:0007059,chromosome segregation|GO:0043086,negative regulation of catalytic activity|GO:0007049,cell cycle|GO:0051248,negative regulation of protein metabolic process|GO:0043161,proteasome-mediated ubiquitin-dependent protein catabolic process|GO:0051246,regulation of protein metabolic process|GO:1903320,regulation of protein modification by small protein conjugation or removal|GO:1903321,negative regulation of protein modification by small protein conjugation or removal|GO:0031399,regulation of protein modification process|GO:0031396,regulation of protein ubiquitination|GO:0031397,negative regulation of protein ubiquitination|GO:0060564,negative regulation of APC-Cdc20 complex activity|GO:0090231,regulation of spindle checkpoint|GO:0031330,negative regulation of cellular catabolic process|GO:1903050,regulation of proteolysis involved in cellular protein catabolic process|GO:1903051,negative regulation of proteolysis involved in cellular protein catabolic process|GO:1903504,regulation of mitotic spindle checkpoint|GO:0031145,anaphase-promoting complex-dependent catabolic process|GO:0045839,negative regulation of mitotic nuclear division|GO:0051985,negative regulation of chromosome segregation|GO:0006508,proteolysis|GO:0051983,regulation of chromosome segregation|GO:0032269,negative regulation of cellular protein metabolic process|GO:0032268,regulation of cellular protein metabolic process|GO:0000819,sister chromatid segregation|GO:0098813,nuclear chromosome segregation|GO:0044772,mitotic cell cycle phase transition|GO:0043170,macromolecule metabolic process|GO:0022402,cell cycle process|GO:0045861,negative regulation of proteolysis|GO:0031329,regulation of cellular catabolic process|GO:0031324,negative regulation of cellular metabolic process|GO:0031323,regulation of cellular metabolic process|GO:1903047,mitotic cell cycle process|GO:0044770,cell cycle phase transition|GO:0010948,negative regulation of cell cycle process|GO:0051439,regulation of ubiquitin-protein ligase activity involved in mitotic cell cycle|GO:0051438,regulation of ubiquitin-protein transferase activity|GO:0051306,mitotic sister chromatid separation|GO:0043632,modification-dependent macromolecule catabolic process|GO:0051436,negative regulation of ubiquitin-protein ligase activity involved in mitotic cell cycle|GO:0051302,regulation of cell division|GO:0051301,cell division|GO:0051304,chromosome separation|GO:0050789,regulation of biological process|GO:0071704,organic substance metabolic process|GO:0045930,negative regulation of mitotic cell cycle|GO:0009987,cellular process|GO:0000278,mitotic cell cycle|GO:1903362,regulation of cellular protein catabolic process|GO:1903363,negative regulation of cellular protein catabolic process|GO:0061136,regulation of proteasomal protein catabolic process|GO:1901575,organic substance catabolic process|GO:0006464,cellular protein modification process|GO:0044265,cellular macromolecule catabolic process|GO:0044763,single-organism cellular process|GO:0019941,modification-dependent protein catabolic process|GO:0009056,catabolic process|GO:0009057,macromolecule catabolic process|GO:0006996,organelle organization|GO:0044238,primary metabolic process|GO:0000070,mitotic sister chromatid segregation|GO:0051276,chromosome organization|GO:0000075,cell cycle checkpoint|GO:0051726,regulation of cell cycle|GO:0044237,cellular metabolic process|GO:1902589,single-organism organelle organization|GO:0048285,organelle fission|GO:1902100,negative regulation of metaphase/anaphase transition of cell cycle|GO:0007094,mitotic spindle assembly checkpoint|GO:0007091,metaphase/anaphase transition of mitotic cell cycle|GO:0048523,negative regulation of cellular process|GO:0007093,mitotic cell cycle checkpoint|GO:1901564,organonitrogen compound metabolic process|GO:1901565,organonitrogen compound catabolic process|GO:0006807,nitrogen compound metabolic process|GO:1904666,regulation of ubiquitin protein ligase activity|GO:1904667,negative regulation of ubiquitin protein ligase activity	GO:0031974,membrane-enclosed lumen|GO:0043229,intracellular organelle|GO:0043228,non-membrane-bounded organelle|GO:0043227,membrane-bounded organelle|GO:0043226,organelle|GO:0005856,cytoskeleton|GO:0044446,intracellular organelle part|GO:0031981,nuclear lumen|GO:0005730,nucleolus|GO:0005634,nucleus|GO:0015630,microtubule cytoskeleton|GO:0043231,intracellular membrane-bounded organelle|GO:0043232,intracellular non-membrane-bounded organelle|GO:0043233,organelle lumen|GO:0044464,cell part|GO:0005623,cell|GO:0005622,intracellular|GO:0070013,intracellular organelle lumen|GO:0044428,nuclear part|GO:0044424,intracellular part|GO:0044422,organelle part|GO:0005654,nucleoplasm	GO:0016787,hydrolase activity|GO:0043167,ion binding|GO:0004175,endopeptidase activity|GO:0019783,ubiquitin-like protein-specific protease activity|GO:0046914,transition metal ion binding|GO:0043169,cation binding|GO:0004197,cysteine-type endopeptidase activity|GO:0008234,cysteine-type peptidase activity|GO:0003824,catalytic activity|GO:0036459,thiol-dependent ubiquitinyl hydrolase activity|GO:0070011,peptidase activity, acting on L-amino acid peptides|GO:0008270,zinc ion binding|GO:0005515,protein binding|GO:0008233,peptidase activity|GO:0046872,metal ion binding|GO:0004843,thiol-dependent ubiquitin-specific protease activity|GO:0005488,binding|GO:0101005,ubiquitinyl hydrolase activity
FAM222B		GO:0044446,intracellular organelle part|GO:0043231,intracellular membrane-bounded organelle|GO:0031981,nuclear lumen|GO:0043233,organelle lumen|GO:0005634,nucleus|GO:0044464,cell part|GO:0031974,membrane-enclosed lumen|GO:0005622,intracellular|GO:0005654,nucleoplasm|GO:0070013,intracellular organelle lumen|GO:0043229,intracellular organelle|GO:0044428,nuclear part|GO:0005623,cell|GO:0044424,intracellular part|GO:0043227,membrane-bounded organelle|GO:0043226,organelle|GO:0044422,organelle part	
IGDCC3	GO:0032501,multicellular organismal process|GO:0050885,neuromuscular process controlling balance|GO:0044707,single-multicellular organism process|GO:0050877,neurological system process|GO:0050905,neuromuscular process|GO:0044699,single-organism process|GO:0003008,system process	GO:0031226,intrinsic component of plasma membrane|GO:0016021,integral component of membrane|GO:0016020,membrane|GO:0044464,cell part|GO:0005623,cell|GO:0071944,cell periphery|GO:0005887,integral component of plasma membrane|GO:0005886,plasma membrane|GO:0044425,membrane part|GO:0044459,plasma membrane part|GO:0031224,intrinsic component of membrane	
CDR1as			
KCNJ6	GO:0051049,regulation of transport|GO:0023052,signaling|GO:0034765,regulation of ion transmembrane transport|GO:0010107,potassium ion import|GO:0050789,regulation of biological process|GO:0043269,regulation of ion transport|GO:0051234,establishment of localization|GO:0071804,cellular potassium ion transport|GO:0071805,potassium ion transmembrane transport|GO:0051179,localization|GO:0065007,biological regulation|GO:0034762,regulation of transmembrane transport|GO:0098660,inorganic ion transmembrane transport|GO:0015672,monovalent inorganic cation transport|GO:0098662,inorganic cation transmembrane transport|GO:0006810,transport|GO:0006813,potassium ion transport|GO:0006812,cation transport|GO:0006811,ion transport|GO:0009987,cellular process|GO:0050794,regulation of cellular process|GO:0034220,ion transmembrane transport|GO:0044765,single-organism transport|GO:0044763,single-organism cellular process|GO:0007268,chemical synaptic transmission|GO:0007267,cell-cell signaling|GO:0007154,cell communication|GO:0032879,regulation of localization|GO:0055085,transmembrane transport|GO:1902578,single-organism localization|GO:0044700,single organism signaling|GO:0044699,single-organism process|GO:0030001,metal ion transport|GO:0098655,cation transmembrane transport	GO:0043229,intracellular organelle|GO:0034705,potassium channel complex|GO:0034702,ion channel complex|GO:0034703,cation channel complex|GO:0031226,intrinsic component of plasma membrane|GO:0043226,organelle|GO:0031224,intrinsic component of membrane|GO:0005737,cytoplasm|GO:0043227,membrane-bounded organelle|GO:0016021,integral component of membrane|GO:0016020,membrane|GO:0044459,plasma membrane part|GO:0005794,Golgi apparatus|GO:1902495,transmembrane transporter complex|GO:1990351,transporter complex|GO:0008076,voltage-gated potassium channel complex|GO:0012505,endomembrane system|GO:0005887,integral component of plasma membrane|GO:0005886,plasma membrane|GO:0043234,protein complex|GO:0032991,macromolecular complex|GO:0043231,intracellular membrane-bounded organelle|GO:0044464,cell part|GO:0005623,cell|GO:0005622,intracellular|GO:0098797,plasma membrane protein complex|GO:0098796,membrane protein complex|GO:0044444,cytoplasmic part|GO:0071944,cell periphery|GO:0044424,intracellular part|GO:0044425,membrane part	GO:0005267,potassium channel activity|GO:0005261,cation channel activity|GO:0015467,G-protein activated inward rectifier potassium channel activity|GO:0005488,binding|GO:0015276,ligand-gated ion channel activity|GO:0022857,transmembrane transporter activity|GO:0022843,voltage-gated cation channel activity|GO:0022803,passive transmembrane transporter activity|GO:0046873,metal ion transmembrane transporter activity|GO:0005515,protein binding|GO:0005215,transporter activity|GO:0005216,ion channel activity|GO:0008324,cation transmembrane transporter activity|GO:0022891,substrate-specific transmembrane transporter activity|GO:0022890,inorganic cation transmembrane transporter activity|GO:0022892,substrate-specific transporter activity|GO:0005244,voltage-gated ion channel activity|GO:0015075,ion transmembrane transporter activity|GO:0022832,voltage-gated channel activity|GO:0015077,monovalent inorganic cation transmembrane transporter activity|GO:0015267,channel activity|GO:0022836,gated channel activity|GO:0005249,voltage-gated potassium channel activity|GO:0022834,ligand-gated channel activity|GO:0022838,substrate-specific channel activity|GO:0015079,potassium ion transmembrane transporter activity|GO:0005242,inward rectifier potassium channel activity
GIPC3		GO:0043227,membrane-bounded organelle|GO:0031982,vesicle|GO:0043226,organelle	
S100B	GO:0048584,positive regulation of response to stimulus|GO:0048583,regulation of response to stimulus|GO:0007613,memory|GO:0007610,behavior|GO:0007611,learning or memory|GO:0007165,signal transduction|GO:0032989,cellular component morphogenesis|GO:0071840,cellular component organization or biogenesis|GO:0007517,muscle organ development|GO:0009966,regulation of signal transduction|GO:0009967,positive regulation of signal transduction|GO:0007519,skeletal muscle tissue development|GO:0048513,animal organ development|GO:0048518,positive regulation of biological process|GO:0048519,negative regulation of biological process|GO:0043122,regulation of I-kappaB kinase/NF-kappaB signaling|GO:0043123,positive regulation of I-kappaB kinase/NF-kappaB signaling|GO:0042127,regulation of cell proliferation|GO:0016202,regulation of striated muscle tissue development|GO:0048468,cell development|GO:0048634,regulation of muscle organ development|GO:0042221,response to chemical|GO:0008360,regulation of cell shape|GO:0051597,response to methylmercury|GO:0051128,regulation of cellular component organization|GO:0010033,response to organic substance|GO:0050794,regulation of cellular process|GO:0003008,system process|GO:0044700,single organism signaling|GO:0044707,single-multicellular organism process|GO:0044708,single-organism behavior|GO:0002376,immune system process|GO:0033554,cellular response to stress|GO:0022604,regulation of cell morphogenesis|GO:0016265,obsolete death|GO:0045843,negative regulation of striated muscle tissue development|GO:0051716,cellular response to stimulus|GO:0070887,cellular response to chemical stimulus|GO:0022603,regulation of anatomical structure morphogenesis|GO:0048167,regulation of synaptic plasticity|GO:0031175,neuron projection development|GO:2001014,regulation of skeletal muscle cell differentiation|GO:2001015,negative regulation of skeletal muscle cell differentiation|GO:0035556,intracellular signal transduction|GO:0042981,regulation of apoptotic process|GO:0048168,regulation of neuronal synaptic plasticity|GO:0000904,cell morphogenesis involved in differentiation|GO:0000902,cell morphogenesis|GO:0016043,cellular component organization|GO:0071453,cellular response to oxygen levels|GO:0065007,biological regulation|GO:0014070,response to organic cyclic compound|GO:0071456,cellular response to hypoxia|GO:0035914,skeletal muscle cell differentiation|GO:0048641,regulation of skeletal muscle tissue development|GO:0048642,negative regulation of skeletal muscle tissue development|GO:0065008,regulation of biological quality|GO:1901861,regulation of muscle tissue development|GO:1901862,negative regulation of muscle tissue development|GO:0061564,axon development|GO:0050793,regulation of developmental process|GO:0042063,gliogenesis|GO:0048708,astrocyte differentiation|GO:0006952,defense response|GO:0012501,programmed cell death|GO:0006950,response to stress|GO:0051239,regulation of multicellular organismal process|GO:0006955,immune response|GO:1902533,positive regulation of intracellular signal transduction|GO:1902531,regulation of intracellular signal transduction|GO:0001666,response to hypoxia|GO:0036293,response to decreased oxygen levels|GO:0050896,response to stimulus|GO:0050890,cognition|GO:0036294,cellular response to decreased oxygen levels|GO:0048812,neuron projection morphogenesis|GO:0048869,cellular developmental process|GO:0050803,regulation of synapse structure or activity|GO:0030154,cell differentiation|GO:0048635,negative regulation of muscle organ development|GO:0050806,positive regulation of synaptic transmission|GO:0050804,modulation of synaptic transmission|GO:0023056,positive regulation of signaling|GO:0023052,signaling|GO:0010001,glial cell differentiation|GO:0023051,regulation of signaling|GO:0061061,muscle structure development|GO:0010647,positive regulation of cell communication|GO:0010646,regulation of cell communication|GO:0044699,single-organism process|GO:0007417,central nervous system development|GO:0009719,response to endogenous stimulus|GO:0009653,anatomical structure morphogenesis|GO:0051241,negative regulation of multicellular organismal process|GO:0008284,positive regulation of cell proliferation|GO:0032501,multicellular organismal process|GO:0008283,cell proliferation|GO:0050877,neurological system process|GO:0009987,cellular process|GO:0045596,negative regulation of cell differentiation|GO:0045595,regulation of cell differentiation|GO:0007409,axonogenesis|GO:0032990,cell part morphogenesis|GO:0051093,negative regulation of developmental process|GO:0060291,long-term synaptic potentiation|GO:0009725,response to hormone|GO:0007249,I-kappaB kinase/NF-kappaB signaling|GO:0048731,system development|GO:0048545,response to steroid hormone|GO:0032502,developmental process|GO:0051384,response to glucocorticoid|GO:0030030,cell projection organization|GO:1990267,response to transition metal nanoparticle|GO:0014706,striated muscle tissue development|GO:0010942,positive regulation of cell death|GO:0008219,cell death|GO:0010941,regulation of cell death|GO:0007275,multicellular organism development|GO:0009888,tissue development|GO:0033993,response to lipid|GO:0050789,regulation of biological process|GO:0043065,positive regulation of apoptotic process|GO:0043067,regulation of programmed cell death|GO:0031960,response to corticosteroid|GO:0043068,positive regulation of programmed cell death|GO:0048666,neuron development|GO:0048667,cell morphogenesis involved in neuron differentiation|GO:0030182,neuron differentiation|GO:0045087,innate immune response|GO:0006915,apoptotic process|GO:0044767,single-organism developmental process|GO:0044763,single-organism cellular process|GO:0007268,chemical synaptic transmission|GO:0007267,cell-cell signaling|GO:0007154,cell communication|GO:0022008,neurogenesis|GO:0070482,response to oxygen levels|GO:0009628,response to abiotic stimulus|GO:0048699,generation of neurons|GO:0048858,cell projection morphogenesis|GO:0007399,nervous system development|GO:0048856,anatomical structure development|GO:2000026,regulation of multicellular organismal development|GO:0060537,muscle tissue development|GO:0060538,skeletal muscle organ development|GO:0048523,negative regulation of cellular process|GO:0048522,positive regulation of cellular process|GO:0008366,axon ensheathment|GO:0098916,anterograde trans-synaptic signaling|GO:0042552,myelination|GO:0031644,regulation of neurological system process|GO:0031646,positive regulation of neurological system process|GO:0031641,regulation of myelination|GO:0031643,positive regulation of myelination|GO:0051962,positive regulation of nervous system development|GO:0051960,regulation of nervous system development|GO:0099536,synaptic signaling|GO:0099537,trans-synaptic signaling|GO:0044057,regulation of system process|GO:0051240,positive regulation of multicellular organismal	GO:0043229,intracellular organelle|GO:0043227,membrane-bounded organelle|GO:0043226,organelle|GO:0005737,cytoplasm|GO:0001726,ruffle|GO:0005615,extracellular space|GO:0005634,nucleus|GO:0097458,neuron part|GO:0048471,perinuclear region of cytoplasm|GO:0043025,neuronal cell body|GO:0044297,cell body|GO:0031252,cell leading edge|GO:0036477,somatodendritic compartment|GO:0042995,cell projection|GO:0043231,intracellular membrane-bounded organelle|GO:0044464,cell part|GO:0005623,cell|GO:0005622,intracellular|GO:0044444,cytoplasmic part|GO:0005576,extracellular region|GO:0044424,intracellular part|GO:0044421,extracellular region part	GO:0005488,binding|GO:0043169,cation binding|GO:0046914,transition metal ion binding|GO:0044548,S100 protein binding|GO:0008092,cytoskeletal protein binding|GO:0048156,tau protein binding|GO:0043167,ion binding|GO:0048306,calcium-dependent protein binding|GO:0042803,protein homodimerization activity|GO:0050786,RAGE receptor binding|GO:0008270,zinc ion binding|GO:0005515,protein binding|GO:0046983,protein dimerization activity|GO:0046872,metal ion binding|GO:0005102,receptor binding|GO:0042802,identical protein binding|GO:0005509,calcium ion binding
GSG1L		GO:0016021,integral component of membrane|GO:0016020,membrane|GO:0044464,cell part|GO:0005623,cell|GO:0071944,cell periphery|GO:0030054,cell junction|GO:0005886,plasma membrane|GO:0044425,membrane part|GO:0032279,asymmetric synapse|GO:0031224,intrinsic component of membrane|GO:0045202,synapse|GO:0099572,postsynaptic specialization|GO:0043229,intracellular organelle|GO:0043228,non-membrane-bounded organelle|GO:0098984,neuron to neuron synapse|GO:0043226,organelle|GO:0044456,synapse part|GO:0098794,postsynapse|GO:0014069,postsynaptic density|GO:0043232,intracellular non-membrane-bounded organelle|GO:0005622,intracellular|GO:0044424,intracellular part	GO:0005488,binding|GO:0005515,protein binding
BSPH1	GO:0030154,cell differentiation|GO:0048468,cell development|GO:0048469,cell maturation|GO:0007276,gamete generation|GO:0021700,developmental maturation|GO:0044699,single-organism process|GO:0000003,reproduction|GO:0048869,cellular developmental process|GO:0048240,sperm capacitation|GO:0048515,spermatid differentiation|GO:0032502,developmental process|GO:0032501,multicellular organismal process|GO:0048609,multicellular organismal reproductive process|GO:0032504,multicellular organism reproduction|GO:0009987,cellular process|GO:0019953,sexual reproduction|GO:0044767,single-organism developmental process|GO:0003006,developmental process involved in reproduction|GO:0007283,spermatogenesis|GO:0044763,single-organism cellular process|GO:0007338,single fertilization|GO:0022412,cellular process involved in reproduction in multicellular organism|GO:0048232,male gamete generation|GO:0051704,multi-organism process|GO:0007281,germ cell development|GO:0044703,multi-organism reproductive process|GO:0044702,single organism reproductive process|GO:0022414,reproductive process|GO:0048856,anatomical structure development|GO:0009566,fertilization|GO:0007286,spermatid development	GO:0009986,cell surface|GO:0005576,extracellular region|GO:0044464,cell part|GO:0005623,cell	GO:1901681,sulfur compound binding|GO:0043168,anion binding|GO:0097367,carbohydrate derivative binding|GO:0043167,ion binding|GO:0005539,glycosaminoglycan binding|GO:0005488,binding|GO:0008201,heparin binding
UBE2L6	GO:0019221,cytokine-mediated signaling pathway|GO:0007165,signal transduction|GO:0007166,cell surface receptor signaling pathway|GO:1901362,organic cyclic compound biosynthetic process|GO:1901360,organic cyclic compound metabolic process|GO:0032020,ISG15-protein conjugation|GO:0044710,single-organism metabolic process|GO:0044711,single-organism biosynthetic process|GO:0006301,postreplication repair|GO:0070647,protein modification by small protein conjugation or removal|GO:0032446,protein modification by small protein conjugation|GO:0048519,negative regulation of biological process|GO:0006281,DNA repair|GO:0030163,protein catabolic process|GO:0010033,response to organic substance|GO:0046483,heterocycle metabolic process|GO:0044700,single organism signaling|GO:0044707,single-multicellular organism process|GO:0019538,protein metabolic process|GO:0019985,translesion synthesis|GO:0002376,immune system process|GO:0016567,protein ubiquitination|GO:0033554,cellular response to stress|GO:0019438,aromatic compound biosynthetic process|GO:0034645,cellular macromolecule biosynthetic process|GO:0006807,nitrogen compound metabolic process|GO:0050789,regulation of biological process|GO:1901576,organic substance biosynthetic process|GO:1901575,organic substance catabolic process|GO:0044265,cellular macromolecule catabolic process|GO:0044260,cellular macromolecule metabolic process|GO:0065007,biological regulation|GO:0018130,heterocycle biosynthetic process|GO:0034097,response to cytokine|GO:0051716,cellular response to stimulus|GO:0050794,regulation of cellular process|GO:0006952,defense response|GO:0006950,response to stress|GO:0032480,negative regulation of type I interferon production|GO:0008152,metabolic process|GO:0006955,immune response|GO:0034654,nucleobase-containing compound biosynthetic process|GO:0051603,proteolysis involved in cellular protein catabolic process|GO:0044271,cellular nitrogen compound biosynthetic process|GO:0071345,cellular response to cytokine stimulus|GO:0050896,response to stimulus|GO:0043412,macromolecule modification|GO:0036211,protein modification process|GO:0001818,negative regulation of cytokine production|GO:0051239,regulation of multicellular organismal process|GO:0044248,cellular catabolic process|GO:0044249,cellular biosynthetic process|GO:0034641,cellular nitrogen compound metabolic process|GO:0023052,signaling|GO:0070887,cellular response to chemical stimulus|GO:0007154,cell communication|GO:0044699,single-organism process|GO:0006139,nucleobase-containing compound metabolic process|GO:0051241,negative regulation of multicellular organismal process|GO:0006508,proteolysis|GO:0032501,multicellular organismal process|GO:0009987,cellular process|GO:0006725,cellular aromatic compound metabolic process|GO:0006974,cellular response to DNA damage stimulus|GO:0044257,cellular protein catabolic process|GO:0043170,macromolecule metabolic process|GO:0001816,cytokine production|GO:0001817,regulation of cytokine production|GO:0032479,regulation of type I interferon production|GO:0071897,DNA biosynthetic process|GO:0032606,type I interferon production|GO:0090304,nucleic acid metabolic process|GO:0043632,modification-dependent macromolecule catabolic process|GO:0071704,organic substance metabolic process|GO:0071310,cellular response to organic substance|GO:0044267,cellular protein metabolic process|GO:0019941,modification-dependent protein catabolic process|GO:0045087,innate immune response|GO:0006464,cellular protein modification process|GO:0009058,biosynthetic process|GO:0009059,macromolecule biosynthetic process|GO:0044763,single-organism cellular process|GO:0042221,response to chemical|GO:0009056,catabolic process|GO:0009057,macromolecule catabolic process|GO:0044238,primary metabolic process|GO:0044237,cellular metabolic process|GO:0006259,DNA metabolic process|GO:1901564,organonitrogen compound metabolic process|GO:0000731,DNA synthesis involved in DNA repair|GO:1901565,organonitrogen compound catabolic process	GO:0005737,cytoplasm|GO:0044446,intracellular organelle part|GO:0005829,cytosol|GO:0043231,intracellular membrane-bounded organelle|GO:0031981,nuclear lumen|GO:0043233,organelle lumen|GO:0005634,nucleus|GO:0044464,cell part|GO:0044444,cytoplasmic part|GO:0031974,membrane-enclosed lumen|GO:0005622,intracellular|GO:0005654,nucleoplasm|GO:0070013,intracellular organelle lumen|GO:0043229,intracellular organelle|GO:0044428,nuclear part|GO:0005623,cell|GO:0044424,intracellular part|GO:0043227,membrane-bounded organelle|GO:0043226,organelle|GO:0044422,organelle part	GO:0019787,ubiquitin-like protein transferase activity|GO:0004842,ubiquitin-protein transferase activity|GO:0042296,ISG15 transferase activity|GO:0003824,catalytic activity|GO:0016740,transferase activity|GO:0005488,binding|GO:0005515,protein binding|GO:0016874,ligase activity|GO:0032182,ubiquitin-like protein binding|GO:0043130,ubiquitin binding
FBXL12	GO:0044248,cellular catabolic process|GO:0043632,modification-dependent macromolecule catabolic process|GO:0044267,cellular protein metabolic process|GO:1901575,organic substance catabolic process|GO:0044265,cellular macromolecule catabolic process|GO:0044260,cellular macromolecule metabolic process|GO:0071704,organic substance metabolic process|GO:0043161,proteasome-mediated ubiquitin-dependent protein catabolic process|GO:0070647,protein modification by small protein conjugation or removal|GO:0032446,protein modification by small protein conjugation|GO:0006508,proteolysis|GO:0044238,primary metabolic process|GO:0009987,cellular process|GO:0019941,modification-dependent protein catabolic process|GO:0031146,SCF-dependent proteasomal ubiquitin-dependent protein catabolic process|GO:0006464,cellular protein modification process|GO:0043412,macromolecule modification|GO:0036211,protein modification process|GO:0030163,protein catabolic process|GO:0008152,metabolic process|GO:0044257,cellular protein catabolic process|GO:0009056,catabolic process|GO:0009057,macromolecule catabolic process|GO:0051603,proteolysis involved in cellular protein catabolic process|GO:0019538,protein metabolic process|GO:0010498,proteasomal protein catabolic process|GO:0044237,cellular metabolic process|GO:0043170,macromolecule metabolic process|GO:0016567,protein ubiquitination|GO:0006511,ubiquitin-dependent protein catabolic process|GO:0006807,nitrogen compound metabolic process|GO:0000209,protein polyubiquitination|GO:0043687,post-translational protein modification|GO:1901564,organonitrogen compound metabolic process|GO:1901565,organonitrogen compound catabolic process	GO:0043234,protein complex|GO:0005737,cytoplasm|GO:0032991,macromolecular complex|GO:1902494,catalytic complex|GO:0044464,cell part|GO:0000151,ubiquitin ligase complex|GO:0005623,cell|GO:0005622,intracellular|GO:0019005,SCF ubiquitin ligase complex|GO:1990234,transferase complex|GO:0044424,intracellular part|GO:0031461,cullin-RING ubiquitin ligase complex|GO:0005829,cytosol|GO:0044444,cytoplasmic part	GO:0019787,ubiquitin-like protein transferase activity|GO:0061630,ubiquitin protein ligase activity|GO:0003824,catalytic activity|GO:0016740,transferase activity|GO:0005488,binding|GO:0005515,protein binding|GO:0004842,ubiquitin-protein transferase activity|GO:0061659,ubiquitin-like protein ligase activity
C22orf46		GO:0005576,extracellular region	
ZNF23	GO:0080090,regulation of primary metabolic process|GO:0019222,regulation of metabolic process|GO:0031326,regulation of cellular biosynthetic process|GO:0031323,regulation of cellular metabolic process|GO:0090304,nucleic acid metabolic process|GO:0044249,cellular biosynthetic process|GO:0034641,cellular nitrogen compound metabolic process|GO:0006807,nitrogen compound metabolic process|GO:0034645,cellular macromolecule biosynthetic process|GO:1901362,organic cyclic compound biosynthetic process|GO:0050789,regulation of biological process|GO:0097659,nucleic acid-templated transcription|GO:0032774,RNA biosynthetic process|GO:0006139,nucleobase-containing compound metabolic process|GO:0044260,cellular macromolecule metabolic process|GO:0071704,organic substance metabolic process|GO:2000112,regulation of cellular macromolecule biosynthetic process|GO:0060255,regulation of macromolecule metabolic process|GO:0010467,gene expression|GO:0065007,biological regulation|GO:1901360,organic cyclic compound metabolic process|GO:0010468,regulation of gene expression|GO:0018130,heterocycle biosynthetic process|GO:1901576,organic substance biosynthetic process|GO:0019219,regulation of nucleobase-containing compound metabolic process|GO:0006725,cellular aromatic compound metabolic process|GO:0009987,cellular process|GO:0009889,regulation of biosynthetic process|GO:1903506,regulation of nucleic acid-templated transcription|GO:0050794,regulation of cellular process|GO:0009058,biosynthetic process|GO:0009059,macromolecule biosynthetic process|GO:0051171,regulation of nitrogen compound metabolic process|GO:0008152,metabolic process|GO:2001141,regulation of RNA biosynthetic process|GO:0034654,nucleobase-containing compound biosynthetic process|GO:0046483,heterocycle metabolic process|GO:0016070,RNA metabolic process|GO:0044238,primary metabolic process|GO:0044271,cellular nitrogen compound biosynthetic process|GO:0051252,regulation of RNA metabolic process|GO:0044237,cellular metabolic process|GO:0043170,macromolecule metabolic process|GO:0006355,regulation of transcription, DNA-templated|GO:0010556,regulation of macromolecule biosynthetic process|GO:0006351,transcription, DNA-templated|GO:0019438,aromatic compound biosynthetic process	GO:0043231,intracellular membrane-bounded organelle|GO:0005634,nucleus|GO:0044464,cell part|GO:0005623,cell|GO:0005622,intracellular|GO:0043229,intracellular organelle|GO:0044424,intracellular part|GO:0043227,membrane-bounded organelle|GO:0043226,organelle	GO:0043169,cation binding|GO:0003700,transcription factor activity, sequence-specific DNA binding|GO:0097159,organic cyclic compound binding|GO:0043167,ion binding|GO:0005488,binding|GO:0003676,nucleic acid binding|GO:0003677,DNA binding|GO:0046872,metal ion binding|GO:1901363,heterocyclic compound binding|GO:0001071,nucleic acid binding transcription factor activity|GO:0005515,protein binding|GO:0000981,RNA polymerase II transcription factor activity, sequence-specific DNA binding
SH3PXD2A	GO:0030154,cell differentiation|GO:0006801,superoxide metabolic process|GO:0000768,syncytium formation by plasma membrane fusion|GO:0006949,syncytium formation|GO:0009653,anatomical structure morphogenesis|GO:0007275,multicellular organism development|GO:0044699,single-organism process|GO:0048869,cellular developmental process|GO:0002573,myeloid leukocyte differentiation|GO:0048513,animal organ development|GO:0048534,hematopoietic or lymphoid organ development|GO:0048646,anatomical structure formation involved in morphogenesis|GO:0032502,developmental process|GO:0032501,multicellular organismal process|GO:0030316,osteoclast differentiation|GO:0072593,reactive oxygen species metabolic process|GO:0044767,single-organism developmental process|GO:0072675,osteoclast fusion|GO:0044763,single-organism cellular process|GO:0008152,metabolic process|GO:0030097,hemopoiesis|GO:0002521,leukocyte differentiation|GO:0002520,immune system development|GO:0072674,multinuclear osteoclast differentiation|GO:0044707,single-multicellular organism process|GO:0048856,anatomical structure development|GO:0044237,cellular metabolic process|GO:0002376,immune system process|GO:0030099,myeloid cell differentiation|GO:0048731,system development|GO:0009987,cellular process|GO:0022607,cellular component assembly|GO:0070271,protein complex biogenesis|GO:0043933,macromolecular complex subunit organization|GO:0043085,positive regulation of catalytic activity|GO:0071800,podosome assembly|GO:0071822,protein complex subunit organization|GO:0016043,cellular component organization|GO:0065003,macromolecular complex assembly|GO:0030198,extracellular matrix organization|GO:0065007,biological regulation|GO:0071840,cellular component organization or biogenesis|GO:0044093,positive regulation of molecular function|GO:0043062,extracellular structure organization|GO:0065009,regulation of molecular function|GO:0050790,regulation of catalytic activity|GO:0006461,protein complex assembly|GO:0044710,single-organism metabolic process|GO:0070925,organelle assembly|GO:0055114,oxidation-reduction process|GO:0006996,organelle organization|GO:0044085,cellular component biogenesis	GO:0005856,cytoskeleton|GO:0043234,protein complex|GO:0002102,podosome|GO:0043228,non-membrane-bounded organelle|GO:0015629,actin cytoskeleton|GO:0043232,intracellular non-membrane-bounded organelle|GO:0044464,cell part|GO:0005623,cell|GO:0005737,cytoplasm|GO:0044446,intracellular organelle part|GO:0043229,intracellular organelle|GO:0044430,cytoskeletal part|GO:0030054,cell junction|GO:0044424,intracellular part|GO:0032991,macromolecular complex|GO:0042995,cell projection|GO:0043226,organelle|GO:0044422,organelle part|GO:0005622,intracellular|GO:0005829,cytosol|GO:0044444,cytoplasmic part	GO:0043168,anion binding|GO:0035091,phosphatidylinositol binding|GO:0005543,phospholipid binding|GO:0008289,lipid binding|GO:0043167,ion binding|GO:0005488,binding|GO:0005515,protein binding|GO:0030234,enzyme regulator activity|GO:0016176,superoxide-generating NADPH oxidase activator activity|GO:0008047,enzyme activator activity|GO:0098772,molecular function regulator|GO:0070273,phosphatidylinositol-4-phosphate binding|GO:0010314,phosphatidylinositol-5-phosphate binding|GO:0032266,phosphatidylinositol-3-phosphate binding|GO:0005546,phosphatidylinositol-4,5-bisphosphate binding|GO:1902936,phosphatidylinositol bisphosphate binding|GO:0043325,phosphatidylinositol-3,4-bisphosphate binding|GO:0019899,enzyme binding|GO:0002020,protease binding|GO:1901981,phosphatidylinositol phosphate binding

STXBP1	GO:0090087,regulation of peptide transport|GO:0008104,protein localization|GO:0007599,hemostasis|GO:0051046,regulation of secretion|GO:0051047,positive regulation of secretion|GO:0010807,regulation of synaptic vesicle priming|GO:0051049,regulation of transport|GO:0006906,vesicle fusion|GO:0048468,cell development|GO:0061025,membrane fusion|GO:0061024,membrane organization|GO:0007009,plasma membrane organization|GO:0043523,regulation of neuron apoptotic process|GO:0043524,negative regulation of neuron apoptotic process|GO:0044281,small molecule metabolic process|GO:0051641,cellular localization|GO:0051656,establishment of organelle localization|GO:0051650,establishment of vesicle localization|GO:0071840,cellular component organization or biogenesis|GO:0044710,single-organism metabolic process|GO:0072659,protein localization to plasma membrane|GO:0070727,cellular macromolecule localization|GO:0016188,synaptic vesicle maturation|GO:0048869,cellular developmental process|GO:0070271,protein complex biogenesis|GO:0010256,endomembrane system organization|GO:0009611,response to wounding|GO:0046879,hormone secretion|GO:0030168,platelet activation|GO:0006904,vesicle docking involved in exocytosis|GO:0048518,positive regulation of biological process|GO:0048519,negative regulation of biological process|GO:0097479,synaptic vesicle localization|GO:0033036,macromolecule localization|GO:0001505,regulation of neurotransmitter levels|GO:0006865,amino acid transport|GO:0051050,positive regulation of transport|GO:0060548,negative regulation of cell death|GO:0071702,organic substance transport|GO:0015711,organic anion transport|GO:0045184,establishment of protein localization|GO:0048278,vesicle docking|GO:0090276,regulation of peptide hormone secretion|GO:0006835,dicarboxylic acid transport|GO:0072657,protein localization to membrane|GO:0006836,neurotransmitter transport|GO:0051128,regulation of cellular component organization|GO:0055114,oxidation-reduction process|GO:0002576,platelet degranulation|GO:0044700,single organism signaling|GO:0016192,vesicle-mediated transport|GO:0044707,single-multicellular organism process|GO:0090174,organelle membrane fusion|GO:0006820,anion transport|GO:0032229,negative regulation of synaptic transmission, GABAergic|GO:0032228,regulation of synaptic transmission, GABAergic|GO:1903530,regulation of secretion by cell|GO:0022607,cellular component assembly|GO:0006950,response to stress|GO:0023051,regulation of signaling|GO:0046717,acid secretion|GO:0050821,protein stabilization|GO:0048167,regulation of synaptic plasticity|GO:0065007,biological regulation|GO:0016050,vesicle organization|GO:0006091,generation of precursor metabolites and energy|GO:0031175,neuron projection development|GO:0051588,regulation of neurotransmitter transport|GO:0051223,regulation of protein transport|GO:0042981,regulation of apoptotic process|GO:0050789,regulation of biological process|GO:0030072,peptide hormone secretion|GO:0030073,insulin secretion|GO:0000904,cell morphogenesis involved in differentiation|GO:0050708,regulation of protein secretion|GO:0000902,cell morphogenesis|GO:0070997,neuron death|GO:0006887,exocytosis|GO:1990778,protein localization to cell periphery|GO:0016043,cellular component organization|GO:0065003,macromolecular complex assembly|GO:0098609,cell-cell adhesion|GO:0006112,energy reserve metabolic process|GO:0065008,regulation of biological quality|GO:0034613,cellular protein localization|GO:0016265,obsolete death|GO:0070201,regulation of establishment of protein localization|GO:0098602,single organism cell adhesion|GO:0061564,axon development|GO:0009306,protein secretion|GO:0006811,ion transport|GO:0006810,transport|GO:0050796,regulation of insulin secretion|GO:0042060,wound healing|GO:0050794,regulation of cellular process|GO:0035542,regulation of SNARE complex assembly|GO:0001775,cell activation|GO:0050817,coagulation|GO:0044802,single-organism membrane organization|GO:0008152,metabolic process|GO:0044801,single-organism membrane fusion|GO:1901214,regulation of neuron death|GO:1901215,negative regulation of neuron death|GO:0014047,glutamate secretion|GO:0046903,secretion|GO:0015980,energy derivation by oxidation of organic compounds|GO:0012501,programmed cell death|GO:0016079,synaptic vesicle exocytosis|GO:0050896,response to stimulus|GO:0031647,regulation of protein stability|GO:0044765,single-organism transport|GO:0048812,neuron projection morphogenesis|GO:0035493,SNARE complex assembly|GO:2000300,regulation of synaptic vesicle exocytosis|GO:0051648,vesicle localization|GO:0033043,regulation of organelle organization|GO:0050803,regulation of synapse structure or activity|GO:0030154,cell differentiation|GO:0050805,negative regulation of synaptic transmission|GO:0050804,modulation of synaptic transmission|GO:0007596,blood coagulation|GO:0023057,negative regulation of signaling|GO:0015833,peptide transport|GO:0023052,signaling|GO:0010648,negative regulation of cell communication|GO:1903532,positive regulation of secretion by cell|GO:0007412,axon target recognition|GO:0010646,regulation of cell communication|GO:0043254,regulation of protein complex assembly|GO:0044699,single-organism process|GO:0032880,regulation of protein localization|GO:0042886,amide transport|GO:0051234,establishment of localization|GO:0051649,establishment of localization in cell|GO:0009653,anatomical structure morphogenesis|GO:0044085,cellular component biogenesis|GO:0007155,cell adhesion|GO:0017158,regulation of calcium ion-dependent exocytosis|GO:0043933,macromolecular complex subunit organization|GO:0051640,organelle localization|GO:0022610,biological adhesion|GO:0017156,calcium ion regulated exocytosis|GO:0017157,regulation of exocytosis|GO:0031338,regulation of vesicle fusion|GO:0032501,multicellular organismal process|GO:0050878,regulation of body fluid levels|GO:0016082,synaptic vesicle priming|GO:0060627,regulation of vesicle-mediated transport|GO:0046928,regulation of neurotransmitter secretion|GO:0007409,axonogenesis|GO:0032879,regulation of localization|GO:0032990,cell part morphogenesis|GO:0006461,protein complex assembly|GO:0060292,long term synaptic depression|GO:0032940,secretion by cell|GO:0031629,synaptic vesicle fusion to presynaptic active zone membrane|GO:0097480,establishment of synaptic vesicle localization|GO:0048731,system development|GO:0032502,developmental process|GO:0045921,positive regulation of exocytosis|GO:0016337,single organismal cell-cell adhesion|GO:0060341,regulation of cellular localization|GO:0030030,cell projection organization|GO:0022406,membrane docking|GO:0034622,cellular macromolecular complex assembly|GO:0007270,neuron-neuron synaptic transmission|GO:0008219,cell death|GO:0010941,regulation of cell death|GO:0007274,neuromuscular synaptic transmission|GO:0007275,multicellular organism development|GO:0031630,regulation of synaptic vesicle fusion to presynaptic active zone membrane|GO:0071822,protein complex subunit organization|GO:0021700,developmental maturation|GO:0051932,synaptic transmission, GABAergic|GO:0032989,cellular component morphogenesis|GO:0071705,nitrogen compound transport|GO:0045956,positive regulation of calcium ion-dependent exocytosis|GO:0043067,regulation of programmed cell death|GO:0043066,negative regulation of apoptotic process|GO:0015849,organic acid transport|GO:0048489,synaptic vesicle transport|GO:0043069,negative regulation of programmed cell death|GO:0097285,obsolete cell-type specific apoptotic process|GO:0048666,neuron development|GO:0048667,cell morphogenesis involved in neuron differentiation|GO:0009987,cellular process|GO:0030182,neuron differentiation|GO:0034109,homotypic cell-cell adhesion|GO:0006915,apoptotic process|GO:0023061,signal release|GO:0010817,regulation of hormone levels|GO:0044767,single-organism developmental process|GO:0051402,neuron apoptotic process|GO:0044763,single-organism cellular process|GO:0007269,neurotransmitter secretion|GO:0007268,chemical synaptic transmission|GO:0007267,cell-cell signaling|GO:0007154,cell communication|GO:0022008,neurogenesis|GO:0043623,cellular protein complex assembly|GO:0051179,localization|GO:1902578,single-organism localization|GO:0046942,carboxylic acid transport|GO:0006996,organelle organization|GO:0048699,generation of neurons|GO:0048858,cell projection morphogenesis|GO:0007399,nervous system development|GO:0002790,peptide secretion|GO:0002791,regulation of peptide secretion|GO:0048856,anatomical	GO:0042995,cell projection|GO:0031974,membrane-enclosed lumen|GO:0030424,axon|GO:0043229,intracellular organelle|GO:0071944,cell periphery|GO:0045202,synapse|GO:0043227,membrane-bounded organelle|GO:0043226,organelle|GO:0005737,cytoplasm|GO:0043679,axon terminus|GO:0044306,neuron projection terminus|GO:0031091,platelet alpha granule|GO:0070062,extracellular exosome|GO:0031981,nuclear lumen|GO:0016023,cytoplasmic, membrane-bounded vesicle|GO:0031410,cytoplasmic vesicle|GO:0005634,nucleus|GO:0016020,membrane|GO:0005739,mitochondrion|GO:0031988,membrane-bounded vesicle|GO:0005654,nucleoplasm|GO:0097458,neuron part|GO:0044456,synapse part|GO:0043005,neuron projection|GO:0043209,myelin sheath|GO:0032991,macromolecular complex|GO:0030141,secretory granule|GO:0012505,endomembrane system|GO:0005886,plasma membrane|GO:0043230,extracellular organelle|GO:1903561,extracellular vesicle|GO:0031982,vesicle|GO:0043234,protein complex|GO:0043195,terminal bouton|GO:0043231,intracellular membrane-bounded organelle|GO:0043233,organelle lumen|GO:0005829,cytosol|GO:0044463,cell projection part|GO:0044464,cell part|GO:0005623,cell|GO:0005622,intracellular|GO:0044446,intracellular organelle part|GO:0070013,intracellular organelle lumen|GO:0044444,cytoplasmic part|GO:0005576,extracellular region|GO:0044428,nuclear part|GO:0033267,axon part|GO:0044424,intracellular part|GO:0065010,extracellular membrane-bounded organelle|GO:0044421,extracellular region part|GO:0044422,organelle part|GO:0098590,plasma membrane region|GO:0043228,non-membrane-bounded organelle|GO:0097060,synaptic membrane|GO:0048787,presynaptic active zone membrane|GO:0048786,presynaptic active zone|GO:0098793,presynapse|GO:0048471,perinuclear region of cytoplasm|GO:0044459,plasma membrane part|GO:0005856,cytoskeleton|GO:0099503,secretory vesicle|GO:0098589,membrane region|GO:0097708,intracellular vesicle|GO:0098805,whole membrane|GO:0043232,intracellular non-membrane-bounded organelle|GO:0042734,presynaptic membrane|GO:0098794,postsynapse|GO:0044425,membrane part|GO:0030139,endocytic vesicle|GO:0045335,phagocytic vesicle	GO:0005488,binding|GO:0005515,protein binding|GO:0047485,protein N-terminus binding|GO:0097159,organic cyclic compound binding|GO:0044822,poly(A) RNA binding|GO:0019899,enzyme binding|GO:0019901,protein kinase binding|GO:0019900,kinase binding|GO:0019905,syntaxin binding|GO:0003723,RNA binding|GO:0003676,nucleic acid binding|GO:0000149,SNARE binding|GO:0017075,syntaxin-1 binding|GO:1901363,heterocyclic compound binding|GO:0042802,identical protein binding|GO:0019904,protein domain specific binding|GO:0043274,phospholipase binding
PPP1R1C	GO:0043549,regulation of kinase activity|GO:0019220,regulation of phosphate metabolic process|GO:0080090,regulation of primary metabolic process|GO:0019222,regulation of metabolic process|GO:0000082,G1/S transition of mitotic cell cycle|GO:0007165,signal transduction|GO:0071840,cellular component organization or biogenesis|GO:0051716,cellular response to stimulus|GO:0010605,negative regulation of macromolecule metabolic process|GO:0045787,positive regulation of cell cycle|GO:0016310,phosphorylation|GO:0048518,positive regulation of biological process|GO:0048519,negative regulation of biological process|GO:0060255,regulation of macromolecule metabolic process|GO:0045859,regulation of protein kinase activity|GO:0042325,regulation of phosphorylation|GO:0044700,single organism signaling|GO:0042326,negative regulation of phosphorylation|GO:0019538,protein metabolic process|GO:0007154,cell communication|GO:0033673,negative regulation of kinase activity|GO:0009892,negative regulation of metabolic process|GO:0044092,negative regulation of molecular function|GO:0035556,intracellular signal transduction|GO:0050789,regulation of biological process|GO:0090068,positive regulation of cell cycle process|GO:0007346,regulation of mitotic cell cycle|GO:0016049,cell growth|GO:0044260,cellular macromolecule metabolic process|GO:0051348,negative regulation of transferase activity|GO:0016043,cellular component organization|GO:0065007,biological regulation|GO:0044699,single-organism process|GO:0044267,cellular protein metabolic process|GO:0065009,regulation of molecular function|GO:0050790,regulation of catalytic activity|GO:0050794,regulation of cellular process|GO:1901987,regulation of cell cycle phase transition|GO:0043412,macromolecule modification|GO:0036211,protein modification process|GO:0008152,metabolic process|GO:1901989,positive regulation of cell cycle phase transition|GO:0031400,negative regulation of protein modification process|GO:0051338,regulation of transferase activity|GO:0051246,regulation of protein metabolic process|GO:0030307,positive regulation of cell growth|GO:0051128,regulation of cellular component organization|GO:0023052,signaling|GO:1901990,regulation of mitotic cell cycle phase transition|GO:1901992,positive regulation of mitotic cell cycle phase transition|GO:0043086,negative regulation of catalytic activity|GO:0007049,cell cycle|GO:0051248,negative regulation of protein metabolic process|GO:0010563,negative regulation of phosphorus metabolic process|GO:0010564,regulation of cell cycle process|GO:0031399,regulation of protein modification process|GO:0009987,cellular process|GO:0001558,regulation of cell growth|GO:0032269,negative regulation of cellular protein metabolic process|GO:0032268,regulation of cellular protein metabolic process|GO:2000045,regulation of G1/S transition of mitotic cell cycle|GO:0043170,macromolecule metabolic process|GO:0050896,response to stimulus|GO:0045927,positive regulation of growth|GO:0031324,negative regulation of cellular metabolic process|GO:0031323,regulation of cellular metabolic process|GO:1903047,mitotic cell cycle process|GO:0044770,cell cycle phase transition|GO:0044772,mitotic cell cycle phase transition|GO:0022402,cell cycle process|GO:0051301,cell division|GO:0040007,growth|GO:0040008,regulation of growth|GO:0071704,organic substance metabolic process|GO:1900087,positive regulation of G1/S transition of mitotic cell cycle|GO:0045931,positive regulation of mitotic cell cycle|GO:0006468,protein phosphorylation|GO:0006469,negative regulation of protein kinase activity|GO:0000278,mitotic cell cycle|GO:0045936,negative regulation of phosphate metabolic process|GO:0006464,cellular protein modification process|GO:0051174,regulation of phosphorus metabolic process|GO:0044763,single-organism cellular process|GO:0044843,cell cycle G1/S phase transition|GO:0044238,primary metabolic process|GO:0051726,regulation of cell cycle|GO:0044237,cellular metabolic process|GO:1902806,regulation of cell cycle G1/S phase transition|GO:0006796,phosphate-containing compound metabolic process|GO:1902808,positive regulation of cell cycle G1/S phase transition|GO:0006793,phosphorus metabolic process|GO:0001933,negative regulation of protein phosphorylation|GO:0001932,regulation of protein phosphorylation|GO:0048523,negative regulation of cellular process|GO:0048522,positive regulation of cellular process|GO:1901564,organonitrogen compound metabolic process|GO:0006807,nitrogen compound metabolic process	GO:0005737,cytoplasm|GO:0044464,cell part|GO:0005623,cell|GO:0005622,intracellular|GO:0044424,intracellular part	GO:0019212,phosphatase inhibitor activity|GO:0019888,protein phosphatase regulator activity|GO:0019208,phosphatase regulator activity|GO:0004864,protein phosphatase inhibitor activity|GO:0004865,protein serine/threonine phosphatase inhibitor activity|GO:0004857,enzyme inhibitor activity|GO:0098772,molecular function regulator|GO:0030234,enzyme regulator activity
CIR1	GO:0009892,negative regulation of metabolic process|GO:0080090,regulation of primary metabolic process|GO:0009890,negative regulation of biosynthetic process|GO:0031327,negative regulation of cellular biosynthetic process|GO:0031326,regulation of cellular biosynthetic process|GO:0031324,negative regulation of cellular metabolic process|GO:0031323,regulation of cellular metabolic process|GO:0090304,nucleic acid metabolic process|GO:0044249,cellular biosynthetic process|GO:0010629,negative regulation of gene expression|GO:0006807,nitrogen compound metabolic process|GO:0034645,cellular macromolecule biosynthetic process|GO:1901576,organic substance biosynthetic process|GO:0043170,macromolecule metabolic process|GO:1901362,organic cyclic compound biosynthetic process|GO:0050789,regulation of biological process|GO:0097659,nucleic acid-templated transcription|GO:0032774,RNA biosynthetic process|GO:0006139,nucleobase-containing compound metabolic process|GO:0010605,negative regulation of macromolecule metabolic process|GO:0019222,regulation of metabolic process|GO:0044260,cellular macromolecule metabolic process|GO:0071704,organic substance metabolic process|GO:0006396,RNA processing|GO:2000112,regulation of cellular macromolecule biosynthetic process|GO:2000113,negative regulation of cellular macromolecule biosynthetic process|GO:0060255,regulation of macromolecule metabolic process|GO:0010467,gene expression|GO:0010556,regulation of macromolecule biosynthetic process|GO:0065007,biological regulation|GO:1901360,organic cyclic compound metabolic process|GO:0006366,transcription from RNA polymerase II promoter|GO:0048519,negative regulation of biological process|GO:0010468,regulation of gene expression|GO:0018130,heterocycle biosynthetic process|GO:0045934,negative regulation of nucleobase-containing compound metabolic process|GO:0044238,primary metabolic process|GO:0019219,regulation of nucleobase-containing compound metabolic process|GO:0006725,cellular aromatic compound metabolic process|GO:0009987,cellular process|GO:0009889,regulation of biosynthetic process|GO:1903506,regulation of nucleic acid-templated transcription|GO:1903507,negative regulation of nucleic acid-templated transcription|GO:1902679,negative regulation of RNA biosynthetic process|GO:0009058,biosynthetic process|GO:0009059,macromolecule biosynthetic process|GO:0045892,negative regulation of transcription, DNA-templated|GO:0051171,regulation of nitrogen compound metabolic process|GO:0051172,negative regulation of nitrogen compound metabolic process|GO:2001141,regulation of RNA biosynthetic process|GO:0034654,nucleobase-containing compound biosynthetic process|GO:0050794,regulation of cellular process|GO:0008152,metabolic process|GO:0046483,heterocycle metabolic process|GO:0016070,RNA metabolic process|GO:0016071,mRNA metabolic process|GO:0044271,cellular nitrogen compound biosynthetic process|GO:0051253,negative regulation of RNA metabolic process|GO:0051252,regulation of RNA metabolic process|GO:0034641,cellular nitrogen compound metabolic process|GO:0044237,cellular metabolic process|GO:0008380,RNA splicing|GO:0006355,regulation of transcription, DNA-templated|GO:0006357,regulation of transcription from RNA polymerase II promoter|GO:0006351,transcription, DNA-templated|GO:0019438,aromatic compound biosynthetic process|GO:0010558,negative regulation of macromolecule biosynthetic process|GO:0048523,negative regulation of cellular	GO:0031974,membrane-enclosed lumen|GO:0043229,intracellular organelle|GO:0043228,non-membrane-bounded organelle|GO:0005622,intracellular|GO:0043227,membrane-bounded organelle|GO:0043226,organelle|GO:0016607,nuclear speck|GO:0016604,nuclear body|GO:0031981,nuclear lumen|GO:0005634,nucleus|GO:0005815,microtubule organizing center|GO:0005654,nucleoplasm|GO:0044430,cytoskeletal part|GO:0044451,nucleoplasm part|GO:0005856,cytoskeleton|GO:0015630,microtubule cytoskeleton|GO:1902494,catalytic complex|GO:0000118,histone deacetylase complex|GO:0043234,protein complex|GO:0032991,macromolecular complex|GO:0043231,intracellular membrane-bounded organelle|GO:0043232,intracellular non-membrane-bounded organelle|GO:0043233,organelle lumen|GO:0044464,cell part|GO:0005623,cell|GO:0005737,cytoplasm|GO:0044446,intracellular organelle part|GO:0070013,intracellular organelle lumen|GO:0044428,nuclear part|GO:0044424,intracellular part|GO:0044422,organelle part	GO:0005515,protein binding|GO:0003700,transcription factor activity, sequence-specific DNA binding|GO:0003714,transcription corepressor activity|GO:0003712,transcription cofactor activity|GO:0003705,transcription factor activity, RNA polymerase II distal enhancer sequence-specific binding|GO:0005488,binding|GO:0000989,transcription factor activity, transcription factor binding|GO:0000988,transcription factor activity, protein binding|GO:0000981,RNA polymerase II transcription factor activity, sequence-specific DNA binding|GO:0001071,nucleic acid binding transcription factor activity|GO:0019901,protein kinase binding|GO:0019900,kinase binding|GO:0019899,enzyme binding
DIAPH2	GO:0022607,cellular component assembly|GO:0048856,anatomical structure development|GO:0070271,protein complex biogenesis|GO:0043933,macromolecular complex subunit organization|GO:0030154,cell differentiation|GO:0048468,cell development|GO:0019953,sexual reproduction|GO:0030036,actin cytoskeleton organization|GO:0007292,female gamete generation|GO:0022402,cell cycle process|GO:0007165,signal transduction|GO:0007276,gamete generation|GO:0034622,cellular macromolecular complex assembly|GO:0035556,intracellular signal transduction|GO:0007275,multicellular organism development|GO:0007049,cell cycle|GO:0051716,cellular response to stimulus|GO:0032504,multicellular organism reproduction|GO:0071822,protein complex subunit organization|GO:0048869,cellular developmental process|GO:0071840,cellular component organization or biogenesis|GO:0016043,cellular component organization|GO:0050789,regulation of biological process|GO:0065003,macromolecular complex assembly|GO:0065007,biological regulation|GO:0051301,cell division|GO:0051704,multi-organism process|GO:0048477,oogenesis|GO:0032502,developmental process|GO:0006996,organelle organization|GO:0032501,multicellular organismal process|GO:0048609,multicellular organismal reproductive process|GO:0030029,actin filament-based process|GO:0007015,actin filament organization|GO:0006461,protein complex assembly|GO:0044703,multi-organism reproductive process|GO:0050794,regulation of cellular process|GO:0007281,germ cell development|GO:0003006,developmental process involved in reproduction|GO:0044763,single-organism cellular process|GO:0022412,cellular process involved in reproduction in multicellular organism|GO:0030041,actin filament polymerization|GO:0007154,cell communication|GO:0007264,small GTPase mediated signal transduction|GO:0008154,actin polymerization or depolymerization|GO:0000003,reproduction|GO:0000910,cytokinesis|GO:0044700,single organism signaling|GO:0044699,single-organism process|GO:0044702,single organism reproductive process|GO:0007010,cytoskeleton organization|GO:0044707,single-multicellular organism process|GO:0022414,reproductive process|GO:0050896,response to stimulus|GO:0051258,protein polymerization|GO:0043623,cellular protein complex assembly|GO:1902589,single-organism organelle organization|GO:0044085,cellular component biogenesis|GO:0044767,single-organism developmental process|GO:0023052,signaling|GO:0009987,cellular process|GO:0097435,fibril organization|GO:0043062,extracellular structure organization|GO:0030198,extracellular matrix organization	GO:0031974,membrane-enclosed lumen|GO:0043229,intracellular organelle|GO:0043228,non-membrane-bounded organelle|GO:0043227,membrane-bounded organelle|GO:0043226,organelle|GO:0005737,cytoplasm|GO:0031981,nuclear lumen|GO:0005730,nucleolus|GO:0005634,nucleus|GO:0005739,mitochondrion|GO:0005794,Golgi apparatus|GO:0012505,endomembrane system|GO:0043231,intracellular membrane-bounded organelle|GO:0043232,intracellular non-membrane-bounded organelle|GO:0043233,organelle lumen|GO:0005829,cytosol|GO:0044464,cell part|GO:0005623,cell|GO:0005622,intracellular|GO:0044446,intracellular organelle part|GO:0070013,intracellular organelle lumen|GO:0044444,cytoplasmic part|GO:0044428,nuclear part|GO:0044424,intracellular part|GO:0005768,endosome|GO:0005769,early endosome|GO:0044422,organelle part|GO:0005783,endoplasmic reticulum|GO:0031982,vesicle|GO:0031410,cytoplasmic vesicle|GO:0097708,intracellular vesicle	GO:0008092,cytoskeletal protein binding|GO:0017048,Rho GTPase binding|GO:0017016,Ras GTPase binding|GO:0003779,actin binding|GO:0031267,small GTPase binding|GO:0051020,GTPase binding|GO:0019899,enzyme binding|GO:0005515,protein binding|GO:0005102,receptor binding|GO:0005488,binding
ZNF667	GO:0080090,regulation of primary metabolic process|GO:0019222,regulation of metabolic process|GO:0031326,regulation of cellular biosynthetic process|GO:0031323,regulation of cellular metabolic process|GO:0090304,nucleic acid metabolic process|GO:0044249,cellular biosynthetic process|GO:0034641,cellular nitrogen compound metabolic process|GO:0006807,nitrogen compound metabolic process|GO:0034645,cellular macromolecule biosynthetic process|GO:1901362,organic cyclic compound biosynthetic process|GO:0050789,regulation of biological process|GO:0097659,nucleic acid-templated transcription|GO:0032774,RNA biosynthetic process|GO:0006139,nucleobase-containing compound metabolic process|GO:0044260,cellular macromolecule metabolic process|GO:0071704,organic substance metabolic process|GO:2000112,regulation of cellular macromolecule biosynthetic process|GO:0060255,regulation of macromolecule metabolic process|GO:0010467,gene expression|GO:0006357,regulation of transcription from RNA polymerase II promoter|GO:0065007,biological regulation|GO:1901360,organic cyclic compound metabolic process|GO:0006366,transcription from RNA polymerase II promoter|GO:0010468,regulation of gene expression|GO:0018130,heterocycle biosynthetic process|GO:1901576,organic substance biosynthetic process|GO:0019219,regulation of nucleobase-containing compound metabolic process|GO:0006725,cellular aromatic compound metabolic process|GO:0009987,cellular process|GO:0009889,regulation of biosynthetic process|GO:1903506,regulation of nucleic acid-templated transcription|GO:0050794,regulation of cellular process|GO:0009058,biosynthetic process|GO:0009059,macromolecule biosynthetic process|GO:0051171,regulation of nitrogen compound metabolic process|GO:0008152,metabolic process|GO:2001141,regulation of RNA biosynthetic process|GO:0034654,nucleobase-containing compound biosynthetic process|GO:0046483,heterocycle metabolic process|GO:0016070,RNA metabolic process|GO:0044238,primary metabolic process|GO:0044271,cellular nitrogen compound biosynthetic process|GO:0051252,regulation of RNA metabolic process|GO:0044237,cellular metabolic process|GO:0043170,macromolecule metabolic process|GO:0006355,regulation of transcription, DNA-templated|GO:0010556,regulation of macromolecule biosynthetic	GO:0043231,intracellular membrane-bounded organelle|GO:0005634,nucleus|GO:0044464,cell part|GO:0005623,cell|GO:0005622,intracellular|GO:0043229,intracellular organelle|GO:0044424,intracellular part|GO:0043227,membrane-bounded organelle|GO:0043226,organelle	GO:0003676,nucleic acid binding|GO:0097159,organic cyclic compound binding|GO:0003712,transcription cofactor activity|GO:0005488,binding|GO:0000989,transcription factor activity, transcription factor binding|GO:0000988,transcription factor activity, protein binding|GO:1901363,heterocyclic compound binding
CLLU1OS			
UQCRB	GO:0009141,nucleoside triphosphate metabolic process|GO:0009144,purine nucleoside triphosphate metabolic process|GO:0009167,purine ribonucleoside monophosphate metabolic process|GO:0034641,cellular nitrogen compound metabolic process|GO:0009060,aerobic respiration|GO:0044281,small molecule metabolic process|GO:0009161,ribonucleoside monophosphate metabolic process|GO:0009123,nucleoside monophosphate metabolic process|GO:0006091,generation of precursor metabolites and energy|GO:0009126,purine nucleoside monophosphate metabolic process|GO:0009259,ribonucleotide metabolic process|GO:0044699,single-organism process|GO:0006139,nucleobase-containing compound metabolic process|GO:0044710,single-organism metabolic process|GO:0042775,mitochondrial ATP synthesis coupled electron transport|GO:1901360,organic cyclic compound metabolic process|GO:0042278,purine nucleoside metabolic process|GO:0071704,organic substance metabolic process|GO:0009199,ribonucleoside triphosphate metabolic process|GO:0022900,electron transport chain|GO:0055086,nucleobase-containing small molecule metabolic process|GO:0022904,respiratory electron transport chain|GO:0006119,oxidative phosphorylation|GO:0009205,purine ribonucleoside triphosphate metabolic process|GO:0046034,ATP metabolic process|GO:0009987,cellular process|GO:0006725,cellular aromatic compound metabolic process|GO:0009150,purine ribonucleotide metabolic process|GO:0072521,purine-containing compound metabolic process|GO:0045333,cellular respiration|GO:0009116,nucleoside metabolic process|GO:0008152,metabolic process|GO:0009119,ribonucleoside metabolic process|GO:0046128,purine ribonucleoside metabolic process|GO:0042773,ATP synthesis coupled electron transport|GO:0055114,oxidation-reduction process|GO:0046483,heterocycle metabolic process|GO:0044238,primary metabolic process|GO:1901564,organonitrogen compound metabolic process|GO:0015980,energy derivation by oxidation of organic compounds|GO:0016310,phosphorylation|GO:0044763,single-organism cellular process|GO:0006122,mitochondrial electron transport, ubiquinol to cytochrome c|GO:0019693,ribose phosphate metabolic process|GO:0006163,purine nucleotide metabolic process|GO:1901657,glycosyl compound metabolic process|GO:0006796,phosphate-containing compound metabolic process|GO:0006807,nitrogen compound metabolic process|GO:0006793,phosphorus metabolic process|GO:0019637,organophosphate metabolic process|GO:0009117,nucleotide metabolic process|GO:0044237,cellular metabolic process|GO:0006753,nucleoside phosphate metabolic process|GO:1901135,carbohydrate derivative metabolic process|GO:0022607,cellular component assembly|GO:0070271,protein complex biogenesis|GO:0043933,macromolecular complex subunit organization|GO:0006810,transport|GO:0098660,inorganic ion transmembrane transport|GO:0017004,cytochrome complex assembly|GO:0034622,cellular macromolecular complex assembly|GO:0071840,cellular component organization or biogenesis|GO:0051234,establishment of localization|GO:0015992,proton transport|GO:0071822,protein complex subunit organization|GO:0016043,cellular component organization|GO:0055085,transmembrane transport|GO:0065003,macromolecular complex assembly|GO:1902600,hydrogen ion transmembrane transport|GO:0007005,mitochondrion organization|GO:0034551,mitochondrial respiratory chain complex III assembly|GO:1902578,single-organism localization|GO:0015672,monovalent inorganic cation transport|GO:0033108,mitochondrial respiratory chain complex assembly|GO:0006818,hydrogen transport|GO:0006811,ion transport|GO:0006461,protein complex assembly|GO:0034220,ion transmembrane transport|GO:0044765,single-organism transport|GO:0051179,localization|GO:0043623,cellular protein complex assembly|GO:0006996,organelle organization|GO:0098662,inorganic cation transmembrane transport|GO:0098655,cation transmembrane transport|GO:0097033,mitochondrial respiratory chain complex III biogenesis|GO:0044085,cellular	GO:0044464,cell part|GO:1990204,oxidoreductase complex|GO:0043229,intracellular organelle|GO:0070069,cytochrome complex|GO:0043227,membrane-bounded organelle|GO:0043226,organelle|GO:0031224,intrinsic component of membrane|GO:0005737,cytoplasm|GO:0031090,organelle membrane|GO:0098796,membrane protein complex|GO:0016021,integral component of membrane|GO:0016020,membrane|GO:0098803,respiratory chain complex|GO:0044444,cytoplasmic part|GO:0005739,mitochondrion|GO:0098800,inner mitochondrial membrane protein complex|GO:0044455,mitochondrial membrane part|GO:0005750,mitochondrial respiratory chain complex III|GO:1902495,transmembrane transporter complex|GO:1902494,catalytic complex|GO:1990351,transporter complex|GO:0031967,organelle envelope|GO:0031966,mitochondrial membrane|GO:0031975,envelope|GO:0043234,protein complex|GO:0032991,macromolecular complex|GO:0043231,intracellular membrane-bounded organelle|GO:0045275,respiratory chain complex III|GO:0019866,organelle inner membrane|GO:0005623,cell|GO:0005622,intracellular|GO:0044446,intracellular organelle part|GO:0005743,mitochondrial inner membrane|GO:0005740,mitochondrial envelope|GO:0005746,mitochondrial respiratory chain|GO:0044429,mitochondrial part|GO:0044424,intracellular	GO:0005488,binding|GO:0005515,protein binding|GO:0022891,substrate-specific transmembrane transporter activity|GO:0022890,inorganic cation transmembrane transporter activity|GO:0022892,substrate-specific transporter activity|GO:0005215,transporter activity|GO:0008324,cation transmembrane transporter activity|GO:0008121,ubiquinol-cytochrome-c reductase activity|GO:0022857,transmembrane transporter activity|GO:0015075,ion transmembrane transporter activity|GO:0003824,catalytic activity|GO:0015077,monovalent inorganic cation transmembrane transporter activity|GO:0016679,oxidoreductase activity, acting on diphenols and related substances as donors|GO:0015078,hydrogen ion transmembrane transporter activity|GO:0016681,oxidoreductase activity, acting on diphenols and related substances as donors, cytochrome as acceptor|GO:0016491,oxidoreductase activity
FAM83F			
THEM5	GO:0006631,fatty acid metabolic process|GO:0006637,acyl-CoA metabolic process|GO:0006650,glycerophospholipid metabolic process|GO:0006732,coenzyme metabolic process|GO:0032048,cardiolipin metabolic process|GO:0035383,thioester metabolic process|GO:0019752,carboxylic acid metabolic process|GO:0044281,small molecule metabolic process|GO:0035336,long-chain fatty-acyl-CoA metabolic process|GO:0035337,fatty-acyl-CoA metabolic process|GO:0044255,cellular lipid metabolic process|GO:0044699,single-organism process|GO:0044710,single-organism metabolic process|GO:0051186,cofactor metabolic process|GO:0071704,organic substance metabolic process|GO:0006644,phospholipid metabolic process|GO:0006629,lipid metabolic process|GO:0009987,cellular process|GO:0032787,monocarboxylic acid metabolic process|GO:0044763,single-organism cellular process|GO:0008152,metabolic process|GO:0043436,oxoacid metabolic process|GO:0046486,glycerolipid metabolic process|GO:0044238,primary metabolic process|GO:0006082,organic acid metabolic process|GO:0044237,cellular metabolic process|GO:0035965,cardiolipin acyl-chain remodeling|GO:0006796,phosphate-containing compound metabolic process|GO:0006790,sulfur compound metabolic process|GO:0006793,phosphorus metabolic process|GO:0019637,organophosphate metabolic process|GO:0046471,phosphatidylglycerol metabolic process|GO:1901568,fatty acid derivative metabolic process	GO:0005737,cytoplasm|GO:0043231,intracellular membrane-bounded organelle|GO:0043233,organelle lumen|GO:0044464,cell part|GO:0044444,cytoplasmic part|GO:0031974,membrane-enclosed lumen|GO:0005622,intracellular|GO:0044446,intracellular organelle part|GO:0070013,intracellular organelle lumen|GO:0043229,intracellular organelle|GO:0005739,mitochondrion|GO:0044429,mitochondrial part|GO:0005759,mitochondrial matrix|GO:0044424,intracellular part|GO:0005623,cell|GO:0043227,membrane-bounded organelle|GO:0043226,organelle|GO:0044422,organelle part	GO:0016787,hydrolase activity|GO:0016289,CoA hydrolase activity|GO:0016790,thiolester hydrolase activity|GO:0016788,hydrolase activity, acting on ester bonds|GO:0003824,catalytic activity|GO:0016290,palmitoyl-CoA hydrolase activity|GO:0005488,binding|GO:0005515,protein binding|GO:0047617,acyl-CoA hydrolase activity
AL627309	GO:0009593,detection of chemical stimulus|GO:0050906,detection of stimulus involved in sensory perception|GO:0007165,signal transduction|GO:0042221,response to chemical|GO:0050789,regulation of biological process|GO:0044699,single-organism process|GO:0051716,cellular response to stimulus|GO:0065007,biological regulation|GO:0007186,G-protein coupled receptor signaling pathway|GO:0032501,multicellular organismal process|GO:0007608,sensory perception of smell|GO:0050877,neurological system process|GO:0007606,sensory perception of chemical stimulus|GO:0007600,sensory perception|GO:0050794,regulation of cellular process|GO:0050911,detection of chemical stimulus involved in sensory perception of smell|GO:0044763,single-organism cellular process|GO:0007154,cell communication|GO:0003008,system process|GO:0044700,single organism signaling|GO:0051606,detection of stimulus|GO:0044707,single-multicellular organism process|GO:0050896,response to stimulus|GO:0023052,signaling|GO:0009987,cellular process|GO:0050907,detection of chemical stimulus involved in sensory perception	GO:0016021,integral component of membrane|GO:0016020,membrane|GO:0044464,cell part|GO:0005623,cell|GO:0071944,cell periphery|GO:0005886,plasma membrane|GO:0044425,membrane part|GO:0031224,intrinsic component of membrane	GO:0004930,G-protein coupled receptor activity|GO:0038023,signaling receptor activity|GO:0060089,molecular transducer activity|GO:0004888,transmembrane signaling receptor activity|GO:0004872,receptor activity|GO:0004871,signal transducer activity|GO:0004984,olfactory receptor activity|GO:0099600,transmembrane receptor activity
LHPP	GO:0071704,organic substance metabolic process|GO:0044267,cellular protein metabolic process|GO:0044260,cellular macromolecule metabolic process|GO:0044238,primary metabolic process|GO:0016311,dephosphorylation|GO:0006470,protein dephosphorylation|GO:0009987,cellular process|GO:0043412,macromolecule modification|GO:0006464,cellular protein modification process|GO:0043170,macromolecule metabolic process|GO:0019538,protein metabolic process|GO:0006796,phosphate-containing compound metabolic process|GO:0036211,protein modification process|GO:0044237,cellular metabolic process|GO:0008152,metabolic process|GO:0006793,phosphorus metabolic process|GO:0009124,nucleoside monophosphate biosynthetic process|GO:0009167,purine ribonucleoside monophosphate metabolic process|GO:0034641,cellular nitrogen compound metabolic process|GO:0006807,nitrogen compound metabolic process|GO:0044281,small molecule metabolic process|GO:0009161,ribonucleoside monophosphate metabolic process|GO:0009123,nucleoside monophosphate metabolic process|GO:0009126,purine nucleoside monophosphate metabolic process|GO:0009127,purine nucleoside monophosphate biosynthetic process|GO:0044699,single-organism process|GO:0006139,nucleobase-containing compound metabolic process|GO:0044710,single-organism metabolic process|GO:0044711,single-organism biosynthetic process|GO:1901362,organic cyclic compound biosynthetic process|GO:1901360,organic cyclic compound metabolic process|GO:1901576,organic substance biosynthetic process|GO:0009168,purine ribonucleoside monophosphate biosynthetic process|GO:0018130,heterocycle biosynthetic process|GO:0009156,ribonucleoside monophosphate biosynthetic process|GO:0006725,cellular aromatic compound metabolic process|GO:0044763,single-organism cellular process|GO:0034654,nucleobase-containing compound biosynthetic process|GO:1901564,organonitrogen compound metabolic process|GO:0090407,organophosphate biosynthetic process|GO:0055086,nucleobase-containing small molecule metabolic process|GO:0046483,heterocycle metabolic process|GO:0044271,cellular nitrogen compound biosynthetic process|GO:0044249,cellular biosynthetic process|GO:0009058,biosynthetic process|GO:1901293,nucleoside phosphate biosynthetic process|GO:0019637,organophosphate metabolic process|GO:0019438,aromatic compound biosynthetic process|GO:0006753,nucleoside phosphate metabolic process	GO:0005737,cytoplasm|GO:0044446,intracellular organelle part|GO:0005829,cytosol|GO:0043231,intracellular membrane-bounded organelle|GO:0031981,nuclear lumen|GO:0043233,organelle lumen|GO:0005634,nucleus|GO:0044464,cell part|GO:0044444,cytoplasmic part|GO:0031974,membrane-enclosed lumen|GO:0005622,intracellular|GO:0005654,nucleoplasm|GO:0070013,intracellular organelle lumen|GO:0043229,intracellular organelle|GO:0044428,nuclear part|GO:0005623,cell|GO:0044424,intracellular part|GO:0043227,membrane-bounded organelle|GO:0043226,organelle|GO:0044422,organelle part|GO:0016607,nuclear speck|GO:0016604,nuclear body|GO:0044451,nucleoplasm part	GO:0008969,phosphohistidine phosphatase activity|GO:0016787,hydrolase activity|GO:0016818,hydrolase activity, acting on acid anhydrides, in phosphorus-containing anhydrides|GO:0016791,phosphatase activity|GO:0004427,inorganic diphosphatase activity|GO:0003824,catalytic activity|GO:0000287,magnesium ion binding|GO:0016817,hydrolase activity, acting on acid anhydrides|GO:0016788,hydrolase activity, acting on ester bonds|GO:0043167,ion binding|GO:0042578,phosphoric ester hydrolase activity|GO:0016462,pyrophosphatase activity|GO:0043169,cation binding|GO:0005488,binding|GO:0005515,protein binding|GO:0046983,protein dimerization activity|GO:0046872,metal ion binding|GO:0042802,identical protein binding|GO:0042803,protein homodimerization activity|GO:0101006,protein histidine phosphatase activity|GO:0004721,phosphoprotein phosphatase activity
FLJ45079			
IKBKG	GO:0019220,regulation of phosphate metabolic process|GO:0080090,regulation of primary metabolic process|GO:0019222,regulation of metabolic process|GO:0048584,positive regulation of response to stimulus|GO:0048583,regulation of response to stimulus|GO:0032147,activation of protein kinase activity|GO:0031349,positive regulation of defense response|GO:0007165,signal transduction|GO:0007166,cell surface receptor signaling pathway|GO:1901576,organic substance biosynthetic process|GO:0051656,establishment of organelle localization|GO:1901362,organic cyclic compound biosynthetic process|GO:0051650,establishment of vesicle localization|GO:0023014,signal transduction by protein phosphorylation|GO:0032774,RNA biosynthetic process|GO:0044710,single-organism metabolic process|GO:0010604,positive regulation of macromolecule metabolic process|GO:0043207,response to external biotic stimulus|GO:0009966,regulation of signal transduction|GO:0009967,positive regulation of signal transduction|GO:0000165,MAPK cascade|GO:0044419,interspecies interaction between organisms|GO:0044093,positive regulation of molecular function|GO:0016310,phosphorylation|GO:0035666,TRIF-dependent toll-like receptor signaling pathway|GO:0048519,negative regulation of biological process|GO:0051704,multi-organism process|GO:0043122,regulation of I-kappaB kinase/NF-kappaB signaling|GO:0043123,positive regulation of I-kappaB kinase/NF-kappaB signaling|GO:0060255,regulation of macromolecule metabolic process|GO:0060548,negative regulation of cell death|GO:0045859,regulation of protein kinase activity|GO:0006952,defense response|GO:0034162,toll-like receptor 9 signaling pathway|GO:0032268,regulation of cellular protein metabolic process|GO:0034166,toll-like receptor 10 signaling pathway|GO:2001141,regulation of RNA biosynthetic process|GO:0051707,response to other organism|GO:0065009,regulation of molecular function|GO:0046483,heterocycle metabolic process|GO:0042325,regulation of phosphorylation|GO:0044700,single organism signaling|GO:0042327,positive regulation of phosphorylation|GO:0002429,immune response-activating cell surface receptor signaling pathway|GO:0009607,response to biotic stimulus|GO:0048872,homeostasis of number of cells|GO:0044707,single-multicellular organism process|GO:0038123,toll-like receptor TLR1:TLR2 signaling pathway|GO:0019538,protein metabolic process|GO:0070423,nucleotide-binding oligomerization domain containing signaling pathway|GO:0034641,cellular nitrogen compound metabolic process|GO:0002376,immune system process|GO:0033554,cellular response to stress|GO:0019438,aromatic compound biosynthetic process|GO:0009893,positive regulation of metabolic process|GO:0009891,positive regulation of biosynthetic process|GO:0006950,response to stress|GO:0038061,NIK/NF-kappaB signaling|GO:0006468,protein phosphorylation|GO:0050778,positive regulation of immune response|GO:0008152,metabolic process|GO:0010628,positive regulation of gene expression|GO:0071902,positive regulation of protein serine/threonine kinase activity|GO:0035556,intracellular signal transduction|GO:0071900,regulation of protein serine/threonine kinase activity|GO:0050789,regulation of biological process|GO:0097659,nucleic acid-templated transcription|GO:0009605,response to external stimulus|GO:0001816,cytokine production|GO:0090304,nucleic acid metabolic process|GO:0051347,positive regulation of transferase activity|GO:0002764,immune response-regulating signaling pathway|GO:0044260,cellular macromolecule metabolic process|GO:0070997,neuron death|GO:0002768,immune response-regulating cell surface receptor signaling pathway|GO:0043549,regulation of kinase activity|GO:0002684,positive regulation of immune system process|GO:0002682,regulation of immune system process|GO:0031347,regulation of defense response|GO:0006366,transcription from RNA polymerase II promoter|GO:0051174,regulation of phosphorus metabolic process|GO:0065008,regulation of biological quality|GO:0018130,heterocycle biosynthetic process|GO:0016265,obsolete death|GO:0050790,regulation of catalytic activity|GO:0009889,regulation of biosynthetic process|GO:0051716,cellular response to stimulus|GO:0050794,regulation of cellular process|GO:0043410,positive regulation of MAPK cascade|GO:0012501,programmed cell death|GO:0034146,toll-like receptor 5 signaling pathway|GO:0036211,protein modification process|GO:0034142,toll-like receptor 4 signaling pathway|GO:0006955,immune response|GO:0034654,nucleobase-containing compound biosynthetic process|GO:1902533,positive regulation of intracellular signal transduction|GO:1901214,regulation of neuron death|GO:1902531,regulation of intracellular signal transduction|GO:0016070,RNA metabolic process|GO:0002755,MyD88-dependent toll-like receptor signaling pathway|GO:0002756,MyD88-independent toll-like receptor signaling pathway|GO:0002757,immune response-activating signal transduction|GO:0044271,cellular nitrogen compound biosynthetic process|GO:0038093,Fc receptor signaling pathway|GO:0002753,cytoplasmic pattern recognition receptor signaling pathway|GO:0043412,macromolecule modification|GO:0080134,regulation of response to stress|GO:0031401,positive regulation of protein modification process|GO:0002758,innate immune response-activating signal transduction|GO:0032481,positive regulation of type I interferon production|GO:0051338,regulation of transferase activity|GO:0006355,regulation of transcription, DNA-templated|GO:0010557,positive regulation of macromolecule biosynthetic process|GO:0006357,regulation of transcription from RNA polymerase II promoter|GO:0006351,transcription, DNA-templated|GO:0009615,response to virus|GO:0051240,positive regulation of multicellular organismal process|GO:0006954,inflammatory response|GO:0051239,regulation of multicellular organismal process|GO:0044699,single-organism process|GO:0051649,establishment of localization in cell|GO:0031098,stress-activated protein kinase signaling cascade|GO:0023056,positive regulation of signaling|GO:0043406,positive regulation of MAP kinase activity|GO:0043405,regulation of MAP kinase activity|GO:0023052,signaling|GO:0038124,toll-like receptor TLR6:TLR2 signaling pathway|GO:0023051,regulation of signaling|GO:0010647,positive regulation of cell communication|GO:0010646,regulation of cell communication|GO:0043085,positive regulation of catalytic activity|GO:0043408,regulation of MAPK cascade|GO:0006139,nucleobase-containing compound metabolic process|GO:0007254,JNK cascade|GO:1901215,negative regulation of neuron death|GO:0051247,positive regulation of protein metabolic process|GO:0007250,activation of NF-kappaB-inducing kinase activity|GO:0010562,positive regulation of phosphorus metabolic process|GO:0033674,positive regulation of kinase activity|GO:0051246,regulation of protein metabolic process|GO:0035872,nucleotide-binding domain, leucine rich repeat containing receptor signaling pathway|GO:0032270,positive regulation of cellular protein metabolic process|GO:0031399,regulation of protein modification process|GO:0002224,toll-like receptor signaling pathway|GO:0002220,innate immune response activating cell surface receptor signaling pathway|GO:0002221,pattern recognition receptor signaling pathway|GO:0002223,stimulatory C-type lectin receptor signaling pathway|GO:1903508,positive regulation of nucleic acid-templated transcription|GO:0032501,multicellular organismal process|GO:0050776,regulation of immune response|GO:0006725,cellular aromatic compound metabolic process|GO:1903506,regulation of nucleic acid-templated transcription|GO:0006974,cellular response to DNA damage stimulus|GO:0048518,positive regulation of biological process|GO:0034645,cellular macromolecule biosynthetic process|GO:0038095,Fc-epsilon receptor signaling pathway|GO:0045893,positive regulation of transcription, DNA-templated|GO:0016032,viral process|GO:0044764,multi-organism cellular process|GO:0002260,lymphocyte homeostasis|GO:0051092,positive regulation of NF-kappaB transcription factor activity|GO:0051091,positive regulation of sequence-specific DNA binding transcription factor activity|GO:0051090,regulation of sequence-specific DNA binding transcription factor activity|GO:0051252,regulation of RNA metabolic process|GO:0007249,I-kappaB kinase/NF-kappaB signaling|GO:0051254,positive regulation of RNA metabolic process|GO:0009059,macromolecule biosynthetic process|GO:0043170,macromolecule metabolic process|GO:1902680,positive regulation of RNA biosynthetic process|GO:0001817,regulation of cytokine production|GO:0006807,nitrogen compound metabolic process|GO:0045944,positive regulation of transcription from RNA polymerase II promoter|GO:0032479,regulation of type I interferon production|GO:0045860,positive regulation of protein kinase activity|GO:0050896,response to stimulus|GO:0001819,positive	GO:0043229,intracellular organelle|GO:0043228,non-membrane-bounded organelle|GO:0043227,membrane-bounded organelle|GO:0043226,organelle|GO:0005856,cytoskeleton|GO:0072686,mitotic spindle|GO:1902911,protein kinase complex|GO:0005634,nucleus|GO:0000922,spindle pole|GO:0044430,cytoskeletal part|GO:0008385,IkappaB kinase complex|GO:0005737,cytoplasm|GO:0015630,microtubule cytoskeleton|GO:1902494,catalytic complex|GO:0061695,transferase complex, transferring phosphorus-containing groups|GO:1902554,serine/threonine protein kinase complex|GO:0005819,spindle|GO:1990234,transferase complex|GO:0043234,protein complex|GO:0032991,macromolecular complex|GO:0043231,intracellular membrane-bounded organelle|GO:0043232,intracellular non-membrane-bounded organelle|GO:0005829,cytosol|GO:0044464,cell part|GO:0000151,ubiquitin ligase complex|GO:0005623,cell|GO:0005622,intracellular|GO:0044446,intracellular organelle part|GO:0044444,cytoplasmic part|GO:0044445,cytosolic part|GO:0044424,intracellular part|GO:0044422,organelle part	GO:1990450,linear polyubiquitin binding|GO:0044389,ubiquitin-like protein ligase binding|GO:0019899,enzyme binding|GO:0060089,molecular transducer activity|GO:0005488,binding|GO:0004871,signal transducer activity|GO:0031593,polyubiquitin binding|GO:0043169,cation binding|GO:0043167,ion binding|GO:0070530,K63-linked polyubiquitin binding|GO:0046872,metal ion binding|GO:0042802,identical protein binding|GO:0042803,protein homodimerization activity|GO:0019904,protein domain specific binding|GO:0005102,receptor binding|GO:0031625,ubiquitin protein ligase binding|GO:0032182,ubiquitin-like protein binding|GO:0043130,ubiquitin binding|GO:0005515,protein binding|GO:0042975,peroxisome proliferator activated receptor binding|GO:0046983,protein dimerization activity|GO:0046982,protein heterodimerization activity

<table>
<tr><td>CYP4A22</td><td>GO:0008152,metabolic process|GO:0044710,single-organism metabolic process|GO:0044699,single-organism process|GO:0055114,oxidation-reduction process|GO:0044238,primary metabolic process|GO:0006629,lipid metabolic process|GO:0009987,cellular process|GO:0044237,cellular metabolic process|GO:0071704,organic substance metabolic process|GO:0044763,single-organism cellular process|GO:0044255,cellular lipid metabolic process|GO:0030258,lipid modification|GO:0002933,lipid hydroxylation</td><td>GO:0005783,endoplasmic reticulum|GO:0005789,endoplasmic reticulum membrane|GO:0042175,nuclear outer membrane-endoplasmic reticulum membrane network|GO:0043229,intracellular organelle|GO:0043227,membrane-bounded organelle|GO:0043226,organelle|GO:0005737,cytoplasm|GO:0044446,intracellular organelle part|GO:0031090,organelle membrane|GO:0016020,membrane|GO:0044432,endoplasmic reticulum part|GO:0098588,bounding membrane of organelle|GO:0012505,endomembrane system|GO:0043231,intracellular membrane-bounded organelle|GO:0044464,cell part|GO:0005623,cell|GO:0005622,intracellular|GO:0044444,cytoplasmic part|GO:0044424,intracellular part|GO:0044425,membrane part|GO:0044422,organelle part|GO:0031984,organelle subcompartment|GO:0098827,endoplasmic reticulum subcompartment|GO:0005576,extracellular</td><td>GO:0005488,binding|GO:0043169,cation binding|GO:0004497,monooxygenase activity|GO:0046914,transition metal ion binding|GO:0020037,heme binding|GO:0046906,tetrapyrrole binding|GO:0097159,organic cyclic compound binding|GO:0016705,oxidoreductase activity, acting on paired donors, with incorporation or reduction of molecular oxygen|GO:0016713,oxidoreductase activity, acting on paired donors, with incorporation or reduction of molecular oxygen, reduced iron-sulfur protein as one donor, and incorporation of one atom of oxygen|GO:0043167,ion binding|GO:0003824,catalytic activity|GO:0018685,alkane 1-monooxygenase activity|GO:0052869,arachidonic acid omega-hydroxylase activity|GO:0005506,iron ion binding|GO:0046872,metal ion binding|GO:1901363,heterocyclic compound binding|GO:0016491,oxidoreductase activity</td></tr>
<tr><td>ABHD1</td><td>GO:0008152,metabolic process|GO:0044238,primary metabolic process|GO:0044710,single-organism metabolic process|GO:0006629,lipid metabolic process|GO:0009987,cellular process|GO:0044237,cellular metabolic process|GO:0071704,organic substance metabolic process|GO:0044763,single-organism cellular process|GO:0044255,cellular lipid metabolic process|GO:0044699,single-organism process</td><td>GO:0044425,membrane part|GO:0016021,integral component of membrane|GO:0016020,membrane|GO:0031224,intrinsic component of membrane</td><td>GO:0016787,hydrolase activity|GO:0052689,carboxylic ester hydrolase activity|GO:0016788,hydrolase activity, acting on ester bonds|GO:0003824,catalytic activity|GO:0005488,binding|GO:0005515,protein binding|GO:0016298,lipase activity</td></tr>
<tr><td>NDUFB4</td><td>GO:0050896,response to stimulus|GO:0009141,nucleoside triphosphate metabolic process|GO:0009144,purine nucleoside triphosphate metabolic process|GO:0009167,purine ribonucleoside monophosphate metabolic process|GO:0034641,cellular nitrogen compound metabolic process|GO:0006807,nitrogen compound metabolic process|GO:0044281,small molecule metabolic process|GO:0009161,ribonucleoside monophosphate metabolic process|GO:0009123,nucleoside monophosphate metabolic process|GO:0006091,generation of precursor metabolites and energy|GO:0009126,purine nucleoside monophosphate metabolic process|GO:0009259,ribonucleotide metabolic process|GO:0044699,single-organism process|GO:0006139,nucleobase-containing compound metabolic process|GO:0044710,single-organism metabolic process|GO:0042775,mitochondrial ATP synthesis coupled electron transport|GO:1901360,organic cyclic compound metabolic process|GO:0042278,purine nucleoside metabolic process|GO:0006979,response to oxidative stress|GO:0071704,organic substance metabolic process|GO:0009199,ribonucleoside triphosphate metabolic process|GO:0022900,electron transport chain|GO:0055086,nucleobase-containing small molecule metabolic process|GO:0022904,respiratory electron transport chain|GO:0006119,oxidative phosphorylation|GO:0009205,purine ribonucleoside triphosphate metabolic process|GO:0046034,ATP metabolic process|GO:0009987,cellular process|GO:0006725,cellular aromatic compound metabolic process|GO:0009150,purine ribonucleotide metabolic process|GO:0072521,purine-containing compound metabolic process|GO:0006950,response to stress|GO:0045333,cellular respiration|GO:0009116,nucleoside metabolic process|GO:0008152,metabolic process|GO:0009119,ribonucleoside metabolic process|GO:0046128,purine ribonucleoside metabolic process|GO:0042773,ATP synthesis coupled electron transport|GO:0055114,oxidation-reduction process|GO:0046483,heterocycle metabolic process|GO:0044238,primary metabolic process|GO:1901564,organonitrogen compound metabolic process|GO:0015980,energy derivation by oxidation of organic compounds|GO:0016310,phosphorylation|GO:0044763,single-organism cellular process|GO:0019693,ribose phosphate metabolic process|GO:0006120,mitochondrial electron transport, NADH to ubiquinone|GO:0006163,purine nucleotide metabolic process|GO:1901657,glycosyl compound metabolic process|GO:0006796,phosphate-containing compound metabolic process|GO:0006793,phosphorus metabolic process|GO:0019637,organophosphate metabolic process|GO:0009117,nucleotide metabolic process|GO:0044237,cellular metabolic process|GO:0006753,nucleoside phosphate metabolic process|GO:1901135,carbohydrate derivative metabolic process|GO:0022607,cellular component assembly|GO:0070271,protein complex biogenesis|GO:0043933,macromolecular complex subunit organization|GO:0034622,cellular macromolecular complex assembly|GO:0071840,cellular component organization or biogenesis|GO:0071822,protein complex subunit organization|GO:0032981,mitochondrial respiratory chain complex I assembly|GO:0010257,NADH dehydrogenase complex assembly|GO:0016043,cellular component organization|GO:0065003,macromolecular complex assembly|GO:0007005,mitochondrion organization|GO:0033108,mitochondrial respiratory chain complex assembly|GO:0006461,protein complex assembly|GO:0043623,cellular protein complex assembly|GO:0006996,organelle organization|GO:0097031,mitochondrial respiratory chain complex I biogenesis|GO:0044085,cellular component biogenesis</td><td>GO:0098796,membrane protein complex|GO:0044421,extracellular region part|GO:0044464,cell part|GO:0031974,membrane-enclosed lumen|GO:1990204,oxidoreductase complex|GO:0043229,intracellular organelle|GO:0031988,membrane-bounded vesicle|GO:0043227,membrane-bounded organelle|GO:0043226,organelle|GO:0031224,intrinsic component of membrane|GO:0005737,cytoplasm|GO:0005654,nucleoplasm|GO:0070062,extracellular exosome|GO:0031981,nuclear lumen|GO:0005743,mitochondrial inner membrane|GO:0031975,envelope|GO:0005634,nucleus|GO:0005635,nuclear envelope|GO:0098803,respiratory chain complex|GO:0044444,cytoplasmic part|GO:0005739,mitochondrion|GO:0098800,inner mitochondrial membrane protein complex|GO:0044455,mitochondrial membrane part|GO:0044428,nuclear part|GO:0044429,mitochondrial part|GO:1902494,catalytic complex|GO:0031965,nuclear membrane|GO:0031967,organelle envelope|GO:0031966,mitochondrial membrane|GO:0012505,endomembrane system|GO:0032991,macromolecular complex|GO:0016020,membrane|GO:1903561,extracellular vesicle|GO:0031982,vesicle|GO:0043234,protein complex|GO:0031090,organelle membrane|GO:0045271,respiratory chain complex I|GO:0043230,extracellular organelle|GO:0043231,intracellular membrane-bounded organelle|GO:0043233,organelle lumen|GO:0030964,NADH dehydrogenase complex|GO:0019866,organelle inner membrane|GO:0005623,cell|GO:0005622,intracellular|GO:0044446,intracellular organelle part|GO:0070013,intracellular organelle lumen|GO:0005740,mitochondrial envelope|GO:0005576,extracellular region|GO:0005746,mitochondrial respiratory chain|GO:0005747,mitochondrial respiratory chain complex I|GO:0044424,intracellular part|GO:0044425,membrane part|GO:0070469,respiratory chain|GO:0065010,extracellular membrane-bounded organelle|GO:0016021,integral component of membrane|GO:0044422,organelle part|GO:0098798,mitochondrial protein complex</td><td>GO:0003824,catalytic activity|GO:0008137,NADH dehydrogenase (ubiquinone) activity|GO:0016655,oxidoreductase activity, acting on NAD(P)H, quinone or similar compound as acceptor|GO:0050136,NADH dehydrogenase (quinone) activity|GO:0016651,oxidoreductase activity, acting on NAD(P)H|GO:0003954,NADH dehydrogenase activity|GO:0016491,oxidoreductase activity</td></tr>
<tr><td>SIRPG</td><td>GO:0042127,regulation of cell proliferation|GO:0016337,single organismal cell-cell adhesion|GO:0034112,positive regulation of homotypic cell-cell adhesion|GO:0034110,regulation of homotypic cell-cell adhesion|GO:0050865,regulation of cell activation|GO:0050867,positive regulation of cell activation|GO:0007596,blood coagulation|GO:0050863,regulation of T cell activation|GO:0006928,movement of cell or subcellular component|GO:0022407,regulation of cell-cell adhesion|GO:0050900,leukocyte migration|GO:0065007,biological regulation|GO:0023052,signaling|GO:0007165,signal transduction|GO:0007599,hemostasis|GO:0035556,intracellular signal transduction|GO:0050789,regulation of biological process|GO:0044699,single-organism process|GO:0022409,positive regulation of cell-cell adhesion|GO:0051716,cellular response to stimulus|GO:0051249,regulation of lymphocyte activation|GO:0045785,positive regulation of cell adhesion|GO:0007155,cell adhesion|GO:0009611,response to wounding|GO:1903039,positive regulation of leukocyte cell-cell adhesion|GO:0002684,positive regulation of immune system process|GO:0002682,regulation of immune system process|GO:0048518,positive regulation of biological process|GO:0048519,negative regulation of biological process|GO:0071593,lymphocyte aggregation|GO:0030155,regulation of cell adhesion|GO:1903037,regulation of leukocyte cell-cell adhesion|GO:0008284,positive regulation of cell proliferation|GO:0008285,negative regulation of cell proliferation|GO:0098602,single organism cell adhesion|GO:0050878,regulation of body fluid levels|GO:0008283,cell proliferation|GO:0098609,cell-cell adhesion|GO:0034109,homotypic cell-cell adhesion|GO:0032501,multicellular organismal process|GO:0009987,cellular process|GO:0050870,positive regulation of T cell activation|GO:0050794,regulation of cellular process|GO:0001775,cell activation|GO:0046649,lymphocyte activation|GO:0070489,T cell aggregation|GO:0042110,T cell activation|GO:0007267,cell-cell signaling|GO:0007154,cell communication|GO:0070486,leukocyte aggregation|GO:0051179,localization|GO:0045321,leukocyte activation|GO:0040011,locomotion|GO:0044700,single organism signaling|GO:0016477,cell migration|GO:0051674,localization of cell|GO:0044707,single-multicellular organism process|GO:0050817,coagulation|GO:0048870,cell motility|GO:0051251,positive regulation of lymphocyte activation|GO:0022610,biological adhesion|GO:0050896,response to stimulus|GO:0044763,single-organism cellular process|GO:0006950,response to stress|GO:0002376,immune system process|GO:0002694,regulation of leukocyte activation|GO:0065008,regulation of biological quality|GO:0002696,positive regulation of leukocyte activation|GO:0042060,wound healing|GO:0007159,leukocyte cell-cell adhesion|GO:0048523,negative regulation of cellular process|GO:0048522,positive regulation of</td><td>GO:0016021,integral component of membrane|GO:0016020,membrane|GO:0044464,cell part|GO:0005623,cell|GO:0005622,intracellular|GO:0071944,cell periphery|GO:0005886,plasma membrane|GO:0044425,membrane part|GO:0031224,intrinsic component of membrane</td><td>GO:0005488,binding|GO:0005515,protein binding</td></tr>
<tr><td>ATXN10</td><td>GO:0032502,developmental process|GO:0022607,cellular component assembly|GO:0070271,protein complex biogenesis|GO:0030030,cell projection organization|GO:0030154,cell differentiation|GO:0048468,cell development|GO:0031175,neuron projection development|GO:0007275,multicellular organism development|GO:0044699,single-organism process|GO:0071822,protein complex subunit organization|GO:0048869,cellular developmental process|GO:0051260,protein homooligomerization|GO:0016043,cellular component organization|GO:0065003,macromolecular complex assembly|GO:0043933,macromolecular complex subunit organization|GO:0071840,cellular component organization or biogenesis|GO:0048666,neuron development|GO:0032501,multicellular organismal process|GO:0070207,protein homotrimerization|GO:0070206,protein trimerization|GO:0030182,neuron differentiation|GO:0006461,protein complex assembly|GO:0044767,single-organism developmental process|GO:0044763,single-organism cellular process|GO:0022008,neurogenesis|GO:0051259,protein oligomerization|GO:0048699,generation of neurons|GO:0007399,nervous system development|GO:0044707,single-multicellular organism process|GO:0048856,anatomical structure development|GO:0044085,cellular component biogenesis|GO:0048731,system development|GO:0009987,cellular process|GO:0009653,anatomical structure morphogenesis|GO:0000902,cell morphogenesis|GO:0032989,cellular component morphogenesis|GO:0060271,cilium morphogenesis|GO:0048858,cell projection morphogenesis|GO:0032990,cell part morphogenesis</td><td>GO:0030425,dendrite|GO:0071944,cell periphery|GO:0005737,cytoplasm|GO:0005615,extracellular space|GO:0016020,membrane|GO:0097458,neuron part|GO:0043005,neuron projection|GO:0048471,perinuclear region of cytoplasm|GO:0043025,neuronal cell body|GO:0044297,cell body|GO:0036477,somatodendritic compartment|GO:0005886,plasma membrane|GO:0042995,cell projection|GO:0005829,cytosol|GO:0044464,cell part|GO:0005623,cell|GO:0005622,intracellular|GO:0044444,cytoplasmic part|GO:0005576,extracellular region|GO:0044424,intracellular part|GO:0044421,extracellular region part</td><td>GO:0005488,binding|GO:0042802,identical protein binding|GO:0019899,enzyme binding|GO:0005515,protein binding</td></tr>
<tr><td>PMCH</td><td>GO:0007631,feeding behavior|GO:0030154,cell differentiation|GO:0019953,sexual reproduction|GO:0007610,behavior|GO:0023052,signaling|GO:0007165,signal transduction|GO:0007275,multicellular organism development|GO:0007276,gamete generation|GO:0007218,neuropeptide signaling pathway|GO:0000003,reproduction|GO:0048869,cellular developmental process|GO:0050789,regulation of biological process|GO:0065007,biological regulation|GO:0044699,single-organism process|GO:0007186,G-protein coupled receptor signaling pathway|GO:0032502,developmental process|GO:0032501,multicellular organismal process|GO:0048609,multicellular organismal reproductive process|GO:0032504,multicellular organism reproduction|GO:0009987,cellular process|GO:0051716,cellular response to stimulus|GO:0050794,regulation of cellular process|GO:0044767,single-organism developmental process|GO:0022414,reproductive process|GO:0007283,spermatogenesis|GO:0044763,single-organism cellular process|GO:0007268,chemical synaptic transmission|GO:0048232,male gamete generation|GO:0007267,cell-cell signaling|GO:0007154,cell communication|GO:0051704,multi-organism process|GO:0044700,single organism signaling|GO:0044703,multi-organism reproductive process|GO:0044702,single organism reproductive process|GO:0044707,single-multicellular organism process|GO:0050896,response to stimulus|GO:0048856,anatomical structure development|GO:0098916,anterograde trans-synaptic signaling|GO:0099536,synaptic signaling|GO:0099537,trans-synaptic signaling</td><td>GO:0043231,intracellular membrane-bounded organelle|GO:0005634,nucleus|GO:0044464,cell part|GO:0005623,cell|GO:0005622,intracellular|GO:0043229,intracellular organelle|GO:0005576,extracellular region|GO:0044424,intracellular part|GO:0043227,membrane-bounded organelle|GO:0043226,organelle</td><td>GO:0005179,hormone activity|GO:0030354,melanin-concentrating hormone activity|GO:0005515,protein binding|GO:0005102,receptor binding|GO:0005488,binding</td></tr>
<tr><td>IKBIP</td><td>GO:0010165,response to X-ray|GO:0010212,response to ionizing radiation|GO:0009628,response to abiotic stimulus|GO:0009314,response to radiation|GO:0050896,response to stimulus</td><td>GO:0005783,endoplasmic reticulum|GO:0005789,endoplasmic reticulum membrane|GO:0042175,nuclear outer membrane-endoplasmic reticulum membrane network|GO:0043229,intracellular organelle|GO:0043227,membrane-bounded organelle|GO:0043226,organelle|GO:0031224,intrinsic component of membrane|GO:0005737,cytoplasm|GO:0031090,organelle membrane|GO:0016021,integral component of membrane|GO:0016020,membrane|GO:0044432,endoplasmic reticulum part|GO:0098588,bounding membrane of organelle|GO:0012505,endomembrane system|GO:0043231,intracellular membrane-bounded organelle|GO:0044464,cell part|GO:0005623,cell|GO:0005622,intracellular|GO:0044446,intracellular organelle part|GO:0044444,cytoplasmic part|GO:0044424,intracellular part|GO:0044425,membrane part|GO:0044422,organelle part|GO:0031984,organelle subcompartment|GO:0098827,endoplasmic reticulum subcompartment</td><td>GO:0005488,binding|GO:0005515,protein binding</td></tr>
<tr><td>CENPJ</td><td>GO:0022607,cellular component assembly|GO:0044839,cell cycle G2/M phase transition|GO:0033043,regulation of organelle organization|GO:0000086,G2/M transition of mitotic cell cycle|GO:0051493,regulation of cytoskeleton organization|GO:0034622,cellular macromolecular complex assembly|GO:1903047,mitotic cell cycle process|GO:0051128,regulation of cellular component organization|GO:0044770,cell cycle phase transition|GO:0010824,regulation of centrosome duplication|GO:0044772,mitotic cell cycle phase transition|GO:0022402,cell cycle process|GO:0051301,cell division|GO:0098534,centriole assembly|GO:0032886,regulation of microtubule-based process|GO:0050789,regulation of biological process|GO:0007049,cell cycle|GO:0051298,centrosome duplication|GO:0007020,microtubule nucleation|GO:0071822,protein complex subunit organization|GO:0071840,cellular component organization or biogenesis|GO:0070271,protein complex biogenesis|GO:0010564,regulation of cell cycle process|GO:0016043,cellular component organization|GO:0065003,macromolecular complex assembly|GO:0043933,macromolecular complex subunit organization|GO:0046785,microtubule polymerization|GO:0065007,biological regulation|GO:1902115,regulation of organelle assembly|GO:0046599,regulation of centriole replication|GO:0031023,microtubule organizing center organization|GO:0000278,mitotic cell cycle|GO:0051297,centrosome organization|GO:0006461,protein complex assembly|GO:0031109,microtubule polymerization or depolymerization|GO:0050794,regulation of cellular process|GO:0046605,regulation of centrosome cycle|GO:0044763,single-organism cellular process|GO:0070925,organelle assembly|GO:0043623,cellular protein complex assembly|GO:0006996,organelle organization|GO:0051258,protein polymerization|GO:0044699,single-organism process|GO:0007017,microtubule-based process|GO:0007010,cytoskeleton organization|GO:0051726,regulation of cell cycle|GO:0044087,regulation of cellular component biogenesis|GO:1902589,single-organism organelle organization|GO:0044085,cellular component biogenesis|GO:0007098,centrosome cycle|GO:0007099,centriole replication|GO:0000226,microtubule cytoskeleton organization|GO:0009987,cellular process|GO:0070507,regulation of microtubule cytoskeleton organization|GO:0000082,G1/S transition of mitotic cell cycle|GO:0060491,regulation of cell projection assembly|GO:0007165,signal transduction|GO:0007166,cell surface receptor signaling pathway|GO:1902402,signal transduction involved in mitotic DNA damage checkpoint|GO:1902403,signal transduction involved in mitotic DNA integrity checkpoint|GO:1902400,intracellular signal transduction involved in G1 DNA damage checkpoint|GO:0031344,regulation of cell projection organization|GO:0051716,cellular response to stimulus|GO:0072413,signal transduction involved in mitotic cell cycle checkpoint|GO:2000134,negative regulation of G1/S transition of mitotic cell cycle|GO:0045787,positive regulation of cell cycle|GO:0072422,signal transduction involved in DNA damage checkpoint|GO:0048869,cellular developmental process|GO:0048522,positive regulation of cellular process|GO:0048518,positive regulation of biological process|GO:0048519,negative regulation of biological process|GO:0043062,extracellular structure organization|GO:0044700,single organism signaling|GO:0010638,positive regulation of organelle organization|GO:0007154,cell communication|GO:0030198,extracellular matrix organization|GO:0033554,cellular response to stress|GO:1901991,negative regulation of mitotic cell cycle phase transition|GO:0044783,G1 DNA damage checkpoint|GO:0022604,regulation of cell morphogenesis|GO:0022603,regulation of anatomical structure morphogenesis|GO:0035556,intracellular signal transduction|GO:0072331,signal transduction by p53 class mediator|GO:0072401,signal transduction involved in DNA integrity checkpoint|GO:0090068,positive regulation of cell cycle process|GO:0007346,regulation of mitotic cell cycle|GO:0000902,cell morphogenesis|GO:0031570,DNA integrity checkpoint|GO:0031571,mitotic G1 DNA damage checkpoint|GO:0045724,positive regulation of cilium assembly|GO:1902017,regulation of cilium assembly|GO:0048646,anatomical structure formation involved in morphogenesis|GO:0051130,positive regulation of cellular component organization|GO:0042384,cilium assembly|GO:0060271,cilium morphogenesis|GO:0050793,regulation of developmental process|GO:0007050,cell cycle arrest|GO:1901987,regulation of cell cycle phase transition|GO:0006950,response to stress|GO:0045786,negative regulation of cell cycle|GO:1901988,negative regulation of cell cycle phase transition|GO:0044782,cilium organization|GO:0007224,smoothened signaling pathway|GO:0050896,response to stimulus|GO:0097435,fibril organization|GO:0010927,cellular component assembly involved in morphogenesis|GO:0023052,signaling|GO:1901990,regulation of mitotic cell cycle phase transition|GO:0009653,anatomical structure morphogenesis|GO:0044819,mitotic G1/S transition checkpoint|GO:0072395,signal transduction involved in cell cycle checkpoint|GO:1902855,regulation of nonmotile primary cilium assembly|GO:0044458,motile cilium assembly|GO:1902857,positive regulation of nonmotile primary cilium assembly|GO:0032502,developmental process|GO:0006974,cellular response to DNA damage stimulus|GO:0006977,DNA damage response, signal transduction by p53 class mediator resulting in cell cycle arrest|GO:0032990,cell part morphogenesis|GO:2000045,regulation of G1/S transition of mitotic cell cycle|GO:0072431,signal transduction involved in mitotic G1 DNA damage checkpoint|GO:0030030,cell projection organization|GO:0030031,cell projection assembly|GO:0061511,centriole elongation|GO:0010948,negative regulation of cell cycle process|GO:0044773,mitotic DNA damage checkpoint|GO:0044774,mitotic DNA integrity checkpoint|GO:1902807,negative regulation of cell cycle G1/S phase transition|GO:0032989,cellular component morphogenesis|GO:0071158,positive regulation of cell cycle arrest|GO:1902117,positive regulation of organelle assembly|GO:0071156,regulation of cell cycle arrest|GO:0045930,negative regulation of mitotic cell cycle|GO:0035058,nonmotile primary cilium assembly|GO:0030330,DNA damage response, signal transduction by p53 class mediator|GO:0044767,single-organism developmental process|GO:0044843,cell cycle G1/S phase transition|GO:0042770,signal transduction in response to DNA damage|GO:0000077,DNA damage checkpoint|GO:0048858,cell projection morphogenesis|GO:0000075,cell cycle checkpoint|GO:0048856,anatomical structure development|GO:1902806,regulation of cell cycle G1/S phase transition|GO:0044089,positive regulation of cellular</td><td>GO:0008275,gamma-tubulin small complex|GO:0043229,intracellular organelle|GO:0043228,non-membrane-bounded organelle|GO:0005623,cell|GO:0005874,microtubule|GO:0043226,organelle|GO:0005856,cytoskeleton|GO:0005813,centrosome|GO:0005814,centriole|GO:0005815,microtubule organizing center|GO:0044430,cytoskeletal part|GO:0044450,microtubule organizing center part|GO:0005737,cytoplasm|GO:0015630,microtubule cytoskeleton|GO:0043234,protein complex|GO:0032991,macromolecular complex|GO:0043232,intracellular non-membrane-bounded organelle|GO:0005829,cytosol|GO:0044464,cell part|GO:0000930,gamma-tubulin complex|GO:0005622,intracellular|GO:0044446,intracellular organelle part|GO:0044444,cytoplasmic part|GO:0044424,intracellular part|GO:0044422,organelle part|GO:0099512,supramolecular fiber|GO:0099513,polymeric cytoskeletal fiber|GO:0031974,membrane-enclosed lumen|GO:0005929,cilium|GO:0036064,ciliary basal body|GO:0031981,nuclear lumen|GO:0005634,nucleus|GO:0016020,membrane|GO:0005654,nucleoplasm|GO:0005886,plasma membrane|GO:0043231,intracellular membrane-bounded organelle|GO:0043233,organelle lumen|GO:0070013,intracellular organelle lumen|GO:0043227,membrane-bounded organelle|GO:0071944,cell periphery|GO:0044428,nuclear part|GO:0044441,ciliary part</td><td>GO:0015631,tubulin binding|GO:0008092,cytoskeletal protein binding|GO:0019899,enzyme binding|GO:0019901,protein kinase binding|GO:0019900,kinase binding|GO:0019904,protein domain specific binding|GO:0005515,protein binding|GO:0005488,binding|GO:0042802,identical protein binding</td></tr>
<tr><td>NOTCH2N</td><td>GO:0030154,cell differentiation|GO:0023052,signaling|GO:0007165,signal transduction|GO:0007166,cell surface receptor signaling pathway|GO:0007275,multicellular organism development|GO:0044699,single-organism process|GO:0051716,cellular response to stimulus|GO:0007219,Notch signaling pathway|GO:0048869,cellular developmental process|GO:0050789,regulation of biological process|GO:0065007,biological regulation|GO:0032502,developmental process|GO:0032501,multicellular organismal process|GO:0009987,cellular process|GO:0050794,regulation of cellular process|GO:0044767,single-organism developmental process|GO:0007154,cell communication|GO:0044700,single organism signaling|GO:0044707,single-multicellular organism process|GO:0050896,response to stimulus|GO:0044763,single-organism cellular process|GO:0048856,anatomical structure development</td><td>GO:0005737,cytoplasm|GO:0044464,cell part|GO:0005623,cell|GO:0005622,intracellular|GO:0005576,extracellular region|GO:0044424,intracellular part</td><td>GO:0043169,cation binding|GO:0043167,ion binding|GO:0005488,binding|GO:0005515,protein binding|GO:0046872,metal ion binding|GO:0005509,calcium ion binding</td></tr>
<tr><td>RPS28</td><td>GO:0051169,nuclear transport|GO:0051168,nuclear export|GO:0061024,membrane organization|GO:0008104,protein localization|GO:1901362,organic cyclic compound biosynthetic process|GO:1901360,organic cyclic compound metabolic process|GO:1901361,organic cyclic compound catabolic process|GO:0070727,cellular macromolecule localization|GO:0043043,peptide biosynthetic process|GO:0044419,interspecies interaction between organisms|GO:0019058,viral life cycle|GO:0033036,macromolecule localization|GO:0006364,rRNA processing|GO:0034470,ncRNA processing|GO:0051704,multi-organism process|GO:0006605,protein targeting|GO:0045184,establishment of protein localization|GO:0072657,protein localization to membrane|GO:0044403,symbiosis, encompassing mutualism through parasitism|GO:0046483,heterocycle metabolic process|GO:1901564,organonitrogen compound metabolic process|GO:1901566,organonitrogen compound biosynthetic process|GO:0019538,protein metabolic process|GO:0019439,aromatic compound catabolic process|GO:0019438,aromatic compound biosynthetic process|GO:0019080,viral gene expression|GO:0006613,cotranslational protein targeting to membrane|GO:0006612,protein targeting to membrane|GO:0006614,SRP-dependent cotranslational protein targeting to membrane|GO:0006807,nitrogen compound metabolic process|GO:0044033,multi-organism metabolic process|GO:0034660,ncRNA metabolic process|GO:0051029,rRNA transport|GO:0044267,cellular protein metabolic process|GO:1901575,organic substance catabolic process|GO:0044265,cellular macromolecule catabolic process|GO:0044260,cellular macromolecule metabolic process|GO:0006886,intracellular protein transport|GO:0042254,ribosome biogenesis|GO:0016043,cellular component organization|GO:0071840,cellular component organization or biogenesis|GO:0018130,heterocycle biosynthetic process|GO:0006810,transport|GO:0046700,heterocycle catabolic process|GO:0044802,single-organism membrane organization|GO:0008152,metabolic process|GO:0034654,nucleobase-containing compound biosynthetic process|GO:0034655,nucleobase-containing compound catabolic process|GO:0051234,establishment of localization|GO:0016070,RNA metabolic process|GO:0016071,mRNA metabolic process|GO:0016072,rRNA metabolic process|GO:0044271,cellular nitrogen compound biosynthetic process|GO:0044270,cellular nitrogen compound catabolic process|GO:0046907,intracellular transport|GO:0009058,biosynthetic process|GO:0045047,protein targeting to ER|GO:0044764,multi-organism cellular process|GO:0022411,cellular component disassembly|GO:0006518,peptide metabolic process|GO:0015931,nucleobase-containing compound transport|GO:0071166,ribonucleoprotein complex localization|GO:0032774,RNA biosynthetic process|GO:0044248,cellular catabolic process|GO:0044249,cellular biosynthetic process|GO:0034641,cellular nitrogen compound metabolic process|GO:0034645,cellular macromolecule biosynthetic process|GO:0051236,establishment of RNA localization|GO:0043241,protein complex disassembly|GO:0044699,single-organism process|GO:0006139,nucleobase-containing compound metabolic process|GO:0070972,protein localization to endoplasmic reticulum|GO:0042274,ribosomal small subunit biogenesis|GO:0051179,localization|GO:0022613,ribonucleoprotein complex biogenesis|GO:0019083,viral transcription|GO:0072599,establishment of protein localization to endoplasmic reticulum|GO:0072594,establishment of protein localization to organelle|GO:0009987,cellular process|GO:0006725,cellular aromatic compound metabolic process|GO:0050658,RNA transport|GO:0016482,cytosolic transport|GO:0043604,amide biosynthetic process|GO:0043603,cellular amide metabolic process|GO:0043170,macromolecule metabolic process|GO:0000956,nuclear-transcribed mRNA catabolic process|GO:0033365,protein localization to organelle|GO:0000184,nuclear-transcribed mRNA catabolic process, nonsense-mediated decay|GO:0043933,macromolecular complex subunit organization|GO:0050657,nucleic acid transport|GO:0090304,nucleic acid metabolic process|GO:0032984,macromolecular complex disassembly|GO:0071822,protein complex subunit organization|GO:0071705,nitrogen compound transport|GO:0071704,organic substance metabolic process|GO:0010467,gene expression|GO:0071702,organic substance transport|GO:0006401,RNA catabolic process|GO:0006402,mRNA catabolic process|GO:0006403,RNA localization|GO:0006405,RNA export from nucleus|GO:0006407,rRNA export from nucleus|GO:1901576,organic substance biosynthetic process|GO:0034613,cellular protein localization|GO:0006913,nucleocytoplasmic transport|GO:0044765,single-organism transport|GO:0009059,macromolecule biosynthetic process|GO:0044763,single-organism cellular process|GO:0051649,establishment of localization in cell|GO:0043624,cellular protein complex disassembly|GO:0009056,catabolic process|GO:0009057,macromolecule catabolic process|GO:1902578,single-organism localization|GO:0051641,cellular localization|GO:0044238,primary metabolic process|GO:1902580,single-organism cellular localization|GO:0071426,ribonucleoprotein complex export from nucleus|GO:0071428,rRNA-containing ribonucleoprotein complex export from nucleus|GO:0090150,establishment of protein localization to membrane|GO:0044237,cellular metabolic process|GO:0044085,cellular component biogenesis|GO:0016032,viral process|GO:0015031,protein transport|GO:1902582,single-organism intracellular transport|GO:0006415,translational termination|GO:0006414,translational elongation|GO:0006413,translational initiation|GO:0006412,translation|GO:0006396,RNA processing|GO:0019222,regulation of metabolic process|GO:0010605,negative regulation of macromolecule metabolic process|GO:0048519,negative regulation of biological process|GO:0060255,regulation of macromolecule metabolic process|GO:0015833,peptide transport|GO:0022607,cellular component assembly|GO:0009892,negative regulation of metabolic process|GO:0000028,ribosomal small subunit assembly|GO:0010629,negative regulation of gene expression|GO:0050789,regulation of biological process|GO:0042255,ribosome assembly|GO:0065003,macromolecular complex assembly|GO:0065007,biological regulation|GO:0042886,amide transport|GO:0022618,ribonucleoprotein complex assembly|GO:0030490,maturation of SSU-rRNA|GO:0034622,cellular macromolecular complex assembly|GO:0071826,ribonucleoprotein complex subunit organization|GO:0010468,regulation of gene expression|GO:0070925,organelle assembly|GO:0006996,organelle organization|GO:0002181,cytoplasmic translation</td><td>GO:0022626,cytosolic ribosome|GO:0022627,cytosolic small ribosomal subunit|GO:0043229,intracellular organelle|GO:0043228,non-membrane-bounded organelle|GO:0030529,intracellular ribonucleoprotein complex|GO:0043227,membrane-bounded organelle|GO:0043226,organelle|GO:0015935,small ribosomal subunit|GO:0005737,cytoplasm|GO:0031982,vesicle|GO:0031988,membrane-bounded vesicle|GO:0005576,extracellular region|GO:0044391,ribosomal subunit|GO:0032991,macromolecular complex|GO:1903561,extracellular vesicle|GO:0070062,extracellular exosome|GO:0043230,extracellular organelle|GO:0005840,ribosome|GO:0043232,intracellular non-membrane-bounded organelle|GO:0005829,cytosol|GO:0044464,cell part|GO:0005623,cell|GO:0005622,intracellular|GO:0044446,intracellular organelle part|GO:0044444,cytoplasmic part|GO:0044445,cytosolic part|GO:0044424,intracellular part|GO:0065010,extracellular membrane-bounded organelle|GO:0044421,extracellular region part|GO:0044422,organelle part|GO:0031974,membrane-enclosed lumen|GO:0031981,nuclear lumen|GO:0005634,nucleus|GO:0005654,nucleoplasm|GO:0043231,intracellular membrane-bounded organelle|GO:0043233,organelle lumen|GO:1990904,ribonucleoprotein complex|GO:0070013,intracellular organelle lumen|GO:0044428,nuclear part|GO:0005844,polysome|GO:0031984,organelle subcompartment|GO:0005791,rough endoplasmic reticulum|GO:0005783,endoplasmic reticulum|GO:0044432,endoplasmic reticulum part|GO:0098588,bounding membrane of organelle|GO:0016020,membrane|GO:0030867,rough endoplasmic reticulum membrane|GO:0005789,endoplasmic reticulum membrane|GO:0098554,cytoplasmic side of endoplasmic reticulum membrane|GO:0098827,endoplasmic reticulum subcompartment|GO:0098556,cytoplasmic side of rough endoplasmic reticulum membrane|GO:0012505,endomembrane system|GO:0098552,side of membrane|GO:0042175,nuclear outer membrane-endoplasmic reticulum membrane network|GO:0044425,membrane part|GO:0042788,polysomal ribosome|GO:0098562,cytoplasmic side of membrane|GO:0031090,organelle membrane</td><td>GO:0005515,protein binding|GO:0097159,organic cyclic compound binding|GO:0044822,poly(A) RNA binding|GO:0005198,structural molecule activity|GO:0003735,structural constituent of ribosome|GO:0003723,RNA binding|GO:0003676,nucleic acid binding|GO:1901363,heterocyclic compound binding|GO:0005488,binding</td></tr>
<tr><td>GABARAP</td><td>GO:0008104,protein localization|GO:0080090,regulation of primary metabolic process|GO:0019222,regulation of metabolic process|GO:0061025,membrane fusion|GO:0061024,membrane organization|GO:0007005,mitochondrion organization|GO:0044712,single-organism catabolic process|GO:0051716,cellular response to stimulus|GO:0010605,negative regulation of macromolecule metabolic process|GO:1901799,negative regulation of proteasomal protein catabolic process|GO:0048518,positive regulation of biological process|GO:0048519,negative regulation of biological process|GO:0033036,macromolecule localization|GO:0022607,cellular component assembly|GO:0060255,regulation of macromolecule metabolic process|GO:0032781,positive regulation of ATPase activity|GO:0032269,negative regulation of cellular protein metabolic process|GO:0045184,establishment of protein localization|GO:0030162,regulation of proteolysis|GO:0030163,protein catabolic process|GO:0031668,cellular response to extracellular stimulus|GO:0031669,cellular response to nutrient levels|GO:0016192,vesicle-mediated transport|GO:0009605,response to external stimulus|GO:0019538,protein metabolic process|GO:0031667,response to nutrient levels|GO:0033554,cellular response to stress|GO:0009894,regulation of catabolic process|GO:0009895,negative regulation of catabolic process|GO:0009892,negative regulation of metabolic process|GO:0009893,positive regulation of metabolic process|GO:1903008,organelle disassembly|GO:0051179,localization|GO:0050789,regulation of biological process|GO:0044267,cellular protein metabolic process|GO:1901575,organic substance catabolic process|GO:0044265,cellular macromolecule catabolic process|GO:0051345,positive regulation of hydrolase activity|GO:1903363,negative regulation of cellular protein catabolic process|GO:0016043,cellular component organization|GO:0065007,biological regulation|GO:0071840,cellular component organization or biogenesis|GO:0065009,regulation of molecular function|GO:0006810,transport|GO:0050790,regulation of catalytic activity|GO:0044710,single-organism metabolic process|GO:0050794,regulation of cellular process|GO:0044804,nucleophagy|GO:0009267,cellular response to starvation|GO:0051234,establishment of localization|GO:0051336,regulation of hydrolase activity|GO:0051603,proteolysis involved in cellular protein catabolic process|GO:0006891,intra-Golgi vesicle-mediated transport|GO:0046907,intracellular transport|GO:0050896,response to stimulus|GO:0010498,proteasomal protein catabolic process|GO:0006950,response to stress|GO:0022411,cellular component disassembly|GO:0008152,metabolic process|GO:0044248,cellular catabolic process|GO:0042176,regulation of protein catabolic process|GO:0042177,negative regulation of protein catabolic process|GO:0043085,positive regulation of catalytic activity|GO:0051248,negative regulation of protein metabolic process|GO:0043562,cellular response to nitrogen levels|GO:0051246,regulation of protein metabolic process|GO:0000045,autophagosome assembly|GO:0006508,proteolysis|GO:0071496,cellular response to external stimulus|GO:0044093,positive regulation of molecular function|GO:0031330,negative regulation of cellular catabolic process|GO:0016236,macroautophagy|GO:1903050,regulation of proteolysis involved in cellular protein catabolic process|GO:0009987,cellular process|GO:0044257,cellular protein catabolic process|GO:0007033,vacuole organization|GO:0044699,single-organism process|GO:0032268,regulation of cellular protein metabolic process|GO:0000422,mitophagy|GO:0043170,macromolecule metabolic process|GO:0045861,negative regulation of proteolysis|GO:0009991,response to extracellular stimulus|GO:0031329,regulation of cellular catabolic process|GO:0031324,negative regulation of cellular metabolic process|GO:0031323,regulation of cellular metabolic process|GO:0042594,response to starvation|GO:0043462,regulation of ATPase activity|GO:0071704,organic substance metabolic process|GO:0071702,organic substance transport|GO:1903051,negative regulation of proteolysis involved in cellular protein catabolic process|GO:1903362,regulation of cellular protein catabolic process|GO:0048193,Golgi vesicle transport|GO:0061726,mitochondrion disassembly|GO:0061136,regulation of proteasomal protein catabolic process|GO:0006914,autophagy|GO:0044765,single-organism transport|GO:0044763,single-organism cellular process|GO:0051649,establishment of localization in cell|GO:0007154,cell communication|GO:0070925,organelle assembly|GO:0009056,catabolic process|GO:0009057,macromolecule catabolic process|GO:1902578,single-organism localization|GO:0051641,cellular localization|GO:0006996,organelle organization|GO:0044238,primary metabolic process|GO:0006995,cellular response to nitrogen starvation|GO:0044260,cellular macromolecule metabolic process|GO:0044237,cellular metabolic process|GO:1902589,single-organism organelle organization|GO:0044085,cellular component biogenesis|GO:0015031,protein transport|GO:1902582,single-organism intracellular transport|GO:0048523,negative regulation of cellular process|GO:0097576,vacuole fusion|GO:1901564,organonitrogen compound metabolic process|GO:1901565,organonitrogen compound catabolic process|GO:0015833,peptide transport|GO:0006807,nitrogen compound metabolic process|GO:0042886,amide transport|GO:1905037,autophagosome organization|GO:0071705,nitrogen compound transport|GO:0097352,autophagosome maturation|GO:0048284,organelle fusion</td><td>GO:0019898,extrinsic component of membrane|GO:0043229,intracellular organelle|GO:0005622,intracellular|GO:0043227,membrane-bounded organelle|GO:0043226,organelle|GO:0005737,cytoplasm|GO:0000407,pre-autophagosomal structure|GO:0031090,organelle membrane|GO:0005773,vacuole|GO:0031410,cytoplasmic vesicle|GO:0098805,whole membrane|GO:0016020,membrane|GO:0005776,autophagosome|GO:0005774,vacuolar membrane|GO:0044431,Golgi apparatus part|GO:0044437,vacuolar part|GO:0005794,Golgi apparatus|GO:0098588,bounding membrane of organelle|GO:0031982,vesicle|GO:0012505,endomembrane system|GO:0000421,autophagosome membrane|GO:0043231,intracellular membrane-bounded organelle|GO:0005829,cytosol|GO:0044464,cell part|GO:0005623,cell|GO:0000139,Golgi membrane|GO:0044446,intracellular organelle part|GO:0044444,cytoplasmic part|GO:0044424,intracellular part|GO:0044425,membrane part|GO:0044422,organelle part|GO:0031984,organelle subcompartment|GO:0097708,intracellular vesicle|GO:0098791,Golgi subcompartment</td><td>GO:0015631,tubulin binding|GO:0008092,cytoskeletal protein binding|GO:0051117,ATPase binding|GO:0008017,microtubule binding|GO:0019899,enzyme binding|GO:0048487,beta-tubulin binding|GO:0044877,macromolecular complex binding|GO:0050811,GABA receptor binding|GO:0005515,protein binding|GO:0032403,protein complex binding|GO:0005102,receptor binding|GO:0000149,SNARE binding|GO:0005488,binding|GO:0031625,ubiquitin protein ligase binding|GO:0044389,ubiquitin-like protein ligase binding</td></tr>
</table>

<table>
<tr><th></th><th></th><th></th><th></th></tr>
<tr><td>RER1</td><td>GO:0050808,synapse organization|GO:0051234,establishment of localization|GO:0060341,regulation of cellular localization|GO:0051649,establishment of localization in cell|GO:0051128,regulation of cellular component organization|GO:1903827,regulation of cellular protein localization|GO:0061024,membrane organization|GO:0007009,plasma membrane organization|GO:0008104,protein localization|GO:0072657,protein localization to membrane|GO:0090004,positive regulation of establishment of protein localization to plasma membrane|GO:0090003,regulation of establishment of protein localization to plasma membrane|GO:0050789,regulation of biological process|GO:1903829,positive regulation of cellular protein localization|GO:0032880,regulation of protein localization|GO:0032879,regulation of localization|GO:0070727,cellular macromolecule localization|GO:0001941,postsynaptic membrane organization|GO:0071840,cellular component organization or biogenesis|GO:0010256,endomembrane system organization|GO:0016043,cellular component organization|GO:0090002,establishment of protein localization to plasma membrane|GO:0044765,single-organism transport|GO:0065007,biological regulation|GO:0044699,single-organism process|GO:0048518,positive regulation of biological process|GO:0033036,macromolecule localization|GO:0034613,cellular protein localization|GO:0051130,positive regulation of cellular component organization|GO:0070201,regulation of establishment of protein localization|GO:0006810,transport|GO:1903078,positive regulation of protein localization to plasma membrane|GO:0048193,Golgi vesicle transport|GO:1990778,protein localization to cell periphery|GO:1903076,regulation of protein localization to plasma membrane|GO:0009987,cellular process|GO:1904377,positive regulation of protein localization to cell periphery|GO:0050794,regulation of cellular process|GO:0007528,neuromuscular junction development|GO:0072659,protein localization to plasma membrane|GO:0044802,single-organism membrane organization|GO:1903729,regulation of plasma membrane organization|GO:1902582,single-organism intracellular transport|GO:1904375,regulation of protein localization to cell periphery|GO:0051179,localization|GO:1902578,single-organism localization|GO:0051641,cellular localization|GO:0045184,establishment of protein localization|GO:0006890,retrograde vesicle-mediated transport, Golgi to ER|GO:0016192,vesicle-mediated transport|GO:0046907,intracellular transport|GO:0090150,establishment of protein localization to membrane|GO:0071340,skeletal muscle acetylcholine-gated channel clustering|GO:0044763,single-organism cellular process|GO:0043113,receptor clustering|GO:1902580,single-organism cellular localization|GO:0048522,positive regulation of cellular process|GO:1904951,positive regulation of establishment of protein localization|GO:0051668,localization within membrane</td><td>GO:0043229,intracellular organelle|GO:0031301,integral component of organelle membrane|GO:0031300,intrinsic component of organelle membrane|GO:0030173,integral component of Golgi membrane|GO:0005622,intracellular|GO:0043227,membrane-bounded organelle|GO:0043226,organelle|GO:0031224,intrinsic component of membrane|GO:0005737,cytoplasm|GO:0031090,organelle membrane|GO:0016021,integral component of membrane|GO:0016020,membrane|GO:0044431,Golgi apparatus part|GO:0009986,cell surface|GO:0005794,Golgi apparatus|GO:0005793,endoplasmic reticulum-Golgi intermediate compartment|GO:0098588,bounding membrane of organelle|GO:0012505,endomembrane system|GO:0043231,intracellular membrane-bounded organelle|GO:0044464,cell part|GO:0031228,intrinsic component of Golgi membrane|GO:0005623,cell|GO:0000139,Golgi membrane|GO:0044446,intracellular organelle part|GO:0044444,cytoplasmic part|GO:0044424,intracellular part|GO:0044425,membrane part|GO:0044422,organelle part|GO:0071944,cell periphery|GO:0031984,organelle subcompartment|GO:0005886,plasma</td><td>GO:0005102,receptor binding|GO:0033130,acetylcholine receptor binding|GO:0005488,binding|GO:0005515,protein binding</td></tr>
<tr><td>KRTAP20</td><td></td><td></td><td></td></tr>
<tr><td>LIN54</td><td>GO:0080090,regulation of primary metabolic process|GO:0019222,regulation of metabolic process|GO:0000086,G2/M transition of mitotic cell cycle|GO:0031326,regulation of cellular biosynthetic process|GO:0031323,regulation of cellular metabolic process|GO:1903047,mitotic cell cycle process|GO:0044770,cell cycle phase transition|GO:0044249,cellular biosynthetic process|GO:0044772,mitotic cell cycle phase transition|GO:0006807,nitrogen compound metabolic process|GO:0022402,cell cycle process|GO:0034645,cellular macromolecule biosynthetic process|GO:1901576,organic substance biosynthetic process|GO:1901362,organic cyclic compound biosynthetic process|GO:0050789,regulation of biological process|GO:0007049,cell cycle|GO:0032774,RNA biosynthetic process|GO:0006139,nucleobase-containing compound metabolic process|GO:0044839,cell cycle G2/M phase transition|GO:0044260,cellular macromolecule metabolic process|GO:0071704,organic substance metabolic process|GO:1901360,organic cyclic compound metabolic process|GO:2000112,regulation of cellular macromolecule biosynthetic process|GO:0097659,nucleic acid-templated transcription|GO:0060255,regulation of macromolecule metabolic process|GO:0010467,gene expression|GO:0065007,biological regulation|GO:0044699,single-organism process|GO:0010468,regulation of gene expression|GO:0018130,heterocycle biosynthetic process|GO:0000278,mitotic cell cycle|GO:0044238,primary metabolic process|GO:0019219,regulation of nucleobase-containing compound metabolic process|GO:0006725,cellular aromatic compound metabolic process|GO:0009987,cellular process|GO:0009889,regulation of biosynthetic process|GO:1903506,regulation of nucleic acid-templated transcription|GO:0050794,regulation of cellular process|GO:0009058,biosynthetic process|GO:0009059,macromolecule biosynthetic process|GO:0044763,single-organism cellular process|GO:0051171,regulation of nitrogen compound metabolic process|GO:0008152,metabolic process|GO:2001141,regulation of RNA biosynthetic process|GO:0034654,nucleobase-containing compound biosynthetic process|GO:0046483,heterocycle metabolic process|GO:0016070,RNA metabolic process|GO:0090304,nucleic acid metabolic process|GO:0044271,cellular nitrogen compound biosynthetic process|GO:0051726,regulation of cell cycle|GO:0051252,regulation of RNA metabolic process|GO:0034641,cellular nitrogen compound metabolic process|GO:0044237,cellular metabolic process|GO:0043170,macromolecule metabolic process|GO:0006355,regulation of transcription, DNA-templated|GO:0010556,regulation of macromolecule biosynthetic process|GO:0006351,transcription, DNA-</td><td>GO:0044446,intracellular organelle part|GO:0043231,intracellular membrane-bounded organelle|GO:0031981,nuclear lumen|GO:0043233,organelle lumen|GO:0005634,nucleus|GO:0044464,cell part|GO:0031974,membrane-enclosed lumen|GO:0005622,intracellular|GO:0005654,nucleoplasm|GO:0070013,intracellular organelle lumen|GO:0043229,intracellular organelle|GO:0044428,nuclear part|GO:0005623,cell|GO:0044424,intracellular part|GO:0043227,membrane-bounded organelle|GO:0043226,organelle|GO:0044422,organelle part</td><td>GO:0003676,nucleic acid binding|GO:0097159,organic cyclic compound binding|GO:0005488,binding|GO:0005515,protein binding|GO:0003677,DNA binding|GO:1901363,heterocyclic compound binding|GO:0043169,cation binding|GO:0046872,metal ion binding|GO:0043167,ion binding</td></tr>
<tr><td>RNF146</td><td>GO:0044238,primary metabolic process|GO:0090263,positive regulation of canonical Wnt signaling pathway|GO:0048584,positive regulation of response to stimulus|GO:0048583,regulation of response to stimulus|GO:0023056,positive regulation of signaling|GO:0030111,regulation of Wnt signaling pathway|GO:0023052,signaling|GO:0007165,signal transduction|GO:0007166,cell surface receptor signaling pathway|GO:0023051,regulation of signaling|GO:0010647,positive regulation of cell communication|GO:0010646,regulation of cell communication|GO:0050789,regulation of biological process|GO:0044699,single-organism process|GO:0044267,cellular protein metabolic process|GO:0051716,cellular response to stimulus|GO:0070936,protein K48-linked ubiquitination|GO:0044260,cellular macromolecule metabolic process|GO:0071704,organic substance metabolic process|GO:0009966,regulation of signal transduction|GO:0009967,positive regulation of signal transduction|GO:0070647,protein modification by small protein conjugation or removal|GO:0000209,protein polyubiquitination|GO:0032446,protein modification by small protein conjugation|GO:0006511,ubiquitin-dependent protein catabolic process|GO:0065007,biological regulation|GO:0036211,protein modification process|GO:0048518,positive regulation of biological process|GO:0042787,protein ubiquitination involved in ubiquitin-dependent protein catabolic process|GO:0043632,modification-dependent macromolecule catabolic process|GO:0051603,proteolysis involved in cellular protein catabolic process|GO:0009987,cellular process|GO:0019941,modification-dependent protein catabolic process|GO:1901575,organic substance catabolic process|GO:0006464,cellular protein modification process|GO:0050794,regulation of cellular process|GO:0060070,canonical Wnt signaling pathway|GO:0043412,macromolecule modification|GO:0044265,cellular macromolecule catabolic process|GO:0044763,single-organism cellular process|GO:0030163,protein catabolic process|GO:0008152,metabolic process|GO:0006508,proteolysis|GO:0044257,cellular protein catabolic process|GO:0007154,cell communication|GO:0009056,catabolic process|GO:0009057,macromolecule catabolic process|GO:0044248,cellular catabolic process|GO:0044700,single organism signaling|GO:0019538,protein metabolic process|GO:0050896,response to stimulus|GO:0016055,Wnt signaling pathway|GO:0030177,positive regulation of Wnt signaling pathway|GO:0044237,cellular metabolic process|GO:0043170,macromolecule metabolic process|GO:0016567,protein ubiquitination|GO:0051865,protein autoubiquitination|GO:0060828,regulation of canonical Wnt signaling pathway|GO:0048522,positive regulation of cellular process|GO:0006807,nitrogen compound metabolic process|GO:1905114,cell surface receptor signaling pathway involved in cell-cell signaling|GO:0198738,cell-cell signaling by wnt|GO:0070646,protein modification by small protein removal|GO:0016579,protein deubiquitination|GO:0007267,cell-cell signaling|GO:1901564,organonitrogen compound metabolic process|GO:1901565,organonitrogen compound catabolic process</td><td>GO:0005737,cytoplasm|GO:0043231,intracellular membrane-bounded organelle|GO:0005829,cytosol|GO:0044464,cell part|GO:0044444,cytoplasmic part|GO:0005623,cell|GO:0043226,organelle|GO:0043229,intracellular organelle|GO:0044424,intracellular part|GO:0005622,intracellular|GO:0043227,membrane-bounded organelle|GO:0005634,nucleus|GO:0031974,membrane-enclosed lumen|GO:0071944,cell periphery|GO:0031981,nuclear lumen|GO:0016020,membrane|GO:0005654,nucleoplasm|GO:0005886,plasma membrane|GO:0043233,organelle lumen|GO:0044446,intracellular organelle part|GO:0070013,intracellular organelle lumen|GO:0044428,nuclear part|GO:0044422,organelle part</td><td>GO:0019787,ubiquitin-like protein transferase activity|GO:0043169,cation binding|GO:0046914,transition metal ion binding|GO:0072572,poly-ADP-D-ribose binding|GO:0097367,carbohydrate derivative binding|GO:0016874,ligase activity|GO:0061630,ubiquitin protein ligase activity|GO:0043167,ion binding|GO:0003824,catalytic activity|GO:0016740,transferase activity|GO:0008270,zinc ion binding|GO:0005515,protein binding|GO:0005488,binding|GO:0046872,metal ion binding|GO:0004842,ubiquitin-protein transferase activity|GO:0061659,ubiquitin-like protein ligase activity</td></tr>
<tr><td>IL17RD</td><td>GO:0044700,single organism signaling|GO:0051716,cellular response to stimulus|GO:0019221,cytokine-mediated signaling pathway|GO:0071345,cellular response to cytokine stimulus|GO:0050896,response to stimulus|GO:0009987,cellular process|GO:0044763,single-organism cellular process|GO:0010033,response to organic substance|GO:0050794,regulation of cellular process|GO:0071310,cellular response to organic substance|GO:0065007,biological regulation|GO:0023052,signaling|GO:0007165,signal transduction|GO:0007166,cell surface receptor signaling pathway|GO:0042221,response to chemical|GO:0007154,cell communication|GO:0070887,cellular response to chemical stimulus|GO:0050789,regulation of biological process|GO:0044699,single-organism process|GO:0034097,response to cytokine|GO:0016310,phosphorylation|GO:0006807,nitrogen compound metabolic process|GO:0035556,intracellular signal transduction|GO:0044267,cellular protein metabolic process|GO:0044710,single-organism metabolic process|GO:0044260,cellular macromolecule metabolic process|GO:0000165,MAPK cascade|GO:0071704,organic substance metabolic process|GO:0023014,signal transduction by protein phosphorylation|GO:0006468,protein phosphorylation|GO:0044238,primary metabolic process|GO:0006464,cellular protein modification process|GO:0043412,macromolecule modification|GO:0036211,protein modification process|GO:0008152,metabolic process|GO:1901564,organonitrogen compound metabolic process|GO:0044237,cellular metabolic process|GO:0043170,macromolecule metabolic process|GO:0019538,protein metabolic process|GO:0006796,phosphate-containing compound metabolic process|GO:0006793,phosphorus metabolic process</td><td>GO:0031974,membrane-enclosed lumen|GO:0043229,intracellular organelle|GO:0071944,cell periphery|GO:0005622,intracellular|GO:0031226,intrinsic component of plasma membrane|GO:0043226,organelle|GO:0031224,intrinsic component of membrane|GO:0005737,cytoplasm|GO:0031090,organelle membrane|GO:0031981,nuclear lumen|GO:0043227,membrane-bounded organelle|GO:0005634,nucleus|GO:0016020,membrane|GO:0005654,nucleoplasm|GO:0044431,Golgi apparatus part|GO:0044459,plasma membrane part|GO:0005794,Golgi apparatus|GO:0098588,bounding membrane of organelle|GO:0012505,endomembrane system|GO:0005887,integral component of plasma membrane|GO:0005886,plasma membrane|GO:0043231,intracellular membrane-bounded organelle|GO:0043233,organelle lumen|GO:0044464,cell part|GO:0005623,cell|GO:0000139,Golgi membrane|GO:0044446,intracellular organelle part|GO:0070013,intracellular organelle lumen|GO:0044444,cytoplasmic part|GO:0044428,nuclear part|GO:0044424,intracellular part|GO:0044425,membrane part|GO:0016021,integral component of membrane|GO:0044422,organelle part|GO:0031984,organelle subcompartment|GO:0098791,Golgi subcompartment</td><td>GO:0038023,signaling receptor activity|GO:0060089,molecular transducer activity|GO:0004888,transmembrane signaling receptor activity|GO:0004872,receptor activity|GO:0004871,signal transducer activity|GO:0030368,interleukin-17 receptor activity|GO:0004896,cytokine receptor activity|GO:0099600,transmembrane receptor activity</td></tr>
<tr><td>TWISTNB</td><td>GO:0009892,negative regulation of metabolic process|GO:0032774,RNA biosynthetic process|GO:0019222,regulation of metabolic process|GO:0090304,nucleic acid metabolic process|GO:0044249,cellular biosynthetic process|GO:0010629,negative regulation of gene expression|GO:0006807,nitrogen compound metabolic process|GO:0034645,cellular macromolecule biosynthetic process|GO:0010467,gene expression|GO:1901362,organic cyclic compound biosynthetic process|GO:0050789,regulation of biological process|GO:0097659,nucleic acid-templated transcription|GO:0006139,nucleobase-containing compound metabolic process|GO:0010605,negative regulation of macromolecule metabolic process|GO:0044260,cellular macromolecule metabolic process|GO:0071704,organic substance metabolic process|GO:0040029,regulation of gene expression, epigenetic|GO:0006361,transcription initiation from RNA polymerase I promoter|GO:0006360,transcription from RNA polymerase I promoter|GO:0006363,termination of RNA polymerase I transcription|GO:0006362,transcription elongation from RNA polymerase I promoter|GO:0065007,biological regulation|GO:1901360,organic cyclic compound metabolic process|GO:0048519,negative regulation of biological process|GO:0045814,negative regulation of gene expression, epigenetic|GO:0010468,regulation of gene expression|GO:0018130,heterocycle biosynthetic process|GO:1901576,organic substance biosynthetic process|GO:0060255,regulation of macromolecule metabolic process|GO:0009987,cellular process|GO:0006725,cellular aromatic compound metabolic process|GO:0009058,biosynthetic process|GO:0009059,macromolecule biosynthetic process|GO:0008152,metabolic process|GO:0034654,nucleobase-containing compound biosynthetic process|GO:0046483,heterocycle metabolic process|GO:0016070,RNA metabolic process|GO:0044238,primary metabolic process|GO:0044271,cellular nitrogen compound biosynthetic process|GO:0034641,cellular nitrogen compound metabolic process|GO:0044237,cellular metabolic process|GO:0043170,macromolecule metabolic process|GO:0006354,DNA-templated transcription, elongation|GO:0006351,transcription, DNA-templated|GO:0006352,DNA-templated transcription, initiation|GO:0006353,DNA-templated transcription, termination|GO:0019438,aromatic compound biosynthetic process|GO:0010628,positive regulation of gene expression|GO:0009893,positive regulation of metabolic process|GO:0010604,positive regulation of macromolecule metabolic process|GO:0048518,positive regulation of biological process|GO:0045815,positive regulation of gene expression, epigenetic|GO:0051716,cellular response to stimulus|GO:1990830,cellular response to leukemia inhibitory factor|GO:0034097,response to cytokine|GO:0071345,cellular response to cytokine stimulus|GO:0050896,response to stimulus|GO:0071310,cellular response to organic substance|GO:0070887,cellular response to chemical stimulus|GO:1990823,response to leukemia inhibitory factor|GO:0042221,response to chemical|GO:0010033,response to organic substance</td><td>GO:0030880,RNA polymerase complex|GO:0044464,cell part|GO:0031974,membrane-enclosed lumen|GO:0043229,intracellular organelle|GO:0043228,non-membrane-bounded organelle|GO:0043227,membrane-bounded organelle|GO:0043226,organelle|GO:0005856,cytoskeleton|GO:0005736,DNA-directed RNA polymerase I complex|GO:0031981,nuclear lumen|GO:0005730,nucleolus|GO:0005634,nucleus|GO:0061695,transferase complex, transferring phosphorus-containing groups|GO:0005654,nucleoplasm|GO:0044452,nucleolar part|GO:0015630,microtubule cytoskeleton|GO:1902494,catalytic complex|GO:0055029,nuclear DNA-directed RNA polymerase complex|GO:1990234,transferase complex|GO:0043234,protein complex|GO:0032991,macromolecular complex|GO:0043231,intracellular membrane-bounded organelle|GO:0043232,intracellular non-membrane-bounded organelle|GO:0043233,organelle lumen|GO:0000428,DNA-directed RNA polymerase complex|GO:0005623,cell|GO:0005622,intracellular|GO:0044446,intracellular organelle part|GO:0070013,intracellular organelle lumen|GO:0044428,nuclear part|GO:0044424,intracellular part|GO:0044422,organelle part</td><td>GO:0016779,nucleotidyltransferase activity|GO:0016772,transferase activity, transferring phosphorus-containing groups|GO:0034062,5'-3' RNA polymerase activity|GO:0003824,catalytic activity|GO:0016740,transferase activity|GO:0001054,RNA polymerase I activity|GO:0003899,DNA-directed 5'-3' RNA polymerase activity</td></tr>
<tr><td>MMD2</td><td>GO:0008104,protein localization|GO:0019220,regulation of phosphate metabolic process|GO:0080090,regulation of primary metabolic process|GO:0019222,regulation of metabolic process|GO:0048584,positive regulation of response to stimulus|GO:0048583,regulation of response to stimulus|GO:0007165,signal transduction|GO:0051716,cellular response to stimulus|GO:0010604,positive regulation of macromolecule metabolic process|GO:0009966,regulation of signal transduction|GO:0009967,positive regulation of signal transduction|GO:0045664,regulation of neuron differentiation|GO:0045666,positive regulation of neuron differentiation|GO:0010720,positive regulation of cell development|GO:0048518,positive regulation of biological process|GO:0033036,macromolecule localization|GO:0051057,positive regulation of small GTPase mediated signal transduction|GO:0051056,regulation of small GTPase mediated signal transduction|GO:0060255,regulation of macromolecule metabolic process|GO:0048468,cell development|GO:0045859,regulation of protein kinase activity|GO:0042325,regulation of phosphorylation|GO:0044700,single organism signaling|GO:0042327,positive regulation of phosphorylation|GO:0044707,single-multicellular organism process|GO:0019538,protein metabolic process|GO:0009893,positive regulation of metabolic process|GO:0033674,positive regulation of kinase activity|GO:0035556,intracellular signal transduction|GO:0050789,regulation of biological process|GO:0044267,cellular protein metabolic process|GO:0051347,positive regulation of transferase activity|GO:0045937,positive regulation of phosphate metabolic process|GO:0044260,cellular macromolecule metabolic process|GO:0043549,regulation of kinase activity|GO:0065007,biological regulation|GO:0044699,single-organism process|GO:0065009,regulation of molecular function|GO:0050793,regulation of developmental process|GO:0050790,regulation of catalytic activity|GO:0050794,regulation of cellular process|GO:0043412,macromolecule modification|GO:0036211,protein modification process|GO:0051239,regulation of multicellular organismal process|GO:1902533,positive regulation of intracellular signal transduction|GO:1902531,regulation of intracellular signal transduction|GO:0051174,regulation of phosphorus metabolic process|GO:0050896,response to stimulus|GO:0031401,positive regulation of protein modification process|GO:0051338,regulation of transferase activity|GO:0051962,positive regulation of nervous system development|GO:0051960,regulation of nervous system development|GO:0048869,cellular developmental process|GO:0051240,positive regulation of multicellular organismal process|GO:0008152,metabolic process|GO:0016310,phosphorylation|GO:0030154,cell differentiation|GO:0046578,regulation of Ras protein signal transduction|GO:0046579,positive regulation of Ras protein signal transduction|GO:0023056,positive regulation of signaling|GO:0023052,signaling|GO:0023051,regulation of signaling|GO:0010647,positive regulation of cell communication|GO:0010646,regulation of cell communication|GO:0007265,Ras protein signal transduction|GO:0043085,positive regulation of catalytic activity|GO:0032880,regulation of protein localization|GO:0050767,regulation of neurogenesis|GO:0010562,positive regulation of phosphorus metabolic process|GO:0051246,regulation of protein metabolic process|GO:0051247,positive regulation of protein metabolic process|GO:0060284,regulation of cell development|GO:0050769,positive regulation of neurogenesis|GO:0032270,positive regulation of cellular protein metabolic process|GO:0031399,regulation of protein modification process|GO:0032502,developmental process|GO:0032501,multicellular organismal process|GO:0044093,positive regulation of molecular function|GO:0009987,cellular process|GO:0045597,positive regulation of cell differentiation|GO:0045595,regulation of cell differentiation|GO:0032879,regulation of localization|GO:0032268,regulation of cellular protein metabolic process|GO:0051094,positive regulation of developmental process|GO:0043170,macromolecule metabolic process|GO:0048731,system development|GO:0045860,positive regulation of protein kinase activity|GO:0031325,positive regulation of cellular metabolic process|GO:0031323,regulation of cellular metabolic process|GO:0007275,multicellular organism development|GO:0071704,organic substance metabolic process|GO:0006468,protein phosphorylation|GO:0030182,neuron differentiation|GO:0006464,cellular protein modification process|GO:0044767,single-organism developmental process|GO:0044763,single-organism cellular process|GO:0007154,cell communication|GO:0022008,neurogenesis|GO:0007264,small GTPase mediated signal transduction|GO:0051179,localization|GO:0044238,primary metabolic process|GO:0048699,generation of neurons|GO:0007399,nervous system development|GO:0048856,anatomical structure development|GO:0044237,cellular metabolic process|GO:0006796,phosphate-containing compound metabolic process|GO:2000026,regulation of multicellular organismal development|GO:0006793,phosphorus metabolic process|GO:0001932,regulation of protein phosphorylation|GO:0001934,positive regulation of protein phosphorylation|GO:0048522,positive regulation of cellular process|GO:1901564,organonitrogen compound metabolic process|GO:0006807,nitrogen compound</td><td>GO:0043229,intracellular organelle|GO:0005622,intracellular|GO:0043227,membrane-bounded organelle|GO:0043226,organelle|GO:0031224,intrinsic component of membrane|GO:0005737,cytoplasm|GO:0031090,organelle membrane|GO:0016021,integral component of membrane|GO:0016020,membrane|GO:0044431,Golgi apparatus part|GO:0048471,perinuclear region of cytoplasm|GO:0005794,Golgi apparatus|GO:0098588,bounding membrane of organelle|GO:0012505,endomembrane system|GO:0043231,intracellular membrane-bounded organelle|GO:0044464,cell part|GO:0005623,cell|GO:0000139,Golgi membrane|GO:0044446,intracellular organelle part|GO:0044444,cytoplasmic part|GO:0044424,intracellular part|GO:0044425,membrane part|GO:0044422,organelle part|GO:0031984,organelle subcompartment|GO:0098791,Golgi subcompartment</td><td>GO:0016773,phosphotransferase activity, alcohol group as acceptor|GO:0016772,transferase activity, transferring phosphorus-containing groups|GO:0016301,kinase activity|GO:0003824,catalytic activity|GO:0016740,transferase activity|GO:0004672,protein kinase activity</td></tr>
<tr><td>NT5C1A</td><td>GO:0046434,organophosphate catabolic process|GO:0016311,dephosphorylation|GO:0044237,cellular metabolic process|GO:0009166,nucleotide catabolic process|GO:0072529,pyrimidine-containing compound catabolic process|GO:0009128,purine nucleoside monophosphate catabolic process|GO:0006807,nitrogen compound metabolic process|GO:0044281,small molecule metabolic process|GO:0009123,nucleoside monophosphate metabolic process|GO:0046135,pyrimidine nucleoside catabolic process|GO:0006195,purine nucleotide catabolic process|GO:0009126,purine nucleoside monophosphate metabolic process|GO:0046085,adenosine metabolic process|GO:0044699,single-organism process|GO:0009125,nucleoside monophosphate catabolic process|GO:0044712,single-organism catabolic process|GO:0006139,nucleobase-containing compound metabolic process|GO:0044710,single-organism metabolic process|GO:0072523,purine-containing compound catabolic process|GO:0006206,pyrimidine nucleobase metabolic process|GO:0042278,purine nucleoside metabolic process|GO:0071704,organic substance metabolic process|GO:1901360,organic cyclic compound metabolic process|GO:0019637,organophosphate metabolic process|GO:0072527,pyrimidine-containing compound metabolic process|GO:0044238,primary metabolic process|GO:1901361,organic cyclic compound catabolic process|GO:0019439,aromatic compound catabolic process|GO:0009987,cellular process|GO:0006725,cellular aromatic compound metabolic process|GO:1901575,organic substance catabolic process|GO:0046700,heterocycle catabolic process|GO:0072521,purine-containing compound metabolic process|GO:0009112,nucleobase metabolic process|GO:0009117,nucleotide metabolic process|GO:0009116,nucleoside metabolic process|GO:0008152,metabolic process|GO:0034655,nucleobase-containing compound catabolic process|GO:0009119,ribonucleoside metabolic process|GO:0046128,purine ribonucleoside metabolic process|GO:0009056,catabolic process|GO:0055086,nucleobase-containing small molecule metabolic process|GO:0046483,heterocycle metabolic process|GO:0044248,cellular catabolic process|GO:1901564,organonitrogen compound metabolic process|GO:0044270,cellular nitrogen compound catabolic process|GO:1901136,carbohydrate derivative catabolic process|GO:1901135,carbohydrate derivative metabolic process|GO:0009164,nucleoside catabolic process|GO:0006213,pyrimidine nucleoside metabolic process|GO:0006163,purine nucleotide metabolic process|GO:1901657,glycosyl compound metabolic process|GO:0006796,phosphate-containing compound metabolic process|GO:1901292,nucleoside phosphate catabolic process|GO:0006793,phosphorus metabolic process|GO:0034641,cellular nitrogen compound metabolic process|GO:0044763,single-organism cellular process|GO:0006753,nucleoside phosphate metabolic process|GO:0006144,purine nucleobase metabolic process|GO:1901658,glycosyl compound catabolic process|GO:1901565,organonitrogen compound catabolic process</td><td>GO:0005737,cytoplasm|GO:0005829,cytosol|GO:0044464,cell part|GO:0005623,cell|GO:0005622,intracellular|GO:0044444,cytoplasmic part|GO:0044424,intracellular part</td><td>GO:0016787,hydrolase activity|GO:0043167,ion binding|GO:0016791,phosphatase activity|GO:0043169,cation binding|GO:0097159,organic cyclic compound binding|GO:0000287,magnesium ion binding|GO:0008253,5'-nucleotidase activity|GO:0008252,nucleotidase activity|GO:0016788,hydrolase activity, acting on ester bonds|GO:0000166,nucleotide binding|GO:0042578,phosphoric ester hydrolase activity|GO:0003824,catalytic activity|GO:0036094,small molecule binding|GO:0005488,binding|GO:0046872,metal ion binding|GO:1901363,heterocyclic compound binding|GO:1901265,nucleoside phosphate binding|GO:0005515,protein binding</td></tr>
<tr><td>RNF138</td><td>GO:0009896,positive regulation of catabolic process|GO:0009894,regulation of catabolic process|GO:0044238,primary metabolic process|GO:0032436,positive regulation of proteasomal ubiquitin-dependent protein catabolic process|GO:0009893,positive regulation of metabolic process|GO:0032434,regulation of proteasomal ubiquitin-dependent protein catabolic process|GO:0070647,protein modification by small protein conjugation or removal|GO:0031325,positive regulation of cellular metabolic process|GO:0031323,regulation of cellular metabolic process|GO:0044248,cellular catabolic process|GO:0042176,regulation of protein catabolic process|GO:0023052,signaling|GO:0007165,signal transduction|GO:0007166,cell surface receptor signaling pathway|GO:0010498,proteasomal protein catabolic process|GO:0043632,modification-dependent macromolecule catabolic process|GO:0050789,regulation of biological process|GO:0044699,single-organism process|GO:0080090,regulation of primary metabolic process|GO:0044267,cellular protein metabolic process|GO:0051716,cellular response to stimulus|GO:0044265,cellular macromolecule catabolic process|GO:0031329,regulation of cellular catabolic process|GO:0044260,cellular macromolecule metabolic process|GO:0016567,protein ubiquitination|GO:0043161,proteasome-mediated ubiquitin-dependent protein catabolic process|GO:0051246,regulation of protein metabolic process|GO:0051247,positive regulation of protein metabolic process|GO:0000209,protein polyubiquitination|GO:0060255,regulation of macromolecule metabolic process|GO:0032270,positive regulation of cellular protein metabolic process|GO:0061136,regulation of proteasomal protein catabolic process|GO:0048518,positive regulation of biological process|GO:0065007,biological regulation|GO:1903364,positive regulation of cellular protein catabolic process|GO:0009987,cellular process|GO:1903362,regulation of cellular protein catabolic process|GO:0051603,proteolysis involved in cellular protein catabolic process|GO:1903052,positive regulation of proteolysis involved in cellular protein catabolic process|GO:0031331,positive regulation of cellular catabolic process|GO:1903050,regulation of proteolysis involved in cellular protein catabolic process|GO:1901800,positive regulation of proteasomal protein catabolic process|GO:0019941,modification-dependent protein catabolic process|GO:1901575,organic substance catabolic process|GO:0006464,cellular protein modification process|GO:0050794,regulation of cellular process|GO:0071704,organic substance metabolic process|GO:0043412,macromolecule modification|GO:0036211,protein modification process|GO:0030162,regulation of proteolysis|GO:0030163,protein catabolic process|GO:0008152,metabolic process|GO:0006508,proteolysis|GO:0044257,cellular protein catabolic process|GO:0007154,cell communication|GO:0019222,regulation of metabolic process|GO:0009056,catabolic process|GO:0009057,macromolecule catabolic process|GO:0010604,positive regulation of macromolecule metabolic process|GO:0044700,single organism signaling|GO:0032268,regulation of cellular protein metabolic process|GO:0032446,protein modification by small protein conjugation|GO:0019538,protein metabolic process|GO:0050896,response to stimulus|GO:0016055,Wnt signaling pathway|GO:0044763,single-organism cellular process|GO:0044237,cellular metabolic process|GO:0043170,macromolecule metabolic process|GO:0045732,positive regulation of protein catabolic process|GO:0006511,ubiquitin-dependent protein catabolic process|GO:0045862,positive regulation of proteolysis|GO:0048522,positive regulation of cellular process|GO:1905114,cell surface receptor signaling pathway involved in cell-cell signaling|GO:1901360,organic cyclic compound metabolic process|GO:0006281,DNA repair|GO:0046483,heterocycle metabolic process|GO:1901564,organonitrogen compound metabolic process|GO:1901565,organonitrogen compound catabolic process|GO:0034641,cellular nitrogen compound metabolic process|GO:0033554,cellular response to stress|GO:0006807,nitrogen compound metabolic process|GO:0010792,DNA double-strand break processing involved in repair via single-strand annealing|GO:0006302,double-strand break repair|GO:0006950,response to stress|GO:0006139,nucleobase-containing compound metabolic process|GO:0000726,non-recombinational repair|GO:0000725,recombinational repair|GO:0000724,double-strand break repair via homologous recombination|GO:0000729,DNA double-strand break processing|GO:0006974,cellular response to DNA damage stimulus|GO:0090304,nucleic acid metabolic process|GO:0006725,cellular aromatic compound metabolic process|GO:0198738,cell-cell signaling by wnt|GO:0007267,cell-cell signaling|GO:0006310,DNA recombination|GO:0045002,double-strand break repair via single-strand annealing|GO:0006259,DNA metabolic process|GO:1990830,cellular response to leukemia inhibitory factor|GO:0034097,response to cytokine|GO:0071345,cellular response to cytokine stimulus|GO:0071310,cellular response to organic substance|GO:0070887,cellular response to chemical stimulus|GO:1990823,response to leukemia inhibitory factor|GO:0042221,response to chemical|GO:0010033,response to organic substance</td><td>GO:0043231,intracellular membrane-bounded organelle|GO:0005634,nucleus|GO:0044464,cell part|GO:0005623,cell|GO:0005622,intracellular|GO:0043229,intracellular organelle|GO:0044424,intracellular part|GO:0043227,membrane-bounded organelle|GO:0043226,organelle|GO:0043232,intracellular non-membrane-bounded organelle|GO:0043228,non-membrane-bounded organelle|GO:0005694,chromosome</td><td>GO:0019787,ubiquitin-like protein transferase activity|GO:0043169,cation binding|GO:0031624,ubiquitin conjugating enzyme binding|GO:0046914,transition metal ion binding|GO:0019900,kinase binding|GO:0016874,ligase activity|GO:0061630,ubiquitin protein ligase activity|GO:0019899,enzyme binding|GO:0044390,ubiquitin-like protein conjugating enzyme binding|GO:0043167,ion binding|GO:0019901,protein kinase binding|GO:0003824,catalytic activity|GO:0016740,transferase activity|GO:0008270,zinc ion binding|GO:0005515,protein binding|GO:0005488,binding|GO:0046872,metal ion binding|GO:0004842,ubiquitin-protein transferase activity|GO:0061659,ubiquitin-like protein ligase activity|GO:0003676,nucleic acid binding|GO:0003677,DNA binding|GO:1901363,heterocyclic compound binding|GO:0003697,single-stranded DNA binding|GO:0097159,organic cyclic compound binding</td></tr>
<tr><td>MRPL48</td><td></td><td>GO:0031974,membrane-enclosed lumen|GO:0043229,intracellular organelle|GO:0043228,non-membrane-bounded organelle|GO:0043227,membrane-bounded organelle|GO:0043226,organelle|GO:0005737,cytoplasm|GO:0005739,mitochondrion|GO:0030529,intracellular ribonucleoprotein complex|GO:0005759,mitochondrial matrix|GO:0044429,mitochondrial part|GO:0000313,organellar ribosome|GO:0005840,ribosome|GO:0032991,macromolecular complex|GO:0043231,intracellular membrane-bounded organelle|GO:0043232,intracellular non-membrane-bounded organelle|GO:0043233,organelle lumen|GO:0044464,cell part|GO:0005623,cell|GO:0005622,intracellular|GO:0044446,intracellular organelle part|GO:0070013,intracellular organelle lumen|GO:0044444,cytoplasmic part|GO:0005761,mitochondrial ribosome|GO:0044424,intracellular part|GO:0044422,organelle part|GO:1990904,ribonucleoprotein complex</td><td></td></tr>
<tr><td>IFNW1</td><td>GO:0019220,regulation of phosphate metabolic process|GO:0019221,cytokine-mediated signaling pathway|GO:0019222,regulation of metabolic process|GO:0048583,regulation of response to stimulus|GO:0007165,signal transduction|GO:0007166,cell surface receptor signaling pathway|GO:0080090,regulation of primary metabolic process|GO:0051716,cellular response to stimulus|GO:0045786,negative regulation of cell cycle|GO:0010604,positive regulation of macromolecule metabolic process|GO:0043207,response to external biotic stimulus|GO:0009966,regulation of signal transduction|GO:0048869,cellular developmental process|GO:0002263,cell activation involved in immune response|GO:0018193,peptidyl-amino acid modification|GO:0010033,response to organic substance|GO:0048513,animal organ development|GO:0048518,positive regulation of biological process|GO:0048519,negative regulation of biological process|GO:0033141,positive regulation of peptidyl-serine phosphorylation of STAT protein|GO:0060255,regulation of macromolecule metabolic process|GO:0006952,defense response|GO:0042221,response to chemical|GO:0046649,lymphocyte activation|GO:0009607,response to biotic stimulus|GO:0051707,response to other organism|GO:0002323,natural killer cell activation involved in immune response|GO:0051704,multi-organism process|GO:0042325,regulation of phosphorylation|GO:0044700,single organism signaling|GO:0042327,positive regulation of phosphorylation|GO:0018209,peptidyl-serine modification|GO:0009605,response to external stimulus|GO:0044707,single-multicellular organism process|GO:0019538,protein metabolic process|GO:0002376,immune system process|GO:0043331,response to dsRNA|GO:0043330,response to exogenous dsRNA|GO:0009893,positive regulation of metabolic process|GO:0006950,response to stress|GO:0032943,mononuclear cell proliferation|GO:0014070,response to organic cyclic compound|GO:0046651,lymphocyte proliferation|GO:0035556,intracellular signal transduction|GO:0050789,regulation of biological process|GO:0044267,cellular protein metabolic process|GO:0002286,T cell activation involved in immune response|GO:0044260,cellular macromolecule metabolic process|GO:0002285,lymphocyte activation involved in immune response|GO:0046425,regulation of JAK-STAT cascade|GO:0002366,leukocyte activation involved in immune response|GO:0065007,biological regulation|GO:0044699,single-organism process|GO:0071593,lymphocyte aggregation|GO:0098602,single organism cell adhesion|GO:0034097,response to cytokine|GO:0098609,cell-cell adhesion|GO:0050794,regulation of cellular process|GO:0007050,cell cycle arrest|GO:0001775,cell activation|GO:0036211,protein modification process|GO:0008152,metabolic process|GO:0030097,hemopoiesis|GO:1902531,regulation of intracellular signal transduction|GO:0002521,leukocyte differentiation|GO:0002520,immune system development|GO:0044767,single-organism developmental process|GO:0051607,defense response to virus|GO:0071345,cellular response to cytokine stimulus|GO:1901698,response to nitrogen compound|GO:0031401,positive regulation of protein modification process|GO:0043412,macromolecule modification|GO:0009615,response to virus|GO:0007159,leukocyte cell-cell adhesion|GO:0016310,phosphorylation|GO:0030154,cell differentiation|GO:0023052,signaling|GO:0070887,cellular response to chemical stimulus|GO:0023051,regulation of signaling|GO:0016337,single organismal cell-cell adhesion|GO:0010646,regulation of cell communication|GO:0007049,cell cycle|GO:0006959,humoral immune response|GO:0010562,positive regulation of phosphorus metabolic process|GO:0051246,regulation of protein metabolic process|GO:0051247,positive regulation of protein metabolic process|GO:0007259,JAK-STAT cascade|GO:0032270,positive regulation of cellular protein metabolic process|GO:0031399,regulation of protein modification process|GO:0022610,biological adhesion|GO:0032502,developmental process|GO:0032501,multicellular organismal process|GO:0008283,cell proliferation|GO:0009987,cellular process|GO:0098542,defense response to other organism|GO:0006955,immune response|GO:0032268,regulation of cellular protein metabolic process|GO:0045321,leukocyte activation|GO:0044763,single-organism cellular process|GO:0043170,macromolecule metabolic process|GO:0030098,lymphocyte differentiation|GO:0048731,system development|GO:0050896,response to stimulus|GO:0018105,peptidyl-serine phosphorylation|GO:0033138,positive regulation of peptidyl-serine phosphorylation|GO:0033139,regulation of peptidyl-serine phosphorylation of STAT protein|GO:0033135,regulation of peptidyl-serine phosphorylation|GO:0031325,positive regulation of cellular metabolic process|GO:0031323,regulation of cellular metabolic process|GO:0042113,B cell activation|GO:0042110,T cell activation|GO:0022402,cell cycle process|GO:0007275,multicellular organism development|GO:0070661,leukocyte proliferation|GO:0071704,organic substance metabolic process|GO:0071310,cellular response to organic substance|GO:0048534,hematopoietic or lymphoid organ development|GO:0006468,protein phosphorylation|GO:0045937,positive regulation of phosphate metabolic process|GO:0030183,B cell differentiation|GO:0034109,homotypic cell-cell adhesion|GO:0002252,immune effector process|GO:0042100,B cell proliferation|GO:0006464,cellular protein modification process|GO:0051174,regulation of phosphorus metabolic process|GO:0070489,T cell aggregation|GO:0007155,cell adhesion|GO:0007154,cell communication|GO:0070486,leukocyte aggregation|GO:0030101,natural killer cell activation|GO:0044238,primary metabolic process|GO:0051726,regulation of cell cycle|GO:0042501,serine phosphorylation of STAT protein|GO:0048856,anatomical structure development|GO:0044237,cellular metabolic process|GO:0006796,phosphate-containing compound metabolic process|GO:0002250,adaptive immune response|GO:0006793,phosphorus metabolic process|GO:0001932,regulation of protein phosphorylation|GO:0001934,positive regulation of protein phosphorylation|GO:0048523,negative regulation of cellular process|GO:0048522,positive regulation of cellular process|GO:1901564,organonitrogen compound metabolic process|GO:1904892,regulation of STAT cascade|GO:0006807,nitrogen compound metabolic process|GO:0097696,STAT cascade|GO:0045087,innate immune response</td><td>GO:0005576,extracellular region|GO:0005615,extracellular space|GO:0044421,extracellular region part</td><td>GO:0005126,cytokine receptor binding|GO:0005125,cytokine activity|GO:0005132,type I interferon receptor binding|GO:0044877,macromolecular complex binding|GO:0005488,binding|GO:0005515,protein binding|GO:0032403,protein complex binding|GO:0005102,receptor binding</td></tr>
<tr><td>FAM84A</td><td></td><td></td><td></td></tr>
</table>

LMLN	GO:1903047,mitotic cell cycle process|GO:0022402,cell cycle process|GO:0051301,cell division|GO:0007067,mitotic nuclear division|GO:0044699,single-organism process|GO:0000280,nuclear division|GO:0016043,cellular component organization|GO:0071704,organic substance metabolic process|GO:0071840,cellular component organization or biogenesis|GO:0006508,proteolysis|GO:0022610,biological adhesion|GO:0000278,mitotic cell cycle|GO:0009987,cellular process|GO:0044763,single-organism cellular process|GO:0008152,metabolic process|GO:0007155,cell adhesion|GO:0006996,organelle organization|GO:0044238,primary metabolic process|GO:0007049,cell cycle|GO:0019538,protein metabolic process|GO:0043170,macromolecule metabolic process|GO:1902589,single-organism organelle organization|GO:0048285,organelle fission|GO:1901564,organonitrogen compound metabolic process|GO:0006807,nitrogen compound metabolic process	GO:0005737,cytoplasm|GO:0016020,membrane|GO:0070161,anchoring junction|GO:0043232,intracellular non-membrane-bounded organelle|GO:0005811,lipid particle|GO:0044464,cell part|GO:0005623,cell|GO:0005622,intracellular|GO:0043229,intracellular organelle|GO:0043228,non-membrane-bounded organelle|GO:0005912,adherens junction|GO:0044424,intracellular part|GO:0005924,cell-substrate adherens junction|GO:0005925,focal adhesion|GO:0043226,organelle|GO:0030054,cell junction|GO:0030055,cell-substrate junction|GO:0005829,cytosol|GO:0044444,cytoplasmic part	GO:0016787,hydrolase activity|GO:0004175,endopeptidase activity|GO:0004222,metalloendopeptidase activity|GO:0043169,cation binding|GO:0043167,ion binding|GO:0003824,catalytic activity|GO:0070011,peptidase activity, acting on L-amino acid peptides|GO:0005488,binding|GO:0008233,peptidase activity|GO:0046872,metal ion binding|GO:0008237,metallopeptidase activity
ELSPBP1	GO:0044703,multi-organism reproductive process|GO:0044702,single organism reproductive process|GO:0000003,reproduction|GO:0009566,fertilization|GO:0019953,sexual reproduction|GO:0022414,reproductive process|GO:0007338,single fertilization|GO:0044699,single-organism process|GO:0051704,multi-organism process|GO:0048232,male gamete generation|GO:0022412,cellular process involved in reproduction in multicellular organism|GO:0048468,cell development|GO:0032501,multicellular organismal process|GO:0048240,sperm capacitation|GO:0007276,gamete generation|GO:0048869,cellular developmental process|GO:0048515,spermatid differentiation|GO:0032502,developmental process|GO:0030154,cell differentiation|GO:0048609,multicellular organismal reproductive process|GO:0032504,multicellular organism reproduction|GO:0009987,cellular process|GO:0048469,cell maturation|GO:0007281,germ cell development|GO:0007283,spermatogenesis|GO:0044763,single-organism cellular process|GO:0007286,spermatid development|GO:0044767,single-organism developmental process|GO:0003006,developmental process involved in reproduction|GO:0048856,anatomical structure development|GO:0021700,developmental maturation	GO:0005576,extracellular region|GO:0009986,cell surface|GO:0044464,cell part|GO:0005623,cell	GO:1901681,sulfur compound binding|GO:0043168,anion binding|GO:0097367,carbohydrate derivative binding|GO:0043167,ion binding|GO:0005539,glycosaminoglycan binding|GO:0005488,binding|GO:0008201,heparin binding
FAM89A			
IFNA7	GO:0019220,regulation of phosphate metabolic process|GO:0019221,cytokine-mediated signaling pathway|GO:0019222,regulation of metabolic process|GO:0007596,blood coagulation|GO:0048583,regulation of response to stimulus|GO:0007165,signal transduction|GO:0007166,cell surface receptor signaling pathway|GO:0007599,hemostasis|GO:0031347,regulation of defense response|GO:0080090,regulation of primary metabolic process|GO:0051716,cellular response to stimulus|GO:0010604,positive regulation of macromolecule metabolic process|GO:0043207,response to external biotic stimulus|GO:0009966,regulation of signal transduction|GO:0048869,cellular developmental process|GO:0002263,cell activation involved in immune response|GO:0009611,response to wounding|GO:0018193,peptidyl-amino acid modification|GO:0010033,response to organic substance|GO:0048513,animal organ development|GO:0048518,positive regulation of biological process|GO:0065007,biological regulation|GO:0033141,positive regulation of peptidyl-serine phosphorylation of STAT protein|GO:0060255,regulation of macromolecule metabolic process|GO:0042221,response to chemical|GO:0032268,regulation of cellular protein metabolic process|GO:0009607,response to biotic stimulus|GO:0051707,response to other organism|GO:0002323,natural killer cell activation involved in immune response|GO:0051704,multi-organism process|GO:0042325,regulation of phosphorylation|GO:0044700,single organism signaling|GO:0042327,positive regulation of phosphorylation|GO:0065008,regulation of biological quality|GO:0018209,peptidyl-serine modification|GO:0009605,response to external stimulus|GO:0044707,single-multicellular organism process|GO:0019538,protein metabolic process|GO:0002376,immune system process|GO:0043331,response to dsRNA|GO:0043330,response to exogenous dsRNA|GO:0050896,response to stimulus|GO:0009893,positive regulation of metabolic process|GO:0006950,response to stress|GO:0032943,mononuclear cell proliferation|GO:0046651,lymphocyte proliferation|GO:0035556,intracellular signal transduction|GO:0050789,regulation of biological process|GO:0044267,cellular protein metabolic process|GO:0002286,T cell activation involved in immune response|GO:0044260,cellular macromolecule metabolic process|GO:0002285,lymphocyte activation involved in immune response|GO:0071357,cellular response to type I interferon|GO:0046425,regulation of JAK-STAT cascade|GO:0002366,leukocyte activation involved in immune response|GO:0002682,regulation of immune system process|GO:0014070,response to organic cyclic compound|GO:0071593,lymphocyte aggregation|GO:0098602,single organism cell adhesion|GO:0034097,response to cytokine|GO:0098609,cell-cell adhesion|GO:0042060,wound healing|GO:0050794,regulation of cellular process|GO:0006952,defense response|GO:0001775,cell activation|GO:0050817,coagulation|GO:0034340,response to type I interferon|GO:0008152,metabolic process|GO:0030097,hemopoiesis|GO:1902531,regulation of intracellular signal transduction|GO:0002521,leukocyte differentiation|GO:0002520,immune system development|GO:0044767,single-organism developmental process|GO:0051607,defense response to virus|GO:0071345,cellular response to cytokine stimulus|GO:0080134,regulation of response to stress|GO:0031401,positive regulation of protein modification process|GO:0043412,macromolecule modification|GO:0036211,protein modification process|GO:0009615,response to virus|GO:0007159,leukocyte cell-cell adhesion|GO:0016310,phosphorylation|GO:0030154,cell differentiation|GO:0007155,cell adhesion|GO:0023052,signaling|GO:0060759,regulation of response to cytokine stimulus|GO:0070887,cellular response to chemical stimulus|GO:0023051,regulation of signaling|GO:0016337,single organismal cell-cell adhesion|GO:0010646,regulation of cell communication|GO:0044699,single-organism process|GO:0006959,humoral immune response|GO:0010562,positive regulation of phosphorus metabolic process|GO:0051246,regulation of protein metabolic process|GO:0051247,positive regulation of protein metabolic process|GO:0007259,JAK-STAT cascade|GO:0032270,positive regulation of cellular protein metabolic process|GO:0031399,regulation of protein modification process|GO:0022610,biological adhesion|GO:0032502,developmental process|GO:0032501,multicellular organismal process|GO:0050878,regulation of body fluid levels|GO:0008283,cell proliferation|GO:0009987,cellular process|GO:0098542,defense response to other organism|GO:0006955,immune response|GO:0050776,regulation of immune response|GO:0001959,regulation of cytokine-mediated signaling pathway|GO:0045321,leukocyte activation|GO:0044763,single-organism cellular process|GO:0043170,macromolecule metabolic process|GO:0030098,lymphocyte differentiation|GO:0048731,system development|GO:1901698,response to nitrogen compound|GO:0018105,peptidyl-serine phosphorylation|GO:0033138,positive regulation of peptidyl-serine phosphorylation|GO:0033139,regulation of peptidyl-serine phosphorylation of STAT protein|GO:0033135,regulation of peptidyl-serine phosphorylation|GO:0031325,positive regulation of cellular metabolic process|GO:0031323,regulation of cellular metabolic process|GO:0042113,B cell activation|GO:0042110,T cell activation|GO:0007275,multicellular organism development|GO:0070661,leukocyte proliferation|GO:0071704,organic substance metabolic process|GO:0071310,cellular response to organic substance|GO:0048534,hematopoietic or lymphoid organ development|GO:0006468,protein phosphorylation|GO:0045088,regulation of innate immune response|GO:0045937,positive regulation of phosphate metabolic process|GO:0030183,B cell differentiation|GO:0034109,homotypic cell-cell adhesion|GO:0045087,innate immune response|GO:0002252,immune effector process|GO:0042100,B cell proliferation|GO:0006464,cellular protein modification process|GO:0051174,regulation of phosphorus metabolic process|GO:0070489,T cell aggregation|GO:0060337,type I interferon signaling pathway|GO:0007267,cell-cell signaling|GO:0007154,cell communication|GO:0060338,regulation of type I interferon-mediated signaling pathway|GO:0070486,leukocyte aggregation|GO:0030101,natural killer cell activation|GO:0044238,primary metabolic process|GO:0042501,serine phosphorylation of STAT protein|GO:0048856,anatomical structure development|GO:0044237,cellular metabolic process|GO:0006796,phosphate-containing compound metabolic process|GO:0046649,lymphocyte activation|GO:0002250,adaptive immune response|GO:0006793,phosphorus metabolic process|GO:0001932,regulation of protein phosphorylation|GO:0001934,positive regulation of protein phosphorylation|GO:0048522,positive regulation of cellular process|GO:1901564,organonitrogen compound metabolic process|GO:1904892,regulation of STAT cascade|GO:0006807,nitrogen compound metabolic process|GO:0097696,STAT cascade	GO:0005576,extracellular region|GO:0005615,extracellular space|GO:0044421,extracellular region part	GO:0005126,cytokine receptor binding|GO:0005125,cytokine activity|GO:0005132,type I interferon receptor binding|GO:0044877,macromolecular complex binding|GO:0005488,binding|GO:0005515,protein binding|GO:0032403,protein complex binding|GO:0005102,receptor binding
GATA1	GO:2001234,negative regulation of apoptotic signaling pathway|GO:2001236,regulation of extrinsic apoptotic signaling pathway|GO:2001237,negative regulation of extrinsic apoptotic signaling pathway|GO:0019220,regulation of phosphate metabolic process|GO:0080090,regulation of primary metabolic process|GO:0019222,regulation of metabolic process|GO:2001233,regulation of apoptotic signaling pathway|GO:0048585,negative regulation of response to stimulus|GO:0007596,blood coagulation|GO:0048583,regulation of response to stimulus|GO:2001239,regulation of extrinsic apoptotic signaling pathway in absence of ligand|GO:0032844,regulation of homeostatic process|GO:0043933,macromolecular complex subunit organization|GO:0001503,ossification|GO:0007165,signal transduction|GO:0007166,cell surface receptor signaling pathway|GO:0030500,regulation of bone mineralization|GO:0007599,hemostasis|GO:0030502,negative regulation of bone mineralization|GO:0032989,cellular component morphogenesis|GO:1901362,organic cyclic compound biosynthetic process|GO:0071840,cellular component organization or biogenesis|GO:0051716,cellular response to stimulus|GO:0010605,negative regulation of macromolecule metabolic process|GO:0010604,positive regulation of macromolecule metabolic process|GO:0009968,negative regulation of signal transduction|GO:0097067,cellular response to thyroid hormone stimulus|GO:0009966,regulation of signal transduction|GO:0060318,definitive erythrocyte differentiation|GO:0002262,myeloid cell homeostasis|GO:0018212,peptidyl-tyrosine modification|GO:0002244,hematopoietic progenitor cell differentiation|GO:0045137,development of primary sexual characteristics|GO:0010033,response to organic substance|GO:0071704,organic substance metabolic process|GO:0010724,regulation of definitive erythrocyte differentiation|GO:0043066,negative regulation of apoptotic process|GO:0045639,positive regulation of myeloid cell differentiation|GO:0048518,positive regulation of biological process|GO:0048519,negative regulation of biological process|GO:0042127,regulation of cell proliferation|GO:0030154,cell differentiation|GO:0010467,gene expression|GO:0060255,regulation of macromolecule metabolic process|GO:0060548,negative regulation of cell death|GO:1903018,regulation of glycoprotein metabolic process|GO:0032846,positive regulation of homeostatic process|GO:0034645,cellular macromolecule biosynthetic process|GO:0003006,developmental process involved in reproduction|GO:1903708,positive regulation of hemopoiesis|GO:0007155,cell adhesion|GO:2001141,regulation of RNA biosynthetic process|GO:0008584,male gonad development|GO:1903706,regulation of hemopoiesis|GO:0018193,peptidyl-amino acid modification|GO:0030168,platelet activation|GO:0046483,heterocycle metabolic process|GO:0042325,regulation of phosphorylation|GO:0044700,single organism signaling|GO:0042327,positive regulation of phosphorylation|GO:0044702,single organism reproductive process|GO:0048872,homeostasis of number of cells|GO:0044707,single-multicellular organism process|GO:0009790,embryo development|GO:0050731,positive regulation of peptidyl-tyrosine phosphorylation|GO:0050730,regulation of peptidyl-tyrosine phosphorylation|GO:0071310,cellular response to organic substance|GO:0002376,immune system process|GO:0019538,protein metabolic process|GO:0007154,cell communication|GO:0042060,wound healing|GO:0019438,aromatic compound biosynthetic process|GO:0098609,cell-cell adhesion|GO:1901135,carbohydrate derivative metabolic process|GO:0051098,regulation of binding|GO:0009892,negative regulation of metabolic process|GO:0009893,positive regulation of metabolic process|GO:0009890,negative regulation of biosynthetic process|GO:0009891,positive regulation of biosynthetic process|GO:0009101,glycoprotein biosynthetic process|GO:0048568,embryonic organ development|GO:0051101,regulation of DNA binding|GO:0051100,negative regulation of binding|GO:0033690,positive regulation of osteoblast proliferation|GO:0008152,metabolic process|GO:0010629,negative regulation of gene expression|GO:0010628,positive regulation of gene expression|GO:0042981,regulation of apoptotic process|GO:0050789,regulation of biological process|GO:0097659,nucleic acid-templated transcription|GO:2000677,regulation of transcription regulatory region DNA binding|GO:0000904,cell morphogenesis involved in differentiation|GO:0000902,cell morphogenesis|GO:0044260,cellular macromolecule metabolic process|GO:0045648,positive regulation of erythrocyte differentiation|GO:0036344,platelet morphogenesis|GO:0045646,regulation of erythrocyte differentiation|GO:0008406,gonad development|GO:0016043,cellular component organization|GO:0035854,eosinophil fate commitment|GO:0002684,positive regulation of immune system process|GO:0002682,regulation of immune system process|GO:0090579,dsDNA loop formation|GO:0018108,peptidyl-tyrosine phosphorylation|GO:0006366,transcription from RNA polymerase II promoter|GO:0065009,regulation of molecular function|GO:0065008,regulation of biological quality|GO:0048646,anatomical structure formation involved in morphogenesis|GO:0018130,heterocycle biosynthetic process|GO:0016265,obsolete death|GO:0070168,negative regulation of biomineral tissue development|GO:0098602,single organism cell adhesion|GO:0046546,development of primary male sexual characteristics|GO:0050793,regulation of developmental process|GO:0009611,response to wounding|GO:0051240,positive regulation of multicellular organismal process|GO:0009889,regulation of biosynthetic process|GO:0070167,regulation of biomineral tissue development|GO:0048468,cell development|GO:0050794,regulation of cellular process|GO:0012501,programmed cell death|GO:0001775,cell activation|GO:0050817,coagulation|GO:0043392,negative regulation of DNA binding|GO:0051239,regulation of multicellular organismal process|GO:0048731,system development|GO:0034654,nucleobase-containing compound biosynthetic process|GO:0030278,regulation of ossification|GO:0030279,negative regulation of ossification|GO:0046661,male sex differentiation|GO:0002521,leukocyte differentiation|GO:0002520,immune system development|GO:0016070,RNA metabolic process|GO:1902679,negative regulation of RNA biosynthetic process|GO:0007548,sex differentiation|GO:0045165,cell fate commitment|GO:0044271,cellular nitrogen compound biosynthetic process|GO:0097066,response to thyroid hormone|GO:0050896,response to stimulus|GO:0031401,positive regulation of protein modification process|GO:0043412,macromolecule modification|GO:0030221,basophil differentiation|GO:0065007,biological regulation|GO:0006355,regulation of transcription, DNA-templated|GO:0010557,positive regulation of macromolecule biosynthetic process|GO:0010556,regulation of macromolecule biosynthetic process|GO:0044767,single-organism developmental process|GO:0006351,transcription, DNA-templated|GO:0048869,cellular developmental process|GO:1901576,organic substance biosynthetic process|GO:0010559,regulation of glycoprotein biosynthetic process|GO:0010558,negative regulation of macromolecule biosynthetic process|GO:0032774,RNA biosynthetic process|GO:0016310,phosphorylation|GO:0031214,biomineral tissue development|GO:0030220,platelet formation|GO:0090202,gene looping|GO:0023057,negative regulation of signaling|GO:0034641,cellular nitrogen compound metabolic process|GO:0009792,embryo development ending in birth or egg hatching|GO:0023052,signaling|GO:0010648,negative regulation of cell communication|GO:0070887,cellular response to chemical stimulus|GO:0023051,regulation of signaling|GO:0010646,regulation of cell communication|GO:0044699,single-organism process|GO:0009719,response to endogenous stimulus|GO:0006139,nucleobase-containing compound metabolic process|GO:0034101,erythrocyte homeostasis|GO:0001701,in utero embryonic development|GO:0033688,regulation of osteoblast proliferation|GO:0000122,negative regulation of transcription from RNA polymerase II promoter|GO:0009653,anatomical structure morphogenesis|GO:0033687,osteoblast proliferation|GO:0010562,positive regulation of phosphorus metabolic process|GO:0051241,negative regulation of multicellular organismal process|GO:0051246,regulation of protein metabolic process|GO:0051247,positive regulation of protein metabolic process|GO:0061458,reproductive system development|GO:0030851,granulocyte differentiation|GO:0032270,positive regulation of cellular protein metabolic process|GO:0048821,erythrocyte development|GO:0036211,protein modification process|GO:0031399,regulation of protein modification process|GO:0043009,chordate embryonic development|GO:0022610,biological	GO:0031974,membrane-enclosed lumen|GO:0043229,intracellular organelle|GO:0043227,membrane-bounded organelle|GO:0043226,organelle|GO:0031981,nuclear lumen|GO:0005634,nucleus|GO:0005654,nucleoplasm|GO:0017053,transcriptional repressor complex|GO:0043234,protein complex|GO:0032991,macromolecular complex|GO:0043231,intracellular membrane-bounded organelle|GO:0043233,organelle lumen|GO:0044464,cell part|GO:0005623,cell|GO:0005622,intracellular|GO:0044446,intracellular organelle part|GO:0070013,intracellular organelle lumen|GO:0044428,nuclear part|GO:0005667,transcription factor complex|GO:0044424,intracellular part|GO:0044422,organelle part|GO:0032993,protein-DNA complex	GO:0005488,binding|GO:0001012,RNA polymerase II regulatory region DNA binding|GO:0001159,core promoter proximal region DNA binding|GO:0001158,enhancer sequence-specific DNA binding|GO:0031490,chromatin DNA binding|GO:0001077,transcriptional activator activity, RNA polymerase II core promoter proximal region sequence-specific binding|GO:0044877,macromolecular complex binding|GO:0008270,zinc ion binding|GO:0003676,nucleic acid binding|GO:0003677,DNA binding|GO:0001078,transcriptional repressor activity, RNA polymerase II core promoter proximal region sequence-specific binding|GO:1901363,heterocyclic compound binding|GO:0043565,sequence-specific DNA binding|GO:0043566,structure-specific DNA binding|GO:0046914,transition metal ion binding|GO:0043167,ion binding|GO:0070742,C2H2 zinc finger domain binding|GO:0000987,core promoter proximal region sequence-specific DNA binding|GO:0046872,metal ion binding|GO:0000982,transcription factor activity, RNA polymerase II core promoter proximal region sequence-specific binding|GO:0000981,RNA polymerase II transcription factor activity, sequence-specific DNA binding|GO:0043169,cation binding|GO:0008134,transcription factor binding|GO:0001067,regulatory region nucleic acid binding|GO:0008301,DNA binding, bending|GO:0035326,enhancer binding|GO:0001047,core promoter binding|GO:0001046,core promoter sequence-specific DNA binding|GO:0001085,RNA polymerase II transcription factor binding|GO:0044212,transcription regulatory region DNA binding|GO:0001227,transcriptional repressor activity, RNA polymerase II transcription regulatory region sequence-specific binding|GO:0003682,chromatin binding|GO:0003700,transcription factor activity, sequence-specific DNA binding|GO:0097159,organic cyclic compound binding|GO:0001071,nucleic acid binding transcription factor activity|GO:0001228,transcriptional activator activity, RNA polymerase II transcription regulatory region sequence-specific binding|GO:0000976,transcription regulatory region sequence-specific DNA binding|GO:0000977,RNA polymerase II regulatory region sequence-specific DNA binding|GO:0005515,protein binding|GO:0000975,regulatory region DNA binding|GO:0000978,RNA polymerase II core promoter proximal region sequence-specific DNA binding|GO:0000979,RNA polymerase II core promoter sequence-specific DNA binding|GO:0002039,p53 binding|GO:0019904,protein domain specific binding|GO:1990837,sequence-specific double-stranded DNA binding|GO:0003690,double-stranded DNA binding|GO:0000980,RNA polymerase II distal enhancer sequence-specific DNA binding
IFNA14	GO:0019220,regulation of phosphate metabolic process|GO:0019221,cytokine-mediated signaling pathway|GO:0019222,regulation of metabolic process|GO:0007596,blood coagulation|GO:0048583,regulation of response to stimulus|GO:0007165,signal transduction|GO:0007166,cell surface receptor signaling pathway|GO:0007599,hemostasis|GO:0031347,regulation of defense response|GO:0080090,regulation of primary metabolic process|GO:0051716,cellular response to stimulus|GO:0010604,positive regulation of macromolecule metabolic process|GO:0043207,response to external biotic stimulus|GO:0009966,regulation of signal transduction|GO:0048869,cellular developmental process|GO:0002263,cell activation involved in immune response|GO:0009611,response to wounding|GO:0018193,peptidyl-amino acid modification|GO:0010033,response to organic substance|GO:0048513,animal organ development|GO:0048518,positive regulation of biological process|GO:0065007,biological regulation|GO:0033141,positive regulation of peptidyl-serine phosphorylation of STAT protein|GO:0060255,regulation of macromolecule metabolic process|GO:0042221,response to chemical|GO:0032268,regulation of cellular protein metabolic process|GO:0009607,response to biotic stimulus|GO:0051707,response to other organism|GO:0002323,natural killer cell activation involved in immune response|GO:0051704,multi-organism process|GO:0042325,regulation of phosphorylation|GO:0044700,single organism signaling|GO:0042327,positive regulation of phosphorylation|GO:0065008,regulation of biological quality|GO:0018209,peptidyl-serine modification|GO:0009605,response to external stimulus|GO:0044707,single-multicellular organism process|GO:0019538,protein metabolic process|GO:0002376,immune system process|GO:0043331,response to dsRNA|GO:0043330,response to exogenous dsRNA|GO:0050896,response to stimulus|GO:0009893,positive regulation of metabolic process|GO:0006950,response to stress|GO:0032943,mononuclear cell proliferation|GO:0046651,lymphocyte proliferation|GO:0035556,intracellular signal transduction|GO:0050789,regulation of biological process|GO:0044267,cellular protein metabolic process|GO:0002286,T cell activation involved in immune response|GO:0044260,cellular macromolecule metabolic process|GO:0002285,lymphocyte activation involved in immune response|GO:0071357,cellular response to type I interferon|GO:0046425,regulation of JAK-STAT cascade|GO:0002366,leukocyte activation involved in immune response|GO:0002682,regulation of immune system process|GO:0014070,response to organic cyclic compound|GO:0071593,lymphocyte aggregation|GO:0098602,single organism cell adhesion|GO:0034097,response to cytokine|GO:0098609,cell-cell adhesion|GO:0042060,wound healing|GO:0050794,regulation of cellular process|GO:0006952,defense response|GO:0001775,cell activation|GO:0050817,coagulation|GO:0034340,response to type I interferon|GO:0008152,metabolic process|GO:0030097,hemopoiesis|GO:1902531,regulation of intracellular signal transduction|GO:0002521,leukocyte differentiation|GO:0002520,immune system development|GO:0044767,single-organism developmental process|GO:0051607,defense response to virus|GO:0071345,cellular response to cytokine stimulus|GO:0080134,regulation of response to stress|GO:0031401,positive regulation of protein modification process|GO:0043412,macromolecule modification|GO:0036211,protein modification process|GO:0009615,response to virus|GO:0007159,leukocyte cell-cell adhesion|GO:0016310,phosphorylation|GO:0030154,cell differentiation|GO:0023052,signaling|GO:0060759,regulation of response to cytokine stimulus|GO:0070887,cellular response to chemical stimulus|GO:0023051,regulation of signaling|GO:0016337,single organismal cell-cell adhesion|GO:0010646,regulation of cell communication|GO:0044699,single-organism process|GO:0006959,humoral immune response|GO:0010562,positive regulation of phosphorus metabolic process|GO:0051246,regulation of protein metabolic process|GO:0051247,positive regulation of protein metabolic process|GO:0007259,JAK-STAT cascade|GO:0032270,positive regulation of cellular protein metabolic process|GO:0031399,regulation of protein modification process|GO:0022610,biological adhesion|GO:0032502,developmental process|GO:0032501,multicellular organismal process|GO:0050878,regulation of body fluid levels|GO:0008283,cell proliferation|GO:0009987,cellular process|GO:0098542,defense response to other organism|GO:0006955,immune response|GO:0050776,regulation of immune response|GO:0001959,regulation of cytokine-mediated signaling pathway|GO:0045321,leukocyte activation|GO:0044763,single-organism cellular process|GO:0043170,macromolecule metabolic process|GO:0030098,lymphocyte differentiation|GO:0048731,system development|GO:1901698,response to nitrogen compound|GO:0018105,peptidyl-serine phosphorylation|GO:0033138,positive regulation of peptidyl-serine phosphorylation|GO:0033139,regulation of peptidyl-serine phosphorylation of STAT protein|GO:0033135,regulation of peptidyl-serine phosphorylation|GO:0031325,positive regulation of cellular metabolic process|GO:0031323,regulation of cellular metabolic process|GO:0042113,B cell activation|GO:0042110,T cell activation|GO:0007275,multicellular organism development|GO:0070661,leukocyte proliferation|GO:0071704,organic substance metabolic process|GO:0071310,cellular response to organic substance|GO:0048534,hematopoietic or lymphoid organ development|GO:0006468,protein phosphorylation|GO:0045088,regulation of innate immune response|GO:0045937,positive regulation of phosphate metabolic process|GO:0030183,B cell differentiation|GO:0034109,homotypic cell-cell adhesion|GO:0045087,innate immune response|GO:0002252,immune effector process|GO:0042100,B cell proliferation|GO:0006464,cellular protein modification process|GO:0051174,regulation of phosphorus metabolic process|GO:0070489,T cell aggregation|GO:0060337,type I interferon signaling pathway|GO:0007155,cell adhesion|GO:0007154,cell communication|GO:0060338,regulation of type I interferon-mediated signaling pathway|GO:0070486,leukocyte aggregation|GO:0030101,natural killer cell activation|GO:0044238,primary metabolic process|GO:0042501,serine phosphorylation of STAT protein|GO:0048856,anatomical structure development|GO:0044237,cellular metabolic process|GO:0006796,phosphate-containing compound metabolic process|GO:0046649,lymphocyte activation|GO:0002250,adaptive immune response|GO:0006793,phosphorus metabolic process|GO:0001932,regulation of protein phosphorylation|GO:0001934,positive regulation of protein phosphorylation|GO:0048522,positive regulation of cellular	GO:0005576,extracellular region|GO:0005615,extracellular space|GO:0044421,extracellular region part	GO:0005126,cytokine receptor binding|GO:0005125,cytokine activity|GO:0005132,type I interferon receptor binding|GO:0044877,macromolecular complex binding|GO:0005488,binding|GO:0005515,protein binding|GO:0032403,protein complex binding|GO:0005102,receptor binding
TMEM78		GO:0044425,membrane part|GO:0016021,integral component of membrane|GO:0016020,membrane|GO:0031224,intrinsic component of membrane	
GABARAP	GO:0009991,response to extracellular stimulus|GO:0022607,cellular component assembly|GO:0008152,metabolic process|GO:0051234,establishment of localization|GO:0006950,response to stress|GO:0006810,transport|GO:0051649,establishment of localization in cell|GO:0016236,macroautophagy|GO:0097191,extrinsic apoptotic signaling pathway|GO:0031667,response to nutrient levels|GO:1903008,organelle disassembly|GO:0061024,membrane organization|GO:0008104,protein localization|GO:0023052,signaling|GO:0007165,signal transduction|GO:0007166,cell surface receptor signaling pathway|GO:0008219,cell death|GO:0050789,regulation of biological process|GO:0044699,single-organism process|GO:0044712,single-organism catabolic process|GO:0044710,single-organism metabolic process|GO:0008625,extrinsic apoptotic signaling pathway via death domain receptors|GO:0070727,cellular macromolecule localization|GO:0071840,cellular component organization or biogenesis|GO:0016043,cellular component organization|GO:0000045,autophagosome assembly|GO:0042594,response to starvation|GO:0065007,biological regulation|GO:0007005,mitochondrion organization|GO:0044248,cellular catabolic process|GO:0071702,organic substance transport|GO:0033036,macromolecule localization|GO:0006914,autophagy|GO:0034613,cellular protein localization|GO:0016265,obsolete death|GO:0009987,cellular process|GO:0007033,vacuole organization|GO:0061726,mitochondrion disassembly|GO:0097190,apoptotic signaling pathway|GO:0061025,membrane fusion|GO:0006915,apoptotic process|GO:0051716,cellular response to stimulus|GO:0050794,regulation of cellular process|GO:0045184,establishment of protein localization|GO:0012501,programmed cell death|GO:0044765,single-organism transport|GO:0007017,microtubule-based process|GO:0022411,cellular component disassembly|GO:0009267,cellular response to starvation|GO:0007268,chemical synaptic transmission|GO:0007267,cell-cell signaling|GO:0007154,cell communication|GO:0006605,protein targeting|GO:0009056,catabolic process|GO:0051179,localization|GO:1902578,single-organism localization|GO:0051641,cellular localization|GO:0006996,organelle organization|GO:0044700,single organism signaling|GO:0031668,cellular response to extracellular stimulus|GO:0031669,cellular response to nutrient levels|GO:0007010,cytoskeleton organization|GO:0046907,intracellular transport|GO:0000422,mitophagy|GO:0050896,response to stimulus|GO:0044763,single-organism cellular process|GO:0044804,nucleophagy|GO:0044237,cellular metabolic process|GO:0071496,cellular response to external stimulus|GO:0009605,response to external stimulus|GO:1902582,single-organism intracellular transport|GO:1902589,single-organism organelle organization|GO:0044085,cellular component biogenesis|GO:0070925,organelle assembly|GO:0033554,cellular response to stress|GO:0015031,protein transport|GO:0000226,microtubule cytoskeleton organization|GO:0006886,intracellular protein transport|GO:0015833,peptide transport|GO:0071705,nitrogen compound transport|GO:0043562,cellular response to nitrogen levels|GO:1905037,autophagosome organization|GO:0098916,anterograde trans-synaptic signaling|GO:0097352,autophagosome maturation|GO:0097576,vacuole fusion|GO:0006995,cellular response to nitrogen starvation|GO:0048284,organelle fusion|GO:0042886,amide transport|GO:0099536,synaptic signaling|GO:0099537,trans-synaptic signaling	GO:0000323,lytic vacuole|GO:0015629,actin cytoskeleton|GO:0044444,cytoplasmic part|GO:0005737,cytoplasm|GO:0031090,organelle membrane|GO:0019898,extrinsic component of membrane|GO:0043226,organelle|GO:0043229,intracellular organelle|GO:0043228,non-membrane-bounded organelle|GO:0005929,cilium|GO:0005622,intracellular|GO:0005874,microtubule|GO:0005875,microtubule associated complex|GO:0005783,endoplasmic reticulum|GO:0005856,cytoskeleton|GO:0005764,lysosome|GO:0000407,pre-autophagosomal structure|GO:0031982,vesicle|GO:0005773,vacuole|GO:0031410,cytoplasmic vesicle|GO:0098805,whole membrane|GO:0016020,membrane|GO:0005776,autophagosome|GO:0005774,vacuolar membrane|GO:0044431,Golgi apparatus part|GO:0044430,cytoskeletal part|GO:0044437,vacuolar part|GO:0048471,perinuclear region of cytoplasm|GO:0005794,Golgi apparatus|GO:0015630,microtubule cytoskeleton|GO:0005790,smooth endoplasmic reticulum|GO:0032838,cell projection cytoplasm|GO:0098588,bounding membrane of organelle|GO:0044297,cell body|GO:0012505,endomembrane system|GO:0005886,plasma membrane|GO:0042995,cell projection|GO:0005930,axoneme|GO:0000421,autophagosome membrane|GO:0043234,protein complex|GO:0032991,macromolecular complex|GO:0043231,intracellular membrane-bounded organelle|GO:0043232,intracellular non-membrane-bounded organelle|GO:0005829,cytosol|GO:0044463,cell projection part|GO:0044464,cell part|GO:0005623,cell|GO:0000139,Golgi membrane|GO:0044446,intracellular organelle part|GO:0043227,membrane-bounded organelle|GO:0071944,cell periphery|GO:0097014,ciliary plasm|GO:0044441,ciliary part|GO:0044424,intracellular part|GO:0044425,membrane part|GO:0044422,organelle part|GO:0099512,supramolecular fiber|GO:0099513,polymeric cytoskeletal fiber|GO:0097223,sperm part|GO:0031984,organelle subcompartment|GO:0097225,sperm midpiece|GO:0097708,intracellular vesicle|GO:0098791,Golgi subcompartment|GO:0036126,sperm flagellum	GO:0015631,tubulin binding|GO:0008092,cytoskeletal protein binding|GO:0008017,microtubule binding|GO:0048487,beta-tubulin binding|GO:0044877,macromolecular complex binding|GO:0050811,GABA receptor binding|GO:0005515,protein binding|GO:0032403,protein complex binding|GO:0005102,receptor binding|GO:0005488,binding|GO:0031625,ubiquitin protein ligase binding|GO:0044389,ubiquitin-like protein ligase binding|GO:0019899,enzyme binding
SOWAHD			
RPL30	GO:0008104,protein localization|GO:0061024,membrane organization|GO:1901362,organic cyclic compound biosynthetic process|GO:1901360,organic cyclic compound metabolic process|GO:1901361,organic cyclic compound catabolic process|GO:0070727,cellular macromolecule localization|GO:0043043,peptide biosynthetic process|GO:0044419,interspecies interaction between organisms|GO:0019058,viral life cycle|GO:0033036,macromolecule localization|GO:0051704,multi-organism process|GO:0006605,protein targeting|GO:0045184,establishment of protein localization|GO:0072657,protein localization to membrane|GO:0044403,symbiosis, encompassing mutualism through parasitism|GO:0046483,heterocycle metabolic process|GO:1901564,organonitrogen compound metabolic process|GO:1901566,organonitrogen compound biosynthetic process|GO:0019538,protein metabolic process|GO:0019439,aromatic compound catabolic process|GO:0019438,aromatic compound biosynthetic process|GO:0019080,viral gene expression|GO:0006613,cotranslational protein targeting to membrane|GO:0006612,protein targeting to membrane|GO:0006614,SRP-dependent cotranslational protein targeting to membrane|GO:0006807,nitrogen compound metabolic process|GO:0044033,multi-organism metabolic process|GO:1901576,organic substance biosynthetic process|GO:1901575,organic substance catabolic process|GO:0044265,cellular macromolecule catabolic process|GO:0044260,cellular macromolecule metabolic process|GO:0006886,intracellular protein transport|GO:0016043,cellular component organization|GO:0071840,cellular component organization or biogenesis|GO:0018130,heterocycle biosynthetic process|GO:0006810,transport|GO:0046700,heterocycle catabolic process|GO:0044802,single-organism membrane organization|GO:0008152,metabolic process|GO:0034654,nucleobase-containing compound biosynthetic process|GO:0034655,nucleobase-containing compound catabolic process|GO:0051234,establishment of localization|GO:0016070,RNA metabolic process|GO:0016071,mRNA metabolic process|GO:0044271,cellular nitrogen compound biosynthetic process|GO:0044270,cellular nitrogen compound catabolic process|GO:0046907,intracellular transport|GO:0044765,single-organism transport|GO:0045047,protein targeting to ER|GO:0044764,multi-organism cellular process|GO:0022411,cellular component disassembly|GO:0006518,peptide metabolic process|GO:0032774,RNA biosynthetic process|GO:0044248,cellular catabolic process|GO:0044249,cellular biosynthetic process|GO:0034641,cellular nitrogen compound metabolic process|GO:0034645,cellular macromolecule biosynthetic process|GO:0043241,protein complex disassembly|GO:0044699,single-organism process|GO:0006139,nucleobase-containing compound metabolic process|GO:0070972,protein localization to endoplasmic reticulum|GO:0051179,localization|GO:0002181,cytoplasmic translation|GO:0019083,viral transcription|GO:0072599,establishment of protein localization to endoplasmic reticulum|GO:0072594,establishment of protein localization to organelle|GO:0009987,cellular process|GO:0006725,cellular aromatic compound metabolic process|GO:0016482,cytosolic transport|GO:0043604,amide biosynthetic process|GO:0043603,cellular amide metabolic process|GO:0043170,macromolecule metabolic process|GO:0000956,nuclear-transcribed mRNA catabolic process|GO:0033365,protein localization to organelle|GO:0000184,nuclear-transcribed mRNA catabolic process, nonsense-mediated decay|GO:0043933,macromolecular complex subunit organization|GO:0090304,nucleic acid metabolic process|GO:0032984,macromolecular complex disassembly|GO:0071822,protein complex subunit organization|GO:0071704,organic substance metabolic process|GO:0010467,gene expression|GO:0071702,organic substance transport|GO:0006401,RNA catabolic process|GO:0006402,mRNA catabolic process|GO:0044267,cellular protein metabolic process|GO:0034613,cellular protein localization|GO:0009058,biosynthetic process|GO:0009059,macromolecule biosynthetic process|GO:0044763,single-organism cellular process|GO:0051649,establishment of localization in cell|GO:0043624,cellular protein complex disassembly|GO:0009056,catabolic process|GO:0009057,macromolecule catabolic process|GO:1902578,single-organism localization|GO:0051641,cellular localization|GO:0044238,primary metabolic process|GO:1902580,single-organism cellular localization|GO:0090150,establishment of protein localization to membrane|GO:0044237,cellular metabolic process|GO:0016032,viral process|GO:0015031,protein transport|GO:1902582,single-organism intracellular transport|GO:0006415,translational termination|GO:0006414,translational elongation|GO:0006413,translational initiation|GO:0006412,translation|GO:0045901,positive regulation of translational elongation|GO:0019222,regulation of metabolic process|GO:0080090,regulation of primary metabolic process|GO:0010605,negative regulation of macromolecule metabolic process|GO:0010604,positive regulation of macromolecule metabolic process|GO:0043207,response to external biotic stimulus|GO:0010608,posttranscriptional regulation of gene expression|GO:0009617,response to bacterium|GO:0048513,animal organ development|GO:0048518,positive regulation of biological process|GO:0048519,negative regulation of biological process|GO:0001906,cell killing|GO:0034470,ncRNA processing|GO:0031100,animal organ regeneration|GO:0060255,regulation of macromolecule metabolic process|GO:0001514,selenocysteine incorporation|GO:0009607,response to biotic stimulus|GO:0051707,response to other organism|GO:0044707,single-multicellular organism process|GO:0015833,peptide transport|GO:0006451,translational readthrough|GO:0009892,negative regulation of metabolic process|GO:0009893,positive regulation of metabolic process|GO:0009891,positive regulation of biosynthetic process|GO:0010629,negative regulation of gene expression|GO:0010628,positive regulation of gene expression|GO:0042742,defense response to bacterium|GO:0050829,defense response to Gram-negative bacterium|GO:0034660,ncRNA metabolic process|GO:0050789,regulation of biological process|GO:0009605,response to external stimulus|GO:0042254,ribosome biogenesis|GO:0065007,biological regulation|GO:0006364,rRNA processing|GO:0045727,positive regulation of translation|GO:0009889,regulation of biosynthetic process|GO:0050794,regulation of cellular process|GO:0006952,defense response|GO:0006950,response to stress|GO:0016072,rRNA metabolic process|GO:0097421,liver regeneration|GO:0050896,response to stimulus|GO:0031640,killing of cells of other organism|GO:0010557,positive regulation of macromolecule biosynthetic process|GO:0010556,regulation of macromolecule biosynthetic process|GO:0034250,positive regulation of cellular amide metabolic process|GO:0031099,regeneration|GO:0042886,amide transport|GO:0044364,disruption of cells of other organism|GO:0061008,hepaticobiliary system development|GO:0051246,regulation of protein metabolic process|GO:0051247,positive regulation of protein metabolic process|GO:0032270,positive regulation of cellular protein metabolic process|GO:0022613,ribonucleoprotein complex biogenesis|GO:0032502,developmental process|GO:0032501,multicellular organismal process|GO:0034248,regulation of cellular amide metabolic process|GO:0098542,defense response to other organism|GO:0032268,regulation of cellular protein metabolic process|GO:0048731,system development|GO:0048732,gland development|GO:0031328,positive regulation of cellular biosynthetic process|GO:0031326,regulation of cellular biosynthetic process|GO:0031325,positive regulation of cellular metabolic process|GO:0031323,regulation of cellular metabolic process|GO:0001889,liver development|GO:0006448,regulation of translational elongation|GO:0007275,multicellular organism development|GO:2000112,regulation of cellular macromolecule biosynthetic process|GO:0071705,nitrogen compound transport|GO:1904571,positive regulation of selenocysteine incorporation|GO:0010468,regulation of gene expression|GO:0044767,single-organism developmental process|GO:0051171,regulation of nitrogen compound metabolic process|GO:0051173,positive regulation of nitrogen compound metabolic process|GO:0048856,anatomical structure development|GO:0035821,modification of morphology or physiology of other organism|GO:0044085,cellular component biogenesis|GO:1904569,regulation of selenocysteine incorporation|GO:0006417,regulation of	GO:0015934,large ribosomal subunit|GO:0022625,cytosolic large ribosomal subunit|GO:0043229,intracellular organelle|GO:0043228,non-membrane-bounded organelle|GO:0022626,cytosolic ribosome|GO:0005924,cell-substrate adherens junction|GO:0005925,focal adhesion|GO:0043227,membrane-bounded organelle|GO:0043226,organelle|GO:0030054,cell junction|GO:0030055,cell-substrate junction|GO:0005737,cytoplasm|GO:0031982,vesicle|GO:0005634,nucleus|GO:0016020,membrane|GO:0031988,membrane-bounded vesicle|GO:0005576,extracellular region|GO:0030529,intracellular ribonucleoprotein complex|GO:0070161,anchoring junction|GO:0044391,ribosomal subunit|GO:0005912,adherens junction|GO:0032991,macromolecular complex|GO:1903561,extracellular vesicle|GO:0005840,ribosome|GO:0043230,extracellular organelle|GO:0043231,intracellular membrane-bounded organelle|GO:0043232,intracellular non-membrane-bounded organelle|GO:0070062,extracellular exosome|GO:0005829,cytosol|GO:0044464,cell part|GO:0005623,cell|GO:0005622,intracellular|GO:0044446,intracellular organelle part|GO:0044444,cytoplasmic part|GO:0044445,cytosolic part|GO:0044424,intracellular part|GO:0065010,extracellular membrane-bounded organelle|GO:0044421,extracellular region part|GO:0044422,organelle part|GO:0031012,extracellular matrix|GO:1990904,ribonucleoprotein complex|GO:0005844,polysome|GO:0042788,polysomal ribosome	GO:0005515,protein binding|GO:0097159,organic cyclic compound binding|GO:0044822,poly(A) RNA binding|GO:0005198,structural molecule activity|GO:0003735,structural constituent of ribosome|GO:0003723,RNA binding|GO:0003676,nucleic acid binding|GO:1901363,heterocyclic compound binding|GO:0005488,binding|GO:0035368,selenocysteine insertion sequence binding|GO:0003729,mRNA binding

ZCCHC16	GO:0032501,multicellular organismal process|GO:1901564,organonitrogen compound metabolic process|GO:0050877,neurological system process|GO:0009712,catechol-containing compound metabolic process|GO:0050890,cognition|GO:0006584,catecholamine metabolic process|GO:0044237,cellular metabolic process|GO:0071704,organic substance metabolic process|GO:0042415,norepinephrine metabolic process|GO:0006807,nitrogen compound metabolic process|GO:0008152,metabolic process|GO:0006725,cellular aromatic compound metabolic process|GO:0018958,phenol-containing compound metabolic process|GO:0009987,cellular process|GO:1901615,organic hydroxy compound metabolic process|GO:1901360,organic cyclic compound metabolic process|GO:0003008,system process		GO:0043169,cation binding|GO:0046914,transition metal ion binding|GO:0097159,organic cyclic compound binding|GO:0043167,ion binding|GO:0008270,zinc ion binding|GO:0003676,nucleic acid binding|GO:0046872,metal ion binding|GO:1901363,heterocyclic compound binding|GO:0005488,binding
MTRNR2L9		GO:0005737,cytoplasm|GO:0044464,cell part|GO:0005623,cell|GO:0005622,intracellular|GO:0005576,extracellular region|GO:0044424,intracellular part	
KRTAP11			
COPS2	GO:0080090,regulation of primary metabolic process|GO:0019222,regulation of metabolic process|GO:0007165,signal transduction|GO:1901362,organic cyclic compound biosynthetic process|GO:1901360,organic cyclic compound metabolic process|GO:0007517,muscle organ development|GO:0010605,negative regulation of macromolecule metabolic process|GO:0048869,cellular developmental process|GO:0007519,skeletal muscle tissue development|GO:0070646,protein modification by small protein removal|GO:0048513,animal organ development|GO:0048519,negative regulation of biological process|GO:0060255,regulation of macromolecule metabolic process|GO:2001141,regulation of RNA biosynthetic process|GO:0046483,heterocycle metabolic process|GO:0044700,single organism signaling|GO:0044707,single-multicellular organism process|GO:0019538,protein metabolic process|GO:0019438,aromatic compound biosynthetic process|GO:0051716,cellular response to stimulus|GO:0009892,negative regulation of metabolic process|GO:0009890,negative regulation of biosynthetic process|GO:0010629,negative regulation of gene expression|GO:0006807,nitrogen compound metabolic process|GO:0050789,regulation of biological process|GO:0097659,nucleic acid-templated transcription|GO:0044267,cellular protein metabolic process|GO:0044260,cellular macromolecule metabolic process|GO:0065007,biological regulation|GO:0006366,transcription from RNA polymerase II promoter|GO:0035914,skeletal muscle cell differentiation|GO:0018130,heterocycle biosynthetic process|GO:0000338,protein deneddylation|GO:0009889,regulation of biosynthetic process|GO:0009888,tissue development|GO:0050794,regulation of cellular process|GO:0043412,macromolecule modification|GO:0036211,protein modification process|GO:0008152,metabolic process|GO:0034654,nucleobase-containing compound biosynthetic process|GO:0016070,RNA metabolic process|GO:0044767,single-organism developmental process|GO:0044271,cellular nitrogen compound biosynthetic process|GO:0050896,response to stimulus|GO:0006355,regulation of transcription, DNA-templated|GO:0010556,regulation of macromolecule biosynthetic process|GO:0006351,transcription, DNA-templated|GO:0010558,negative regulation of macromolecule biosynthetic process|GO:0032774,RNA biosynthetic process|GO:0070647,protein modification by small protein conjugation or removal|GO:0030154,cell differentiation|GO:0044249,cellular biosynthetic process|GO:0034641,cellular nitrogen compound metabolic process|GO:0023052,signaling|GO:0034645,cellular macromolecule biosynthetic process|GO:0061061,muscle structure development|GO:0044699,single-organism process|GO:0006139,nucleobase-containing compound metabolic process|GO:0006508,proteolysis|GO:0032502,developmental process|GO:0032501,multicellular organismal process|GO:0008283,cell proliferation|GO:0009987,cellular process|GO:0006725,cellular aromatic compound metabolic process|GO:1903506,regulation of nucleic acid-templated transcription|GO:1903507,negative regulation of nucleic acid-templated transcription|GO:0045892,negative regulation of transcription, DNA-templated|GO:0051253,negative regulation of RNA metabolic process|GO:0051252,regulation of RNA metabolic process|GO:0043170,macromolecule metabolic process|GO:0048731,system development|GO:0031327,negative regulation of cellular biosynthetic process|GO:0031326,regulation of cellular biosynthetic process|GO:0031324,negative regulation of cellular metabolic process|GO:0031323,regulation of cellular metabolic process|GO:0090304,nucleic acid metabolic process|GO:0014706,striated muscle tissue development|GO:0007275,multicellular organism development|GO:2000112,regulation of cellular macromolecule biosynthetic process|GO:2000113,negative regulation of cellular macromolecule biosynthetic process|GO:0071704,organic substance metabolic process|GO:0010467,gene expression|GO:0010468,regulation of gene expression|GO:0045934,negative regulation of nucleobase-containing compound metabolic process|GO:1901576,organic substance biosynthetic process|GO:0019219,regulation of nucleobase-containing compound metabolic process|GO:0006464,cellular protein modification process|GO:1902679,negative regulation of RNA biosynthetic process|GO:0009058,biosynthetic process|GO:0009059,macromolecule biosynthetic process|GO:0044763,single-organism cellular process|GO:0051171,regulation of nitrogen compound metabolic process|GO:0051172,negative regulation of nitrogen compound metabolic process|GO:0007154,cell communication|GO:0010388,cullin deneddylation|GO:0044238,primary metabolic process|GO:0048856,anatomical structure development|GO:0044237,cellular metabolic process|GO:0060537,muscle tissue development|GO:0060538,skeletal muscle organ development|GO:0048523,negative regulation of cellular process|GO:0071840,cellular component organization or biogenesis|GO:0006283,transcription-coupled nucleotide-excision repair|GO:0006281,DNA repair|GO:0006289,nucleotide-excision repair|GO:1901564,organonitrogen compound metabolic process|GO:0000715,nucleotide-excision repair, DNA damage recognition|GO:0033554,cellular response to stress|GO:0016043,cellular component organization|GO:0006950,response to stress|GO:0000122,negative regulation of transcription from RNA polymerase II promoter|GO:0043687,post-translational protein modification|GO:0006974,cellular response to DNA damage stimulus|GO:0006357,regulation of transcription from RNA polymerase II promoter|GO:0030182,neuron differentiation|GO:0022008,neurogenesis|GO:0006996,organelle organization|GO:0048699,generation of neurons|GO:0051276,chromosome organization|GO:0007399,nervous system development|GO:0006259,DNA metabolic process	GO:0043234,protein complex|GO:0005737,cytoplasm|GO:0032991,macromolecular complex|GO:0043231,intracellular membrane-bounded organelle|GO:0005634,nucleus|GO:0044464,cell part|GO:0005623,cell|GO:0005622,intracellular|GO:0044446,intracellular organelle part|GO:0043229,intracellular organelle|GO:0044428,nuclear part|GO:0044424,intracellular part|GO:0043227,membrane-bounded organelle|GO:0043226,organelle|GO:0044422,organelle part|GO:0008180,COP9 signalosome|GO:0031974,membrane-enclosed lumen|GO:0031981,nuclear lumen|GO:0005654,nucleoplasm|GO:0043233,organelle lumen|GO:0005829,cytosol|GO:0070013,intracellular organelle lumen|GO:0044444,cytoplasmic part	GO:0004871,signal transducer activity|GO:0003714,transcription corepressor activity|GO:0003712,transcription cofactor activity|GO:0000989,transcription factor activity, transcription factor binding|GO:0060089,molecular transducer activity|GO:0005488,binding|GO:0005515,protein binding|GO:0000988,transcription factor activity, protein binding
DNASE2B	GO:0006921,cellular component disassembly involved in execution phase of apoptosis|GO:0097194,execution phase of apoptosis|GO:0090304,nucleic acid metabolic process|GO:0090305,nucleic acid phosphodiester bond hydrolysis|GO:0034641,cellular nitrogen compound metabolic process|GO:0030262,apoptotic nuclear changes|GO:0016043,cellular component organization|GO:0008219,cell death|GO:0071840,cellular component organization or biogenesis|GO:1901361,organic cyclic compound catabolic process|GO:0006139,nucleobase-containing compound metabolic process|GO:1901575,organic substance catabolic process|GO:0044265,cellular macromolecule catabolic process|GO:0044260,cellular macromolecule metabolic process|GO:1901360,organic cyclic compound metabolic process|GO:0006309,apoptotic DNA fragmentation|GO:0006308,DNA catabolic process|GO:0071704,organic substance metabolic process|GO:0044699,single-organism process|GO:0016265,obsolete death|GO:0009987,cellular process|GO:0044238,primary metabolic process|GO:0019439,aromatic compound catabolic process|GO:0006915,apoptotic process|GO:0006725,cellular aromatic compound metabolic process|GO:0046700,heterocycle catabolic process|GO:0012501,programmed cell death|GO:0022411,cellular component disassembly|GO:0008152,metabolic process|GO:0034655,nucleobase-containing compound catabolic process|GO:0009056,catabolic process|GO:0009057,macromolecule catabolic process|GO:0046483,heterocycle metabolic process|GO:0044248,cellular catabolic process|GO:0044270,cellular nitrogen compound catabolic process|GO:0000737,DNA catabolic process, endonucleolytic|GO:0044237,cellular metabolic process|GO:0043170,macromolecule metabolic process|GO:0006807,nitrogen compound metabolic process|GO:0006259,DNA metabolic process|GO:0044763,single-organism cellular process	GO:0005737,cytoplasm|GO:0000323,lytic vacuole|GO:0043231,intracellular membrane-bounded organelle|GO:0005773,vacuole|GO:0044464,cell part|GO:0044444,cytoplasmic part|GO:0005623,cell|GO:0005622,intracellular|GO:0005764,lysosome|GO:0043229,intracellular organelle|GO:0044424,intracellular part|GO:0043227,membrane-bounded organelle|GO:0043226,organelle|GO:0005576,extracellular region	GO:0016787,hydrolase activity|GO:0003824,catalytic activity|GO:0004536,deoxyribonuclease activity|GO:0004519,endonuclease activity|GO:0004520,endodeoxyribonuclease activity|GO:0016788,hydrolase activity, acting on ester bonds|GO:0004531,deoxyribonuclease II activity|GO:0004518,nuclease activity|GO:0016894,endonuclease activity, active with either ribo- or deoxyribonucleic acids and producing 3'-phosphomonoesters|GO:0016889,endodeoxyribonuclease activity, producing 3'-phosphomonoesters
LSM3	GO:0090304,nucleic acid metabolic process|GO:0034641,cellular nitrogen compound metabolic process|GO:0006807,nitrogen compound metabolic process|GO:0044237,cellular metabolic process|GO:1901360,organic cyclic compound metabolic process|GO:1901361,organic cyclic compound catabolic process|GO:0006139,nucleobase-containing compound metabolic process|GO:1901575,organic substance catabolic process|GO:0044265,cellular macromolecule catabolic process|GO:0000288,nuclear-transcribed mRNA catabolic process, deadenylation-dependent decay|GO:0044260,cellular macromolecule metabolic process|GO:0009056,catabolic process|GO:0071704,organic substance metabolic process|GO:0010467,gene expression|GO:0006401,RNA catabolic process|GO:0006402,mRNA catabolic process|GO:0008380,RNA splicing|GO:0044238,primary metabolic process|GO:0009987,cellular process|GO:0006725,cellular aromatic compound metabolic process|GO:0046700,heterocycle catabolic process|GO:0000375,RNA splicing, via transesterification reactions|GO:0000377,RNA splicing, via transesterification reactions with bulged adenosine as nucleophile|GO:0008152,metabolic process|GO:0034655,nucleobase-containing compound catabolic process|GO:0043928,exonucleolytic nuclear-transcribed mRNA catabolic process involved in deadenylation-dependent decay|GO:0009057,macromolecule catabolic process|GO:0044248,cellular catabolic process|GO:0046483,heterocycle metabolic process|GO:0016070,RNA metabolic process|GO:0016071,mRNA metabolic process|GO:0000291,nuclear-transcribed mRNA catabolic process, exonucleolytic|GO:0044270,cellular nitrogen compound catabolic process|GO:0000398,mRNA splicing, via spliceosome|GO:0043170,macromolecule metabolic process|GO:0000956,nuclear-transcribed mRNA catabolic process|GO:0019439,aromatic compound catabolic process|GO:0006396,RNA processing|GO:0006397,mRNA processing|GO:0022607,cellular component assembly|GO:0009892,negative regulation of metabolic process|GO:0019222,regulation of metabolic process|GO:0043933,macromolecular complex subunit organization|GO:0010629,negative regulation of gene expression|GO:0034622,cellular macromolecular complex assembly|GO:0050789,regulation of biological process|GO:0071840,cellular component organization or biogenesis|GO:0071826,ribonucleoprotein complex subunit organization|GO:0016043,cellular component organization|GO:0065003,macromolecular complex assembly|GO:0022618,ribonucleoprotein complex assembly|GO:0065007,biological regulation|GO:0022613,ribonucleoprotein complex biogenesis|GO:0048519,negative regulation of biological process|GO:0010468,regulation of gene expression|GO:0033962,cytoplasmic mRNA processing body assembly|GO:0060255,regulation of macromolecule metabolic process|GO:0010605,negative regulation of macromolecule metabolic process|GO:0070925,organelle assembly|GO:0006996,organelle organization|GO:0044085,cellular component biogenesis	GO:0005737,cytoplasm|GO:0005829,cytosol|GO:0005622,intracellular|GO:0032991,macromolecular complex|GO:0043231,intracellular membrane-bounded organelle|GO:0071013,catalytic step 2 spliceosome|GO:0044464,cell part|GO:0044444,cytoplasmic part|GO:0005623,cell|GO:0043226,organelle|GO:0044446,intracellular organelle part|GO:0043229,intracellular organelle|GO:0044428,nuclear part|GO:0044422,organelle part|GO:0044424,intracellular part|GO:0030529,intracellular ribonucleoprotein complex|GO:0043227,membrane-bounded organelle|GO:0005634,nucleus|GO:0005681,spliceosomal complex|GO:0035770,ribonucleoprotein granule|GO:1990904,ribonucleoprotein complex|GO:0031974,membrane-enclosed lumen|GO:0043228,non-membrane-bounded organelle|GO:0036464,cytoplasmic ribonucleoprotein granule|GO:0097525,spliceosomal snRNP complex|GO:0031981,nuclear lumen|GO:0005654,nucleoplasm|GO:0046540,U4/U6 x U5 tri-snRNP complex|GO:0071011,precatalytic spliceosome|GO:1990726,Lsm1-7-Pat1 complex|GO:0043234,protein complex|GO:0043232,intracellular non-membrane-bounded organelle|GO:0043233,organelle lumen|GO:0030532,small nuclear ribonucleoprotein complex|GO:0000932,P-body|GO:0070013,intracellular organelle lumen|GO:0097526,spliceosomal tri-snRNP complex|GO:0005688,U6 snRNP	GO:0003676,nucleic acid binding|GO:0097159,organic cyclic compound binding|GO:0044822,poly(A) RNA binding|GO:0003723,RNA binding|GO:0005515,protein binding|GO:1901363,heterocyclic compound binding|GO:0005488,binding
ATP6V1D	GO:0008104,protein localization|GO:0055072,iron ion homeostasis|GO:0006826,iron ion transport|GO:0007165,signal transduction|GO:0007166,cell surface receptor signaling pathway|GO:0055076,transition metal ion homeostasis|GO:0098771,inorganic ion homeostasis|GO:0007169,transmembrane receptor protein tyrosine kinase signaling pathway|GO:0043434,response to peptide hormone|GO:0071840,cellular component organization or biogenesis|GO:0051716,cellular response to stimulus|GO:0070727,cellular macromolecule localization|GO:0048869,cellular developmental process|GO:0044419,interspecies interaction between organisms|GO:0033036,macromolecule localization|GO:0019725,cellular homeostasis|GO:0015992,proton transport|GO:0045184,establishment of protein localization|GO:0051701,interaction with host|GO:0010033,response to organic substance|GO:0051704,multi-organism process|GO:0044700,single organism signaling|GO:0010243,response to organonitrogen compound|GO:0048878,chemical homeostasis|GO:0044782,cilium organization|GO:0022607,cellular component assembly|GO:0007167,enzyme linked receptor protein signaling pathway|GO:0042384,cilium assembly|GO:0050789,regulation of biological process|GO:0046916,cellular transition metal ion homeostasis|GO:0000902,cell morphogenesis|GO:0016043,cellular component organization|GO:0065007,biological regulation|GO:0065008,regulation of biological quality|GO:0048646,anatomical structure formation involved in morphogenesis|GO:0006818,hydrogen transport|GO:0060271,cilium morphogenesis|GO:0006812,cation transport|GO:0006811,ion transport|GO:0006810,transport|GO:0050794,regulation of cellular process|GO:0051234,establishment of localization|GO:1901698,response to nitrogen compound|GO:1901699,cellular response to nitrogen compound|GO:0033572,transferrin transport|GO:0050801,ion homeostasis|GO:0010927,cellular component assembly involved in morphogenesis|GO:0023052,signaling|GO:0070887,cellular response to chemical stimulus|GO:0042221,response to chemical|GO:0009653,anatomical structure morphogenesis|GO:0044699,single-organism process|GO:0009719,response to endogenous stimulus|GO:0071375,cellular response to peptide hormone stimulus|GO:0000041,transition metal ion transport|GO:0015672,monovalent inorganic cation transport|GO:0032502,developmental process|GO:1901700,response to oxygen-containing compound|GO:0008286,insulin receptor signaling pathway|GO:0006879,cellular iron ion homeostasis|GO:0006875,cellular metal ion homeostasis|GO:0009987,cellular process|GO:0006873,cellular ion homeostasis|GO:0032870,cellular response to hormone stimulus|GO:0030001,metal ion transport|GO:0030003,cellular cation homeostasis|GO:0055080,cation homeostasis|GO:0055082,cellular chemical homeostasis|GO:0055085,transmembrane transport|GO:0048858,cell projection morphogenesis|GO:0072512,trivalent inorganic cation transport|GO:0009725,response to hormone|GO:0015682,ferric iron transport|GO:0033365,protein localization to organelle|GO:0050896,response to stimulus|GO:0071495,cellular response to endogenous stimulus|GO:0030030,cell projection organization|GO:0030031,cell projection assembly|GO:0042592,homeostatic process|GO:0061512,protein localization to cilium|GO:0032869,cellular response to insulin stimulus|GO:0032868,response to insulin|GO:0071417,cellular response to organonitrogen compound|GO:0090382,phagosome maturation|GO:0032989,cellular component morphogenesis|GO:0071310,cellular response to organic substance|GO:0071702,organic substance transport|GO:0034613,cellular protein localization|GO:0044767,single-organism developmental process|GO:0034220,ion transmembrane transport|GO:0044765,single-organism transport|GO:0044763,single-organism cellular process|GO:0055065,metal ion homeostasis|GO:0007154,cell communication|GO:0070925,organelle assembly|GO:0051179,localization|GO:1902578,single-organism localization|GO:0051641,cellular localization|GO:0006996,organelle organization|GO:1901701,cellular response to oxygen-containing compound|GO:0032990,cell part morphogenesis|GO:0048856,anatomical structure development|GO:1902589,single-organism organelle organization|GO:0044085,cellular component biogenesis|GO:1901652,response to peptide|GO:1901653,cellular response to peptide|GO:0015031,protein transport|GO:0044403,symbiosis, encompassing mutualism through parasitism|GO:0019222,regulation of metabolic process|GO:0090383,phagosome acidification|GO:0002263,cell activation involved in immune response|GO:0045851,pH reduction|GO:0016192,vesicle-mediated transport|GO:0002275,myeloid cell activation involved in immune response|GO:0002274,myeloid leukocyte activation|GO:0002376,immune system process|GO:0009894,regulation of catabolic process|GO:0045321,leukocyte activation|GO:0032940,secretion by cell|GO:0002283,neutrophil activation involved in immune response|GO:0006885,regulation of pH|GO:0006887,exocytosis|GO:0045055,regulated exocytosis|GO:0002366,leukocyte activation involved in immune response|GO:0001775,cell activation|GO:0008152,metabolic process|GO:0006955,immune response|GO:0046903,secretion|GO:0043312,neutrophil degranulation|GO:0044248,cellular catabolic process|GO:0015833,peptide transport|GO:0051453,regulation of intracellular pH|GO:0051452,intracellular pH reduction|GO:0042886,amide transport|GO:0002444,myeloid leukocyte mediated immunity|GO:0002446,neutrophil mediated immunity|GO:0016236,macroautophagy|GO:0030004,cellular monovalent inorganic cation homeostasis|GO:0036230,granulocyte activation|GO:0031329,regulation of cellular catabolic process|GO:0042119,neutrophil activation|GO:0031323,regulation of cellular metabolic process|GO:0030641,regulation of cellular pH|GO:0071705,nitrogen compound transport|GO:0010506,regulation of autophagy|GO:0016241,regulation of macroautophagy|GO:0006914,autophagy|GO:0055067,monovalent inorganic cation homeostasis|GO:0002443,leukocyte mediated immunity|GO:0009056,catabolic process|GO:0044237,cellular metabolic process|GO:0043299,leukocyte degranulation|GO:0002252,immune	GO:0000323,lytic vacuole|GO:0033176,proton-transporting V-type ATPase complex|GO:0043229,intracellular organelle|GO:0043228,non-membrane-bounded organelle|GO:0005929,cilium|GO:0031988,membrane-bounded vesicle|GO:0043227,membrane-bounded organelle|GO:0043226,organelle|GO:0005856,cytoskeleton|GO:0005764,lysosome|GO:0042995,cell projection|GO:0031982,vesicle|GO:0098796,membrane protein complex|GO:0005773,vacuole|GO:0005813,centrosome|GO:0098805,whole membrane|GO:0016020,membrane|GO:0005774,vacuolar membrane|GO:0044430,cytoskeletal part|GO:0044437,vacuolar part|GO:0005737,cytoplasm|GO:0015630,microtubule cytoskeleton|GO:0098588,bounding membrane of organelle|GO:0032991,macromolecular complex|GO:1903561,extracellular vesicle|GO:0070062,extracellular exosome|GO:0043234,protein complex|GO:0043230,extracellular organelle|GO:0043231,intracellular membrane-bounded organelle|GO:0043232,intracellular non-membrane-bounded organelle|GO:0065010,extracellular membrane-bounded organelle|GO:0005829,cytosol|GO:0044464,cell part|GO:0005623,cell|GO:0005622,intracellular|GO:0044446,intracellular organelle part|GO:0005765,lysosomal membrane|GO:0044444,cytoplasmic part|GO:0005576,extracellular region|GO:0031090,organelle membrane|GO:0044424,intracellular part|GO:0044425,membrane part|GO:0016469,proton-transporting two-sector ATPase complex|GO:0005815,microtubule organizing center|GO:0044421,extracellular region part|GO:0044422,organelle part|GO:0035579,specific granule membrane|GO:0030667,secretory granule membrane|GO:0044433,cytoplasmic vesicle part|GO:0099503,secretory vesicle|GO:0016023,cytoplasmic, membrane-bounded vesicle|GO:0030141,secretory granule|GO:0071944,cell periphery|GO:0042581,specific granule|GO:0030659,cytoplasmic vesicle membrane|GO:0097708,intracellular vesicle|GO:0012506,vesicle membrane|GO:0005886,plasma membrane|GO:0031410,cytoplasmic vesicle|GO:0098852,lytic vacuole	GO:0016787,hydrolase activity|GO:0042626,ATPase activity, coupled to transmembrane movement of substances|GO:0016820,hydrolase activity, acting on acid anhydrides, catalyzing transmembrane movement of substances|GO:0016818,hydrolase activity, acting on acid anhydrides, in phosphorus-containing anhydrides|GO:0042623,ATPase activity, coupled|GO:0005215,transporter activity|GO:0043492,ATPase activity, coupled to movement of substances|GO:0017111,nucleoside-triphosphatase activity|GO:0003824,catalytic activity|GO:0016817,hydrolase activity, acting on acid anhydrides|GO:0015399,primary active transmembrane transporter activity|GO:0022857,transmembrane transporter activity|GO:0005515,protein binding|GO:0016887,ATPase activity|GO:0016462,pyrophosphatase activity|GO:0015405,P-P-bond-hydrolysis-driven transmembrane transporter activity|GO:0022804,active transmembrane transporter activity|GO:0005488,binding
CDKN2C	GO:0043549,regulation of kinase activity|GO:0019220,regulation of phosphate metabolic process|GO:0080090,regulation of primary metabolic process|GO:0019222,regulation of metabolic process|GO:0000082,G1/S transition of mitotic cell cycle|GO:0071840,cellular component organization or biogenesis|GO:0010605,negative regulation of macromolecule metabolic process|GO:0048869,cellular developmental process|GO:0044092,negative regulation of molecular function|GO:0048519,negative regulation of biological process|GO:0042127,regulation of cell proliferation|GO:0060255,regulation of macromolecule metabolic process|GO:0045859,regulation of protein kinase activity|GO:0042325,regulation of phosphorylation|GO:0042326,negative regulation of phosphorylation|GO:0044707,single-multicellular organism process|GO:0019538,protein metabolic process|GO:1904029,regulation of cyclin-dependent protein kinase activity|GO:0033673,negative regulation of kinase activity|GO:0009892,negative regulation of metabolic process|GO:0071901,negative regulation of protein serine/threonine kinase activity|GO:0071900,regulation of protein serine/threonine kinase activity|GO:0050789,regulation of biological process|GO:0044267,cellular protein metabolic process|GO:0016049,cell growth|GO:0044260,cellular macromolecule metabolic process|GO:0051348,negative regulation of transferase activity|GO:0016043,cellular component organization|GO:0065007,biological regulation|GO:0007049,cell cycle|GO:0065009,regulation of molecular function|GO:0050790,regulation of catalytic activity|GO:0048709,oligodendrocyte differentiation|GO:0042063,gliogenesis|GO:0050794,regulation of cellular process|GO:0007050,cell cycle arrest|GO:0043412,macromolecule modification|GO:0036211,protein modification process|GO:0008152,metabolic process|GO:0045786,negative regulation of cell cycle|GO:0044767,single-organism developmental process|GO:0031400,negative regulation of protein modification process|GO:0051338,regulation of transferase activity|GO:0030308,negative regulation of cell growth|GO:0016310,phosphorylation|GO:0030154,cell differentiation|GO:0051128,regulation of cellular component organization|GO:0010001,glial cell differentiation|GO:0043086,negative regulation of catalytic activity|GO:0044699,single-organism process|GO:0007417,central nervous system development|GO:0051248,negative regulation of protein metabolic process|GO:0010563,negative regulation of phosphorus metabolic process|GO:0051246,regulation of protein metabolic process|GO:0031399,regulation of protein modification process|GO:0032502,developmental process|GO:0008285,negative regulation of cell proliferation|GO:0032501,multicellular organismal process|GO:0008283,cell proliferation|GO:0009987,cellular process|GO:0001558,regulation of cell growth|GO:0032269,negative regulation of cellular protein metabolic process|GO:0032268,regulation of cellular protein metabolic process|GO:0043170,macromolecule metabolic process|GO:0048731,system development|GO:0045926,negative regulation of growth|GO:0031324,negative regulation of cellular metabolic process|GO:0031323,regulation of cellular metabolic process|GO:1903047,mitotic cell cycle process|GO:0044770,cell cycle phase transition|GO:0044772,mitotic cell cycle phase transition|GO:0022402,cell cycle process|GO:0007275,multicellular organism development|GO:0040007,growth|GO:0040008,regulation of growth|GO:0071704,organic substance metabolic process|GO:0006468,protein phosphorylation|GO:0006469,negative regulation of protein kinase activity|GO:0000278,mitotic cell cycle|GO:0045936,negative regulation of phosphate metabolic process|GO:0006464,cellular protein modification process|GO:0051174,regulation of phosphorus metabolic process|GO:0044763,single-organism cellular process|GO:0044843,cell cycle G1/S phase transition|GO:0022008,neurogenesis|GO:0044238,primary metabolic process|GO:0007399,nervous system development|GO:0051726,regulation of cell cycle|GO:0000079,regulation of cyclin-dependent protein serine/threonine kinase activity|GO:0048856,anatomical structure development|GO:0044237,cellular metabolic process|GO:0006796,phosphate-containing compound metabolic process|GO:0006793,phosphorus metabolic process|GO:0001933,negative regulation of protein phosphorylation|GO:0001932,regulation of protein phosphorylation|GO:0048523,negative regulation of cellular process|GO:1901564,organonitrogen compound metabolic process|GO:0006807,nitrogen compound metabolic process|GO:1904030,negative regulation of cyclin-dependent protein kinase activity|GO:0045736,negative regulation of cyclin-dependent protein serine/threonine kinase activity|GO:0007346,regulation of mitotic cell cycle|GO:0031657,regulation of cyclin-dependent protein serine/threonine kinase activity involved in G1/S transition of mitotic cell cycle|GO:2000045,regulation of G1/S transition of mitotic cell cycle|GO:0031658,negative regulation of cyclin-dependent protein serine/threonine kinase activity involved in G1/S transition of mitotic cell cycle|GO:0010564,regulation of cell cycle process|GO:1902806,regulation of cell cycle G1/S phase transition|GO:1901987,regulation of cell cycle phase transition|GO:1901990,regulation of mitotic cell cycle phase transition	GO:0005737,cytoplasm|GO:0043231,intracellular membrane-bounded organelle|GO:0005829,cytosol|GO:0044464,cell part|GO:0044444,cytoplasmic part|GO:0005623,cell|GO:0043226,organelle|GO:0043229,intracellular organelle|GO:0044424,intracellular part|GO:0005622,intracellular|GO:0043227,membrane-bounded organelle|GO:0005634,nucleus	GO:0019210,kinase inhibitor activity|GO:0019207,kinase regulator activity|GO:0019900,kinase binding|GO:0019887,protein kinase regulator activity|GO:0019901,protein kinase binding|GO:0030234,enzyme regulator activity|GO:0016538,cyclin-dependent protein serine/threonine kinase regulator activity|GO:0019899,enzyme binding|GO:0004860,protein kinase inhibitor activity|GO:0004861,cyclin-dependent protein serine/threonine kinase inhibitor activity|GO:0005488,binding|GO:0005515,protein binding|GO:0030291,protein serine/threonine kinase inhibitor activity|GO:0004857,enzyme inhibitor activity|GO:0098772,molecular function regulator
NMNAT2	GO:0019674,NAD metabolic process|GO:0006732,coenzyme metabolic process|GO:0006733,oxidoreduction coenzyme metabolic process|GO:0046496,nicotinamide nucleotide metabolic process|GO:0009435,NAD biosynthetic process|GO:0034641,cellular nitrogen compound metabolic process|GO:0009165,nucleotide biosynthetic process|GO:0009108,coenzyme biosynthetic process|GO:0044281,small molecule metabolic process|GO:0072524,pyridine-containing compound metabolic process|GO:0072525,pyridine-containing compound biosynthetic process|GO:0044699,single-organism process|GO:0006139,nucleobase-containing compound metabolic process|GO:0044710,single-organism metabolic process|GO:0044711,single-organism biosynthetic process|GO:1901362,organic cyclic compound biosynthetic process|GO:0051188,cofactor biosynthetic process|GO:0071704,organic substance metabolic process|GO:1901360,organic cyclic compound metabolic process|GO:0019359,nicotinamide nucleotide biosynthetic process|GO:0018130,heterocycle biosynthetic process|GO:0006767,water-soluble vitamin metabolic process|GO:0006766,vitamin metabolic process|GO:1901576,organic substance biosynthetic process|GO:0009987,cellular process|GO:0006725,cellular aromatic compound metabolic process|GO:0009058,biosynthetic process|GO:0051186,cofactor metabolic process|GO:0044763,single-organism cellular process|GO:0008152,metabolic process|GO:0019438,aromatic compound biosynthetic process|GO:0034654,nucleobase-containing compound biosynthetic process|GO:1901564,organonitrogen compound metabolic process|GO:0090407,organophosphate biosynthetic process|GO:0055086,nucleobase-containing small molecule metabolic process|GO:0046483,heterocycle metabolic process|GO:0044238,primary metabolic process|GO:0044271,cellular nitrogen compound biosynthetic process|GO:1901566,organonitrogen compound biosynthetic process|GO:0044249,cellular biosynthetic process|GO:0044237,cellular metabolic process|GO:0006796,phosphate-containing compound metabolic process|GO:0006807,nitrogen compound metabolic process|GO:1901293,nucleoside phosphate biosynthetic process|GO:0006793,phosphorus metabolic process|GO:0019637,organophosphate metabolic process|GO:0009117,nucleotide metabolic process|GO:0006753,nucleoside phosphate metabolic process|GO:0019363,pyridine nucleotide biosynthetic process|GO:0019362,pyridine nucleotide metabolic process|GO:0019752,carboxylic acid metabolic process|GO:0034627,'de novo' NAD biosynthetic process|GO:0034628,'de novo' NAD biosynthetic process from aspartate|GO:0006520,cellular amino acid metabolic process|GO:0019355,nicotinamide nucleotide biosynthetic process from aspartate|GO:1901605,alpha-amino acid metabolic process|GO:0006531,aspartate metabolic process|GO:0043436,oxoacid metabolic process|GO:0043648,dicarboxylic acid metabolic process|GO:0006082,organic acid metabolic process|GO:0009066,aspartate family amino acid metabolic process	GO:0016020,membrane|GO:0043229,intracellular organelle|GO:0045202,synapse|GO:0005622,intracellular|GO:0043227,membrane-bounded organelle|GO:0043226,organelle|GO:0005737,cytoplasm|GO:0031984,organelle subcompartment|GO:0031090,organelle membrane|GO:0005770,late endosome|GO:0044431,Golgi apparatus part|GO:0005794,Golgi apparatus|GO:0098588,bounding membrane of organelle|GO:0012505,endomembrane system|GO:0043231,intracellular membrane-bounded organelle|GO:0005802,trans-Golgi network|GO:0044464,cell part|GO:0005623,cell|GO:0000139,Golgi membrane|GO:0044446,intracellular organelle part|GO:0044444,cytoplasmic part|GO:0098791,Golgi subcompartment|GO:0044424,intracellular part|GO:0005768,endosome|GO:0044422,organelle part|GO:0031410,cytoplasmic vesicle|GO:0097708,intracellular vesicle|GO:0031982,vesicle|GO:0012506,vesicle membrane|GO:0097458,neuron part|GO:0030424,axon|GO:0044433,cytoplasmic vesicle part|GO:0030659,cytoplasmic vesicle membrane|GO:0043005,neuron projection	GO:0000309,nicotinamide-nucleotide adenylyltransferase activity|GO:0035639,purine ribonucleoside triphosphate binding|GO:0005488,binding|GO:0043167,ion binding|GO:1901363,heterocyclic compound binding|GO:1901265,nucleoside phosphate binding|GO:0001882,nucleoside binding|GO:0043168,anion binding|GO:0000166,nucleotide binding|GO:0016740,transferase activity|GO:0001883,purine nucleoside binding|GO:0032549,ribonucleoside binding|GO:0017076,purine nucleotide binding|GO:0005524,ATP binding|GO:0036094,small molecule binding|GO:0003824,catalytic activity|GO:0016779,nucleotidyltransferase activity|GO:0004515,nicotinate-nucleotide adenylyltransferase activity|GO:0030554,adenyl nucleotide binding|GO:0097367,carbohydrate derivative binding|GO:0016772,transferase activity, transferring phosphorus-containing groups|GO:0070566,adenylyltransferase activity|GO:0032559,adenyl ribonucleotide binding|GO:0097159,organic cyclic compound binding|GO:0032555,purine ribonucleotide binding|GO:0032550,purine ribonucleoside binding|GO:0032553,ribonucleotide binding
MYL6B	GO:0006928,movement of cell or subcellular component|GO:0003012,muscle system process|GO:0061061,muscle structure development|GO:0007275,multicellular organism development|GO:0044699,single-organism process|GO:0007517,muscle organ development|GO:0030029,actin filament-based process|GO:0007519,skeletal muscle tissue development|GO:0033275,actin-myosin filament sliding|GO:0048513,animal organ development|GO:0032502,developmental process|GO:0032501,multicellular organismal process|GO:0006936,muscle contraction|GO:0009987,cellular process|GO:0009888,tissue development|GO:0030049,muscle filament sliding|GO:0030048,actin filament-based movement|GO:0044763,single-organism cellular process|GO:0003008,system process|GO:0044767,single-organism developmental process|GO:0044707,single-multicellular organism process|GO:0048856,anatomical structure development|GO:0014706,striated muscle tissue development|GO:0060537,muscle tissue development|GO:0060538,skeletal muscle organ development|GO:0048731,system development|GO:0070252,actin-mediated cell contraction	GO:0015629,actin cytoskeleton|GO:0043229,intracellular organelle|GO:0043228,non-membrane-bounded organelle|GO:0044430,cytoskeletal part|GO:0031988,membrane-bounded vesicle|GO:0043227,membrane-bounded organelle|GO:0043226,organelle|GO:0005856,cytoskeleton|GO:0031982,vesicle|GO:0044444,cytoplasmic part|GO:0005859,muscle myosin complex|GO:0016459,myosin complex|GO:0005576,extracellular region|GO:0005737,cytoplasm|GO:0044424,intracellular part|GO:0032991,macromolecular complex|GO:1903561,extracellular vesicle|GO:0070062,extracellular exosome|GO:0043234,protein complex|GO:0043230,extracellular organelle|GO:0043232,intracellular non-membrane-bounded organelle|GO:0005829,cytosol|GO:0044464,cell part|GO:0005623,cell|GO:0005622,intracellular|GO:0044446,intracellular organelle part|GO:0016461,unconventional myosin complex|GO:0016460,myosin II complex|GO:0043292,contractile fiber|GO:0065010,extracellular membrane-bounded organelle|GO:0044421,extracellular region part|GO:0044422,organelle part|GO:0044449,contractile fiber part|GO:0099512,supramolecular fiber	GO:0016787,hydrolase activity|GO:0016818,hydrolase activity, acting on acid anhydrides, in phosphorus-containing anhydrides|GO:0003824,catalytic activity|GO:0043167,ion binding|GO:0017111,nucleoside-triphosphatase activity|GO:0016817,hydrolase activity, acting on acid anhydrides|GO:0005198,structural molecule activity|GO:0016462,pyrophosphatase activity|GO:0008307,structural constituent of muscle|GO:0043169,cation binding|GO:0005509,calcium ion binding|GO:0005515,protein binding|GO:0046872,metal ion binding|GO:0003774,motor activity|GO:0005488,binding
CYSTM1	GO:0006887,exocytosis|GO:0042119,neutrophil activation|GO:0001775,cell activation|GO:0045321,leukocyte activation|GO:0032940,secretion by cell|GO:0044699,single-organism process|GO:0002283,neutrophil activation involved in immune response|GO:0002443,leukocyte mediated immunity|GO:0002263,cell activation involved in immune response|GO:0045055,regulated exocytosis|GO:0002366,leukocyte activation involved in immune response|GO:0002446,neutrophil mediated immunity|GO:0009987,cellular process|GO:0006810,transport|GO:0044765,single-organism transport|GO:0036230,granulocyte activation|GO:0006955,immune response|GO:0051234,establishment of localization|GO:0002444,myeloid leukocyte mediated immunity|GO:0051179,localization|GO:1902578,single-organism localization|GO:0046903,secretion|GO:0016192,vesicle-mediated transport|GO:0002275,myeloid cell activation involved in immune response|GO:0002274,myeloid leukocyte activation|GO:0043312,neutrophil degranulation|GO:0002376,immune system process|GO:0043299,leukocyte degranulation|GO:0002252,immune effector process|GO:0044763,single-organism cellular process|GO:0050896,response to stimulus	GO:0043230,extracellular organelle|GO:0031982,vesicle|GO:0044421,extracellular region part|GO:0043227,membrane-bounded organelle|GO:0016021,integral component of membrane|GO:0016020,membrane|GO:0031988,membrane-bounded vesicle|GO:0005576,extracellular region|GO:0044425,membrane part|GO:1903561,extracellular vesicle|GO:0065010,extracellular membrane-bounded organelle|GO:0043226,organelle|GO:0031224,intrinsic component of membrane|GO:0070062,extracellular exosome|GO:0043229,intracellular organelle|GO:0071944,cell periphery|GO:0005737,cytoplasm|GO:0030667,secretory granule membrane|GO:0070820,tertiary granule|GO:0016023,cytoplasmic, membrane-bounded vesicle|GO:0031410,cytoplasmic vesicle|GO:0044433,cytoplasmic vesicle part|GO:0099503,secretory vesicle|GO:0030141,secretory granule|GO:0098588,bounding membrane of organelle|GO:0070821,tertiary granule membrane|GO:0030659,cytoplasmic vesicle membrane|GO:0097708,intracellular vesicle|GO:0012506,vesicle membrane|GO:0005886,plasma membrane|GO:0031090,organelle membrane|GO:0043231,intracellular membrane-bounded organelle|GO:0044464,cell part|GO:0005623,cell|GO:0005622,intracellular|GO:0044446,intracellular organelle part|GO:0044444,cytoplasmic part|GO:0044424,intracellular part|GO:0098805,whole membrane|GO:0044422,organelle part|GO:0012505,endomembrane system	
PGPEP1L	GO:0044238,primary metabolic process|GO:1901564,organonitrogen compound metabolic process|GO:0019538,protein metabolic process|GO:0043170,macromolecule metabolic process|GO:0071704,organic substance metabolic process|GO:0006807,nitrogen compound metabolic process|GO:0008152,metabolic process|GO:0006508,proteolysis	GO:0005737,cytoplasm|GO:0005829,cytosol|GO:0044464,cell part|GO:0005623,cell|GO:0005622,intracellular|GO:0044444,cytoplasmic part|GO:0044424,intracellular part	GO:0016787,hydrolase activity|GO:0003824,catalytic activity|GO:0008242,omega peptidase activity|GO:0008238,exopeptidase activity|GO:0070011,peptidase activity, acting on L-amino acid peptides|GO:0008233,peptidase activity|GO:0016920,pyroglutamyl-peptidase activity
PPWD1	GO:0044267,cellular protein metabolic process|GO:0000413,protein peptidyl-prolyl isomerization|GO:0018208,peptidyl-proline modification|GO:0044260,cellular macromolecule metabolic process|GO:0044238,primary metabolic process|GO:0019538,protein metabolic process|GO:0044237,cellular metabolic process|GO:0009987,cellular process|GO:0006464,cellular protein modification process|GO:0043170,macromolecule metabolic process|GO:0071704,organic substance metabolic process|GO:0043412,macromolecule modification|GO:0036211,protein modification process|GO:0018193,peptidyl-amino acid modification|GO:0008152,metabolic process		GO:0003824,catalytic activity|GO:0016853,isomerase activity|GO:0003755,peptidyl-prolyl cis-trans isomerase activity|GO:0016859,cis-trans isomerase activity
MAB21L1	GO:0042127,regulation of cell proliferation|GO:0009653,anatomical structure morphogenesis|GO:0007275,multicellular organism development|GO:0044699,single-organism process|GO:0050789,regulation of biological process|GO:0048513,animal organ development|GO:0065007,biological regulation|GO:0048518,positive regulation of biological process|GO:0008284,positive regulation of cell proliferation|GO:0032501,multicellular organismal process|GO:0008283,cell proliferation|GO:0009987,cellular process|GO:0050794,regulation of cellular process|GO:0044767,single-organism developmental process|GO:0001654,eye development|GO:0007423,sensory organ development|GO:0044707,single-multicellular organism process|GO:0048856,anatomical structure development|GO:0032502,developmental process|GO:0048731,system development|GO:0043010,camera-type eye development|GO:0048522,positive regulation of cellular process	GO:0043231,intracellular membrane-bounded organelle|GO:0005634,nucleus|GO:0044464,cell part|GO:0005623,cell|GO:0005622,intracellular|GO:0043229,intracellular organelle|GO:0044424,intracellular part|GO:0043227,membrane-bounded organelle|GO:0043226,organelle	GO:0005488,binding|GO:0005515,protein binding|GO:0035639,purine ribonucleoside triphosphate binding|GO:0043167,ion binding|GO:1901363,heterocyclic compound binding|GO:1901265,nucleoside phosphate binding|GO:0001882,nucleoside binding|GO:0043168,anion binding|GO:0043169,cation binding|GO:0000166,nucleotide binding|GO:0032561,guanyl ribonucleotide binding|GO:0016740,transferase activity|GO:0001883,purine nucleoside binding|GO:0032549,ribonucleoside binding|GO:0017076,purine nucleotide binding|GO:0046872,metal ion binding|GO:0005524,ATP binding|GO:0005525,GTP binding|GO:0036094,small molecule binding|GO:0003824,catalytic activity|GO:0016779,nucleotidyltransferase activity|GO:0030554,adenyl nucleotide binding|GO:0097367,carbohydrate derivative binding|GO:0016772,transferase activity, transferring phosphorus-containing groups|GO:0019001,guanyl nucleotide binding|GO:0032559,adenyl ribonucleotide binding|GO:0097159,organic cyclic compound binding|GO:0032555,purine ribonucleotide binding|GO:0032550,purine ribonucleoside

B3GALT2	GO:0009311,oligosaccharide metabolic process|GO:0009312,oligosaccharide biosynthetic process|GO:0044249,cellular biosynthetic process|GO:0034645,cellular macromolecule biosynthetic process|GO:0043170,macromolecule metabolic process|GO:0009100,glycoprotein metabolic process|GO:0009101,glycoprotein biosynthetic process|GO:0044699,single-organism process|GO:0044267,cellular protein metabolic process|GO:0044710,single-organism metabolic process|GO:0044711,single-organism biosynthetic process|GO:0044260,cellular macromolecule metabolic process|GO:0071704,organic substance metabolic process|GO:1901576,organic substance biosynthetic process|GO:0009987,cellular process|GO:0070085,glycosylation|GO:0006464,cellular protein modification process|GO:0043412,macromolecule modification|GO:0036211,protein modification process|GO:0044763,single-organism cellular process|GO:0008152,metabolic process|GO:0044723,single-organism carbohydrate metabolic process|GO:0009059,macromolecule biosynthetic process|GO:0044238,primary metabolic process|GO:0005975,carbohydrate metabolic process|GO:0006486,protein glycosylation|GO:0019538,protein metabolic process|GO:1901135,carbohydrate derivative metabolic process|GO:0009058,biosynthetic process|GO:0044237,cellular metabolic process|GO:0016051,carbohydrate biosynthetic process|GO:1901137,carbohydrate derivative biosynthetic process|GO:0043413,macromolecule glycosylation|GO:0006807,nitrogen compound metabolic process|GO:1901564,organonitrogen compound metabolic process|GO:1901566,organonitrogen compound biosynthetic process|GO:0034641,cellular nitrogen compound metabolic process|GO:0006629,lipid metabolic process|GO:0006682,galactosylceramide biosynthetic process|GO:0006681,galactosylceramide metabolic process|GO:0006687,glycosphingolipid metabolic process|GO:0046467,membrane lipid biosynthetic process|GO:0019374,galactolipid metabolic process|GO:0019375,galactolipid biosynthetic process|GO:0006664,glycolipid metabolic process|GO:0006665,sphingolipid metabolic process|GO:0030148,sphingolipid biosynthetic process|GO:1903509,liposaccharide metabolic process|GO:0006643,membrane lipid metabolic process|GO:0009247,glycolipid biosynthetic process|GO:0044255,cellular lipid metabolic process|GO:0006688,glycosphingolipid biosynthetic process|GO:0008610,lipid biosynthetic process|GO:0043604,amide biosynthetic process|GO:0044271,cellular nitrogen compound biosynthetic process|GO:0043603,cellular amide metabolic process|GO:0046513,ceramide biosynthetic process|GO:0006677,glycosylceramide metabolic process|GO:0046476,glycosylceramide biosynthetic process|GO:0006672,ceramide metabolic process	GO:0043229,intracellular organelle|GO:0005622,intracellular|GO:0043227,membrane-bounded organelle|GO:0043226,organelle|GO:0031224,intrinsic component of membrane|GO:0005737,cytoplasm|GO:0031090,organelle membrane|GO:0016021,integral component of membrane|GO:0016020,membrane|GO:0044431,Golgi apparatus part|GO:0005794,Golgi apparatus|GO:0098588,bounding membrane of organelle|GO:0012505,endomembrane system|GO:0043231,intracellular membrane-bounded organelle|GO:0044464,cell part|GO:0005623,cell|GO:0000139,Golgi membrane|GO:0044446,intracellular organelle part|GO:0044444,cytoplasmic part|GO:0044424,intracellular part|GO:0044425,membrane part|GO:0044422,organelle part|GO:0031984,organelle subcompartment|GO:0098791,Golgi subcompartment	GO:0035250,UDP-galactosyltransferase activity|GO:0003824,catalytic activity|GO:0016740,transferase activity|GO:0008378,galactosyltransferase activity|GO:0016757,transferase activity, transferring glycosyl groups|GO:0008499,UDP-galactose:beta-N-acetylglucosamine beta-1,3-galactosyltransferase activity|GO:0016758,transferase activity, transferring hexosyl groups|GO:0008194,UDP-glycosyltransferase activity|GO:0048531,beta-1,3-galactosyltransferase activity|GO:0047275,glucosaminylgalactosylglucosylceramide beta-galactosyltransferase activity
IMPA1	GO:0006650,glycerophospholipid metabolic process|GO:0016311,dephosphorylation|GO:0016310,phosphorylation|GO:0046173,polyol biosynthetic process|GO:0046174,polyol catabolic process|GO:0019751,polyol metabolic process|GO:1901615,organic hydroxy compound metabolic process|GO:0044249,cellular biosynthetic process|GO:0023052,signaling|GO:0007165,signal transduction|GO:0044282,small molecule catabolic process|GO:0044283,small molecule biosynthetic process|GO:0009056,catabolic process|GO:1901576,organic substance biosynthetic process|GO:0044255,cellular lipid metabolic process|GO:0050789,regulation of biological process|GO:0044699,single-organism process|GO:0046434,organophosphate catabolic process|GO:0044712,single-organism catabolic process|GO:0045017,glycerolipid biosynthetic process|GO:0044710,single-organism metabolic process|GO:0044711,single-organism biosynthetic process|GO:0044262,cellular carbohydrate metabolic process|GO:1901575,organic substance catabolic process|GO:0043647,inositol phosphate metabolic process|GO:0090407,organophosphate biosynthetic process|GO:0046834,lipid phosphorylation|GO:0065007,biological regulation|GO:0046855,inositol phosphate dephosphorylation|GO:0046854,phosphatidylinositol phosphorylation|GO:0006661,phosphatidylinositol biosynthetic process|GO:0044237,cellular metabolic process|GO:0046838,phosphorylated carbohydrate dephosphorylation|GO:0006644,phospholipid metabolic process|GO:0009987,cellular process|GO:0006629,lipid metabolic process|GO:0044238,primary metabolic process|GO:0046165,alcohol biosynthetic process|GO:0046164,alcohol catabolic process|GO:0051716,cellular response to stimulus|GO:0050794,regulation of cellular process|GO:0034637,cellular carbohydrate biosynthetic process|GO:0009058,biosynthetic process|GO:0046488,phosphatidylinositol metabolic process|GO:0008152,metabolic process|GO:0044723,single-organism carbohydrate metabolic process|GO:0007154,cell communication|GO:0046486,glycerolipid metabolic process|GO:0030258,lipid modification|GO:0008610,lipid biosynthetic process|GO:0071704,organic substance metabolic process|GO:0044700,single organism signaling|GO:0005975,carbohydrate metabolic process|GO:0008654,phospholipid biosynthetic process|GO:0050896,response to stimulus|GO:0044763,single-organism cellular process|GO:0071545,inositol phosphate catabolic process|GO:0016051,carbohydrate biosynthetic process|GO:0006066,alcohol metabolic process|GO:0006796,phosphate-containing compound metabolic process|GO:0006793,phosphorus metabolic process|GO:0019637,organophosphate metabolic process|GO:0046474,glycerophospholipid biosynthetic process|GO:0006020,inositol metabolic process|GO:0006021,inositol biosynthetic process|GO:1901616,organic hydroxy compound catabolic process|GO:1901617,organic hydroxy compound biosynthetic process|GO:0044281,small molecule metabolic process	GO:0005737,cytoplasm|GO:0005622,intracellular|GO:0043230,extracellular organelle|GO:0031982,vesicle|GO:0044421,extracellular region part|GO:0043227,membrane-bounded organelle|GO:0005829,cytosol|GO:0044464,cell part|GO:0005623,cell|GO:0031988,membrane-bounded vesicle|GO:0044444,cytoplasmic part|GO:0005576,extracellular region|GO:0044424,intracellular part|GO:1903561,extracellular vesicle|GO:0065010,extracellular membrane-bounded organelle|GO:0043226,organelle|GO:0070062,extracellular exosome	GO:0016791,phosphatase activity|GO:0042578,phosphoric ester hydrolase activity|GO:0005488,binding|GO:0043169,cation binding|GO:0046914,transition metal ion binding|GO:0000287,magnesium ion binding|GO:0043167,ion binding|GO:0052834,inositol monophosphate phosphatase activity|GO:0052832,inositol monophosphate 3-phosphatase activity|GO:0052833,inositol monophosphate 4-phosphatase activity|GO:0046872,metal ion binding|GO:0042802,identical protein binding|GO:0042803,protein homodimerization activity|GO:0016787,hydrolase activity|GO:0008934,inositol monophosphate 1-phosphatase activity|GO:0030145,manganese ion binding|GO:0016788,hydrolase activity, acting on ester bonds|GO:0003824,catalytic activity|GO:0031403,lithium ion binding|GO:0031420,alkali metal ion binding|GO:0052745,inositol phosphate phosphatase activity|GO:0005515,protein binding|GO:0046983,protein dimerization activity
C4orf32			
DPY19L3	GO:0018103,protein C-linked glycosylation|GO:0044249,cellular biosynthetic process|GO:0034645,cellular macromolecule biosynthetic process|GO:0035268,protein mannosylation|GO:0009100,glycoprotein metabolic process|GO:0009101,glycoprotein biosynthetic process|GO:0044699,single-organism process|GO:0044267,cellular protein metabolic process|GO:0044710,single-organism metabolic process|GO:0006486,protein glycosylation|GO:0018317,protein C-linked glycosylation via tryptophan|GO:0018193,peptidyl-amino acid modification|GO:0018211,peptidyl-tryptophan modification|GO:0071704,organic substance metabolic process|GO:0018406,protein C-linked glycosylation via 2'-alpha-mannosyl-L-tryptophan|GO:1901576,organic substance biosynthetic process|GO:0009987,cellular process|GO:0070085,glycosylation|GO:0006464,cellular protein modification process|GO:0009058,biosynthetic process|GO:0043412,macromolecule modification|GO:0043413,macromolecule glycosylation|GO:0044763,single-organism cellular process|GO:0008152,metabolic process|GO:0044723,single-organism carbohydrate metabolic process|GO:0009059,macromolecule biosynthetic process|GO:0044238,primary metabolic process|GO:0005975,carbohydrate metabolic process|GO:0044260,cellular macromolecule metabolic process|GO:0019538,protein metabolic process|GO:1901135,carbohydrate derivative metabolic process|GO:0097502,mannosylation|GO:0044237,cellular metabolic process|GO:0043170,macromolecule metabolic process|GO:1901137,carbohydrate derivative biosynthetic process|GO:0036211,protein modification process|GO:0006807,nitrogen compound metabolic process|GO:1901564,organonitrogen compound metabolic process|GO:1901566,organonitrogen compound biosynthetic process	GO:0005635,nuclear envelope|GO:0044464,cell part|GO:0031975,envelope|GO:0043229,intracellular organelle|GO:0043227,membrane-bounded organelle|GO:0043226,organelle|GO:0031224,intrinsic component of membrane|GO:0031090,organelle membrane|GO:0005637,nuclear inner membrane|GO:0016021,integral component of membrane|GO:0016020,membrane|GO:0031965,nuclear membrane|GO:0031967,organelle envelope|GO:0012505,endomembrane system|GO:0043231,intracellular membrane-bounded organelle|GO:0019866,organelle inner membrane|GO:0005623,cell|GO:0005622,intracellular|GO:0044446,intracellular organelle part|GO:0044428,nuclear part|GO:0044424,intracellular part|GO:0044425,membrane part|GO:0005634,nucleus|GO:0044422,organelle part	GO:0003824,catalytic activity|GO:0016740,transferase activity|GO:0016757,transferase activity, transferring glycosyl groups|GO:0016758,transferase activity, transferring hexosyl groups|GO:0000030,mannosyltransferase activity
ZNF829	GO:0080090,regulation of primary metabolic process|GO:0019222,regulation of metabolic process|GO:0031326,regulation of cellular biosynthetic process|GO:0031323,regulation of cellular metabolic process|GO:0090304,nucleic acid metabolic process|GO:0044249,cellular biosynthetic process|GO:0034641,cellular nitrogen compound metabolic process|GO:0006807,nitrogen compound metabolic process|GO:0034645,cellular macromolecule biosynthetic process|GO:1901362,organic cyclic compound biosynthetic process|GO:0050789,regulation of biological process|GO:0097659,nucleic acid-templated transcription|GO:0032774,RNA biosynthetic process|GO:0006139,nucleobase-containing compound metabolic process|GO:0044260,cellular macromolecule metabolic process|GO:0071704,organic substance metabolic process|GO:2000112,regulation of cellular macromolecule biosynthetic process|GO:0060255,regulation of macromolecule metabolic process|GO:0010467,gene expression|GO:0065007,biological regulation|GO:1901360,organic cyclic compound metabolic process|GO:0010468,regulation of gene expression|GO:0018130,heterocycle biosynthetic process|GO:1901576,organic substance biosynthetic process|GO:0019219,regulation of nucleobase-containing compound metabolic process|GO:0006725,cellular aromatic compound metabolic process|GO:0009987,cellular process|GO:0009889,regulation of biosynthetic process|GO:1903506,regulation of nucleic acid-templated transcription|GO:0050794,regulation of cellular process|GO:0009058,biosynthetic process|GO:0009059,macromolecule biosynthetic process|GO:0051171,regulation of nitrogen compound metabolic process|GO:0008152,metabolic process|GO:2001141,regulation of RNA biosynthetic process|GO:0034654,nucleobase-containing compound biosynthetic process|GO:0046483,heterocycle metabolic process|GO:0016070,RNA metabolic process|GO:0044238,primary metabolic process|GO:0044271,cellular nitrogen compound biosynthetic process|GO:0051252,regulation of RNA metabolic process|GO:0044237,cellular metabolic process|GO:0043170,macromolecule metabolic process|GO:0006355,regulation of transcription, DNA-templated|GO:0010556,regulation of macromolecule biosynthetic process|GO:0006351,transcription, DNA-templated|GO:0019438,aromatic compound biosynthetic process	GO:0043231,intracellular membrane-bounded organelle|GO:0005634,nucleus|GO:0044464,cell part|GO:0005623,cell|GO:0005622,intracellular|GO:0043229,intracellular organelle|GO:0044424,intracellular part|GO:0043227,membrane-bounded organelle|GO:0043226,organelle	GO:0043169,cation binding|GO:0097159,organic cyclic compound binding|GO:0043167,ion binding|GO:0005488,binding|GO:0003676,nucleic acid binding|GO:0003677,DNA binding|GO:0046872,metal ion binding|GO:1901363,heterocyclic compound binding|GO:0003700,transcription factor activity, sequence-specific DNA binding|GO:0001071,nucleic acid binding transcription factor activity|GO:0000981,RNA polymerase II transcription factor activity, sequence-specific DNA binding
FAM219B			
SMCP	GO:0019953,sexual reproduction|GO:0006928,movement of cell or subcellular component|GO:0051674,localization of cell|GO:0000003,reproduction|GO:0044699,single-organism process|GO:0007341,penetration of zona pellucida|GO:0032501,multicellular organismal process|GO:0048609,multicellular organismal reproductive process|GO:0032504,multicellular organism reproduction|GO:0030317,flagellated sperm motility|GO:0009987,cellular process|GO:0022414,reproductive process|GO:0044763,single-organism cellular process|GO:0007338,single fertilization|GO:0051179,localization|GO:0051704,multi-organism process|GO:0040011,locomotion|GO:0044703,multi-organism reproductive process|GO:0044702,single organism reproductive process|GO:0048870,cell motility|GO:0044706,multi-multicellular organism process|GO:0009566,fertilization	GO:0005737,cytoplasm|GO:0005622,intracellular|GO:0043231,intracellular membrane-bounded organelle|GO:0005623,cell|GO:0043227,membrane-bounded organelle|GO:0016020,membrane|GO:0044444,cytoplasmic part|GO:0043229,intracellular organelle|GO:0005739,mitochondrion|GO:0031966,mitochondrial membrane|GO:0044446,intracellular organelle part|GO:0005740,mitochondrial envelope|GO:0044429,mitochondrial part|GO:0044424,intracellular part|GO:0044464,cell part|GO:0031975,envelope|GO:0031967,organelle	GO:0005488,binding|GO:0005515,protein binding
OR4C11	GO:0009593,detection of chemical stimulus|GO:0050906,detection of stimulus involved in sensory perception|GO:0007165,signal transduction|GO:0042221,response to chemical|GO:0050789,regulation of biological process|GO:0044699,single-organism process|GO:0051716,cellular response to stimulus|GO:0065007,biological regulation|GO:0007186,G-protein coupled receptor signaling pathway|GO:0032501,multicellular organismal process|GO:0007608,sensory perception of smell|GO:0050877,neurological system process|GO:0007606,sensory perception of chemical stimulus|GO:0007600,sensory perception|GO:0050794,regulation of cellular process|GO:0050911,detection of chemical stimulus involved in sensory perception of smell|GO:0044763,single-organism cellular process|GO:0007154,cell communication|GO:0003008,system process|GO:0044700,single organism signaling|GO:0051606,detection of stimulus|GO:0044707,single-multicellular organism process|GO:0050896,response to stimulus|GO:0023052,signaling|GO:0009987,cellular process|GO:0050907,detection of chemical stimulus involved in sensory perception	GO:0016021,integral component of membrane|GO:0016020,membrane|GO:0044464,cell part|GO:0005623,cell|GO:0071944,cell periphery|GO:0005886,plasma membrane|GO:0044425,membrane part|GO:0031224,intrinsic component of membrane	GO:0004930,G-protein coupled receptor activity|GO:0038023,signaling receptor activity|GO:0060089,molecular transducer activity|GO:0004888,transmembrane signaling receptor activity|GO:0004872,receptor activity|GO:0004871,signal transducer activity|GO:0004984,olfactory receptor activity|GO:0099600,transmembrane receptor activity
DYRK4	GO:0044267,cellular protein metabolic process|GO:0006468,protein phosphorylation|GO:0008152,metabolic process|GO:0044260,cellular macromolecule metabolic process|GO:0044238,primary metabolic process|GO:0019538,protein metabolic process|GO:0016310,phosphorylation|GO:0009987,cellular process|GO:0018212,peptidyl-tyrosine modification|GO:0043412,macromolecule modification|GO:0006464,cellular protein modification process|GO:0043170,macromolecule metabolic process|GO:0071704,organic substance metabolic process|GO:0006796,phosphate-containing compound metabolic process|GO:0036211,protein modification process|GO:0018193,peptidyl-amino acid modification|GO:0018108,peptidyl-tyrosine phosphorylation|GO:0006793,phosphorus metabolic process|GO:0044237,cellular metabolic process|GO:0006807,nitrogen compound metabolic process|GO:1901564,organonitrogen compound metabolic process	GO:0005737,cytoplasm|GO:0043231,intracellular membrane-bounded organelle|GO:0005634,nucleus|GO:0044464,cell part|GO:0005623,cell|GO:0005622,intracellular|GO:0043229,intracellular organelle|GO:0044424,intracellular part|GO:0043227,membrane-bounded organelle|GO:0043226,organelle	GO:0035639,purine ribonucleoside triphosphate binding|GO:0004672,protein kinase activity|GO:0043167,ion binding|GO:1901363,heterocyclic compound binding|GO:0001883,purine nucleoside binding|GO:0001882,nucleoside binding|GO:0043168,anion binding|GO:0043169,cation binding|GO:0000166,nucleotide binding|GO:0004674,protein serine/threonine kinase activity|GO:0016740,transferase activity|GO:0004712,protein serine/threonine/tyrosine kinase activity|GO:0004713,protein tyrosine kinase activity|GO:0032549,ribonucleoside binding|GO:0017076,purine nucleotide binding|GO:0046872,metal ion binding|GO:0005524,ATP binding|GO:0016301,kinase activity|GO:1901265,nucleoside phosphate binding|GO:0036094,small molecule binding|GO:0003824,catalytic activity|GO:0030554,adenyl nucleotide binding|GO:0016773,phosphotransferase activity, alcohol group as acceptor|GO:0016772,transferase activity, transferring phosphorus-containing groups|GO:0097367,carbohydrate derivative binding|GO:0032559,adenyl ribonucleotide binding|GO:0005515,protein binding|GO:0097159,organic cyclic compound binding|GO:0032555,purine ribonucleotide binding|GO:0032550,purine ribonucleoside binding|GO:0032553,ribonucleotide binding|GO:0005488,binding
RAB24	GO:0008104,protein localization|GO:0044248,cellular catabolic process|GO:0023052,signaling|GO:0007165,signal transduction|GO:0035556,intracellular signal transduction|GO:0050789,regulation of biological process|GO:0044699,single-organism process|GO:0051716,cellular response to stimulus|GO:0051234,establishment of localization|GO:0070727,cellular macromolecule localization|GO:0006886,intracellular protein transport|GO:0065007,biological regulation|GO:0071702,organic substance transport|GO:0033036,macromolecule localization|GO:0034613,cellular protein localization|GO:0006810,transport|GO:0006914,autophagy|GO:0009987,cellular process|GO:0050794,regulation of cellular process|GO:0045184,establishment of protein localization|GO:0032482,Rab protein signal transduction|GO:0044763,single-organism cellular process|GO:0008152,metabolic process|GO:0051649,establishment of localization in cell|GO:0007154,cell communication|GO:0007265,Ras protein signal transduction|GO:0007264,small GTPase mediated signal transduction|GO:0009056,catabolic process|GO:0051179,localization|GO:0051641,cellular localization|GO:0044700,single organism signaling|GO:0046907,intracellular transport|GO:0050896,response to stimulus|GO:0044237,cellular metabolic process|GO:0015031,protein transport|GO:0006887,exocytosis|GO:0042119,neutrophil activation|GO:0001775,cell activation|GO:0045321,leukocyte activation|GO:0032940,secretion by cell|GO:0002283,neutrophil activation involved in immune response|GO:0042886,amide transport|GO:0002443,leukocyte mediated immunity|GO:0002444,myeloid leukocyte mediated immunity|GO:0002263,cell activation involved in immune response|GO:0045055,regulated exocytosis|GO:0071705,nitrogen compound transport|GO:0002366,leukocyte activation involved in immune response|GO:0002446,neutrophil mediated immunity|GO:0015833,peptide transport|GO:0044765,single-organism transport|GO:0036230,granulocyte activation|GO:0006955,immune response|GO:1902578,single-organism localization|GO:0046903,secretion|GO:0016192,vesicle-mediated transport|GO:0002275,myeloid cell activation involved in immune response|GO:0002274,myeloid leukocyte activation|GO:0043312,neutrophil degranulation|GO:0002376,immune system process|GO:0043299,leukocyte degranulation|GO:0002252,immune effector process	GO:0030139,endocytic vesicle|GO:0043229,intracellular organelle|GO:0043227,membrane-bounded organelle|GO:0043226,organelle|GO:0005737,cytoplasm|GO:0031982,vesicle|GO:0016023,cytoplasmic, membrane-bounded vesicle|GO:0031410,cytoplasmic vesicle|GO:0016020,membrane|GO:0005776,autophagosome|GO:0005739,mitochondrion|GO:0031988,membrane-bounded vesicle|GO:0012505,endomembrane system|GO:0043231,intracellular membrane-bounded organelle|GO:0005829,cytosol|GO:0044464,cell part|GO:0005623,cell|GO:0005622,intracellular|GO:0005773,vacuole|GO:0044444,cytoplasmic part|GO:0044424,intracellular part|GO:0005768,endosome|GO:0071944,cell periphery|GO:0030667,secretory granule membrane|GO:0031090,organelle membrane|GO:0098805,whole membrane|GO:0044433,cytoplasmic vesicle part|GO:0099503,secretory vesicle|GO:0030141,secretory granule|GO:0098588,bounding membrane of organelle|GO:0030659,cytoplasmic vesicle membrane|GO:0097708,intracellular vesicle|GO:0012506,vesicle membrane|GO:0005886,plasma membrane|GO:0044446,intracellular organelle part|GO:0044422,organelle part	GO:0035639,purine ribonucleoside triphosphate binding|GO:0005488,binding|GO:0043167,ion binding|GO:1901363,heterocyclic compound binding|GO:1901265,nucleoside phosphate binding|GO:0001882,nucleoside binding|GO:0043168,anion binding|GO:0016462,pyrophosphatase activity|GO:0000166,nucleotide binding|GO:0032561,guanyl ribonucleotide binding|GO:0001883,purine nucleoside binding|GO:0032549,ribonucleoside binding|GO:0017076,purine nucleotide binding|GO:0005525,GTP binding|GO:0016787,hydrolase activity|GO:0003924,GTPase activity|GO:0017111,nucleoside-triphosphatase activity|GO:0036094,small molecule binding|GO:0003824,catalytic activity|GO:0016818,hydrolase activity, acting on acid anhydrides, in phosphorus-containing anhydrides|GO:0097367,carbohydrate derivative binding|GO:0097159,organic cyclic compound binding|GO:0016817,hydrolase activity, acting on acid anhydrides|GO:0019001,guanyl nucleotide binding|GO:0005515,protein binding|GO:0032555,purine ribonucleotide binding|GO:0032550,purine ribonucleoside binding|GO:0032553,ribonucleotide binding
AL353791	GO:0007155,cell adhesion|GO:0022610,biological adhesion	GO:0044425,membrane part|GO:0016021,integral component of membrane|GO:0016020,membrane|GO:0031224,intrinsic component of membrane	
TMEM52B		GO:0043230,extracellular organelle|GO:0031982,vesicle|GO:0044421,extracellular region part|GO:0043227,membrane-bounded organelle|GO:0016021,integral component of membrane|GO:0016020,membrane|GO:0031988,membrane-bounded vesicle|GO:0005576,extracellular region|GO:0044425,membrane part|GO:1903561,extracellular vesicle|GO:0065010,extracellular membrane-bounded organelle|GO:0043226,organelle|GO:0031224,intrinsic component of membrane|GO:0070062,extracellular exosome	
TMPRSS11BNL			
MUC19	GO:0044249,cellular biosynthetic process|GO:0034645,cellular macromolecule biosynthetic process|GO:0009100,glycoprotein metabolic process|GO:0009101,glycoprotein biosynthetic process|GO:0044699,single-organism process|GO:0044267,cellular protein metabolic process|GO:0044710,single-organism metabolic process|GO:0044260,cellular macromolecule metabolic process|GO:0071704,organic substance metabolic process|GO:0016266,O-glycan processing|GO:1901576,organic substance biosynthetic process|GO:0043687,post-translational protein modification|GO:0009987,cellular process|GO:0070085,glycosylation|GO:0006464,cellular protein modification process|GO:0043412,macromolecule modification|GO:0036211,protein modification process|GO:0044763,single-organism cellular process|GO:0008152,metabolic process|GO:0044723,single-organism carbohydrate metabolic process|GO:0005975,carbohydrate metabolic process|GO:0009059,macromolecule biosynthetic process|GO:0044238,primary metabolic process|GO:0006493,protein O-linked glycosylation|GO:0006486,protein glycosylation|GO:0019538,protein metabolic process|GO:1901135,carbohydrate derivative metabolic process|GO:0009058,biosynthetic process|GO:0044237,cellular metabolic process|GO:0043170,macromolecule metabolic process|GO:1901137,carbohydrate derivative biosynthetic process|GO:0043413,macromolecule glycosylation|GO:0006950,response to stress|GO:0050896,response to stimulus|GO:0048584,positive regulation of response to stimulus|GO:0048583,regulation of response to stimulus|GO:1901564,organonitrogen compound metabolic process|GO:0031349,positive regulation of defense response|GO:0006807,nitrogen compound metabolic process|GO:0023052,signaling|GO:0007165,signal transduction|GO:0007166,cell surface receptor signaling pathway|GO:0006952,defense response|GO:0050789,regulation of biological process|GO:0031347,regulation of defense response|GO:0002764,immune response-regulating signaling pathway|GO:0002768,immune response-regulating cell surface receptor signaling pathway|GO:0002684,positive regulation of immune system process|GO:0002682,regulation of immune system process|GO:0048518,positive regulation of biological process|GO:0065007,biological regulation|GO:0002220,innate immune response activating cell surface receptor signaling pathway|GO:0002223,stimulatory C-type lectin receptor signaling pathway|GO:0045089,positive regulation of innate immune response|GO:0045088,regulation of innate immune response|GO:0002757,immune response-activating signal transduction|GO:0045087,innate immune response|GO:0051716,cellular response to stimulus|GO:0050794,regulation of cellular process|GO:0002429,immune response-activating cell surface receptor signaling pathway|GO:0006955,immune response|GO:0007154,cell communication|GO:0044700,single organism signaling|GO:0050776,regulation of immune response|GO:1901566,organonitrogen compound biosynthetic process|GO:0080134,regulation of response to stress|GO:0002758,innate immune response-activating signal transduction|GO:0050778,positive regulation of immune response|GO:0002376,immune system	GO:0031974,membrane-enclosed lumen|GO:0043229,intracellular organelle|GO:0043227,membrane-bounded organelle|GO:0043226,organelle|GO:0005737,cytoplasm|GO:0070062,extracellular exosome|GO:0031988,membrane-bounded vesicle|GO:0044431,Golgi apparatus part|GO:0005794,Golgi apparatus|GO:0005796,Golgi lumen|GO:0012505,endomembrane system|GO:1903561,extracellular vesicle|GO:0031982,vesicle|GO:0043230,extracellular organelle|GO:0043231,intracellular membrane-bounded organelle|GO:0043233,organelle lumen|GO:0044464,cell part|GO:0005623,cell|GO:0005622,intracellular|GO:0044446,intracellular organelle part|GO:0070013,intracellular organelle lumen|GO:0044444,cytoplasmic part|GO:0005576,extracellular region|GO:0044424,intracellular part|GO:0065010,extracellular membrane-bounded organelle|GO:0044421,extracellular region part|GO:0044422,organelle part|GO:0071944,cell periphery|GO:0016020,membrane|GO:0005886,plasma membrane	
CACNG8	GO:0019226,transmission of nerve impulse|GO:0032412,regulation of ion transmembrane transporter activity|GO:0051049,regulation of transport|GO:0050877,neurological system process|GO:0048583,regulation of response to stimulus|GO:0023052,signaling|GO:0007165,signal transduction|GO:0007166,cell surface receptor signaling pathway|GO:0023051,regulation of signaling|GO:0034765,regulation of ion transmembrane transport|GO:0051179,localization|GO:0010646,regulation of cell communication|GO:0072511,divalent inorganic cation transport|GO:0050789,regulation of biological process|GO:0043269,regulation of ion transport|GO:0051716,cellular response to stimulus|GO:0051234,establishment of localization|GO:0035637,multicellular organismal signaling|GO:0009966,regulation of signal transduction|GO:0007215,glutamate receptor signaling pathway|GO:0032409,regulation of transporter activity|GO:0065007,biological regulation|GO:0034762,regulation of transmembrane transport|GO:0006810,transport|GO:0098660,inorganic ion transmembrane transport|GO:2000311,regulation of alpha-amino-3-hydroxy-5-methyl-4-isoxazole propionate selective glutamate receptor activity|GO:0065009,regulation of molecular function|GO:0010469,regulation of receptor activity|GO:0098662,inorganic cation transmembrane transport|GO:0032501,multicellular organismal process|GO:0006812,cation transport|GO:0006811,ion transport|GO:0009987,cellular process|GO:0006816,calcium ion transport|GO:0050794,regulation of cellular process|GO:0034220,ion transmembrane transport|GO:0044765,single-organism transport|GO:0044763,single-organism cellular process|GO:0007268,chemical synaptic transmission|GO:0007267,cell-cell signaling|GO:1900449,regulation of glutamate receptor signaling pathway|GO:0032879,regulation of localization|GO:0055085,transmembrane transport|GO:1902578,single-organism localization|GO:0003008,system process|GO:0044700,single organism signaling|GO:0044699,single-organism process|GO:0070838,divalent metal ion transport|GO:0044707,single-multicellular organism process|GO:0022898,regulation of transmembrane transporter activity|GO:0050896,response to stimulus|GO:0070588,calcium ion transmembrane transport|GO:0007154,cell communication|GO:0030001,metal ion transport|GO:0098655,cation transmembrane transport|GO:0060047,heart contraction|GO:0044057,regulation of system process|GO:0003015,heart process|GO:0061337,cardiac conduction|GO:0008015,blood circulation|GO:0008016,regulation of heart contraction|GO:1903522,regulation of blood circulation|GO:0051239,regulation of multicellular organismal process|GO:0003013,circulatory system process|GO:0099601,regulation of neurotransmitter receptor activity	GO:0098590,plasma membrane region|GO:0032281,AMPA glutamate receptor complex|GO:0005887,integral component of plasma membrane|GO:0030139,endocytic vesicle|GO:0043229,intracellular organelle|GO:0005891,voltage-gated calcium channel complex|GO:0034702,ion channel complex|GO:0045202,synapse|GO:0043227,membrane-bounded organelle|GO:0043226,organelle|GO:0030054,cell junction|GO:0097060,synaptic membrane|GO:0098797,plasma membrane protein complex|GO:0030666,endocytic vesicle membrane|GO:0031982,vesicle|GO:0016023,cytoplasmic, membrane-bounded vesicle|GO:0031410,cytoplasmic vesicle|GO:0016021,integral component of membrane|GO:0016020,membrane|GO:0098802,plasma membrane receptor complex|GO:0031988,membrane-bounded vesicle|GO:0044433,cytoplasmic vesicle part|GO:0044456,synapse part|GO:0031224,intrinsic component of membrane|GO:0044459,plasma membrane part|GO:0005737,cytoplasm|GO:0031226,intrinsic component of plasma membrane|GO:1902495,transmembrane transporter complex|GO:0008328,ionotropic glutamate receptor complex|GO:0098588,bounding membrane of organelle|GO:0098589,membrane region|GO:1990351,transporter complex|GO:0030659,cytoplasmic vesicle membrane|GO:0014069,postsynaptic density|GO:0012506,vesicle membrane|GO:0005886,plasma membrane|GO:0031090,organelle membrane|GO:0043234,protein complex|GO:0043235,receptor complex|GO:0032991,macromolecular complex|GO:0043231,intracellular membrane-bounded organelle|GO:0045211,postsynaptic membrane|GO:0071944,cell periphery|GO:0044464,cell part|GO:0034704,calcium channel complex|GO:0005623,cell|GO:0005622,intracellular|GO:0044446,intracellular organelle part|GO:0098796,membrane protein complex|GO:0044444,cytoplasmic part|GO:0098794,postsynapse|GO:0044424,intracellular part|GO:0044425,membrane part|GO:0098805,whole membrane|GO:0044422,organelle part|GO:0034703,cation channel complex|GO:0099572,postsynaptic specialization|GO:0043228,non-membrane-bounded organelle|GO:0098984,neuron to neuron synapse|GO:0032279,asymmetric synapse|GO:0097708,intracellular vesicle|GO:0043232,intracellular non-membrane-	GO:0005261,cation channel activity|GO:0005262,calcium channel activity|GO:0098772,molecular function regulator|GO:0015085,calcium ion transmembrane transporter activity|GO:0072509,divalent inorganic cation transmembrane transporter activity|GO:0022843,voltage-gated cation channel activity|GO:0016247,channel regulator activity|GO:0022803,passive transmembrane transporter activity|GO:0046873,metal ion transmembrane transporter activity|GO:0005215,transporter activity|GO:0005216,ion channel activity|GO:0008324,cation transmembrane transporter activity|GO:0022891,substrate-specific transmembrane transporter activity|GO:0022890,inorganic cation transmembrane transporter activity|GO:0022892,substrate-specific transporter activity|GO:0005244,voltage-gated ion channel activity|GO:0015075,ion transmembrane transporter activity|GO:0022832,voltage-gated channel activity|GO:0022857,transmembrane transporter activity|GO:0015267,channel activity|GO:0022836,gated channel activity|GO:0005245,voltage-gated calcium channel activity|GO:0022838,substrate-specific channel activity
F7	GO:0007599,hemostasis|GO:0007598,blood coagulation, extrinsic pathway|GO:0048584,positive regulation of response to stimulus|GO:0048583,regulation of response to stimulus|GO:0007610,behavior|GO:0050926,regulation of positive chemotaxis|GO:0050927,positive regulation of positive chemotaxis|GO:0050920,regulation of chemotaxis|GO:0050921,positive regulation of chemotaxis|GO:0007166,cell surface receptor signaling pathway|GO:0007167,enzyme linked receptor protein signaling pathway|GO:0007169,transmembrane receptor protein tyrosine kinase signaling pathway|GO:0051897,positive regulation of protein kinase B signaling|GO:0051896,regulation of protein kinase B signaling|GO:0044710,single-organism metabolic process|GO:0042330,taxis|GO:0009966,regulation of signal transduction|GO:0009967,positive regulation of signal transduction|GO:0018214,protein carboxylation|GO:0002684,positive regulation of immune system process|GO:0018193,peptidyl-amino acid modification|GO:0048511,rhythmic process|GO:0048513,animal organ development|GO:0048518,positive regulation of biological process|GO:0065007,biological regulation|GO:0030595,leukocyte chemotaxis|GO:0007596,blood coagulation|GO:0006935,chemotaxis|GO:0031100,animal organ regeneration|GO:0050918,positive chemotaxis|GO:0042221,response to chemical|GO:0043434,response to peptide hormone|GO:0010033,response to organic substance|GO:0044700,single organism signaling|GO:0065008,regulation of biological quality|GO:0009605,response to external stimulus|GO:0044707,single-multicellular organism process|GO:0048870,cell motility|GO:0019538,protein metabolic process|GO:0010243,response to organonitrogen compound|GO:0017187,peptidyl-glutamic acid carboxylation|GO:0018200,peptidyl-glutamic acid modification|GO:0002376,immune system process|GO:0030193,regulation of blood coagulation|GO:0060416,response to growth hormone|GO:0030194,positive regulation of blood coagulation|GO:0060326,cell chemotaxis|GO:0006928,movement of cell or subcellular component|GO:0008152,metabolic process|GO:0050820,positive regulation of coagulation|GO:0048008,platelet-derived growth factor receptor signaling pathway|GO:0051674,localization of cell|GO:0035556,intracellular signal transduction|GO:0050789,regulation of biological process|GO:0044267,cellular protein metabolic process|GO:0044260,cellular macromolecule metabolic process|GO:0002687,positive regulation of leukocyte migration|GO:1900046,regulation of hemostasis|GO:0002685,regulation of leukocyte migration|GO:0002682,regulation of immune system process|GO:0014070,response to organic cyclic compound|GO:0016477,cell migration|GO:1900048,positive regulation of hemostasis|GO:0002688,regulation of leukocyte chemotaxis|GO:0007623,circadian rhythm|GO:0051716,cellular response to stimulus|GO:0050795,regulation of behavior|GO:0050794,regulation of cellular process|GO:0043412,macromolecule modification|GO:0050817,coagulation|GO:0042060,wound healing|GO:0051239,regulation of multicellular organismal process|GO:1902533,positive regulation of intracellular signal transduction|GO:1902531,regulation of intracellular signal transduction|GO:0050818,regulation of coagulation|GO:0043491,protein kinase B signaling|GO:0080134,regulation of response to stress|GO:0006950,response to stress|GO:0036211,protein modification process|GO:2000145,regulation of cell motility|GO:0002690,positive regulation of leukocyte chemotaxis|GO:2000147,positive regulation of cell motility|GO:0032103,positive regulation of response to external stimulus|GO:0032101,regulation of response to external stimulus|GO:0050896,response to stimulus|GO:0009611,response to wounding|GO:0023056,positive regulation of signaling|GO:0023052,signaling|GO:0070887,cellular response to chemical stimulus|GO:0023051,regulation of signaling|GO:0010647,positive regulation of cell communication|GO:0010646,regulation of cell communication|GO:0010641,positive regulation of platelet-derived growth factor receptor signaling pathway|GO:0010640,regulation of platelet-derived growth factor receptor signaling pathway|GO:0044699,single-organism process|GO:0009719,response to endogenous stimulus|GO:0051240,positive regulation of multicellular organismal process|GO:0031099,regeneration|GO:0033273,response to vitamin|GO:0006508,proteolysis|GO:1903034,regulation of response to wounding|GO:1903036,positive regulation of response to wounding|GO:0032502,developmental process|GO:1901700,response to oxygen-containing compound|GO:0032501,multicellular organismal process|GO:0050878,regulation of body fluid levels|GO:0044238,primary metabolic process|GO:0043687,post-translational protein modification|GO:0009987,cellular process|GO:0051270,regulation of cellular component movement|GO:0032879,regulation of localization|GO:0009725,response to hormone|GO:0061041,regulation of wound healing|GO:0043170,macromolecule metabolic process|GO:0032571,response to vitamin K|GO:0048731,system development|GO:1901698,response to nitrogen compound|GO:0048545,response to steroid hormone|GO:0009991,response to extracellular stimulus|GO:0007165,signal transduction|GO:0050900,leukocyte migration|GO:0090303,positive regulation of wound healing|GO:0007275,multicellular organism development|GO:0031667,response to nutrient levels|GO:0072376,protein activation cascade|GO:0072378,blood coagulation, fibrin clot formation|GO:0071704,organic substance metabolic process|GO:0030335,positive regulation of cell migration|GO:0030334,regulation of cell migration|GO:0007584,response to nutrient|GO:0006464,cellular protein modification process|GO:0044767,single-organism developmental process|GO:0044763,single-organism cellular process|GO:0043627,response to estrogen|GO:0007154,cell communication|GO:0051179,localization|GO:0040011,locomotion|GO:0051272,positive regulation of cellular component movement|GO:0040012,regulation of locomotion|GO:0040017,positive regulation of locomotion|GO:0048856,anatomical structure development|GO:0044237,cellular metabolic process|GO:1901654,response to ketone|GO:1901652,response to peptide|GO:0033993,response to lipid|GO:0048520,positive regulation of behavior|GO:0048522,positive regulation of cellular process|GO:0009628,response to abiotic stimulus|GO:1905217,response to astaxanthin|GO:0043200,response to amino acid|GO:0070723,response to cholesterol|GO:0010037,response to carbon dioxide|GO:1901564,organonitrogen compound metabolic process|GO:0016192,vesicle-mediated transport|GO:0006807,nitrogen compound metabolic process|GO:0001666,response to hypoxia|GO:0006888,ER to Golgi vesicle-mediated transport|GO:0006810,transport|GO:0097066,response to thyroid hormone|GO:0051604,protein maturation|GO:0097068,response to thyroxine|GO:0006518,peptide metabolic process|GO:0033594,response to hydroxyisoflavone|GO:0033595,response to genistein|GO:0061476,response to anticoagulant|GO:0034641,cellular nitrogen compound metabolic process|GO:0051234,establishment of localization|GO:1904400,response to Thyroid stimulating hormone|GO:0070482,response to oxygen levels|GO:0001101,response to acid chemical|GO:0016485,protein processing|GO:1904386,response to L-phenylalanine derivative|GO:0046907,intracellular transport|GO:0043603,cellular amide metabolic process|GO:0042493,response to drug|GO:1905225,response to thyrotropin-releasing hormone|GO:0036293,response to decreased oxygen levels|GO:0032355,response to estradiol|GO:1904612,response to 2,3,7,8-tetrachlorodibenzodioxine|GO:0010467,gene expression|GO:0080184,response to phenylpropanoid|GO:0048193,Golgi vesicle transport|GO:0006465,signal peptide processing|GO:0051649,establishment of localization in cell|GO:0036314,response to sterol|GO:0051641,cellular localization	GO:0005783,endoplasmic reticulum|GO:0005788,endoplasmic reticulum lumen|GO:0031974,membrane-enclosed lumen|GO:0043229,intracellular organelle|GO:0071944,cell periphery|GO:0043227,membrane-bounded organelle|GO:0043226,organelle|GO:0005737,cytoplasm|GO:0031982,vesicle|GO:0016020,membrane|GO:0044432,endoplasmic reticulum part|GO:0044431,Golgi apparatus part|GO:0005794,Golgi apparatus|GO:0005796,Golgi lumen|GO:0012505,endomembrane system|GO:0005886,plasma membrane|GO:0005615,extracellular space|GO:0043231,intracellular membrane-bounded organelle|GO:0043233,organelle lumen|GO:0044464,cell part|GO:0005623,cell|GO:0005622,intracellular|GO:0044446,intracellular organelle part|GO:0070013,intracellular organelle lumen|GO:0044444,cytoplasmic part|GO:0005576,extracellular region|GO:0044424,intracellular part|GO:0044421,extracellular region part|GO:0044422,organelle part	GO:0001948,glycoprotein binding|GO:0004252,serine-type endopeptidase activity|GO:0004175,endopeptidase activity|GO:0017171,serine hydrolase activity|GO:0097367,carbohydrate derivative binding|GO:0016787,hydrolase activity|GO:0043169,cation binding|GO:0043167,ion binding|GO:0003824,catalytic activity|GO:0070011,peptidase activity, acting on L-amino acid peptides|GO:0005509,calcium ion binding|GO:0005515,protein binding|GO:0005488,binding|GO:0008233,peptidase activity|GO:0046872,metal ion binding|GO:0005102,receptor binding|GO:0008236,serine-type peptidase activity

TLCD2		GO:0044425,membrane part|GO:0016021,integral component of membrane|GO:0016020,membrane|GO:0031224,intrinsic component of membrane	
PRX	GO:0032502,developmental process|GO:0032501,multicellular organismal process|GO:0044707,single-multicellular organism process|GO:0007399,nervous system development|GO:0009987,cellular process|GO:0048856,anatomical structure development|GO:0044767,single-organism developmental process|GO:0044763,single-organism cellular process|GO:0008366,axon ensheathment|GO:0048731,system development|GO:0007272,ensheathment of neurons|GO:0007275,multicellular organism development|GO:0044699,single-organism process	GO:0005737,cytoplasm|GO:0043231,intracellular membrane-bounded organelle|GO:0005634,nucleus|GO:0016020,membrane|GO:0044464,cell part|GO:0005623,cell|GO:0005622,intracellular|GO:0043229,intracellular organelle|GO:0071944,cell periphery|GO:0005886,plasma membrane|GO:0044424,intracellular part|GO:0043227,membrane-bounded organelle|GO:0043226,organelle|GO:0030054,cell junction	GO:0005488,binding|GO:0005515,protein binding
C1orf220			
PSKH1	GO:0044267,cellular protein metabolic process|GO:0006468,protein phosphorylation|GO:0044260,cellular macromolecule metabolic process|GO:0044238,primary metabolic process|GO:0019538,protein metabolic process|GO:0016310,phosphorylation|GO:0009987,cellular process|GO:0043412,macromolecule modification|GO:0006464,cellular protein modification process|GO:0043170,macromolecule metabolic process|GO:0071704,organic substance metabolic process|GO:0006796,phosphate-containing compound metabolic process|GO:0036211,protein modification process|GO:0044237,cellular metabolic process|GO:0008152,metabolic process|GO:0006793,phosphorus metabolic process|GO:0018107,peptidyl-threonine phosphorylation|GO:0018105,peptidyl-serine phosphorylation|GO:1901564,organonitrogen compound metabolic process|GO:0072359,circulatory system development|GO:0006807,nitrogen compound metabolic process|GO:0023052,signaling|GO:0007165,signal transduction|GO:0009799,specification of symmetry|GO:0035556,intracellular signal transduction|GO:0018193,peptidyl-amino acid modification|GO:0007275,multicellular organism development|GO:0044699,single-organism process|GO:0007389,pattern specification process|GO:0051716,cellular response to stimulus|GO:0007368,determination of left/right symmetry|GO:0018210,peptidyl-threonine modification|GO:0050789,regulation of biological process|GO:0048513,animal organ development|GO:0065007,biological regulation|GO:0032502,developmental process|GO:0032501,multicellular organismal process|GO:0007507,heart development|GO:0050794,regulation of cellular process|GO:0044767,single-organism developmental process|GO:0007154,cell communication|GO:0044700,single organism signaling|GO:0018209,peptidyl-serine modification|GO:0044707,single-multicellular organism process|GO:0050896,response to stimulus|GO:0048856,anatomical structure development|GO:0009855,determination of bilateral symmetry|GO:0048731,system development	GO:0005783,endoplasmic reticulum|GO:0005794,Golgi apparatus|GO:0031974,membrane-enclosed lumen|GO:0005789,endoplasmic reticulum membrane|GO:0042175,nuclear outer membrane-endoplasmic reticulum membrane network|GO:0043229,intracellular organelle|GO:0043228,non-membrane-bounded organelle|GO:0005622,intracellular|GO:0043227,membrane-bounded organelle|GO:0043226,organelle|GO:0016607,nuclear speck|GO:0016604,nuclear body|GO:0031090,organelle membrane|GO:0031981,nuclear lumen|GO:0005634,nucleus|GO:0016020,membrane|GO:0005815,microtubule organizing center|GO:0005654,nucleoplasm|GO:0044432,endoplasmic reticulum part|GO:0044430,cytoskeletal part|GO:0044451,nucleoplasm part|GO:0005856,cytoskeleton|GO:0015630,microtubule cytoskeleton|GO:0098588,bounding membrane of organelle|GO:0012505,endomembrane system|GO:0005886,plasma membrane|GO:0043231,intracellular membrane-bounded organelle|GO:0043232,intracellular non-membrane-bounded organelle|GO:0043233,organelle lumen|GO:0044464,cell part|GO:0005623,cell|GO:0005737,cytoplasm|GO:0044446,intracellular organelle part|GO:0070013,intracellular organelle lumen|GO:0044444,cytoplasmic part|GO:0071944,cell periphery|GO:0044428,nuclear part|GO:0044424,intracellular part|GO:0044425,membrane part|GO:0044422,organelle part|GO:0098827,endoplasmic reticulum subcompartment|GO:0031984,organelle subcompartment	GO:0035639,purine ribonucleoside triphosphate binding|GO:0004672,protein kinase activity|GO:0043167,ion binding|GO:1901363,heterocyclic compound binding|GO:0001883,purine nucleoside binding|GO:0001882,nucleoside binding|GO:0043168,anion binding|GO:0000166,nucleotide binding|GO:0004674,protein serine/threonine kinase activity|GO:0016740,transferase activity|GO:1901265,nucleoside phosphate binding|GO:0032549,ribonucleoside binding|GO:0017076,purine nucleotide binding|GO:0005524,ATP binding|GO:0016301,kinase activity|GO:0036094,small molecule binding|GO:0003824,catalytic activity|GO:0030554,adenyl nucleotide binding|GO:0016773,phosphotransferase activity, alcohol group as acceptor|GO:0016772,transferase activity, transferring phosphorus-containing groups|GO:0097367,carbohydrate derivative binding|GO:0032559,adenyl ribonucleotide binding|GO:0005515,protein binding|GO:0097159,organic cyclic compound binding|GO:0032555,purine ribonucleotide binding|GO:0032550,purine ribonucleoside binding|GO:0032553,ribonucleotide binding|GO:0005488,binding
SPSB1	GO:0023052,signaling|GO:0007165,signal transduction|GO:0035556,intracellular signal transduction|GO:0050789,regulation of biological process|GO:0044699,single-organism process|GO:0044267,cellular protein metabolic process|GO:0051716,cellular response to stimulus|GO:0044260,cellular macromolecule metabolic process|GO:0071704,organic substance metabolic process|GO:0070647,protein modification by small protein conjugation or removal|GO:0032446,protein modification by small protein conjugation|GO:0065007,biological regulation|GO:0044238,primary metabolic process|GO:0009987,cellular process|GO:0006464,cellular protein modification process|GO:0050794,regulation of cellular process|GO:0043412,macromolecule modification|GO:0036211,protein modification process|GO:0044763,single-organism cellular process|GO:0008152,metabolic process|GO:0007154,cell communication|GO:0044700,single organism signaling|GO:0019538,protein metabolic process|GO:0050896,response to stimulus|GO:0044237,cellular metabolic process|GO:0043170,macromolecule metabolic process|GO:0016567,protein ubiquitination|GO:0006807,nitrogen compound metabolic process|GO:0000209,protein polyubiquitination|GO:0043687,post-translational protein modification|GO:1901564,organonitrogen compound metabolic process	GO:0005737,cytoplasm|GO:0044464,cell part|GO:0005623,cell|GO:0005622,intracellular|GO:0044424,intracellular part|GO:0005829,cytosol|GO:0044444,cytoplasmic part	GO:0005488,binding|GO:0005515,protein binding|GO:0019787,ubiquitin-like protein transferase activity|GO:0003824,catalytic activity|GO:0016740,transferase activity|GO:0004842,ubiquitin-protein transferase activity
CD99L2	GO:0007155,cell adhesion|GO:0022610,biological adhesion|GO:0097530,granulocyte migration|GO:0016337,single organismal cell-cell adhesion|GO:0002685,regulation of leukocyte migration|GO:0098602,single organism cell adhesion|GO:1990266,neutrophil migration|GO:0006928,movement of cell or subcellular component|GO:1902624,positive regulation of neutrophil migration|GO:0065007,biological regulation|GO:1902622,regulation of neutrophil migration|GO:0050904,diapedesis|GO:0050789,regulation of biological process|GO:0044699,single-organism process|GO:2000391,positive regulation of neutrophil extravasation|GO:0072683,T cell extravasation|GO:0002687,positive regulation of leukocyte migration|GO:2000401,regulation of lymphocyte migration|GO:0002684,positive regulation of immune system process|GO:2000403,positive regulation of lymphocyte migration|GO:0002682,regulation of immune system process|GO:2000406,positive regulation of T cell migration|GO:2000407,regulation of T cell extravasation|GO:0048518,positive regulation of biological process|GO:2000404,regulation of T cell migration|GO:0016477,cell migration|GO:0030335,positive regulation of cell migration|GO:0030334,regulation of cell migration|GO:0002693,positive regulation of cellular extravasation|GO:0098609,cell-cell adhesion|GO:0034109,homotypic cell-cell adhesion|GO:0009987,cellular process|GO:0050794,regulation of cellular process|GO:0072676,lymphocyte migration|GO:0040012,regulation of locomotion|GO:0044763,single-organism cellular process|GO:0072672,neutrophil extravasation|GO:2000409,positive regulation of T cell extravasation|GO:0032879,regulation of localization|GO:0072678,T cell migration|GO:0051179,localization|GO:0040011,locomotion|GO:0051272,positive regulation of cellular component movement|GO:0051270,regulation of cellular component movement|GO:0040017,positive regulation of locomotion|GO:0048870,cell motility|GO:0045123,cellular extravasation|GO:0050900,leukocyte migration|GO:2000389,regulation of neutrophil extravasation|GO:0002376,immune system process|GO:0051674,localization of cell|GO:0002691,regulation of cellular extravasation|GO:2000145,regulation of cell motility|GO:2000147,positive regulation of cell motility|GO:0097529,myeloid leukocyte migration|GO:0048522,positive regulation of cellular process	GO:0016020,membrane|GO:0016021,integral component of membrane|GO:0070161,anchoring junction|GO:0044464,cell part|GO:0005623,cell|GO:0031224,intrinsic component of membrane|GO:0071944,cell periphery|GO:0005912,adherens junction|GO:0005886,plasma membrane|GO:0044425,membrane part|GO:0005924,cell-substrate adherens junction|GO:0005925,focal adhesion|GO:0030054,cell junction|GO:0030055,cell-substrate junction|GO:0009986,cell surface|GO:0005913,cell-cell adherens junction|GO:0005911,cell-cell junction	GO:0005488,binding|GO:0005515,protein binding
AC008267	GO:0006478,peptidyl-tyrosine sulfation|GO:0044238,primary metabolic process|GO:0044260,cellular macromolecule metabolic process|GO:0044267,cellular protein metabolic process|GO:0019538,protein metabolic process|GO:0051923,sulfation|GO:0006477,protein sulfation|GO:0009987,cellular process|GO:0018212,peptidyl-tyrosine modification|GO:0006464,cellular protein modification process|GO:0043170,macromolecule metabolic process|GO:0071704,organic substance metabolic process|GO:0043412,macromolecule modification|GO:0036211,protein modification process|GO:0006790,sulfur compound metabolic process|GO:0018193,peptidyl-amino acid modification|GO:0008152,metabolic process|GO:0044237,cellular metabolic process|GO:0006807,nitrogen compound metabolic process|GO:1901564,organonitrogen compound metabolic process		GO:0016782,transferase activity, transferring sulfur-containing groups|GO:0008146,sulfotransferase activity|GO:0003824,catalytic activity|GO:0016740,transferase activity|GO:0008476,protein-tyrosine sulfotransferase activity
SRP9	GO:0008104,protein localization|GO:0045900,negative regulation of translational elongation|GO:0080090,regulation of primary metabolic process|GO:0019222,regulation of metabolic process|GO:0061024,membrane organization|GO:0071840,cellular component organization or biogenesis|GO:0010605,negative regulation of macromolecule metabolic process|GO:0070727,cellular macromolecule localization|GO:0010608,posttranscriptional regulation of gene expression|GO:0043043,peptide biosynthetic process|GO:0048519,negative regulation of biological process|GO:0033036,macromolecule localization|GO:0006448,regulation of translational elongation|GO:0060255,regulation of macromolecule metabolic process|GO:0006605,protein targeting|GO:0045184,establishment of protein localization|GO:0072657,protein localization to membrane|GO:1901564,organonitrogen compound metabolic process|GO:1901566,organonitrogen compound biosynthetic process|GO:0019538,protein metabolic process|GO:0009892,negative regulation of metabolic process|GO:0009890,negative regulation of biosynthetic process|GO:0006613,cotranslational protein targeting to membrane|GO:0006612,protein targeting to membrane|GO:0006614,SRP-dependent cotranslational protein targeting to membrane|GO:0010629,negative regulation of gene expression|GO:0006807,nitrogen compound metabolic process|GO:0050789,regulation of biological process|GO:0044267,cellular protein metabolic process|GO:0044260,cellular macromolecule metabolic process|GO:0006886,intracellular protein transport|GO:0016043,cellular component organization|GO:0065007,biological regulation|GO:0006810,transport|GO:0009889,regulation of biosynthetic process|GO:0050794,regulation of cellular process|GO:0044802,single-organism membrane organization|GO:0008152,metabolic process|GO:0051234,establishment of localization|GO:0044271,cellular nitrogen compound biosynthetic process|GO:0046907,intracellular transport|GO:0044765,single-organism transport|GO:0045047,protein targeting to ER|GO:0010556,regulation of macromolecule biosynthetic process|GO:0006518,peptide metabolic process|GO:0010558,negative regulation of macromolecule biosynthetic process|GO:0044249,cellular biosynthetic process|GO:0034641,cellular nitrogen compound metabolic process|GO:0034645,cellular macromolecule biosynthetic process|GO:0044699,single-organism process|GO:0051248,negative regulation of protein metabolic process|GO:0070972,protein localization to endoplasmic reticulum|GO:0051246,regulation of protein metabolic process|GO:0072599,establishment of protein localization to endoplasmic reticulum|GO:0072594,establishment of protein localization to organelle|GO:0009987,cellular process|GO:0034249,negative regulation of cellular amide metabolic process|GO:0034248,regulation of cellular amide metabolic process|GO:0016482,cytosolic transport|GO:0043604,amide biosynthetic process|GO:0032269,negative regulation of cellular protein metabolic process|GO:0032268,regulation of cellular protein metabolic process|GO:0043603,cellular amide metabolic process|GO:0043170,macromolecule metabolic process|GO:0017148,negative regulation of translation|GO:0033365,protein localization to organelle|GO:0031327,negative regulation of cellular biosynthetic process|GO:0031326,regulation of cellular biosynthetic process|GO:0031324,negative regulation of cellular metabolic process|GO:0031323,regulation of cellular metabolic process|GO:2000112,regulation of cellular macromolecule biosynthetic process|GO:2000113,negative regulation of cellular macromolecule biosynthetic process|GO:0071704,organic substance metabolic process|GO:0010467,gene expression|GO:0071702,organic substance transport|GO:0010468,regulation of gene expression|GO:1901576,organic substance biosynthetic process|GO:0034613,cellular protein localization|GO:0006417,regulation of translation|GO:0009058,biosynthetic process|GO:0009059,macromolecule biosynthetic process|GO:0044763,single-organism cellular process|GO:0051171,regulation of nitrogen compound metabolic process|GO:0051172,negative regulation of nitrogen compound metabolic process|GO:0051649,establishment of localization in cell|GO:0051179,localization|GO:1902578,single-organism localization|GO:0051641,cellular localization|GO:0044238,primary metabolic process|GO:1902580,single-organism cellular localization|GO:0090150,establishment of protein localization to membrane|GO:0044237,cellular metabolic process|GO:0015031,protein transport|GO:1902582,single-organism intracellular transport|GO:0006414,translational elongation|GO:0006412,translation|GO:0048523,negative regulation of cellular process|GO:0006616,SRP-dependent cotranslational protein targeting to membrane, translocation|GO:0065002,intracellular protein transmembrane transport|GO:0042886,amide transport|GO:0015833,peptide transport|GO:0071806,protein transmembrane transport|GO:0055085,transmembrane transport|GO:0071705,nitrogen compound transport	GO:0005786,signal recognition particle, endoplasmic reticulum targeting|GO:0005785,signal recognition particle receptor complex|GO:0005783,endoplasmic reticulum|GO:0044464,cell part|GO:0005789,endoplasmic reticulum membrane|GO:0042175,nuclear outer membrane-endoplasmic reticulum membrane network|GO:0043229,intracellular organelle|GO:0030529,intracellular ribonucleoprotein complex|GO:0043227,membrane-bounded organelle|GO:0043226,organelle|GO:0005737,cytoplasm|GO:0031982,vesicle|GO:0016020,membrane|GO:0031988,membrane-bounded vesicle|GO:0044432,endoplasmic reticulum part|GO:0005791,rough endoplasmic reticulum|GO:0098588,bounding membrane of organelle|GO:0012505,endomembrane system|GO:0032991,macromolecular complex|GO:1903561,extracellular vesicle|GO:0031090,organelle membrane|GO:0043234,protein complex|GO:0043230,extracellular organelle|GO:0043231,intracellular membrane-bounded organelle|GO:0070062,extracellular exosome|GO:0005829,cytosol|GO:0030867,rough endoplasmic reticulum membrane|GO:0005623,cell|GO:0005622,intracellular|GO:0044446,intracellular organelle part|GO:0098796,membrane protein complex|GO:0044444,cytoplasmic part|GO:0005576,extracellular region|GO:0048500,signal recognition particle|GO:0044424,intracellular part|GO:0044425,membrane part|GO:0065010,extracellular membrane-bounded organelle|GO:0044421,extracellular region part|GO:0044422,organelle part|GO:1990904,ribonucleoprotein complex|GO:0031984,organelle subcompartment|GO:0098827,endoplasmic reticulum subcompartment	GO:0097159,organic cyclic compound binding|GO:0005047,signal recognition particle binding|GO:0008312,7S RNA binding|GO:0003723,RNA binding|GO:0003676,nucleic acid binding|GO:0043021,ribonucleoprotein complex binding|GO:0044877,macromolecular complex binding|GO:1901363,heterocyclic compound binding|GO:0005488,binding|GO:0005515,protein binding
RAD54B	GO:0080090,regulation of primary metabolic process|GO:0019222,regulation of metabolic process|GO:0031326,regulation of cellular biosynthetic process|GO:0031323,regulation of cellular metabolic process|GO:0090304,nucleic acid metabolic process|GO:0044249,cellular biosynthetic process|GO:0034641,cellular nitrogen compound metabolic process|GO:0006807,nitrogen compound metabolic process|GO:0034645,cellular macromolecule biosynthetic process|GO:1901362,organic cyclic compound biosynthetic process|GO:0050789,regulation of biological process|GO:0097659,nucleic acid-templated transcription|GO:0032774,RNA biosynthetic process|GO:0006139,nucleobase-containing compound metabolic process|GO:0044260,cellular macromolecule metabolic process|GO:0071704,organic substance metabolic process|GO:2000112,regulation of cellular macromolecule biosynthetic process|GO:0060255,regulation of macromolecule metabolic process|GO:0010467,gene expression|GO:0065007,biological regulation|GO:1901360,organic cyclic compound metabolic process|GO:0010468,regulation of gene expression|GO:0018130,heterocycle biosynthetic process|GO:1901576,organic substance biosynthetic process|GO:0019219,regulation of nucleobase-containing compound metabolic process|GO:0006725,cellular aromatic compound metabolic process|GO:0009987,cellular process|GO:0009889,regulation of biosynthetic process|GO:1903506,regulation of nucleic acid-templated transcription|GO:0050794,regulation of cellular process|GO:0009058,biosynthetic process|GO:0009059,macromolecule biosynthetic process|GO:0051171,regulation of nitrogen compound metabolic process|GO:0008152,metabolic process|GO:2001141,regulation of RNA biosynthetic process|GO:0034654,nucleobase-containing compound biosynthetic process|GO:0046483,heterocycle metabolic process|GO:0016070,RNA metabolic process|GO:0044238,primary metabolic process|GO:0044271,cellular nitrogen compound biosynthetic process|GO:0051252,regulation of RNA metabolic process|GO:0044237,cellular metabolic process|GO:0043170,macromolecule metabolic process|GO:0006355,regulation of transcription, DNA-templated|GO:0010556,regulation of macromolecule biosynthetic process|GO:0006351,transcription, DNA-templated|GO:0019438,aromatic compound biosynthetic process	GO:0043231,intracellular membrane-bounded organelle|GO:0005634,nucleus|GO:0044464,cell part|GO:0005623,cell|GO:0005622,intracellular|GO:0043229,intracellular organelle|GO:0044424,intracellular part|GO:0043227,membrane-bounded organelle|GO:0043226,organelle	GO:0005515,protein binding|GO:0042802,identical protein binding|GO:0005488,binding
MIF	GO:0080090,regulation of primary metabolic process|GO:0019222,regulation of metabolic process|GO:2000242,negative regulation of reproductive process|GO:2000241,regulation of reproductive process|GO:0048468,cell development|GO:0008585,female gonad development|GO:0007292,female gamete generation|GO:1901362,organic cyclic compound biosynthetic process|GO:1901360,organic cyclic compound metabolic process|GO:0010604,positive regulation of macromolecule metabolic process|GO:0048869,cellular developmental process|GO:0045137,development of primary sexual characteristics|GO:0048511,rhythmic process|GO:0048513,animal organ development|GO:0044093,positive regulation of molecular function|GO:0048518,positive regulation of biological process|GO:0048519,negative regulation of biological process|GO:0051704,multi-organism process|GO:0030154,cell differentiation|GO:0060255,regulation of macromolecule metabolic process|GO:0007281,germ cell development|GO:0003006,developmental process involved in reproduction|GO:0042221,response to chemical|GO:2001141,regulation of RNA biosynthetic process|GO:0010033,response to organic substance|GO:0046483,heterocycle metabolic process|GO:0044700,single organism signaling|GO:0044703,multi-organism reproductive process|GO:0044702,single organism reproductive process|GO:0044707,single-multicellular organism process|GO:0042698,ovulation cycle|GO:0019438,aromatic compound biosynthetic process|GO:0022605,oogenesis stage|GO:0009893,positive regulation of metabolic process|GO:0022602,ovulation cycle process|GO:0009891,positive regulation of biosynthetic process|GO:0008152,metabolic process|GO:0010628,positive regulation of gene expression|GO:0048162,multi-layer follicle stage|GO:0050789,regulation of biological process|GO:0097659,nucleic acid-templated transcription|GO:0007530,sex determination|GO:1901576,organic substance biosynthetic process|GO:0044260,cellular macromolecule metabolic process|GO:0008406,gonad development|GO:0065007,biological regulation|GO:0014070,response to organic cyclic compound|GO:0065009,regulation of molecular function|GO:0048477,oogenesis|GO:0018130,heterocycle biosynthetic process|GO:0046545,development of primary female sexual characteristics|GO:0050793,regulation of developmental process|GO:0009889,regulation of biosynthetic process|GO:0009888,tissue development|GO:0050794,regulation of cellular process|GO:0051239,regulation of multicellular organismal process|GO:0034654,nucleobase-containing compound biosynthetic process|GO:0046660,female sex differentiation|GO:0046661,male sex differentiation|GO:0000003,reproduction|GO:0016070,RNA metabolic process|GO:0007548,sex differentiation|GO:0044271,cellular nitrogen compound biosynthetic process|GO:0050896,response to stimulus|GO:0006355,regulation of transcription, DNA-templated|GO:0010557,positive regulation of macromolecule biosynthetic process|GO:0010556,regulation of macromolecule biosynthetic process|GO:0006351,transcription, DNA-templated|GO:0032774,RNA biosynthetic process|GO:0007498,mesoderm development|GO:0022412,cellular process involved in reproduction in multicellular organism|GO:0019953,sexual reproduction|GO:0044249,cellular biosynthetic process|GO:0034641,cellular nitrogen compound metabolic process|GO:0023052,signaling|GO:0034645,cellular macromolecule biosynthetic process|GO:0007154,cell communication|GO:0009653,anatomical structure morphogenesis|GO:0044699,single-organism process|GO:0006139,nucleobase-containing compound metabolic process|GO:0001546,preantral ovarian follicle growth|GO:0001541,ovarian follicle development|GO:0051241,negative regulation of multicellular organismal process|GO:1903508,positive regulation of nucleic acid-templated transcription|GO:2000194,regulation of female gonad development|GO:2000195,negative regulation of female gonad development|GO:0048608,reproductive structure development|GO:0048609,multicellular organismal reproductive process|GO:0032504,multicellular organism reproduction|GO:2000354,regulation of ovarian follicle development|GO:2000355,negative regulation of ovarian follicle development|GO:0009987,cellular process|GO:0006725,cellular aromatic compound metabolic process|GO:1903506,regulation of nucleic acid-templated transcription|GO:0001655,urogenital system development|GO:0051093,negative regulation of developmental process|GO:0051092,positive regulation of NF-kappaB transcription factor activity|GO:0051091,positive regulation of sequence-specific DNA binding transcription factor activity|GO:0051090,regulation of sequence-specific DNA binding transcription factor activity|GO:0007568,aging|GO:0051252,regulation of RNA metabolic process|GO:0042493,response to drug|GO:0051254,positive regulation of RNA metabolic process|GO:0043170,macromolecule metabolic process|GO:1902680,positive regulation of RNA biosynthetic process|GO:0006807,nitrogen compound metabolic process|GO:0048731,system development|GO:0032502,developmental process|GO:0031328,positive regulation of cellular biosynthetic process|GO:0061458,reproductive system development|GO:0031326,regulation of cellular biosynthetic process|GO:0031325,positive regulation of cellular metabolic process|GO:0031323,regulation of cellular metabolic process|GO:0090304,nucleic acid metabolic process|GO:0032501,multicellular organismal process|GO:0001880,Mullerian duct regression|GO:0007276,gamete generation|GO:0040007,growth|GO:2000112,regulation of cellular macromolecule biosynthetic process|GO:0007275,multicellular organism development|GO:0071704,organic substance metabolic process|GO:0010467,gene expression|GO:0010468,regulation of gene expression|GO:0045935,positive regulation of nucleobase-containing compound metabolic process|GO:0019219,regulation of nucleobase-containing compound metabolic process|GO:0060033,anatomical structure regression|GO:0044767,single-organism developmental process|GO:0022414,reproductive process|GO:0009058,biosynthetic process|GO:0009059,macromolecule biosynthetic process|GO:0044763,single-organism cellular process|GO:0051171,regulation of nitrogen compound metabolic process|GO:0051173,positive regulation of nitrogen compound metabolic process|GO:0007267,cell-cell signaling|GO:0060485,mesenchyme development|GO:0007506,gonadal mesoderm development|GO:0044238,primary metabolic process|GO:0048856,anatomical structure development|GO:0044237,cellular metabolic process|GO:2000026,regulation of multicellular organismal development|GO:0048522,positive regulation of cellular process|GO:0045893,positive regulation of transcription, DNA-templated|GO:0030509,BMP signaling pathway|GO:0007165,signal transduction|GO:0007166,cell surface receptor signaling pathway|GO:0007167,enzyme linked receptor protein signaling pathway|GO:0051716,cellular response to stimulus|GO:0071363,cellular response to growth factor stimulus|GO:0007178,transmembrane receptor protein serine/threonine kinase signaling pathway|GO:0006366,transcription from RNA polymerase II promoter|GO:0071772,response to BMP|GO:0071773,cellular response to BMP stimulus|GO:0070848,response to growth factor|GO:0070887,cellular response to chemical stimulus|GO:0009719,response to endogenous stimulus|GO:0071310,cellular response to organic substance|GO:0006357,regulation of transcription from RNA polymerase II promoter|GO:0071495,cellular response to endogenous stimulus	GO:0005737,cytoplasm|GO:0005615,extracellular space|GO:0044464,cell part|GO:0005623,cell|GO:0005622,intracellular|GO:0005576,extracellular region|GO:0044424,intracellular part|GO:0044421,extracellular region part	GO:0005126,cytokine receptor binding|GO:0005179,hormone activity|GO:0008083,growth factor activity|GO:0005160,transforming growth factor beta receptor binding|GO:0005488,binding|GO:0005515,protein binding|GO:0005102,receptor binding
GDF2	GO:0032924,activin receptor signaling pathway|GO:0080090,regulation of primary metabolic process|GO:0019222,regulation of metabolic process|GO:0001667,ameboidal-type cell migration|GO:0048584,positive regulation of response to stimulus|GO:0048583,regulation of response to stimulus|GO:0050680,negative regulation of epithelial cell proliferation|GO:0034645,cellular macromolecule biosynthetic process|GO:0045765,regulation of angiogenesis|GO:0030509,BMP signaling pathway|GO:0072359,circulatory system development|GO:0072358,cardiovascular system development|GO:0007165,signal transduction|GO:0007166,cell surface receptor signaling pathway|GO:0007167,enzyme linked receptor protein signaling pathway|GO:0098771,inorganic ion homeostasis|GO:0019220,regulation of phosphate metabolic process|GO:1901362,organic cyclic compound biosynthetic process|GO:0044707,single-multicellular organism process|GO:0023014,signal transduction by protein phosphorylation|GO:0002009,morphogenesis of an epithelium|GO:0044710,single-organism metabolic process|GO:0010605,negative regulation of macromolecule metabolic process|GO:0010604,positive regulation of macromolecule metabolic process|GO:0010596,negative regulation of endothelial cell migration|GO:0009966,regulation of signal transduction|GO:0009967,positive regulation of signal transduction|GO:0000165,MAPK cascade|GO:0043067,regulation of programmed cell death|GO:0001936,regulation of endothelial cell proliferation|GO:0045667,regulation of osteoblast differentiation|GO:0048513,animal organ development|GO:0030856,regulation of epithelial cell differentiation|GO:0048869,cellular developmental process|GO:0030855,epithelial cell differentiation|GO:0048518,positive regulation of biological process|GO:0048519,negative regulation of biological process|GO:0030858,positive regulation of epithelial cell differentiation|GO:0007178,transmembrane receptor protein serine/threonine kinase signaling pathway|GO:0045669,positive regulation of osteoblast differentiation|GO:0042127,regulation of cell proliferation|GO:0051053,negative regulation of DNA metabolic process|GO:0051052,regulation of DNA metabolic process|GO:0010467,gene expression|GO:0060255,regulation of macromolecule metabolic process|GO:0048468,cell development|GO:0003158,endothelium development|GO:0043537,negative regulation of blood vessel endothelial cell migration|GO:0043535,regulation of blood vessel endothelial cell migration|GO:0043534,blood vessel endothelial cell migration|GO:0042221,response to chemical|GO:2001141,regulation of RNA biosynthetic process|GO:0071363,cellular response to growth factor stimulus|GO:0030513,positive regulation of BMP signaling pathway|GO:0010033,response to organic substance|GO:0030510,regulation of BMP signaling pathway|GO:0042325,regulation of phosphorylation|GO:0044700,single organism signaling|GO:0042327,positive regulation of phosphorylation|GO:0051216,cartilage development|GO:0051716,cellular response to stimulus|GO:0010631,epithelial cell migration|GO:0010632,regulation of epithelial cell migration|GO:0010633,negative regulation of epithelial cell migration|GO:0019538,protein metabolic process|GO:0060395,SMAD protein signal transduction|GO:0048870,cell motility|GO:0006275,regulation of DNA replication|GO:0060393,regulation of pathway-restricted SMAD protein phosphorylation|GO:0007154,cell communication|GO:0001503,ossification|GO:0019438,aromatic compound biosynthetic process|GO:0048878,chemical homeostasis|GO:0001570,vasculogenesis|GO:0009892,negative regulation of metabolic process|GO:0009893,positive regulation of metabolic process|GO:0009890,negative regulation of biosynthetic process|GO:0009891,positive regulation of biosynthetic process|GO:0055065,metal ion homeostasis|GO:0055076,transition metal ion homeostasis|GO:0006468,protein phosphorylation|GO:0006928,movement of cell or subcellular component|GO:0008152,metabolic process|GO:1901342,regulation of vasculature development|GO:0010628,positive regulation of gene expression|GO:0006807,nitrogen compound metabolic process|GO:0035556,intracellular signal transduction|GO:0010862,positive regulation of pathway-restricted SMAD protein phosphorylation|GO:0042981,regulation of apoptotic process|GO:0050789,regulation of biological process|GO:0097659,nucleic acid-templated transcription|GO:0043542,endothelial cell migration|GO:0001816,cytokine production|GO:0009653,anatomical structure morphogenesis|GO:0046916,cellular transition metal ion homeostasis|GO:0090100,positive regulation of transmembrane receptor protein serine/threonine kinase signaling pathway|GO:0060389,pathway-restricted SMAD protein phosphorylation|GO:0001569,branching involved in blood vessel morphogenesis|GO:0016043,cellular component organization|GO:0006873,cellular ion homeostasis|GO:0071840,cellular component organization or biogenesis|GO:0006366,transcription from RNA polymerase II promoter|GO:0019725,cellular homeostasis|GO:0044267,cellular protein metabolic process|GO:0016477,cell migration|GO:0022603,regulation of anatomical structure morphogenesis|GO:0048646,anatomical structure formation involved in morphogenesis|GO:0018130,heterocycle biosynthetic process|GO:0016265,obsolete death|GO:0032637,interleukin-8 production|GO:0050793,regulation of developmental process|GO:2000279,negative regulation of DNA biosynthetic process|GO:2000278,regulation of DNA biosynthetic process|GO:0009889,regulation of biosynthetic process|GO:0009888,tissue development|GO:0050794,regulation of cellular process|GO:0012501,programmed cell death|GO:0008156,negative regulation of DNA replication|GO:0001501,skeletal system development|GO:0051239,regulation of multicellular organismal process|GO:0045446,endothelial cell differentiation|GO:0034654,nucleobase-containing compound biosynthetic process|GO:0030278,regulation of ossification|GO:1902531,regulation of intracellular signal transduction|GO:0016070,RNA metabolic process|GO:0061036,positive regulation of cartilage development|GO:0044271,cellular nitrogen compound biosynthetic process|GO:0071495,cellular response to endogenous stimulus|GO:0051240,positive regulation of multicellular organismal process|GO:0050896,response to stimulus|GO:0031401,positive regulation of protein modification process|GO:0043412,macromolecule modification|GO:0032502,developmental process|GO:0006355,regulation of transcription, DNA-templated|GO:0010557,positive regulation of macromolecule biosynthetic process|GO:0010556,regulation of macromolecule biosynthetic process|GO:2000145,regulation of cell motility|GO:0006351,transcription, DNA-templated|GO:2000146,negative regulation of cell motility|GO:0016525,negative regulation of angiogenesis|GO:0010558,negative regulation of macromolecule biosynthetic process|GO:0071772,response to BMP|GO:0071773,cellular response to BMP stimulus|GO:0032774,RNA biosynthetic process|GO:0030308,negative regulation of cell growth|GO:0001944,vasculature development|GO:0050801,ion homeostasis|GO:0030154,cell differentiation|GO:0051128,regulation of cellular component organization|GO:0023056,positive regulation of signaling|GO:0044249,cellular biosynthetic process|GO:0034641,cellular nitrogen compound metabolic process|GO:0023052,signaling|GO:0070887,cellular response to chemical stimulus|GO:0023051,regulation of signaling|GO:2000181,negative regulation of blood vessel morphogenesis|GO:0010647,positive regulation of cell communication|GO:0010646,regulation of cell communication|GO:0070848,response to growth factor|GO:0044699,single-organism process|GO:0043408,regulation of MAPK cascade|GO:0009719,response to endogenous stimulus|GO:0006139,nucleobase-containing compound metabolic process|GO:0090287,regulation of cellular response to growth factor stimulus|GO:1904018,positive regulation of vasculature development|GO:0051674,localization of cell|GO:0010562,positive regulation of phosphorus metabolic process|GO:0051241,negative regulation of multicellular organismal process|GO:0051246,regulation of protein metabolic process|GO:0051247,positive regulation of protein metabolic process|GO:0032270,positive regulation of cellular protein metabolic process|GO:0036211,protein modification process|GO:0031399,regulation of protein modification process|GO:0048514,blood vessel morphogenesis|GO:0046483,heterocycle metabolic process|GO:0071310,cellular response to organic substance|GO:0032677,regulation of interleukin-8 production|GO:1903508,positive regulation of nucleic acid-templated transcription|GO:0008285,negative regulation of cell proliferation|GO:0032501,multicellular	GO:0043230,extracellular organelle|GO:0031982,vesicle|GO:0044421,extracellular region part|GO:0043227,membrane-bounded organelle|GO:0005623,cell|GO:0031988,membrane-bounded vesicle|GO:0005576,extracellular region|GO:0005615,extracellular space|GO:1903561,extracellular vesicle|GO:0065010,extracellular membrane-bounded organelle|GO:0043226,organelle|GO:0070062,extracellular exosome	GO:0005126,cytokine receptor binding|GO:0005125,cytokine activity|GO:0008083,growth factor activity|GO:0005160,transforming growth factor beta receptor binding|GO:0005488,binding|GO:0005515,protein binding|GO:0005102,receptor binding

PSMD6	GO:0080090,regulation of primary metabolic process|GO:0019222,regulation of metabolic process|GO:0048585,negative regulation of response to stimulus|GO:0048584,positive regulation of response to stimulus|GO:0048583,regulation of response to stimulus|GO:0031349,positive regulation of defense response|GO:0030111,regulation of Wnt signaling pathway|GO:0007165,signal transduction|GO:0007166,cell surface receptor signaling pathway|GO:1902402,signal transduction involved in mitotic DNA damage checkpoint|GO:1902403,signal transduction involved in mitotic DNA integrity checkpoint|GO:1902400,intracellular signal transduction involved in G1 DNA damage checkpoint|GO:0031347,regulation of defense response|GO:0044710,single-organism metabolic process|GO:0010605,negative regulation of macromolecule metabolic process|GO:0010604,positive regulation of macromolecule metabolic process|GO:0009968,negative regulation of signal transduction|GO:0072422,signal transduction involved in DNA damage checkpoint|GO:0009966,regulation of signal transduction|GO:0009967,positive regulation of signal transduction|GO:0070647,protein modification by small protein conjugation or removal|GO:0010467,gene expression|GO:0044419,interspecies interaction between organisms|GO:0032446,protein modification by small protein conjugation|GO:0033238,regulation of cellular amine metabolic process|GO:0043632,modification-dependent macromolecule catabolic process|GO:0044093,positive regulation of molecular function|GO:0044092,negative regulation of molecular function|GO:0048518,positive regulation of biological process|GO:0048519,negative regulation of biological process|GO:0060255,regulation of macromolecule metabolic process|GO:0060548,negative regulation of cell death|GO:0006952,defense response|GO:0050776,regulation of immune response|GO:0030162,regulation of proteolysis|GO:0030163,protein catabolic process|GO:0043436,oxoacid metabolic process|GO:0072413,signal transduction involved in mitotic cell cycle checkpoint|GO:0051704,multi-organism process|GO:0044700,single organism signaling|GO:0002429,immune response-activating cell surface receptor signaling pathway|GO:1901564,organonitrogen compound metabolic process|GO:0019538,protein metabolic process|GO:0022402,cell cycle process|GO:0016055,Wnt signaling pathway|GO:0051351,positive regulation of ligase activity|GO:0051352,negative regulation of ligase activity|GO:0050789,regulation of biological process|GO:0016567,protein ubiquitination|GO:0007154,cell communication|GO:0012501,programmed cell death|GO:0033554,cellular response to stress|GO:1901991,negative regulation of mitotic cell cycle phase transition|GO:0044783,G1 DNA damage checkpoint|GO:0060828,regulation of canonical Wnt signaling pathway|GO:1901990,regulation of mitotic cell cycle phase transition|GO:0009896,positive regulation of catabolic process|GO:0090090,negative regulation of canonical Wnt signaling pathway|GO:0009894,regulation of catabolic process|GO:0009892,negative regulation of metabolic process|GO:0009893,positive regulation of metabolic process|GO:0090263,positive regulation of canonical Wnt signaling pathway|GO:0030177,positive regulation of Wnt signaling pathway|GO:0065007,biological regulation|GO:0050896,response to stimulus|GO:0035556,intracellular signal transduction|GO:0072331,signal transduction by p53 class mediator|GO:0030178,negative regulation of Wnt signaling pathway|GO:0072401,signal transduction involved in DNA integrity checkpoint|GO:0000278,mitotic cell cycle|GO:0090068,positive regulation of cell cycle process|GO:0007346,regulation of mitotic cell cycle|GO:0051340,regulation of ligase activity|GO:0051347,positive regulation of transferase activity|GO:0002764,immune response-regulating signaling pathway|GO:0031570,DNA integrity checkpoint|GO:0031571,mitotic G1 DNA damage checkpoint|GO:0002768,immune response-regulating cell surface receptor signaling pathway|GO:0051348,negative regulation of transferase activity|GO:0000209,protein polyubiquitination|GO:0002684,positive regulation of immune system process|GO:0002682,regulation of immune system process|GO:0007049,cell cycle|GO:0044267,cellular protein metabolic process|GO:0065009,regulation of molecular function|GO:0044281,small molecule metabolic process|GO:0000082,G1/S transition of mitotic cell cycle|GO:0016265,obsolete death|GO:0051338,regulation of transferase activity|GO:0019882,antigen processing and presentation|GO:0019884,antigen processing and presentation of exogenous antigen|GO:0009308,amine metabolic process|GO:0050790,regulation of catalytic activity|GO:0051716,cellular response to stimulus|GO:0050794,regulation of cellular process|GO:0007050,cell cycle arrest|GO:0060070,canonical Wnt signaling pathway|GO:0043412,macromolecule modification|GO:0036211,protein modification process|GO:0002376,immune system process|GO:0008152,metabolic process|GO:0006955,immune response|GO:2000134,negative regulation of G1/S transition of mitotic cell cycle|GO:0034641,cellular nitrogen compound metabolic process|GO:1901988,negative regulation of cell cycle phase transition|GO:0045787,positive regulation of cell cycle|GO:0051444,negative regulation of ubiquitin-protein transferase activity|GO:0051603,proteolysis involved in cellular protein catabolic process|GO:0002757,immune response-activating signal transduction|GO:0051443,positive regulation of ubiquitin-protein transferase activity|GO:0031400,negative regulation of protein modification process|GO:0031401,positive regulation of protein modification process|GO:0002758,innate immune response-activating signal transduction|GO:0006950,response to stress|GO:2000060,positive regulation of protein ubiquitination involved in ubiquitin-dependent protein catabolic process|GO:0045732,positive regulation of protein catabolic process|GO:0006511,ubiquitin-dependent protein catabolic process|GO:0010564,regulation of cell cycle process|GO:0044699,single-organism process|GO:0010565,regulation of cellular ketone metabolic process|GO:0023056,positive regulation of signaling|GO:0023057,negative regulation of signaling|GO:0042176,regulation of protein catabolic process|GO:0023052,signaling|GO:0010648,negative regulation of cell communication|GO:0023051,regulation of signaling|GO:0010647,positive regulation of cell communication|GO:0010646,regulation of cell communication|GO:0044819,mitotic G1/S transition checkpoint|GO:0043086,negative regulation of catalytic activity|GO:0043085,positive regulation of catalytic activity|GO:0051248,negative regulation of protein metabolic process|GO:0043161,proteasome-mediated ubiquitin-dependent protein catabolic process|GO:0072395,signal transduction involved in cell cycle checkpoint|GO:0051246,regulation of protein metabolic process|GO:0051247,positive regulation of protein metabolic process|GO:1903320,regulation of protein modification by small protein conjugation or removal|GO:1903321,negative regulation of protein modification by small protein conjugation or removal|GO:1903322,positive regulation of protein modification by small protein conjugation or removal|GO:0032270,positive regulation of cellular protein metabolic process|GO:0031398,positive regulation of protein ubiquitination|GO:0031399,regulation of protein modification process|GO:0031396,regulation of protein ubiquitination|GO:0031397,negative regulation of protein ubiquitination|GO:1901987,regulation of cell cycle phase transition|GO:0002220,innate immune response activating cell surface receptor signaling pathway|GO:0002223,stimulatory C-type lectin receptor signaling pathway|GO:1903052,positive regulation of proteolysis involved in cellular protein catabolic process|GO:0031331,positive regulation of cellular catabolic process|GO:1903050,regulation of proteolysis involved in cellular protein catabolic process|GO:0009987,cellular process|GO:0019941,modification-dependent protein catabolic process|GO:0031145,anaphase-promoting complex-dependent catabolic process|GO:0044260,cellular macromolecule metabolic process|GO:0006974,cellular response to DNA damage stimulus|GO:0044106,cellular amine metabolic process|GO:0006977,DNA damage response, signal transduction by p53 class mediator resulting in cell cycle arrest|GO:1903047,mitotic cell cycle process|GO:0006508,proteolysis|GO:0044257,cellular protein catabolic process|GO:0016032,viral process|GO:0044764,multi-organism cellular process|GO:0032269,negative regulation of cellular protein metabolic process|GO:0032268,regulation of cellular protein metabolic process|GO:2000045,regulation of G1/S transition of mitotic cell cycle|GO:0006082,organic acid metabolic process|GO:0051726,regulation of cell cycle|GO:0050778,positive regulation of immune response|GO:0042590,antigen processing and presentation of exogenous peptide antigen via [illegible]	GO:0022624,proteasome accessory complex|GO:0031974,membrane-enclosed lumen|GO:0043229,intracellular organelle|GO:0043227,membrane-bounded organelle|GO:0043226,organelle|GO:0005737,cytoplasm|GO:0005838,proteasome regulatory particle|GO:0070062,extracellular exosome|GO:0031981,nuclear lumen|GO:0005634,nucleus|GO:0031988,membrane-bounded vesicle|GO:0005654,nucleoplasm|GO:0032991,macromolecular complex|GO:0000502,proteasome complex|GO:1903561,extracellular vesicle|GO:0031982,vesicle|GO:0043234,protein complex|GO:0043230,extracellular organelle|GO:0043231,intracellular membrane-bounded organelle|GO:0043233,organelle lumen|GO:0005829,cytosol|GO:0044464,cell part|GO:0005623,cell|GO:0005622,intracellular|GO:0044446,intracellular organelle part|GO:0070013,intracellular organelle lumen|GO:0044444,cytoplasmic part|GO:0005576,extracellular region|GO:0044428,nuclear part|GO:0044424,intracellular part|GO:0065010,extracellular membrane-bounded organelle|GO:0044421,extracellular region part|GO:0044422,organelle part|GO:0101002,ficolin-1-rich granule|GO:1904813,ficolin-1-rich granule lumen|GO:0060205,cytoplasmic membrane-bounded vesicle lumen|GO:0044433,cytoplasmic vesicle part|GO:0031983,vesicle lumen|GO:0016023,cytoplasmic, membrane-bounded vesicle|GO:0031410,cytoplasmic vesicle|GO:0099503,secretory vesicle|GO:0030141,secretory granule|GO:0097708,intracellular vesicle|GO:0034774,secretory granule lumen|GO:0012505,endomembrane system	GO:0016787,hydrolase activity|GO:0016818,hydrolase activity, acting on acid anhydrides, in phosphorus-containing anhydrides|GO:0005515,protein binding|GO:0003824,catalytic activity|GO:0017111,nucleoside-triphosphatase activity|GO:0016817,hydrolase activity, acting on acid anhydrides|GO:0016462,pyrophosphatase activity|GO:0005488,binding|GO:0016887,ATPase activity|GO:0030234,enzyme regulator activity|GO:0098772,molecular function regulator
ETFDH	GO:0008152,metabolic process|GO:0044710,single-organism metabolic process|GO:0044699,single-organism process|GO:0055114,oxidation-reduction process		GO:0003824,catalytic activity|GO:0016491,oxidoreductase activity
RNF175	GO:0044238,primary metabolic process|GO:0036503,ERAD pathway|GO:0044248,cellular catabolic process|GO:0044257,cellular protein catabolic process|GO:0023052,signaling|GO:0007165,signal transduction|GO:0070887,cellular response to chemical stimulus|GO:0042221,response to chemical|GO:0043632,modification-dependent macromolecule catabolic process|GO:0050789,regulation of biological process|GO:0044699,single-organism process|GO:0044712,single-organism catabolic process|GO:0044267,cellular protein metabolic process|GO:0044710,single-organism metabolic process|GO:0044265,cellular macromolecule catabolic process|GO:0044260,cellular macromolecule metabolic process|GO:0071704,organic substance metabolic process|GO:0043161,proteasome-mediated ubiquitin-dependent protein catabolic process|GO:0070647,protein modification by small protein conjugation or removal|GO:0010033,response to organic substance|GO:0032446,protein modification by small protein conjugation|GO:0071310,cellular response to organic substance|GO:0035966,response to topologically incorrect protein|GO:0065007,biological regulation|GO:1901575,organic substance catabolic process|GO:0006508,proteolysis|GO:0051603,proteolysis involved in cellular protein catabolic process|GO:0009987,cellular process|GO:0019941,modification-dependent protein catabolic process|GO:0051716,cellular response to stimulus|GO:0006464,cellular protein modification process|GO:0050794,regulation of cellular process|GO:0006986,response to unfolded protein|GO:0043412,macromolecule modification|GO:0036211,protein modification process|GO:0044763,single-organism cellular process|GO:0030163,protein catabolic process|GO:0008152,metabolic process|GO:0030433,ubiquitin-dependent ERAD pathway|GO:0034620,cellular response to unfolded protein|GO:0007154,cell communication|GO:0009056,catabolic process|GO:0009057,macromolecule catabolic process|GO:0044700,single organism signaling|GO:0030968,endoplasmic reticulum unfolded protein response|GO:0019538,protein metabolic process|GO:0050896,response to stimulus|GO:0010498,proteasomal protein catabolic process|GO:0006950,response to stress|GO:0044237,cellular metabolic process|GO:0043170,macromolecule metabolic process|GO:0016567,protein ubiquitination|GO:0035967,cellular response to topologically incorrect protein|GO:0006511,ubiquitin-dependent protein catabolic process|GO:0033554,cellular response to stress|GO:0034976,response to endoplasmic reticulum stress|GO:0006807,nitrogen compound metabolic process|GO:1901564,organonitrogen compound metabolic process|GO:1901565,organonitrogen compound catabolic process|GO:0010243,response to organonitrogen compound|GO:1901698,response to nitrogen compound	GO:0005783,endoplasmic reticulum|GO:0005789,endoplasmic reticulum membrane|GO:0042175,nuclear outer membrane-endoplasmic reticulum membrane network|GO:0043229,intracellular organelle|GO:0005622,intracellular|GO:0043227,membrane-bounded organelle|GO:0043226,organelle|GO:0031224,intrinsic component of membrane|GO:0005737,cytoplasm|GO:0031090,organelle membrane|GO:0016021,integral component of membrane|GO:0016020,membrane|GO:0044432,endoplasmic reticulum part|GO:0044431,Golgi apparatus part|GO:0005794,Golgi apparatus|GO:0098588,bounding membrane of organelle|GO:0012505,endomembrane system|GO:0043231,intracellular membrane-bounded organelle|GO:0044464,cell part|GO:0005623,cell|GO:0000139,Golgi membrane|GO:0044446,intracellular organelle part|GO:0044444,cytoplasmic part|GO:0044424,intracellular part|GO:0044425,membrane part|GO:0044422,organelle part|GO:0031984,organelle subcompartment|GO:0098827,endoplasmic reticulum subcompartment|GO:0098791,Golgi subcompartment	GO:0019787,ubiquitin-like protein transferase activity|GO:0043169,cation binding|GO:0046914,transition metal ion binding|GO:0061630,ubiquitin protein ligase activity|GO:0043167,ion binding|GO:0003824,catalytic activity|GO:0016740,transferase activity|GO:0008270,zinc ion binding|GO:0005515,protein binding|GO:0005488,binding|GO:0046872,metal ion binding|GO:0004842,ubiquitin-protein transferase activity|GO:0061659,ubiquitin-like protein ligase activity
LRIG2	GO:0007167,enzyme linked receptor protein signaling pathway|GO:0048583,regulation of response to stimulus|GO:0048008,platelet-derived growth factor receptor signaling pathway|GO:0023052,signaling|GO:0007165,signal transduction|GO:0007166,cell surface receptor signaling pathway|GO:0023051,regulation of signaling|GO:0007169,transmembrane receptor protein tyrosine kinase signaling pathway|GO:0010646,regulation of cell communication|GO:0050789,regulation of biological process|GO:0044699,single-organism process|GO:0051716,cellular response to stimulus|GO:0060384,innervation|GO:0009966,regulation of signal transduction|GO:0007275,multicellular organism development|GO:0065007,biological regulation|GO:0050877,neurological system process|GO:0032502,developmental process|GO:0032501,multicellular organismal process|GO:0007605,sensory perception of sound|GO:0050954,sensory perception of mechanical stimulus|GO:0009987,cellular process|GO:0007600,sensory perception|GO:0021675,nerve development|GO:0050794,regulation of cellular process|GO:0044767,single-organism developmental process|GO:0044763,single-organism cellular process|GO:0007154,cell communication|GO:0003008,system process|GO:0044700,single organism signaling|GO:0010640,regulation of platelet-derived growth factor receptor signaling pathway|GO:0044707,single-multicellular organism process|GO:0007399,nervous system development|GO:0050896,response to stimulus|GO:0048856,anatomical structure development|GO:0048731,system development|GO:0051043,regulation of membrane protein ectodomain proteolysis|GO:0051045,negative regulation of membrane protein ectodomain proteolysis|GO:0019222,regulation of metabolic process|GO:0048585,negative regulation of response to stimulus|GO:0048679,regulation of axon regeneration|GO:0048678,response to axon injury|GO:0008104,protein localization|GO:0031345,negative regulation of cell projection organization|GO:0031344,regulation of cell projection organization|GO:0071840,cellular component organization or biogenesis|GO:0080090,regulation of primary metabolic process|GO:0010605,negative regulation of macromolecule metabolic process|GO:0071704,organic substance metabolic process|GO:0048869,cellular developmental process|GO:0009611,response to wounding|GO:0045665,negative regulation of neuron differentiation|GO:0045664,regulation of neuron differentiation|GO:0010721,negative regulation of cell development|GO:0048518,positive regulation of biological process|GO:0048519,negative regulation of biological process|GO:0033036,macromolecule localization|GO:0031103,axon regeneration|GO:0031102,neuron projection regeneration|GO:0060255,regulation of macromolecule metabolic process|GO:0030162,regulation of proteolysis|GO:0030163,protein catabolic process|GO:0010975,regulation of neuron projection development|GO:1901564,organonitrogen compound metabolic process|GO:1901565,organonitrogen compound catabolic process|GO:0048870,cell motility|GO:0019538,protein metabolic process|GO:0033554,cellular response to stress|GO:2000008,regulation of protein localization to cell surface|GO:0009894,regulation of catabolic process|GO:0009895,negative regulation of catabolic process|GO:0009892,negative regulation of metabolic process|GO:0006928,movement of cell or subcellular component|GO:0006807,nitrogen compound metabolic process|GO:0001764,neuron migration|GO:0031175,neuron projection development|GO:0044267,cellular protein metabolic process|GO:1901575,organic substance catabolic process|GO:0044260,cellular macromolecule metabolic process|GO:2000010,positive regulation of protein localization to cell surface|GO:0016043,cellular component organization|GO:2001222,regulation of neuron migration|GO:0016477,cell migration|GO:0061564,axon development|GO:0050793,regulation of developmental process|GO:0048468,cell development|GO:0006950,response to stress|GO:0051239,regulation of multicellular organismal process|GO:0080134,regulation of response to stress|GO:0080135,regulation of cellular response to stress|GO:0051961,negative regulation of nervous system development|GO:0051960,regulation of nervous system development|GO:2000145,regulation of cell motility|GO:0032102,negative regulation of response to external stimulus|GO:0008152,metabolic process|GO:0032101,regulation of response to external stimulus|GO:0031099,regeneration|GO:0030154,cell differentiation|GO:0033619,membrane protein proteolysis|GO:0051129,negative regulation of cellular component organization|GO:0051128,regulation of cellular component organization|GO:1903827,regulation of cellular protein localization|GO:0042176,regulation of protein catabolic process|GO:0042177,negative regulation of protein catabolic process|GO:0060284,regulation of cell development|GO:1903829,positive regulation of cellular protein localization|GO:0032880,regulation of protein localization|GO:0050767,regulation of neurogenesis|GO:0051248,negative regulation of protein metabolic process|GO:0060341,regulation of cellular localization|GO:0051241,negative regulation of multicellular organismal process|GO:0051246,regulation of protein metabolic process|GO:0034394,protein localization to cell surface|GO:0050768,negative regulation of neurogenesis|GO:0051179,localization|GO:0070571,negative regulation of neuron projection regeneration|GO:0070570,regulation of neuron projection regeneration|GO:0006509,membrane protein ectodomain proteolysis|GO:0006508,proteolysis|GO:1903034,regulation of response to wounding|GO:1903035,negative regulation of response to wounding|GO:0031330,negative regulation of cellular catabolic process|GO:0045596,negative regulation of cell differentiation|GO:0045595,regulation of cell differentiation|GO:0040012,regulation of locomotion|GO:0032879,regulation of localization|GO:0051093,negative regulation of developmental process|GO:0032269,negative regulation of cellular protein metabolic process|GO:0032268,regulation of cellular protein metabolic process|GO:0043170,macromolecule metabolic process|GO:0051674,localization of cell|GO:0045861,negative regulation of proteolysis|GO:0031329,regulation of cellular catabolic process|GO:0030030,cell projection organization|GO:0031324,negative regulation of cellular metabolic process|GO:0031323,regulation of cellular metabolic process|GO:0048681,negative regulation of axon regeneration|GO:0009605,response to external stimulus|GO:0048666,neuron development|GO:0030334,regulation of cell migration|GO:0030182,neuron differentiation|GO:0034613,cellular protein localization|GO:0022008,neurogenesis|GO:0009056,catabolic process|GO:0009057,macromolecule catabolic process|GO:0044248,cellular catabolic process|GO:0051641,cellular localization|GO:0040011,locomotion|GO:0044238,primary metabolic process|GO:0048699,generation of neurons|GO:0051270,regulation of cellular component movement|GO:0044237,cellular metabolic process|GO:0070727,cellular macromolecule localization|GO:2000026,regulation of multicellular organismal development|GO:0010977,negative regulation of neuron projection development|GO:0048523,negative regulation of cellular process|GO:0048522,positive regulation of cellular process	GO:0005737,cytoplasm|GO:0016021,integral component of membrane|GO:0016020,membrane|GO:0044464,cell part|GO:0005623,cell|GO:0005622,intracellular|GO:0071944,cell periphery|GO:0005886,plasma membrane|GO:0044424,intracellular part|GO:0044425,membrane part|GO:0031224,intrinsic component of membrane|GO:0030427,site of polarized growth|GO:0030426,growth cone|GO:0043227,membrane-bounded organelle|GO:0043226,organelle|GO:0031982,vesicle|GO:0097458,neuron part|GO:0043005,neuron projection|GO:0043025,neuronal cell body|GO:0044297,cell body|GO:0097708,intracellular vesicle|GO:0036477,somatodendritic compartment	GO:0005102,receptor binding|GO:0005488,binding|GO:0005515,protein binding
STRADB	GO:2001234,negative regulation of apoptotic signaling pathway|GO:2001236,regulation of extrinsic apoptotic signaling pathway|GO:2001237,negative regulation of extrinsic apoptotic signaling pathway|GO:0019220,regulation of phosphate metabolic process|GO:0080090,regulation of primary metabolic process|GO:0019222,regulation of metabolic process|GO:2001233,regulation of apoptotic signaling pathway|GO:0048585,negative regulation of response to stimulus|GO:0048583,regulation of response to stimulus|GO:2001239,regulation of extrinsic apoptotic signaling pathway in absence of ligand|GO:0007266,Rho protein signal transduction|GO:0051168,nuclear export|GO:0007165,signal transduction|GO:0007166,cell surface receptor signaling pathway|GO:0007167,enzyme linked receptor protein signaling pathway|GO:0007169,transmembrane receptor protein tyrosine kinase signaling pathway|GO:0023014,signal transduction by protein phosphorylation|GO:0044710,single-organism metabolic process|GO:0045786,negative regulation of cell cycle|GO:0010604,positive regulation of macromolecule metabolic process|GO:0009968,negative regulation of signal transduction|GO:0070727,cellular macromolecule localization|GO:0009966,regulation of signal transduction|GO:0048869,cellular developmental process|GO:0071840,cellular component organization or biogenesis|GO:0000165,MAPK cascade|GO:0071310,cellular response to organic substance|GO:0008104,protein localization|GO:0044093,positive regulation of molecular function|GO:0048518,positive regulation of biological process|GO:0048519,negative regulation of biological process|GO:0033036,macromolecule localization|GO:0060255,regulation of macromolecule metabolic process|GO:0060548,negative regulation of cell death|GO:0045859,regulation of protein kinase activity|GO:0032147,activation of protein kinase activity|GO:0045184,establishment of protein localization|GO:0042221,response to chemical|GO:0043434,response to peptide hormone|GO:0010033,response to organic substance|GO:0042325,regulation of phosphorylation|GO:0044700,single organism signaling|GO:0042327,positive regulation of phosphorylation|GO:0048870,cell motility|GO:0019538,protein metabolic process|GO:0010243,response to organonitrogen compound|GO:0007154,cell communication|GO:0033554,cellular response to stress|GO:0009893,positive regulation of metabolic process|GO:0033674,positive regulation of kinase activity|GO:0006611,protein export from nucleus|GO:0006928,movement of cell or subcellular component|GO:0051674,localization of cell|GO:0030029,actin filament-based process|GO:0035556,intracellular signal transduction|GO:0042981,regulation of apoptotic process|GO:0050789,regulation of biological process|GO:0044267,cellular protein metabolic process|GO:0007346,regulation of mitotic cell cycle|GO:0009653,anatomical structure morphogenesis|GO:0051347,positive regulation of transferase activity|GO:0000902,cell morphogenesis|GO:0044260,cellular macromolecule metabolic process|GO:0006886,intracellular protein transport|GO:0043549,regulation of kinase activity|GO:0016043,cellular component organization|GO:0065007,biological regulation|GO:0007049,cell cycle|GO:0065009,regulation of molecular function|GO:0016477,cell migration|GO:0034613,cellular protein localization|GO:0016265,obsolete death|GO:0006810,transport|GO:0050790,regulation of catalytic activity|GO:0051716,cellular response to stimulus|GO:0050794,regulation of cellular process|GO:0007050,cell cycle arrest|GO:0012501,programmed cell death|GO:0043412,macromolecule modification|GO:0036211,protein modification process|GO:1901700,response to oxygen-containing compound|GO:0008152,metabolic process|GO:1902531,regulation of intracellular signal transduction|GO:0044767,single-organism developmental process|GO:0046907,intracellular transport|GO:1901698,response to nitrogen compound|GO:0031401,positive regulation of protein modification process|GO:0006950,response to stress|GO:0051338,regulation of transferase activity|GO:0051169,nuclear transport|GO:0016310,phosphorylation|GO:0031098,stress-activated protein kinase signaling cascade|GO:0023057,negative regulation of signaling|GO:0023052,signaling|GO:0010648,negative regulation of cell communication|GO:0070887,cellular response to chemical stimulus|GO:0023051,regulation of signaling|GO:0010646,regulation of cell communication|GO:0043085,positive regulation of catalytic activity|GO:0043408,regulation of MAPK cascade|GO:0009719,response to endogenous stimulus|GO:0051234,establishment of localization|GO:0071375,cellular response to peptide hormone stimulus|GO:0010562,positive regulation of phosphorus metabolic process|GO:0051246,regulation of protein metabolic process|GO:0051247,positive regulation of protein metabolic process|GO:0032270,positive regulation of cellular protein metabolic process|GO:0031399,regulation of protein modification process|GO:2001240,negative regulation of extrinsic apoptotic signaling pathway in absence of ligand|GO:0032502,developmental process|GO:0040011,locomotion|GO:0008286,insulin receptor signaling pathway|GO:0038034,signal transduction in absence of ligand|GO:0044238,primary metabolic process|GO:0031323,regulation of cellular metabolic process|GO:0009987,cellular process|GO:0032870,cellular response to hormone stimulus|GO:0030036,actin cytoskeleton organization|GO:0016482,cytosolic transport|GO:0044699,single-organism process|GO:0032268,regulation of cellular protein metabolic process|GO:0009725,response to hormone|GO:0043170,macromolecule metabolic process|GO:0045860,positive regulation of protein kinase activity|GO:0050896,response to stimulus|GO:0071495,cellular response to endogenous stimulus|GO:1901699,cellular response to nitrogen compound|GO:0031325,positive regulation of cellular metabolic process|GO:0097190,apoptotic signaling pathway|GO:0097191,extrinsic apoptotic signaling pathway|GO:0097192,extrinsic apoptotic signaling pathway in absence of ligand|GO:0022402,cell cycle process|GO:0032869,cellular response to insulin stimulus|GO:0032868,response to insulin|GO:0008219,cell death|GO:0010941,regulation of cell death|GO:0071417,cellular response to organonitrogen compound|GO:0006796,phosphate-containing compound metabolic process|GO:0032989,cellular component morphogenesis|GO:0071704,organic substance metabolic process|GO:0043067,regulation of programmed cell death|GO:0043066,negative regulation of apoptotic process|GO:0071702,organic substance transport|GO:0043069,negative regulation of programmed cell death|GO:0006468,protein phosphorylation|GO:0000278,mitotic cell cycle|GO:0045937,positive regulation of phosphate metabolic process|GO:0006915,apoptotic process|GO:0006913,nucleocytoplasmic transport|GO:0006464,cellular protein modification process|GO:0051174,regulation of phosphorus metabolic process|GO:0044765,single-organism transport|GO:0044763,single-organism cellular process|GO:0051649,establishment of localization in cell|GO:1901099,negative regulation of signal transduction in absence of ligand|GO:0007265,Ras protein signal transduction|GO:0007264,small GTPase mediated signal transduction|GO:0051179,localization|GO:1902578,single-organism localization|GO:0051641,cellular localization|GO:0006996,organelle organization|GO:1901701,cellular response to oxygen-containing compound|GO:0007010,cytoskeleton organization|GO:0051726,regulation of cell cycle|GO:0048856,anatomical structure development|GO:0044237,cellular metabolic process|GO:1902589,single-organism organelle organization|GO:1901652,response to peptide|GO:1901653,cellular response to peptide|GO:0006793,phosphorus metabolic process|GO:0015031,protein transport|GO:1902582,single-organism intracellular transport|GO:0001932,regulation of protein phosphorylation|GO:0001934,positive regulation of protein phosphorylation|GO:0048523,negative regulation of cellular process|GO:0048522,positive regulation of cellular process|GO:1901564,organonitrogen compound metabolic process|GO:0006807,nitrogen compound metabolic process|GO:0015833,peptide transport|GO:0042886,amide transport|GO:0071705,nitrogen compound transport	GO:0005737,cytoplasm|GO:0043231,intracellular membrane-bounded organelle|GO:0005829,cytosol|GO:0044464,cell part|GO:0044444,cytoplasmic part|GO:0005623,cell|GO:0043226,organelle|GO:0043229,intracellular organelle|GO:0044424,intracellular part|GO:0005622,intracellular|GO:0043227,membrane-bounded organelle|GO:0005634,nucleus|GO:0018995,host|GO:0044217,other organism part|GO:0044215,other organism|GO:0043657,host cell|GO:0016234,inclusion body|GO:0016235,aggresome|GO:0044216,other organism cell	GO:0035639,purine ribonucleoside triphosphate binding|GO:0017048,Rho GTPase binding|GO:0031267,small GTPase binding|GO:0051020,GTPase binding|GO:0004672,protein kinase activity|GO:0043167,ion binding|GO:1901363,heterocyclic compound binding|GO:0001883,purine nucleoside binding|GO:0001882,nucleoside binding|GO:0043168,anion binding|GO:0000166,nucleotide binding|GO:0004674,protein serine/threonine kinase activity|GO:0016740,transferase activity|GO:1901265,nucleoside phosphate binding|GO:0032549,ribonucleoside binding|GO:0017076,purine nucleotide binding|GO:0005524,ATP binding|GO:0016301,kinase activity|GO:0017016,Ras GTPase binding|GO:0036094,small molecule binding|GO:0003824,catalytic activity|GO:0019899,enzyme binding|GO:0030554,adenyl nucleotide binding|GO:0016773,phosphotransferase activity, alcohol group as acceptor|GO:0016772,transferase activity, transferring phosphorus-containing groups|GO:0097367,carbohydrate derivative binding|GO:0048365,Rac GTPase binding|GO:0032559,adenyl ribonucleotide binding|GO:0005515,protein binding|GO:0097159,organic cyclic compound binding|GO:0032555,purine ribonucleotide binding|GO:0032550,purine ribonucleoside binding|GO:0032553,ribonucleotide binding|GO:0005488,binding
SNCB	GO:0050808,synapse organization|GO:0009892,negative regulation of metabolic process|GO:0019222,regulation of metabolic process|GO:0043523,regulation of neuron apoptotic process|GO:0023052,signaling|GO:0018958,phenol-containing compound metabolic process|GO:0008219,cell death|GO:0010941,regulation of cell death|GO:0042981,regulation of apoptotic process|GO:0050789,regulation of biological process|GO:0044699,single-organism process|GO:0070997,neuron death|GO:0097164,ammonium ion metabolic process|GO:1901360,organic cyclic compound metabolic process|GO:0009712,catechol-containing compound metabolic process|GO:0016043,cellular component organization|GO:0043086,negative regulation of catalytic activity|GO:0071704,organic substance metabolic process|GO:0043067,regulation of programmed cell death|GO:0043066,negative regulation of apoptotic process|GO:0065007,biological regulation|GO:0071840,cellular component organization or biogenesis|GO:0044092,negative regulation of molecular function|GO:0048519,negative regulation of biological process|GO:0065009,regulation of molecular function|GO:0043069,negative regulation of programmed cell death|GO:0097285,obsolete cell-type specific apoptotic process|GO:0016265,obsolete death|GO:0006915,apoptotic process|GO:0060548,negative regulation of cell death|GO:0050790,regulation of catalytic activity|GO:0006725,cellular aromatic compound metabolic process|GO:0006584,catecholamine metabolic process|GO:0050794,regulation of cellular process|GO:0042417,dopamine metabolic process|GO:0012501,programmed cell death|GO:0051402,neuron apoptotic process|GO:0044763,single-organism cellular process|GO:0008152,metabolic process|GO:0007268,chemical synaptic transmission|GO:0007267,cell-cell signaling|GO:0007154,cell communication|GO:1901214,regulation of neuron death|GO:1901215,negative regulation of neuron death|GO:0044700,single organism signaling|GO:1901564,organonitrogen compound metabolic process|GO:0044237,cellular metabolic process|GO:0006807,nitrogen compound metabolic process|GO:0043524,negative regulation of neuron apoptotic process|GO:0009987,cellular process|GO:0048523,negative regulation of cellular process|GO:1901615,organic hydroxy compound metabolic process|GO:0042221,response to chemical|GO:0010038,response to metal ion|GO:0010035,response to inorganic substance|GO:0050896,response to stimulus|GO:0098916,anterograde trans-synaptic signaling|GO:0099536,synaptic signaling|GO:0099537,trans-synaptic signaling	GO:0016234,inclusion body|GO:0043229,intracellular organelle|GO:0045202,synapse|GO:0043227,membrane-bounded organelle|GO:0043226,organelle|GO:0005737,cytoplasm|GO:0031981,nuclear lumen|GO:0005634,nucleus|GO:0005739,mitochondrion|GO:0005654,nucleoplasm|GO:0005794,Golgi apparatus|GO:0031974,membrane-enclosed lumen|GO:0043231,intracellular membrane-bounded organelle|GO:0043233,organelle lumen|GO:0044464,cell part|GO:0005623,cell|GO:0005622,intracellular|GO:0044446,intracellular organelle part|GO:0070013,intracellular organelle lumen|GO:0044444,cytoplasmic part|GO:0044428,nuclear part|GO:0044424,intracellular part|GO:0044422,organelle part|GO:0012505,endomembrane system|GO:0005829,cytosol	GO:0043169,cation binding|GO:0055102,lipase inhibitor activity|GO:0030234,enzyme regulator activity|GO:0043167,ion binding|GO:0046872,metal ion binding|GO:0004859,phospholipase inhibitor activity|GO:0005509,calcium ion binding|GO:0004857,enzyme inhibitor activity|GO:0098772,molecular function regulator|GO:0005488,binding|GO:0046914,transition metal ion binding|GO:1903136,cuprous ion binding|GO:0005507,copper ion binding
CACHD1	GO:0070838,divalent metal ion transport|GO:0006812,cation transport|GO:0006811,ion transport|GO:0006810,transport|GO:0006816,calcium ion transport|GO:0044765,single-organism transport|GO:0030001,metal ion transport|GO:0044699,single-organism process|GO:0072511,divalent inorganic cation transport|GO:0051234,establishment of localization|GO:0051179,localization|GO:1902578,single-organism localization	GO:0044425,membrane part|GO:0016021,integral component of membrane|GO:0016020,membrane|GO:0031224,intrinsic component of membrane	
STARD3	GO:0033036,macromolecule localization|GO:0051234,establishment of localization|GO:0015918,sterol transport|GO:0006869,lipid transport|GO:0030301,cholesterol transport|GO:0044249,cellular biosynthetic process|GO:0016125,sterol metabolic process|GO:0044281,small molecule metabolic process|GO:0051641,cellular localization|GO:0044283,small molecule biosynthetic process|GO:1901362,organic cyclic compound biosynthetic process|GO:0044699,single-organism process|GO:0042445,hormone metabolic process|GO:0044710,single-organism metabolic process|GO:0044711,single-organism biosynthetic process|GO:1901360,organic cyclic compound metabolic process|GO:0015850,organic hydroxy compound transport|GO:0042448,progesterone metabolic process|GO:0051179,localization|GO:0071704,organic substance metabolic process|GO:0065007,biological regulation|GO:1902578,single-organism localization|GO:0006810,transport|GO:0071702,organic substance transport|GO:0008610,lipid biosynthetic process|GO:0006701,progesterone biosynthetic process|GO:0006700,C21-steroid hormone biosynthetic process|GO:0006629,lipid metabolic process|GO:1901576,organic substance biosynthetic process|GO:0009987,cellular process|GO:0042446,hormone biosynthetic process|GO:0010817,regulation of hormone levels|GO:0044765,single-organism transport|GO:0044763,single-organism cellular process|GO:0008152,metabolic process|GO:0051649,establishment of localization in cell|GO:0006839,mitochondrial transport|GO:0008207,C21-steroid hormone metabolic process|GO:0010876,lipid localization|GO:0034754,cellular hormone metabolic process|GO:0008202,steroid metabolic process|GO:0008203,cholesterol metabolic process|GO:0044238,primary metabolic process|GO:0065008,regulation of biological quality|GO:0042181,ketone biosynthetic process|GO:0042180,cellular ketone metabolic process|GO:0046907,intracellular transport|GO:0009058,biosynthetic process|GO:0044237,cellular metabolic process|GO:0006066,alcohol metabolic process|GO:0006694,steroid biosynthetic process|GO:1902582,single-organism intracellular transport|GO:1901615,organic hydroxy compound metabolic process|GO:1902652,secondary alcohol metabolic process	GO:0016020,membrane|GO:0000323,lytic vacuole|GO:0043229,intracellular organelle|GO:0043227,membrane-bounded organelle|GO:0043226,organelle|GO:0031224,intrinsic component of membrane|GO:0010008,endosome membrane|GO:0005737,cytoplasm|GO:0044446,intracellular organelle part|GO:0031090,organelle membrane|GO:0005773,vacuole|GO:0016021,integral component of membrane|GO:0005770,late endosome|GO:0005739,mitochondrion|GO:0005774,vacuolar membrane|GO:0044437,vacuolar part|GO:0031902,late endosome membrane|GO:0098588,bounding membrane of organelle|GO:0012505,endomembrane system|GO:0043231,intracellular membrane-bounded organelle|GO:0044464,cell part|GO:0005623,cell|GO:0005622,intracellular|GO:0005764,lysosome|GO:0005765,lysosomal membrane|GO:0044444,cytoplasmic part|GO:0044440,endosomal part|GO:0044424,intracellular part|GO:0044425,membrane part|GO:0005768,endosome|GO:0098805,whole membrane|GO:0044422,organelle part|GO:0005783,endoplasmic reticulum|GO:0005789,endoplasmic reticulum membrane|GO:0042175,nuclear outer membrane-endoplasmic reticulum membrane network|GO:0031984,organelle subcompartment|GO:0031982,vesicle|GO:0031410,cytoplasmic vesicle|GO:0044433,cytoplasmic vesicle part|GO:0044432,endoplasmic reticulum part|GO:0098827,endoplasmic reticulum subcompartment|GO:0097708,intracellular vesicle|GO:0005829,cytosol|GO:0044232,organelle membrane contact site|GO:0098852,lytic vacuole membrane	GO:0005319,lipid transporter activity|GO:0022892,substrate-specific transporter activity|GO:0015485,cholesterol binding|GO:0043178,alcohol binding|GO:0005215,transporter activity|GO:0008289,lipid binding|GO:0017127,cholesterol transporter activity|GO:0036094,small molecule binding|GO:0032934,sterol binding|GO:0005488,binding|GO:0005515,protein binding|GO:0097159,organic cyclic compound binding|GO:0005496,steroid binding|GO:0015248,sterol transporter activity|GO:0046983,protein dimerization activity|GO:0042802,identical protein binding|GO:0042803,protein homodimerization activity
PRR15L			
SYT2	GO:0051046,regulation of secretion|GO:0048588,developmental cell growth|GO:0051049,regulation of transport|GO:0048468,cell development|GO:0001505,regulation of neurotransmitter levels|GO:1903861,positive regulation of dendrite extension|GO:0051656,establishment of organelle localization|GO:0031344,regulation of cell projection organization|GO:0071840,cellular component organization or biogenesis|GO:0031346,positive regulation of cell projection organization|GO:0048589,developmental growth|GO:0048869,cellular developmental process|GO:0045664,regulation of neuron differentiation|GO:0045666,positive regulation of neuron differentiation|GO:0010720,positive regulation of cell development|GO:0048518,positive regulation of biological process|GO:0097479,synaptic vesicle localization|GO:1990138,neuron projection extension|GO:0010976,positive regulation of neuron projection development|GO:0010975,regulation of neuron projection development|GO:0006836,neurotransmitter transport|GO:0097484,dendrite extension|GO:0097480,establishment of synaptic vesicle localization|GO:0044700,single organism signaling|GO:0016192,vesicle-mediated transport|GO:0044707,single-multicellular organism process|GO:0022604,regulation of cell morphogenesis|GO:0022603,regulation of anatomical structure morphogenesis|GO:0032940,secretion by cell|GO:0036465,synaptic vesicle recycling|GO:0031175,neuron projection development|GO:0050789,regulation of biological process|GO:0048488,synaptic vesicle endocytosis|GO:0048489,synaptic vesicle transport|GO:0016049,cell growth|GO:0000902,cell morphogenesis|GO:0006887,exocytosis|GO:0016043,cellular component organization|GO:0051650,establishment of vesicle localization|GO:0065007,biological regulation|GO:0065008,regulation of biological quality|GO:0051130,positive regulation of cellular component organization|GO:0050793,regulation of developmental process|GO:0006810,transport|GO:0050794,regulation of cellular process|GO:0051239,regulation of multicellular organismal process|GO:0051234,establishment of localization|GO:0046903,secretion|GO:0006897,endocytosis|GO:0016079,synaptic vesicle exocytosis|GO:0048791,calcium ion-regulated exocytosis of neurotransmitter|GO:0051962,positive regulation of nervous system development|GO:0048812,neuron projection morphogenesis|GO:0048639,positive regulation of developmental growth|GO:0048638,regulation of developmental growth|GO:0051648,vesicle localization|GO:0030307,positive regulation of cell growth|GO:0030154,cell differentiation|GO:0051128,regulation of cellular component organization|GO:0023052,signaling|GO:1903530,regulation of secretion by cell|GO:0009653,anatomical structure morphogenesis|GO:0044699,single-organism process|GO:0050767,regulation of neurogenesis|GO:0051649,establishment of localization in cell|GO:0051240,positive regulation of multicellular organismal process|GO:0060284,regulation of cell development|GO:0050769,positive regulation of neurogenesis|GO:0017158,regulation of calcium ion-dependent exocytosis|GO:0051640,organelle localization|GO:0060560,developmental growth involved in morphogenesis|GO:0017156,calcium ion regulated exocytosis|GO:0017157,regulation of exocytosis|GO:0032502,developmental process|GO:0032501,multicellular organismal process|GO:0009987,cellular process|GO:0060627,regulation of vesicle-mediated transport|GO:0045597,positive regulation of cell differentiation|GO:0045595,regulation of cell differentiation|GO:0001558,regulation of cell growth|GO:0032879,regulation of localization|GO:0048858,cell projection morphogenesis|GO:0051094,positive regulation of developmental process|GO:1903859,regulation of dendrite extension|GO:0048731,system development|GO:0045927,positive regulation of growth|GO:0060341,regulation of cellular localization|GO:0030030,cell projection organization|GO:0007275,multicellular organism development|GO:0040007,growth|GO:0040008,regulation of growth|GO:0032989,cellular component morphogenesis|GO:0051960,regulation of nervous system development|GO:0048666,neuron development|GO:0030182,neuron differentiation|GO:0023061,signal release|GO:0044767,single-organism developmental process|GO:0044765,single-organism transport|GO:0044763,single-organism cellular process|GO:0007269,neurotransmitter secretion|GO:0007268,chemical synaptic transmission|GO:0007267,cell-cell signaling|GO:0007154,cell communication|GO:0022008,neurogenesis|GO:0051179,localization|GO:1902578,single-organism localization|GO:0051641,cellular localization|GO:0048699,generation of neurons|GO:0032990,cell part morphogenesis|GO:0007399,nervous system development|GO:0048856,anatomical structure development|GO:2000026,regulation of multicellular organismal development|GO:1902580,single-organism cellular localization|GO:0048522,positive regulation of cellular process|GO:0006906,vesicle fusion|GO:0061025,membrane fusion|GO:0061024,membrane organization|GO:0099504,synaptic vesicle cycle|GO:0090174,organelle membrane fusion|GO:0016050,vesicle organization|GO:0098916,anterograde trans-synaptic signaling|GO:0045055,regulated exocytosis|GO:1903305,regulation of regulated secretory pathway|GO:0044802,single-organism membrane organization|GO:0044801,single-organism membrane fusion|GO:0046907,intracellular transport|GO:0006898,receptor-mediated endocytosis|GO:0099531,presynaptic process involved in chemical synaptic transmission|GO:0099536,synaptic signaling|GO:0099537,trans-synaptic signaling|GO:0099643,signal release from synapse|GO:0072583,clathrin-mediated endocytosis|GO:0006996,organelle organization|GO:1902589,single-organism organelle organization|GO:0048284,organelle fusion|GO:1902582,single-organism intracellular transport	GO:0008021,synaptic vesicle|GO:0043229,intracellular organelle|GO:0071944,cell periphery|GO:0045202,synapse|GO:0043227,membrane-bounded organelle|GO:0043226,organelle|GO:0030054,cell junction|GO:0005737,cytoplasm|GO:0030667,secretory granule membrane|GO:0031982,vesicle|GO:0016023,cytoplasmic, membrane-bounded vesicle|GO:0031410,cytoplasmic vesicle|GO:0016021,integral component of membrane|GO:0016020,membrane|GO:0031988,membrane-bounded vesicle|GO:0044433,cytoplasmic vesicle part|GO:0097458,neuron part|GO:0044456,synapse part|GO:0031224,intrinsic component of membrane|GO:0030141,secretory granule|GO:0098588,bounding membrane of organelle|GO:0030659,cytoplasmic vesicle membrane|GO:0042583,chromaffin granule|GO:0042584,chromaffin granule membrane|GO:0012506,vesicle membrane|GO:0005886,plasma membrane|GO:0031090,organelle membrane|GO:0030672,synaptic vesicle membrane|GO:0043231,intracellular membrane-bounded organelle|GO:0044464,cell part|GO:0005623,cell|GO:0005622,intracellular|GO:0044446,intracellular organelle part|GO:0044444,cytoplasmic part|GO:0098793,presynapse|GO:0044424,intracellular part|GO:0044425,membrane part|GO:0098805,whole membrane|GO:0044422,organelle part|GO:0012505,endomembrane system|GO:0030133,transport vesicle|GO:0030135,coated vesicle|GO:0030136,clathrin-coated vesicle|GO:0030665,clathrin-coated vesicle membrane|GO:0030662,coated vesicle membrane|GO:0070382,exocytic vesicle|GO:0099501,exocytic vesicle membrane|GO:0099503,secretory vesicle|GO:0030658,transport vesicle membrane|GO:0097708,intracellular vesicle	GO:0043168,anion binding|GO:0043169,cation binding|GO:0000149,SNARE binding|GO:0005543,phospholipid binding|GO:0005544,calcium-dependent phospholipid binding|GO:0008289,lipid binding|GO:0043167,ion binding|GO:0019905,syntaxin binding|GO:0005509,calcium ion binding|GO:0005515,protein binding|GO:0030276,clathrin binding|GO:0046872,metal ion binding|GO:0005488,binding|GO:0043178,alcohol binding|GO:0036094,small molecule binding|GO:0043533,inositol 1,3,4,5 tetrakisphosphate binding

BRPF3			GO:0043169,cation binding|GO:0046914,transition metal ion binding|GO:0043167,ion binding|GO:0005488,binding|GO:0046872,metal ion binding|GO:0008270,zinc ion binding
NLRC5			
C12orf76		GO:0044425,membrane part|GO:0016021,integral component of membrane|GO:0016020,membrane|GO:0031224,intrinsic component of membrane	
BOD1	GO:0006996,organelle organization|GO:0044699,single-organism process|GO:0000278,mitotic cell cycle|GO:0071840,cellular component organization or biogenesis|GO:0009987,cellular process|GO:0000280,nuclear division|GO:1903047,mitotic cell cycle process|GO:0016043,cellular component organization|GO:1902589,single-organism organelle organization|GO:0022402,cell cycle process|GO:0051301,cell division|GO:0044763,single-organism cellular process|GO:0007067,mitotic nuclear division|GO:0007049,cell cycle|GO:0048285,organelle fission|GO:0033036,macromolecule localization|GO:0008104,protein localization|GO:0032515,negative regulation of phosphoprotein phosphatase activity|GO:0080090,regulation of primary metabolic process|GO:0019222,regulation of metabolic process|GO:0016311,dephosphorylation|GO:0010921,regulation of phosphatase activity|GO:0031134,sister chromatid biorientation|GO:0010923,negative regulation of phosphatase activity|GO:0031323,regulation of cellular metabolic process|GO:1901564,organonitrogen compound metabolic process|GO:0008608,attachment of spindle microtubules to kinetochore|GO:1990758,mitotic sister chromatid biorientation|GO:0044092,negative regulation of molecular function|GO:0006807,nitrogen compound metabolic process|GO:0044237,cellular metabolic process|GO:0051641,cellular localization|GO:0007064,mitotic sister chromatid cohesion|GO:0009892,negative regulation of metabolic process|GO:0051179,localization|GO:0051656,establishment of organelle localization|GO:0051303,establishment of chromosome localization|GO:0050789,regulation of biological process|GO:0019220,regulation of phosphate metabolic process|GO:0071962,mitotic sister chromatid cohesion, centromeric|GO:0044267,cellular protein metabolic process|GO:0051248,negative regulation of protein metabolic process|GO:0010605,negative regulation of macromolecule metabolic process|GO:0051346,negative regulation of hydrolase activity|GO:0070601,centromeric sister chromatid cohesion|GO:0070727,cellular macromolecule localization|GO:0010563,negative regulation of phosphorus metabolic process|GO:0051246,regulation of protein metabolic process|GO:0051315,attachment of mitotic spindle microtubules to kinetochore|GO:0071704,organic substance metabolic process|GO:0007080,mitotic metaphase plate congression|GO:0065007,biological regulation|GO:0051640,organelle localization|GO:0031399,regulation of protein modification process|GO:0071459,protein localization to chromosome, centromeric region|GO:0048519,negative regulation of biological process|GO:0065009,regulation of molecular function|GO:0007062,sister chromatid cohesion|GO:0034613,cellular protein localization|GO:0031324,negative regulation of cellular metabolic process|GO:0045936,negative regulation of phosphate metabolic process|GO:0060255,regulation of macromolecule metabolic process|GO:0050000,chromosome localization|GO:0044260,cellular macromolecule metabolic process|GO:0050790,regulation of catalytic activity|GO:0006464,cellular protein modification process|GO:0050794,regulation of cellular process|GO:0051174,regulation of phosphorus metabolic process|GO:0035308,negative regulation of protein dephosphorylation|GO:0043412,macromolecule modification|GO:0000070,mitotic sister chromatid segregation|GO:0008152,metabolic process|GO:0051649,establishment of localization in cell|GO:0051310,metaphase plate congression|GO:0007059,chromosome segregation|GO:0035303,regulation of dephosphorylation|GO:0051234,establishment of localization|GO:0035305,negative regulation of dephosphorylation|GO:0035304,regulation of protein dephosphorylation|GO:0034502,protein localization to chromosome|GO:0051276,chromosome organization|GO:0051336,regulation of hydrolase activity|GO:0044238,primary metabolic process|GO:0032269,negative regulation of cellular protein metabolic process|GO:0032268,regulation of cellular protein metabolic process|GO:0000819,sister chromatid segregation|GO:0043666,regulation of phosphoprotein phosphatase activity|GO:0019538,protein metabolic process|GO:0031400,negative regulation of protein modification process|GO:0098813,nuclear chromosome segregation|GO:0043086,negative regulation of catalytic activity|GO:0043170,macromolecule metabolic process|GO:0006796,phosphate-containing compound metabolic process|GO:0036211,protein modification process|GO:0006470,protein dephosphorylation|GO:0006793,phosphorus metabolic process|GO:0033365,protein localization to organelle|GO:0048523,negative regulation of cellular process	GO:0043229,intracellular organelle|GO:0043228,non-membrane-bounded organelle|GO:0043226,organelle|GO:0005856,cytoskeleton|GO:0005815,microtubule organizing center|GO:0044430,cytoskeletal part|GO:0000776,kinetochore|GO:0005694,chromosome|GO:0005737,cytoplasm|GO:0015630,microtubule cytoskeleton|GO:0000793,condensed chromosome|GO:0000775,chromosome, centromeric region|GO:0098687,chromosomal region|GO:0000777,condensed chromosome kinetochore|GO:0043234,protein complex|GO:0032991,macromolecular complex|GO:0043232,intracellular non-membrane-bounded organelle|GO:0000779,condensed chromosome, centromeric region|GO:0044464,cell part|GO:0005623,cell|GO:0005622,intracellular|GO:0044446,intracellular organelle part|GO:0044424,intracellular part|GO:0044427,chromosomal part|GO:0044422,organelle part|GO:0005819,spindle|GO:0099512,supramolecular fiber|GO:0099513,polymeric cytoskeletal fiber|GO:0005813,centrosome|GO:0000940,condensed chromosome outer kinetochore|GO:0000922,spindle pole|GO:0005874,microtubule|GO:0005876,spindle microtubule	GO:0051721,protein phosphatase 2A binding|GO:0019212,phosphatase inhibitor activity|GO:0019888,protein phosphatase regulator activity|GO:0030234,enzyme regulator activity|GO:0019899,enzyme binding|GO:0019903,protein phosphatase binding|GO:0019902,phosphatase binding|GO:0004864,protein phosphatase inhibitor activity|GO:0005488,binding|GO:0005515,protein binding|GO:0004857,enzyme inhibitor activity|GO:0098772,molecular function regulator|GO:0019208,phosphatase regulator activity
WASL	GO:2000369,regulation of clathrin-mediated endocytosis|GO:0044238,primary metabolic process|GO:0006909,phagocytosis|GO:0080090,regulation of primary metabolic process|GO:0019222,regulation of metabolic process|GO:0051049,regulation of transport|GO:0048584,positive regulation of response to stimulus|GO:0048583,regulation of response to stimulus|GO:0006900,membrane budding|GO:0008064,regulation of actin polymerization or depolymerization|GO:0061024,membrane organization|GO:0060491,regulation of cell projection assembly|GO:0008104,protein localization|GO:0007165,signal transduction|GO:0007166,cell surface receptor signaling pathway|GO:0007167,enzyme linked receptor protein signaling pathway|GO:0007169,transmembrane receptor protein tyrosine kinase signaling pathway|GO:0048260,positive regulation of receptor-mediated endocytosis|GO:0051656,establishment of organelle localization|GO:1901362,organic cyclic compound biosynthetic process|GO:0031344,regulation of cell projection organization|GO:0051653,spindle localization|GO:0031346,positive regulation of cell projection organization|GO:0065003,macromolecular complex assembly|GO:0044710,single-organism metabolic process|GO:0042330,taxis|GO:0070727,cellular macromolecule localization|GO:0048869,cellular developmental process|GO:0051495,positive regulation of cytoskeleton organization|GO:0070271,protein complex biogenesis|GO:0051493,regulation of cytoskeleton organization|GO:0002684,positive regulation of immune system process|GO:0051491,positive regulation of filopodium assembly|GO:0019438,aromatic compound biosynthetic process|GO:0048518,positive regulation of biological process|GO:0048519,negative regulation of biological process|GO:0033036,macromolecule localization|GO:0040011,locomotion|GO:0048699,generation of neurons|GO:0006935,chemotaxis|GO:0051050,positive regulation of transport|GO:0060255,regulation of macromolecule metabolic process|GO:0048468,cell development|GO:0006952,defense response|GO:0072583,clathrin-mediated endocytosis|GO:0030048,actin filament-based movement|GO:2001141,regulation of RNA biosynthetic process|GO:0030041,actin filament polymerization|GO:0097485,neuron projection guidance|GO:0051707,response to other organism|GO:0046483,heterocycle metabolic process|GO:0031503,protein complex localization|GO:0044700,single organism signaling|GO:0002429,immune response-activating cell surface receptor signaling pathway|GO:0009607,response to biotic stimulus|GO:0016192,vesicle-mediated transport|GO:0044707,single-multicellular organism process|GO:0048870,cell motility|GO:0010638,positive regulation of organelle organization|GO:0007009,plasma membrane organization|GO:0016050,vesicle organization|GO:0002376,immune system process|GO:0034315,regulation of Arp2/3 complex-mediated actin nucleation|GO:0046847,filopodium assembly|GO:0032535,regulation of cellular component size|GO:0044281,small molecule metabolic process|GO:0022607,cellular component assembly|GO:0048667,cell morphogenesis involved in neuron differentiation|GO:0006950,response to stress|GO:0006928,movement of cell or subcellular component|GO:0065007,biological regulation|GO:0006807,nitrogen compound metabolic process|GO:0031175,neuron projection development|GO:0030029,actin filament-based process|GO:0035556,intracellular signal transduction|GO:0006955,immune response|GO:0050789,regulation of biological process|GO:0097659,nucleic acid-templated transcription|GO:0009605,response to external stimulus|GO:1901576,organic substance biosynthetic process|GO:0051341,regulation of oxidoreductase activity|GO:0000904,cell morphogenesis involved in differentiation|GO:0002764,immune response-regulating signaling pathway|GO:0044260,cellular macromolecule metabolic process|GO:0002431,Fc receptor mediated stimulatory signaling pathway|GO:0002768,immune response-regulating cell surface receptor signaling pathway|GO:0002433,immune response-regulating cell surface receptor signaling pathway involved in phagocytosis|GO:0016043,cellular component organization|GO:0090066,regulation of anatomical structure size|GO:0002686,negative regulation of leukocyte migration|GO:2000401,regulation of lymphocyte migration|GO:2000402,negative regulation of lymphocyte migration|GO:0002685,regulation of leukocyte migration|GO:0002682,regulation of immune system process|GO:0002683,negative regulation of immune system process|GO:0002252,immune effector process|GO:0065009,regulation of molecular function|GO:0016477,cell migration|GO:2000370,positive regulation of clathrin-mediated endocytosis|GO:0034613,cellular protein localization|GO:0018130,heterocycle biosynthetic process|GO:0051130,positive regulation of cellular component organization|GO:0006810,transport|GO:0061564,axon development|GO:0032768,regulation of monooxygenase activity|GO:0050790,regulation of catalytic activity|GO:0009889,regulation of biosynthetic process|GO:0051716,cellular response to stimulus|GO:0050794,regulation of cellular process|GO:1903526,negative regulation of membrane tubulation|GO:0072676,lymphocyte migration|GO:1903525,regulation of membrane tubulation|GO:0044802,single-organism membrane organization|GO:1903729,regulation of plasma membrane organization|GO:0008152,metabolic process|GO:0032956,regulation of actin cytoskeleton organization|GO:0034654,nucleobase-containing compound biosynthetic process|GO:0048013,ephrin receptor signaling pathway|GO:0051234,establishment of localization|GO:0038096,Fc-gamma receptor signaling pathway involved in phagocytosis|GO:0008154,actin polymerization or depolymerization|GO:0038094,Fc-gamma receptor signaling pathway|GO:0002757,immune response-activating signal transduction|GO:0044271,cellular nitrogen compound biosynthetic process|GO:0038093,Fc receptor signaling pathway|GO:0050896,response to stimulus|GO:0006898,receptor-mediated endocytosis|GO:0009058,biosynthetic process|GO:0006355,regulation of transcription, DNA-templated|GO:0010556,regulation of macromolecule biosynthetic process|GO:2000145,regulation of cell motility|GO:0006351,transcription, DNA-templated|GO:2000146,negative regulation of cell motility|GO:0043207,response to external biotic stimulus|GO:0051127,positive regulation of actin nucleation|GO:0051125,regulation of actin nucleation|GO:0009617,response to bacterium|GO:0032774,RNA biosynthetic process|GO:0033043,regulation of organelle organization|GO:0048259,regulation of receptor-mediated endocytosis|GO:0010256,endomembrane system organization|GO:0030154,cell differentiation|GO:0016070,RNA metabolic process|GO:0051129,negative regulation of cellular component organization|GO:0051128,regulation of cellular component organization|GO:0044249,cellular biosynthetic process|GO:0034641,cellular nitrogen compound metabolic process|GO:0023052,signaling|GO:0034314,Arp2/3 complex-mediated actin nucleation|GO:0034645,cellular macromolecule biosynthetic process|GO:0042221,response to chemical|GO:0007411,axon guidance|GO:0009653,anatomical structure morphogenesis|GO:0007067,mitotic nuclear division|GO:0043254,regulation of protein complex assembly|GO:0007049,cell cycle|GO:0032880,regulation of protein localization|GO:0006139,nucleobase-containing compound metabolic process|GO:0043623,cellular protein complex assembly|GO:0000280,nuclear division|GO:0043933,macromolecular complex subunit organization|GO:0032271,regulation of protein polymerization|GO:0032273,positive regulation of protein polymerization|GO:0051641,cellular localization|GO:0032502,developmental process|GO:0006996,organelle organization|GO:2000601,positive regulation of Arp2/3 complex-mediated actin nucleation|GO:0007015,actin filament organization|GO:0032970,regulation of actin filament-based process|GO:0031334,positive regulation of protein complex assembly|GO:0040013,negative regulation of locomotion|GO:1903506,regulation of nucleic acid-templated transcription|GO:0060627,regulation of vesicle-mediated transport|GO:0040012,regulation of locomotion|GO:1903047,mitotic cell cycle process|GO:0007409,axonogenesis|GO:0006897,endocytosis|GO:0032879,regulation of localization|GO:0032990,cell part morphogenesis|GO:0030031,cell projection assembly|GO:0051258,protein polymerization|GO:0044699,single-organism process|GO:0050776,regulation of immune response|GO:0051674,localization of cell|GO:0065008,regulation of biological quality|GO:0051252,regulation of RNA metabolic process|GO:0051650,establishment of vesicle localization|GO:0050778,positive regulation of immune response|GO:0043170,macromolecule metabolic process|GO:0097320,membrane tubulation|GO:0048731,system development|GO:0009987,cellular process|GO:0072593,reactive oxygen species metabolic	GO:0042995,cell projection|GO:0015629,actin cytoskeleton|GO:0044421,extracellular region part|GO:0030139,endocytic vesicle|GO:0043229,intracellular organelle|GO:0043228,non-membrane-bounded organelle|GO:0030864,cortical actin cytoskeleton|GO:0043227,membrane-bounded organelle|GO:0043226,organelle|GO:0005856,cytoskeleton|GO:0030666,endocytic vesicle membrane|GO:0070062,extracellular exosome|GO:0016023,cytoplasmic, membrane-bounded vesicle|GO:0031410,cytoplasmic vesicle|GO:0005634,nucleus|GO:0016020,membrane|GO:0005737,cytoplasm|GO:0031988,membrane-bounded vesicle|GO:0044433,cytoplasmic vesicle part|GO:0030027,lamellipodium|GO:0044431,Golgi apparatus part|GO:0044430,cytoskeletal part|GO:0005576,extracellular region|GO:0005794,Golgi apparatus|GO:0005622,intracellular|GO:0030478,actin cap|GO:0098588,bounding membrane of organelle|GO:0031252,cell leading edge|GO:0030659,cytoplasmic vesicle membrane|GO:0005938,cell cortex|GO:0012506,vesicle membrane|GO:0005886,plasma membrane|GO:1903561,extracellular vesicle|GO:0031982,vesicle|GO:0043230,extracellular organelle|GO:0043231,intracellular membrane-bounded organelle|GO:0043232,intracellular non-membrane-bounded organelle|GO:0030863,cortical cytoskeleton|GO:0005829,cytosol|GO:0044464,cell part|GO:0005623,cell|GO:0000139,Golgi membrane|GO:0044446,intracellular organelle part|GO:0044448,cell cortex part|GO:0044444,cytoplasmic part|GO:0071944,cell periphery|GO:0031090,organelle membrane|GO:0044424,intracellular part|GO:0065010,extracellular membrane-bounded organelle|GO:0098805,whole membrane|GO:0044422,organelle part|GO:0012505,endomembrane system|GO:0099568,cytoplasmic region|GO:0097708,intracellular vesicle	GO:0008092,cytoskeletal protein binding|GO:0030234,enzyme regulator activity|GO:0003779,actin binding|GO:0030695,GTPase regulator activity|GO:0005488,binding|GO:0005515,protein binding|GO:0098772,molecular function regulator|GO:0060589,nucleoside-triphosphatase regulator activity
RHOBTB3	GO:0044700,single organism signaling|GO:0051716,cellular response to stimulus|GO:0050896,response to stimulus|GO:0009987,cellular process|GO:0044763,single-organism cellular process|GO:0050794,regulation of cellular process|GO:0065007,biological regulation|GO:0023052,signaling|GO:0007165,signal transduction|GO:0007154,cell communication|GO:0035556,intracellular signal transduction|GO:0007264,small GTPase mediated signal transduction|GO:0050789,regulation of biological process|GO:0044699,single-organism process|GO:0006810,transport|GO:0016192,vesicle-mediated transport|GO:0051234,establishment of localization|GO:0051179,localization	GO:0044464,cell part|GO:0005623,cell|GO:0005622,intracellular	GO:0043168,anion binding|GO:0005488,binding|GO:0019001,guanyl nucleotide binding|GO:0035639,purine ribonucleoside triphosphate binding|GO:0097367,carbohydrate derivative binding|GO:0043167,ion binding|GO:1901363,heterocyclic compound binding|GO:1901265,nucleoside phosphate binding|GO:0000166,nucleotide binding|GO:0036094,small molecule binding|GO:0032561,guanyl ribonucleotide binding|GO:0032553,ribonucleotide binding|GO:0032549,ribonucleoside binding|GO:0032555,purine ribonucleotide binding|GO:0017076,purine nucleotide binding|GO:0005525,GTP binding|GO:0097159,organic cyclic compound binding|GO:0032550,purine ribonucleoside binding|GO:0001883,purine nucleoside binding|GO:0001882,nucleoside binding|GO:0019899,enzyme binding|GO:0031267,small GTPase binding|GO:0051020,GTPase binding|GO:0016887,ATPase activity|GO:0016787,hydrolase activity|GO:0003924,GTPase activity|GO:0017111,nucleoside-triphosphatase activity|GO:0017137,Rab GTPase binding|GO:0003824,catalytic activity|GO:0017016,Ras GTPase binding|GO:0016818,hydrolase activity, acting on acid anhydrides, in phosphorus-containing anhydrides|GO:0016817,hydrolase activity, acting on acid anhydrides|GO:0016462,pyrophosphatase activity|GO:0005515,protein binding
PKN2	GO:0080090,regulation of primary metabolic process|GO:0019222,regulation of metabolic process|GO:0007165,signal transduction|GO:1901576,organic substance biosynthetic process|GO:1901362,organic cyclic compound biosynthetic process|GO:0071840,cellular component organization or biogenesis|GO:0051716,cellular response to stimulus|GO:0045787,positive regulation of cell cycle|GO:0090132,epithelium migration|GO:0044419,interspecies interaction between organisms|GO:0019058,viral life cycle|GO:0016310,phosphorylation|GO:0048518,positive regulation of biological process|GO:0051704,multi-organism process|GO:0060255,regulation of macromolecule metabolic process|GO:2001141,regulation of RNA biosynthetic process|GO:0046483,heterocycle metabolic process|GO:0000910,cytokinesis|GO:0044700,single organism signaling|GO:0010631,epithelial cell migration|GO:0044707,single-multicellular organism process|GO:0048870,cell motility|GO:0019538,protein metabolic process|GO:0051781,positive regulation of cell division|GO:0007043,cell-cell junction assembly|GO:0022607,cellular component assembly|GO:0006928,movement of cell or subcellular component|GO:0006807,nitrogen compound metabolic process|GO:0035556,intracellular signal transduction|GO:0050789,regulation of biological process|GO:0097659,nucleic acid-templated transcription|GO:0090068,positive regulation of cell cycle process|GO:0007346,regulation of mitotic cell cycle|GO:0044260,cellular macromolecule metabolic process|GO:0016043,cellular component organization|GO:0065007,biological regulation|GO:0044699,single-organism process|GO:0016477,cell migration|GO:0018130,heterocycle biosynthetic process|GO:0016265,obsolete death|GO:0050792,regulation of viral process|GO:0009889,regulation of biosynthetic process|GO:0050794,regulation of cellular process|GO:0043902,positive regulation of multi-organism process|GO:0043903,regulation of symbiosis, encompassing mutualism through parasitism|GO:0043900,regulation of multi-organism process|GO:0036211,protein modification process|GO:0008152,metabolic process|GO:0019438,aromatic compound biosynthetic process|GO:0034654,nucleobase-containing compound biosynthetic process|GO:0016070,RNA metabolic process|GO:0044271,cellular nitrogen compound biosynthetic process|GO:0012501,programmed cell death|GO:0050896,response to stimulus|GO:0043412,macromolecule modification|GO:0006355,regulation of transcription, DNA-templated|GO:0010556,regulation of macromolecule biosynthetic process|GO:2000145,regulation of cell motility|GO:0006351,transcription, DNA-templated|GO:0032774,RNA biosynthetic process|GO:0045070,positive regulation of viral genome replication|GO:0044249,cellular biosynthetic process|GO:0034641,cellular nitrogen compound metabolic process|GO:0023052,signaling|GO:0034645,cellular macromolecule biosynthetic process|GO:0001667,ameboidal-type cell migration|GO:0007049,cell cycle|GO:0006139,nucleobase-containing compound metabolic process|GO:1903900,regulation of viral life cycle|GO:1903902,positive regulation of viral life cycle|GO:0010564,regulation of cell cycle process|GO:0022610,biological adhesion|GO:0032501,multicellular organismal process|GO:0019079,viral genome replication|GO:0009987,cellular process|GO:0006725,cellular aromatic compound metabolic process|GO:1903506,regulation of nucleic acid-templated transcription|GO:0051270,regulation of cellular component movement|GO:0045216,cell-cell junction organization|GO:0032879,regulation of localization|GO:0034329,cell junction assembly|GO:0090130,tissue migration|GO:0045069,regulation of viral genome replication|GO:0051252,regulation of RNA metabolic process|GO:0009059,macromolecule biosynthetic process|GO:0043170,macromolecule metabolic process|GO:0051674,localization of cell|GO:0031326,regulation of cellular biosynthetic process|GO:0034330,cell junction organization|GO:0031323,regulation of cellular metabolic process|GO:0090304,nucleic acid metabolic process|GO:0022402,cell cycle process|GO:0008219,cell death|GO:0051302,regulation of cell division|GO:0051301,cell division|GO:1901360,organic cyclic compound metabolic process|GO:2000112,regulation of cellular macromolecule biosynthetic process|GO:0071704,organic substance metabolic process|GO:0010467,gene expression|GO:0032465,regulation of cytokinesis|GO:0032467,positive regulation of cytokinesis|GO:0010468,regulation of gene expression|GO:0045931,positive regulation of mitotic cell cycle|GO:0006468,protein phosphorylation|GO:0000278,mitotic cell cycle|GO:0044267,cellular protein metabolic process|GO:0019219,regulation of nucleobase-containing compound metabolic process|GO:0006915,apoptotic process|GO:0006464,cellular protein modification process|GO:0009058,biosynthetic process|GO:0044764,multi-organism cellular process|GO:0044763,single-organism cellular process|GO:0051171,regulation of nitrogen compound metabolic process|GO:0044403,symbiosis, encompassing mutualism through parasitism|GO:0007155,cell adhesion|GO:0007154,cell communication|GO:0007264,small GTPase mediated signal transduction|GO:0051179,localization|GO:0040011,locomotion|GO:0044238,primary metabolic process|GO:0040012,regulation of locomotion|GO:0051726,regulation of cell cycle|GO:0044237,cellular metabolic process|GO:0006796,phosphate-containing compound metabolic process|GO:0044085,cellular component biogenesis|GO:0016032,viral process|GO:0006793,phosphorus metabolic process|GO:0048524,positive regulation of viral process|GO:0043297,apical junction assembly|GO:0048522,positive regulation of cellular process|GO:0018193,peptidyl-amino acid modification|GO:1901564,organonitrogen compound metabolic process|GO:0018209,peptidyl-serine modification|GO:0018105,peptidyl-serine phosphorylation|GO:0030030,cell projection organization	GO:0044464,cell part|GO:0031974,membrane-enclosed lumen|GO:0043229,intracellular organelle|GO:0043228,non-membrane-bounded organelle|GO:0097610,cell surface furrow|GO:0043227,membrane-bounded organelle|GO:0043226,organelle|GO:0030054,cell junction|GO:0005856,cytoskeleton|GO:0045111,intermediate filament cytoskeleton|GO:0031981,nuclear lumen|GO:0005813,centrosome|GO:0005634,nucleus|GO:0016020,membrane|GO:0005815,microtubule organizing center|GO:0005654,nucleoplasm|GO:0044430,cytoskeletal part|GO:0048471,perinuclear region of cytoplasm|GO:0009986,cell surface|GO:0005737,cytoplasm|GO:0015630,microtubule cytoskeleton|GO:0030027,lamellipodium|GO:0032153,cell division site|GO:0032155,cell division site part|GO:0032154,cleavage furrow|GO:0031252,cell leading edge|GO:0005911,cell-cell junction|GO:0005886,plasma membrane|GO:0042995,cell projection|GO:0043234,protein complex|GO:0032991,macromolecular complex|GO:0043231,intracellular membrane-bounded organelle|GO:0043232,intracellular non-membrane-bounded organelle|GO:0043233,organelle lumen|GO:0005829,cytosol|GO:0030496,midbody|GO:0005623,cell|GO:0005622,intracellular|GO:0044446,intracellular organelle part|GO:0070013,intracellular organelle lumen|GO:0044444,cytoplasmic part|GO:0071944,cell periphery|GO:0044428,nuclear part|GO:0044424,intracellular part|GO:0043296,apical junction complex|GO:0044422,organelle part|GO:0016604,nuclear body|GO:0044451,nucleoplasm part	GO:0035639,purine ribonucleoside triphosphate binding|GO:0019899,enzyme binding|GO:0004672,protein kinase activity|GO:0003676,nucleic acid binding|GO:0043167,ion binding|GO:0004697,protein kinase C activity|GO:1901363,heterocyclic compound binding|GO:0001883,purine nucleoside binding|GO:0001882,nucleoside binding|GO:0043168,anion binding|GO:0070063,RNA polymerase binding|GO:0000166,nucleotide binding|GO:0004674,protein serine/threonine kinase activity|GO:0016740,transferase activity|GO:1901265,nucleoside phosphate binding|GO:0032549,ribonucleoside binding|GO:0017076,purine nucleotide binding|GO:0042826,histone deacetylase binding|GO:0005524,ATP binding|GO:0044822,poly(A) RNA binding|GO:0016301,kinase activity|GO:0036094,small molecule binding|GO:0003824,catalytic activity|GO:0030554,adenyl nucleotide binding|GO:0016773,phosphotransferase activity, alcohol group as acceptor|GO:0016772,transferase activity, transferring phosphorus-containing groups|GO:0097367,carbohydrate derivative binding|GO:0032559,adenyl ribonucleotide binding|GO:0003723,RNA binding|GO:0005515,protein binding|GO:0097159,organic cyclic compound binding|GO:0032555,purine ribonucleotide binding|GO:0032550,purine ribonucleoside binding|GO:0032553,ribonucleotide binding|GO:0005488,binding|GO:0050839,cell adhesion molecule binding|GO:0045296,cadherin binding|GO:0031267,small GTPase binding|GO:0051020,GTPase binding|GO:0017016,Ras GTPase binding|GO:0017048,Rho GTPase binding|GO:0017049,GTP-Rho binding
HSBP1	GO:0080090,regulation of primary metabolic process|GO:0019222,regulation of metabolic process|GO:0034605,cellular response to heat|GO:0048583,regulation of response to stimulus|GO:0003012,muscle system process|GO:1901362,organic cyclic compound biosynthetic process|GO:1901360,organic cyclic compound metabolic process|GO:0051716,cellular response to stimulus|GO:0010605,negative regulation of macromolecule metabolic process|GO:0048869,cellular developmental process|GO:0007369,gastrulation|GO:0048519,negative regulation of biological process|GO:0003008,system process|GO:0006936,muscle contraction|GO:0060255,regulation of macromolecule metabolic process|GO:2001141,regulation of RNA biosynthetic process|GO:0046483,heterocycle metabolic process|GO:0044707,single-multicellular organism process|GO:0044249,cellular biosynthetic process|GO:0033554,cellular response to stress|GO:0019438,aromatic compound biosynthetic process|GO:0009892,negative regulation of metabolic process|GO:0009890,negative regulation of biosynthetic process|GO:0010629,negative regulation of gene expression|GO:0006807,nitrogen compound metabolic process|GO:0050789,regulation of biological process|GO:0097659,nucleic acid-templated transcription|GO:1901576,organic substance biosynthetic process|GO:0044260,cellular macromolecule metabolic process|GO:0065007,biological regulation|GO:0006366,transcription from RNA polymerase II promoter|GO:0048646,anatomical structure formation involved in morphogenesis|GO:0018130,heterocycle biosynthetic process|GO:0009889,regulation of biosynthetic process|GO:0009888,tissue development|GO:0050794,regulation of cellular process|GO:0006950,response to stress|GO:0008152,metabolic process|GO:0009266,response to temperature stimulus|GO:0034654,nucleobase-containing compound biosynthetic process|GO:0035987,endodermal cell differentiation|GO:0016070,RNA metabolic process|GO:0044767,single-organism developmental process|GO:0044271,cellular nitrogen compound biosynthetic process|GO:0080134,regulation of response to stress|GO:0080135,regulation of cellular response to stress|GO:0006355,regulation of transcription, DNA-templated|GO:0010556,regulation of macromolecule biosynthetic process|GO:0006351,transcription, DNA-templated|GO:0010558,negative regulation of macromolecule biosynthetic process|GO:0007492,endoderm development|GO:0032774,RNA biosynthetic process|GO:0030154,cell differentiation|GO:0009790,embryo development|GO:0034641,cellular nitrogen compound metabolic process|GO:0034645,cellular macromolecule biosynthetic process|GO:0009653,anatomical structure morphogenesis|GO:0044699,single-organism process|GO:0006139,nucleobase-containing compound metabolic process|GO:0001706,endoderm formation|GO:0001704,formation of primary germ layer|GO:0000122,negative regulation of transcription from RNA polymerase II promoter|GO:0032502,developmental process|GO:0032501,multicellular organismal process|GO:0009628,response to abiotic stimulus|GO:0009987,cellular process|GO:0006725,cellular aromatic compound metabolic process|GO:1903506,regulation of nucleic acid-templated transcription|GO:1903507,negative regulation of nucleic acid-templated transcription|GO:0045892,negative regulation of transcription, DNA-templated|GO:0009408,response to heat|GO:1900034,regulation of cellular response to heat|GO:0051253,negative regulation of RNA metabolic process|GO:0051252,regulation of RNA metabolic process|GO:0043170,macromolecule metabolic process|GO:0050896,response to stimulus|GO:0031327,negative regulation of cellular biosynthetic process|GO:0031326,regulation of cellular biosynthetic process|GO:0031324,negative regulation of cellular metabolic process|GO:0031323,regulation of cellular metabolic process|GO:0090304,nucleic acid metabolic process|GO:0007275,multicellular organism development|GO:2000112,regulation of cellular macromolecule biosynthetic process|GO:2000113,negative regulation of cellular macromolecule biosynthetic process|GO:0071704,organic substance metabolic process|GO:0010467,gene expression|GO:0006357,regulation of transcription from RNA polymerase II promoter|GO:0010468,regulation of gene expression|GO:0048598,embryonic morphogenesis|GO:0045934,negative regulation of nucleobase-containing compound metabolic process|GO:0019219,regulation of nucleobase-containing compound metabolic process|GO:1902679,negative regulation of RNA biosynthetic process|GO:0009058,biosynthetic process|GO:0009059,macromolecule biosynthetic process|GO:0044763,single-organism cellular process|GO:0051171,regulation of nitrogen compound metabolic process|GO:0051172,negative regulation of nitrogen compound metabolic process|GO:0044238,primary metabolic process|GO:0048856,anatomical structure development|GO:0044237,cellular metabolic process|GO:0048523,negative regulation of cellular process	GO:0005856,cytoskeleton|GO:0044446,intracellular organelle part|GO:0043231,intracellular membrane-bounded organelle|GO:0031981,nuclear lumen|GO:0043233,organelle lumen|GO:0005634,nucleus|GO:0044464,cell part|GO:0043232,intracellular non-membrane-bounded organelle|GO:0031974,membrane-enclosed lumen|GO:0005622,intracellular|GO:0005654,nucleoplasm|GO:0070013,intracellular organelle lumen|GO:0043229,intracellular organelle|GO:0043228,non-membrane-bounded organelle|GO:0044428,nuclear part|GO:0005623,cell|GO:0044424,intracellular part|GO:0043227,membrane-bounded organelle|GO:0043226,organelle|GO:0044422,organelle part	GO:0005515,protein binding|GO:0003714,transcription corepressor activity|GO:0003712,transcription cofactor activity|GO:0005488,binding|GO:0000989,transcription factor activity, transcription factor binding|GO:0000988,transcription factor activity, protein binding|GO:0042802,identical protein binding
TLCD1		GO:0016021,integral component of membrane|GO:0016020,membrane|GO:0044464,cell part|GO:0005623,cell|GO:0071944,cell periphery|GO:0005886,plasma membrane|GO:0044425,membrane part|GO:0031224,intrinsic component of membrane	
ZFPM2	GO:0007599,hemostasis|GO:0080090,regulation of primary metabolic process|GO:0019222,regulation of metabolic process|GO:2000242,negative regulation of reproductive process|GO:2000243,positive regulation of reproductive process|GO:2000241,regulation of reproductive process|GO:0008585,female gonad development|GO:0008584,male gonad development|GO:0072359,circulatory system development|GO:0072358,cardiovascular system development|GO:1901362,organic cyclic compound biosynthetic process|GO:1901360,organic cyclic compound metabolic process|GO:0003151,outflow tract morphogenesis|GO:0007517,muscle organ development|GO:0010605,negative regulation of macromolecule metabolic process|GO:0010604,positive regulation of macromolecule metabolic process|GO:0048869,cellular developmental process|GO:0009611,response to wounding|GO:0045137,development of primary sexual characteristics|GO:0048513,animal organ development|GO:0048514,blood vessel morphogenesis|GO:0048518,positive regulation of biological process|GO:0048519,negative regulation of biological process|GO:0060541,respiratory system development|GO:0007596,blood coagulation|GO:0060255,regulation of macromolecule metabolic process|GO:0060548,negative regulation of cell death|GO:0003007,heart morphogenesis|GO:0003006,developmental process involved in reproduction|GO:0044702,single organism reproductive process|GO:2001141,regulation of RNA biosynthetic process|GO:0046483,heterocycle metabolic process|GO:0003215,cardiac right ventricle morphogenesis|GO:0044707,single-multicellular organism process|GO:0044249,cellular biosynthetic process|GO:0060412,ventricular septum morphogenesis|GO:0060411,cardiac septum morphogenesis|GO:0060415,muscle tissue morphogenesis|GO:0009892,negative regulation of metabolic process|GO:0009893,positive regulation of metabolic process|GO:0009890,negative regulation of biosynthetic process|GO:0009891,positive regulation of biosynthetic process|GO:0048568,embryonic organ development|GO:0003281,ventricular septum development|GO:0008152,metabolic process|GO:0010629,negative regulation of gene expression|GO:0010628,positive regulation of gene expression|GO:0050789,regulation of biological process|GO:0097659,nucleic acid-templated transcription|GO:0003205,cardiac chamber development|GO:0048738,cardiac muscle tissue development|GO:2000018,regulation of male gonad development|GO:0044260,cellular macromolecule metabolic process|GO:0001568,blood vessel development|GO:0008406,gonad development|GO:0003208,cardiac ventricle morphogenesis|GO:0065007,biological regulation|GO:0044699,single-organism process|GO:0006366,transcription from RNA polymerase II promoter|GO:0065008,regulation of biological quality|GO:0048644,muscle organ morphogenesis|GO:0018130,heterocycle biosynthetic process|GO:0016265,obsolete death|GO:0009887,animal organ morphogenesis|GO:0046545,development of primary female sexual characteristics|GO:0046546,development of primary male sexual characteristics|GO:0050793,regulation of developmental process|GO:0009889,regulation of biosynthetic process|GO:0009888,tissue development|GO:0042060,wound healing|GO:0050794,regulation of cellular process|GO:0006950,response to stress|GO:0050817,coagulation|GO:0051239,regulation of multicellular organismal process|GO:0019438,aromatic compound biosynthetic process|GO:0034654,nucleobase-containing compound biosynthetic process|GO:0003231,cardiac ventricle development|GO:0046660,female sex differentiation|GO:0046661,male sex differentiation|GO:0016070,RNA metabolic process|GO:0001570,vasculogenesis|GO:0007548,sex differentiation|GO:0044271,cellular nitrogen compound biosynthetic process|GO:0050896,response to stimulus|GO:0006355,regulation of transcription, DNA-templated|GO:0010557,positive regulation of macromolecule biosynthetic process|GO:0010556,regulation of macromolecule biosynthetic process|GO:0044767,single-organism developmental process|GO:0006351,transcription, DNA-templated|GO:0010558,negative regulation of macromolecule biosynthetic process|GO:0032774,RNA biosynthetic process|GO:0007498,mesoderm development|GO:0030154,cell differentiation|GO:0009790,embryo development|GO:0034641,cellular nitrogen compound metabolic process|GO:0009792,embryo development ending in birth or egg hatching|GO:0003229,ventricular cardiac muscle tissue development|GO:0061061,muscle structure development|GO:0009653,anatomical structure morphogenesis|GO:0003221,right ventricular cardiac muscle tissue morphogenesis|GO:0006139,nucleobase-containing compound metabolic process|GO:0001701,in utero embryonic development|GO:0000122,negative regulation of transcription from RNA polymerase II promoter|GO:0051240,positive regulation of multicellular organismal process|GO:0051241,negative regulation of multicellular organismal process|GO:0001944,vasculature development|GO:0061458,reproductive system development|GO:0043009,chordate embryonic development|GO:1903508,positive regulation of nucleic acid-templated transcription|GO:2000194,regulation of female gonad development|GO:2000195,negative regulation of female gonad development|GO:0048608,reproductive structure development|GO:0050878,regulation of body fluid levels|GO:0044238,primary metabolic process|GO:0055008,cardiac muscle tissue morphogenesis|GO:0034645,cellular macromolecule biosynthetic process|GO:0009987,cellular process|GO:0006725,cellular aromatic compound metabolic process|GO:1903506,regulation of nucleic acid-templated transcription|GO:1903507,negative regulation of nucleic acid-templated transcription|GO:0045596,negative regulation of cell differentiation|GO:0045595,regulation of cell differentiation|GO:0045892,negative regulation of transcription, DNA-templated|GO:0045893,positive regulation of transcription, DNA-templated|GO:0045599,negative regulation of fat cell differentiation|GO:0045598,regulation of fat cell differentiation|GO:0051093,negative regulation of developmental process|GO:0051094,positive regulation of developmental process|GO:0051253,negative regulation of RNA metabolic process|GO:0051252,regulation of RNA metabolic process|GO:0051254,positive regulation of RNA metabolic process|GO:0009059,macromolecule biosynthetic process|GO:0043170,macromolecule metabolic process|GO:1902680,positive regulation of RNA biosynthetic process|GO:0006807,nitrogen compound metabolic process|GO:0045944,positive regulation of transcription from RNA polymerase II promoter|GO:0048731,system development|GO:0032502,developmental process|GO:0030323,respiratory tube development|GO:0030324,lung development|GO:0031328,positive regulation of cellular biosynthetic process|GO:0031327,negative regulation of cellular biosynthetic process|GO:0031326,regulation of cellular biosynthetic process|GO:0031325,positive regulation of cellular metabolic process|GO:0031324,negative regulation of cellular metabolic process|GO:0031323,regulation of cellular metabolic process|GO:0090304,nucleic acid metabolic process|GO:0014706,striated muscle tissue development|GO:0032501,multicellular organismal process|GO:0045444,fat cell differentiation|GO:0008219,cell death|GO:0010941,regulation of cell death|GO:0007275,multicellular organism development|GO:2000112,regulation of cellular macromolecule biosynthetic process|GO:2000113,negative regulation of cellular macromolecule biosynthetic process|GO:0071704,organic substance metabolic process|GO:0010467,gene expression|GO:0006357,regulation of transcription from RNA polymerase II promoter|GO:0048729,tissue morphogenesis|GO:2000026,regulation of multicellular organismal development|GO:0010468,regulation of gene expression|GO:0045935,positive regulation of nucleobase-containing compound metabolic process|GO:0045934,negative regulation of nucleobase-containing compound metabolic process|GO:1901576,organic substance biosynthetic process|GO:0019219,regulation of nucleobase-containing compound metabolic process|GO:1902679,negative regulation of RNA biosynthetic process|GO:0022414,reproductive process|GO:0009058,biosynthetic process|GO:0003206,cardiac chamber morphogenesis|GO:0044763,single-organism cellular process|GO:0051171,regulation of nitrogen compound metabolic process|GO:0051172,negative regulation of nitrogen compound metabolic process|GO:0051173,positive regulation of nitrogen compound metabolic process|GO:0060485,mesenchyme development|GO:0035295,tube development|GO:0007506,gonadal mesoderm development|GO:0007507,heart development|GO:0003279,cardiac septum development|GO:0003148,outflow tract septum morphogenesis|GO:0048856,anatomical structure	GO:0005737,cytoplasm|GO:0044446,intracellular organelle part|GO:0043231,intracellular membrane-bounded organelle|GO:0031981,nuclear lumen|GO:0043233,organelle lumen|GO:0005634,nucleus|GO:0044464,cell part|GO:0031974,membrane-enclosed lumen|GO:0005622,intracellular|GO:0005654,nucleoplasm|GO:0070013,intracellular organelle lumen|GO:0043229,intracellular organelle|GO:0044428,nuclear part|GO:0005623,cell|GO:0044424,intracellular part|GO:0043227,membrane-bounded organelle|GO:0043226,organelle|GO:0044422,organelle part	GO:0001076,transcription factor activity, RNA polymerase II transcription factor binding|GO:0008270,zinc ion binding|GO:0003676,nucleic acid binding|GO:0003677,DNA binding|GO:0001078,transcriptional repressor activity, RNA polymerase II core promoter proximal region sequence-specific binding|GO:1901363,heterocyclic compound binding|GO:0043169,cation binding|GO:0001190,transcriptional activator activity, RNA polymerase II transcription factor binding|GO:0046914,transition metal ion binding|GO:0003714,transcription corepressor activity|GO:0003713,transcription coactivator activity|GO:0003712,transcription cofactor activity|GO:0043167,ion binding|GO:0000989,transcription factor activity, transcription factor binding|GO:0000988,transcription factor activity, protein binding|GO:0046872,metal ion binding|GO:0000982,transcription factor activity, RNA polymerase II core promoter proximal region sequence-specific binding|GO:0000981,RNA polymerase II transcription factor activity, sequence-specific DNA binding|GO:0008134,transcription factor binding|GO:0001104,RNA polymerase II transcription cofactor activity|GO:0001105,RNA polymerase II transcription coactivator activity|GO:0001085,RNA polymerase II transcription factor binding|GO:0001227,transcriptional repressor activity, RNA polymerase II transcription regulatory region sequence-specific binding|GO:0003700,transcription factor activity, sequence-specific DNA binding|GO:0097159,organic cyclic compound binding|GO:0001071,nucleic acid binding transcription factor activity|GO:0005515,protein binding|GO:0005488,binding|GO:0001191,transcriptional repressor activity, RNA polymerase II transcription factor binding|GO:0098811,transcriptional repressor activity, RNA polymerase II activating transcription factor binding

TVP23B	GO:0033036,macromolecule localization|GO:0046903,secretion|GO:0006810,transport|GO:0016192,vesicle-mediated transport|GO:0051649,establishment of localization in cell|GO:0009306,protein secretion|GO:0009987,cellular process|GO:0044765,single-organism transport|GO:0045184,establishment of protein localization|GO:0032940,secretion by cell|GO:0008104,protein localization|GO:0044763,single-organism cellular process|GO:0044699,single-organism process|GO:0071702,organic substance transport|GO:0015031,protein transport|GO:0051234,establishment of localization|GO:0051179,localization|GO:1902578,single-organism localization|GO:0051641,cellular localization|GO:0015833,peptide transport|GO:0042886,amide transport|GO:0071705,nitrogen compound transport|GO:0002790,peptide secretion	GO:0043229,intracellular organelle|GO:0031301,integral component of organelle membrane|GO:0031300,intrinsic component of organelle membrane|GO:0030173,integral component of Golgi membrane|GO:0005622,intracellular|GO:0043227,membrane-bounded organelle|GO:0043226,organelle|GO:0031224,intrinsic component of membrane|GO:0005737,cytoplasm|GO:0031090,organelle membrane|GO:0016021,integral component of membrane|GO:0016020,membrane|GO:0044431,Golgi apparatus part|GO:0005794,Golgi apparatus|GO:0098588,bounding membrane of organelle|GO:0012505,endomembrane system|GO:0043231,intracellular membrane-bounded organelle|GO:0044464,cell part|GO:0031228,intrinsic component of Golgi membrane|GO:0005623,cell|GO:0000139,Golgi membrane|GO:0044446,intracellular organelle part|GO:0044444,cytoplasmic part|GO:0044424,intracellular part|GO:0044425,membrane part|GO:0044422,organelle part|GO:0031984,organelle subcompartment|GO:0098791,Golgi subcompartment	
CNEP1R1	GO:0006997,nucleus organization|GO:0019220,regulation of phosphate metabolic process|GO:0080090,regulation of primary metabolic process|GO:0019222,regulation of metabolic process|GO:0006470,protein dephosphorylation|GO:0061024,membrane organization|GO:0008104,protein localization|GO:0071840,cellular component organization or biogenesis|GO:0044710,single-organism metabolic process|GO:0044711,single-organism biosynthetic process|GO:0010604,positive regulation of macromolecule metabolic process|GO:0070727,cellular macromolecule localization|GO:0010256,endomembrane system organization|GO:0045834,positive regulation of lipid metabolic process|GO:0048518,positive regulation of biological process|GO:0033036,macromolecule localization|GO:0006639,acylglycerol metabolic process|GO:0060255,regulation of macromolecule metabolic process|GO:0007077,mitotic nuclear envelope disassembly|GO:0046486,glycerolipid metabolic process|GO:0019538,protein metabolic process|GO:0019432,triglyceride biosynthetic process|GO:0009893,positive regulation of metabolic process|GO:0009891,positive regulation of biosynthetic process|GO:0050789,regulation of biological process|GO:0010867,positive regulation of triglyceride biosynthetic process|GO:0010866,regulation of triglyceride biosynthetic process|GO:1901576,organic substance biosynthetic process|GO:0044260,cellular macromolecule metabolic process|GO:0016043,cellular component organization|GO:0065007,biological regulation|GO:0007049,cell cycle|GO:0044267,cellular protein metabolic process|GO:0006629,lipid metabolic process|GO:0009889,regulation of biosynthetic process|GO:0050794,regulation of cellular process|GO:0043412,macromolecule modification|GO:0036211,protein modification process|GO:0044802,single-organism membrane organization|GO:0008152,metabolic process|GO:0035303,regulation of dephosphorylation|GO:0035304,regulation of protein dephosphorylation|GO:0035307,positive regulation of protein dephosphorylation|GO:0035306,positive regulation of dephosphorylation|GO:0031401,positive regulation of protein modification process|GO:0022411,cellular component disassembly|GO:0090208,positive regulation of triglyceride metabolic process|GO:0016311,dephosphorylation|GO:0090207,regulation of triglyceride metabolic process|GO:0006638,neutral lipid metabolic process|GO:0051247,positive regulation of protein metabolic process|GO:0044249,cellular biosynthetic process|GO:0044699,single-organism process|GO:0010562,positive regulation of phosphorus metabolic process|GO:0051246,regulation of protein metabolic process|GO:0051081,nuclear envelope disassembly|GO:0032270,positive regulation of cellular protein metabolic process|GO:0031399,regulation of protein modification process|GO:0051641,cellular localization|GO:0046463,acylglycerol biosynthetic process|GO:0046460,neutral lipid biosynthetic process|GO:0006641,triglyceride metabolic process|GO:0009987,cellular process|GO:0046889,positive regulation of lipid biosynthetic process|GO:0044255,cellular lipid metabolic process|GO:0030397,membrane disassembly|GO:0032268,regulation of cellular protein metabolic process|GO:0043170,macromolecule metabolic process|GO:0033365,protein localization to organelle|GO:0034504,protein localization to nucleus|GO:0031328,positive regulation of cellular biosynthetic process|GO:0031326,regulation of cellular biosynthetic process|GO:0031325,positive regulation of cellular metabolic process|GO:0031323,regulation of cellular metabolic process|GO:1903047,mitotic cell cycle process|GO:0022402,cell cycle process|GO:0006998,nuclear envelope organization|GO:0046890,regulation of lipid biosynthetic process|GO:0045017,glycerolipid biosynthetic process|GO:0071704,organic substance metabolic process|GO:0000278,mitotic cell cycle|GO:0045937,positive regulation of phosphate metabolic process|GO:0019216,regulation of lipid metabolic process|GO:0034613,cellular protein localization|GO:0006464,cellular protein modification process|GO:0051174,regulation of phosphorus metabolic process|GO:0009058,biosynthetic process|GO:0044763,single-organism cellular process|GO:0051179,localization|GO:0008610,lipid biosynthetic process|GO:0006996,organelle organization|GO:0044238,primary metabolic process|GO:0044237,cellular metabolic process|GO:0006796,phosphate-containing compound metabolic process|GO:0006793,phosphorus metabolic process|GO:0048522,positive regulation of cellular process|GO:1901564,organonitrogen compound metabolic process|GO:0006807,nitrogen compound metabolic process	GO:0005635,nuclear envelope|GO:1903293,phosphatase complex|GO:0031975,envelope|GO:0042175,nuclear outer membrane-endoplasmic reticulum membrane network|GO:0043229,intracellular organelle|GO:0043227,membrane-bounded organelle|GO:0043226,organelle|GO:0031224,intrinsic component of membrane|GO:0005737,cytoplasm|GO:0031090,organelle membrane|GO:0016021,integral component of membrane|GO:0016020,membrane|GO:0071595,Nem1-Spo7 phosphatase complex|GO:0008287,protein serine/threonine phosphatase complex|GO:1902494,catalytic complex|GO:0031965,nuclear membrane|GO:0031967,organelle envelope|GO:0012505,endomembrane system|GO:0043234,protein complex|GO:0032991,macromolecular complex|GO:0043231,intracellular membrane-bounded organelle|GO:0044464,cell part|GO:0005623,cell|GO:0005622,intracellular|GO:0044446,intracellular organelle part|GO:0098796,membrane protein complex|GO:0044428,nuclear part|GO:0044424,intracellular part|GO:0044425,membrane part|GO:0005634,nucleus|GO:0044422,organelle part|GO:0005829,cytosol|GO:0044444,cytoplasmic part	GO:0005488,binding|GO:0005515,protein binding
S100A7A		GO:0005737,cytoplasm|GO:0044464,cell part|GO:0005623,cell|GO:0005622,intracellular|GO:0044424,intracellular part	GO:0043169,cation binding|GO:0043167,ion binding|GO:0005509,calcium ion binding|GO:0005515,protein binding|GO:0046872,metal ion binding|GO:0043621,protein self-association|GO:0005488,binding|GO:0046914,transition metal ion binding
RGS4	GO:0043549,regulation of kinase activity|GO:0019220,regulation of phosphate metabolic process|GO:0080090,regulation of primary metabolic process|GO:0019222,regulation of metabolic process|GO:0048585,negative regulation of response to stimulus|GO:0048583,regulation of response to stimulus|GO:0008277,regulation of G-protein coupled receptor protein signaling pathway|GO:0007165,signal transduction|GO:0023014,signal transduction by protein phosphorylation|GO:0044710,single-organism metabolic process|GO:0010605,negative regulation of macromolecule metabolic process|GO:0009968,negative regulation of signal transduction|GO:0009966,regulation of signal transduction|GO:0000165,MAPK cascade|GO:0044093,positive regulation of molecular function|GO:0044092,negative regulation of molecular function|GO:0048518,positive regulation of biological process|GO:0048519,negative regulation of biological process|GO:0060255,regulation of macromolecule metabolic process|GO:0045859,regulation of protein kinase activity|GO:0042325,regulation of phosphorylation|GO:0044700,single organism signaling|GO:0042326,negative regulation of phosphorylation|GO:0019538,protein metabolic process|GO:0033673,negative regulation of kinase activity|GO:0009892,negative regulation of metabolic process|GO:0009893,positive regulation of metabolic process|GO:0035556,intracellular signal transduction|GO:0071900,regulation of protein serine/threonine kinase activity|GO:0050789,regulation of biological process|GO:0044267,cellular protein metabolic process|GO:0043547,positive regulation of GTPase activity|GO:0044260,cellular macromolecule metabolic process|GO:0051348,negative regulation of transferase activity|GO:0065007,biological regulation|GO:0043409,negative regulation of MAPK cascade|GO:0065009,regulation of molecular function|GO:0007186,G-protein coupled receptor signaling pathway|GO:0043085,positive regulation of catalytic activity|GO:0050790,regulation of catalytic activity|GO:0051716,cellular response to stimulus|GO:0050794,regulation of cellular process|GO:0023021,termination of signal transduction|GO:0036211,protein modification process|GO:0008152,metabolic process|GO:1902532,negative regulation of intracellular signal transduction|GO:1902531,regulation of intracellular signal transduction|GO:0051336,regulation of hydrolase activity|GO:0031400,negative regulation of protein modification process|GO:0043412,macromolecule modification|GO:0051338,regulation of transferase activity|GO:0016310,phosphorylation|GO:0043407,negative regulation of MAP kinase activity|GO:0023057,negative regulation of signaling|GO:0043405,regulation of MAP kinase activity|GO:0023052,signaling|GO:0010648,negative regulation of cell communication|GO:0023051,regulation of signaling|GO:0010646,regulation of cell communication|GO:0043087,regulation of GTPase activity|GO:0043086,negative regulation of catalytic activity|GO:0044699,single-organism process|GO:0043408,regulation of MAPK cascade|GO:0051248,negative regulation of protein metabolic process|GO:0010563,negative regulation of phosphorus metabolic process|GO:0051246,regulation of protein metabolic process|GO:0045744,negative regulation of G-protein coupled receptor protein signaling pathway|GO:0031399,regulation of protein modification process|GO:0038032,termination of G-protein coupled receptor signaling pathway|GO:0009987,cellular process|GO:0032269,negative regulation of cellular protein metabolic process|GO:0032268,regulation of cellular protein metabolic process|GO:0043170,macromolecule metabolic process|GO:0050896,response to stimulus|GO:0031324,negative regulation of cellular metabolic process|GO:0031323,regulation of cellular metabolic process|GO:0000188,inactivation of MAPK activity|GO:0071901,negative regulation of protein serine/threonine kinase activity|GO:0071704,organic substance metabolic process|GO:0006468,protein phosphorylation|GO:0006469,negative regulation of protein kinase activity|GO:0045936,negative regulation of phosphate metabolic process|GO:0006464,cellular protein modification process|GO:0051174,regulation of phosphorus metabolic process|GO:0044763,single-organism cellular process|GO:0007154,cell communication|GO:0044238,primary metabolic process|GO:0051345,positive regulation of hydrolase activity|GO:0044237,cellular metabolic process|GO:0006796,phosphate-containing compound metabolic process|GO:0006793,phosphorus metabolic process|GO:0001933,negative regulation of protein phosphorylation|GO:0001932,regulation of protein phosphorylation|GO:0048523,negative regulation of cellular process|GO:1901564,organonitrogen compound metabolic process|GO:0006807,nitrogen compound metabolic process|GO:0048589,developmental growth|GO:0048588,developmental cell growth|GO:0051049,regulation of transport|GO:0048584,positive regulation of response to stimulus|GO:0048468,cell development|GO:0072359,circulatory system development|GO:0060322,head development|GO:0003015,heart process|GO:0007166,cell surface receptor signaling pathway|GO:0035265,organ growth|GO:0034765,regulation of ion transmembrane transport|GO:0034766,negative regulation of ion transmembrane transport|GO:1905114,cell surface receptor signaling pathway involved in cell-cell signaling|GO:0034762,regulation of transmembrane transport|GO:0060159,regulation of dopamine receptor signaling pathway|GO:0007517,muscle organ development|GO:0007212,dopamine receptor signaling pathway|GO:0009967,positive regulation of signal transduction|GO:0048513,animal organ development|GO:0042220,response to cocaine|GO:0010721,negative regulation of cell development|GO:0051952,regulation of amine transport|GO:0051953,negative regulation of amine transport|GO:0051956,negative regulation of amino acid transport|GO:0051955,regulation of amino acid transport|GO:0016202,regulation of striated muscle tissue development|GO:0006865,amino acid transport|GO:2000725,regulation of cardiac muscle cell differentiation|GO:1905207,regulation of cardiocyte differentiation|GO:2000726,negative regulation of cardiac muscle cell differentiation|GO:1905208,negative regulation of cardiocyte differentiation|GO:0071702,organic substance transport|GO:0015711,organic anion transport|GO:0048634,regulation of muscle organ development|GO:0098662,inorganic cation transmembrane transport|GO:1901379,regulation of potassium ion transmembrane transport|GO:0040007,growth|GO:0051128,regulation of cellular component organization|GO:0010033,response to organic substance|GO:0043271,negative regulation of ion transport|GO:0003008,system process|GO:0065008,regulation of biological quality|GO:0044707,single-multicellular organism process|GO:0010243,response to organonitrogen compound|GO:0008015,blood circulation|GO:0003012,muscle system process|GO:0060419,heart growth|GO:0098916,anterograde trans-synaptic signaling|GO:0045823,positive regulation of heart contraction|GO:0042692,muscle cell differentiation|GO:0003013,circulatory system process|GO:0014741,negative regulation of muscle hypertrophy|GO:0045843,negative regulation of striated muscle tissue development|GO:0014743,regulation of muscle hypertrophy|GO:0098815,modulation of excitatory postsynaptic potential|GO:0045471,response to ethanol|GO:0016049,cell growth|GO:0016043,cellular component organization|GO:0098655,cation transmembrane transport|GO:0055026,negative regulation of cardiac muscle tissue development|GO:0071840,cellular component organization or biogenesis|GO:0098660,inorganic ion transmembrane transport|GO:0048640,negative regulation of developmental growth|GO:0034763,negative regulation of transmembrane transport|GO:0006811,ion transport|GO:1901861,regulation of muscle tissue development|GO:2000463,positive regulation of excitatory postsynaptic potential|GO:1901862,negative regulation of muscle tissue development|GO:0050793,regulation of developmental process|GO:0006812,cation transport|GO:0055024,regulation of cardiac muscle tissue development|GO:0008016,regulation of heart contraction|GO:0055022,negative regulation of cardiac muscle tissue growth|GO:0009888,tissue development|GO:0060160,negative regulation of dopamine receptor signaling pathway|GO:1903524,positive regulation of blood circulation|GO:0071804,cellular potassium ion transport|GO:1903522,regulation of blood circulation|GO:0051239,regulation of multicellular organismal process|GO:0060079,excitatory postsynaptic potential|GO:0032891,negative regulation of organic acid transport|GO:0055021,regulation of cardiac muscle tissue growth|GO:0006813,potassium ion transport|GO:0044767,single-organism developmental process|GO:0043090,amino acid import|GO:0007420,brain development|GO:0043092,L-amino	GO:0043234,protein complex|GO:0005737,cytoplasm|GO:0005829,cytosol|GO:0032991,macromolecular complex|GO:0043231,intracellular membrane-bounded organelle|GO:0005634,nucleus|GO:0016020,membrane|GO:0044464,cell part|GO:0044444,cytoplasmic part|GO:0005623,cell|GO:0005622,intracellular|GO:0043229,intracellular organelle|GO:0071944,cell periphery|GO:0005886,plasma membrane|GO:0044424,intracellular part|GO:0043227,membrane-bounded organelle|GO:0043226,organelle	GO:0005488,binding|GO:0030234,enzyme regulator activity|GO:0005096,GTPase activator activity|GO:0030695,GTPase regulator activity|GO:0001965,G-protein alpha-subunit binding|GO:0008047,enzyme activator activity|GO:0005515,protein binding|GO:0098772,molecular function regulator|GO:0060589,nucleoside-triphosphatase regulator activity|GO:0005516,calmodulin binding
PARP11	GO:0008152,metabolic process		GO:0003824,catalytic activity|GO:0016740,transferase activity|GO:0016757,transferase activity, transferring glycosyl groups|GO:0016763,transferase activity, transferring pentosyl groups|GO:0003950,NAD+ ADP-ribosyltransferase activity
PDX1	GO:0035774,positive regulation of insulin secretion involved in cellular response to glucose stimulus|GO:0032024,positive regulation of insulin secretion|GO:0009746,response to hexose|GO:0044238,primary metabolic process|GO:0051234,establishment of localization|GO:0080090,regulation of primary metabolic process|GO:0019222,regulation of metabolic process|GO:2001233,regulation of apoptotic signaling pathway|GO:0051049,regulation of transport|GO:0048585,negative regulation of response to stimulus|GO:0048584,positive regulation of response to stimulus|GO:0048583,regulation of response to stimulus|GO:0032846,positive regulation of homeostatic process|GO:0032844,regulation of homeostatic process|GO:0008104,protein localization|GO:0007165,signal transduction|GO:0007166,cell surface receptor signaling pathway|GO:0032940,secretion by cell|GO:0009593,detection of chemical stimulus|GO:1903573,negative regulation of response to endoplasmic reticulum stress|GO:0010040,response to iron(II) ion|GO:1901362,organic cyclic compound biosynthetic process|GO:1901360,organic cyclic compound metabolic process|GO:0032774,RNA biosynthetic process|GO:0043201,response to leucine|GO:0043200,response to amino acid|GO:0044710,single-organism metabolic process|GO:0048863,stem cell differentiation|GO:0010605,negative regulation of macromolecule metabolic process|GO:0010604,positive regulation of macromolecule metabolic process|GO:0009968,negative regulation of signal transduction|GO:0009966,regulation of signal transduction|GO:0048869,cellular developmental process|GO:0035773,insulin secretion involved in cellular response to glucose stimulus|GO:0071310,cellular response to organic substance|GO:0046879,hormone secretion|GO:0043066,negative regulation of apoptotic process|GO:0071331,cellular response to hexose stimulus|GO:0044093,positive regulation of molecular function|GO:0030855,epithelial cell differentiation|GO:0048518,positive regulation of biological process|GO:0048519,negative regulation of biological process|GO:0033036,macromolecule localization|GO:0002067,glandular epithelial cell differentiation|GO:0002065,columnar/cuboidal epithelial cell differentiation|GO:0042127,regulation of cell proliferation|GO:0030154,cell differentiation|GO:0035094,response to nicotine|GO:0009749,response to glucose|GO:0051050,positive regulation of transport|GO:0060255,regulation of macromolecule metabolic process|GO:0060548,negative regulation of cell death|GO:0006366,transcription from RNA polymerase II promoter|GO:0034645,cellular macromolecule biosynthetic process|GO:0035270,endocrine system development|GO:0090276,regulation of peptide hormone secretion|GO:0090277,positive regulation of peptide hormone secretion|GO:0042221,response to chemical|GO:0051594,detection of glucose|GO:2001141,regulation of RNA biosynthetic process|GO:0034287,detection of monosaccharide stimulus|GO:0009725,response to hormone|GO:0010033,response to organic substance|GO:0046483,heterocycle metabolic process|GO:0044700,single organism signaling|GO:0065008,regulation of biological quality|GO:0051716,cellular response to stimulus|GO:0044707,single-multicellular organism process|GO:2001234,negative regulation of apoptotic signaling pathway|GO:0009790,embryo development|GO:0071322,cellular response to carbohydrate stimulus|GO:0010243,response to organonitrogen compound|GO:0034284,response to monosaccharide|GO:0071326,cellular response to monosaccharide stimulus|GO:0097285,obsolete cell-type specific apoptotic process|GO:0033554,cellular response to stress|GO:0060290,transdifferentiation|GO:0019438,aromatic compound biosynthetic process|GO:0048878,chemical homeostasis|GO:0051099,positive regulation of binding|GO:1903530,regulation of secretion by cell|GO:0001101,response to acid chemical|GO:0051098,regulation of binding|GO:0009892,negative regulation of metabolic process|GO:0009893,positive regulation of metabolic process|GO:0009890,negative regulation of biosynthetic process|GO:0009891,positive regulation of biosynthetic process|GO:1903532,positive regulation of secretion by cell|GO:1902235,regulation of endoplasmic reticulum stress-induced intrinsic apoptotic signaling pathway|GO:0009887,animal organ morphogenesis|GO:1902236,negative regulation of endoplasmic reticulum stress-induced intrinsic apoptotic signaling pathway|GO:0008152,metabolic process|GO:0010629,negative regulation of gene expression|GO:0010628,positive regulation of gene expression|GO:0006807,nitrogen compound metabolic process|GO:0019932,second-messenger-mediated signaling|GO:0043388,positive regulation of DNA binding|GO:0035556,intracellular signal transduction|GO:0051223,regulation of protein transport|GO:0042981,regulation of apoptotic process|GO:0050789,regulation of biological process|GO:0030072,peptide hormone secretion|GO:0030073,insulin secretion|GO:0009605,response to external stimulus|GO:1901576,organic substance biosynthetic process|GO:0090304,nucleic acid metabolic process|GO:0009653,anatomical structure morphogenesis|GO:0050708,regulation of protein secretion|GO:0044260,cellular macromolecule metabolic process|GO:0009987,cellular process|GO:0009306,protein secretion|GO:1904035,regulation of epithelial cell apoptotic process|GO:0014070,response to organic cyclic compound|GO:1904036,negative regulation of epithelial cell apoptotic process|GO:0019725,cellular homeostasis|GO:0065009,regulation of molecular function|GO:0007417,central nervous system development|GO:0055123,digestive system development|GO:0018130,heterocycle biosynthetic process|GO:0016265,obsolete death|GO:0070201,regulation of establishment of protein localization|GO:0034097,response to cytokine|GO:0050714,positive regulation of protein secretion|GO:0019318,hexose metabolic process|GO:0006810,transport|GO:0009889,regulation of biosynthetic process|GO:0050796,regulation of insulin secretion|GO:0050794,regulation of cellular process|GO:0012501,programmed cell death|GO:0006950,response to stress|GO:1901700,response to oxygen-containing compound|GO:0001678,cellular glucose homeostasis|GO:1902532,negative regulation of intracellular signal transduction|GO:0034641,cellular nitrogen compound metabolic process|GO:0061178,regulation of insulin secretion involved in cellular response to glucose stimulus|GO:0048732,gland development|GO:0016070,RNA metabolic process|GO:0044767,single-organism developmental process|GO:0046903,secretion|GO:0010260,animal organ senescence|GO:0044271,cellular nitrogen compound biosynthetic process|GO:0006091,generation of precursor metabolites and energy|GO:0010157,response to chlorate|GO:0070059,intrinsic apoptotic signaling pathway in response to endoplasmic reticulum stress|GO:0080134,regulation of response to stress|GO:0080135,regulation of cellular response to stress|GO:0010038,response to metal ion|GO:0009058,biosynthetic process|GO:0065007,biological regulation|GO:0006355,regulation of transcription, DNA-templated|GO:0010557,positive regulation of macromolecule biosynthetic process|GO:0006357,regulation of transcription from RNA polymerase II promoter|GO:0006351,transcription, DNA-templated|GO:0051046,regulation of secretion|GO:0042886,amide transport|GO:0010558,negative regulation of macromolecule biosynthetic process|GO:0031018,endocrine pancreas development|GO:0031960,response to corticosteroid|GO:0050896,response to stimulus|GO:0031017,exocrine pancreas development|GO:0031016,pancreas development|GO:0033500,carbohydrate homeostasis|GO:0009991,response to extracellular stimulus|GO:0009611,response to wounding|GO:0023056,positive regulation of signaling|GO:0023057,negative regulation of signaling|GO:0015833,peptide transport|GO:0051384,response to glucocorticoid|GO:0023052,signaling|GO:0010648,negative regulation of cell communication|GO:0070887,cellular response to chemical stimulus|GO:0023051,regulation of signaling|GO:0051047,positive regulation of secretion|GO:0048565,digestive tract development|GO:0010647,positive regulation of cell communication|GO:0010646,regulation of cell communication|GO:0044699,single-organism process|GO:0032880,regulation of protein localization|GO:0009719,response to endogenous stimulus|GO:0006139,nucleobase-containing compound metabolic process|GO:1904019,epithelial cell apoptotic process|GO:1902531,regulation of intracellular signal transduction|GO:0003309,type B pancreatic cell differentiation|GO:0051649,establishment of localization in cell|GO:0000122,negative regulation of transcription from RNA polymerase II promoter|GO:0010039,response to iron ion|GO:0061008,hepaticobiliary system development|GO:0031099,regeneration|GO:0048513,animal organ development|GO:0033273,response to vitamin|GO:0010035,response to	GO:0031974,membrane-enclosed lumen|GO:0043229,intracellular organelle|GO:0043227,membrane-bounded organelle|GO:0043226,organelle|GO:0016607,nuclear speck|GO:0016604,nuclear body|GO:0031981,nuclear lumen|GO:0005634,nucleus|GO:0005654,nucleoplasm|GO:0044451,nucleoplasm part|GO:0005737,cytoplasm|GO:0043231,intracellular membrane-bounded organelle|GO:0043233,organelle lumen|GO:0005829,cytosol|GO:0044464,cell part|GO:0005623,cell|GO:0005622,intracellular|GO:0044446,intracellular organelle part|GO:0070013,intracellular organelle lumen|GO:0044444,cytoplasmic part|GO:0044428,nuclear part|GO:0044424,intracellular part|GO:0044422,organelle part	GO:0001012,RNA polymerase II regulatory region DNA binding|GO:0001159,core promoter proximal region DNA binding|GO:0044877,macromolecular complex binding|GO:0005488,binding|GO:0003676,nucleic acid binding|GO:0003677,DNA binding|GO:1901363,heterocyclic compound binding|GO:0043565,sequence-specific DNA binding|GO:0000987,core promoter proximal region sequence-specific DNA binding|GO:0000981,RNA polymerase II transcription factor activity, sequence-specific DNA binding|GO:0008134,transcription factor binding|GO:0001067,regulatory region nucleic acid binding|GO:0032403,protein complex binding|GO:0001047,core promoter binding|GO:0001046,core promoter sequence-specific DNA binding|GO:0000978,RNA polymerase II core promoter proximal region sequence-specific DNA binding|GO:0044212,transcription regulatory region DNA binding|GO:0003682,chromatin binding|GO:0003700,transcription factor activity, sequence-specific DNA binding|GO:0097159,organic cyclic compound binding|GO:0003705,transcription factor activity, RNA polymerase II distal enhancer sequence-specific binding|GO:0000976,transcription regulatory region sequence-specific DNA binding|GO:0000977,RNA polymerase II regulatory region sequence-specific DNA binding|GO:0005515,protein binding|GO:0000975,regulatory region DNA binding|GO:0046983,protein dimerization activity|GO:0046982,protein heterodimerization activity|GO:0001071,nucleic acid binding transcription factor activity|GO:1990837,sequence-specific double-stranded DNA binding|GO:0003690,double-stranded DNA binding
SLC36A1	GO:1902600,hydrogen ion transmembrane transport|GO:0006865,amino acid transport|GO:0015816,glycine transport|GO:1903825,organic acid transmembrane transport|GO:0044699,single-organism process|GO:0015992,proton transport|GO:0051179,localization|GO:0071705,nitrogen compound transport|GO:0098662,inorganic cation transmembrane transport|GO:0006810,transport|GO:0098660,inorganic ion transmembrane transport|GO:1902578,single-organism localization|GO:0015672,monovalent inorganic cation transport|GO:0006818,hydrogen transport|GO:0015849,organic acid transport|GO:0006811,ion transport|GO:0009987,cellular process|GO:0015711,organic anion transport|GO:0015804,neutral amino acid transport|GO:0034220,ion transmembrane transport|GO:0044765,single-organism transport|GO:0015807,L-amino acid transport|GO:0044763,single-organism cellular process|GO:0015824,proline transport|GO:0051234,establishment of localization|GO:0015808,L-alanine transport|GO:0055085,transmembrane transport|GO:0006812,cation transport|GO:0046942,carboxylic acid transport|GO:0071702,organic substance transport|GO:0035524,proline transmembrane transport|GO:0003333,amino acid transmembrane transport|GO:0006820,anion transport|GO:0098656,anion transmembrane transport|GO:0032328,alanine transport|GO:0098655,cation transmembrane transport|GO:1905039,carboxylic acid transmembrane transport	GO:0000323,lytic vacuole|GO:0005783,endoplasmic reticulum|GO:0043229,intracellular organelle|GO:0071944,cell periphery|GO:0043227,membrane-bounded organelle|GO:0043226,organelle|GO:0031224,intrinsic component of membrane|GO:0005737,cytoplasm|GO:0044446,intracellular organelle part|GO:0031090,organelle membrane|GO:0005773,vacuole|GO:0016021,integral component of membrane|GO:0016020,membrane|GO:0005774,vacuolar membrane|GO:0044437,vacuolar part|GO:0098588,bounding membrane of organelle|GO:0012505,endomembrane system|GO:0005886,plasma membrane|GO:0043231,intracellular membrane-bounded organelle|GO:0044464,cell part|GO:0005623,cell|GO:0005622,intracellular|GO:0005764,lysosome|GO:0005765,lysosomal membrane|GO:0044444,cytoplasmic part|GO:0044424,intracellular part|GO:0044425,membrane part|GO:0098805,whole membrane|GO:0044422,organelle part|GO:0098852,lytic vacuole membrane	GO:0005342,organic acid transmembrane transporter activity|GO:0015187,glycine transmembrane transporter activity|GO:0015180,L-alanine transmembrane transporter activity|GO:0005416,cation:amino acid symporter activity|GO:0008509,anion transmembrane transporter activity|GO:0022857,transmembrane transporter activity|GO:0022804,active transmembrane transporter activity|GO:0015295,solute:proton symporter activity|GO:0015294,solute:cation symporter activity|GO:0015291,secondary active transmembrane transporter activity|GO:0015293,symporter activity|GO:0005215,transporter activity|GO:0008324,cation transmembrane transporter activity|GO:0015193,L-proline transmembrane transporter activity|GO:0046943,carboxylic acid transmembrane transporter activity|GO:0022891,substrate-specific transmembrane transporter activity|GO:0022890,inorganic cation transmembrane transporter activity|GO:0022892,substrate-specific transporter activity|GO:0008514,organic anion transmembrane transporter activity|GO:0005280,hydrogen:amino acid symporter activity|GO:0015078,hydrogen ion transmembrane transporter activity|GO:0015075,ion transmembrane transporter activity|GO:0015077,monovalent inorganic cation transmembrane transporter activity|GO:0015179,L-amino acid transmembrane transporter activity|GO:0015175,neutral amino acid transmembrane transporter activity|GO:0022858,alanine transmembrane transporter activity|GO:0015171,amino acid transmembrane transporter activity
BET1L	GO:0008104,protein localization|GO:0060341,regulation of cellular localization|GO:0051049,regulation of transport|GO:0032386,regulation of intracellular transport|GO:0061025,membrane fusion|GO:0061024,membrane organization|GO:0050789,regulation of biological process|GO:0044699,single-organism process|GO:0051234,establishment of localization|GO:0016043,cellular component organization|GO:0065007,biological regulation|GO:0071840,cellular component organization or biogenesis|GO:0071702,organic substance transport|GO:2000156,regulation of retrograde vesicle-mediated transport, Golgi to ER|GO:0033036,macromolecule localization|GO:0006810,transport|GO:0048193,Golgi vesicle transport|GO:0009987,cellular process|GO:0050794,regulation of cellular process|GO:0045184,establishment of protein localization|GO:0044765,single-organism transport|GO:0042147,retrograde transport, endosome to Golgi|GO:0051649,establishment of localization in cell|GO:0032879,regulation of localization|GO:0051179,localization|GO:1902578,single-organism localization|GO:0051641,cellular localization|GO:0016197,endosomal transport|GO:0006890,retrograde vesicle-mediated transport, Golgi to ER|GO:0016192,vesicle-mediated transport|GO:0046907,intracellular transport|GO:0060627,regulation of vesicle-mediated transport|GO:0015031,protein transport|GO:1902582,single-organism intracellular transport|GO:0015833,peptide transport|GO:0042886,amide transport|GO:0006888,ER to Golgi vesicle-mediated transport|GO:0071705,nitrogen compound transport|GO:0016482,cytosolic transport	GO:0043229,intracellular organelle|GO:0005622,intracellular|GO:0043227,membrane-bounded organelle|GO:0031201,SNARE complex|GO:0031224,intrinsic component of membrane|GO:0005737,cytoplasm|GO:0031984,organelle subcompartment|GO:0031090,organelle membrane|GO:0016021,integral component of membrane|GO:0016020,membrane|GO:0043226,organelle|GO:0044431,Golgi apparatus part|GO:0005795,Golgi stack|GO:0005794,Golgi apparatus|GO:0098588,bounding membrane of organelle|GO:0012505,endomembrane system|GO:0043234,protein complex|GO:0032991,macromolecular complex|GO:0043231,intracellular membrane-bounded organelle|GO:0044464,cell part|GO:0005623,cell|GO:0000139,Golgi membrane|GO:0044446,intracellular organelle part|GO:0098796,membrane protein complex|GO:0044444,cytoplasmic part|GO:0098791,Golgi subcompartment|GO:0044424,intracellular part|GO:0044425,membrane part|GO:0005768,endosome|GO:0044422,organelle part|GO:0031410,cytoplasmic vesicle|GO:0097708,intracellular vesicle|GO:0031982,vesicle|GO:0005829,cytosol	GO:0005484,SNAP receptor activity|GO:0005488,binding|GO:0005515,protein binding
GOLGA3	GO:0006891,intra-Golgi vesicle-mediated transport|GO:0016192,vesicle-mediated transport|GO:0046907,intracellular transport|GO:0048193,Golgi vesicle transport|GO:0006810,transport|GO:0044765,single-organism transport|GO:1902578,single-organism localization|GO:0051649,establishment of localization in cell|GO:1902582,single-organism intracellular transport|GO:0051234,establishment of localization|GO:0051179,localization|GO:0044699,single-organism process|GO:0051641,cellular localization|GO:0044703,multi-organism reproductive process|GO:0044702,single organism reproductive process|GO:0048609,multicellular organismal reproductive process|GO:0032504,multicellular organism reproduction|GO:0019953,sexual reproduction|GO:0022414,reproductive process|GO:0007283,spermatogenesis|GO:0032501,multicellular organismal process|GO:0048232,male gamete generation|GO:0051704,multi-organism process|GO:0007276,gamete generation|GO:0000003,reproduction	GO:0031974,membrane-enclosed lumen|GO:0019898,extrinsic component of membrane|GO:0043229,intracellular organelle|GO:0043228,non-membrane-bounded organelle|GO:0005622,intracellular|GO:0043227,membrane-bounded organelle|GO:0043226,organelle|GO:0005737,cytoplasm|GO:0031985,Golgi cisterna|GO:0031984,organelle subcompartment|GO:0031090,organelle membrane|GO:0031981,nuclear lumen|GO:0005730,nucleolus|GO:0005634,nucleus|GO:0016020,membrane|GO:0005654,nucleoplasm|GO:0044431,Golgi apparatus part|GO:0090498,extrinsic component of Golgi membrane|GO:0005795,Golgi stack|GO:0005794,Golgi apparatus|GO:0017119,Golgi transport complex|GO:0098588,bounding membrane of organelle|GO:0031312,extrinsic component of organelle membrane|GO:0012505,endomembrane system|GO:0032580,Golgi cisterna membrane|GO:0043234,protein complex|GO:0032991,macromolecular complex|GO:0043231,intracellular membrane-bounded organelle|GO:0043232,intracellular non-membrane-bounded organelle|GO:0043233,organelle lumen|GO:0005829,cytosol|GO:0044464,cell part|GO:0005623,cell|GO:0000139,Golgi membrane|GO:0044446,intracellular organelle part|GO:0070013,intracellular organelle lumen|GO:0044444,cytoplasmic part|GO:0044428,nuclear part|GO:0098791,Golgi subcompartment|GO:0044424,intracellular part|GO:0044425,membrane part|GO:0044422,organelle part	GO:0005215,transporter activity|GO:0005488,binding|GO:0005515,protein binding|GO:0045296,cadherin binding|GO:0050839,cell adhesion molecule binding

GOSR1	GO:0008104,protein localization|GO:0061025,membrane fusion|GO:0061024,membrane organization|GO:0051641,cellular localization|GO:0071840,cellular component organization or biogenesis|GO:0006888,ER to Golgi vesicle-mediated transport|GO:0016043,cellular component organization|GO:0044699,single-organism process|GO:0071702,organic substance transport|GO:0033036,macromolecule localization|GO:0009987,cellular process|GO:0048193,Golgi vesicle transport|GO:0006810,transport|GO:0045184,establishment of protein localization|GO:0044765,single-organism transport|GO:0042147,retrograde transport, endosome to Golgi|GO:0051649,establishment of localization in cell|GO:0051234,establishment of localization|GO:0051179,localization|GO:1902578,single-organism localization|GO:0016482,cytosolic transport|GO:0016197,endosomal transport|GO:0006891,intra-Golgi vesicle-mediated transport|GO:0016192,vesicle-mediated transport|GO:0046907,intracellular transport|GO:0015031,protein transport|GO:1902582,single-organism intracellular transport|GO:0048209,regulation of vesicle targeting, to, from or within Golgi|GO:0051648,vesicle localization|GO:0060341,regulation of cellular localization|GO:0051049,regulation of transport|GO:0006906,vesicle fusion|GO:0006903,vesicle targeting|GO:0015833,peptide transport|GO:0051656,establishment of organelle localization|GO:0050789,regulation of biological process|GO:0042886,amide transport|GO:0032879,regulation of localization|GO:0051650,establishment of vesicle localization|GO:0071705,nitrogen compound transport|GO:0065007,biological regulation|GO:0048199,vesicle targeting, to, from or within Golgi|GO:0050794,regulation of cellular process|GO:0044802,single-organism membrane organization|GO:0044801,single-organism membrane fusion|GO:0060627,regulation of vesicle-mediated transport|GO:0051640,organelle localization|GO:0006996,organelle organization|GO:0090174,organelle membrane fusion|GO:0016050,vesicle organization|GO:1902589,single-organism organelle organization|GO:0048284,organelle fusion|GO:0044763,single-organism cellular process	GO:0043229,intracellular organelle|GO:0005622,intracellular|GO:0043227,membrane-bounded organelle|GO:0031201,SNARE complex|GO:0031224,intrinsic component of membrane|GO:0005737,cytoplasm|GO:0031090,organelle membrane|GO:0016021,integral component of membrane|GO:0016020,membrane|GO:0043226,organelle|GO:0044431,Golgi apparatus part|GO:0005794,Golgi apparatus|GO:0098588,bounding membrane of organelle|GO:0012505,endomembrane system|GO:0043234,protein complex|GO:0032991,macromolecular complex|GO:0043231,intracellular membrane-bounded organelle|GO:0005801,cis-Golgi network|GO:0044464,cell part|GO:0005623,cell|GO:0000139,Golgi membrane|GO:0044446,intracellular organelle part|GO:0098796,membrane protein complex|GO:0044444,cytoplasmic part|GO:0044424,intracellular part|GO:0044425,membrane part|GO:0044422,organelle part|GO:0030133,transport vesicle|GO:0031985,Golgi cisterna|GO:0031984,organelle subcompartment|GO:0031410,cytoplasmic vesicle|GO:0005795,Golgi stack|GO:0005797,Golgi medial cisterna|GO:0097708,intracellular vesicle|GO:0031982,vesicle|GO:0005829,cytosol|GO:0098791,Golgi subcompartment	GO:0005484,SNAP receptor activity|GO:0005488,binding|GO:0005515,protein binding|GO:0000149,SNARE binding
PTPRG	GO:0044707,single-multicellular organism process|GO:0008152,metabolic process|GO:0051234,establishment of localization|GO:0016311,dephosphorylation|GO:0006470,protein dephosphorylation|GO:0030155,regulation of cell adhesion|GO:1901998,toxin transport|GO:0035335,peptidyl-tyrosine dephosphorylation|GO:0006928,movement of cell or subcellular component|GO:0032501,multicellular organismal process|GO:0023052,signaling|GO:0007165,signal transduction|GO:0007166,cell surface receptor signaling pathway|GO:0007167,enzyme linked receptor protein signaling pathway|GO:0007169,transmembrane receptor protein tyrosine kinase signaling pathway|GO:0001667,ameboidal-type cell migration|GO:0050789,regulation of biological process|GO:0044699,single-organism process|GO:0051179,localization|GO:0044267,cellular protein metabolic process|GO:0051716,cellular response to stimulus|GO:0007156,homophilic cell adhesion via plasma membrane adhesion molecules|GO:0044260,cellular macromolecule metabolic process|GO:0051241,negative regulation of multicellular organismal process|GO:0040012,regulation of locomotion|GO:0071704,organic substance metabolic process|GO:0065007,biological regulation|GO:0006810,transport|GO:0048519,negative regulation of biological process|GO:0016477,cell migration|GO:0030336,negative regulation of cell migration|GO:0030334,regulation of cell migration|GO:0044238,primary metabolic process|GO:0098609,cell-cell adhesion|GO:0006793,phosphorus metabolic process|GO:0051271,negative regulation of cellular component movement|GO:0006464,cellular protein modification process|GO:0050794,regulation of cellular process|GO:0043412,macromolecule modification|GO:1903385,regulation of homophilic cell adhesion|GO:0044763,single-organism cellular process|GO:0051239,regulation of multicellular organismal process|GO:0007155,cell adhesion|GO:0007154,cell communication|GO:0032879,regulation of localization|GO:0090132,epithelium migration|GO:0098742,cell-cell adhesion via plasma-membrane adhesion molecules|GO:0090130,tissue migration|GO:0040011,locomotion|GO:0044700,single organism signaling|GO:0040013,negative regulation of locomotion|GO:0051270,regulation of cellular component movement|GO:0051674,localization of cell|GO:0010631,epithelial cell migration|GO:0010632,regulation of epithelial cell migration|GO:0010633,negative regulation of epithelial cell migration|GO:0019538,protein metabolic process|GO:0022610,biological adhesion|GO:0050896,response to stimulus|GO:0048870,cell motility|GO:0044237,cellular metabolic process|GO:0043170,macromolecule metabolic process|GO:0006796,phosphate-containing compound metabolic process|GO:0036211,protein modification process|GO:2000145,regulation of cell motility|GO:2000146,negative regulation of cell motility|GO:0009987,cellular process|GO:0048523,negative regulation of cellular process|GO:0048468,cell development|GO:0060322,head development|GO:0031345,negative regulation of cell projection organization|GO:0031344,regulation of cell projection organization|GO:0071840,cellular component organization or biogenesis|GO:0048869,cellular developmental process|GO:0045665,negative regulation of neuron differentiation|GO:0045664,regulation of neuron differentiation|GO:0048513,animal organ development|GO:0010721,negative regulation of cell development|GO:0048699,generation of neurons|GO:0010977,negative regulation of neuron projection development|GO:0010975,regulation of neuron projection development|GO:1901564,organonitrogen compound metabolic process|GO:0006807,nitrogen compound metabolic process|GO:0031175,neuron projection development|GO:0016043,cellular component organization|GO:0050793,regulation of developmental process|GO:0007420,brain development|GO:0051961,negative regulation of nervous system development|GO:0051960,regulation of nervous system development|GO:0030154,cell differentiation|GO:0051129,negative regulation of cellular component organization|GO:0051128,regulation of cellular component organization|GO:0060284,regulation of cell development|GO:0007417,central nervous system development|GO:0050767,regulation of neurogenesis|GO:0050768,negative regulation of neurogenesis|GO:0032502,developmental process|GO:0045596,negative regulation of cell differentiation|GO:0045595,regulation of cell differentiation|GO:0051093,negative regulation of developmental process|GO:0048731,system development|GO:0030030,cell projection organization|GO:0007275,multicellular organism development|GO:0048666,neuron development|GO:0030182,neuron differentiation|GO:0044767,single-organism developmental process|GO:0022008,neurogenesis|GO:0007399,nervous system development|GO:0048856,anatomical structure development|GO:2000026,regulation of multicellular organismal development	GO:0071944,cell periphery|GO:0031226,intrinsic component of plasma membrane|GO:0043226,organelle|GO:0031224,intrinsic component of membrane|GO:0031982,vesicle|GO:0043227,membrane-bounded organelle|GO:0016021,integral component of membrane|GO:0016020,membrane|GO:0031988,membrane-bounded vesicle|GO:0044459,plasma membrane part|GO:0005887,integral component of plasma membrane|GO:0005886,plasma membrane|GO:1903561,extracellular vesicle|GO:0070062,extracellular exosome|GO:0043230,extracellular organelle|GO:0044464,cell part|GO:0005623,cell|GO:0005576,extracellular region|GO:0044425,membrane part|GO:0065010,extracellular membrane-bounded organelle|GO:0044421,extracellular region part|GO:0005615,extracellular space	GO:0042578,phosphoric ester hydrolase activity|GO:0038023,signaling receptor activity|GO:0005515,protein binding|GO:0016788,hydrolase activity, acting on ester bonds|GO:0016787,hydrolase activity|GO:0004725,protein tyrosine phosphatase activity|GO:0004721,phosphoprotein phosphatase activity|GO:0005001,transmembrane receptor protein tyrosine phosphatase activity|GO:0060089,molecular transducer activity|GO:0004888,transmembrane signaling receptor activity|GO:0042802,identical protein binding|GO:0004872,receptor activity|GO:0004871,signal transducer activity|GO:0016791,phosphatase activity|GO:0019198,transmembrane receptor protein phosphatase activity|GO:0003824,catalytic activity|GO:0005488,binding|GO:0099600,transmembrane receptor activity
NLRC3	GO:0080090,regulation of primary metabolic process|GO:0019222,regulation of metabolic process|GO:0048585,negative regulation of response to stimulus|GO:0048583,regulation of response to stimulus|GO:0007165,signal transduction|GO:1901362,organic cyclic compound biosynthetic process|GO:1901360,organic cyclic compound metabolic process|GO:0051716,cellular response to stimulus|GO:0010605,negative regulation of macromolecule metabolic process|GO:0009968,negative regulation of signal transduction|GO:0009966,regulation of signal transduction|GO:0009617,response to bacterium|GO:0070647,protein modification by small protein conjugation or removal|GO:0010033,response to organic substance|GO:0032446,protein modification by small protein conjugation|GO:0044092,negative regulation of molecular function|GO:0043124,negative regulation of I-kappaB kinase/NF-kappaB signaling|GO:0048519,negative regulation of biological process|GO:0051704,multi-organism process|GO:0043122,regulation of I-kappaB kinase/NF-kappaB signaling|GO:0060255,regulation of macromolecule metabolic process|GO:0042221,response to chemical|GO:0046649,lymphocyte activation|GO:2001141,regulation of RNA biosynthetic process|GO:0051707,response to other organism|GO:0043433,negative regulation of sequence-specific DNA binding transcription factor activity|GO:0046483,heterocycle metabolic process|GO:0044700,single organism signaling|GO:0009607,response to biotic stimulus|GO:0009605,response to external stimulus|GO:0044707,single-multicellular organism process|GO:0044249,cellular biosynthetic process|GO:0019538,protein metabolic process|GO:0002376,immune system process|GO:0016567,protein ubiquitination|GO:0019438,aromatic compound biosynthetic process|GO:0070489,T cell aggregation|GO:0009892,negative regulation of metabolic process|GO:0032640,tumor necrosis factor production|GO:0009890,negative regulation of biosynthetic process|GO:0008152,metabolic process|GO:0045321,leukocyte activation|GO:0010629,negative regulation of gene expression|GO:0006807,nitrogen compound metabolic process|GO:1903556,negative regulation of tumor necrosis factor superfamily cytokine production|GO:1903555,regulation of tumor necrosis factor superfamily cytokine production|GO:0035556,intracellular signal transduction|GO:0050789,regulation of biological process|GO:0097659,nucleic acid-templated transcription|GO:0044267,cellular protein metabolic process|GO:0044260,cellular macromolecule metabolic process|GO:0065007,biological regulation|GO:0034109,homotypic cell-cell adhesion|GO:0065009,regulation of molecular function|GO:0071593,lymphocyte aggregation|GO:0018130,heterocycle biosynthetic process|GO:0098602,single organism cell adhesion|GO:0098609,cell-cell adhesion|GO:0009889,regulation of biosynthetic process|GO:0050794,regulation of cellular process|GO:0001775,cell activation|GO:0036211,protein modification process|GO:0051239,regulation of multicellular organismal process|GO:1902532,negative regulation of intracellular signal transduction|GO:1902531,regulation of intracellular signal transduction|GO:0016070,RNA metabolic process|GO:0044271,cellular nitrogen compound biosynthetic process|GO:0043207,response to external biotic stimulus|GO:0050896,response to stimulus|GO:0043412,macromolecule modification|GO:0032088,negative regulation of NF-kappaB transcription factor activity|GO:0006355,regulation of transcription, DNA-templated|GO:0032715,negative regulation of interleukin-6 production|GO:0010556,regulation of macromolecule biosynthetic process|GO:0006351,transcription, DNA-templated|GO:0007159,leukocyte cell-cell adhesion|GO:0010558,negative regulation of macromolecule biosynthetic process|GO:0032774,RNA biosynthetic process|GO:0023057,negative regulation of signaling|GO:0034641,cellular nitrogen compound metabolic process|GO:0023052,signaling|GO:0010648,negative regulation of cell communication|GO:0034645,cellular macromolecule biosynthetic process|GO:0023051,regulation of signaling|GO:0010646,regulation of cell communication|GO:0044699,single-organism process|GO:0006139,nucleobase-containing compound metabolic process|GO:0051241,negative regulation of multicellular organismal process|GO:0051246,regulation of protein metabolic process|GO:1903320,regulation of protein modification by small protein conjugation or removal|GO:0031399,regulation of protein modification process|GO:0031396,regulation of protein ubiquitination|GO:0022610,biological adhesion|GO:0032675,regulation of interleukin-6 production|GO:0032501,multicellular organismal process|GO:0009987,cellular process|GO:0006725,cellular aromatic compound metabolic process|GO:1903506,regulation of nucleic acid-templated transcription|GO:1903507,negative regulation of nucleic acid-templated transcription|GO:0045892,negative regulation of transcription, DNA-templated|GO:0032268,regulation of cellular protein metabolic process|GO:0051253,negative regulation of RNA metabolic process|GO:0051252,regulation of RNA metabolic process|GO:0007249,I-kappaB kinase/NF-kappaB signaling|GO:0043170,macromolecule metabolic process|GO:0001816,cytokine production|GO:0001817,regulation of cytokine production|GO:0051090,regulation of sequence-specific DNA binding transcription factor activity|GO:0002237,response to molecule of bacterial origin|GO:0032720,negative regulation of tumor necrosis factor production|GO:0001818,negative regulation of cytokine production|GO:0016337,single organismal cell-cell adhesion|GO:0031327,negative regulation of cellular biosynthetic process|GO:0031326,regulation of cellular biosynthetic process|GO:0031324,negative regulation of cellular metabolic process|GO:0031323,regulation of cellular metabolic process|GO:0042110,T cell activation|GO:0090304,nucleic acid metabolic process|GO:0032496,response to lipopolysaccharide|GO:0033993,response to lipid|GO:2000112,regulation of cellular macromolecule biosynthetic process|GO:2000113,negative regulation of cellular macromolecule biosynthetic process|GO:0071704,organic substance metabolic process|GO:0010467,gene expression|GO:0071706,tumor necrosis factor superfamily cytokine production|GO:0010468,regulation of gene expression|GO:0045934,negative regulation of nucleobase-containing compound metabolic process|GO:1901576,organic substance biosynthetic process|GO:0019219,regulation of nucleobase-containing compound metabolic process|GO:0032635,interleukin-6 production|GO:0006464,cellular protein modification process|GO:1902679,negative regulation of RNA biosynthetic process|GO:0009058,biosynthetic process|GO:0009059,macromolecule biosynthetic process|GO:0044763,single-organism cellular process|GO:0051171,regulation of nitrogen compound metabolic process|GO:0051172,negative regulation of nitrogen compound metabolic process|GO:0007155,cell adhesion|GO:0007154,cell communication|GO:0070486,leukocyte aggregation|GO:1901700,response to oxygen-containing compound|GO:0044238,primary metabolic process|GO:0044237,cellular metabolic process|GO:0032680,regulation of tumor necrosis factor production|GO:0034654,nucleobase-containing compound biosynthetic process|GO:0048523,negative regulation of cellular process|GO:0050680,negative regulation of epithelial cell proliferation|GO:0031348,negative regulation of defense response|GO:0031347,regulation of defense response|GO:0050728,negative regulation of inflammatory response|GO:0071840,cellular component organization or biogenesis|GO:0070271,protein complex biogenesis|GO:0050727,regulation of inflammatory response|GO:0042127,regulation of cell proliferation|GO:0044546,NLRP3 inflammasome complex assembly|GO:0045824,negative regulation of innate immune response|GO:0032647,regulation of interferon-alpha production|GO:0022607,cellular component assembly|GO:0038061,NIK/NF-kappaB signaling|GO:0032648,regulation of interferon-beta production|GO:0050673,epithelial cell proliferation|GO:0002534,cytokine production involved in inflammatory response|GO:0016043,cellular component organization|GO:0065003,macromolecular complex assembly|GO:0002682,regulation of immune system process|GO:0002683,negative regulation of immune system process|GO:0006952,defense response|GO:0032480,negative regulation of type I interferon production|GO:0006955,immune response|GO:0043254,regulation of protein complex assembly|GO:0048015,phosphatidylinositol-mediated signaling|GO:0048017,inositol lipid-mediated signaling|GO:0080134,regulation of response to stress|GO:0006950,response to stress|GO:0009611,response to wounding|GO:0006954,inflammatory response|GO:0032102,negative regulation of response to external stimulus|GO:0032101,regulation of response to external stimulus|GO:0048147,negative regulation	GO:0005737,cytoplasm|GO:0044464,cell part|GO:0005623,cell|GO:0005622,intracellular|GO:0044424,intracellular part|GO:0005856,cytoskeleton|GO:0015630,microtubule cytoskeleton|GO:0043228,non-membrane-bounded organelle|GO:0043232,intracellular non-membrane-bounded organelle|GO:0005829,cytosol|GO:0044444,cytoplasmic part|GO:0005815,microtubule organizing center|GO:0044446,intracellular organelle part|GO:0043229,intracellular organelle|GO:0044430,cytoskeletal part|GO:0048471,perinuclear region of cytoplasm|GO:0043226,organelle|GO:0044422,organelle part	GO:0043168,anion binding|GO:0005488,binding|GO:0030554,adenyl nucleotide binding|GO:0035639,purine ribonucleoside triphosphate binding|GO:0097367,carbohydrate derivative binding|GO:1901363,heterocyclic compound binding|GO:1901265,nucleoside phosphate binding|GO:0000166,nucleotide binding|GO:0036094,small molecule binding|GO:0032553,ribonucleotide binding|GO:0032559,adenyl ribonucleotide binding|GO:0005524,ATP binding|GO:0032549,ribonucleoside binding|GO:0032555,purine ribonucleotide binding|GO:0017076,purine nucleotide binding|GO:0043167,ion binding|GO:0097159,organic cyclic compound binding|GO:0032550,purine ribonucleoside binding|GO:0001883,purine nucleoside binding|GO:0001882,nucleoside binding|GO:0043548,phosphatidylinositol 3-kinase binding|GO:0005515,protein binding|GO:0036312,phosphatidylinositol 3-kinase regulatory subunit binding
S100A8	GO:0090087,regulation of peptide transport|GO:2001235,positive regulation of apoptotic signaling pathway|GO:0051234,establishment of localization|GO:0080090,regulation of primary metabolic process|GO:0019222,regulation of metabolic process|GO:2001233,regulation of apoptotic signaling pathway|GO:0051049,regulation of transport|GO:0048584,positive regulation of response to stimulus|GO:0048583,regulation of response to stimulus|GO:0031349,positive regulation of defense response|GO:0016485,protein processing|GO:0007165,signal transduction|GO:0060326,cell chemotaxis|GO:0098771,inorganic ion homeostasis|GO:1901576,organic substance biosynthetic process|GO:1901362,organic cyclic compound biosynthetic process|GO:0031347,regulation of defense response|GO:0032774,RNA biosynthetic process|GO:0071705,nitrogen compound transport|GO:0051716,cellular response to stimulus|GO:0050729,positive regulation of inflammatory response|GO:0010604,positive regulation of macromolecule metabolic process|GO:0042330,taxis|GO:0009966,regulation of signal transduction|GO:0009967,positive regulation of signal transduction|GO:0071840,cellular component organization or biogenesis|GO:0051047,positive regulation of secretion|GO:0051493,regulation of cytoskeleton organization|GO:0050727,regulation of inflammatory response|GO:0018193,peptidyl-amino acid modification|GO:0033043,regulation of organelle organization|GO:0044093,positive regulation of molecular function|GO:0043281,regulation of cysteine-type endopeptidase activity involved in apoptotic process|GO:0043280,positive regulation of cysteine-type endopeptidase activity involved in apoptotic process|GO:0002526,acute inflammatory response|GO:0051704,multi-organism process|GO:0019725,cellular homeostasis|GO:0030595,leukocyte chemotaxis|GO:0030593,neutrophil chemotaxis|GO:0006935,chemotaxis|GO:0051050,positive regulation of transport|GO:0060255,regulation of macromolecule metabolic process|GO:0050832,defense response to fungus|GO:0042221,response to chemical|GO:0051090,regulation of sequence-specific DNA binding transcription factor activity|GO:0030162,regulation of proteolysis|GO:0010038,response to metal ion|GO:2001141,regulation of RNA biosynthetic process|GO:0010035,response to inorganic substance|GO:0051707,response to other organism|GO:0010033,response to organic substance|GO:0046483,heterocycle metabolic process|GO:0044700,single organism signaling|GO:0065008,regulation of biological quality|GO:0009607,response to biotic stimulus|GO:0009605,response to external stimulus|GO:0044707,single-multicellular organism process|GO:0048870,cell motility|GO:0019538,protein metabolic process|GO:0034641,cellular nitrogen compound metabolic process|GO:0043068,positive regulation of programmed cell death|GO:0002376,immune system process|GO:0007154,cell communication|GO:0019438,aromatic compound biosynthetic process|GO:0009893,positive regulation of metabolic process|GO:0009891,positive regulation of biosynthetic process|GO:0006950,response to stress|GO:0006810,transport|GO:0055076,transition metal ion homeostasis|GO:0006928,movement of cell or subcellular component|GO:0010043,response to zinc ion|GO:0010628,positive regulation of gene expression|GO:0042742,defense response to bacterium|GO:0035556,intracellular signal transduction|GO:0048878,chemical homeostasis|GO:0042981,regulation of apoptotic process|GO:0050789,regulation of biological process|GO:0097659,nucleic acid-templated transcription|GO:0001816,cytokine production|GO:0090304,nucleic acid metabolic process|GO:0046916,cellular transition metal ion homeostasis|GO:0051345,positive regulation of hydrolase activity|GO:0052547,regulation of peptidase activity|GO:0006955,immune response|GO:0006882,cellular zinc ion homeostasis|GO:0019219,regulation of nucleobase-containing compound metabolic process|GO:0006873,cellular ion homeostasis|GO:0044699,single-organism process|GO:0044267,cellular protein metabolic process|GO:0065009,regulation of molecular function|GO:0016477,cell migration|GO:0045087,innate immune response|GO:0018130,heterocycle biosynthetic process|GO:0016265,obsolete death|GO:0098602,single organism cell adhesion|GO:0006139,nucleobase-containing compound metabolic process|GO:0098609,cell-cell adhesion|GO:0050790,regulation of catalytic activity|GO:0009889,regulation of biosynthetic process|GO:0017014,protein nitrosylation|GO:0042060,wound healing|GO:0050794,regulation of cellular process|GO:0006952,defense response|GO:0012501,programmed cell death|GO:0043412,macromolecule modification|GO:0036211,protein modification process|GO:1901700,response to oxygen-containing compound|GO:0008152,metabolic process|GO:0051238,sequestering of metal ion|GO:0034654,nucleobase-containing compound biosynthetic process|GO:1902533,positive regulation of intracellular signal transduction|GO:0051235,maintenance of location|GO:1902531,regulation of intracellular signal transduction|GO:0002523,leukocyte migration involved in inflammatory response|GO:0006725,cellular aromatic compound metabolic process|GO:0018198,peptidyl-cysteine modification|GO:0051336,regulation of hydrolase activity|GO:0046903,secretion|GO:0055069,zinc ion homeostasis|GO:0044271,cellular nitrogen compound biosynthetic process|GO:0043207,response to external biotic stimulus|GO:0051604,protein maturation|GO:0080134,regulation of response to stress|GO:0009058,biosynthetic process|GO:0065007,biological regulation|GO:0006355,regulation of transcription, DNA-templated|GO:0010557,positive regulation of macromolecule biosynthetic process|GO:0010556,regulation of macromolecule biosynthetic process|GO:0006351,transcription, DNA-templated|GO:0044763,single-organism cellular process|GO:0051046,regulation of secretion|GO:0097305,response to alcohol|GO:0009611,response to wounding|GO:0070488,neutrophil aggregation|GO:0006954,inflammatory response|GO:0009617,response to bacterium|GO:0032103,positive regulation of response to external stimulus|GO:0032101,regulation of response to external stimulus|GO:0030307,positive regulation of cell growth|GO:0050801,ion homeostasis|GO:0055065,metal ion homeostasis|GO:0051128,regulation of cellular component organization|GO:0023056,positive regulation of signaling|GO:0044249,cellular biosynthetic process|GO:0015833,peptide transport|GO:0023052,signaling|GO:0070887,cellular response to chemical stimulus|GO:0023051,regulation of signaling|GO:0010647,positive regulation of cell communication|GO:0010646,regulation of cell communication|GO:0043085,positive regulation of catalytic activity|GO:0016043,cellular component organization|GO:0072507,divalent inorganic cation homeostasis|GO:0042886,amide transport|GO:0070486,leukocyte aggregation|GO:0072503,cellular divalent inorganic cation homeostasis|GO:0002544,chronic inflammatory response|GO:0051246,regulation of protein metabolic process|GO:0051247,positive regulation of protein metabolic process|GO:0032270,positive regulation of cellular protein metabolic process|GO:0097530,granulocyte migration|GO:2001244,positive regulation of intrinsic apoptotic signaling pathway|GO:0022610,biological adhesion|GO:1903034,regulation of response to wounding|GO:1990267,response to transition metal nanoparticle|GO:1903036,positive regulation of response to wounding|GO:2001242,regulation of intrinsic apoptotic signaling pathway|GO:1903508,positive regulation of nucleic acid-templated transcription|GO:0016070,RNA metabolic process|GO:0032602,chemokine production|GO:0006875,cellular metal ion homeostasis|GO:0031323,regulation of cellular metabolic process|GO:0009987,cellular process|GO:0032119,sequestering of zinc ion|GO:1903506,regulation of nucleic acid-templated transcription|GO:0048518,positive regulation of biological process|GO:0034645,cellular macromolecule biosynthetic process|GO:0001558,regulation of cell growth|GO:0098542,defense response to other organism|GO:0045893,positive regulation of transcription, DNA-templated|GO:0030003,cellular cation homeostasis|GO:0006508,proteolysis|GO:0055080,cation homeostasis|GO:0055082,cellular chemical homeostasis|GO:0032879,regulation of localization|GO:0051092,positive regulation of NF-kappaB transcription factor activity|GO:0051091,positive regulation of sequence-specific DNA binding transcription factor activity|GO:0032268,regulation of cellular protein metabolic process|GO:0051674,localization of cell|GO:0051252,regulation of RNA metabolic process|GO:0051254,positive regulation of RNA metabolic process|GO:0043170,macromolecule metabolic process|GO:1902680,positive regulation of RNA biosynthetic process|GO:0006807,nitrogen compound metabolic process|GO:0002237,response to molecule of bacterial origin|GO:0009620,response to fungus|GO:0045862,positive regulation of	GO:0043229,intracellular organelle|GO:0071944,cell periphery|GO:0043227,membrane-bounded organelle|GO:0043226,organelle|GO:0005856,cytoskeleton|GO:0031982,vesicle|GO:0005615,extracellular space|GO:0005634,nucleus|GO:0016020,membrane|GO:0031988,membrane-bounded vesicle|GO:0005576,extracellular region|GO:0005737,cytoplasm|GO:0005886,plasma membrane|GO:1903561,extracellular vesicle|GO:0070062,extracellular exosome|GO:0043230,extracellular organelle|GO:0043231,intracellular membrane-bounded organelle|GO:0043232,intracellular non-membrane-bounded organelle|GO:0005829,cytosol|GO:0044464,cell part|GO:0005623,cell|GO:0005622,intracellular|GO:0044444,cytoplasmic part|GO:0043228,non-membrane-bounded organelle|GO:0044424,intracellular part|GO:0065010,extracellular membrane-bounded organelle|GO:0044421,extracellular region part|GO:0031974,membrane-enclosed lumen|GO:0060205,cytoplasmic membrane-bounded vesicle lumen|GO:0045111,intermediate filament cytoskeleton|GO:0031983,vesicle lumen|GO:0016023,cytoplasmic, membrane-bounded vesicle|GO:0031410,cytoplasmic vesicle|GO:0044433,cytoplasmic vesicle part|GO:0099503,secretory vesicle|GO:0030141,secretory granule|GO:0097708,intracellular vesicle|GO:0034774,secretory granule lumen|GO:0043233,organelle lumen|GO:0044446,intracellular organelle part|GO:0044422,organelle part|GO:0012505,endomembrane system	GO:0050786,RAGE receptor binding|GO:0044877,macromolecular complex binding|GO:0008270,zinc ion binding|GO:0036041,long-chain fatty acid binding|GO:0043168,anion binding|GO:0043169,cation binding|GO:0046914,transition metal ion binding|GO:0043167,ion binding|GO:0005509,calcium ion binding|GO:0005504,fatty acid binding|GO:0046872,metal ion binding|GO:0035662,Toll-like receptor 4 binding|GO:0015631,tubulin binding|GO:0008017,microtubule binding|GO:0008289,lipid binding|GO:0036094,small molecule binding|GO:0032403,protein complex binding|GO:0035325,Toll-like receptor binding|GO:0033293,monocarboxylic acid binding|GO:0050542,icosanoid binding|GO:0050543,icosatetraenoic acid binding|GO:0050544,arachidonic acid binding|GO:1901567,fatty acid derivative binding|GO:0043177,organic acid binding|GO:0008092,cytoskeletal protein binding|GO:0031406,carboxylic acid binding|GO:0005515,protein binding|GO:0005102,receptor binding|GO:0005488,binding
DPM2	GO:0018410,C-terminal protein amino acid modification|GO:0006650,glycerophospholipid metabolic process|GO:0019222,regulation of metabolic process|GO:0016093,polyprenol metabolic process|GO:0044249,cellular biosynthetic process|GO:0065007,biological regulation|GO:0044281,small molecule metabolic process|GO:0034645,cellular macromolecule biosynthetic process|GO:1901576,organic substance biosynthetic process|GO:0035268,protein mannosylation|GO:0035269,protein O-linked mannosylation|GO:0044255,cellular lipid metabolic process|GO:0050789,regulation of biological process|GO:0044699,single-organism process|GO:0006629,lipid metabolic process|GO:0042157,lipoprotein metabolic process|GO:0045017,glycerolipid biosynthetic process|GO:0044710,single-organism metabolic process|GO:0044711,single-organism biosynthetic process|GO:0070085,glycosylation|GO:0006486,protein glycosylation|GO:0006487,protein N-linked glycosylation|GO:0006488,dolichol-linked oligosaccharide biosynthetic process|GO:0009100,glycoprotein metabolic process|GO:0018196,peptidyl-asparagine modification|GO:0018193,peptidyl-amino acid modification|GO:0009101,glycoprotein biosynthetic process|GO:0006501,C-terminal protein lipidation|GO:0071704,organic substance metabolic process|GO:0006505,GPI anchor metabolic process|GO:0042158,lipoprotein biosynthetic process|GO:0006506,GPI anchor biosynthetic process|GO:0046467,membrane lipid biosynthetic process|GO:0065009,regulation of molecular function|GO:0065008,regulation of biological quality|GO:0006664,glycolipid metabolic process|GO:0018279,protein N-linked glycosylation via asparagine|GO:0009059,macromolecule biosynthetic process|GO:1903509,liposaccharide metabolic process|GO:0006644,phospholipid metabolic process|GO:0009987,cellular process|GO:0006643,membrane lipid metabolic process|GO:0044267,cellular protein metabolic process|GO:0006720,isoprenoid metabolic process|GO:0043687,post-translational protein modification|GO:0050790,regulation of catalytic activity|GO:0009247,glycolipid biosynthetic process|GO:0006464,cellular protein modification process|GO:0009058,biosynthetic process|GO:0043412,macromolecule modification|GO:0043413,macromolecule glycosylation|GO:0046488,phosphatidylinositol metabolic process|GO:0008152,metabolic process|GO:0006661,phosphatidylinositol biosynthetic process|GO:0044723,single-organism carbohydrate metabolic process|GO:0005975,carbohydrate metabolic process|GO:0046486,glycerolipid metabolic process|GO:0090407,organophosphate biosynthetic process|GO:0008610,lipid biosynthetic process|GO:0006497,protein lipidation|GO:0044238,primary metabolic process|GO:0006493,protein O-linked glycosylation|GO:0044260,cellular macromolecule metabolic process|GO:0008654,phospholipid biosynthetic process|GO:0019538,protein metabolic process|GO:1901135,carbohydrate derivative metabolic process|GO:0031647,regulation of protein stability|GO:0044763,single-organism cellular process|GO:0097502,mannosylation|GO:0044237,cellular metabolic process|GO:0043170,macromolecule metabolic process|GO:0006066,alcohol metabolic process|GO:1901137,carbohydrate derivative biosynthetic process|GO:0006796,phosphate-containing compound metabolic process|GO:0036211,protein modification process|GO:0006793,phosphorus metabolic process|GO:0019637,organophosphate metabolic process|GO:0046474,glycerophospholipid biosynthetic process|GO:1901615,organic hydroxy compound metabolic process|GO:0016254,preassembly of GPI anchor in ER membrane|GO:0019348,dolichol metabolic process|GO:1901564,organonitrogen compound metabolic process|GO:0006807,nitrogen compound metabolic process|GO:1901566,organonitrogen compound biosynthetic process	GO:0005783,endoplasmic reticulum|GO:0005789,endoplasmic reticulum membrane|GO:0042175,nuclear outer membrane-endoplasmic reticulum membrane network|GO:0043229,intracellular organelle|GO:0030176,integral component of endoplasmic reticulum membrane|GO:0031301,integral component of organelle membrane|GO:0031300,intrinsic component of organelle membrane|GO:0043227,membrane-bounded organelle|GO:0043226,organelle|GO:0031224,intrinsic component of membrane|GO:0005737,cytoplasm|GO:0031090,organelle membrane|GO:0016021,integral component of membrane|GO:0016020,membrane|GO:0031227,intrinsic component of endoplasmic reticulum membrane|GO:0044432,endoplasmic reticulum part|GO:0033185,dolichol-phosphate-mannose synthase complex|GO:1902494,catalytic complex|GO:0098588,bounding membrane of organelle|GO:0012505,endomembrane system|GO:1990234,transferase complex|GO:0000506,glycosylphosphatidylinositol-N-acetylglucosaminyltransferase (GPI-GnT) complex|GO:0043234,protein complex|GO:0031501,mannosyltransferase complex|GO:0032991,macromolecular complex|GO:0043231,intracellular membrane-bounded organelle|GO:0044464,cell part|GO:0005623,cell|GO:0005622,intracellular|GO:0044446,intracellular organelle part|GO:0044444,cytoplasmic part|GO:0044424,intracellular part|GO:0044425,membrane part|GO:0044422,organelle part|GO:0031984,organelle subcompartment|GO:0098827,endoplasmic reticulum subcompartment	GO:0005515,protein binding|GO:0030234,enzyme regulator activity|GO:0003824,catalytic activity|GO:0016740,transferase activity|GO:0005488,binding|GO:0016757,transferase activity, transferring glycosyl groups|GO:0016758,transferase activity, transferring hexosyl groups|GO:0098772,molecular function regulator|GO:0004582,dolichyl-phosphate beta-D-mannosyltransferase activity|GO:0000030,mannosyltransferase activity
KIAA1614			
CMPK2	GO:0009211,pyrimidine deoxyribonucleoside triphosphate metabolic process|GO:0009212,pyrimidine deoxyribonucleoside triphosphate biosynthetic process|GO:0009219,pyrimidine deoxyribonucleotide metabolic process|GO:0009165,nucleotide biosynthetic process|GO:0044281,small molecule metabolic process|GO:1901362,organic cyclic compound biosynthetic process|GO:1901360,organic cyclic compound metabolic process|GO:0044710,single-organism metabolic process|GO:0044711,single-organism biosynthetic process|GO:0043207,response to external biotic stimulus|GO:0009617,response to bacterium|GO:0051704,multi-organism process|GO:0009197,pyrimidine deoxyribonucleoside diphosphate biosynthetic process|GO:0009196,pyrimidine deoxyribonucleoside diphosphate metabolic process|GO:0009202,deoxyribonucleoside triphosphate biosynthetic process|GO:0009200,deoxyribonucleoside triphosphate metabolic process|GO:0009607,response to biotic stimulus|GO:0051707,response to other organism|GO:0010033,response to organic substance|GO:0046483,heterocycle metabolic process|GO:1901564,organonitrogen compound metabolic process|GO:0009605,response to external stimulus|GO:1901566,organonitrogen compound biosynthetic process|GO:0006165,nucleoside diphosphate phosphorylation|GO:0019637,organophosphate metabolic process|GO:0019438,aromatic compound biosynthetic process|GO:0009148,pyrimidine nucleoside triphosphate biosynthetic process|GO:0009141,nucleoside triphosphate metabolic process|GO:0009142,nucleoside triphosphate biosynthetic process|GO:0009147,pyrimidine nucleoside triphosphate metabolic process|GO:0006807,nitrogen compound metabolic process|GO:0032496,response to lipopolysaccharide|GO:1901576,organic substance biosynthetic process|GO:0071219,cellular response to molecule of bacterial origin|GO:0071216,cellular response to biotic stimulus|GO:0018130,heterocycle biosynthetic process|GO:0006139,nucleobase-containing compound metabolic process|GO:0051716,cellular response to stimulus|GO:0009263,deoxyribonucleotide biosynthetic process|GO:0009262,deoxyribonucleotide metabolic process|GO:0009265,2'-deoxyribonucleotide biosynthetic process|GO:0008152,metabolic process|GO:0034654,nucleobase-containing compound biosynthetic process|GO:0090407,organophosphate biosynthetic process|GO:0044271,cellular nitrogen compound biosynthetic process|GO:0050896,response to stimulus|GO:1901293,nucleoside phosphate biosynthetic process|GO:0044763,single-organism cellular process|GO:0006753,nucleoside phosphate metabolic process|GO:0016310,phosphorylation|GO:0044249,cellular biosynthetic process|GO:0034641,cellular nitrogen compound metabolic process|GO:0070887,cellular response to chemical stimulus|GO:0044699,single-organism process|GO:0006220,pyrimidine nucleotide metabolic process|GO:0006221,pyrimidine nucleotide biosynthetic process|GO:0046385,deoxyribose phosphate biosynthetic process|GO:0006227,dUDP biosynthetic process|GO:0046939,nucleotide phosphorylation|GO:1901701,cellular response to oxygen-containing compound|GO:0009987,cellular process|GO:0006725,cellular aromatic compound metabolic process|GO:0009139,pyrimidine nucleoside diphosphate biosynthetic process|GO:0009138,pyrimidine nucleoside diphosphate metabolic process|GO:0046072,dTDP metabolic process|GO:0046075,dTTP metabolic process|GO:0046077,dUDP metabolic process|GO:0009133,nucleoside diphosphate biosynthetic process|GO:0009132,nucleoside diphosphate metabolic process|GO:0055086,nucleobase-containing small molecule metabolic process|GO:0006235,dTTP biosynthetic process|GO:0006233,dTDP biosynthetic process|GO:1901137,carbohydrate derivative biosynthetic process|GO:1901135,carbohydrate derivative metabolic process|GO:0002237,response to molecule of bacterial origin|GO:0009186,deoxyribonucleoside diphosphate metabolic process|GO:0071222,cellular response to lipopolysaccharide|GO:0009189,deoxyribonucleoside diphosphate biosynthetic process|GO:0072528,pyrimidine-containing compound biosynthetic process|GO:0072527,pyrimidine-containing compound metabolic process|GO:0033993,response to lipid|GO:0071704,organic substance metabolic process|GO:0071310,cellular response to organic substance|GO:0071396,cellular response to lipid|GO:0009221,pyrimidine deoxyribonucleotide biosynthetic process|GO:0009058,biosynthetic process|GO:0009117,nucleotide metabolic process|GO:0042221,response to chemical|GO:1901700,response to oxygen-containing compound|GO:0044238,primary metabolic process|GO:0009394,2'-deoxyribonucleotide metabolic process|GO:0044237,cellular metabolic process|GO:0019692,deoxyribose phosphate metabolic process|GO:0006796,phosphate-containing compound metabolic process|GO:0006793,phosphorus metabolic process	GO:0031974,membrane-enclosed lumen|GO:0043229,intracellular organelle|GO:0043227,membrane-bounded organelle|GO:0043226,organelle|GO:0005737,cytoplasm|GO:0031981,nuclear lumen|GO:0005634,nucleus|GO:0005739,mitochondrion|GO:0005654,nucleoplasm|GO:0043231,intracellular membrane-bounded organelle|GO:0043233,organelle lumen|GO:0005829,cytosol|GO:0044464,cell part|GO:0005623,cell|GO:0005622,intracellular|GO:0044446,intracellular organelle part|GO:0070013,intracellular organelle lumen|GO:0044444,cytoplasmic part|GO:0044428,nuclear part|GO:0044424,intracellular part|GO:0044422,organelle part	GO:0019201,nucleotide kinase activity|GO:0019205,nucleobase-containing compound kinase activity|GO:0035639,purine ribonucleoside triphosphate binding|GO:0005488,binding|GO:0004798,thymidylate kinase activity|GO:0009041,uridylate kinase activity|GO:0043167,ion binding|GO:1901363,heterocyclic compound binding|GO:0001883,purine nucleoside binding|GO:0001882,nucleoside binding|GO:0043168,anion binding|GO:0000166,nucleotide binding|GO:0016740,transferase activity|GO:1901265,nucleoside phosphate binding|GO:0032549,ribonucleoside binding|GO:0017076,purine nucleotide binding|GO:0005524,ATP binding|GO:0016301,kinase activity|GO:0004127,cytidylate kinase activity|GO:0036094,small molecule binding|GO:0003824,catalytic activity|GO:0032559,adenyl ribonucleotide binding|GO:0030554,adenyl nucleotide binding|GO:0033862,UMP kinase activity|GO:0016772,transferase activity, transferring phosphorus-containing groups|GO:0016776,phosphotransferase activity, phosphate group as acceptor|GO:0097367,carbohydrate derivative binding|GO:0004550,nucleoside diphosphate kinase activity|GO:0097159,organic cyclic compound binding|GO:0032555,purine ribonucleotide binding|GO:0032550,purine ribonucleoside binding|GO:0032553,ribonucleotide binding

TAP2	GO:0046719,regulation by virus of viral protein levels in host cell|GO:0019060,intracellular transport of viral protein in host cell|GO:0008104,protein localization|GO:0030581,symbiont intracellular protein transport in host|GO:0002479,antigen processing and presentation of exogenous peptide antigen via MHC class I, TAP-dependent|GO:0002483,antigen processing and presentation of endogenous peptide antigen|GO:0002591,positive regulation of antigen processing and presentation of peptide antigen via MHC class I|GO:0042592,homeostatic process|GO:0015833,peptide transport|GO:0044403,symbiosis, encompassing mutualism through parasitism|GO:0051641,cellular localization|GO:0048002,antigen processing and presentation of peptide antigen|GO:0019884,antigen processing and presentation of exogenous antigen|GO:0051179,localization|GO:0050789,regulation of biological process|GO:0044699,single-organism process|GO:0019885,antigen processing and presentation of endogenous peptide antigen via MHC class I|GO:0002475,antigen processing and presentation via MHC class Ib|GO:0044419,interspecies interaction between organisms|GO:0042886,amide transport|GO:0002476,antigen processing and presentation of endogenous peptide antigen via MHC class Ib|GO:0002579,positive regulation of antigen processing and presentation|GO:0019883,antigen processing and presentation of endogenous antigen|GO:0070727,cellular macromolecule localization|GO:0006886,intracellular protein transport|GO:0002577,regulation of antigen processing and presentation|GO:0002484,antigen processing and presentation of endogenous peptide antigen via MHC class I via ER pathway|GO:0002489,antigen processing and presentation of endogenous peptide antigen via MHC class Ib via ER pathway, TAP-dependent|GO:0075733,intracellular transport of virus|GO:0071705,nitrogen compound transport|GO:0002684,positive regulation of immune system process|GO:0019058,viral life cycle|GO:0002682,regulation of immune system process|GO:0071702,organic substance transport|GO:0048518,positive regulation of biological process|GO:0065007,biological regulation|GO:0051704,multi-organism process|GO:0065008,regulation of biological quality|GO:0002478,antigen processing and presentation of exogenous peptide antigen|GO:0034613,cellular protein localization|GO:0019882,antigen processing and presentation|GO:0006810,transport|GO:0002589,regulation of antigen processing and presentation of peptide antigen via MHC class I|GO:1902583,multi-organism intracellular transport|GO:0002474,antigen processing and presentation of peptide antigen via MHC class I|GO:0002585,positive regulation of antigen processing and presentation of peptide antigen|GO:0050792,regulation of viral process|GO:0002485,antigen processing and presentation of endogenous peptide antigen via MHC class I via ER pathway, TAP-dependent|GO:0009987,cellular process|GO:0002477,antigen processing and presentation of exogenous peptide antigen via MHC class Ib|GO:0002583,regulation of antigen processing and presentation of peptide antigen|GO:0050794,regulation of cellular process|GO:0046967,cytosol to ER transport|GO:0043903,regulation of symbiosis, encompassing mutualism through parasitism|GO:0043900,regulation of multi-organism process|GO:0044764,multi-organism cellular process|GO:0044763,single-organism cellular process|GO:0002481,antigen processing and presentation of exogenous protein antigen via MHC class Ib, TAP-dependent|GO:0008152,metabolic process|GO:0051649,establishment of localization in cell|GO:0051701,interaction with host|GO:0051234,establishment of localization|GO:0002488,antigen processing and presentation of endogenous peptide antigen via MHC class Ib via ER pathway|GO:0055085,transmembrane transport|GO:1902578,single-organism localization|GO:0046968,peptide antigen transport|GO:0045184,establishment of protein localization|GO:0002428,antigen processing and presentation of peptide antigen via MHC class Ib|GO:0033036,macromolecule localization|GO:0046907,intracellular transport|GO:0044766,multi-organism transport|GO:0044765,single-organism transport|GO:0042590,antigen processing and presentation of exogenous peptide antigen via MHC class I|GO:0048878,chemical homeostasis|GO:0002376,immune system process|GO:0051708,intracellular protein transport in other organism involved in symbiotic interaction|GO:0046794,transport of virus|GO:0016032,viral process|GO:0015031,protein transport|GO:1902582,single-organism intracellular transport|GO:1902579,multi-organism localization|GO:0016482,cytosolic transport|GO:0006906,vesicle fusion|GO:0061025,membrane fusion|GO:0061024,membrane organization|GO:0044802,single-organism membrane organization|GO:0016043,cellular component organization|GO:0071840,cellular component organization or biogenesis|GO:1990668,vesicle fusion with endoplasmic reticulum-Golgi intermediate compartment (ERGIC) membrane|GO:0006955,immune response|GO:0044801,single-organism membrane fusion|GO:0006996,organelle organization|GO:0016192,vesicle-mediated transport|GO:0090174,organelle membrane fusion|GO:0050896,response to stimulus|GO:0016050,vesicle organization|GO:1902589,single-organism organelle organization|GO:0048284,organelle fusion|GO:0002250,adaptive immune response	GO:0005783,endoplasmic reticulum|GO:0005789,endoplasmic reticulum membrane|GO:0042175,nuclear outer membrane-endoplasmic reticulum membrane network|GO:0043229,intracellular organelle|GO:0030176,integral component of endoplasmic reticulum membrane|GO:0031301,integral component of organelle membrane|GO:0031300,intrinsic component of organelle membrane|GO:0043227,membrane-bounded organelle|GO:0043226,organelle|GO:0031224,intrinsic component of membrane|GO:0005737,cytoplasm|GO:0031090,organelle membrane|GO:0016021,integral component of membrane|GO:0016020,membrane|GO:0043657,host cell|GO:0031227,intrinsic component of endoplasmic reticulum membrane|GO:0044432,endoplasmic reticulum part|GO:0042824,MHC class I peptide loading complex|GO:0042825,TAP complex|GO:0018995,host|GO:0098588,bounding membrane of organelle|GO:0012505,endomembrane system|GO:0032991,macromolecular complex|GO:0044216,other organism cell|GO:0043234,protein complex|GO:0044217,other organism part|GO:0043231,intracellular membrane-bounded organelle|GO:0044215,other organism|GO:0044464,cell part|GO:0005623,cell|GO:0005622,intracellular|GO:0044446,intracellular organelle part|GO:0098796,membrane protein complex|GO:0044444,cytoplasmic part|GO:0044424,intracellular part|GO:0044425,membrane part|GO:0044422,organelle part|GO:0033116,endoplasmic reticulum-Golgi intermediate compartment membrane|GO:0031974,membrane-enclosed lumen|GO:0030139,endocytic vesicle|GO:0016607,nuclear speck|GO:0005654,nucleoplasm|GO:0016604,nuclear body|GO:0031982,vesicle|GO:0031981,nuclear lumen|GO:0031410,cytoplasmic vesicle|GO:0005634,nucleus|GO:0044433,cytoplasmic vesicle part|GO:0098827,endoplasmic reticulum subcompartment|GO:0044451,nucleoplasm part|GO:0030666,endocytic vesicle membrane|GO:0005793,endoplasmic reticulum-Golgi intermediate compartment|GO:0030659,cytoplasmic vesicle membrane|GO:0045335,phagocytic vesicle|GO:0031984,organelle subcompartment|GO:0097708,intracellular vesicle|GO:0012506,vesicle membrane|GO:0098805,whole membrane|GO:0030670,phagocytic vesicle membrane|GO:0043233,organelle lumen|GO:0070013,intracellular organelle lumen|GO:0044428,nuclear part	GO:0035639,purine ribonucleoside triphosphate binding|GO:0015440,peptide-transporting ATPase activity|GO:0005488,binding|GO:0016887,ATPase activity|GO:0046977,TAP binding|GO:0046978,TAP1 binding|GO:0043167,ion binding|GO:1901363,heterocyclic compound binding|GO:0001883,purine nucleoside binding|GO:0001882,nucleoside binding|GO:0043168,anion binding|GO:0042626,ATPase activity, coupled to transmembrane movement of substances|GO:0042887,amide transmembrane transporter activity|GO:0042623,ATPase activity, coupled|GO:0000166,nucleotide binding|GO:0015399,primary active transmembrane transporter activity|GO:1901265,nucleoside phosphate binding|GO:0032549,ribonucleoside binding|GO:0017076,purine nucleotide binding|GO:0022804,active transmembrane transporter activity|GO:0016787,hydrolase activity|GO:0015433,peptide antigen-transporting ATPase activity|GO:0005215,transporter activity|GO:0017111,nucleoside-triphosphatase activity|GO:0036094,small molecule binding|GO:0003824,catalytic activity|GO:0016820,hydrolase activity, acting on acid anhydrides, catalyzing transmembrane movement of substances|GO:0015197,peptide transporter activity|GO:0023029,MHC class Ib protein binding|GO:0042287,MHC protein binding|GO:0022891,substrate-specific transmembrane transporter activity|GO:0016818,hydrolase activity, acting on acid anhydrides, in phosphorus-containing anhydrides|GO:0022892,substrate-specific transporter activity|GO:0030554,adenyl nucleotide binding|GO:0097367,carbohydrate derivative binding|GO:0097159,organic cyclic compound binding|GO:0043492,ATPase activity, coupled to movement of substances|GO:0016817,hydrolase activity, acting on acid anhydrides|GO:0032550,purine ribonucleoside binding|GO:0016462,pyrophosphatase activity|GO:0022857,transmembrane transporter activity|GO:0032559,adenyl ribonucleotide binding|GO:0005524,ATP binding|GO:0005515,protein binding|GO:0032555,purine ribonucleotide binding|GO:0046980,tapasin binding|GO:0005102,receptor binding|GO:0015405,P-P-bond-hydrolysis-driven transmembrane transporter activity|GO:0032553,ribonucleotide binding|GO:1904680,peptide transmembrane transporter activity
FAM86C1	GO:0032259,methylation|GO:0008152,metabolic process	GO:0043234,protein complex|GO:0032991,macromolecular complex	GO:0003824,catalytic activity|GO:0016740,transferase activity|GO:0016741,transferase activity, transferring one-carbon groups|GO:0005488,binding|GO:0005515,protein binding|GO:0008168,methyltransferase activity
ATG12	GO:0045087,innate immune response|GO:0009991,response to extracellular stimulus|GO:0022607,cellular component assembly|GO:0018410,C-terminal protein amino acid modification|GO:0051239,regulation of multicellular organismal process|GO:0006950,response to stress|GO:0002376,immune system process|GO:0016236,macroautophagy|GO:1903008,organelle disassembly|GO:0032606,type I interferon production|GO:0044248,cellular catabolic process|GO:0044249,cellular biosynthetic process|GO:0008152,metabolic process|GO:0042157,lipoprotein metabolic process|GO:0034645,cellular macromolecule biosynthetic process|GO:1901576,organic substance biosynthetic process|GO:0042158,lipoprotein biosynthetic process|GO:0050789,regulation of biological process|GO:0044699,single-organism process|GO:0031667,response to nutrient levels|GO:0044712,single-organism catabolic process|GO:0044267,cellular protein metabolic process|GO:0044710,single-organism metabolic process|GO:0043562,cellular response to nitrogen levels|GO:0009056,catabolic process|GO:0071840,cellular component organization or biogenesis|GO:0051241,negative regulation of multicellular organismal process|GO:0016043,cellular component organization|GO:0000045,autophagosome assembly|GO:0006501,C-terminal protein lipidation|GO:0071704,organic substance metabolic process|GO:0044085,cellular component biogenesis|GO:0065007,biological regulation|GO:0007005,mitochondrion organization|GO:0036211,protein modification process|GO:0048519,negative regulation of biological process|GO:0006914,autophagy|GO:0006497,protein lipidation|GO:0032501,multicellular organismal process|GO:0044238,primary metabolic process|GO:0061726,mitochondrion disassembly|GO:0043687,post-translational protein modification|GO:0009987,cellular process|GO:0006952,defense response|GO:0051716,cellular response to stimulus|GO:0006464,cellular protein modification process|GO:0044260,cellular macromolecule metabolic process|GO:0009058,biosynthetic process|GO:0071496,cellular response to external stimulus|GO:0044804,nucleophagy|GO:0032480,negative regulation of type I interferon production|GO:0022411,cellular component disassembly|GO:0009267,cellular response to starvation|GO:0006955,immune response|GO:0007154,cell communication|GO:0009605,response to external stimulus|GO:0070925,organelle assembly|GO:0009059,macromolecule biosynthetic process|GO:0006996,organelle organization|GO:0007033,vacuole organization|GO:0031668,cellular response to extracellular stimulus|GO:0006995,cellular response to nitrogen starvation|GO:0044707,single-multicellular organism process|GO:0000422,mitophagy|GO:0019538,protein metabolic process|GO:0050896,response to stimulus|GO:0043412,macromolecule modification|GO:0044237,cellular metabolic process|GO:0043170,macromolecule metabolic process|GO:0001816,cytokine production|GO:0001817,regulation of cytokine production|GO:1902589,single-organism organelle organization|GO:0031669,cellular response to nutrient levels|GO:0033554,cellular response to stress|GO:0032479,regulation of type I interferon production|GO:0044763,single-organism cellular process|GO:0042594,response to starvation|GO:0001818,negative regulation of cytokine production|GO:0006807,nitrogen compound metabolic process|GO:1905037,autophagosome organization|GO:1901564,organonitrogen compound metabolic process|GO:1901566,organonitrogen compound biosynthetic process	GO:0043234,protein complex|GO:0005737,cytoplasm|GO:0032991,macromolecular complex|GO:0031090,organelle membrane|GO:0098588,bounding membrane of organelle|GO:0016020,membrane|GO:0044464,cell part|GO:0005623,cell|GO:0098805,whole membrane|GO:0034045,pre-autophagosomal structure membrane|GO:0044444,cytoplasmic part|GO:0034274,Atg12-Atg5-Atg16 complex|GO:0044424,intracellular part|GO:0005622,intracellular|GO:0043227,membrane-bounded organelle|GO:0043226,organelle|GO:0044422,organelle part|GO:0000407,pre-autophagosomal structure|GO:0012506,vesicle membrane|GO:0030139,endocytic vesicle|GO:0043229,intracellular organelle|GO:0030666,endocytic vesicle membrane|GO:0031982,vesicle|GO:0031410,cytoplasmic vesicle|GO:0044433,cytoplasmic vesicle part|GO:1902494,catalytic complex|GO:0030659,cytoplasmic vesicle membrane|GO:0045335,phagocytic vesicle|GO:0097708,intracellular vesicle|GO:1990234,transferase complex|GO:0030670,phagocytic vesicle membrane|GO:0005829,cytosol|GO:0044446,intracellular organelle part|GO:0005773,vacuole|GO:0005776,autophagosome|GO:0043231,intracellular membrane-bounded organelle	GO:0019776,Atg8 ligase activity|GO:0003824,catalytic activity|GO:0016740,transferase activity|GO:0005488,binding|GO:0005515,protein binding
LINC00923			
AC103801	GO:0022604,regulation of cell morphogenesis|GO:0032502,developmental process|GO:0006921,cellular component disassembly involved in execution phase of apoptosis|GO:0022603,regulation of anatomical structure morphogenesis|GO:0097194,execution phase of apoptosis|GO:0051128,regulation of cellular component organization|GO:0022402,cell cycle process|GO:0008219,cell death|GO:0009653,anatomical structure morphogenesis|GO:0050789,regulation of biological process|GO:0007049,cell cycle|GO:0045786,negative regulation of cell cycle|GO:0000902,cell morphogenesis|GO:0048869,cellular developmental process|GO:0071840,cellular component organization or biogenesis|GO:0016043,cellular component organization|GO:0032989,cellular component morphogenesis|GO:0065007,biological regulation|GO:0044699,single-organism process|GO:0048519,negative regulation of biological process|GO:0065008,regulation of biological quality|GO:0016265,obsolete death|GO:0009987,cellular process|GO:0050793,regulation of developmental process|GO:0006915,apoptotic process|GO:0050794,regulation of cellular process|GO:0007050,cell cycle arrest|GO:0012501,programmed cell death|GO:0008360,regulation of cell shape|GO:0022411,cellular component disassembly|GO:0044767,single-organism developmental process|GO:0051726,regulation of cell cycle|GO:0048856,anatomical structure development|GO:0044763,single-organism cellular process|GO:0048523,negative regulation of cellular process	GO:0005856,cytoskeleton|GO:0043234,protein complex|GO:0043228,non-membrane-bounded organelle|GO:0015629,actin cytoskeleton|GO:0043232,intracellular non-membrane-bounded organelle|GO:0005829,cytosol|GO:0016020,membrane|GO:0044464,cell part|GO:0044444,cytoplasmic part|GO:0005623,cell|GO:0005737,cytoplasm|GO:0044446,intracellular organelle part|GO:0043229,intracellular organelle|GO:0044430,cytoskeletal part|GO:0005884,actin filament|GO:0044424,intracellular part|GO:0032991,macromolecular complex|GO:0005622,intracellular|GO:0043226,organelle|GO:0044422,organelle part|GO:0099512,supramolecular fiber|GO:0099513,polymeric cytoskeletal fiber	GO:0015631,tubulin binding|GO:0008092,cytoskeletal protein binding|GO:0008017,microtubule binding|GO:0005488,binding|GO:0005515,protein binding
TRADD	GO:2001235,positive regulation of apoptotic signaling pathway|GO:2001236,regulation of extrinsic apoptotic signaling pathway|GO:0019221,cytokine-mediated signaling pathway|GO:0019222,regulation of metabolic process|GO:2001233,regulation of apoptotic signaling pathway|GO:0048584,positive regulation of response to stimulus|GO:0048583,regulation of response to stimulus|GO:0007165,signal transduction|GO:0007166,cell surface receptor signaling pathway|GO:1901576,organic substance biosynthetic process|GO:0098773,skin epidermis development|GO:1901362,organic cyclic compound biosynthetic process|GO:0071840,cellular component organization or biogenesis|GO:0080090,regulation of primary metabolic process|GO:0043065,positive regulation of apoptotic process|GO:0051716,cellular response to stimulus|GO:0010605,negative regulation of macromolecule metabolic process|GO:0010604,positive regulation of macromolecule metabolic process|GO:0009966,regulation of signal transduction|GO:0009967,positive regulation of signal transduction|GO:0071310,cellular response to organic substance|GO:0043066,negative regulation of apoptotic process|GO:0051798,positive regulation of hair follicle development|GO:0044093,positive regulation of molecular function|GO:0044092,negative regulation of molecular function|GO:0043281,regulation of cysteine-type endopeptidase activity involved in apoptotic process|GO:0043280,positive regulation of cysteine-type endopeptidase activity involved in apoptotic process|GO:0051797,regulation of hair follicle development|GO:0043122,regulation of I-kappaB kinase/NF-kappaB signaling|GO:0043123,positive regulation of I-kappaB kinase/NF-kappaB signaling|GO:0010467,gene expression|GO:0060255,regulation of macromolecule metabolic process|GO:0060548,negative regulation of cell death|GO:0051291,protein heterooligomerization|GO:0032269,negative regulation of cellular protein metabolic process|GO:0042221,response to chemical|GO:0051090,regulation of sequence-specific DNA binding transcription factor activity|GO:0030162,regulation of proteolysis|GO:2001141,regulation of RNA biosynthetic process|GO:0010033,response to organic substance|GO:0046483,heterocycle metabolic process|GO:0044700,single organism signaling|GO:0044707,single-multicellular organism process|GO:0044249,cellular biosynthetic process|GO:0019538,protein metabolic process|GO:0043068,positive regulation of programmed cell death|GO:0007154,cell communication|GO:0043588,skin development|GO:0019438,aromatic compound biosynthetic process|GO:0022607,cellular component assembly|GO:0009892,negative regulation of metabolic process|GO:0009893,positive regulation of metabolic process|GO:0009891,positive regulation of biosynthetic process|GO:0008152,metabolic process|GO:0010628,positive regulation of gene expression|GO:0035556,intracellular signal transduction|GO:0042981,regulation of apoptotic process|GO:0050789,regulation of biological process|GO:0097659,nucleic acid-templated transcription|GO:0044267,cellular protein metabolic process|GO:0008544,epidermis development|GO:0051346,negative regulation of hydrolase activity|GO:0051345,positive regulation of hydrolase activity|GO:0071356,cellular response to tumor necrosis factor|GO:0052547,regulation of peptidase activity|GO:0016043,cellular component organization|GO:0065003,macromolecular complex assembly|GO:0019219,regulation of nucleobase-containing compound metabolic process|GO:0065007,biological regulation|GO:0044699,single-organism process|GO:0065009,regulation of molecular function|GO:0018130,heterocycle biosynthetic process|GO:0016265,obsolete death|GO:0006461,protein complex assembly|GO:0034097,response to cytokine|GO:0050793,regulation of developmental process|GO:0050790,regulation of catalytic activity|GO:0009889,regulation of biosynthetic process|GO:0009888,tissue development|GO:0050794,regulation of cellular process|GO:0012501,programmed cell death|GO:0051239,regulation of multicellular organismal process|GO:0048731,system development|GO:0042633,hair cycle|GO:1902533,positive regulation of intracellular signal transduction|GO:1902531,regulation of intracellular signal transduction|GO:0042303,molting cycle|GO:0042635,positive regulation of hair cycle|GO:0042634,regulation of hair cycle|GO:0051336,regulation of hydrolase activity|GO:0044271,cellular nitrogen compound biosynthetic process|GO:0051604,protein maturation|GO:0071345,cellular response to cytokine stimulus|GO:0050896,response to stimulus|GO:0032502,developmental process|GO:0006355,regulation of transcription, DNA-templated|GO:0010557,positive regulation of macromolecule biosynthetic process|GO:0010556,regulation of macromolecule biosynthetic process|GO:0006351,transcription, DNA-templated|GO:0097300,programmed necrotic cell death|GO:0001942,hair follicle development|GO:1902041,regulation of extrinsic apoptotic signaling pathway via death domain receptors|GO:0032774,RNA biosynthetic process|GO:0070271,protein complex biogenesis|GO:0033209,tumor necrosis factor-mediated signaling pathway|GO:0023056,positive regulation of signaling|GO:0034654,nucleobase-containing compound biosynthetic process|GO:0034641,cellular nitrogen compound metabolic process|GO:0023052,signaling|GO:0070887,cellular response to chemical stimulus|GO:0023051,regulation of signaling|GO:0010647,positive regulation of cell communication|GO:0010646,regulation of cell communication|GO:0043086,negative regulation of catalytic activity|GO:0043085,positive regulation of catalytic activity|GO:0006139,nucleobase-containing compound metabolic process|GO:0051248,negative regulation of protein metabolic process|GO:0008625,extrinsic apoptotic signaling pathway via death domain receptors|GO:0051240,positive regulation of multicellular organismal process|GO:0051246,regulation of protein metabolic process|GO:0051247,positive regulation of protein metabolic process|GO:0048513,animal organ development|GO:0032270,positive regulation of cellular protein metabolic process|GO:0006508,proteolysis|GO:1903508,positive regulation of nucleic acid-templated transcription|GO:0016070,RNA metabolic process|GO:0070266,necroptotic process|GO:0010939,regulation of necrotic cell death|GO:0031323,regulation of cellular metabolic process|GO:0032501,multicellular organismal process|GO:0060429,epithelium development|GO:0006725,cellular aromatic compound metabolic process|GO:1903506,regulation of nucleic acid-templated transcription|GO:0048518,positive regulation of biological process|GO:0034645,cellular macromolecule biosynthetic process|GO:0045893,positive regulation of transcription, DNA-templated|GO:0048519,negative regulation of biological process|GO:0016485,protein processing|GO:0051259,protein oligomerization|GO:0051092,positive regulation of NF-kappaB transcription factor activity|GO:0051091,positive regulation of sequence-specific DNA binding transcription factor activity|GO:0032268,regulation of cellular protein metabolic process|GO:0051094,positive regulation of developmental process|GO:0051252,regulation of RNA metabolic process|GO:0007249,I-kappaB kinase/NF-kappaB signaling|GO:0051254,positive regulation of RNA metabolic process|GO:0043170,macromolecule metabolic process|GO:1902680,positive regulation of RNA biosynthetic process|GO:0006807,nitrogen compound metabolic process|GO:0045862,positive regulation of proteolysis|GO:0045861,negative regulation of proteolysis|GO:0070265,necrotic cell death|GO:0031328,positive regulation of cellular biosynthetic process|GO:0043933,macromolecular complex subunit organization|GO:0031326,regulation of cellular biosynthetic process|GO:0031325,positive regulation of cellular metabolic process|GO:0031324,negative regulation of cellular metabolic process|GO:0097190,apoptotic signaling pathway|GO:0097191,extrinsic apoptotic signaling pathway|GO:2001056,positive regulation of cysteine-type endopeptidase activity|GO:0090304,nucleic acid metabolic process|GO:0022404,molting cycle process|GO:0022405,hair cycle process|GO:0010942,positive regulation of cell death|GO:0045682,regulation of epidermis development|GO:0008219,cell death|GO:0010941,regulation of cell death|GO:0007275,multicellular organism development|GO:0045684,positive regulation of epidermis development|GO:0097202,activation of cysteine-type endopeptidase activity|GO:0071822,protein complex subunit organization|GO:0043154,negative regulation of cysteine-type endopeptidase activity involved in apoptotic process|GO:2000116,regulation of cysteine-type endopeptidase activity|GO:2000117,negative regulation of cysteine-type endopeptidase activity|GO:1901360,organic cyclic compound metabolic process|GO:2000112,regulation of cellular macromolecule biosynthetic process|GO:0031638,zymogen activation|GO:2001267,regulation of cysteine-type	GO:0031264,death-inducing signaling complex|GO:0043228,non-membrane-bounded organelle|GO:0043227,membrane-bounded organelle|GO:0043226,organelle|GO:0005856,cytoskeleton|GO:0005634,nucleus|GO:0016020,membrane|GO:0044459,plasma membrane part|GO:0005737,cytoplasm|GO:0098589,membrane region|GO:0005886,plasma membrane|GO:0043234,protein complex|GO:0043235,receptor complex|GO:0032991,macromolecular complex|GO:0043231,intracellular membrane-bounded organelle|GO:0043232,intracellular non-membrane-bounded organelle|GO:0005829,cytosol|GO:0045121,membrane raft|GO:0044464,cell part|GO:0043229,intracellular organelle|GO:0005623,cell|GO:0005622,intracellular|GO:0098797,plasma membrane protein complex|GO:0098796,membrane protein complex|GO:0044444,cytoplasmic part|GO:0071944,cell periphery|GO:0044424,intracellular part|GO:0044425,membrane part|GO:0098805,whole membrane|GO:0098857,membrane microdomain	GO:0005126,cytokine receptor binding|GO:0032813,tumor necrosis factor receptor superfamily binding|GO:0005515,protein binding|GO:0019900,kinase binding|GO:0019904,protein domain specific binding|GO:0019899,enzyme binding|GO:0070513,death domain binding|GO:0060089,molecular transducer activity|GO:0044877,macromolecular complex binding|GO:0005164,tumor necrosis factor receptor binding|GO:0004871,signal transducer activity|GO:0032403,protein complex binding|GO:0005488,binding|GO:0005102,receptor binding|GO:0042802,identical protein binding|GO:0060090,binding, bridging
IFT140	GO:0022607,cellular component assembly|GO:0051234,establishment of localization|GO:0030030,cell projection organization|GO:0030031,cell projection assembly|GO:0006810,transport|GO:0042073,intraciliary transport|GO:0010927,cellular component assembly involved in morphogenesis|GO:0032989,cellular component morphogenesis|GO:0044699,single-organism process|GO:0070925,organelle assembly|GO:0000902,cell morphogenesis|GO:0009653,anatomical structure morphogenesis|GO:0048869,cellular developmental process|GO:0016043,cellular component organization|GO:0030705,cytoskeleton-dependent intracellular transport|GO:0071840,cellular component organization or biogenesis|GO:0035721,intraciliary retrograde transport|GO:0051641,cellular localization|GO:0048646,anatomical structure formation involved in morphogenesis|GO:0032502,developmental process|GO:0042384,cilium assembly|GO:0060271,cilium morphogenesis|GO:0009987,cellular process|GO:0044767,single-organism developmental process|GO:0044765,single-organism transport|GO:0010970,transport along microtubule|GO:0044763,single-organism cellular process|GO:0051649,establishment of localization in cell|GO:0006928,movement of cell or subcellular component|GO:0051179,localization|GO:1902578,single-organism localization|GO:0048858,cell projection morphogenesis|GO:0006996,organelle organization|GO:0007017,microtubule-based process|GO:0032990,cell part morphogenesis|GO:0046907,intracellular transport|GO:0048856,anatomical structure development|GO:0007018,microtubule-based movement|GO:1902589,single-organism organelle organization|GO:0044085,cellular component biogenesis|GO:1902582,single-organism intracellular transport|GO:0044782,cilium organization	GO:0005737,cytoplasm|GO:0044463,cell projection part|GO:0005622,intracellular|GO:0032838,cell projection cytoplasm|GO:0005929,cilium|GO:0044464,cell part|GO:0005623,cell|GO:0005930,axoneme|GO:0044444,cytoplasmic part|GO:0097014,ciliary plasm|GO:0044441,ciliary part|GO:0044424,intracellular part|GO:0042995,cell projection|GO:0043226,organelle|GO:0044422,organelle part	
ELMO3	GO:0016265,obsolete death|GO:0040011,locomotion|GO:0009987,cellular process|GO:0006909,phagocytosis|GO:0016192,vesicle-mediated transport|GO:0051234,establishment of localization|GO:0048870,cell motility|GO:0006915,apoptotic process|GO:0006928,movement of cell or subcellular component|GO:0012501,programmed cell death|GO:0044765,single-organism transport|GO:0051674,localization of cell|GO:0044763,single-organism cellular process|GO:0044699,single-organism process|GO:0006810,transport|GO:0006897,endocytosis|GO:0008219,cell death|GO:0016477,cell migration|GO:0051179,localization|GO:1902578,single-organism localization	GO:0005737,cytoplasm|GO:0044464,cell part|GO:0005623,cell|GO:0005622,intracellular|GO:0044424,intracellular part	GO:0005488,binding|GO:0017124,SH3 domain binding|GO:0005515,protein binding|GO:0019904,protein domain specific binding
FAM86B2	GO:0032259,methylation|GO:0008152,metabolic process	GO:0043234,protein complex|GO:0032991,macromolecular complex	GO:0008168,methyltransferase activity|GO:0016740,transferase activity|GO:0016741,transferase activity, transferring one-carbon groups|GO:0003824,catalytic activity|GO:0005488,binding|GO:0005515,protein binding
FAM169B			
VAMP1	GO:0022607,cellular component assembly|GO:0070271,protein complex biogenesis|GO:0043933,macromolecular complex subunit organization|GO:0006906,vesicle fusion|GO:0061025,membrane fusion|GO:0061024,membrane organization|GO:0032940,secretion by cell|GO:0023052,signaling|GO:0034622,cellular macromolecular complex assembly|GO:0044699,single-organism process|GO:0071822,protein complex subunit organization|GO:0006887,exocytosis|GO:0016043,cellular component organization|GO:0065003,macromolecular complex assembly|GO:0071840,cellular component organization or biogenesis|GO:0006810,transport|GO:0051649,establishment of localization in cell|GO:0009987,cellular process|GO:0006461,protein complex assembly|GO:0044765,single-organism transport|GO:0044763,single-organism cellular process|GO:0007268,chemical synaptic transmission|GO:0044801,single-organism membrane fusion|GO:0007267,cell-cell signaling|GO:0007154,cell communication|GO:0051234,establishment of localization|GO:0043623,cellular protein complex assembly|GO:0051179,localization|GO:1902578,single-organism localization|GO:0051641,cellular localization|GO:0006996,organelle organization|GO:0044700,single organism signaling|GO:0046903,secretion|GO:0016192,vesicle-mediated transport|GO:0090174,organelle membrane fusion|GO:0016050,vesicle organization|GO:1902589,single-organism organelle organization|GO:0044085,cellular component biogenesis|GO:0048284,organelle fusion|GO:0044802,single-organism membrane organization|GO:0035493,SNARE complex assembly	GO:0008021,synaptic vesicle|GO:0042995,cell projection|GO:0000323,lytic vacuole|GO:0005887,integral component of plasma membrane|GO:0044444,cytoplasmic part|GO:0043679,axon terminus|GO:0031975,envelope|GO:0043226,organelle|GO:0043229,intracellular organelle|GO:0035579,specific granule membrane|GO:0045202,synapse|GO:0005773,vacuole|GO:0031201,SNARE complex|GO:0005774,vacuolar membrane|GO:0043227,membrane-bounded organelle|GO:0035577,azurophil granule membrane|GO:0030054,cell junction|GO:0005737,cytoplasm|GO:0005764,lysosome|GO:0070821,tertiary granule membrane|GO:0070820,tertiary granule|GO:0031982,vesicle|GO:0098796,membrane protein complex|GO:0016023,cytoplasmic, membrane-bounded vesicle|GO:0031410,cytoplasmic vesicle|GO:0016021,integral component of membrane|GO:0016020,membrane|GO:0005766,primary lysosome|GO:0005739,mitochondrion|GO:0031988,membrane-bounded vesicle|GO:0044433,cytoplasmic vesicle part|GO:0097458,neuron part|GO:0044456,synapse part|GO:0044437,vacuolar part|GO:0031224,intrinsic component of membrane|GO:0043005,neuron projection|GO:0044459,plasma membrane part|GO:0032991,macromolecular complex|GO:0031968,organelle outer membrane|GO:0031226,intrinsic component of plasma membrane|GO:0030141,secretory granule|GO:0098588,bounding membrane of organelle|GO:0071944,cell periphery|GO:0030667,secretory granule membrane|GO:0031967,organelle envelope|GO:0031966,mitochondrial membrane|GO:0042581,specific granule|GO:0030659,cytoplasmic vesicle membrane|GO:0042582,azurophil granule|GO:0012505,endomembrane system|GO:0012506,vesicle membrane|GO:0005886,plasma membrane|GO:0043195,terminal bouton|GO:0030424,axon|GO:0009986,cell surface|GO:0031090,organelle membrane|GO:0043234,protein complex|GO:0030672,synaptic vesicle membrane|GO:0043231,intracellular membrane-bounded organelle|GO:0005829,cytosol|GO:0044463,cell projection part|GO:0044464,cell part|GO:0019867,outer membrane|GO:0005623,cell|GO:0005622,intracellular|GO:0044446,intracellular organelle part|GO:0005765,lysosomal membrane|GO:0005740,mitochondrial envelope|GO:0005741,mitochondrial outer membrane|GO:0098793,presynapse|GO:0044429,mitochondrial part|GO:0044306,neuron projection terminus|GO:0033267,axon part|GO:0044424,intracellular part|GO:0044425,membrane part|GO:0098805,whole membrane|GO:0044422,organelle part|GO:0030133,transport vesicle|GO:0070382,exocytic vesicle|GO:0099501,exocytic vesicle membrane|GO:0099503,secretory vesicle|GO:0030658,transport vesicle membrane|GO:0097708,intracellular vesicle|GO:0098852,lytic vacuole membrane	GO:0005484,SNAP receptor activity|GO:0000149,SNARE binding|GO:0005488,binding|GO:0005515,protein binding|GO:0019905,syntaxin binding
SRRM4	GO:0048856,anatomical structure development|GO:0080090,regulation of primary metabolic process|GO:0019222,regulation of metabolic process|GO:0050684,regulation of mRNA processing|GO:0030154,cell differentiation|GO:0031323,regulation of cellular metabolic process|GO:0090304,nucleic acid metabolic process|GO:0034641,cellular nitrogen compound metabolic process|GO:0006807,nitrogen compound metabolic process|GO:0044237,cellular metabolic process|GO:0048024,regulation of mRNA splicing, via spliceosome|GO:0043170,macromolecule metabolic process|GO:0007275,multicellular organism development|GO:0044699,single-organism process|GO:0006139,nucleobase-containing compound metabolic process|GO:0000381,regulation of alternative mRNA splicing, via spliceosome|GO:0000380,alternative mRNA splicing, via spliceosome|GO:0044260,cellular macromolecule metabolic process|GO:0071704,organic substance metabolic process|GO:0048869,cellular developmental process|GO:0043484,regulation of RNA splicing|GO:0050789,regulation of biological process|GO:0060255,regulation of macromolecule metabolic process|GO:0010467,gene expression|GO:0065007,biological regulation|GO:1901360,organic cyclic compound metabolic process|GO:0050877,neurological system process|GO:0046483,heterocycle metabolic process|GO:0010468,regulation of gene expression|GO:0032502,developmental process|GO:0032501,multicellular organismal process|GO:0044238,primary metabolic process|GO:0019219,regulation of nucleobase-containing compound metabolic process|GO:0050954,sensory perception of mechanical stimulus|GO:0009987,cellular process|GO:0006725,cellular aromatic compound metabolic process|GO:0007600,sensory perception|GO:0050794,regulation of cellular process|GO:0044767,single-organism developmental process|GO:0000375,RNA splicing, via transesterification reactions|GO:0000377,RNA splicing, via transesterification reactions with bulged adenosine as nucleophile|GO:0044763,single-organism cellular process|GO:0051171,regulation of nitrogen compound metabolic process|GO:0008152,metabolic process|GO:0007605,sensory perception of sound|GO:0003008,system process|GO:0016070,RNA metabolic process|GO:0016071,mRNA metabolic process|GO:0044707,single-multicellular organism process|GO:0007399,nervous system development|GO:0051252,regulation of RNA metabolic process|GO:0000398,mRNA splicing, via spliceosome|GO:0008380,RNA splicing|GO:0048731,system development|GO:1903311,regulation of mRNA metabolic process|GO:0006396,RNA processing|GO:0006397,mRNA processing	GO:0043231,intracellular membrane-bounded organelle|GO:0005634,nucleus|GO:0044464,cell part|GO:0005623,cell|GO:0005622,intracellular|GO:0043229,intracellular organelle|GO:0044424,intracellular part|GO:0043227,membrane-bounded organelle|GO:0043226,organelle	GO:0005488,binding|GO:0097159,organic cyclic compound binding|GO:0044822,poly(A) RNA binding|GO:0003723,RNA binding|GO:0003676,nucleic acid binding|GO:1901363,heterocyclic compound binding|GO:0003729,mRNA binding
FLJ00104			
NPAP1	GO:0030154,cell differentiation|GO:0019953,sexual reproduction|GO:0007275,multicellular organism development|GO:0044699,single-organism process|GO:0000003,reproduction|GO:0048869,cellular developmental process|GO:0007276,gamete generation|GO:0032502,developmental process|GO:0032501,multicellular organismal process|GO:0048609,multicellular organismal reproductive process|GO:0032504,multicellular organism reproduction|GO:0009987,cellular process|GO:0044767,single-organism developmental process|GO:0022414,reproductive process|GO:0007283,spermatogenesis|GO:0044763,single-organism cellular process|GO:0048232,male gamete generation|GO:0051704,multi-organism process|GO:0044703,multi-organism reproductive process|GO:0044702,single organism reproductive process|GO:0044707,single-multicellular organism process|GO:0034504,protein localization to nucleus|GO:0015931,nucleobase-containing compound transport|GO:0051169,nuclear transport|GO:0051168,nuclear export|GO:0050657,nucleic acid transport|GO:0015833,peptide transport|GO:0008104,protein localization|GO:0051236,establishment of RNA localization|GO:0033036,macromolecule localization|GO:0042886,amide transport|GO:0051649,establishment of localization in cell|GO:0070727,cellular macromolecule localization|GO:0006886,intracellular protein transport|GO:0071705,nitrogen compound transport|GO:0006810,transport|GO:0071702,organic substance transport|GO:0006913,nucleocytoplasmic transport|GO:0006403,RNA localization|GO:0006405,RNA export from nucleus|GO:0034613,cellular protein localization|GO:0072594,establishment of protein localization to organelle|GO:0017038,protein import|GO:0006606,protein import into nucleus|GO:0050658,RNA transport|GO:0006605,protein targeting|GO:0044765,single-organism transport|GO:0044744,protein targeting to nucleus|GO:0051234,establishment of localization|GO:0051179,localization|GO:1902578,single-organism localization|GO:0051641,cellular localization|GO:0045184,establishment of protein localization|GO:0046907,intracellular transport|GO:0048856,anatomical structure development|GO:0033365,protein localization to organelle|GO:0015031,protein transport|GO:1902582,single-organism intracellular transport	GO:0016020,membrane|GO:0044464,cell part|GO:0031974,membrane-enclosed lumen|GO:0031975,envelope|GO:0043229,intracellular organelle|GO:0043227,membrane-bounded organelle|GO:0043226,organelle|GO:0031090,organelle membrane|GO:0031981,nuclear lumen|GO:0005637,nuclear inner membrane|GO:0005634,nucleus|GO:0005635,nuclear envelope|GO:0031988,membrane-bounded vesicle|GO:0005654,nucleoplasm|GO:0031965,nuclear membrane|GO:0031982,vesicle|GO:0031967,organelle envelope|GO:0012505,endomembrane system|GO:1903561,extracellular vesicle|GO:0070062,extracellular exosome|GO:0043230,extracellular organelle|GO:0043231,intracellular membrane-bounded organelle|GO:0043233,organelle lumen|GO:0019866,organelle inner membrane|GO:0005623,cell|GO:0005622,intracellular|GO:0044446,intracellular organelle part|GO:0070013,intracellular organelle lumen|GO:0005576,extracellular region|GO:0044428,nuclear part|GO:0044424,intracellular part|GO:0065010,extracellular membrane-bounded organelle|GO:0044421,extracellular region part|GO:0044422,organelle part|GO:0044217,other organism part|GO:0044216,other organism cell|GO:0044615,nuclear pore nuclear basket|GO:0044613,nuclear pore central transport channel|GO:0043657,host cell|GO:0018995,host|GO:0032991,macromolecular complex|GO:0043234,protein complex|GO:0044215,other organism|GO:0005643,nuclear pore	GO:0017056,structural constituent of nuclear pore|GO:0042277,peptide binding|GO:0005048,signal sequence binding|GO:0008139,nuclear localization sequence binding|GO:0005215,transporter activity|GO:0005198,structural molecule activity|GO:0005488,binding|GO:0033218,amide binding|GO:0005487,nucleocytoplasmic transporter activity

HGSNAT	GO:0022607,cellular component assembly|GO:0030203,glycosaminoglycan metabolic process|GO:0070271,protein complex biogenesis|GO:0043933,macromolecular complex subunit organization|GO:0006807,nitrogen compound metabolic process|GO:0044281,small molecule metabolic process|GO:0007041,lysosomal transport|GO:0044699,single-organism process|GO:0044710,single-organism metabolic process|GO:0071822,protein complex subunit organization|GO:0016043,cellular component organization|GO:0051179,localization|GO:0065003,macromolecular complex assembly|GO:0071704,organic substance metabolic process|GO:0071840,cellular component organization or biogenesis|GO:0006810,transport|GO:0009987,cellular process|GO:0006461,protein complex assembly|GO:1901575,organic substance catabolic process|GO:0044765,single-organism transport|GO:0008152,metabolic process|GO:0051649,establishment of localization in cell|GO:1901564,organonitrogen compound metabolic process|GO:0051234,establishment of localization|GO:0009056,catabolic process|GO:0009057,macromolecule catabolic process|GO:1902578,single-organism localization|GO:0051641,cellular localization|GO:0051259,protein oligomerization|GO:0044238,primary metabolic process|GO:0005975,carbohydrate metabolic process|GO:1901565,organonitrogen compound catabolic process|GO:0007034,vacuolar transport|GO:1901136,carbohydrate derivative catabolic process|GO:1901135,carbohydrate derivative metabolic process|GO:0043170,macromolecule metabolic process|GO:0044085,cellular component biogenesis|GO:0046907,intracellular transport|GO:0006022,aminoglycan metabolic process|GO:1902582,single-organism intracellular transport|GO:0006026,aminoglycan catabolic process|GO:0006027,glycosaminoglycan catabolic process|GO:0042119,neutrophil activation|GO:0050896,response to stimulus|GO:0006955,immune response|GO:0045321,leukocyte activation|GO:0032940,secretion by cell|GO:0002446,neutrophil mediated immunity|GO:0002283,neutrophil activation involved in immune response|GO:0002443,leukocyte mediated immunity|GO:0002444,myeloid leukocyte mediated immunity|GO:0045055,regulated exocytosis|GO:0002366,leukocyte activation involved in immune response|GO:0001775,cell activation|GO:0036230,granulocyte activation|GO:0006887,exocytosis|GO:0043299,leukocyte degranulation|GO:0002263,cell activation involved in immune response|GO:0046903,secretion|GO:0002275,myeloid cell activation involved in immune response|GO:0002274,myeloid leukocyte activation|GO:0043312,neutrophil degranulation|GO:0002376,immune system process|GO:0002252,immune effector process|GO:0044763,single-organism cellular process|GO:0016192,vesicle-mediated transport	GO:0000323,lytic vacuole|GO:0043229,intracellular organelle|GO:0043227,membrane-bounded organelle|GO:0043226,organelle|GO:0031224,intrinsic component of membrane|GO:0005737,cytoplasm|GO:0044446,intracellular organelle part|GO:0031090,organelle membrane|GO:0005773,vacuole|GO:0016021,integral component of membrane|GO:0016020,membrane|GO:0005774,vacuolar membrane|GO:0044437,vacuolar part|GO:0098588,bounding membrane of organelle|GO:0043231,intracellular membrane-bounded organelle|GO:0044464,cell part|GO:0005623,cell|GO:0005622,intracellular|GO:0005764,lysosome|GO:0005765,lysosomal membrane|GO:0044444,cytoplasmic part|GO:0044424,intracellular part|GO:0044425,membrane part|GO:0098805,whole membrane|GO:0044422,organelle part|GO:0035579,specific granule membrane|GO:0031988,membrane-bounded vesicle|GO:0070821,tertiary granule membrane|GO:0070820,tertiary granule|GO:0031982,vesicle|GO:0031410,cytoplasmic vesicle|GO:0044433,cytoplasmic vesicle part|GO:0099503,secretory vesicle|GO:0016023,cytoplasmic, membrane-bounded vesicle|GO:0030141,secretory granule|GO:0030667,secretory granule membrane|GO:0042581,specific granule|GO:0030659,cytoplasmic vesicle membrane|GO:0097708,intracellular vesicle|GO:0012506,vesicle membrane|GO:0005886,plasma membrane|GO:0071944,cell periphery|GO:0098852,lytic vacuole membrane|GO:0012505,endomembrane system	GO:0008080,N-acetyltransferase activity|GO:0016740,transferase activity|GO:0003824,catalytic activity|GO:0016407,acetyltransferase activity|GO:0016746,transferase activity, transferring acyl groups|GO:0016747,transferase activity, transferring acyl groups other than amino-acyl groups|GO:0016410,N-acyltransferase activity|GO:0015019,heparan-alpha-glucosaminide N-acetyltransferase activity
MUC3A		GO:0044425,membrane part|GO:0016021,integral component of membrane|GO:0016020,membrane|GO:0031224,intrinsic component of membrane	
ZNF213	GO:0080090,regulation of primary metabolic process|GO:0019222,regulation of metabolic process|GO:0031326,regulation of cellular biosynthetic process|GO:0031323,regulation of cellular metabolic process|GO:0090304,nucleic acid metabolic process|GO:0044249,cellular biosynthetic process|GO:0034641,cellular nitrogen compound metabolic process|GO:0006807,nitrogen compound metabolic process|GO:0034645,cellular macromolecule biosynthetic process|GO:1901362,organic cyclic compound biosynthetic process|GO:0050789,regulation of biological process|GO:0097659,nucleic acid-templated transcription|GO:0032774,RNA biosynthetic process|GO:0006139,nucleobase-containing compound metabolic process|GO:0044260,cellular macromolecule metabolic process|GO:0071704,organic substance metabolic process|GO:2000112,regulation of cellular macromolecule biosynthetic process|GO:0060255,regulation of macromolecule metabolic process|GO:0010467,gene expression|GO:0065007,biological regulation|GO:1901360,organic cyclic compound metabolic process|GO:0010468,regulation of gene expression|GO:0018130,heterocycle biosynthetic process|GO:1901576,organic substance biosynthetic process|GO:0019219,regulation of nucleobase-containing compound metabolic process|GO:0006725,cellular aromatic compound metabolic process|GO:0009987,cellular process|GO:0009889,regulation of biosynthetic process|GO:1903506,regulation of nucleic acid-templated transcription|GO:0050794,regulation of cellular process|GO:0009058,biosynthetic process|GO:0009059,macromolecule biosynthetic process|GO:0051171,regulation of nitrogen compound metabolic process|GO:0008152,metabolic process|GO:2001141,regulation of RNA biosynthetic process|GO:0034654,nucleobase-containing compound biosynthetic process|GO:0046483,heterocycle metabolic process|GO:0016070,RNA metabolic process|GO:0044238,primary metabolic process|GO:0044271,cellular nitrogen compound biosynthetic process|GO:0051252,regulation of RNA metabolic process|GO:0044237,cellular metabolic process|GO:0043170,macromolecule metabolic process|GO:0006355,regulation of transcription, DNA-templated|GO:0010556,regulation of macromolecule biosynthetic process|GO:0006351,transcription, DNA-templated|GO:0019438,aromatic compound biosynthetic process	GO:0043231,intracellular membrane-bounded organelle|GO:0005634,nucleus|GO:0044464,cell part|GO:0005623,cell|GO:0005622,intracellular|GO:0043229,intracellular organelle|GO:0044424,intracellular part|GO:0043227,membrane-bounded organelle|GO:0043226,organelle	GO:0043169,cation binding|GO:0003700,transcription factor activity, sequence-specific DNA binding|GO:0097159,organic cyclic compound binding|GO:0043167,ion binding|GO:0005488,binding|GO:0003676,nucleic acid binding|GO:0003677,DNA binding|GO:0046872,metal ion binding|GO:1901363,heterocyclic compound binding|GO:0001071,nucleic acid binding transcription factor activity|GO:0000981,RNA polymerase II transcription factor activity, sequence-specific DNA binding
KB			
CDK2AP2	GO:0033043,regulation of organelle organization|GO:0051493,regulation of cytoskeleton organization|GO:0000082,G1/S transition of mitotic cell cycle|GO:1903047,mitotic cell cycle process|GO:0051128,regulation of cellular component organization|GO:0044770,cell cycle phase transition|GO:0010948,negative regulation of cell cycle process|GO:0065007,biological regulation|GO:1901991,negative regulation of mitotic cell cycle phase transition|GO:1901990,regulation of mitotic cell cycle phase transition|GO:0051301,cell division|GO:0044843,cell cycle G1/S phase transition|GO:0032886,regulation of microtubule-based process|GO:0051302,regulation of cell division|GO:0071840,cellular component organization or biogenesis|GO:0007346,regulation of mitotic cell cycle|GO:2000134,negative regulation of G1/S transition of mitotic cell cycle|GO:0010564,regulation of cell cycle process|GO:0016043,cellular component organization|GO:0050789,regulation of biological process|GO:2000035,regulation of stem cell division|GO:0007049,cell cycle|GO:0048519,negative regulation of biological process|GO:0045930,negative regulation of mitotic cell cycle|GO:0000278,mitotic cell cycle|GO:0009987,cellular process|GO:0050794,regulation of cellular process|GO:1901987,regulation of cell cycle phase transition|GO:0044763,single-organism cellular process|GO:0045786,negative regulation of cell cycle|GO:1901988,negative regulation of cell cycle phase transition|GO:0006996,organelle organization|GO:0044699,single-organism process|GO:0007017,microtubule-based process|GO:0007010,cytoskeleton organization|GO:2000045,regulation of G1/S transition of mitotic cell cycle|GO:0051726,regulation of cell cycle|GO:0022402,cell cycle process|GO:0044772,mitotic cell cycle phase transition|GO:1902806,regulation of cell cycle G1/S phase transition|GO:1902807,negative regulation of cell cycle G1/S phase transition|GO:0000226,microtubule cytoskeleton organization|GO:0017145,stem cell division|GO:0070507,regulation of microtubule cytoskeleton organization|GO:0048523,negative regulation of cellular process	GO:0005856,cytoskeleton|GO:0005737,cytoplasm|GO:0015630,microtubule cytoskeleton|GO:0099512,supramolecular fiber|GO:0099513,polymeric cytoskeletal fiber|GO:0043232,intracellular non-membrane-bounded organelle|GO:0043227,membrane-bounded organelle|GO:0005634,nucleus|GO:0044464,cell part|GO:0005623,cell|GO:0005622,intracellular|GO:0044446,intracellular organelle part|GO:0043229,intracellular organelle|GO:0044430,cytoskeletal part|GO:0044424,intracellular part|GO:0043228,non-membrane-bounded organelle|GO:0005874,microtubule|GO:0043226,organelle|GO:0044422,organelle part|GO:0043231,intracellular membrane-bounded organelle	GO:0005488,binding|GO:0005515,protein binding
C6orf15			
TUBB1	GO:0022607,cellular component assembly|GO:0006457,protein folding|GO:0070271,protein complex biogenesis|GO:0043933,macromolecular complex subunit organization|GO:0006458,'de novo' protein folding|GO:0000226,microtubule cytoskeleton organization|GO:0034622,cellular macromolecular complex assembly|GO:0051225,spindle assembly|GO:0044699,single-organism process|GO:0044267,cellular protein metabolic process|GO:0071822,protein complex subunit organization|GO:0051084,'de novo' posttranslational protein folding|GO:0071840,cellular component organization or biogenesis|GO:0016043,cellular component organization|GO:0065003,macromolecular complex assembly|GO:0071704,organic substance metabolic process|GO:0007049,cell cycle|GO:0009987,cellular process|GO:0044238,primary metabolic process|GO:0006461,protein complex assembly|GO:0007051,spindle organization|GO:0044763,single-organism cellular process|GO:0008152,metabolic process|GO:0070925,organelle assembly|GO:0043623,cellular protein complex assembly|GO:0006996,organelle organization|GO:0051258,protein polymerization|GO:0007017,microtubule-based process|GO:0007010,cytoskeleton organization|GO:0044260,cellular macromolecule metabolic process|GO:0019538,protein metabolic process|GO:0044237,cellular metabolic process|GO:0043170,macromolecule metabolic process|GO:1902589,single-organism organelle organization|GO:0044085,cellular component biogenesis|GO:0022402,cell cycle process	GO:0043229,intracellular organelle|GO:0043228,non-membrane-bounded organelle|GO:0005874,microtubule|GO:0043226,organelle|GO:0005856,cytoskeleton|GO:0031982,vesicle|GO:0043227,membrane-bounded organelle|GO:0031988,membrane-bounded vesicle|GO:0044430,cytoskeletal part|GO:0005737,cytoplasm|GO:0015630,microtubule cytoskeleton|GO:0032991,macromolecular complex|GO:1903561,extracellular vesicle|GO:0070062,extracellular exosome|GO:0043234,protein complex|GO:0043230,extracellular organelle|GO:0043232,intracellular non-membrane-bounded organelle|GO:0044464,cell part|GO:0005623,cell|GO:0005622,intracellular|GO:0044446,intracellular organelle part|GO:0005576,extracellular region|GO:0044424,intracellular part|GO:0065010,extracellular membrane-bounded organelle|GO:0044421,extracellular region part|GO:0044422,organelle part|GO:0099512,supramolecular fiber|GO:0099513,polymeric cytoskeletal fiber	GO:0005200,structural constituent of cytoskeleton|GO:0035639,purine ribonucleoside triphosphate binding|GO:0005488,binding|GO:0005198,structural molecule activity|GO:1901363,heterocyclic compound binding|GO:1901265,nucleoside phosphate binding|GO:0001882,nucleoside binding|GO:0043168,anion binding|GO:0016462,pyrophosphatase activity|GO:0000166,nucleotide binding|GO:0032561,guanyl ribonucleotide binding|GO:0001883,purine nucleoside binding|GO:0032549,ribonucleoside binding|GO:0017076,purine nucleotide binding|GO:0005525,GTP binding|GO:0016787,hydrolase activity|GO:0003924,GTPase activity|GO:0017111,nucleoside-triphosphatase activity|GO:0036094,small molecule binding|GO:0003824,catalytic activity|GO:0016818,hydrolase activity, acting on acid anhydrides, in phosphorus-containing anhydrides|GO:0043167,ion binding|GO:0097367,carbohydrate derivative binding|GO:0097159,organic cyclic compound binding|GO:0016817,hydrolase activity, acting on acid anhydrides|GO:0019001,guanyl nucleotide binding|GO:0032555,purine ribonucleotide binding|GO:0032550,purine ribonucleoside binding|GO:0032553,ribonucleotide binding
AKTIP	GO:0008104,protein localization|GO:0019220,regulation of phosphate metabolic process|GO:0080090,regulation of primary metabolic process|GO:0019222,regulation of metabolic process|GO:0071840,cellular component organization or biogenesis|GO:0010604,positive regulation of macromolecule metabolic process|GO:0010256,endomembrane system organization|GO:0032446,protein modification by small protein conjugation|GO:0044093,positive regulation of molecular function|GO:0048518,positive regulation of biological process|GO:0033036,macromolecule localization|GO:0042325,regulation of phosphorylation|GO:0060255,regulation of macromolecule metabolic process|GO:0045184,establishment of protein localization|GO:0045022,early endosome to late endosome transport|GO:0070647,protein modification by small protein conjugation or removal|GO:0016197,endosomal transport|GO:0042327,positive regulation of phosphorylation|GO:0016192,vesicle-mediated transport|GO:0019538,protein metabolic process|GO:0016567,protein ubiquitination|GO:0009893,positive regulation of metabolic process|GO:0050789,regulation of biological process|GO:0044267,cellular protein metabolic process|GO:0032092,positive regulation of protein binding|GO:0044260,cellular macromolecule metabolic process|GO:0016043,cellular component organization|GO:0065007,biological regulation|GO:0065009,regulation of molecular function|GO:0016265,obsolete death|GO:0006810,transport|GO:0050794,regulation of cellular process|GO:0012501,programmed cell death|GO:0043412,macromolecule modification|GO:0036211,protein modification process|GO:0043393,regulation of protein binding|GO:0008152,metabolic process|GO:0051234,establishment of localization|GO:0046907,intracellular transport|GO:0031401,positive regulation of protein modification process|GO:0016310,phosphorylation|GO:0008333,endosome to lysosome transport|GO:0007041,lysosomal transport|GO:0007040,lysosome organization|GO:0044699,single-organism process|GO:0010562,positive regulation of phosphorus metabolic process|GO:0051246,regulation of protein metabolic process|GO:0051247,positive regulation of protein metabolic process|GO:0032270,positive regulation of cellular protein metabolic process|GO:0031399,regulation of protein modification process|GO:0009987,cellular process|GO:0016482,cytosolic transport|GO:0007032,endosome organization|GO:0007033,vacuole organization|GO:0032268,regulation of cellular protein metabolic process|GO:0007034,vacuolar transport|GO:0051099,positive regulation of binding|GO:0051098,regulation of binding|GO:0043170,macromolecule metabolic process|GO:0031325,positive regulation of cellular metabolic process|GO:0031323,regulation of cellular metabolic process|GO:0008219,cell death|GO:0071704,organic substance metabolic process|GO:0071702,organic substance transport|GO:0006468,protein phosphorylation|GO:0045937,positive regulation of phosphate metabolic process|GO:0006915,apoptotic process|GO:0006464,cellular protein modification process|GO:0051174,regulation of phosphorus metabolic process|GO:0044765,single-organism transport|GO:0044763,single-organism cellular process|GO:0051649,establishment of localization in cell|GO:0051179,localization|GO:1902578,single-organism localization|GO:0051641,cellular localization|GO:0006996,organelle organization|GO:0044238,primary metabolic process|GO:0044237,cellular metabolic process|GO:0006796,phosphate-containing compound metabolic process|GO:0080171,lytic vacuole organization|GO:0006793,phosphorus metabolic process|GO:0015031,protein transport|GO:1902582,single-organism intracellular transport|GO:0001932,regulation of protein phosphorylation|GO:0001934,positive regulation of protein phosphorylation|GO:0048522,positive regulation of cellular process|GO:1901564,organonitrogen compound metabolic process|GO:0016050,vesicle organization|GO:0006807,nitrogen compound metabolic process|GO:0098927,vesicle-mediated transport between endosomal compartments|GO:0015833,peptide transport|GO:0042886,amide transport|GO:0071705,nitrogen compound transport	GO:0030897,HOPS complex|GO:0031974,membrane-enclosed lumen|GO:0043229,intracellular organelle|GO:0043228,non-membrane-bounded organelle|GO:0043227,membrane-bounded organelle|GO:0043226,organelle|GO:0005737,cytoplasm|GO:0031981,nuclear lumen|GO:0005730,nucleolus|GO:0005634,nucleus|GO:0016020,membrane|GO:0005794,Golgi apparatus|GO:0070013,intracellular organelle lumen|GO:0012505,endomembrane system|GO:0005886,plasma membrane|GO:0043234,protein complex|GO:0032991,macromolecular complex|GO:0043231,intracellular membrane-bounded organelle|GO:0043232,intracellular non-membrane-bounded organelle|GO:0043233,organelle lumen|GO:0044464,cell part|GO:0005623,cell|GO:0005622,intracellular|GO:0044446,intracellular organelle part|GO:0098796,membrane protein complex|GO:0044444,cytoplasmic part|GO:0071944,cell periphery|GO:0044428,nuclear part|GO:0044424,intracellular part|GO:0044425,membrane part|GO:0070695,FHF complex|GO:0044422,organelle part|GO:0005829,cytosol	GO:0019787,ubiquitin-like protein transferase activity|GO:0031625,ubiquitin protein ligase binding|GO:0044389,ubiquitin-like protein ligase binding|GO:0061630,ubiquitin protein ligase activity|GO:0019899,enzyme binding|GO:0003824,catalytic activity|GO:0016740,transferase activity|GO:0005488,binding|GO:0005515,protein binding|GO:0004842,ubiquitin-protein transferase activity|GO:0061659,ubiquitin-like protein ligase activity
ZNF711	GO:0032774,RNA biosynthetic process|GO:0080090,regulation of primary metabolic process|GO:0019222,regulation of metabolic process|GO:0009891,positive regulation of biosynthetic process|GO:0031326,regulation of cellular biosynthetic process|GO:0031325,positive regulation of cellular metabolic process|GO:0051173,positive regulation of nitrogen compound metabolic process|GO:0031323,regulation of cellular metabolic process|GO:0090304,nucleic acid metabolic process|GO:0044249,cellular biosynthetic process|GO:0034641,cellular nitrogen compound metabolic process|GO:0010628,positive regulation of gene expression|GO:0006807,nitrogen compound metabolic process|GO:0034645,cellular macromolecule biosynthetic process|GO:1901362,organic cyclic compound biosynthetic process|GO:0050789,regulation of biological process|GO:0097659,nucleic acid-templated transcription|GO:0009893,positive regulation of metabolic process|GO:0006139,nucleobase-containing compound metabolic process|GO:0010604,positive regulation of macromolecule metabolic process|GO:0044260,cellular macromolecule metabolic process|GO:0071704,organic substance metabolic process|GO:0031328,positive regulation of cellular biosynthetic process|GO:2000112,regulation of cellular macromolecule biosynthetic process|GO:0060255,regulation of macromolecule metabolic process|GO:0010467,gene expression|GO:0065007,biological regulation|GO:1901360,organic cyclic compound metabolic process|GO:0048518,positive regulation of biological process|GO:0010468,regulation of gene expression|GO:0018130,heterocycle biosynthetic process|GO:1903508,positive regulation of nucleic acid-templated transcription|GO:0045935,positive regulation of nucleobase-containing compound metabolic process|GO:1901576,organic substance biosynthetic process|GO:0019219,regulation of nucleobase-containing compound metabolic process|GO:0006725,cellular aromatic compound metabolic process|GO:0009987,cellular process|GO:0009889,regulation of biosynthetic process|GO:1903506,regulation of nucleic acid-templated transcription|GO:0050794,regulation of cellular process|GO:0009058,biosynthetic process|GO:0009059,macromolecule biosynthetic process|GO:0045893,positive regulation of transcription, DNA-templated|GO:0008152,metabolic process|GO:2001141,regulation of RNA biosynthetic process|GO:0034654,nucleobase-containing compound biosynthetic process|GO:0046483,heterocycle metabolic process|GO:0016070,RNA metabolic process|GO:0044238,primary metabolic process|GO:0044271,cellular nitrogen compound biosynthetic process|GO:0051252,regulation of RNA metabolic process|GO:0051254,positive regulation of RNA metabolic process|GO:0044237,cellular metabolic process|GO:0043170,macromolecule metabolic process|GO:1902680,positive regulation of RNA biosynthetic process|GO:0006355,regulation of transcription, DNA-templated|GO:0010557,positive regulation of macromolecule biosynthetic process|GO:0010556,regulation of macromolecule biosynthetic process|GO:0006351,transcription, DNA-templated|GO:0019438,aromatic compound biosynthetic process|GO:0048522,positive regulation of cellular process|GO:0051171,regulation of nitrogen compound metabolic process	GO:0031974,membrane-enclosed lumen|GO:0043229,intracellular organelle|GO:0043227,membrane-bounded organelle|GO:0043226,organelle|GO:0070062,extracellular exosome|GO:0031981,nuclear lumen|GO:0005634,nucleus|GO:0031988,membrane-bounded vesicle|GO:0005654,nucleoplasm|GO:1903561,extracellular vesicle|GO:0031982,vesicle|GO:0043230,extracellular organelle|GO:0043231,intracellular membrane-bounded organelle|GO:0043233,organelle lumen|GO:0044464,cell part|GO:0005623,cell|GO:0005622,intracellular|GO:0044446,intracellular organelle part|GO:0070013,intracellular organelle lumen|GO:0005576,extracellular region|GO:0044428,nuclear part|GO:0044424,intracellular part|GO:0065010,extracellular membrane-bounded organelle|GO:0044421,extracellular region part|GO:0044422,organelle part	GO:0044212,transcription regulatory region DNA binding|GO:0001071,nucleic acid binding transcription factor activity|GO:0005515,protein binding|GO:0043565,sequence-specific DNA binding|GO:0003700,transcription factor activity, sequence-specific DNA binding|GO:0001012,RNA polymerase II regulatory region DNA binding|GO:0003677,DNA binding|GO:0001067,regulatory region nucleic acid binding|GO:0043169,cation binding|GO:0043167,ion binding|GO:0000976,transcription regulatory region sequence-specific DNA binding|GO:0000977,RNA polymerase II regulatory region sequence-specific DNA binding|GO:0003676,nucleic acid binding|GO:0000975,regulatory region DNA binding|GO:0046872,metal ion binding|GO:0097159,organic cyclic compound binding|GO:1901363,heterocyclic compound binding|GO:0005488,binding|GO:0000981,RNA polymerase II transcription factor activity, sequence-specific DNA binding
C2orf88			
TCL1A	GO:0030154,cell differentiation|GO:0048468,cell development|GO:0007275,multicellular organism development|GO:0044699,single-organism process|GO:0048863,stem cell differentiation|GO:0048864,stem cell development|GO:0048869,cellular developmental process|GO:0050789,regulation of biological process|GO:0065007,biological regulation|GO:0048519,negative regulation of biological process|GO:0032502,developmental process|GO:0032501,multicellular organismal process|GO:0050793,regulation of developmental process|GO:0009987,cellular process|GO:0050794,regulation of cellular process|GO:0044767,single-organism developmental process|GO:0098727,maintenance of cell number|GO:0045595,regulation of cell differentiation|GO:0044763,single-organism cellular process|GO:0051093,negative regulation of developmental process|GO:0019827,stem cell population maintenance|GO:0044707,single-multicellular organism process|GO:0045596,negative regulation of cell differentiation|GO:0048856,anatomical structure development|GO:0048523,negative regulation of cellular process|GO:0019220,regulation of phosphate metabolic process|GO:0080090,regulation of primary metabolic process|GO:0019222,regulation of metabolic process|GO:0071840,cellular component organization or biogenesis|GO:0051716,cellular response to stimulus|GO:0010604,positive regulation of macromolecule metabolic process|GO:0071310,cellular response to organic substance|GO:0018193,peptidyl-amino acid modification|GO:0044093,positive regulation of molecular function|GO:0048522,positive regulation of cellular process|GO:0048518,positive regulation of biological process|GO:0042127,regulation of cell proliferation|GO:0060255,regulation of macromolecule metabolic process|GO:0060548,negative regulation of cell death|GO:0045859,regulation of protein kinase activity|GO:1901564,organonitrogen compound metabolic process|GO:0010033,response to organic substance|GO:0042325,regulation of phosphorylation|GO:0042327,positive regulation of phosphorylation|GO:0018209,peptidyl-serine modification|GO:0019538,protein metabolic process|GO:0043069,negative regulation of programmed cell death|GO:0042391,regulation of membrane potential|GO:0022607,cellular component assembly|GO:0009893,positive regulation of metabolic process|GO:0033674,positive regulation of kinase activity|GO:0006807,nitrogen compound metabolic process|GO:0071902,positive regulation of protein serine/threonine kinase activity|GO:0071900,regulation of protein serine/threonine kinase activity|GO:0042981,regulation of apoptotic process|GO:0034097,response to cytokine|GO:0044267,cellular protein metabolic process|GO:0051347,positive regulation of transferase activity|GO:0044260,cellular macromolecule metabolic process|GO:0071356,cellular response to tumor necrosis factor|GO:0043549,regulation of kinase activity|GO:0016043,cellular component organization|GO:0065003,macromolecular complex assembly|GO:0043085,positive regulation of catalytic activity|GO:0065009,regulation of molecular function|GO:0065008,regulation of biological quality|GO:0051130,positive regulation of cellular component organization|GO:0006915,apoptotic process|GO:0070207,protein homotrimerization|GO:0070206,protein trimerization|GO:0050790,regulation of catalytic activity|GO:0012501,programmed cell death|GO:0043412,macromolecule modification|GO:0036211,protein modification process|GO:0008152,metabolic process|GO:0043254,regulation of protein complex assembly|GO:0010918,positive regulation of mitochondrial membrane potential|GO:0071345,cellular response to cytokine stimulus|GO:0050896,response to stimulus|GO:0031401,positive regulation of protein modification process|GO:0051338,regulation of transferase activity|GO:0070271,protein complex biogenesis|GO:0016310,phosphorylation|GO:0051128,regulation of cellular component organization|GO:0070887,cellular response to chemical stimulus|GO:0010562,positive regulation of phosphorus metabolic process|GO:0051246,regulation of protein metabolic process|GO:0051247,positive regulation of protein metabolic process|GO:0032270,positive regulation of cellular protein metabolic process|GO:0031399,regulation of protein modification process|GO:0008284,positive regulation of cell proliferation|GO:0008283,cell proliferation|GO:0031334,positive regulation of protein complex assembly|GO:0045838,positive regulation of membrane potential|GO:0051259,protein oligomerization|GO:0032268,regulation of cellular protein metabolic process|GO:0043170,macromolecule metabolic process|GO:0045860,positive regulation of protein kinase activity|GO:0018105,peptidyl-serine phosphorylation|GO:0033138,positive regulation of peptidyl-serine phosphorylation|GO:0043933,macromolecular complex subunit organization|GO:0033135,regulation of peptidyl-serine phosphorylation|GO:0031325,positive regulation of cellular metabolic process|GO:0031323,regulation of cellular metabolic process|GO:0008219,cell death|GO:0010941,regulation of cell death|GO:0071822,protein complex subunit organization|GO:0051260,protein homooligomerization|GO:0071704,organic substance metabolic process|GO:0043067,regulation of programmed cell death|GO:0043066,negative regulation of apoptotic process|GO:0032461,positive regulation of protein oligomerization|GO:0006468,protein phosphorylation|GO:0045937,positive regulation of phosphate metabolic process|GO:0034612,response to tumor necrosis factor|GO:0006461,protein complex assembly|GO:0006464,cellular protein modification process|GO:0051174,regulation of phosphorus metabolic process|GO:0042221,response to chemical|GO:0044238,primary metabolic process|GO:0051881,regulation of mitochondrial membrane potential|GO:0044237,cellular metabolic process|GO:0044087,regulation of cellular component biogenesis|GO:0006796,phosphate-containing compound metabolic process|GO:0044085,cellular component biogenesis|GO:0006793,phosphorus metabolic process|GO:0032459,regulation of protein oligomerization|GO:0001932,regulation of protein phosphorylation|GO:0001934,positive regulation of protein phosphorylation|GO:0044089,positive regulation of cellular component	GO:0005737,cytoplasm|GO:0012505,endomembrane system|GO:0005783,endoplasmic reticulum|GO:0045120,pronucleus|GO:0044464,cell part|GO:0044444,cytoplasmic part|GO:0005623,cell|GO:0043226,organelle|GO:0043229,intracellular organelle|GO:0071944,cell periphery|GO:0005938,cell cortex|GO:0044424,intracellular part|GO:0005622,intracellular|GO:0043227,membrane-bounded organelle|GO:0005634,nucleus|GO:0043231,intracellular membrane-bounded organelle|GO:0043234,protein complex|GO:0032991,macromolecular complex	GO:0005488,binding|GO:0005515,protein binding|GO:0019207,kinase regulator activity|GO:0019887,protein kinase regulator activity|GO:0030234,enzyme regulator activity|GO:0019209,kinase activator activity|GO:0019899,enzyme binding|GO:0019901,protein kinase binding|GO:0019900,kinase binding|GO:0042802,identical protein binding|GO:0008047,enzyme activator activity|GO:0098772,molecular function regulator|GO:0030295,protein kinase activator activity|GO:0043539,protein serine/threonine kinase activator activity
ZNF225	GO:0051716,cellular response to stimulus|GO:0033233,regulation of protein sumoylation|GO:0071455,cellular response to hyperoxia|GO:0060548,negative regulation of cell death|GO:0046483,heterocycle metabolic process|GO:0071593,lymphocyte aggregation|GO:0009605,response to external stimulus|GO:0034284,response to monosaccharide|GO:0019538,protein metabolic process|GO:0072359,circulatory system development|GO:0051253,negative regulation of RNA metabolic process|GO:0009892,negative regulation of metabolic process|GO:0009893,positive regulation of metabolic process|GO:0009890,negative regulation of biosynthetic process|GO:0009891,positive regulation of biosynthetic process|GO:1901342,regulation of vasculature development|GO:0071867,response to monoamine|GO:0071869,response to catecholamine|GO:0050789,regulation of biological process|GO:0018130,heterocycle biosynthetic process|GO:0098602,single organism cell adhesion|GO:0007623,circadian rhythm|GO:0098609,cell-cell adhesion|GO:0048709,oligodendrocyte differentiation|GO:0090193,positive regulation of glomerulus development|GO:0090192,regulation of glomerulus development|GO:0043412,macromolecule modification|GO:0002521,leukocyte differentiation|GO:0002520,immune system development|GO:0016070,RNA metabolic process|GO:0072301,regulation of metanephric glomerular mesangial cell proliferation|GO:0071345,cellular response to cytokine stimulus|GO:0071347,cellular response to interleukin-1|GO:0001975,response to amphetamine|GO:0010557,positive regulation of macromolecule biosynthetic process|GO:0010556,regulation of macromolecule biosynthetic process|GO:0097305,response to alcohol|GO:0010558,negative regulation of macromolecule biosynthetic process|GO:0051246,regulation of protein metabolic process|GO:0010001,glial cell differentiation|GO:0014074,response to purine-containing compound|GO:0014075,response to amine|GO:0014072,response to isoquinoline alkaloid|GO:0014070,response to organic cyclic compound|GO:1904018,positive regulation of vasculature development|GO:0032446,protein modification by small protein conjugation|GO:0008284,positive regulation of cell proliferation|GO:0008283,cell proliferation|GO:0050877,neurological system process|GO:0034698,response to gonadotropin|GO:0001655,urogenital system development|GO:0001656,metanephros development|GO:0072298,regulation of metanephric glomerulus development|GO:0000122,negative regulation of transcription from RNA polymerase II promoter|GO:0097237,cellular response to toxic substance|GO:0035690,cellular response to drug|GO:0070498,interleukin-1-mediated signaling pathway|GO:0008219,cell death|GO:0055093,response to hyperoxia|GO:0007275,multicellular organism development|GO:0009888,tissue development|GO:0032354,response to follicle-stimulating hormone|GO:0033993,response to lipid|GO:0072203,cell proliferation involved in metanephros development|GO:0009636,response to toxic substance|GO:2000113,negative regulation of cellular macromolecule biosynthetic process|GO:0043065,positive regulation of apoptotic process|GO:0043067,regulation of programmed cell death|GO:0043066,negative regulation of apoptotic process|GO:0043069,negative regulation of programmed cell death|GO:0043068,positive regulation of programmed cell death|GO:0090090,negative regulation of canonical Wnt signaling pathway|GO:0019219,regulation of nucleobase-containing compound metabolic process|GO:0045087,innate immune response|GO:0006464,cellular protein modification process|GO:0044767,single-organism developmental process|GO:0044763,single-organism cellular process|GO:0060337,type I interferon signaling pathway|GO:1901700,response to oxygen-containing compound|GO:1901701,cellular response to oxygen-containing compound|GO:0048856,anatomical structure development|GO:2000026,regulation of multicellular organismal development|GO:0034465,response to carbon monoxide|GO:0048523,negative regulation of cellular process|GO:0048522,positive regulation of cellular process|GO:2000112,regulation of cellular macromolecule biosynthetic process|GO:0007610,behavior|GO:0007611,learning or memory|GO:0043523,regulation of neuron apoptotic process|GO:0007165,signal transduction|GO:0007166,cell surface receptor signaling pathway|GO:0007167,enzyme linked receptor protein signaling pathway|GO:0072111,cell proliferation involved in kidney development|GO:0072110,glomerular mesangial cell proliferation|GO:0070848,response to growth factor|GO:0007178,transmembrane receptor protein serine/threonine kinase signaling pathway|GO:0030217,T cell differentiation|GO:2001141,regulation of RNA biosynthetic process|GO:0010033,response to organic substance|GO:0044249,cellular biosynthetic process|GO:0018205,peptidyl-lysine modification|GO:0031667,response to nutrient levels|GO:0097285,obsolete cell-type specific apoptotic process|GO:0071320,cellular response to cAMP|GO:0045321,leukocyte activation|GO:0010629,negative regulation of gene expression|GO:0010628,positive regulation of gene expression|GO:0061418,regulation of transcription from RNA polymerase II promoter in response to hypoxia|GO:0044267,cellular protein metabolic process|GO:0010646,regulation of cell communication|GO:0044260,cellular macromolecule metabolic process|GO:0070997,neuron death|GO:0001568,blood vessel development|GO:0006366,transcription from RNA polymerase II promoter|GO:0050793,regulation of developmental process|GO:0009889,regulation of biosynthetic process|GO:0071479,cellular response to ionizing radiation|GO:0050794,regulation of cellular process|GO:0072300,positive regulation of metanephric glomerulus development|GO:0060070,canonical Wnt signaling pathway|GO:0072303,positive regulation of glomerular metanephric mesangial cell proliferation|GO:0071873,response to norepinephrine|GO:0051239,regulation of multicellular organismal process|GO:1901216,positive regulation of neuron death|GO:1901214,regulation of neuron death|GO:0071506,cellular response to mycophenolic acid|GO:0071505,response to mycophenolic acid|GO:0071504,cellular response to heparin|GO:0071503,response to heparin|GO:0050896,response to stimulus|GO:0050890,cognition|GO:0071480,cellular response to gamma radiation|GO:0009314,response to radiation|GO:0070887,cellular response to chemical stimulus|GO:0044699,single-organism process|GO:0071371,cellular response to gonadotropin stimulus|GO:0071372,cellular response to follicle-stimulating hormone stimulus|GO:0071478,cellular response to radiation|GO:0010212,response to ionizing radiation|GO:0071375,cellular response to peptide hormone stimulus|GO:0072012,glomerulus vasculature development|GO:0051240,positive regulation of multicellular organismal process|GO:0001944,vasculature development|GO:0031399,regulation of protein modification process|GO:0043620,regulation of DNA-templated transcription in response to stress|GO:0071396,cellular response to lipid|GO:0043279,response to alkaloid|GO:0060291,long-term synaptic potentiation|GO:0042493,response to drug|GO:1902680,positive regulation of RNA biosynthetic process|GO:0048731,system development|GO:0016337,single organismal cell-cell adhesion|GO:0014706,striated muscle tissue development|GO:0048169,regulation of long-term neuronal synaptic plasticity|GO:0030178,negative regulation of Wnt signaling pathway|GO:0045935,positive regulation of nucleobase-containing compound metabolic process|GO:0045934,negative regulation of nucleobase-containing compound metabolic process|GO:0045471,response to ethanol|GO:0070489,T cell aggregation|GO:0007268,chemical synaptic transmission|GO:0042220,response to cocaine|GO:0042221,response to chemical|GO:0022008,neurogenesis|GO:0070486,leukocyte aggregation|GO:0070482,response to oxygen levels|GO:0009746,response to hexose|GO:0044238,primary metabolic process|GO:0072239,metanephric glomerulus vasculature development|GO:0044237,cellular metabolic process|GO:0009749,response to glucose|GO:0007267,cell-cell signaling|GO:0060537,muscle tissue development|GO:0060538,skeletal muscle organ development|GO:0001101,response to acid chemical|GO:0019221,cytokine-mediated signaling pathway|GO:0019222,regulation of metabolic process|GO:0048585,negative regulation of response to stimulus|GO:0048583,regulation of response to stimulus|GO:0030509,BMP signaling pathway|GO:0030111,regulation of Wnt signaling pathway|GO:0072358,cardiovascular system development|GO:1901362,organic cyclic compound biosynthetic process|GO:1901360,organic cyclic compound metabolic process|GO:0009968,negative regulation of signal	GO:0005737,cytoplasm|GO:0044446,intracellular organelle part|GO:0043231,intracellular membrane-bounded organelle|GO:0031981,nuclear lumen|GO:0043233,organelle lumen|GO:0005634,nucleus|GO:0044464,cell part|GO:0031974,membrane-enclosed lumen|GO:0005622,intracellular|GO:0005654,nucleoplasm|GO:0070013,intracellular organelle lumen|GO:0043229,intracellular organelle|GO:0044428,nuclear part|GO:0005623,cell|GO:0044424,intracellular part|GO:0043227,membrane-bounded organelle|GO:0043226,organelle|GO:0044422,organelle part	GO:0001012,RNA polymerase II regulatory region DNA binding|GO:0019899,enzyme binding|GO:0001077,transcriptional activator activity, RNA polymerase II core promoter proximal region sequence-specific binding|GO:0005488,binding|GO:0003676,nucleic acid binding|GO:0003677,DNA binding|GO:1901363,heterocyclic compound binding|GO:0043565,sequence-specific DNA binding|GO:0043566,structure-specific DNA binding|GO:0043167,ion binding|GO:0003690,double-stranded DNA binding|GO:0046872,metal ion binding|GO:0000982,transcription factor activity, RNA polymerase II core promoter proximal region sequence-specific binding|GO:0000981,RNA polymerase II transcription factor activity, sequence-specific DNA binding|GO:0043169,cation binding|GO:0008134,transcription factor binding|GO:0001067,regulatory region nucleic acid binding|GO:0035035,histone acetyltransferase binding|GO:0001047,core promoter binding|GO:0001046,core promoter sequence-specific DNA binding|GO:0044212,transcription regulatory region DNA binding|GO:0003700,transcription factor activity, sequence-specific DNA binding|GO:0097159,organic cyclic compound binding|GO:0001228,transcriptional activator activity, RNA polymerase II transcription regulatory region sequence-specific binding|GO:0000976,transcription regulatory region sequence-specific DNA binding|GO:0000977,RNA polymerase II regulatory region sequence-specific DNA binding|GO:0005515,protein binding|GO:0000975,regulatory region DNA binding|GO:0000979,RNA polymerase II core promoter sequence-specific DNA binding|GO:0001071,nucleic acid binding transcription factor activity|GO:0008270,zinc ion binding|GO:1990837,sequence-specific double-stranded DNA binding|GO:0046914,transition metal ion binding|GO:0010385,double-stranded methylated DNA binding|GO:0044729,hemi-methylated DNA-binding|GO:1990841,promoter-specific chromatin binding|GO:0003682,chromatin binding
ZNF728	GO:0080090,regulation of primary metabolic process|GO:0019222,regulation of metabolic process|GO:0031326,regulation of cellular biosynthetic process|GO:0031323,regulation of cellular metabolic process|GO:0090304,nucleic acid metabolic process|GO:0044249,cellular biosynthetic process|GO:0034641,cellular nitrogen compound metabolic process|GO:0006807,nitrogen compound metabolic process|GO:0034645,cellular macromolecule biosynthetic process|GO:1901362,organic cyclic compound biosynthetic process|GO:0050789,regulation of biological process|GO:0097659,nucleic acid-templated transcription|GO:0032774,RNA biosynthetic process|GO:0006139,nucleobase-containing compound metabolic process|GO:0044260,cellular macromolecule metabolic process|GO:0071704,organic substance metabolic process|GO:2000112,regulation of cellular macromolecule biosynthetic process|GO:0060255,regulation of macromolecule metabolic process|GO:0010467,gene expression|GO:0065007,biological regulation|GO:1901360,organic cyclic compound metabolic process|GO:0010468,regulation of gene expression|GO:0018130,heterocycle biosynthetic process|GO:1901576,organic substance biosynthetic process|GO:0019219,regulation of nucleobase-containing compound metabolic process|GO:0006725,cellular aromatic compound metabolic process|GO:0009987,cellular process|GO:0009889,regulation of biosynthetic process|GO:1903506,regulation of nucleic acid-templated transcription|GO:0050794,regulation of cellular process|GO:0009058,biosynthetic process|GO:0009059,macromolecule biosynthetic process|GO:0051171,regulation of nitrogen compound metabolic process|GO:0008152,metabolic process|GO:2001141,regulation of RNA biosynthetic process|GO:0034654,nucleobase-containing compound biosynthetic process|GO:0046483,heterocycle metabolic process|GO:0016070,RNA metabolic process|GO:0044238,primary metabolic process|GO:0044271,cellular nitrogen compound biosynthetic process|GO:0051252,regulation of RNA metabolic process|GO:0044237,cellular metabolic process|GO:0043170,macromolecule metabolic process|GO:0006355,regulation of transcription, DNA-templated|GO:0010556,regulation of macromolecule biosynthetic process|GO:0006351,transcription, DNA-templated|GO:0019438,aromatic compound biosynthetic process	GO:0044464,cell part|GO:0005623,cell|GO:0005622,intracellular	GO:0043169,cation binding|GO:0097159,organic cyclic compound binding|GO:0043167,ion binding|GO:0005488,binding|GO:0003676,nucleic acid binding|GO:0046872,metal ion binding|GO:1901363,heterocyclic compound binding|GO:0003700,transcription factor activity, sequence-specific DNA binding|GO:0001071,nucleic acid binding transcription factor activity
TAPBPL	GO:0002474,antigen processing and presentation of peptide antigen via MHC class I|GO:0019882,antigen processing and presentation|GO:0002589,regulation of antigen processing and presentation of peptide antigen via MHC class I|GO:0002584,negative regulation of antigen processing and presentation of peptide antigen|GO:0002577,regulation of antigen processing and presentation|GO:0002583,regulation of antigen processing and presentation of peptide antigen|GO:0002376,immune system process|GO:0065007,biological regulation|GO:0002682,regulation of immune system process|GO:0002590,negative regulation of antigen processing and presentation of peptide antigen via MHC class I|GO:0048519,negative regulation of biological process|GO:0048002,antigen processing and presentation of peptide antigen|GO:0002578,negative regulation of antigen processing and presentation|GO:0050789,regulation of biological process|GO:0002683,negative regulation of immune system process	GO:0005783,endoplasmic reticulum|GO:0005789,endoplasmic reticulum membrane|GO:0042175,nuclear outer membrane-endoplasmic reticulum membrane network|GO:0043229,intracellular organelle|GO:0071944,cell periphery|GO:0005622,intracellular|GO:0043227,membrane-bounded organelle|GO:0043226,organelle|GO:0031224,intrinsic component of membrane|GO:0005737,cytoplasm|GO:0031090,organelle membrane|GO:0016021,integral component of membrane|GO:0016020,membrane|GO:0044432,endoplasmic reticulum part|GO:0044431,Golgi apparatus part|GO:0005794,Golgi apparatus|GO:0098588,bounding membrane of organelle|GO:0012505,endomembrane system|GO:0005886,plasma membrane|GO:0043231,intracellular membrane-bounded organelle|GO:0044464,cell part|GO:0005623,cell|GO:0000139,Golgi membrane|GO:0044446,intracellular organelle part|GO:0044444,cytoplasmic part|GO:0044424,intracellular part|GO:0044425,membrane part|GO:0044422,organelle part|GO:0031984,organelle subcompartment|GO:0098827,endoplasmic reticulum subcompartment|GO:0098791,Golgi subcompartment	GO:0032403,protein complex binding|GO:0044877,macromolecular complex binding|GO:0005488,binding|GO:0005515,protein binding

ARL1	GO:0060193,positive regulation of lipase activity|GO:0060191,regulation of lipase activity|GO:0051234,establishment of localization|GO:0009893,positive regulation of metabolic process|GO:0019222,regulation of metabolic process|GO:0034067,protein localization to Golgi apparatus|GO:0006810,transport|GO:0008152,metabolic process|GO:0008104,protein localization|GO:0023052,signaling|GO:0007165,signal transduction|GO:0051641,cellular localization|GO:0035556,intracellular signal transduction|GO:0050789,regulation of biological process|GO:0043085,positive regulation of catalytic activity|GO:0044710,single-organism metabolic process|GO:0007264,small GTPase mediated signal transduction|GO:0051345,positive regulation of hydrolase activity|GO:0070727,cellular macromolecule localization|GO:0071840,cellular component organization or biogenesis|GO:0010256,endomembrane system organization|GO:0016043,cellular component organization|GO:0065007,biological regulation|GO:0044699,single-organism process|GO:0044093,positive regulation of molecular function|GO:0048518,positive regulation of biological process|GO:0065009,regulation of molecular function|GO:0033036,macromolecule localization|GO:0034613,cellular protein localization|GO:0016197,endosomal transport|GO:0019748,secondary metabolic process|GO:0048193,Golgi vesicle transport|GO:0050790,regulation of catalytic activity|GO:0051716,cellular response to stimulus|GO:0031584,activation of phospholipase D activity|GO:0044765,single-organism transport|GO:0044763,single-organism cellular process|GO:0009404,toxin metabolic process|GO:0042147,retrograde transport, endosome to Golgi|GO:0051649,establishment of localization in cell|GO:0007154,cell communication|GO:0050794,regulation of cellular process|GO:0051179,localization|GO:1902578,single-organism localization|GO:0016192,vesicle-mediated transport|GO:0051336,regulation of hydrolase activity|GO:0044700,single organism signaling|GO:0007030,Golgi organization|GO:0006996,organelle organization|GO:0046907,intracellular transport|GO:0050896,response to stimulus|GO:0044237,cellular metabolic process|GO:0010518,positive regulation of phospholipase activity|GO:0033365,protein localization to organelle|GO:1902582,single-organism intracellular transport|GO:0009987,cellular process|GO:0010517,regulation of phospholipase activity|GO:0016482,cytosolic transport	GO:0043229,intracellular organelle|GO:0005622,intracellular|GO:0043227,membrane-bounded organelle|GO:0043226,organelle|GO:0005737,cytoplasm|GO:0031984,organelle subcompartment|GO:0031982,vesicle|GO:0016020,membrane|GO:0031988,membrane-bounded vesicle|GO:0044431,Golgi apparatus part|GO:0005794,Golgi apparatus|GO:0098588,bounding membrane of organelle|GO:0012505,endomembrane system|GO:1903561,extracellular vesicle|GO:0031090,organelle membrane|GO:0043230,extracellular organelle|GO:0043231,intracellular membrane-bounded organelle|GO:0070062,extracellular exosome|GO:0005802,trans-Golgi network|GO:0044464,cell part|GO:0005623,cell|GO:0000139,Golgi membrane|GO:0044446,intracellular organelle part|GO:0044444,cytoplasmic part|GO:0005576,extracellular region|GO:0098791,Golgi subcompartment|GO:0044424,intracellular part|GO:0065010,extracellular membrane-bounded organelle|GO:0044421,extracellular region part|GO:0044422,organelle part|GO:0032588,trans-Golgi network membrane|GO:0005829,cytosol	GO:0035639,purine ribonucleoside triphosphate binding|GO:0008047,enzyme activator activity|GO:0098772,molecular function regulator|GO:1901363,heterocyclic compound binding|GO:0001883,purine nucleoside binding|GO:0001882,nucleoside binding|GO:0043168,anion binding|GO:0043169,cation binding|GO:0016462,pyrophosphatase activity|GO:0000166,nucleotide binding|GO:0032561,guanyl ribonucleotide binding|GO:1901265,nucleoside phosphate binding|GO:0032549,ribonucleoside binding|GO:0017076,purine nucleotide binding|GO:0046872,metal ion binding|GO:0005525,GTP binding|GO:0016787,hydrolase activity|GO:0030234,enzyme regulator activity|GO:0003924,GTPase activity|GO:0017111,nucleoside-triphosphatase activity|GO:0036094,small molecule binding|GO:0003824,catalytic activity|GO:0019904,protein domain specific binding|GO:0016818,hydrolase activity, acting on acid anhydrides, in phosphorus-containing anhydrides|GO:0043167,ion binding|GO:0097367,carbohydrate derivative binding|GO:0097159,organic cyclic compound binding|GO:0016817,hydrolase activity, acting on acid anhydrides|GO:0019001,guanyl nucleotide binding|GO:0005515,protein binding|GO:0032555,purine ribonucleotide binding|GO:0032550,purine
RAB7L1	GO:0033157,regulation of intracellular protein transport|GO:0008104,protein localization|GO:0032388,positive regulation of intracellular transport|GO:0060341,regulation of cellular localization|GO:0051049,regulation of transport|GO:0032386,regulation of intracellular transport|GO:0030154,cell differentiation|GO:1903827,regulation of cellular protein localization|GO:0023052,signaling|GO:0007165,signal transduction|GO:0051641,cellular localization|GO:0051234,establishment of localization|GO:0051222,positive regulation of protein transport|GO:0051223,regulation of protein transport|GO:0035556,intracellular signal transduction|GO:1903829,positive regulation of cellular protein localization|GO:0032880,regulation of protein localization|GO:0051716,cellular response to stimulus|GO:0032879,regulation of localization|GO:0006888,ER to Golgi vesicle-mediated transport|GO:0070727,cellular macromolecule localization|GO:0048869,cellular developmental process|GO:0071840,cellular component organization or biogenesis|GO:0048193,Golgi vesicle transport|GO:0010256,endomembrane system organization|GO:0016043,cellular component organization|GO:0050789,regulation of biological process|GO:0046907,intracellular transport|GO:0033059,cellular pigmentation|GO:0065007,biological regulation|GO:0044699,single-organism process|GO:0048753,pigment granule organization|GO:0048518,positive regulation of biological process|GO:0006892,post-Golgi vesicle-mediated transport|GO:0032438,melanosome organization|GO:0034613,cellular protein localization|GO:0043473,pigmentation|GO:0032502,developmental process|GO:0006996,organelle organization|GO:0070201,regulation of establishment of protein localization|GO:0006810,transport|GO:0051050,positive regulation of transport|GO:0045184,establishment of protein localization|GO:0009987,cellular process|GO:0090316,positive regulation of intracellular protein transport|GO:0050794,regulation of cellular process|GO:0044767,single-organism developmental process|GO:0032482,Rab protein signal transduction|GO:0044765,single-organism transport|GO:0035526,retrograde transport, plasma membrane to Golgi|GO:0044763,single-organism cellular process|GO:0071702,organic substance transport|GO:0008152,metabolic process|GO:0051649,establishment of localization in cell|GO:0007154,cell communication|GO:0007265,Ras protein signal transduction|GO:0007264,small GTPase mediated signal transduction|GO:0051179,localization|GO:1902578,single-organism localization|GO:0016482,cytosolic transport|GO:0016197,endosomal transport|GO:0044700,single organism signaling|GO:0007030,Golgi organization|GO:0033036,macromolecule localization|GO:0016192,vesicle-mediated transport|GO:0006895,Golgi to endosome transport|GO:0050896,response to stimulus|GO:0016050,vesicle organization|GO:0015031,protein transport|GO:1902582,single-organism intracellular transport|GO:0006886,intracellular protein transport|GO:0019222,regulation of metabolic process|GO:0048584,positive regulation of response to stimulus|GO:0048583,regulation of response to stimulus|GO:0061024,membrane organization|GO:0007009,plasma membrane organization|GO:0007166,cell surface receptor signaling pathway|GO:0030182,neuron differentiation|GO:0031345,negative regulation of cell projection organization|GO:0031344,regulation of cell projection organization|GO:0007005,mitochondrion organization|GO:0010604,positive regulation of macromolecule metabolic process|GO:0043207,response to external biotic stimulus|GO:0009966,regulation of signal transduction|GO:0009967,positive regulation of signal transduction|GO:0009617,response to bacterium|GO:0045665,negative regulation of neuron differentiation|GO:0045664,regulation of neuron differentiation|GO:0019058,viral life cycle|GO:1990778,protein localization to cell periphery|GO:0010721,negative regulation of cell development|GO:0048519,negative regulation of biological process|GO:1903441,protein localization to ciliary membrane|GO:0060255,regulation of macromolecule metabolic process|GO:0048468,cell development|GO:0042147,retrograde transport, endosome to Golgi|GO:0046649,lymphocyte activation|GO:0010977,negative regulation of neuron projection development|GO:0072657,protein localization to membrane|GO:0010975,regulation of neuron projection development|GO:0072659,protein localization to plasma membrane|GO:0051707,response to other organism|GO:0051704,multi-organism process|GO:0002429,immune response-activating cell surface receptor signaling pathway|GO:0009607,response to biotic stimulus|GO:0044707,single-multicellular organism process|GO:0039694,viral RNA genome replication|GO:0002376,immune system process|GO:0001919,regulation of receptor recycling|GO:0022607,cellular component assembly|GO:0009893,positive regulation of metabolic process|GO:0045321,leukocyte activation|GO:0031175,neuron projection development|GO:0010646,regulation of cell communication|GO:0002764,immune response-regulating signaling pathway|GO:0044260,cellular macromolecule metabolic process|GO:0070997,neuron death|GO:0002768,immune response-regulating cell surface receptor signaling pathway|GO:0002684,positive regulation of immune system process|GO:0002682,regulation of immune system process|GO:0007416,synapse assembly|GO:0044419,interspecies interaction between organisms|GO:0050793,regulation of developmental process|GO:0001775,cell activation|GO:0044802,single-organism membrane organization|GO:0051239,regulation of multicellular organismal process|GO:0006955,immune response|GO:1901214,regulation of neuron death|GO:0002757,immune response-activating signal transduction|GO:0051961,negative regulation of nervous system development|GO:0051960,regulation of nervous system development|GO:0050808,synapse organization|GO:0051129,negative regulation of cellular component organization|GO:0051128,regulation of cellular component organization|GO:0023056,positive regulation of signaling|GO:0015833,peptide transport|GO:0060284,regulation of cell development|GO:0023051,regulation of signaling|GO:0010647,positive regulation of cell communication|GO:1904951,positive regulation of establishment of protein localization|GO:0050767,regulation of neurogenesis|GO:0042886,amide transport|GO:0051241,negative regulation of multicellular organismal process|GO:0050768,negative regulation of neurogenesis|GO:0032501,multicellular organismal process|GO:0019079,viral genome replication|GO:0045596,negative regulation of cell differentiation|GO:0045595,regulation of cell differentiation|GO:0051093,negative regulation of developmental process|GO:0050776,regulation of immune response|GO:0050778,positive regulation of immune response|GO:0043170,macromolecule metabolic process|GO:0033365,protein localization to organelle|GO:0048731,system development|GO:0030030,cell projection organization|GO:0031325,positive regulation of cellular metabolic process|GO:0031323,regulation of cellular metabolic process|GO:0042110,T cell activation|GO:0050862,positive regulation of T cell receptor signaling pathway|GO:0061512,protein localization to cilium|GO:0008219,cell death|GO:0010941,regulation of cell death|GO:0001881,receptor recycling|GO:0007275,multicellular organism development|GO:0071705,nitrogen compound transport|GO:0071704,organic substance metabolic process|GO:0001921,positive regulation of receptor recycling|GO:0009605,response to external stimulus|GO:0048666,neuron development|GO:0050851,antigen receptor-mediated signaling pathway|GO:0050852,T cell receptor signaling pathway|GO:0050854,regulation of antigen receptor-mediated signaling pathway|GO:0016032,viral process|GO:0050856,regulation of T cell receptor signaling pathway|GO:0050857,positive regulation of antigen receptor-mediated signaling pathway|GO:0022008,neurogenesis|GO:0048699,generation of neurons|GO:0043112,receptor metabolic process|GO:0007399,nervous system development|GO:0048856,anatomical structure development|GO:0044237,cellular metabolic process|GO:0044085,cellular component biogenesis|GO:2000026,regulation of	GO:0048770,pigment granule|GO:0043229,intracellular organelle|GO:0043228,non-membrane-bounded organelle|GO:0005622,intracellular|GO:0043227,membrane-bounded organelle|GO:0043226,organelle|GO:0005856,cytoskeleton|GO:0031984,organelle subcompartment|GO:0070062,extracellular exosome|GO:0016023,cytoplasmic, membrane-bounded vesicle|GO:0031410,cytoplasmic vesicle|GO:0016020,membrane|GO:0005739,mitochondrion|GO:0031988,membrane-bounded vesicle|GO:0044431,Golgi apparatus part|GO:0005576,extracellular region|GO:0048471,perinuclear region of cytoplasm|GO:0005794,Golgi apparatus|GO:0012505,endomembrane system|GO:0005886,plasma membrane|GO:1903561,extracellular vesicle|GO:0031982,vesicle|GO:0042470,melanosome|GO:0043230,extracellular organelle|GO:0043231,intracellular membrane-bounded organelle|GO:0043232,intracellular non-membrane-bounded organelle|GO:0005802,trans-Golgi network|GO:0044464,cell part|GO:0005623,cell|GO:0005737,cytoplasm|GO:0044446,intracellular organelle part|GO:0005773,vacuole|GO:0044444,cytoplasmic part|GO:0071944,cell periphery|GO:0098791,Golgi subcompartment|GO:0044424,intracellular part|GO:0065010,extracellular membrane-bounded organelle|GO:0044421,extracellular region part|GO:0044422,organelle part|GO:0020003,symbiont-containing vacuole|GO:0055037,recycling endosome|GO:0044216,other organism cell|GO:0043656,intracellular region of host|GO:0043657,host cell|GO:0033643,host cell part|GO:0044215,other organism|GO:0033655,host cell cytoplasm part|GO:0033646,host intracellular part|GO:0030430,host cell cytoplasm|GO:0097708,intracellular vesicle|GO:0044217,other organism part|GO:0018995,host|GO:0005801,cis-Golgi network|GO:0005829,cytosol|GO:0005768,endosome|GO:0005769,early endosome	GO:0035639,purine ribonucleoside triphosphate binding|GO:0005488,binding|GO:0043167,ion binding|GO:1901363,heterocyclic compound binding|GO:1901265,nucleoside phosphate binding|GO:0001882,nucleoside binding|GO:0043168,anion binding|GO:0016462,pyrophosphatase activity|GO:0000166,nucleotide binding|GO:0032561,guanyl ribonucleotide binding|GO:0001883,purine nucleoside binding|GO:0032549,ribonucleoside binding|GO:0017076,purine nucleotide binding|GO:0005525,GTP binding|GO:0016787,hydrolase activity|GO:0003924,GTPase activity|GO:0017111,nucleoside-triphosphatase activity|GO:0036094,small molecule binding|GO:0003824,catalytic activity|GO:0016818,hydrolase activity, acting on acid anhydrides, in phosphorus-containing anhydrides|GO:0097367,carbohydrate derivative binding|GO:0097159,organic cyclic compound binding|GO:0016817,hydrolase activity, acting on acid anhydrides|GO:0019001,guanyl nucleotide binding|GO:0005515,protein binding|GO:0032555,purine ribonucleotide binding|GO:0032550,purine ribonucleoside binding|GO:0032553,ribonucleotide binding|GO:0019894,kinesin binding|GO:0019899,enzyme binding|GO:0031267,small GTPase binding|GO:0051020,GTPase binding|GO:0044877,macromolecular complex binding|GO:0070840,dynein complex binding|GO:0032403,protein complex binding|GO:0017137,Rab GTPase binding|GO:0017016,Ras GTPase binding|GO:0008092,cytoskeletal protein binding|GO:0019003,GDP binding
COQ4	GO:0006732,coenzyme metabolic process|GO:0006733,oxidoreduction coenzyme metabolic process|GO:0044249,cellular biosynthetic process|GO:0009108,coenzyme biosynthetic process|GO:0044281,small molecule metabolic process|GO:0044283,small molecule biosynthetic process|GO:0044699,single-organism process|GO:1901576,organic substance biosynthetic process|GO:0044710,single-organism metabolic process|GO:0044711,single-organism biosynthetic process|GO:1901663,quinone biosynthetic process|GO:0051188,cofactor biosynthetic process|GO:1901661,quinone metabolic process|GO:0071704,organic substance metabolic process|GO:0006744,ubiquinone biosynthetic process|GO:0006743,ubiquinone metabolic process|GO:0009987,cellular process|GO:0009058,biosynthetic process|GO:0051186,cofactor metabolic process|GO:0044763,single-organism cellular process|GO:0008152,metabolic process|GO:0042181,ketone biosynthetic process|GO:0042180,cellular ketone metabolic process|GO:0044237,cellular metabolic process	GO:0044464,cell part|GO:0031975,envelope|GO:0043229,intracellular organelle|GO:0043227,membrane-bounded organelle|GO:0043226,organelle|GO:0005737,cytoplasm|GO:0031090,organelle membrane|GO:0016020,membrane|GO:0005740,mitochondrial envelope|GO:0005739,mitochondrion|GO:0044455,mitochondrial membrane part|GO:0031967,organelle envelope|GO:0031966,mitochondrial membrane|GO:0031314,extrinsic component of mitochondrial inner membrane|GO:0031312,extrinsic component of organelle membrane|GO:0019898,extrinsic component of membrane|GO:0043231,intracellular membrane-bounded organelle|GO:0019866,organelle inner membrane|GO:0005623,cell|GO:0005622,intracellular|GO:0044446,intracellular organelle part|GO:0005743,mitochondrial inner membrane|GO:0044444,cytoplasmic part|GO:0044429,mitochondrial part|GO:0044424,intracellular part|GO:0044425,membrane part|GO:0044422,organelle	
KTI12			GO:0035639,purine ribonucleoside triphosphate binding|GO:0005488,binding|GO:0043167,ion binding|GO:1901363,heterocyclic compound binding|GO:1901265,nucleoside phosphate binding|GO:0001882,nucleoside binding|GO:0043168,anion binding|GO:0000166,nucleotide binding|GO:0001883,purine nucleoside binding|GO:0032549,ribonucleoside binding|GO:0017076,purine nucleotide binding|GO:0005524,ATP binding|GO:0036094,small molecule binding|GO:0030554,adenyl nucleotide binding|GO:0097367,carbohydrate derivative binding|GO:0097159,organic cyclic compound binding|GO:0032559,adenyl ribonucleotide binding|GO:0005515,protein binding|GO:0032555,purine ribonucleotide binding|GO:0032550,purine ribonucleoside binding|GO:0032553,ribonucleotide binding
C11orf65			GO:0005488,binding|GO:0005515,protein binding
ISOC1	GO:0008152,metabolic process	GO:0043231,intracellular membrane-bounded organelle|GO:0005737,cytoplasm|GO:0005622,intracellular|GO:0043230,extracellular organelle|GO:0031982,vesicle|GO:0044421,extracellular region part|GO:0043227,membrane-bounded organelle|GO:0005777,peroxisome|GO:0044444,cytoplasmic part|GO:0005623,cell|GO:0031988,membrane-bounded vesicle|GO:0042579,microbody|GO:0043229,intracellular organelle|GO:0005576,extracellular region|GO:0044424,intracellular part|GO:0044464,cell part|GO:1903561,extracellular vesicle|GO:0065010,extracellular membrane-bounded organelle|GO:0043226,organelle|GO:0070062,extracellular exosome	GO:0003824,catalytic activity|GO:0005488,binding|GO:0005515,protein binding
TNFRSF13	GO:0080090,regulation of primary metabolic process|GO:0019222,regulation of metabolic process|GO:0034110,regulation of homotypic cell-cell adhesion|GO:0048584,positive regulation of response to stimulus|GO:0048583,regulation of response to stimulus|GO:0042035,regulation of cytokine biosynthetic process|GO:0034112,positive regulation of homotypic cell-cell adhesion|GO:0010604,positive regulation of macromolecule metabolic process|GO:0045785,positive regulation of cell adhesion|GO:0002260,lymphocyte homeostasis|GO:0032729,positive regulation of interferon-gamma production|GO:0048518,positive regulation of biological process|GO:0065007,biological regulation|GO:0031296,B cell costimulation|GO:0031295,T cell costimulation|GO:0031294,lymphocyte costimulation|GO:0042127,regulation of cell proliferation|GO:0060255,regulation of macromolecule metabolic process|GO:0002824,positive regulation of adaptive immune response based on somatic recombination of immune receptors built from immunoglobulin superfamily domains|GO:0002822,regulation of adaptive immune response based on somatic recombination of immune receptors built from immunoglobulin superfamily domains|GO:0042129,regulation of T cell proliferation|GO:0002821,positive regulation of adaptive immune response|GO:0046649,lymphocyte activation|GO:0048872,homeostasis of number of cells|GO:0065008,regulation of biological quality|GO:0042089,cytokine biosynthetic process|GO:0044707,single-multicellular organism process|GO:0044249,cellular biosynthetic process|GO:0019538,protein metabolic process|GO:0002376,immune system process|GO:0009893,positive regulation of metabolic process|GO:0009891,positive regulation of biosynthetic process|GO:0032649,regulation of interferon-gamma production|GO:0050778,positive regulation of immune response|GO:0032943,mononuclear cell proliferation|GO:0032946,positive regulation of mononuclear cell proliferation|GO:0032944,regulation of mononuclear cell proliferation|GO:0046651,lymphocyte proliferation|GO:0050789,regulation of biological process|GO:1901576,organic substance biosynthetic process|GO:0042095,interferon-gamma biosynthetic process|GO:0042098,T cell proliferation|GO:0098609,cell-cell adhesion|GO:0002684,positive regulation of immune system process|GO:0002682,regulation of immune system process|GO:0071593,lymphocyte aggregation|GO:0048646,anatomical structure formation involved in morphogenesis|GO:0098602,single organism cell adhesion|GO:0030890,positive regulation of B cell proliferation|GO:0050793,regulation of developmental process|GO:0009889,regulation of biosynthetic process|GO:0050794,regulation of cellular process|GO:0001776,leukocyte homeostasis|GO:0001775,cell activation|GO:0051239,regulation of multicellular organismal process|GO:0006955,immune response|GO:0050896,response to stimulus|GO:0002694,regulation of leukocyte activation|GO:0010557,positive regulation of macromolecule biosynthetic process|GO:0002696,positive regulation of leukocyte activation|GO:0007159,leukocyte cell-cell adhesion|GO:0008152,metabolic process|GO:0030155,regulation of cell adhesion|GO:0030888,regulation of B cell proliferation|GO:0002819,regulation of adaptive immune response|GO:0007155,cell adhesion|GO:0009653,anatomical structure morphogenesis|GO:0044699,single-organism process|GO:0051249,regulation of lymphocyte activation|GO:0045078,positive regulation of interferon-gamma biosynthetic process|GO:0051240,positive regulation of multicellular organismal process|GO:0022603,regulation of anatomical structure morphogenesis|GO:0051246,regulation of protein metabolic process|GO:0051247,positive regulation of protein metabolic process|GO:0045072,regulation of interferon-gamma biosynthetic process|GO:1903039,positive regulation of leukocyte cell-cell adhesion|GO:0050864,regulation of B cell activation|GO:0001782,B cell homeostasis|GO:0022610,biological adhesion|GO:0050867,positive regulation of cell activation|GO:1903037,regulation of leukocyte cell-cell adhesion|GO:0008284,positive regulation of cell proliferation|GO:0032501,multicellular organismal process|GO:0008283,cell proliferation|GO:0009987,cellular process|GO:0050870,positive regulation of T cell activation|GO:0050871,positive regulation of B cell activation|GO:0002636,positive regulation of germinal center formation|GO:0002634,regulation of germinal center formation|GO:0042110,T cell activation|GO:0002467,germinal center formation|GO:0050776,regulation of immune response|GO:0002460,adaptive immune response based on somatic recombination of immune receptors built from immunoglobulin superfamily domains|GO:0051094,positive regulation of developmental process|GO:0051251,positive regulation of lymphocyte activation|GO:0050670,regulation of lymphocyte proliferation|GO:0050671,positive regulation of lymphocyte proliferation|GO:0043170,macromolecule metabolic process|GO:0001816,cytokine production|GO:0001817,regulation of cytokine production|GO:0001819,positive regulation of cytokine production|GO:0032609,interferon-gamma production|GO:0032502,developmental process|GO:0016337,single organismal cell-cell adhesion|GO:0031328,positive regulation of cellular biosynthetic process|GO:0050865,regulation of cell activation|GO:0031326,regulation of cellular biosynthetic process|GO:0031325,positive regulation of cellular metabolic process|GO:0031323,regulation of cellular metabolic process|GO:0042113,B cell activation|GO:0050863,regulation of T cell activation|GO:0042592,homeostatic process|GO:0022407,regulation of cell-cell adhesion|GO:0022409,positive regulation of cell-cell adhesion|GO:0070661,leukocyte proliferation|GO:0071704,organic substance metabolic process|GO:0070663,regulation of leukocyte proliferation|GO:0010556,regulation of macromolecule biosynthetic process|GO:0070665,positive regulation of leukocyte proliferation|GO:0042108,positive regulation of cytokine biosynthetic process|GO:0034109,homotypic cell-cell adhesion|GO:0042107,cytokine metabolic process|GO:0042100,B cell proliferation|GO:0042102,positive regulation of T cell proliferation|GO:0009058,biosynthetic process|GO:0009059,macromolecule biosynthetic process|GO:0070489,T cell aggregation|GO:0045321,leukocyte activation|GO:0070486,leukocyte aggregation|GO:0044238,primary metabolic process|GO:0048856,anatomical structure development|GO:0044237,cellular metabolic process|GO:0044763,single-organism cellular process|GO:0002250,adaptive immune response|GO:0048522,positive regulation of cellular process|GO:0019221,cytokine-mediated signaling pathway|GO:0007165,signal transduction|GO:0007166,cell surface receptor signaling pathway|GO:0051716,cellular response to stimulus|GO:0071310,cellular response to organic substance|GO:0010033,response to organic substance|GO:0044700,single organism signaling|GO:1901564,organonitrogen compound metabolic process|GO:0006807,nitrogen compound metabolic process|GO:0071356,cellular response to tumor necrosis factor|GO:0034612,response to tumor necrosis factor|GO:0034097,response to cytokine|GO:0071345,cellular response to cytokine stimulus|GO:0033209,tumor necrosis factor-mediated signaling pathway|GO:0023052,signaling|GO:0070887,cellular response to chemical stimulus|GO:0042221,response to chemical|GO:0007154,cell communication	GO:0009897,external side of plasma membrane|GO:0016020,membrane|GO:0016021,integral component of membrane|GO:0098552,side of membrane|GO:0044464,cell part|GO:0005623,cell|GO:0071944,cell periphery|GO:0005886,plasma membrane|GO:0044425,membrane part|GO:0044459,plasma membrane part|GO:0009986,cell surface|GO:0031224,intrinsic component of membrane	
ELN		GO:0044421,extracellular region part|GO:0005576,extracellular region|GO:0005578,proteinaceous extracellular matrix|GO:0031012,extracellular matrix	GO:0005201,extracellular matrix structural constituent|GO:0005198,structural molecule activity
STX4	GO:0035774,positive regulation of insulin secretion involved in cellular response to glucose stimulus|GO:0032024,positive regulation of insulin secretion|GO:2001236,regulation of extrinsic apoptotic signaling pathway|GO:0006887,exocytosis|GO:0007599,hemostasis|GO:0019222,regulation of metabolic process|GO:2001233,regulation of apoptotic signaling pathway|GO:0051049,regulation of transport|GO:0008104,protein localization|GO:0048584,positive regulation of response to stimulus|GO:0048583,regulation of response to stimulus|GO:0032846,positive regulation of homeostatic process|GO:0007610,behavior|GO:0032844,regulation of homeostatic process|GO:0061025,membrane fusion|GO:0061024,membrane organization|GO:0002705,positive regulation of leukocyte mediated immunity|GO:1902566,regulation of eosinophil activation|GO:0050920,regulation of chemotaxis|GO:0050921,positive regulation of chemotaxis|GO:0007166,cell surface receptor signaling pathway|GO:0002700,regulation of production of molecular mediator of immune response|GO:0090004,positive regulation of establishment of protein localization to plasma membrane|GO:0090003,regulation of establishment of protein localization to plasma membrane|GO:1902568,positive regulation of eosinophil activation|GO:0071840,cellular component organization or biogenesis|GO:0051716,cellular response to stimulus|GO:0072659,protein localization to plasma membrane|GO:0002440,production of molecular mediator of immune response|GO:0051046,regulation of secretion|GO:0042330,taxis|GO:0045785,positive regulation of cell adhesion|GO:0009966,regulation of signal transduction|GO:0051047,positive regulation of secretion|GO:0010256,endomembrane system organization|GO:0071310,cellular response to organic substance|GO:0046879,hormone secretion|GO:0009746,response to hexose|GO:0071331,cellular response to hexose stimulus|GO:0044093,positive regulation of molecular function|GO:0071333,cellular response to glucose stimulus|GO:0048518,positive regulation of biological process|GO:0065007,biological regulation|GO:0097479,synaptic vesicle localization|GO:0033036,macromolecule localization|GO:0090002,establishment of protein localization to plasma membrane|GO:0042127,regulation of cell proliferation|GO:0006906,vesicle fusion|GO:0006935,chemotaxis|GO:0051050,positive regulation of transport|GO:1901701,cellular response to oxygen-containing compound|GO:0045184,establishment of protein localization|GO:0048278,vesicle docking|GO:0090276,regulation of peptide hormone secretion|GO:0090277,positive regulation of peptide hormone secretion|GO:0007155,cell adhesion|GO:0072657,protein localization to membrane|GO:0002443,leukocyte mediated immunity|GO:0006836,neurotransmitter transport|GO:0051128,regulation of cellular component organization|GO:0010033,response to organic substance|GO:0030168,platelet activation|GO:0044700,single organism signaling|GO:0016477,cell migration|GO:0016192,vesicle-mediated transport|GO:0044707,single-multicellular organism process|GO:0048870,cell motility|GO:0071322,cellular response to carbohydrate stimulus|GO:0002275,myeloid cell activation involved in immune response|GO:0002278,eosinophil activation involved in immune response|GO:0007009,plasma membrane organization|GO:0016050,vesicle organization|GO:0048878,chemical homeostasis|GO:0002376,immune system process|GO:0002377,immunoglobulin production|GO:0002703,regulation of leukocyte mediated immunity|GO:2000008,regulation of protein localization to cell surface|GO:0002702,positive regulation of production of molecular mediator of immune response|GO:0016265,obsolete death|GO:0002274,myeloid leukocyte activation|GO:0009893,positive regulation of metabolic process|GO:1903532,positive regulation of secretion by cell|GO:0006928,movement of cell or subcellular component|GO:0008152,metabolic process|GO:0051023,regulation of immunoglobulin secretion|GO:0048167,regulation of synaptic plasticity|GO:0051674,localization of cell|GO:1903530,regulation of secretion by cell|GO:0051024,positive regulation of immunoglobulin secretion|GO:0051222,positive regulation of protein transport|GO:0051223,regulation of protein transport|GO:0042981,regulation of apoptotic process|GO:0050789,regulation of biological process|GO:0030072,peptide hormone secretion|GO:0030073,insulin secretion|GO:0043308,eosinophil degranulation|GO:0043309,regulation of eosinophil degranulation|GO:0051656,establishment of organelle localization|GO:0050708,regulation of protein secretion|GO:0002447,eosinophil mediated immunity|GO:0043300,regulation of leukocyte degranulation|GO:0043302,positive regulation of leukocyte degranulation|GO:2000010,positive regulation of protein localization to cell surface|GO:1990778,protein localization to cell periphery|GO:0016043,cellular component organization|GO:0045055,regulated exocytosis|GO:0065003,macromolecular complex assembly|GO:0002684,positive regulation of immune system process|GO:0002366,leukocyte activation involved in immune response|GO:0002682,regulation of immune system process|GO:1903829,positive regulation of cellular protein localization|GO:0090174,organelle membrane fusion|GO:0006886,intracellular protein transport|GO:1903307,positive regulation of regulated secretory pathway|GO:0065009,regulation of molecular function|GO:1903305,regulation of regulated secretory pathway|GO:0034613,cellular protein localization|GO:0051130,positive regulation of cellular component organization|GO:0070201,regulation of establishment of protein localization|GO:0006461,protein complex assembly|GO:0009306,protein secretion|GO:0050790,regulation of catalytic activity|GO:0001505,regulation of neurotransmitter levels|GO:0050796,regulation of insulin secretion|GO:0050795,regulation of behavior|GO:0050794,regulation of cellular process|GO:0002888,positive regulation of myeloid leukocyte mediated immunity|GO:0012501,programmed cell death|GO:0001775,cell activation|GO:0050817,coagulation|GO:0044802,single-organism membrane organization|GO:1903729,regulation of plasma membrane organization|GO:0001678,cellular glucose homeostasis|GO:0044801,single-organism membrane fusion|GO:0061178,regulation of insulin secretion involved in cellular response to glucose stimulus|GO:0002886,regulation of myeloid leukocyte mediated immunity|GO:0042060,wound healing|GO:0002263,cell activation involved in immune response|GO:0046903,secretion|GO:0046907,intracellular transport|GO:0050714,positive regulation of protein secretion|GO:0016079,synaptic vesicle exocytosis|GO:0050896,response to stimulus|GO:0070727,cellular macromolecule localization|GO:0006950,response to stress|GO:0043311,positive regulation of eosinophil degranulation|GO:0007596,blood coagulation|GO:0002694,regulation of leukocyte activation|GO:0002697,regulation of immune effector process|GO:0002696,positive regulation of leukocyte activation|GO:2000145,regulation of cell motility|GO:1901700,response to oxygen-containing compound|GO:2000147,positive regulation of cell motility|GO:0002699,positive regulation of immune effector process|GO:0009611,response to wounding|GO:0035493,SNARE complex assembly|GO:1902041,regulation of extrinsic apoptotic signaling pathway via death domain receptors|GO:0048305,immunoglobulin secretion|GO:0032103,positive regulation of response to external stimulus|GO:0032101,regulation of response to external stimulus|GO:0050803,regulation of synapse structure or activity|GO:0030155,regulation of cell adhesion|GO:0051649,establishment of localization in cell|GO:0050806,positive regulation of synaptic transmission|GO:0050804,modulation of synaptic transmission|GO:0023056,positive regulation of signaling|GO:0007267,cell-cell signaling|GO:0015833,peptide transport|GO:0022607,cellular component assembly|GO:0023052,signaling|GO:0007165,signal transduction|GO:0070887,cellular response to chemical stimulus|GO:0023051,regulation of signaling|GO:0010647,positive regulation of cell communication|GO:0010646,regulation of cell communication|GO:0071326,cellular response to monosaccharide stimulus|GO:0043085,positive regulation of catalytic activity|GO:0032880,regulation of protein localization|GO:0042886,amide transport|GO:0008625,extrinsic apoptotic signaling pathway via death domain receptors|GO:0009749,response to glucose|GO:0002444,myeloid leukocyte mediated immunity|GO:0034394,protein localization to cell surface|GO:0043933,macromolecular complex subunit organization|GO:0051640,organelle localization|GO:0006810,transport|GO:0006892,post-Golgi vesicle-mediated transport|GO:0017157,regulation of exocytosis|GO:0008284,positive regulation of cell proliferation|GO:0040011,locomotion|GO:0032501,multicellular organismal process|GO:0050878,regulation of body fluid levels|GO:0008283,cell	GO:0008021,synaptic vesicle|GO:0042995,cell projection|GO:0043219,lateral loop|GO:0098590,plasma membrane region|GO:0044421,extracellular region part|GO:0030425,dendrite|GO:0043229,intracellular organelle|GO:0071944,cell periphery|GO:0045202,synapse|GO:0031201,SNARE complex|GO:0043227,membrane-bounded organelle|GO:0043226,organelle|GO:0031224,intrinsic component of membrane|GO:0005737,cytoplasm|GO:0031984,organelle subcompartment|GO:0070062,extracellular exosome|GO:0044463,cell projection part|GO:0098796,membrane protein complex|GO:0016023,cytoplasmic, membrane-bounded vesicle|GO:0031410,cytoplasmic vesicle|GO:0016021,integral component of membrane|GO:0016020,membrane|GO:0044309,neuron spine|GO:0031988,membrane-bounded vesicle|GO:0030027,lamellipodium|GO:0044431,Golgi apparatus part|GO:0044456,synapse part|GO:0005615,extracellular space|GO:0043005,neuron projection|GO:0043209,myelin sheath|GO:0048471,perinuclear region of cytoplasm|GO:0044459,plasma membrane part|GO:0005576,extracellular region|GO:0005794,Golgi apparatus|GO:0097458,neuron part|GO:0035749,myelin sheath adaxonal region|GO:0030141,secretory granule|GO:0009986,cell surface|GO:0098589,membrane region|GO:0031252,cell leading edge|GO:0016323,basolateral plasma membrane|GO:0042581,specific granule|GO:0012505,endomembrane system|GO:0005886,plasma membrane|GO:0032991,macromolecular complex|GO:1903561,extracellular vesicle|GO:0031982,vesicle|GO:0043234,protein complex|GO:0043230,extracellular organelle|GO:0043231,intracellular membrane-bounded organelle|GO:0043197,dendritic spine|GO:0005768,endosome|GO:0005829,cytosol|GO:0005802,trans-Golgi network|GO:0044464,cell part|GO:0005623,cell|GO:0005622,intracellular|GO:0044446,intracellular organelle part|GO:0005773,vacuole|GO:0044444,cytoplasmic part|GO:0098794,postsynapse|GO:0098793,presynapse|GO:0098791,Golgi subcompartment|GO:0044424,intracellular part|GO:0044425,membrane part|GO:0065010,extracellular membrane-bounded organelle|GO:0098805,whole membrane|GO:0044422,organelle part|GO:0036477,somatodendritic compartment|GO:0000322,storage vacuole|GO:0030139,endocytic vesicle|GO:0030133,transport vesicle|GO:0044433,cytoplasmic vesicle part|GO:0070382,exocytic vesicle|GO:0099503,secretory vesicle|GO:0098588,bounding membrane of organelle|GO:0030659,cytoplasmic vesicle membrane|GO:0045335,phagocytic vesicle|GO:0030666,endocytic vesicle membrane|GO:0097708,intracellular vesicle|GO:0012506,vesicle membrane|GO:0030670,phagocytic vesicle membrane|GO:0031090,organelle membrane	GO:0005488,binding|GO:0016230,sphingomyelin phosphodiesterase activator activity|GO:0060229,lipase activator activity|GO:0008047,enzyme activator activity|GO:0005515,protein binding|GO:0000149,SNARE binding|GO:0005484,SNAP receptor activity|GO:0098772,molecular function regulator|GO:0030234,enzyme regulator activity|GO:0016004,phospholipase activator activity
LUZP1	GO:0032502,developmental process|GO:0016331,morphogenesis of embryonic epithelium|GO:0044707,single-multicellular organism process|GO:0060571,morphogenesis of an epithelial fold|GO:0003281,ventricular septum development|GO:0035148,tube formation|GO:0009790,embryo development|GO:0072175,epithelial tube formation|GO:0009792,embryo development ending in birth or egg hatching|GO:0021915,neural tube development|GO:0009653,anatomical structure morphogenesis|GO:0007275,multicellular organism development|GO:0044699,single-organism process|GO:0003205,cardiac chamber development|GO:0002009,morphogenesis of an epithelium|GO:0001568,blood vessel development|GO:0001944,vasculature development|GO:0048513,animal organ development|GO:0048729,tissue morphogenesis|GO:0001841,neural tube formation|GO:0060562,epithelial tube morphogenesis|GO:0043009,chordate embryonic development|GO:0001842,neural fold formation|GO:0048646,anatomical structure formation involved in morphogenesis|GO:0048598,embryonic morphogenesis|GO:0032501,multicellular organismal process|GO:0035239,tube morphogenesis|GO:0007507,heart development|GO:0060429,epithelium development|GO:0021503,neural fold bending|GO:0009888,tissue development|GO:0044767,single-organism developmental process|GO:0003231,cardiac ventricle development|GO:0035295,tube development|GO:0001838,embryonic epithelial tube formation|GO:0014020,primary neural tube formation|GO:0003279,cardiac septum development|GO:0007399,nervous system development|GO:0048856,anatomical structure development|GO:0072359,circulatory system development|GO:0072358,cardiovascular system development|GO:0048731,system	GO:0043231,intracellular membrane-bounded organelle|GO:0005622,intracellular|GO:0043230,extracellular organelle|GO:0031982,vesicle|GO:0044421,extracellular region part|GO:0043227,membrane-bounded organelle|GO:0005634,nucleus|GO:0016020,membrane|GO:0044464,cell part|GO:0005623,cell|GO:0031988,membrane-bounded vesicle|GO:0043229,intracellular organelle|GO:0005576,extracellular region|GO:0044424,intracellular part|GO:1903561,extracellular vesicle|GO:0065010,extracellular membrane-bounded organelle|GO:0043226,organelle|GO:0070062,extracellular	

DCTN3	GO:0044839,cell cycle G2/M phase transition|GO:0000086,G2/M transition of mitotic cell cycle|GO:1903047,mitotic cell cycle process|GO:0044770,cell cycle phase transition|GO:0044772,mitotic cell cycle phase transition|GO:0022402,cell cycle process|GO:0051301,cell division|GO:0048002,antigen processing and presentation of peptide antigen|GO:0007067,mitotic nuclear division|GO:0044699,single-organism process|GO:0000280,nuclear division|GO:0002478,antigen processing and presentation of exogenous peptide antigen|GO:0016043,cellular component organization|GO:0071840,cellular component organization or biogenesis|GO:0006996,organelle organization|GO:0019882,antigen processing and presentation|GO:0019884,antigen processing and presentation of exogenous antigen|GO:0000278,mitotic cell cycle|GO:0019886,antigen processing and presentation of exogenous peptide antigen via MHC class II|GO:0009987,cellular process|GO:0044763,single-organism cellular process|GO:0002495,antigen processing and presentation of peptide antigen via MHC class II|GO:0000910,cytokinesis|GO:0002504,antigen processing and presentation of peptide or polysaccharide antigen via MHC class II|GO:0007049,cell cycle|GO:0007017,microtubule-based process|GO:0002376,immune system process|GO:1902589,single-organism organelle organization|GO:0048285,organelle fission|GO:0006888,ER to Golgi vesicle-mediated transport|GO:0006810,transport|GO:0048193,Golgi vesicle transport|GO:0051649,establishment of localization in cell|GO:0051234,establishment of localization|GO:0051179,localization|GO:0051641,cellular localization|GO:0016192,vesicle-mediated transport|GO:0046907,intracellular transport|GO:0007346,regulation of mitotic cell cycle|GO:0051726,regulation of cell cycle|GO:0010564,regulation of cell cycle process|GO:0050794,regulation of cellular process|GO:0050789,regulation of biological process|GO:1901987,regulation of cell cycle phase transition|GO:1902749,regulation of cell cycle G2/M phase transition|GO:1901990,regulation of mitotic cell cycle phase transition|GO:0065007,biological regulation|GO:0010389,regulation of G2/M transition of mitotic cell cycle|GO:0061640,cytoskeleton-dependent cytokinesis	GO:0015629,actin cytoskeleton|GO:0044464,cell part|GO:0043226,organelle|GO:0043229,intracellular organelle|GO:0043228,non-membrane-bounded organelle|GO:0097610,cell surface furrow|GO:0005875,microtubule associated complex|GO:0005856,cytoskeleton|GO:0005819,spindle|GO:0005813,centrosome|GO:0005815,microtubule organizing center|GO:0044430,cytoskeletal part|GO:0000776,kinetochore|GO:0048471,perinuclear region of cytoplasm|GO:0005694,chromosome|GO:0009986,cell surface|GO:0005737,cytoplasm|GO:0015630,microtubule cytoskeleton|GO:0032153,cell division site|GO:0032155,cell division site part|GO:0032154,cleavage furrow|GO:0000793,condensed chromosome|GO:0005869,dynactin complex|GO:0000775,chromosome, centromeric region|GO:0098687,chromosomal region|GO:0000777,condensed chromosome kinetochore|GO:0043234,protein complex|GO:0032991,macromolecular complex|GO:0043232,intracellular non-membrane-bounded organelle|GO:0000779,condensed chromosome, centromeric region|GO:0005829,cytosol|GO:0030496,midbody|GO:0005623,cell|GO:0005622,intracellular|GO:0044446,intracellular organelle part|GO:0044444,cytoplasmic part|GO:0044424,intracellular part|GO:0044427,chromosomal part|GO:0044422,organelle part|GO:0031974,membrane-enclosed lumen|GO:0043227,membrane-bounded organelle|GO:0031981,nuclear lumen|GO:0005730,nucleolus|GO:0005634,nucleus|GO:0043231,intracellular membrane-bounded organelle|GO:0043233,organelle lumen|GO:0070013,intracellular organelle lumen|GO:0044428,nuclear part|GO:0099512,supramolecular fiber|GO:0005874,microtubule|GO:0099513,polymeric	GO:0005488,binding|GO:0005515,protein binding|GO:0005198,structural molecule activity
RADIL	GO:0044700,single organism signaling|GO:0051716,cellular response to stimulus|GO:0050896,response to stimulus|GO:0009987,cellular process|GO:0044763,single-organism cellular process|GO:0050794,regulation of cellular process|GO:0065007,biological regulation|GO:0023052,signaling|GO:0007165,signal transduction|GO:0007154,cell communication|GO:0050789,regulation of biological process|GO:0044699,single-organism process		
OCSTAMP	GO:0000768,syncytium formation by plasma membrane fusion|GO:0071840,cellular component organization or biogenesis|GO:0051716,cellular response to stimulus|GO:0048869,cellular developmental process|GO:0070663,regulation of leukocyte proliferation|GO:0048513,animal organ development|GO:0048518,positive regulation of biological process|GO:0002682,regulation of immune system process|GO:0042127,regulation of cell proliferation|GO:1903708,positive regulation of hemopoiesis|GO:0060143,positive regulation of syncytium formation by plasma membrane fusion|GO:0060142,regulation of syncytium formation by plasma membrane fusion|GO:1903706,regulation of hemopoiesis|GO:0010033,response to organic substance|GO:0044707,single-multicellular organism process|GO:0002376,immune system process|GO:0071407,cellular response to organic cyclic compound|GO:0022603,regulation of anatomical structure morphogenesis|GO:0050789,regulation of biological process|GO:0002761,regulation of myeloid leukocyte differentiation|GO:0002763,positive regulation of myeloid leukocyte differentiation|GO:0071356,cellular response to tumor necrosis factor|GO:0016043,cellular component organization|GO:0002684,positive regulation of immune system process|GO:0065007,biological regulation|GO:0044699,single-organism process|GO:0048646,anatomical structure formation involved in morphogenesis|GO:0051130,positive regulation of cellular component organization|GO:0034097,response to cytokine|GO:0050793,regulation of developmental process|GO:0050794,regulation of cellular process|GO:0072674,multinuclear osteoclast differentiation|GO:0051239,regulation of multicellular organismal process|GO:0030097,hemopoiesis|GO:0002521,leukocyte differentiation|GO:0002520,immune system development|GO:0090290,positive regulation of osteoclast proliferation|GO:0071345,cellular response to cytokine stimulus|GO:0050896,response to stimulus|GO:0032502,developmental process|GO:0045639,positive regulation of myeloid cell differentiation|GO:0045637,regulation of myeloid cell differentiation|GO:0030154,cell differentiation|GO:0051128,regulation of cellular component organization|GO:0070887,cellular response to chemical stimulus|GO:0006949,syncytium formation|GO:0009653,anatomical structure morphogenesis|GO:0014070,response to organic cyclic compound|GO:0009719,response to endogenous stimulus|GO:0051240,positive regulation of multicellular organismal process|GO:0090289,regulation of osteoclast proliferation|GO:0008284,positive regulation of cell proliferation|GO:0032501,multicellular organismal process|GO:0034241,positive regulation of macrophage fusion|GO:0008283,cell proliferation|GO:0030316,osteoclast differentiation|GO:0071396,cellular response to lipid|GO:0045597,positive regulation of cell differentiation|GO:0032870,cellular response to hormone stimulus|GO:0051094,positive regulation of developmental process|GO:0009725,response to hormone|GO:0030099,myeloid cell differentiation|GO:0048731,system development|GO:0009987,cellular process|GO:0048545,response to steroid hormone|GO:0071495,cellular response to endogenous stimulus|GO:0071383,cellular response to steroid hormone stimulus|GO:0034238,macrophage fusion|GO:0034239,regulation of macrophage fusion|GO:0007275,multicellular organism development|GO:0033993,response to lipid|GO:0002573,myeloid leukocyte differentiation|GO:0070661,leukocyte proliferation|GO:0071310,cellular response to organic substance|GO:0070665,positive regulation of leukocyte proliferation|GO:0045672,positive regulation of osteoclast differentiation|GO:0048534,hematopoietic or lymphoid organ development|GO:0071391,cellular response to estrogen stimulus|GO:0034612,response to tumor necrosis factor|GO:0044767,single-organism developmental process|GO:0044763,single-organism cellular process|GO:0002158,osteoclast proliferation|GO:0043627,response to estrogen|GO:0042221,response to chemical|GO:0048856,anatomical structure development|GO:0045595,regulation of cell differentiation|GO:1902107,positive regulation of leukocyte differentiation|GO:1902105,regulation of leukocyte differentiation|GO:2000026,regulation of multicellular organismal development|GO:0045670,regulation of osteoclast differentiation|GO:0048522,positive regulation of cellular process	GO:0044425,membrane part|GO:0016021,integral component of membrane|GO:0016020,membrane|GO:0031224,intrinsic component of membrane	
GATS			
BSPRY	GO:0070838,divalent metal ion transport|GO:0006812,cation transport|GO:0006811,ion transport|GO:0006810,transport|GO:0006816,calcium ion transport|GO:0044765,single-organism transport|GO:0030001,metal ion transport|GO:0044699,single-organism process|GO:0072511,divalent inorganic cation transport|GO:0051234,establishment of localization|GO:0051179,localization|GO:1902578,single-organism localization|GO:0042221,response to chemical|GO:0051716,cellular response to stimulus|GO:1990830,cellular response to leukemia inhibitory factor|GO:0034097,response to cytokine|GO:0071345,cellular response to cytokine stimulus|GO:0009987,cellular process|GO:0010033,response to organic substance|GO:0050896,response to stimulus|GO:0071310,cellular response to organic substance|GO:0070887,cellular response to chemical stimulus|GO:1990823,response to leukemia inhibitory factor	GO:0031974,membrane-enclosed lumen|GO:0043229,intracellular organelle|GO:0005924,cell-substrate adherens junction|GO:0005925,focal adhesion|GO:0043227,membrane-bounded organelle|GO:0043226,organelle|GO:0030054,cell junction|GO:0030055,cell-substrate junction|GO:0005737,cytoplasm|GO:0031981,nuclear lumen|GO:0005634,nucleus|GO:0016020,membrane|GO:0005654,nucleoplasm|GO:0048471,perinuclear region of cytoplasm|GO:0070161,anchoring junction|GO:0031252,cell leading edge|GO:0005912,adherens junction|GO:0043231,intracellular membrane-bounded organelle|GO:0043233,organelle lumen|GO:0044464,cell part|GO:0005623,cell|GO:0005622,intracellular|GO:0044446,intracellular organelle part|GO:0070013,intracellular	GO:0043169,cation binding|GO:0046914,transition metal ion binding|GO:0043167,ion binding|GO:0005488,binding|GO:0046872,metal ion binding|GO:0008270,zinc ion binding
GPRIN2			GO:0005488,binding|GO:0005515,protein binding
CSF2	GO:2001234,negative regulation of apoptotic signaling pathway|GO:2001236,regulation of extrinsic apoptotic signaling pathway|GO:2001237,negative regulation of extrinsic apoptotic signaling pathway|GO:0019220,regulation of phosphate metabolic process|GO:0080090,regulation of primary metabolic process|GO:0019222,regulation of metabolic process|GO:2001233,regulation of apoptotic signaling pathway|GO:0048585,negative regulation of response to stimulus|GO:0048584,positive regulation of response to stimulus|GO:0048583,regulation of response to stimulus|GO:2001239,regulation of extrinsic apoptotic signaling pathway in absence of ligand|GO:0043933,macromolecular complex subunit organization|GO:0007165,signal transduction|GO:0007166,cell surface receptor signaling pathway|GO:0032627,interleukin-23 production|GO:0051234,establishment of localization|GO:0071840,cellular component organization or biogenesis|GO:0051716,cellular response to stimulus|GO:0010604,positive regulation of macromolecule metabolic process|GO:0009968,negative regulation of signal transduction|GO:0009966,regulation of signal transduction|GO:0009967,positive regulation of signal transduction|GO:0070271,protein complex biogenesis|GO:0018212,peptidyl-tyrosine modification|GO:0042531,positive regulation of tyrosine phosphorylation of STAT protein|GO:0018193,peptidyl-amino acid modification|GO:0048513,animal organ development|GO:0043011,myeloid dendritic cell differentiation|GO:0048522,positive regulation of cellular process|GO:0048518,positive regulation of biological process|GO:0048519,negative regulation of biological process|GO:0051704,multi-organism process|GO:0042127,regulation of cell proliferation|GO:0042325,regulation of phosphorylation|GO:0051054,positive regulation of DNA metabolic process|GO:0051052,regulation of DNA metabolic process|GO:0009058,biosynthetic process|GO:0042522,regulation of tyrosine phosphorylation of Stat5 protein|GO:0060255,regulation of macromolecule metabolic process|GO:0060548,negative regulation of cell death|GO:0034645,cellular macromolecule biosynthetic process|GO:0003006,developmental process involved in reproduction|GO:0042221,response to chemical|GO:0051707,response to other organism|GO:0010033,response to organic substance|GO:0046483,heterocycle metabolic process|GO:0042523,positive regulation of tyrosine phosphorylation of Stat5 protein|GO:0044700,single organism signaling|GO:0042327,positive regulation of phosphorylation|GO:0044702,single organism reproductive process|GO:0009607,response to biotic stimulus|GO:0009605,response to external stimulus|GO:0044707,single-multicellular organism process|GO:0009790,embryo development|GO:0050731,positive regulation of peptidyl-tyrosine phosphorylation|GO:0050730,regulation of peptidyl-tyrosine phosphorylation|GO:0002274,myeloid leukocyte activation|GO:0010638,positive regulation of organelle organization|GO:0090077,foam cell differentiation|GO:0006275,regulation of DNA replication|GO:0071310,cellular response to organic substance|GO:0002376,immune system process|GO:0019538,protein metabolic process|GO:0007154,cell communication|GO:0016265,obsolete death|GO:0022607,cellular component assembly|GO:0032496,response to lipopolysaccharide|GO:0009893,positive regulation of metabolic process|GO:0009891,positive regulation of biosynthetic process|GO:0048568,embryonic organ development|GO:0045321,leukocyte activation|GO:0010628,positive regulation of gene expression|GO:0035556,intracellular signal transduction|GO:0042981,regulation of apoptotic process|GO:0050789,regulation of biological process|GO:0019835,cytolysis|GO:0044267,cellular protein metabolic process|GO:0044260,cellular macromolecule metabolic process|GO:0016043,cellular component organization|GO:0065003,macromolecular complex assembly|GO:0045918,negative regulation of cytolysis|GO:0065007,biological regulation|GO:1901360,organic cyclic compound metabolic process|GO:0071219,cellular response to molecule of bacterial origin|GO:0071216,cellular response to biotic stimulus|GO:0051130,positive regulation of cellular component organization|GO:0006461,protein complex assembly|GO:0006139,nucleobase-containing compound metabolic process|GO:0050793,regulation of developmental process|GO:0006810,transport|GO:0009889,regulation of biosynthetic process|GO:0050794,regulation of cellular process|GO:0012501,programmed cell death|GO:0001775,cell activation|GO:0001701,in utero embryonic development|GO:0001773,myeloid dendritic cell activation|GO:1901700,response to oxygen-containing compound|GO:0051239,regulation of multicellular organismal process|GO:0048731,system development|GO:0042044,fluid transport|GO:1902533,positive regulation of intracellular signal transduction|GO:0043254,regulation of protein complex assembly|GO:1902531,regulation of intracellular signal transduction|GO:0044238,primary metabolic process|GO:0002521,leukocyte differentiation|GO:0002520,immune system development|GO:0044767,single-organism developmental process|GO:0042268,regulation of cytolysis|GO:0043207,response to external biotic stimulus|GO:0050896,response to stimulus|GO:0031401,positive regulation of protein modification process|GO:0043412,macromolecule modification|GO:0010557,positive regulation of macromolecule biosynthetic process|GO:0010556,regulation of macromolecule biosynthetic process|GO:0048869,cellular developmental process|GO:1901576,organic substance biosynthetic process|GO:0051240,positive regulation of multicellular organismal process|GO:0009617,response to bacterium|GO:0008152,metabolic process|GO:0033043,regulation of organelle organization|GO:0016310,phosphorylation|GO:0030154,cell differentiation|GO:0051128,regulation of cellular component organization|GO:0023056,positive regulation of signaling|GO:0023057,negative regulation of signaling|GO:0034641,cellular nitrogen compound metabolic process|GO:0009792,embryo development ending in birth or egg hatching|GO:0023052,signaling|GO:0010648,negative regulation of cell communication|GO:0070887,cellular response to chemical stimulus|GO:0023051,regulation of signaling|GO:0010647,positive regulation of cell communication|GO:0010646,regulation of cell communication|GO:0044699,single-organism process|GO:0071803,positive regulation of podosome assembly|GO:0071800,podosome assembly|GO:0071801,regulation of podosome assembly|GO:0010562,positive regulation of phosphorus metabolic process|GO:0051246,regulation of protein metabolic process|GO:0051247,positive regulation of protein metabolic process|GO:0007259,JAK-STAT cascade|GO:0042116,macrophage activation|GO:0032270,positive regulation of cellular protein metabolic process|GO:0045740,positive regulation of DNA replication|GO:0031399,regulation of protein modification process|GO:0006260,DNA replication|GO:0043009,chordate embryonic development|GO:0097028,dendritic cell differentiation|GO:2001240,negative regulation of extrinsic apoptotic signaling pathway in absence of ligand|GO:0008284,positive regulation of cell proliferation|GO:0006996,organelle organization|GO:0032747,positive regulation of interleukin-23 production|GO:0038034,signal transduction in absence of ligand|GO:0042045,epithelial fluid transport|GO:0008283,cell proliferation|GO:0031323,regulation of cellular metabolic process|GO:0009987,cellular process|GO:0031334,positive regulation of protein complex assembly|GO:0036211,protein modification process|GO:0045597,positive regulation of cell differentiation|GO:0045595,regulation of cell differentiation|GO:0018108,peptidyl-tyrosine phosphorylation|GO:0006955,immune response|GO:0001892,embryonic placenta development|GO:0055085,transmembrane transport|GO:0001890,placenta development|GO:0032268,regulation of cellular protein metabolic process|GO:0051094,positive regulation of developmental process|GO:0043170,macromolecule metabolic process|GO:0001816,cytokine production|GO:0001817,regulation of cytokine production|GO:0006807,nitrogen compound metabolic process|GO:0002237,response to molecule of bacterial origin|GO:0030099,myeloid cell differentiation|GO:0032667,regulation of interleukin-23 production|GO:0030097,hemopoiesis|GO:0001819,positive regulation of cytokine production|GO:0032502,developmental process|GO:0071222,cellular response to lipopolysaccharide|GO:0031328,positive regulation of cellular biosynthetic process|GO:0061458,reproductive system development|GO:0031326,regulation of cellular biosynthetic process|GO:0031325,positive regulation of cellular metabolic process|GO:0097190,apoptotic signaling pathway|GO:0097191,extrinsic apoptotic signaling pathway|GO:0097192,extrinsic apoptotic signaling pathway in absence of ligand|GO:0090304,nucleic acid metabolic process|GO:0032501,multicellular organismal process|GO:0010467,gene	GO:0005576,extracellular region|GO:0005615,extracellular space|GO:0044421,extracellular region part|GO:0043231,intracellular membrane-bounded organelle|GO:0044464,cell part|GO:0005623,cell|GO:0005622,intracellular|GO:0043229,intracellular organelle|GO:0044424,intracellular part|GO:0043227,membrane-bounded organelle|GO:0043226,organelle	GO:0005126,cytokine receptor binding|GO:0005125,cytokine activity|GO:0008083,growth factor activity|GO:0070851,growth factor receptor binding|GO:0005129,granulocyte macrophage colony-stimulating factor receptor binding|GO:0005488,binding|GO:0005515,protein binding|GO:0005102,receptor binding|GO:0005088,Ras guanyl-nucleotide exchange factor activity|GO:0016773,phosphotransferase activity, alcohol group as acceptor|GO:0016772,transferase activity, transferring phosphorus-containing groups|GO:0016301,kinase activity|GO:0005085,guanyl-nucleotide exchange factor activity|GO:0003824,catalytic activity|GO:0016740,transferase activity|GO:0004713,protein tyrosine kinase activity|GO:0004672,protein kinase activity|GO:0098772,molecular function regulator
PCDP1	GO:0032502,developmental process|GO:0007017,microtubule-based process|GO:0000902,cell morphogenesis|GO:0060271,cilium morphogenesis|GO:0030030,cell projection organization|GO:0048869,cellular developmental process|GO:0048856,anatomical structure development|GO:0007018,microtubule-based movement|GO:0016043,cellular component organization|GO:0032989,cellular component morphogenesis|GO:0044767,single-organism developmental process|GO:0044763,single-organism cellular process|GO:0009653,anatomical structure morphogenesis|GO:0006928,movement of cell or subcellular component|GO:0071840,cellular component organization or biogenesis|GO:0003341,cilium movement|GO:0009987,cellular process|GO:0032990,cell part morphogenesis|GO:0044699,single-organism process|GO:0048858,cell projection morphogenesis|GO:0044458,motile cilium assembly	GO:0005856,cytoskeleton|GO:0005737,cytoplasm|GO:0044463,cell projection part|GO:0032838,cell projection cytoplasm|GO:0043232,intracellular non-membrane-bounded organelle|GO:0005929,cilium|GO:0044464,cell part|GO:0044444,cytoplasmic part|GO:0005623,cell|GO:0043226,organelle|GO:0043229,intracellular organelle|GO:0043228,non-membrane-bounded organelle|GO:0097014,ciliary plasm|GO:0044441,ciliary part|GO:0044424,intracellular part|GO:0042995,cell projection|GO:0005930,axoneme|GO:0044422,organelle part|GO:0005622,intracellular|GO:0044446,intracellular organelle part|GO:0044430,cytoskeletal part	GO:0005516,calmodulin binding|GO:0005515,protein binding|GO:0005488,binding
C1orf158			
LL22NC03			
OSBPL5	GO:0033036,macromolecule localization|GO:0006869,lipid transport|GO:0006810,transport|GO:0044765,single-organism transport|GO:1902578,single-organism localization|GO:0071702,organic substance transport|GO:0051234,establishment of localization|GO:0010876,lipid localization|GO:0051179,localization|GO:0044699,single-organism process		
RIBC2		GO:0043231,intracellular membrane-bounded organelle|GO:0005634,nucleus|GO:0044464,cell part|GO:0005623,cell|GO:0005622,intracellular|GO:0043229,intracellular organelle|GO:0044424,intracellular part|GO:0043227,membrane-bounded organelle|GO:0043226,organelle	GO:0005488,binding|GO:0005515,protein binding
PYCR1	GO:0042391,regulation of membrane potential|GO:0044238,primary metabolic process|GO:0008152,metabolic process|GO:1901566,organonitrogen compound biosynthetic process|GO:0048585,negative regulation of response to stimulus|GO:0009064,glutamine family amino acid metabolic process|GO:0048583,regulation of response to stimulus|GO:0019752,carboxylic acid metabolic process|GO:0000302,response to reactive oxygen species|GO:0044249,cellular biosynthetic process|GO:0034641,cellular nitrogen compound metabolic process|GO:0006807,nitrogen compound metabolic process|GO:0044281,small molecule metabolic process|GO:0070887,cellular response to chemical stimulus|GO:0044283,small molecule biosynthetic process|GO:0055129,L-proline biosynthetic process|GO:0010941,regulation of cell death|GO:1901362,organic cyclic compound biosynthetic process|GO:0050789,regulation of biological process|GO:0008219,cell death|GO:1901576,organic substance biosynthetic process|GO:0044710,single-organism metabolic process|GO:0044711,single-organism biosynthetic process|GO:0006561,proline biosynthetic process|GO:0006560,proline metabolic process|GO:1901360,organic cyclic compound metabolic process|GO:0006520,cellular amino acid metabolic process|GO:0006979,response to oxidative stress|GO:1903206,negative regulation of hydrogen peroxide-induced cell death|GO:1903205,regulation of hydrogen peroxide-induced cell death|GO:0065007,biological regulation|GO:1903202,negative regulation of oxidative stress-induced cell death|GO:1903201,regulation of oxidative stress-induced cell death|GO:0048519,negative regulation of biological process|GO:0065008,regulation of biological quality|GO:1901605,alpha-amino acid metabolic process|GO:1901607,alpha-amino acid biosynthetic process|GO:0018130,heterocycle biosynthetic process|GO:0016265,obsolete death|GO:1901700,response to oxygen-containing compound|GO:0009987,cellular process|GO:1901701,cellular response to oxygen-containing compound|GO:0060548,negative regulation of cell death|GO:0070301,cellular response to hydrogen peroxide|GO:0034614,cellular response to reactive oxygen species|GO:0051716,cellular response to stimulus|GO:0050794,regulation of cellular process|GO:0036473,cell death in response to oxidative stress|GO:0009058,biosynthetic process|GO:0006950,response to stress|GO:0044763,single-organism cellular process|GO:0036474,cell death in response to hydrogen peroxide|GO:0043436,oxoacid metabolic process|GO:0042221,response to chemical|GO:0034599,cellular response to oxidative stress|GO:0010035,response to inorganic substance|GO:1902882,regulation of response to oxidative stress|GO:1902883,negative regulation of response to oxidative stress|GO:0055114,oxidation-reduction process|GO:0046483,heterocycle metabolic process|GO:0071704,organic substance metabolic process|GO:0008652,cellular amino acid biosynthetic process|GO:0044699,single-organism process|GO:1900407,regulation of cellular response to oxidative stress|GO:1901564,organonitrogen compound metabolic process|GO:0051881,regulation of mitochondrial membrane potential|GO:0006082,organic acid metabolic process|GO:0042542,response to hydrogen peroxide|GO:0046394,carboxylic acid biosynthetic process|GO:0080134,regulation of response to stress|GO:0080135,regulation of cellular response to stress|GO:1900408,negative regulation of cellular response to oxidative stress|GO:0016053,organic acid biosynthetic process|GO:0044237,cellular metabolic process|GO:1901032,negative regulation of response to reactive oxygen species|GO:0033554,cellular response to stress|GO:1901031,regulation of response to reactive oxygen species|GO:0009084,glutamine family amino acid biosynthetic process|GO:0050896,response to stimulus|GO:0048523,negative regulation of	GO:0005737,cytoplasm|GO:0043231,intracellular membrane-bounded organelle|GO:0043233,organelle lumen|GO:0044464,cell part|GO:0044444,cytoplasmic part|GO:0031974,membrane-enclosed lumen|GO:0005622,intracellular|GO:0044446,intracellular organelle part|GO:0070013,intracellular organelle lumen|GO:0043229,intracellular organelle|GO:0005739,mitochondrion|GO:0044429,mitochondrial part|GO:0005759,mitochondrial matrix|GO:0044424,intracellular part|GO:0005623,cell|GO:0043227,membrane-bounded organelle|GO:0043226,organelle|GO:0044422,organelle part	GO:0004735,pyrroline-5-carboxylate reductase activity|GO:0016645,oxidoreductase activity, acting on the CH-NH group of donors|GO:0016646,oxidoreductase activity, acting on the CH-NH group of donors, NAD or NADP as acceptor|GO:0003824,catalytic activity|GO:0005488,binding|GO:0005515,protein binding|GO:0042802,identical protein binding|GO:0016491,oxidoreductase activity
CDKN2A	GO:0016310,phosphorylation|GO:0009987,cellular process|GO:0044237,cellular metabolic process|GO:0006796,phosphate-containing compound metabolic process|GO:0008152,metabolic process|GO:0006793,phosphorus metabolic process		GO:0016772,transferase activity, transferring phosphorus-containing groups|GO:0016740,transferase activity|GO:0016301,kinase activity|GO:0003824,catalytic activity
CYP2B6	GO:0006631,fatty acid metabolic process|GO:0019752,carboxylic acid metabolic process|GO:0044248,cellular catabolic process|GO:0006805,xenobiotic metabolic process|GO:0009410,response to xenobiotic stimulus|GO:0044281,small molecule metabolic process|GO:0070887,cellular response to chemical stimulus|GO:0044699,single-organism process|GO:0044710,single-organism metabolic process|GO:1901360,organic cyclic compound metabolic process|GO:0071704,organic substance metabolic process|GO:0055114,oxidation-reduction process|GO:0001676,long-chain fatty acid metabolic process|GO:0019373,epoxygenase P450 pathway|GO:0006629,lipid metabolic process|GO:0009987,cellular process|GO:0051716,cellular response to stimulus|GO:0032787,monocarboxylic acid metabolic process|GO:0044763,single-organism cellular process|GO:0008152,metabolic process|GO:0043436,oxoacid metabolic process|GO:0042221,response to chemical|GO:0044255,cellular lipid metabolic process|GO:0009056,catabolic process|GO:0008202,steroid metabolic process|GO:0042737,drug catabolic process|GO:0044238,primary metabolic process|GO:0017144,drug metabolic process|GO:0042180,cellular ketone metabolic process|GO:0006082,organic acid metabolic process|GO:1901568,fatty acid derivative metabolic process|GO:0042738,exogenous drug catabolic process|GO:0042493,response to drug|GO:0044237,cellular metabolic process|GO:0006690,icosanoid metabolic process|GO:0071466,cellular response to xenobiotic stimulus|GO:0019369,arachidonic acid metabolic process|GO:0033559,unsaturated fatty acid metabolic process|GO:0050896,response to stimulus	GO:0005783,endoplasmic reticulum|GO:0005789,endoplasmic reticulum membrane|GO:0042175,nuclear outer membrane-endoplasmic reticulum membrane network|GO:0043229,intracellular organelle|GO:0043227,membrane-bounded organelle|GO:0043226,organelle|GO:0005737,cytoplasm|GO:0044446,intracellular organelle part|GO:0031090,organelle membrane|GO:0016020,membrane|GO:0044432,endoplasmic reticulum part|GO:0098588,bounding membrane of organelle|GO:0012505,endomembrane system|GO:0043231,intracellular membrane-bounded organelle|GO:0044464,cell part|GO:0005623,cell|GO:0005622,intracellular|GO:0044444,cytoplasmic part|GO:0044424,intracellular part|GO:0044425,membrane part|GO:0044422,organelle part|GO:0031984,organelle subcompartment|GO:0098827,endoplasmic reticulum subcompartment	GO:0016491,oxidoreductase activity|GO:0043169,cation binding|GO:0004497,monooxygenase activity|GO:0046872,metal ion binding|GO:0046914,transition metal ion binding|GO:0020037,heme binding|GO:0046906,tetrapyrrole binding|GO:0097159,organic cyclic compound binding|GO:0016705,oxidoreductase activity, acting on paired donors, with incorporation or reduction of molecular oxygen|GO:0016712,oxidoreductase activity, acting on paired donors, with incorporation or reduction of molecular oxygen, reduced flavin or flavoprotein as one donor, and incorporation of one atom of oxygen|GO:0003824,catalytic activity|GO:0019825,oxygen binding|GO:0043167,ion binding|GO:0005488,binding|GO:0005506,iron ion binding|GO:0008395,steroid hydroxylase activity|GO:0008392,arachidonic acid epoxygenase activity|GO:1901363,heterocyclic compound
MLXIP	GO:0051169,nuclear transport|GO:0080090,regulation of primary metabolic process|GO:0019222,regulation of metabolic process|GO:0031326,regulation of cellular biosynthetic process|GO:0100024,regulation of carbohydrate metabolic process by transcription from RNA polymerase II promoter|GO:0031323,regulation of cellular metabolic process|GO:0090304,nucleic acid metabolic process|GO:0044249,cellular biosynthetic process|GO:0034641,cellular nitrogen compound metabolic process|GO:0006807,nitrogen compound metabolic process|GO:0034645,cellular macromolecule biosynthetic process|GO:1901362,organic cyclic compound biosynthetic process|GO:0050789,regulation of biological process|GO:0097659,nucleic acid-templated transcription|GO:0032774,RNA biosynthetic process|GO:0006139,nucleobase-containing compound metabolic process|GO:0044260,cellular macromolecule metabolic process|GO:0071704,organic substance metabolic process|GO:2000112,regulation of cellular macromolecule biosynthetic process|GO:0051641,cellular localization|GO:0060255,regulation of macromolecule metabolic process|GO:0010467,gene expression|GO:0006357,regulation of transcription from RNA polymerase II promoter|GO:0065007,biological regulation|GO:1901360,organic cyclic compound metabolic process|GO:0006366,transcription from RNA polymerase II promoter|GO:0016482,cytosolic transport|GO:0010468,regulation of gene expression|GO:0018130,heterocycle biosynthetic process|GO:0006810,transport|GO:1901576,organic substance biosynthetic process|GO:0019219,regulation of nucleobase-containing compound metabolic process|GO:0006725,cellular aromatic compound metabolic process|GO:0009987,cellular process|GO:0009889,regulation of biosynthetic process|GO:0006913,nucleocytoplasmic transport|GO:1903506,regulation of nucleic acid-templated transcription|GO:0050794,regulation of cellular process|GO:0009058,biosynthetic process|GO:0009059,macromolecule biosynthetic process|GO:0051171,regulation of nitrogen compound metabolic process|GO:0008152,metabolic process|GO:2001141,regulation of RNA biosynthetic process|GO:0034654,nucleobase-containing compound biosynthetic process|GO:0005975,carbohydrate metabolic process|GO:0051234,establishment of localization|GO:0051179,localization|GO:0046483,heterocycle metabolic process|GO:0016070,RNA metabolic process|GO:0044238,primary metabolic process|GO:0044271,cellular nitrogen compound biosynthetic process|GO:0046907,intracellular transport|GO:1900402,regulation of carbohydrate metabolic process by regulation of transcription from RNA polymerase II promoter|GO:0051252,regulation of RNA metabolic process|GO:0044237,cellular metabolic process|GO:0043170,macromolecule metabolic process|GO:0006355,regulation of transcription, DNA-templated|GO:0010556,regulation of macromolecule biosynthetic process|GO:0006351,transcription, DNA-templated|GO:0019438,aromatic compound biosynthetic process|GO:0006109,regulation of carbohydrate metabolic process|GO:0051649,establishment of localization in cell|GO:1903508,positive regulation of nucleic acid-templated transcription|GO:0045935,positive regulation of nucleobase-containing compound metabolic process|GO:0010604,positive regulation of macromolecule metabolic process|GO:0031328,positive regulation of cellular biosynthetic process|GO:0031325,positive regulation of cellular metabolic process|GO:0009891,positive regulation of biosynthetic process|GO:0051254,positive regulation of RNA metabolic process|GO:1902680,positive regulation of RNA biosynthetic process|GO:0010557,positive regulation of macromolecule biosynthetic process|GO:0010628,positive regulation of gene expression|GO:0045944,positive regulation of transcription from RNA polymerase II promoter|GO:0045893,positive regulation of transcription, DNA-templated|GO:0051173,positive regulation of nitrogen compound metabolic process|GO:0048518,positive regulation of biological process|GO:0048522,positive regulation of cellular process|GO:0009893,positive regulation of metabolic process	GO:0031975,envelope|GO:0043229,intracellular organelle|GO:0043227,membrane-bounded organelle|GO:0043226,organelle|GO:0005737,cytoplasm|GO:0031090,organelle membrane|GO:0005634,nucleus|GO:0016020,membrane|GO:0005740,mitochondrial envelope|GO:0005739,mitochondrion|GO:0031968,organelle outer membrane|GO:0098588,bounding membrane of organelle|GO:0031967,organelle envelope|GO:0031966,mitochondrial membrane|GO:0043231,intracellular membrane-bounded organelle|GO:0044464,cell part|GO:0019867,outer membrane|GO:0005623,cell|GO:0005622,intracellular|GO:0044446,intracellular organelle part|GO:0044444,cytoplasmic part|GO:0005741,mitochondrial outer membrane|GO:0044429,mitochondrial part|GO:0044424,intracellular part|GO:0098805,whole membrane|GO:0044422,organelle part	GO:0003676,nucleic acid binding|GO:0003700,transcription factor activity, sequence-specific DNA binding|GO:0097159,organic cyclic compound binding|GO:0005488,binding|GO:0005515,protein binding|GO:0003677,DNA binding|GO:0046983,protein dimerization activity|GO:1901363,heterocyclic compound binding|GO:0001071,nucleic acid binding transcription factor activity|GO:0000989,transcription factor activity, transcription factor binding|GO:0000988,transcription factor activity, protein binding|GO:0044212,transcription regulatory region DNA binding|GO:1990837,sequence-specific double-stranded DNA binding|GO:0043565,sequence-specific DNA binding|GO:0001067,regulatory region nucleic acid binding|GO:0001012,RNA polymerase II regulatory region DNA binding|GO:0001228,transcriptional activator activity, RNA polymerase II transcription regulatory region sequence-specific binding|GO:0000976,transcription regulatory region sequence-specific DNA binding|GO:0000977,RNA polymerase II regulatory region sequence-specific DNA binding|GO:0000975,regulatory region DNA binding|GO:0003690,double-stranded DNA binding|GO:0000981,RNA polymerase II transcription factor activity, sequence-specific DNA binding
OR52E8	GO:0009593,detection of chemical stimulus|GO:0050906,detection of stimulus involved in sensory perception|GO:0007165,signal transduction|GO:0042221,response to chemical|GO:0050789,regulation of biological process|GO:0044699,single-organism process|GO:0051716,cellular response to stimulus|GO:0065007,biological regulation|GO:0007186,G-protein coupled receptor signaling pathway|GO:0032501,multicellular organismal process|GO:0007608,sensory perception of smell|GO:0050877,neurological system process|GO:0007606,sensory perception of chemical stimulus|GO:0007600,sensory perception|GO:0050794,regulation of cellular process|GO:0050911,detection of chemical stimulus involved in sensory perception of smell|GO:0044763,single-organism cellular process|GO:0007154,cell communication|GO:0003008,system process|GO:0044700,single organism signaling|GO:0051606,detection of stimulus|GO:0044707,single-multicellular organism process|GO:0050896,response to stimulus|GO:0023052,signaling|GO:0009987,cellular process|GO:0050907,detection of chemical stimulus involved in sensory perception	GO:0016021,integral component of membrane|GO:0016020,membrane|GO:0044464,cell part|GO:0005623,cell|GO:0071944,cell periphery|GO:0005886,plasma membrane|GO:0044425,membrane part|GO:0031224,intrinsic component of membrane	GO:0004930,G-protein coupled receptor activity|GO:0038023,signaling receptor activity|GO:0060089,molecular transducer activity|GO:0004888,transmembrane signaling receptor activity|GO:0004872,receptor activity|GO:0004871,signal transducer activity|GO:0004984,olfactory receptor activity|GO:0099600,transmembrane receptor activity
FCGR2C	GO:0044700,single organism signaling|GO:0051716,cellular response to stimulus|GO:0050896,response to stimulus|GO:0009987,cellular process|GO:0044763,single-organism cellular process|GO:0050794,regulation of cellular process|GO:0002376,immune system process|GO:0065007,biological regulation|GO:0023052,signaling|GO:0007165,signal transduction|GO:0006955,immune response|GO:0007154,cell communication|GO:0050789,regulation of biological process|GO:0044699,single-organism process	GO:0005737,cytoplasm|GO:0016021,integral component of membrane|GO:0016020,membrane|GO:0044464,cell part|GO:0005623,cell|GO:0005622,intracellular|GO:0071944,cell periphery|GO:0005886,plasma membrane|GO:0044424,intracellular part|GO:0044425,membrane part|GO:0031224,intrinsic component of membrane	GO:0038023,signaling receptor activity|GO:0005515,protein binding|GO:0019864,IgG binding|GO:0019865,immunoglobulin binding|GO:0060089,molecular transducer activity|GO:0004888,transmembrane signaling receptor activity|GO:0044877,macromolecular complex binding|GO:0004872,receptor activity|GO:0004871,signal transducer activity|GO:0032403,protein complex binding|GO:0005488,binding|GO:0099600,transmembrane receptor activity
TMEM143	GO:0032502,developmental process|GO:0002376,immune system process|GO:0048856,anatomical structure development|GO:0044707,single-multicellular organism process|GO:0009987,cellular process|GO:0048869,cellular developmental process|GO:0032501,multicellular organismal process|GO:0030154,cell differentiation|GO:0044767,single-organism developmental process|GO:0002244,hematopoietic progenitor cell differentiation|GO:0048513,animal organ development|GO:0044763,single-organism cellular process|GO:0048731,system development|GO:0030097,hemopoiesis|GO:0048534,hematopoietic or lymphoid organ development|GO:0007275,multicellular organism development|GO:0044699,single-organism process|GO:0002520,immune system development	GO:0005737,cytoplasm|GO:0043231,intracellular membrane-bounded organelle|GO:0016021,integral component of membrane|GO:0016020,membrane|GO:0044464,cell part|GO:0044444,cytoplasmic part|GO:0005739,mitochondrion|GO:0005622,intracellular|GO:0043229,intracellular organelle|GO:0005623,cell|GO:0044424,intracellular part|GO:0044425,membrane part|GO:0043227,membrane-bounded organelle|GO:0043226,organelle|GO:0031224,intrinsic component of membrane	

DNAJC19	GO:0033036,macromolecule localization|GO:0008104,protein localization|GO:0006457,protein folding|GO:0009893,positive regulation of metabolic process|GO:0019222,regulation of metabolic process|GO:0061458,reproductive system development|GO:0006810,transport|GO:0072655,establishment of protein localization to mitochondrion|GO:0030150,protein import into mitochondrial matrix|GO:0032501,multicellular organismal process|GO:0051641,cellular localization|GO:0072594,establishment of protein localization to organelle|GO:0007005,mitochondrion organization|GO:0007275,multicellular organism development|GO:0043085,positive regulation of catalytic activity|GO:0051179,localization|GO:0044267,cellular protein metabolic process|GO:0043462,regulation of ATPase activity|GO:0071806,protein transmembrane transport|GO:0051345,positive regulation of hydrolase activity|GO:0071704,organic substance metabolic process|GO:0071840,cellular component organization or biogenesis|GO:0016043,cellular component organization|GO:0050789,regulation of biological process|GO:0065002,intracellular protein transmembrane transport|GO:1990542,mitochondrial transmembrane transport|GO:0048513,animal organ development|GO:0048806,genitalia development|GO:1902578,single-organism localization|GO:0044093,positive regulation of molecular function|GO:0050877,neurological system process|GO:0048518,positive regulation of biological process|GO:0065007,biological regulation|GO:0065009,regulation of molecular function|GO:0016482,cytosolic transport|GO:0034613,cellular protein localization|GO:0032502,developmental process|GO:0006996,organelle organization|GO:0017038,protein import|GO:0048608,reproductive structure development|GO:0070585,protein localization to mitochondrion|GO:0045184,establishment of protein localization|GO:0032781,positive regulation of ATPase activity|GO:0006626,protein targeting to mitochondrion|GO:0050790,regulation of catalytic activity|GO:0007601,visual perception|GO:0007600,sensory perception|GO:0050953,sensory perception of light stimulus|GO:0006605,protein targeting|GO:0044767,single-organism developmental process|GO:0003006,developmental process involved in reproduction|GO:0044765,single-organism transport|GO:0044763,single-organism cellular process|GO:0071702,organic substance transport|GO:0008152,metabolic process|GO:1902582,single-organism intracellular transport|GO:0006839,mitochondrial transport|GO:0007548,sex differentiation|GO:0051234,establishment of localization|GO:0055085,transmembrane transport|GO:0044743,protein transmembrane import into intracellular organelle|GO:0003008,system process|GO:0051336,regulation of hydrolase activity|GO:0044238,primary metabolic process|GO:0044699,single-organism process|GO:0044702,single organism reproductive process|GO:0044260,cellular macromolecule metabolic process|GO:0044707,single-multicellular organism process|GO:0022414,reproductive process|GO:0019538,protein metabolic process|GO:0048856,anatomical structure development|GO:0044237,cellular metabolic process|GO:0043170,macromolecule metabolic process|GO:0033365,protein localization to organelle|GO:0070727,cellular macromolecule localization|GO:0046907,intracellular transport|GO:0015031,protein transport|GO:0048731,system development|GO:0009987,cellular process|GO:1902580,single-organism cellular localization|GO:0006886,intracellular protein transport|GO:0051649,establishment of localization in cell|GO:0015833,peptide transport|GO:0042886,amide transport|GO:0000003,reproduction|GO:0071705,nitrogen compound transport	GO:0001405,presequence translocase-associated import motor|GO:0031975,envelope|GO:0043229,intracellular organelle|GO:0043227,membrane-bounded organelle|GO:0043226,organelle|GO:0031224,intrinsic component of membrane|GO:0005737,cytoplasm|GO:0031090,organelle membrane|GO:0005743,mitochondrial inner membrane|GO:0016021,integral component of membrane|GO:0016020,membrane|GO:0044444,cytoplasmic part|GO:0005739,mitochondrion|GO:0098800,inner mitochondrial membrane protein complex|GO:0044455,mitochondrial membrane part|GO:0044464,cell part|GO:0031967,organelle envelope|GO:0031966,mitochondrial membrane|GO:0043234,protein complex|GO:0032991,macromolecular complex|GO:0043231,intracellular membrane-bounded organelle|GO:0019866,organelle inner membrane|GO:0005623,cell|GO:0005622,intracellular|GO:0044446,intracellular organelle part|GO:0098796,membrane protein complex|GO:0005740,mitochondrial envelope|GO:0044429,mitochondrial part|GO:0005744,mitochondrial inner membrane presequence translocase complex|GO:0044424,intracellular part|GO:0044425,membrane part|GO:0044422,organelle part|GO:0098798,mitochondrial protein complex	GO:0008565,protein transporter activity|GO:0022892,substrate-specific transporter activity|GO:0005515,protein binding|GO:0005215,transporter activity|GO:0030234,enzyme regulator activity|GO:0008047,enzyme activator activity|GO:0060590,ATPase regulator activity|GO:0005488,binding|GO:0098772,molecular function regulator|GO:0060589,nucleoside-triphosphatase regulator activity|GO:0001671,ATPase activator activity
ICOS	GO:0016337,single organismal cell-cell adhesion|GO:0034112,positive regulation of homotypic cell-cell adhesion|GO:0034110,regulation of homotypic cell-cell adhesion|GO:0050865,regulation of cell activation|GO:0050867,positive regulation of cell activation|GO:0006955,immune response|GO:0050863,regulation of T cell activation|GO:0022407,regulation of cell-cell adhesion|GO:0032501,multicellular organismal process|GO:0050789,regulation of biological process|GO:0044699,single-organism process|GO:0022409,positive regulation of cell-cell adhesion|GO:0002517,T cell tolerance induction|GO:0051249,regulation of lymphocyte activation|GO:0045785,positive regulation of cell adhesion|GO:0007275,multicellular organism development|GO:1903039,positive regulation of leukocyte cell-cell adhesion|GO:0002684,positive regulation of immune system process|GO:0002682,regulation of immune system process|GO:0048518,positive regulation of biological process|GO:0065007,biological regulation|GO:0071593,lymphocyte aggregation|GO:0030155,regulation of cell adhesion|GO:0031295,T cell costimulation|GO:1903037,regulation of leukocyte cell-cell adhesion|GO:0032502,developmental process|GO:0098602,single organism cell adhesion|GO:0098609,cell-cell adhesion|GO:0034109,homotypic cell-cell adhesion|GO:0009987,cellular process|GO:0050870,positive regulation of T cell activation|GO:0050794,regulation of cellular process|GO:0044767,single-organism developmental process|GO:0001775,cell activation|GO:0046649,lymphocyte activation|GO:0070489,T cell aggregation|GO:0042110,T cell activation|GO:0048731,system development|GO:0007155,cell adhesion|GO:0070486,leukocyte aggregation|GO:0002520,immune system development|GO:0002507,tolerance induction|GO:0022610,biological adhesion|GO:0044707,single-multicellular organism process|GO:0045321,leukocyte activation|GO:0051251,positive regulation of lymphocyte activation|GO:0050896,response to stimulus|GO:0048856,anatomical structure development|GO:0044763,single-organism cellular process|GO:0002376,immune system process|GO:0002694,regulation of leukocyte activation|GO:0002696,positive regulation of leukocyte activation|GO:0007159,leukocyte cell-cell adhesion|GO:0031294,lymphocyte costimulation|GO:0048522,positive regulation of cellular process|GO:0048583,regulation of response to stimulus|GO:0007165,signal transduction|GO:0044710,single-organism metabolic process|GO:0009966,regulation of signal transduction|GO:0046488,phosphatidylinositol metabolic process|GO:0046486,glycerolipid metabolic process|GO:0044700,single organism signaling|GO:0019637,organophosphate metabolic process|GO:0035556,intracellular signal transduction|GO:0046854,phosphatidylinositol phosphorylation|GO:0006629,lipid metabolic process|GO:0051716,cellular response to stimulus|GO:0008152,metabolic process|GO:1902531,regulation of intracellular signal transduction|GO:0048015,phosphatidylinositol-mediated signaling|GO:0048017,inositol lipid-mediated signaling|GO:0016310,phosphorylation|GO:0023052,signaling|GO:0023051,regulation of signaling|GO:0010646,regulation of cell communication|GO:0046834,lipid phosphorylation|GO:0006644,phospholipid metabolic process|GO:0014065,phosphatidylinositol 3-kinase signaling|GO:0044255,cellular lipid metabolic process|GO:0014066,regulation of phosphatidylinositol 3-kinase signaling|GO:0030258,lipid modification|GO:0006650,glycerophospholipid metabolic process|GO:0071704,organic substance metabolic process|GO:0007154,cell communication|GO:0044238,primary metabolic process|GO:0044237,cellular metabolic process|GO:0006796,phosphate-containing compound metabolic process|GO:0006793,phosphorus metabolic process|GO:0043491,protein kinase B signaling|GO:0048584,positive regulation of response to stimulus|GO:0023056,positive regulation of signaling|GO:0009967,positive regulation of signal transduction|GO:1902533,positive regulation of intracellular signal transduction|GO:0010647,positive regulation of cell communication|GO:0051897,positive regulation of protein kinase B signaling|GO:0051896,regulation of protein kinase B signaling	GO:0009897,external side of plasma membrane|GO:0016020,membrane|GO:0031226,intrinsic component of plasma membrane|GO:0016021,integral component of membrane|GO:0098552,side of membrane|GO:0044464,cell part|GO:0005623,cell|GO:0071944,cell periphery|GO:0005887,integral component of plasma membrane|GO:0005886,plasma membrane|GO:0044425,membrane part|GO:0044459,plasma membrane part|GO:0009986,cell surface|GO:0031224,intrinsic component of membrane|GO:0005576,extracellular region|GO:0005622,intracellular	GO:0046934,phosphatidylinositol-4,5-bisphosphate 3-kinase activity|GO:0003824,catalytic activity|GO:0016773,phosphotransferase activity, alcohol group as acceptor|GO:0016772,transferase activity, transferring phosphorus-containing groups|GO:0016301,kinase activity|GO:0052813,phosphatidylinositol bisphosphate kinase activity|GO:0016740,transferase activity|GO:0035004,phosphatidylinositol 3-kinase activity
FOXP3	GO:0051046,regulation of secretion|GO:0051048,negative regulation of secretion|GO:0051049,regulation of transport|GO:0002707,negative regulation of lymphocyte mediated immunity|GO:0002706,regulation of lymphocyte mediated immunity|GO:0002705,positive regulation of leukocyte mediated immunity|GO:0002704,negative regulation of leukocyte mediated immunity|GO:0042035,regulation of cytokine biosynthetic process|GO:0002701,negative regulation of production of molecular mediator of immune response|GO:0042036,negative regulation of cytokine biosynthetic process|GO:0002709,regulation of T cell mediated immunity|GO:0002708,positive regulation of lymphocyte mediated immunity|GO:0051716,cellular response to stimulus|GO:0043207,response to external biotic stimulus|GO:0071604,transforming growth factor beta production|GO:0065007,biological regulation|GO:0006473,protein acetylation|GO:0006475,internal protein amino acid acetylation|GO:0002824,positive regulation of adaptive immune response based on somatic recombination of immune receptors built from immunoglobulin superfamily domains|GO:0002822,regulation of adaptive immune response based on somatic recombination of immune receptors built from immunoglobulin superfamily domains|GO:0002823,negative regulation of adaptive immune response based on somatic recombination of immune receptors built from immunoglobulin superfamily domains|GO:0002820,negative regulation of adaptive immune response|GO:0002821,positive regulation of adaptive immune response|GO:0002677,negative regulation of chronic inflammatory response|GO:0002676,regulation of chronic inflammatory response|GO:0046483,heterocycle metabolic process|GO:0065008,regulation of biological quality|GO:0009607,response to biotic stimulus|GO:0042089,cytokine biosynthetic process|GO:0009605,response to external stimulus|GO:0019538,protein metabolic process|GO:0002369,T cell cytokine production|GO:0010638,positive regulation of organelle organization|GO:0010639,negative regulation of organelle organization|GO:0002703,regulation of leukocyte mediated immunity|GO:0098609,cell-cell adhesion|GO:0045829,negative regulation of isotype switching|GO:0009892,negative regulation of metabolic process|GO:0009893,positive regulation of metabolic process|GO:0009890,negative regulation of biosynthetic process|GO:0009891,positive regulation of biosynthetic process|GO:0002700,regulation of production of molecular mediator of immune response|GO:0032792,negative regulation of CREB transcription factor activity|GO:0050671,positive regulation of lymphocyte proliferation|GO:0046651,lymphocyte proliferation|GO:0051223,regulation of protein transport|GO:0051224,negative regulation of protein transport|GO:0050789,regulation of biological process|GO:2000398,regulation of thymocyte aggregation|GO:0032700,negative regulation of interleukin-17 production|GO:0002684,positive regulation of immune system process|GO:0032703,negative regulation of interleukin-2 production|GO:0002682,regulation of immune system process|GO:0002683,negative regulation of immune system process|GO:1903308,regulation of chromatin modification|GO:1903309,negative regulation of chromatin modification|GO:0045910,negative regulation of DNA recombination|GO:0018130,heterocycle biosynthetic process|GO:0070201,regulation of establishment of protein localization|GO:0098602,single organism cell adhesion|GO:0033077,T cell differentiation in thymus|GO:0033079,immature T cell proliferation|GO:0043412,macromolecule modification|GO:0009059,macromolecule biosynthetic process|GO:0002521,leukocyte differentiation|GO:0002520,immune system development|GO:0016070,RNA metabolic process|GO:0002293,alpha-beta T cell differentiation involved in immune response|GO:0002292,T cell differentiation involved in immune response|GO:0002294,CD4-positive, alpha-beta T cell differentiation involved in immune response|GO:0010557,positive regulation of macromolecule biosynthetic process|GO:0010556,regulation of macromolecule biosynthetic process|GO:2001252,positive regulation of chromosome organization|GO:0010558,negative regulation of macromolecule biosynthetic process|GO:2001251,negative regulation of chromosome organization|GO:0032609,interferon-gamma production|GO:0051129,negative regulation of cellular component organization|GO:0051128,regulation of cellular component organization|GO:0098732,macromolecule deacylation|GO:0006955,immune response|GO:0072538,T-helper 17 type immune response|GO:0000122,negative regulation of transcription from RNA polymerase II promoter|GO:0045076,regulation of interleukin-2 biosynthetic process|GO:0045077,negative regulation of interferon-gamma biosynthetic process|GO:0045072,regulation of interferon-gamma biosynthetic process|GO:0002286,T cell activation involved in immune response|GO:0045621,positive regulation of lymphocyte differentiation|GO:0045620,negative regulation of lymphocyte differentiation|GO:0045623,negative regulation of T-helper cell differentiation|GO:0032673,regulation of interleukin-4 production|GO:0032674,regulation of interleukin-5 production|GO:0032675,regulation of interleukin-6 production|GO:0008284,positive regulation of cell proliferation|GO:0008285,negative regulation of cell proliferation|GO:0035601,protein deacylation|GO:0008283,cell proliferation|GO:0050870,positive regulation of T cell activation|GO:0002637,regulation of immunoglobulin production|GO:0002440,production of molecular mediator of immune response|GO:0002638,negative regulation of immunoglobulin production|GO:0006338,chromatin remodeling|GO:0002870,T cell anergy|GO:0060341,regulation of cellular localization|GO:0042592,homeostatic process|GO:0022407,regulation of cell-cell adhesion|GO:0032635,interleukin-6 production|GO:0007275,multicellular organism development|GO:0022408,negative regulation of cell-cell adhesion|GO:0022409,positive regulation of cell-cell adhesion|GO:0002643,regulation of tolerance induction|GO:0002645,positive regulation of tolerance induction|GO:2000112,regulation of cellular macromolecule biosynthetic process|GO:2000113,negative regulation of cellular macromolecule biosynthetic process|GO:0070661,leukocyte proliferation|GO:0070663,regulation of leukocyte proliferation|GO:0002696,positive regulation of leukocyte activation|GO:0070665,positive regulation of leukocyte proliferation|GO:0070664,negative regulation of leukocyte proliferation|GO:0045085,negative regulation of interleukin-2 biosynthetic process|GO:0032914,positive regulation of transforming growth factor beta1 production|GO:0090311,regulation of protein deacetylation|GO:0006464,cellular protein modification process|GO:0044767,single-organism developmental process|GO:0044765,single-organism transport|GO:0044763,single-organism cellular process|GO:0033091,positive regulation of immature T cell proliferation|GO:0051276,chromosome organization|GO:0048856,anatomical structure development|GO:0002698,negative regulation of immune effector process|GO:1902107,positive regulation of leukocyte differentiation|GO:1902106,negative regulation of leukocyte differentiation|GO:1902105,regulation of leukocyte differentiation|GO:2000026,regulation of multicellular organismal development|GO:2000756,regulation of peptidyl-lysine acetylation|GO:2000757,negative regulation of peptidyl-lysine acetylation|GO:0032753,positive regulation of interleukin-4 production|GO:0048523,negative regulation of cellular process|GO:0048522,positive regulation of cellular process|GO:0042130,negative regulation of T cell proliferation|GO:0033089,positive regulation of T cell differentiation in thymus|GO:0032620,interleukin-17 production|GO:0034112,positive regulation of homotypic cell-cell adhesion|GO:0034111,negative regulation of homotypic cell-cell adhesion|GO:0034110,regulation of homotypic cell-cell adhesion|GO:0033080,immature T cell proliferation in thymus|GO:0033081,regulation of T cell differentiation in thymus|GO:0033083,regulation of immature T cell proliferation|GO:0033084,regulation of immature T cell proliferation in thymus|GO:0031348,negative regulation of defense response|GO:0007162,negative regulation of cell adhesion|GO:0007165,signal transduction|GO:0007166,cell surface receptor signaling pathway|GO:0002458,peripheral T cell tolerance induction|GO:0002456,T cell mediated immunity|GO:0031347,regulation of defense response|GO:0002517,T cell tolerance induction|GO:0050728,negative regulation of inflammatory response|GO:0002513,tolerance induction to self antigen|GO:0045785,positive regulation of cell adhesion|GO:0002260,lymphocyte homeostasis|GO:0002262,myeloid cell homeostasis|GO:0032101,regulation of response to external stimulus|GO:0050727,regulation of inflammatory response|GO:0048294,negative [illegible]	GO:0043234,protein complex|GO:0005737,cytoplasm|GO:0032991,macromolecular complex|GO:0043231,intracellular membrane-bounded organelle|GO:0005634,nucleus|GO:0044464,cell part|GO:0005623,cell|GO:0005622,intracellular|GO:0043229,intracellular organelle|GO:0044424,intracellular part|GO:0043227,membrane-bounded organelle|GO:0043226,organelle|GO:0044446,intracellular organelle part|GO:0031981,nuclear lumen|GO:0043233,organelle lumen|GO:0031974,membrane-enclosed lumen|GO:0005654,nucleoplasm|GO:0070013,intracellular organelle lumen|GO:0044428,nuclear part|GO:0044422,organelle part	GO:0019899,enzyme binding|GO:0005488,binding|GO:0003676,nucleic acid binding|GO:0003677,DNA binding|GO:1901363,heterocyclic compound binding|GO:0043565,sequence-specific DNA binding|GO:0051525,NFAT protein binding|GO:0003714,transcription corepressor activity|GO:0003712,transcription cofactor activity|GO:0043167,ion binding|GO:0000989,transcription factor activity, transcription factor binding|GO:0000988,transcription factor activity, protein binding|GO:0046872,metal ion binding|GO:0042826,histone deacetylase binding|GO:0042802,identical protein binding|GO:0042803,protein homodimerization activity|GO:0043169,cation binding|GO:0008134,transcription factor binding|GO:0001067,regulatory region nucleic acid binding|GO:0035035,histone acetyltransferase binding|GO:0051059,NF-kappaB binding|GO:0001047,core promoter binding|GO:0044212,transcription regulatory region DNA binding|GO:0003700,transcription factor activity, sequence-specific DNA binding|GO:0097159,organic cyclic compound binding|GO:0003705,transcription factor activity, RNA polymerase II distal enhancer sequence-specific binding|GO:0000981,RNA polymerase II transcription factor activity, sequence-specific DNA binding|GO:0005515,protein binding|GO:0000975,regulatory region DNA binding|GO:0046983,protein dimerization activity|GO:0001071,nucleic acid binding transcription factor activity|GO:1990837,sequence-specific double-stranded DNA binding|GO:0001012,RNA polymerase II regulatory region DNA binding|GO:0001159,core promoter proximal region DNA binding|GO:0000976,transcription regulatory region sequence-specific DNA binding|GO:0000977,RNA polymerase II regulatory region sequence-specific DNA binding|GO:0000987,core promoter proximal region sequence-specific DNA binding|GO:0000978,RNA polymerase II core promoter proximal region sequence-specific DNA binding|GO:0003690,double-stranded DNA binding
TP73	GO:0019220,regulation of phosphate metabolic process|GO:0051716,cellular response to stimulus|GO:0048589,developmental growth|GO:0000165,MAPK cascade|GO:0006281,DNA repair|GO:0060548,negative regulation of cell death|GO:0045859,regulation of protein kinase activity|GO:0008361,regulation of cell size|GO:0046483,heterocycle metabolic process|GO:0042325,regulation of phosphorylation|GO:0042327,positive regulation of phosphorylation|GO:0042326,negative regulation of phosphorylation|GO:0019538,protein metabolic process|GO:0072358,cardiovascular system development|GO:0021537,telencephalon development|GO:0009892,negative regulation of metabolic process|GO:0009893,positive regulation of metabolic process|GO:0009890,negative regulation of biosynthetic process|GO:0009891,positive regulation of biosynthetic process|GO:0071902,positive regulation of protein serine/threonine kinase activity|GO:0035556,intracellular signal transduction|GO:0071900,regulation of protein serine/threonine kinase activity|GO:0072331,signal transduction by p53 class mediator|GO:0050789,regulation of biological process|GO:0072332,intrinsic apoptotic signaling pathway by p53 class mediator|GO:0051347,positive regulation of transferase activity|GO:0051348,negative regulation of transferase activity|GO:1901360,organic cyclic compound metabolic process|GO:0018130,heterocycle biosynthetic process|GO:1901861,regulation of muscle tissue development|GO:1901862,negative regulation of muscle tissue development|GO:0048709,oligodendrocyte differentiation|GO:0043410,positive regulation of MAPK cascade|GO:0021543,pallium development|GO:0043412,macromolecule modification|GO:0016070,RNA metabolic process|GO:0010557,positive regulation of macromolecule biosynthetic process|GO:0010556,regulation of macromolecule biosynthetic process|GO:0048869,cellular developmental process|GO:0010558,negative regulation of macromolecule biosynthetic process|GO:0048638,regulation of developmental growth|GO:0048635,negative regulation of muscle organ development|GO:0048634,regulation of muscle organ development|GO:0021766,hippocampus development|GO:0009411,response to UV|GO:1901991,negative regulation of mitotic cell cycle phase transition|GO:1901990,regulation of mitotic cell cycle phase transition|GO:0010001,glial cell differentiation|GO:0021761,limbic system development|GO:0044819,mitotic G1/S transition checkpoint|GO:1901214,regulation of neuron death|GO:0051234,establishment of localization|GO:0000122,negative regulation of transcription from RNA polymerase II promoter|GO:0060284,regulation of cell development|GO:0022008,neurogenesis|GO:0008285,negative regulation of cell proliferation|GO:0035239,tube morphogenesis|GO:0050878,regulation of body fluid levels|GO:0008283,cell proliferation|GO:0006974,cellular response to DNA damage stimulus|GO:0001655,urogenital system development|GO:0006978,DNA damage response, signal transduction by p53 class mediator resulting in transcription of p21 class mediator|GO:0044699,single-organism process|GO:0045926,negative regulation of growth|GO:0097190,apoptotic signaling pathway|GO:0097193,intrinsic apoptotic signaling pathway|GO:0022402,cell cycle process|GO:0008219,cell death|GO:0007275,multicellular organism development|GO:2000112,regulation of cellular macromolecule biosynthetic process|GO:2000113,negative regulation of cellular macromolecule biosynthetic process|GO:0043065,positive regulation of apoptotic process|GO:0043067,regulation of programmed cell death|GO:0043066,negative regulation of apoptotic process|GO:0043069,negative regulation of programmed cell death|GO:0097285,obsolete cell-type specific apoptotic process|GO:0006468,protein phosphorylation|GO:0006469,negative regulation of protein kinase activity|GO:0000278,mitotic cell cycle|GO:0019219,regulation of nucleobase-containing compound metabolic process|GO:0006461,protein complex assembly|GO:0006464,cellular protein modification process|GO:0044767,single-organism developmental process|GO:0044765,single-organism transport|GO:0044764,multi-organism cellular process|GO:0044763,single-organism cellular process|GO:0048856,anatomical structure development|GO:0006796,phosphate-containing compound metabolic process|GO:2000026,regulation of multicellular organismal development|GO:0006793,phosphorus metabolic process|GO:0014855,striated muscle cell proliferation|GO:0048523,negative regulation of cellular process|GO:0048522,positive regulation of cellular process|GO:0033326,cerebrospinal fluid secretion|GO:0000082,G1/S transition of mitotic cell cycle|GO:0032147,activation of protein kinase activity|GO:0043523,regulation of neuron apoptotic process|GO:0043524,negative regulation of neuron apoptotic process|GO:0007165,signal transduction|GO:0044710,single-organism metabolic process|GO:0045786,negative regulation of cell cycle|GO:0045665,negative regulation of neuron differentiation|GO:0045664,regulation of neuron differentiation|GO:0044093,positive regulation of molecular function|GO:1901798,positive regulation of signal transduction by p53 class mediator|GO:1901796,regulation of signal transduction by p53 class mediator|GO:0016202,regulation of striated muscle tissue development|GO:1902229,regulation of intrinsic apoptotic signaling pathway in response to DNA damage|GO:2001141,regulation of RNA biosynthetic process|GO:2001020,regulation of response to DNA damage stimulus|GO:2001022,positive regulation of response to DNA damage stimulus|GO:0010033,response to organic substance|GO:0051704,multi-organism process|GO:0043407,negative regulation of MAP kinase activity|GO:0044249,cellular biosynthetic process|GO:0043068,positive regulation of programmed cell death|GO:1902255,positive regulation of intrinsic apoptotic signaling pathway by p53 class mediator|GO:1902253,regulation of intrinsic apoptotic signaling pathway by p53 class mediator|GO:0045843,negative regulation of striated muscle tissue development|GO:0034644,cellular response to UV|GO:0006298,mismatch repair|GO:0010629,negative regulation of gene expression|GO:0010628,positive regulation of gene expression|GO:0046329,negative regulation of JNK cascade|GO:0046328,regulation of JNK cascade|GO:0044267,cellular protein metabolic process|GO:0010646,regulation of cell communication|GO:0044260,cellular macromolecule metabolic process|GO:0070997,neuron death|GO:0007589,body fluid secretion|GO:0007049,cell cycle|GO:0006366,transcription from RNA polymerase II promoter|GO:0050793,regulation of developmental process|GO:0050790,regulation of catalytic activity|GO:0009889,regulation of biosynthetic process|GO:0009888,tissue development|GO:0050794,regulation of cellular process|GO:0051239,regulation of multicellular organismal process|GO:1902533,positive regulation of intracellular signal transduction|GO:1901215,negative regulation of neuron death|GO:1902679,negative regulation of RNA biosynthetic process|GO:0050896,response to stimulus|GO:0051338,regulation of transferase activity|GO:0051962,positive regulation of nervous system development|GO:0051961,negative regulation of nervous system development|GO:0051960,regulation of nervous system development|GO:0010562,positive regulation of phosphorus metabolic process|GO:0010563,negative regulation of phosphorus metabolic process|GO:0071482,cellular response to light stimulus|GO:0070271,protein complex biogenesis|GO:0051246,regulation of protein metabolic process|GO:0031098,stress-activated protein kinase signaling cascade|GO:0055123,digestive system development|GO:0043406,positive regulation of MAP kinase activity|GO:0043405,regulation of MAP kinase activity|GO:0044843,cell cycle G1/S phase transition|GO:0048565,digestive tract development|GO:0043409,negative regulation of MAPK cascade|GO:0043408,regulation of MAPK cascade|GO:0050767,regulation of neurogenesis|GO:0051248,negative regulation of protein metabolic process|GO:0071478,cellular response to radiation|GO:0010212,response to ionizing radiation|GO:0051240,positive regulation of multicellular organismal process|GO:0051241,negative regulation of multicellular organismal process|GO:0010564,regulation of cell cycle process|GO:0051247,positive regulation of protein metabolic process|GO:0050768,negative regulation of neurogenesis|GO:0050769,positive regulation of neurogenesis|GO:0031399,regulation of protein modification process|GO:2001244,positive regulation of intrinsic apoptotic signaling pathway|GO:2001242,regulation of intrinsic apoptotic signaling pathway|GO:0070302,regulation of stress-activated protein kinase signaling cascade|GO:0070303,negative regulation of stress-activated protein kinase signaling cascade|GO:0009416,response to light stimulus|GO:0042493,response to [illegible]	GO:0031974,membrane-enclosed lumen|GO:0043229,intracellular organelle|GO:0043228,non-membrane-bounded organelle|GO:0000785,chromatin|GO:0043227,membrane-bounded organelle|GO:0043226,organelle|GO:0030054,cell junction|GO:0005737,cytoplasm|GO:0031981,nuclear lumen|GO:0005634,nucleus|GO:0005654,nucleoplasm|GO:0005694,chromosome|GO:0005794,Golgi apparatus|GO:0012505,endomembrane system|GO:0043234,protein complex|GO:0032991,macromolecular complex|GO:0043231,intracellular membrane-bounded organelle|GO:0043232,intracellular non-membrane-bounded organelle|GO:0043233,organelle lumen|GO:0005829,cytosol|GO:0044464,cell part|GO:0005623,cell|GO:0005622,intracellular|GO:0044446,intracellular organelle part|GO:0070013,intracellular organelle lumen|GO:0044444,cytoplasmic part|GO:0044428,nuclear part|GO:0005667,transcription factor complex|GO:0044424,intracellular part|GO:0044427,chromosomal part|GO:0044422,organelle part|GO:0005739,mitochondrion	GO:0001012,RNA polymerase II regulatory region DNA binding|GO:0001159,core promoter proximal region DNA binding|GO:0001077,transcriptional activator activity, RNA polymerase II core promoter proximal region sequence-specific binding|GO:0044877,macromolecular complex binding|GO:0005488,binding|GO:0003676,nucleic acid binding|GO:0003677,DNA binding|GO:1901363,heterocyclic compound binding|GO:0043565,sequence-specific DNA binding|GO:0043566,structure-specific DNA binding|GO:0043167,ion binding|GO:0000987,core promoter proximal region sequence-specific DNA binding|GO:0003690,double-stranded DNA binding|GO:0046872,metal ion binding|GO:0000982,transcription factor activity, RNA polymerase II core promoter proximal region sequence-specific binding|GO:0042802,identical protein binding|GO:0043169,cation binding|GO:0008134,transcription factor binding|GO:0001067,regulatory region nucleic acid binding|GO:0019901,protein kinase binding|GO:0019900,kinase binding|GO:0019899,enzyme binding|GO:0044212,transcription regulatory region DNA binding|GO:0003700,transcription factor activity, sequence-specific DNA binding|GO:0097159,organic cyclic compound binding|GO:0001071,nucleic acid binding transcription factor activity|GO:0001228,transcriptional activator activity, RNA polymerase II transcription regulatory region sequence-specific binding|GO:0000976,transcription regulatory region sequence-specific DNA binding|GO:0000977,RNA polymerase II regulatory region sequence-specific DNA binding|GO:0005515,protein binding|GO:0000975,regulatory region DNA binding|GO:0000978,RNA polymerase II core promoter proximal region sequence-specific DNA binding|GO:0003682,chromatin binding|GO:0002039,p53 binding|GO:0003684,damaged DNA binding|GO:0000981,RNA polymerase II transcription factor activity, sequence-specific DNA binding|GO:1990837,sequence-specific double-stranded DNA binding|GO:0097371,MDM2/MDM4 family protein binding
FAM109B	GO:0008152,metabolic process|GO:0023052,signaling|GO:0023051,regulation of signaling|GO:0001881,receptor recycling|GO:0050789,regulation of biological process|GO:0071840,cellular component organization or biogenesis|GO:0044260,cellular macromolecule metabolic process|GO:0010256,endomembrane system organization|GO:0016043,cellular component organization|GO:0071704,organic substance metabolic process|GO:0065007,biological regulation|GO:0044699,single-organism process|GO:0051641,cellular localization|GO:0007032,endosome organization|GO:0006810,transport|GO:0009987,cellular process|GO:0044765,single-organism transport|GO:0042147,retrograde transport, endosome to Golgi|GO:0051649,establishment of localization in cell|GO:0051234,establishment of localization|GO:0051179,localization|GO:1902578,single-organism localization|GO:0016192,vesicle-mediated transport|GO:0016197,endosomal transport|GO:0006996,organelle organization|GO:0046907,intracellular transport|GO:0044237,cellular metabolic process|GO:0043170,macromolecule metabolic process|GO:1902582,single-organism intracellular transport|GO:0043112,receptor metabolic process|GO:0016050,vesicle organization|GO:0016482,cytosolic transport	GO:0030135,coated vesicle|GO:0055037,recycling endosome|GO:0043229,intracellular organelle|GO:0043227,membrane-bounded organelle|GO:0043226,organelle|GO:0030136,clathrin-coated vesicle|GO:0005737,cytoplasm|GO:0031984,organelle subcompartment|GO:0031982,vesicle|GO:0016023,cytoplasmic, membrane-bounded vesicle|GO:0031410,cytoplasmic vesicle|GO:0031988,membrane-bounded vesicle|GO:0044431,Golgi apparatus part|GO:0005794,Golgi apparatus|GO:0012505,endomembrane system|GO:0043231,intracellular membrane-bounded organelle|GO:0005802,trans-Golgi network|GO:0044464,cell part|GO:0005623,cell|GO:0005622,intracellular|GO:0044446,intracellular organelle part|GO:0044444,cytoplasmic part|GO:0098791,Golgi subcompartment|GO:0044424,intracellular part|GO:0005768,endosome|GO:0005769,early endosome|GO:0044422,organelle part|GO:0097708,intracellular vesicle|GO:0005829,cytosol	GO:0005488,binding|GO:0005515,protein binding|GO:0046983,protein dimerization activity|GO:0042802,identical protein binding|GO:0042803,protein homodimerization activity
ABHD6	GO:0080090,regulation of primary metabolic process|GO:0019222,regulation of metabolic process|GO:0048583,regulation of response to stimulus|GO:0008277,regulation of G-protein coupled receptor protein signaling pathway|GO:0007165,signal transduction|GO:0046434,organophosphate catabolic process|GO:0044712,single-organism catabolic process|GO:0051716,cellular response to stimulus|GO:0044711,single-organism biosynthetic process|GO:0071926,endocannabinoid signaling pathway|GO:0009966,regulation of signal transduction|GO:0045834,positive regulation of lipid metabolic process|GO:0048518,positive regulation of biological process|GO:0048519,negative regulation of biological process|GO:0046503,glycerolipid catabolic process|GO:0038171,cannabinoid signaling pathway|GO:0042221,response to chemical|GO:0046486,glycerolipid metabolic process|GO:0010033,response to organic substance|GO:0044700,single organism signaling|GO:2000124,regulation of endocannabinoid signaling pathway|GO:0048870,cell motility|GO:0019637,organophosphate metabolic process|GO:0009893,positive regulation of metabolic process|GO:0009891,positive regulation of biosynthetic process|GO:0006928,movement of cell or subcellular component|GO:0048167,regulation of synaptic plasticity|GO:0051674,localization of cell|GO:0050789,regulation of biological process|GO:1901576,organic substance biosynthetic process|GO:1901575,organic substance catabolic process|GO:0016042,lipid catabolic process|GO:0065007,biological regulation|GO:0016477,cell migration|GO:0007186,G-protein coupled receptor signaling pathway|GO:0006629,lipid metabolic process|GO:0009889,regulation of biosynthetic process|GO:0044710,single-organism metabolic process|GO:0050794,regulation of cellular process|GO:0008152,metabolic process|GO:0050896,response to stimulus|GO:2000145,regulation of cell motility|GO:2000146,negative regulation of cell motility|GO:0050803,regulation of synapse structure or activity|GO:0006639,acylglycerol metabolic process|GO:0006638,neutral lipid metabolic process|GO:0050805,negative regulation of synaptic transmission|GO:0050804,modulation of synaptic transmission|GO:0044248,cellular catabolic process|GO:0023057,negative regulation of signaling|GO:0023052,signaling|GO:0010648,negative regulation of cell communication|GO:0023051,regulation of signaling|GO:0044242,cellular lipid catabolic process|GO:0010646,regulation of cell communication|GO:0044699,single-organism process|GO:0009395,phospholipid catabolic process|GO:0046464,acylglycerol catabolic process|GO:0046461,neutral lipid catabolic process|GO:1901700,response to oxygen-containing compound|GO:0006644,phospholipid metabolic process|GO:0009987,cellular process|GO:0040013,negative regulation of locomotion|GO:0051270,regulation of cellular component movement|GO:0046889,positive regulation of lipid biosynthetic process|GO:0044255,cellular lipid metabolic process|GO:0032879,regulation of localization|GO:0065008,regulation of biological quality|GO:0060292,long term synaptic depression|GO:0046890,regulation of lipid biosynthetic process|GO:0071704,organic substance metabolic process|GO:0030336,negative regulation of cell migration|GO:0030334,regulation of cell migration|GO:0019216,regulation of lipid metabolic process|GO:0009058,biosynthetic process|GO:0044763,single-organism cellular process|GO:0007268,chemical synaptic transmission|GO:0007267,cell-cell signaling|GO:0007154,cell communication|GO:0009056,catabolic process|GO:0051179,localization|GO:0008610,lipid biosynthetic process|GO:0040011,locomotion|GO:0044238,primary metabolic process|GO:0051271,negative regulation of cellular component movement|GO:0040012,regulation of locomotion|GO:0044237,cellular metabolic process|GO:0006796,phosphate-containing compound metabolic process|GO:0006793,phosphorus metabolic process|GO:0048523,negative regulation of cellular process|GO:0098916,anterograde trans-synaptic signaling|GO:0099536,synaptic signaling|GO:0099537,trans-synaptic signaling	GO:0032281,AMPA glutamate receptor complex|GO:0043229,intracellular organelle|GO:0071944,cell periphery|GO:0034702,ion channel complex|GO:0031226,intrinsic component of plasma membrane|GO:0043226,organelle|GO:0031224,intrinsic component of membrane|GO:0005737,cytoplasm|GO:0031982,vesicle|GO:0043227,membrane-bounded organelle|GO:0016021,integral component of membrane|GO:0016020,membrane|GO:0098802,plasma membrane receptor complex|GO:0005739,mitochondrion|GO:0031988,membrane-bounded vesicle|GO:0044459,plasma membrane part|GO:1902495,transmembrane transporter complex|GO:0008328,ionotropic glutamate receptor complex|GO:1990351,transporter complex|GO:0005887,integral component of plasma membrane|GO:0005886,plasma membrane|GO:0032991,macromolecular complex|GO:1903561,extracellular vesicle|GO:0070062,extracellular exosome|GO:0043234,protein complex|GO:0043235,receptor complex|GO:0043230,extracellular organelle|GO:0043231,intracellular membrane-bounded organelle|GO:0044464,cell part|GO:0005623,cell|GO:0005622,intracellular|GO:0098797,plasma membrane protein complex|GO:0098796,membrane protein complex|GO:0044444,cytoplasmic part|GO:0005576,extracellular region|GO:0044424,intracellular part|GO:0044425,membrane part|GO:0065010,extracellular membrane-bounded organelle|GO:0044421,extracellular region part|GO:0034703,cation channel complex|GO:0098878,neurotransmitter	GO:0016787,hydrolase activity|GO:0016788,hydrolase activity, acting on ester bonds|GO:0003824,catalytic activity|GO:0052689,carboxylic ester hydrolase activity|GO:0004620,phospholipase activity|GO:0016298,lipase activity|GO:0047372,acylglycerol lipase activity
HCFC1	GO:0080090,regulation of primary metabolic process|GO:0019222,regulation of metabolic process|GO:0006473,protein acetylation|GO:0006475,internal protein amino acid acetylation|GO:1901576,organic substance biosynthetic process|GO:1901362,organic cyclic compound biosynthetic process|GO:0007005,mitochondrion organization|GO:0044710,single-organism metabolic process|GO:0010605,negative regulation of macromolecule metabolic process|GO:0010604,positive regulation of macromolecule metabolic process|GO:0010467,gene expression|GO:0018193,peptidyl-amino acid modification|GO:0044419,interspecies interaction between organisms|GO:0048518,positive regulation of biological process|GO:0048519,negative regulation of biological process|GO:0051704,multi-organism process|GO:0016570,histone modification|GO:0043967,histone H4 acetylation|GO:0060255,regulation of macromolecule metabolic process|GO:2001141,regulation of RNA biosynthetic process|GO:0010033,response to organic substance|GO:0046483,heterocycle metabolic process|GO:0018205,peptidyl-lysine modification|GO:0019538,protein metabolic process|GO:0019438,aromatic compound biosynthetic process|GO:0016568,chromatin modification|GO:0016569,covalent chromatin modification|GO:0022607,cellular component assembly|GO:0009892,negative regulation of metabolic process|GO:0009893,positive regulation of metabolic process|GO:0009890,negative regulation of biosynthetic process|GO:0009891,positive regulation of biosynthetic process|GO:0050821,protein stabilization|GO:0010629,negative regulation of gene expression|GO:0010628,positive regulation of gene expression|GO:0050789,regulation of biological process|GO:0097659,nucleic acid-templated transcription|GO:0043543,protein acylation|GO:0044260,cellular macromolecule metabolic process|GO:0016043,cellular component organization|GO:0065003,macromolecular complex assembly|GO:0065007,biological regulation|GO:0071840,cellular component organization or biogenesis|GO:0016573,histone acetylation|GO:0006366,transcription from RNA polymerase II promoter|GO:0065008,regulation of biological quality|GO:0018130,heterocycle biosynthetic process|GO:0018393,internal peptidyl-lysine acetylation|GO:0018394,peptidyl-lysine acetylation|GO:0009889,regulation of biosynthetic process|GO:0051716,cellular response to stimulus|GO:0050794,regulation of cellular process|GO:0043412,macromolecule modification|GO:0036211,protein modification process|GO:0008152,metabolic process|GO:0034654,nucleobase-containing compound biosynthetic process|GO:0043254,regulation of protein complex assembly|GO:0045787,positive regulation of cell cycle|GO:0016070,RNA metabolic process|GO:0044271,cellular nitrogen compound biosynthetic process|GO:0043982,histone H4-K8 acetylation|GO:0050896,response to stimulus|GO:0043981,histone H4-K5 acetylation|GO:0043984,histone H4-K16 acetylation|GO:0006355,regulation of transcription, DNA-templated|GO:0010557,positive regulation of macromolecule biosynthetic process|GO:0010556,regulation of macromolecule biosynthetic process|GO:0006351,transcription, DNA-templated|GO:0010558,negative regulation of macromolecule biosynthetic process|GO:0032774,RNA biosynthetic process|GO:0070271,protein complex biogenesis|GO:0051128,regulation of cellular component organization|GO:0044249,cellular biosynthetic process|GO:0034641,cellular nitrogen compound metabolic process|GO:0014070,response to organic cyclic compound|GO:0070887,cellular response to chemical stimulus|GO:0007049,cell cycle|GO:0006139,nucleobase-containing compound metabolic process|GO:0000122,negative regulation of transcription from RNA polymerase II promoter|GO:0043933,macromolecular complex subunit organization|GO:1903508,positive regulation of nucleic acid-templated transcription|GO:0009987,cellular process|GO:0006725,cellular aromatic compound metabolic process|GO:1903506,regulation of nucleic acid-templated transcription|GO:1903507,negative regulation of nucleic acid-templated transcription|GO:0034645,cellular macromolecule biosynthetic process|GO:0045892,negative regulation of transcription, DNA-templated|GO:0045893,positive regulation of transcription, DNA-templated|GO:0044699,single-organism process|GO:0071407,cellular response to organic cyclic compound|GO:0051253,negative regulation of RNA metabolic process|GO:0051252,regulation of RNA metabolic process|GO:0051254,positive regulation of RNA metabolic process|GO:0009059,macromolecule biosynthetic process|GO:0043170,macromolecule metabolic process|GO:1902680,positive regulation of RNA biosynthetic process|GO:0006807,nitrogen compound metabolic process|GO:0045944,positive regulation of transcription from RNA polymerase II promoter|GO:0019046,release from viral latency|GO:0031647,regulation of protein stability|GO:0019042,viral latency|GO:0031328,positive regulation of cellular biosynthetic process|GO:0031327,negative regulation of cellular biosynthetic process|GO:0031326,regulation of cellular biosynthetic process|GO:0031325,positive regulation of cellular metabolic process|GO:0031324,negative regulation of cellular metabolic process|GO:0031323,regulation of cellular metabolic process|GO:0090304,nucleic acid metabolic process|GO:0071822,protein complex subunit organization|GO:0006325,chromatin organization|GO:1901360,organic cyclic compound metabolic process|GO:2000112,regulation of cellular macromolecule biosynthetic process|GO:2000113,negative regulation of cellular macromolecule biosynthetic process|GO:0071704,organic substance metabolic process|GO:0071310,cellular response to organic substance|GO:0006357,regulation of transcription from RNA polymerase II promoter|GO:0010468,regulation of gene expression|GO:0045935,positive regulation of nucleobase-containing compound metabolic process|GO:0045934,negative regulation of nucleobase-containing compound metabolic process|GO:0044267,cellular protein metabolic process|GO:0019219,regulation of nucleobase-containing compound metabolic process|GO:0006461,protein complex assembly|GO:0006464,cellular protein modification process|GO:1902679,negative regulation of RNA biosynthetic process|GO:0009058,biosynthetic process|GO:0044764,multi-organism cellular process|GO:0044763,single-organism cellular process|GO:0051171,regulation of nitrogen compound metabolic process|GO:0051172,negative regulation of nitrogen compound metabolic process|GO:0051173,positive regulation of nitrogen compound metabolic process|GO:0042221,response to chemical|GO:0006996,organelle organization|GO:0044238,primary metabolic process|GO:0051276,chromosome organization|GO:0051726,regulation of cell cycle|GO:0044237,cellular metabolic process|GO:0044087,regulation of cellular component biogenesis|GO:1902589,single-organism organelle organization|GO:0044085,cellular component biogenesis|GO:0016032,viral process|GO:0044403,symbiosis, encompassing mutualism through parasitism|GO:0048523,negative regulation of cellular process|GO:0048522,positive regulation of cellular process|GO:0070647,protein modification by small protein conjugation or removal|GO:0070646,protein modification by small protein removal|GO:1901564,organonitrogen compound metabolic	GO:0031248,protein acetyltransferase complex|GO:0031974,membrane-enclosed lumen|GO:0030424,axon|GO:0043229,intracellular organelle|GO:0005623,cell|GO:0043227,membrane-bounded organelle|GO:0043226,organelle|GO:0034708,methyltransferase complex|GO:0005737,cytoplasm|GO:0031981,nuclear lumen|GO:0005634,nucleus|GO:0016020,membrane|GO:0044444,cytoplasmic part|GO:0005739,mitochondrion|GO:0005654,nucleoplasm|GO:0097458,neuron part|GO:0005671,Ada2/Gcn5/Ada3 transcription activator complex|GO:0044451,nucleoplasm part|GO:0043005,neuron projection|GO:0070688,MLL5-L complex|GO:0043025,neuronal cell body|GO:0071339,MLL1 complex|GO:1902493,acetyltransferase complex|GO:1902494,catalytic complex|GO:0044297,cell body|GO:0030425,dendrite|GO:1990234,transferase complex|GO:0042995,cell projection|GO:0036477,somatodendritic compartment|GO:0043231,intracellular membrane-bounded organelle|GO:0043234,protein complex|GO:0032991,macromolecular complex|GO:0000123,histone acetyltransferase complex|GO:0043233,organelle lumen|GO:0044464,cell part|GO:0035097,histone methyltransferase complex|GO:0005622,intracellular|GO:0044446,intracellular organelle part|GO:0070013,intracellular organelle lumen|GO:0070461,SAGA-type complex|GO:0048188,Set1C/COMPASS complex|GO:0044428,nuclear part|GO:0044424,intracellular part|GO:0044422,organelle part|GO:0044665,MLL1/2 complex|GO:1902562,H4 histone acetyltransferase complex	GO:0043995,histone acetyltransferase activity (H4-K5 specific)|GO:0003705,transcription factor activity, RNA polymerase II distal enhancer sequence-specific binding|GO:0046972,histone acetyltransferase activity (H4-K16 specific)|GO:0044877,macromolecular complex binding|GO:0005488,binding|GO:0010485,H4 histone acetyltransferase activity|GO:0008080,N-acetyltransferase activity|GO:0003713,transcription coactivator activity|GO:0003712,transcription cofactor activity|GO:0043996,histone acetyltransferase activity (H4-K8 specific)|GO:0016740,transferase activity|GO:0016746,transferase activity, transferring acyl groups|GO:0016747,transferase activity, transferring acyl groups other than amino-acyl groups|GO:0016410,N-acyltransferase activity|GO:0000988,transcription factor activity, protein binding|GO:0090595,acetyl-CoA:L-lysine N6-acetyltransferase|GO:0042802,identical protein binding|GO:0000989,transcription factor activity, transcription factor binding|GO:0003824,catalytic activity|GO:0001205,transcriptional activator activity, RNA polymerase II distal enhancer sequence-specific binding|GO:0003700,transcription factor activity, sequence-specific DNA binding|GO:0001071,nucleic acid binding transcription factor activity|GO:0001228,transcriptional activator activity, RNA polymerase II transcription regulatory region sequence-specific binding|GO:0016407,acetyltransferase activity|GO:0000981,RNA polymerase II transcription factor activity, sequence-specific DNA binding|GO:0005515,protein binding|GO:0003682,chromatin binding|GO:0004402,histone acetyltransferase activity|GO:0033613,activating transcription factor binding|GO:0061733,peptide-lysine-N-acetyltransferase activity|GO:0034212,peptide N-acetyltransferase activity|GO:0008134,transcription factor binding|GO:0050839,cell adhesion molecule binding|GO:0045296,cadherin binding|GO:0030674,protein binding, bridging|GO:0060090,binding, bridging

ST3GAL3	GO:0030203,glycosaminoglycan metabolic process|GO:0018146,keratan sulfate biosynthetic process|GO:1903510,mucopolysaccharide metabolic process|GO:0044723,single-organism carbohydrate metabolic process|GO:0006807,nitrogen compound metabolic process|GO:0044281,small molecule metabolic process|GO:0034645,cellular macromolecule biosynthetic process|GO:0009100,glycoprotein metabolic process|GO:0009101,glycoprotein biosynthetic process|GO:0044699,single-organism process|GO:0044267,cellular protein metabolic process|GO:0044710,single-organism metabolic process|GO:0044711,single-organism biosynthetic process|GO:1901564,organonitrogen compound metabolic process|GO:0006486,protein glycosylation|GO:0006487,protein N-linked glycosylation|GO:0006488,dolichol-linked oligosaccharide biosynthetic process|GO:0018196,peptidyl-asparagine modification|GO:0018193,peptidyl-amino acid modification|GO:0042339,keratan sulfate metabolic process|GO:0071704,organic substance metabolic process|GO:1901566,organonitrogen compound biosynthetic process|GO:0018279,protein N-linked glycosylation via asparagine|GO:0016266,O-glycan processing|GO:1901576,organic substance biosynthetic process|GO:0043687,post-translational protein modification|GO:0009987,cellular process|GO:0070085,glycosylation|GO:0006464,cellular protein modification process|GO:0043412,macromolecule modification|GO:0043413,macromolecule glycosylation|GO:0044763,single-organism cellular process|GO:0008152,metabolic process|GO:0043436,oxoacid metabolic process|GO:0005975,carbohydrate metabolic process|GO:0009059,macromolecule biosynthetic process|GO:0044238,primary metabolic process|GO:0006493,protein O-linked glycosylation|GO:0044260,cellular macromolecule metabolic process|GO:0006082,organic acid metabolic process|GO:0044272,sulfur compound biosynthetic process|GO:0019538,protein metabolic process|GO:1901135,carbohydrate derivative metabolic process|GO:0097503,sialylation|GO:0009058,biosynthetic process|GO:0044237,cellular metabolic process|GO:0043170,macromolecule metabolic process|GO:1901137,carbohydrate derivative biosynthetic process|GO:0036211,protein modification process|GO:0006790,sulfur compound metabolic process|GO:0006022,aminoglycan metabolic process|GO:0006023,aminoglycan biosynthetic process|GO:0044249,cellular biosynthetic process|GO:0006024,glycosaminoglycan biosynthetic process|GO:0009311,oligosaccharide metabolic process	GO:0043229,intracellular organelle|GO:0031301,integral component of organelle membrane|GO:0031300,intrinsic component of organelle membrane|GO:0030173,integral component of Golgi membrane|GO:0005622,intracellular|GO:0043227,membrane-bounded organelle|GO:0043226,organelle|GO:0031224,intrinsic component of membrane|GO:0005737,cytoplasm|GO:0031985,Golgi cisterna|GO:0031984,organelle subcompartment|GO:0031090,organelle membrane|GO:0016021,integral component of membrane|GO:0016020,membrane|GO:0044431,Golgi apparatus part|GO:0005795,Golgi stack|GO:0005794,Golgi apparatus|GO:0098588,bounding membrane of organelle|GO:0012505,endomembrane system|GO:0032580,Golgi cisterna membrane|GO:0043231,intracellular membrane-bounded organelle|GO:0044464,cell part|GO:0031228,intrinsic component of Golgi membrane|GO:0005623,cell|GO:0000139,Golgi membrane|GO:0044446,intracellular organelle part|GO:0044444,cytoplasmic part|GO:0005576,extracellular region|GO:0098791,Golgi subcompartment|GO:0044424,intracellular part|GO:0044425,membrane	GO:0008118,N-acetyllactosaminide alpha-2,3-sialyltransferase activity|GO:0003836,beta-galactoside (CMP) alpha-2,3-sialyltransferase activity|GO:0003824,catalytic activity|GO:0016740,transferase activity|GO:0016757,transferase activity, transferring glycosyl groups|GO:0008373,sialyltransferase activity
GALNT16	GO:0044249,cellular biosynthetic process|GO:0034645,cellular macromolecule biosynthetic process|GO:0009100,glycoprotein metabolic process|GO:0009101,glycoprotein biosynthetic process|GO:0044699,single-organism process|GO:0044267,cellular protein metabolic process|GO:0044710,single-organism metabolic process|GO:0044260,cellular macromolecule metabolic process|GO:0071704,organic substance metabolic process|GO:1901576,organic substance biosynthetic process|GO:0009987,cellular process|GO:0070085,glycosylation|GO:0006464,cellular protein modification process|GO:0043412,macromolecule modification|GO:0036211,protein modification process|GO:0044763,single-organism cellular process|GO:0008152,metabolic process|GO:0044723,single-organism carbohydrate metabolic process|GO:0009059,macromolecule biosynthetic process|GO:0044238,primary metabolic process|GO:0005975,carbohydrate metabolic process|GO:0006486,protein glycosylation|GO:0019538,protein metabolic process|GO:1901135,carbohydrate derivative metabolic process|GO:0009058,biosynthetic process|GO:0044237,cellular metabolic process|GO:0043170,macromolecule metabolic process|GO:1901137,carbohydrate derivative biosynthetic process|GO:0043413,macromolecule glycosylation|GO:0006807,nitrogen compound metabolic process|GO:1901564,organonitrogen compound metabolic process|GO:1901566,organonitrogen compound biosynthetic process|GO:0018209,peptidyl-serine modification|GO:0018193,peptidyl-amino acid modification|GO:0018243,protein O-linked glycosylation via threonine|GO:0018242,protein O-linked glycosylation via serine|GO:0018210,peptidyl-threonine modification|GO:0006493,protein O-linked glycosylation	GO:0043229,intracellular organelle|GO:0005622,intracellular|GO:0043227,membrane-bounded organelle|GO:0043226,organelle|GO:0031224,intrinsic component of membrane|GO:0005737,cytoplasm|GO:0031982,vesicle|GO:0016021,integral component of membrane|GO:0016020,membrane|GO:0031988,membrane-bounded vesicle|GO:0044431,Golgi apparatus part|GO:0005794,Golgi apparatus|GO:0098588,bounding membrane of organelle|GO:0012505,endomembrane system|GO:1903561,extracellular vesicle|GO:0031090,organelle membrane|GO:0043230,extracellular organelle|GO:0043231,intracellular membrane-bounded organelle|GO:0070062,extracellular exosome|GO:0044464,cell part|GO:0005623,cell|GO:0000139,Golgi membrane|GO:0044446,intracellular organelle part|GO:0044444,cytoplasmic part|GO:0005576,extracellular region|GO:0044424,intracellular part|GO:0044425,membrane part|GO:0065010,extracellular membrane-bounded organelle|GO:0044421,extracellular region part|GO:0044422,organelle part|GO:0031984,organelle subcompartment|GO:0098791,Golgi subcompartment	GO:0043169,cation binding|GO:0004653,polypeptide N-acetylgalactosaminyltransferase activity|GO:0030246,carbohydrate binding|GO:0043167,ion binding|GO:0003824,catalytic activity|GO:0016740,transferase activity|GO:0005488,binding|GO:0016757,transferase activity, transferring glycosyl groups|GO:0016758,transferase activity, transferring hexosyl groups|GO:0008376,acetylgalactosaminyltransferase activity|GO:0046872,metal ion binding|GO:0008194,UDP-glycosyltransferase activity
PPM1F	GO:0043549,regulation of kinase activity|GO:0019220,regulation of phosphate metabolic process|GO:0080090,regulation of primary metabolic process|GO:0019222,regulation of metabolic process|GO:1901890,positive regulation of cell junction assembly|GO:0006470,protein dephosphorylation|GO:0048584,positive regulation of response to stimulus|GO:0048583,regulation of response to stimulus|GO:0007610,behavior|GO:0007160,cell-matrix adhesion|GO:0043933,macromolecular complex subunit organization|GO:0006325,chromatin organization|GO:0050920,regulation of chemotaxis|GO:0050921,positive regulation of chemotaxis|GO:0051893,regulation of focal adhesion assembly|GO:1901576,organic substance biosynthetic process|GO:1901362,organic cyclic compound biosynthetic process|GO:0044707,single-multicellular organism process|GO:0071840,cellular component organization or biogenesis|GO:0051894,positive regulation of focal adhesion assembly|GO:0032231,regulation of actin filament bundle assembly|GO:0044710,single-organism metabolic process|GO:0032233,positive regulation of actin filament bundle assembly|GO:0010605,negative regulation of macromolecule metabolic process|GO:0010604,positive regulation of macromolecule metabolic process|GO:0042330,taxis|GO:0045785,positive regulation of cell adhesion|GO:0051496,positive regulation of stress fiber assembly|GO:0051495,positive regulation of cytoskeleton organization|GO:0033043,regulation of organelle organization|GO:0051493,regulation of cytoskeleton organization|GO:0051492,regulation of stress fiber assembly|GO:0018193,peptidyl-amino acid modification|GO:0090132,epithelium migration|GO:0071704,organic substance metabolic process|GO:0010467,gene expression|GO:0044093,positive regulation of molecular function|GO:0048522,positive regulation of cellular process|GO:0043281,regulation of cysteine-type endopeptidase activity involved in apoptotic process|GO:0043280,positive regulation of cysteine-type endopeptidase activity involved in apoptotic process|GO:0040011,locomotion|GO:0010634,positive regulation of epithelial cell migration|GO:0031589,cell-substrate adhesion|GO:0022607,cellular component assembly|GO:0006935,chemotaxis|GO:1901888,regulation of cell junction assembly|GO:0060255,regulation of macromolecule metabolic process|GO:0045859,regulation of protein kinase activity|GO:0006807,nitrogen compound metabolic process|GO:0042221,response to chemical|GO:0030162,regulation of proteolysis|GO:2001141,regulation of RNA biosynthetic process|GO:0065009,regulation of molecular function|GO:0046483,heterocycle metabolic process|GO:0042325,regulation of phosphorylation|GO:0044700,single organism signaling|GO:0061572,actin filament bundle organization|GO:0042326,negative regulation of phosphorylation|GO:0018209,peptidyl-serine modification|GO:0010631,epithelial cell migration|GO:0010632,regulation of epithelial cell migration|GO:0048870,cell motility|GO:0019538,protein metabolic process|GO:0010638,positive regulation of organelle organization|GO:0034641,cellular nitrogen compound metabolic process|GO:0043068,positive regulation of programmed cell death|GO:0019438,aromatic compound biosynthetic process|GO:0018130,heterocycle biosynthetic process|GO:0016568,chromatin modification|GO:0007165,signal transduction|GO:0033673,negative regulation of kinase activity|GO:0016265,obsolete death|GO:0001954,positive regulation of cell-matrix adhesion|GO:0009892,negative regulation of metabolic process|GO:0009893,positive regulation of metabolic process|GO:0009890,negative regulation of biosynthetic process|GO:0006468,protein phosphorylation|GO:0006928,movement of cell or subcellular component|GO:0008152,metabolic process|GO:0010629,negative regulation of gene expression|GO:0010628,positive regulation of gene expression|GO:1903391,regulation of adherens junction organization|GO:1903393,positive regulation of adherens junction organization|GO:0035556,intracellular signal transduction|GO:0010810,regulation of cell-substrate adhesion|GO:0042981,regulation of apoptotic process|GO:0050789,regulation of biological process|GO:0097659,nucleic acid-templated transcription|GO:0009605,response to external stimulus|GO:0044267,cellular protein metabolic process|GO:0100002,negative regulation of protein kinase activity by protein phosphorylation|GO:0051345,positive regulation of hydrolase activity|GO:0051348,negative regulation of transferase activity|GO:0016043,cellular component organization|GO:0090109,regulation of cell-substrate junction assembly|GO:0019219,regulation of nucleobase-containing compound metabolic process|GO:0016576,histone dephosphorylation|GO:0065007,biological regulation|GO:0016570,histone modification|GO:0032970,regulation of actin filament-based process|GO:0016477,cell migration|GO:0043149,stress fiber assembly|GO:0051130,positive regulation of cellular component organization|GO:0044260,cellular macromolecule metabolic process|GO:0050790,regulation of catalytic activity|GO:0009889,regulation of biosynthetic process|GO:0051716,cellular response to stimulus|GO:0050795,regulation of behavior|GO:0050794,regulation of cellular process|GO:0012501,programmed cell death|GO:0043412,macromolecule modification|GO:0036211,protein modification process|GO:0006464,cellular protein modification process|GO:0051239,regulation of multicellular organismal process|GO:0032956,regulation of actin cytoskeleton organization|GO:0034654,nucleobase-containing compound biosynthetic process|GO:0044238,primary metabolic process|GO:0006725,cellular aromatic compound metabolic process|GO:0051336,regulation of hydrolase activity|GO:1902679,negative regulation of RNA biosynthetic process|GO:0044271,cellular nitrogen compound biosynthetic process|GO:0031400,negative regulation of protein modification process|GO:0051338,regulation of transferase activity|GO:0034332,adherens junction organization|GO:0006355,regulation of transcription, DNA-templated|GO:0010556,regulation of macromolecule biosynthetic process|GO:2000145,regulation of cell motility|GO:0006351,transcription, DNA-templated|GO:2000147,positive regulation of cell motility|GO:0016569,covalent chromatin modification|GO:0010558,negative regulation of macromolecule biosynthetic process|GO:0032103,positive regulation of response to external stimulus|GO:0032101,regulation of response to external stimulus|GO:0016311,dephosphorylation|GO:0044699,single-organism process|GO:0030155,regulation of cell adhesion|GO:0051128,regulation of cellular component organization|GO:0044249,cellular biosynthetic process|GO:0007045,cell-substrate adherens junction assembly|GO:0007044,cell-substrate junction assembly|GO:0023052,signaling|GO:0070887,cellular response to chemical stimulus|GO:0007154,cell communication|GO:0001667,ameboidal-type cell migration|GO:0043086,negative regulation of catalytic activity|GO:0043085,positive regulation of catalytic activity|GO:0031032,actomyosin structure organization|GO:0006139,nucleobase-containing compound metabolic process|GO:0051248,negative regulation of protein metabolic process|GO:0051240,positive regulation of multicellular organismal process|GO:0010563,negative regulation of phosphorus metabolic process|GO:0051246,regulation of protein metabolic process|GO:0051247,positive regulation of protein metabolic process|GO:0034333,adherens junction assembly|GO:0032270,positive regulation of cellular protein metabolic process|GO:0035970,peptidyl-threonine dephosphorylation|GO:0033135,regulation of peptidyl-serine phosphorylation|GO:0006508,proteolysis|GO:0022610,biological adhesion|GO:0031325,positive regulation of cellular metabolic process|GO:0016070,RNA metabolic process|GO:0031324,negative regulation of cellular metabolic process|GO:0007015,actin filament organization|GO:0031323,regulation of cellular metabolic process|GO:0032501,multicellular organismal process|GO:0031399,regulation of protein modification process|GO:0051017,actin filament bundle assembly|GO:1903506,regulation of nucleic acid-templated transcription|GO:1903507,negative regulation of nucleic acid-templated transcription|GO:0048518,positive regulation of biological process|GO:0034645,cellular macromolecule biosynthetic process|GO:0040012,regulation of locomotion|GO:0045892,negative regulation of transcription, DNA-templated|GO:0030036,actin cytoskeleton organization|GO:0045216,cell-cell junction organization|GO:0048519,negative regulation of biological process|GO:0032879,regulation of localization|GO:0034329,cell junction assembly|GO:0090130,tissue migration|GO:0032269,negative regulation of cellular protein metabolic process|GO:0032268,regulation of cellular protein metabolic process|GO:0051674,localization of cell|GO:0051253,negative regulation of RNA metabolic process|GO:0051252,regulation of RNA metabolic	GO:0043234,protein complex|GO:0005737,cytoplasm|GO:0032991,macromolecular complex|GO:0005829,cytosol|GO:0044464,cell part|GO:0005623,cell|GO:0005622,intracellular|GO:0044444,cytoplasmic part|GO:0044424,intracellular part|GO:0048471,perinuclear region of cytoplasm	GO:0042578,phosphoric ester hydrolase activity|GO:0016791,phosphatase activity|GO:0016787,hydrolase activity|GO:0004723,calcium-dependent protein serine/threonine phosphatase activity|GO:0004722,protein serine/threonine phosphatase activity|GO:0004721,phosphoprotein phosphatase activity|GO:0043167,ion binding|GO:0033192,calmodulin-dependent protein phosphatase activity|GO:0003824,catalytic activity|GO:0043169,cation binding|GO:0016788,hydrolase activity, acting on ester bonds|GO:0005515,protein binding|GO:0046872,metal ion binding|GO:0005488,binding
C21orf33		GO:0005737,cytoplasm|GO:0043231,intracellular membrane-bounded organelle|GO:0044464,cell part|GO:0043229,intracellular organelle|GO:0005739,mitochondrion|GO:0005622,intracellular|GO:0044444,cytoplasmic part|GO:0005623,cell|GO:0044424,intracellular part|GO:0043227,membrane-bounded organelle|GO:0043226,organelle	
MYLK2	GO:0019222,regulation of metabolic process|GO:0051049,regulation of transport|GO:0032386,regulation of intracellular transport|GO:0072359,circulatory system development|GO:0072358,cardiovascular system development|GO:0003015,heart process|GO:0007517,muscle organ development|GO:0010604,positive regulation of macromolecule metabolic process|GO:0048869,cellular developmental process|GO:0007519,skeletal muscle tissue development|GO:0018210,peptidyl-threonine modification|GO:0048513,animal organ development|GO:0048518,positive regulation of biological process|GO:0006936,muscle contraction|GO:0006937,regulation of muscle contraction|GO:0060255,regulation of macromolecule metabolic process|GO:0030049,muscle filament sliding|GO:0030048,actin filament-based movement|GO:0003008,system process|GO:0044700,single organism signaling|GO:0044707,single-multicellular organism process|GO:0019538,protein metabolic process|GO:0003012,muscle system process|GO:0003013,circulatory system process|GO:0060415,muscle tissue morphogenesis|GO:0009893,positive regulation of metabolic process|GO:0014816,skeletal muscle satellite cell differentiation|GO:0006928,movement of cell or subcellular component|GO:0010628,positive regulation of gene expression|GO:0050789,regulation of biological process|GO:0044267,cellular protein metabolic process|GO:0044260,cellular macromolecule metabolic process|GO:0065007,biological regulation|GO:0035914,skeletal muscle cell differentiation|GO:0048644,muscle organ morphogenesis|GO:0009887,animal organ morphogenesis|GO:0008015,blood circulation|GO:0006810,transport|GO:0009888,tissue development|GO:0050794,regulation of cellular process|GO:0043412,macromolecule modification|GO:0036211,protein modification process|GO:0051239,regulation of multicellular organismal process|GO:0051234,establishment of localization|GO:0046907,intracellular transport|GO:0008152,metabolic process|GO:0003007,heart morphogenesis|GO:0016310,phosphorylation|GO:0030154,cell differentiation|GO:0006941,striated muscle contraction|GO:0060047,heart contraction|GO:0023052,signaling|GO:0018193,peptidyl-amino acid modification|GO:0061061,muscle structure development|GO:0009653,anatomical structure morphogenesis|GO:0060048,cardiac muscle contraction|GO:0046777,protein autophosphorylation|GO:0044699,single-organism process|GO:0044057,regulation of system process|GO:0033275,actin-myosin filament sliding|GO:0032502,developmental process|GO:0032501,multicellular organismal process|GO:1903115,regulation of actin filament-based movement|GO:0044238,primary metabolic process|GO:0055008,cardiac muscle tissue morphogenesis|GO:0032971,regulation of muscle filament sliding|GO:0032970,regulation of actin filament-based process|GO:0032879,regulation of localization|GO:0043170,macromolecule metabolic process|GO:0048738,cardiac muscle tissue development|GO:0048731,system development|GO:0018107,peptidyl-threonine phosphorylation|GO:0060341,regulation of cellular localization|GO:0014706,striated muscle tissue development|GO:0007274,neuromuscular synaptic transmission|GO:0007275,multicellular organism development|GO:0071704,organic substance metabolic process|GO:0010467,gene expression|GO:0048729,tissue morphogenesis|GO:0051649,establishment of localization in cell|GO:0010468,regulation of gene expression|GO:0006468,protein phosphorylation|GO:0009987,cellular process|GO:0030029,actin filament-based process|GO:0060537,muscle tissue development|GO:0006464,cellular protein modification process|GO:0044767,single-organism developmental process|GO:0044763,single-organism cellular process|GO:0090257,regulation of muscle system process|GO:0007268,chemical synaptic transmission|GO:0007267,cell-cell signaling|GO:0007154,cell communication|GO:0051179,localization|GO:0051641,cellular localization|GO:0007507,heart development|GO:0051270,regulation of cellular component movement|GO:0048856,anatomical structure development|GO:0044237,cellular metabolic process|GO:0006796,phosphate-containing compound metabolic process|GO:0006793,phosphorus metabolic process|GO:0060538,skeletal muscle organ development|GO:0070252,actin-mediated cell contraction|GO:0007165,signal transduction|GO:1901564,organonitrogen compound metabolic process|GO:0018209,peptidyl-serine modification|GO:0098916,anterograde trans-synaptic signaling|GO:0051716,cellular response to stimulus|GO:0035556,intracellular signal transduction|GO:0050896,response to stimulus|GO:0099536,synaptic signaling|GO:0099537,trans-synaptic signaling|GO:0006807,nitrogen compound metabolic process|GO:0018105,peptidyl-serine phosphorylation	GO:0005737,cytoplasm|GO:0043231,intracellular membrane-bounded organelle|GO:0043232,intracellular non-membrane-bounded organelle|GO:0005634,nucleus|GO:0044464,cell part|GO:0044444,cytoplasmic part|GO:0005623,cell|GO:0005622,intracellular|GO:0030016,myofibril|GO:0030017,sarcomere|GO:0043229,intracellular organelle|GO:0043228,non-membrane-bounded organelle|GO:0043292,contractile fiber|GO:0044424,intracellular part|GO:0043227,membrane-bounded organelle|GO:0043226,organelle|GO:0044422,organelle part|GO:0044449,contractile fiber part|GO:0099512,supramolecular fiber	GO:0035639,purine ribonucleoside triphosphate binding|GO:0004672,protein kinase activity|GO:0043167,ion binding|GO:1901363,heterocyclic compound binding|GO:0001883,purine nucleoside binding|GO:0001882,nucleoside binding|GO:0043168,anion binding|GO:0000166,nucleotide binding|GO:0004674,protein serine/threonine kinase activity|GO:0016740,transferase activity|GO:1901265,nucleoside phosphate binding|GO:0032549,ribonucleoside binding|GO:0017076,purine nucleotide binding|GO:0005524,ATP binding|GO:0016301,kinase activity|GO:0036094,small molecule binding|GO:0003824,catalytic activity|GO:0004687,myosin light chain kinase activity|GO:0004683,calmodulin-dependent protein kinase activity|GO:0016773,phosphotransferase activity, alcohol group as acceptor|GO:0016772,transferase activity, transferring phosphorus-containing groups|GO:0030554,adenyl nucleotide binding|GO:0097367,carbohydrate derivative binding|GO:0032559,adenyl ribonucleotide binding|GO:0005516,calmodulin binding|GO:0005515,protein binding|GO:0097159,organic cyclic compound binding|GO:0032555,purine ribonucleotide binding|GO:0032550,purine ribonucleoside binding|GO:0032553,ribonucleotide binding|GO:0005488,binding
OSBPL2	GO:0033036,macromolecule localization|GO:0006869,lipid transport|GO:0006810,transport|GO:0044765,single-organism transport|GO:1902578,single-organism localization|GO:0071702,organic substance transport|GO:0051234,establishment of localization|GO:0010876,lipid localization|GO:0051179,localization|GO:0044699,single-organism process|GO:0015918,sterol transport|GO:0015850,organic hydroxy compound transport|GO:0019752,carboxylic acid metabolic process|GO:0044249,cellular biosynthetic process|GO:0044281,small molecule metabolic process|GO:0044283,small molecule biosynthetic process|GO:1901362,organic cyclic compound biosynthetic process|GO:0072330,monocarboxylic acid biosynthetic process|GO:1901576,organic substance biosynthetic process|GO:0044710,single-organism metabolic process|GO:0044711,single-organism biosynthetic process|GO:1901360,organic cyclic compound metabolic process|GO:0071704,organic substance metabolic process|GO:0006629,lipid metabolic process|GO:0009987,cellular process|GO:0032787,monocarboxylic acid metabolic process|GO:0009058,biosynthetic process|GO:0044763,single-organism cellular process|GO:0008152,metabolic process|GO:0043436,oxoacid metabolic process|GO:0008206,bile acid metabolic process|GO:0008202,steroid metabolic process|GO:0008610,lipid biosynthetic process|GO:0044238,primary metabolic process|GO:0006082,organic acid metabolic process|GO:0046394,carboxylic acid biosynthetic process|GO:0016053,organic acid biosynthetic process|GO:0044237,cellular metabolic process|GO:0006694,steroid biosynthetic process|GO:0006699,bile acid biosynthetic process|GO:1901617,organic hydroxy compound biosynthetic process|GO:1901615,organic hydroxy compound metabolic process	GO:0005737,cytoplasm|GO:0005829,cytosol|GO:0044464,cell part|GO:0005623,cell|GO:0005622,intracellular|GO:0044444,cytoplasmic part|GO:0044424,intracellular part	GO:0032934,sterol binding|GO:0015485,cholesterol binding|GO:0043178,alcohol binding|GO:0097159,organic cyclic compound binding|GO:0008289,lipid binding|GO:0036094,small molecule binding|GO:0005488,binding|GO:0005515,protein binding|GO:0005496,steroid binding|GO:0005319,lipid transporter activity|GO:0022892,substrate-specific transporter activity|GO:0005215,transporter activity|GO:0015248,sterol transporter activity
DR1	GO:0080090,regulation of primary metabolic process|GO:0019222,regulation of metabolic process|GO:0006473,protein acetylation|GO:0006475,internal protein amino acid acetylation|GO:1901362,organic cyclic compound biosynthetic process|GO:1901360,organic cyclic compound metabolic process|GO:0044710,single-organism metabolic process|GO:0010605,negative regulation of macromolecule metabolic process|GO:0018193,peptidyl-amino acid modification|GO:0048519,negative regulation of biological process|GO:0016570,histone modification|GO:0043966,histone H3 acetylation|GO:0060255,regulation of macromolecule metabolic process|GO:2001141,regulation of RNA biosynthetic process|GO:0046483,heterocycle metabolic process|GO:0019538,protein metabolic process|GO:0018205,peptidyl-lysine modification|GO:0019438,aromatic compound biosynthetic process|GO:0016568,chromatin modification|GO:0016569,covalent chromatin modification|GO:0009892,negative regulation of metabolic process|GO:0009890,negative regulation of biosynthetic process|GO:0010629,negative regulation of gene expression|GO:0006807,nitrogen compound metabolic process|GO:0050789,regulation of biological process|GO:0097659,nucleic acid-templated transcription|GO:0043543,protein acylation|GO:0044260,cellular macromolecule metabolic process|GO:0016043,cellular component organization|GO:0065007,biological regulation|GO:0071840,cellular component organization or biogenesis|GO:0016573,histone acetylation|GO:0006366,transcription from RNA polymerase II promoter|GO:0044267,cellular protein metabolic process|GO:0018130,heterocycle biosynthetic process|GO:0018393,internal peptidyl-lysine acetylation|GO:0018394,peptidyl-lysine acetylation|GO:0009889,regulation of biosynthetic process|GO:0050794,regulation of cellular process|GO:0043412,macromolecule modification|GO:0036211,protein modification process|GO:0008152,metabolic process|GO:0034654,nucleobase-containing compound biosynthetic process|GO:0016070,RNA metabolic process|GO:0044271,cellular nitrogen compound biosynthetic process|GO:0006355,regulation of transcription, DNA-templated|GO:0010556,regulation of macromolecule biosynthetic process|GO:0006351,transcription, DNA-templated|GO:0010558,negative regulation of macromolecule biosynthetic process|GO:0032774,RNA biosynthetic process|GO:0044249,cellular biosynthetic process|GO:0034641,cellular nitrogen compound metabolic process|GO:0034645,cellular macromolecule biosynthetic process|GO:0044699,single-organism process|GO:0006139,nucleobase-containing compound metabolic process|GO:0000122,negative regulation of transcription from RNA polymerase II promoter|GO:0043933,macromolecular complex subunit organization|GO:0009987,cellular process|GO:0006725,cellular aromatic compound metabolic process|GO:1903506,regulation of nucleic acid-templated transcription|GO:1903507,negative regulation of nucleic acid-templated transcription|GO:0045892,negative regulation of transcription, DNA-templated|GO:0051253,negative regulation of RNA metabolic process|GO:0051252,regulation of RNA metabolic process|GO:0043170,macromolecule metabolic process|GO:0031327,negative regulation of cellular biosynthetic process|GO:0031326,regulation of cellular biosynthetic process|GO:0031324,negative regulation of cellular metabolic process|GO:0031323,regulation of cellular metabolic process|GO:0090304,nucleic acid metabolic process|GO:0006325,chromatin organization|GO:2000112,regulation of cellular macromolecule biosynthetic process|GO:2000113,negative regulation of cellular macromolecule biosynthetic process|GO:0071704,organic substance metabolic process|GO:0010467,gene expression|GO:0006357,regulation of transcription from RNA polymerase II promoter|GO:0010468,regulation of gene expression|GO:0045934,negative regulation of nucleobase-containing compound metabolic process|GO:1901576,organic substance biosynthetic process|GO:0019219,regulation of nucleobase-containing compound metabolic process|GO:0006464,cellular protein modification process|GO:1902679,negative regulation of RNA biosynthetic process|GO:0009058,biosynthetic process|GO:0009059,macromolecule biosynthetic process|GO:0044763,single-organism cellular process|GO:0051171,regulation of nitrogen compound metabolic process|GO:0051172,negative regulation of nitrogen compound metabolic process|GO:0006996,organelle organization|GO:0044238,primary metabolic process|GO:0051276,chromosome organization|GO:0044237,cellular metabolic process|GO:1902589,single-organism organelle organization|GO:0048523,negative regulation of cellular process|GO:1901564,organonitrogen compound	GO:0031248,protein acetyltransferase complex|GO:0031974,membrane-enclosed lumen|GO:0043229,intracellular organelle|GO:0043227,membrane-bounded organelle|GO:0043226,organelle|GO:0000123,histone acetyltransferase complex|GO:0005634,nucleus|GO:0005654,nucleoplasm|GO:0005671,Ada2/Gcn5/Ada3 transcription activator complex|GO:0044451,nucleoplasm part|GO:1902493,acetyltransferase complex|GO:1902494,catalytic complex|GO:1990234,transferase complex|GO:0043231,intracellular membrane-bounded organelle|GO:0043234,protein complex|GO:0032991,macromolecular complex|GO:0031981,nuclear lumen|GO:0043233,organelle lumen|GO:0044464,cell part|GO:0005623,cell|GO:0005622,intracellular|GO:0044446,intracellular organelle part|GO:0070013,intracellular organelle lumen|GO:0044428,nuclear part|GO:0044424,intracellular part|GO:0044422,organelle part|GO:1902562,H4 histone acetyltransferase complex	GO:0000988,transcription factor activity, protein binding|GO:0043565,sequence-specific DNA binding|GO:0005515,protein binding|GO:0017025,TBP-class protein binding|GO:0008134,transcription factor binding|GO:0003714,transcription corepressor activity|GO:0097159,organic cyclic compound binding|GO:0003712,transcription cofactor activity|GO:0000989,transcription factor activity, transcription factor binding|GO:0005488,binding|GO:0003676,nucleic acid binding|GO:0003677,DNA binding|GO:0046983,protein dimerization activity|GO:0046982,protein heterodimerization activity|GO:1901363,heterocyclic compound binding|GO:0003700,transcription factor activity, sequence-specific DNA binding|GO:0001071,nucleic acid binding transcription factor activity|GO:0000981,RNA polymerase II transcription factor activity, sequence-specific DNA binding
CYBRD1	GO:0044710,single-organism metabolic process|GO:0010038,response to metal ion|GO:1990267,response to transition metal nanoparticle|GO:0055114,oxidation-reduction process|GO:0008152,metabolic process|GO:0010039,response to iron ion|GO:0042221,response to chemical|GO:0010035,response to inorganic substance|GO:0050896,response to stimulus|GO:0044699,single-organism process	GO:0098590,plasma membrane region|GO:0042995,cell projection|GO:0016020,membrane|GO:0044464,cell part|GO:0071944,cell periphery|GO:0016021,integral component of membrane|GO:0098589,membrane region|GO:0031253,cell projection membrane|GO:0005623,cell|GO:0031526,brush border membrane|GO:0005903,brush border|GO:0005886,plasma membrane|GO:0044425,membrane part|GO:0044463,cell projection part|GO:0044459,plasma membrane part|GO:0098805,whole membrane|GO:0031224,intrinsic component of membrane|GO:0098862,cluster of actin-based cell projections	GO:0000293,ferric-chelate reductase activity|GO:0016722,oxidoreductase activity, oxidizing metal ions|GO:0016723,oxidoreductase activity, oxidizing metal ions, NAD or NADP as acceptor|GO:0003824,catalytic activity|GO:0016491,oxidoreductase activity
DHFR	GO:0080090,regulation of primary metabolic process|GO:0019222,regulation of metabolic process|GO:0000082,G1/S transition of mitotic cell cycle|GO:0000083,regulation of transcription involved in G1/S transition of mitotic cell cycle|GO:0009165,nucleotide biosynthetic process|GO:0044281,small molecule metabolic process|GO:0044283,small molecule biosynthetic process|GO:1901362,organic cyclic compound biosynthetic process|GO:1901360,organic cyclic compound metabolic process|GO:0044710,single-organism metabolic process|GO:0044711,single-organism biosynthetic process|GO:0006545,glycine biosynthetic process|GO:0006544,glycine metabolic process|GO:0006767,water-soluble vitamin metabolic process|GO:0006766,vitamin metabolic process|GO:0006760,folic acid-containing compound metabolic process|GO:0043604,amide biosynthetic process|GO:0060255,regulation of macromolecule metabolic process|GO:2001141,regulation of RNA biosynthetic process|GO:0043436,oxoacid metabolic process|GO:0009070,serine family amino acid biosynthetic process|GO:0010033,response to organic substance|GO:0055114,oxidation-reduction process|GO:0046483,heterocycle metabolic process|GO:0043648,dicarboxylic acid metabolic process|GO:1901564,organonitrogen compound metabolic process|GO:0006082,organic acid metabolic process|GO:1901566,organonitrogen compound biosynthetic process|GO:0010243,response to organonitrogen compound|GO:0016053,organic acid biosynthetic process|GO:0019637,organophosphate metabolic process|GO:0019438,aromatic compound biosynthetic process|GO:0031427,response to methotrexate|GO:0042398,cellular modified amino acid biosynthetic process|GO:0014070,response to organic cyclic compound|GO:0006807,nitrogen compound metabolic process|GO:0046653,tetrahydrofolate metabolic process|GO:0046655,folic acid metabolic process|GO:0050789,regulation of biological process|GO:0097659,nucleic acid-templated transcription|GO:1901576,organic substance biosynthetic process|GO:0051341,regulation of oxidoreductase activity|GO:0051186,cofactor metabolic process|GO:0044260,cellular macromolecule metabolic process|GO:0051188,cofactor biosynthetic process|GO:0042558,pteridine-containing compound metabolic process|GO:0042559,pteridine-containing compound biosynthetic process|GO:0065007,biological regulation|GO:0044699,single-organism process|GO:0006366,transcription from RNA polymerase II promoter|GO:0065009,regulation of molecular function|GO:0018130,heterocycle biosynthetic process|GO:0032768,regulation of monooxygenase activity|GO:0050790,regulation of catalytic activity|GO:0009889,regulation of biosynthetic process|GO:0050794,regulation of cellular process|GO:0008152,metabolic process|GO:0034654,nucleobase-containing compound biosynthetic process|GO:0090407,organophosphate biosynthetic process|GO:0006575,cellular modified amino acid metabolic process|GO:0016070,RNA metabolic process|GO:0008652,cellular amino acid biosynthetic process|GO:0044271,cellular nitrogen compound biosynthetic process|GO:0046394,carboxylic acid biosynthetic process|GO:1901698,response to nitrogen compound|GO:0006355,regulation of transcription, DNA-templated|GO:0006357,regulation of transcription from RNA polymerase II promoter|GO:0006351,transcription, DNA-templated|GO:0044763,single-organism cellular process|GO:0006753,nucleoside phosphate metabolic process|GO:0032774,RNA biosynthetic process|GO:0006732,coenzyme metabolic process|GO:0006730,one-carbon metabolic process|GO:0044249,cellular biosynthetic process|GO:0034641,cellular nitrogen compound metabolic process|GO:0034645,cellular macromolecule biosynthetic process|GO:0042221,response to chemical|GO:0007049,cell cycle|GO:0009719,response to endogenous stimulus|GO:0006139,nucleobase-containing compound metabolic process|GO:0009396,folic acid-containing compound biosynthetic process|GO:1901605,alpha-amino acid metabolic process|GO:1901607,alpha-amino acid biosynthetic process|GO:1901700,response to oxygen-containing compound|GO:0009987,cellular process|GO:0006725,cellular aromatic compound metabolic process|GO:1903506,regulation of nucleic acid-templated transcription|GO:0019752,carboxylic acid metabolic process|GO:0055086,nucleobase-containing small molecule metabolic process|GO:0090304,nucleic acid metabolic process|GO:0043603,cellular amide metabolic process|GO:0051252,regulation of RNA metabolic process|GO:0043170,macromolecule metabolic process|GO:0009108,coenzyme biosynthetic process|GO:0050896,response to stimulus|GO:0072593,reactive oxygen species metabolic process|GO:0031326,regulation of cellular biosynthetic process|GO:0031323,regulation of cellular metabolic process|GO:1903047,mitotic cell cycle process|GO:2001057,reactive nitrogen species metabolic process|GO:0044770,cell cycle phase transition|GO:0044772,mitotic cell cycle phase transition|GO:0022402,cell cycle process|GO:0046209,nitric oxide metabolic process|GO:0009069,serine family amino acid metabolic process|GO:0006520,cellular amino acid metabolic process|GO:2000112,regulation of cellular macromolecule biosynthetic process|GO:0046654,tetrahydrofolate biosynthetic process|GO:0071704,organic substance metabolic process|GO:0010467,gene expression|GO:0010556,regulation of macromolecule biosynthetic process|GO:0010468,regulation of gene expression|GO:1901293,nucleoside phosphate biosynthetic process|GO:0000278,mitotic cell cycle|GO:0019219,regulation of nucleobase-containing compound metabolic process|GO:0009058,biosynthetic process|GO:0009059,macromolecule biosynthetic process|GO:0009117,nucleotide metabolic process|GO:0051171,regulation of nitrogen compound metabolic process|GO:0044843,cell cycle G1/S phase transition|GO:0050999,regulation of nitric-oxide synthase activity|GO:0044238,primary metabolic process|GO:0044237,cellular metabolic process|GO:0006796,phosphate-containing compound metabolic process|GO:0006793,phosphorus metabolic process|GO:0001101,response to acid chemical|GO:0009636,response to toxic substance|GO:0048583,regulation of response to stimulus|GO:0010605,negative regulation of macromolecule metabolic process|GO:0010608,posttranscriptional regulation of gene expression|GO:0043043,peptide biosynthetic process|GO:0009611,response to wounding|GO:0098869,cellular oxidant detoxification|GO:0048869,cellular developmental process|GO:0044093,positive regulation of molecular function|GO:0048518,positive regulation of biological process|GO:0048519,negative regulation of biological process|GO:0031103,axon regeneration|GO:0048468,cell development|GO:0048678,response to axon injury|GO:1990748,cellular detoxification|GO:0032268,regulation of cellular protein metabolic process|GO:0010035,response to inorganic substance|GO:0044707,single-multicellular organism process|GO:2000121,regulation of removal of superoxide radicals|GO:0019538,protein metabolic process|GO:0051353,positive regulation of oxidoreductase activity|GO:0019430,removal of superoxide radicals|GO:0033554,cellular response to stress|GO:0051716,cellular response to stimulus|GO:0009892,negative regulation of metabolic process|GO:0009893,positive regulation of metabolic process|GO:0009890,negative regulation of biosynthetic process|GO:0000303,response to superoxide|GO:0000302,response to reactive oxygen species|GO:0010629,negative regulation of gene expression|GO:0006801,superoxide metabolic process|GO:0098754,detoxification|GO:0071451,cellular response to superoxide|GO:0071450,cellular response to oxygen radical|GO:0061564,axon development|GO:2000377,regulation of reactive oxygen species metabolic process|GO:0006950,response to stress|GO:0046146,tetrahydrobiopterin metabolic process|GO:0080134,regulation of response to stress|GO:0080135,regulation of cellular response to stress|GO:0007275,multicellular organism development|GO:0006518,peptide metabolic process|GO:0010558,negative regulation of macromolecule biosynthetic process|GO:0032770,positive regulation of monooxygenase activity|GO:0031099,regeneration|GO:0030154,cell differentiation|GO:0090322,regulation of superoxide metabolic process|GO:0051000,positive regulation of nitric-oxide synthase activity|GO:0070887,cellular response to chemical stimulus|GO:0034599,cellular response to oxidative stress|GO:0043085,positive regulation of catalytic activity|GO:0051248,negative regulation of protein metabolic process|GO:0051246,regulation of protein metabolic process|GO:0000305,response to oxygen radical|GO:0017148,negative regulation of translation|GO:0032502,developmental process|GO:0032501,multicellular organismal process|GO:0034249,negative regulation of cellular amide metabolic process|GO:0034248,regulation of cellular amide	GO:0005737,cytoplasm|GO:0044446,intracellular organelle part|GO:0005829,cytosol|GO:0043231,intracellular membrane-bounded organelle|GO:0031981,nuclear lumen|GO:0043233,organelle lumen|GO:0005634,nucleus|GO:0044464,cell part|GO:0044444,cytoplasmic part|GO:0031974,membrane-enclosed lumen|GO:0005622,intracellular|GO:0005654,nucleoplasm|GO:0070013,intracellular organelle lumen|GO:0043229,intracellular organelle|GO:0044428,nuclear part|GO:0005623,cell|GO:0044424,intracellular part|GO:0043227,membrane-bounded organelle|GO:0043226,organelle|GO:0044422,organelle part|GO:0005739,mitochondrion	GO:0005488,binding|GO:0050662,coenzyme binding|GO:0050661,NADP binding|GO:0000166,nucleotide binding|GO:0044822,poly(A) RNA binding|GO:0016646,oxidoreductase activity, acting on the CH-NH group of donors, NAD or NADP as acceptor|GO:0016645,oxidoreductase activity, acting on the CH-NH group of donors|GO:0008144,drug binding|GO:0036094,small molecule binding|GO:0003824,catalytic activity|GO:0048037,cofactor binding|GO:0003723,RNA binding|GO:0003676,nucleic acid binding|GO:0097159,organic cyclic compound binding|GO:0004146,dihydrofolate reductase activity|GO:0003729,mRNA binding|GO:1901363,heterocyclic compound binding|GO:1901265,nucleoside phosphate binding|GO:0016491,oxidoreductase activity|GO:0043167,ion binding|GO:0043168,anion binding|GO:0000900,translation repressor activity, nucleic acid binding|GO:0005542,folic acid binding|GO:0033560,folate reductase activity|GO:0051870,methotrexate binding|GO:0033218,amide binding|GO:0030371,translation repressor activity|GO:0045182,translation regulator activity|GO:1990825,sequence-specific mRNA binding|GO:0090079,translation regulator activity, nucleic acid binding|GO:0072341,modified amino acid binding|GO:0043177,organic acid binding|GO:0031406,carboxylic acid binding|GO:0019842,vitamin binding|GO:0070402,NADPH binding
CTC			

PDCD5	GO:2001235,positive regulation of apoptotic signaling pathway|GO:0008104,protein localization|GO:0032388,positive regulation of intracellular transport|GO:0080090,regulation of primary metabolic process|GO:0019222,regulation of metabolic process|GO:2001233,regulation of apoptotic signaling pathway|GO:0051049,regulation of transport|GO:0032386,regulation of intracellular transport|GO:0048584,positive regulation of response to stimulus|GO:0048583,regulation of response to stimulus|GO:0061024,membrane organization|GO:0007008,outer mitochondrial membrane organization|GO:0007165,signal transduction|GO:0070585,protein localization to mitochondrion|GO:0034764,positive regulation of transmembrane transport|GO:0007006,mitochondrial membrane organization|GO:0034762,regulation of transmembrane transport|GO:0007005,mitochondrion organization|GO:0051716,cellular response to stimulus|GO:0010605,negative regulation of macromolecule metabolic process|GO:0010604,positive regulation of macromolecule metabolic process|GO:0070727,cellular macromolecule localization|GO:0009966,regulation of signal transduction|GO:0009967,positive regulation of signal transduction|GO:0071840,cellular component organization or biogenesis|GO:1990542,mitochondrial transmembrane transport|GO:0044093,positive regulation of molecular function|GO:0043281,regulation of cysteine-type endopeptidase activity involved in apoptotic process|GO:0043280,positive regulation of cysteine-type endopeptidase activity involved in apoptotic process|GO:0016482,cytosolic transport|GO:0042127,regulation of cell proliferation|GO:0051050,positive regulation of transport|GO:0060255,regulation of macromolecule metabolic process|GO:0071702,organic substance transport|GO:0008637,apoptotic mitochondrial changes|GO:0006605,protein targeting|GO:0045184,establishment of protein localization|GO:1903955,positive regulation of protein targeting to mitochondrion|GO:0030162,regulation of proteolysis|GO:0072657,protein localization to membrane|GO:0072655,establishment of protein localization to mitochondrion|GO:0006839,mitochondrial transport|GO:0010033,response to organic substance|GO:0010467,gene expression|GO:1903827,regulation of cellular protein localization|GO:0019538,protein metabolic process|GO:0010638,positive regulation of organelle organization|GO:0043068,positive regulation of programmed cell death|GO:0007154,cell communication|GO:0016265,obsolete death|GO:0009892,negative regulation of metabolic process|GO:0009893,positive regulation of metabolic process|GO:1903651,positive regulation of cytoplasmic transport|GO:0010628,positive regulation of gene expression|GO:0051222,positive regulation of protein transport|GO:0051223,regulation of protein transport|GO:0042981,regulation of apoptotic process|GO:0050789,regulation of biological process|GO:0044267,cellular protein metabolic process|GO:0033157,regulation of intracellular protein transport|GO:0051345,positive regulation of hydrolase activity|GO:0006886,intracellular protein transport|GO:0016043,cellular component organization|GO:0065002,intracellular protein transmembrane transport|GO:0070887,cellular response to chemical stimulus|GO:0065007,biological regulation|GO:1903829,positive regulation of cellular protein localization|GO:0065009,regulation of molecular function|GO:0006626,protein targeting to mitochondrion|GO:0034613,cellular protein localization|GO:0051130,positive regulation of cellular component organization|GO:0070201,regulation of establishment of protein localization|GO:1903644,regulation of chaperone-mediated protein folding|GO:0090199,regulation of release of cytochrome c from mitochondria|GO:1903649,regulation of cytoplasmic transport|GO:1903645,negative regulation of chaperone-mediated protein folding|GO:0050790,regulation of catalytic activity|GO:0050794,regulation of cellular process|GO:0012501,programmed cell death|GO:0044802,single-organism membrane organization|GO:0008152,metabolic process|GO:0051234,establishment of localization|GO:0061077,chaperone-mediated protein folding|GO:0051336,regulation of hydrolase activity|GO:0046907,intracellular transport|GO:0045040,protein import into mitochondrial outer membrane|GO:0050896,response to stimulus|GO:0033043,regulation of organelle organization|GO:0044699,single-organism process|GO:0071310,cellular response to organic substance|GO:0090200,positive regulation of release of cytochrome c from mitochondria|GO:0051128,regulation of cellular component organization|GO:0023056,positive regulation of signaling|GO:1903638,positive regulation of protein import into mitochondrial outer membrane|GO:0023052,signaling|GO:1903533,regulation of protein targeting|GO:0023051,regulation of signaling|GO:0006457,protein folding|GO:0010647,positive regulation of cell communication|GO:0010646,regulation of cell communication|GO:1903636,regulation of protein import into mitochondrial outer membrane|GO:0070848,response to growth factor|GO:0043085,positive regulation of catalytic activity|GO:0032880,regulation of protein localization|GO:0009719,response to endogenous stimulus|GO:0051248,negative regulation of protein metabolic process|GO:0071806,protein transmembrane transport|GO:0051246,regulation of protein metabolic process|GO:0051247,positive regulation of protein metabolic process|GO:0032270,positive regulation of cellular protein metabolic process|GO:0006810,transport|GO:0006508,proteolysis|GO:0071495,cellular response to endogenous stimulus|GO:0008285,negative regulation of cell proliferation|GO:0008283,cell proliferation|GO:0031323,regulation of cellular metabolic process|GO:0017038,protein import|GO:0048518,positive regulation of biological process|GO:0048519,negative regulation of biological process|GO:0032879,regulation of localization|GO:0055085,transmembrane transport|GO:0044743,protein transmembrane import into intracellular organelle|GO:1903749,positive regulation of establishment of protein localization to mitochondrion|GO:0042221,response to chemical|GO:0032269,negative regulation of cellular protein metabolic process|GO:0032268,regulation of cellular protein metabolic process|GO:0071363,cellular response to growth factor stimulus|GO:0043170,macromolecule metabolic process|GO:0071560,cellular response to transforming growth factor beta stimulus|GO:1903333,negative regulation of protein folding|GO:1903332,regulation of protein folding|GO:0033365,protein localization to organelle|GO:0045862,positive regulation of proteolysis|GO:0060341,regulation of cellular localization|GO:0044700,single organism signaling|GO:0031325,positive regulation of cellular metabolic process|GO:0031324,negative regulation of cellular metabolic process|GO:0097190,apoptotic signaling pathway|GO:2001056,positive regulation of cysteine-type endopeptidase activity|GO:0010821,regulation of mitochondrion organization|GO:0044237,cellular metabolic process|GO:0010822,positive regulation of mitochondrion organization|GO:0010942,positive regulation of cell death|GO:0008219,cell death|GO:0010941,regulation of cell death|GO:0033036,macromolecule localization|GO:2000116,regulation of cysteine-type endopeptidase activity|GO:0071559,response to transforming growth factor beta|GO:0043065,positive regulation of apoptotic process|GO:0071704,organic substance metabolic process|GO:0043067,regulation of programmed cell death|GO:0072594,establishment of protein localization to organelle|GO:0010468,regulation of gene expression|GO:0009987,cellular process|GO:0052547,regulation of peptidase activity|GO:0052548,regulation of endopeptidase activity|GO:0006915,apoptotic process|GO:0090316,positive regulation of intracellular protein transport|GO:0044765,single-organism transport|GO:0044763,single-organism cellular process|GO:0051649,establishment of localization in cell|GO:0010950,positive regulation of endopeptidase activity|GO:0010952,positive regulation of peptidase activity|GO:0051179,localization|GO:1902578,single-organism localization|GO:0051641,cellular localization|GO:0006996,organelle organization|GO:0044238,primary metabolic process|GO:0044260,cellular macromolecule metabolic process|GO:1903747,regulation of establishment of protein localization to mitochondrion|GO:0090151,establishment of protein localization to mitochondrial membrane|GO:0090150,establishment of protein localization to membrane|GO:0001836,release of cytochrome c from mitochondria|GO:1902589,single-organism organelle organization|GO:1903214,regulation of protein targeting to mitochondrion|GO:0015031,protein transport|GO:1902582,single-organism intracellular transport|GO:1902580,single-organism cellular localization|GO:0048523,negative regulation of cellular process|GO:0048522,positive regulation of	GO:0043231,intracellular membrane-bounded organelle|GO:0005737,cytoplasm|GO:0005622,intracellular|GO:0043230,extracellular organelle|GO:0031982,vesicle|GO:0044421,extracellular region part|GO:0043227,membrane-bounded organelle|GO:0005634,nucleus|GO:0044464,cell part|GO:0005623,cell|GO:0031988,membrane-bounded vesicle|GO:0043229,intracellular organelle|GO:0005576,extracellular region|GO:0044424,intracellular part|GO:1903561,extracellular vesicle|GO:0065010,extracellular membrane-bounded organelle|GO:0043226,organelle|GO:0070062,extracellular exosome|GO:0005829,cytosol|GO:0044444,cytoplasmic part	GO:1901681,sulfur compound binding|GO:0043168,anion binding|GO:0015631,tubulin binding|GO:0005515,protein binding|GO:0048487,beta-tubulin binding|GO:0097367,carbohydrate derivative binding|GO:0097159,organic cyclic compound binding|GO:0030234,enzyme regulator activity|GO:0008092,cytoskeletal protein binding|GO:0008201,heparin binding|GO:0005539,glycosaminoglycan binding|GO:0043167,ion binding|GO:0010698,acetyltransferase activator activity|GO:0008047,enzyme activator activity|GO:0003676,nucleic acid binding|GO:0003677,DNA binding|GO:0098772,molecular function regulator|GO:1901363,heterocyclic compound binding|GO:0005488,binding
SMIM9		GO:0016021,integral component of membrane|GO:0016020,membrane|GO:0044464,cell part|GO:0005623,cell|GO:0071944,cell periphery|GO:0005886,plasma membrane|GO:0044425,membrane part|GO:0031224,intrinsic component of membrane	
SCFD1	GO:0008104,protein localization|GO:0006909,phagocytosis|GO:0019222,regulation of metabolic process|GO:0051049,regulation of transport|GO:0048585,negative regulation of response to stimulus|GO:0048583,regulation of response to stimulus|GO:0071840,cellular component organization or biogenesis|GO:0044712,single-organism catabolic process|GO:0044710,single-organism metabolic process|GO:0001666,response to hypoxia|GO:0048869,cellular developmental process|GO:0033043,regulation of organelle organization|GO:0006904,vesicle docking involved in exocytosis|GO:0048519,negative regulation of biological process|GO:0033036,macromolecule localization|GO:0032386,regulation of intracellular transport|GO:0045184,establishment of protein localization|GO:0048278,vesicle docking|GO:0051129,negative regulation of cellular component organization|GO:0031668,cellular response to extracellular stimulus|GO:0031669,cellular response to nutrient levels|GO:0016192,vesicle-mediated transport|GO:0009605,response to external stimulus|GO:0010639,negative regulation of organelle organization|GO:0010507,negative regulation of autophagy|GO:0033554,cellular response to stress|GO:0009894,regulation of catabolic process|GO:0009895,negative regulation of catabolic process|GO:0009892,negative regulation of metabolic process|GO:0031667,response to nutrient levels|GO:0008152,metabolic process|GO:0032940,secretion by cell|GO:0051223,regulation of protein transport|GO:0050789,regulation of biological process|GO:0009653,anatomical structure morphogenesis|GO:0006888,ER to Golgi vesicle-mediated transport|GO:0000902,cell morphogenesis|GO:0006887,exocytosis|GO:0016043,cellular component organization|GO:0065007,biological regulation|GO:0070201,regulation of establishment of protein localization|GO:1903649,regulation of cytoplasmic transport|GO:0006810,transport|GO:0051716,cellular response to stimulus|GO:0050794,regulation of cellular process|GO:0006950,response to stress|GO:0009267,cellular response to starvation|GO:0051234,establishment of localization|GO:0006892,post-Golgi vesicle-mediated transport|GO:0046903,secretion|GO:0006890,retrograde vesicle-mediated transport, Golgi to ER|GO:0006897,endocytosis|GO:0046907,intracellular transport|GO:0036293,response to decreased oxygen levels|GO:0080134,regulation of response to stress|GO:0080135,regulation of cellular response to stress|GO:0032107,regulation of response to nutrient levels|GO:0032104,regulation of response to extracellular stimulus|GO:0032105,negative regulation of response to extracellular stimulus|GO:0032102,negative regulation of response to external stimulus|GO:2000785,regulation of autophagosome assembly|GO:0032101,regulation of response to external stimulus|GO:0032108,negative regulation of response to nutrient levels|GO:0051128,regulation of cellular component organization|GO:0044248,cellular catabolic process|GO:0022607,cellular component assembly|GO:0010648,negative regulation of cell communication|GO:0042221,response to chemical|GO:0010646,regulation of cell communication|GO:1901998,toxin transport|GO:0044699,single-organism process|GO:0032880,regulation of protein localization|GO:0060341,regulation of cellular localization|GO:0000045,autophagosome assembly|GO:0070482,response to oxygen levels|GO:0060628,regulation of ER to Golgi vesicle-mediated transport|GO:0071496,cellular response to external stimulus|GO:0031330,negative regulation of cellular catabolic process|GO:0016236,macroautophagy|GO:0009987,cellular process|GO:0060627,regulation of vesicle-mediated transport|GO:0032879,regulation of localization|GO:0016482,cytosolic transport|GO:0007033,vacuole organization|GO:1902902,negative regulation of autophagosome assembly|GO:0050896,response to stimulus|GO:0032502,developmental process|GO:0009991,response to extracellular stimulus|GO:0031329,regulation of cellular catabolic process|GO:0031324,negative regulation of cellular metabolic process|GO:0031323,regulation of cellular metabolic process|GO:0022406,membrane docking|GO:0042594,response to starvation|GO:0009636,response to toxic substance|GO:0032989,cellular component morphogenesis|GO:1902115,regulation of organelle assembly|GO:1902116,negative regulation of organelle assembly|GO:0071702,organic substance transport|GO:0010506,regulation of autophagy|GO:0016242,negative regulation of macroautophagy|GO:0016241,regulation of macroautophagy|GO:0048193,Golgi vesicle transport|GO:0006914,autophagy|GO:0044767,single-organism developmental process|GO:0044765,single-organism transport|GO:0044763,single-organism cellular process|GO:0051649,establishment of localization in cell|GO:0007154,cell communication|GO:0070925,organelle assembly|GO:0009056,catabolic process|GO:0051179,localization|GO:1902578,single-organism localization|GO:0051641,cellular localization|GO:0006996,organelle organization|GO:0009628,response to abiotic stimulus|GO:0048856,anatomical structure development|GO:0044237,cellular metabolic process|GO:0044087,regulation of cellular component biogenesis|GO:0044085,cellular component biogenesis|GO:0048523,negative regulation of cellular process|GO:0015031,protein transport|GO:1902582,single-organism intracellular transport|GO:0044088,regulation of vacuole organization|GO:0006901,vesicle coating|GO:0006900,membrane budding|GO:0006903,vesicle targeting|GO:0061024,membrane organization|GO:0051656,establishment of organelle localization|GO:0051650,establishment of vesicle localization|GO:0070271,protein complex biogenesis|GO:1905037,autophagosome organization|GO:0051668,localization within membrane|GO:0016050,vesicle organization|GO:0065003,macromolecular complex assembly|GO:0044802,single-organism membrane organization|GO:0090114,COPII-coated vesicle budding|GO:0015833,peptide transport|GO:0042886,amide transport|GO:0051640,organelle localization|GO:1902591,single-organism membrane budding|GO:0048208,COPII vesicle coating|GO:0043933,macromolecular complex subunit organization|GO:0048207,vesicle targeting, rough ER to cis-Golgi|GO:0071822,protein complex subunit organization|GO:0071705,nitrogen compound transport|GO:0006461,protein complex assembly|GO:0048199,vesicle targeting, to, from or within Golgi|GO:0051648,vesicle localization|GO:1902589,single-organism organelle organization|GO:1902580,single-organism cellular localization	GO:0005783,endoplasmic reticulum|GO:0005789,endoplasmic reticulum membrane|GO:0042175,nuclear outer membrane-endoplasmic reticulum membrane network|GO:0043229,intracellular organelle|GO:0071944,cell periphery|GO:0031201,SNARE complex|GO:0005622,intracellular|GO:0043227,membrane-bounded organelle|GO:0043226,organelle|GO:0005737,cytoplasm|GO:0031985,Golgi cisterna|GO:0031984,organelle subcompartment|GO:0031982,vesicle|GO:0016023,cytoplasmic, membrane-bounded vesicle|GO:0031410,cytoplasmic vesicle|GO:0016020,membrane|GO:0031988,membrane-bounded vesicle|GO:0044432,endoplasmic reticulum part|GO:0044431,Golgi apparatus part|GO:0005795,Golgi stack|GO:0005794,Golgi apparatus|GO:0017119,Golgi transport complex|GO:0098588,bounding membrane of organelle|GO:0005798,Golgi-associated vesicle|GO:0012505,endomembrane system|GO:0005886,plasma membrane|GO:0032580,Golgi cisterna membrane|GO:0031090,organelle membrane|GO:0043234,protein complex|GO:0032991,macromolecular complex|GO:0043231,intracellular membrane-bounded organelle|GO:0005801,cis-Golgi network|GO:0044464,cell part|GO:0005623,cell|GO:0000139,Golgi membrane|GO:0044446,intracellular organelle part|GO:0098796,membrane protein complex|GO:0044444,cytoplasmic part|GO:0098791,Golgi subcompartment|GO:0044424,intracellular part|GO:0044425,membrane part|GO:0044422,organelle part|GO:0098827,endoplasmic reticulum subcompartment|GO:0097708,intracellular vesicle|GO:0005829,cytosol	GO:0047485,protein N-terminus binding|GO:0000149,SNARE binding|GO:0005488,binding|GO:0005515,protein binding|GO:0019905,syntaxin binding
MRPL13	GO:0043933,macromolecular complex subunit organization|GO:0044249,cellular biosynthetic process|GO:0034641,cellular nitrogen compound metabolic process|GO:0006807,nitrogen compound metabolic process|GO:0034645,cellular macromolecule biosynthetic process|GO:1901576,organic substance biosynthetic process|GO:0043241,protein complex disassembly|GO:1901566,organonitrogen compound biosynthetic process|GO:0071840,cellular component organization or biogenesis|GO:0044267,cellular protein metabolic process|GO:0044710,single-organism metabolic process|GO:0044711,single-organism biosynthetic process|GO:0071822,protein complex subunit organization|GO:0043043,peptide biosynthetic process|GO:0016043,cellular component organization|GO:0071704,organic substance metabolic process|GO:0010467,gene expression|GO:0007005,mitochondrion organization|GO:0032543,mitochondrial translation|GO:0070126,mitochondrial translational termination|GO:0070124,mitochondrial translational initiation|GO:0070125,mitochondrial translational elongation|GO:0044238,primary metabolic process|GO:0009987,cellular process|GO:0032984,macromolecular complex disassembly|GO:0009058,biosynthetic process|GO:0009059,macromolecule biosynthetic process|GO:0022411,cellular component disassembly|GO:0008152,metabolic process|GO:1901564,organonitrogen compound metabolic process|GO:0043624,cellular protein complex disassembly|GO:0006518,peptide metabolic process|GO:0006996,organelle organization|GO:0043604,amide biosynthetic process|GO:0044699,single-organism process|GO:0044271,cellular nitrogen compound biosynthetic process|GO:0044260,cellular macromolecule metabolic process|GO:0043603,cellular amide metabolic process|GO:0019538,protein metabolic process|GO:0044237,cellular metabolic process|GO:0043170,macromolecule metabolic process|GO:1902589,single-organism organelle organization|GO:0044763,single-organism cellular process|GO:0006415,translational termination|GO:0006414,translational elongation|GO:0006413,translational initiation|GO:0006412,translation	GO:0015934,large ribosomal subunit|GO:0044464,cell part|GO:0031974,membrane-enclosed lumen|GO:0031975,envelope|GO:0043229,intracellular organelle|GO:0043228,non-membrane-bounded organelle|GO:0043227,membrane-bounded organelle|GO:0043226,organelle|GO:0005737,cytoplasm|GO:0031090,organelle membrane|GO:0070013,intracellular organelle lumen|GO:0016020,membrane|GO:0044444,cytoplasmic part|GO:0005739,mitochondrion|GO:0030529,intracellular ribonucleoprotein complex|GO:0005759,mitochondrial matrix|GO:0044429,mitochondrial part|GO:0000315,organellar large ribosomal subunit|GO:0000313,organellar ribosome|GO:0031967,organelle envelope|GO:0044391,ribosomal subunit|GO:0031966,mitochondrial membrane|GO:0005840,ribosome|GO:0032991,macromolecular complex|GO:0043231,intracellular membrane-bounded organelle|GO:0043232,intracellular non-membrane-bounded organelle|GO:0043233,organelle lumen|GO:0019866,organelle inner membrane|GO:0005623,cell|GO:0005622,intracellular|GO:0044446,intracellular organelle part|GO:0005743,mitochondrial inner membrane|GO:0005740,mitochondrial envelope|GO:0005761,mitochondrial ribosome|GO:0005762,mitochondrial large ribosomal subunit|GO:0044424,intracellular part|GO:0044422,organelle part|GO:1990904,ribonucleoprotein complex	GO:0005488,binding|GO:0005515,protein binding|GO:0097159,organic cyclic compound binding|GO:0044822,poly(A) RNA binding|GO:0005198,structural molecule activity|GO:0003735,structural constituent of ribosome|GO:0003723,RNA binding|GO:0003676,nucleic acid binding|GO:1901363,heterocyclic compound binding|GO:0003729,mRNA binding
MAP3K2	GO:0019220,regulation of phosphate metabolic process|GO:0080090,regulation of primary metabolic process|GO:0019222,regulation of metabolic process|GO:0048584,positive regulation of response to stimulus|GO:0048583,regulation of response to stimulus|GO:0032147,activation of protein kinase activity|GO:0007165,signal transduction|GO:1901362,organic cyclic compound biosynthetic process|GO:0023014,signal transduction by protein phosphorylation|GO:0044710,single-organism metabolic process|GO:0010604,positive regulation of macromolecule metabolic process|GO:0009966,regulation of signal transduction|GO:0009967,positive regulation of signal transduction|GO:0000165,MAPK cascade|GO:0009612,response to mechanical stimulus|GO:0044093,positive regulation of molecular function|GO:0048518,positive regulation of biological process|GO:0060255,regulation of macromolecule metabolic process|GO:0045859,regulation of protein kinase activity|GO:0046330,positive regulation of JNK cascade|GO:2001141,regulation of RNA biosynthetic process|GO:0046483,heterocycle metabolic process|GO:0042325,regulation of phosphorylation|GO:0044700,single organism signaling|GO:0042327,positive regulation of phosphorylation|GO:0009605,response to external stimulus|GO:0044249,cellular biosynthetic process|GO:0019538,protein metabolic process|GO:0034641,cellular nitrogen compound metabolic process|GO:0033554,cellular response to stress|GO:0019438,aromatic compound biosynthetic process|GO:0009893,positive regulation of metabolic process|GO:0009891,positive regulation of biosynthetic process|GO:0071260,cellular response to mechanical stimulus|GO:0010628,positive regulation of gene expression|GO:0046328,regulation of JNK cascade|GO:0035556,intracellular signal transduction|GO:0071900,regulation of protein serine/threonine kinase activity|GO:0050789,regulation of biological process|GO:0097659,nucleic acid-templated transcription|GO:0044267,cellular protein metabolic process|GO:0051347,positive regulation of transferase activity|GO:0044260,cellular macromolecule metabolic process|GO:0043549,regulation of kinase activity|GO:0065007,biological regulation|GO:0044699,single-organism process|GO:0065009,regulation of molecular function|GO:0071214,cellular response to abiotic stimulus|GO:0018130,heterocycle biosynthetic process|GO:0006139,nucleobase-containing compound metabolic process|GO:0050790,regulation of catalytic activity|GO:0009889,regulation of biosynthetic process|GO:0051716,cellular response to stimulus|GO:0050794,regulation of cellular process|GO:0043410,positive regulation of MAPK cascade|GO:0043412,macromolecule modification|GO:0036211,protein modification process|GO:0008152,metabolic process|GO:0034654,nucleobase-containing compound biosynthetic process|GO:1902533,positive regulation of intracellular signal transduction|GO:1902531,regulation of intracellular signal transduction|GO:0016070,RNA metabolic process|GO:0044271,cellular nitrogen compound biosynthetic process|GO:0080134,regulation of response to stress|GO:0031401,positive regulation of protein modification process|GO:0006950,response to stress|GO:0051338,regulation of transferase activity|GO:0006355,regulation of transcription, DNA-templated|GO:0010557,positive regulation of macromolecule biosynthetic process|GO:0010556,regulation of macromolecule biosynthetic process|GO:0006351,transcription, DNA-templated|GO:0051171,regulation of nitrogen compound metabolic process|GO:0032774,RNA biosynthetic process|GO:0016310,phosphorylation|GO:0031098,stress-activated protein kinase signaling cascade|GO:0023056,positive regulation of signaling|GO:0043406,positive regulation of MAP kinase activity|GO:0043405,regulation of MAP kinase activity|GO:0023052,signaling|GO:0034645,cellular macromolecule biosynthetic process|GO:0023051,regulation of signaling|GO:0010647,positive regulation of cell communication|GO:0010646,regulation of cell communication|GO:0043085,positive regulation of catalytic activity|GO:0043408,regulation of MAPK cascade|GO:0007257,activation of JUN kinase activity|GO:0007254,JNK cascade|GO:0010562,positive regulation of phosphorus metabolic process|GO:0033674,positive regulation of kinase activity|GO:0051246,regulation of protein metabolic process|GO:0051247,positive regulation of protein metabolic process|GO:0032270,positive regulation of cellular protein metabolic process|GO:0031399,regulation of protein modification process|GO:1903508,positive regulation of nucleic acid-templated transcription|GO:0071496,cellular response to external stimulus|GO:0009628,response to abiotic stimulus|GO:0070302,regulation of stress-activated protein kinase signaling cascade|GO:0009987,cellular process|GO:0006725,cellular aromatic compound metabolic process|GO:0070304,positive regulation of stress-activated protein kinase signaling cascade|GO:0032872,regulation of stress-activated MAPK cascade|GO:0045893,positive regulation of transcription, DNA-templated|GO:0032874,positive regulation of stress-activated MAPK cascade|GO:0032268,regulation of cellular protein metabolic process|GO:0051252,regulation of RNA metabolic process|GO:0051254,positive regulation of RNA metabolic process|GO:0009059,macromolecule biosynthetic process|GO:0043170,macromolecule metabolic process|GO:1902680,positive regulation of RNA biosynthetic process|GO:0006807,nitrogen compound metabolic process|GO:0045860,positive regulation of protein kinase activity|GO:0050896,response to stimulus|GO:0000187,activation of MAPK activity|GO:0000186,activation of MAPKK activity|GO:0080135,regulation of cellular response to stress|GO:0031328,positive regulation of cellular biosynthetic process|GO:0031326,regulation of cellular biosynthetic process|GO:0031325,positive regulation of cellular metabolic process|GO:0031323,regulation of cellular metabolic process|GO:0090304,nucleic acid metabolic process|GO:0071902,positive regulation of protein serine/threonine kinase activity|GO:0043506,regulation of JUN kinase activity|GO:0043507,positive regulation of JUN kinase activity|GO:1901360,organic cyclic compound metabolic process|GO:2000112,regulation of cellular macromolecule biosynthetic process|GO:0071704,organic substance metabolic process|GO:0010467,gene expression|GO:0010468,regulation of gene expression|GO:0006468,protein phosphorylation|GO:0045935,positive regulation of nucleobase-containing compound metabolic process|GO:0045937,positive regulation of phosphate metabolic process|GO:1901576,organic substance biosynthetic process|GO:0019219,regulation of nucleobase-containing compound metabolic process|GO:0006464,cellular protein modification process|GO:0051174,regulation of phosphorus metabolic process|GO:0009058,biosynthetic process|GO:0051403,stress-activated MAPK cascade|GO:0044763,single-organism cellular process|GO:1903506,regulation of nucleic acid-templated transcription|GO:0051173,positive regulation of nitrogen compound metabolic process|GO:0007154,cell communication|GO:0044238,primary metabolic process|GO:0044237,cellular metabolic process|GO:0006796,phosphate-containing compound metabolic process|GO:0006793,phosphorus metabolic process|GO:0001932,regulation of protein phosphorylation|GO:0001934,positive regulation of protein phosphorylation|GO:0048522,positive regulation of cellular process|GO:1901564,organonitrogen compound metabolic process|GO:0042981,regulation of apoptotic process|GO:0007346,regulation of mitotic cell cycle|GO:0007049,cell cycle|GO:0012501,programmed cell death|GO:0008219,cell	GO:0005737,cytoplasm|GO:0044446,intracellular organelle part|GO:0005829,cytosol|GO:0043231,intracellular membrane-bounded organelle|GO:0031981,nuclear lumen|GO:0043233,organelle lumen|GO:0005634,nucleus|GO:0044464,cell part|GO:0044444,cytoplasmic part|GO:0031974,membrane-enclosed lumen|GO:0005622,intracellular|GO:0005654,nucleoplasm|GO:0070013,intracellular organelle lumen|GO:0043229,intracellular organelle|GO:0044428,nuclear part|GO:0005623,cell|GO:0044424,intracellular part|GO:0043227,membrane-bounded organelle|GO:0043226,organelle|GO:0044422,organelle part	GO:0035639,purine ribonucleoside triphosphate binding|GO:0019899,enzyme binding|GO:0060089,molecular transducer activity|GO:0005488,binding|GO:0004871,signal transducer activity|GO:0043167,ion binding|GO:1901363,heterocyclic compound binding|GO:0001883,purine nucleoside binding|GO:0001882,nucleoside binding|GO:0043168,anion binding|GO:0043169,cation binding|GO:0019900,kinase binding|GO:0005057,signal transducer activity, downstream of receptor|GO:0032549,ribonucleoside binding|GO:0000166,nucleotide binding|GO:0004674,protein serine/threonine kinase activity|GO:0016740,transferase activity|GO:0004712,protein serine/threonine/tyrosine kinase activity|GO:1901265,nucleoside phosphate binding|GO:0004672,protein kinase activity|GO:0017076,purine nucleotide binding|GO:0046872,metal ion binding|GO:0005524,ATP binding|GO:0016301,kinase activity|GO:0036094,small molecule binding|GO:0003824,catalytic activity|GO:0019901,protein kinase binding|GO:0030554,adenyl nucleotide binding|GO:0016773,phosphotransferase activity, alcohol group as acceptor|GO:0016772,transferase activity, transferring phosphorus-containing groups|GO:0004708,MAP kinase kinase activity|GO:0097367,carbohydrate derivative binding|GO:0032559,adenyl ribonucleotide binding|GO:0005515,protein binding|GO:0004702,signal transducer, downstream of receptor, with serine/threonine kinase activity|GO:0032555,purine ribonucleotide binding|GO:0097159,organic cyclic compound binding|GO:0004709,MAP kinase kinase kinase activity|GO:0032550,purine ribonucleoside binding|GO:0032553,ribonucleotide binding
DRAXIN	GO:0048675,axon extension|GO:0048589,developmental growth|GO:0048588,developmental cell growth|GO:0021515,cell differentiation in spinal cord|GO:0048585,negative regulation of response to stimulus|GO:0021516,dorsal spinal cord development|GO:0048583,regulation of response to stimulus|GO:0021510,spinal cord development|GO:0030111,regulation of Wnt signaling pathway|GO:0043523,regulation of neuron apoptotic process|GO:0043524,negative regulation of neuron apoptotic process|GO:0007165,signal transduction|GO:0007166,cell surface receptor signaling pathway|GO:0061387,regulation of extent of cell growth|GO:0031345,negative regulation of cell projection organization|GO:0031344,regulation of cell projection organization|GO:0071840,cellular component organization or biogenesis|GO:0051716,cellular response to stimulus|GO:0009968,negative regulation of signal transduction|GO:0009966,regulation of signal transduction|GO:0048869,cellular developmental process|GO:0045665,negative regulation of neuron differentiation|GO:0045664,regulation of neuron differentiation|GO:0048513,animal organ development|GO:0010721,negative regulation of cell development|GO:0048519,negative regulation of biological process|GO:1990138,neuron projection extension|GO:0021528,commissural neuron differentiation in spinal cord|GO:0060548,negative regulation of cell death|GO:0008361,regulation of cell size|GO:0010977,negative regulation of neuron projection development|GO:0010975,regulation of neuron projection development|GO:0030517,negative regulation of axon extension|GO:0030516,regulation of axon extension|GO:0097485,neuron projection guidance|GO:0044700,single organism signaling|GO:0009605,response to external stimulus|GO:0044707,single-multicellular organism process|GO:0016055,Wnt signaling pathway|GO:0060322,head development|GO:0060828,regulation of canonical Wnt signaling pathway|GO:0010648,negative regulation of cell communication|GO:0022604,regulation of cell morphogenesis|GO:0048666,neuron development|GO:0022603,regulation of anatomical structure morphogenesis|GO:0006928,movement of cell or subcellular component|GO:0031175,neuron projection development|GO:0042981,regulation of apoptotic process|GO:0030178,negative regulation of Wnt signaling pathway|GO:0000904,cell morphogenesis involved in differentiation|GO:0016049,cell growth|GO:0000902,cell morphogenesis|GO:0070997,neuron death|GO:0016043,cellular component organization|GO:0090066,regulation of anatomical structure size|GO:0065007,biological regulation|GO:0048640,negative regulation of developmental growth|GO:0065008,regulation of biological quality|GO:0016265,obsolete death|GO:0061564,axon development|GO:0050793,regulation of developmental process|GO:0048468,cell development|GO:0050794,regulation of cellular process|GO:0060070,canonical Wnt signaling pathway|GO:0051239,regulation of multicellular organismal process|GO:1901214,regulation of neuron death|GO:1901215,negative regulation of neuron death|GO:0007420,brain development|GO:0012501,programmed cell death|GO:0050896,response to stimulus|GO:0051961,negative regulation of nervous system development|GO:0051960,regulation of nervous system development|GO:0032535,regulation of cellular component size|GO:0048638,regulation of developmental growth|GO:0030308,negative regulation of cell growth|GO:0030154,cell differentiation|GO:0051129,negative regulation of cellular component organization|GO:0051128,regulation of cellular component organization|GO:0023057,negative regulation of signaling|GO:0023052,signaling|GO:0060284,regulation of cell development|GO:0042330,taxis|GO:0023051,regulation of signaling|GO:0007411,axon guidance|GO:0010646,regulation of cell communication|GO:0044699,single-organism process|GO:0007417,central nervous system development|GO:0050767,regulation of neurogenesis|GO:0009653,anatomical structure morphogenesis|GO:0051241,negative regulation of multicellular organismal process|GO:0050768,negative regulation of neurogenesis|GO:0010769,regulation of cell morphogenesis involved in differentiation|GO:0060560,developmental growth involved in morphogenesis|GO:0021953,central nervous system neuron differentiation|GO:0032502,developmental process|GO:0032501,multicellular organismal process|GO:0009987,cellular process|GO:0045596,negative regulation of cell differentiation|GO:0045595,regulation of cell differentiation|GO:0001558,regulation of cell growth|GO:0007409,axonogenesis|GO:0032990,cell part morphogenesis|GO:0051093,negative regulation of developmental process|GO:0042221,response to chemical|GO:0050771,negative regulation of axonogenesis|GO:0050770,regulation of axonogenesis|GO:0010771,negative regulation of cell morphogenesis involved in differentiation|GO:0048731,system development|GO:0045926,negative regulation of growth|GO:0048812,neuron projection morphogenesis|GO:0030030,cell projection organization|GO:0008219,cell death|GO:0010941,regulation of cell death|GO:0007275,multicellular organism development|GO:0006935,chemotaxis|GO:0040007,growth|GO:0040008,regulation of growth|GO:0050789,regulation of biological process|GO:0043067,regulation of programmed cell death|GO:0043066,negative regulation of apoptotic process|GO:2000026,regulation of multicellular organismal development|GO:0043069,negative regulation of programmed cell death|GO:0097285,obsolete cell-type specific apoptotic process|GO:0090090,negative regulation of canonical Wnt signaling pathway|GO:0048667,cell morphogenesis involved in neuron differentiation|GO:0030182,neuron differentiation|GO:0006915,apoptotic process|GO:0044767,single-organism developmental process|GO:0051402,neuron apoptotic process|GO:0044763,single-organism cellular process|GO:0007154,cell communication|GO:0022008,neurogenesis|GO:0040011,locomotion|GO:0048699,generation of neurons|GO:0048858,cell projection morphogenesis|GO:0007399,nervous system development|GO:0048856,anatomical structure development|GO:0030900,forebrain development|GO:0032989,cellular component morphogenesis|GO:0048523,negative regulation of cellular process|GO:1905114,cell surface receptor signaling pathway involved in cell-cell signaling|GO:0198738,cell-cell signaling by wnt|GO:0007267,cell-cell signaling	GO:0005576,extracellular region	GO:0005488,binding|GO:0005515,protein binding
PLEKHG4B	GO:0009893,positive regulation of metabolic process|GO:0019222,regulation of metabolic process|GO:0035023,regulation of Rho protein signal transduction|GO:0048583,regulation of response to stimulus|GO:0023052,signaling|GO:0007165,signal transduction|GO:0023051,regulation of signaling|GO:0035556,intracellular signal transduction|GO:0010646,regulation of cell communication|GO:0043087,regulation of GTPase activity|GO:0050789,regulation of biological process|GO:0043085,positive regulation of catalytic activity|GO:0051716,cellular response to stimulus|GO:1902531,regulation of intracellular signal transduction|GO:0043547,positive regulation of GTPase activity|GO:0051345,positive regulation of hydrolase activity|GO:0009966,regulation of signal transduction|GO:0065007,biological regulation|GO:0044699,single-organism process|GO:0044093,positive regulation of molecular function|GO:0048518,positive regulation of biological process|GO:0065009,regulation of molecular function|GO:0051056,regulation of small GTPase mediated signal transduction|GO:0009987,cellular process|GO:0046578,regulation of Ras protein signal transduction|GO:0050790,regulation of catalytic activity|GO:0050794,regulation of cellular process|GO:0044763,single-organism cellular process|GO:0008152,metabolic process|GO:0007266,Rho protein signal transduction|GO:0007265,Ras protein signal transduction|GO:0007264,small GTPase mediated signal transduction|GO:0051336,regulation of hydrolase activity|GO:0044700,single organism signaling|GO:0050896,response to stimulus|GO:0007154,cell communication		GO:0005089,Rho guanyl-nucleotide exchange factor activity|GO:0005088,Ras guanyl-nucleotide exchange factor activity|GO:0098772,molecular function regulator|GO:0005085,guanyl-nucleotide exchange factor activity|GO:0005488,binding|GO:0005515,protein binding
NFIC	GO:0080090,regulation of primary metabolic process|GO:0019222,regulation of metabolic process|GO:1901362,organic cyclic compound biosynthetic process|GO:1901360,organic cyclic compound metabolic process|GO:0010605,negative regulation of macromolecule metabolic process|GO:0010604,positive regulation of macromolecule metabolic process|GO:0048513,animal organ development|GO:0006260,DNA replication|GO:0048518,positive regulation of biological process|GO:0048519,negative regulation of biological process|GO:0060255,regulation of macromolecule metabolic process|GO:2001141,regulation of RNA biosynthetic process|GO:0046483,heterocycle metabolic process|GO:0044707,single-multicellular organism process|GO:0019438,aromatic compound biosynthetic process|GO:0009892,negative regulation of metabolic process|GO:0009893,positive regulation of metabolic process|GO:0009890,negative regulation of biosynthetic process|GO:0009891,positive regulation of biosynthetic process|GO:0010629,negative regulation of gene expression|GO:0010628,positive regulation of gene expression|GO:0050789,regulation of biological process|GO:0097659,nucleic acid-templated transcription|GO:1901576,organic substance biosynthetic process|GO:0044260,cellular macromolecule metabolic process|GO:0065007,biological regulation|GO:0006366,transcription from RNA polymerase II promoter|GO:0018130,heterocycle biosynthetic process|GO:0009887,animal organ morphogenesis|GO:0009889,regulation of biosynthetic process|GO:0050794,regulation of cellular process|GO:0008152,metabolic process|GO:0034654,nucleobase-containing compound biosynthetic process|GO:0016070,RNA metabolic process|GO:1902679,negative regulation of RNA biosynthetic process|GO:0044271,cellular nitrogen compound biosynthetic process|GO:0006355,regulation of transcription, DNA-templated|GO:0010557,positive regulation of macromolecule biosynthetic process|GO:0010556,regulation of macromolecule biosynthetic process|GO:0006351,transcription, DNA-templated|GO:0010558,negative regulation of macromolecule biosynthetic process|GO:0032774,RNA biosynthetic process|GO:0044249,cellular biosynthetic process|GO:0034641,cellular nitrogen compound metabolic process|GO:0034645,cellular macromolecule biosynthetic process|GO:0009653,anatomical structure morphogenesis|GO:0044699,single-organism process|GO:0006139,nucleobase-containing compound metabolic process|GO:0000122,negative regulation of transcription from RNA polymerase II promoter|GO:0032502,developmental process|GO:0032501,multicellular organismal process|GO:0009987,cellular process|GO:0006725,cellular aromatic compound metabolic process|GO:1903506,regulation of nucleic acid-templated transcription|GO:1903507,negative regulation of nucleic acid-templated transcription|GO:0045892,negative regulation of transcription, DNA-templated|GO:0045893,positive regulation of transcription, DNA-templated|GO:0051253,negative regulation of RNA metabolic process|GO:0051252,regulation of RNA metabolic process|GO:0051254,positive regulation of RNA metabolic process|GO:0043170,macromolecule metabolic process|GO:1902680,positive regulation of RNA biosynthetic process|GO:0006807,nitrogen compound metabolic process|GO:0045944,positive regulation of transcription from RNA polymerase II promoter|GO:0048731,system development|GO:1903508,positive regulation of nucleic acid-templated transcription|GO:0031328,positive regulation of cellular biosynthetic process|GO:0031327,negative regulation of cellular biosynthetic process|GO:0031326,regulation of cellular biosynthetic process|GO:0031325,positive regulation of cellular metabolic process|GO:0031324,negative regulation of cellular metabolic process|GO:0031323,regulation of cellular metabolic process|GO:0090304,nucleic acid metabolic process|GO:0007275,multicellular organism development|GO:2000112,regulation of cellular macromolecule biosynthetic process|GO:2000113,negative regulation of cellular macromolecule biosynthetic process|GO:0071704,organic substance metabolic process|GO:0010467,gene expression|GO:0006357,regulation of transcription from RNA polymerase II promoter|GO:0010468,regulation of gene expression|GO:0045935,positive regulation of nucleobase-containing compound metabolic process|GO:0045934,negative regulation of nucleobase-containing compound metabolic process|GO:0019219,regulation of nucleobase-containing compound metabolic process|GO:0044767,single-organism developmental process|GO:0009058,biosynthetic process|GO:0009059,macromolecule biosynthetic process|GO:0051171,regulation of nitrogen compound metabolic process|GO:0051172,negative regulation of nitrogen compound metabolic process|GO:0051173,positive regulation of nitrogen compound metabolic process|GO:0044238,primary metabolic process|GO:0042475,odontogenesis of dentin-containing tooth|GO:0042476,odontogenesis|GO:0048856,anatomical structure development|GO:0044237,cellular metabolic process|GO:0006259,DNA	GO:0044446,intracellular organelle part|GO:0043231,intracellular membrane-bounded organelle|GO:0031981,nuclear lumen|GO:0043233,organelle lumen|GO:0005634,nucleus|GO:0005730,nucleolus|GO:0044464,cell part|GO:0043232,intracellular non-membrane-bounded organelle|GO:0031974,membrane-enclosed lumen|GO:0005622,intracellular|GO:0005654,nucleoplasm|GO:0070013,intracellular organelle lumen|GO:0043229,intracellular organelle|GO:0043228,non-membrane-bounded organelle|GO:0044428,nuclear part|GO:0005623,cell|GO:0044424,intracellular part|GO:0043227,membrane-bounded organelle|GO:0043226,organelle|GO:0044422,organelle part|GO:0044452,nucleolar part|GO:0001650,fibrillar center	GO:0001012,RNA polymerase II regulatory region DNA binding|GO:0001159,core promoter proximal region DNA binding|GO:0001077,transcriptional activator activity, RNA polymerase II core promoter proximal region sequence-specific binding|GO:0005488,binding|GO:0003676,nucleic acid binding|GO:0003677,DNA binding|GO:1901363,heterocyclic compound binding|GO:0043565,sequence-specific DNA binding|GO:0043566,structure-specific DNA binding|GO:0000987,core promoter proximal region sequence-specific DNA binding|GO:0003690,double-stranded DNA binding|GO:0000982,transcription factor activity, RNA polymerase II core promoter proximal region sequence-specific binding|GO:0000981,RNA polymerase II transcription factor activity, sequence-specific DNA binding|GO:0001067,regulatory region nucleic acid binding|GO:0044212,transcription regulatory region DNA binding|GO:0003700,transcription factor activity, sequence-specific DNA binding|GO:0097159,organic cyclic compound binding|GO:0001228,transcriptional activator activity, RNA polymerase II transcription regulatory region sequence-specific binding|GO:0000976,transcription regulatory region sequence-specific DNA binding|GO:0000977,RNA polymerase II regulatory region sequence-specific DNA binding|GO:0000975,regulatory region DNA binding|GO:0000978,RNA polymerase II core promoter proximal region sequence-specific DNA binding|GO:0001071,nucleic acid binding transcription factor activity|GO:1990837,sequence-specific double-stranded DNA binding

MGRN1	GO:0044238,primary metabolic process|GO:0007041,lysosomal transport|GO:0048585,negative regulation of response to stimulus|GO:0019933,cAMP-mediated signaling|GO:0048583,regulation of response to stimulus|GO:0023057,negative regulation of signaling|GO:0008277,regulation of G-protein coupled receptor protein signaling pathway|GO:0023052,signaling|GO:0007165,signal transduction|GO:0019932,second-messenger-mediated signaling|GO:0023051,regulation of signaling|GO:0035556,intracellular signal transduction|GO:0010646,regulation of cell communication|GO:0050789,regulation of biological process|GO:0044699,single-organism process|GO:0044267,cellular protein metabolic process|GO:0051716,cellular response to stimulus|GO:0051234,establishment of localization|GO:0009968,negative regulation of signal transduction|GO:0016567,protein ubiquitination|GO:0009966,regulation of signal transduction|GO:0070647,protein modification by small protein conjugation or removal|GO:0000209,protein polyubiquitination|GO:0032446,protein modification by small protein conjugation|GO:0045744,negative regulation of G-protein coupled receptor protein signaling pathway|GO:0065007,biological regulation|GO:0006810,transport|GO:0048519,negative regulation of biological process|GO:0051641,cellular localization|GO:0007186,G-protein coupled receptor signaling pathway|GO:0008333,endosome to lysosome transport|GO:0043949,regulation of cAMP-mediated signaling|GO:0043951,negative regulation of cAMP-mediated signaling|GO:0009987,cellular process|GO:0006464,cellular protein modification process|GO:0050794,regulation of cellular process|GO:0071704,organic substance metabolic process|GO:0044765,single-organism transport|GO:0036211,protein modification process|GO:0044763,single-organism cellular process|GO:0008152,metabolic process|GO:0051649,establishment of localization in cell|GO:1902532,negative regulation of intracellular signal transduction|GO:0007154,cell communication|GO:1902531,regulation of intracellular signal transduction|GO:0051179,localization|GO:1902578,single-organism localization|GO:0016482,cytosolic transport|GO:0016197,endosomal transport|GO:0044700,single organism signaling|GO:0044260,cellular macromolecule metabolic process|GO:0007034,vacuolar transport|GO:0019935,cyclic-nucleotide-mediated signaling|GO:0019538,protein metabolic process|GO:0050896,response to stimulus|GO:0043412,macromolecule modification|GO:0044237,cellular metabolic process|GO:0043170,macromolecule metabolic process|GO:0006513,protein monoubiquitination|GO:0046907,intracellular transport|GO:1902582,single-organism intracellular transport|GO:0048523,negative regulation of cellular process|GO:0010648,negative regulation of cell communication|GO:0006807,nitrogen compound metabolic process|GO:0016192,vesicle-mediated transport|GO:1901564,organonitrogen compound metabolic process|GO:0008589,regulation of smoothened signaling pathway|GO:0045879,negative regulation of smoothened signaling pathway|GO:0007166,cell surface receptor signaling pathway|GO:0007224,smoothened signaling pathway	GO:0044421,extracellular region part|GO:0043229,intracellular organelle|GO:0071944,cell periphery|GO:0043227,membrane-bounded organelle|GO:0043226,organelle|GO:0005737,cytoplasm|GO:0031982,vesicle|GO:0005634,nucleus|GO:0016020,membrane|GO:0031988,membrane-bounded vesicle|GO:0012505,endomembrane system|GO:0005886,plasma membrane|GO:1903561,extracellular vesicle|GO:0070062,extracellular exosome|GO:0043230,extracellular organelle|GO:0043231,intracellular membrane-bounded organelle|GO:0065010,extracellular membrane-bounded organelle|GO:0005829,cytosol|GO:0044464,cell part|GO:0005623,cell|GO:0005622,intracellular|GO:0044444,cytoplasmic part|GO:0005576,extracellular region|GO:0044424,intracellular part|GO:0005768,endosome|GO:0005769,early endosome|GO:0031410,cytoplasmic vesicle|GO:0097708,intracellular vesicle	GO:0019787,ubiquitin-like protein transferase activity|GO:0043169,cation binding|GO:0046914,transition metal ion binding|GO:0016874,ligase activity|GO:0061630,ubiquitin protein ligase activity|GO:0043167,ion binding|GO:0003824,catalytic activity|GO:0016740,transferase activity|GO:0008270,zinc ion binding|GO:0005515,protein binding|GO:0005488,binding|GO:0046872,metal ion binding|GO:0004842,ubiquitin-protein transferase activity|GO:0061659,ubiquitin-like protein ligase activity
TRUB2	GO:0009451,RNA modification|GO:0090304,nucleic acid metabolic process|GO:0034641,cellular nitrogen compound metabolic process|GO:0006807,nitrogen compound metabolic process|GO:0008033,tRNA processing|GO:0034660,ncRNA metabolic process|GO:0001522,pseudouridine synthesis|GO:1901360,organic cyclic compound metabolic process|GO:0006139,nucleobase-containing compound metabolic process|GO:0044260,cellular macromolecule metabolic process|GO:0071704,organic substance metabolic process|GO:0010467,gene expression|GO:0034470,ncRNA processing|GO:0009987,cellular process|GO:0006725,cellular aromatic compound metabolic process|GO:0043412,macromolecule modification|GO:0008152,metabolic process|GO:0046483,heterocycle metabolic process|GO:0016070,RNA metabolic process|GO:0044238,primary metabolic process|GO:0044237,cellular metabolic process|GO:0043170,macromolecule metabolic process|GO:0006399,tRNA metabolic process|GO:0006396,RNA processing|GO:0034250,positive regulation of cellular amide metabolic process|GO:0070131,positive regulation of mitochondrial translation|GO:0009893,positive regulation of metabolic process|GO:0019222,regulation of metabolic process|GO:0031328,positive regulation of cellular biosynthetic process|GO:0051246,regulation of protein metabolic process|GO:0031325,positive regulation of cellular metabolic process|GO:0031323,regulation of cellular metabolic process|GO:0051128,regulation of cellular component organization|GO:0044249,cellular biosynthetic process|GO:0010628,positive regulation of gene expression|GO:0010821,regulation of mitochondrion organization|GO:0034645,cellular macromolecule biosynthetic process|GO:0010822,positive regulation of mitochondrion organization|GO:0043043,peptide biosynthetic process|GO:0050789,regulation of biological process|GO:0007005,mitochondrion organization|GO:0080090,regulation of primary metabolic process|GO:0044267,cellular protein metabolic process|GO:0010604,positive regulation of macromolecule metabolic process|GO:0010608,posttranscriptional regulation of gene expression|GO:0009891,positive regulation of biosynthetic process|GO:2000112,regulation of cellular macromolecule biosynthetic process|GO:0051247,positive regulation of protein metabolic process|GO:0016043,cellular component organization|GO:0033043,regulation of organelle organization|GO:0032270,positive regulation of cellular protein metabolic process|GO:0071840,cellular component organization or biogenesis|GO:0031326,regulation of cellular biosynthetic process|GO:0048518,positive regulation of biological process|GO:0065007,biological regulation|GO:0045727,positive regulation of translation|GO:0010468,regulation of gene expression|GO:0051130,positive regulation of cellular component organization|GO:0043604,amide biosynthetic process|GO:0060255,regulation of macromolecule metabolic process|GO:0009889,regulation of biosynthetic process|GO:0070129,regulation of mitochondrial translation|GO:0009058,biosynthetic process|GO:0009059,macromolecule biosynthetic process|GO:0051171,regulation of nitrogen compound metabolic process|GO:0051173,positive regulation of nitrogen compound metabolic process|GO:1901564,organonitrogen compound metabolic process|GO:0034248,regulation of cellular amide metabolic process|GO:0050794,regulation of cellular process|GO:0006996,organelle organization|GO:0016071,mRNA metabolic process|GO:0032268,regulation of cellular protein metabolic process|GO:0044271,cellular nitrogen compound biosynthetic process|GO:1901566,organonitrogen compound biosynthetic process|GO:0043603,cellular amide metabolic process|GO:0019538,protein metabolic process|GO:0010638,positive regulation of organelle organization|GO:0032543,mitochondrial translation|GO:0010557,positive regulation of macromolecule biosynthetic process|GO:0010556,regulation of macromolecule biosynthetic process|GO:1901576,organic substance biosynthetic process|GO:0006417,regulation of translation|GO:0006518,peptide metabolic process|GO:0006397,mRNA	GO:0005737,cytoplasm|GO:0044464,cell part|GO:0005623,cell|GO:0005622,intracellular|GO:0044424,intracellular part|GO:0005829,cytosol|GO:0044444,cytoplasmic part|GO:0043231,intracellular membrane-bounded organelle|GO:0043233,organelle lumen|GO:0031974,membrane-enclosed lumen|GO:0044446,intracellular organelle part|GO:0070013,intracellular organelle lumen|GO:0043229,intracellular organelle|GO:0005739,mitochondrion|GO:0044429,mitochondrial part|GO:0005759,mitochondrial matrix|GO:0043227,membrane-bounded organelle|GO:0043226,organelle|GO:0044422,organelle part	GO:0016853,isomerase activity|GO:0097159,organic cyclic compound binding|GO:0044822,poly(A) RNA binding|GO:0009982,pseudouridine synthase activity|GO:0003824,catalytic activity|GO:0003723,RNA binding|GO:0003676,nucleic acid binding|GO:0016866,intramolecular transferase activity|GO:1901363,heterocyclic compound binding|GO:0005488,binding
FAM160B2			
SCAI	GO:0080090,regulation of primary metabolic process|GO:0019222,regulation of metabolic process|GO:0048585,negative regulation of response to stimulus|GO:0048583,regulation of response to stimulus|GO:0007266,Rho protein signal transduction|GO:0007165,signal transduction|GO:1901362,organic cyclic compound biosynthetic process|GO:1901360,organic cyclic compound metabolic process|GO:0051716,cellular response to stimulus|GO:0010605,negative regulation of macromolecule metabolic process|GO:0009968,negative regulation of signal transduction|GO:0009966,regulation of signal transduction|GO:0048519,negative regulation of biological process|GO:0051056,regulation of small GTPase mediated signal transduction|GO:0060255,regulation of macromolecule metabolic process|GO:0051058,negative regulation of small GTPase mediated signal transduction|GO:2001141,regulation of RNA biosynthetic process|GO:0046483,heterocycle metabolic process|GO:0044700,single organism signaling|GO:0044249,cellular biosynthetic process|GO:0048870,cell motility|GO:0019438,aromatic compound biosynthetic process|GO:0009892,negative regulation of metabolic process|GO:0009890,negative regulation of biosynthetic process|GO:0023051,regulation of signaling|GO:0006928,movement of cell or subcellular component|GO:0006807,nitrogen compound metabolic process|GO:0035556,intracellular signal transduction|GO:0050789,regulation of biological process|GO:0097659,nucleic acid-templated transcription|GO:1901576,organic substance biosynthetic process|GO:0044260,cellular macromolecule metabolic process|GO:0065007,biological regulation|GO:0016477,cell migration|GO:0018130,heterocycle biosynthetic process|GO:0009889,regulation of biosynthetic process|GO:0050794,regulation of cellular process|GO:0008152,metabolic process|GO:1902532,negative regulation of intracellular signal transduction|GO:1902531,regulation of intracellular signal transduction|GO:0016070,RNA metabolic process|GO:0044271,cellular nitrogen compound biosynthetic process|GO:0050896,response to stimulus|GO:0006355,regulation of transcription, DNA-templated|GO:0010556,regulation of macromolecule biosynthetic process|GO:2000145,regulation of cell motility|GO:0006351,transcription, DNA-templated|GO:2000146,negative regulation of cell motility|GO:0010558,negative regulation of macromolecule biosynthetic process|GO:0051171,regulation of nitrogen compound metabolic process|GO:0032774,RNA biosynthetic process|GO:0046578,regulation of Ras protein signal transduction|GO:0035024,negative regulation of Rho protein signal transduction|GO:0023057,negative regulation of signaling|GO:0034641,cellular nitrogen compound metabolic process|GO:0023052,signaling|GO:0010648,negative regulation of cell communication|GO:0034645,cellular macromolecule biosynthetic process|GO:0035023,regulation of Rho protein signal transduction|GO:0010646,regulation of cell communication|GO:0044699,single-organism process|GO:0006139,nucleobase-containing compound metabolic process|GO:0009987,cellular process|GO:0006725,cellular aromatic compound metabolic process|GO:0040013,negative regulation of locomotion|GO:1903506,regulation of nucleic acid-templated transcription|GO:1903507,negative regulation of nucleic acid-templated transcription|GO:0051270,regulation of cellular component movement|GO:0032879,regulation of localization|GO:0051253,negative regulation of RNA metabolic process|GO:0051252,regulation of RNA metabolic process|GO:0043170,macromolecule metabolic process|GO:0051674,localization of cell|GO:0031327,negative regulation of cellular biosynthetic process|GO:0031326,regulation of cellular biosynthetic process|GO:0031324,negative regulation of cellular metabolic process|GO:0031323,regulation of cellular metabolic process|GO:0090304,nucleic acid metabolic process|GO:2000112,regulation of cellular macromolecule biosynthetic process|GO:0071704,organic substance metabolic process|GO:0010467,gene expression|GO:0010468,regulation of gene expression|GO:0030336,negative regulation of cell migration|GO:0030334,regulation of cell migration|GO:0045934,negative regulation of nucleobase-containing compound metabolic process|GO:0019219,regulation of nucleobase-containing compound metabolic process|GO:1902679,negative regulation of RNA biosynthetic process|GO:0009058,biosynthetic process|GO:0009059,macromolecule biosynthetic process|GO:0044763,single-organism cellular process|GO:0046580,negative regulation of Ras protein signal transduction|GO:0051172,negative regulation of nitrogen compound metabolic process|GO:0007154,cell communication|GO:0007265,Ras protein signal transduction|GO:0007264,small GTPase mediated signal transduction|GO:0051179,localization|GO:0040011,locomotion|GO:0044238,primary metabolic process|GO:0051271,negative regulation of cellular component movement|GO:0040012,regulation of locomotion|GO:0044237,cellular metabolic	GO:0016020,membrane|GO:0031974,membrane-enclosed lumen|GO:0031975,envelope|GO:0043229,intracellular organelle|GO:0043227,membrane-bounded organelle|GO:0043226,organelle|GO:0031224,intrinsic component of membrane|GO:0005737,cytoplasm|GO:0031090,organelle membrane|GO:0031981,nuclear lumen|GO:0016021,integral component of membrane|GO:0005635,nuclear envelope|GO:0005654,nucleoplasm|GO:0031965,nuclear membrane|GO:0031967,organelle envelope|GO:0012505,endomembrane system|GO:0043231,intracellular membrane-bounded organelle|GO:0043233,organelle lumen|GO:0044464,cell part|GO:0005623,cell|GO:0005622,intracellular|GO:0044446,intracellular organelle part|GO:0070013,intracellular organelle lumen|GO:0044428,nuclear part|GO:0044424,intracellular part|GO:0044425,membrane part|GO:0005634,nucleus|GO:0044422,organelle part	GO:0005515,protein binding|GO:0003714,transcription corepressor activity|GO:0003712,transcription cofactor activity|GO:0005488,binding|GO:0000989,transcription factor activity, transcription factor binding|GO:0000988,transcription factor activity, protein binding
DBNL	GO:0032502,developmental process|GO:0044707,single-multicellular organism process|GO:0050808,synapse organization|GO:0030030,cell projection organization|GO:0030031,cell projection assembly|GO:0030154,cell differentiation|GO:0048468,cell development|GO:0022607,cellular component assembly|GO:0031175,neuron projection development|GO:0009653,anatomical structure morphogenesis|GO:0007275,multicellular organism development|GO:0007416,synapse assembly|GO:0000902,cell morphogenesis|GO:0048869,cellular developmental process|GO:0071840,cellular component organization or biogenesis|GO:0048858,cell projection morphogenesis|GO:0016043,cellular component organization|GO:0032989,cellular component morphogenesis|GO:0044085,cellular component biogenesis|GO:0044699,single-organism process|GO:0006810,transport|GO:0048666,neuron development|GO:0032501,multicellular organismal process|GO:0030182,neuron differentiation|GO:0009987,cellular process|GO:0044767,single-organism developmental process|GO:0044763,single-organism cellular process|GO:0022008,neurogenesis|GO:0051234,establishment of localization|GO:0051179,localization|GO:0032990,cell part morphogenesis|GO:0031529,ruffle organization|GO:0048699,generation of neurons|GO:0006897,endocytosis|GO:0097178,ruffle assembly|GO:0007399,nervous system development|GO:0006898,receptor-mediated endocytosis|GO:0048856,anatomical structure development|GO:0048812,neuron projection morphogenesis|GO:0048731,system development|GO:0016192,vesicle-mediated transport		GO:0008092,cytoskeletal protein binding|GO:0051015,actin filament binding|GO:0003779,actin binding|GO:0044877,macromolecular complex binding|GO:0005488,binding|GO:0005515,protein binding|GO:0032403,protein complex binding
ZNF479	GO:0080090,regulation of primary metabolic process|GO:0019222,regulation of metabolic process|GO:0031326,regulation of cellular biosynthetic process|GO:0031323,regulation of cellular metabolic process|GO:0090304,nucleic acid metabolic process|GO:0044249,cellular biosynthetic process|GO:0034641,cellular nitrogen compound metabolic process|GO:0006807,nitrogen compound metabolic process|GO:0034645,cellular macromolecule biosynthetic process|GO:1901362,organic cyclic compound biosynthetic process|GO:0050789,regulation of biological process|GO:0097659,nucleic acid-templated transcription|GO:0032774,RNA biosynthetic process|GO:0006139,nucleobase-containing compound metabolic process|GO:0044260,cellular macromolecule metabolic process|GO:0071704,organic substance metabolic process|GO:2000112,regulation of cellular macromolecule biosynthetic process|GO:0060255,regulation of macromolecule metabolic process|GO:0010467,gene expression|GO:0065007,biological regulation|GO:1901360,organic cyclic compound metabolic process|GO:0010468,regulation of gene expression|GO:0018130,heterocycle biosynthetic process|GO:1901576,organic substance biosynthetic process|GO:0019219,regulation of nucleobase-containing compound metabolic process|GO:0006725,cellular aromatic compound metabolic process|GO:0009987,cellular process|GO:0009889,regulation of biosynthetic process|GO:1903506,regulation of nucleic acid-templated transcription|GO:0050794,regulation of cellular process|GO:0009058,biosynthetic process|GO:0009059,macromolecule biosynthetic process|GO:0051171,regulation of nitrogen compound metabolic process|GO:0008152,metabolic process|GO:2001141,regulation of RNA biosynthetic process|GO:0034654,nucleobase-containing compound biosynthetic process|GO:0046483,heterocycle metabolic process|GO:0016070,RNA metabolic process|GO:0044238,primary metabolic process|GO:0044271,cellular nitrogen compound biosynthetic process|GO:0051252,regulation of RNA metabolic process|GO:0044237,cellular metabolic process|GO:0043170,macromolecule metabolic process|GO:0006355,regulation of transcription, DNA-templated|GO:0010556,regulation of macromolecule biosynthetic process|GO:0006351,transcription, DNA-templated|GO:0019438,aromatic compound biosynthetic process	GO:0043231,intracellular membrane-bounded organelle|GO:0005634,nucleus|GO:0044464,cell part|GO:0005623,cell|GO:0005622,intracellular|GO:0043229,intracellular organelle|GO:0044424,intracellular part|GO:0043227,membrane-bounded organelle|GO:0043226,organelle	GO:0043169,cation binding|GO:0097159,organic cyclic compound binding|GO:0043167,ion binding|GO:0005488,binding|GO:0003676,nucleic acid binding|GO:0003677,DNA binding|GO:0046872,metal ion binding|GO:1901363,heterocyclic compound binding|GO:0003700,transcription factor activity, sequence-specific DNA binding|GO:0001071,nucleic acid binding transcription factor activity|GO:0000981,RNA polymerase II transcription factor activity, sequence-specific DNA binding
FAM83G	GO:0042221,response to chemical|GO:0080090,regulation of primary metabolic process|GO:0019222,regulation of metabolic process|GO:0031326,regulation of cellular biosynthetic process|GO:0031323,regulation of cellular metabolic process|GO:0070848,response to growth factor|GO:0090304,nucleic acid metabolic process|GO:0030509,BMP signaling pathway|GO:0034641,cellular nitrogen compound metabolic process|GO:0006807,nitrogen compound metabolic process|GO:0023052,signaling|GO:0007165,signal transduction|GO:0007166,cell surface receptor signaling pathway|GO:0007167,enzyme linked receptor protein signaling pathway|GO:1901576,organic substance biosynthetic process|GO:0070887,cellular response to chemical stimulus|GO:1901362,organic cyclic compound biosynthetic process|GO:0050789,regulation of biological process|GO:0044699,single-organism process|GO:0032774,RNA biosynthetic process|GO:0009719,response to endogenous stimulus|GO:0006139,nucleobase-containing compound metabolic process|GO:0051716,cellular response to stimulus|GO:0044271,cellular nitrogen compound biosynthetic process|GO:0044260,cellular macromolecule metabolic process|GO:0071704,organic substance metabolic process|GO:1901360,organic cyclic compound metabolic process|GO:2000112,regulation of cellular macromolecule biosynthetic process|GO:0010467,gene expression|GO:0060255,regulation of macromolecule metabolic process|GO:0071310,cellular response to organic substance|GO:0010556,regulation of macromolecule biosynthetic process|GO:0065007,biological regulation|GO:0097659,nucleic acid-templated transcription|GO:0006366,transcription from RNA polymerase II promoter|GO:0046483,heterocycle metabolic process|GO:0010468,regulation of gene expression|GO:0018130,heterocycle biosynthetic process|GO:0071495,cellular response to endogenous stimulus|GO:0044238,primary metabolic process|GO:0019219,regulation of nucleobase-containing compound metabolic process|GO:0006725,cellular aromatic compound metabolic process|GO:0009987,cellular process|GO:0009889,regulation of biosynthetic process|GO:1903506,regulation of nucleic acid-templated transcription|GO:0050794,regulation of cellular process|GO:0034645,cellular macromolecule biosynthetic process|GO:0009059,macromolecule biosynthetic process|GO:0044763,single-organism cellular process|GO:0051171,regulation of nitrogen compound metabolic process|GO:0008152,metabolic process|GO:2001141,regulation of RNA biosynthetic process|GO:0034654,nucleobase-containing compound biosynthetic process|GO:0007154,cell communication|GO:0010033,response to organic substance|GO:0007178,transmembrane receptor protein serine/threonine kinase signaling pathway|GO:0016070,RNA metabolic process|GO:0044700,single organism signaling|GO:0071363,cellular response to growth factor stimulus|GO:0044249,cellular biosynthetic process|GO:0050896,response to stimulus|GO:0051252,regulation of RNA metabolic process|GO:0009058,biosynthetic process|GO:0044237,cellular metabolic process|GO:0043170,macromolecule metabolic process|GO:0006355,regulation of transcription, DNA-templated|GO:0006357,regulation of transcription from RNA polymerase II promoter|GO:0006351,transcription, DNA-templated|GO:0019438,aromatic compound biosynthetic process|GO:0071772,response to BMP|GO:0071773,cellular response to BMP stimulus	GO:0005737,cytoplasm|GO:0043231,intracellular membrane-bounded organelle|GO:0005829,cytosol|GO:0044464,cell part|GO:0044444,cytoplasmic part|GO:0005623,cell|GO:0043226,organelle|GO:0043229,intracellular organelle|GO:0044424,intracellular part|GO:0005622,intracellular|GO:0043227,membrane-bounded organelle|GO:0005634,nucleus	GO:0005488,binding|GO:0005515,protein binding
BTD	GO:0019752,carboxylic acid metabolic process|GO:0034641,cellular nitrogen compound metabolic process|GO:0032501,multicellular organismal process|GO:0044281,small molecule metabolic process|GO:0044707,single-multicellular organism process|GO:0044699,single-organism process|GO:0007417,central nervous system development|GO:0044710,single-organism metabolic process|GO:0051186,cofactor metabolic process|GO:1901360,organic cyclic compound metabolic process|GO:0007275,multicellular organism development|GO:0071704,organic substance metabolic process|GO:0006082,organic acid metabolic process|GO:0032502,developmental process|GO:0006767,water-soluble vitamin metabolic process|GO:0006766,vitamin metabolic process|GO:0009987,cellular process|GO:0006768,biotin metabolic process|GO:0032787,monocarboxylic acid metabolic process|GO:0044767,single-organism developmental process|GO:0044763,single-organism cellular process|GO:0008152,metabolic process|GO:0043436,oxoacid metabolic process|GO:0046483,heterocycle metabolic process|GO:1901564,organonitrogen compound metabolic process|GO:0007399,nervous system development|GO:0043603,cellular amide metabolic process|GO:0048856,anatomical structure development|GO:0044237,cellular metabolic process|GO:0006807,nitrogen compound metabolic process|GO:0006790,sulfur compound metabolic process|GO:0048731,system development	GO:0031974,membrane-enclosed lumen|GO:0043229,intracellular organelle|GO:0043227,membrane-bounded organelle|GO:0043226,organelle|GO:0005737,cytoplasm|GO:0070062,extracellular exosome|GO:0005615,extracellular space|GO:0005739,mitochondrion|GO:0031988,membrane-bounded vesicle|GO:0005759,mitochondrial matrix|GO:1903561,extracellular vesicle|GO:0031982,vesicle|GO:0043230,extracellular organelle|GO:0043231,intracellular membrane-bounded organelle|GO:0043233,organelle lumen|GO:0044464,cell part|GO:0005623,cell|GO:0005622,intracellular|GO:0044446,intracellular organelle part|GO:0070013,intracellular organelle lumen|GO:0044444,cytoplasmic part|GO:0005576,extracellular region|GO:0044429,mitochondrial part|GO:0044424,intracellular part|GO:0065010,extracellular membrane-bounded organelle|GO:0044421,extracellular region part|GO:0044422,organelle part	GO:0016787,hydrolase activity|GO:0047708,biotinidase activity|GO:0016810,hydrolase activity, acting on carbon-nitrogen (but not peptide) bonds|GO:0016811,hydrolase activity, acting on carbon-nitrogen (but not peptide) bonds, in linear amides|GO:0003824,catalytic activity
OTOR	GO:0032502,developmental process|GO:0001501,skeletal system development|GO:0001502,cartilage condensation|GO:0009653,anatomical structure morphogenesis|GO:0007275,multicellular organism development|GO:0044699,single-organism process|GO:0009888,tissue development|GO:0048513,animal organ development|GO:0050877,neurological system process|GO:0098743,cell aggregation|GO:0048705,skeletal system morphogenesis|GO:0009887,animal organ morphogenesis|GO:0032501,multicellular organismal process|GO:0007605,sensory perception of sound|GO:0050954,sensory perception of mechanical stimulus|GO:0007600,sensory perception|GO:0061448,connective tissue development|GO:0044767,single-organism developmental process|GO:0003008,system process|GO:0051216,cartilage development|GO:0044707,single-multicellular organism process|GO:0048856,anatomical structure development|GO:0048731,system development	GO:0005576,extracellular region	
CCND3	GO:0009987,cellular process|GO:0044763,single-organism cellular process|GO:0044699,single-organism process|GO:0007049,cell cycle	GO:0043231,intracellular membrane-bounded organelle|GO:0005634,nucleus|GO:0044464,cell part|GO:0005623,cell|GO:0005622,intracellular|GO:0043229,intracellular organelle|GO:0044424,intracellular part|GO:0043227,membrane-bounded organelle|GO:0043226,organelle|GO:0071944,cell periphery|GO:0005924,cell-substrate adherens junction|GO:0005925,focal adhesion|GO:0030054,cell junction|GO:0030055,cell-substrate junction|GO:0005737,cytoplasm|GO:0016020,membrane|GO:0070161,anchoring junction|GO:0005912,adherens junction|GO:0005886,plasma membrane|GO:0005829,cytosol|GO:0044444,cytoplasmic part	
PPARA	GO:0046434,organophosphate catabolic process|GO:0051049,regulation of transport|GO:0009166,nucleotide catabolic process|GO:0009167,purine ribonucleoside monophosphate metabolic process|GO:0044281,small molecule metabolic process|GO:0044282,small molecule catabolic process|GO:0009161,ribonucleoside monophosphate metabolic process|GO:0051716,cellular response to stimulus|GO:0001666,response to hypoxia|GO:0030258,lipid modification|GO:0009199,ribonucleoside triphosphate metabolic process|GO:0060548,negative regulation of cell death|GO:0046483,heterocycle metabolic process|GO:0051196,regulation of coenzyme metabolic process|GO:0042326,negative regulation of phosphorylation|GO:0051193,regulation of cofactor metabolic process|GO:0009605,response to external stimulus|GO:0019538,protein metabolic process|GO:0051198,negative regulation of coenzyme metabolic process|GO:0010887,negative regulation of cholesterol storage|GO:0010885,regulation of cholesterol storage|GO:0010883,regulation of lipid storage|GO:0045820,negative regulation of glycolytic process|GO:0010889,regulation of sequestering of triglyceride|GO:0010888,negative regulation of lipid storage|GO:0009896,positive regulation of catabolic process|GO:0009894,regulation of catabolic process|GO:0009895,negative regulation of catabolic process|GO:0009892,negative regulation of metabolic process|GO:0009893,positive regulation of metabolic process|GO:0009890,negative regulation of biosynthetic process|GO:0009891,positive regulation of biosynthetic process|GO:0010906,regulation of glucose metabolic process|GO:0010907,positive regulation of glucose metabolic process|GO:0050789,regulation of biological process|GO:0032091,negative regulation of protein binding|GO:0032094,response to food|GO:0032095,regulation of response to food|GO:0032096,negative regulation of response to food|GO:0032098,regulation of appetite|GO:0032099,negative regulation of appetite|GO:0006110,regulation of glycolytic process|GO:0006111,regulation of gluconeogenesis|GO:0045913,positive regulation of carbohydrate metabolic process|GO:0045912,negative regulation of carbohydrate metabolic process|GO:0018130,heterocycle biosynthetic process|GO:0007623,circadian rhythm|GO:0006629,lipid metabolic process|GO:0009889,regulation of biosynthetic process|GO:0070166,enamel mineralization|GO:0009888,tissue development|GO:0044723,single-organism carbohydrate metabolic process|GO:0019395,fatty acid oxidation|GO:0044724,single-organism carbohydrate catabolic process|GO:0016070,RNA metabolic process|GO:0035095,behavioral response to nicotine|GO:0035094,response to nicotine|GO:0010557,positive regulation of macromolecule biosynthetic process|GO:0010556,regulation of macromolecule biosynthetic process|GO:1901292,nucleoside phosphate catabolic process|GO:0006753,nucleoside phosphate metabolic process|GO:0010558,negative regulation of macromolecule biosynthetic process|GO:0006757,ATP generation from ADP|GO:0034505,tooth mineralization|GO:0051235,maintenance of location|GO:0014070,response to organic cyclic compound|GO:0051234,establishment of localization|GO:0000122,negative regulation of transcription from RNA polymerase II promoter|GO:0030812,negative regulation of nucleotide catabolic process|GO:0030811,regulation of nucleotide catabolic process|GO:0072369,regulation of lipid transport by positive regulation of transcription from RNA polymerase II promoter|GO:0010675,regulation of cellular carbohydrate metabolic process|GO:0010676,positive regulation of cellular carbohydrate metabolic process|GO:0010677,negative regulation of cellular carbohydrate metabolic process|GO:0044255,cellular lipid metabolic process|GO:0009132,nucleoside diphosphate metabolic process|GO:0009135,purine nucleoside diphosphate metabolic process|GO:0045923,positive regulation of fatty acid metabolic process|GO:0008217,regulation of blood pressure|GO:0006091,generation of precursor metabolites and energy|GO:0006090,pyruvate metabolic process|GO:0008219,cell death|GO:0006094,gluconeogenesis|GO:0033993,response to lipid|GO:2000112,regulation of cellular macromolecule biosynthetic process|GO:2000113,negative regulation of cellular macromolecule biosynthetic process|GO:0006096,glycolytic process|GO:0015849,organic acid transport|GO:0019217,regulation of fatty acid metabolic process|GO:0019216,regulation of lipid metabolic process|GO:0019219,regulation of nucleobase-containing compound metabolic process|GO:0044767,single-organism developmental process|GO:0044765,single-organism transport|GO:0044763,single-organism cellular process|GO:1901700,response to oxygen-containing compound|GO:0030534,adult behavior|GO:0048856,anatomical structure development|GO:0050994,regulation of lipid catabolic process|GO:0019693,ribose phosphate metabolic process|GO:0050996,positive regulation of lipid catabolic process|GO:0006796,phosphate-containing compound metabolic process|GO:0006793,phosphorus metabolic process|GO:0006140,regulation of nucleotide metabolic process|GO:0043112,receptor metabolic process|GO:0048523,negative regulation of cellular process|GO:0019362,pyridine nucleotide metabolic process|GO:0034440,lipid oxidation|GO:0032104,regulation of response to extracellular stimulus|GO:0007610,behavior|GO:1903579,negative regulation of ATP metabolic process|GO:1903578,regulation of ATP metabolic process|GO:0003013,circulatory system process|GO:0007165,signal transduction|GO:0019915,lipid storage|GO:0044712,single-organism catabolic process|GO:0044710,single-organism metabolic process|GO:0044711,single-organism biosynthetic process|GO:0044092,negative regulation of molecular function|GO:0033036,macromolecule localization|GO:0042325,regulation of phosphorylation|GO:0046034,ATP metabolic process|GO:0046031,ADP metabolic process|GO:0051195,negative regulation of cofactor metabolic process|GO:2001141,regulation of RNA biosynthetic process|GO:0009179,purine ribonucleoside diphosphate metabolic process|GO:0010033,response to organic substance|GO:0090077,foam cell differentiation|GO:0010243,response to organonitrogen compound|GO:0043401,steroid hormone mediated signaling pathway|GO:0046321,positive regulation of fatty acid oxidation|GO:0046320,regulation of fatty acid oxidation|GO:0010629,negative regulation of gene expression|GO:0010628,positive regulation of gene expression|GO:0046496,nicotinamide nucleotide metabolic process|GO:0051186,cofactor metabolic process|GO:0044262,cellular carbohydrate metabolic process|GO:0044260,cellular macromolecule metabolic process|GO:0070997,neuron death|GO:0006367,transcription initiation from RNA polymerase II promoter|GO:0006366,transcription from RNA polymerase II promoter|GO:0009887,animal organ morphogenesis|GO:0050793,regulation of developmental process|GO:0019318,hexose metabolic process|GO:0019319,hexose biosynthetic process|GO:0050794,regulation of cellular process|GO:1901214,regulation of neuron death|GO:1901215,negative regulation of neuron death|GO:1902679,negative regulation of RNA biosynthetic process|GO:0032368,regulation of lipid transport|GO:0046395,carboxylic acid catabolic process|GO:0050896,response to stimulus|GO:0006109,regulation of carbohydrate metabolic process|GO:0051171,regulation of nitrogen compound metabolic process|GO:0032107,regulation of response to nutrient levels|GO:0006631,fatty acid metabolic process|GO:0032105,negative regulation of response to extracellular stimulus|GO:0032102,negative regulation of response to external stimulus|GO:0006635,fatty acid beta-oxidation|GO:0032101,regulation of response to external stimulus|GO:0006732,coenzyme metabolic process|GO:0006733,oxidoreduction coenzyme metabolic process|GO:0032108,negative regulation of response to nutrient levels|GO:0070887,cellular response to chemical stimulus|GO:0009259,ribonucleotide metabolic process|GO:0044699,single-organism process|GO:0010563,negative regulation of phosphorus metabolic process|GO:0010565,regulation of cellular ketone metabolic process|GO:0051101,regulation of DNA binding|GO:0071396,cellular response to lipid|GO:0043279,response to alkaloid|GO:0010869,regulation of receptor biosynthetic process|GO:0043470,regulation of carbohydrate catabolic process|GO:0043471,regulation of cellular carbohydrate catabolic process|GO:1901135,carbohydrate derivative metabolic process|GO:1901362,organic cyclic compound biosynthetic process|GO:1902680,positive regulation of RNA biosynthetic process|GO:0048731,system development|GO:0046364,monosaccharide biosynthetic process|GO:0072521,purine-containing compound metabolic process|GO:0072524,pyridine-containing compound metabolic process|GO:0045935,positive regulation of nucleobase-containing compound metabolic process|GO:0097186,amelogenesis|GO:0045936,negative regulation of phosphate	GO:0044446,intracellular organelle part|GO:0043231,intracellular membrane-bounded organelle|GO:0031981,nuclear lumen|GO:0043233,organelle lumen|GO:0005634,nucleus|GO:0044464,cell part|GO:0031974,membrane-enclosed lumen|GO:0005622,intracellular|GO:0005654,nucleoplasm|GO:0070013,intracellular organelle lumen|GO:0043229,intracellular organelle|GO:0044428,nuclear part|GO:0005623,cell|GO:0044424,intracellular part|GO:0043227,membrane-bounded organelle|GO:0043226,organelle|GO:0044422,organelle part	GO:0005488,binding|GO:0038023,signaling receptor activity|GO:0004871,signal transducer activity|GO:0001012,RNA polymerase II regulatory region DNA binding|GO:0001159,core promoter proximal region DNA binding|GO:0008144,drug binding|GO:0060089,molecular transducer activity|GO:0001076,transcription factor activity, RNA polymerase II transcription factor binding|GO:0001077,transcriptional activator activity, RNA polymerase II core promoter proximal region sequence-specific binding|GO:0044877,macromolecular complex binding|GO:0008270,zinc ion binding|GO:0003676,nucleic acid binding|GO:0003677,DNA binding|GO:0001078,transcriptional repressor activity, RNA polymerase II core promoter proximal region sequence-specific binding|GO:1901363,heterocyclic compound binding|GO:0019904,protein domain specific binding|GO:0070491,repressing transcription factor binding|GO:0043565,sequence-specific DNA binding|GO:0001190,transcriptional activator activity, RNA polymerase II transcription factor binding|GO:0046914,transition metal ion binding|GO:0097371,MDM2/MDM4 family protein binding|GO:0001227,transcriptional repressor activity, RNA polymerase II transcription regulatory region sequence-specific binding|GO:0043167,ion binding|GO:0004879,RNA polymerase II transcription factor activity, ligand-activated sequence-specific DNA binding|GO:0000989,transcription factor activity, transcription factor binding|GO:0000988,transcription factor activity, protein binding|GO:0000987,core promoter proximal region sequence-specific DNA binding|GO:0046872,metal ion binding|GO:0000982,transcription factor activity, RNA polymerase II core promoter proximal region sequence-specific binding|GO:0000981,RNA polymerase II transcription factor activity, sequence-specific DNA binding|GO:0043169,cation binding|GO:0008134,transcription factor binding|GO:0001067,regulatory region nucleic acid binding|GO:0008289,lipid binding|GO:0044390,ubiquitin-like protein conjugating enzyme binding|GO:0032403,protein complex binding|GO:0019902,phosphatase binding|GO:0019899,enzyme binding|GO:0001103,RNA polymerase II repressing transcription factor binding|GO:0001085,RNA polymerase II transcription factor binding|GO:0044212,transcription regulatory region DNA binding|GO:0031624,ubiquitin conjugating enzyme binding|GO:0051525,NFAT protein binding|GO:0003700,transcription factor activity, sequence-specific DNA binding|GO:0097159,organic cyclic compound binding|GO:0001071,nucleic acid binding transcription factor activity|GO:0098531,transcription factor activity, direct ligand regulated sequence-specific DNA binding|GO:0003707,steroid hormone receptor activity|GO:0000976,transcription regulatory region sequence-specific DNA binding|GO:0000977,RNA polymerase II regulatory region sequence-specific DNA binding|GO:0005515,protein binding|GO:0000975,regulatory region DNA binding|GO:0000978,RNA polymerase II core promoter proximal region sequence-specific DNA binding|GO:0001228,transcriptional activator activity [illegible]
AOC3	GO:0006954,inflammatory response|GO:0006807,nitrogen compound metabolic process|GO:0046677,response to antibiotic|GO:0044699,single-organism process|GO:0044710,single-organism metabolic process|GO:0009636,response to toxic substance|GO:0055085,transmembrane transport|GO:0071704,organic substance metabolic process|GO:0055114,oxidation-reduction process|GO:0022610,biological adhesion|GO:0006810,transport|GO:0009308,amine metabolic process|GO:0006812,cation transport|GO:0006811,ion transport|GO:0009987,cellular process|GO:0006952,defense response|GO:0034220,ion transmembrane transport|GO:0044765,single-organism transport|GO:0044763,single-organism cellular process|GO:0008152,metabolic process|GO:0007155,cell adhesion|GO:0042221,response to chemical|GO:0051234,establishment of localization|GO:0051179,localization|GO:1902578,single-organism localization|GO:1901564,organonitrogen compound metabolic process|GO:0050896,response to stimulus|GO:0006950,response to stress|GO:0098655,cation transmembrane transport|GO:1902283,negative regulation of primary amine oxidase activity|GO:0009892,negative regulation of metabolic process|GO:0019222,regulation of metabolic process|GO:0006805,xenobiotic metabolic process|GO:0009410,response to xenobiotic stimulus|GO:0070887,cellular response to chemical stimulus|GO:0043086,negative regulation of catalytic activity|GO:0051341,regulation of oxidoreductase activity|GO:0050789,regulation of biological process|GO:0065007,biological regulation|GO:0044092,negative regulation of molecular function|GO:0048519,negative regulation of biological process|GO:0065009,regulation of molecular function|GO:0050790,regulation of catalytic activity|GO:0051716,cellular response to stimulus|GO:0051354,negative regulation of oxidoreductase activity|GO:0044237,cellular metabolic process|GO:0071466,cellular response to xenobiotic stimulus	GO:0005783,endoplasmic reticulum|GO:0005902,microvillus|GO:0071944,cell periphery|GO:0043227,membrane-bounded organelle|GO:0043226,organelle|GO:0031224,intrinsic component of membrane|GO:0005737,cytoplasm|GO:0016021,integral component of membrane|GO:0016020,membrane|GO:0005794,Golgi apparatus|GO:0009986,cell surface|GO:0012505,endomembrane system|GO:0005886,plasma membrane|GO:0042995,cell projection|GO:0043231,intracellular membrane-bounded organelle|GO:0044464,cell part|GO:0043229,intracellular organelle|GO:0005623,cell|GO:0005622,intracellular|GO:0044444,cytoplasmic part|GO:0044424,intracellular part|GO:0044425,membrane part|GO:0005768,endosome|GO:0005769,early endosome|GO:0031982,vesicle|GO:0031410,cytoplasmic vesicle|GO:0097708,intracellular vesicle|GO:0098858,actin-based cell projection	GO:0005261,cation channel activity|GO:0046872,metal ion binding|GO:0005488,binding|GO:0016491,oxidoreductase activity|GO:0052593,tryptamine:oxygen oxidoreductase (deaminating) activity|GO:0043169,cation binding|GO:0052596,phenethylamine:oxygen oxidoreductase (deaminating) activity|GO:0052595,aliphatic-amine oxidase activity|GO:0052594,aminoacetone:oxygen oxidoreductase(deaminating) activity|GO:0016641,oxidoreductase activity, acting on the CH-NH2 group of donors, oxygen as acceptor|GO:0043167,ion binding|GO:0005509,calcium ion binding|GO:0022803,passive transmembrane transporter activity|GO:0005507,copper ion binding|GO:0042802,identical protein binding|GO:0042803,protein homodimerization activity|GO:0046982,protein heterodimerization activity|GO:0005215,transporter activity|GO:0005216,ion channel activity|GO:0008324,cation transmembrane transporter activity|GO:0008131,primary amine oxidase activity|GO:0003824,catalytic activity|GO:0048037,cofactor binding|GO:0048038,quinone binding|GO:0022891,substrate-specific transmembrane transporter activity|GO:0022892,substrate-specific transporter activity|GO:0046914,transition metal ion binding|GO:0016638,oxidoreductase activity, acting on the CH-NH2 group of donors|GO:0015075,ion transmembrane transporter activity|GO:0022857,transmembrane transporter activity|GO:0015267,channel activity|GO:0005515,protein binding|GO:0046983,protein dimerization activity|GO:0022838,substrate-specific
FBXO27	GO:0071704,organic substance metabolic process|GO:0044267,cellular protein metabolic process|GO:0044238,primary metabolic process|GO:1901564,organonitrogen compound metabolic process|GO:0044260,cellular macromolecule metabolic process|GO:0032446,protein modification by small protein conjugation|GO:0019538,protein metabolic process|GO:0043687,post-translational protein modification|GO:0009987,cellular process|GO:0070647,protein modification by small protein conjugation or removal|GO:0006464,cellular protein modification process|GO:0043170,macromolecule metabolic process|GO:0016567,protein ubiquitination|GO:0043412,macromolecule modification|GO:0036211,protein modification process|GO:0006807,nitrogen compound metabolic process|GO:0044237,cellular metabolic process|GO:0008152,metabolic process|GO:0000209,protein polyubiquitination	GO:0043234,protein complex|GO:0032991,macromolecular complex|GO:1902494,catalytic complex|GO:0044464,cell part|GO:0000151,ubiquitin ligase complex|GO:0005623,cell|GO:0005622,intracellular|GO:0019005,SCF ubiquitin ligase complex|GO:1990234,transferase complex|GO:0044424,intracellular part|GO:0031461,cullin-RING ubiquitin ligase complex|GO:0005737,cytoplasm|GO:0005829,cytosol|GO:0044444,cytoplasmic part	GO:0001948,glycoprotein binding|GO:0097367,carbohydrate derivative binding|GO:0005488,binding|GO:0005515,protein binding|GO:0019787,ubiquitin-like protein transferase activity|GO:0003824,catalytic activity|GO:0016740,transferase activity|GO:0004842,ubiquitin-protein transferase activity

<table>
<tr><td>HRSP12</td><td>GO:0080090,regulation of primary metabolic process|GO:0019222,regulation of metabolic process|GO:0050680,negative regulation of epithelial cell proliferation|GO:0060322,head development|GO:0071840,cellular component organization or biogenesis|GO:0010605,negative regulation of macromolecule metabolic process|GO:0010608,posttranscriptional regulation of gene expression|GO:0043043,peptide biosynthetic process|GO:0001822,kidney development|GO:0048513,animal organ development|GO:0048519,negative regulation of biological process|GO:1902074,response to salt|GO:0060541,respiratory system development|GO:0006449,regulation of translational termination|GO:0060255,regulation of macromolecule metabolic process|GO:0032268,regulation of cellular protein metabolic process|GO:0010033,response to organic substance|GO:0046483,heterocycle metabolic process|GO:1901564,organonitrogen compound metabolic process|GO:1901566,organonitrogen compound biosynthetic process|GO:0044707,single-multicellular organism process|GO:0019538,protein metabolic process|GO:0009892,negative regulation of metabolic process|GO:0009890,negative regulation of biosynthetic process|GO:0010629,negative regulation of gene expression|GO:0006807,nitrogen compound metabolic process|GO:0043170,macromolecule metabolic process|GO:0050789,regulation of biological process|GO:0044267,cellular protein metabolic process|GO:0044260,cellular macromolecule metabolic process|GO:0016043,cellular component organization|GO:0065007,biological regulation|GO:0044699,single-organism process|GO:0009889,regulation of biosynthetic process|GO:0050794,regulation of cellular process|GO:0008152,metabolic process|GO:0016070,RNA metabolic process|GO:0044271,cellular nitrogen compound biosynthetic process|GO:0007420,brain development|GO:0050896,response to stimulus|GO:0010556,regulation of macromolecule biosynthetic process|GO:0044763,single-organism cellular process|GO:0006518,peptide metabolic process|GO:0010558,negative regulation of macromolecule biosynthetic process|GO:0051128,regulation of cellular component organization|GO:0044249,cellular biosynthetic process|GO:0034641,cellular nitrogen compound metabolic process|GO:0034645,cellular macromolecule biosynthetic process|GO:0043244,regulation of protein complex disassembly|GO:0043241,protein complex disassembly|GO:0007049,cell cycle|GO:0007417,central nervous system development|GO:0006139,nucleobase-containing compound metabolic process|GO:0051248,negative regulation of protein metabolic process|GO:0051246,regulation of protein metabolic process|GO:0043933,macromolecular complex subunit organization|GO:0032502,developmental process|GO:0008285,negative regulation of cell proliferation|GO:0032501,multicellular organismal process|GO:0008283,cell proliferation|GO:0009987,cellular process|GO:0006725,cellular aromatic compound metabolic process|GO:0034249,negative regulation of cellular amide metabolic process|GO:0034248,regulation of cellular amide metabolic process|GO:0001655,urogenital system development|GO:0072001,renal system development|GO:0090502,RNA phosphodiester bond hydrolysis, endonucleolytic|GO:0043604,amide biosynthetic process|GO:0032269,negative regulation of cellular protein metabolic process|GO:0090501,RNA phosphodiester bond hydrolysis|GO:0050678,regulation of epithelial cell proliferation|GO:0043603,cellular amide metabolic process|GO:0050673,epithelial cell proliferation|GO:0017148,negative regulation of translation|GO:0048731,system development|GO:0042127,regulation of cell proliferation|GO:0030323,respiratory tube development|GO:0030324,lung development|GO:0031327,negative regulation of cellular biosynthetic process|GO:0031326,regulation of cellular biosynthetic process|GO:0031324,negative regulation of cellular metabolic process|GO:0031323,regulation of cellular metabolic process|GO:0070314,G1 to G0 transition|GO:0090304,nucleic acid metabolic process|GO:0090305,nucleic acid phosphodiester bond hydrolysis|GO:0022402,cell cycle process|GO:0007275,multicellular organism development|GO:0032984,macromolecular complex disassembly|GO:0071822,protein complex subunit organization|GO:0033993,response to lipid|GO:1901360,organic cyclic compound metabolic process|GO:2000112,regulation of cellular macromolecule biosynthetic process|GO:2000113,negative regulation of cellular macromolecule biosynthetic process|GO:0071704,organic substance metabolic process|GO:0010467,gene expression|GO:0010468,regulation of gene expression|GO:1901576,organic substance biosynthetic process|GO:0044767,single-organism developmental process|GO:0009058,biosynthetic process|GO:0009059,macromolecule biosynthetic process|GO:0022411,cellular component disassembly|GO:0051171,regulation of nitrogen compound metabolic process|GO:0051172,negative regulation of nitrogen compound metabolic process|GO:0042221,response to chemical|GO:0035295,tube development|GO:0043624,cellular protein complex disassembly|GO:0044238,primary metabolic process|GO:0007399,nervous system development|GO:0048856,anatomical structure development|GO:0044237,cellular metabolic process|GO:0006417,regulation of translation|GO:0006415,translational termination|GO:0006412,translation|GO:0048523,negative regulation of cellular process|GO:1901565,organonitrogen compound catabolic process|GO:1901575,organic substance catabolic process|GO:0009056,catabolic process|GO:0006567,threonine catabolic process|GO:0006566,threonine metabolic process|GO:0044710,single-organism metabolic process|GO:0006082,organic acid metabolic process|GO:0019518,L-threonine catabolic process to glycine|GO:0046395,carboxylic acid catabolic process|GO:0016054,organic acid catabolic process|GO:0006520,cellular amino acid metabolic process|GO:0019752,carboxylic acid metabolic process|GO:0044281,small molecule metabolic process|GO:0009063,cellular amino acid catabolic process|GO:0044712,single-organism catabolic process|GO:0009066,aspartate family amino acid metabolic process|GO:0044282,small molecule catabolic process|GO:0006544,glycine metabolic process|GO:0043436,oxoacid metabolic process|GO:0009069,serine family amino acid metabolic process|GO:0009068,aspartate family amino acid catabolic process|GO:1901605,alpha-amino acid metabolic process|GO:0044248,cellular catabolic process|GO:1901606,alpha-amino acid catabolic process</td><td>GO:0042579,microbody|GO:0043229,intracellular organelle|GO:0043227,membrane-bounded organelle|GO:0043226,organelle|GO:0005737,cytoplasm|GO:0031982,vesicle|GO:0005634,nucleus|GO:0005777,peroxisome|GO:0005739,mitochondrion|GO:0031988,membrane-bounded vesicle|GO:1903561,extracellular vesicle|GO:0070062,extracellular exosome|GO:0043230,extracellular organelle|GO:0043231,intracellular membrane-bounded organelle|GO:0005829,cytosol|GO:0044464,cell part|GO:0005623,cell|GO:0005622,intracellular|GO:0044444,cytoplasmic part|GO:0005576,extracellular region|GO:0044424,intracellular part|GO:0065010,extracellular membrane-bounded organelle|GO:0044421,extracellular region part|GO:0031974,membrane-enclosed lumen|GO:0005759,mitochondrial matrix|GO:0043233,organelle lumen|GO:0044446,intracellular organelle part|GO:0070013,intracellular organelle lumen|GO:0044429,mitochondrial part|GO:0044422,organelle part</td><td>GO:0005488,binding|GO:0003676,nucleic acid binding|GO:1901363,heterocyclic compound binding|GO:0036041,long-chain fatty acid binding|GO:0043168,anion binding|GO:0043169,cation binding|GO:0046914,transition metal ion binding|GO:1904013,xenon atom binding|GO:1904012,platinum binding|GO:0004540,ribonuclease activity|GO:0004521,endoribonuclease activity|GO:0043167,ion binding|GO:0019239,deaminase activity|GO:0005504,fatty acid binding|GO:0046872,metal ion binding|GO:0042802,identical protein binding|GO:0042803,protein homodimerization activity|GO:0016787,hydrolase activity|GO:0044822,poly(A) RNA binding|GO:0008289,lipid binding|GO:0016788,hydrolase activity, acting on ester bonds|GO:0016892,endoribonuclease activity, producing 3'-phosphomonoesters|GO:0003824,catalytic activity|GO:0016894,endonuclease activity, active with either ribo- or deoxyribonucleic acids and producing 3'-phosphomonoesters|GO:0033293,monocarboxylic acid binding|GO:0036094,small molecule binding|GO:0004518,nuclease activity|GO:0004519,endonuclease activity|GO:0031406,carboxylic acid binding|GO:0043177,organic acid binding|GO:0003723,RNA binding|GO:0005515,protein binding|GO:0097159,organic cyclic compound binding|GO:0046983,protein dimerization activity</td></tr>
<tr><td>AC093157</td><td>GO:0044249,cellular biosynthetic process|GO:0043170,macromolecule metabolic process|GO:0044699,single-organism process|GO:0044267,cellular protein metabolic process|GO:0044710,single-organism metabolic process|GO:0044711,single-organism biosynthetic process|GO:0044260,cellular macromolecule metabolic process|GO:0018193,peptidyl-amino acid modification|GO:0071704,organic substance metabolic process|GO:0032259,methylation|GO:0009987,cellular process|GO:0006464,cellular protein modification process|GO:0009058,biosynthetic process|GO:0036211,protein modification process|GO:0044763,single-organism cellular process|GO:0008152,metabolic process|GO:0044238,primary metabolic process|GO:0017183,peptidyl-diphthamide biosynthetic process from peptidyl-histidine|GO:0017182,peptidyl-diphthamide metabolic process|GO:0043412,macromolecule modification|GO:0044237,cellular metabolic process|GO:0018202,peptidyl-histidine modification|GO:0019538,protein metabolic process</td><td></td><td>GO:0004164,diphthine synthase activity|GO:0008757,S-adenosylmethionine-dependent methyltransferase activity|GO:0003824,catalytic activity|GO:0016740,transferase activity|GO:0016741,transferase activity, transferring one-carbon groups|GO:0008168,methyltransferase activity</td></tr>
<tr><td>CYP4A11</td><td>GO:0051046,regulation of secretion|GO:0051047,positive regulation of secretion|GO:0051049,regulation of transport|GO:0043436,oxoacid metabolic process|GO:0006820,anion transport|GO:0003013,circulatory system process|GO:0003014,renal system process|GO:0044281,small molecule metabolic process|GO:0044282,small molecule catabolic process|GO:0098771,inorganic ion homeostasis|GO:0055078,sodium ion homeostasis|GO:0044712,single-organism catabolic process|GO:0044710,single-organism metabolic process|GO:0003091,renal water homeostasis|GO:0003095,pressure natriuresis|GO:0097267,omega-hydroxylase P450 pathway|GO:0048518,positive regulation of biological process|GO:0033036,macromolecule localization|GO:0015718,monocarboxylic acid transport|GO:0051050,positive regulation of transport|GO:0015849,organic acid transport|GO:0015711,organic anion transport|GO:0032787,monocarboxylic acid metabolic process|GO:0072329,monocarboxylic acid catabolic process|GO:0042758,long-chain fatty acid catabolic process|GO:0010876,lipid localization|GO:0055114,oxidation-reduction process|GO:0003008,system process|GO:0048871,multicellular organismal homeostasis|GO:0044707,single-multicellular organism process|GO:1901568,fatty acid derivative metabolic process|GO:1901569,fatty acid derivative catabolic process|GO:0016054,organic acid catabolic process|GO:0048878,chemical homeostasis|GO:0032305,positive regulation of icosanoid secretion|GO:0032303,regulation of icosanoid secretion|GO:0033559,unsaturated fatty acid metabolic process|GO:0032309,icosanoid secretion|GO:1903793,positive regulation of anion transport|GO:0046717,acid secretion|GO:0009062,fatty acid catabolic process|GO:0050789,regulation of biological process|GO:0015908,fatty acid transport|GO:0003073,regulation of systemic arterial blood pressure|GO:1901575,organic substance catabolic process|GO:0003071,renal system process involved in regulation of systemic arterial blood pressure|GO:1901571,fatty acid derivative transport|GO:0036102,leukotriene B4 metabolic process|GO:0036100,leukotriene catabolic process|GO:0036101,leukotriene B4 catabolic process|GO:0016042,lipid catabolic process|GO:0065007,biological regulation|GO:0065008,regulation of biological quality|GO:0006629,lipid metabolic process|GO:0008015,blood circulation|GO:0006811,ion transport|GO:0006810,transport|GO:0051716,cellular response to stimulus|GO:0008152,metabolic process|GO:0001676,long-chain fatty acid metabolic process|GO:0032892,positive regulation of organic acid transport|GO:0051234,establishment of localization|GO:0046903,secretion|GO:0032368,regulation of lipid transport|GO:0046395,carboxylic acid catabolic process|GO:0050896,response to stimulus|GO:0050891,multicellular organismal water homeostasis|GO:0006691,leukotriene metabolic process|GO:0006690,icosanoid metabolic process|GO:0001977,renal system process involved in regulation of blood volume|GO:1901616,organic hydroxy compound catabolic process|GO:1901615,organic hydroxy compound metabolic process|GO:0006631,fatty acid metabolic process|GO:0006869,lipid transport|GO:0050801,ion homeostasis|GO:0044248,cellular catabolic process|GO:0032890,regulation of organic acid transport|GO:0009410,response to xenobiotic stimulus|GO:0070887,cellular response to chemical stimulus|GO:0044242,cellular lipid catabolic process|GO:0044699,single-organism process|GO:0032370,positive regulation of lipid transport|GO:0032501,multicellular organismal process|GO:2000193,positive regulation of fatty acid transport|GO:0050878,regulation of body fluid levels|GO:2000191,regulation of fatty acid transport|GO:0009987,cellular process|GO:0043270,positive regulation of ion transport|GO:0055080,cation homeostasis|GO:0044255,cellular lipid metabolic process|GO:0032879,regulation of localization|GO:0006082,organic acid metabolic process|GO:0006805,xenobiotic metabolic process|GO:0071715,icosanoid transport|GO:1901523,icosanoid catabolic process|GO:0019752,carboxylic acid metabolic process|GO:0042592,homeostatic process|GO:0008217,regulation of blood pressure|GO:0044070,regulation of anion transport|GO:0043269,regulation of ion transport|GO:0071466,cellular response to xenobiotic stimulus|GO:0071704,organic substance metabolic process|GO:0071702,organic substance transport|GO:0019373,epoxygenase P450 pathway|GO:0044765,single-organism transport|GO:0044763,single-organism cellular process|GO:0055067,monovalent inorganic cation homeostasis|GO:0055065,metal ion homeostasis|GO:0030104,water homeostasis|GO:0042221,response to chemical|GO:0009056,catabolic process|GO:0051179,localization|GO:1902578,single-organism localization|GO:0046942,carboxylic acid transport|GO:0044238,primary metabolic process|GO:0044237,cellular metabolic process|GO:0019369,arachidonic acid metabolic process|GO:0080090,regulation of primary metabolic process|GO:0019222,regulation of metabolic process|GO:0019216,regulation of lipid</td><td>GO:0098590,plasma membrane region|GO:0005783,endoplasmic reticulum|GO:0005789,endoplasmic reticulum membrane|GO:0042175,nuclear outer membrane-endoplasmic reticulum membrane network|GO:0043229,intracellular organelle|GO:0071944,cell periphery|GO:0043227,membrane-bounded organelle|GO:0043226,organelle|GO:0005737,cytoplasm|GO:0045177,apical part of cell|GO:0031982,vesicle|GO:0098805,whole membrane|GO:0016020,membrane|GO:0031988,membrane-bounded vesicle|GO:0044432,endoplasmic reticulum part|GO:0044459,plasma membrane part|GO:0016324,apical plasma membrane|GO:0098588,bounding membrane of organelle|GO:0098589,membrane region|GO:0012505,endomembrane system|GO:0005886,plasma membrane|GO:1903561,extracellular vesicle|GO:0031090,organelle membrane|GO:0043230,extracellular organelle|GO:0043231,intracellular membrane-bounded organelle|GO:0070062,extracellular exosome|GO:0044464,cell part|GO:0005623,cell|GO:0005622,intracellular|GO:0044446,intracellular organelle part|GO:0044444,cytoplasmic part|GO:0005576,extracellular region|GO:0044424,intracellular part|GO:0044425,membrane part|GO:0065010,extracellular membrane-bounded organelle|GO:0044421,extracellular region part|GO:0044422,organelle part|GO:0031984,organelle subcompartment|GO:0098827,endoplasmic reticulum subcompartment</td><td>GO:0052869,arachidonic acid omega-hydroxylase activity|GO:0004497,monooxygenase activity|GO:0005488,binding|GO:1901363,heterocyclic compound binding|GO:0016491,oxidoreductase activity|GO:0043169,cation binding|GO:0046914,transition metal ion binding|GO:0016709,oxidoreductase activity, acting on paired donors, with incorporation or reduction of molecular oxygen, NAD(P)H as one donor, and incorporation of one atom of oxygen|GO:0050051,leukotriene-B4 20-monooxygenase activity|GO:0016705,oxidoreductase activity, acting on paired donors, with incorporation or reduction of molecular oxygen|GO:0043167,ion binding|GO:0005506,iron ion binding|GO:0046872,metal ion binding|GO:0020037,heme binding|GO:0003824,catalytic activity|GO:0046906,tetrapyrrole binding|GO:0097159,organic cyclic compound binding|GO:0016713,oxidoreductase activity, acting on paired donors, with incorporation or reduction of molecular oxygen, reduced iron-sulfur protein as one donor, and incorporation of one atom of oxygen|GO:0018685,alkane 1-monooxygenase activity|GO:0008392,arachidonic acid epoxygenase activity|GO:0008391,arachidonic acid monooxygenase activity|GO:0019825,oxygen binding</td></tr>
<tr><td>FAM98B</td><td>GO:0090304,nucleic acid metabolic process|GO:0034641,cellular nitrogen compound metabolic process|GO:0006807,nitrogen compound metabolic process|GO:0034660,ncRNA metabolic process|GO:0008033,tRNA processing|GO:1901360,organic cyclic compound metabolic process|GO:0006139,nucleobase-containing compound metabolic process|GO:0044260,cellular macromolecule metabolic process|GO:0071704,organic substance metabolic process|GO:0010467,gene expression|GO:0006388,tRNA splicing, via endonucleolytic cleavage and ligation|GO:0008380,RNA splicing|GO:0034470,ncRNA processing|GO:0009987,cellular process|GO:0006725,cellular aromatic compound metabolic process|GO:0008152,metabolic process|GO:0046483,heterocycle metabolic process|GO:0016070,RNA metabolic process|GO:0044238,primary metabolic process|GO:0000394,RNA splicing, via endonucleolytic cleavage and ligation|GO:0044237,cellular metabolic process|GO:0043170,macromolecule metabolic process|GO:0006399,tRNA metabolic process|GO:0006396,RNA processing|GO:0006479,protein methylation|GO:0042127,regulation of cell proliferation|GO:0009893,positive regulation of metabolic process|GO:0019222,regulation of metabolic process|GO:0008213,protein alkylation|GO:0010628,positive regulation of gene expression|GO:0050789,regulation of biological process|GO:0044699,single-organism process|GO:0044267,cellular protein metabolic process|GO:0010604,positive regulation of macromolecule metabolic process|GO:0065007,biological regulation|GO:0048518,positive regulation of biological process|GO:0032259,methylation|GO:0010468,regulation of gene expression|GO:0008284,positive regulation of cell proliferation|GO:0008283,cell proliferation|GO:0060255,regulation of macromolecule metabolic process|GO:0006464,cellular protein modification process|GO:0050794,regulation of cellular process|GO:0043412,macromolecule modification|GO:0036211,protein modification process|GO:0043414,macromolecule methylation|GO:1901564,organonitrogen compound metabolic</td><td>GO:0043234,protein complex|GO:0005737,cytoplasm|GO:0072669,tRNA-splicing ligase complex|GO:0032991,macromolecular complex|GO:0043231,intracellular membrane-bounded organelle|GO:0005634,nucleus|GO:0044464,cell part|GO:0005623,cell|GO:0005622,intracellular|GO:0043229,intracellular organelle|GO:0044424,intracellular part|GO:0043227,membrane-bounded organelle|GO:0043226,organelle|GO:0031974,membrane-enclosed lumen|GO:0031981,nuclear lumen|GO:0005654,nucleoplasm|GO:0043233,organelle lumen|GO:0044446,intracellular organelle part|GO:0070013,intracellular organelle lumen|GO:0044428,nuclear part|GO:0044422,organelle part</td><td>GO:0097159,organic cyclic compound binding|GO:0044822,poly(A) RNA binding|GO:0005488,binding|GO:0003676,nucleic acid binding|GO:1901363,heterocyclic compound binding|GO:0003723,RNA binding|GO:0003824,catalytic activity|GO:0016741,transferase activity, transferring one-carbon groups|GO:0016740,transferase activity|GO:0008276,protein methyltransferase activity|GO:0005515,protein binding|GO:0008168,methyltransferase activity|GO:0042802,identical protein binding</td></tr>
<tr><td>ZG16</td><td>GO:0033036,macromolecule localization|GO:0008104,protein localization|GO:0006810,transport|GO:0045184,establishment of protein localization|GO:0071702,organic substance transport|GO:0015031,protein transport|GO:0051234,establishment of localization|GO:0051179,localization|GO:0042886,amide transport|GO:0071705,nitrogen compound transport|GO:0015833,peptide transport</td><td>GO:0044421,extracellular region part|GO:0031974,membrane-enclosed lumen|GO:0031012,extracellular matrix|GO:0043229,intracellular organelle|GO:0060205,cytoplasmic membrane-bounded vesicle lumen|GO:0043227,membrane-bounded organelle|GO:0043226,organelle|GO:0005737,cytoplasm|GO:0030667,secretory granule membrane|GO:0031983,vesicle lumen|GO:0031982,vesicle|GO:0016023,cytoplasmic, membrane-bounded vesicle|GO:0031410,cytoplasmic vesicle|GO:0098805,whole membrane|GO:0016020,membrane|GO:0031988,membrane-bounded vesicle|GO:0044433,cytoplasmic vesicle part|GO:0044431,Golgi apparatus part|GO:0005794,Golgi apparatus|GO:0005796,Golgi lumen|GO:0030141,secretory granule|GO:0098588,bounding membrane of organelle|GO:0030659,cytoplasmic vesicle membrane|GO:0012505,endomembrane system|GO:0012506,vesicle membrane|GO:0042589,zymogen granule membrane|GO:0042588,zymogen granule|GO:0031090,organelle membrane|GO:0043231,intracellular membrane-bounded organelle|GO:0043233,organelle lumen|GO:0044464,cell part|GO:0005623,cell|GO:0005622,intracellular|GO:0044446,intracellular organelle part|GO:0070013,intracellular organelle lumen|GO:0044444,cytoplasmic part|GO:0005576,extracellular region|GO:0044424,intracellular part|GO:0005578,proteinaceous extracellular matrix|GO:0044422,organelle part|GO:0099503,secretory</td><td>GO:0030246,carbohydrate binding|GO:0005515,protein binding|GO:0005488,binding</td></tr>
<tr><td>FAM213A</td><td>GO:0008152,metabolic process|GO:0030154,cell differentiation|GO:0050789,regulation of biological process|GO:0044699,single-organism process|GO:0002761,regulation of myeloid leukocyte differentiation|GO:0044710,single-organism metabolic process|GO:0048869,cellular developmental process|GO:0002573,myeloid leukocyte differentiation|GO:0048513,animal organ development|GO:0002682,regulation of immune system process|GO:0055114,oxidation-reduction process|GO:0065007,biological regulation|GO:0048534,hematopoietic or lymphoid organ development|GO:0032502,developmental process|GO:0032501,multicellular organismal process|GO:0050793,regulation of developmental process|GO:0030316,osteoclast differentiation|GO:0050794,regulation of cellular process|GO:0044767,single-organism developmental process|GO:0045595,regulation of cell differentiation|GO:0044763,single-organism cellular process|GO:0051239,regulation of multicellular organismal process|GO:0048731,system development|GO:1903706,regulation of hemopoiesis|GO:0002521,leukocyte differentiation|GO:0002520,immune system development|GO:0044707,single-multicellular organism process|GO:0048856,anatomical structure development|GO:0002376,immune system process|GO:1902105,regulation of leukocyte differentiation|GO:2000026,regulation of multicellular organismal development|GO:0045670,regulation of osteoclast differentiation|GO:0030099,myeloid cell differentiation|GO:0007275,multicellular organism development|GO:0030097,hemopoiesis|GO:0009987,cellular process|GO:0045637,regulation of myeloid cell differentiation|GO:0098754,detoxification|GO:0009636,response to toxic substance|GO:0098869,cellular oxidant detoxification|GO:1990748,cellular detoxification|GO:0042221,response to chemical|GO:0050896,response to stimulus</td><td>GO:0043231,intracellular membrane-bounded organelle|GO:0005737,cytoplasm|GO:0005622,intracellular|GO:0043230,extracellular organelle|GO:0031982,vesicle|GO:0044421,extracellular region part|GO:0043227,membrane-bounded organelle|GO:0044464,cell part|GO:0044444,cytoplasmic part|GO:0005739,mitochondrion|GO:0031988,membrane-bounded vesicle|GO:0043229,intracellular organelle|GO:0005576,extracellular region|GO:0005623,cell|GO:0044424,intracellular part|GO:1903561,extracellular vesicle|GO:0065010,extracellular membrane-bounded organelle|GO:0043226,organelle|GO:0070062,extracellular exosome</td><td>GO:0016209,antioxidant activity</td></tr>
<tr><td>RABL5</td><td>GO:0008104,protein localization|GO:0023052,signaling|GO:0007165,signal transduction|GO:0035556,intracellular signal transduction|GO:0050789,regulation of biological process|GO:0071840,cellular component organization or biogenesis|GO:0051716,cellular response to stimulus|GO:0051234,establishment of localization|GO:0070727,cellular macromolecule localization|GO:0006886,intracellular protein transport|GO:0016043,cellular component organization|GO:0065007,biological regulation|GO:0044699,single-organism process|GO:0071702,organic substance transport|GO:0033036,macromolecule localization|GO:0034613,cellular protein localization|GO:0006810,transport|GO:0009987,cellular process|GO:0050794,regulation of cellular process|GO:0045184,establishment of protein localization|GO:0032482,Rab protein signal transduction|GO:0044763,single-organism cellular process|GO:0051649,establishment of localization in cell|GO:0007154,cell communication|GO:0007265,Ras protein signal transduction|GO:0007264,small GTPase mediated signal transduction|GO:0051179,localization|GO:0051641,cellular localization|GO:0006996,organelle organization|GO:0044700,single organism signaling|GO:0046907,intracellular transport|GO:0050896,response to stimulus|GO:0015031,protein transport|GO:0030030,cell projection organization|GO:0042073,intraciliary transport|GO:0030705,cytoskeleton-dependent intracellular transport|GO:0015833,peptide transport|GO:0009653,anatomical structure morphogenesis|GO:0000902,cell morphogenesis|GO:0048869,cellular developmental process|GO:0032989,cellular component morphogenesis|GO:0071705,nitrogen compound transport|GO:0098840,protein transport along microtubule|GO:0032502,developmental process|GO:0031503,protein complex localization|GO:0060271,cilium morphogenesis|GO:0042886,amide transport|GO:0044767,single-organism developmental process|GO:0044765,single-organism transport|GO:0010970,transport along microtubule|GO:1902578,single-organism localization|GO:0032990,cell part morphogenesis|GO:0048858,cell projection morphogenesis|GO:0048856,anatomical structure development|GO:1902589,single-organism organelle organization|GO:0035735,intraciliary transport involved in cilium assembly|GO:1902582,single-organism intracellular transport|GO:0044782,cilium organization</td><td>GO:0043234,protein complex|GO:0030992,intraciliary transport particle B|GO:0042995,cell projection|GO:0072372,primary cilium|GO:0032991,macromolecular complex|GO:0044441,ciliary part|GO:0044464,cell part|GO:0005623,cell|GO:0005622,intracellular|GO:0097542,ciliary tip|GO:0030990,intraciliary transport particle|GO:0005929,cilium|GO:0044463,cell projection part|GO:0043226,organelle|GO:0044422,organelle part|GO:0043229,intracellular organelle|GO:0043228,non-membrane-bounded organelle|GO:0005856,cytoskeleton|GO:0005813,centrosome|GO:0005815,microtubule organizing center|GO:0044430,cytoskeletal part|GO:0015630,microtubule cytoskeleton|GO:0043232,intracellular non-membrane-bounded organelle|GO:0044446,intracellular organelle part|GO:0044424,intracellular part</td><td>GO:0035639,purine ribonucleoside triphosphate binding|GO:0005488,binding|GO:0043167,ion binding|GO:1901363,heterocyclic compound binding|GO:1901265,nucleoside phosphate binding|GO:0001882,nucleoside binding|GO:0043168,anion binding|GO:0016462,pyrophosphatase activity|GO:0000166,nucleotide binding|GO:0032561,guanyl ribonucleotide binding|GO:0001883,purine nucleoside binding|GO:0032549,ribonucleoside binding|GO:0017076,purine nucleotide binding|GO:0005525,GTP binding|GO:0016787,hydrolase activity|GO:0003924,GTPase activity|GO:0017111,nucleoside-triphosphatase activity|GO:0036094,small molecule binding|GO:0003824,catalytic activity|GO:0016818,hydrolase activity, acting on acid anhydrides, in phosphorus-containing anhydrides|GO:0097367,carbohydrate derivative binding|GO:0097159,organic cyclic compound binding|GO:0016817,hydrolase activity, acting on acid anhydrides|GO:0019001,guanyl nucleotide binding|GO:0032555,purine ribonucleotide binding|GO:0032550,purine ribonucleoside binding|GO:0032553,ribonucleotide binding</td></tr>
</table>
