# Supplementary material for: Exosomal Circular RNA as a Biomarker Platform for the Early Diagnosis of Immune-Mediated Demyelinating Disease
Source: Front Genet. 2019 Sep 27;10:860. doi: 10.3389/fgene.2019.00860 (PMC6777646; doi:10.3389/fgene.2019.00860)
Supplement: Supplementary Table 6 — KEGG enrichment on potential target genes that may be regulated by differentially expressed circRNAs. [file Table_6.pdf]

Supplementary Table 6

Pathway Name	Pathway ID	Pvalue	Pvalue_adjusted	Genes	Count	Pop Hit	List_Total	Background Genes	Class
RIG-I-like receptor signaling pathway	hsa04622	1.93E-03	3.53E-01	IFNK|1;IKBKG|1;IFNW1|1;IFNA7|1;IFNA14|1;ATG12|1;TRADD|1	7	70	180	7057	Organismal Systems
Regulation of autophagy	hsa04140	3.26E-03	3.53E-01	GABARAPL2|1;IFNA7|1;IFNA14|1;GABARAP|1;ATG12|1	5	40	180	7057	Cellular Processes
p53 signaling pathway	hsa04115	8.03E-03	4.03E-01	BBC3|1;CDK6|1;CCNG1|1;CDKN2A|1;TP73|1;CCND3|1	6	69	180	7057	Cellular Processes
SNARE interactions in vesicular transport	hsa04130	1.04E-02	4.03E-01	BET1L|1;GOSR1|1;VAMP1|1;STX4|1	4	34	180	7057	Genetic Information Processing
Metabolic pathways	hsa01100	1.12E-02	4.03E-01	COX5A|1;NDUFB8|1;PANK1|1;HIBCH|1;TYMS|1;NDUFA4|1;CHPF2|1;FAH|1;ALOX12B|1;FUT9|1;ADH1B|1;GPAT3|1;ACSM2A|1;FOLH1|1;TMEM23|1;LIPT1|1;CMPK1|1;CYP3A5|1;ATP5C1|1;PSPH|1;NAT8L|1;PTS|1;GALNT18|1;MAN2A1|1;MCEE|1;UQCRB|1;NDUFB4|1;TWISTNB|1;NT5C1A|1;ATP6V1D|1;NMNAT2|1;B3GALT2|1;IMPA1|1;DPM2|1;CMPK2|1;HGSNAT|1;PYCR1|1;CYP2B6|1;ST3GAL3|1;GALNT16|1;DHFR|1;BTD|1;AOC3|1;CYP4A11|1	44	1239	180	7057	Metabolism
One carbon pool by folate	hsa00670	1.35E-02	4.03E-01	TYMS|1;MTFMT|1;DHFR|1	3	20	180	7057	Metabolism
Primary immunodeficiency	hsa05340	1.40E-02	4.03E-01	IKBKG|1;TAP2|1;TNFRSF13C|1;ICOS|1	4	37	180	7057	Human Diseases
Non-alcoholic fatty liver disease (NAFLD)	hsa04932	1.49E-02	4.03E-01	COX5A|1;NDUFB8|1;NDUFA4|1;DDIT3|1;FASLG|1;UQCRB|1;NDUFB4|1;MLXIP|1;PPARA|1	9	151	180	7057	Human Diseases
Oxidative phosphorylation	hsa00190	2.01E-02	4.83E-01	COX5A|1;NDUFB8|1;NDUFA4|1;ATP5C1|1;UQCRB|1;LHPP|1;NDUFB4|1;ATP6V1D|	8	133	180	7057	Metabolism
Measles	hsa05162	2.27E-02	4.83E-01	BBC3|1;CDK6|1;FASLG|1;RAB9A|1;IFNA7|1;IFNA14|1;TP73|1;CCND3|1	8	136	180	7057	Human Diseases
GABAergic synapse	hsa04727	2.45E-02	4.83E-01	GABRA4|1;GNG12|1;GNG13|1;KCNJ6|1;GABARAPL2|1;GABARAP|1	6	88	180	7057	Organismal Systems
Glycosphingolipid biosynthesis - lacto and neolacto series	hsa00601	2.76E-02	4.99E-01	FUT9|1;B3GALT2|1;ST3GAL3|1	3	26	180	7057	Metabolism
Transcriptional misregulation in cancer	hsa05202	4.03E-02	6.73E-01	REL|1;HDAC2|1;H3F3A|1;FLI1|1;DDIT3|1;ATF1|1;HMGA2|1;CDKN2C|1;CSF2|1	9	180	180	7057	Human Diseases
Retrograde endocannabinoid signaling	hsa04723	4.38E-02	6.79E-01	GABRA4|1;GNG12|1;GNG13|1;GRIA4|1;KCNJ6|1;ABHD6|1	6	101	180	7057	Organismal Systems
Folate biosynthesis	hsa00790	4.81E-02	6.97E-01	PTS|1;DHFR|1	2	14	180	7057	Metabolism
Huntington's disease	hsa05016	5.81E-02	7.51E-01	COX5A|1;NDUFB8|1;HDAC2|1;BBC3|1;NDUFA4|1;GRIN2B|1;ATP5C1|1;UQCRB|1;NDUFB4|1	9	193	180	7057	Human Diseases
Tyrosine metabolism	hsa00350	5.89E-02	7.51E-01	FAH|1;ADH1B|1;AOC3|1	3	35	180	7057	Metabolism
Parkinson's disease	hsa05012	7.05E-02	7.62E-01	COX5A|1;NDUFB8|1;NDUFA4|1;ATP5C1|1;UBE2L6|1;UQCRB|1;NDUFB4|1	7	142	180	7057	Human Diseases
Glutamatergic synapse	hsa04724	7.07E-02	7.62E-01	GNG12|1;GNG13|1;GRIN2B|1;GRIA4|1;SLC1A6|1;GRIK1|1	6	114	180	7057	Organismal Systems
Biotin metabolism	hsa00780	7.46E-02	7.62E-01	BTD|1	1	3	180	7057	Metabolism
Lipoic acid metabolism	hsa00785	7.46E-02	7.62E-01	LIPT1|1	1	3	180	7057	Metabolism
Nicotine addiction	hsa05033	8.11E-02	7.62E-01	GABRA4|1;GRIN2B|1;GRIA4|1	3	40	180	7057	Human Diseases
Retinol metabolism	hsa00830	8.36E-02	7.62E-01	ADH1B|1;CYP3A5|1;CYP2B6|1;CYP4A11|1	4	65	180	7057	Metabolism
Circadian entrainment	hsa04713	9.48E-02	7.62E-01	GNG12|1;GNG13|1;GRIN2B|1;GRIA4|1;KCNJ6|1	5	95	180	7057	Organismal Systems
Cell cycle	hsa04110	9.67E-02	7.62E-01	HDAC2|1;CDK6|1;CDC26|1;CDKN2C|1;CDKN2A|1;CCND3|1	6	124	180	7057	Cellular Processes
Hippo signaling pathway	hsa04390	9.83E-02	7.62E-01	FZD3|1;TEAD3|1;BBC3|1;BMP7|1;AMH|1;TP73|1;CCND3|1	7	154	180	7057	Environmental Information Processing
Drug metabolism - cytochrome P450	hsa00982	9.89E-02	7.62E-01	MGST1|1;ADH1B|1;CYP3A5|1;CYP2B6|1	4	69	180	7057	Metabolism
Fatty acid degradation	hsa00071	1.01E-01	7.62E-01	ADH1B|1;CYP4A22|1;CYP4A11|1	3	44	180	7057	Metabolism
Chronic myeloid leukemia	hsa05220	1.15E-01	7.62E-01	HDAC2|1;CDK6|1;IKBKG|1;CDKN2A|1	4	73	180	7057	Human Diseases
Metabolism of xenobiotics by cytochrome P450	hsa00980	1.15E-01	7.62E-01	MGST1|1;ADH1B|1;CYP3A5|1;CYP2B6|1	4	73	180	7057	Metabolism
Protein export	hsa03060	1.16E-01	7.62E-01	SEC61G|1;SRP9|1	2	23	180	7057	Genetic Information Processing
Notch signaling pathway	hsa04330	1.23E-01	7.62E-01	HDAC2|1;NUMBL|1;CIR1|1	3	48	180	7057	Environmental Information Processing
Hepatitis C	hsa05160	1.24E-01	7.62E-01	OCLN|1;IKBKG|1;IFNA7|1;IFNA14|1;TRADD|1;PPARA|1	6	133	180	7057	Human Diseases
Vitamin digestion and absorption	hsa04977	1.24E-01	7.62E-01	FOLH1|1;BTD|1	2	24	180	7057	Organismal Systems
HTLV-I infection	hsa05166	1.25E-01	7.62E-01	FZD3|1;CDC26|1;ATF1|1;IKBKG|1;CDKN2C|1;EGR1|1;TNFRSF13C|1;CSF2|1;CDKN2A|1;CCND3|1	10	259	180	7057	Human Diseases
Pyrimidine metabolism	hsa00240	1.30E-01	7.62E-01	TYMS|1;CMPK1|1;TWISTNB|1;NT5C1A|1;CMPK2|1	5	105	180	7057	Metabolism
Pathways in cancer	hsa05200	1.36E-01	7.62E-01	FZD3|1;GNG12|1;APPL1|1;ELOC|1;HDAC2|1;LPAR4|1;GNG13|1;CDK6|1;STK36|1;FASLG|1;FGF7|1;CKS1B|1;IKBKG|1;CDKN2A|1	14	397	180	7057	Human Diseases
Alzheimer's disease	hsa05010	1.37E-01	7.62E-01	COX5A|1;NDUFB8|1;NDUFA4|1;GRIN2B|1;ATP5C1|1;UQCRB|1;NDUFB4|1	7	168	180	7057	Human Diseases
Cytokine-cytokine receptor interaction	hsa04060	1.39E-01	7.62E-01	TNFRSF11A|1;BMP7|1;FASLG|1;IFNK|1;IFNW1|1;IFNA7|1;IFNA14|1;AMH|1;TNFRSF13C|1;CSF2|1	10	265	180	7057	Environmental Information Processing
Ribosome	hsa03010	1.40E-01	7.62E-01	RPL36A|1;RPL31|1;RPL37|1;RPS28|1;RPL30|1;MRPL13|1	6	138	180	7057	Genetic Information Processing
Chemical carcinogenesis	hsa05204	1.56E-01	8.11E-01	MGST1|1;CYP3A43|1;ADH1B|1;CYP3A5|1	4	82	180	7057	Human Diseases
Autoimmune thyroid disease	hsa05320	1.59E-01	8.11E-01	FASLG|1;IFNA7|1;IFNA14|1	3	54	180	7057	Human Diseases
PI3K-Akt signaling pathway	hsa04151	1.63E-01	8.11E-01	GNG12|1;LPAR4|1;GNG13|1;CDK6|1;FASLG|1;FGF7|1;IKBKG|1;IFNA7|1;IFNA14|1;PKN2|1;TCL1A|1;CCND3|1	12	342	180	7057	Environmental Information Processing
Pathogenic Escherichia coli infection	hsa05130	1.65E-01	8.11E-01	OCLN|1;WASL|1;TUBB1|1	3	55	180	7057	Human Diseases
Nicotinate and nicotinamide metabolism	hsa00760	1.77E-01	8.11E-01	NT5C1A|1;NMNAT2|1	2	30	180	7057	Metabolism
Other types of O-glycan biosynthesis	hsa00514	1.87E-01	8.11E-01	FUT9|1;ST3GAL3|1	2	31	180	7057	Metabolism
beta-Alanine metabolism	hsa00410	1.87E-01	8.11E-01	HIBCH|1;AOC3|1	2	31	180	7057	Metabolism
Mucin type O-Glycan biosynthesis	hsa00512	1.87E-01	8.11E-01	GALNT18|1;GALNT16|1	2	31	180	7057	Metabolism
Vascular smooth muscle contraction	hsa04270	1.92E-01	8.11E-01	ACTG2|1;CYP4A22|1;MYL6B|1;MYLK2|1;CYP4A11|1	5	120	180	7057	Organismal Systems
Propanoate metabolism	hsa00640	1.96E-01	8.11E-01	HIBCH|1;MCEE|1	2	32	180	7057	Metabolism
Herpes simplex infection	hsa05168	1.97E-01	8.11E-01	SRSF6|1;FASLG|1;IKBKG|1;IFNA7|1;IFNA14|1;TAP2|1;HCFC1|1	7	186	180	7057	Human Diseases
Protein digestion and absorption	hsa04974	1.97E-01	8.11E-01	SLC6A19|1;MEP1A|1;SLC36A1|1;ELN|1	4	90	180	7057	Organismal Systems

Morphine addiction	hsa05032	2.02E-01	8.11E-01	GABRA4|1;GNG12|1;GNG13|1;KCNJ6|1	4	91	180	7057	Human Diseases
Phagosome	hsa04145	2.04E-01	8.11E-01	TFRC|1;SEC61G|1;ATP6V1D|1;TAP2|1;TUBB1|1;FCGR2C|1	6	155	180	7057	Cellular Processes
Arachidonic acid metabolism	hsa00590	2.10E-01	8.11E-01	ALOX12B|1;CYP2B6|1;CYP4A11|1	3	62	180	7057	Metabolism
NF-kappa B signaling pathway	hsa04064	2.13E-01	8.11E-01	TNFRSF11A|1;IKBKG|1;TRADD|1;TNFRSF13C|1	4	93	180	7057	Environmental Information Processing
Jak-STAT signaling pathway	hsa04630	2.16E-01	8.11E-01	IFNK|1;IFNW1|1;IFNA7|1;IFNA14|1;CSF2|1;CCND3|1	6	158	180	7057	Environmental Information Processing
Cytosolic DNA-sensing pathway	hsa04623	2.23E-01	8.11E-01	IKBKG|1;IFNA7|1;IFNA14|1	3	64	180	7057	Organismal Systems
Alanine, aspartate and glutamate metabolism	hsa00250	2.24E-01	8.11E-01	FOLH1|1;NAT8L|1	2	35	180	7057	Metabolism
Prion diseases	hsa05020	2.24E-01	8.11E-01	NCAM1|1;EGR1|1	2	35	180	7057	Human Diseases
Shigellosis	hsa05131	2.30E-01	8.18E-01	IKBKG|1;WASL|1;ELMO3|1	3	65	180	7057	Human Diseases
Pancreatic cancer	hsa05212	2.37E-01	8.18E-01	CDK6|1;IKBKG|1;CDKN2A|1	3	66	180	7057	Human Diseases
Dopaminergic synapse	hsa04728	2.37E-01	8.18E-01	GNG12|1;GNG13|1;GRIN2B|1;GRIA4|1;KCNJ6|1	5	130	180	7057	Organismal Systems
Osteoclast differentiation	hsa04380	2.47E-01	8.37E-01	TNFRSF11A|1;TYROBP|1;IKBKG|1;SIRPG|1;FCGR2C|1	5	132	180	7057	Organismal Systems
Natural killer cell mediated cytotoxicity	hsa04650	2.61E-01	8.42E-01	FASLG|1;TYROBP|1;IFNA7|1;IFNA14|1;CSF2|1	5	135	180	7057	Organismal Systems
Adipocytokine signaling pathway	hsa04920	2.64E-01	8.42E-01	IKBKG|1;TRADD|1;PPARA|1	3	70	180	7057	Organismal Systems
Viral carcinogenesis	hsa05203	2.69E-01	8.42E-01	REL|1;HDAC2|1;CDK6|1;IKBKG|1;TRADD|1;CDKN2A|1;CCND3|1	7	205	180	7057	Human Diseases
Ubiquitin mediated proteolysis	hsa04120	2.71E-01	8.42E-01	ELOC|1;FBXW7|1;CDC26|1;UBE2L6|1;MGRN1|1	5	137	180	7057	Genetic Information Processing
Melanoma	hsa05218	2.71E-01	8.42E-01	CDK6|1;FGF7|1;CDKN2A|1	3	71	180	7057	Human Diseases
Glycine, serine and threonine metabolism	hsa00260	2.72E-01	8.42E-01	PSPH|1;AOC3|1	2	40	180	7057	Metabolism
Apoptosis	hsa04210	2.86E-01	8.51E-01	BBC3|1;DDIT3|1;FASLG|1;IKBKG|1;TRADD|1	5	140	180	7057	Cellular Processes
Glycosphingolipid biosynthesis - globo series	hsa00603	3.04E-01	8.51E-01	FUT9|1	1	14	180	7057	Metabolism
Alcoholism	hsa05034	3.06E-01	8.51E-01	GNG12|1;HDAC2|1;H3F3A|1;GNG13|1;GRIN2B|1;HIST1H2AG|1	6	179	180	7057	Human Diseases
TNF signaling pathway	hsa04668	3.08E-01	8.51E-01	EDN1|1;IKBKG|1;TRADD|1;CSF2|1	4	110	180	7057	Environmental Information Processing
Regulation of actin cytoskeleton	hsa04810	3.09E-01	8.51E-01	GNG12|1;NCKAP1|1;TMSB4X|1;FGF7|1;DIAPH2|1;WASL|1;MYLK2|1	7	215	180	7057	Cellular Processes
Proteasome	hsa03050	3.10E-01	8.51E-01	PSMA8|1;PSMD6|1	2	44	180	7057	Genetic Information Processing
Hepatitis B	hsa05161	3.16E-01	8.51E-01	CDK6|1;FASLG|1;IKBKG|1;IFNA7|1;IFNA14|1	5	146	180	7057	Human Diseases
ABC transporters	hsa02010	3.19E-01	8.51E-01	ABCC9|1;TAP2|1	2	45	180	7057	Environmental Information Processing
Serotonergic synapse	hsa04726	3.20E-01	8.51E-01	GNG12|1;GNG13|1;ALOX12B|1;KCNJ6|1	4	112	180	7057	Organismal Systems
Cardiac muscle contraction	hsa04260	3.20E-01	8.51E-01	COX5A|1;UQCRB|1;CACNG8|1	3	78	180	7057	Organismal Systems
Bacterial invasion of epithelial cells	hsa05100	3.20E-01	8.51E-01	CAV1|1;WASL|1;ELMO3|1	3	78	180	7057	Human Diseases
Glycosaminoglycan biosynthesis - keratan sulfate	hsa00533	3.22E-01	8.51E-01	ST3GAL3|1	1	15	180	7057	-
MicroRNAs in cancer	hsa05206	3.46E-01	8.88E-01	FZD3|1;ZEB2|1;FSCN1|1;CDK6|1;CCNG1|1;HMGA2|1;TRIM71|1;ZFPM2|1;CDKN2A|	9	297	180	7057	Human Diseases
Valine, leucine and isoleucine degradation	hsa00280	3.47E-01	8.88E-01	HIBCH|1;MCEE|1	2	48	180	7057	Metabolism
Phenylalanine metabolism	hsa00360	3.56E-01	8.88E-01	AOC3|1	1	17	180	7057	Metabolism
N-Glycan biosynthesis	hsa00510	3.57E-01	8.88E-01	MAN2A1|1;DPM2|1	2	49	180	7057	Metabolism
Ras signaling pathway	hsa04014	3.63E-01	8.88E-01	REL|1;GNG12|1;GNG13|1;GRIN2B|1;FASLG|1;FGF7|1;IKBKG|1	7	228	180	7057	Environmental Information Processing
Intestinal immune network for IgA production	hsa04672	3.66E-01	8.88E-01	TNFRSF13C|1;ICOS|1	2	50	180	7057	Organismal Systems
Pantothenate and CoA biosynthesis	hsa00770	3.72E-01	8.88E-01	PANK1|1	1	18	180	7057	Metabolism
Vibrio cholerae infection	hsa05110	3.75E-01	8.88E-01	SEC61G|1;ATP6V1D|1	2	51	180	7057	Human Diseases
Salmonella infection	hsa05132	3.77E-01	8.88E-01	WASL|1;PKN2|1;CSF2|1	3	86	180	7057	Human Diseases
Small cell lung cancer	hsa05222	3.77E-01	8.88E-01	CDK6|1;CKS1B|1;IKBKG|1	3	86	180	7057	Human Diseases
Glutathione metabolism	hsa00480	3.84E-01	8.92E-01	MGST1|1;GGCT|1	2	52	180	7057	Metabolism
Glycosaminoglycan degradation	hsa00531	3.88E-01	8.92E-01	HGSNAT|1	1	19	180	7057	Metabolism
Hematopoietic cell lineage	hsa04640	3.90E-01	8.92E-01	TFRC|1;GYPA|1;CSF2|1	3	88	180	7057	Organismal Systems
Glycosaminoglycan biosynthesis - chondroitin sulfate / dermatan sulfate	hsa00532	4.04E-01	9.11E-01	CHPF2|1	1	20	180	7057	-
Rheumatoid arthritis	hsa05323	4.11E-01	9.11E-01	TNFRSF11A|1;ATP6V1D|1;CSF2|1	3	91	180	7057	Human Diseases
Basal cell carcinoma	hsa05217	4.11E-01	9.11E-01	FZD3|1;STK36|1	2	55	180	7057	Human Diseases
Non-small cell lung cancer	hsa05223	4.20E-01	9.21E-01	CDK6|1;CDKN2A|1	2	56	180	7057	Human Diseases
Staphylococcus aureus infection	hsa05150	4.29E-01	9.31E-01	FGG|1;FCGR2C|1	2	57	180	7057	Human Diseases
FoxO signaling pathway	hsa04068	4.48E-01	9.62E-01	FASLG|1;GABARAPL2|1;GABARAP|1;ATG12|1	4	134	180	7057	Environmental Information Processing
Glycosylphosphatidylinositol(GPI)-anchor biosynthesis	hsa00563	4.76E-01	9.74E-01	DPM2|1	1	25	180	7057	Metabolism
MAPK signaling pathway	hsa04010	4.77E-01	9.74E-01	GNG12|1;DDIT3|1;FASLG|1;FGF7|1;IKBKG|1;CACNG8|1;MAP3K2|1	7	255	180	7057	Environmental Information Processing
Synaptic vesicle cycle	hsa04721	4.81E-01	9.74E-01	STXBP1|1;ATP6V1D|1	2	63	180	7057	Organismal Systems
Maturity onset diabetes of the young	hsa04950	4.90E-01	9.74E-01	PDX1|1	1	26	180	7057	Human Diseases
HIF-1 signaling pathway	hsa04066	4.92E-01	9.74E-01	ELOC|1;TFRC|1;EDN1|1	3	103	180	7057	Environmental Information Processing
Glioma	hsa05214	4.97E-01	9.74E-01	CDK6|1;CDKN2A|1	2	65	180	7057	Human Diseases
Collecting duct acid secretion	hsa04966	5.03E-01	9.74E-01	ATP6V1D|1	1	27	180	7057	Organismal Systems
T cell receptor signaling pathway	hsa04660	5.04E-01	9.74E-01	IKBKG|1;CSF2|1;ICOS|1	3	105	180	7057	Organismal Systems
Toll-like receptor signaling pathway	hsa04620	5.11E-01	9.74E-01	IKBKG|1;IFNA7|1;IFNA14|1	3	106	180	7057	Organismal Systems
Amphetamine addiction	hsa05031	5.13E-01	9.74E-01	GRIN2B|1;GRIA4|1	2	67	180	7057	Human Diseases
Cell adhesion molecules (CAMs)	hsa04514	5.14E-01	9.74E-01	LRRC4C|1;OCLN|1;NCAM1|1;ICOS|1	4	146	180	7057	Environmental Information Processing
Glyoxylate and dicarboxylate metabolism	hsa00630	5.16E-01	9.74E-01	MCEE|1	1	28	180	7057	Metabolism
Butanoate metabolism	hsa00650	5.16E-01	9.74E-01	ACSM2A|1	1	28	180	7057	Metabolism
Epithelial cell signaling in Helicobacter pylori infection	hsa05120	5.21E-01	9.74E-01	IKBKG|1;ATP6V1D|1	2	68	180	7057	Human Diseases

Hippo signaling pathway -multiple species	hsa04392	5.28E-01	9.74E-01	TEAD3|1	1	29	180	7057	-
Homologous recombination	hsa03440	5.28E-01	9.74E-01	FSBP|1	1	29	180	7057	Genetic Information Processing
Insulin resistance	hsa04931	5.30E-01	9.74E-01	SLC27A6|1;MLXIP|1;PPARA|1	3	109	180	7057	-
Cholinergic synapse	hsa04725	5.42E-01	9.88E-01	GNG12|1;GNG13|1;KCNJ6|1	3	111	180	7057	Organismal Systems
PPAR signaling pathway	hsa03320	5.52E-01	9.89E-01	SLC27A6|1;PPARA|1	2	72	180	7057	Organismal Systems
Carbon metabolism	hsa01200	5.54E-01	9.89E-01	HIBCH|1;PSPH|1;MCEE|1	3	113	180	7057	Metabolism
RNA polymerase	hsa03020	5.63E-01	9.89E-01	TWISTNB|1	1	32	180	7057	Genetic Information Processing
Apoptosis - multiple species	hsa04215	5.75E-01	9.89E-01	BBC3|1	1	33	180	7057	-
Biosynthesis of amino acids	hsa01230	5.75E-01	9.89E-01	PSPH|1;PYCR1|1	2	75	180	7057	Metabolism
Oxytocin signaling pathway	hsa04921	5.77E-01	9.89E-01	KCNJ6|1;MYL6B|1;CACNG8|1;MYLK2|1	4	158	180	7057	Organismal Systems
RNA degradation	hsa03018	5.89E-01	9.89E-01	DCP1A|1;LSM3|1	2	77	180	7057	Genetic Information Processing
African trypanosomiasis	hsa05143	5.96E-01	9.89E-01	FASLG|1	1	35	180	7057	Human Diseases
Complement and coagulation cascades	hsa04610	6.03E-01	9.89E-01	FGG|1;F7|1	2	79	180	7057	Organismal Systems
Taste transduction	hsa04742	6.30E-01	9.89E-01	GABRA4|1;GNG13|1	2	83	180	7057	Organismal Systems
Allograft rejection	hsa05330	6.36E-01	9.89E-01	FASLG|1	1	39	180	7057	Human Diseases
TGF-beta signaling pathway	hsa04350	6.37E-01	9.89E-01	BMP7|1;AMH|1	2	84	180	7057	Environmental Information Processing
Bladder cancer	hsa05219	6.54E-01	9.89E-01	CDKN2A|1	1	41	180	7057	Human Diseases
Gap junction	hsa04540	6.62E-01	9.89E-01	TUBB1|1;MAP3K2|1	2	88	180	7057	Cellular Processes
Graft-versus-host disease	hsa05332	6.63E-01	9.89E-01	FASLG|1	1	42	180	7057	Human Diseases
Spliceosome	hsa03040	6.70E-01	9.89E-01	SRSF6|1;HNRNPU|1;LSM3|1	3	134	180	7057	Genetic Information Processing
Tuberculosis	hsa05152	6.75E-01	9.89E-01	IFNA7|1;IFNA14|1;TRADD|1;FCGR2C|1	4	179	180	7057	Human Diseases
Systemic lupus erythematosus	hsa05322	6.80E-01	9.89E-01	H3F3A|1;GRIN2B|1;HIST1H2AG|1	3	136	180	7057	Human Diseases
Vasopressin-regulated water reabsorption	hsa04962	6.80E-01	9.89E-01	STX4|1	1	44	180	7057	Organismal Systems
Type I diabetes mellitus	hsa04940	6.80E-01	9.89E-01	FASLG|1	1	44	180	7057	Human Diseases
Sphingolipid metabolism	hsa00600	7.04E-01	9.89E-01	TMEM23|1	1	47	180	7057	Metabolism
Chemokine signaling pathway	hsa04062	7.08E-01	9.89E-01	GNG12|1;GNG13|1;IKBKG|1;WASL|1	4	187	180	7057	Organismal Systems
Type II diabetes mellitus	hsa04930	7.12E-01	9.89E-01	PDX1|1	1	48	180	7057	Human Diseases
Wnt signaling pathway	hsa04310	7.12E-01	9.89E-01	FZD3|1;NOTUM|1;CCND3|1	3	143	180	7057	Environmental Information Processing
Malaria	hsa05144	7.19E-01	9.89E-01	GYPA|1	1	49	180	7057	Human Diseases
Cocaine addiction	hsa05030	7.19E-01	9.89E-01	GRIN2B|1	1	49	180	7057	Human Diseases
Neuroactive ligand-receptor interaction	hsa04080	7.19E-01	9.89E-01	GABRA4|1;LPAR4|1;GRIN2B|1;GRIA4|1;GRIK1|1;SSTR3|1	6	278	180	7057	Environmental Information Processing
Arginine and proline metabolism	hsa00330	7.26E-01	9.89E-01	PYCR1|1	1	50	180	7057	Metabolism
Melanogenesis	hsa04916	7.29E-01	9.89E-01	FZD3|1;EDN1|1	2	100	180	7057	Organismal Systems
Amyotrophic lateral sclerosis (ALS)	hsa05014	7.34E-01	9.89E-01	GRIN2B|1	1	51	180	7057	Human Diseases
AGE-RAGE signaling pathway in diabetic complications	hsa04933	7.34E-01	9.89E-01	EDN1|1;EGR1|1	2	101	180	7057	-
Mineral absorption	hsa04978	7.40E-01	9.89E-01	SLC6A19|1	1	52	180	7057	Organismal Systems
Chagas disease (American trypanosomiasis)	hsa05142	7.49E-01	9.89E-01	FASLG|1;IKBKG|1	2	104	180	7057	Human Diseases
cAMP signaling pathway	hsa04024	7.53E-01	9.89E-01	GRIN2B|1;GRIA4|1;AMH|1;PPARA|1	4	199	180	7057	Environmental Information Processing
Epstein-Barr virus infection	hsa05169	7.70E-01	9.89E-01	HDAC2|1;IKBKG|1;PSMD6|1;TRADD|1	4	204	180	7057	Human Diseases
NOD-like receptor signaling pathway	hsa04621	7.72E-01	9.89E-01	IKBKG|1	1	57	180	7057	Organismal Systems
Acute myeloid leukemia	hsa05221	7.72E-01	9.89E-01	IKBKG|1	1	57	180	7057	Human Diseases
Steroid hormone biosynthesis	hsa00140	7.78E-01	9.89E-01	CYP3A5|1	1	58	180	7057	Metabolism
Glycerolipid metabolism	hsa00561	7.84E-01	9.89E-01	GPAT3|1	1	59	180	7057	Metabolism
Viral myocarditis	hsa05416	7.89E-01	9.89E-01	CAV1|1	1	60	180	7057	Human Diseases
Colorectal cancer	hsa05210	8.00E-01	9.89E-01	APPL1|1	1	62	180	7057	Human Diseases
Longevity regulating pathway - multiple species	hsa04213	8.10E-01	9.89E-01	HDAC2|1	1	64	180	7057	-
Neurotrophin signaling pathway	hsa04722	8.16E-01	9.89E-01	FASLG|1;TP73|1	2	120	180	7057	Organismal Systems
Sphingolipid signaling pathway	hsa04071	8.20E-01	9.89E-01	TMEM23|1;TRADD|1	2	121	180	7057	Environmental Information Processing
Long-term potentiation	hsa04720	8.20E-01	9.89E-01	GRIN2B|1	1	66	180	7057	Organismal Systems
Inflammatory bowel disease (IBD)	hsa05321	8.20E-01	9.89E-01	FOXP3|1	1	66	180	7057	Human Diseases
Aminoacyl-tRNA biosynthesis	hsa00970	8.20E-01	9.89E-01	MTFMT|1	1	66	180	7057	Genetic Information Processing
Platelet activation	hsa04611	8.23E-01	9.89E-01	FGG|1;MYLK2|1	2	122	180	7057	Organismal Systems
Glycolysis / Gluconeogenesis	hsa00010	8.24E-01	9.89E-01	ADH1B|1	1	67	180	7057	Metabolism
Renal cell carcinoma	hsa05211	8.24E-01	9.89E-01	ELOC|1	1	67	180	7057	Human Diseases
Lysosome	hsa04142	8.27E-01	9.89E-01	DNASE2B|1;HGSNAT|1	2	123	180	7057	Cellular Processes
Fc epsilon RI signaling pathway	hsa04664	8.29E-01	9.89E-01	CSF2|1	1	68	180	7057	Organismal Systems
Influenza A	hsa05164	8.32E-01	9.89E-01	FASLG|1;IFNA7|1;IFNA14|1	3	176	180	7057	Human Diseases
Axon guidance	hsa04360	8.32E-01	9.89E-01	FZD3|1;LRRC4C|1;BMP7|1	3	176	180	7057	Organismal Systems
Inositol phosphate metabolism	hsa00562	8.42E-01	9.89E-01	IMPA1|1	1	71	180	7057	Metabolism
Prolactin signaling pathway	hsa04917	8.46E-01	9.89E-01	TNFRSF11A|1	1	72	180	7057	Organismal Systems
B cell receptor signaling pathway	hsa04662	8.50E-01	9.89E-01	IKBKG|1	1	73	180	7057	Organismal Systems
Leishmaniasis	hsa05140	8.54E-01	9.89E-01	FCGR2C|1	1	74	180	7057	Human Diseases
Adherens junction	hsa04520	8.54E-01	9.89E-01	WASL|1	1	74	180	7057	Cellular Processes
Gastric acid secretion	hsa04971	8.54E-01	9.89E-01	MYLK2|1	1	74	180	7057	Organismal Systems

Arrhythmogenic right ventricular cardiomyopathy (ARVC)	hsa05412	8.54E-01	9.89E-01	CACNG8|1	1	74	180	7057	Human Diseases
Antigen processing and presentation	hsa04612	8.68E-01	9.89E-01	TAP2|1	1	78	180	7057	Organismal Systems
Aldosterone synthesis and secretion	hsa04925	8.78E-01	9.89E-01	ATF1|1	1	81	180	7057	-
Hypertrophic cardiomyopathy (HCM)	hsa05410	8.84E-01	9.89E-01	CACNG8|1	1	83	180	7057	Human Diseases
Insulin secretion	hsa04911	8.90E-01	9.89E-01	PDX1|1	1	85	180	7057	Organismal Systems
Focal adhesion	hsa04510	8.96E-01	9.89E-01	CAV1|1;MYLK2|1;CCND3|1	3	203	180	7057	Cellular Processes
ErbB signaling pathway	hsa04012	8.99E-01	9.89E-01	EREG|1	1	88	180	7057	Environmental Information Processing
Proteoglycans in cancer	hsa05205	9.00E-01	9.89E-01	FZD3|1;CAV1|1;FASLG|1	3	205	180	7057	Human Diseases
Ribosome biogenesis in eukaryotes	hsa03008	9.01E-01	9.89E-01	UTP4|1	1	89	180	7057	Genetic Information Processing
Salivary secretion	hsa04970	9.01E-01	9.89E-01	STATH|1	1	89	180	7057	Organismal Systems
Prostate cancer	hsa05215	9.01E-01	9.89E-01	IKBKG|1	1	89	180	7057	Human Diseases
Dilated cardiomyopathy	hsa05414	9.04E-01	9.89E-01	CACNG8|1	1	90	180	7057	-
Endocytosis	hsa04144	9.04E-01	9.89E-01	EPN1|1;TFRC|1;CAV1|1;WASL|1	4	260	180	7057	Cellular Processes
GnRH signaling pathway	hsa04912	9.06E-01	9.89E-01	MAP3K2|1	1	91	180	7057	Organismal Systems
Rap1 signaling pathway	hsa04015	9.10E-01	9.89E-01	LPAR4|1;GRIN2B|1;FGF7|1	3	211	180	7057	Environmental Information Processing
Fc gamma R-mediated phagocytosis	hsa04666	9.11E-01	9.89E-01	WASL|1	1	93	180	7057	Organismal Systems
Longevity regulating pathway	hsa04211	9.13E-01	9.89E-01	APPL1|1	1	94	180	7057	-
Glycerophospholipid metabolism	hsa00564	9.16E-01	9.89E-01	GPAT3|1	1	95	180	7057	Metabolism
Phosphatidylinositol signaling system	hsa04070	9.22E-01	9.89E-01	IMPA1|1	1	98	180	7057	Environmental Information Processing
Progesterone-mediated oocyte maturation	hsa04914	9.22E-01	9.89E-01	CDC26|1	1	98	180	7057	Organismal Systems
Estrogen signaling pathway	hsa04915	9.24E-01	9.89E-01	KCNJ6|1	1	99	180	7057	Organismal Systems
Amoebiasis	hsa05146	9.26E-01	9.89E-01	CSF2|1	1	100	180	7057	Human Diseases
Choline metabolism in cancer	hsa05231	9.28E-01	9.89E-01	WASL|1	1	101	180	7057	Human Diseases
Protein processing in endoplasmic reticulum	hsa04141	9.29E-01	9.89E-01	DDIT3|1;SEC61G|1	2	166	180	7057	Genetic Information Processing
Glucagon signaling pathway	hsa04922	9.30E-01	9.89E-01	PPARA|1	1	102	180	7057	Organismal Systems
Purine metabolism	hsa00230	9.43E-01	9.89E-01	TWISTNB|1;NT5C1A|1	2	176	180	7057	Metabolism
Leukocyte transendothelial migration	hsa04670	9.54E-01	9.89E-01	OCLN|1	1	118	180	7057	Organismal Systems
Thyroid hormone signaling pathway	hsa04919	9.54E-01	9.89E-01	HDAC2|1	1	118	180	7057	Organismal Systems
Toxoplasmosis	hsa05145	9.55E-01	9.89E-01	IKBKG|1	1	119	180	7057	Human Diseases
Oocyte meiosis	hsa04114	9.59E-01	9.89E-01	CDC26|1	1	123	180	7057	Cellular Processes
AMPK signaling pathway	hsa04152	9.62E-01	9.89E-01	STRADB|1	1	125	180	7057	Environmental Information Processing
Olfactory transduction	hsa04740	9.62E-01	9.89E-01	GNG13|1;OR12D2|1;OR1E1|1;OR4F5|1;OR4C11|1;OR52E8|1	6	420	180	7057	Organismal Systems
Tight junction	hsa04530	9.73E-01	9.89E-01	OCLN|1	1	139	180	7057	Cellular Processes
Signaling pathways regulating pluripotency of stem cells	hsa04550	9.75E-01	9.89E-01	FZD3|1	1	142	180	7057	Cellular Processes
Phospholipase D signaling pathway	hsa04072	9.77E-01	9.89E-01	LPAR4|1	1	144	180	7057	-
Adrenergic signaling in cardiomyocytes	hsa04261	9.80E-01	9.89E-01	CACNG8|1	1	149	180	7057	Organismal Systems
cGMP-PKG signaling pathway	hsa04022	9.87E-01	9.91E-01	MYLK2|1	1	167	180	7057	Environmental Information Processing
Calcium signaling pathway	hsa04020	9.91E-01	9.91E-01	MYLK2|1	1	180	180	7057	Environmental Information Processing
